# Ancient polyploidization events influence the evolution of the ginseng family (Araliaceae)

**DOI:** 10.3389/fpls.2025.1595321

**Published:** 2025-06-13

**Authors:** Angélica Gallego-Narbón, Gabriel Johnson, Mario Fernández-Mazuecos, Jun Wen, Virginia Valcárcel

**Affiliations:** ^1^ Department of Botany, National Museum of Natural History, Smithsonian Institution, Washington, DC, United States; ^2^ Departamento de Biología, Universidad Autónoma de Madrid, Madrid, Spain; ^3^ Centro de Investigación en Biodiversidad y Cambio Global (CIBC‐UAM), Universidad Autónoma de Madrid, Madrid, Spain; ^4^ Departamento de Biodiversidad y Conservación, Real Jardín Botánico (RJB), CSIC, Madrid, Spain

**Keywords:** Araliaceae, Asian Palmate group, chromosome evolution, Hyb-Seq, whole genome duplication, polyploidy, hybridization

## Abstract

**Introduction:**

Whole genome duplication events (WGDs) have been recognized as major drivers of evolution in plants, especially when they involve hybridization (allopolyploidization). In this study we evaluated if WGDs acted as evolutionary forces at the origin and early divergence of the Asian Palmate group (AsPG) of the plant family Araliaceae. This clade encompasses most of the generic and species diversity as well as most of the polyploids of the family, and a role of hybridization in its origin has been suggested.

**Methods:**

In order to test this hypothesis, we obtained nuclear and plastid time-calibrated phylogenomic trees including 80% of Araliaceae genera (37 genera, 237 species) using the Hyb-Seq approach. The role of WGDs in the early evolution of the AsPG was tested using ancestral chromosome number reconstructions based on chromosome counts for 62% of the sampled genera, while recent polyploidization events were explored by inferring ploidy of the sequenced species from allelic frequencies.

**Results:**

Phylogenetic analyses of nuclear and plastid sequences provided highly resolved but incongruent topologies consistent with ancient hybridization not only for the origin of the AsPG, but also in the second most highly diverse clade of the family. Our ancestral chromosome number reconstructions supported that one or two WGDs preceded the origin of two of the three main clades of Araliaceae (AsPG and *Polyscias-Pseudopanax*), which could have acted as background variables necessary for the posterior diversification of these lineages. Ploidy inference based on allelic frequencies provided signal of recent polyploidization in the AsPG and the third main clade of Araliaceae (*Aralia*-*Panax*).

**Conclusions:**

In summary, WGDs are linked to the origin of the main clades of the Araliaceae family, but the drivers of the strong diversification of the AsPG remain an open question.

## 1 Introduction

Chromosomes can evolve in a series of ways that range from structural rearrangements like duplications, deletions, inversions, and translocations among individual chromosomes to partial genome duplication and polyploidization or whole genome duplication (WGD, Bado et al., 2015). These changes are common across different plant groups and have evolutionary significance (i.e., they can lead to an increase in speciation or extinction) depending on the characteristics of the lineage involved and its different adaptations to the surrounding environment (Stebbins, 1971). The footprint of WGD events is widespread across angiosperms (Soltis and Soltis, 2009), and polyploidy is currently recognized as one of the main drivers of speciation in plants (Valdés-Florido et al., 2023). WGD can occur as a result of the fusion of unreduced diploid gametes within the same species (autopolyploidy) or by means of hybridization between different species that is associated with genome doubling (allopolyploidy; Landis et al., 2018). While WGDs, and especially allopolyploidization events, were considered an evolutionary dead-end in the past (Stebbins, 1971), its evolutionary potential is now widely recognized (Soltis et al., 2015). In fact, there is increasing evidence that WGD was a trigger of diversification in several species-rich lineages of angiosperms (Schranz et al., 2012; Tank et al., 2015; Landis et al., 2018). Allopolyploidization events could be especially relevant for speciation, given that they often provide genetic and morphological novelty (Qiu et al., 2020). In this regard, multiple gene copies from duplicate genomes can lead to the neofunctionalization of homologues to develop new functions in molecular pathways and allow greater genomic plasticity (Tao et al., 2021).

The ginseng family (Araliaceae, Apiales) constitutes an ideal study case to assess the role of chromosome number changes and WGDs in evolution. This diverse family encompasses approximately 46 genera and 1700 species, with a widespread distribution across five continents but mostly occupying tropical and subtropical areas in Asia, the Americas and Oceania (Wen et al., 2001; Nicolas and Plunkett, 2014; Valcárcel and Wen, 2019). The family is characterized by chromosome numbers from *n*=12 to *n*=96, with *n*=12 and *n*=24 as the numbers present in most genera, and polyploids present in several lineages (e.g.: *Aralia*, *Hedera*, and *Panax*; Wen and Zimmer, 1996; Vargas et al., 1999; Yi et al., 2004; Zhang et al., 2025). In addition, the signal of an ancient WGD has been detected in the early evolution of Apiales (Landis et al., 2018), which could be related to the high diversity currently found in the most diversified families Apiaceae and Araliaceae. Altogether, the chromosome counts and ploidy variability in the family and the presence of ancient WGDs in the history of Apiales suggest that polyploidization could be an important force in the evolution of Araliaceae. Yi et al. (2004) compiled chromosome counts in the family and observed that *n*=12 was the most common chromosome number in most Araliaceae lineages, while *n*=24 was the most common number in the Asian Palmate group (AsPG; Wen et al., 2001; Plunkett et al., 2004a), a clade that encompasses around 50% of the generic and species diversity of the family. These results pointed to an early WGD related to the origin of the AsPG. Gallego-Narbón et al. (2022) tested this hypothesis on highly resolved nuclear and plastid trees of these genera by performing ancestral chromosome number reconstructions which revealed a WGD at the origin of the AsPG. The authors additionally inferred a hybridization event involved in the origin of the AsPG and hypothesized that the early evolution of the AsPG was driven by an allopolyploidization event. However, broader taxon sampling and higher phylogenetic resolution are necessary to test this hypothesis. Therefore, the placement of the detected WGD in the evolutionary history of Araliaceae and its impact on the early divergence of AsPG genera could not be assessed.

In this study, we aim to assess the relevance of chromosome evolution and ploidy changes in the origin and subsequent evolution of the Asian Palmate group of Araliaceae. We hypothesized that an ancient allopolyploidization event underlays the origin of the clade. Additionally, given the evidence of polyploidization in several Araliaceae lineages, we considered that WGDs may have also been relevant in the origin and subsequent evolution of the other two main clades of Araliaceae (*Polyscias*-*Pseudopanax* and *Aralia*-*Panax*; Wen, 2001; Plunkett et al., 2004b). These hypotheses were explored by: 1) disentangling the evolutionary relationships in the family Araliaceae using nuclear and plastid time-calibrated phylogenies with an extensive taxon sampling (96% of AsPG genera and 80% of Araliaceae genera), 2) assessing the link between WGDs and speciation events in the early evolution of Araliaceae and determining their role in the evolution of the most speciose clades based on ancestral chromosome number reconstructions, and 3) evaluating the signal of recent polyploidization in the evolution of Araliaceae.

## 2 Methods

### 2.1 Species sampling and library preparation

A total of 239 samples were included in this study, comprising 238 Araliaceae samples (237 species) and *Mackinlaya schlechteri* (Harms) Philipson (Apiaceae) as the outgroup (**Supplementary Data 1**). We represented the three main clades of Araliaceae (AsPG: 169 taxa, 168 species, *Aralia*-*Panax*: 28 species, and *Polyscias*-*Pseudopanax*: 11 species) and seven of the eleven early-diverging lineages (greater *Raukaua*: 13 species; *Cussonia*-*Seemanaralia*: six species; *Osmoxylon*: three species; two species of *Harmsiopanax* and *Hydrocotyle*, and one species of *Neocussonia*-*Astropanax* and *Trachymene*). Our sampling covered 22 AsPG genera (96% of AsPG genera) and 37 Araliaceae genera (80% of Araliaceae genera). These percentages are based on the taxonomic treatment of the family published by Plunkett et al. (2018) and subsequent taxonomic publications (Lowry et al., 2019, 2020; Fiaschi et al., 2020; Plunkett et al., 2021).

The raw reads of 73 species were obtained from previous studies: 63 species from Gallego-Narbón et al. (2022) and two species from the 1000 Plant Transcriptomes Initiative (www.onekp.com), obtained using an Illumina HiSeq platform sequencing 150 bp paired‐end reads, and 8 species from Shee et al. (2020) obtained using an Illumina MiSeq platform sequencing 250 bp paired-end reads. Genomic data for the remaining 166 taxa were newly generated using both silica-dried materials (132 samples) and herbarium specimens from the United States National Herbarium (US, 34 samples). DNA extraction, library preparation, and target enrichment followed the Hyb-Seq protocol used by Gallego-Narbón et al. (2022), except for the number of samples per indexed pool (12 for silica-dried material and 10 for herbarium material). We used a Araliaceae-specific bait set targeting 936 nuclear exons designed based on two genomes and two transcriptomes representing the three main Araliaceae lineages (AsPG, *Aralia*-*Panax* and *Polyscias*-*Pseudopanax*; Gallego-Narbón et al., 2022) synthesized by Daicel Arbor BioSciences (Ann Arbor MI, USA). Paired-end sequencing with 150-bp reads was performed on the Illumina HiSeq 4000 platform at Novogene (Sacramento, CA, USA).

### 2.2 Sequence assembly and alignment

Resulting reads were trimmed using Trimmommatic 0.39 (Bolger et al., 2014) following the parameters in Gallego-Narbón et al. (2022). Trimmed paired files were assembled using HybPiper 2.1.1 (Johnson et al., 2016) by mapping the reads to the bait targets using BWA (Li and Durbin, 2009) and assembling the contigs with SPAdes (Bankevich et al., 2012). For the nuclear data, exons were assembled for the bait-set loci and flanking regions were retrieved with the “run_intronerate” tool. HybPiper statistics were obtained and paralogy was assessed with the “paralog_retriever” tool. Plastid loci were assembled by mapping reads to a reference plastome (*Eleutherococcus senticosus* (Rupr. & Maxim.) Maxim, GenBank accession JN637765) as described in Gallego-Narbón et al. (2022). Species with low recovery that showed clearly incorrect phylogenetic placements (i.e., recovered in a different genus) in preliminary analyses were not included in the final alignments. Alignments were performed for each gene separately using MAFFT 7.475 with standard parameters (Katoh and Standley, 2013), and those with < 10 species were excluded from further analyses. After removal of the paralogs detected with the “paralog_retriever” HybPiper tool, the nuclear matrix was concatenated (*NuTot*) and the plastid gene alignments were concatenated (*CpTot*) in Geneious Prime 5.1.7 (Kearse et al., 2012). Statistics for these concatenated matrices were generated with AMAS 1.0 (Borowiec, 2016).

### 2.3 Phylogenomic analyses

Concatenation-based and coalescent-based analyses were performed. For concatenation-based analyses, we applied a maximum likelihood (ML) approach using RAxML-HPC v.8.2.10 (Stamatakis, 2014) on the concatenated matrices under a GTRCAT evolutionary model with 1,000 fast bootstrap replicates using one partition per locus. RAxML generates reduced matrices by excluding sites that contain only undetermined values prior to the analysis. These matrices were also used as the input for Bayesian Inference (BI) analyses. A BI approach was also applied exclusively to the *CpTot* matrix in ExaBayes v.1.4.1 (Aberer et al., 2014) using a parsimony starting tree, and two parallel MCMC runs with two coupled chains for 1.2 million generations under a GTR+G evolutionary model. A burn-in of 25% was used and convergence was assessed using Tracer 1.7.2 (Rambaut et al., 2018). A BI analysis was not run using the *NuTot* matrix because of computing limitations. To run coalescent-based analyses, 1,000 fast bootstrap replicates were generated with RAxML under a GTRCAT model for each nuclear locus alignment to obtain gene trees. These were then processed with ASTRAL 5.6.2 with default settings (Zhang et al., 2018) to obtain a coalescent-based tree. Local posterior probabilities (PP_local_) and quartet scores were retrieved as measures of branch support and gene tree discordance for the coalescent-based tree. The quartet scores indicate the proportion of gene trees that support the main topology (Q1), the main alternative (Q2), and the second alternative (Q3) for each quartet connecting four descendant lineages. Low Q1 values are indicative of high gene tree discordance. In this scenario, similar Q2 and Q3 values can be interpreted as a signal of incomplete lineage sorting (ILS), while different Q2 and Q3 values are interpreted as a signal of hybridization (Schumer et al., 2016).

### 2.4 Phylogenetic dating

Divergence times were estimated using a penalized likelihood approach as implemented in treePL (Smith and O’Meara, 2012), with our ML nuclear and plastid trees separately as the input. We used two calibration points: (1) the 95% highest posterior density intervals obtained by Magallón et al. (2015) for the crown node of Araliaceae (49.28-72.9 Ma), and (2) the age of the oldest known macrofossil of *Hedera* (23.0-39.9 Ma, Rim, 1994; Kong, 2000) for the divergence between *Hedera* and *Merrilliopanax*. The three-step calibration was started with a preliminary calibration with the “prime” option to establish the optimal parameter values. Secondly, the optimal parameter values obtained from the previous calibration were used with a random sample cross-validation with 200,000 iterations for penalized likelihood and 5,000 iterations for cross-validation. This second calibration provided the best value for the smoothing parameter, which was used in the third and final calibration. The resulting nuclear and plastid time-calibrated trees were used for downstream analyses. As treePL does not directly provide uncertainty ranges, we performed time calibration for each of the 1,000 bootstrapped trees generated by RAxML analyses of nuclear and plastid matrices with the parameters obtained for the ML trees as described above. Age statistics for the nodes were summarized in TreeAnnotator v.1.10 (Drummond et al., 2012).

### 2.5 Chromosome evolution and chromosomal cladogenesis

To study the role of ancient WGDs in the evolution of Araliaceae, we used ChromEvol 2.0 (Mayrose et al., 2010; Glick and Mayrose, 2014) and ChromoSSE (Freyman and Höhna, 2018) in RevBayes v.1.0.12 (Höhna et al., 2016). Given that the species sampling was highly incomplete for most of the genera both for molecular data and for chromosome counts, chromosomal variation during the evolution of each genus could not be analyzed at the species level. Considering this limitation and our interest in exploring the role of ancient WGDs in the early evolution of Araliaceae, ancestral reconstructions at the genus level were performed. Chromosome counts compiled by Gallego-Narbón et al. (2022) for the Araliaceae family were complemented with additional counts of newly sampled genera from the Chromosome Counts Database (CCDB, Rice et al., 2015), the Index to Plant Chromosome Numbers database (IPCN, Goldblatt and Johnson, 2006), and Yi et al. (2004). This newly compiled cytological database contained 143 counts from *n*=9 to *n*=96 representing 24 Araliaceae genera (62% of sampled genera, **Supplementary Data 2**). When a genus presented several different chromosome numbers (eight genera), the frequency of each chromosome number was estimated based on the proportion of counts for each number, considering the total number of species with chromosome counts per genus (number of species with a certain chromosome number for a genus/total number of species with chromosome counts for that genus). Generic lineages whose chromosome number was not available (15) were treated as missing data as specified in the ChromEvol guidelines. The resulting chromosome numbers or proportions of each number per genus were used to reconstruct ancestral chromosome numbers based on the nuclear and plastid time-calibrated trees pruned to include a single species per genus, except for the non-monophyletic genera for which one tip per linage was retained (40 samples in total, including *Mackinlaya* as the outgroup). ChromEvol tested ten different models of chromosome evolution and estimated ancestral chromosome numbers, whole genome duplication (WGD), half genome duplication, and disploidy events across the phylogeny. The best nuclear and plastid models of chromosome evolution were selected according to their AIC values.

Afterwards, the same inputs were used for a ChromoSSE analysis (Freyman and Höhna, 2018), as implemented in RevBayes v.1.0.12 (Höhna et al., 2016). Using this analysis, we assessed whether the chromosome changes detected early in the evolution of Araliaceae were temporally correlated with speciation events, or if they occurred along branches independently of the speciation events recovered in the phylogeny. To construct our ChromoSSE nuclear and plastid models, the parameters of chromosome evolution were restricted to those included in the best ChromEvol models (chromosome gain constant rate, chromosome loss constant rate, and WGD rate) and speciation rates associated with no chromosome change, chromosome gain, chromosome loss, and WGD were incorporated. Taxon sampling probability was adjusted to 0.8 (percentage of Araliaceae genera sampled). ChromoSSE does not consider the possibility of having multiple chromosome counts for each tip of the phylogeny or the presence of missing data. Therefore, we took a conservative approach to this issue, by using the most frequent count per genus as the input for the eight genera exhibiting multiple values, and the chromosome number inferred by ChromEvol for genera without available chromosome counts. The analyses were run with 5,500 iterations discarding the first 500 as burn-in. We ensured the effective sample sizes (ESS) of all the parameters were higher than 200 for all replicates as suggested by Freyman and Höhna (2018). The ChromoSSE results were visualized using the R package RevGadgets (Tribble et al., 2022).

### 2.6 Assessment of recent polyploidy based on Hyb-Seq data

Recent polyploidy events in Araliaceae were assessed using the pipeline nQuire (Weiß et al., 2018), which estimates ploidy level from target-enriched sequence data without chromosome counts or cytometric data (Viruel et al., 2019, 2023). This allowed the estimation of the ploidy levels for all the species included in the complete nuclear and plastid trees, even those without chromosome counts. This methodology estimates the allelic ratios of the SNPs of a species and compares them with those expected for different ploidy levels, allowing the estimation of the ploidy level for diploids, triploids, and tetraploids, and is especially useful to detect the signal of recent polyploidization events (Viruel et al., 2019, 2023). The trimmed reads for all samples included in the final phylogenetic trees were mapped to a reference using exclusively loci not identified as paralogs by HybPiper. This reference was used to run nQuire, using the “denoise” tool to remove basal noise that could hinder the identification of allelic frequency patterns following the pipeline of Viruel et al. (2019). Ploidy level determination was based on several criteria according to Weiß et al. (2018) and Viruel et al. (2019). The best model for each species was selected by comparing the Δlog of the three models (2*x*, 3*x* and 4*x*) with the free model. The model of best fit must exhibit an elevated R^2^ and y-y slope, as well as low standard error and Norm SSR to ensure sufficient adjustment. When no model exhibited a R^2^≥0.1, then the species did not fit any of the three models, which could be due to either poor data quality or alternative ploidy scenarios, such as higher levels of polyploidy. To further explore the data, we calculated allelic frequencies from the nQuire denoised results and obtained several plots per sample following Viruel et al. (2019): histograms of allelic frequencies per sample, boxplots of allelic frequencies per sample and per SNP, distribution of the allelic ratios per SNP, and density plots of allelic ratio values. Mean, median, and proportions of allelic ratios <2 per sample were calculated. Median values <2 were considered as suggestive of diploidy while those >2 as indicative of polyploidy.

## 3 Results

### 3.1 Sequence capture and alignment

Sequences were recovered for 100% of targeted nuclear regions (936 regions) and 90% of targeted plastid regions (262 regions). After removing samples with low recovery and incorrect phylogenetic placement, 227 samples were included in the *NuTot* alignment and 225 in the *CpTot* alignment. The proportion of recovered length for targeted regions was generally high (average of 86.2% for nuclear regions and 81% for plastid regions). The concatenated nuclear alignment (*NuTot*) included 156 loci and 562,329 bp after paralog removal, while the plastid concatenated alignment (*CpTot*) was 177,454 bp long. Additional information on Hyb-Piper statistics, capture efficiency, paralog retrieval, and alignment statistics can be found in **Supplementary Data 3**.

### 3.2 Phylogenetic relationships and dating

**Figure 1:**
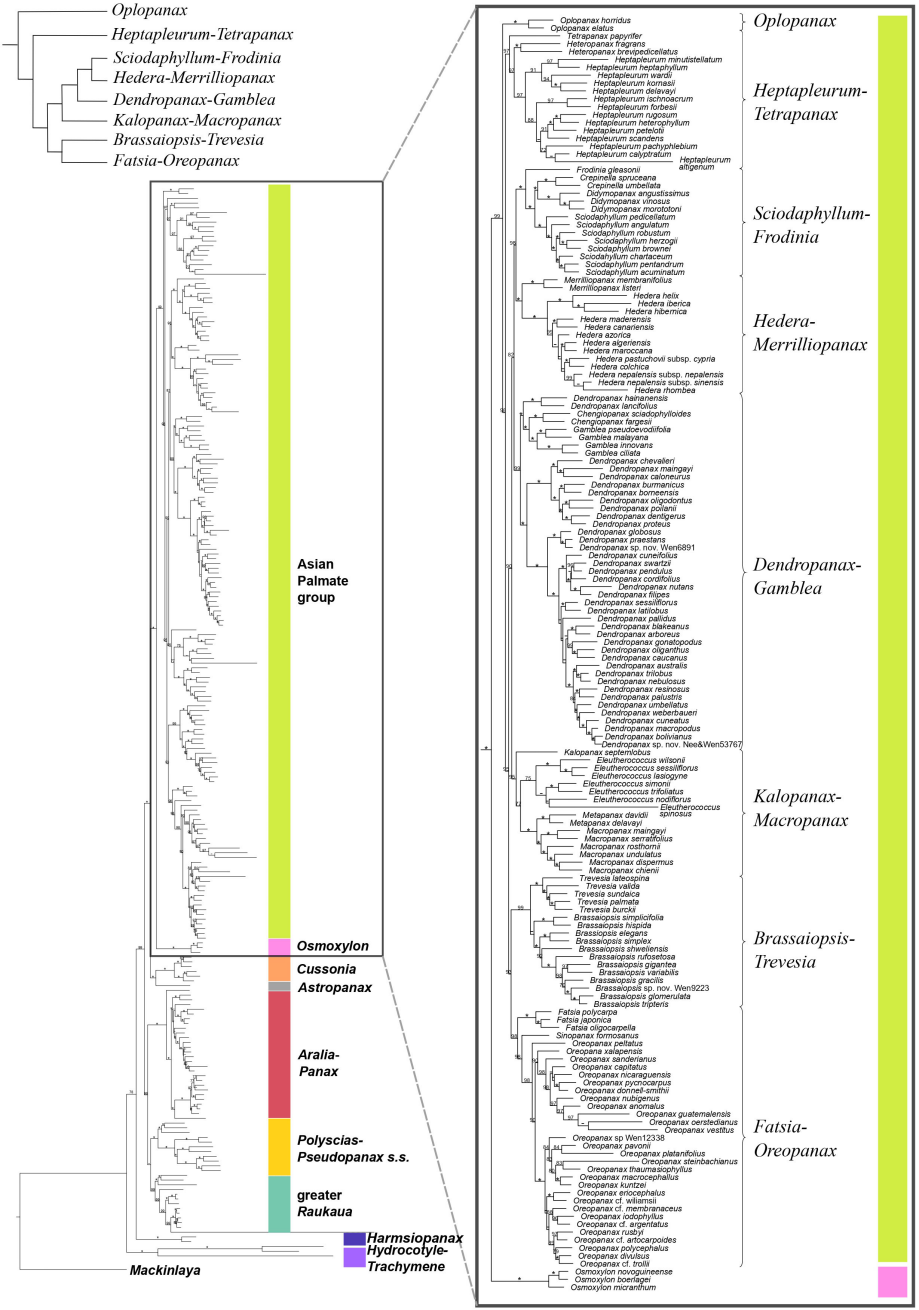
Concatenation-based maximum-likelihood phylogeny of Araliaceae based on the nuclear DNA matrix (*NuTot*) generated through Hyb-Seq, with a detailed view of the phylogenetic relationships among major clades of the Asian Palmate group (AsPG). Numbers above branches are percentage bootstrap support (BS) values, with asterisks indicating BS values equal to 100. Hyphens indicate unsupported branches.

**Figure 2:**
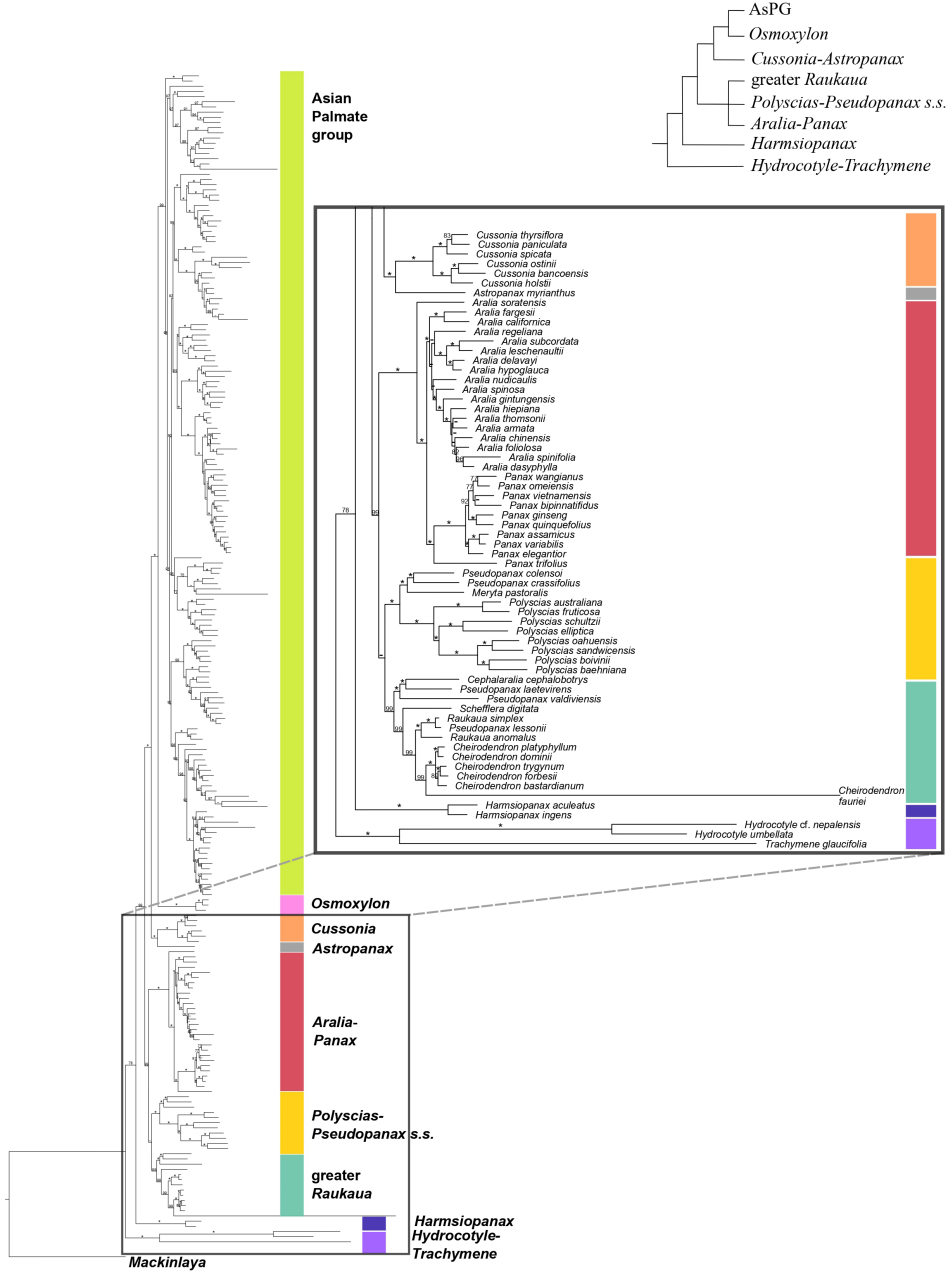
Concatenation-based maximum-likelihood phylogeny of Araliaceae based on the nuclear DNA matrix (*NuTot*) generated through Hyb-Seq, with a detailed view of the early phylogenetic relationships among major clades of Araliaceae. Numbers above branches are percentage bootstrap support (BS) values, with asterisks indicating BS values equal to 100. Hyphens indicate unsupported branches.

The concatenation-based nuclear analysis (**Figures 1**, **2**) showed high support for most phylogenetic relationships among Araliaceae genera and all phylogenetic relationships among the AsPG genera. The dating analysis based on the nuclear phylogeny (**Supplementary Figure 1A**) recovered a crown age in the late Cretaceous or early Paleocene (72.9 [63.8-72.9] Ma) for Araliaceae. The *Hydrocotyle*-*Trachymene* clade exhibited the earliest divergence within Araliaceae, followed by *Harmsiopanax* (Paleocene to early Eocene, 49.4-63.4 Ma), which was sister to a clade including the rest of Araliaceae lineages. This clade was formed by two main clades: one including the clades *Aralia*-*Panax*, *Polyscias*-*Pseudopanax s.s.* and greater *Raukaua* in a polytomy, and another clade including the AsPG-*Osmoxylon* clade sister to the *Cussonia*-*Astropanax* clade. The divergence of *Aralia*-*Panax*, *Polyscias*-*Pseudopanax s.s.*, and the greater *Raukaua* clades occurred between the early and the late Eocene (36.9-53.8 Ma), with crown ages from the early to the late Eocene for greater *Raukaua* (33.8-49.9 Ma), from the late Eocene to the Oligocene for *Polyscias*-*Pseudopanax s.s.* (27.6-35.9 Ma), and from the Oligocene to the early Miocene for *Aralia*-*Panax* (18.8-24.2 Ma). On the other hand, the AsPG and *Osmoxylon* diverged between the late Eocene to the Oligocene (31.7-38.3 Ma), and the *Cussonia*-*Astropanax* clade diverged between the early to the late Eocene (34.9-44.4 Ma) with a crown age between the late Eocene to the Oligocene (27.2-36.6 Ma). The AsPG crown age was placed in the Oligocene (29.8-33.0 Ma). The earliest-diverging lineage within the AsPG was *Oplopanax*, followed by the *Heptapleurum*-*Tetrapanax* clade, which was sister to a clade including the rest of the AsPG lineages. Within this clade we recovered a clade with *Brassaiopsis*-*Trevesia* and *Fatsia*-*Oreopanax* clades as sisters to each other, which was sister to a clade including the *Kalopanax*-*Macropanax* and *Dendropanax*-*Gamblea* clades as consecutive sisters of a clade formed by the *Sciodaphyllum*-*Frodinia* clade as sister to the *Hedera*-*Merrilliopanax* clade. Ten of the sampled AsPG genera diverged during the Oligocene (*Dendropanax*, *Eleutherococcus*, *Fatsia*, *Hedera*, *Heptapleurum*, *Heteropanax*, *Kalopanax*, *Merrilliopanax*, *Oplopanax* and *Tetrapanax*), while the rest diverged during the Miocene.

The topology displayed by the coalescent-based nuclear phylogeny (**Supplementary Figure 2**) was mostly compatible with that of the concatenation-based phylogeny except for the placement of *Harmsiopanax*, which appeared as sister to the *Hydrocotyle*-*Trachymene* clade. This reconstruction maintained high support for recent relationships but displayed lower support for deep relationships as a result of high gene tree discordance, with quartet scores congruent with a pattern of ILS. The coalescent-based nuclear phylogeny recovered an internal polytomy of four Araliaceae lineages: the clade formed by *Harmsiopanax* as sister to the *Hydrocotyle*-*Trachymene* clade, the greater *Raukaua* clade, the clade formed by *Aralia*-*Panax* and *Polyscias*-*Pseudopanax s.s.* clades, and a clade including the *Cussonia*-*Astropanax* clade and *Osmoxylon* as consecutive sisters of the AsPG. Within the AsPG we recovered and internal polytomy involving *Oplopanax*, *Heptapleurum*-*Tetrapanax*, and the clade including the remaining AsPG lineages. The latter exhibited a polytomy comprising five lineages (*Dendropanax*-*Gamblea* clade, *Hedera*-*Merrilliopanax* clade, *Kalopanax*-*Macropanax* clade, *Sciodaphyllum*-*Frodinia* clade, and a clade formed by the *Brassaiopsis*-*Trevesia* and *Fatsia*-*Oreopanax* clades).

**Figure 3:**
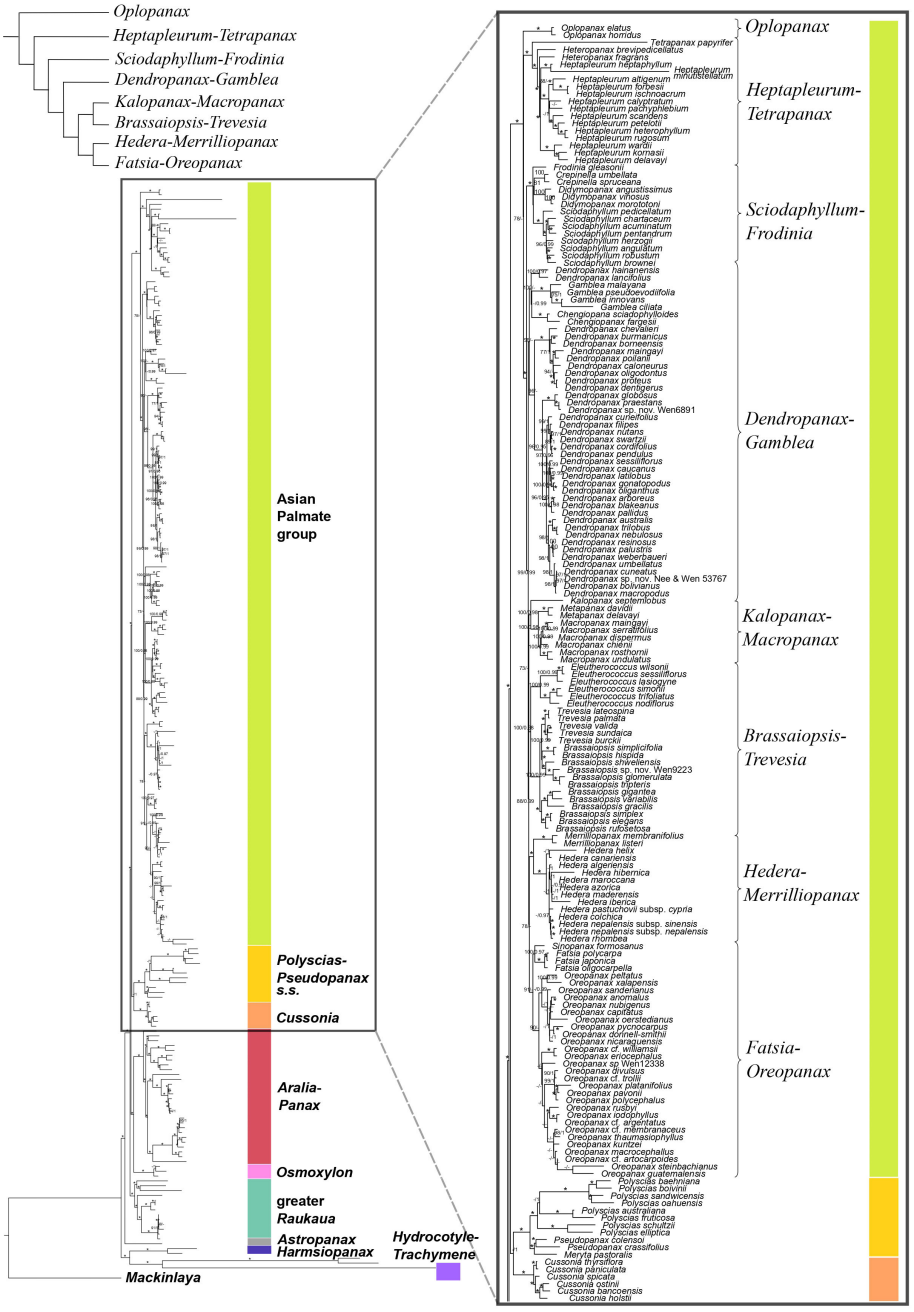
Concatenation-based maximum-likelihood phylogeny of Araliaceae based on the plastid DNA matrix (*CpTot*) generated through Hyb-Seq, with a detailed view of the phylogenetic relationships among major clades of the Asian Palmate group (AsPG). Numbers above branches are percentage bootstrap support (BS) and Bayesian posterior probability (PP) values, with asterisks indicating maximum support (BS = 100, PP = 1.0). Hyphens indicate unsupported branches.

**Figure 4:**
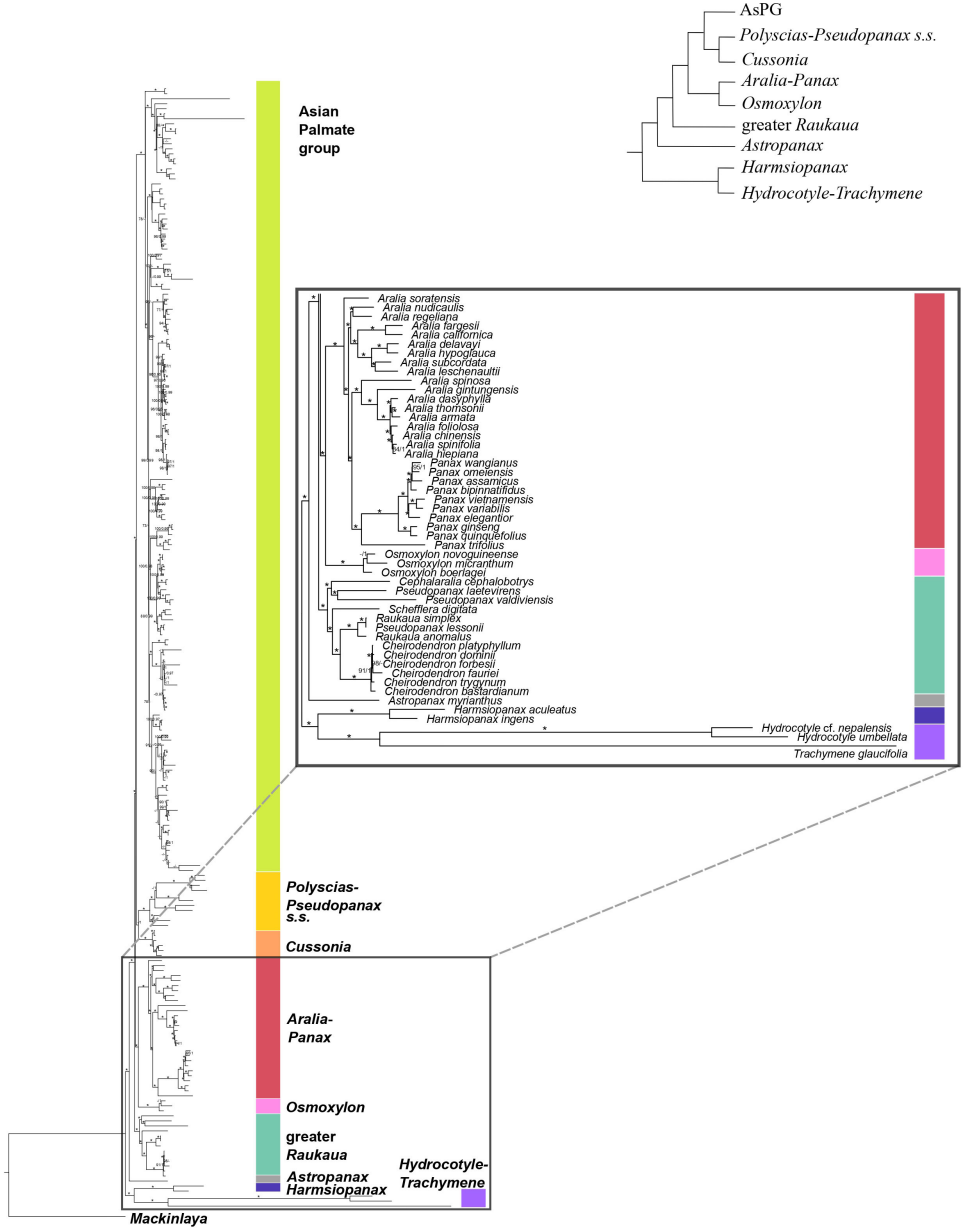
Concatenation-based maximum-likelihood phylogeny of Araliaceae based on the plastid DNA matrix (*CpTot*) generated through Hyb-Seq, with a detailed view of the early phylogenetic relationships among major clades of Araliaceae. Numbers above branches are percentage bootstrap support (BS) and Bayesian posterior probability (PP) values, with asterisks indicating maximum support (BS = 100, PP = 1.0). Hyphens indicate unsupported branches.

The plastid phylogeny (**Figures 3**, **4**) also recovered the main Araliaceae clades and those within the AsPG, and resolved the evolutionary relationships among them. However, the relationships among these clades were highly incongruent between nuclear and plastid phylogenies (**Figures 1**-**4**; **Supplementary Figure 2**). The *Hydrocotyle*-*Trachymene* clade was sister to *Harmsiopanax*, forming a clade that diverged during the late Cretaceous (67.5-72.4 Ma, **Supplementary Figure 1B**), sister to a clade including the rest of Araliaceae lineages. Within this highly diverse clade, *Astropanax* diverged during the Paleocene to Early Eocene (41.6-67.6 Ma), followed by the greater *Raukaua* clade, which diverged from its sister clade between the late Eocene and the Oligocene (31.8-37.4 Ma). The greater *Raukaua* clade was sister to a clade including two main clades: the AsPG as sister to a clade including *Cussonia* and *Polyscias*-*Pseudopanax s.s.* (Oligocene crown age, 30.4-33.1 Ma), and a clade formed by *Aralia*-*Panax* as sister to *Osmoxylon* (Oligocene crown age, 27.5-30.6 Ma). The AsPG, *Polyscias*-*Pseudopanax s.s.*, and *Aralia*-*Panax* exhibited crown ages during the Oligocene (25.7-29.7 Ma), between the Oligocene and early Miocene (22.1-25.9 Ma), and during the Early Miocene (19.8-22.6 Ma), respectively. Within the AsPG, *Oplopanax*, *Heptapleurum*-*Tetrapanax*, *Sciodaphyllum*-*Frodinia* and *Dendropanax*-*Gamblea* were consecutive sisters of a clade including two clades: *Kalopanax*-*Macropanax* as sister to *Brassaiopsis*-*Trevesia* and *Hedera*-*Merrilliopanax* as sister to *Fatsia*-*Oreopanax*.

Our nuclear and plastid phylogenies indicate that several Araliaceae genera are paraphyletic (*Aralia* and *Raukaua*, with *Panax* and *Pseudopanax lessonii* nested within them, respectively) or polyphyletic (*Dendropanax* and *Pseudopanax*). In this regard, *Dendropanax hainanensis* and *D. lancifolius* form a well-supported clade independent from the remaining *Dendropanax* species and sister to the *Chengiopanax*-*Gamblea* clade (*Dendropanax hainanensis*-*lancifolius* lineage). *Pseudopanax s.s* formed a clade with *Meryta* sister to *Polyscias* (*Polyscias*-*Pseudopanax s.s.* clade), whereas the American *Pseudopanax* species formed a clade with *Cephalaralia* that had an early divergence in the greater *Raukaua* clade, and the New Zealander species *P. lessonii* was nested in *Raukaua s.s.*

### 3.3 Ancestral polyploidy in the evolution of Araliaceae

The best-fitting model explaining chromosome evolution was “DysDup” for both nuclear (AIC=40.2499, log-likelihood=-17.1250) and plastid (AIC=40.3929, log-likelihood=-17.1965) analyses (**Supplementary Data 4**). This model considers ascending and descending dysploidy as well as WGD events.

**Figure 5:**
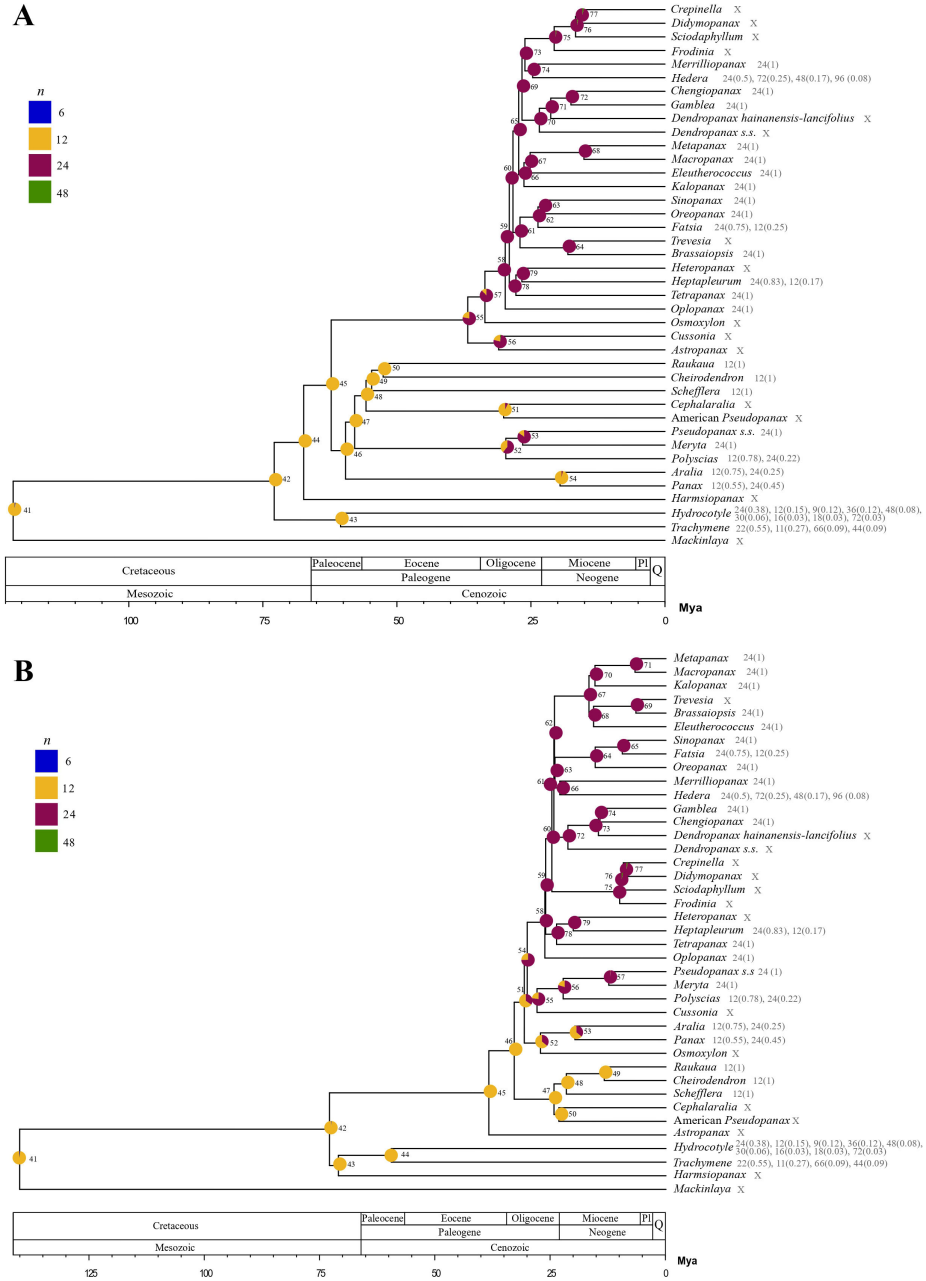
Ancestral chromosome number reconstructions for Araliaceae based on the best-fit ChromEvol models for nuclear **(A)** and plastid **(B)** time-calibrated phylogenetic trees. Ancestral probabilities for each chromosome number are provided as pie charts with different colors for each ancestral number. Only chromosome numbers with inferred ancestral probabilities above zero in at least one ancestral node are shown in the legend. Chromosome counts and their proportion are provided for each genus when available. Genera without available counts are marked with an “x”. ChromEvol node numbering is indicated.

The estimated ancestral chromosome number for Araliaceae was *n*=12 for both nuclear and plastid reconstructions (posterior probabilities of 0.98 and 0.99, respectively; **Figure 5**) and the estimated chromosome numbers for all ancestral nodes were *n*=12 or *n*=24 in both reconstructions (12 nodes with *n*=12 and 27 with *n*=24 for the nuclear phylogeny; 13 nodes with *n*=12 and 26 with *n*=24 for the plastid phylogeny). In addition, both the nuclear and plastid reconstructions recovered a descending disploidy event for *Trachymene* (from *n*=12 to *n*=11). According to the nuclear reconstruction, at least four WGD events occurred during the evolutionary history of Araliaceae (4.22 expected events), with two of them in internal branches and representing transitions from *n*=12 to *n*=24 (**Figure 5A**). One WGD event was retrieved at the base of the clade formed by the AsPG, *Osmoxylon*, *Cussonia* and *Astropanax* (N55, expected transitions=0.78). The second ancient WGD event was recovered at the base of the *Polyscias*-*Pseudopanax s.s.* clade (N52, expected transitions=0.61). The probability of WGD events was also relatively elevated for *Mackinlaya* (expected transitions=0.32) and *Hydrocotyle* (expected transitions=0.30) and the base of the *Pseudopanax s.s.*-*Meryta* clade (N53, expected transitions=0.25). On the other hand, the plastid reconstruction (**Figure 5B**) supported at least two WGD events during the evolutionary history of Araliaceae (2.82 expected events), one of them in an internal branch. This WGD event was recovered at the base of the clade including the AsPG, *Polyscias*-*Pseudopanax* s*.s.* and *Cussonia* (N54, expected transitions=0.40) and represents a change from *n*=12 to *n*=24. The probability of WGD events was also relatively elevated for *Mackinlaya* (expected transitions=0.32) and *Hydrocotyle* (expected transitions=0.26).

**Figure 6:**
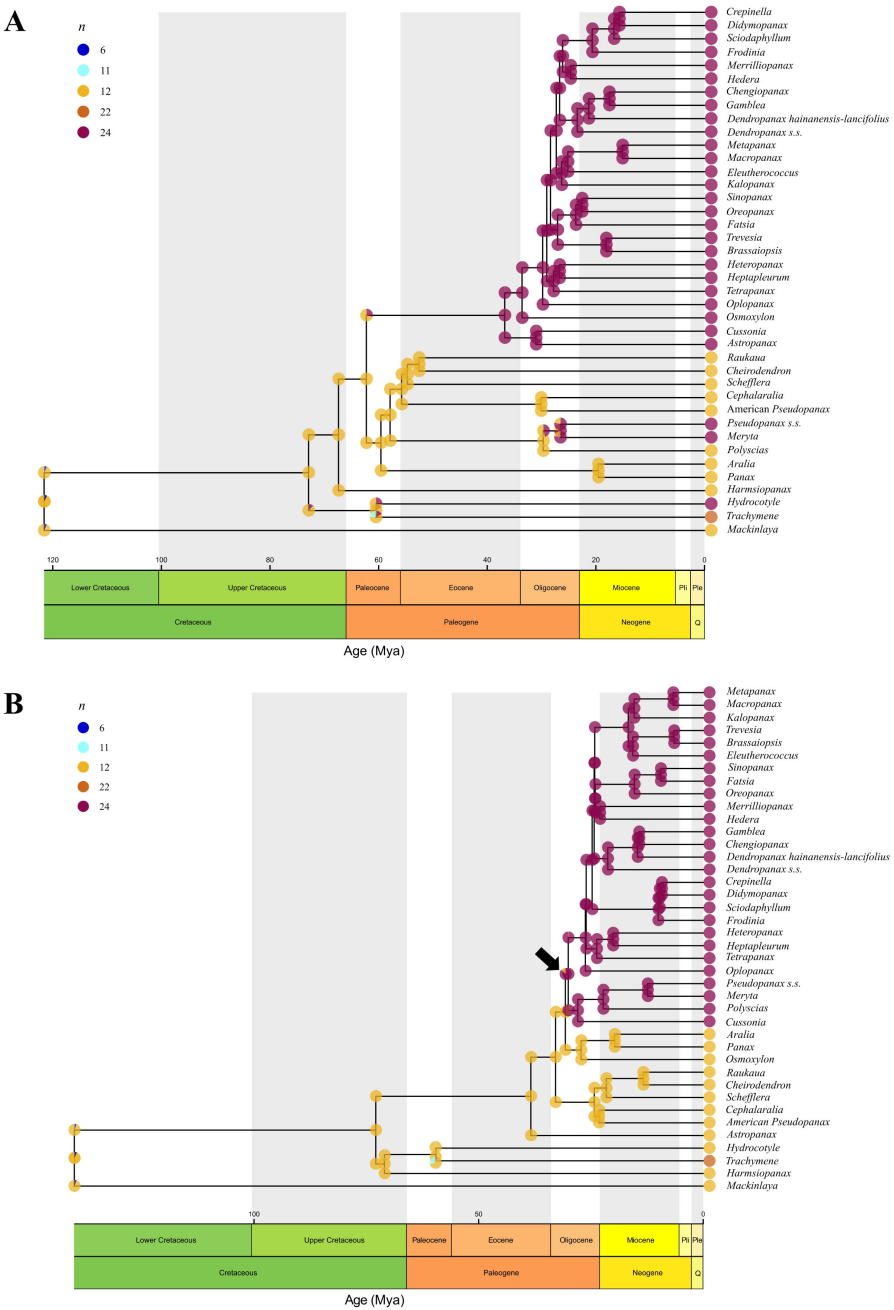
Ancestral chromosome number reconstructions for Araliaceae based on ChromoSSE models for nuclear **(A)** and plastid **(B)** time-calibrated phylogenies. Ancestral probabilities for each chromosome number are provided as pie charts with different colors for each ancestral number. Only chromosome numbers with inferred ancestral probabilities above zero in at least one ancestral node are shown in the legend. Chromosome counts and their proportions are provided for each genus.

Regarding the nature of chromosome number changes, the distribution of the parameters in ChromoSSE models supported that chromosome number changes were mostly unrelated to speciation (**Figure 6**; **Supplementary Figure 3**, **Supplementary Data 5**). For chromosome change events, the highest rates were obtained for the polyploidization parameter, while for the parameters assessing the relationship between chromosome number changes and speciation the highest rates were found for no chromosome changes, several orders of magnitude higher than the rest of the rates. These results indicated that chromosome number changes were mostly asynchronous with speciation in Araliaceae. The ChromoSSE nuclear model (**Figure 6A**) recovered two ancient WGDs unrelated with speciation. The ancient WGD event at the base of the clade formed by the AsPG, *Osmoxylon*, *Cussonia* and *Astropanax*, recovered using ChromEvol, was recovered again by ChromoSSE. However, the ancient WGD event at the base of the *Polyscias*-*Pseudopanax s.s.* clade recovered with ChromEvol was instead recovered within that clade at the base of the *Meryta*-*Pseudopanax s.s.* clade in the ChromoSSE nuclear reconstruction. Our plastid ChromoSSE model recovered a single WGD event that was associated with the origin of the clade formed by the AsPG, *Polyscias*-*Pseudopanax s.s.* and *Cussonia* (**Figure 6B**), congruently with ChromEvol results (**Figure 5B**).

### 3.4 Assessment of recent ploidy events within the evolution of Araliaceae genera

Eighty-eight percent of the analyzed species conformed to one of the ploidy levels tested with nQuire (2*x*, 3*x* or 4*x*; 199 species, **Supplementary Data 6**). Most of the samples were characterized as diploids (174 taxa), while 23 were characterized as tetraploids and two were inferred as triploids (*Hedera colchica* and *Aralia subcordata*). The inferred tetraploids were scattered across different Araliaceae clades, with most of them recovered for *Aralia*-*Panax* (12 species in two genera) and the AsPG (7 species in two genera). Median allelic frequencies ranged from 1.16 to 1.77 for diploids, 1.78 to 2.11 for triploids, and 1.30 to 2.95 for tetraploids (**Supplementary Data 7**). The proportion of diploid loci (allelic ratio <2) ranged from 64.1 to 95.6% in diploids, 36.3 to 62.3% in triploids and 11.0 to 60.7% in tetraploids. Exploratory plots of allelic frequencies for each sample are provided in **Supplementary Figure 4**.

## 4 Discussion

### 4.1 Phylogenomics increases the resolution of the AsPG and Araliaceae backbones

Until recently, the knowledge of early evolutionary relationships in Araliaceae was based on studies using a small number of nuclear and plastid DNA regions (Wen et al., 2001; Plunkett et al., 2004a, b, 2020; Yi et al., 2004; Nicolas and Plunkett, 2009; Mitchell et al., 2012; Valcárcel et al., 2014; Li and Wen, 2016). In addition, phylogenetic studies have been frequently focused on the most diverse clades (AsPG, *Polyscias*-*Pseudopanax s.s*. and *Aralia*-*Panax*), while the lineages with low species diversity have usually been underrepresented. As a result of this limited genetic data and biased generic sampling, the early evolutionary patterns in Araliaceae remain poorly understood. The studies conducted so far at the family level consistently pointed to an early divergence of the genera *Hydrocotyle*, *Trachymene* and/or *Harmsiopanax* as sisters to a clade including the core of Araliaceae (Plunkett et al., 2004a, b; Yi et al., 2004; Nicolas and Plunkett, 2009; Mitchell et al., 2012; Valcárcel et al., 2014; Li and Wen, 2016), and they recovered a large polytomy at the base of the core Araliaceae. The recent application of the Hyb-Seq technique to sequence hundreds of loci along with higher generic sampling (65% of Araliaceae genera; Gallego-Narbón et al., 2022), recovered well resolved phylogenetic trees. These revealed incongruent relationships when comparing the plastid and nuclear trees, suggesting hybridization events were involved in the origin of the core Araliaceae, as well as in the origin of two species-rich clades (AsPG and the *Aralia*-*Panax* clade). After improving the generic sampling to 80% of Araliaceae genera, the results of this study confirmed the previous findings in Gallego-Narbón et al. (2022). On the one hand, the early divergence of the *Hydrocotyle*-*Trachymene* clade was confirmed, as well as the incongruent placement of *Harmsiopanax*, that either appears as sister to this clade or to the core Araliaceae. On the other hand, this study recovered (i) the AsPG as sister to *Osmoxylon* (**Figure 1**) or to the clade formed by *Polyscias*-*Pseudopanax* and *Cussonia* (**Figure 3**), (ii) the *Aralia*-*Panax* clade as sister to *Polyscias*-*Pseudopanax* or to *Osmoxylon*, and (iii) *Astropanax* as sister to *Cussonia*, forming a clade sister to the AsPG-*Osmoxylon* clade or as the earliest-diverging clade within the core. Altogether, these results preclude resolving the early evolutionary relationships in Araliaceae due to extensive phylogenetic incongruence between gene trees (**Supplementary Figure 2**) and between nuclear and plastid data (**Figures 1**, **2** *vs* **3**, **4**). Given that, for most of the nodes, gene tree incongruence supports a pattern of ILS (Schumer et al., 2016), our results provide evidence of early radiation in the evolution of Araliaceae. In addition, previous hybridization analyses in Araliaceae provided evidence of hybridization events involving the AsPG and other Araliaceae clades (*Astropanax* and greater *Raukaua*, Gallego-Narbón et al., 2022). Considering our results in this context, we interpret that they provide evidence of inter-lineage hybridization at a deep evolutionary level in the family with a subsequent evolutionary radiation, similar to the pattern previously inferred for the early evolution of its largest clade, the AsPG (Gallego-Narbón et al., 2022).

The present results improved the phylogenetic resolution in the AsPG. Previous research based on a limited number of loci recovered an internal polytomy involving most of the main clades of the AsPG (Lowry et al., 2004; Plunkett et al., 2004a; Li and Wen, 2013, 2016; Valcárcel et al., 2014). Subsequent studies based on plastid genomes with limited lineage sampling improved the resolution in comparison with Sanger-based studies (Valcárcel and Wen, 2019; Kang et al., 2023), but the deep relationships within the AsPG remained unresolved. The use of Hyb-Seq led to a significant improvement in the resolution, with only one internal node unresolved for the nuclear-based phylogeny of the AsPG and two internal nodes for the plastid-based phylogeny (Gallego-Narbón et al., 2022). Here, the increased generic and species sampling enabled the resolution of all the internal nodes in both nuclear and plastid trees when concatenation-based methods were applied (**Figures 1**, **3**). Both trees supported an early divergence of *Oplopanax* followed by *Heptapleurum*-*Tetrapanax* as sister to a clade including the rest of the AsPG lineages. This topology was supported in previous studies based on nuclear loci (Gallego-Narbón et al., 2022). Research using Sanger sequencing of plastid DNA also suggested this topology (Valcárcel et al., 2014; Li and Wen, 2016; Plunkett et al., 2020), but only Valcárcel and Wen (2019) obtained enough support for these early relationships in the AsPG, which were confirmed here. In addition, our nuclear tree helped clarify the sister relationships of two clades (*Hedera*-*Merrilliopanax* and *Kalopanax*-*Macropanax*), that, according to Gallego-Narbón et al. (2022), were part of a polytomy alongside a clade including the rest of the lineages (*Dendropanax*-*Gamblea* clade, *Sciodaphyllum*-*Frodinia* clade, and the clade formed by *Brassaiopsis*-*Trevesia* and *Fatsia*-*Oreopanax* clades). Specifically, the present nuclear reconstruction (**Figure 1**) supports *Hedera*-*Merrilliopanax* as sister to the *Sciodaphyllum*-*Frodinia* clade, with *Dendropanax*-*Gamblea* and *Kalopanax*-*Macropanax* as consecutive sisters of this clade. However, all these clades are part of a polytomy according to the coalescence-based analysis (**Supplementary Figure 2**), with a distribution of quartet scores congruent with a pattern of ILS (equal values of Q2 and Q3; Schumer et al., 2016). These results provide further support for the pattern of an evolutionary radiation during the early evolution of the AsPG that has been discussed in previous literature (Valcárcel and Wen, 2019; Gallego-Narbón et al., 2022). In addition, our plastid tree (**Figures 3**, **4**) is topologically incongruent with both nuclear trees (**Figures 1**, **2**; **Supplementary Figure 2**), which we interpret as evidence of the hybridization events identified in Gallego-Narbón et al. (2022) for the early evolution of the AsPG.

### 4.2 Polyploidization and evolution in Araliaceae

The ancestral chromosome number reconstructions consistently recovered WGDs around the Eocene-Oligocene that coincided in time with the origin of two of the most species-rich lineages (**Figures 5**, **6**). According to the chromosome reconstruction based on the nuclear tree (**Figure 5A**), one ancient WGD occurred at the base of the clade formed by AsPG-*Osmoxylon* and *Cussonia*-*Astropanax* and a second one at the base of the *Polyscias*-*Pseudopanax s.s.* lineage. However, according to the reconstruction based on the plastid tree, only one ancient WGD occurred around this time, placed at the base of a clade including the AsPG, *Polyscias*-*Pseudopanax s.s.* and *Cussonia* (**Figure 5B**). The link between these WGDs and speciation is unclear. According to the nuclear reconstruction the ancient WGD that preceded the origin of the AsPG cannot be linked to speciation (**Figure 6A**) while the WGD detected in the plastid reconstruction prior to the origin of the AsPG is linked to speciation (**Figure 6B**). The incongruence in the number and placement of WGD events between nuclear and plastid reconstructions was expected, considering the extensive incongruence we detected between the two topologies for these clades (**Figures 1**, **2** *vs* **3**, **4**). Despite these incongruent results, the two reconstructions (nuclear and plastid) agreed on the recovery of ancient WGDs predating the origin of the same lineages, that currently include polyploids and display the highest number of species (AsPG, *Polyscias*-*Pseudopanax s.s.* and *Cussonia*). We interpret the ancient WGD predating the origin of the AsPG as a background factor for the early radiation of this group. This follows the rationale described by Bouchenak-Khelladi et al. (2015) that the factors present before diversification bursts provide the necessary conditions for future evolutionary radiations, which is related to the WGD radiation time-lag model set by Schranz et al. (2012). These authors identified a consistent pattern of ancient WGDs in angiosperms leading to species-rich clades sister to species-poor clades, suggesting that there is a one-node lag between the acquisition of the novelties associated to WGDs and the subsequent radiations. Later on, this hypothesis was expanded by the identification of a general pattern in which the lag between ancient WGDs and the subsequent diversification bursts generally involved several nodes (Landis et al., 2018). The results of the present study are congruent with this time-lag hypothesis, as the ancient WGDs we recovered in Araliaceae predated by one or two-nodes the origin of two of the three most species-rich clades of Araliaceae (AsPG and *Polyscias*-*Pseudopanax s.s.*), that together encompass approximately 70% of the species diversity of the Araliaceae family (Plunkett and Lowry, 2010; Valcárcel and Wen, 2019).

Given the extensive pattern of hybridization found in those Araliaceae clades where hybridization has been studied in more detail (e.g., AsPG, Gallego-Narbón et al., 2022; *Aralia*-*Panax*, Liu et al., 2023; Zhang et al., 2025), and the consistent pattern of genomic incongruence affecting the divergence of the main clades of Araliaceae obtained in this study, we consider that ancient WGDs could be the result of hybridization events (allopolyploidy). Gallego-Narbón et al. (2022) hypothesized that the WGD associated with the origin of the AsPG involved the genera *Polyscias* and *Osmoxylon*. We propose that an ancestor of the African genera of Araliaceae (*Astropanax* and *Cussonia*) was involved in the WGD at the origin of Araliaceae. While our nuclear trees supported the close relationship between the *Astropanax*-*Cussonia* and the AsPG-*Osmoxylon* clade (**Figures 1**, **2**; **Supplementary Figure 2**), the plastid analysis supports that *Astropanax* is an early-diverging lineage in the Araliaceae core clade and *Cussonia* forms a clade with *Polyscias*-*Pseudopanax s.s.* (**Figures 3**, **4**). Gallego-Narbón et al. (2022) performed phylogenetic network analyses for the family Araliaceae, with all networks supporting a hybrid origin for the AsPG. Most of the networks supported *Osmoxylon* as the major sister of the AsPG and *Astropanax* as the minor sister, which would mean that an ancestor of *Osmoxylon* and an ancestor of *Astropanax* were the parental lineages involved in the hybrid origin of the AsPG. However, these analyses generally supported that there was an additional hybridization event involved in the origin of the AsPG, which means that several hybridization events could be involved in the origin of the AsPG. Further research providing additional chromosome counts and phylogenetic trees with increased species sampling across the family Araliaceae are required to precisely determine the placement of allopolyploidization events in the evolution of Araliaceae, and their role in the origin and diversification of the AsPG.

### 4.3 Allelic frequencies and whole genome duplications

The use of allelic frequencies derived from Hyb-Seq data provides a novel approach to infer ploidy levels without requiring fresh material (Weiß et al., 2018). This methodology is promising, and several pipelines have been released in recent years, but their performance has not yet been intensively explored (e.g., nQuire, Weiß et al., 2018; and nQuack, Gaynor et al., 2024). In our study, the use of allelic frequencies derived from Hyb-Seq data provided further insights into the distribution of different ploidy levels across the family Araliaceae and provided evidence of recent polyploidization events. This methodology allowed the detection of polyploids across the three main lineages of Araliaceae (AsPG, *Polyscias*-*Pseudopanax s.s*. and *Aralia*-*Panax*; **Supplementary Figure 4** and **Supplementary Data 6** and **7**), which agrees with previous information based on chromosome counts (Yi et al., 2004). The most relevant result was the detection of abundant tetraploids in the *Aralia*-*Panax* clade, which supports the relevance of recent polyploidization events in the evolution of this clade. However, the correlation between ploidy levels derived from allelic frequencies and those obtained from direct ploidy measurements has not been extensively tested in previous studies and the combination of this methodology with direct measurements is still recommended (Viruel et al., 2019; Gaynor et al., 2024).

In fact, while all existing evidence provided by chromosome counts and ancestral reconstructions supported the polyploid origin of the AsPG (Yi et al., 2004; Gallego-Narbón et al., 2022; **Figures 5**, **6**), our analysis indicates that most of the sampled AsPG species show a pattern of allelic frequencies compatible with diploidy (**Supplementary Data 6**). These results showcase that ploidy inference based on allelic frequencies is not suitable to identify the signal of ancient polyploidization events. This pattern was already pointed out by the authors of the methodology, who emphasized the usefulness of allelic frequencies to study polyploidization at an intraspecific scale (Weiß et al., 2018). Our inference of ancient polyploidization events (**Figures 5**, **6**) and extensive detection of diploids based on allelic frequencies in the AsPG (**Supplementary Data 6**) suggest that this clade might have undergone a process of diploidization, which is common after allopolyploidization in angiosperms (Leitch and Bennett, 2004).

It is also remarkable that we found several incongruences between the ploidy level inferred from allelic frequencies and that obtained from chromosome counts for those genera whose ploidy levels have been studied more extensively. In this regard, chromosome counts of *Hedera* have been used to study ploidy for the twelve species of the genus (Vargas et al., 1999). The use of allelic frequencies allowed the accurate inference of diploidy for five species (*H. azorica*, *H. canariensis*, *H. helix*, *H. maroccana* and *H. nepalensis*) and species known to be hexaploid (*H. iberica*, *H. maderensis*, *H. pastuchovii*) were incompatible with diploidy, triploidy, or tetraploidy, as expected (**Supplementary Data 6**). However, the ploidy levels inferred for the four remaining species of the genus were incorrect, with a diploid and a tetraploid species not fitting into any of the tested models (*H. rhombea* and *H. algeriensis*, respectively), an octoploid characterized as triploid (*H. colchica*) and a tetraploid (*H. hibernica*) showing allelic frequencies typical of a diploid. Previous research on the plant genus *Dioscorea* showed that allelic frequencies of recent autotetraploids may be comparable to those of diploids (Viruel et al., 2019, 2023). However, this is not the case for *H. hibernica*, which is known to be an allotetraploid (Vargas et al., 1999). The incongruence between chromosome counts and allelic frequencies may be the result of the extensive hybridization that has been reported for *Hedera* based on molecular data (Vargas et al., 1999; Ackerfield and Wen, 2003), as hybridization can influence the pattern of allelic frequencies (Gaynor et al., 2024). In addition, the *Hedera* polyploids whose ploidy level was incorrectly inferred generally exhibited early divergences during the evolutionary history of *Hedera* according to previous research (Gallego-Narbón et al., 2023), which provides further support for the application of ploidy inference techniques based on allelic frequencies exclusively for species of recent origin. Nevertheless, we acknowledge that new methodologies for inferring ploidy from allelic frequencies, published during the development of our study and improving upon nQuire (Gaynor et al., 2024), are promising for addressing some of the limitations of our approach.

In addition, we observed an extensive overlap in the median allelic frequencies between species categorized as tetraploids and those categorized as diploids by the models (**Supplementary Data 7**). This overlap is not observed for the proportion of diploid loci (allelic ratio <2) and the distributions of allelic frequencies are clearly different between diploids and tetraploids (**Supplementary Figure 4**). In this regard, model parameters (**Supplementary Data 6**) must be interpreted together with the median and distribution of allelic frequencies and the percentage of diploid loci (**Supplementary Data 7** and **Supplementary Figure 4**) for a more accurate assessment of ploidy, especially for those samples in which different model parameters support different ploidy models. Despite these limitations, our results still support the relevance of WGDs not only in the early evolutionary history of the family, but also in the evolutionary history of several genera across different clades of Araliaceae. Additionally, they provide a preliminary assessment of ploidy in the family that should be evaluated in further detail in future research incorporating novel ploidy inference methodologies and direct ploidy measurements.

## 5 Conclusions

We obtained the first nuclear and plastid phylogenomic trees of the Asian Palmate group of Araliaceae with full internal support as a result of a high taxonomic and genetic sampling. Our ancestral chromosome number reconstructions based on these phylogenies provide further support for the occurrence of a WGD event related to the origin of the AsPG. According to the reconstruction based on the plastid tree, this polyploidization event not only preceded the origin of the AsPG but also the origin of the *Polyscias*-*Pseudopanax s.s.* clade. The use of information provided by allele frequencies allowed the detection of several recent polyploids in Araliaceae, especially for the *Aralia*-*Panax* clade. However, this methodology failed to accurately detect the signal of ancient polyploidization and recent polyploidization in lineages with extensive hybridization (e.g., *Hedera*). Therefore, the application of direct methods to measure ploidy is still recommended in such cases. In summary, our results support that ancient WGD events were involved in the origin of two of the three most diversified lineages of Araliaceae (AsPG and *Polyscias*-*Pseudopanax s.s.*) and polyploidization was involved in the recent evolutionary history of the third most diversified lineage (*Aralia*-*Panax* clade). Further research expanding the taxon sampling and providing additional cytogenetic data will be necessary to disentangle the spatiotemporal context of polyploidization events and their role in the diversification of the family Araliaceae.

## Data Availability

The raw reads (FASTQ files) used in this study are available in the NCBI Sequence Read Archive (SRA) database (Bioproject ID: PRJNA841627).
