# Supplementary material for: Ancient polyploidization events influence the evolution of the ginseng family (Araliaceae)
Source: Front Plant Sci. 2025 Jun 13;16:1595321. doi: 10.3389/fpls.2025.1595321 (PMC12202383; doi:10.3389/fpls.2025.1595321)
Supplement: Supplementary file 1 [file DataSheet1.pdf]

Supplementary Data 1. List of material used in the Hyb-Seq study. Locality, voucher, and accession numbers are provided for each sample and herbarium code is included for herbarium samples.

Species	Locality	Voucher	Accession	Herbarium code
Aralia armata (Wall. ex G.Don) Seem.	China	J. Wen 6290	forthcoming	-
Aralia californica S. Watson	USA	J. Wen 6694-9	forthcoming	-
Aralia chinensis L.	China, Guangdong, Shenzen	J. Wen 13626	forthcoming	-
Aralia dasyphylla Miq.	Indonesia, Java, Cibodas Botanical Garden area	J. Wen 10129*	SRS13357386	-
Aralia delavayi J. Wen	China, Yunnan, Bingchuan	J. Wen 3040*	SRS13357423	-
Aralia fargesii Franch.	China	J. Wen 5001	forthcoming	-
Aralia foliolosa Seem. ex C.B. Clarke	China, Yunnan, Simao Shi	G. M. Plunkett 1983	forthcoming	-
Aralia gintungensis C.Y.Wu ex K.M.Feng	China, Yunnan, Jindong Xian	J. Wen 9075	forthcoming	-
Aralia hiepiana J.Wen & Lowry	Vietnam, Lam Dong Prov.	J. Wen 11004*	SRS13357387	.
Aralia hypoglauca (C.J.Qi & T.R.Cao) J.Wen & Y.F.Deng	China, Hunan	Y. F. Deng s.n.*	SRS13357398	-
Aralia leschenaultii (DC.) J.Wen	-	J. Wen 6374-3	forthcoming	-
Aralia nudicaulis L.	-	J. Wen s.n.	forthcoming	-
Aralia regeliana Marchal	Mexico, Queretaro	M. R. McVaugh 10348	forthcoming	-
Aralia soratensis Marchal	Bolivia, Santa Cruz, Mairana	M. H. Nee 53845	forthcoming	-
Aralia spinifolia Merr.	China	J. Wen 5772-2	forthcoming	-
Aralia spinosa L.	USA, Maryland	J. Wen 17212	forthcoming	-
Aralia subcordata (Wall. ex G.Don) J.Wen	China	J. Wen 5818	forthcoming	-
Aralia thomsonii Seem. ex C.B.Clarke	China, Yunnan, Jindong Xian	J. Wen 9097	forthcoming	-
Astropanax myrianthus (Baker) Lowry, G.M.Plunkett, Gostel & Frodin	Madagascar, Antsiranana, National Montagne d'Ambre	J. Wen 9570*	SRS13357435	-
Brassaiopsis bodinieri (H.Lév.) J.Wen & Lowry	Vietnam	J. Wen 10874	forthcoming	-
Brassaiopsis elegans Ridl.	Malaysia, Selangor. Raub - Kuala Kubu Bharu	J. Wen 8408*	SRS13357420	-
Brassaiopsis gigantea J.Wen & Lowry	Vietnam, Hoa Binh Prov.	J. Wen 10941*	SRS13357431	-
Brassaiopsis glomerulata (Blume) Regel	Vietnam, Vinh Phuc Prov.	J. Wen 10821*	SRS13357443	-

Brassaiopsis gracilis Hand.-Mazz.	Vietnam, Lao Cai Prov.	J. Wen 10859*	SRS13357453	-
Brassaiopsis hispida Seem.	-	J. Wen 5057	forthcoming	-
Brassaiopsis rufosetosa (Ridl.) Jebb	Malaysia, Pahang, Peng Lai Xian Jin Chinese Temple	J. Wen 8406*	SRS13357456	-
Brassaiopsis shweliensis W.W.Sm.	China	J. Wen 6500	forthcoming	-
Brassaiopsis simplex (King) B.C.Stone	Malaysia, Perak, Luzon	J. Wen 8357	forthcoming	-
Brassaiopsis simplicifolia C.B.Clarke	-	X. F. Gao s.n.	forthcoming	-
Brassaiopsis sp. nov.	China, Tibet, Pailong Xiang	J. Wen 9223*	SRS13357455	-
Brassaiopsis tripteris (H.Lév.) Rehder	China, Guangxi, Huanjiang Xian (Niujiaozhai)	J. Wen 13741*	SRS13357388	-
Brassaiopsis variabilis C.B.Shang	Vietnam, Ninh Binh Prov.	J. Wen 10907*	SRS13357389	-
Cephalaralia cephalobotrys (F.Muell.) Harms	Australia, New South Wales, World of the Blue Mountains	J. Wen 12186*	SRS13357390	-
Cheirodendron bastardianum (Decne.) Frodin	French Polynesia, Marchesas Islands, Temetiu	J. Price 205	forthcoming	US3452111
Cheirodendron domini Krajina	United States of America, Hawaii, Alakai swamp	P. van Royen 11723	forthcoming	US2831185
Chengiopanax fargesii (Franch.) C.B.Shang & J.Y.Huang	China, Hunan, Zhiyunshan Nature Preserve	J. Wen 9316*	SRS13357391	-
Cheirodendron fauriei Hochr.	United States of America, Hawaii, Kawaihua-Hanalei	D. H. Lorence 6381	forthcoming	US3260883
Cheirodendron forbesii (Sherff) Lowry	United States of America, Hawaii, Kauai	K. R. Wood 17361	forthcoming	US3722670
Cheirodendron platyphyllum (Hook. & Arn.) Seem.	United States of America, Hawaii, Kauai	T. B. Croat 44895	forthcoming	US3034372
Cheirodendron trigynum (Gaudich.) A.Heller	United States of America, Hawaii, Kilauea	F. R. Fosberg 51745	forthcoming	US3121514
Chengiopanax sciadophylloides (Franch. & Sav.) C.B.Shang & J.Y.Huang	Japan, Honshu	Soejima s.n.*	SRS13357449	-
Crepinella spruceana (Seem.) G.M.Plunkett, Lowry & D.A.Neill	Brazil, São Gabriel da Cachoeira - Amazonas, Campina	C. Farney 1781	forthcoming	US3339772
Crepinella umbellate (N.E.Br.) G.M.Plunkett, Lowry & D.A.Neill	Guyana, Potaro-Siparuni, mount Ayanganna	H. D. Clarke 9301	forthcoming	US3451535
Cussonia bancoensis Aubrév. & Pellegr.	Ivory Coast, Abidjan	J. T. S. Teillier 329	forthcoming	US2595436
Cussonia holstii Harms ex Engl.	Ethiopia, Shoa, mount Zuquala	F. G. Meyer 7635	forthcoming	US2519823
Cussonia ostinii Chiov.	Ethiopia, Kaffa	F. G. Meyer 8074	forthcoming	US2519739
Cussonia paniculata Eckl. & Zeyh.	South Africa	Davies 5872/1	forthcoming	-
Cussonia spicata Thunb.	Cameroon, Sud-Ouest, Southern Bakundu Forest Reserve	D. Thomas 2503	forthcoming	US3057843

Cussonia thyrsiflora Thunb.	South Africa; Western Cape, 2 km W of Knysna	J. Wen 10057	forthcoming	US3537465
Dendropanax arboreus (L.) Decne. & Planch.	Jamaica	J. Wen 11843*	SRS13357392	-
Dendropanax australis Fiaschi & Jung-Mend.	Brazil, São Paulo, Eldorado	P. Fiaschi 3872	forthcoming	US3698039
Dendropanax blakeanus Britton	Jamaica, Portland, Ecclesdown	J. Wen 11857	forthcoming	-
Dendropanax bolivianus Gand.	Bolivia, Santa Cruz, 4.5 Km SSE of Buena Vista	M. H. Nee 53797	forthcoming	US3521525
Dendropanax borneensis (Philipson) Merr.	Malaysia, Borneo, Kinabalu National Park	J. Wen 11699	forthcoming	US3650193
Dendropanax burmanicus Merr.	China, Yunnan, Gongshan Xian	L. Heng 33274	forthcoming	US3562561
Dendropanax caloneurus (Harms) Merr.	Vietnam, Lao Cai, Sa Pa	J. Wen 6063-8	forthcoming	-
Dendropanax caucanus (Harms) Harms	Costa Rica, Heredia	J. Wen 6928	forthcoming	-
Dendropanax chevalieri (R.Vig.) Merr.	Vietnam, Lao Cai Prov.	J. Wen 10844*	SRS13357393	-
Dendropanax cordifolius Britton	Jamaica, St. Thomas, Golden Grove	J. Wen 11910	forthcoming	-
Dendropanax cuneatus (DC.) Decne. & Planch.	Bolivia, Santa Cruz, 4.5 Km SSE of Buena Vista	M. H. Nee 53800	forthcoming	-
Dendropanax cuneifolius (C.Wright ex Griseb.) Seem.	Cuba, Pinar del Rio, La Palma	J. L. Clark 10620	forthcoming	US3558179
Dendropanax dentigerus (Harms) Merr.	China, Fujian, Wuyishan Shi	J. Wen 12034	forthcoming	-
Dendropanax filipes Britton		J. Wen 11896	forthcoming	-
Dendropanax globosus M.J.Cannon & Cannon	Costa Rica, Puntarenas, Santa Elena Cloud Forest Reserve	J. Wen 6848-2	forthcoming	-
Dendropanax gonatopodus (Donn.Sm.) A.C.Sm.	Costa Rica, Heredia	J. Wen 6931	forthcoming	-
Dendropanax hainanensis (Merr. & Chun) Chun	-	Y. F. Deng 16240	forthcoming	-
Dendropanax lancifolius (Marchal ex Urb.) R.C.Schneid.	Malaysia, Luzon, Cameroon Highlands	J. Wen 8362	forthcoming	-
Dendropanax latilobus M.J.Cannon & Cannon	Costa Rica, Puntarenas, Reserva Biologica Monteverde.	J. Wen 6834-6	forthcoming	-
Dendropanax macropodus (Harms) Harms	Bolivia, Chapare, Cochabamba	M .H. Nee 53970	forthcoming	-
Dendropanax maingayi King	Malaysia, Pahang, Luzon	J. Wen 8364	forthcoming	-
Dendropanax nebulosus Fiaschi & Jung-Mend.	Brazil, Rio de Janeiro, Itatiaia	P. Fiaschi 3910	forthcoming	US3677101
Dendropanax nutans (Sw.) Decne. & Planch.	Jamaica, St. Thomas	J. Wen 11876	forthcoming	-
Dendropanax oliganthus (A.C.Sm.) A.C.Sm.	Mexico, Oaxaca, Candalaria Loxicha	J. Wen 8692	forthcoming	-

Dendropanax oligodontus Merr. & Chun	China	J. Wen 6603-3	forthcoming	-
Dendropanax pallidus M.J.Cannon & Cannon	Mexico, Chiapas, Mpio. Ocosingo	J. Wen 8728	forthcoming	-
Dendropanax palustris (Ducke) Harms	-	P. Fine 1006	forthcoming	-
Dendropanax pendulus (Sw.) Decne. & Planch.	Jamaica, Clarendon, Peckham Woods	J. Wen 11893	forthcoming	-
Dendropanax poilanei Bui	Vietnam	J. Wen 11024	forthcoming	-
Dendropanax praestans Standl.	Costa Rica, San Jose, Santa Maria de Dota	J. Wen 6889	forthcoming	US3503739
Dendropanax proteus (Champ. ex Benth.) Benth.	China, Guangdong, Yangshan Xian	J. Wen 11415	forthcoming	-
Dendropanax resinosus (Marchal) Frodin	Brazil	P. Fiaschi 3240	forthcoming	-
Dendropanax sessiliflorus (Standl. & A.C.Sm.) A.C.Sm.	Costa Rica, Puntarenas, Las Tablas	J. Wen 6978-10	forthcoming	-
Dendropanax sp. nov.	Bolivia, Santa Cruz, Buena Vista	M. H. Nee 53767	forthcoming	-
Dendropanax sp. nov.	Costa Rica	J. Wen 6891-18	forthcoming	-
Dendropanax swartzii (Fawc. & Rendle) A.C.Sm.	Jamaica, St. Andrew, Hollywell National Park	J. Wen 11871	forthcoming	-
Dendropanax trilobus (Gardner) Seem.	Brazil, Rio de Janeiro, Teresopolis	P. Fiaschi 3885	forthcoming	US3678700
Dendropanax umbellatus (Ruiz & Pav.) J.F.Macbr.	Peru, Huampal	J. Wen 8617	forthcoming	-
Dendropanax weberbaueri (Harms) Harms	Peru, Huanaco, Honoria	V. J. Schunke 1804	forthcoming	US2865130
Didymopanax angustissimus Marchal	Brazil, Rio de Janeiro, Teresopolis	C. Baez 1167	forthcoming	US3713965
Didymopanax morototoni (Aubl.) Decne. & Planch.	Bolivia, Cochabamba	M. H. Nee 53964	forthcoming	-
Didymopanax vinosus (Cham. & Schltdl.) Marchal	Brazil, Bahia, Rio de Contas	S. A. Mori 12452	forthcoming	US2856970
Eleutherococcus lasiogyne (Harms) S.Y.Hu	China	B. G. Marburg	forthcoming	-
Eleutherococcus nodiflorus	China, Zhejiang, Qingyuan Xian	J. Wen 11269	forthcoming	-
Eleutherococcus sessiliflorus (Dunn) S.Y.Hu	Russia, Moscow, Botanical Garden of Moscow State University	J. Wen 10378	forthcoming	-
Eleutherococcus simonii Simon-Louis ex Mouill.	China, Yunnan, Mengla Xian	J. Wen 15950	forthcoming	-
Eleutherococcus spinosus (L.f.) S.Y.Hu	Japan	J. Wen 5672	forthcoming	-
Eleutherococcus stenophyllus (Harms) Nakai	China, Shanxi, Lishan Nature Preserve	J. Wen 12850	forthcoming	-

Eleutherococcus trifoliatus (L.) S.Y.Hu	China, Hubei, Changyang Xian	J. Wen 14527*	SRS13357394	-
Fatsia japonica (Thunb.) Decne. & Planch.	China, Zhejiang, Hangzhou Botanical Garden	J. Wen 11148*	SRS13357395	-
Fatsia oligocarpella Koidz.	Japan	H. Kato 030041	forthcoming	-
Fatsia polycarpa Hayata	Taiwan, Formosa, Taoyuan Hsiang	J. Wen 9391*	SRS13357396	-
Frodinia gleasonii (Britton & P.Wilson) Lowry & G.M.Plunkett	Puerto Rico, Jayuya, Cerro de Punta	E. L. Little 21909	forthcoming	US2705307
Gamblea ciliate C.B.Clarke	China, Hunan, Luohandong	J. Wen 9334*	SRS13357397	-
Gamblea innovans (Siebold & Zucc.) C.B.Shang, Lowry & Frodin	Japan, Honshu	Soejima 1094*	SRS13357400	-
Gamblea malayana (M.R.Hend.) C.B.Shang, Lowry & Frodin	Malaysia, Pahang, Luzon	J. Wen 8361	forthcoming	-
Gamblea pseudoevodiifolia (K.M.Feng) C.B.Shang, Lowry & Frodin	Vietnam, Lao Cai Prov.	J. Wen 10850*	SRS13357399	-
Harmsiopanax aculeatus (Blume) Warb. ex Boerl.	Indonesia, Java, Cibodas Botanical Garden area	J. Wen 10130*	SRS13357401	-
Harmsiopanax ingens Philipson	Papua New Guinea, Morobe Province, Mt. Kolorong area	J. Wen 12309*	SRS13357402	-
Hedera algeriensis Rantonnet ex C.Morren	Tunisia, Ayn Darahim	J.J. Aldasoro A2890-3	forthcoming	-
Hedera azorica Carrière	Portugal, Faial	V. Fandos s.n.	forthcoming	-
Hedera canariensis Willd.	Spain, La Palma, La Galga	V. Valcárcel 66VV04(10)	forthcoming	-
Hedera colchica (K.Koch) K.Koch	Russia, Krasnodar	J. Wen 10358	forthcoming	-
Hedera helix L.	Spain, Malaga, Ronda	V. Valcárcel 04VV20(1)	forthcoming	-
Hedera hibernica (G. Kirchn.) Bean	Spain, Cordoba, Trasierra	V. Valcárcel 11VV20(1)	forthcoming	-
Hedera iberica (McAll.) Ackerf. & J.Wen	Spain, Aracena, Fuenteheridos	V. Valcárcel 11VV18(1)	forthcoming	-
Hedera maderensis K. Koch ex A. Rutherford	Portugal, Madeira, between Encumeada and Sao Vicente	V. Valcárcel 06VV19(4)	forthcoming	-
Hedera maroccana McAll.	Morocco, Azilal	P. Vargas 67PV05(1)	forthcoming	-
Hedera nepalensis K. Koch subsp. *nepalensis*	-	J. Wen 5796	forthcoming	-
Hedera nepalensis K. Koch subsp. *sinensis*	China, Taiwan, Kagi	J. Wen 10899	forthcoming	-
Hedera pastuchovii Woronow subsp. *cypria*	Cyprus, Platres	V. Valcárcel 12VV05(1)	forthcoming	-
Hedera rhombea (Miq.) Paul	South Korea, South Gyengsang, Geoje Island	O. Gyeongnam s.n.	forthcoming	-

Heptapleurum altigenum (Frodin) Lowry & G.M.Plunkett	Indonesia, Papua, lake Habbema	L. J. Brass 9091†	SRS6106606	ZQ108
Heptapleurum aromaticum (Blume) Boerl.	Indonesia, Java, Cibodas	J. Wen 10682	forthcoming	-
Heptapleurum bougainvilleanum (Harms) Lowry & G.M.Plunkett	Indonesia, Biak	T. M. A. Utteridge 676†	SRS6106553	ZQ035
Heptapleurum bractescens (Ridl.) Lowry & G.M.Plunkett	Indonesia, Papua	A. N. Millar NGF35390A†	SRS6106578	ZQ263
Heptapleurum calyptratum (Hook.f. & Thomson) Y.F.Deng	Vietnam, Lam Dong Prov.	J. Wen 11061*	SRS13357437	-
Heptapleurum delavayi Franch.	China, Sichuan, Mt. Omei	J. Wen 12106*	SRS13357428	-
Heptapleurum forbesii (Ridl.) Lowry & G.M.Plunkett	China, Sichuan, Mt. Omei	J. Wen 12130*	SRS13357430	-
Heptapleurum heptaphyllum (L.) Y.F.Deng	China, Guangdong, Wutongshan	J. Wen 12816*	SRS13357429	-
Heptapleurum heterophyllum (Wall. ex G.Don) Seem.	Malaysia, Selangor, Luzon	J. Wen 8392	forthcoming	-
Heptapleurum ischnoacrum (Harms) Lowry & G.M.Plunkett	Papua New Guinea, Eastern Highlands	J. Wen 12350*	SRS13357432	-
Heptapleurum kornasii (Grushv. & Skvortsova) Lowry & G.M.Plunkett	Vietnam, Lam Dong Prov.	J. Wen 11045*	SRS13357433	-
Heptapleurum minutistellatum (Merr. ex H.L.Li) Y.F.Deng	China, Guangdong, Nankunshan	J. Wen 13291*	SRS13357434	-
Heptapleurum oligodon (Harms) G.M.Plunkett & Lowry	Indonesia, Papua, Mimika Regency	J. H. Beaman 12242†	SRS6106621	-
Heptapleurum pachyphlebium (Merr.) G.M.Plunkett & Lowry	Malaysia, Borneo, Mt. Kinabalu National Park	J. Wen 11692	forthcoming	-
Heptapleurum papuanum (Ridl.) G.M.Plunkett & Lowry	Indonesia, Papua, Mimika Regency	P. J. Edwards 4356†	SRS6106591	-
Heptapleurum pilematophorum (Harms) G.M.Plunkett & Lowry	Papua New Guinea, Morobe, Salawaket range	R. D. Hoogland 9777†	SRS6106634	-
Heptapleurum porphyrantherum (Ridl.) G.M.Plunkett & Lowry	Indonesia, Papua, Mimika Regency	P. J. Rudall 56†	SRS6106592	-
Heptapleurum rugosum (Blume) Boerl.	Indonesia, West Java Province, Bogor Botanical Garden	J. Wen 10158*	SRS13357439	-
Heptapleurum scandens (Blume) Seem.	Indonesia, Java, Cibodas Botanical Garden area	J. Wen 10128*	SRS13357438	-
Heptapleurum setulosum (Harms) G.M.Plunkett & Lowry	Papua New Guinea, Enga, Lagaip Porgera	T. Mala UPNG8684†	SRS6106561	-
Heptapleurum wardii (C.Marquand & Airy Shaw) G.M.Plunkett & Lowry	China, Tibet, Xizang Province	J. Wen 9224*	SRS13357440	-

Heptapleurun petelotii (Merr.) G.M.Plunkett & Lowry	Vietnam, Ninh Binh Prov.	J. Wen 10946*	SRS13357436	-
Heteropanax brevipedicellatus H.L.Li	China, Guangdong, Nankunshan	J. Wen 13266*	SRS13357404	-
Heteropanax fragrans (Roxb.) Seem.	Vietnam, Ninh Binh Prov.	J. Wen 10905*	SRS13357403	-
Hydrocotyle cf. *nepalensis* Hook.	China, Taiwan, Taoyuan Hsiang	J. Wen 9401*	SRS13357405	-
Hydrocotyle umbellata L.	-	-‡	ERS1829705	-
Kalopanax septemlobus (Thunb.) Koidz.	China, Hubei, Yichang Shi	J. Wen 14561*	SRS13357406	-
Mackinlaya schlechteri (Harms) Philipson	Papua New Guinea, Eastern Highlands	J. Wen 12331*	SRS13357410	-
Macropanax chienii G.Hoo	China, Yunnan, Xishuangbanna	J. Wen 8473	forthcoming	-
Macropanax dispermus (Blume) Kuntze	Indonesia, Bali, Gunong Batukau	J. Wen 12422*	SRS13357407	-
Macropanax maingayi (C.B.Clarke) Philipson	Malaysia, Selangor, Luzon	J. Wen 8355	forthcoming	-
Macropanax rosthornii (Harms) C.Y.Wu ex G.Hoo	China, Sichuan, Mt. Emei	J. Wen 12176	forthcoming	-
Macropanax serratifolius K.M.Feng & Y.R.Li	China, Yunnan, Jingping Xian	J. Wen 10530	forthcoming	-
Macropanax undulatus (Wall. ex G.Don) Seem.	China, Yunnan, Tiantou Chun.	J. Wen 10569*	SRS13357408	-
Merrilliopanax listeri (King) H.L.Li	China, Yunnan, Gaoligong Mountains	J. Wen 6326-4*	SRS13357411	-
Merrilliopanax membranifolius	China, Yunnan, Gongshan Xian	Gaoligong Shan Expedition 13781	forthcoming	US3513065
Meryta pastoralis (W.W.Sm.) C.B.Shang	French Polynesia, Marchesas Islands, Feani	S. P. Perlman 18336	forthcoming	US3452030
Metapanax davidii Franch.) J.Wen & Frodin	Thailand, Chiang Mai, Chiang Dao Dist.	J. Wen 5018	forthcoming	-
Metapanax delavayi (Franch.) J.Wen & Frodin	China, Yunnan, Qianjia Cong	J. Wen 9146*	SRS13357412	-
Oplopanax elatus (Nakai) Nakai	-	J. Wen 5407-1	forthcoming	-
Oplopanax horridus (Sm.) Miq.	USA, Alaska	Taylor s.n.	forthcoming	-
Oreopanax anomalus M.J.Cannon & Cannon	Costa Rica, Puntarenas, Estacion Biologica Monteverde	J. Wen 6825	forthcoming	US3498433
Oreopanax capitatus (Jacq.) Decne. & Planch.	-	J. Wen 6284	forthcoming	-
Oreopanax cf. *argentatus* (Kunth) Decne. & Planch.	Peru, Pasco, Oxapampa	J. Wen 8597	forthcoming	-
Oreopanax cf. *artocarpoides* Standl.	Bolivia, Cochabamba	M. H. Nee 53913	forthcoming	-

Oreopanax cf. *membranaceus* Rusby	Bolivia, La Paz, Valle de Zongo	M. H. Nee 53938	forthcoming	-
Oreopanax cf. *trollii* Harms	Bolivia, Cochabamba	M. H. Nee 53921	forthcoming	-
Oreopanax cf. *williamsii* Harms	Peru, Pozuzo	J. Wen 8615	forthcoming	-
Oreopanax divulsus Marchal	Peru, Esperanza	J. Wen 8598	forthcoming	-
Oreopanax donnell-smithii Standl.	Costa Rica, San Jose, Canton de Perez Zeledon	J. Wen 6951	forthcoming	-
Oreopanax eriocephalus Harms		J. Wen 8610	forthcoming	-
Oreopanax guatemalensis (Lem. ex Bosse) Decne. & Planch. ex Witte	Costa Rica, Puntarenas, Estacion Biologica Monteverde	J. Wen 6822	forthcoming	-
Oreopanax iodophyllus Harms	Peru, Esperanza	J. Wen 8607	forthcoming	-
Oreopanax kuntzei Harms	Bolivia, Cochabamba, Río Ivirizu	J. Wen 53910*	SRS13357413	-
Oreopanax macrocephalus Decne. & Planch. ex Wedd.	Bolivia, Cochabamba, Dept. Cochabamba, Monte Punco	J. Wen 53909*	SRS13357414	-
Oreopanax nicaraguensis M.J.Cannon & Cannon	Costa Rica, Puntarenas, 1km west of San Rafael	J. Wen 6958-2	forthcoming	-
Oreopanax nubigenus Standl.	Costa Rica, Puntarenas, Estacion Biologica Monteverde	J. Wen 6832-1	forthcoming	-
Oreopanax oerstedianus Marchal	Costa Rica, Puntarenas, Estacion Biologica Monteverde	J. Wen 6813	forthcoming	-
Oreopanax pavonii Seem.	Peru, Chachos	J. Wen 8588	forthcoming	-
Oreopanax peltatus Linden ex Regel	Mexico, Oaxaca, San Felipe	J. Wen 8682	forthcoming	-
Oreopanax platanifolius (Willd. ex Schult.) Decne. & Planch.	Peru, Esperanza	J. Wen 8606	forthcoming	-
Oreopanax polycephalus Harms	Peru, Chacos	J. Wen 8576	forthcoming	-
Oreopanax pycnocarpus Donn.Sm.	Costa Rica, San Jose, Canton de Perez Zeledon	J. Wen 7023	forthcoming	-
Oreopanax rusbyi Britton	Bolivia, Cochabamba	M. H. Nee 53928	forthcoming	-
Oreopanax sanderianus Hemsl.	Mexico, Oaxaca, El Porvenir	J. Wen 8691*	SRS13357415	-
Oreopanax sp	Mexico	J. Wen 12338*	SRS13357416	-
Oreopanax steinbachianus Harms	Bolivia, Cochabamba	M. H. Nee 53946	forthcoming	-
Oreopanax thaumasiophyllus Harms	Bolivia, La Paz, Valle de Zongo	M. H. Nee 53934	forthcoming	-
Oreopanax vestitus Marchal	Costa Rica, Puntarenas, Estacion Biologica Monteverde	J. Wen 6823	forthcoming	-
Oreopanax xalapensis (Kunth) Decne. & Planch.	Mexico, Oaxaca	J. Wen 8689*	SRS13357417	-
Osmoxylon boerlagei (Warb.) Philipson	Papua New Guinea, Morobe, Salamaua	J. Wen 12274	forthcoming	-
Osmoxylon micranthum (Harms) Philipson	Papua New Guinea, Eastern Highlands	J. Wen 12341*	SRS13357418	-
Osmoxylon novoguineense (Scheff.) Becc.	Indonesia, Irian Barat, Keerom	J. Wen 10706*	SRS13357419	-
Panax assamicus	-	J. Wen s.n.	forthcoming	-

R.N.Banerjee				
Panax bipinnatifidus Seem.	-	J. Wen 5728-6	forthcoming	-
Panax elegantior (Burk.) Hu	-	X. G. Sun 20010455	forthcoming	-
Panax ginseng C.A.Mey.	-	J. Wen 5400-2	forthcoming	-
Panax omeiensis J. Wen	China, Sichuan, Mt. Omei	J. Wen 12095-3	forthcoming	-
Panax quinquefolius L.	-	J. Wen 6244-16	forthcoming	-
Panax trifolius L.	-	J. Wen 627	forthcoming	-
Panax variabilis	-	J. Wen 6525-6	forthcoming	-
Panax vietnamensis Ha & Grushv.	Vietnam, Lam Dong Prov.	J. Liu 49*	SRS13357421	-
Panax wangianus S.C.Sun	China, Sichuan, Mt. Omei	J. Wen 12167*	SRS13357422	-
Polyscias australiana (F.Muell.) Philipson	Indonesia, Irian Barat, Keerom	J. Wen 10710*	SRS13357424	-
Polyscias baehniana (Bernardi) Bernardi	Madagascar, Ihorombe, Ilempo	J. Wen 9512	forthcoming	-
Polyscias boivinii (Seem.) Bernardi	Madagascar, Antsiranana, Montagne des Francais	J. Wen 9633*	SRS13357425	-
Polyscias diversifolia (Blume) Lowry & G.M.Plunkett	Malaysia, Perak, Luzon	J. Wen 8372	forthcoming	-
Polyscias elliptica (Blume) Lowry & G.M.Plunkett	Indonesia, West Java Province, Bogor Botanical Garden	J. Wen 10157*	SRS13357409	-
Polyscias fruticosa (L.) Harms	-	‡	ERS1829704	-
Polyscias oahuensis (A.Gray) Lowry & G.M.Plunkett	United States, Hawaii, Oahu	J. Wen 7073	forthcoming	-
Polyscias samoensis (A.Gray) Harms	United States, Hawaii, Oahu	J. Wen 7078	forthcoming	-
Polyscias sandwicensis (A.Gray) Lowry & G.M.Plunkett	United States, Hawaii, Oahu	J. Wen 7061	forthcoming	-
Polyscias schultzei Harms	Indonesia, Papua, District Jayamijaja	J. Wen 10730	forthcoming	-
Pseudopanax colensoi (Hook.f.) K.Koch	New Zealand, near Arthur´s Pass	L. Bernardi 12297	forthcoming	US2749593
Pseudopanax crassifolius (Sol. ex A.Cunn.) K.Koch	New Zealand, Auckland, cultivated in Ross Michie's garden	E. H. Walker 5321	forthcoming	US1994489
Pseudopanax laetevirens (Gay) Baill.	Chile originally, cult.	J. Wen 2019306*	SRS13357426	-
Pseudopanax lessonii (DC.) K.Koch	New Zealand, Auckland, Woodhill Forest	L. Bernardi 12376	forthcoming	US2749585
Pseudopanax valdiviensis (Gay) Baill.	Thailand, Chiang Mai, Doi Sutep	J. Wen 7448-2	forthcoming	-
Raukaua anomalus (Hook.) A.D.Mitch., Frodin & Heads	New Zealand	A. Mitchell 527931	forthcoming	-

Raukaua simplex (G.Forst.) A.D.Mitch., Frodin & Heads	New Zealand, near Arthur´s Pass	L. Bernardi 12284	forthcoming	US2749586
Schefflera digitata J.R.Forst. & G.Forst.	New Zealand	Steward Oliver s.n.	forthcoming	-
Sciodaphyllum acuminatum Pav. (Poir.)	Peru, Chacos	J. Wen 8575	forthcoming	-
Sciodaphyllum angulatum Pav. (Poir.)	Peru, Oxapampa	J. Wen 8589*	SRS13357427	-
Sciodaphyllum brownei Spreng.	Jamaica, St. Andrew, Holly Well National Parl	J. Wen 11867	forthcoming	-
Sciodaphyllum chartaceum A.C.Sm.	Costa Rica, Puntarenas, Canton de Golfito	J. Wen 6998	forthcoming	-
Sciodaphyllum herzogii (Harms) Lowry, G.M.Plunkett & M.M.Mora	Bolivia, Cochabamba	M. H. Nee 53951	forthcoming	-
Sciodaphyllum pedicellatum Pav. (Poir.)	Peru, Villa Rica	J. Wen 8645	forthcoming	-
Sciodaphyllum pentandrum Pav. (Poir.)	Peru, near Huampal	J. Wen 8619	forthcoming	-
Sciodaphyllum robustum A.C.Sm.	Costa Rica, Gucanaste, Cordillera de Tilaran	J. Wen 6869	forthcoming	-
Sciodaphyllum sodiroi (Harms) Lowry, G.M.Plunkett & M.M.Mora	Ecuador, Pichincha, Mejia	J. L. Clark 9466	forthcoming	-
Sinopanax formosanus (Hayata) H.L.Li	China, Taiwan, Taoyuan Hsiang	J. Wen 9395*	SRS13357441	-
Tetrapanax papyrifer (Hook.) K.Koch	China, Zhejiang, Qingyuan Xian, Wudabao Xiang, Honguang Village	J. Wen 11233*	SRS13357442	-
Trachymene glaucifolia (F.Muell.) Benth.	Australia, Northern Territory, Yuendumu	H. S. McKee 8598	forthcoming	US2380287
Trevesia burckii Boerl.	-	J. Wen 15036	forthcoming	-
Trevesia lateospina Jebb	Thailand, Lampang, Muang Bahn District	J. Wen 7480-3	forthcoming	-
Trevesia palmata (Roxb. ex Lindl.) Vis.	China, Yunnan, Mengla Xian	J. Wen 15943	forthcoming	-
Trevesia sundaica Miq.	Indonesia, West Java Province, Bogor Botanical Garden	J. Wen 10162*	SRS13357445	-
Trevesia valida Craib	-	Maxwell 06-26	forthcoming	-

*Samples obtained from Gallego-Narbón et al. (2022) †Samples obtained from Shee et al. (2020) †‡Samples obtained from OneKP project
