# Supplementary material for: Ancient polyploidization events influence the evolution of the ginseng family (Araliaceae)
Source: Front Plant Sci. 2025 Jun 13;16:1595321. doi: 10.3389/fpls.2025.1595321 (PMC12202383; doi:10.3389/fpls.2025.1595321)
Supplement: Supplementary file 2 [file DataSheet2.pdf]

Supplementary Data 2. Compilation of chromosome counts of the Araliaceae family. Basic chromosome counts (*n*) are provided. The proportion of chromosome counts are indicated for each genus.

**Species**	***n***	**Reference**
***Aralia* L.**	**12 (0.75), 24 (0.25)**	
Aralia cachemirica Decne.	24[†]	Wanscher (1933)
Aralia californica S.Watson	24[†]	Bowden (1945)
Aralia chinensis L.	12[†‡]	Wu (1995)
Aralia continentalis Kitag.	24[†‡]	Gurzenkov *et al.* (1998)
Aralia cordata Thunb.	24[†‡]	Probatova & Sokolovskaya (1986)
Aralia elata (Miq.) Seem.	12[†‡]	Fedorov (1974)
Aralia hispida Vent.	12[†‡]	Fedorov (1974)
Aralia nudicaulis L.	12[†‡], 24	Bowden (1945), Wen (2024)
Aralia parasitica (D.Don) Buch.-Ham. Ex Bosse	12[†]	Yi et al (2004)
Aralia racemosa L.	12[†‡]	Love & Love (1982)
Aralia spinosa L.	12[†]	Darlington & Wylie (1955)
Aralia wangshanensis (W.C.Cheng) Y.F.Deng	12[§]	Yi *et al.* (2004)
***Brassaiopsis* Decne. & Planch.**	**24 (1.0)**	
Brassaiopsis ciliata Dunn	24[†]	Yi *et al.* (2004)
Brassaiopsis hainla (Buch.-Ham.) Seem.	24[†]	Yi *et al.* (2004)
Brassaiopsis shweliensis W.W.Sm.	24[§]	Yi *et al.* (2004)
***Cheirodendron* Nutt. ex Seem.**	**12 (1.0)**	
Cheirodendron trigynum (Gaudich.) A.Heller	12[†]	Fedorov (1974)
***Chengiopanax* C.B.Shang & J.Y.Huang**	**24 (1.0)**	
Chengiopanax sciadophylloides (Franch. & Sav.) C.B.Shang & J.Y.Huang	24[§]	Kawatani & Ohno (1971)
Eleutherococcus Maxim.	**24 (1.0)**	
Eleutherococcus divaricatus (Siebold & Zucc.) S.Y.Hu	24[§]	Yi *et al.* (2004)
Eleutherococcus giraldii (Harms) Nakai	24[§]	Yi *et al.* (2004)

Eleutherococcus hypoleucus (Makino) Nakai	24§	Yi *et al.* (2004)
Eleutherococcus japonicus (Franch. & Sav.) Nakai	24§	Yi *et al.* (2004)
Eleutherococcus koreanus Nakai	24†	Sun *et al.* (1988)
Eleutherococcus lasiogyne (Harms) S.Y.Hu	24†	Yi *et al.* (2004)
Eleutherococcus nikaianus (Koidz. ex Nakai) H.Ohashi	24§	Yi *et al.* (2004)
Eleutherococcus senticosus (Rupr. & Maxim.) Maxim.	24†	Kawatani & Ohno (1971)
Eleutherococcus sessiliflorus (Rupr. & Maxim.) S.Y.Hu	24†	Starodubtsev (1984)
Eleutherococcus sieboldianus (Makino) Koidz.	24†	Kawatani & Ohno (1971)
Eleutherococcus trichodon (Franch. & Sav.) H.Ohashi	24§	Yi *et al.* (2004
Eleutherococcus trifoliatus (L.) S.Y.Hu	24†	Wanscher (1933)
***Fatsia* Decne. & Planch.**	**24 (0.75), 12 (0.25)**	
Fatsia japonica (Thunb.) Decne. & Planch.	12,24†‡	Sun *et al.* (1988, n=24); Tanaka (1974, n=48)
Fatsia oligocarpella Koidz.	24†‡	Ono (1975)
***Gamblea* C.B.Clarke**	**24 (1.0)**	
Gamblea ciliata C.B.Clarke in J.D.Hooker	24†‡	Mehra & Bawa (1969)
***Hedera* L.**	**24 (0.5), 72 (0.25),<br>48 (0.17), 96 (0.08)**	
Hedera algeriensis Rantonnet ex C.Morren	48†‡	Vargas *et al.* (1999)
Hedera azorica Carrière	24†‡	Vargas *et al.* (1999)
Hedera canariensis Willd.	24†‡	Vargas *et al.* (1999)
Hedera colchica (K.Koch) K.Koch	96†‡	Vargas *et al.* (1999)
Hedera cypria McAll.	72†‡	Vargas *et al.* (1999)
Hedera helix L.	24†‡	Vargas *et al.* (1999)
Hedera hibernica Poit.	48†‡	Jacobsen (1954)
Hedera maderensis K.Koch ex A.Rutherf.	72†‡	Vargas *et al.* (1999)
Hedera maroccana McAll.	24†‡	Vargas *et al.* (1999)
Hedera nepalensis K.Koch	24†‡	Singhal *et al*. (1980)
Hedera pastuchovii Woronow in A.A.Grossheim	72†‡	Vargas *et al.* (1999)
Hedera rhombea (Miq.) Paul	24†‡	Sun *et al.* (1988)

***Heptapleurum* Gaertn.**	**24 (0.83), 12 (0.17)**	
Heptapleurum arboricola Hayata	12[†]	Hsu (1968)
Heptapleurum delavayi Franch.	24[†]	Yi *et al.* (2004)
Heptapleurum farinosum (Blume) Lowry & G.M.Plunkett	24[§]	Yi *et al.* (2004)
Heptapleurum rhododendrifolium (Griff.) G.M.Plunkett & Lowry	24[†]	Mehra & Bawa (1969)
Heptapleurum schweliense (W.W.Sm.) G.M.Plunkett & Lowry	24[†]	Yi *et al. (*2004)
Heptapleurum venulosum (Wight & Arn.) Seem.	24[†‡]	Gil *et al.* (1979)
***Hydrocotyle* L.**	**24 (0.38), 12 (0.15), 9 (0.12), 36 (0.12), 48 (0.08), 30 (0.06), 16 (0.03), 18 (0.03), 72 (0.03)**	
Hydrocotyle algida A. Rich.	24[†‡]	Webb & Beuzenberg (1987)
Hydrocotyle americana L.	30[†‡]	Hair (1980)
Hydrocotyle asiatica L.	9[†‡]	Baquar (1967)
Hydrocotyle bonariensis Comm. ex Lam.	48[†]	Constance *et al.* (1971)
Hydrocotyle bowlesioides Mathias & Constance	24[†]	Constance *et al.* (1971)
Hydrocotyle conferta Wight	9[†‡]	Subramanian (1986)
Hydrocotyle dichondroides Makino	12[†‡]	Hsu (1968)
Hydrocotyle dissecta Hook. f.	24[†‡]	Hair (1980)
Hydrocotyle elongata A. Cunn. ex Hook.	24[†‡]	Hair (1980)
Hydrocotyle heteromeria A. Rich.	30[†‡]	Webb & Beuzenberg (1987)
Hydrocotyle himalaica P. K. Mukh.	36[†]	Chatterjee *et al.* (1989)
Hydrocotyle hirta R. Br. ex A. Rich.	12[†‡]	Renard *et al.* (1983)
Hydrocotyle humboldtii A. Rich.	24[†]	Constance *et al.* (1971)
Hydrocotyle hydrophila Petrie	72[†‡]	Webb & Beuzenberg (1987)
Hydrocotyle japonica Makino	48[†‡]	Byung-Yun *et al.* (1996)
Hydrocotyle javanica Thunb.	18[†‡]	Sharma & Sarkar (1967)
Hydrocotyle maritima Honda	36[†‡]	Iwatsubo *et al.* (2006)
Hydrocotyle microphylla A. Cunn.	24[†‡]	Hair (1980)

Hydrocotyle monticola Hook. f.	9[†‡]	Hedberg & Hedberg (1977)
Hydrocotyle moschata G. Forst.	24[†‡]	Hair (1980)
Hydrocotyle novae-zealandiae DC.	24[†‡]	Darlington & Wylie (1955)
Hydrocotyle poeppigii DC.	24[†]	Cave (1958)
Hydrocotyle pterocarpa F. Muell.	24[†‡]	Hair (1980)
Hydrocotyle ramiflora Maxim.	12[†‡]	Iwatsubo *et al.* (2006)
Hydrocotyle ranunculoides L.	24[†‡]	Constance *et al.* (1971)
Hydrocotyle ribifolia Rose & Standl.	16[†]	Constance *et al.* (1971)
Hydrocotyle rotundifolia Roxb.	9[†‡]	Sharma (1970)
Hydrocotyle sibthorpioides Lam.	12[†‡]	Constance *et al.* (1976)
Hydrocotyle steyermarkii Mathias & Constance	24[†]	Constance *et al.* (1971)
Hydrocotyle sulcata C. Webb & P. Johnson	36[†‡]	Webb & Beuzenberg (1987)
Hydrocotyle tripartita A. Rich.	12[†‡]	Webb & Beuzenberg (1987)
Hydrocotyle umbellata L.	24[†]	Darlington & Wylie (1955)
Hydrocotyle vulgaris L.	48[†‡]	Queiros (1978)
Hydrocotyle yabei Makino	36[‡]	Iwatsubo *et al.* (2006)
***Kalopanax* Miq.**	**24 (1.0)**	
Kalopanax septemlobus Miq.	24[†‡]	Starodubtsev (1984)
***Macropanax* Miq.**	**24 (1.0)**	
Macropanax dispermus (Blume) Kuntze	24[†‡]	Mehra & Bawa (1969)
***Merrilliopanax* H.L.Li**	**24 (1.0)**	
Merrilliopanax listeri (King) H.L.Li,	24[†]	Yi *et al.* (2004)
***Meryta* J.R.Forst & G.Forst**	**24 (1.0)**	
Meryta angustifolia (Endl.) Seem.	24[†]	de Lange & Murray (2003)
Meryta latifolia (Endl.) Seem.	24[†]	de Lange & Murray (2003)
Meryta sinclairii (Hook.f.) Seem.	24[†]	Beuzenberg (1983)
***Metapanax* J.Wen & Frodin**	**24 (1.0)**	
Metapanax davidii (Franch.) J.Wen & Frodin	24[†]	Yi *et al.* (2004)

Metapanax delavayi (Franch.) J.Wen & Frodin	24†	Yi *et al.* (2004)
***Oplopanax* Torr. & A.Gray) Miq.**	**24 (1.0)**	
Oplopanax elatus (Nakai) Nakai,	24†	Gurzenkov (1973)
Oplopanax horridus (Sm.) Miq.	24†	Reveal & Spellenberg (1976)
***Oreopanax* Decne. & Planch.**	**24 (1.0)**	
Oreopanax reticulatus (Willd. ex Schult.) Decne. & Planch.	24§	Yi *et al.* (2004)
***Panax* L.**	**12 (0.55), 24 (0.45)**	
Panax assamicus R.N.Banerjee	24§	Yi *et al.* (2004)
Panax bipinnatifidus Seem.	12†, 24§	Kurosawa (1971), Yi *et al.* (2004)
Panax ginseng C.A.Mey.	24†‡	Li *et al.* (1985)
Panax japonicus (T.Nees) C.A.Mey.	24§	Yi *et al.* (2004)
Panax notoginseng (Burkill) F.H.Chen	12†‡	Kondo *et al.* (1992)
Panax pseudoginseng Wall.	12†	Hara (1970)
Panax quinquefolius L.	24†‡	Blair (1975)
Panax stipuleanatus H.T.Tsai & K.M.Feng	12†	Yi *et al.* (2004)
Panax trifolius L.	12†‡	Hu *et al.* (1980)
***Polyscias* J.R.Forst. & G.Forst.**	**12 (0.78), 24 (0.22)**	
Polyscias australiana (F.Muell.) Philipson	12§	Yi *et al.* (2004)
Polyscias bisattenuata (Sherff) Lowry & G.M.Plunkett	24‡	Kiehn (2005)
Polyscias bracteata (R.Vig.) Lowry	12†	Yi *et al.* (2004)
Polyscias crenata (Pancher & Sebert) Frodin	12†	Yi *et al.* (2004)
Polyscias dioica (Vieill. ex Pancher & Sebert) Harms	12†	Yi *et al.* (2004)
Polyscias fruticosa (L.) Harms	12†	Wanscher (1933)
Polyscias guilfoylei (W.Bull) L.H.Bailey	12†	Stone & Loo (1969)
Polyscias oahuensis (A.Gray) Lowry & G.M.Plunkett	24§	Yi *et al.* (2004)
Polyscias otopyrena (Baill.) Lowry & G.M.Plunkett	12§	Yi *et al.* (2004)
Polyscias pancheri (Baill.) Harms	12†	Yi *et al.* (2004)
Polyscias racemosa (C.N.Forbes) Lowry & G.M.Plunkett	24†	Kiehn (1996)

Polyscias scopoliae (Baill.) Lowry	12†	Yi *et al.* (2004)
Polyscias scutellaria (Burm.f.) Fosberg	12†	Stone & Loo (1969)
Polyscias vieillardii (Baill.) Lowry & G.M.Plunkett	12†	Yi *et al.* (2004)
**New Zealander *Pseudopanax***	**24 (1.0)**	
Pseudopanax arboreus (L.f) K.Koch	24†	Beuzenberg (1983)
Pseudopanax chathamicus Kirk	24†	de Lange *et al.* (2004)
Pseudopanax crassifolius (Sol. ex. A.Cunn.) K.Koch	24†	Beuzenberg (1983)
Pseudopanax discolor (Kirk) Harms	24†	Beuzenberg (1983)
Pseudopanax ferox Kirk	24†	Beuzenberg (1983)
Pseudopanax gilliesii Kirk	24†	de Lange *et al.* (2004)
Pseudopanax lessonii (DC.) K.Koch	24†	Cave (1958)
Pseudopanax linearis (Hook.f.) K.Koch	24†	de Lange *et al.* (2004)
***Raukaua* Seem.**	**12 (1.0)**	
Raukaua anomalus (Hook.) A.D.Mitch.	12†	Cave (1958)
Raukaua edgerleyi (Hook.f.) Seem.	12†	Cave (1958)
Raukaua simplex (G.Forst.) A.D.Mitch.	12†	Cave (1958)
***Schefflera* J.R.Forst. & G.Forst.**	**12 (1.0)**	
Schefflera candelabrum Baill.	12†	Yi *et al.* (2004)
Schefflera digitata J.R.Forst. & G.Forst.	12†	Beuzenberg (1983)
Tetrapanax (K.Koch) K.Koch	**24 (1.0)**	
Tetrapanax papyrifer (Hook.) K.Koch	24†	Cave (1962)
***Trachymene* Rudge**	**22(0.55), 11(0.27), 66(0.09), 44(0.09)**	
Trachymene adenodes Buwalda	22†	Constance *et al.* (1971)
Trachymene arfakensis (Gibbs) Buwalda	22†	Constance *et al.* (1971)
Trachymene anisocarpa (Turcz.) B.L.Burtt	66†, 44§	Constance *et al.* (1971), Wanscher (1933)
Trachymene coerulea Graham	11†	Wanscher (1933)
Trachymene cyanantha Boyland	11†	Henderson (1973)
Trachymene humilis (Hook.f.) Benth.	22†	

Trachymene pilbarensis Rye	11†	Rye (1999)
Trachymene pilosa Sm.	22†	Wanscher (1933)
Trachymene scapigera (Domin) B.L.Burtt	22§	Wanscher (1933)
Trachymene tripartita Hoogland	22†	Fedorov (1974)

†Chromosome Counts Database (CCDB; Rice et al., 2014)

‡Index to Plant Chromosome Numbers database (IPCN; Goldblatt & Johnson 2006)

§Yi et al., (2004)

References

Baquar SR. 1967-68. Chromosome numbers in some vascular plants of East Pakistan. *Rev. Biol.* 6: 440-448.

Beuzenberg EJ. 1983. Contributions to a chromosome atlas of the New Zealand flora - 24 Coprosma (Rubiaceae). *New Zealand Journal of Botany* 21: 9–12.

Blair A. 1975. Karyotype of five plant species with disjunct distributions in Virginia and the Carolinas. *American Journal of Botany* 62: 833–837.

Byung-Yun S, Park JH, Kwak MJ, Kim CH, Kim KS. 1996. Chromosome counts from the flora of Korea with emphasis on Apiaceae. *Journal of Plant Biology* 39: 15–22.

Bowden WM. 1945. A list of chromosome numbers in higher plants I. Acanthaceae to Myrtaceae. *Americal Journal of Botany* 32: 81–92.

Cave MS. 1958. *Index to Plant Chromosome Numbers for 1957*. Berkeley: California Botanical Society.

Cave MS. 1963. *Index to Plant Chromosome Numbers for 1962*. Berkeley: California Botanical Society.

Chatterjee A, Ghosh S, Roy SC. 1989. A cytological survey of eastern Himalayan plants III. *Cell Chromosome Research*. 12: 22–29.

Constance L, Chuang T-I, Bell CR. 1971. Chromosome numbers in Umbelliferae. IV. *American. Journal of Botany* 58: 577–587.

Constance L, Chuang T-I, Bell CR. 1976. Chromosome numbers in Umbelliferae. V. *American. Journal of Botany* 63(5): 608–625.

Darlington CD, Wylie AP. 1955. *Chromosome atlas of flowering plants*. London, UK: George Allen and Unwin Ltd.

De Lange PJ, Murray BG. 2003. Chromosome numbers of Norfolk Island endemic plants. *Australian Journal of Botany* 51: 211–215. Cccccccc

De Lange PJ, Murray BG, Datson PM. 2004. Contributions to a chromosome atlas of the New Zealand flora-38. Counts for 50 families. *New Zealand Journal of Botany* 42: 873–904.

Fedorov AA. 1974. *Chromosome numbers of flowering plants*. Leningrad: Academy of Sciences of the USSR. Komarov Botanical Institute (Reprint 1974).

Gill BS, Bir SS, Singhal VK. 1979. In IOPB chromosome number reports LXIV. *TAXON* 28: 403.

Gurzenkov NN. 1973. Studies of chromosome numbers of plants from the south of the Soviet Far East. *Komarov Lectures* 20: 47–61.

Gurzenkov NN, Starodubtsev VN, Kolyada AS. 1998. Karyological peculiarities of the Far East species of the family Araliaceae. In: *Rasteniya v Mussonnom Klimate*. Vladivostok. 121-123.

Hair JB. 1980. Contributions to a chromosome atlas of the New Zealand flora - 21 Umbelliferae (miscellaneous genera). *New Zealand Journal of Botany* 18: 559–562.

Hara H. 1970. On the Asiatic species of the genus *Panax*. *Journal of Japanese Botany* 45: 197–212.

Hedberg I, Hedberg O. 1977. Chromosome numbers of afroalpine and afromontane angiosperms. *Botaniska notiser* 130: 1–24.

Henderson RJF. 1973. In IOPB chromosome number reports XXXIX. *TAXON* 22: 115-118.

Hsu, CC. 1968. Preliminary chromosome studies on the vascular plants of Taiwan (II). *Taiwania* 14: 11–27.

Hu SY., Rudenberg L, Del Tredici P. 1980. Studies of American ginsengs. *Rhodora* 82: 627–636.

Iwatsubo Y, Matsuda M, Sasamura K, Naruhashi N. 2006. Chromosome numbers of six species of *Hydrocotyle* (Umbelliferae) in Japan. *Journal of Japanese Botany* 81: 262–267.

Jacobsen P. 1954. Chromosome numbers in the genus *Hedera* L. *Hereditas* 40: 252–254.

Kawatani T, Ohno T. 1971. Chromosome numbers of *Acanthopanax* growing in Japan. *Journal of Japanese Botany* 46: 349–350.

Kiehn M. 1996. Chromosome counts on angiosperms cultivated at the National Tropical Botanical Garden, Kaua'i, Hawai'i. *Pacific Science* 50: 317–323.

Kiehn M. 2005. Chromosome numbers of Hawaiian angiosperms: new records and comments. *Pacific Science* 59: 363–377.

Kondo K, Taniguchi K, Tanaka R, Gu Z. 1992. Karyomorphological studies in Chinese plant-species involving the Japanese floristic element, I. *The American Camellia Yearbook* 1992: 131–156.

Kurosawa S. 1971. Cytological studies on some eastern Himalayan plants and their related species. In: *The Flora of eastern Himalaya. Second report.* Tokyo: Univ. Tokyo Press. 355–364.

Li F-Y, Sun X, Gong X-C. 1985. The analysis of the chromosomal morphology and Giemsa C-banding pattern in Ginseng. *Scientia Agricultura Sinica* 5: 31–35.

Love A, Love D. 1982. In IOPB chromosome number reports LXXVI. *TAXON* 31: 583–587.

Mehra PN, Bawa KS. 1969. Chromosomal evolution in tropical hardwoods. *Evolution* 23: 466–481.

Ono M. 1975. Chromosome numbers of some endemic species of the Bonin Islands I. *Botanical Magazine (Tokyo)* 88: 323–328.

Probatova NS, Sokolovskaya AP. 1986. Chromosome numbers of the vascular plants from the far east of the USSR. *Botanicheskii Zhurnal SSSR* 71: 1572–1575.

Queiros M. 1978. Numeros cromosomicos para a flora Portuguesa 1-15. *Boletim da Sociedade Broteriana* 52: 69–77.

Renard R, Lambinon J, Reekmans M, Veken PV, Govaert M. 1983. Nombres chromosomiques de quelques Angiospermes du Rwanda, du Burundi et du Kenya. *Bulletin du Jardin Botanique National de Belgique* 53: 342–371.

Reveal JL, Spellenberg R. 1976. Miscellaneous chromosome counts of Western American plants - III. *Rhodora* 78: 37–52.

Rye BL. 1999. A taxonomic revision of the many-flowered species of *Trachymene* (Apiaceae) in western Australia. *Nuytsia* 13: 193–232.

Sharma AK, Sarkar AK. 1967. Chromosome number reports of plants in Annual Report, Cytogenetics Laboratory, Department of Botany, University of Calcutta. *The Research Bulletin* 2: 38–48.

Sharma AK. 1970. Annual report, 1967-1968. *Res. Bull. Univ. Calcutta Cytogenetics Lab*. 2: 1–50.

Singhal VK, Gill BS, Bir SS. 1980. In Chromosome number reports LXIX. *TAXON* 29: 712–713.

Stone BC, Loo AH. 1969. Cytotaxonomic notes on some species of *Polyscias* (Araliaceae). *Journal of Japanese Botany* 44: 321–327.

Subramanian D. 1986. Cytotaxonomical studies in south Indian Apiaceae. *Cytologia* 51: 479–488.

Sun B-Y, Kim C-H, Soh W-Y. 1988. Chromosome numbers of Araliaceae in Korea. *Korean Journal of Plant Taxonomy* 18: 291–296.

Starodubtsev VN. 1984. Chromosome numbers in the species of the flora of Primorye Region. *Botanicheskii Zhurnal* SSSR 69: 1565–1566.

Tanaka R. 1974. Organizational system of meiotic division and the development of reproductive cells in higher plants. *The Cell* 6: 22–25.

Wanscher JH. 1933. Studies on the chromosome numbers of Umbelliferae. III. *Botanisk Tidsskrift* 42: 384–399.

Vargas P, McAllister HA, Morton C, Jury SL, Wilkinson MJ. 1999. Polyploid speciation in *Hedera* (Araliaceae): phylogenetic and biogeographic insights based on chromosome counts and ITS sequences. *Plant Systematics and Evolution* 219: 165–179.

Webb CJ, Beuzenberg EJ. 1987. Contributions to a chromosome atlas of the New Zealand flora -- corrections and additions to number 21 Umbelliferae (*Hydrocotyle*). *New Zealand Journal of Botany* 25: 371–372.

Wen J. 2024. Araliaceae. In: *Flora of North America. Volume 13.* Oxford University Press.

Wu ZM. 1995. Cytological studies on some plants of woody flora in Huangshan, Anhui Province. *Journal of Wuhan Botanical Research* 13: 107–112.

Yi T, Lowry PP, Plunkett GM, Wen J. 2004. Chromosomal evolution in Araliaceae and close relatives. *TAXON* 53: 987–1005.
