# Supplementary material for: Ancient polyploidization events influence the evolution of the ginseng family (Araliaceae)
Source: Front Plant Sci. 2025 Jun 13;16:1595321. doi: 10.3389/fpls.2025.1595321 (PMC12202383; doi:10.3389/fpls.2025.1595321)
Supplement: Supplementary file 4 [file DataSheet4.pdf]

Supplementary Data 4. Parameters or nuclear (A) and plastid (B) ChromEvol models. The parameters not included in each model are marked with hyphens. λ, chromosome gain constant rate; λ_l, chromosome gain linear rate; δ, chromosome loss constant rate; δ_l, chromosome loss linear rate; ρ, whole genome duplication rate; μ, demi-duplication rate; β, monoploid chromosome number; ν, monoploid chromosome number addition rate. The best models appear in bold.

A)

Model	Log-likelihood	AIC	ΔAIC	λ	λ_l	δ	δ_l	ρ	μ	β	ν
**DysDup**	-17.1250	40.2499	NA	$1.81\cdot10^{-10}$	-	0.158942	-	0.400000	-	-	-
DysDupDem=	-18.8600	43.7199	3.4700	$1.81\cdot10^{-10}$	-	0.158241	-	0.190983	-	-	-
DysDupDem	-17.1250	42.2499	2.0000	$1.81\cdot10^{-10}$	-	0.158942	-	0.400000	$2.15\cdot10^{-10}$	-	-
Dys	-48.6737	101.3470	61.097	$1.81\cdot10^{-10}$	-	0.293080	-	-	-	-	-
Dys^{Linear}Dup	-17.0979	44.1958	3.9459	$1.81\cdot10^{-10}$	$2.05\cdot10^{-10}$	0.165368	$-7.4\cdot10^{-4}$	0.400000	-	-	-
Dys^{Linear}DupDem=	-18.8344	47.6688	7.4189	$1.81\cdot10^{-10}$	$1.80\cdot10^{-10}$	0.157493	$-7.0\cdot10^{-4}$	0.190983	-	-	-
Dys^{Linear}DupDem	-17.0936	46.1873	5.9374	$1.81\cdot10^{-10}$	$2.05\cdot10^{-10}$	0.166416	$-7.8\cdot10^{-10}$	0.420843	$2.15\cdot10^{-10}$	-	-
Dys^{Linear}	-61.6105	131.2210	90.9711	$1.81\cdot10^{-10}$	$2.36\cdot10^{-10}$	12.2599	-0.05370	-	-	-	-
DysBnum	-21.5177	51.0353	10.7854	$1.47\cdot10^{-10}$	-	0.163798	-	-	-	12	0.047599
DysDupBnum	-17.1231	44.2462	3.9963	$1.92\cdot10^{-10}$	-	0.145872	-	0.421396	-	12	$1.85\cdot10^{-10}$

B)

Model	Log-likelihood	AIC	ΔAIC	λ	λ_l	δ	δ_l	ρ	μ	β	ν
**DysDup**	-17.1965	40.3929	NA	$1.81\cdot10^{-10}$	-	0.169969	-	0.271935	-	-	-
DysDupDem=	-18.1991	42.3981	2.0052	$1.81\cdot10^{-10}$	-	0.151055	-	0.096436	-	-	-
DysDupDem	-17.1955	42.3909	1.9980	$2.06\cdot10^{-10}$	-	0.149251	-	0.278115	$2.15\cdot10^{-10}$	-	-
Dys	-46.2119	96.4237	56.031	$1.81\cdot10^{-10}$	-	0.141696	-	-	-	-	-
Dys^{Linear}Dup	-17.1675	44.3350	3.9421	$1.81\cdot10^{-10}$	$2.05\cdot10^{-10}$	0.163225	$-7.3\cdot10^{-4}$	0.278115	-	-	-
Dys^{Linear}DupDem=	-18.1713	46.3426	5.9497	$1.81\cdot10^{-10}$	$1.80\cdot10^{-10}$	0.106231	$-7.3\cdot10^{-4}$	0.106231	-	-	-
Dys^{Linear}DupDem	-17.1666	46.3332	5.9403	$1.81\cdot10^{-10}$	$2.04\cdot10^{-10}$	0.164653	$-7.4\cdot10^{-4}$	0.274625	$2.15\cdot10^{-10}$	-	-
Dys^{Linear}	-54.4996	116.999	76.606	$1.81\cdot10^{-10}$	$1.80\cdot10^{-10}$	3.725610	-0.0172	-	-	-	-
DysBnum	-19.5572	47.1143	6.7214	$1.81\cdot10^{-10}$	-	0.152786	-	-	-	12	0.025077
DysDupBnum	-17.1965	44.3929	4.0000	$1.81\cdot10^{-10}$	-	0.169969	-	0.271935	-	12	$1.81\cdot10^{-10}$
