# Supplementary material for: Ancient polyploidization events influence the evolution of the ginseng family (Araliaceae)
Source: Front Plant Sci. 2025 Jun 13;16:1595321. doi: 10.3389/fpls.2025.1595321 (PMC12202383; doi:10.3389/fpls.2025.1595321)
Supplement: Supplementary file 5 [file DataSheet5.pdf]

Supplementary Data 5. Parameters or nuclear (A) and plastid (B) ChromoSSE models. $\gamma_{a,}$ anagenetic chromosome gain rate; $\delta_{a,}$ anagenetic chromosome loss rate; ρ_a, anagenetic whole genome duplication rate; ϕc, cladogenetic no chromosome number change rate; γ_c, cladogenetic chromosome gain rate; δ_c, cladogenetic chromosome loss rate; ρ_c, cladogenetic whole genome duplication rate. CI: Confidence Interval; EES: Effective sample size.

A)

Statistics	Anagenetic rates			Cladogenetic rates			
	γ_a	δ_a	ρ_a	ϕ_c	γ_c	δ_c	ρ_c
Mean	0.0001	0.0007	0.0025	0.0294	0.0001	0.0004	0.0014
Median	0	0.0005	0.0023	0.0291	0	$5.91 \cdot 10^{-5}$	0.0009
CI 95%	0-0.0011	0-0.0025	0-0.0065	0.0205-0.0402	0-0.0015	0-0.0020	0-0.0052
EES	3661	1356	693	2225	4459	2274	1278

B)

Statistics	Anagenetic rates			Cladogenetic rates			
	γ_a	δ_a	ρ_a	ϕ_c	γ_c	δ_c	ρ_c
Mean	0.0002	0.0008	0.0011	0.0385	0.0001	0.0005	0.0018
Median	0	0.0006	0.0007	0.0378	0	0.0001	0.0015
CI 95%	0-0.0015	0-0.0030	0-0.0045	0.0269-0.0540	0-0.0012	0-0.0025	0-0.0051
EES	3567	1215	1997	2087	4408	2232	2484
