# Supplementary material for: Ancient polyploidization events influence the evolution of the ginseng family (Araliaceae)
Source: Front Plant Sci. 2025 Jun 13;16:1595321. doi: 10.3389/fpls.2025.1595321 (PMC12202383; doi:10.3389/fpls.2025.1595321)
Supplement: Supplementary file 6 [file DataSheet6.pdf]

Supplementary Data 6. Ploidy estimation models obtained with nQuire for each of the samples. Those parameters that provide support to the best model are marked in blue while those that provide support to an alternative model appear in gray. The ploidy level estimated for each sample is provided. When none of the models provided a R2≥0.1 we considered that none of the models had a proper adjustment to the distribution of the data.

Species	Model	freeLog	LogL	deltaLog	SSR	y-y slope	std.Err	R2	Ploidy_estimated
Aralia_armata	2x	424.6	352.6	71.9	0.017	0.502	0.055	0.583	2x
	3x	424.6	178	246.5	0.041	-0.149	0.123	0.024	
	4x	424.6	246.2	178.3	0.022	0.17	0.192	0.013	
Aralia_californica	2x	3431	3025.8	405.2	0.002	0.774	0.016	0.974	2x
	3x	3431	1025.4	2405.6	0.067	-0.623	0.124	0.298	
	4x	3431	2055.4	1375.6	0.027	0.300	0.227	0.028	
Aralia_chinensis	2x	502.2	196.3	305.8	0.035	0.321	0.085	0.191	4x
	3x	502.2	182.0	320.2	0.058	-0.487	0.123	0.208	
	4x	502.2	444.7	57.481	0.014	0.957	0.177	0.331	
Aralia_dasyphylla	2x	268.2	65.9	202.2	0.058	-0.055	0.075	0.009	4x
	3x	268.2	125.4	142.8	0.044	-0.314	0.102	0.138	
	4x	268.2	241.4	26.7	0.011	0.648	0.149	0.242	
Aralia_delavayi	2x	463.4	421.4	41.9	0.015	0.516	0.049	0.645	2x
	3x	463.4	208.3	255.0	0.048	-0.334	0.113	0.128	
	4x	463.4	289.3	174.1	0.022	0.085	0.189	0.003	
Aralia_fargesii	2x	406.7	230.0	176.6	0.026	0.406	0.071	0.354	4x
	3x	406.7	160.6	246.1	0.046	-0.233	0.125	0.055	
	4x	406.7	310.1	96.5	0.018	0.521	0.189	0.113	
Aralia_foliolosa	2x	473.1	361.7	111.4	0.037	0.069	0.034	0.065	None
	3x	473.1	350.7	122.4	0.024	-0.077	0.050	0.038	
	4x	473.1	303.8	169.3	0.009	0.057	0.079	0.008	
Aralia_gintungensis	2x	1272.7	807.0	465.7	0.034	0.6	0.034	0.835	2x
	3x	1272.7	429.4	843.2	0.101	-0.555	0.101	0.337	
	4x	1272.7	832.0	440.6	0.175	0.635	0.175	0.182	
Aralia_hiepiana	2x	605.9	385	220.9	0.034	0.111	0.038	0.127	4x
	3x	605.9	396.9	208.9	0.021	0.037	0.059	0.006	
	4x	605.9	413.5	192.3	0.007	0.247	0.086	0.121	

Species	Model	freeLog	LogL	deltaLog	SSR	y-y slope	std.Err	R2	Ploidy_estimated
Aralia_hypoglauca	2x	238.9	210.3	28.6	0.030	0.279	0.065	0.238	2x
	3x	238.9	147.7	91.2	0.038	-0.168	0.106	0.04	
	4x	238.9	155	83.9	0.020	0.043	0.168	0.001	
Aralia_leschenaultii	2x	565.1	496.5	68.5	0.019	0.376	0.041	0.583	2x
	3x	565.1	282.4	282.6	0.038	-0.268	0.086	0.139	
	4x	565.1	307.8	257.2	0.017	0.016	0.145	<0.001	
Aralia_nudicaulis	2x	949.9	682.4	267.5	0.009	0.762	0.055	0.763	2x
	3x	949.9	295.0	654.9	0.071	-0.574	0.147	0.204	
	4x	949.9	644.0	305.9	0.027	0.660	0.242	0.111	
Aralia_regeliana	2x	673.4	466.1	207.3	0.019	0.462	0.056	0.53	2x
	3x	673.4	279.0	394.4	0.052	-0.47	0.103	0.258	
	4x	673.4	456.4	216.9	0.015	0.546	0.173	0.144	
Aralia_soratensis	2x	559.5	413.7	145.7	0.016	0.548	0.06	0.583	2x
	3x	559.5	224.3	335.1	0.053	-0.359	0.127	0.118	
	4x	559.5	383.4	176.1	0.02	0.481	0.202	0.087	
Aralia_spinifolia	2x	12105.1	109.1	11995.9	0.068	-0.001	0.108	<0.001	None
	3x	12105.1	133.2	11971.8	0.044	0.074	0.158	0.003	
	4x	12105.1	107.2	11997.9	0.035	0.028	0.246	<0.001	
Aralia_spinosa	2x	589.4	220.6	368.8	0.034	0.23	0.067	0.164	4x
	3x	589.4	220.1	369.3	0.047	-0.424	0.092	0.262	
	4x	589.4	462.7	126.7	0.007	0.837	0.127	0.421	
Aralia_subcordata	2x	807.4	217.1	590.3	0.090	-0.304	0.097	0.142	3x
	3x	807.4	680.2	127.2	0.011	0.871	0.102	0.551	
	4x	807.4	393.1	4141.3	0.040	-0.446	0.230	0.059	
Aralia_thomsonii	2x	642.0	504.7	137.2	0.009	0.716	0.053	0.754	2x
	3x	642.0	217.3	424.6	0.0629	-0.444	0.145	0.137	
	4x	642.0	432.3	209.6	0.028	0.426	0.237	0.052	
Astropanax_myrianthus	2x	4647.1	4463.7	183.3	0.023	0.266	0.027	0.612	2x
	3x	4647.1	2595.8	2051.3	0.026	-0.084	0.063	0.029	
	4x	4647.1	2252.6	2394.4	0.016	-0.228	0.096	0.087	
Brassaiopsis_elegans	2x	3390.3	3197.1	193.2	0.019	0.321	0.024	0.747	2x
	3x	3390.3	1816.2	1574.1	0.033	-0.237	0.063	0.194	
	4x	3390.3	1811.2	1579.0	0.014	-0.063	0.109	0.005	

Species	Model	freeLog	LogL	deltaLog	SSR	y-y slope	std.Err	R2	Ploidy_estimated
Brassaiopsis_gigantea	2x	3082.3	2967.1	115.2	0.023	0.265	0.026	0.631	**2x**
	3x	3082.3	1793.2	1289.1	0.028	-0.145	0.060	0.089	
	4x	3082.3	1610.6	1471.7	0.015	-0.190	0.095	0.063	
Brassaiopsis_glomerulata	2x	3261.5	2953.2	308.2	0.021	0.308	0.026	0.700	**2x**
	3x	3261.5	1693.9	1567.6	0.032	-0.212	0.064	0.157	
	4x	3261.5	1682.8	1578.6	0.016	-0.204	0.105	0.059	
Brassaiopsis_gracilis	2x	2696.8	2375.4	321.3	0.026	0.207	0.025	0.526	**2x**
	3x	2696.8	1613.7	1083.1	0.028	-0.172	0.049	0.17	
	4x	2696.8	1412.6	1284.1	0.014	-0.189	0.081	0.085	
Brassaiopsis_hispida	2x	3800.1	3694.2	105.9	0.009	0.568	0.030	0.855	**2x**
	3x	3800.1	1451.2	2348.8	0.047	-0.357	0.106	0.159	
	4x	3800.1	2129.2	1670.9	0.023	-0.042	0.181	<0.001	
Brassaiopsis_rufosetosa	2x	3347.1	3295.5	51.5	0.021	0.311	0.027	0.683	**2x**
	3x	3347.1	1831.8	1515.2	0.030	-0.151	0.068	0.075	
	4x	3347.1	1753.7	1593.3	0.017	-0.221	0.107	0.067	
Brassaiopsis_shweliensis	2x	3854.8	3812.2	42.5	0.011	0.524	0.029	0.839	**2x**
	3x	3854.8	1552.2	2302.6	0.044	-0.317	0.100	0.145	
	4x	3854.8	2119.6	1735.1	0.022	-0.119	0.168	0.008	
Brassaiopsis_simplex	2x	3082.4	3008.2	74.2	0.021	0.321	0.029	0.665	**2x**
	3x	3082.4	1609.4	1473.0	0.029	-0.109	0.073	0.036	
	4x	3082.4	1517.4	1565.0	0.017	-0.240	0.111	0.072	
Brassaiopsis_simplicifolia	2x	3998.7	3790.6	208.1	0.020	0.319	0.027	0.696	**2x**
	3x	3998.7	2088.9	1909.7	0.033	-0.228	0.066	0.167	
	4x	3998.7	2077.3	1921.3	0.015	-0.122	0.111	0.200	
Brassaiopsis_spnovWen9223	2x	3113.4	2941.2	172.2	0.029	0.165	0.026	0.400	**2x**
	3x	3113.4	2079.4	1034.0	0.023	-0.044	0.049	0.013	
	4x	3113.4	1594.1	1519.3	0.014	-0.268	0.069	0.204	
Brassaiopsis_tripteris	2x	3008.2	2877.2	131.0	0.025	0.231	0.028	0.530	**2x**
	3x	3008.2	1811.9	1196.3	0.028	-0.141	0.057	0.093	
	4x	3008.2	1543.6	1464.6	0.014	-0.193	0.089	0.073	
Brassaiopsis_variabilis	2x	2372.1	2302.1	70.1	0.028	0.209	0.036	0.361	**2x**
	3x	2372.1	1438.8	933.3	0.026	-0.076	0.065	0.022	
	4x	2372.1	1178.7	1193.4	0.017	-0.322	0.094	0.165	

Species	Model	freeLog	LogL	deltaLog	SSR	y-y slope	std.Err	R2	Ploidy_estimated
Cephalaralia_cephalobotrys	2x	641.8	597.6	44.1	0.016	0.506	0.051	0.619	2x
	3x	641.8	285.7	356.1	0.049	-0.372	0.111	0.158	
	4x	641.8	378.1	263.7	0.022	0.089	0.189	0.003	
Cheirodendron_bastardianum	2x	513.1	392.4	120.6	0.048	0.032	0.067	0.004	None
	3x	513.1	365.3	147.7	0.035	-0.145	0.096	0.036	
	4x	513.1	258.1	254.9	0.019	-0.065	0.153	0.003	
Cheirodendron_dominii	2x	622.3	553.4	68.9	0.023	0.338	0.049	0.446	2x
	3x	622.3	311.3	310.9	0.042	-0.034	0.084	0.221	
	4x	622.3	341.7	280.6	0.017	0.027	0.149	<0.001	
Cheirodendron_fauriei	2x	320.6	295.2	25.3	0.045	0.159	0.084	0.056	None
	3x	320.6	234.5	86.1	0.038	-0.036	0.126	0.001	
	4x	320.6	185.7	134.8	0.028	-0.154	0.196	0.010	
Cheirodendron_forbesii	2x	430.9	359.6	71.2	0.039	0.120	0.058	0.066	None
	3x	430.9	305.7	125.1	0.026	0.026	0.088	0.001	
	4x	430.9	238.4	192.4	0.017	-0.060	0.137	0.003	
Cheirodendron_platyphyllum	2x	695.2	587.5	107.6	0.025	0.256	0.042	0.382	2x
	3x	695.2	373.7	321.4	0.035	-0.251	0.071	0.172	
	4x	695.2	355.4	339.7	0.016	-0.105	0.121	0.012	
Cheirodendron_trygynum	2x	610.3	550.7	59.6	0.022	0.367	0.051	0.472	2x
	3x	610.3	288.3	322.1	0.041	-0.305	0.093	0.154	
	4x	610.3	313.6	296.7	0.019	-0.020	0.157	<0.001	
Chengiopanax_fargesii	2x	3290.2	3044.7	245.4	0.023	0.236	0.016	0.771	2x
	3x	3290.2	2035.4	1254.7	0.027	-0.147	0.047	0.141	
	4x	3290.2	1809.7	1480.4	0.011	-0.071	0.078	0.013	
Chengiopanax_sciadophylloides	2x	3760.9	3554.1	206.7	0.005	0.682	0.022	0.939	2x
	3x	3760.9	1236.9	2523.9	0.059	-0.544	0.113	0.281	
	4x	3760.9	2148.4	1612.5	0.025	0.125	0.207	0.006	
Crepinella_spruceana	2x	2588.0	2434.7	153.2	0.024	0.267	0.038	0.456	2x
	3x	2588.0	1451.9	1136.1	0.024	0.011	0.075	<0.001	
	4x	2588.0	1210.8	1377.1	0.018	-0.262	0.111	0.085	
Crepinella_umbellata	2x	1147.3	1104.9	42.4	0.032	0.214	0.056	0.196	2x
	3x	1147.3	719.2	428.1	0.029	-0.039	0.091	0.003	
	4x	1147.3	589.5	557.8	0.021	-0.283	0.137	0.066	

Species	Model	freeLog	LogL	deltaLog	SSR	y-y slope	std.Err	R2	Ploidy_estimated
Cussonia_bancoensis	2x	6010.9	5916.7	94.1	0.015	0.431	0.031	0.759	2x
	3x	6010.9	2618.4	3392.5	0.038	-0.252	0.087	0.122	
	4x	6010.9	3091.1	2919.7	0.020	-0.205	0.143	0.033	
Cussonia_holstii	2x	5840.1	5648.5	191.4	0.013	0.478	0.031	0.803	2x
	3x	5840.1	2400.8	3439.1	0.042	-0.331	0.091	0.191	
	4x	5840.1	3100.3	2739.6	0.021	-0.138	0.156	0.013	
Cussonia_ostinii	2x	5868.3	5632.1	236.1	0.016	0.405	0.031	0.735	2x
	3x	5868.3	2638.3	3229.9	0.038	-0.285	0.081	0.172	
	4x	5868.3	3073.7	2794.5	0.019	-0.191	0.137	0.031	
Cussonia_paniculata	2x	5448.6	5380.8	67.7	0.009	0.565	0.028	0.869	2x
	3x	5448.6	2052.6	3396.0	0.048	-0.393	0.102	0.198	
	4x	5448.6	3021.5	2427.0	0.024	-0.099	0.178	0.005	
Cussonia_spicata	2x	4878.4	4835.7	41.7	0.020	0.341	0.034	0.626	2x
	3x	4878.4	2385.6	2492.8	0.031	-0.149	0.079	0.057	
	4x	4878.4	2429.3	2449.1	0.020	-0.318	0.119	0.106	
Cussonia_thyrsiflora	2x	5329.6	5128.8	200.7	0.018	0.371	0.030	0.715	2x
	3x	5329.6	2523.3	2806.2	0.034	-0.205	0.078	0.103	
	4x	5329.6	2677.9	2651.7	0.018	-0.202	0.126	0.041	
Dendropanax_arboreus	2x	4315.1	4048.2	266.8	0.020	0.311	0.023	0.748	2x
	3x	4315.1	2335.6	1979.5	0.032	-0.213	0.062	0.165	
	4x	4315.1	2289.4	2025.6	0.014	-0.083	0.105	0.010	
Dendropanax_australis	2x	1978.9	1206.7	772.2	0.039	0.06	0.041	0.035	4x
	3x	1978.9	1290.8	688.1	0.025	-0.062	0.06	0.017	
	4x	1978.9	1327.7	651.2	0.007	0.314	0.086	0.184	
Dendropanax_blakeanus	2x	4261.6	4139.5	122.0	0.023	0.279	0.032	0.563	2x
	3x	4261.6	2329.1	1932.4	0.028	-0.097	0.069	0.032	
	4x	4261.6	2084.2	2177.3	0.017	-0.248	0.105	0.086	
Dendropanax_bolivianus	2x	3431.3	3204.1	227.1	0.019	0.338	0.030	0.683	2x
	3x	3431.3	1736.8	1694.4	0.028	-0.063	0.077	0.011	
	4x	3431.3	1625.6	1805.6	0.017	-0.159	0.119	0.029	
Dendropanax_borneensis	2x	3600.9	3387.2	213.6	0.020	0.306	0.026	0.692	2x
	3x	3600.9	1901.4	1699.5	0.031	-0.202	0.064	0.141	
	4x	3600.9	1838.1	1762.7	0.015	-0.145	0.107	0.030	

Species	Model	freeLog	LogL	deltaLog	SSR	y-y slope	std.Err	R2	Ploidy_estimated
Dendropanax_burmanicus	2x	3874.6	3769.8	104.7	0.015	0.414	0.023	0.838	2x
	3x	3874.6	1854.9	2019.7	0.035	-0.230	0.080	0.122	
	4x	3874.6	2073.4	1801.2	0.017	-0.080	0.133	0.006	
Dendropanax_caloneurus	2x	2148.2	2044.9	103.2	0.026	0.217	0.031	0.461	2x
	3x	2148.2	1337.1	811.1	0.022	0.005	0.061	<0.001	
	4x	2148.2	1067.7	1080.5	0.016	-0.28	0.087	0.149	
Dendropanax_caucanus	2x	3506.5	3360.1	146.4	0.017	0.367	0.027	0.755	2x
	3x	3506.5	1756.8	1749.6	0.035	-0.239	0.073	0.151	
	4x	3506.5	1873.2	1633.2	0.016	-0.082	0.124	0.007	
Dendropanax_chevalieri	2x	2624.2	2239.7	384.4	0.009	0.587	0.031	0.854	2x
	3x	2624.2	966.8	1657.3	0.052	-0.464	0.104	0.251	
	4x	2624.2	1513.5	1110.7	0.021	0.134	0.186	0.008	
Dendropanax_cordifolius	2x	4294.4	4125.6	168.8	0.015	0.432	0.030	0.772	2x
	3x	4294.4	1880.1	2414.2	0.040	-0.322	0.083	0.202	
	4x	4294.4	2280.4	2013.9	0.020	-0.162	0.143	0.021	
Dendropanax_cuneatus	2x	2551.1	2349.1	201.9	0.028	0.180	0.026	0.437	2x
	3x	2551.1	1699.1	851.8	0.021	0.004	0.051	<0.001	
	4x	2551.1	1317.5	1233.4	0.013	-0.190	0.076	0.095	
Dendropanax_cuneifolius	2x	4688.2	4520.5	167.6	0.017	0.364	0.026	0.763	2x
	3x	4688.2	2317.6	2370.5	0.034	-0.232	0.073	0.146	
	4x	4688.2	2439.0	2249.1	0.016	-0.115	0.122	0.015	
Dendropanax_dentigerus	2x	2975.6	2549.3	426.3	0.022	0.280	0.025	0.667	2x
	3x	2975.6	1611.3	1364.2	0.033	-0.253	0.056	0.257	
	4x	2975.6	1561.0	1414.6	0.011	0.055	0.101	0.005	
Dendropanax_filipes	2x	3080.6	2867.7	213.1	0.029	0.168	0.026	0.408	2x
	3x	3080.6	2088.5	992.1	0.021	0.016	0.049	0.001	
	4x	3080.6	1582.9	1497.7	0.014	-0.242	0.071	0.164	
Dendropanax_globosus	2x	4203.7	4144.4	59.3	0.022	0.295	0.035	0.542	2x
	3x	4203.7	2159.9	2043.8	-0.132	-0.132	0.073	0.051	
	4x	4203.7	2061.9	2141.7	-0.357	-0.357	0.108	0.155	
Dendropanax_gonatopodus	2x	848.3	712,7	135.6	0.052	0.046	0.083	0.005	None
	3x	848.3	588.0	260.3	0.034	0.027	0.122	>0.001	
	4x	848.3	370.3	478.0	0.030	-0.372	0.183	0.065	

Species	Model	freeLog	LogL	deltaLog	SSR	y-y slope	std.Err	R2	Ploidy_estimated
Dendropanax_hainanensis	2x	3824.5	3458.3	366.1	0.024	0.236	0.017	0.761	2x
	3x	3824.5	2329.7	1494.7	0.027	-0.142	0.047	0.131	
	4x	3824.5	2053.1	1771.3	0.011	-0.016	0.079	<0.001	
Dendropanax_lancifolius	2x	3991.0	3892.5	98.4	0.025	0.25	0.035	0.454	2x
	3x	3991.0	2217.7	1773.2	0.027	-0.087	0.069	0.026	
	4x	3991.0	1925.4	2065.5	0.018	-0.345	0.099	0.168	
Dendropanax_latilobus	2x	4785.1	4685.4	99.6	0.016	0.403	0.028	0.77	2x
	3x	4785.1	2236.9	2548.1	0.034	-0.196	0.083	0.086	
	4x	4785.1	2474.0	2311.1	0.019	-0.177	0.133	0.028	
Dendropanax_macropodus	2x	4269.2	4056.0	213.2	0.016	0.372	0.020	0.847	2x
	3x	4269.2	2160.7	2108.4	0.033	-0.206	0.071	0.123	
	4x	4269.2	2265.2	2003.9	0.014	-0.033	0.119	0.001	
Dendropanax_maingayi	2x	783.3	679.5	103.8	0.038	0.153	0.062	0.091	None
	3x	783.3	530.6	252.7	0.030	-0.040	0.096	0.003	
	4x	783.3	415.3	368.0	0.018	-0.048	0.149	0.017	
Dendropanax_nebulosus	2x	3821.8	3791.2	30.6	0.018	0.364	0.032	0.686	2x
	3x	3821.8	1864.8	1956.9	0.034	-0.196	0.079	0.094	
	4x	3821.8	1973.7	1848.1	0.019	-0.254	0.125	0.065	
Dendropanax_nutans	2x	650.2	424.2	225.9	0.047	0.027	0.063	0.003	None
	3x	650.2	422.6	227.5	0.034	-0.151	0.090	0.045	
	4x	650.2	353.2	297.0	0.020	-0.207	0.141	0.035	
Dendropanax_oliganthus	2x	2509.8	2429.1	80.7	0.019	0.368	0.039	0.596	2x
	3x	2509.8	1207.9	1301.9	0.036	-0.214	0.086	0.095	
	4x	2509.8	1294.6	1215.2	0.020	-0.223	0.137	0.042	
Dendropanax_oligodontus	2x	1672.7	1597.1	75.6	0.027	0.239	0.043	0.336	2x
	3x	1672.7	977.5	695.1	0.032	-0.189	0.074	0.099	
	4x	1672.7	866.1	806.5	0.017	-0.207	0.118	0.048	
Dendropanax_pallidus	2x	4598.4	4459.3	139.1	0.008	0.587	0.028	0.881	2x
	3x	4598.4	1721.0	2877.4	0.048	-0.357	0.109	0.154	
	4x	4598.4	2531.5	2066.9	0.023	0.004	0.184	<0.001	
Dendropanax_palustris	2x	4520.6	4458.8	61.7	0.015	0.410	0.029	0.770	2x
	3x	4520.6	2061.5	2459.1	0.036	-0.225	0.085	0.110	
	4x	4520.6	2318.4	2202.1	0.019	-0.191	0.135	0.032	

Species	Model	freeLog	LogL	deltaLog	SSR	y-y slope	std.Err	R2	Ploidy_estimated
Dendropanax_pendulus	2x	4255.6	4199.8	55.8	0.021	0.309	0.029	0.646	2x
	3x	4255.6	2276.3	1979.3	0.029	-0.127	0.071	0.051	
	4x	4255.6	2159.1	2096.5	0.017	-0.259	0.108	0.088	
Dendropanax_poilanii	2x	3812.0	3314.1	497.8	0.010	0.539	0.025	0.883	2x
	3x	3812.0	1461.9	2350.1	0.050	-0.483	0.088	0.335	
	4x	3812.0	2151.7	1660.2	0.018	0.128	0.168	0.009	
Dendropanax praestans	2x	4420.4	4397.1	23.2	0.020	0.344	0.035	0.612	2x
	3x	4420.4	2148.6	2271.7	0.032	-0.163	0.081	0.064	
	4x	4420.4	2207.2	2213.1	0.021	-0.334	0.122	0.112	
Dendropanax_proteus	2x	2684.3	2580.4	103.9	0.021	0.339	0.039	0.557	2x
	3x	2684.3	1325.7	1358.6	0.038	-0.297	0.077	0.202	
	4x	2684.3	1421.4	1262.9	0.018	-0.185	0.132	0.032	
Dendropanax_resinosus	2x	3768.6	3663.2	105.4	0.017	0.386	0.034	0.685	2x
	3x	3768.6	1736.8	2031.7	0.037	-0.254	0.082	0.140	
	4x	3768.6	1952.1	1816.5	0.020	-0.239	0.134	0.051	
Dendropanax_sessiliflorus	2x	4600.5	4274.7	325.8	0.008	0.594	0.027	0.885	2x
	3x	4600.5	1696.6	2903.8	0.053	-0.499	0.100	0.294	
	4x	4600.5	2669.3	1931.2	0.022	0.061	0.186	0.001	
Dendropanax_spnovWen6891	2x	4131.2	3946.2	184.8	0.025	0.243	0.034	0.458	2x
	3x	4131.2	2339.5	1791.6	0.030	-0.171	0.064	0.108	
	4x	4131.2	2082.5	2048.6	0.017	-0.271	0.099	0.111	
Dendropanax_spnovWen53767	2x	4194.5	3958.6	235.9	0.007	0.621	0.027	0.893	2x
	3x	4194.5	1481.9	2712.5	0.055	-0.504	0.105	0.277	
	4x	4194.5	2407.8	1786.6	0.023	0.079	0.193	0.002	
Dendropanax_swartzii	2x	2971.4	2828.9	142.5	0.031	0.167	0.040	0.225	2x
	3x	2971.4	1788.5	1182.9	0.024	-0.007	0.067	<0.001	
	4x	2971.4	1370.6	1600.8	0.020	-0.477	0.083	0.454	
Dendropanax_trilobus	2x	3236.7	3014.1	222.5	0.019	0.365	0.035	0.64	2x
	3x	3236.7	1508.8	1727.9	0.039	-0.313	0.076	0.223	
	4x	3236.7	1649.1	1587.6	0.017	-0.090	0.134	0.007	
Dendropanax_umbellatus	2x	3765.6	3713.3	52.2	0.022	0.299	0.035	0.548	2x
	3x	3765.6	1963.3	1802.2	0.031	-0.168	0.073	0.082	
	4x	3765.6	1887.5	1878.1	0.018	-0.288	0.113	0.099	

Species	Model	freeLog	LogL	deltaLog	SSR	y-y slope	std.Err	R2	Ploidy_estimated
Dendropanax_weberbaueri	2x	2204.2	2103.1	101.1	0.019	0.362	0.038	0.605	2x
	3x	2204.2	1082.8	1121.3	0.037	-0.256	0.081	0.143	
	4x	2204.2	1154.1	1050.1	0.017	-0.095	0.136	0.008	
Dydimopanax_angustissimus	2x	5024.2	4916.6	107.5	0.018	0.361	0.031	0.693	2x
	3x	5024.2	2392.8	2631.4	0.034	-0.203	0.077	0.103	
	4x	5024.2	2518.3	25050.8	0.018	-0.221	0.124	0.505	
Dydimopanax_morototoni	2x	4120.4	3959.2	161.1	0.018	0.345	0.026	0.740	2x
	3x	4120.4	2114.8	2005.6	0.033	-0.225	0.070	0.149	
	4x	4120.4	2164.6	1955.8	0.015	-0.110	0.117	0.015	
Dydimopanax_vinosus	2x	4229.7	4104.8	124.9	0.024	0.261	0.035	0.477	2x
	3x	4229.7	2298.9	1930.7	0.029	-0.124	0.069	0.051	
	4x	4229.7	2076.9	2152.8	0.018	-0.350	0.101	0.167	
Eleutherococcus_lasiogyne	2x	3358.1	3283.3	75.7	0.013	0.482	0.032	0.792	2x
	3x	3358.1	1396.1	1961.9	0.043	-0.331	0.093	0.176	
	4x	3358.1	1806.2	1551.8	0.021	-0.144	0.158	0.013	
Eleutherococcus_nodiflorus	2x	3814.1	497.8	3316.2	0.081	-0.369	0.049	0.488	4x
	3x	3814.1	2699.6	1114,4	0.017	0.331	0.090	0.184	
	4x	3814.1	2841.0	973.1	0.012	0.432	0.145	0.130	
Eleutherococcus_sessiliflorus	2x	3205.4	2778.8	426.5	0.009	0.576	0.028	0.871	2x
	3x	3205.4	1166.2	2039.1	0.054	-0.526	0.094	0.342	
	4x	3205.4	1817.8	1387.5	0.021	0.118	0.181	0.007	
Eleutherococcus_simonii	2x	2686.3	2235.7	450.5	0.021	0.297	0.029	0.636	2x
	3x	2686.3	1396.2	1290.1	0.036	-0.321	0.057	0.350	
	4x	2686.3	1438.1	1248.1	0.012	0.083	0.109	0.009	
Eleutherococcus_spinosus	2x	471.6	25.1	446.5	0.086	-0.052	0.132	0.002	None
	3x	471.6	29.1	442.4	0.074	-0.358	0.187	0.057	
	4x	471.6	27.6	444	0.054	-0.329	0.298	0.020	
Eleutherococcus_trifoliatus	2x	2389.4	2056.4	332.9	0.024	0.236	0.026	0.581	2x
	3x	2389.4	1383.5	1005.8	0.031	-0.237	0.049	0.277	
	4x	2389.4	1279.8	1109.5	0.012	-0.049	0.091	0.004	
Eleutherococcus_wilsonii	2x	2647.1	718.8	1928.3	0.034	0.262	0.072	0.180	4x
	3x	2647.1	804.1	1843.0	0.049	-0.412	0.103	0.211	
	4x	2647.1	2561.2	85.9	0.006	1.070	0.117	0.585	

Species	Model	freeLog	LogL	deltaLog	SSR	y-y slope	std.Err	R2	Ploidy_estimated
Fatsia_japonica	2x	4250.7	3858.4	392.2	0.019	0.317	0.020	0.809	**2x**
	3x	4250.7	2230.9	2019.8	0.034	-0.273	0.056	0.283	
	4x	4250.7	2262.3	1988.4	0.013	-0.027	0.104	0.001	
Fatsia_oligocarpella	2x	4643.1	4186.1	456.9	0.012	0.492	0.027	0.846	**2x**
	3x	4643.1	1864.9	2768.1	0.046	-0.433	0.084	0.309	
	4x	4643.1	2540.8	2102.2	0.018	0.019	0.157	<0.001	
Fatsia_polycarpa	2x	3740.3	3669.8	70.5	0.016	0.385	0.023	0.816	**2x**
	3x	3740.3	1849.4	1890.8	0.033	-0.202	0.076	0.106	
	4x	3740.3	1976.2	1764.1	0.017	-0.134	0.124	0.019	
Frodinia_gleasonii	2x	5836.6	5801.9	34.7	0.017	0.390	0.031	0.721	**2x**
	3x	5836.6	2676.6	3160.0	0.034	-0.193	0.083	0.083	
	4x	5836.6	2947.1	2889.4	0.020	-0.278	0.130	0.072	
Gamblea_ciliata	2x	3457.8	3178.5	279.3	0.021	0.292	0.022	0.744	**2x**
	3x	3457.8	1926.6	1531.1	0.031	-0.221	0.057	0.199	
	4x	3457.8	1873.1	1584.6	0.013	-0.082	0.099	0.011	
Gamblea_innovans	2x	3114.0	2935.3	178.6	0.016	0.402	0.029	0.765	**2x**
	3x	3114.0	1436.6	1677.3	0.037	-0.273	0.079	0.167	
	4x	3114.0	1613.5	1500.4	0.017	-0.065	0.135	0.003	
Gamblea_malayana	2x	4454.4	4027.1	427.2	0.013	0.441	0.021	0.878	**2x**
	3x	4454.4	1998.8	2455.5	0.042	-0.389	0.073	0.322	
	4x	4454.4	2497.9	1956.4	0.015	0.080	0.138	0.005	
Gamblea_pseudoevodiifolia	2x	3499.6	3399.4	100.2	0.020	0.321	0.026	0.708	**2x**
	3x	3499.6	º849.5	1650.1	0.030	-0.159	0.060	0.082	
	4x	3499.6	1787.3	1712.3	0.016	-0.172	0.110	0.039	
Harmsiopanax_aculeatus	2x	388.9	339.4	49.4	0.020	0.397	0.050	0.513	**2x**
	3x	388.9	185.4	203.4	0.044	-0.344	0.094	0.182	
	4x	388.9	218.3	170.5	0.019	0.014	0.163	<0.001	
Harmsiopanax_ingens	2x	378.5	348.4	30.1	0.014	0.564	0.053	0.655	**2x**
	3x	378.5	147.7	230.8	0.054	-0.423	0.120	0.174	
	4x	378.5	218.1	160.3	0.025	0.122	0.204	0.005	
Hedera_algeriensis	2x	1487.9	1165.8	322.0	0.04	0.067	0.050	0.029	**None**
	3x	1487.9	972.9	514.9	0.029	-0.0114	0.073	0.039	
	4x	1487.9	698	789.8	0.017	-0.212	0.113	0.056	

Species	Model	freeLog	LogL	deltaLog	SSR	y-y slope	std.Err	R2	Ploidy_estimated
Hedera_azorica	2x	2714.2	2585.8	128.3	0.026	0.235	0.037	0.395	2x
	3x	2714.2	1527.6	1186.5	0.031	-0.172	0.067	0.099	
	4x	2714.2	1348.7	1365.4	0.017	-0.274	0.104	0.104	
Hedera_canariensis	2x	1207.2	1151.1	56.1	0.028	0.237	0.048	0.293	2x
	3x	1207.2	733.8	473.4	0.031	-0.133	0.081	0.043	
	4x	1207.2	647.2	560.0	0.018	-0.171	0.127	0.029	
Hedera_colchica	2x	1604.1	1027.7	576.4	0.057	-0.135	0.049	0.112	3x
	3x	1604.1	1371.9	232.2	0.017	0.212	0.071	0.13	
	4x	1604.1	732.6	871.5	0.021	-0.481	0.101	0.276	
Hedera_helix	2x	3349.6	3284.8	64.8	0.029	0.279	0.060	0.265	2x
	3x	3349.6	1808.3	1541.3	0.033	-0.083	0.102	0.011	
	4x	3349.6	1635.7	1713.9	0.024	-0.306	0.155	0.062	
Hedera_hibernica	2x	2323.3	2253.4	69.9	0.028	0.27	0.055	0.284	2x
	3x	2323.3	1386.7	936.5	0.034	-0.14	0.094	0.036	
	4x	2323.3	1279.8	1043.4	0.021	-0.196	0.147	0.029	
Hedera_iberica	2x	1713.3	1544.7	168.5	0.068	0.058	0.119	0.004	None
	3x	1713.3	1498.1	215.1	0.052	-0.002	0.173	<0.001	
	4x	1713.3	1197.9	515.3	0.044	-0.208	0.268	0.01	
Hedera_maderensis	2x	477.2	299.2	178.0	0.061	-0.029	0.089	0.001	None
	3x	477.2	397.6	79.6	0.033	0.105	0.129	0.011	
	4x	477.2	336.7	140.5	0.025	0.105	0.202	0.004	
Hedera_maroccana	2x	2947.5	2768.9	178.6	0.029	0.199	0.041	0.281	2x
	3x	2947.5	1667.5	1280.0	0.026	-0.056	0.070	0.01	
	4x	2947.5	1376.3	1571.2	0.020	-0.479	0.091	0.316	
Hedera_nepalensis_nepalensis	2x	3320.0	3247	72.0	0.027	0.227	0.038	0.375	2x
	3x	3320.0	1880	1439.0	0.026	-0.062	0.069	0.013	
	4x	3320.0	1598	1721.0	0.019	-0.42	0.095	0.249	
Hedera_nepalensis_sinensis	2x	1050.6	585.7	464.9	0.040	0.137	0.067	0.067	4x
	3x	1050.6	553.9	496.7	0.041	-0.302	0.093	0.093	
	4x	1050.6	682.8	367.8	0.014	0.283	0.153	0.153	
Hedera_pastuchovii_cypria	2x	442.9	256.7	186.1	0.066	-0.075	0.093	0.010	None
	3x	442.9	328.6	114.2	0.034	0.14	0.135	0.017	
	4x	442.9	217.8	225.1	0.033	-0.315	0.208	0.037	

Species	Model	freeLog	LogL	deltaLog	SSR	y-y slope	std.Err	R2	Ploidy_estimated
Hedera_rhombea	2x	441.0	172.1	268.0	0.086	-0.252	0.099	0.099	None
	3x	441.0	395.3	45.7	0.04	0.103	0.151	0.007	
	4x	441.0	318.4	122.6	0.034	-0.088	0.236	0.002	
Heptapleurum_altigenum	2x	67.5	19.4	48.1	0.068	-0.008	0.108	<0.001	4x
	3x	67.5	35.2	32.3	0.050	-0.109	0.157	0.008	
	4x	67.5	62.1	5.3	0.025	0.627	0.231	0.110	
Heptapleurum_calyptratum	2x	4832.9	4666.5	166.4	0.021	0.301	0.024	0.725	2x
	3x	4832.9	2668.3	2164.6	0.029	-0.146	0.064	0.080	
	4x	4832.9	2491.1	2341.9	0.015	-0.150	0.102	0.035	
Heptapleurum_delavayi	2x	2893.3	2598.9	294.3	0.024	0.251	0.031	0.531	2x
	3x	2893.3	1626.7	1266.5	0.031	-0.194	0.060	0.149	
	4x	2893.3	1506.8	1386.5	0.015	-0.212	0.098	0.074	
Heptapleurum_forbesii	2x	2466.6	2369.8	96.7	0.025	0.242	0.033	0.477	2x
	3x	2466.6	1517.1	949.5	0.026	-0.062	0.065	0.015	
	4x	2466.6	1285.3	1181.2	0.015	-0.194	0.100	0.060	
Heptapleurum_heptaphyllum	2x	3516.2	3227.8	288.3	0.031	0.130	0.025	0.313	2x
	3x	3516.2	2471.1	1045.1	0.022	-0.025	0.043	0.005	
	4x	3516.2	1856.9	1659.2	0.014	-0.278	0.058	0.279	
Heptapleurum_heterophyllum	2x	3263.2	2937.2	325.9	0.019	0.331	0.023	0.776	2x
	3x	3263.2	1752.6	1510.6	0.033	-0.224	0.064	0.169	
	4x	3263.2	1819.4	1443.8	0.012	0.084	0.110	0.009	
Heptapleurum_ischnoacrum	2x	3247.3	2946.9	300.4	0.032	0.116	0.024	0.268	2x
	3x	3247.3	2362.7	884.6	0.020	0.010	0.042	0.001	
	4x	3247.3	1721.1	1526.2	0.013	-0.215	0.059	0.179	
Heptapleurum_kornasii	2x	1893.5	1731.4	162.1	0.013	0.480	0.034	0.770	2x
	3x	1893.5	818.5	1074.9	0.040	-0.257	0.098	0.104	
	4x	1893.5	1014.5	878.9	0.018	0.077	0.161	0.004	
Heptapleurum_minutistellatum	2x	539.0	365.8	173.1	0.059	-0.029	0.085	<0.001	None
	3x	539.0	468.9	70.0	0.032	0.009	0.124	0.009	
	4x	539.0	383.5	155.4	0.025	-0.019	0.194	<0.001	
Heptapleurum_pachyphlebium	2x	3596.1	2533.2	1062.9	0.011	0.536	0.034	0.807	2x
	3x	3596.1	1281.7	2313.4	0.055	-0.577	0.084	0.440	
	4x	3596.1	2012.1	1583.9	0.015	0.394	0.168	0.085	

Species	Model	freeLog	LogL	deltaLog	SSR	y-y slope	std.Err	R2	Ploidy_estimated
Heptapleurum_petelotii	2x	3915.3	3773.7	141.6	0.018	0.368	0.029	0.724	2x
	3x	3915.3	1928.6	1986.6	0.033	-0.199	0.077	0.100	
	4x	3915.3	2018.1	1897.2	0.018	-0.187	0.125	0.036	
Heptapleurum_rugosum	2x	2631.2	2521.5	109.7	0.021	0.297	0.028	0.654	2x
	3x	2631.2	1499.3	1131.8	0.028	-0.122	0.067	0.052	
	4x	2631.2	1381.7	1239.4	0.015	-0.129	0.106	0.024	
Heptapleurum_scandens	2x	4335.3	4247.9	87.3	0.016	0.388	0.028	0.757	2x
	3x	4335.3	2047.8	2287.4	0.035	-0.220	0.079	0.115	
	4x	4335.3	2227.6	2107.6	0.018	-0.171	0.129	0.028	
Heptapleurum_wardii	2x	2706.5	2641.3	65.2	0.025	0.260	0.039	0.418	2x
	3x	2706.5	1539.0	1167.5	0.032	-0.179	0.072	0.093	
	4x	2706.5	1420.5	1286.0	0.018	-0.265	0.113	0.084	
Heteropanax_brevipedicellatus	2x	4347.8	4256.1	91.7	0.020	0.330	0.031	0.649	2x
	3x	4347.8	2187.1	2160.7	0.033	-0.202	0.073	0.115	
	4x	4347.8	2207.5	2140.2	0.017	-0.217	0.117	0.054	
Heteropanax_fragrans	2x	5127.8	5072.0	55.8	0.021	0.308	0.032	0.600	2x
	3x	5127.8	2676.6	2451.2	0.030	-0.136	0.073	0.055	
	4x	5127.8	2577.6	2550.1	0.018	-0.293	0.110	0.105	
Hydrocotyle cf nepalensis	2x	2883.9	106.3	2777.5	0.082	-0.340	0.068	0.294	4x
	3x	2883.9	1310.1	1573.8	0.036	-0.037	0.118	0.001	
	4x	2883.9	2634.4	249.4	0.009	0.915	0.141	0.416	
Hydrocotyle umbellata	2x	376.2	263.9	112.2	0.088	-0.010	0.141	<0.001	None
	3x	376.2	328.1	48.1	0.064	0.050	0.205	0.001	
	4x	376.2	286.9	89.3	0.056	-0.080	0.320	0.001	
Kalopanax septemlobus	2x	4267.9	3816.9	450.9	0.006	0.617	0.021	0.934	2x
	3x	4267.9	1530.9	2736.9	0.054	-0.517	0.100	0.310	
	4x	4267.9	2191.6	1776.3	0.022	0.132	0.187	0.008	
Mackinlaya schlechteri	2x	95.7	27.9	66.8	0.092	-0.041	0.144	0.001	None
	3x	95.7	52.5	42.2	0.073	-0.137	0.208	0.007	
	4x	95.7	91.2	3.4	0.046	0.652	0.315	0.067	
Macropanax chienii	2x	1103.3	985.5	117.8	0.034	0.158	0.049	0.147	2x
	3x	1103.3	754.2	349.1	0.030	-0.127	0.076	0.045	
	4x	1103.3	618.9	484.4	0.016	-0.091	0.121	<0.001	

Species	Model	freeLog	LogL	deltaLog	SSR	y-y slope	std.Err	R2	Ploidy_estimated
Macropanax_dispermus	2x	2258.1	1219.5	1038.6	0.020	0.359	0.043	0.533	4x
	3x	2258.1	902.7	1355.4	0.047	-0.488	0.068	0.463	
	4x	2258.1	1640.6	617.4	0.007	0.664	0.116	0.353	
Macropanax_maingayi	2x	3303.0	3104.4	198.5	0.022	0.289	0.029	0.623	2x
	3x	3303.0	1783.1	1519.8	0.030	-0.167	0.065	0.098	
	4x	3303.0	1671.4	1631.5	0.015	-0.170	0.105	0.042	
Macropanax_rosthornii	2x	3408.1	3085.9	322.1	0.016	0.375	0.022	0.826	2x
	3x	3408.1	1701.9	1706.1	0.036	-0.275	0.069	0.209	
	4x	3408.1	1854.1	1553.9	0.013	0.088	0.121	0.008	
Macropanax_serratifolius	2x	3074.9	2737.7	337.1	0.016	0.372	0.022	0.826	2x
	3x	3074.9	1541.1	1533.8	0.034	-0.242	0.071	0.166	
	4x	3074.9	1677.1	1397.9	0.012	0.107	0.120	0.013	
Macropanax_undulatus	2x	3075.9	2955.5	120.3	0.021	0.313	0.031	0.629	2x
	3x	3075.9	1639.9	1436.0	0.027	-0.067	0.074	0.013	
	4x	3075.9	1498.2	1577.7	0.017	-0.214	0.113	0.057	
Merrilliopanax_listeri	2x	3817.8	3529.4	288.3	0.018	0.347	0.021	0.815	2x
	3x	3817.8	1952.4	1865.4	0.034	-0.256	0.064	0.21	
	4x	3817.8	2036.3	1781.4	0.013	0.001	0.113	<0.001	
Merrilliopanax_membranifolius	2x	3099.9	2988.1	111.8	0.017	0.381	0.031	0.718	2x
	3x	3099.9	1465	1634.9	0.035	-0.237	0.079	0.131	
	4x	3099.9	1616.7	1483.2	0.019	-0.209	0.129	0.042	
Meryta_pastoralis	2x	6454.9	6297.4	157.5	0.014	0.440	0.031	0.772	2x
	3x	6454.9	2741.1	3713.7	0.041	-0.319	0.087	0.185	
	4x	6454.9	3412.6	3042.2	0.021	-0.198	0.147	0.029	
Metapanax_davidii	2x	2603.4	2336.7	266.6	0.019	0.332	0.026	0.728	2x
	3x	2603.4	1388.7	1214.6	0.031	-0.170	0.070	0.090	
	4x	2603.4	1386.6	1216.7	0.012	0.061	0.114	0.005	
Metapanax_delavayi	2x	2615.0	2377.2	237.8	0.011	0.509	0.026	0.859	2x
	3x	2615.0	1091.4	1523.5	0.047	-0.430	0.087	0.291	
	4x	2615.0	1510.1	1104.8	0.018	0.070	0.164	0.006	
Oplopanax_elatus	2x	531.4	193.2	338.1	0.057	0.118	0.105	0.021	4x
	3x	531.4	233.2	298.2	0.061	-0.413	0.145	0.119	
	4x	531.4	494.3	37.1	0.023	0.721	0.222	0.151	

Species	Model	freeLog	LogL	deltaLog	SSR	y-y slope	std.Err	R2	Ploidy_estimated
Oplopanax_horridus	2x	3400.0	3171.6	228.3	0.003	0.765	0.019	0.964	2x
	3x	3400.0	1030.2	2369.7	0.065	-0.591	0.126	0.271	
	4x	3400.0	1993.8	1406.1	0.028	0.249	0.227	0.019	
Oreopanax_anomalus	2x	1891.9	1784.8	107.1	0.028	0.212	0.039	0.331	2x
	3x	1891.9	1142.2	749.7	0.027	-0.080	0.069	0.022	
	4x	1891.9	930.6	961.3	0.016	-0.215	0.105	0.066	
Oreopanax_capitatus	2x	3447.9	3343.8	104.1	0.022	0.313	0.036	0.557	2x
	3x	3447.9	1747.2	1700.7	0.033	-0.194	0.075	0.101	
	4x	3447.9	1709.9	1737.9	0.018	-0.218	0.120	0.052	
Oreopanax_cf_argentatus	2x	3884.1	3853.9	30.1	0.018	0.358	0.032	0.678	2x
	3x	3884.1	1911.9	1972.1	0.034	-0.201	0.078	0.101	
	4x	3884.1	2020.9	1863.1	0.019	-0.255	0.123	0.067	
Oreopanax_cf_artocarpoides	2x	4756.7	4598.1	158.6	0.007	0.645	0.029	0.888	2x
	3x	4756.7	1596.0	3160.7	0.057	-0.516	0.110	0.268	
	4x	4756.7	2680.4	2076.2	0.025	0.038	0.201	<0.001	
Oreopanax_cf_membranaceus	2x	3100.1	3030.2	69.8	0.021	0.310	0.032	0.601	2x
	3x	3100.1	1623.5	1476.6	0.029	-0.108	0.074	0.034	
	4x	3100.1	1529.8	1570.3	0.018	-0.261	0.112	0.083	
Oreopanax_cf_trollii	2x	4192.3	3955.6	236.6	0.020	0.303	0.021	0.779	2x
	3x	4192.3	2328.9	1863.3	0.030	-0.188	0.060	0.142	
	4x	4192.3	2223.8	1968.4	0.013	-0.066	0.100	0.007	
Oreopanax_cf_williamsii	2x	3955.8	3903.3	52.5	0.021	0.301	0.027	0.669	2x
	3x	3955.8	2141.2	1814.5	0.028	-0.115	0.068	0.046	
	4x	3955.8	1999.8	1956.0	0.017	-0.281	0.102	0.113	
Oreopanax_divulsus	2x	4004.2	3930.4	73.8	0.017	0.374	0.028	0.751	2x
	3x	4004.2	1936.1	2068.1	0.034	-0.214	0.076	0.116	
	4x	4004.2	2064.3	1939.9	0.018	-0.179	0.125	0.033	
Oreopanax_donnell_smithii	2x	3525.7	3373.8	151.9	0.008	0.603	0.029	0.876	2x
	3x	3525.7	1303.1	2222.7	0.052	-0.438	0.108	0.217	
	4x	3525.7	2029.9	1495.8	0.023	0.066	0.190	0.002	
Oreopanax_eriocephallus	2x	3887.4	3759.9	127.5	0.019	0.333	0.028	0.691	2x
	3x	3887.4	1989.1	1898.3	0.032	-0.183	0.072	0.098	
	4x	3887.4	1963.9	1923.4	0.016	-0.158	0.116	0.030	

Species	Model	freeLog	LogL	deltaLog	SSR	y-y slope	std.Err	R2	Ploidy_estimated
Oreopanax_guatemalensis	2x	110.8	70.0	40.8	0.057	0.106	0.104	0.017	None
	3x	110.8	771.9	38.8	0.049	-0.114	0.152	0.009	
	4x	110.8	76.7	34.1	0.029	0.297	0.235	0.026	
Oreopanax_iodophyllus	2x	2492.1	2726.9	215.1	0.025	0.254	0.033	0.493	2x
	3x	2492.1	1626.4	1315.6	0.027	-0.098	0.066	0.035	
	4x	2492.1	1380.8	1561.3	0.015	-0.189	0.102	0.054	
Oreopanax_kuntzei	2x	3763.8	3621.7	142.1	0.010	0.564	0.032	0.834	2x
	3x	3763.8	1425.6	2338.1	0.049	-0.413	0.104	0.210	
	4x	3763.8	2102.7	1661.0	0.023	-0.008	0.182	<0.001	
Oreopanax_macrocephalus	2x	4156.6	3925.2	231.3	0.023	0.251	0.023	0.656	2x
	3x	4156.6	2446.8	1709.8	0.028	-0.139	0.056	0.095	
	4x	4156.6	2141.1	2015.6	0.013	-0.124	0.090	0.031	
Oreopanax_nicaraguensis	2x	3744.8	3573.5	171.2	0.008	0.604	0.027	0.892	2x
	3x	3744.8	1348.6	2396.1	0.052	-0.465	0.104	0.250	
	4x	3744.8	2116.0	1628.8	0.023	0.030	0.188	<0.001	
Oreopanax_nubigenus	2x	2325.4	1928.4	396.9	0.032	0.118	0.025	0.267	2x
	3x	2325.4	1592.8	732.5	0.025	-0.124	0.040	0.140	
	4x	2325.4	1283.6	1041.7	0.010	-0.027	0.067	0.002	
Oreopanax_oerstedianus	2x	438.7	221.9	216.8	0.068	-0.121	0.087	0.031	None
	3x	438.7	391.1	47.7	0.032	0.132	0.127	0.017	
	4x	438.7	347.5	91.2	0.024	0.118	0.200	0.005	
Oreopanax_pavonii	2x	3245.4	3161.9	83.4	0.023	0.267	0.028	0.600	2x
	3x	3245.4	1837.3	1408.1	0.027	-0.092	0.064	0.033	
	4x	3245.4	1617.8	1627.5	0.016	-0.259	0.096	0.110	
Oreopanax_peltatus	2x	2954.4	2843.6	110.7	0.024	0.270	0.034	0.506	2x
	3x	2954.4	1631.5	1322.8	0.029	-0.125	0.070	0.051	
	4x	2954.4	1463.6	1490.8	0.016	-0.128	0.108	0.064	
Oreopanax_platanifolius+	2x	1097.8	964.5	133.2	0.045	0.070	0.067	0.017	None
	3x	1097.8	826.7	271.1	0.022	0.189	0.096	0.061	
	4x	1097.8	534.5	563.3	0.025	-0.458	0.142	0.148	
Oreopanax_polycephalus	2x	3350.1	3177.9	172.2	0.019	0.339	0.027	0.714	2x
	3x	3350.1	1694.1	1656.1	0.034	-0.232	0.069	0.157	
	4x	3350.1	1735.1	1615.0	0.016	-0.164	0.116	0.032	

Species	Model	freeLog	LogL	deltaLog	SSR	y-y slope	std.Err	R2	Ploidy_estimated
Oreopanax_pycnocarpus	2x	3236.7	3110.2	126.4	0.022	0.282	0.031	0.571	2x
	3x	3236.7	1743.6	1493.1	0.031	-0.168	0.067	0.095	
	4x	3236.7	1658.8	1577.8	0.018	-0.319	0.102	0.142	
Oreopanax_rusbyi	2x	3957.0	3707.9	249.0	0.018	0.347	0.022	0.801	2x
	3x	3957.0	2046.6	1910.3	0.034	-0.241	0.066	0.183	
	4x	3957.0	2114.0	1842.9	0.014	-0.050	0.114	0.003	
Oreopanax_sanderianus	2x	4623.4	4494.3	129.1	0.014	0.450	0.027	0.816	2x
	3x	4623.4	2031.0	2592.4	0.040	-0.309	0.085	0.181	
	4x	4623.4	2500.5	2122.9	0.019	-0.098	0.146	0.007	
Oreopanax_spWen12338	2x	3927.2	3925.1	42.2	0.011	0.523	0.028	0.848	2x
	3x	3927.2	1589.4	2377.8	0.044	-0.341	0.098	0.170	
	4x	3927.2	2160.7	1806.5	0.022	-0.107	0.166	0.006	
Oreopanax_steinbachianus	2x	989.9	921.8	68.1	0.040	0.116	0.061	0.056	None
	3x	989.9	704.2	285.7	0.032	-0.111	0.091	0.024	
	4x	989.9	549.3	440.5	0.021	-0.268	0.139	0.059	
Oreopanax_thaumasiophyllus	2x	2564.8	2422.4	142.4	0.023	0.255	0.027	0.598	2x
	3x	2564.8	1550.3	1014.5	0.028	-0.126	0.060	0.068	
	4x	2564.8	1356.2	1208.5	0.013	-0.105	0.096	0.020	
Oreopanax_vestitus	2x	302.9	110.6	192.2	0.071	-0.136	0.089	0.037	None
	3x	302.9	206.8	96.1	0.044	-0.164	0.131	0.025	
	4x	302.9	251.1	51.8	0.026	0.079	0.207	0.002	
Oreopanax_xalapensis	2x	2908.6	2612.1	296.5	0.009	0.566	0.028	0.870	2x
	3x	2908.6	1129.3	1779.2	0.051	-0.460	0.098	0.271	
	4x	2908.6	1702.8	1205.7	0.019	0.176	0.177	0.016	
Osmoxylon_boerlagei	2x	503.3	429.2	74.0	0.037	0.128	0.053	0.090	None
	3x	503.3	334.2	169.0	0.031	-0.146	0.078	0.055	
	4x	503.3	267.7	235.5	0.017	-0.144	0.124	0.022	
Osmoxylon_micranthum	2x	205.3	101.4	103.9	0.040	0.176	0.074	0.086	4x
	3x	205.3	107.8	97.4	0.040	-0.203	0.110	0.054	
	4x	205.3	178.9	26.4	0.012	0.612	0.157	0.203	
Osmoxylon_novoguineense	2x	540.1	417.8	122.3	0.014	0.550	0.053	0.641	2x
	3x	540.1	217.1	323.0	0.051	-0.350	0.120	0.123	
	4x	540.1	345.4	194.7	0.021	0.360	0.190	0.053	

Species	Model	freeLog	LogL	deltaLog	SSR	y-y slope	std.Err	R2	Ploidy_estimated
Panax_assamicus	2x	290.6	126.2	164.3	0.031	0.312	0.072	0.239	4x
	3x	290.6	102.5	188.6	0.053	-0.495	0.102	0.285	
	4x	290.6	177.4	113.1	0.015	0.537	0.174	0.138	
Panax_bipinnatifidus	2x	1257.4	120.2	1137.2	0.062	-0.093	0.079	0.023	4x
	3x	1257.4	396.4	860.9	0.049	-0.398	0.104	0.198	
	4x	1257.4	1214.1	43.2	0.004	1.204	0.091	0.747	
Panax_elegantior	2x	336.9	135.7	201.2	0.049	-0.056	0.046	0.024	4x
	3x	336.9	236.5	100.4	0.021	0.073	0.068	0.019	
	4x	336.9	282.3	54.6	0.007	0.354	0.096	0.185	
Panax_ginseng	2x	3410.0	3173.5	236.4	0.002	0.780	0.019	0.965	2x
	3x	3410.0	1029.4	2380.5	0.065	-0.573	0.130	0.246	
	4x	3410.0	2037.2	1372.7	0.028	0.278	0.231	0.023	
Panax_omeiensis	2x	1893.0	101.1	1791.8	0.067	-0.096	0.090	0.019	4x
	3x	1893.0	495.7	1397.3	0.053	-0.405	0.121	0.158	
	4x	1893.0	1763.7	129.3	0.006	1.362	0.104	0.741	
Panax_quinquefolius	2x	3765.6	3510.1	255.5	0.001	0.871	0.021	0.965	2x
	3x	3765.6	1041.8	2723.7	0.073	-0.620	0.147	0.231	
	4x	3765.6	2303.7	1461.8	0.033	0.342	0.257	0.028	
Panax_trifolius	2x	537.1	451.5	85.5	0.011	0.567	0.038	0.784	2x
	3x	537.1	217.1	319.9	0.051	-0.419	0.108	0.202	
	4x	537.1	344.9	192.1	0.020	0.249	0.186	0.029	
Panax_variabilis	2x	343.3	142.2	201.1	0.031	0.309	0.071	0.242	4x
	3x	343.3	129.3	214.0	0.053	-0.493	0.099	0.292	
	4x	343.3	303.3	40.0	0.011	0.740	0.157	0.271	
Panax_vietnamensis	2x	135.6	31.0	104.6	0.056	0.056	0.093	0.006	4x
	3x	135.6	54.6	81.0	0.054	-0.390	0.125	0.139	
	4x	135.6	132.2	3.4	0.019	0.559	0.198	0.118	
Panax_wangianus	2x	368.7	293.7	75.0	0.026	0.334	0.062	0.329	2x
	3x	368.7	181.6	187.1	0.047	-0.404	0.097	0.226	
	4x	368.7	212.5	156.1	0.018	0.182	0.170	0.019	
Polyscias_australiana	2x	346.8	286.3	60.4	0.019	0.378	0.042	0.576	2x
	3x	346.8	163.6	183.1	0.040	-0.299	0.086	0.170	
	4x	346.8	187.9	158.8	0.016	0.068	0.146	0.003	

Species	Model	freeLog	LogL	deltaLog	SSR	y-y slope	std.Err	R2	Ploidy_estimated
Polyscias_baehniana	2x	4174.7	2789.9	1384.8	0.005	0.683	0.028	0.908	2x
	3x	4174.7	1280.1	2894.7	0.063	-0.644	0.106	0.381	
	4x	4174.7	2491.2	1683.5	0.018	0.610	0.195	0.141	
Polyscias_boivinii	2x	3345.2	3026.4	318.8	0.014	0.442	0.029	0.792	2x
	3x	3345.2	1474.1	1871.1	0.041	-0.341	0.082	0.223	
	4x	3345.2	1818.4	1526.7	0.019	-0.087	0.146	0.006	
Polyscias_elliptica	2x	1228.3	985.1	243.2	0.005	0.763	0.038	0.869	2x
	3x	1228.3	382.2	846.1	0.066	-0.561	0.136	0.222	
	4x	1228.3	780.5	447.8	0.025	0.554	0.230	0.089	
Polyscias_fruticosa	2x	2260.3	270.1	1990.1	0.071	0.131	0.132	0.016	None
	3x	2260.3	206.0	2054.3	0.067	-0.152	0.194	0.010	
	4x	2260.3	178.9	2081.4	0.057	-0.489	0.296	0.044	
Polyscias_oahuensis	2x	4419.3	4333.5	85.8	0.017	0.365	0.026	0.764	2x
	3x	4419.3	2216.1	2203.1	0.032	-0.177	0.075	0.084	
	4x	4419.3	2280.1	2139.1	0.017	-0.171	0.121	0.032	
Polyscias_sandwicensis	2x	5720.1	5423.7	296.4	0.006	0.653	0.026	0.913	2x
	3x	5720.1	1943.3	3776.8	0.057	-0.526	0.110	0.279	
	4x	5720.1	3297.0	2423.1	0.025	0.085	0.201	0.003	
Polyscias_schultzii	2x	532.6	264.6	268.0	0.033	0.244	0.066	0.186	4x
	3x	532.6	231.3	301.3	0.046	-0.397	0.093	0.233	
	4x	532.6	379.7	152.8	0.010	0.663	0.142	0.268	
Pseudopanax_colensoi	2x	4138.9	3761.1	377.7	0.020	0.306	0.024	0.721	2x
	3x	4138.9	2171.1	1967.8	0.033	-0.251	0.059	0.230	
	4x	4138.9	2147.3	1991.6	0.013	-0.058	0.106	0.005	
Pseudopanax_crassifolius	2x	3879.5	3535.3	344.2	0.006	0.644	0.024	0.920	2x
	3x	3879.5	1378.2	2501.2	0.056	-0.517	0.107	0.280	
	4x	3879.5	2307.7	1571.8	0.023	0.177	0.196	0.013	
Pseudopanax_laetevirens	2x	4470.8	3796.8	673.9	0.003	0.730	0.016	0.970	2x
	3x	4470.8	1412.7	3058.0	0.064	-0.616	0.115	0.326	
	4x	4470.8	2717.2	1753.5	0.025	0.305	0.214	0.033	
Pseudopanax_lessonii	2x	250.6	217.0	33.5	0.048	0.091	0.080	0.021	None
	3x	250.6	194.1	56.4	0.029	0.139	0.117	0.023	
	4x	250.6	143.5	107.1	0.027	-0.255	0.181	0.032	

Species	Model	freeLog	LogL	deltaLog	SSR	y-y slope	std.Err	R2	Ploidy_estimated
Pseudopanax_valdiviensis	2x	6146.2	5693.0	453.2	0.002	0.818	0.023	0.953	2x
	3x	6146.2	1798.0	4348.2	0.070	-0.611	0.137	0.251	
	4x	6146.2	3735.2	2411.0	0.031	0.274	0.244	0.021	
Raukaua_anomalus	2x	380.5	238.8	141.6	0.038	0.053	0.034	0.040	4x
	3x	380.5	264.2	116.2	0.027	-0.158	0.046	0.164	
	4x	380.5	279.4	101.1	0.006	0.243	0.072	0.160	
Raukaua_simplex	2x	716.3	319.9	396.3	0.054	-0.072	0.059	0.024	4x
	3x	716.3	506.5	209.7	0.027	-0.004	0.087	<0.001	
	4x	716.3	575.4	140.8	0.009	0.412	0.125	0.154	
Schefflera_digitata	2x	594.2	425.9	168.3	0.029	0.239	0.049	0.279	2x
	3x	594.2	319.1	275.1	0.037	-0.277	0.077	0.177	
	4x	594.2	332.9	261.3	0.012	0.207	0.130	0.041	
Sciodaphyllum_acuminatum	2x	3672.1	3575.2	96.8	0.013	0.478	0.030	0.811	2x
	3x	3672.1	1575.3	2096.7	0.041	-0.286	0.093	0.137	
	4x	3672.1	1980.3	1691.7	0.020	-0.082	0.156	0.004	
Sciodaphyllum_angulatum	2x	4276.6	4078.1	198.4	0.019	0.323	0.024	0.741	2x
	3x	4276.6	2246.4	2030.2	0.033	-0.237	0.064	0.187	
	4x	4276.6	2271.4	2005.1	0.015	-0.122	0.109	0.020	
Sciodaphyllum_brownei	2x	4565.0	4318.6	246.3	0.018	0.352	0.025	0.761	2x
	3x	4565.0	2276.2	2288.7	0.035	-0.257	0.068	0.191	
	4x	4565.0	2389.9	2175.1	0.015	-0.094	0.118	0.010	
Sciodaphyllum_chartaceum	2x	4584.5	4475.4	109.1	0.019	0.326	0.027	0.709	2x
	3x	4584.5	2378.3	2206.2	0.031	-0.175	0.069	0.097	
	4x	4584.5	2342.0	2242.5	0.016	-0.191	0.111	0.047	
Sciodaphyllum_herzogii	2x	4989.7	4750.4	239.2	0.009	0.544	0.023	0.898	2x
	3x	4989.7	1950.5	3039.1	0.047	-0.405	0.095	0.235	
	4x	4989.7	2789.5	2200.1	0.021	-0.009	0.169	<0.001	
Sciodaphyllum_pedicellatum	2x	4638.7	4622.9	15.7	0.015	0.433	0.031	0.760	2x
	3x	4638.7	2044.5	2594.1	0.037	-0.227	0.089	0.099	
	4x	4638.7	2398.1	2240.6	0.021	-0.247	0.142	0.048	
Sciodaphyllum_pentandrum	2x	4490.0	4179.7	310.2	0.015	0.397	0.024	0.820	2x
	3x	4490.0	2122.9	2367.0	0.037	-0.294	0.073	0.212	
	4x	4490.0	2406.5	2083.4	0.016	-0.046	0.129	0.002	

Species	Model	freeLog	LogL	deltaLog	SSR	y-y slope	std.Err	R2	Ploidy_estimated
Sciodaphyllum_robustum	2x	4130.7	4049.3	81.3	0.019	0.364	0.036	0.632	2x
	3x	4130.7	1951.3	2179.3	0.036	-0.231	0.081	0.121	
	4x	4130.7	2093.3	2037.4	0.019	-0.217	0.132	0.043	
Sinopanax_formosanus	2x	4579.8	4391.9	187.9	0.015	0.421	0.026	0.812	2x
	3x	4579.8	2103.4	2476.3	0.037	-0.257	0.082	0.143	
	4x	4579.8	2431.9	2147.9	0.018	-0.129	0.136	0.015	
Tetrapanax_papyrifer	2x	4490.4	4468.6	21.8	0.019	0.344	0.033	0.639	2x
	3x	4490.4	2216.5	2273.8	0.032	-0.159	0.078	0.064	
	4x	4490.4	2273.5	2216.9	0.020	-0.309	0.120	0.100	
Trachymene_glaucifolia	2x	258.0	195.9	62.1	0.037	0.141	0.058	0.089	None
	3x	258.0	157.0	101.0	0.038	-0.293	0.081	0.181	
	4x	258.0	143.1	114.9	0.015	0.057	0.139	0.002	
Trevesia_burckii	2x	3656.7	3574.3	82.3	0.019	0.321	0.025	0.725	2x
	3x	3656.7	1958.3	1698.3	0.031	-0.171	0.068	0.097	
	4x	3656.7	1900.3	1756.3	0.016	-0.178	0.109	0.043	
Trevesia_lateospina	2x	335.0	180.9	154.0	0.059	-0.069	0.076	0.014	None
	3x	335.0	268.5	66.4	0.030	0.049	0.111	0.003	
	4x	335.0	248.5	86.4	0.017	0.253	0.170	0.036	
Trevesia_palmata	2x	3451.8	2964.4	487.4	0.008	0.579	0.026	0.893	2x
	3x	3451.8	1284.1	2167.7	0.054	-0.531	0.093	0.354	
	4x	3451.8	2014.5	1437.3	0.019	0.219	0.178	0.024	
Trevesia_sundaica	2x	3763.2	3617.2	146.0	0.015	0.396	0.022	0.839	2x
	3x	3763.2	1827.7	1935.5	0.034	-0.217	0.077	0.118	
	4x	3763.2	2004.8	1758.4	0.017	-0.112	0.126	0.013	
Trevesia_valida	2x	1487.8	1338.4	149.3	0.030	0.173	0.039	0.249	2x
	3x	1487.8	976.5	511.2	0.028	-0.117	0.064	0.053	
	4x	1487.8	805.3	682.4	0.015	-0.167	0.100	0.045	
