# Supplementary material for: Ancient polyploidization events influence the evolution of the ginseng family (Araliaceae)
Source: Front Plant Sci. 2025 Jun 13;16:1595321. doi: 10.3389/fpls.2025.1595321 (PMC12202383; doi:10.3389/fpls.2025.1595321)
Supplement: Supplementary file 7 [file DataSheet7.pdf]

Supplementary Data 7. Estimation of the mean and median allelic ratios and the percentage of SNPs with an allelic ratio <2 for each of the samples analyzed with nQuire, together with the inferred ploidy level based on the adjustment of the model.

Species	Mean	Median	Percentage <2	Ploidy
Aralia armata	1.59	1.29	78.4	Diploid
Aralia californica	1.36	1.19	92.6	Diploid
Aralia chinensis	2.13	1.73	51.1	Tetraploid
Aralia dasyphylla	2.47	2.70	38.9	Tetraploid
Aralia delavayi	1.30	1.62	77.7	Diploid
Aralia fargesii	1.87	1.30	55.8	Tetraploid
Aralia foliolosa	1.99	1.78	57.0	Unknown
Aralia gintungensis	1.71	1.22	70.7	Diploid
Aralia hiepiana	2.01	2.02	49.1	Tetraploid
Aralia hypoglauca	1.87	1.50	67.2	Diploid
Aralia leschenaultii	1.71	1.42	77.8	Diploid
Aralia nudicaulis	1.52	1.16	79.8	Diploid
Aralia regeliana	1.78	1.28	70.6	Diploid
Aralia soratensis	1.68	1.23	71.3	Diploid
Aralia spinifolia	2.10	1.88	53.9	Unknown
Aralia spinosa	2.17	2.00	49.6	Tetraploid
Aralia subcordata	2.27	2.11	36.3	Triploid
Aralia thomsonii	1.51	1.18	80.3	Diploid
Astropanax myrianthus	1.65	1.46	76.5	Diploid
Brassaiopsis elegans	1.68	1.42	75.5	Diploid
Brassaiopsis gigantea	1.71	1.46	75.2	Diploid
Brassaiopsis glomerulata	1.71	1.42	77.0	Diploid
Brassaiopsis gracilis	1.82	1.51	71.3	Diploid
Brassaiopsis hispida	1.44	1.27	87.1	Diploid
Brassaiopsis rufosetosa	1.65	1.43	77.3	Diploid
Brassaiopsis shweliensis	1.46	1.27	88.5	Diploid
Brassaiopsis simplex	1.60	1.44	79.2	Diploid
Brassaiopsis simplicifolia	1.65	1.42	77.3	Diploid
Brassaiopsis sp. nov. Wen9223	1.78	1.57	71.0	Diploid
Brassaiopsis tripteris	1.72	1.50	73.7	Diploid
Brassaiopsis variabilis	1.70	1.50	74.8	Diploid
Cephararalia cephalobotrys	1.58	1.32	79.9	Diploid
Cheirodendron bastardianum	1.94	1.64	66.0	Unknown
Cheirodendron dominii	1.70	1.38	74.8	Diploid
Cheirodendron fauriei	1.89	1.69	69.3	Unknown
Cheirodendron forbesii	1.87	1.72	60.9	Unknown
Cheirodendron platyphyllum	1.78	1.47	73.4	Diploid
Cheirodendron trygynum	1.63	1.35	77.7	Diploid
Chengiopanax fargesii	1.78	1.51	70.4	Diploid
Chengiopanax sciadophylloides	1.37	1.21	92.9	Diploid
Crepinella spruceana	1.66	1.50	74.4	Diploid
Crepinella umbellata	1.74	1.47	71.9	Diploid
Cussonia bancoensis	1.50	1.35	86.1	Diploid

Cussonia holstii	1.49	1.30	87.3	Diploid
Cussonia ostinii	1.55	1.34	84.2	Diploid
Cussonia paniculata	1.42	1.27	89.2	Diploid
Cussonia spicata	1.56	1.39	82.3	Diploid
Cussonia thyrsiflora	1.57	1.39	82.2	Diploid
Dendropanax arboreus	1.69	1.43	75.6	Diploid
Dendropanax australis	2.01	1.94	52.3	Tetraploid
Dendropanax blakeanus	1.63	1.45	77.8	Diploid
Dendropanax bolivianus	1.61	1.40	77.3	Diploid
Dendropanax borneensis	1.66	1.44	77.5	Diploid
Dendropanax burmanicus	1.57	1.36	81.4	Diploid
Dendropanax caloneurus	1.73	1.53	70.8	Diploid
Dendropanax caucanus	1.37	1.62	78.6	Diploid
Dendropanax chevalieri	1.52	1.27	84.0	Diploid
Dendropanax cordifolius	1.53	1.34	85.2	Diploid
Dendropanax cuneatus	1.79	1.58	68.3	Diploid
Dendropanax cuneifolius	1.60	1.39	79.6	Diploid
Dendropanax dentigerus	1.76	1.46	72.3	Diploid
Dendropanax filipes	1.79	1.59	69.1	Diploid
Dendropanax globosus	1.59	1.42	82.5	Diploid
Dendropanax gonatopodus	1.79	1.61	72.6	Unknown
Dendropanax hainanensis	1.77	1.51	69.3	Diploid
Dendropanax lancifolius	1.63	1.47	78.2	Diploid
Dendropoanax latilobus	1.54	1.35	81.5	Diploid
Dendropanax macropodus	1.62	1.38	77.8	Diploid
Dendropanax maingayi	1.83	1.64	64.2	Unknown
Dendropanax nebulosus	1.57	1.39	83.0	Diploid
Dendropanax nutans	2.1	1.7	61.8	Unknown
Dendropanax oliganthus	1.58	1.39	81.2	Diploid
Dendropanax oligodontus	1.72	1.47	76.5	Diploid
Dendropanax pallidus	1.42	1.25	87.8	Diploid
Dendropanax palustris	1.53	1.36	83.6	Diploid
Dendropanax pendulus	1.62	1.43	80.2	Diploid
Dendropanax poilanii	1.53	1.28	84.5	Diploid
Dendropanax praestans	1.56	1.40	85.1	Diploid
Dendropanax proteus	1.62	1.39	80.3	Diploid
Dendropanax resinosus	1.55	1.37	83.5	Diploid
Dendropanax sessiliflorus	1.46	1.25	87.5	Diploid
Dendropanax sp. nov. Wen6891	1.69	1.47	77.9	Diploid
Dendropanax sp. nov. Wen53767	1.42	1.25	90.2	Diploid
Dendropanax swartzii	1.68	1.51	77.3	Diploid
Dendropanax trilobus	1.59	1.38	81.1	Diploid
Dendropanax umbellatus	1.61	1.42	79.5	Diploid
Dendropanax weberbaueri	1.60	1.36	79.5	Diploid
Dydimopanax angustissimus	1.55	1.38	82.7	Diploid
Dydimopanax morototoni	1.63	1.40	78.4	Diploid
Dydimopanax vinosus	1.64	1.46	80.5	Diploid
Eleutherococcus lasiogyne	1.49	1.31	88.4	Diploid
Eleutherococcus nodiflorus	2.58	2.52	20.6	Tetraploid

Eleutherococcus sessiliflorus	1.50	1.27	87.4	Diploid
Eleutherococcus simonii	1.78	1.44	73.5	Diploid
Eleutherococcus spinosus	2.32	1.63	57.1	Unknown
Eleutherococcus trifoliatus	1.82	1.53	71.8	Diploid
Eleutherococcus wilsonii	2.24	2.40	41.5	Tetraploid
Fatsia japonica	1.70	1.42	76.2	Diploid
Fatsia oligocarpella	1.54	1.30	85.3	Diploid
Fatsia polycarpa	1.59	1.38	80.8	Diploid
Frodinia gleasonii	1.52	1.37	85.4	Diploid
Gamblea ciliata	1.74	1.45	73.6	Diploid
Gamblea innovans	1.57	1.35	81.0	Diploid
Gamblea malayana	1.61	1.32	80.4	Diploid
Gamblea pseudoevodiifolia	1.63	1.41	77.3	Diploid
Harmsiopanax aculeatus	1.69	1.36	76.8	Diploid
Harmsiopanax ingens	1.50	1.24	86.9	Diploid
Hedera algeriensis	1.89	1.61	70.3	Unknown
Hedera azorica	1.68	1.47	79.8	Diploid
Hedera canariensis	1.75	1.47	73.9	Diploid
Hedera colchica	2.01	1.78	62.3	Unknown
Hedera helix	1.62	1.45	77.7	Diploid
Hedera hibernica	1.74	1.47	70.5	Diploid
Hedera iberica	2.04	1.75	53.5	Unknown
Hedera maderensis	2.11	2.00	48.6	Unknown
Hedera maroccana	1.68	1.50	79.9	Diploid
Hedera nepalensis subsp. *nepalensis*	1.64	1.47	78.2	Diploid
Hedera nepalensis subsp. *sinensis*	2.10	1.73	55.3	Tetraploid
Hedera pastuchovii subsp. *cypria*	2.11	1.82	62.5	Unknown
Hedera rhombea	2.37	2.11	36.7	Unknown
Heptapleurum altigenum	2.42	2.33	30.0	Tetraploid
Heptapleurum calyptratum	1.66	1.44	77.5	Diploid
Heptapleurum delavayi	1.47	1.77	74.2	Diploid
Heptapleurum forbesii	1.50	1.73	71.2	Diploid
Heptapleurum heptaphyllum	1.84	1.61	67.4	Diploid
Heptapleurum heterophyllum	1.40	1.71	71.9	Diploid
Heptapleurum ischnoacrum	1.85	1.62	65.8	Diploid
Heptapleurum kornasii	1.54	1.30	76.8	Diploid
Heptapleurum minutistellatum	2.00	2.13	48.0	Unknown
Heptapleurum pachyphlebium	1.66	1.26	77.6	Diploid
Heptapleurum petelotii	1.60	1.39	81.5	Diploid
Heptapleurum rugosum	1.69	1.45	73.5	Diploid
Heptapleurum scandens	1.55	1.36	82.9	Diploid
Heptapleurum wardii	1.69	1.44	75.9	Diploid
Heteropanax brevipedicellatus	1.59	1.39	81.0	Diploid
Heteropanax fragrans	1.61	1.41	81.4	Diploid
Hydrocotyle cf. *nepalensis*	2.81	2.85	11.1	Tetraploid
Hydrocotyle umbellata	2.17	2.00	43.1	Unknown
Kalopanax septemlobus	1.47	1.24	87.7	Diploid
Mackinlaya schlechteri	2.41	2.33	34.5	Unknown
Macropanax chienii	1.86	1.57	64.1	Diploid

Macropanax dispermus	1.97	1.39	60.7	Tetraploid
Macropanax maingayi	1.67	1.43	77.5	Diploid
Macropanax rosthornii	1.66	1.39	75.6	Diploid
Macropanax serratifolius	1.67	1.38	72.3	Diploid
Macropanax undulatus	1.62	1.43	76.7	Diploid
Merrilliopanax listeri	1.65	1.38	76.3	Diploid
Merrilliopanax membranifolius	1.58	1.36	81.7	Diploid
Meryta pastoralis	1.50	1.34	87.1	Diploid
Metapanax davidii	1.69	1.42	71.6	Diploid
Metapanax delavayi	1.56	1.29	81.7	Diploid
Oplopanax elatus	2.27	2.33	44.0	Tetraploid
Oplopanax horridus	1.33	1.19	93.1	Diploid
Oreopanax anomalus	1.71	1.50	70.6	Diploid
Oreopanax capitatus	1.60	1.41	80.2	Diploid
Oreopanax cf. *argentatus*	1.58	1.38	81.5	Diploid
Oreopanax cf. *artocarpoides*	1.37	1.24	91.7	Diploid
Oreopanax cf. *membranaceus*	1.61	1.42	80.0	Diploid
Oreopanax cf. *trollii*	1.70	1.45	75.9	Diploid
Oreopanax cf. *williamsii*	1.63	1.44	78.9	Diploid
Oreopanax divulsus	1.57	1.38	82.2	Diploid
Oreopanax donnell-smithii	1.43	1.25	88.1	Diploid
Oreopanax eriocephallus	1.61	1.41	70.1	Diploid
Oreopanax guatemalensis	2.08	2.00	47.27	Unknown
Oreopanax iodophyllus	1.66	1.46	75.4	Diploid
Oreopanax kuntzei	1.44	1.27	86.2	Diploid
Oreopanax_macrocephalus	1.72	1.49	73.0	Diploid
Oreopanax nicaraguensis	1.42	1.24	90.2	Diploid
Oreopanax nubigenus	1.90	1.60	64.5	Diploid
Oreopanax oerstedianus	2.27	2.06	39.8	Unknown
Oreopanax pavonii	1.66	1.46	75.8	Diploid
Oreopanax peltatus	1.65	1.43	77.4	Diploid
Oreopanax platanifolius	1.84	1.67	59.7	Unknown
Oreopanax polycephalus	1.64	1.42	80.6	Diploid
Oreopanax pycnocarpus	1.67	1.43	79.6	Diploid
Oreopanax rusbyi	1.66	1.40	76.7	Diploid
Oreopanax sanderianus	1.53	1.31	84.9	Diploid
Oreopanax sp. Wen12338	1.45	1.29	88.0	Diploid
Oreopanax steinbachianus	1.87	1.55	67.0	Unknown
Oreopanax thaumasiophyllus	1.74	1.53	73.6	Diploid
Oreopanax vestitus	2.46	2.25	37.7	Unknown
Oreopanax xalapensis	1.51	1.26	82.8	Diploid
Osmoxylon boerlagei	1.88	1.62	70.9	Unknown
Osmoxylon micranthum	2.16	2.03	47.3	Tetraploid
Osmoxylon novoguineense	1.65	1.28	70.5	Diploid
Panax assamicus	2.09	1.49	60.4	Tetraploid
Panax bipinnatifidus	2.64	2.85	24.3	Tetraploid
Panax elegantior	2.33	2.26	35.3	Tetraploid
Panax ginseng	1.19	1.33	91.1	Diploid
Panax omeiensis	2.73	2.95	19.7	Tetraploid

Panax quinquefolius	1.29	1.16	95.3	Diploid
Panax trifolius	1.61	1.26	76.6	Diploid
Panax variabilis	2.17	1.80	50.2	Tetraploid
Panax vietnamensis	2.56	2.80	36.4	Tetraploid
Panax wangianus	1.79	1.39	69.1	Diploid
Polyscias australiana	1.72	1.39	74.4	Diploid
Polyscias baehniana	1.58	1.20	80.5	Diploid
Polyscias boivinii	1.60	1.34	84.0	Diploid
Polyscias elliptica	1.43	1.18	85.4	Diploid
Polyscias fruticosa	1.98	1.50	63.1	Unknown
Polyscias oahuensis	1.59	1.40	79.8	Diploid
Polyscias sandwicensis	1.39	1.24	91.3	Diploid
Polyscias schultzii	2.05	1.69	56.2	Tetraploid
Pseudopanax colensoi	1.69	1.43	76.0	Diploid
Pseudopanax crassifolius	1.45	1.23	88.3	Diploid
Pseudopanax laetevirens	1.42	1.20	89.3	Diploid
Pseudopanax lessonii	1.93	1.86	67.4	Unknown
Pseudopanax valdiviensis	1.32	1.17	95.6	Diploid
Raukaua anomalus	2.13	1.82	53.8	Tetraploid
Raukaua simplex	2.21	2.25	39.9	Tetraploid
Schefflera digitata	1.91	1.56	67.6	Diploid
Sciodaphyllum acuminatum	1.50	1.30	83.9	Diploid
Sciodaphyllum angulatum	1.66	1.39	77.4	Diploid
Sciodaphyllum brownei	1.63	1.38	80.2	Diploid
Sciodaphyllum chartaceum	1.62	1.41	79.1	Diploid
Sciodaphyllum herzogii	1.47	1.28	87.3	Diploid
Sciodaphyllum pedicellatum	1.50	1.34	86.2	Diploid
Sciodaphyllum pentandrum	1.62	1.36	81.3	Diploid
Sciodaphyllum robustum	1.55	1.38	84.5	Diploid
Sinopanax formosanus	1.56	1.35	84.0	Diploid
Tetrapanax papyrifer	1.57	1.39	83.3	Diploid
Trachymene glaucifolia	1.94	1.56	65.9	Unknown
Trevesia burckii	1.64	1.44	77.9	Diploid
Trevesia lateospina	2.15	2.1	45.3	Unknown
Trevesia palmata	1.52	1.27	83.6	Diploid
Trevesia sundaica	1.60	1.36	79.3	Diploid
Trevesia valida	1.85	1.54	70.2	Diploid
