# Supplementary material for: Ancient polyploidization events influence the evolution of the ginseng family (Araliaceae)
Source: Front Plant Sci. 2025 Jun 13;16:1595321. doi: 10.3389/fpls.2025.1595321 (PMC12202383; doi:10.3389/fpls.2025.1595321)
Supplement: Supplementary file 8 [file Presentation1.pdf]

Supplementary Figure 1. Time-calibrated nuclear (A) and plastid (B) phylogenies obtained with TreePL. Age ranges are represented as node bars for all the supported nodes. Calibration points appear as stars.

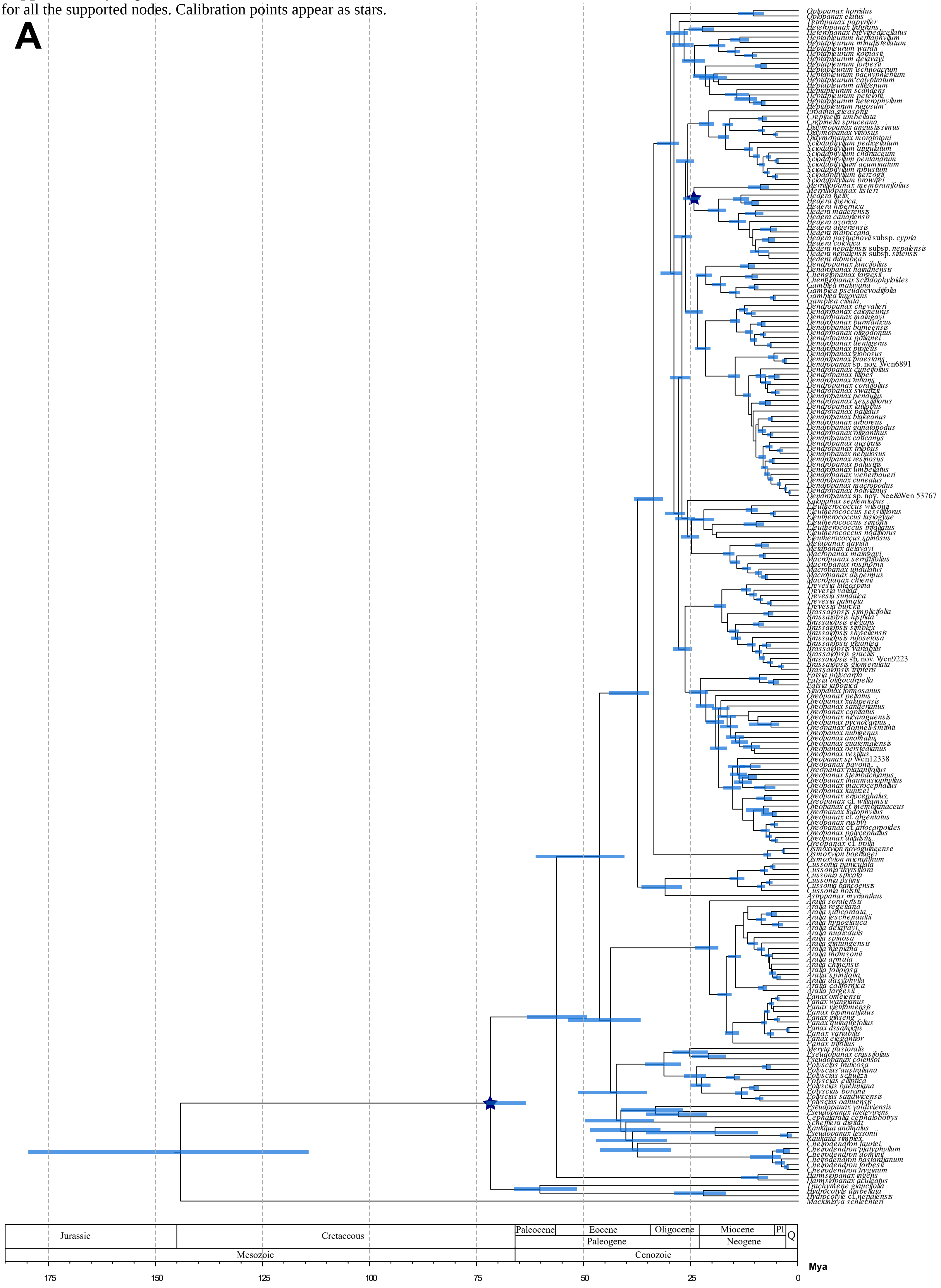

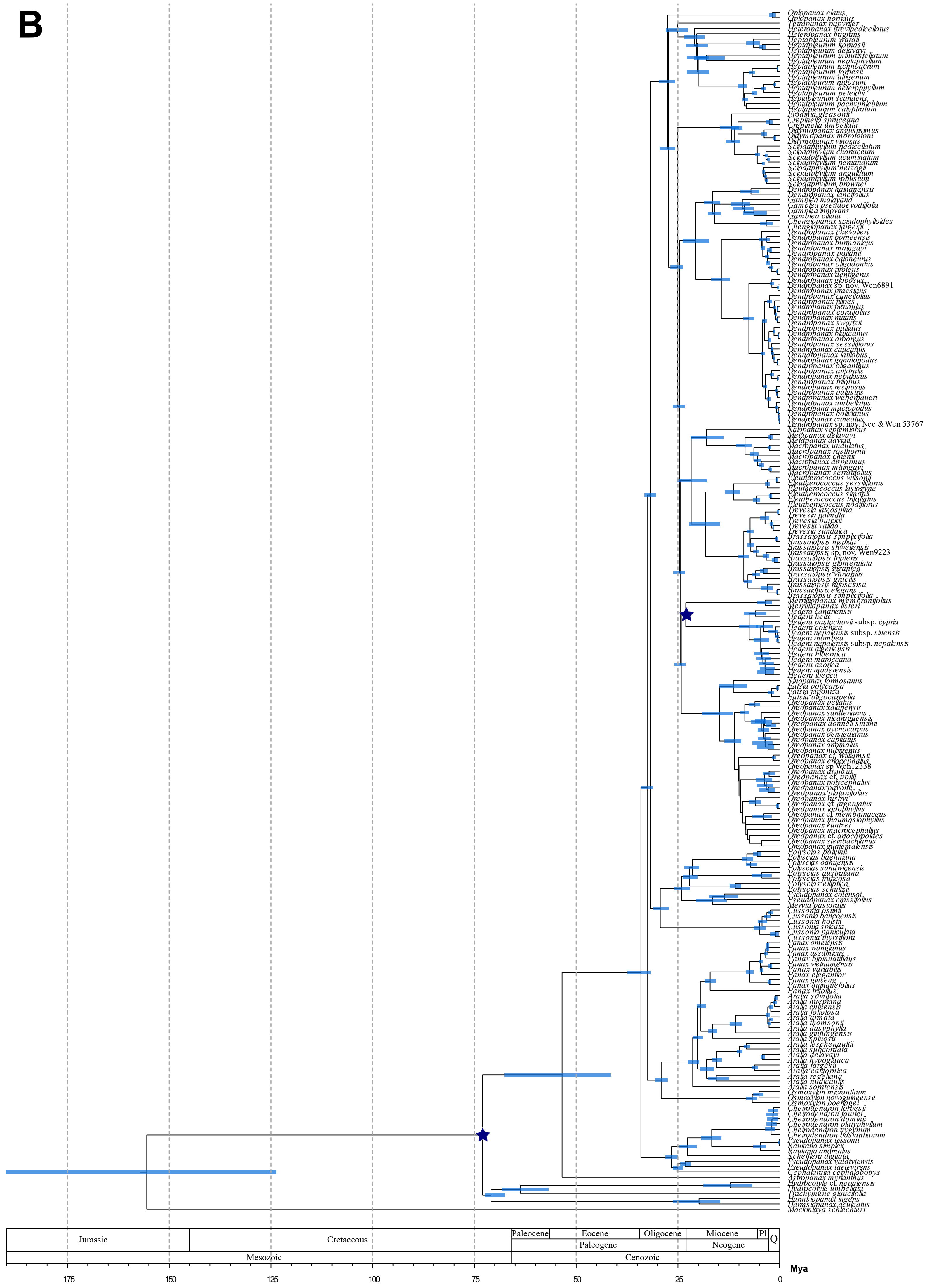

B
Oplopanax elatus
Oplopanax horridus
Tetrapanax papyrifer
Heteropanax brevipedicellatus
Heteropanax fragrans
Heptapleurum wardii
Heptapleurum kornasii
Heptapleurum delavayi
Heptapleurum minutistellatum
Heptapleurum heptaphyllum
Heptapleurum ischnoacrum
Heptapleurum forbesii
Heptapleurum altigenum
Heptapleurum rugosum
Heptapleurum heterophyllum
Heptapleurum petelotii
Heptapleurum scandens
Heptapleurum pachyphlebium
Heptapleurum calyptratum
Frodinia gleasonii
Crepinella spruceana
Crepinella umbellata
Didymopanax angustisimus
Didymopanax morototoni
Didymopanax vinosus
Sciodaphyllum pedicellatum
Sciodaphylum chartaceum
Sciodaphyllum acuminatum
Sciodaphyllum pentandrum
Sciodaphyllum herzogii
Sciodaphyllum angulatum
Sciodaphyllum robustum
Sciodaphyllum brownei
Dendropanax hainanensis
Dendropanax lancifolius
Gamblea malayana
Gamblea pseudoevodiifolia
Gamblea innovans
Gamblea ciliata
Chengiopanax sciadophylloides
Chengiopanax fargesii
Dendropanax chevalieri
Dendropanax borneensis
Dendropanax burmanicus
Dendropanax maingayi
Dendropanax poilanii
Dendropanax caloneurus
Dendropanax oligodontus
Dendropanax proteus
Dendropanax dentigerus
Dendropanax globosus
Dendropanax sp. nov. Wen6891
Dendropanax praestans
Dendropanax cuneifolius
Dendropanax filipes
Dendropanax pendulus
Dendropanax cordifolius
Dendropanax nutans
Dendropanax swartzii
Dendropanax pallidus
Dendropanax blakeanus
Dendropanax arboreus
Dendropanax sessiliflorus
Dendropanax caucanus
Denndropanax latilobus
Dendropanax gonatopodus
Dendropanax oliganthus
Dendropanax australis
Dendropanax nebulosus
Dendropanax trilobus
Dendropanax resinosus
Dendropanax palustris
Dendropanax weberbaueri
Dendropanax umbellatus
Dendropana macropodus
Dendropanax bolivianus
Dendropanax cuneatus
Dendropanax sp. nov. Nee & Wen 53767
Kalopanax septemlobus
Metapanax delavayi
Metapanax davidii
Macropanax undulatus
Macropanax rosthornii
Macropanax chienii
Macropanax dispermus
Macropanax maingayi
Macropanax serratifolius
Eleutherococcus wilsonii
Eleutherococcus sessiliflorus
Eleutherococcus lasiogyne
Eleutherococcus simonii
Eleutherococcus trifoliatus
Eleutherococcus nodiflorus
Trevesia lateospina
Trevesia palmata
Trevesia burckii
Trevesia valida
Trevesia sundaica
Brassaiopsis simplicifolia
Brassaiopsis hispida
Brassaiopsis shweliensis
Brassaiopsis sp. nov. Wen9223
Brassaiopsis tripteris
Brassaiopsis glomerulata
Brassaiopsis gigantea
Brassaiopsis variabilis
Brassaiopsis gracilis
Brassaiopsis rufosetosa
Brassaiopsis elegans
Brassaiopsis simplicifolia
Merrilliopanax membranifolius
Merrilliopanax listeri
Hedera canariensis
Hedera helix
Hedera pastuchovii subsp. cypria
Hedera colchica
Hedera nepalensis subsp. sinensis
Hedera rhombea
Hedera nepalensis subsp. nepalensis
Hedera algeriensis
Hedera hibernica
Hedera maroccana
Hedera azorica
Hedera maderensis
Hedera iberica
Sinopanax formosanus
Fatsia polycarpa
Fatsia japonica
Fatsia oligocarpella
Oreopanax peltatus
Oreopanax xalapensis
Oreopanax sanderianus
Oreopanax nicaraguensis
Oreopanax donnell-smithii
Oreopanax pycnocarpus
Oreopanax oerstedianus
Oreopanax capitatus
Oreopanax anomalus
Oreopanax nubigenus
Oreopanax cf. williamsii
Oreopanax eriocephalus
Oreopanax sp Wen12338
Oreopanax divulsus
Oreopanax cf. trollii
Oreopanax polycephalus
Oreopanax pavonii
Oreopanax platanifolius
Oreopanax rusbyi
Oreopanax cf. argentatus
Oreopanax iodophyllus
Oreopanax cf. membranaceus
Oreopanax thaumasiophyllus
Oreopanax kuntzei
Oreopanax macrocephallus
Oreopanax cf. artocarpoides
Oreopanax steinbachianus
Oreopanax guatemalensis
Polyscias boivinii
Polyscias baehniana
Polyscias oahuensis
Polyscias sandwicensis
Polyscias australiana
Polyscias fruticosa
Polyscias elliptica
Polyscias schultzii
Pseudopanax colensoi
Pseudopanax crassifolius
Meryta pastoralis
Cussonia ostinii
Cussonia bancoensis
Cussonia holstii
Cussonia spicata
Cussonia paniculata
Cussonia thyrsiflora
Panax omeiensis
Panax wangianus
Panax assamicus
Panax bipinnatifidus
Panax vietnamensis
Panax variabilis
Panax elegantior
Panax ginseng
Panax quinquefolius
Panax trifolius
Aralia spinifolia
Aralia huepiana
Aralia chinensis
Aralia foliolosa
Aralia armata
Aralia thomsonii
Aralia dasyphylla
Aralia gintungensis
Aralia spinosa
Aralia leschenaultii
Aralia subcordata
Aralia delavayi
Aralia hypoglauca
Aralia fargesii
Aralia californica
Aralia regeliana
Aralia nudicaulis
Aralia soratensis
Osmoxylon micranthum
Osmoxylon novoguineense
Osmoxylon boerlagei
Cheirodendron forbesii
Cheirodendron fauriei
Cheirodendron dominii
Cheirodendron platyphyllum
Cheirodendron trygynum
Cheirodendron bastardianum
Pseudopanax lessonii
Raukaua simplex
Raukaua anomalus
Schefflera digitata
Pseudopanax valdiviensis
Pseudopanax laetevirens
Cephalaralia cephalobotrys
Astropanax myrianthus
Hydrocotyle cf. nepalensis
Hydrocotyle umbellata
Trachymene glaucifolia
Harmsiopanax ingens
Harmsiopanax aculeatus
Mackinlaya schlechteri
Jurassic
Cretaceous
Paleocene
Eocene
Oligocene
Miocene
Pl
Q
Paleogene
Neogene
Mesozoic
Cenozoic
Mya
175
150
125
100
75
50
25
0
