# Supplementary material for: Ancient polyploidization events influence the evolution of the ginseng family (Araliaceae)
Source: Front Plant Sci. 2025 Jun 13;16:1595321. doi: 10.3389/fpls.2025.1595321 (PMC12202383; doi:10.3389/fpls.2025.1595321)
Supplement: Supplementary file 9 [file Presentation2.pdf]

Supplementary Figure 2. Coalescent-based ASTRAL phylogeny using 156 nuclear loci. Branch support is provided as local posterior probabilities for all supported branches (PPlocal≥0.8). Quartet scores are provided as a measure of gene tree discordance. Quartets scores are also provided: Q1 (blue, most frequent quartet topology recovered for a node across all loci), Q2 (orange, first alternative topology) and Q2 (gray, second alternative topology). A) Detailed view of the evolutionary relationships in the Asian Palmate group (AsPG) presenting the main AsPG clades. B) Detailed view of the early evolutionary relationships in Araliaceae presenting the main Araliaceae clades. The main relationships between Araliaceae clades (A) and AsPG clades (B) are shown.

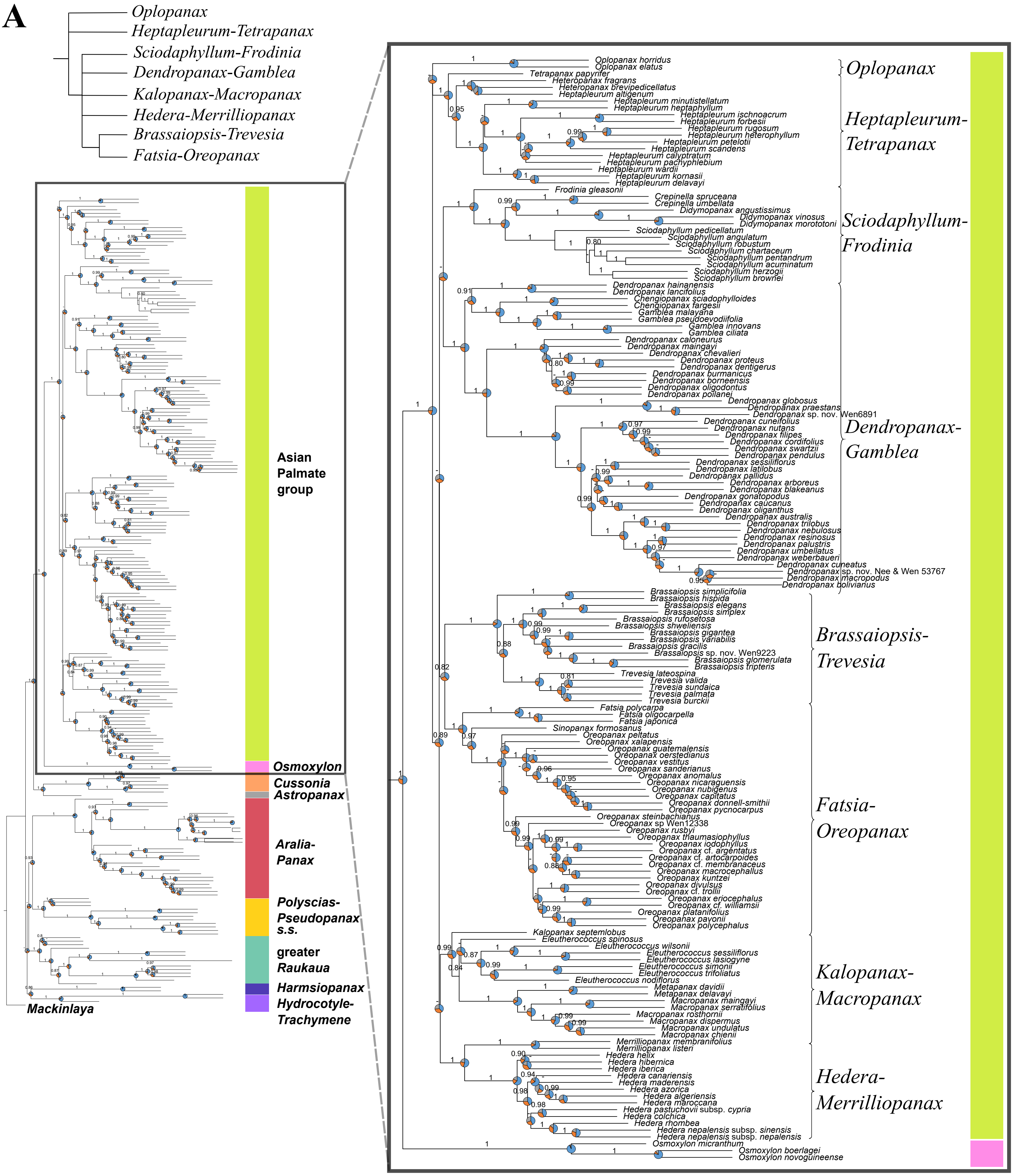

B

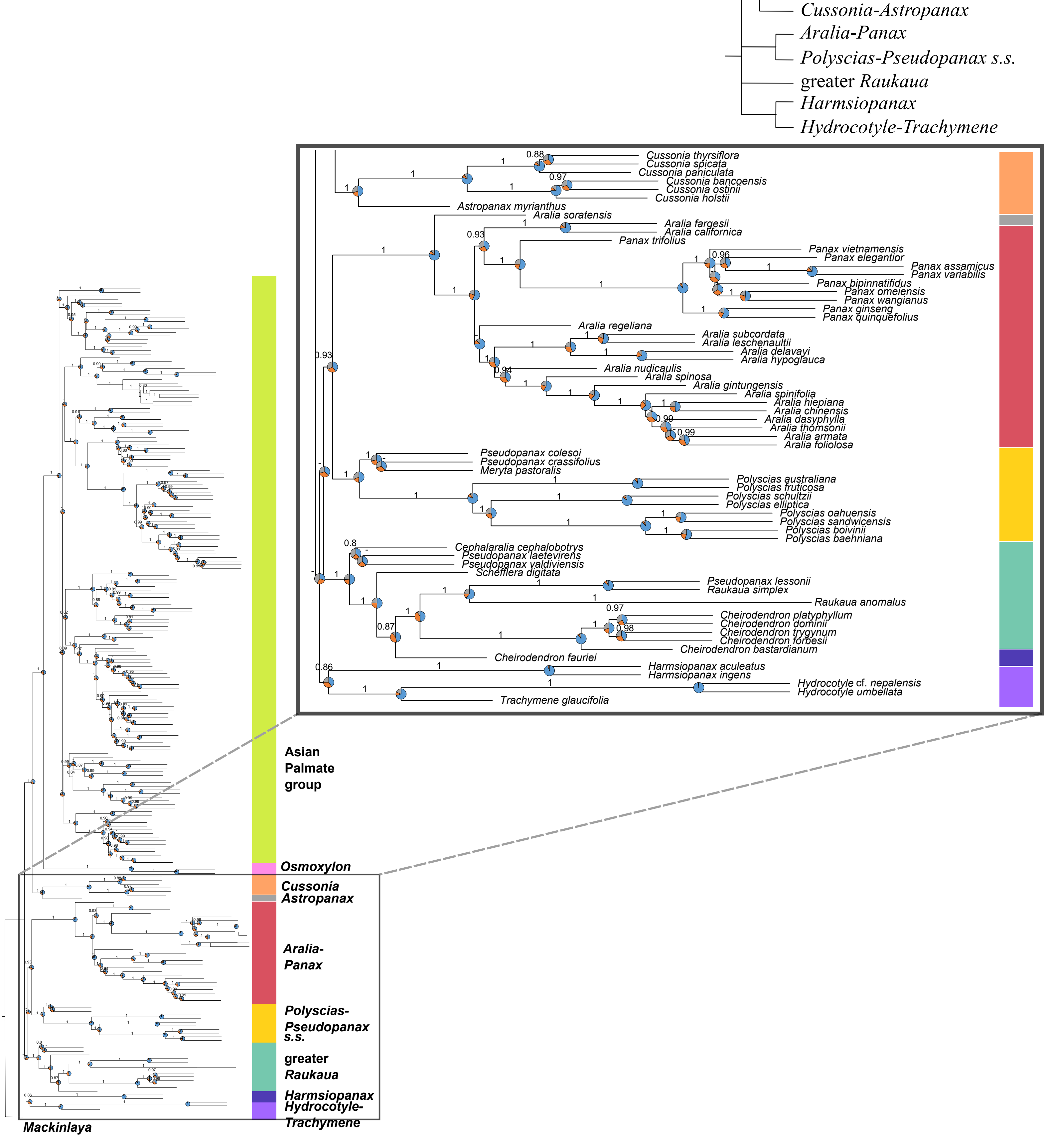
AsPG
Osmoxylon
Cussonia-Astropanax
Aralia-Panax
Polyscias-Pseudopanax s.s.
greater Raukaua
Harmsiopanax
Hydrocotyle-Trachymene
Cussonia thyrsiflora
Cussonia spicata
Cussonia paniculata
Cussonia bancoensis
Cussonia ostinii
Cussonia holstii
Astropanax myrianthus
Aralia soratensis
Aralia fargesii
Aralia californica
Panax trifolius
Panax vietnamensis
Panax elegantior
Panax assamicus
Panax variabilis
Panax bipinnatifidus
Panax omeiensis
Panax wangianus
Panax ginseng
Panax quinquefolius
Aralia regeliana
Aralia subcordata
Aralia leschenaultii
Aralia delavayi
Aralia hypoglauca
Aralia nudicaulis
Aralia spinosa
Aralia gintungensis
Aralia spinifolia
Aralia hiepiana
Aralia chinensis
Aralia dasyphylla
Aralia thomsonii
Aralia armata
Aralia foliolosa
Pseudopanax colesoi
Pseudopanax crassifolius
Meryta pastoralis
Polyscias australiana
Polyscias fruticosa
Polyscias schultzii
Polyscias elliptica
Polyscias oahuensis
Polyscias sandwicensis
Polyscias boivinii
Polyscias baehniana
Cephalaralia cephalobotrys
Pseudopanax laetevirens
Pseudopanax valdiviensis
Schefflera digitata
Pseudopanax lessonii
Raukaua simplex
Raukaua anomalus
Cheirodendron platyphyllum
Cheirodendron dominii
Cheirodendron trygynum
Cheirodendron forbesii
Cheirodendron bastardianum
Cheirodendron fauriei
Harmsiopanax aculeatus
Harmsiopanax ingens
Hydrocotyle cf. nepalensis
Hydrocotyle umbellata
Trachymene glaucifolia
Asian Palmate group
Osmoxylon
Cussonia
Astropanax
Aralia-Panax
Polyscias-Pseudopanax s.s.
greater Raukaua
Harmsiopanax
Hydrocotyle-Trachymene
Mackinlaya
