# Supplementary material for: Ancient polyploidization events influence the evolution of the ginseng family (Araliaceae)
Source: Front Plant Sci. 2025 Jun 13;16:1595321. doi: 10.3389/fpls.2025.1595321 (PMC12202383; doi:10.3389/fpls.2025.1595321)
Supplement: Supplementary file 10 [file Presentation3.pdf]

Figure S3. Density distribution of estimated anagenetic and cladogenetic parameters of nuclear (A) and plastid (B) ChromoSSE models. The distribution of the parameters is shown together and for each parameter separately. Color filling represents 95% confidence intervals. Gamma: chromosome gain anagenetic rate; delta: chromosome loss anagenetic rate; rho: anagenetic polyploidization rate; clado_no_change: no chromosome change cladogenetic rate; clado_fission: chromosome gain cladogenetic rate; clado_fussion: chromosome loss cladogenetic rate; clado_polyploid: cladogenetic polyploidization rate.

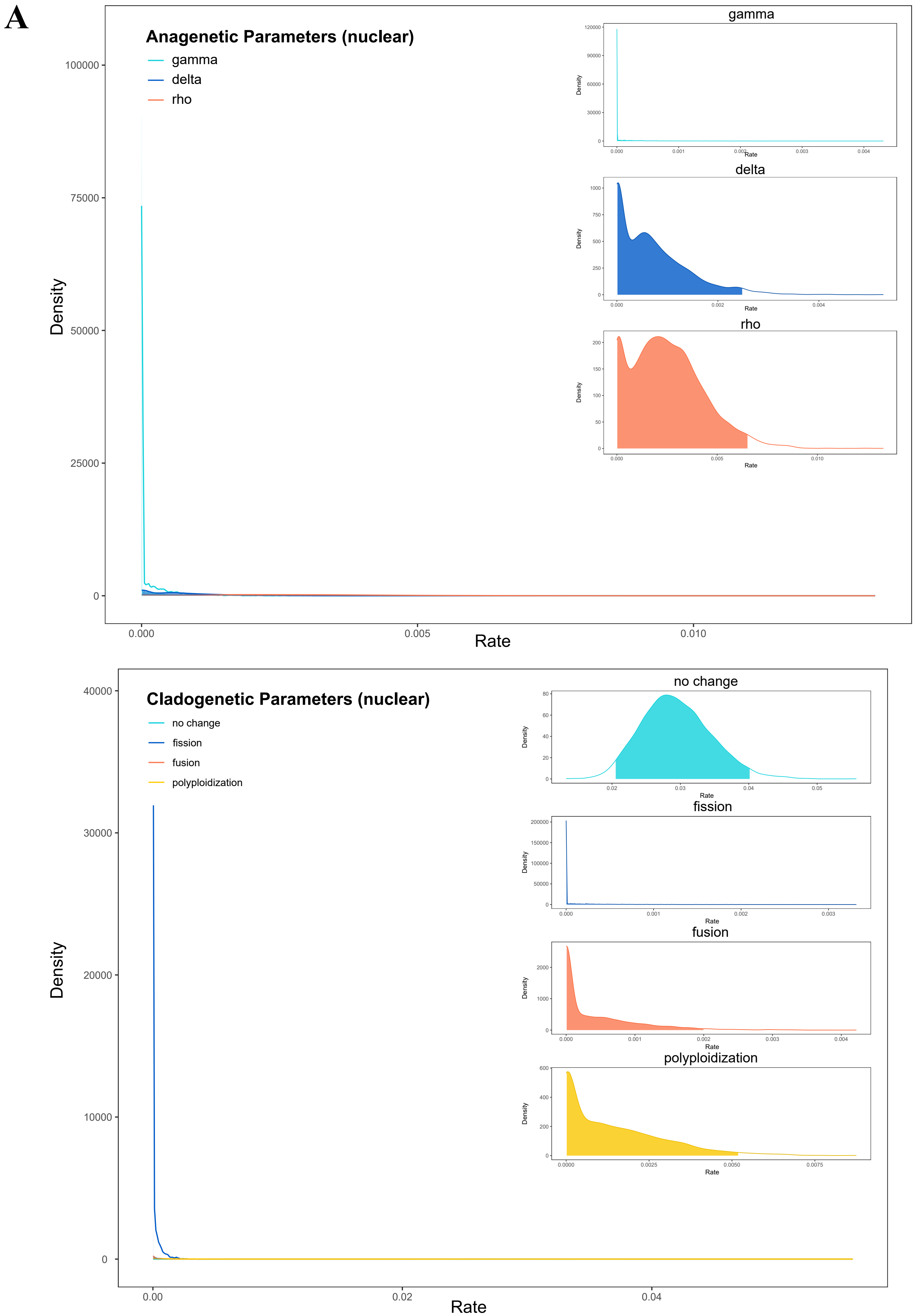

B

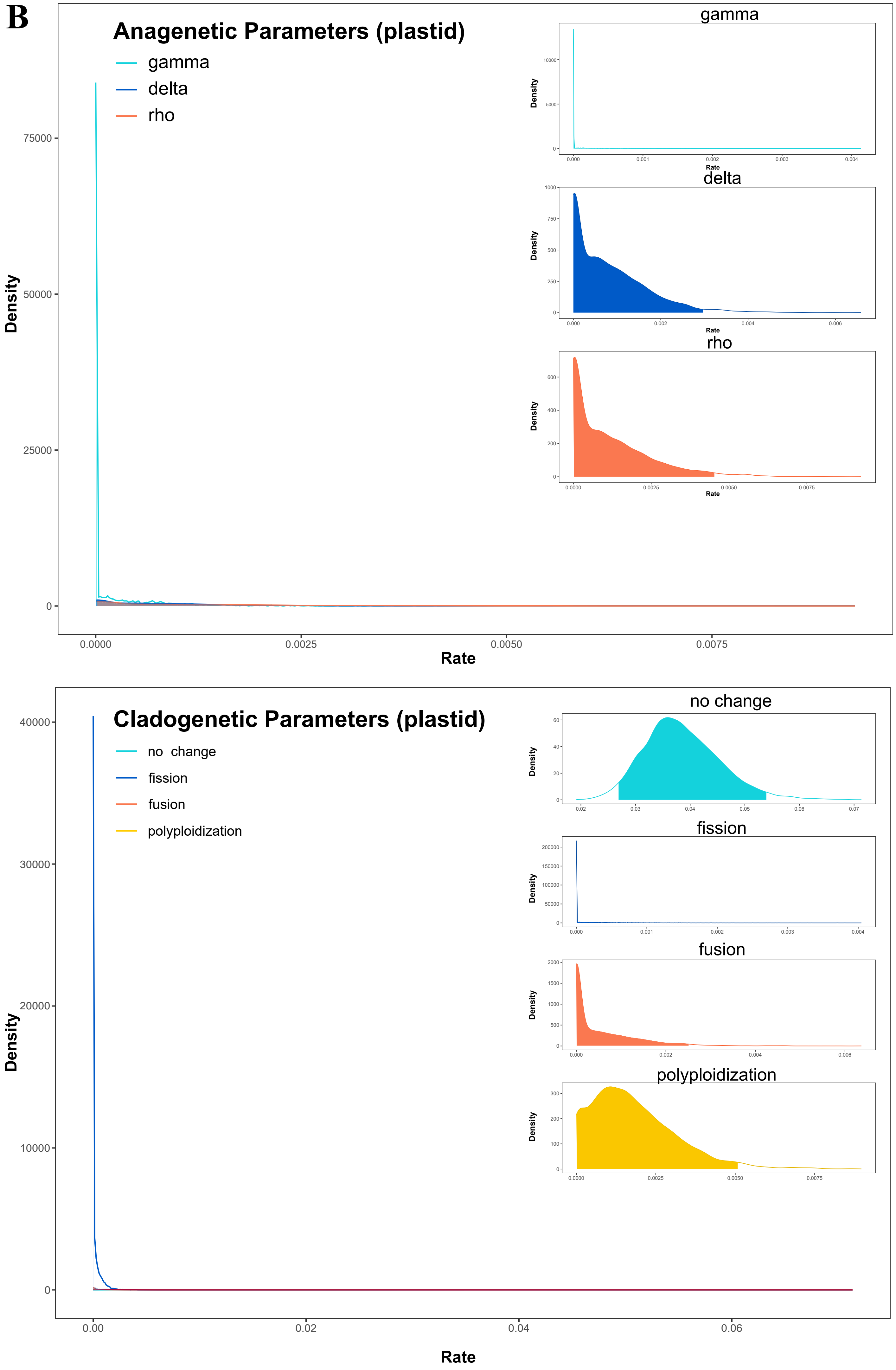
Anagenetic Parameters (plastid)
gamma
delta
rho
Density
Rate
0.0000
0.0025
0.0050
0.0075
0
25000
50000
75000
gamma
delta
rho
Cladogenetic Parameters (plastid)
no change
fission
fusion
polyploidization
Density
Rate
0.00
0.02
0.04
0.06
0
10000
20000
30000
40000
no change
fission
fusion
polyploidization
