# Supplementary material for: Ancient polyploidization events influence the evolution of the ginseng family (Araliaceae)
Source: Front Plant Sci. 2025 Jun 13;16:1595321. doi: 10.3389/fpls.2025.1595321 (PMC12202383; doi:10.3389/fpls.2025.1595321)

Figure S4. Plots obtained for each of the samples from nQuire results following Viruel et al. (2023). The plots from up to down are left to right are as follows: distribution of allelic frequencies, showing the number of SNPs with allelic frequencies from 20-80%; allellic ratios per SNP against the read counts obtained for each SNP; boxplot summarizing allelic ratio values for all the SNPs; density plot of allelic ratios for all the SNPs; boxplots summarizing allelic ratio values per locus providing the median values per locus (blue lines). Red lines indicate the allelic ratio of 2, as diploid samples are expected to present median allelic ratios <2 (Viruel et al., 2019).

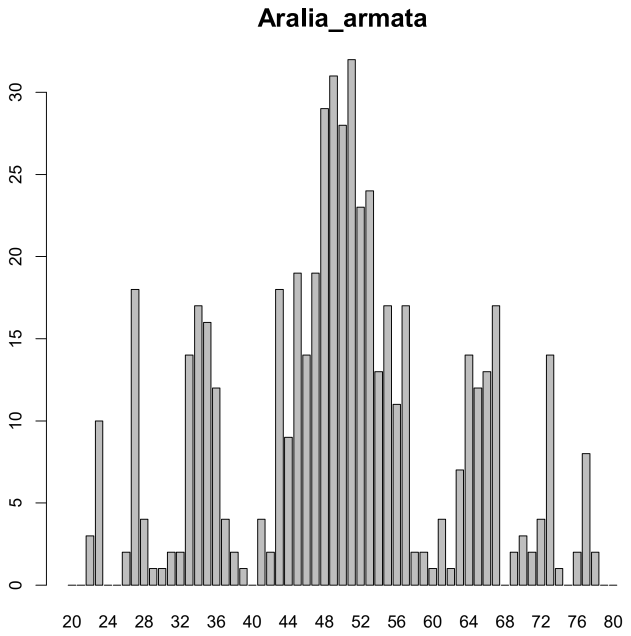
Aralia_armata
0
5
10
15
20
25
30
20 24 28 32 36 40 44 48 52 56 60 64 68 72 76 80

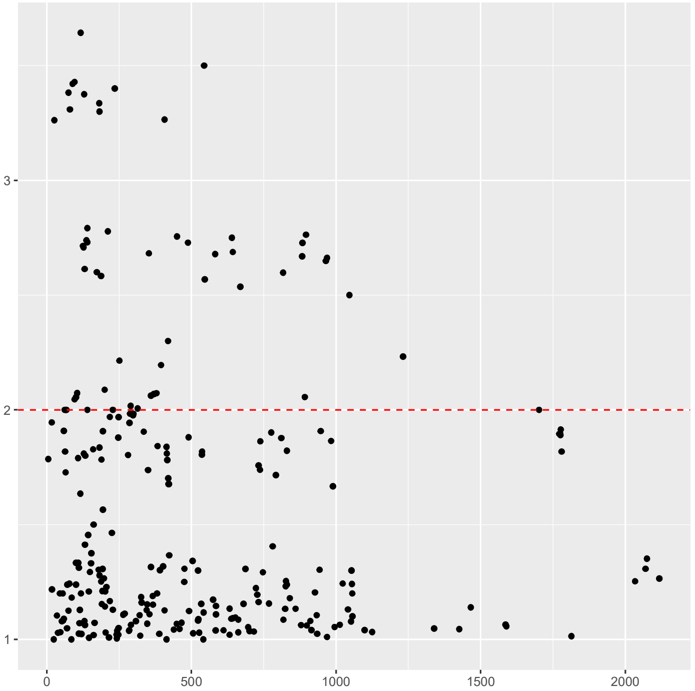
1
2
3
0 500 1000 1500 2000

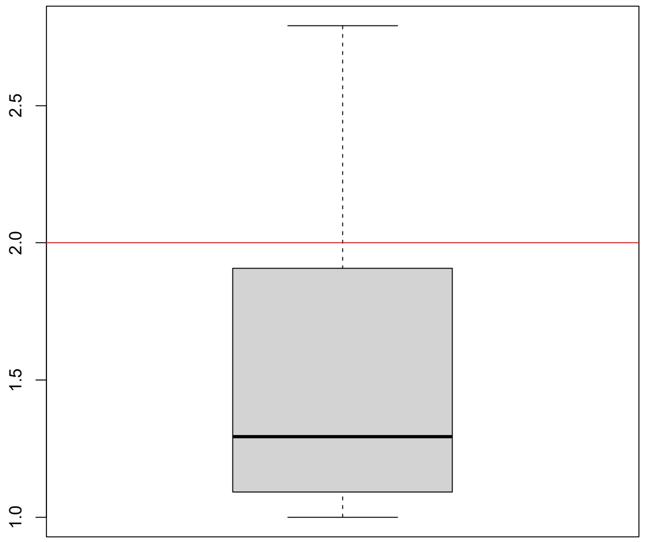
1.0
1.5
2.0
2.5

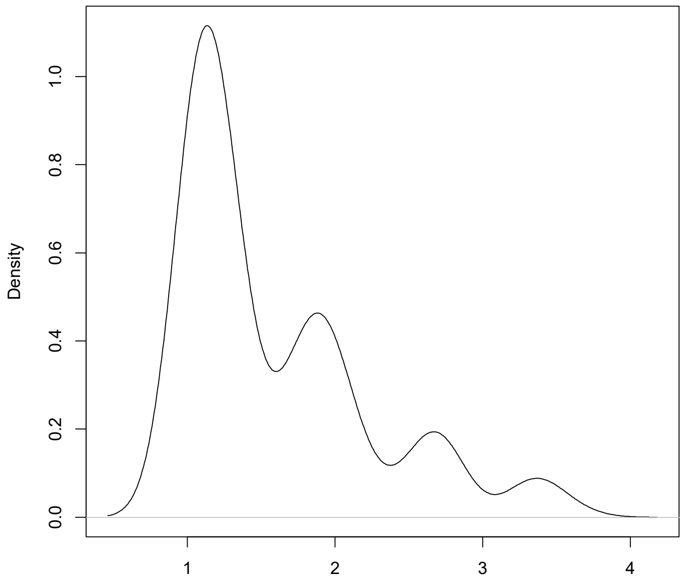
Density
0.0
0.2
0.4
0.6
0.8
1.0
1 2 3 4

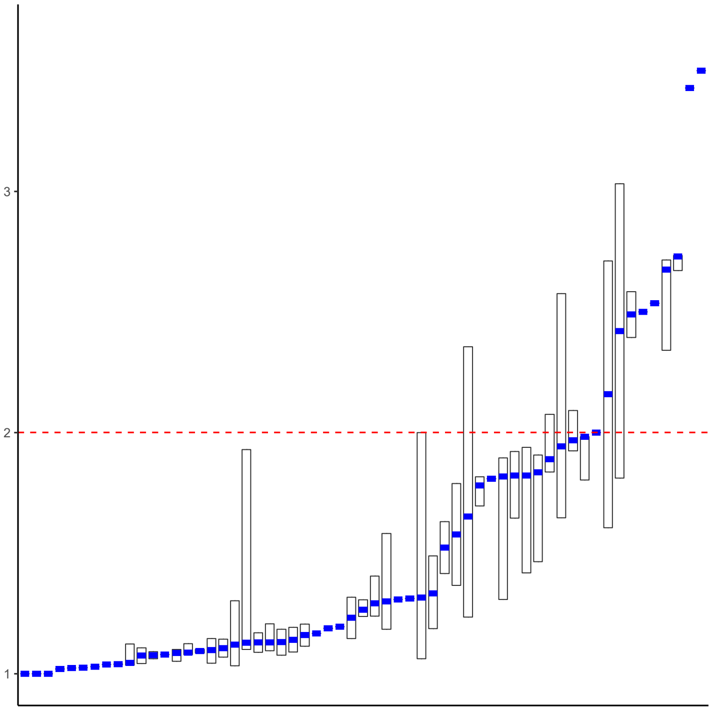
1
2
3

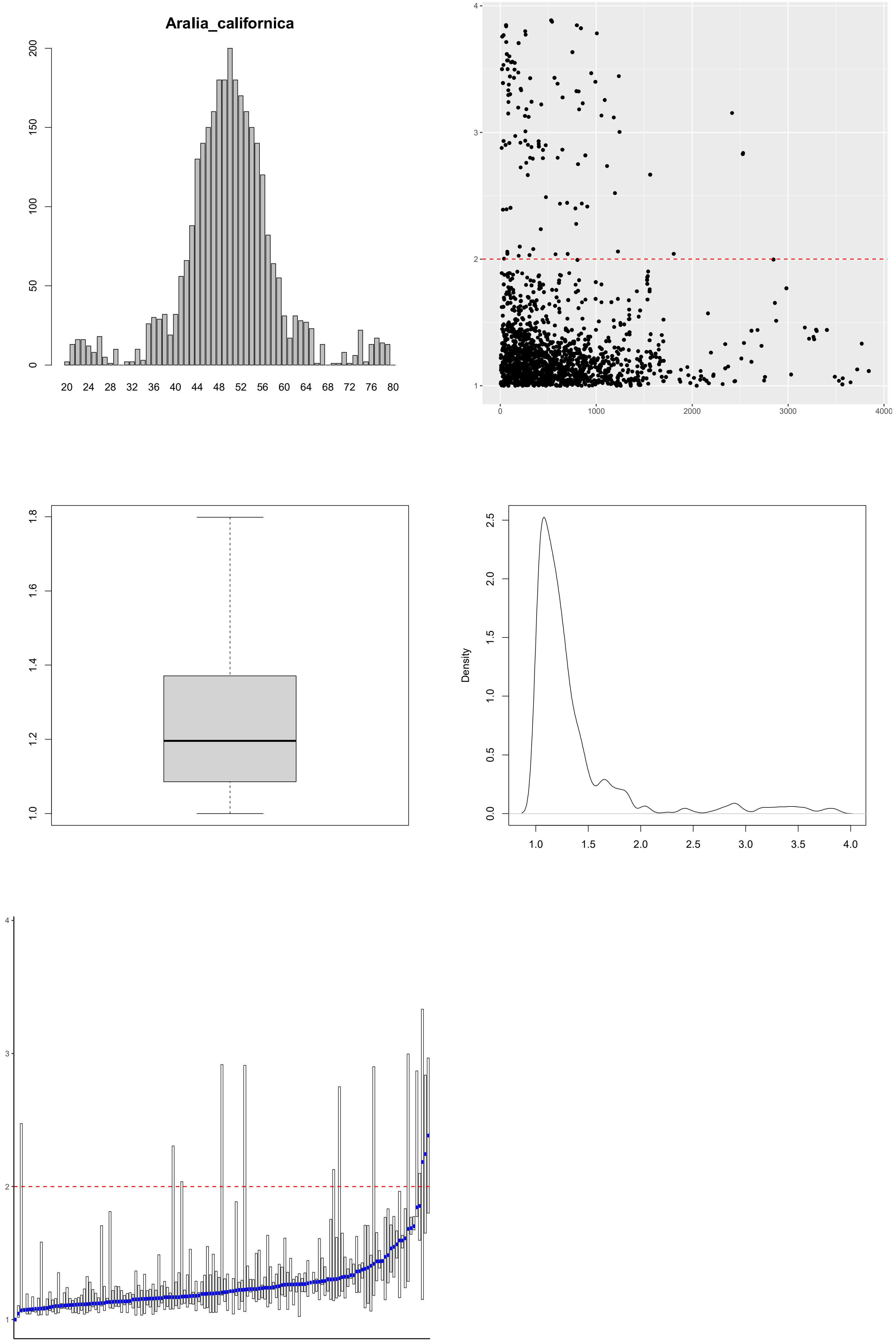
Aralia_californica
0
50
100
150
200
20 24 28 32 36 40 44 48 52 56 60 64 68 72 76 80
1
2
3
4
0 1000 2000 3000 4000
1.0
1.2
1.4
1.6
1.8
Density
0.0
0.5
1.0
1.5
2.0
2.5
1.0 1.5 2.0 2.5 3.0 3.5 4.0
1
2
3
4

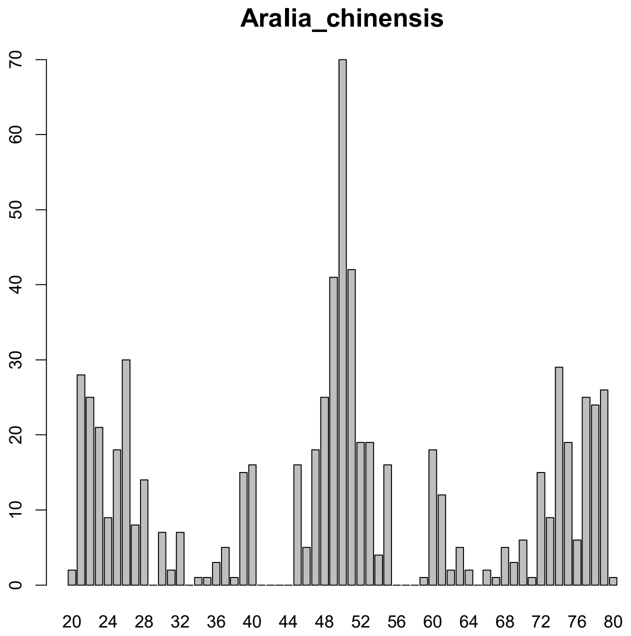
Aralia_chinensis

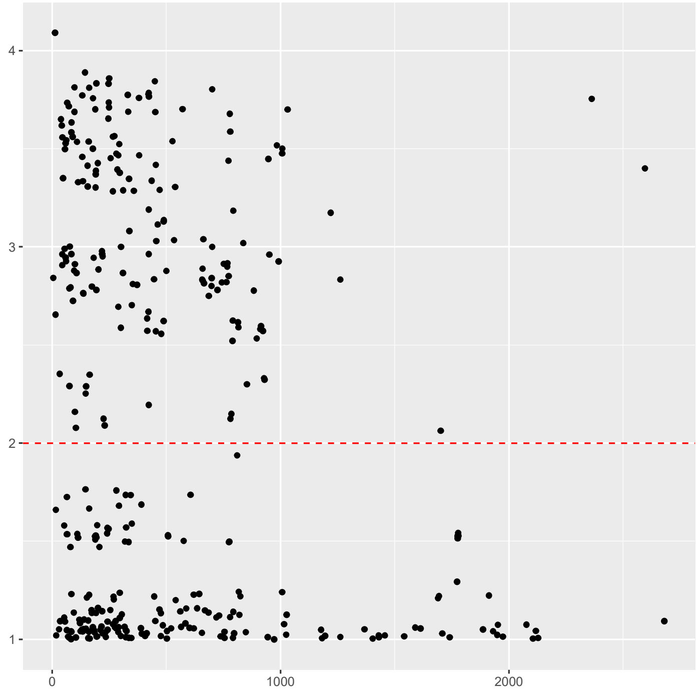

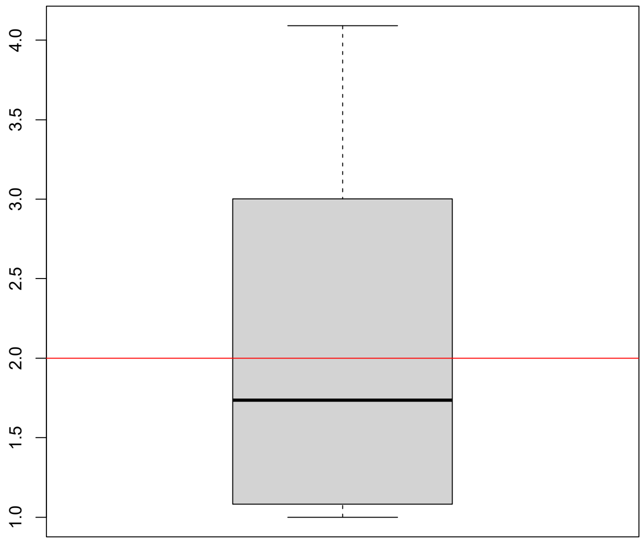

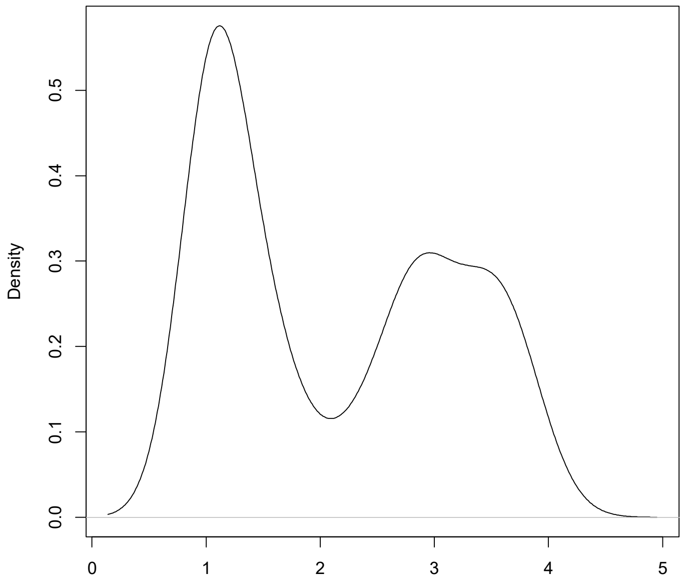
Density

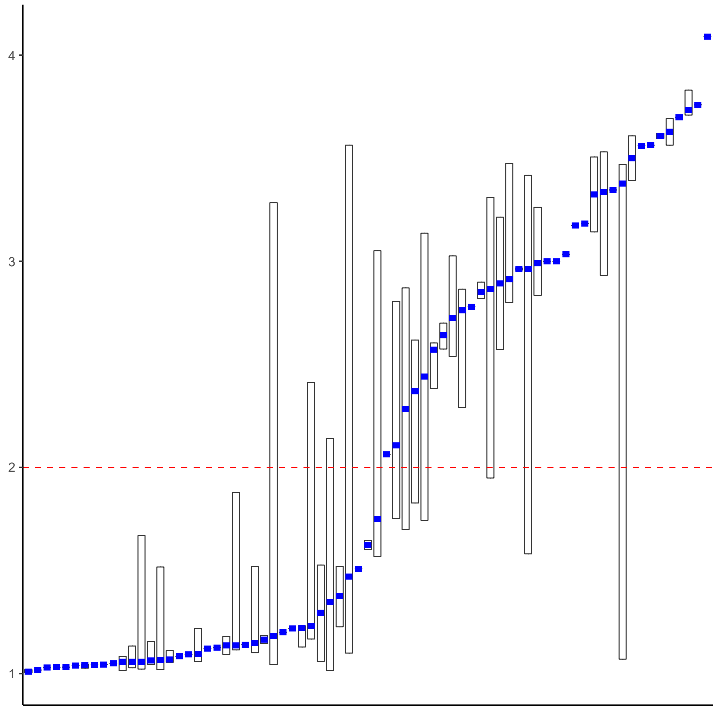

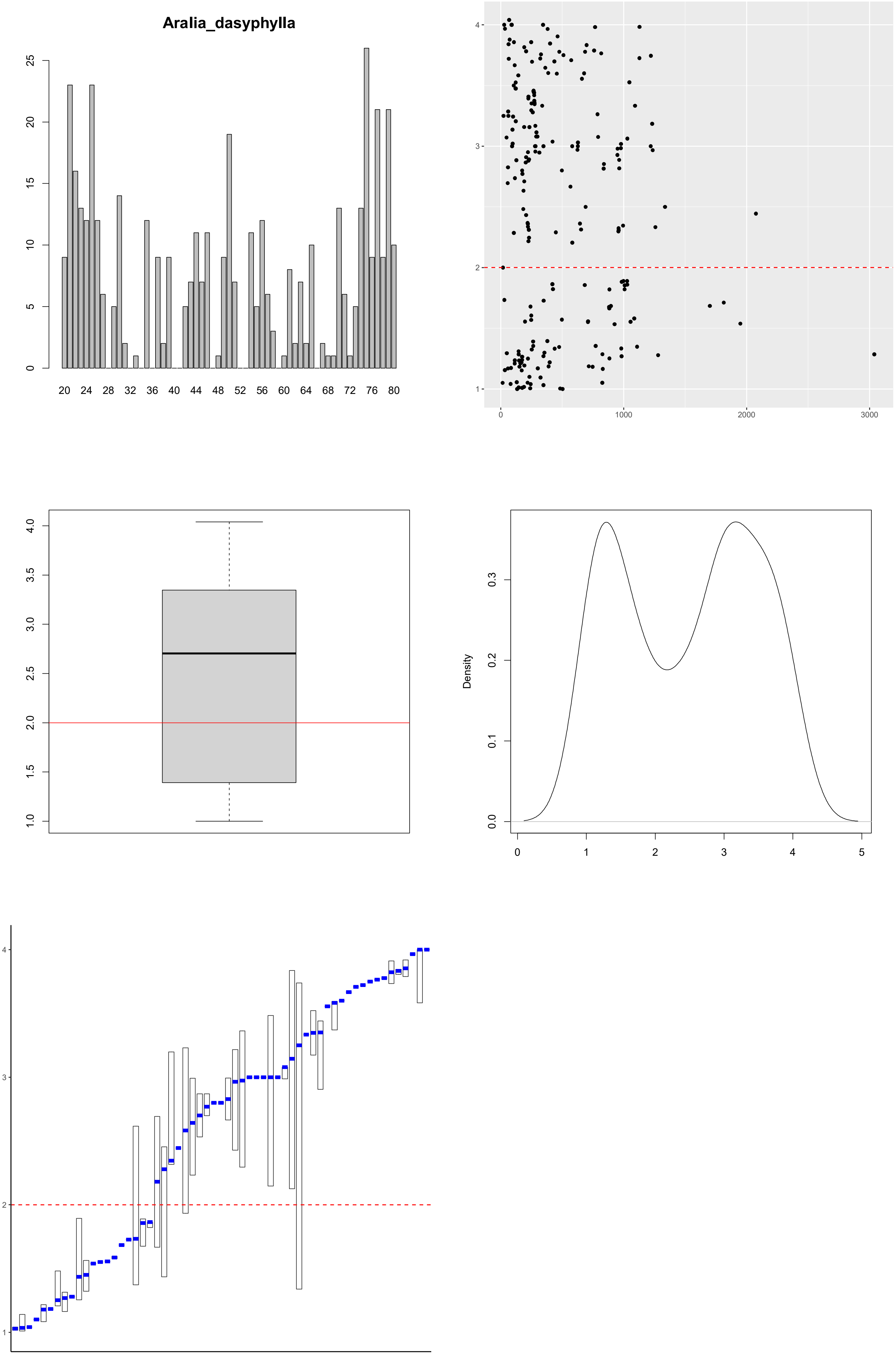

Aralia_dasyphylla
Density

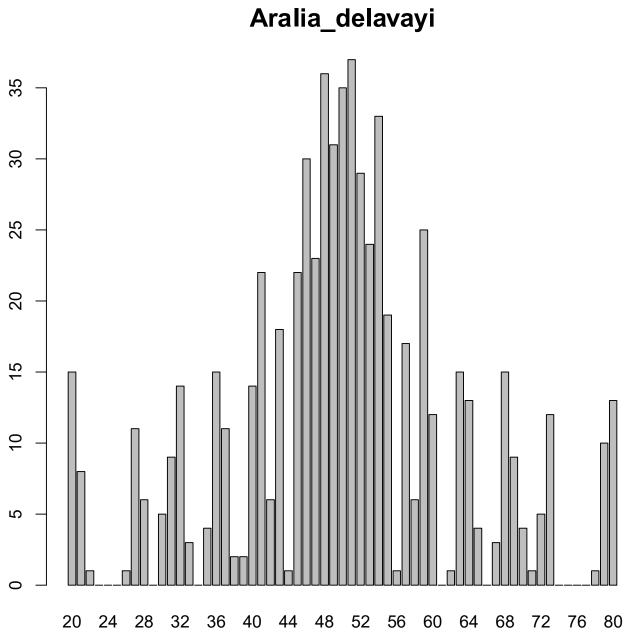
Aralia_delavayi
0
5
10
15
20
25
30
35
20 24 28 32 36 40 44 48 52 56 60 64 68 72 76 80

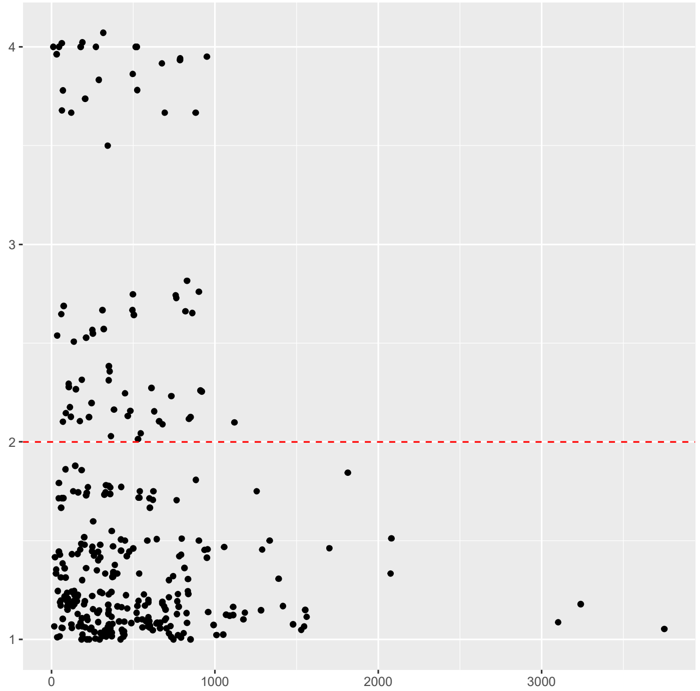
1
2
3
4
0
1000
2000
3000

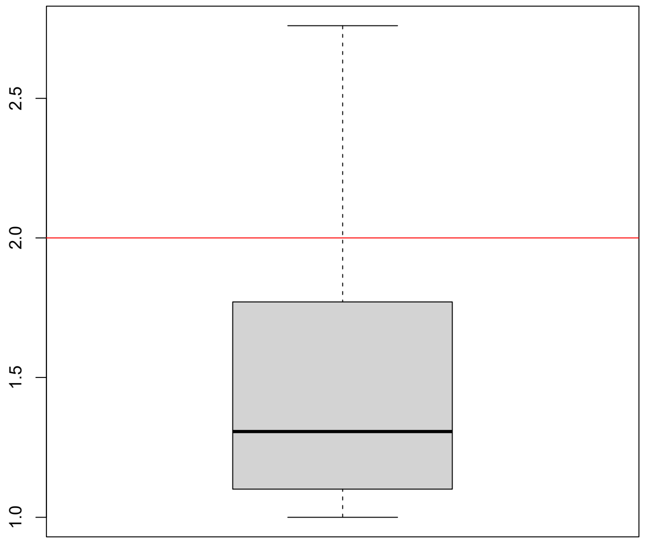
1.0
1.5
2.0
2.5

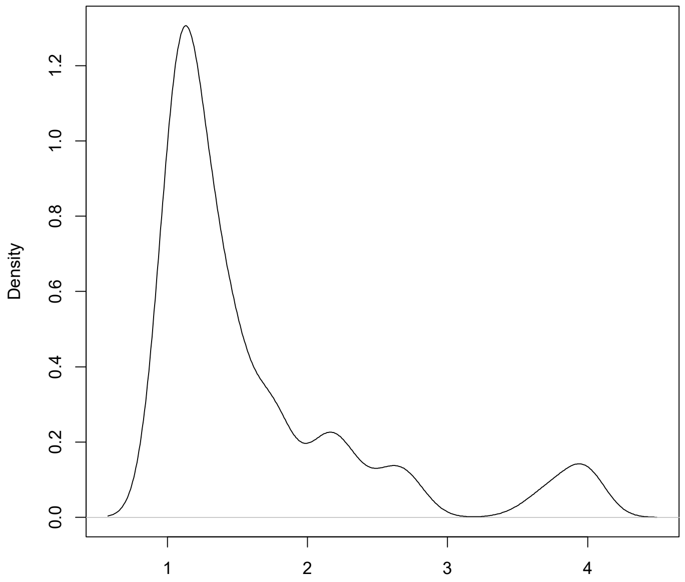
Density
0.0
0.2
0.4
0.6
0.8
1.0
1.2
1 2 3 4

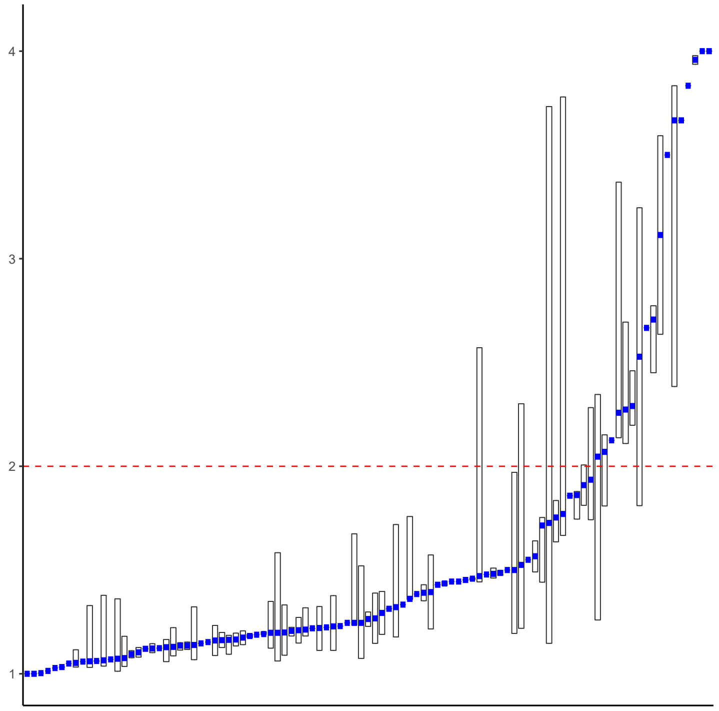
1
2
3
4

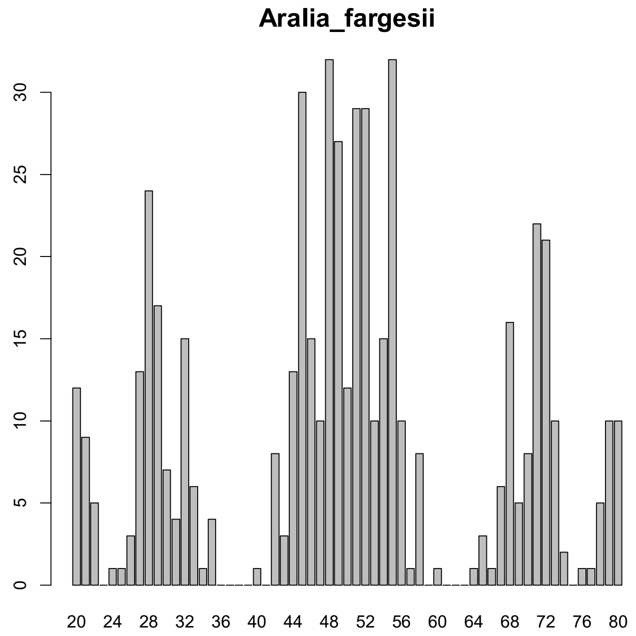
Aralia_fargesii

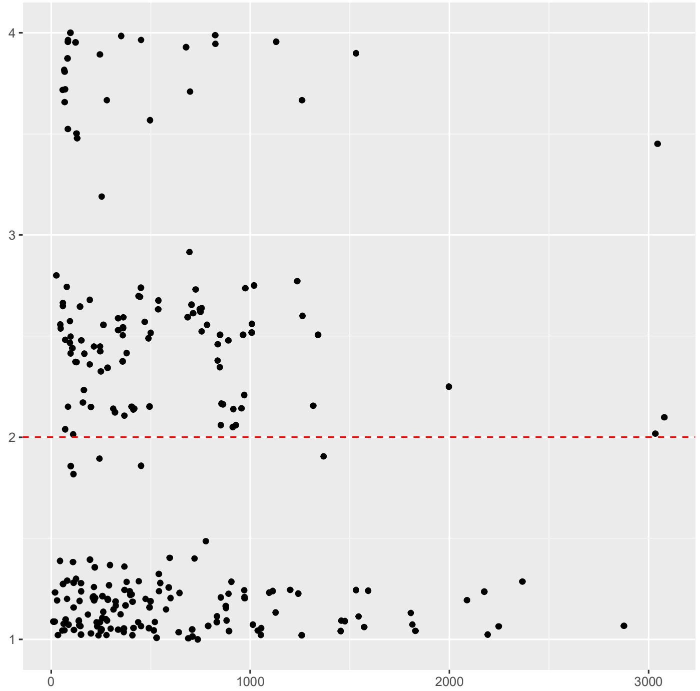

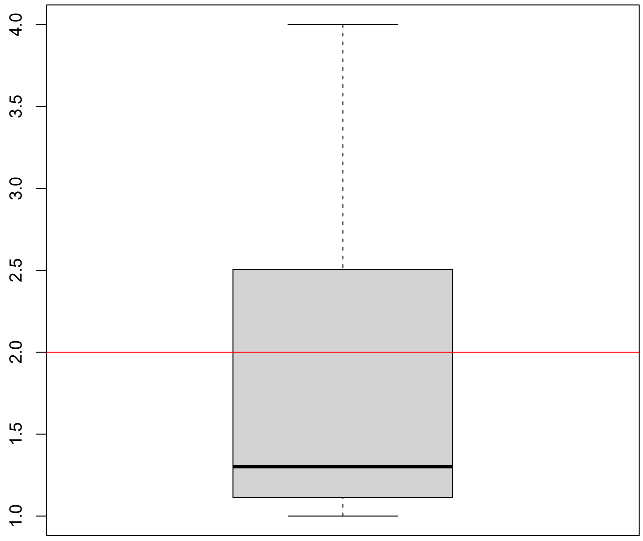

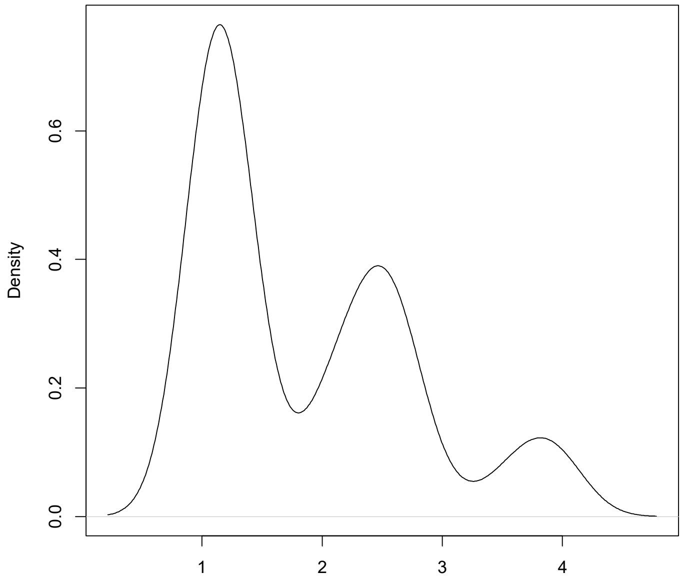
Density

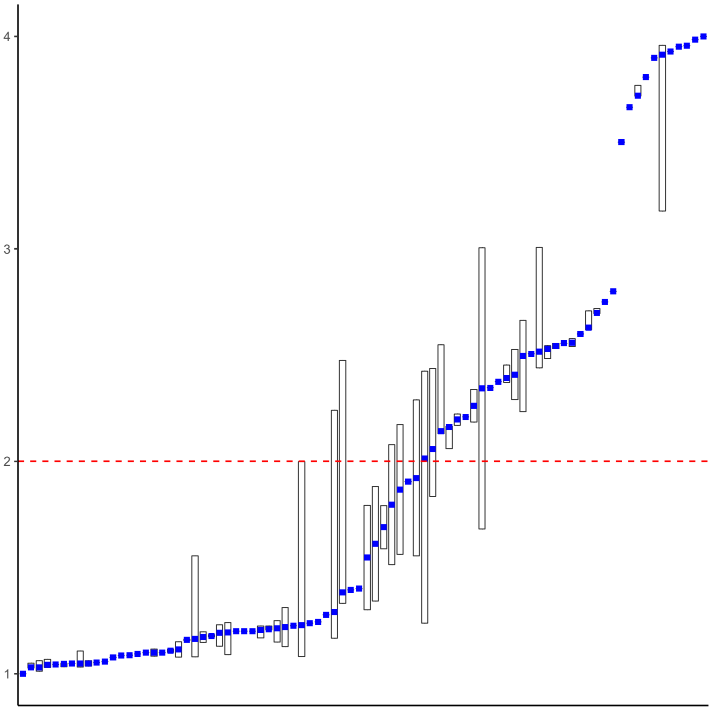

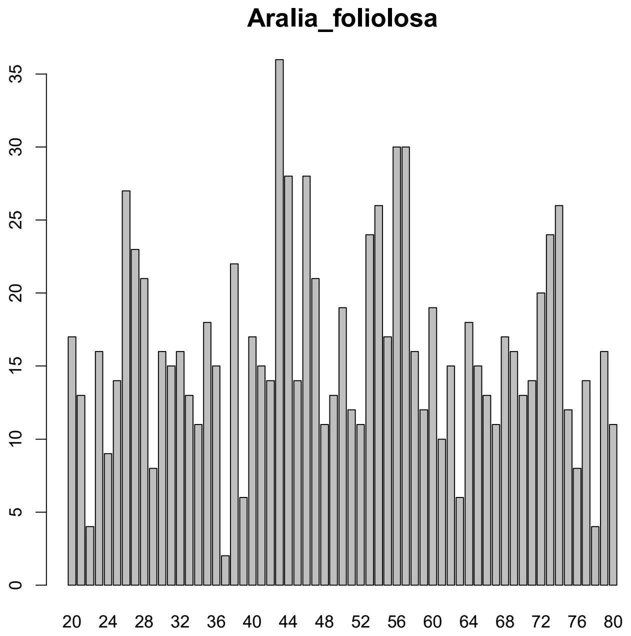
Aralia_foliolosa
0
5
10
15
20
25
30
35
20 24 28 32 36 40 44 48 52 56 60 64 68 72 76 80

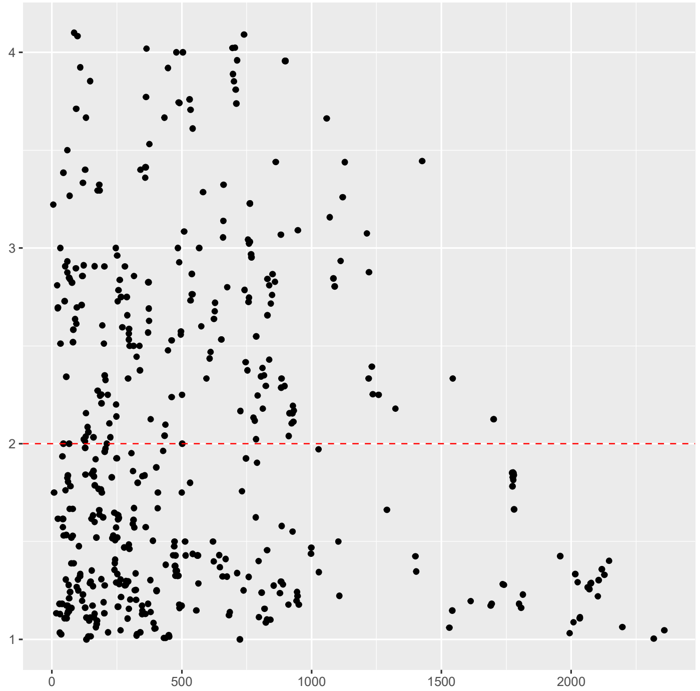
1
2
3
4
0 500 1000 1500 2000

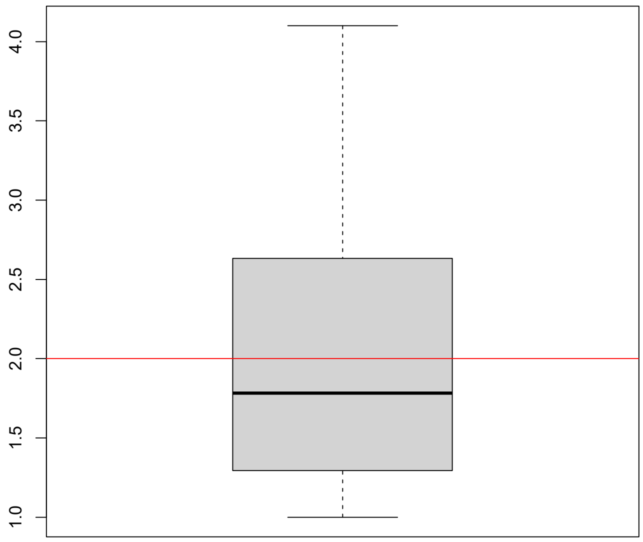
1.0
1.5
2.0
2.5
3.0
3.5
4.0

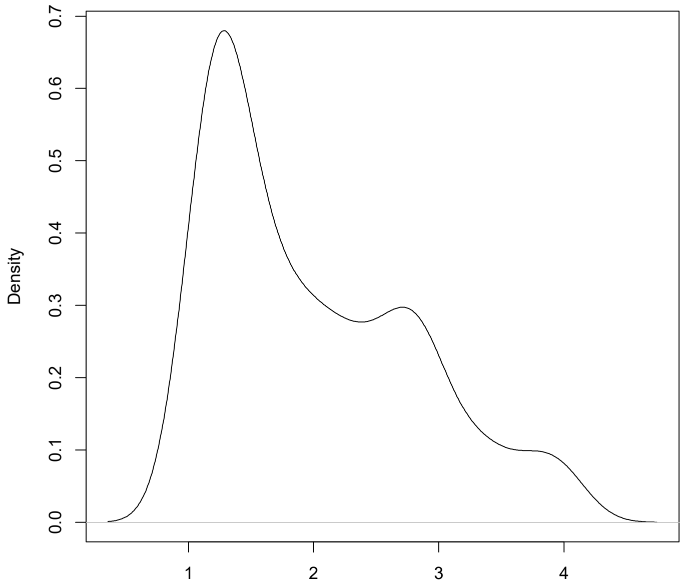
Density
0.0
0.1
0.2
0.3
0.4
0.5
0.6
0.7
1 2 3 4

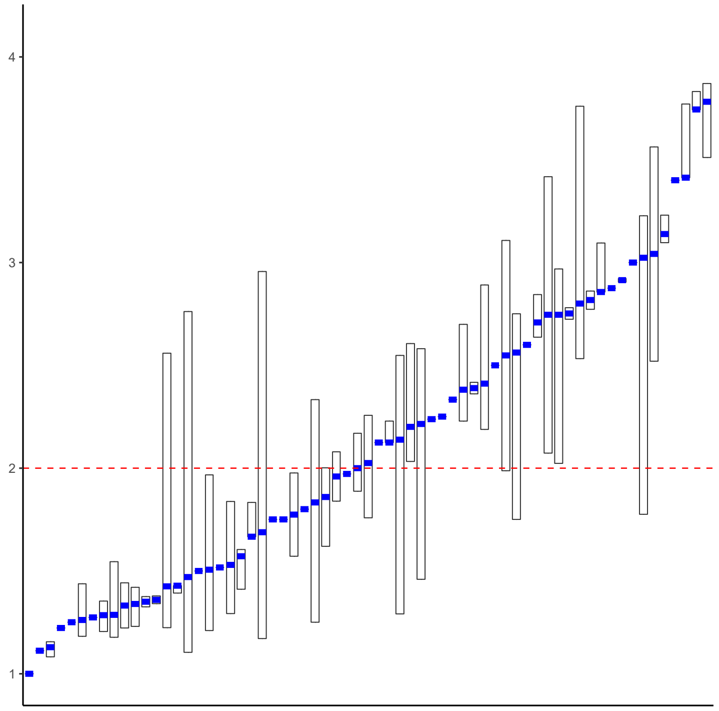
1
2
3
4

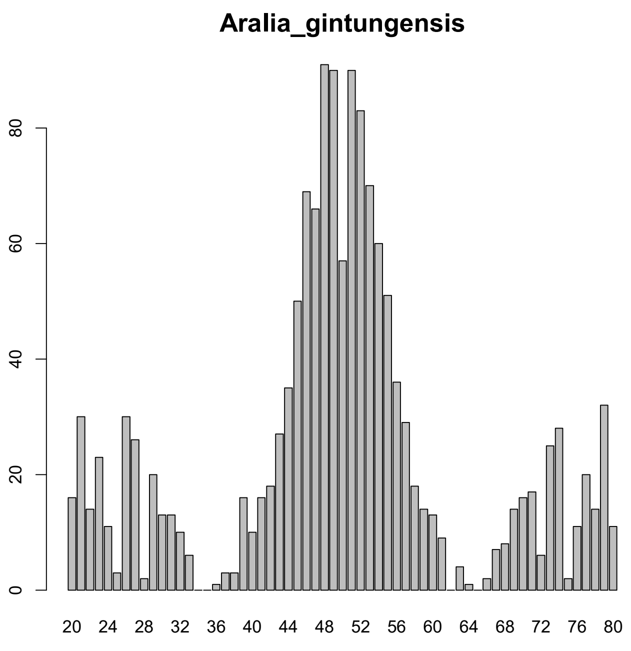
Aralia_gintungensis

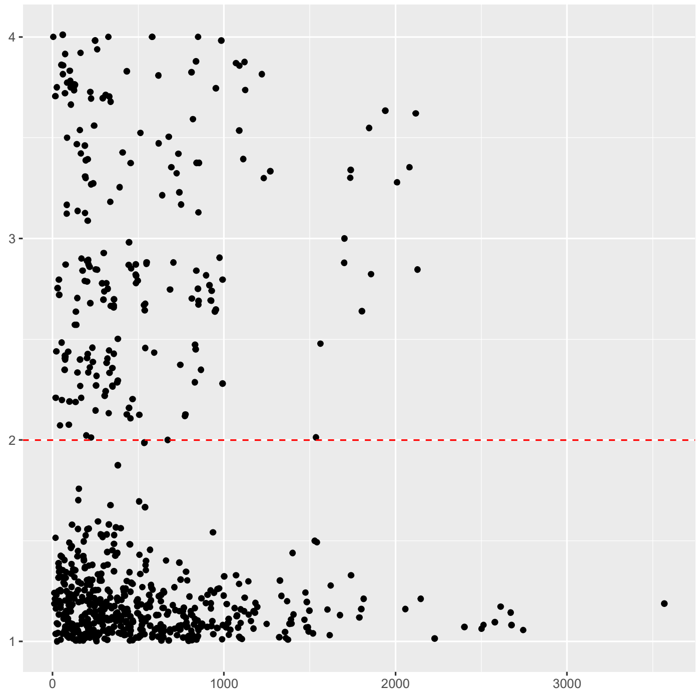

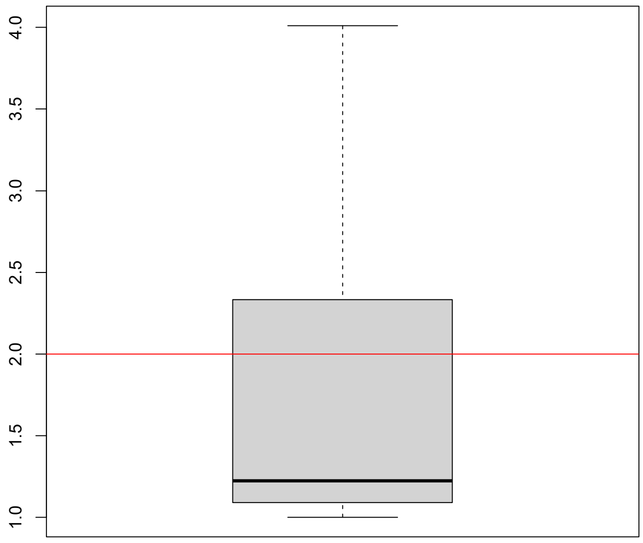

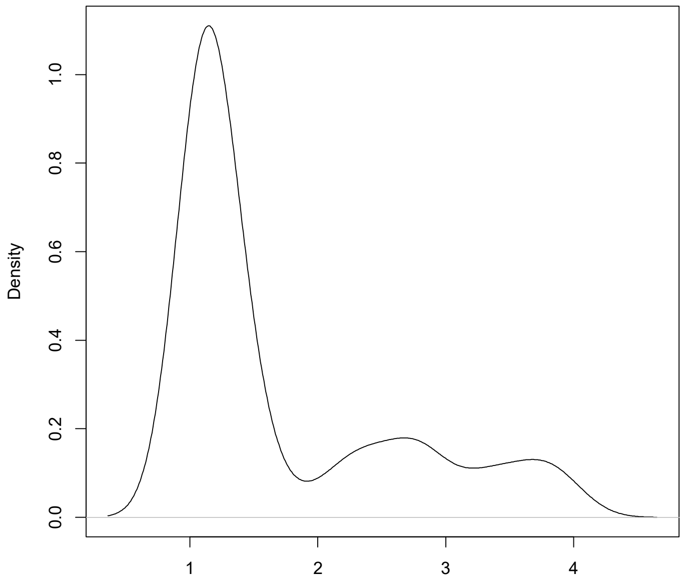
Density

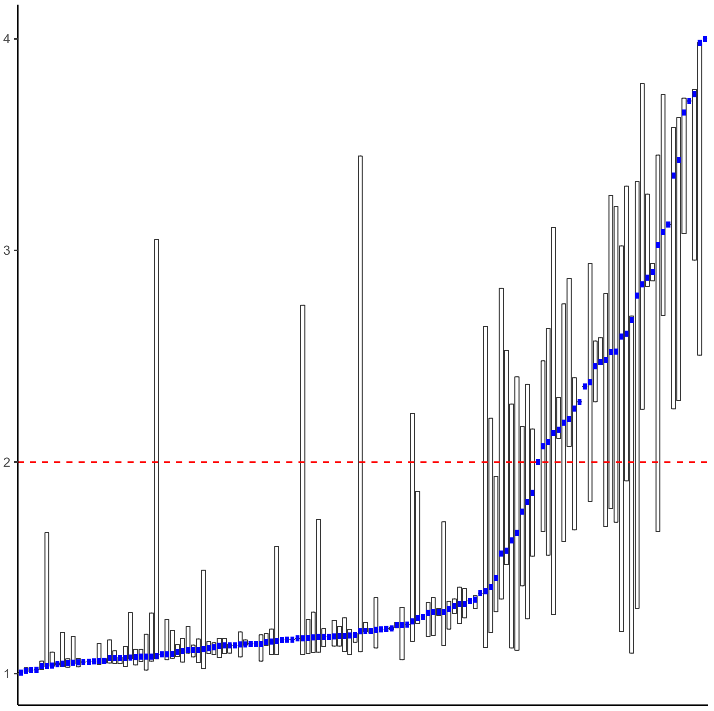

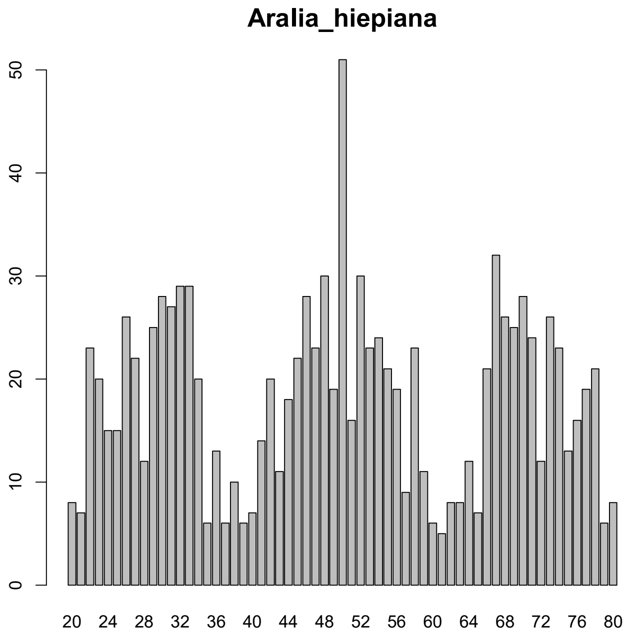
Aralia_hiepiana
0 10 20 30 40 50
20 24 28 32 36 40 44 48 52 56 60 64 68 72 76 80

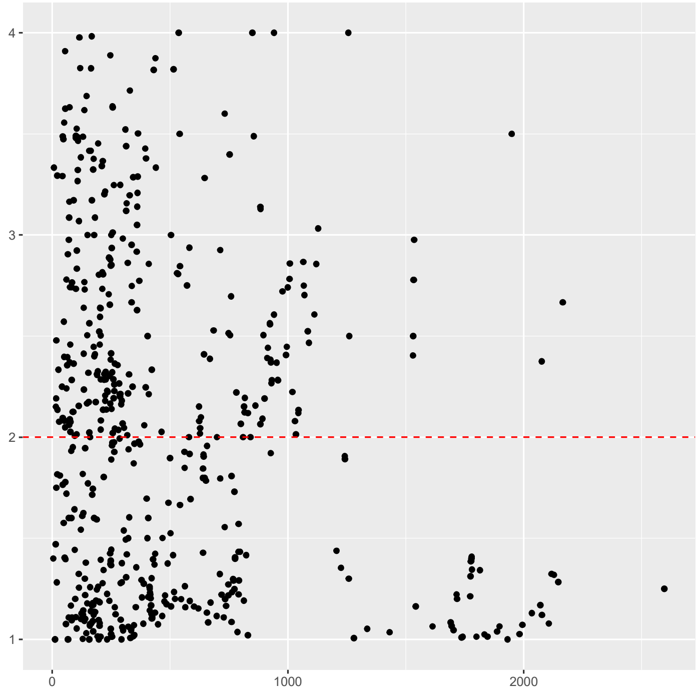
1 2 3 4
0 1000 2000

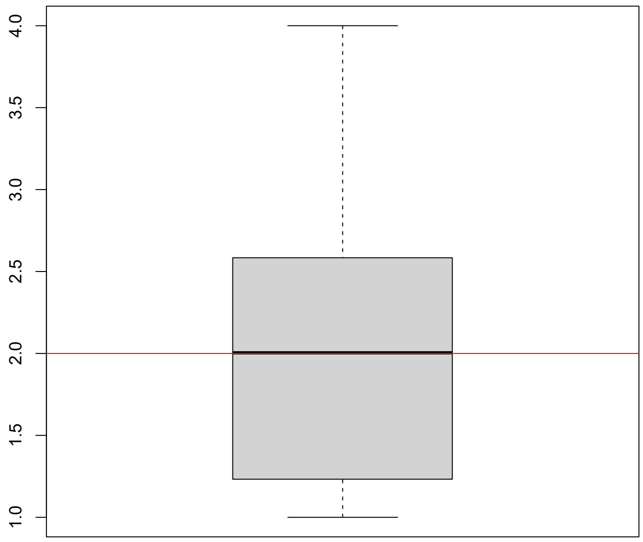
1.0 1.5 2.0 2.5 3.0 3.5 4.0

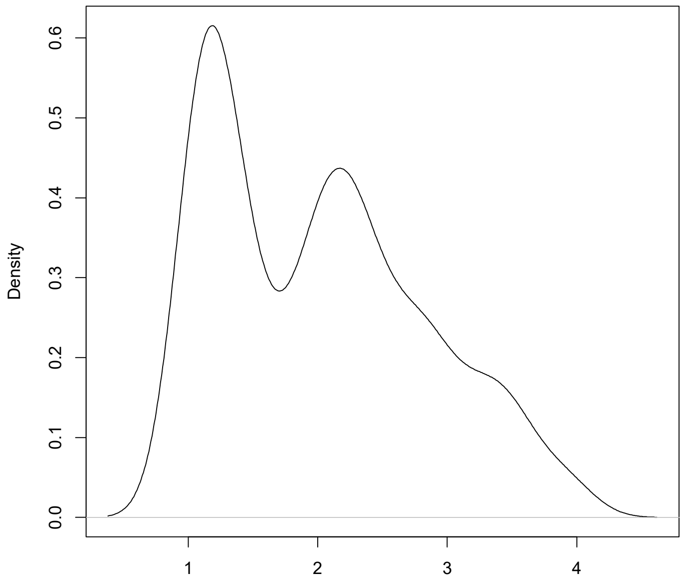
Density
0.0 0.1 0.2 0.3 0.4 0.5 0.6
1 2 3 4

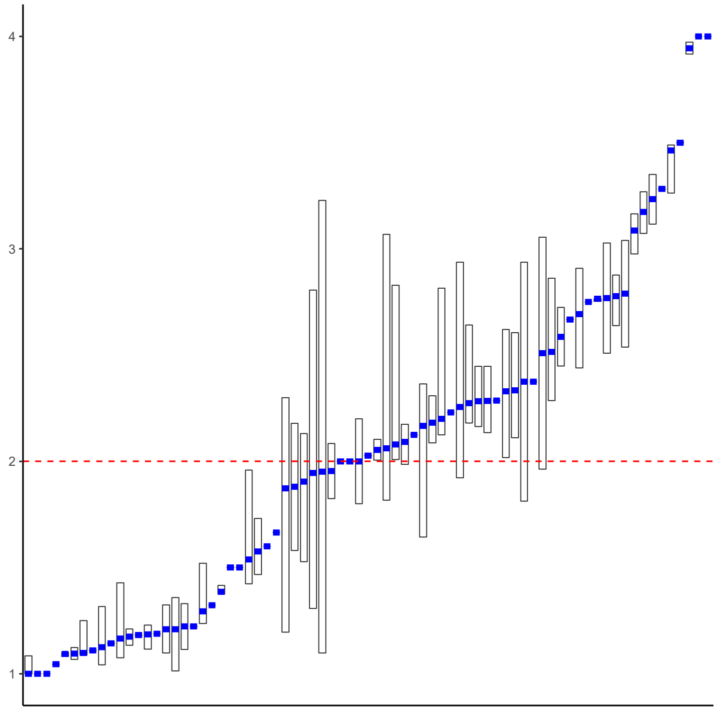
1 2 3 4

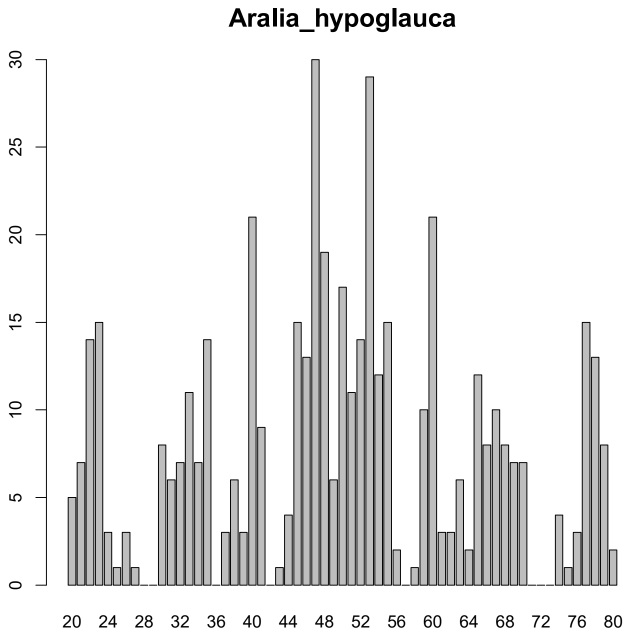
Aralia_hypoglauca

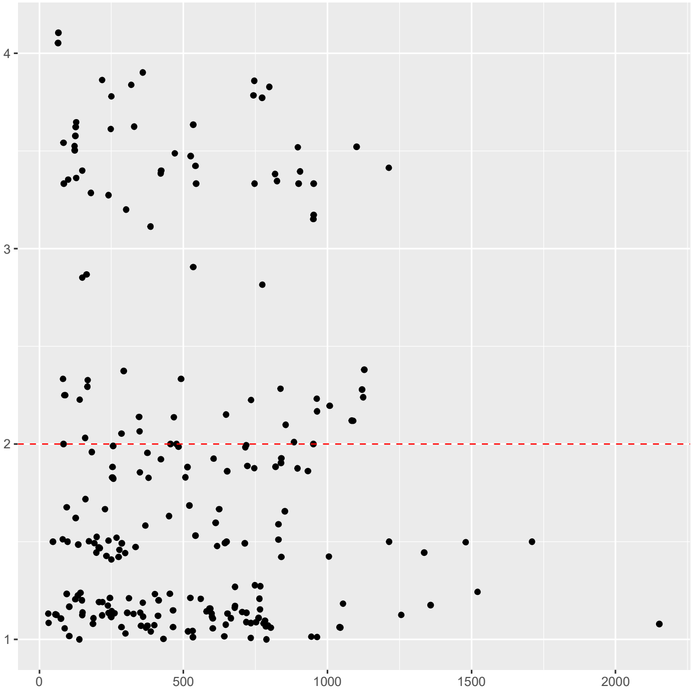

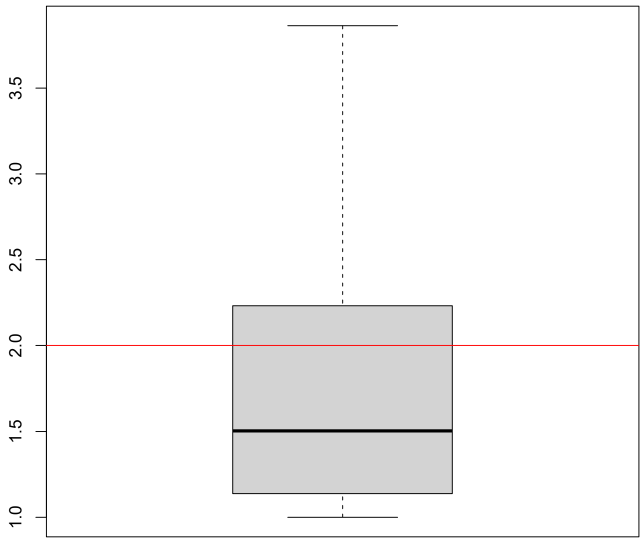

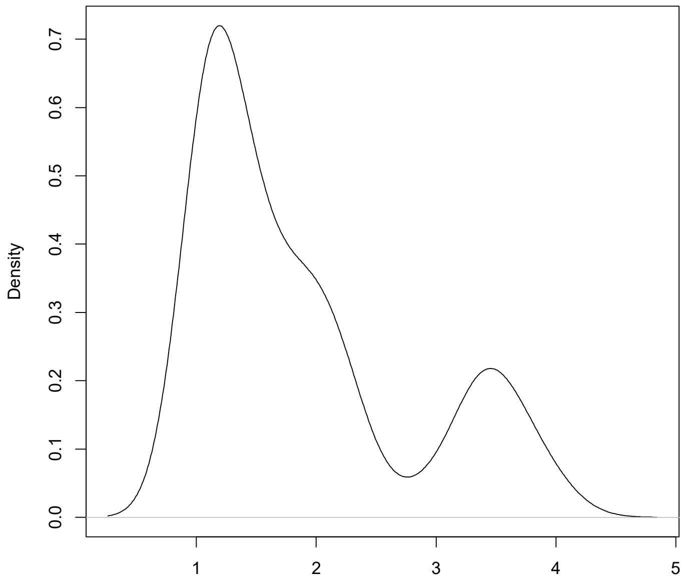
Density

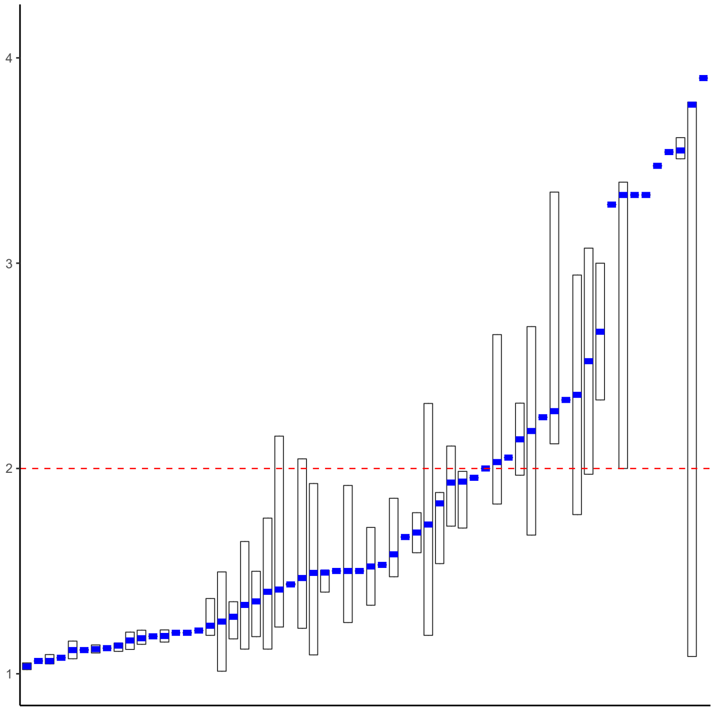

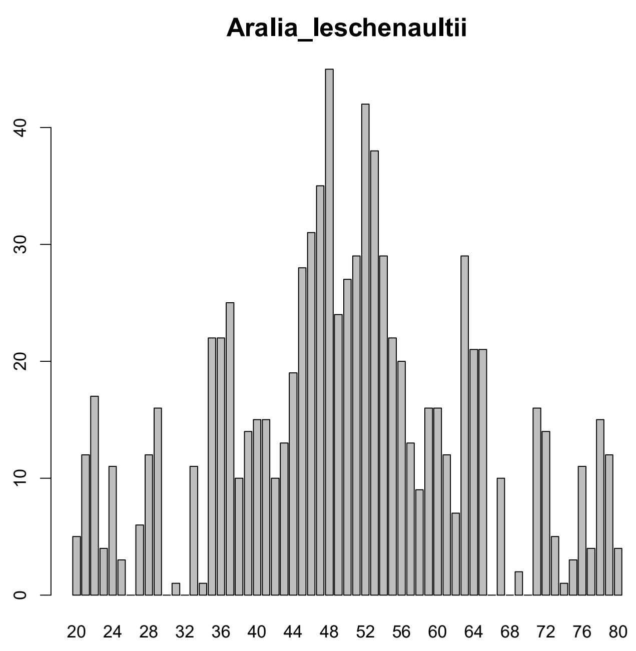
Aralia_leschenaultii
0
10
20
30
40
20 24 28 32 36 40 44 48 52 56 60 64 68 72 76 80

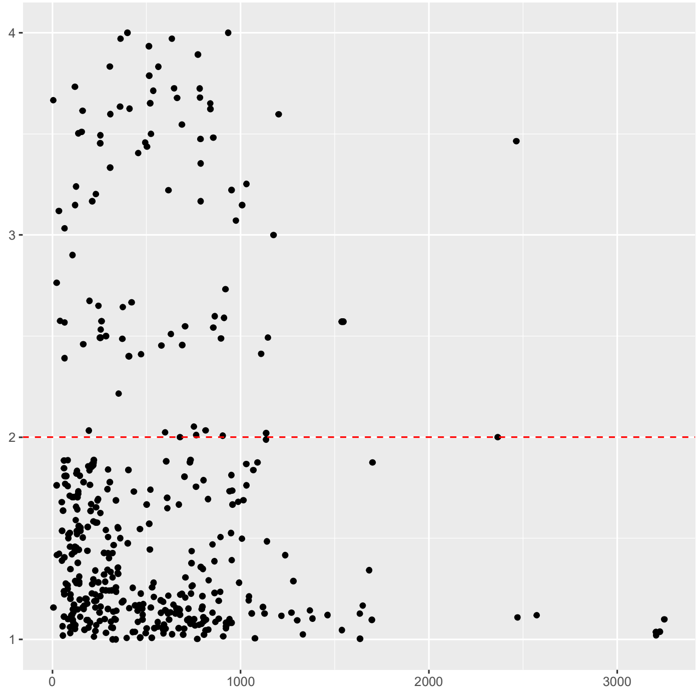
1
2
3
4
0 1000 2000 3000

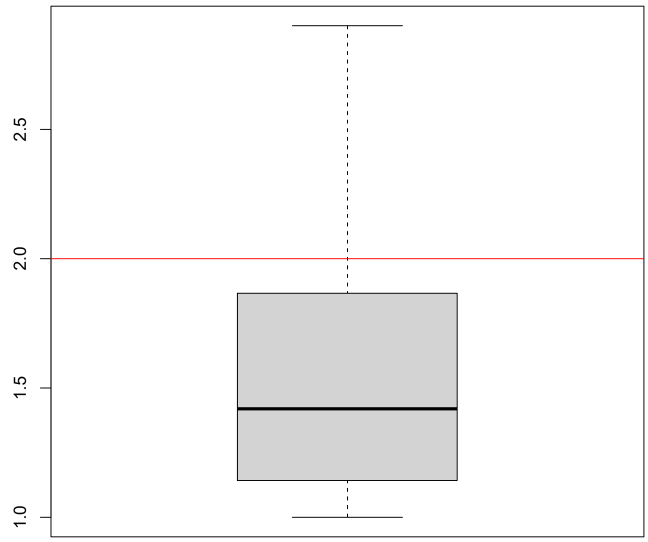
1.0
1.5
2.0
2.5

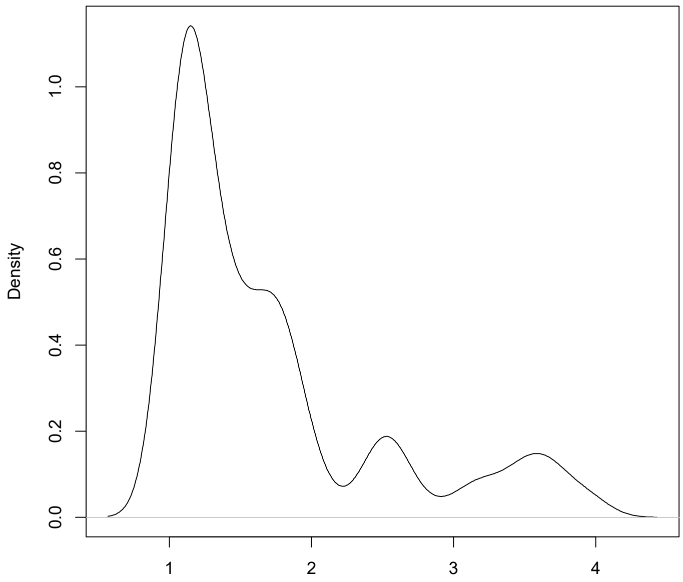
Density
0.0
0.2
0.4
0.6
0.8
1.0
1 2 3 4

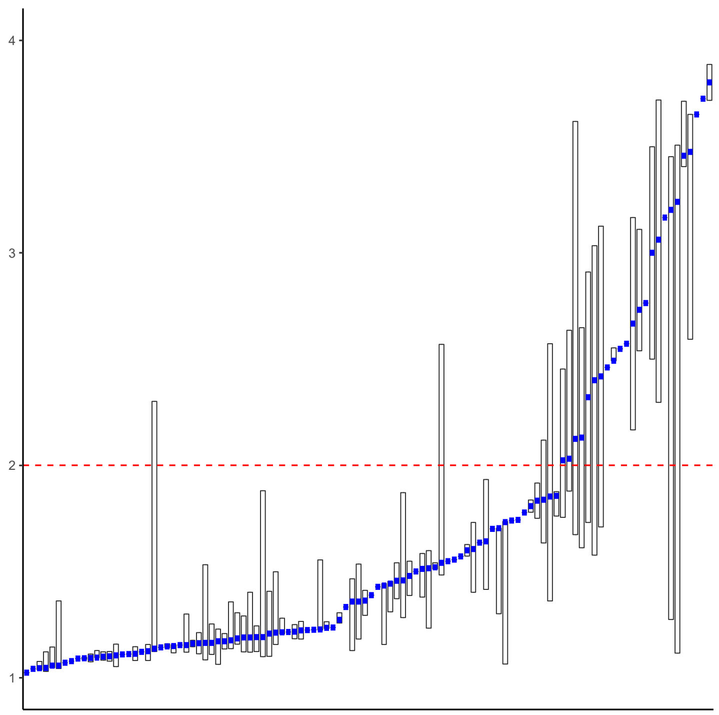
1
2
3
4

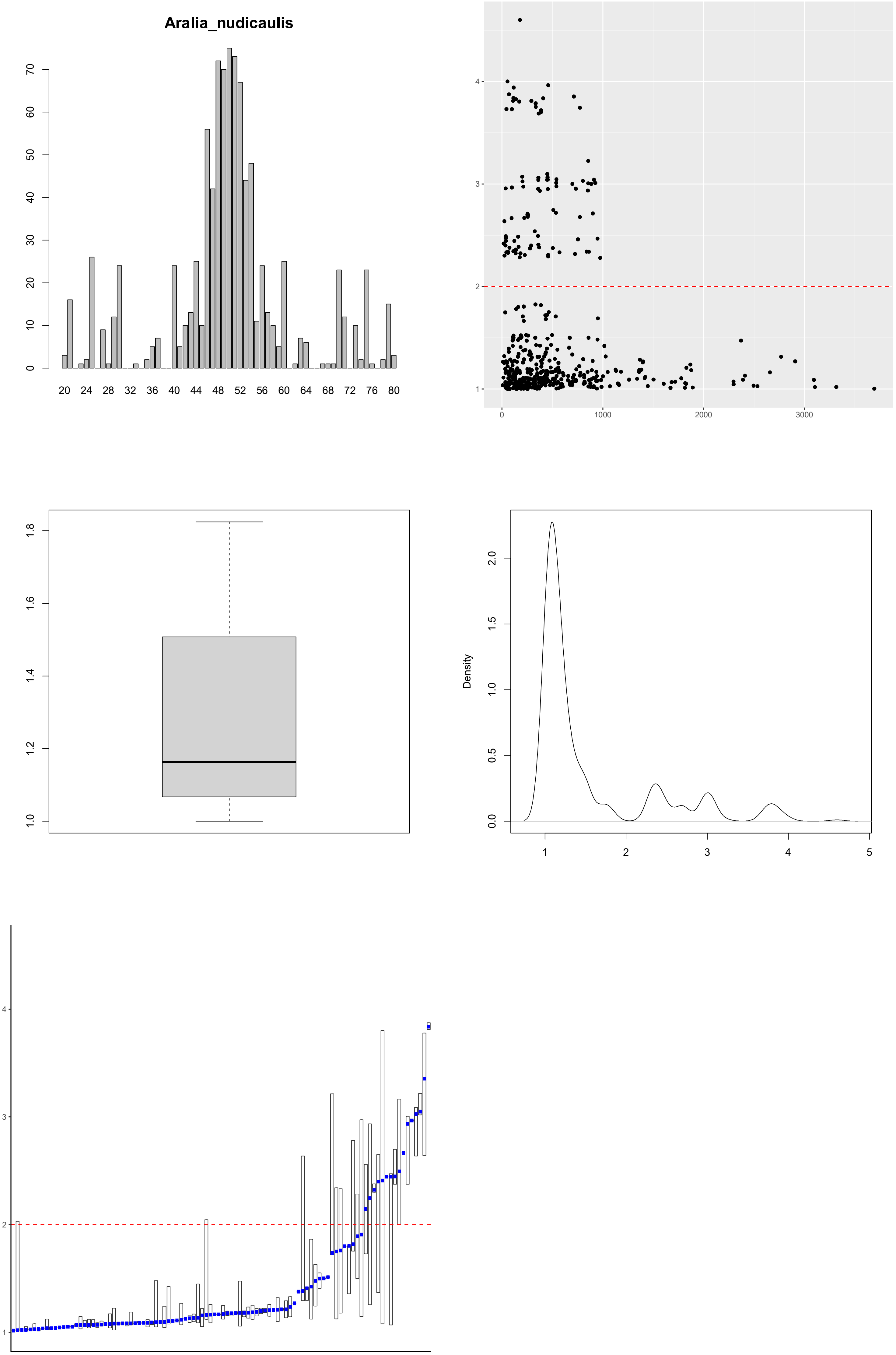

Aralia_nudicaulis
Density

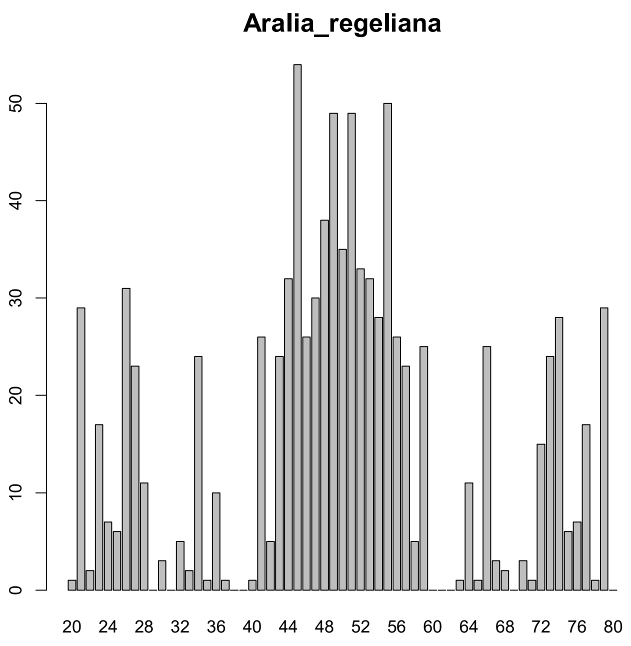
Aralia_regeliana

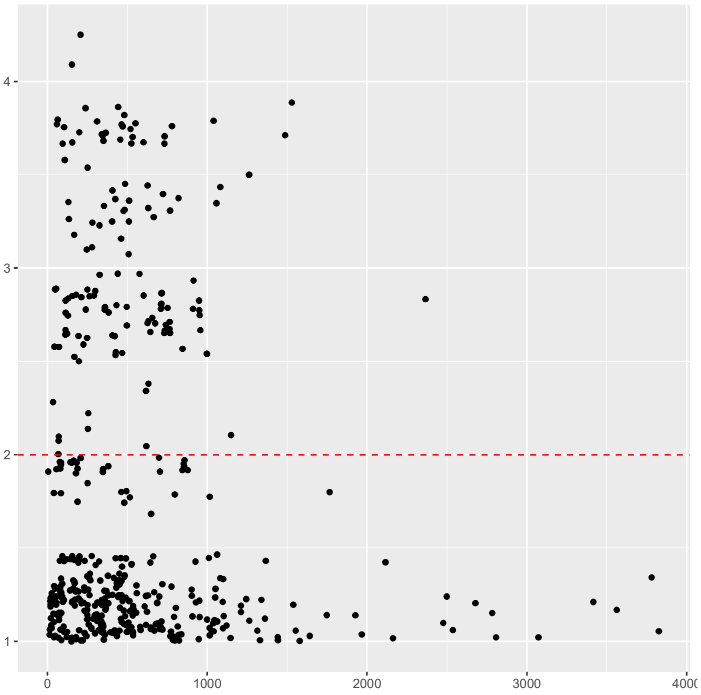

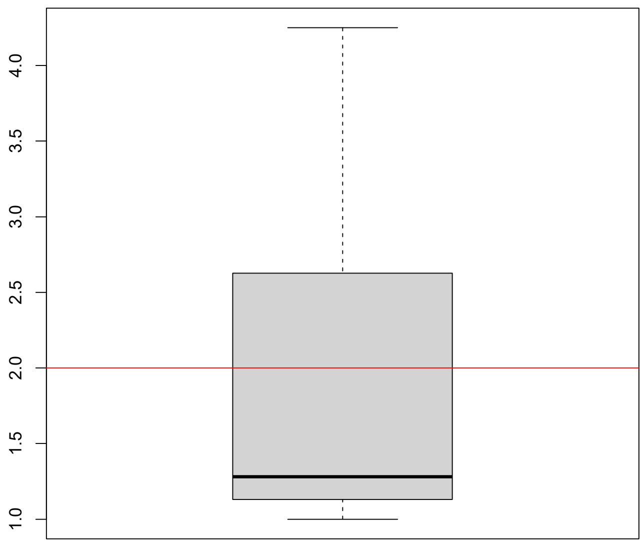

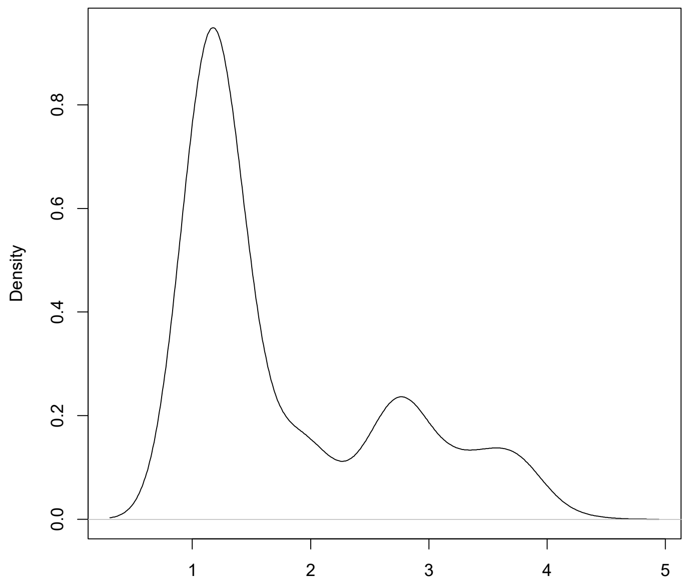
Density

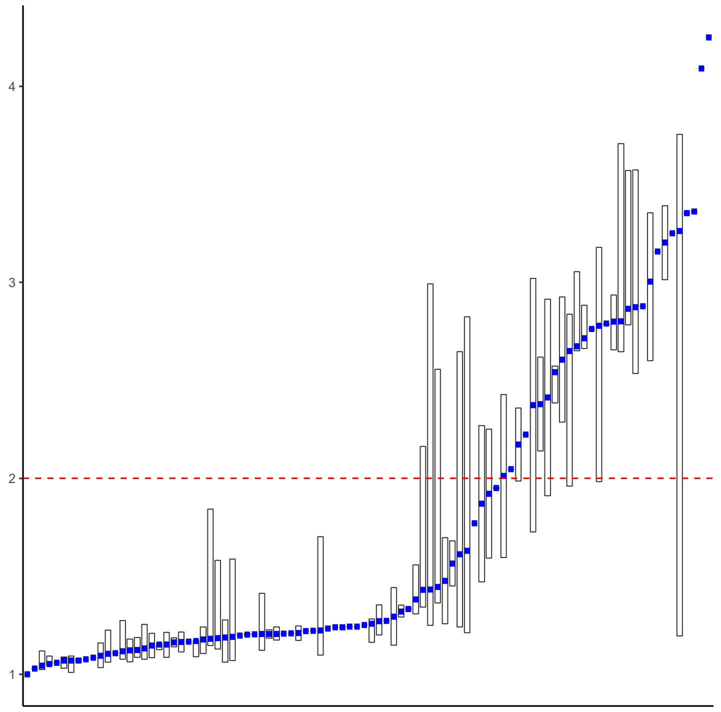

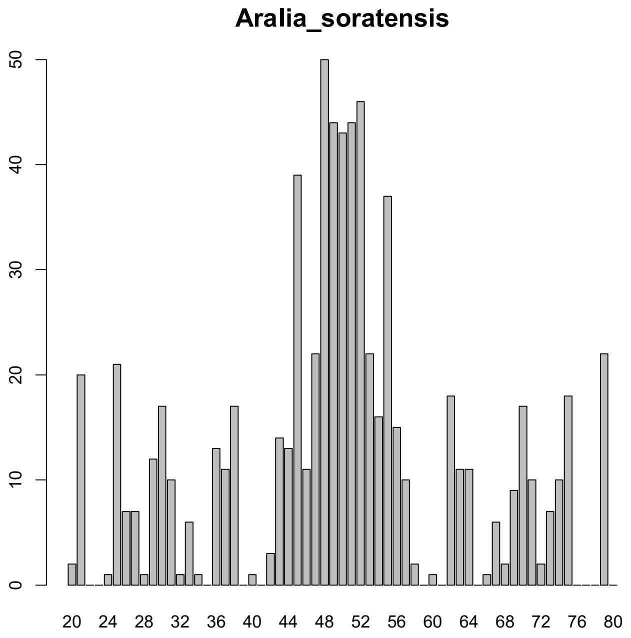
Aralia_soratensis

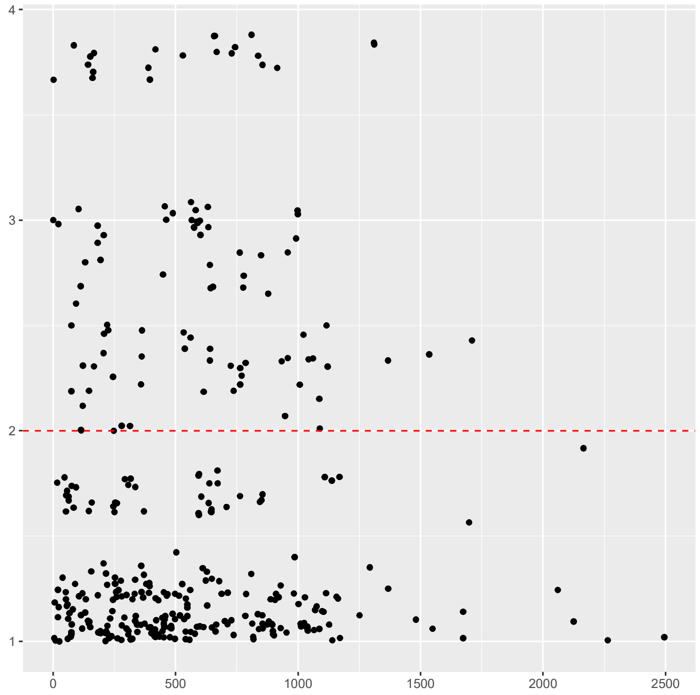

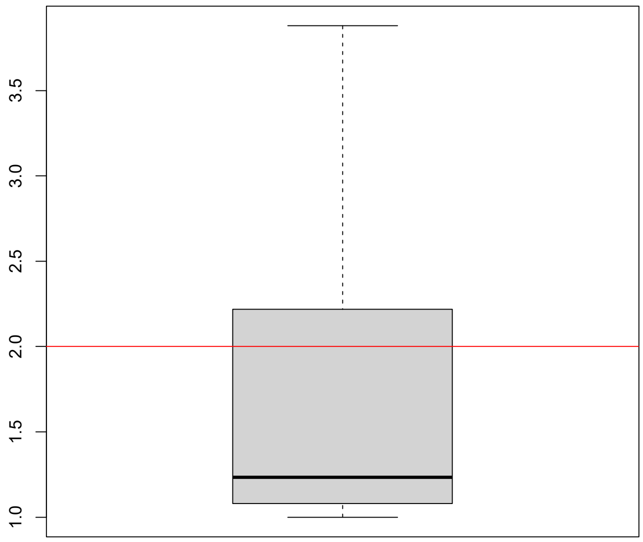

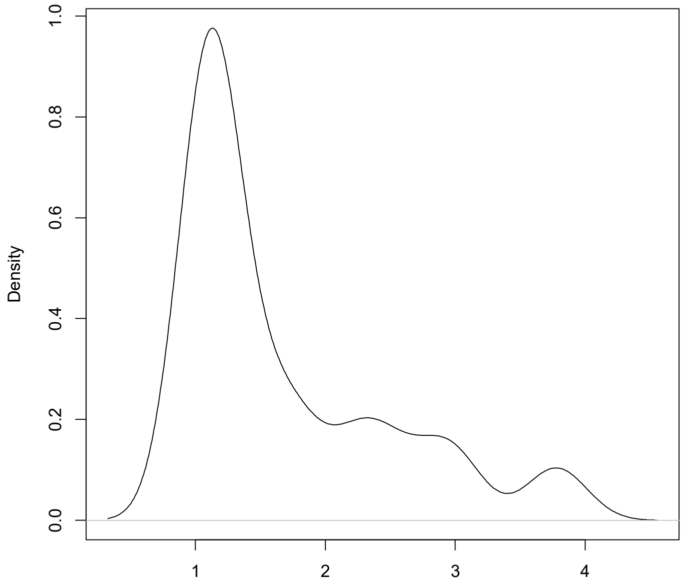
Density

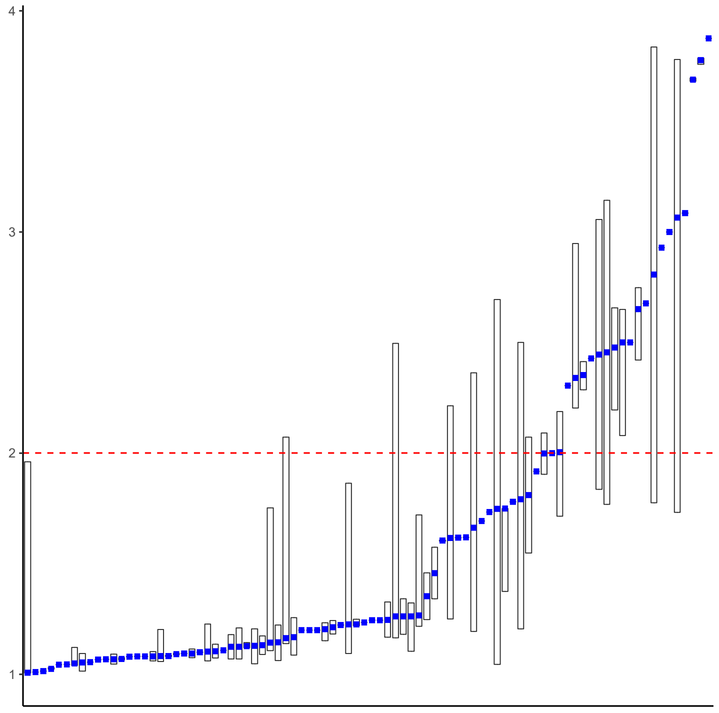

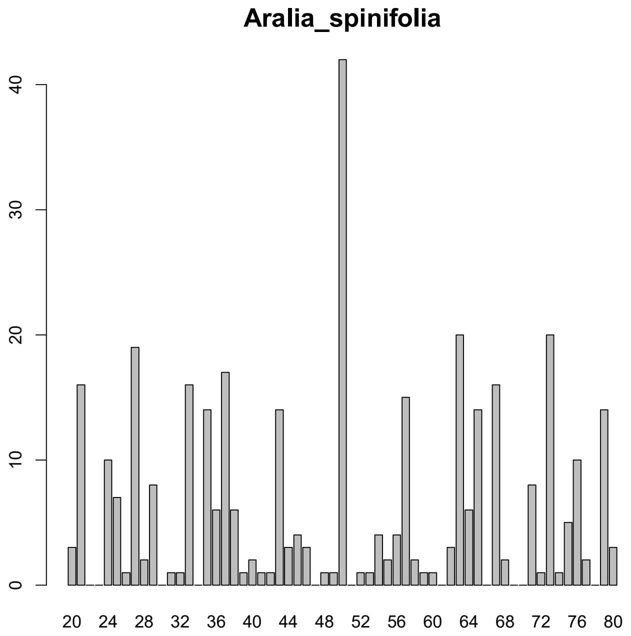
Aralia_spinifolia

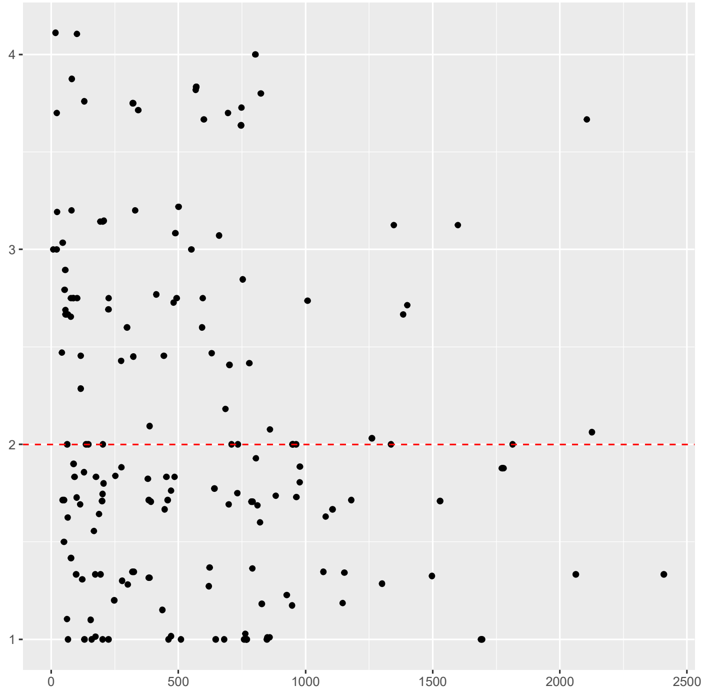

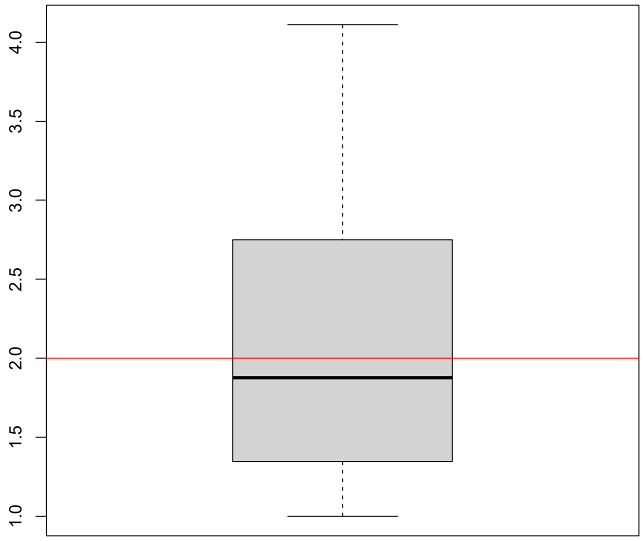

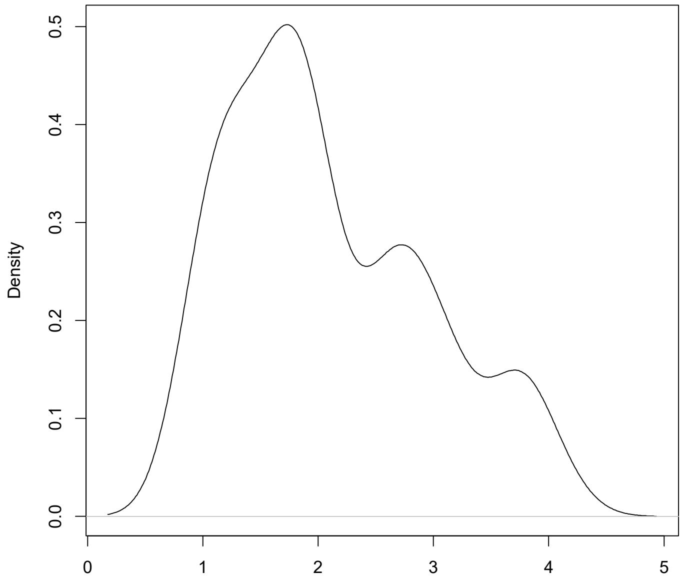
Density

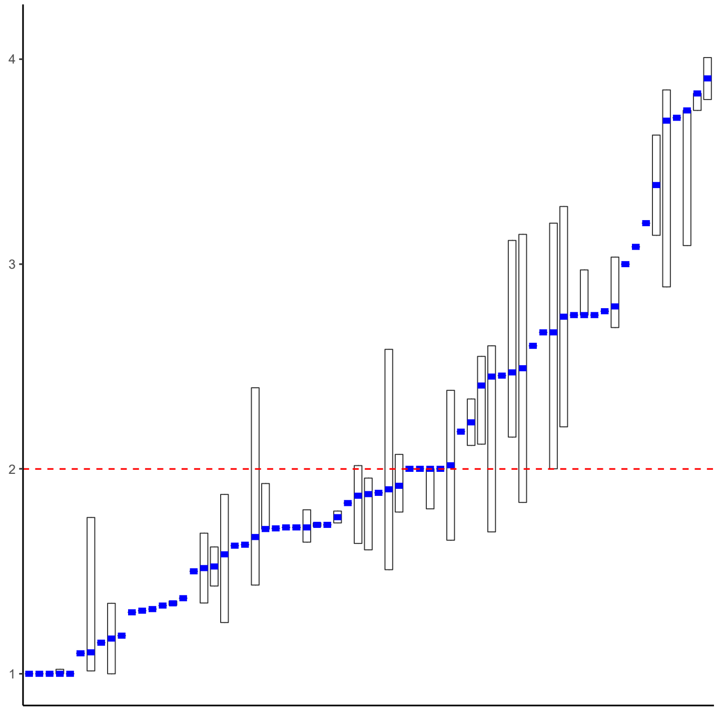

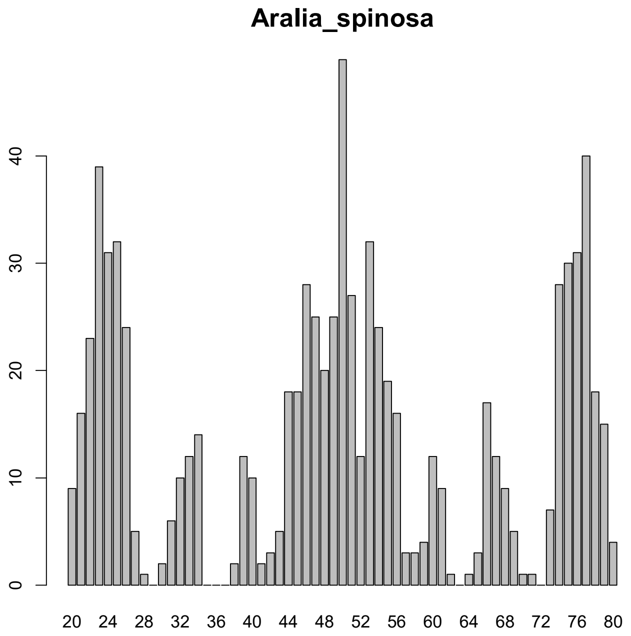
Aralia_spinosa
0
10
20
30
40
20 24 28 32 36 40 44 48 52 56 60 64 68 72 76 80

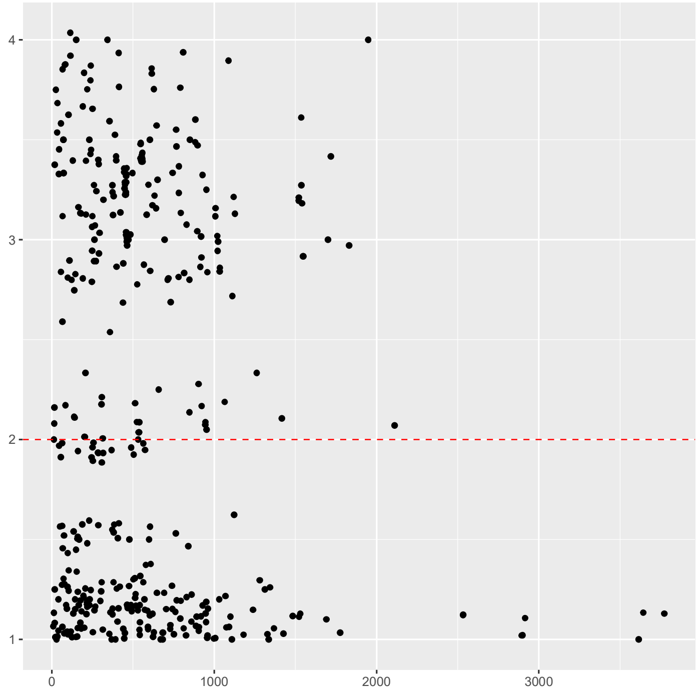
1
2
3
4
0 1000 2000 3000

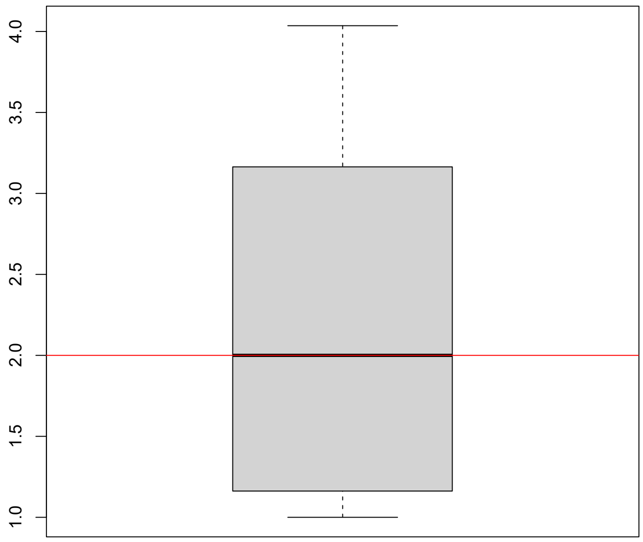
1.0
1.5
2.0
2.5
3.0
3.5
4.0

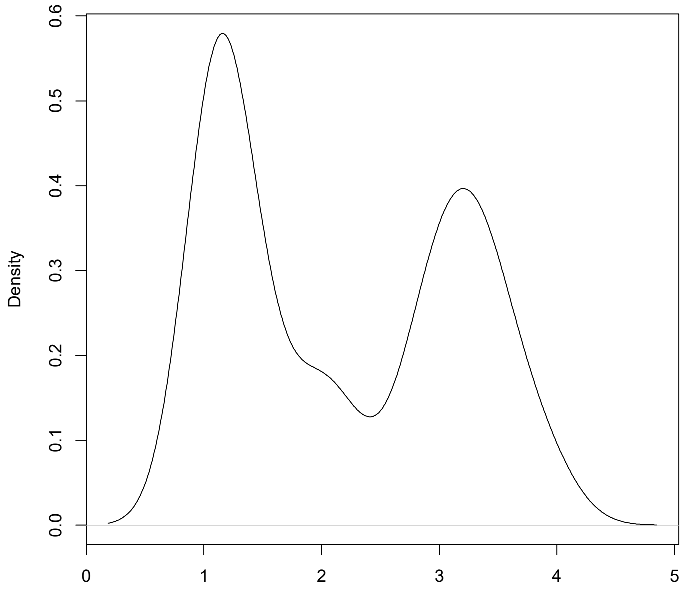
Density
0.0
0.1
0.2
0.3
0.4
0.5
0.6
0 1 2 3 4 5

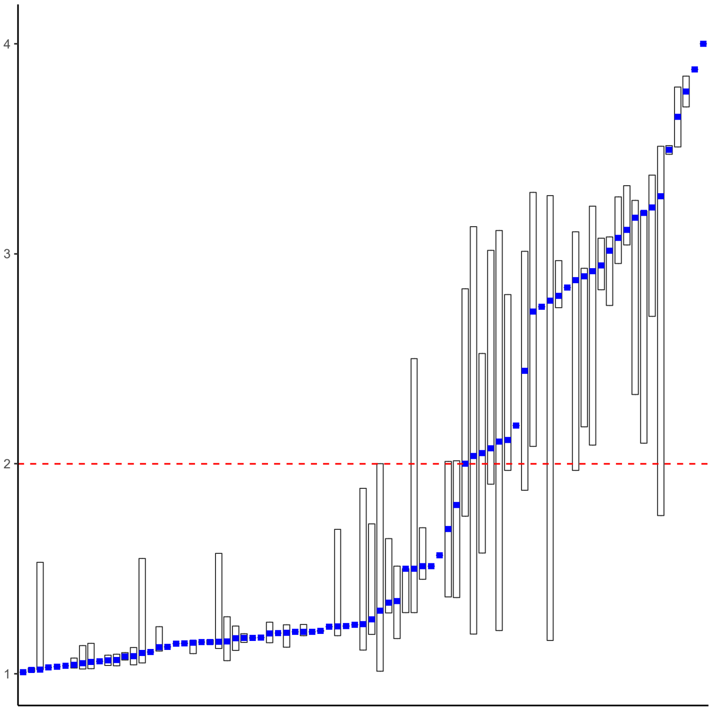
1
2
3
4

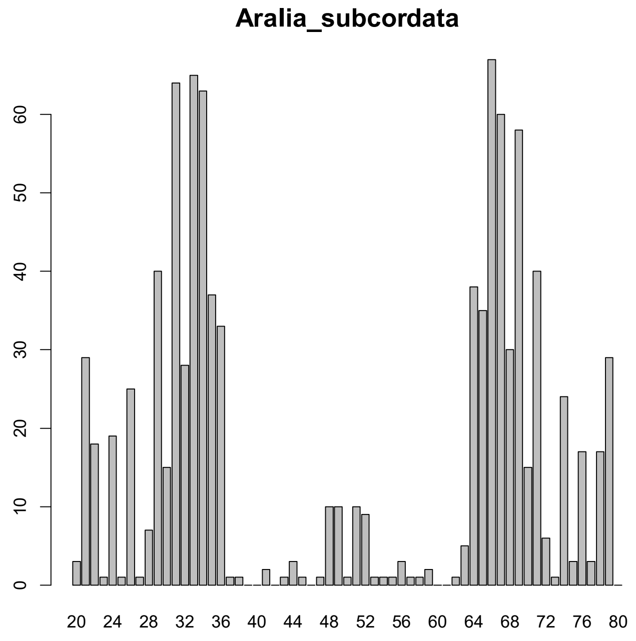
Aralia_subcordata

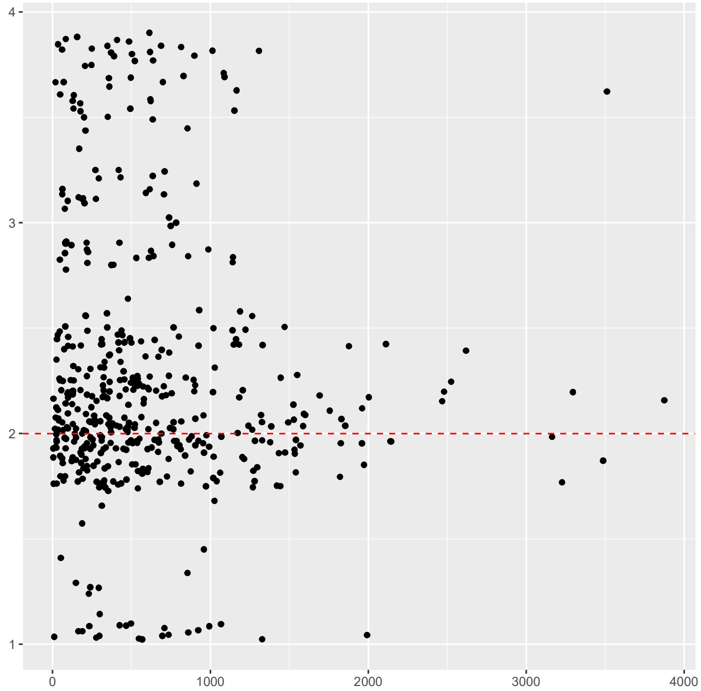

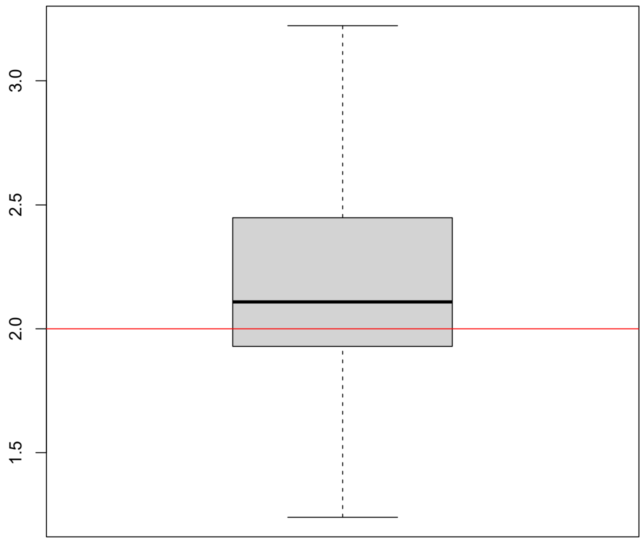

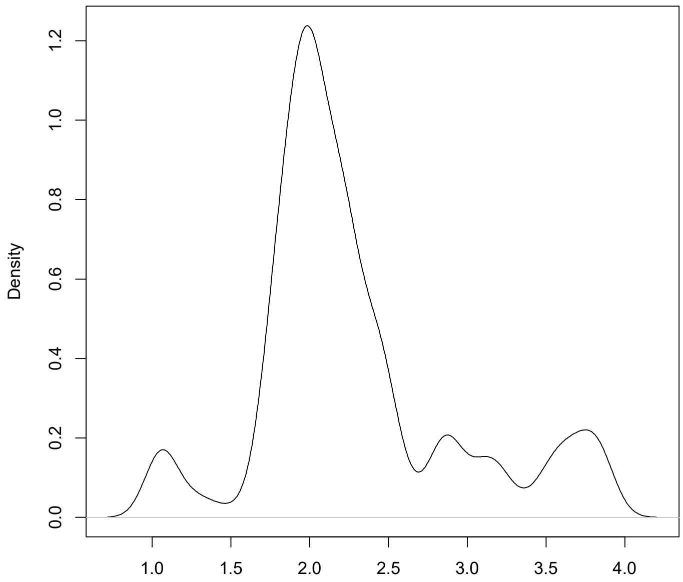
Density

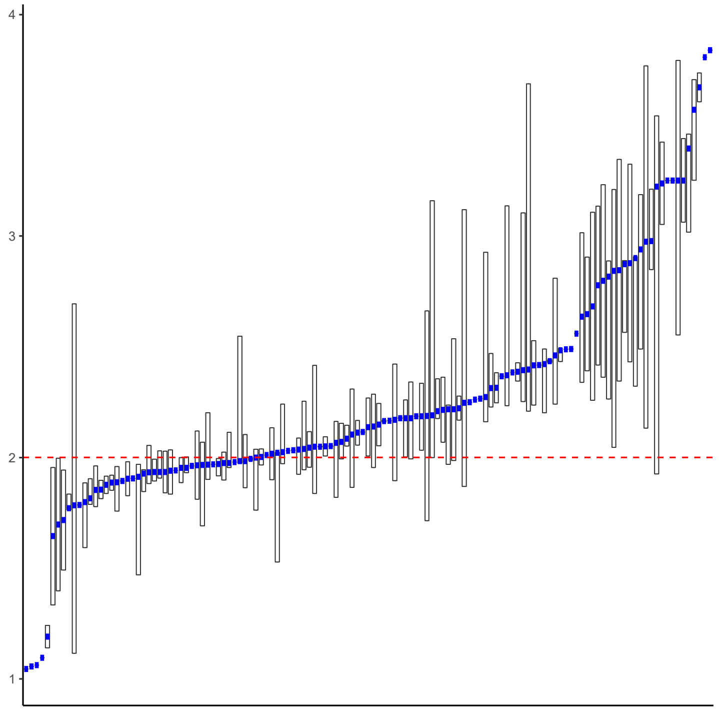

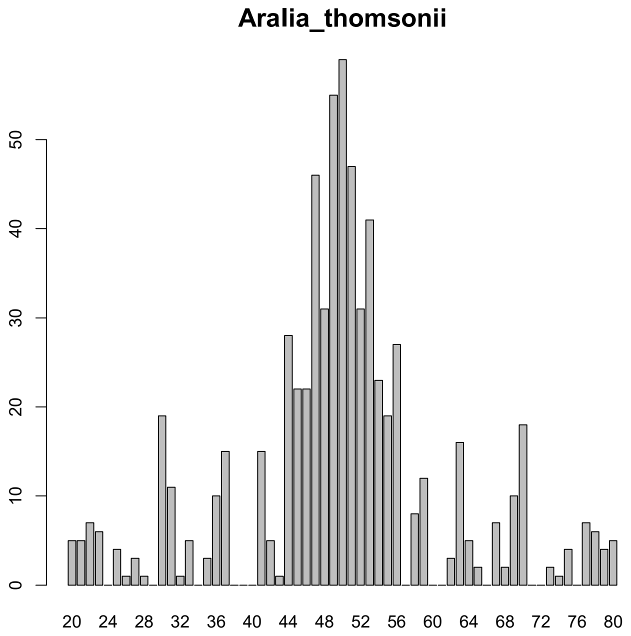
Aralia_thomsonii
0
10
20
30
40
50
20 24 28 32 36 40 44 48 52 56 60 64 68 72 76 80

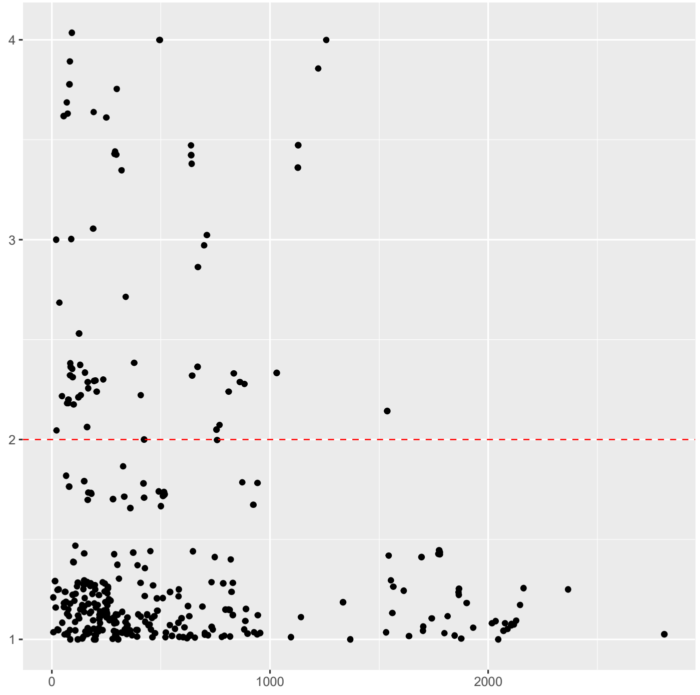
1
2
3
4
0
1000
2000

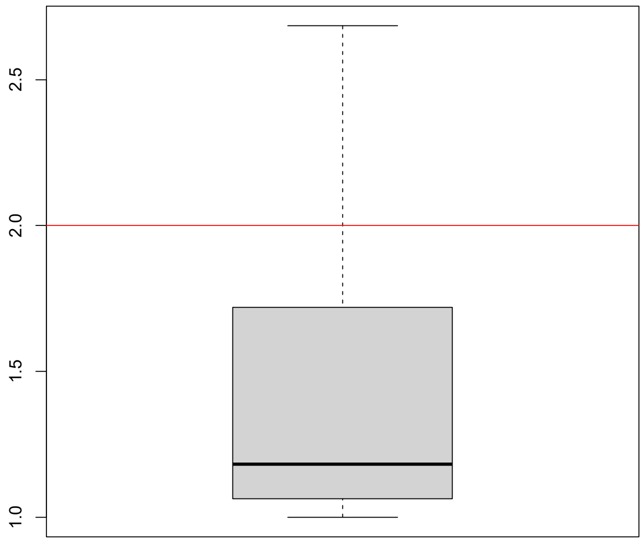
1.0
1.5
2.0
2.5

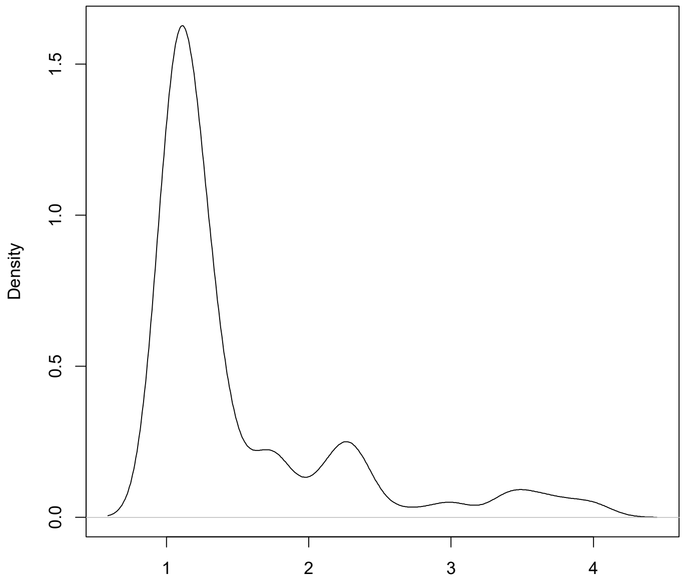
Density
0.0
0.5
1.0
1.5
1 2 3 4

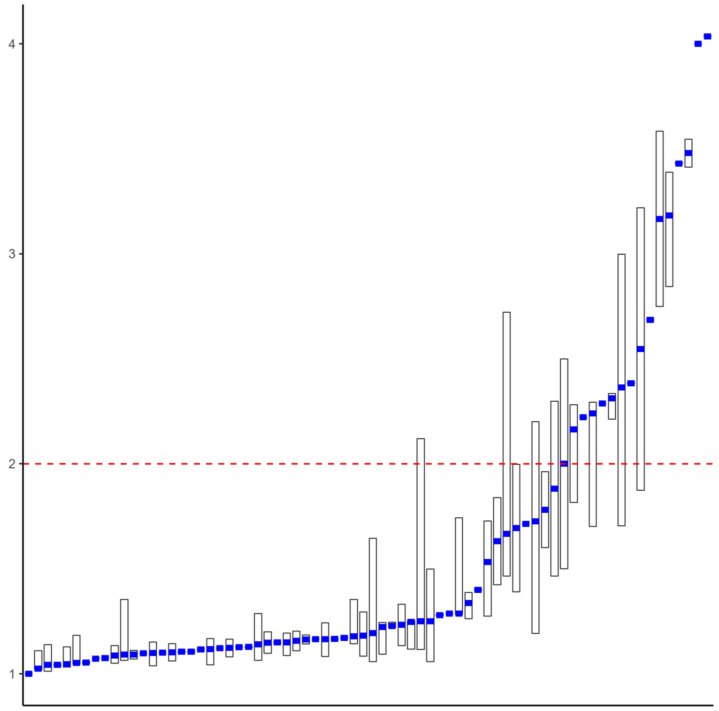
1
2
3
4

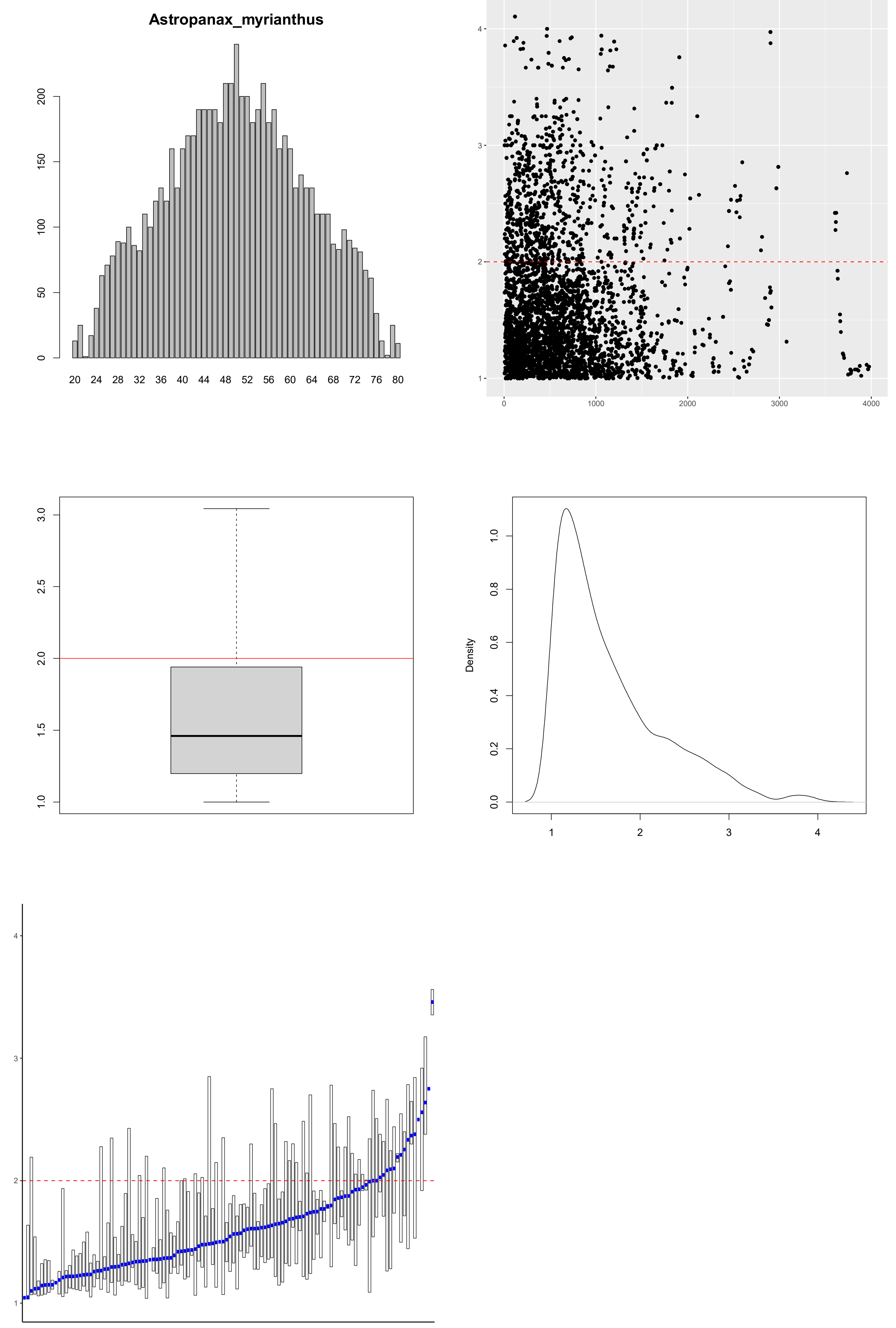
Astropanax_myrianthus
Density

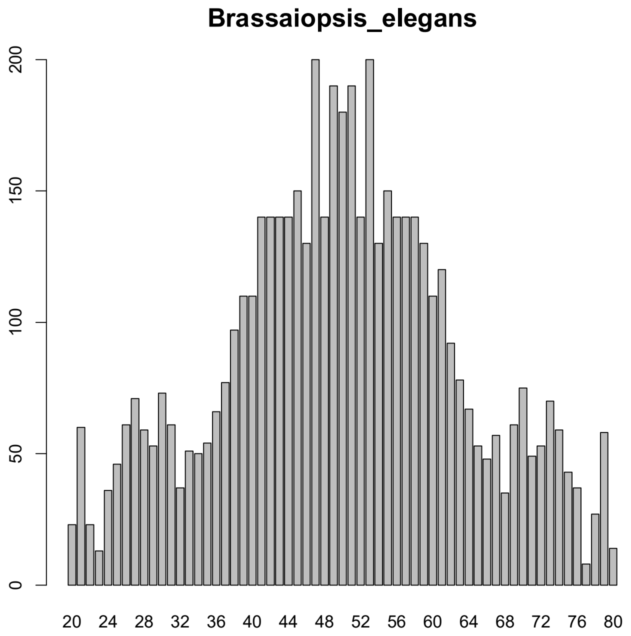
Brassaiopsis_elegans
0
50
100
150
200
20 24 28 32 36 40 44 48 52 56 60 64 68 72 76 80

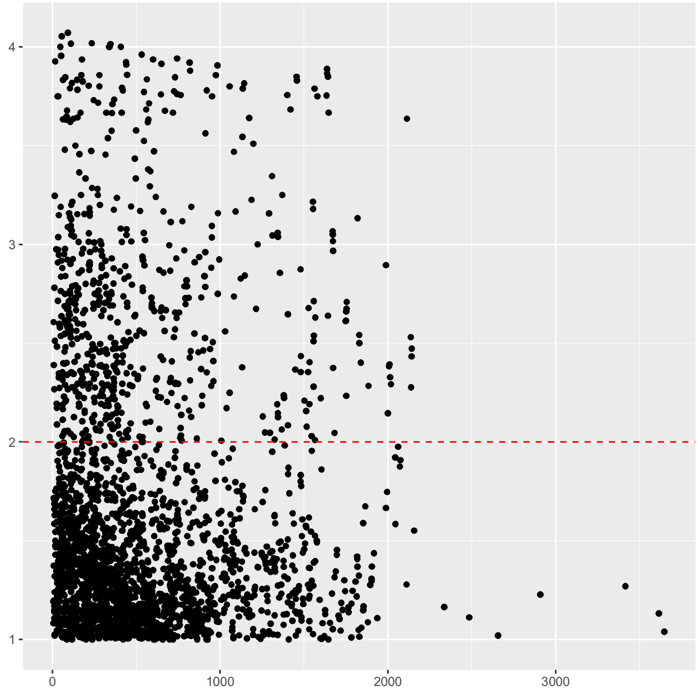
1
2
3
4
0
1000
2000
3000

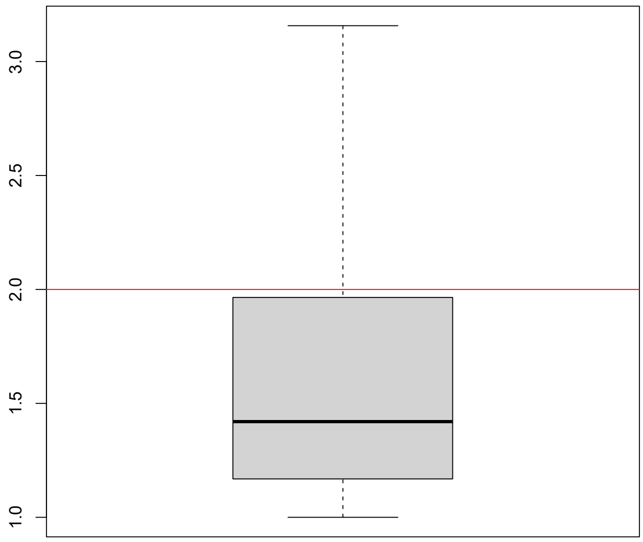
1.0
1.5
2.0
2.5
3.0

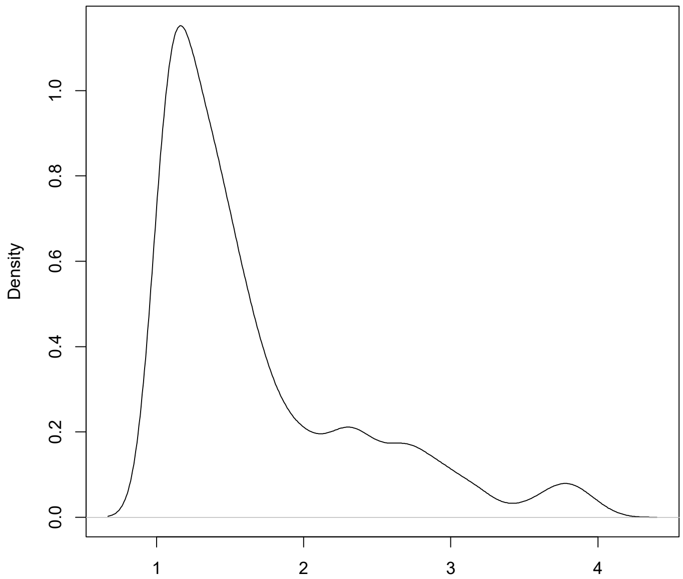
Density
0.0
0.2
0.4
0.6
0.8
1.0
1 2 3 4

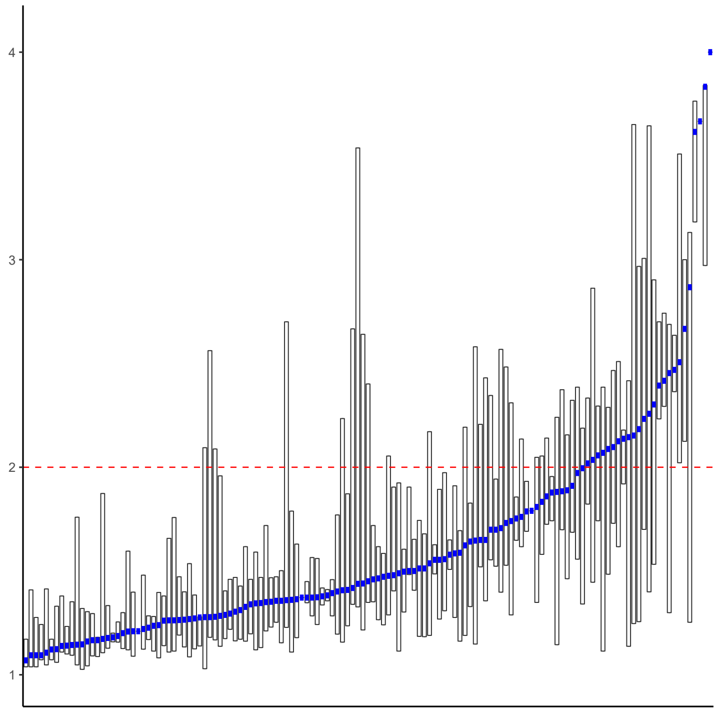
1
2
3
4

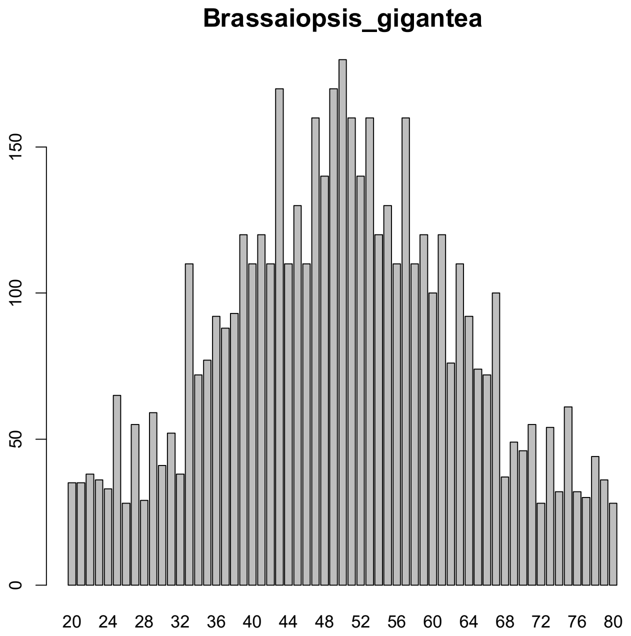
Brassaiopsis_gigantea
0
50
100
150
20 24 28 32 36 40 44 48 52 56 60 64 68 72 76 80

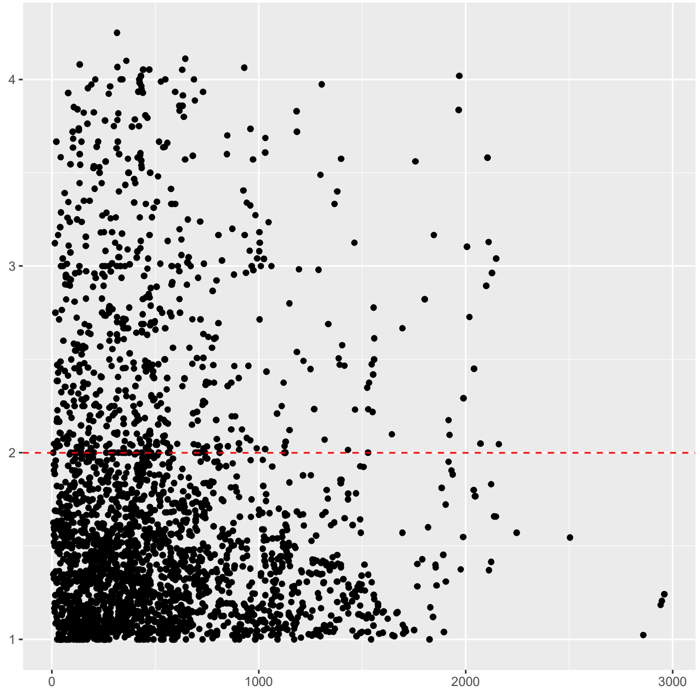
1
2
3
4
0 1000 2000 3000

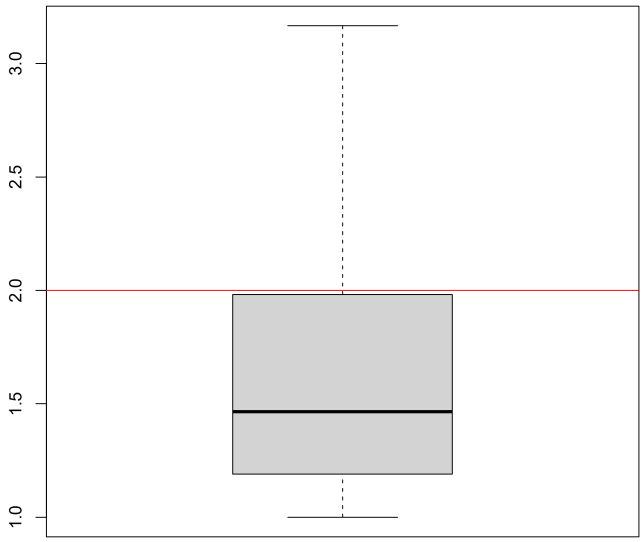
1.0
1.5
2.0
2.5
3.0

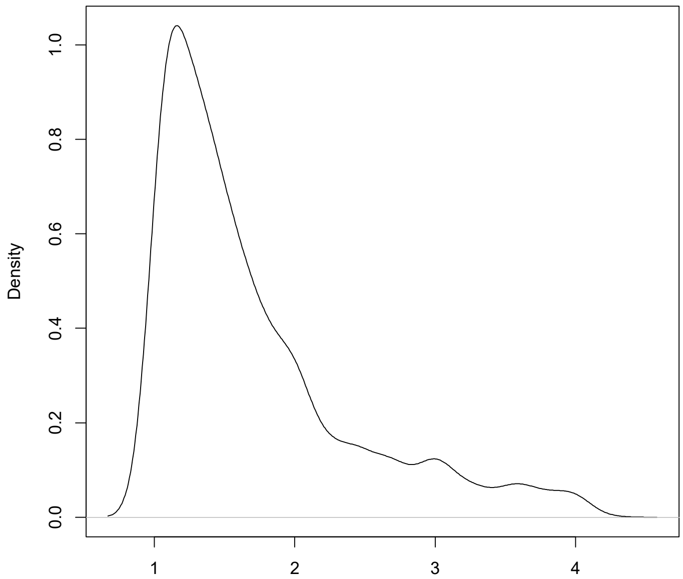
Density
0.0
0.2
0.4
0.6
0.8
1.0
1 2 3 4

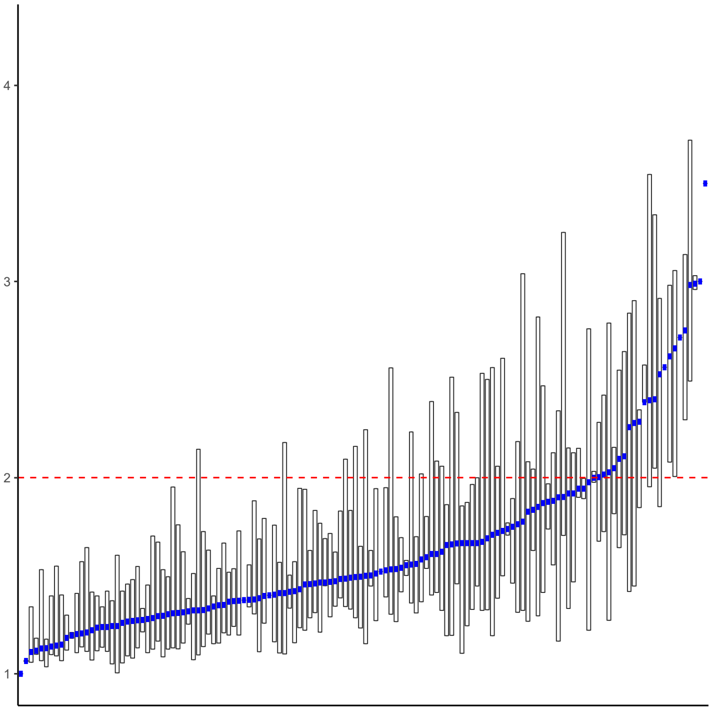
1
2
3
4

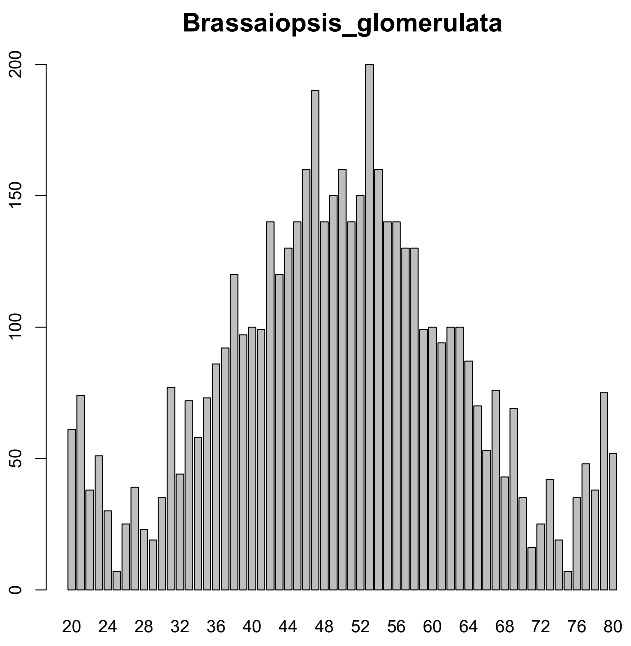
Brassaiopsis_glomerulata
0
50
100
150
200
20 24 28 32 36 40 44 48 52 56 60 64 68 72 76 80

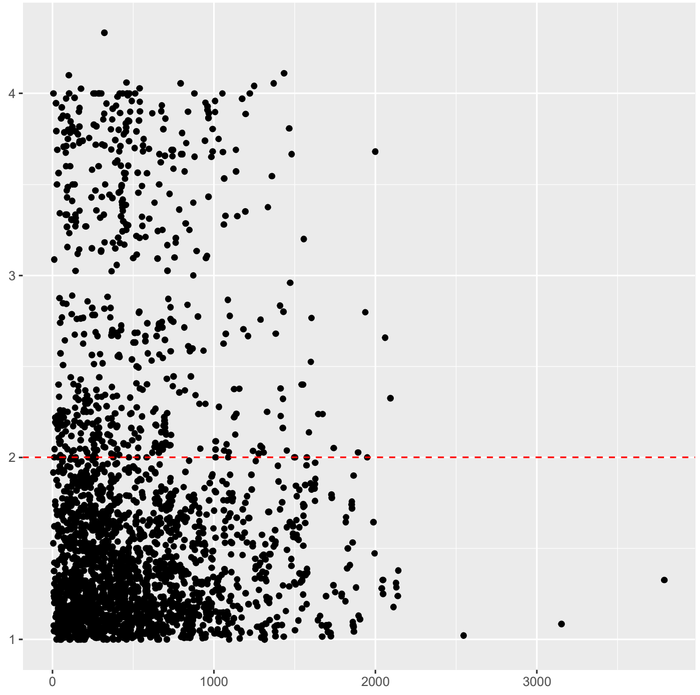
1
2
3
4
0 1000 2000 3000

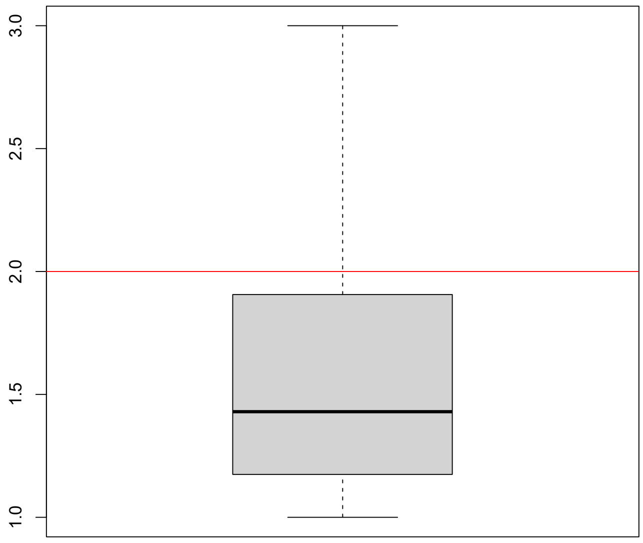
1.0
1.5
2.0
2.5
3.0

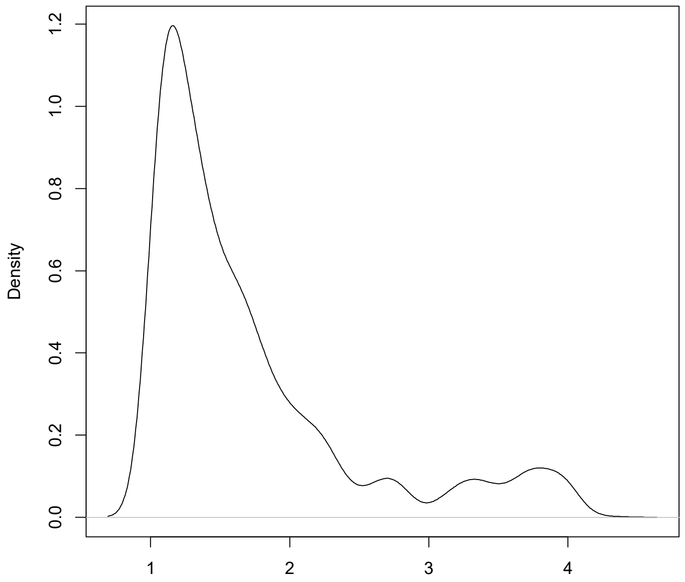
Density
0.0
0.2
0.4
0.6
0.8
1.0
1.2
1 2 3 4

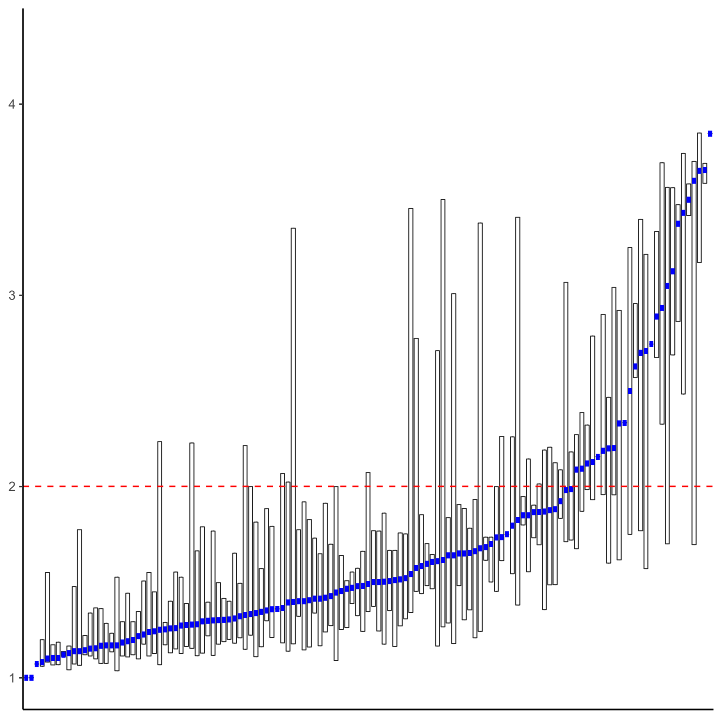
1
2
3
4

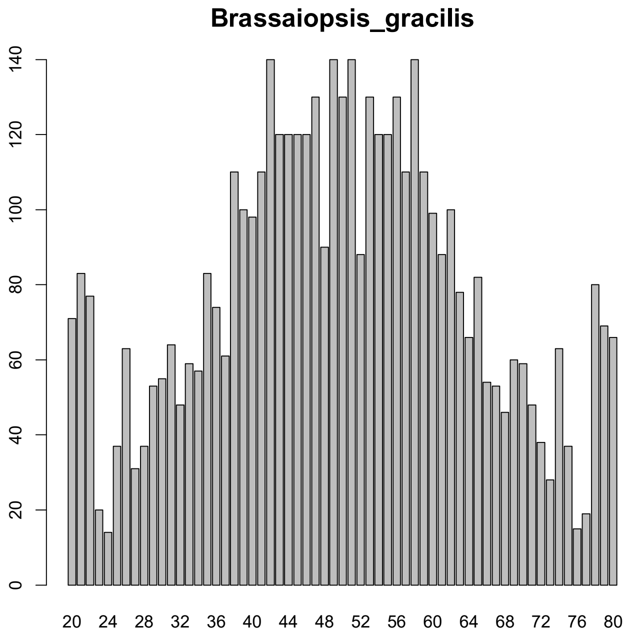
Brassaiopsis_gracilis

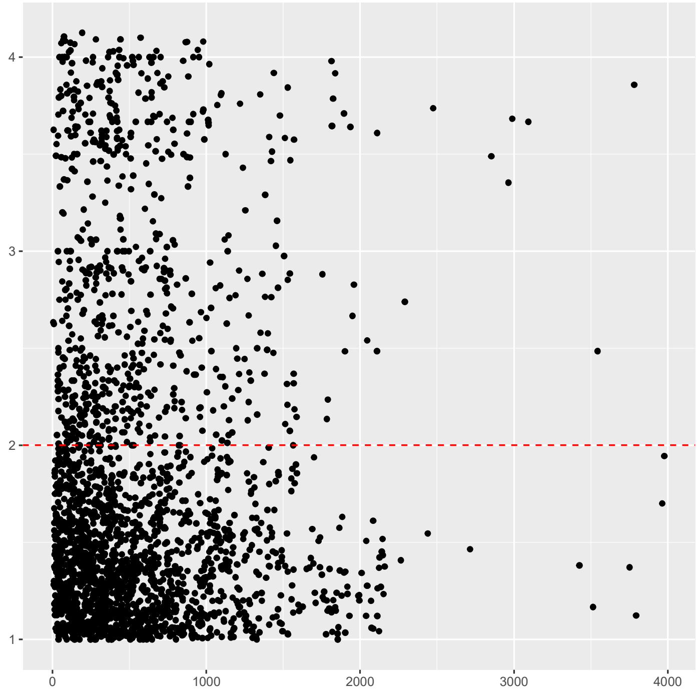

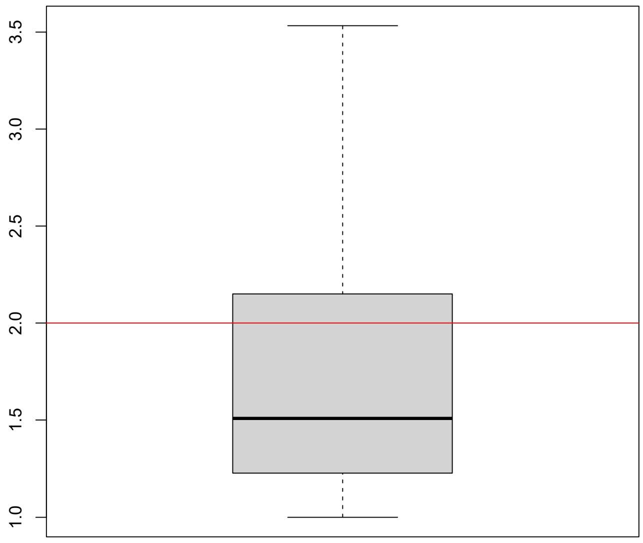

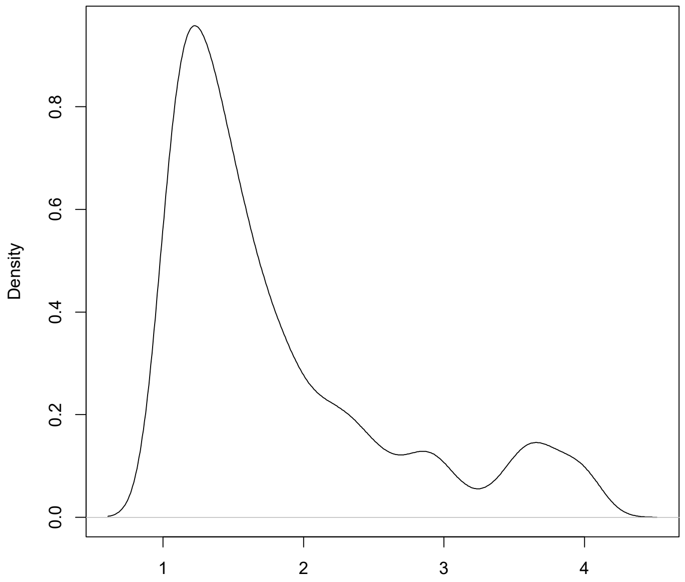
Density

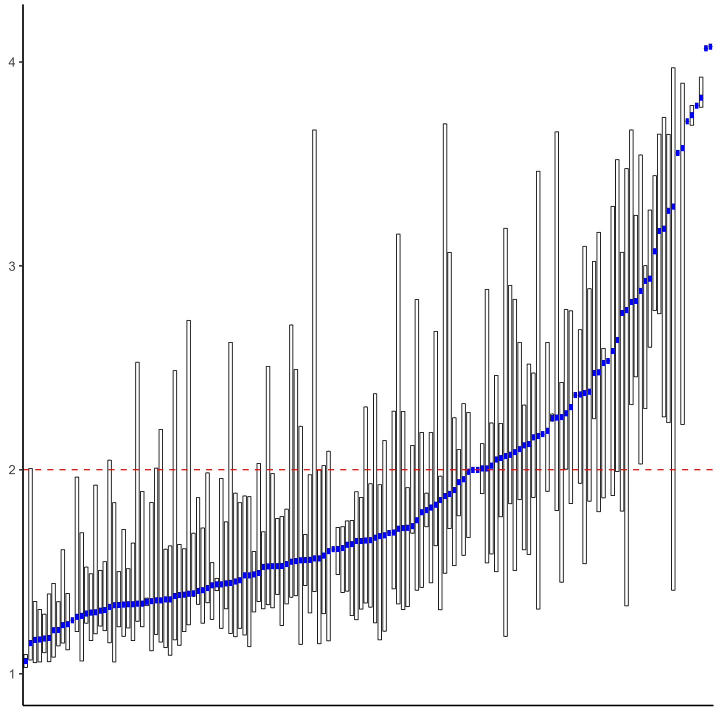

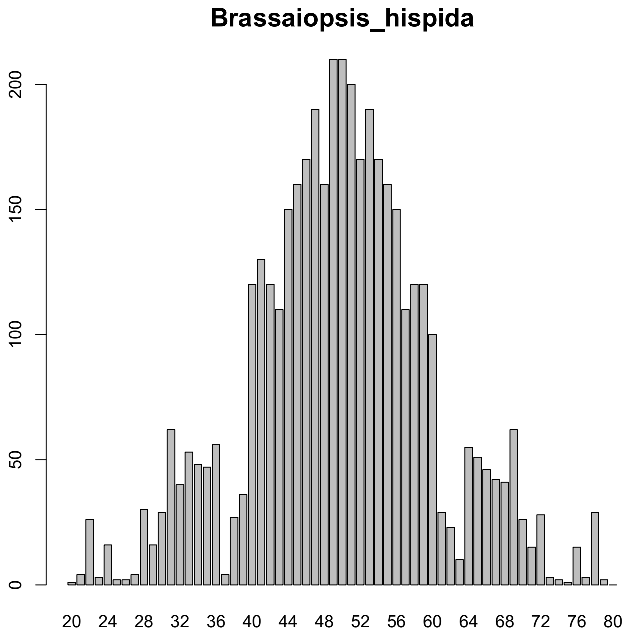
Brassaiopsis_hispida
0
50
100
150
200
20 24 28 32 36 40 44 48 52 56 60 64 68 72 76 80

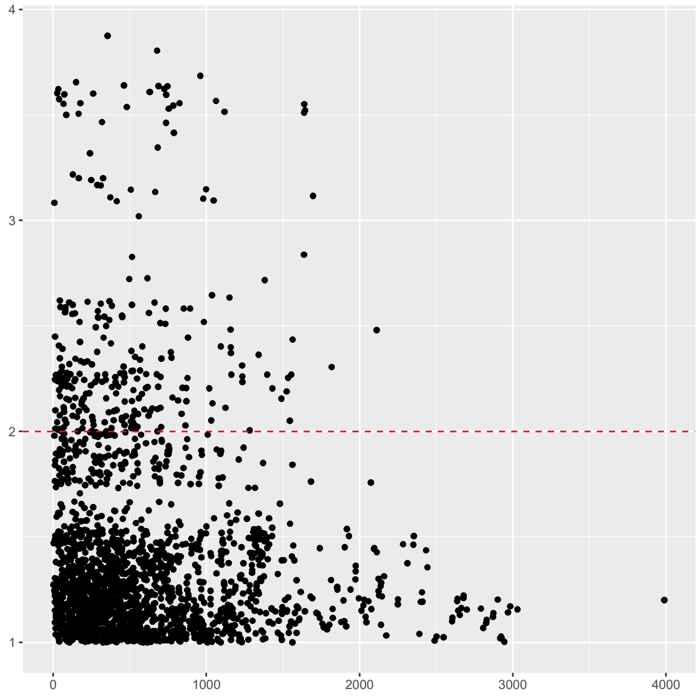
1
2
3
4
0 1000 2000 3000 4000

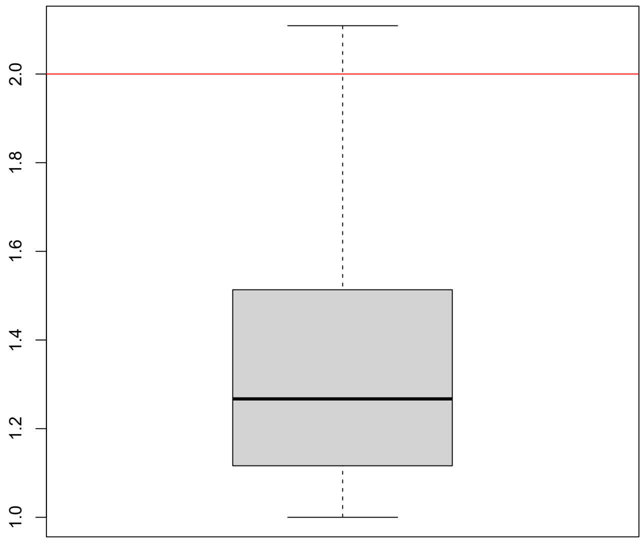
1.0
1.2
1.4
1.6
1.8
2.0

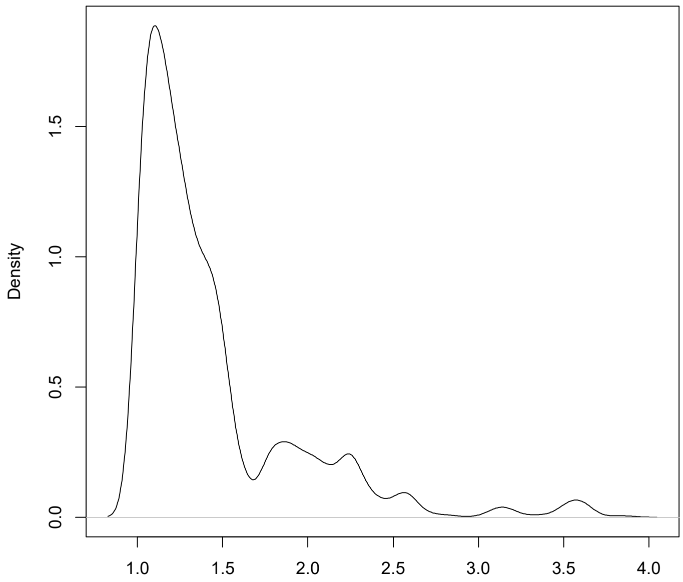
Density
0.0
0.5
1.0
1.5
1.0 1.5 2.0 2.5 3.0 3.5 4.0

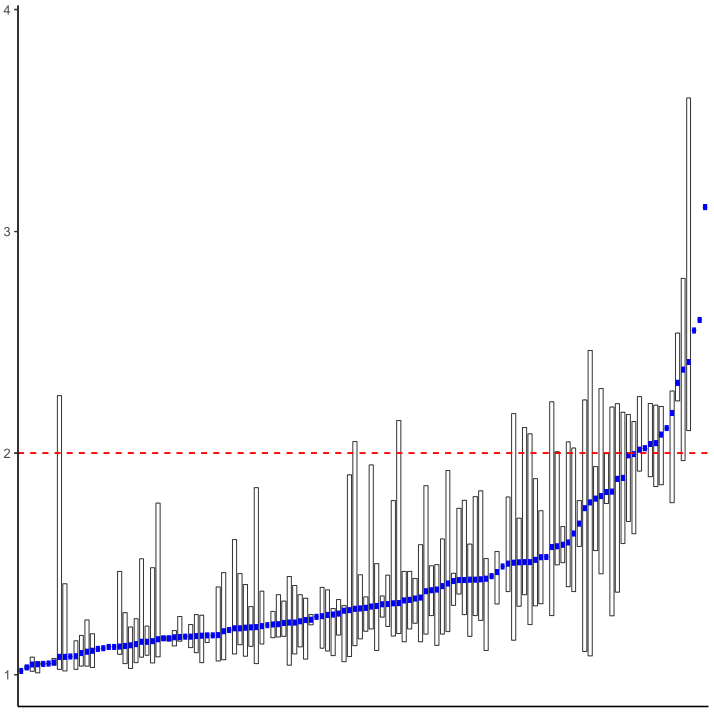
1
2
3
4

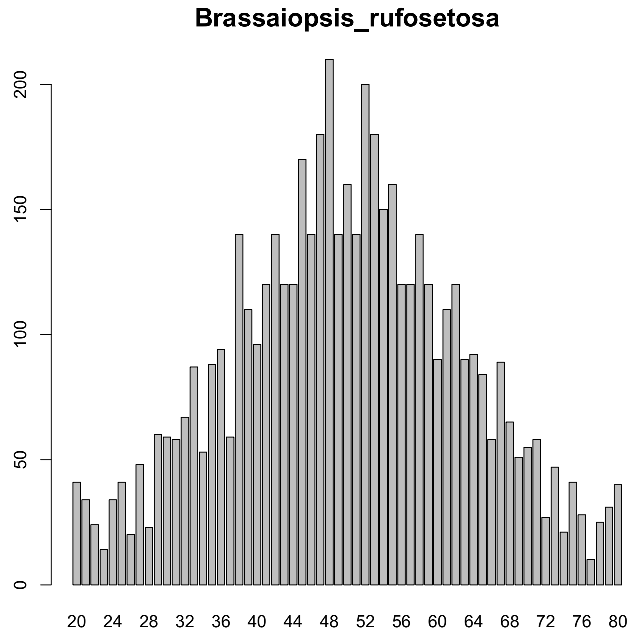
Brassaiopsis_rufosetosa
0
50
100
150
200
20 24 28 32 36 40 44 48 52 56 60 64 68 72 76 80

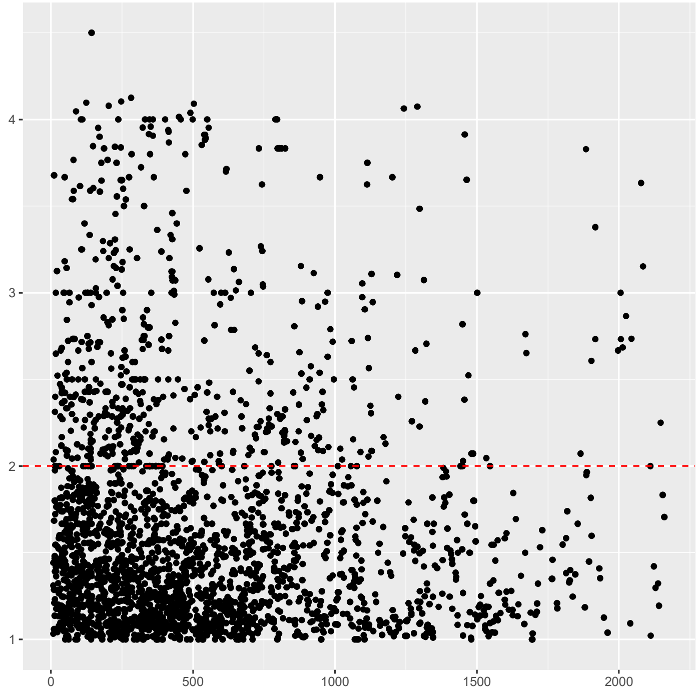
1
2
3
4
0 500 1000 1500 2000

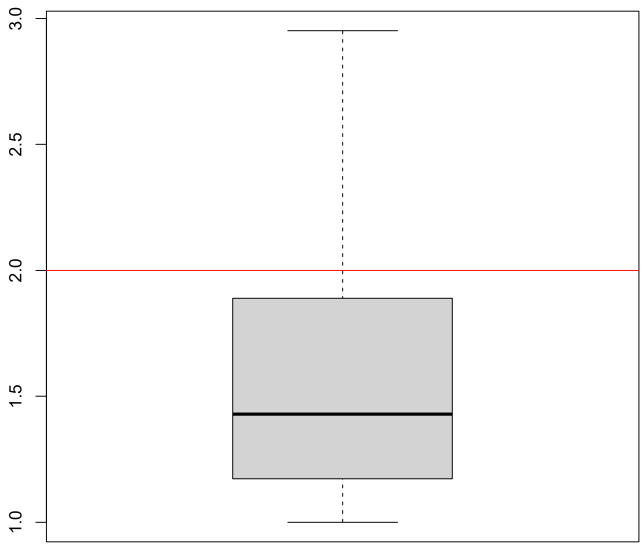
1.0
1.5
2.0
2.5
3.0

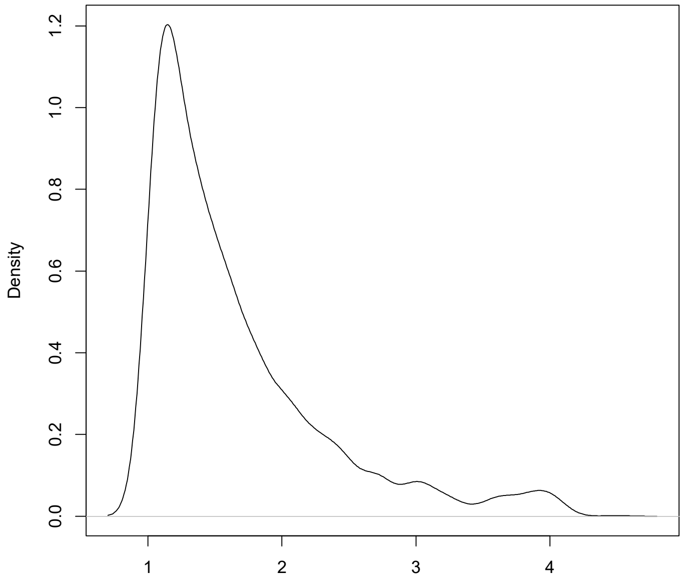
Density
0.0
0.2
0.4
0.6
0.8
1.0
1.2
1 2 3 4

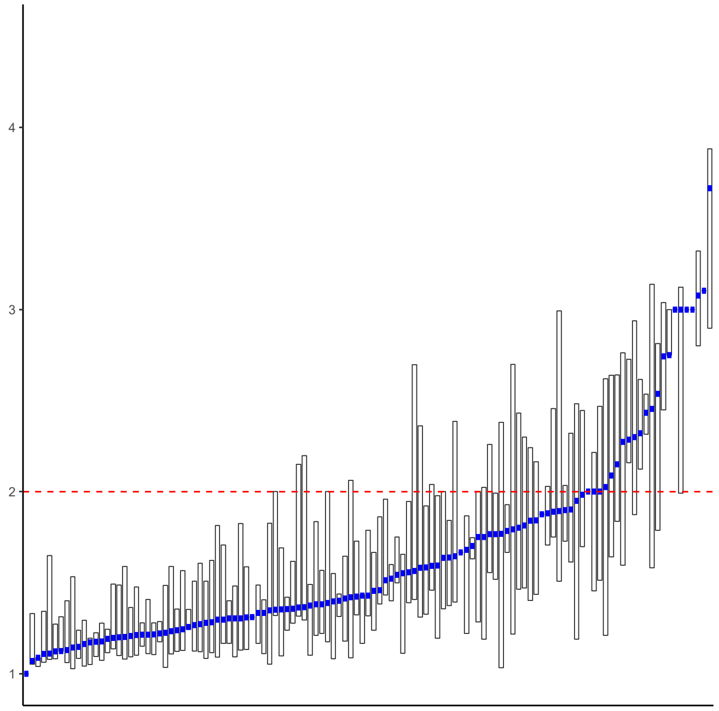
1
2
3
4

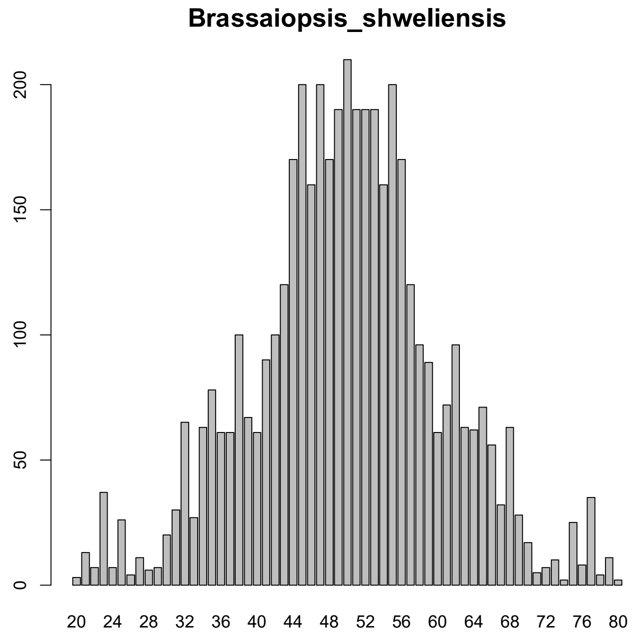
Brassaiopsis_shweliensis

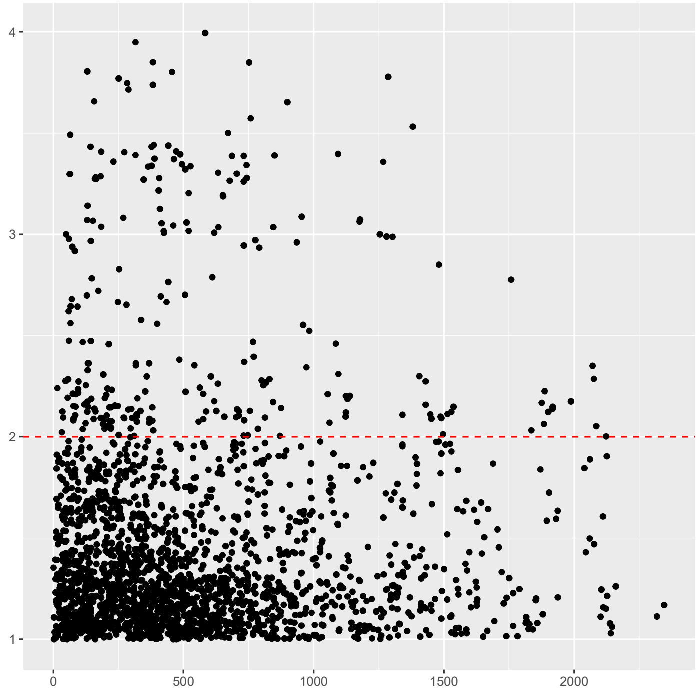

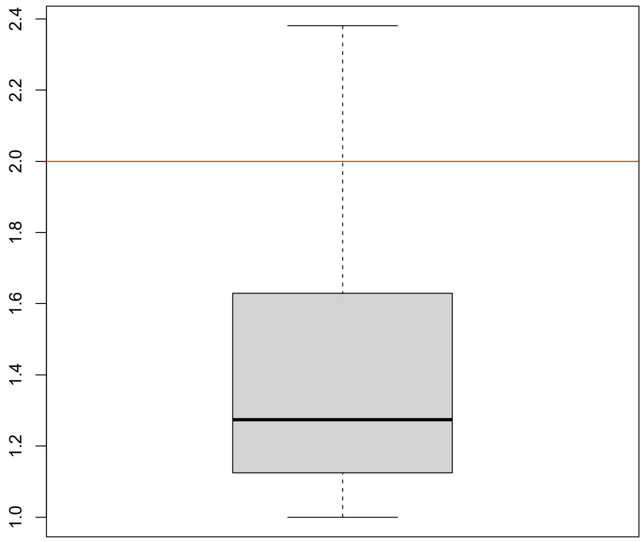

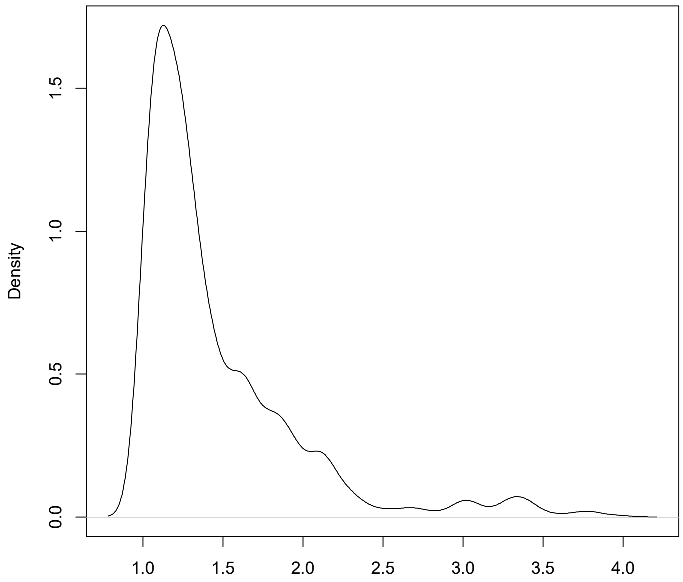
Density

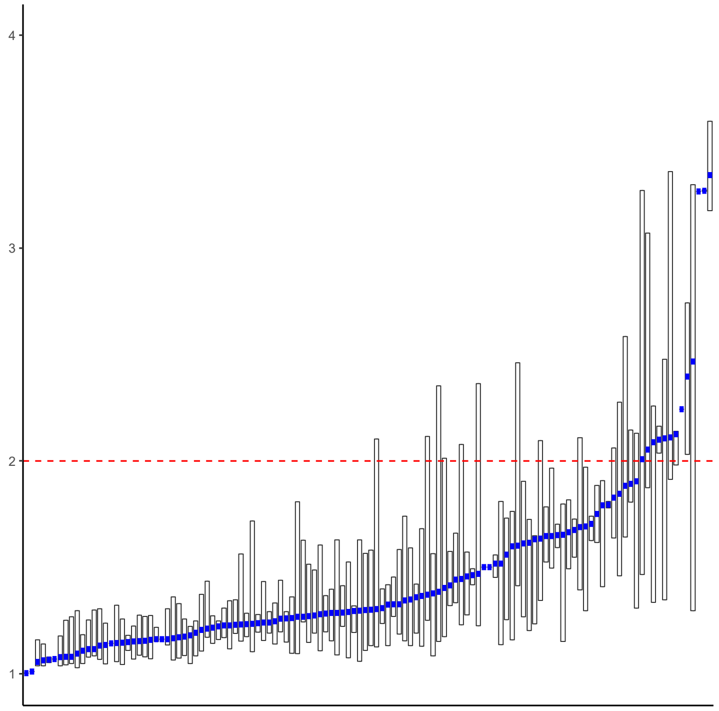

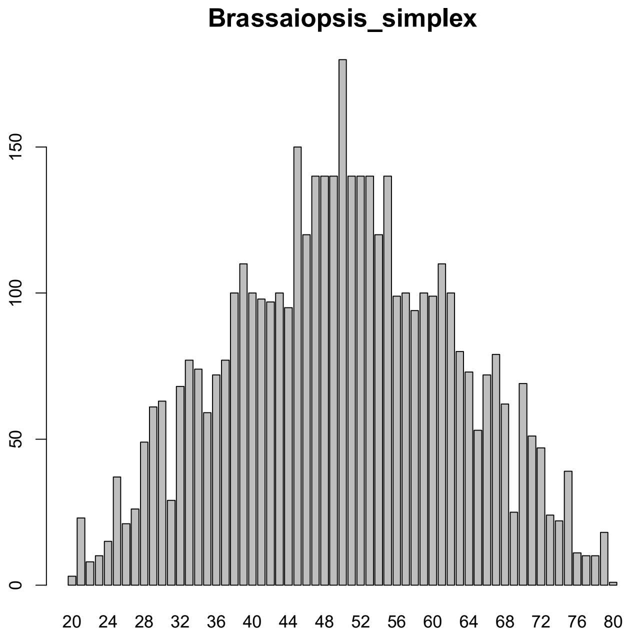
Brassaiopsis_simplex

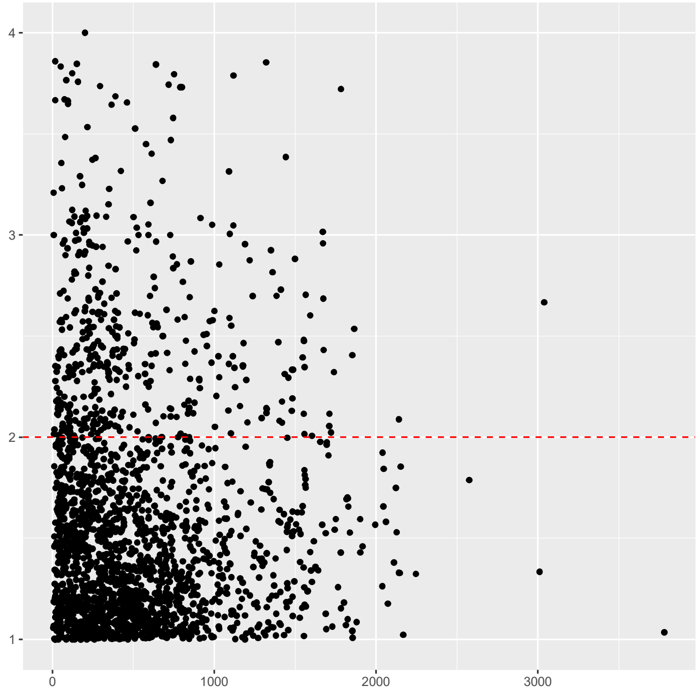

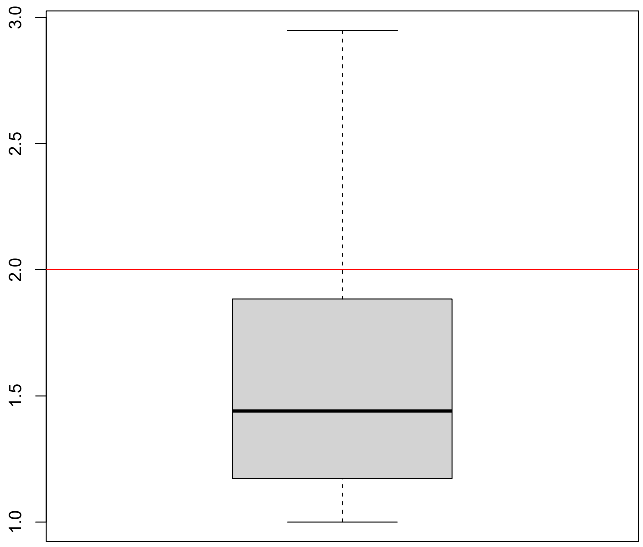

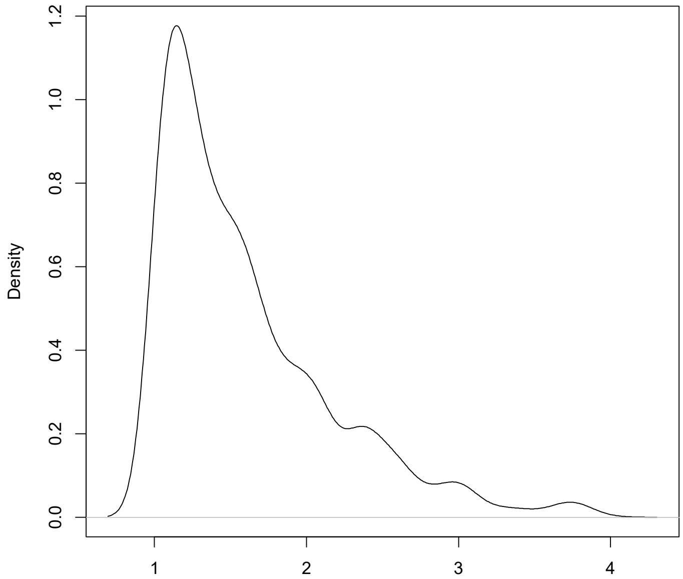
Density

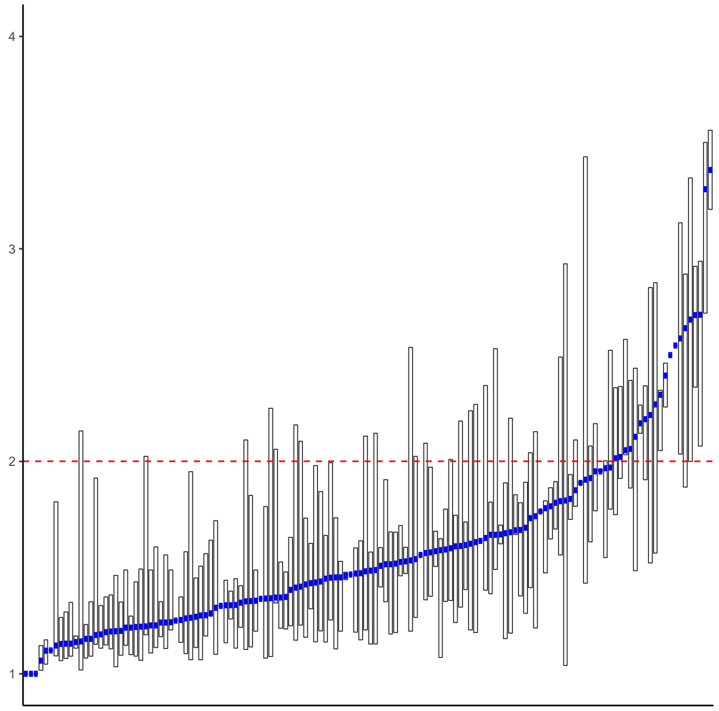

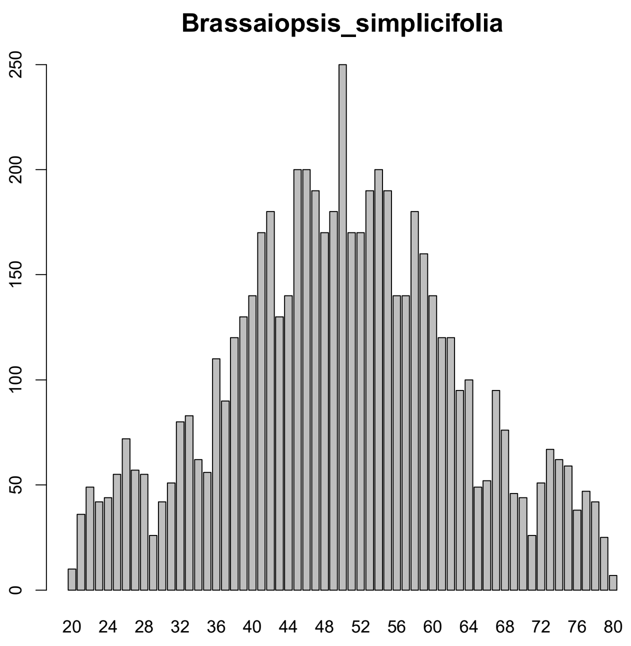
Brassaiopsis_simplicifolia

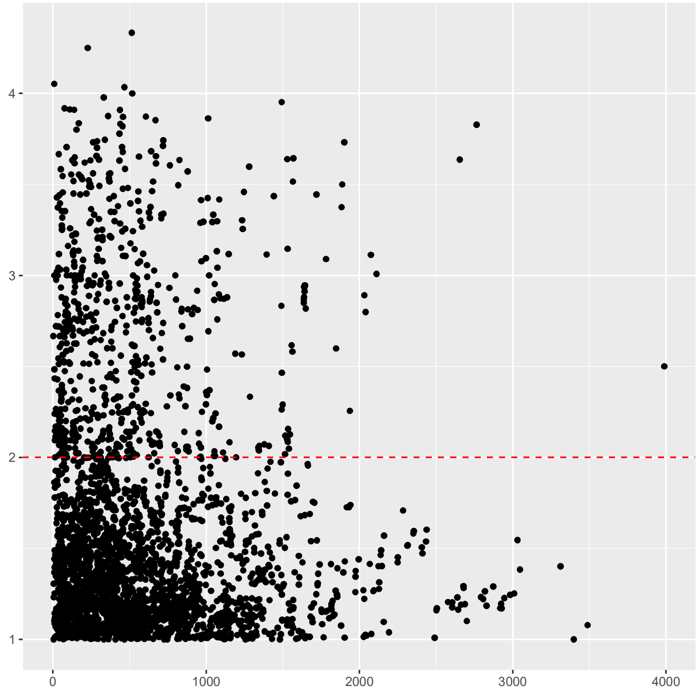

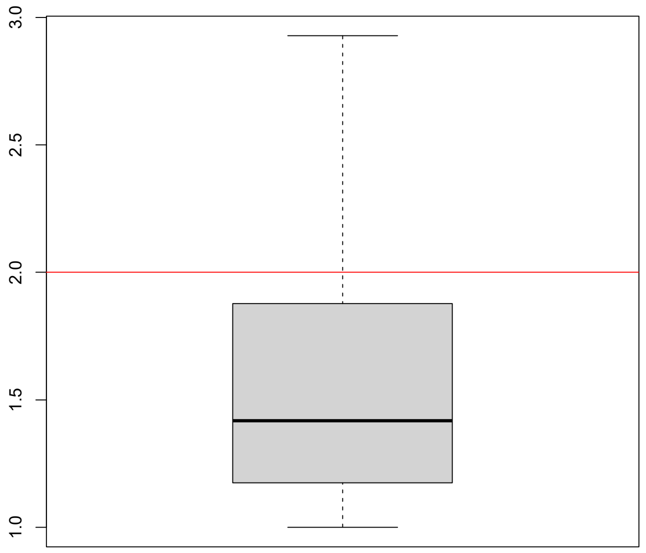

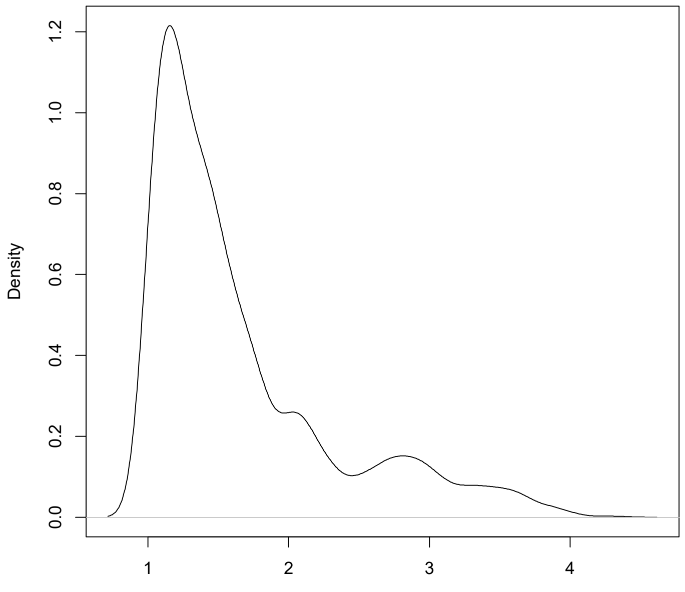
Density

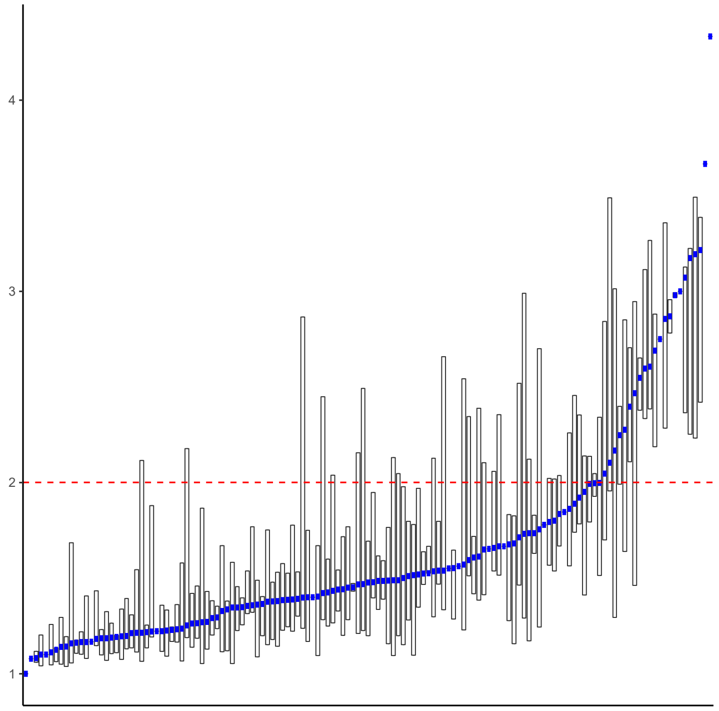

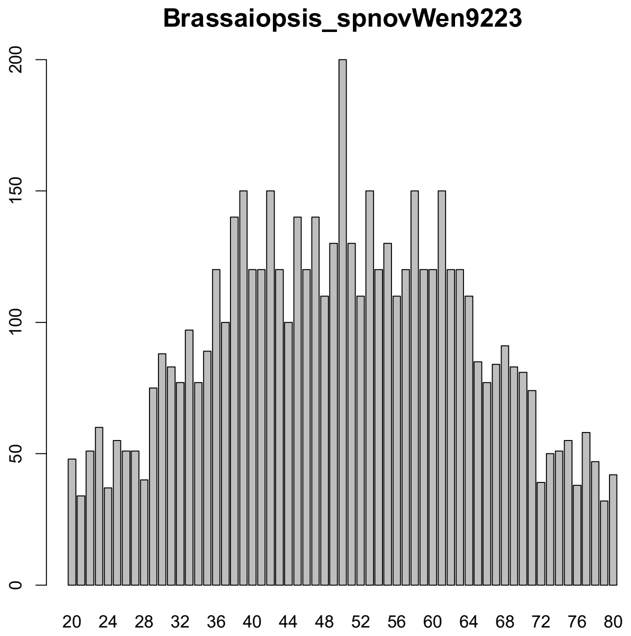
Brassaiopsis_spnovWen9223
0
50
100
150
200
20 24 28 32 36 40 44 48 52 56 60 64 68 72 76 80

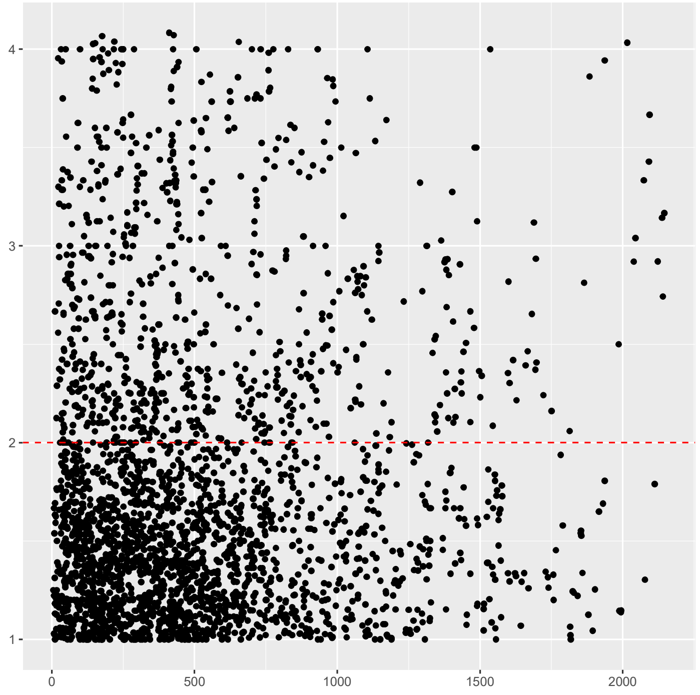
1
2
3
4
0 500 1000 1500 2000

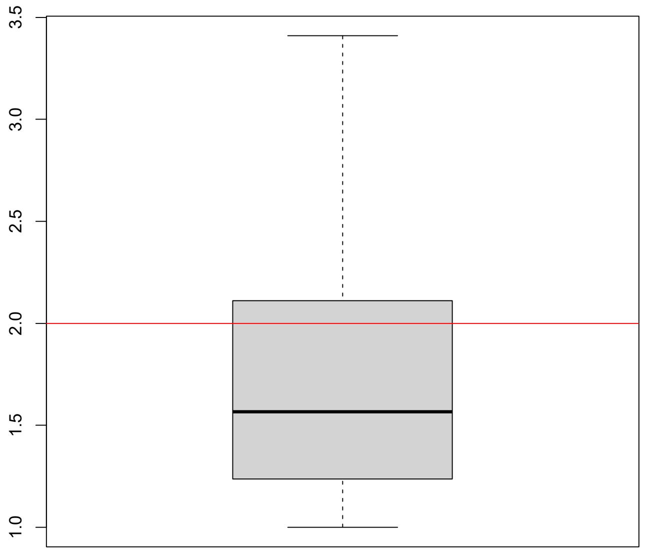
1.0
1.5
2.0
2.5
3.0
3.5

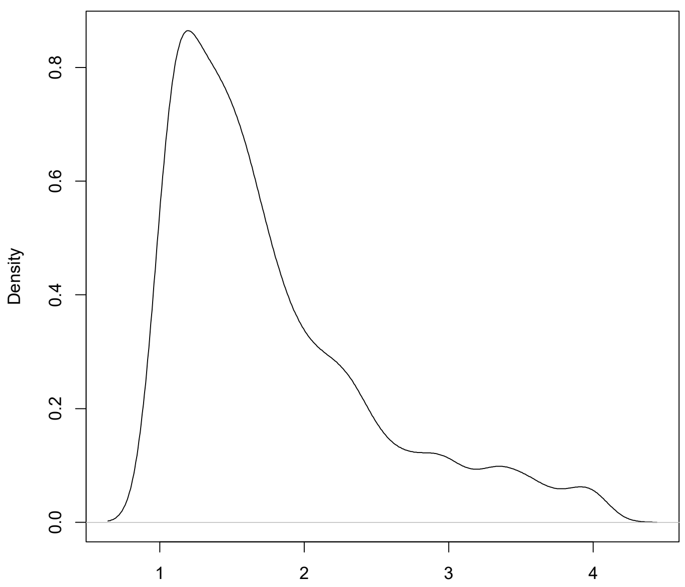
Density
0.0
0.2
0.4
0.6
0.8
1 2 3 4

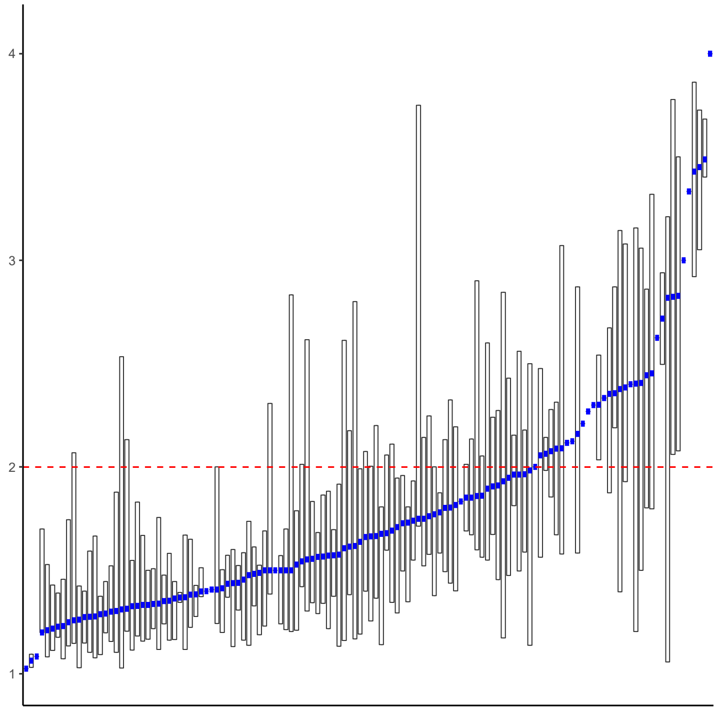
1
2
3
4

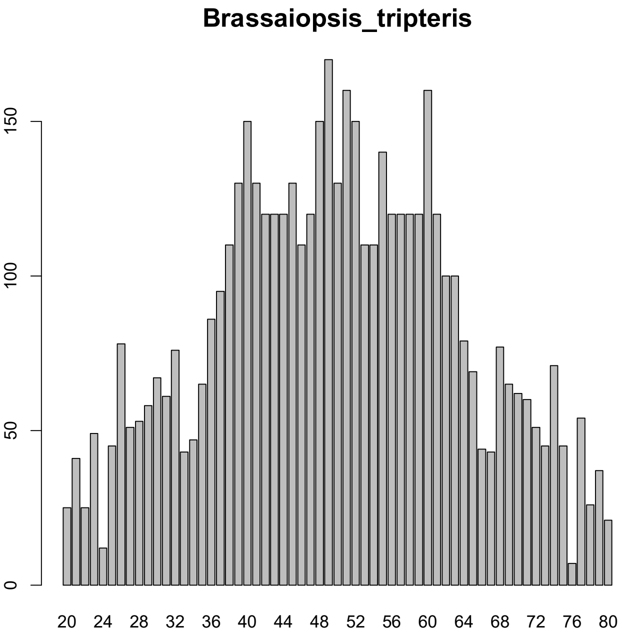
Brassaiopsis_tripteris
0
50
100
150
20 24 28 32 36 40 44 48 52 56 60 64 68 72 76 80

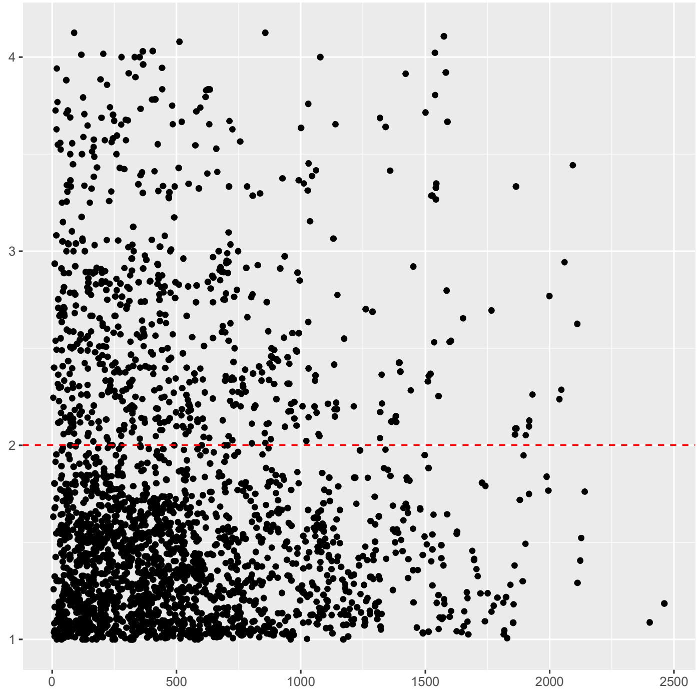
1
2
3
4
0 500 1000 1500 2000 2500

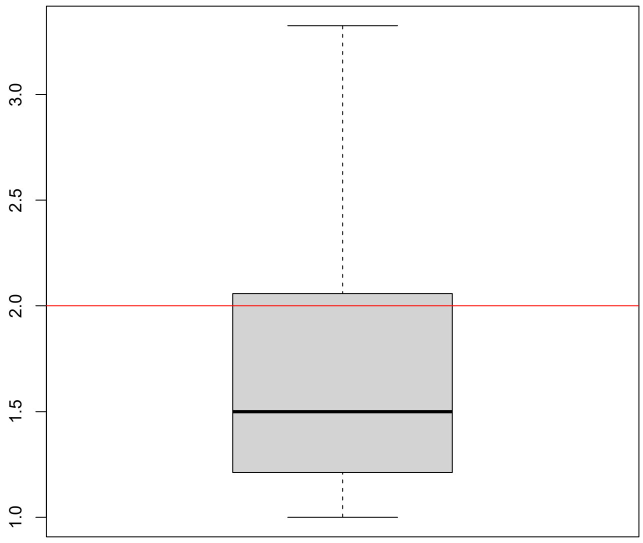
1.0
1.5
2.0
2.5
3.0

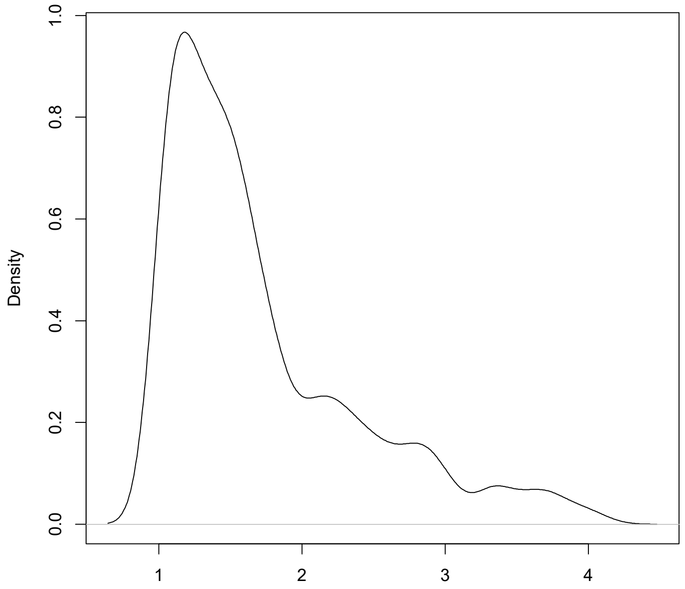
Density
0.0
0.2
0.4
0.6
0.8
1.0
1 2 3 4

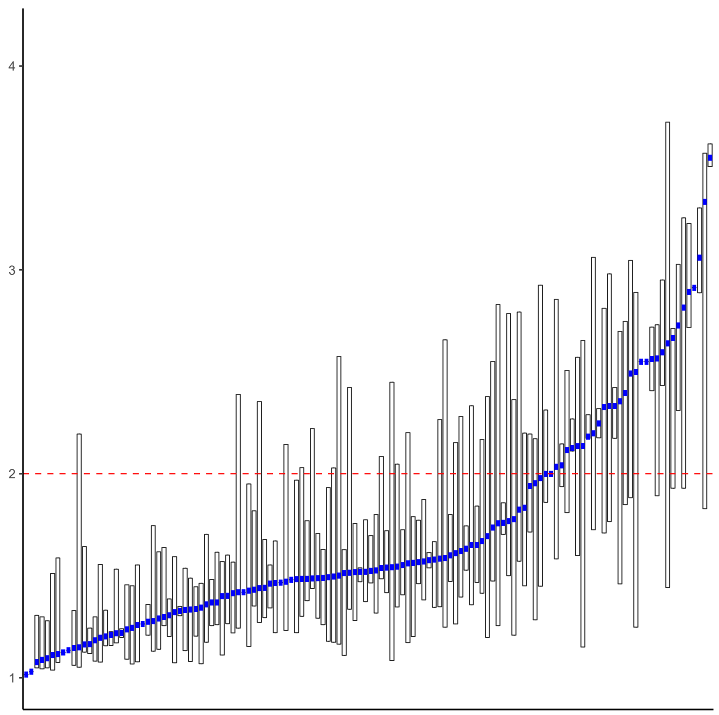
1
2
3
4

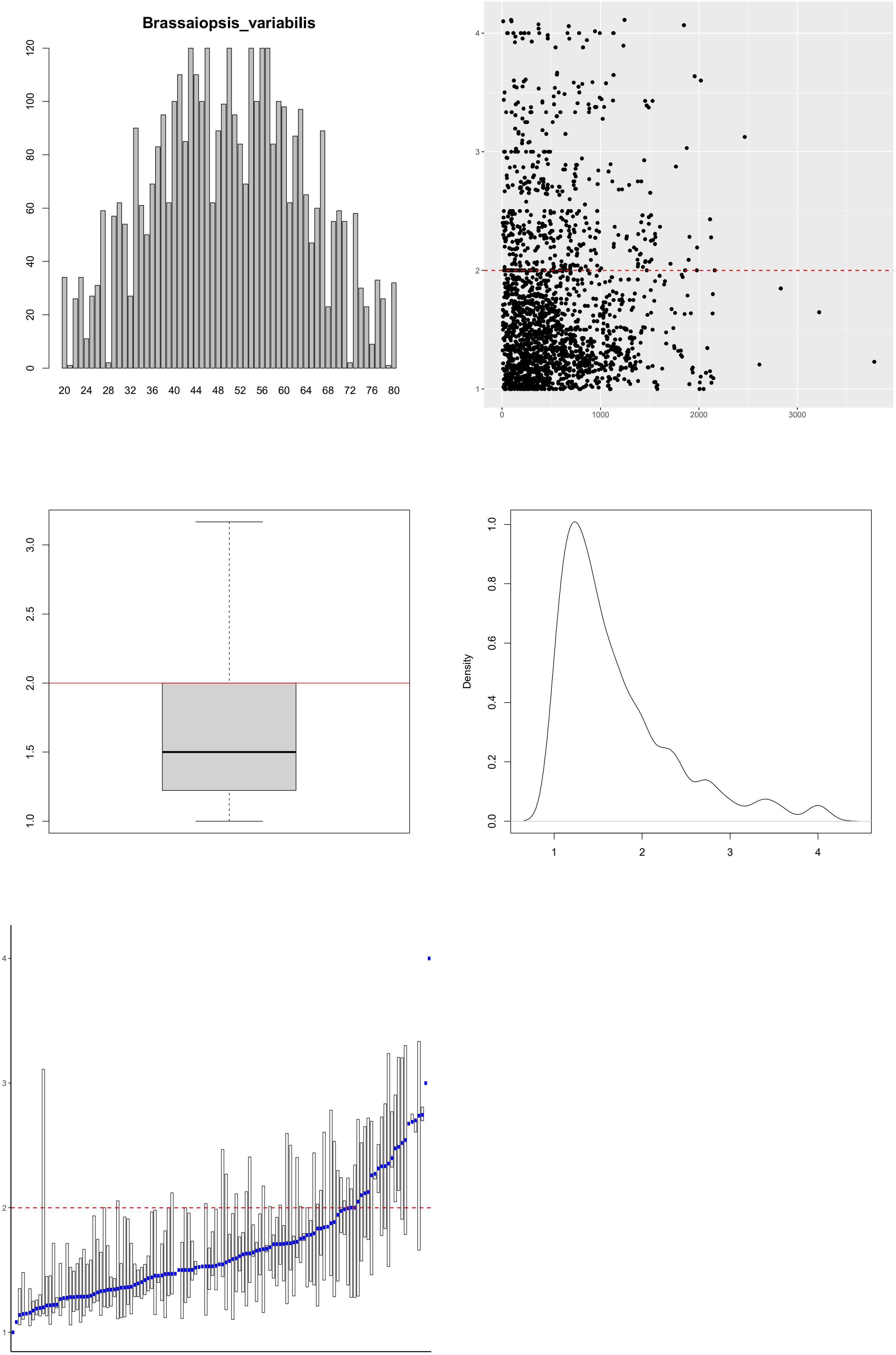
Brassaiopsis_variabilis
Density

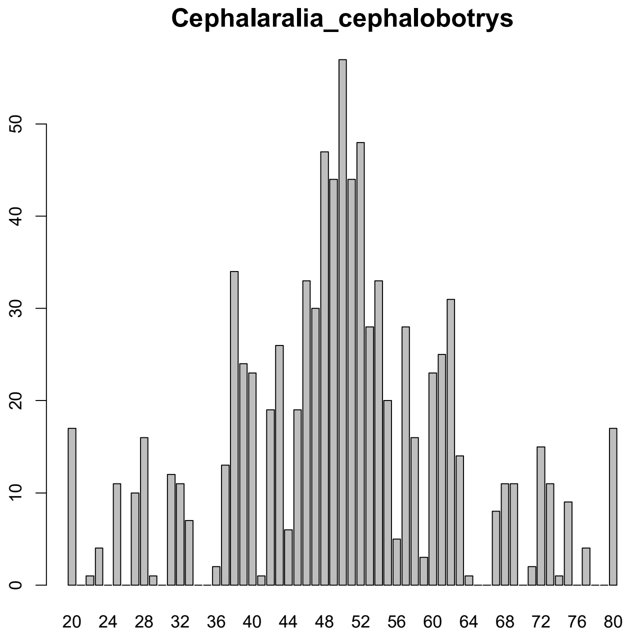
Cephalaralia_cephalobotrys

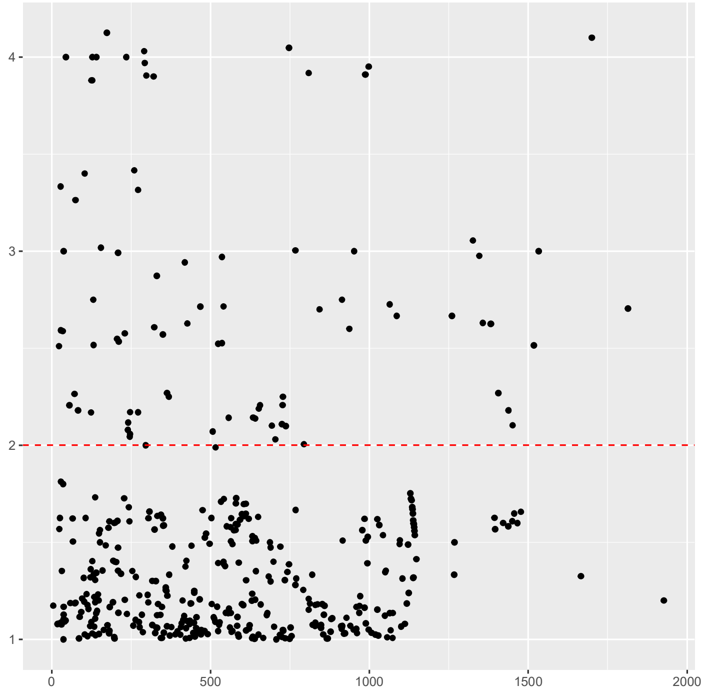

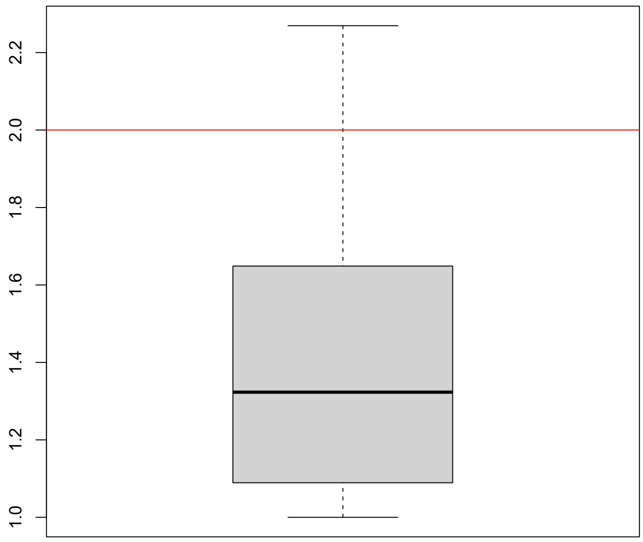

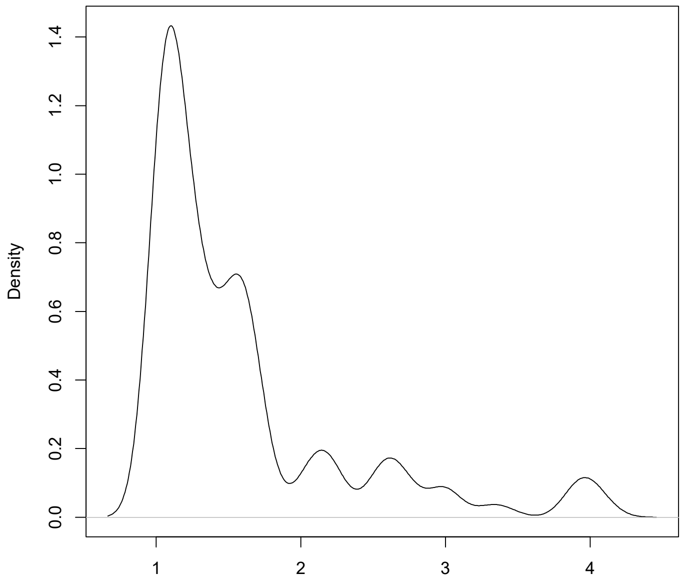
Density

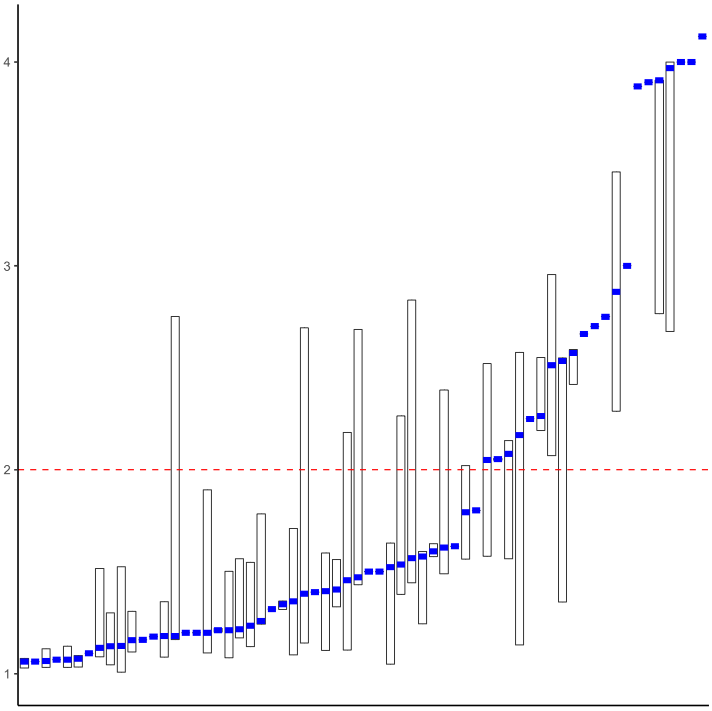

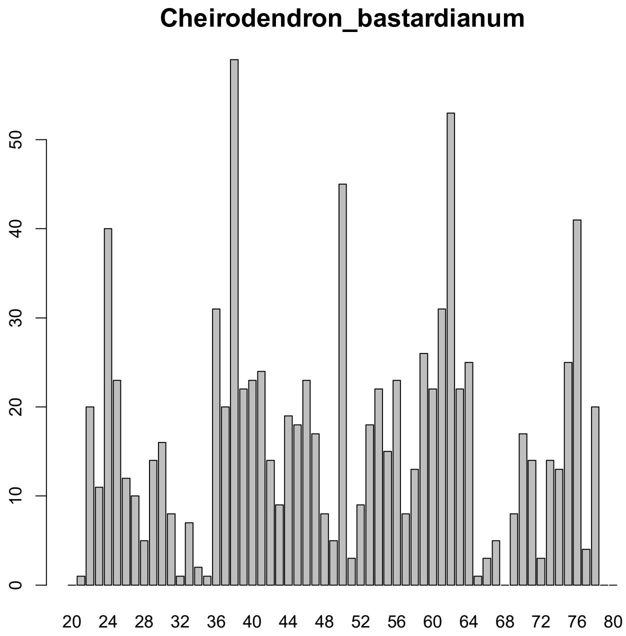
Cheirodendron_bastardianum
0
10
20
30
40
50
20
24
28
32
36
40
44
48
52
56
60
64
68
72
76
80

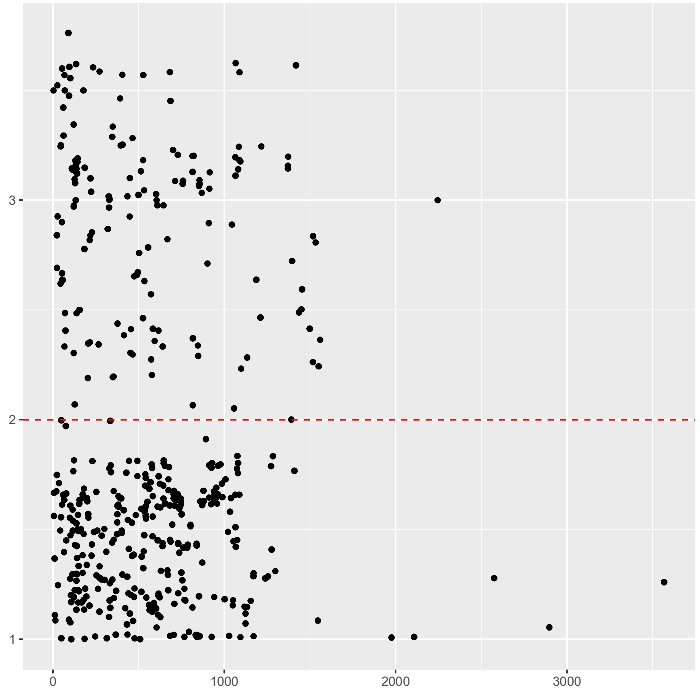
1
2
3
0
1000
2000
3000

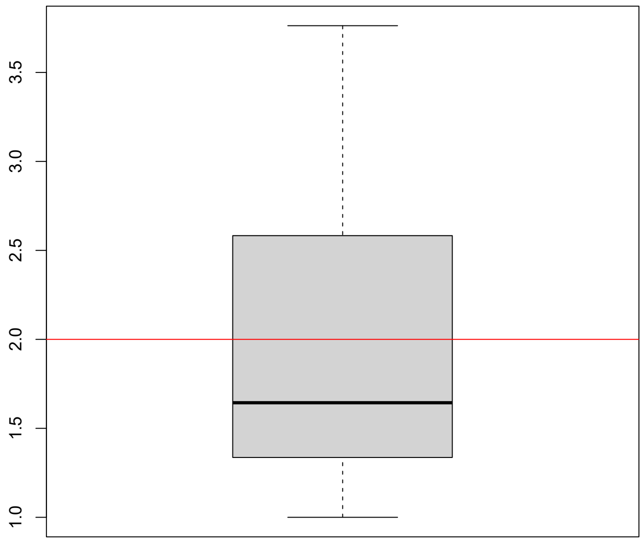
1.0
1.5
2.0
2.5
3.0
3.5

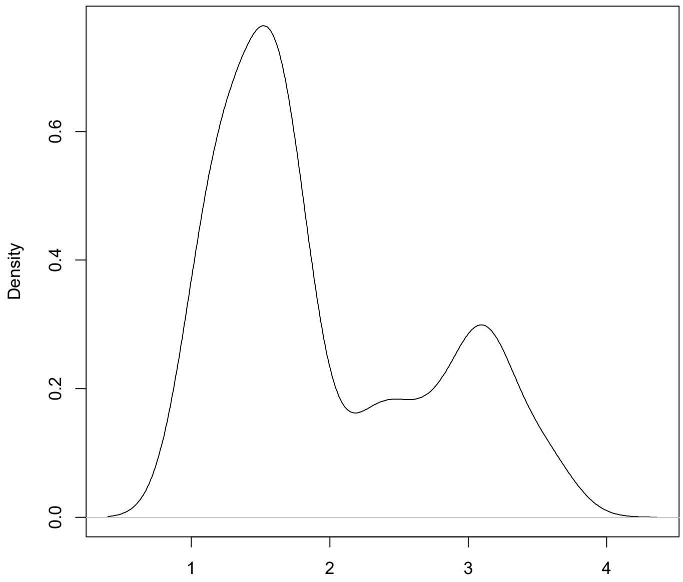
Density
0.0
0.2
0.4
0.6
1
2
3
4

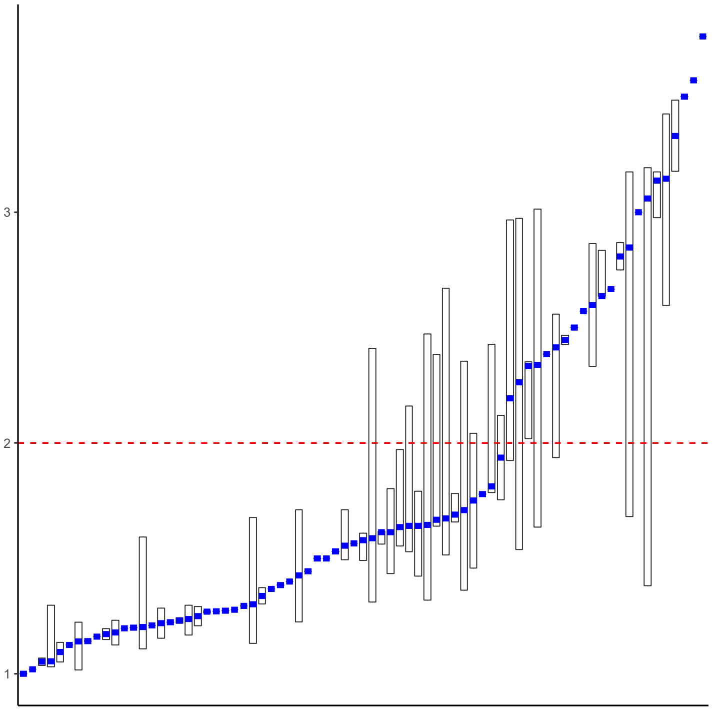
1
2
3

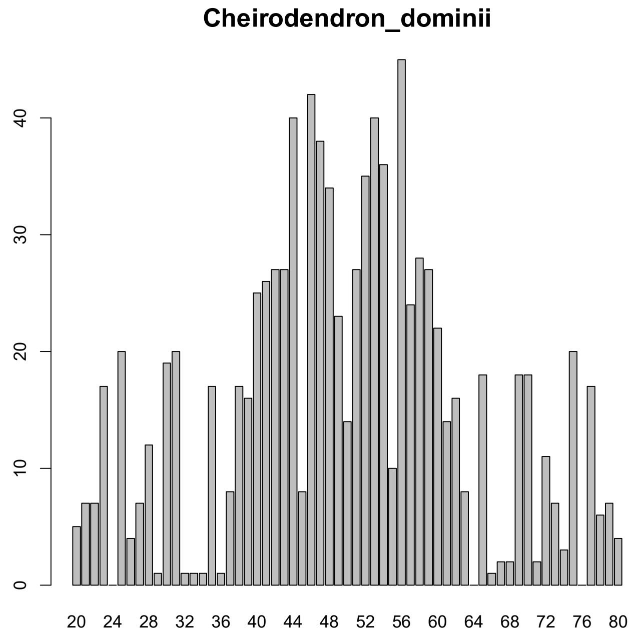
Cheirodendron_dominii

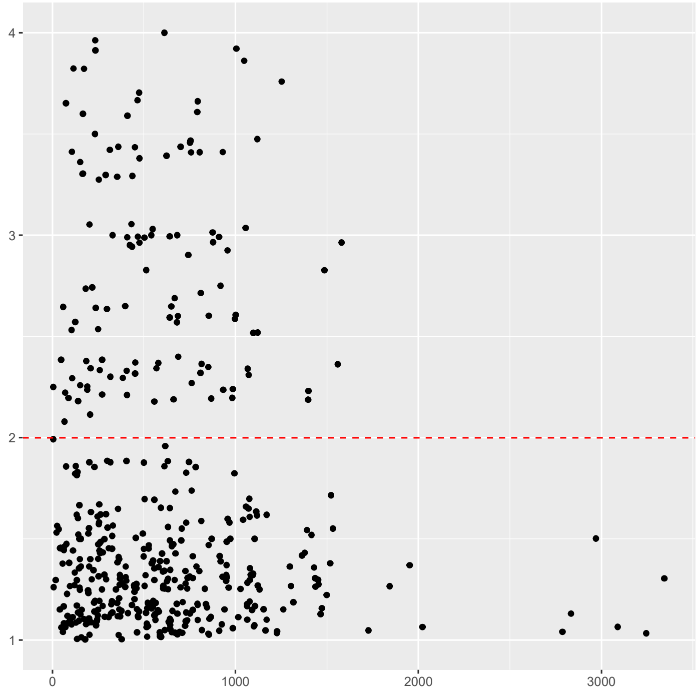

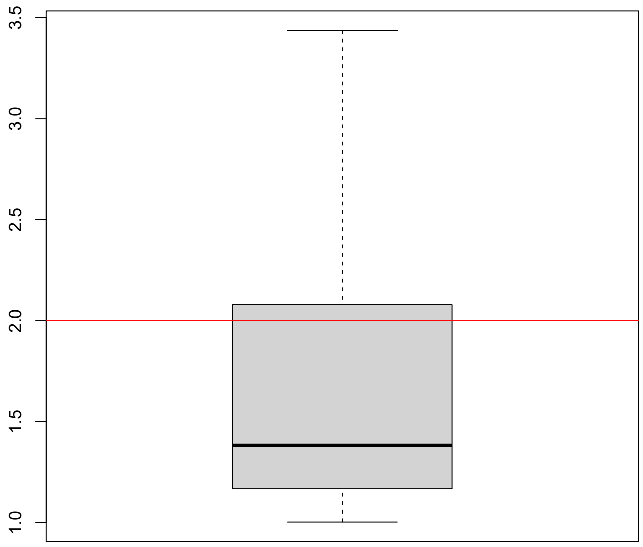

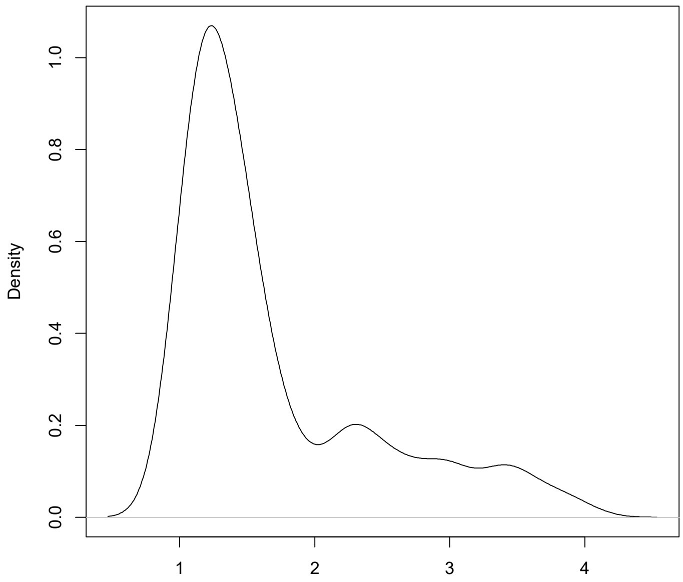
Density

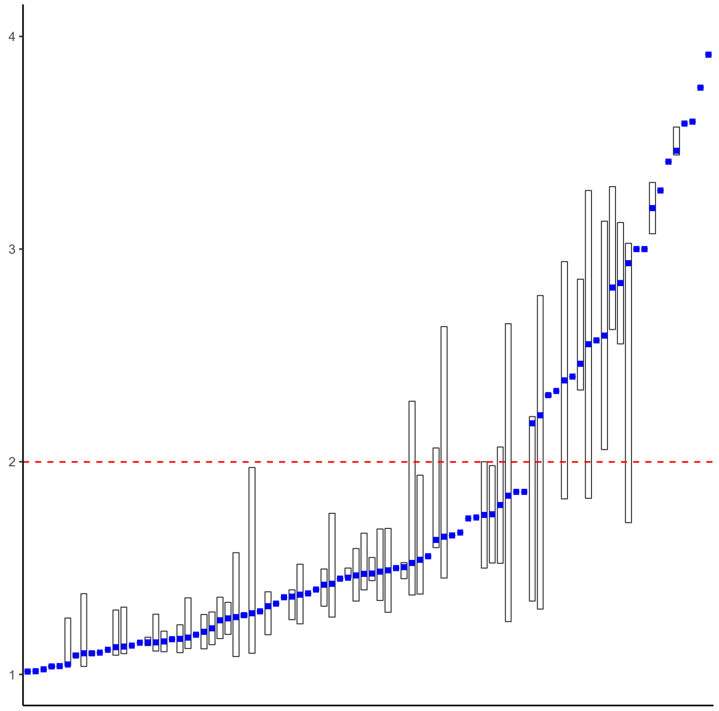

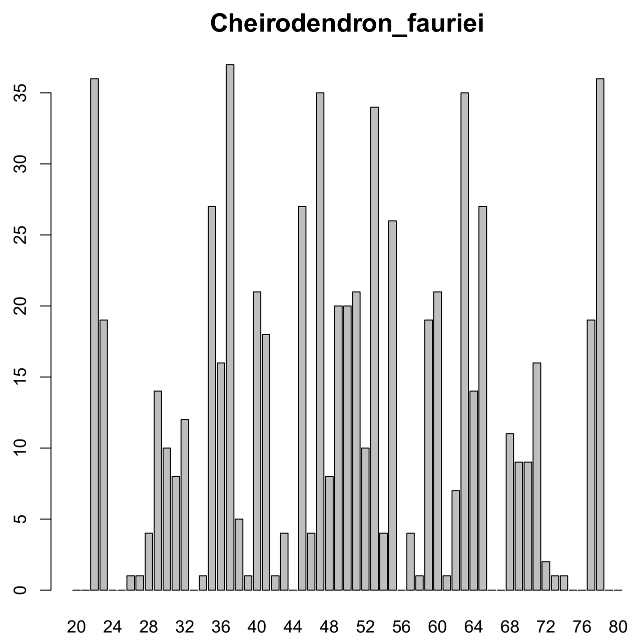
Cheirodendron_fauriei
0 5 10 15 20 25 30 35
20 24 28 32 36 40 44 48 52 56 60 64 68 72 76 80

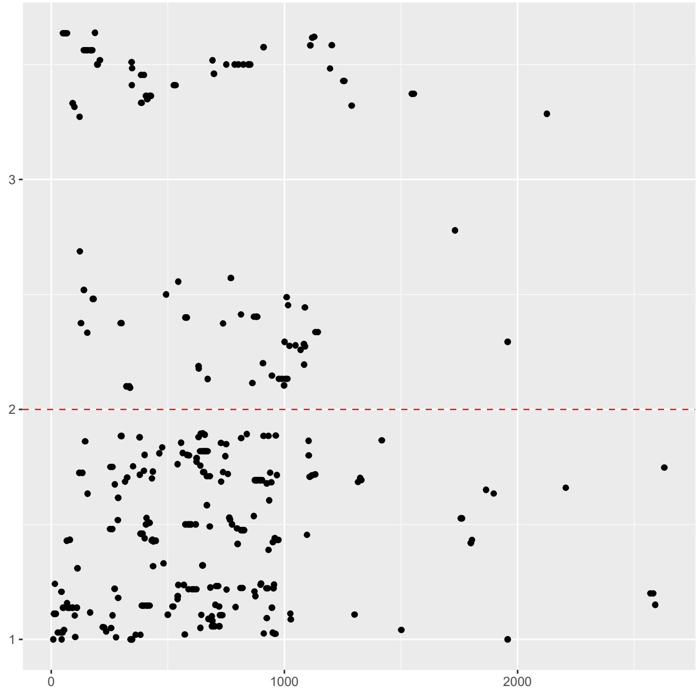
1 2 3
0 1000 2000

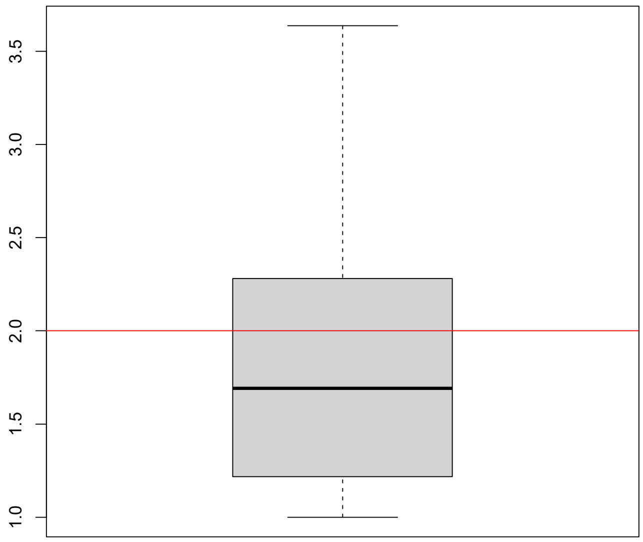
1.0 1.5 2.0 2.5 3.0 3.5

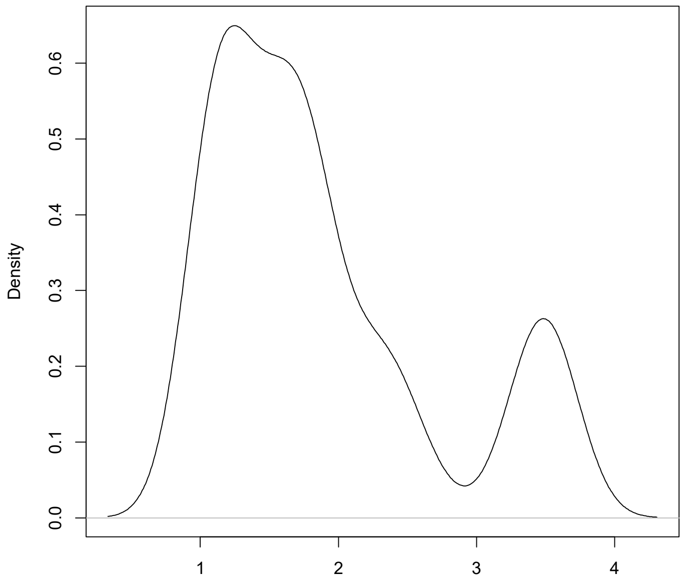
Density
0.0 0.1 0.2 0.3 0.4 0.5 0.6
1 2 3 4

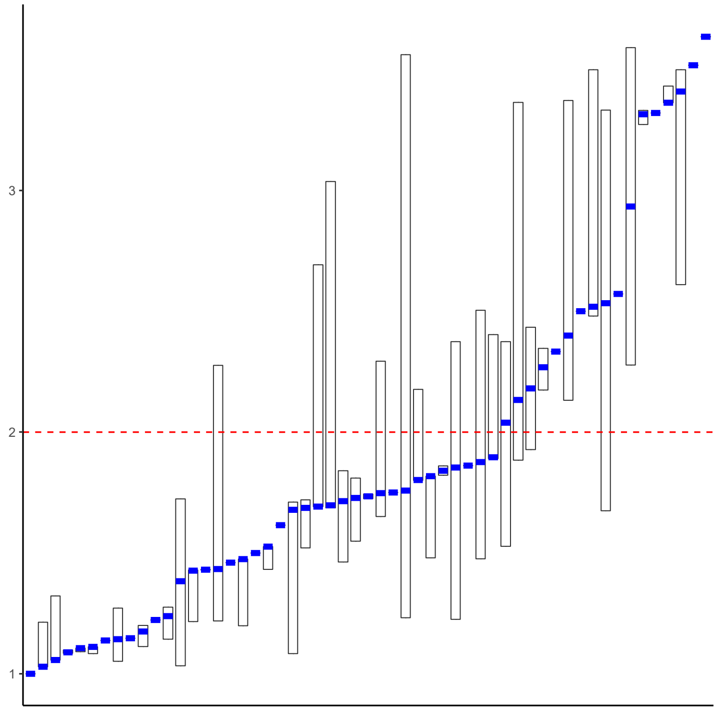
1 2 3

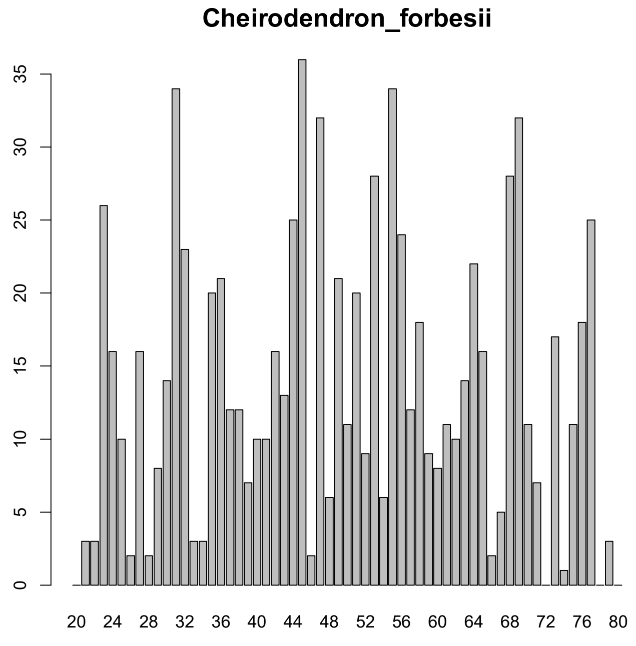
Cheirodendron_forbesii
0 5 10 15 20 25 30 35
20 24 28 32 36 40 44 48 52 56 60 64 68 72 76 80

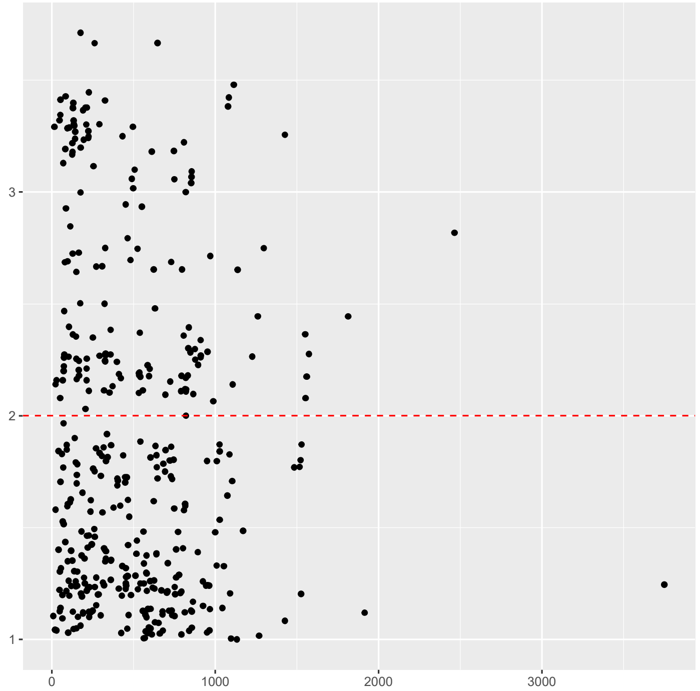
1 2 3
0 1000 2000 3000

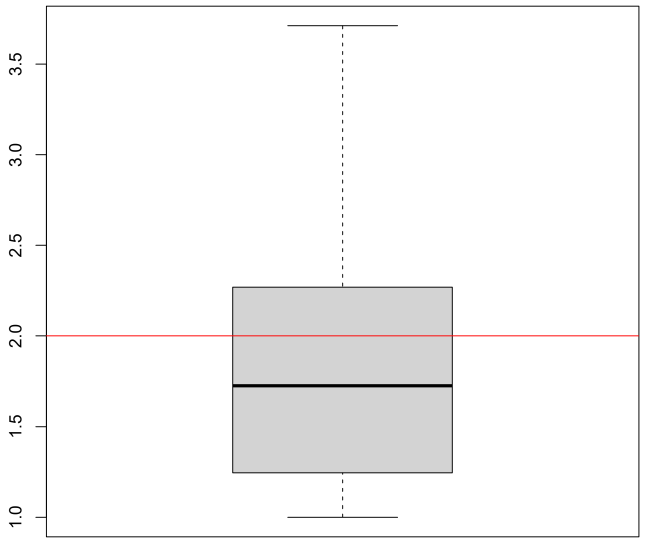
1.0 1.5 2.0 2.5 3.0 3.5

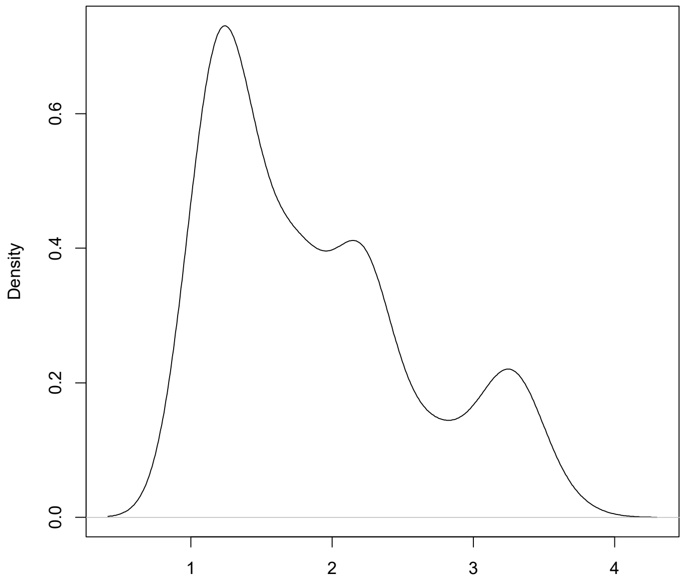
Density
0.0 0.2 0.4 0.6
1 2 3 4

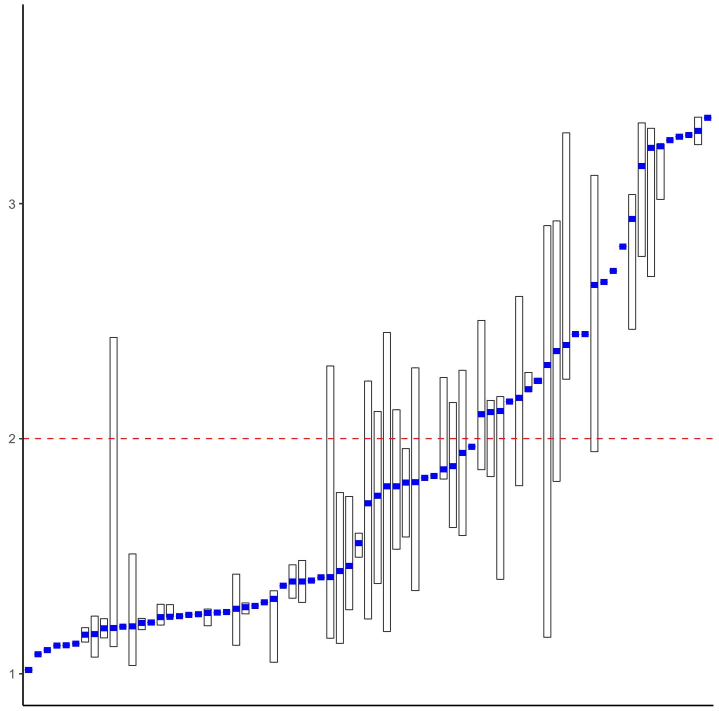
1 2 3

Cheirodendron_platyphyllum

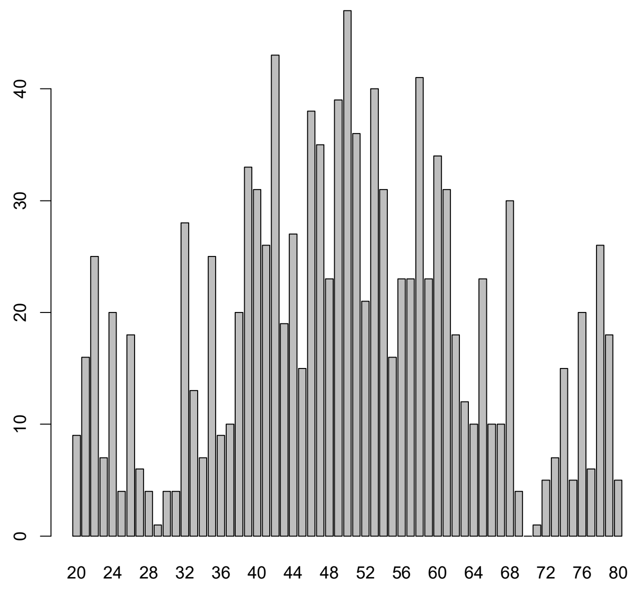

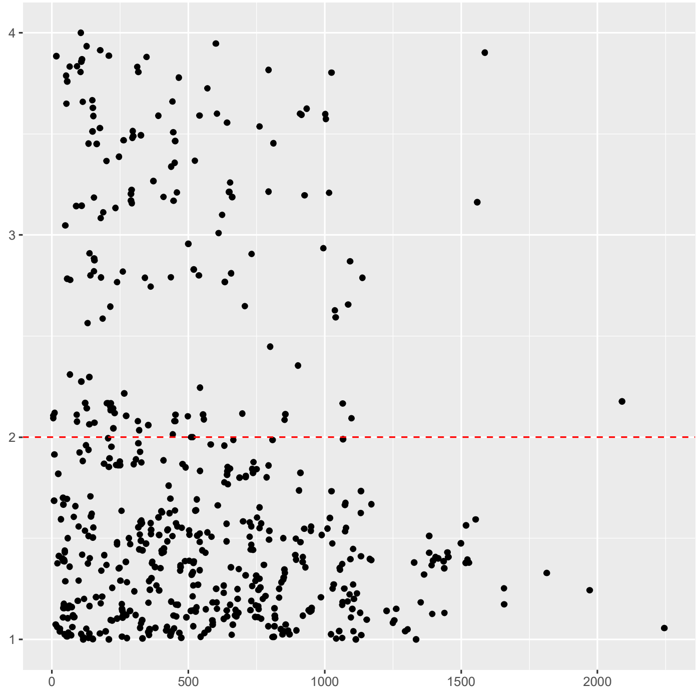

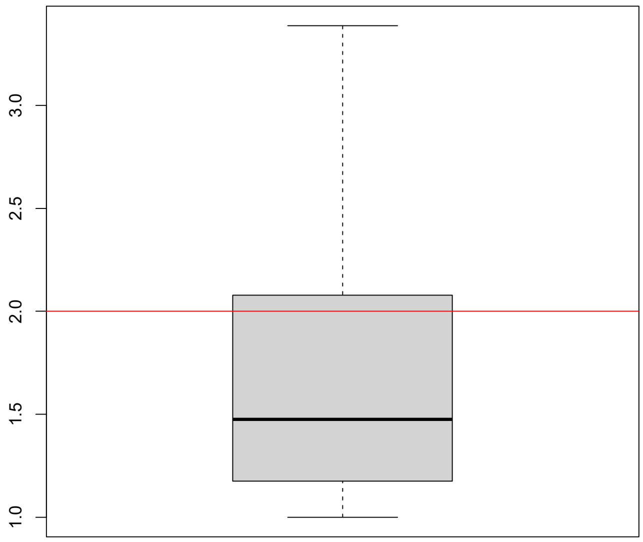

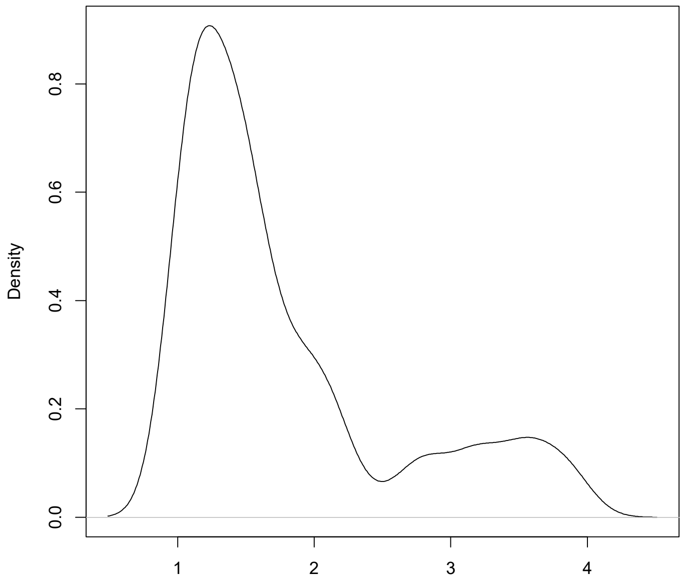

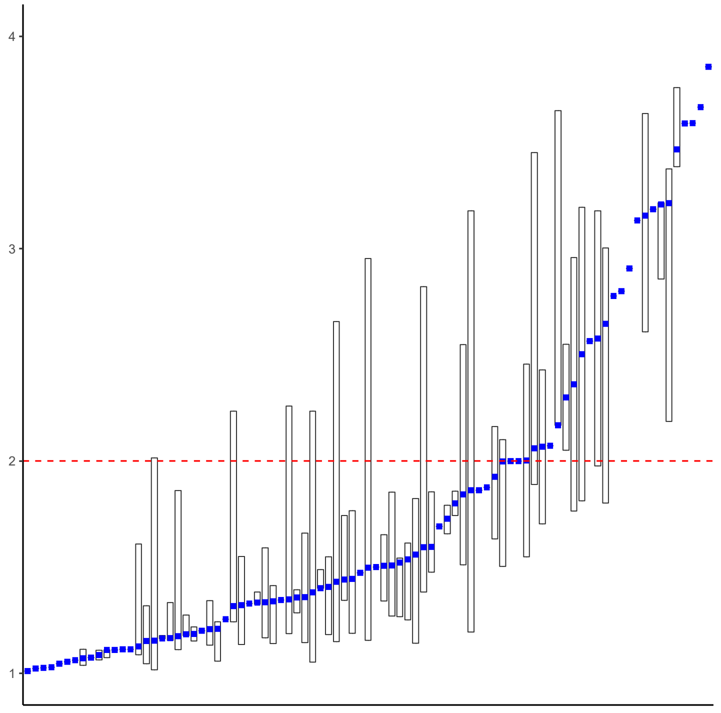

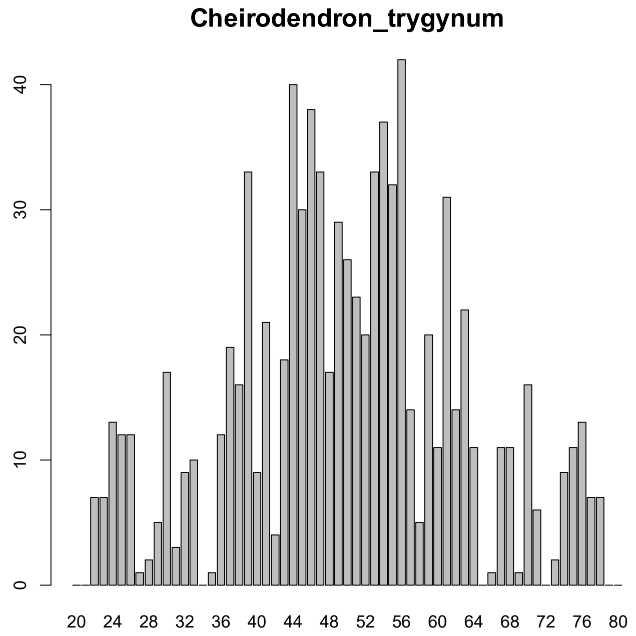
Cheirodendron_trygynum
0
10
20
30
40
20 24 28 32 36 40 44 48 52 56 60 64 68 72 76 80

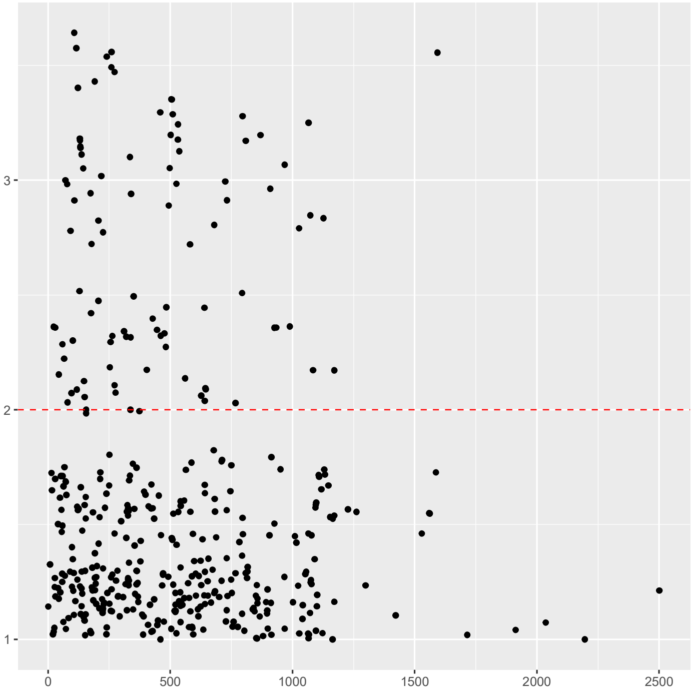
1
2
3
0 500 1000 1500 2000 2500

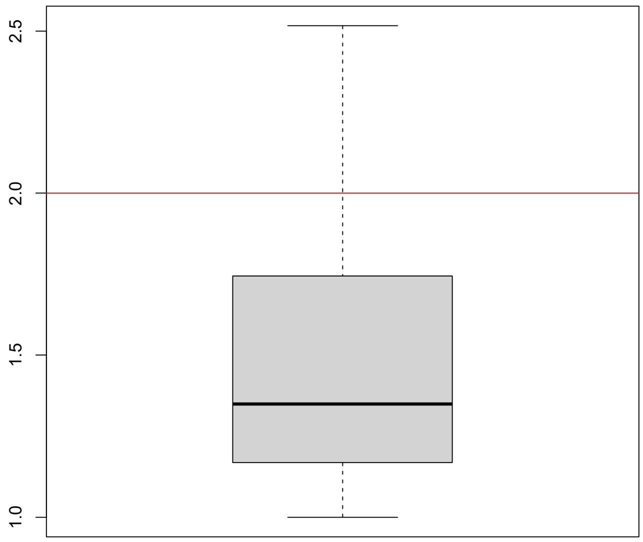
1.0
1.5
2.0
2.5

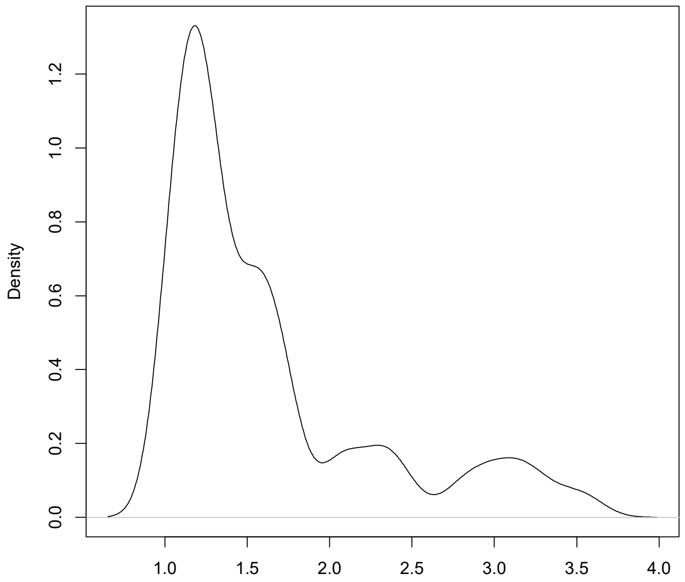
Density
0.0
0.2
0.4
0.6
0.8
1.0
1.2
1.0 1.5 2.0 2.5 3.0 3.5 4.0

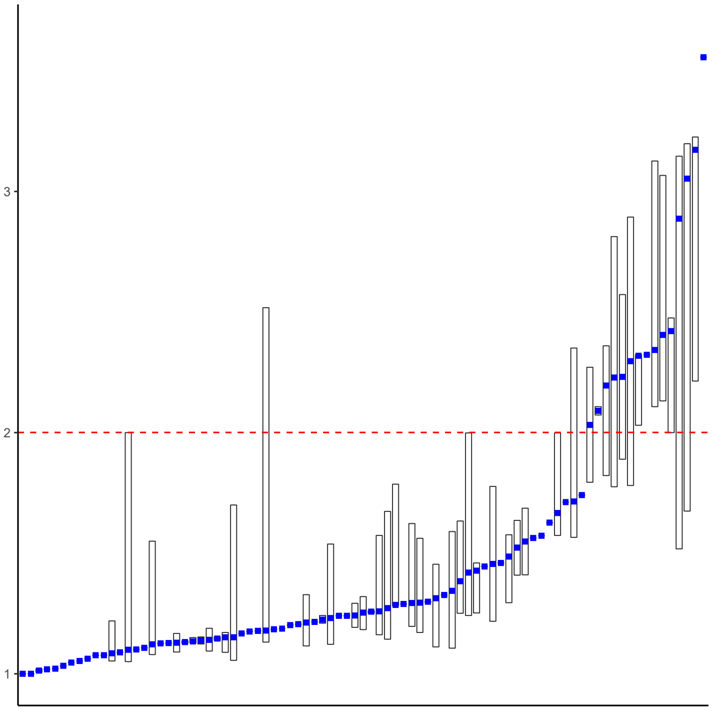
1
2
3

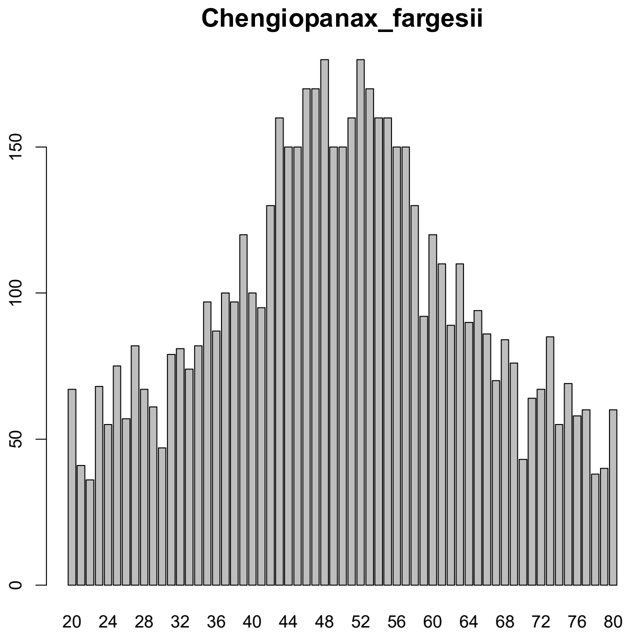
Chengiopanax_fargesii
0
50
100
150
20 24 28 32 36 40 44 48 52 56 60 64 68 72 76 80

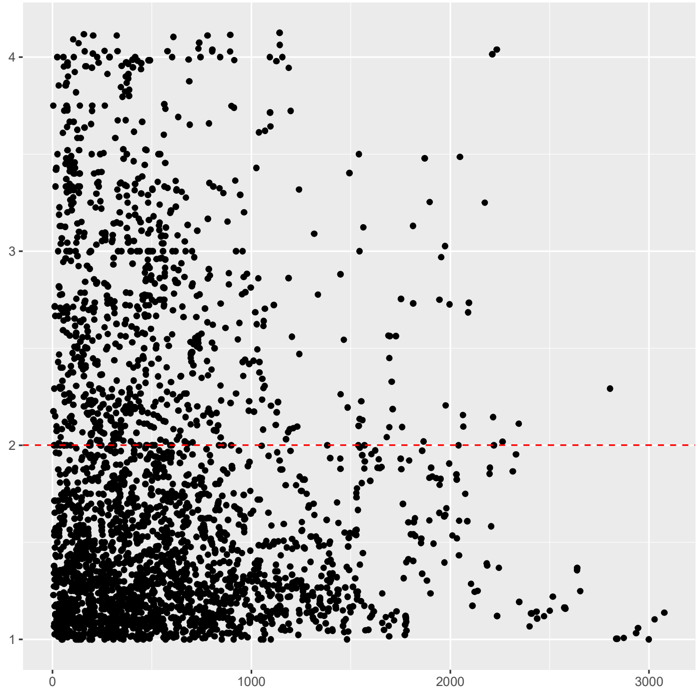
1
2
3
4
0
1000
2000
3000

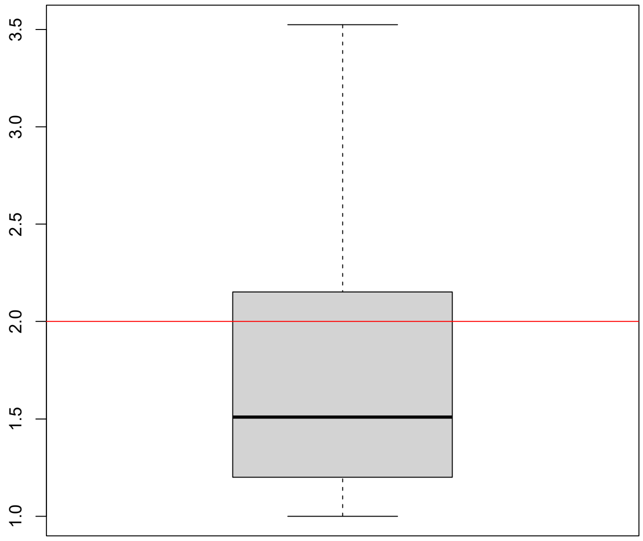
1.0
1.5
2.0
2.5
3.0
3.5

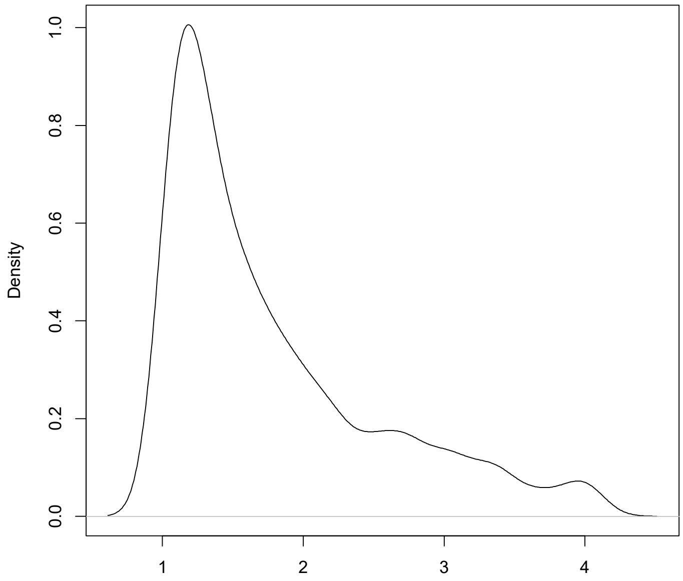
Density
0.0
0.2
0.4
0.6
0.8
1.0
1
2
3
4

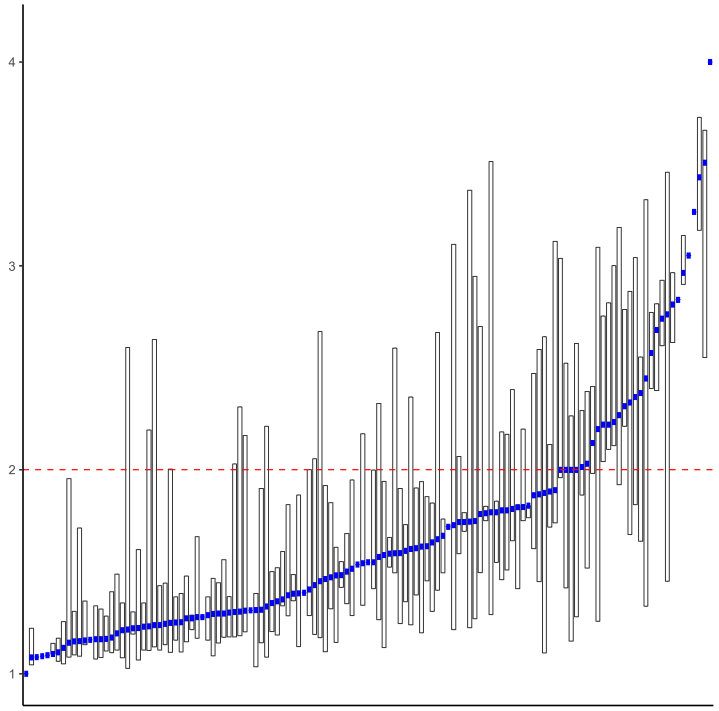
1
2
3
4

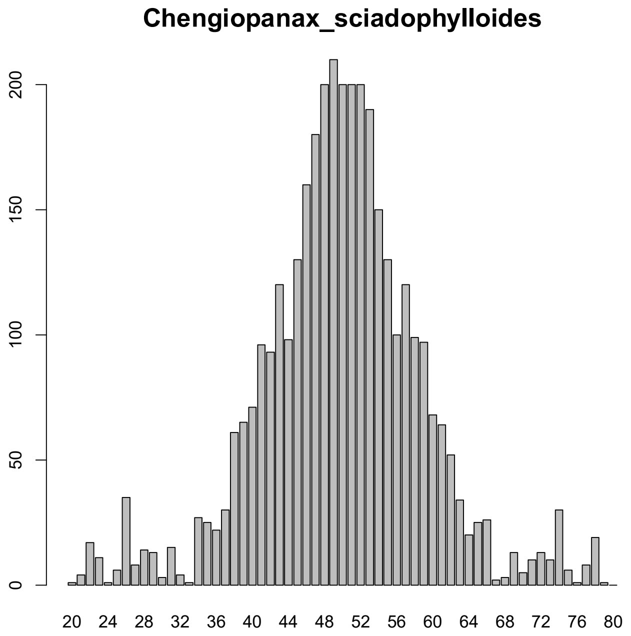
Chengiopanax_sciadophylloides
0
50
100
150
200
20 24 28 32 36 40 44 48 52 56 60 64 68 72 76 80

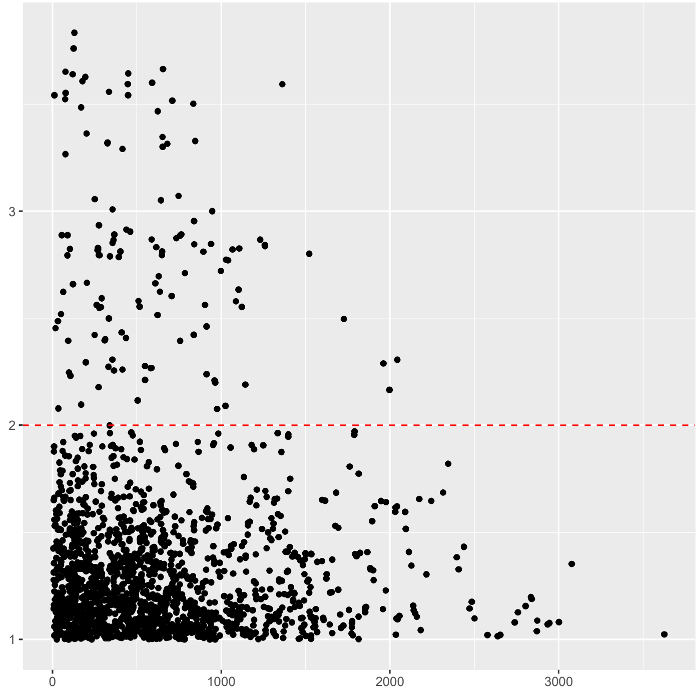
1
2
3
0 1000 2000 3000

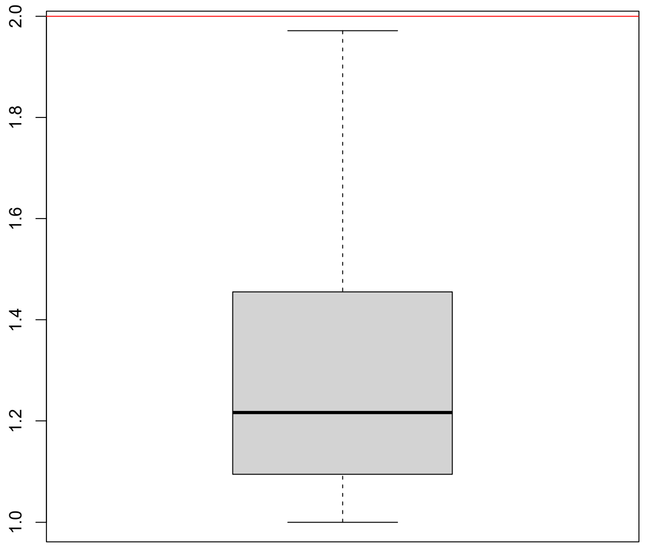
1.0
1.2
1.4
1.6
1.8
2.0

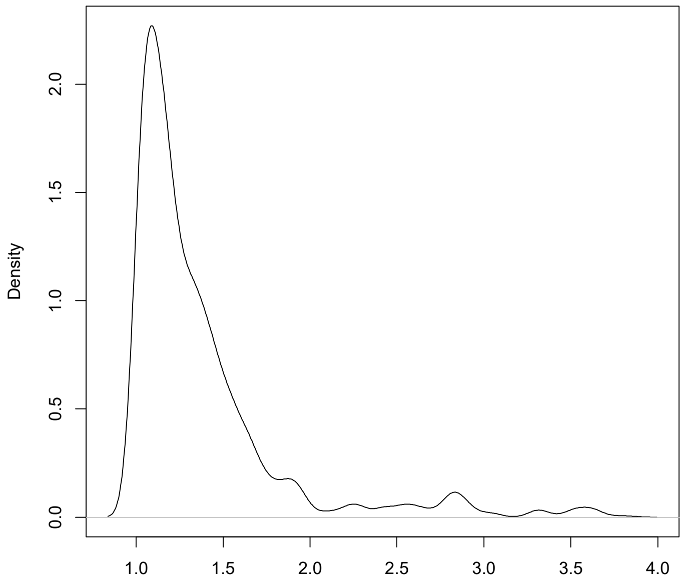
Density
0.0
0.5
1.0
1.5
2.0
1.0 1.5 2.0 2.5 3.0 3.5 4.0

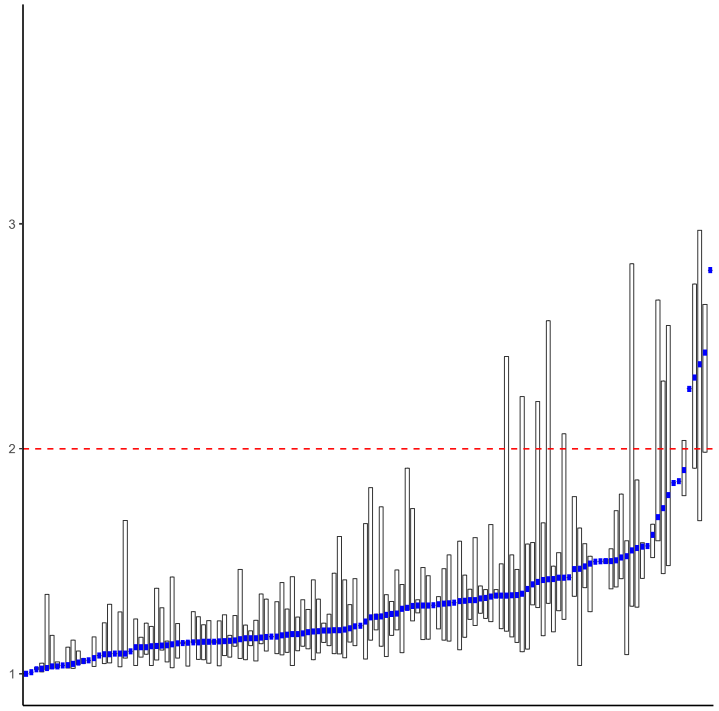
1
2
3

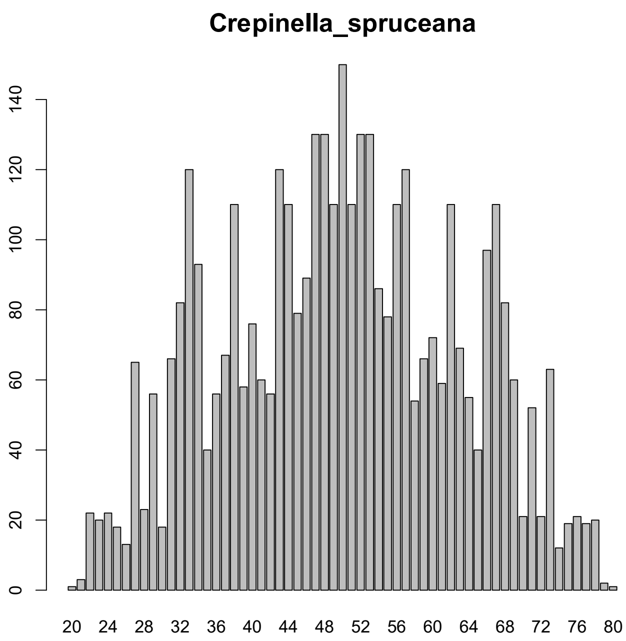
Crepinella_spruceana

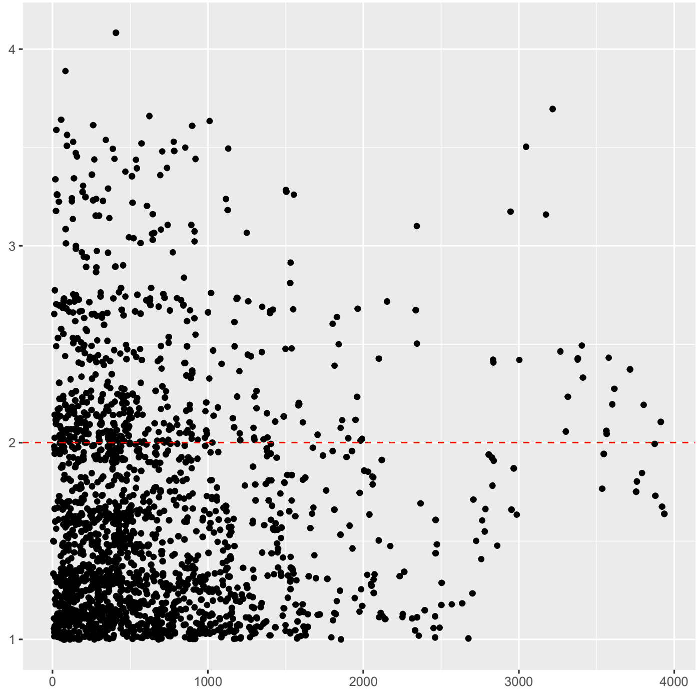

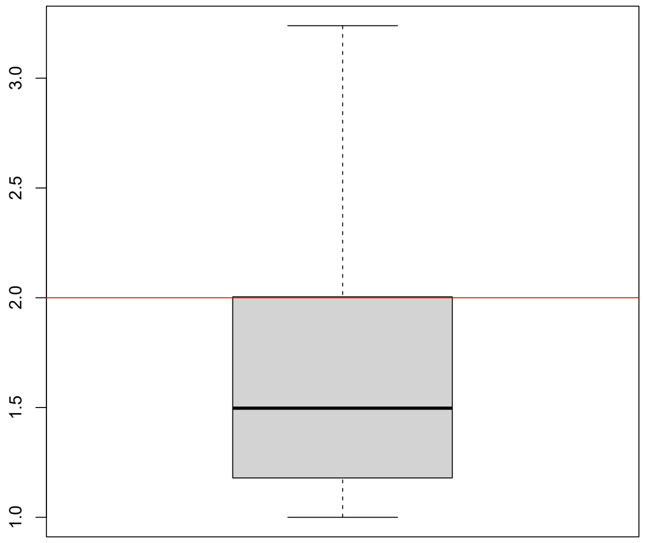

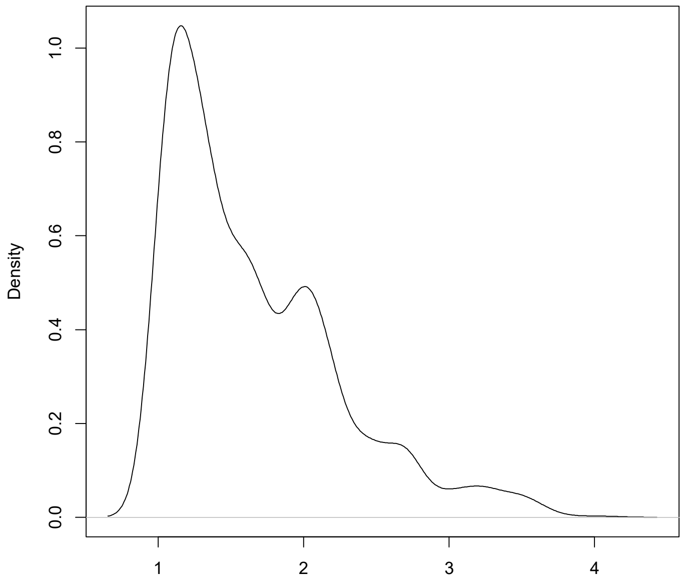
Density

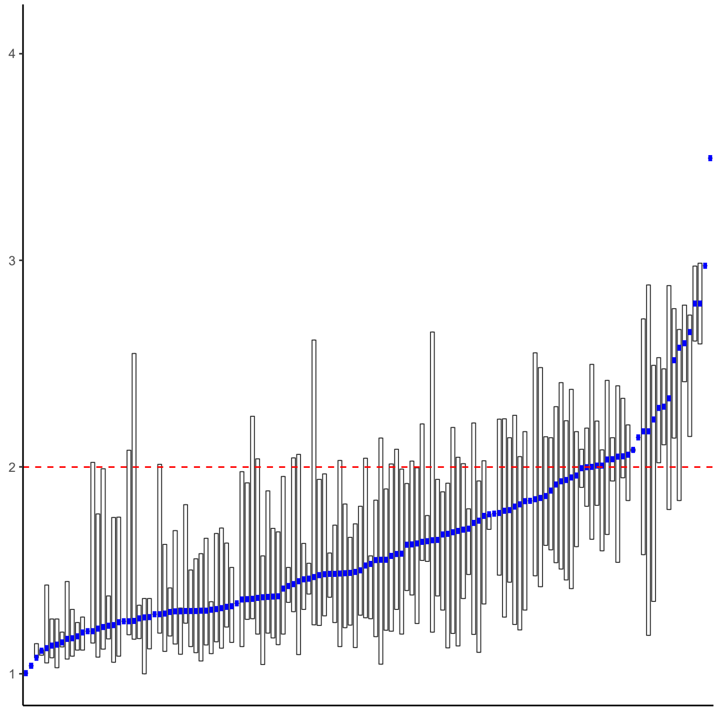

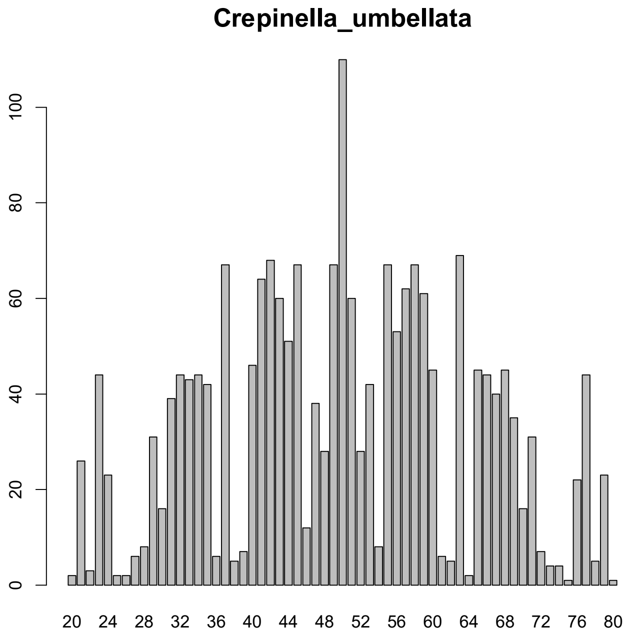
Crepinella_umbellata
0
20
40
60
80
100
20 24 28 32 36 40 44 48 52 56 60 64 68 72 76 80

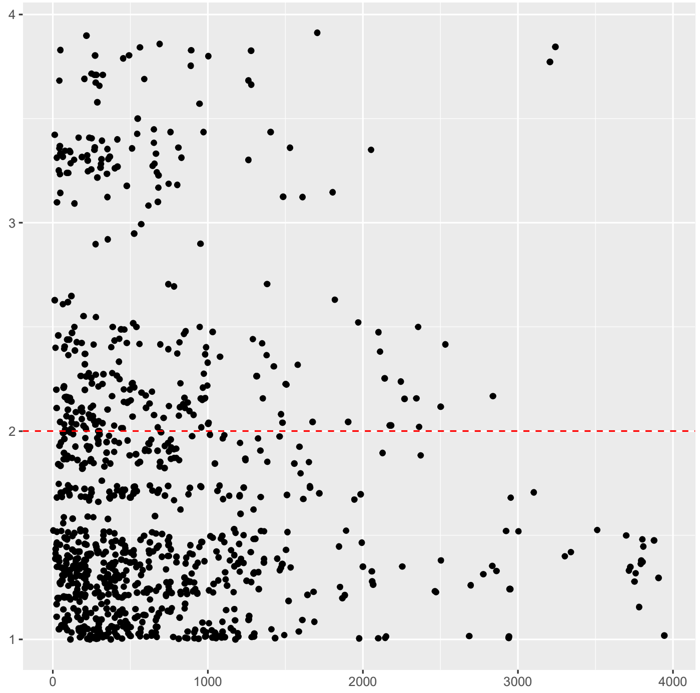
1
2
3
4
0 1000 2000 3000 4000

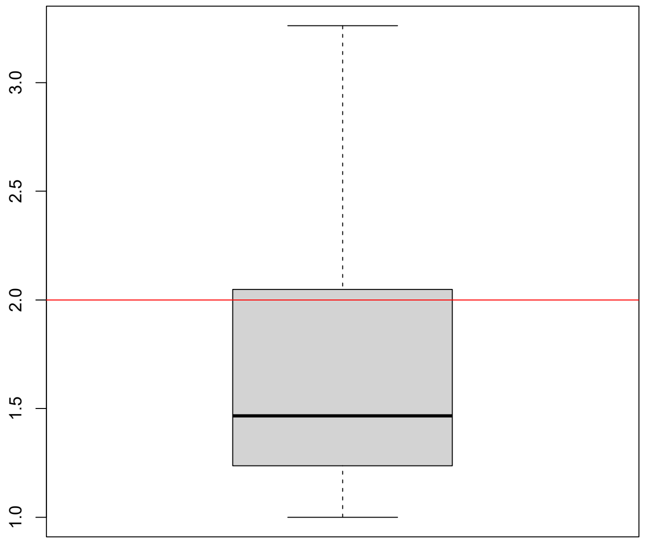
1.0
1.5
2.0
2.5
3.0

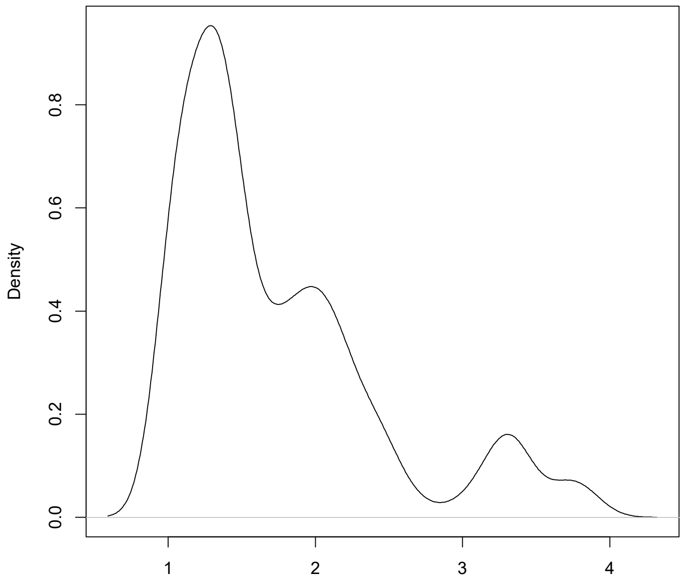
Density
0.0
0.2
0.4
0.6
0.8
1 2 3 4

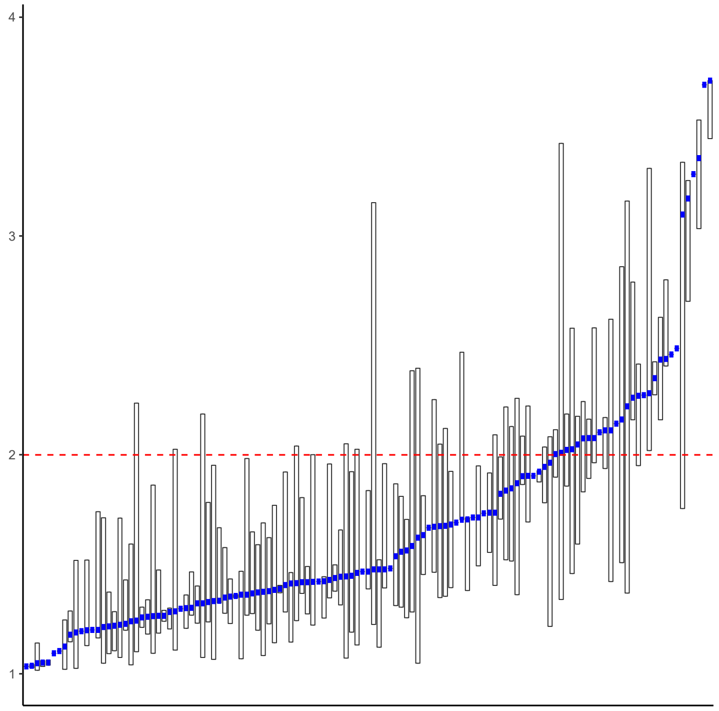
1
2
3
4

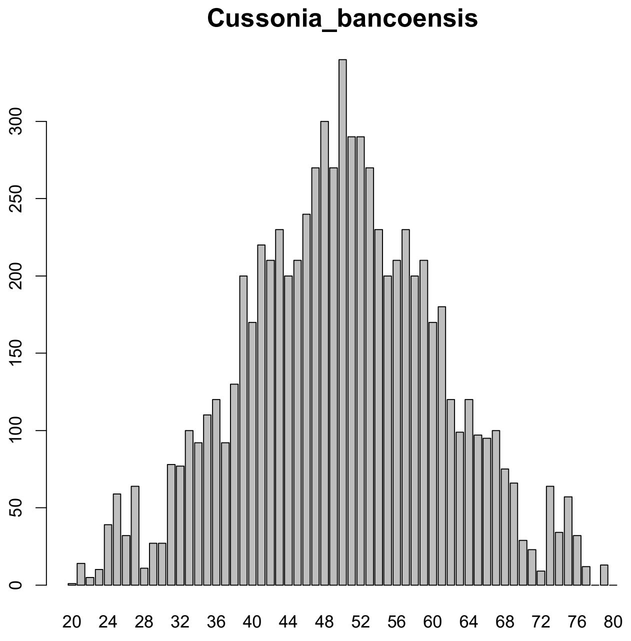
Cussonia_bancoensis
0
50
100
150
200
250
300
20 24 28 32 36 40 44 48 52 56 60 64 68 72 76 80

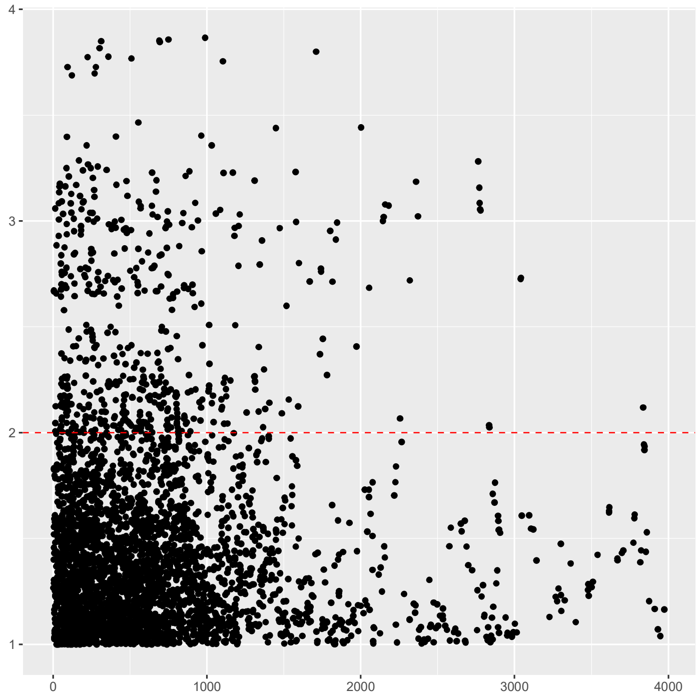
1
2
3
4
0 1000 2000 3000 4000

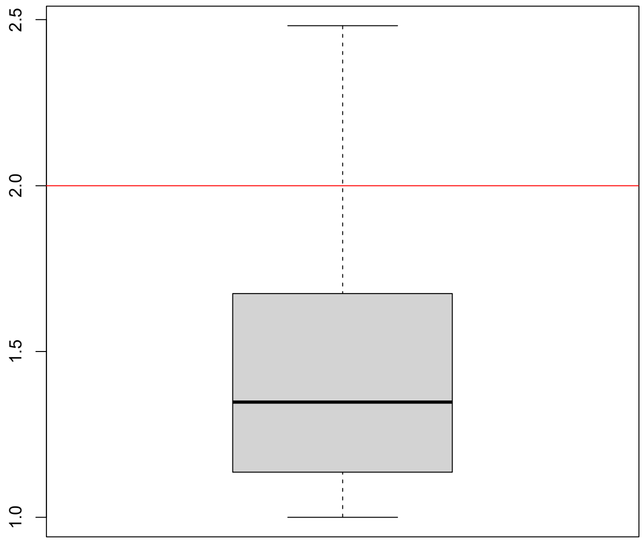
1.0
1.5
2.0
2.5

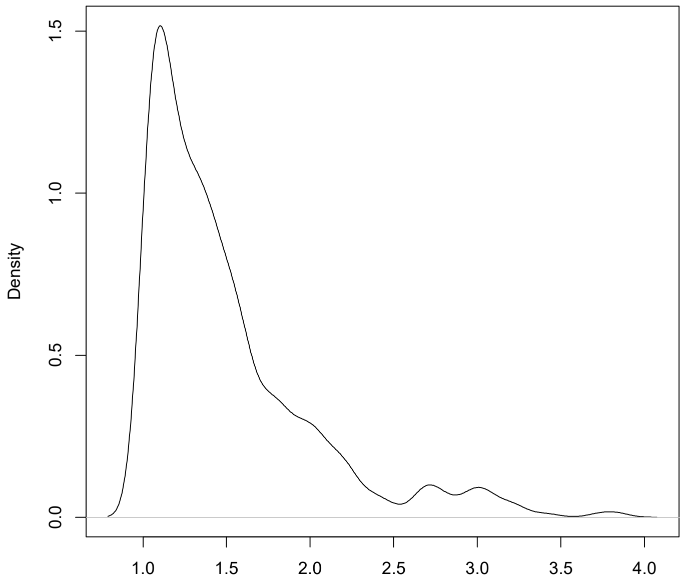
Density
0.0
0.5
1.0
1.5
1.0 1.5 2.0 2.5 3.0 3.5 4.0

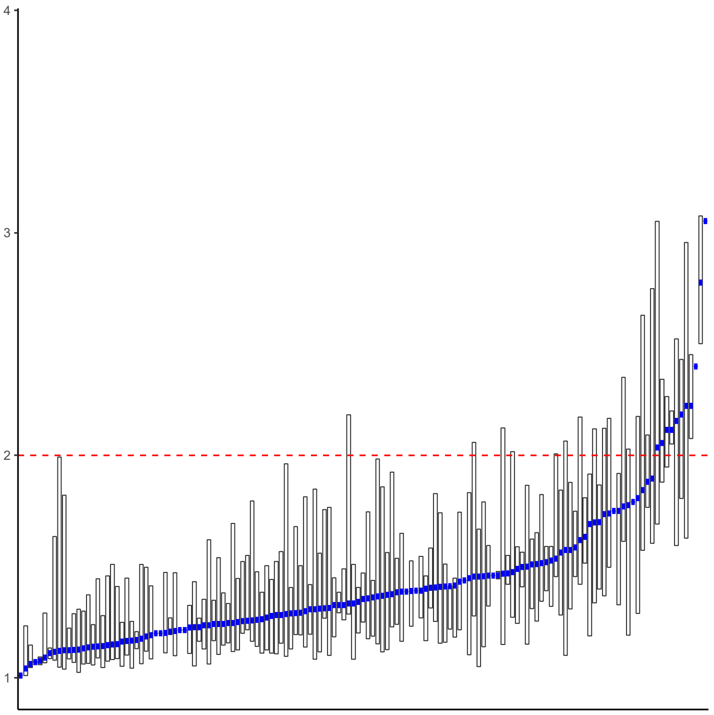
1
2
3
4

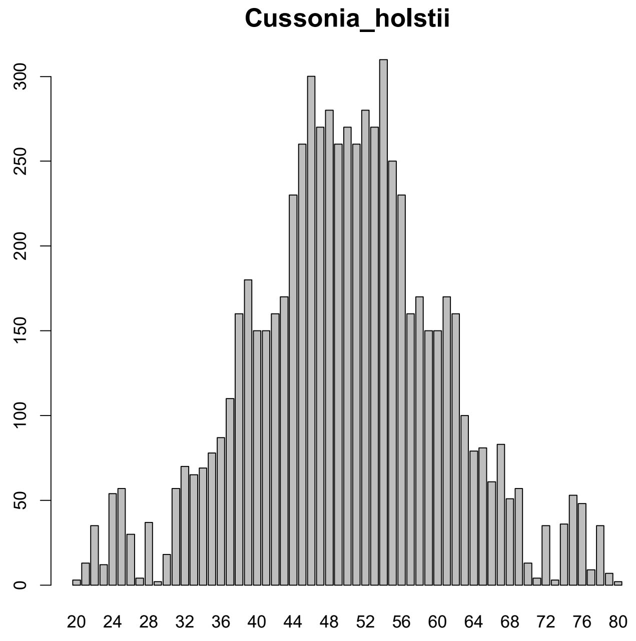
Cussonia_holstii

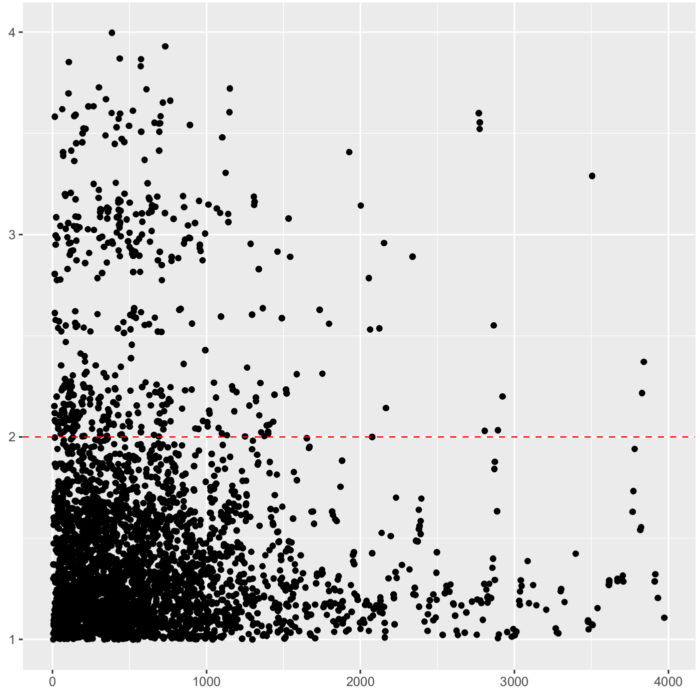

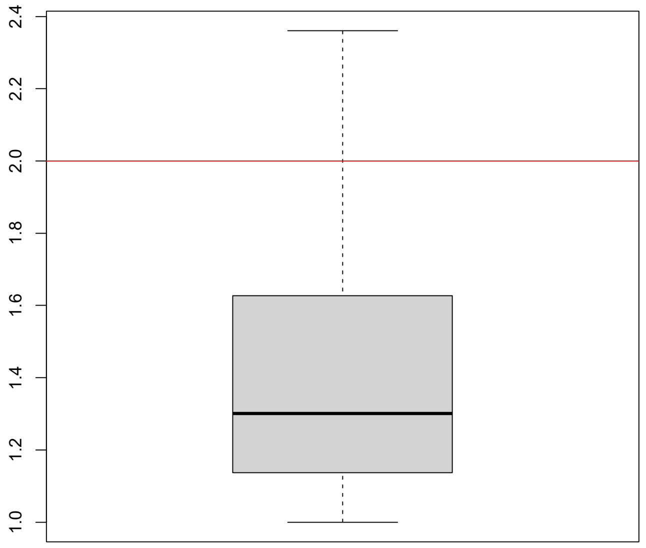

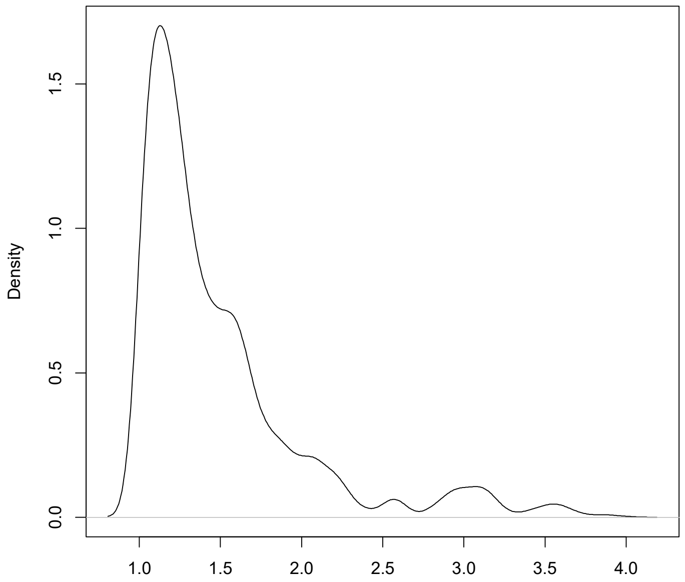
Density

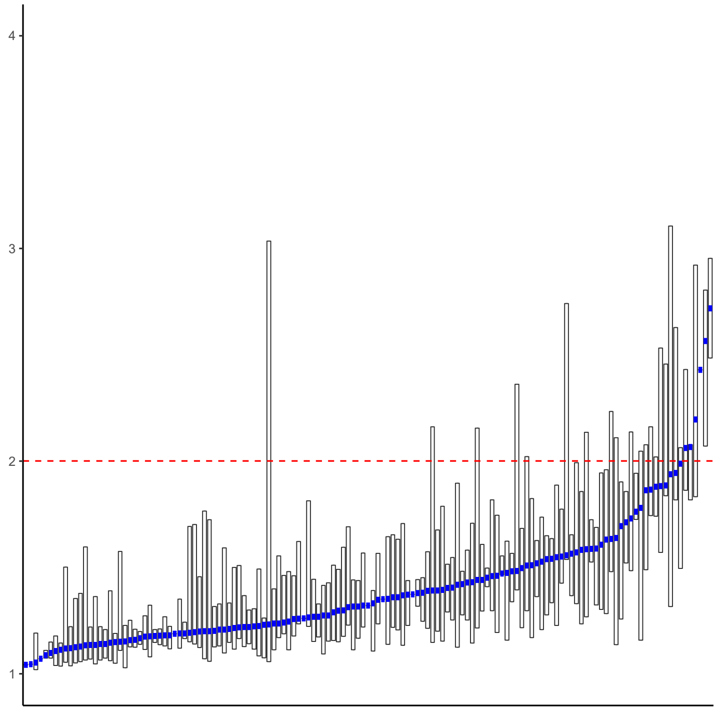

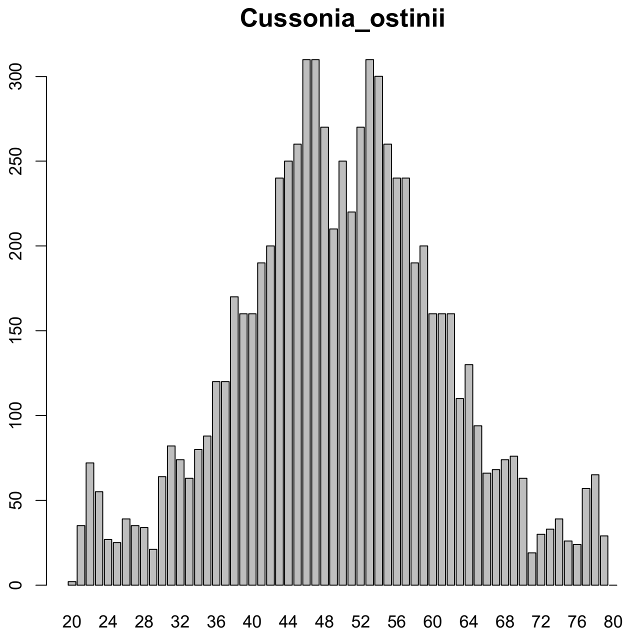
Cussonia_ostinii

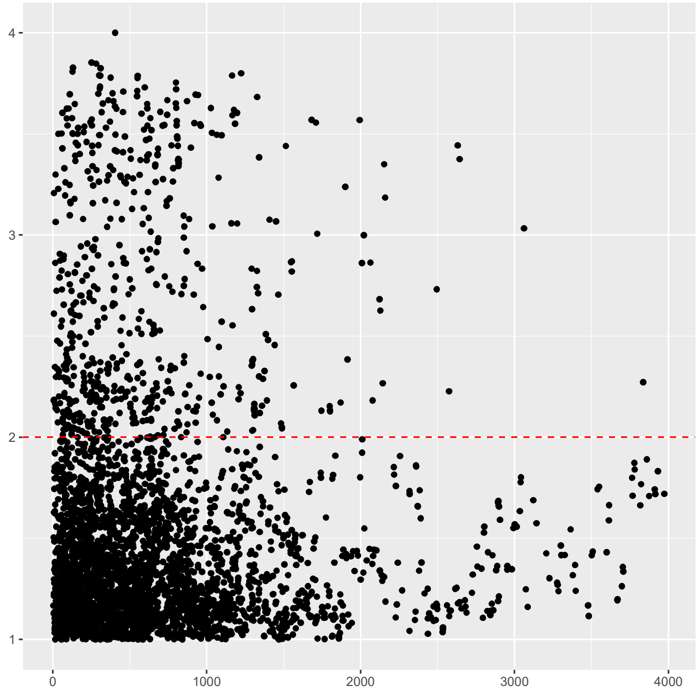

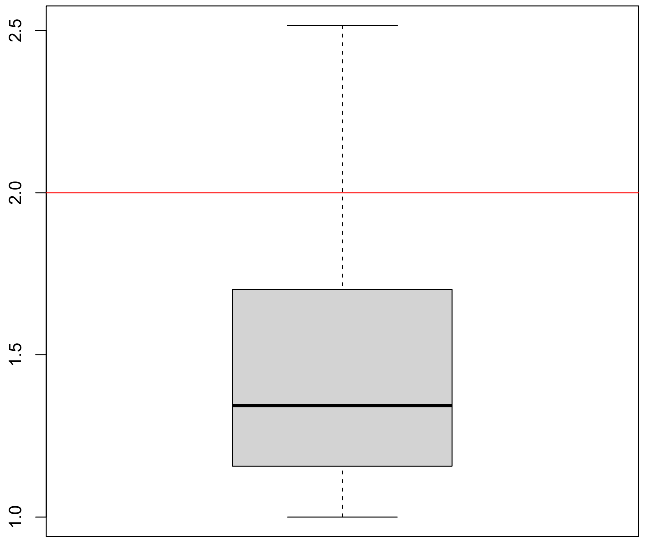

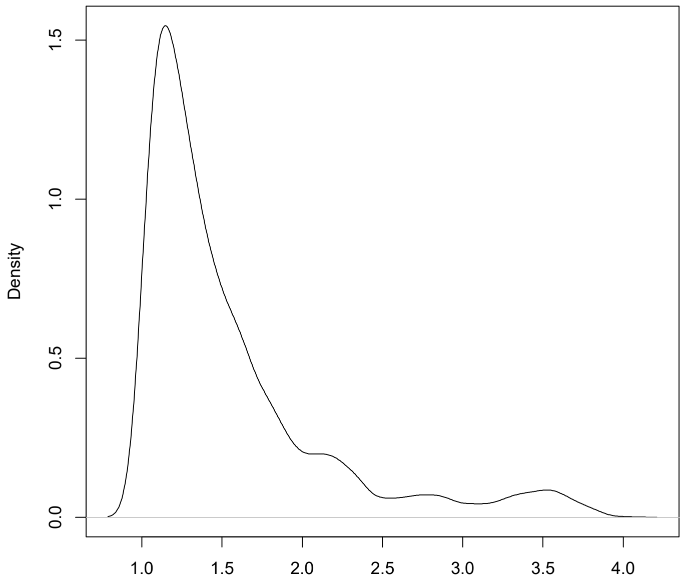
Density

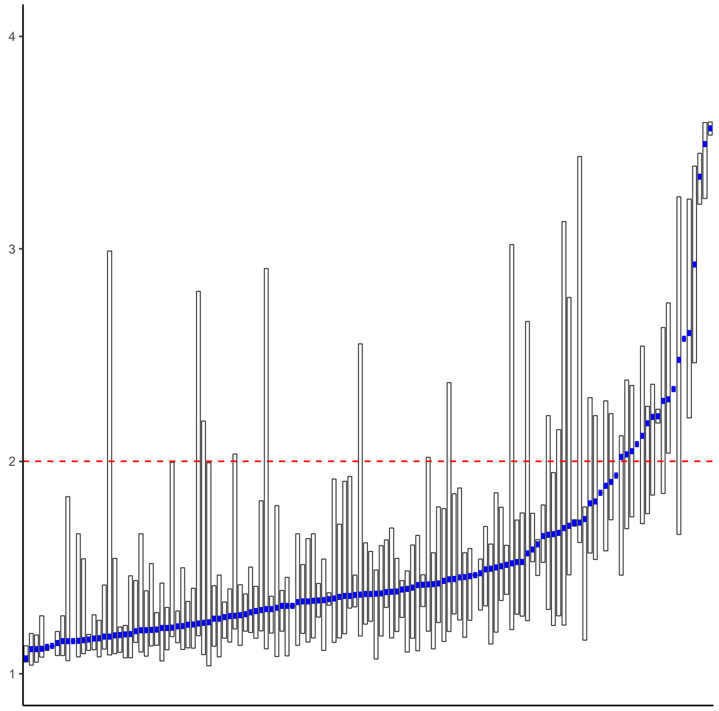

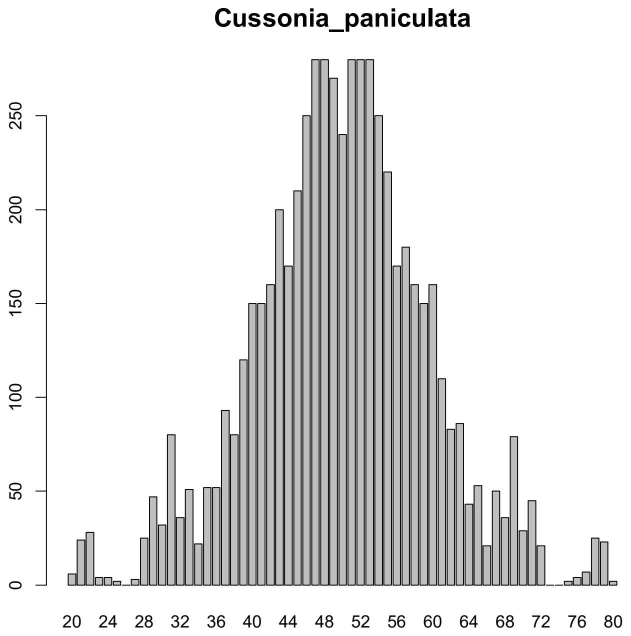
Cussonia_paniculata

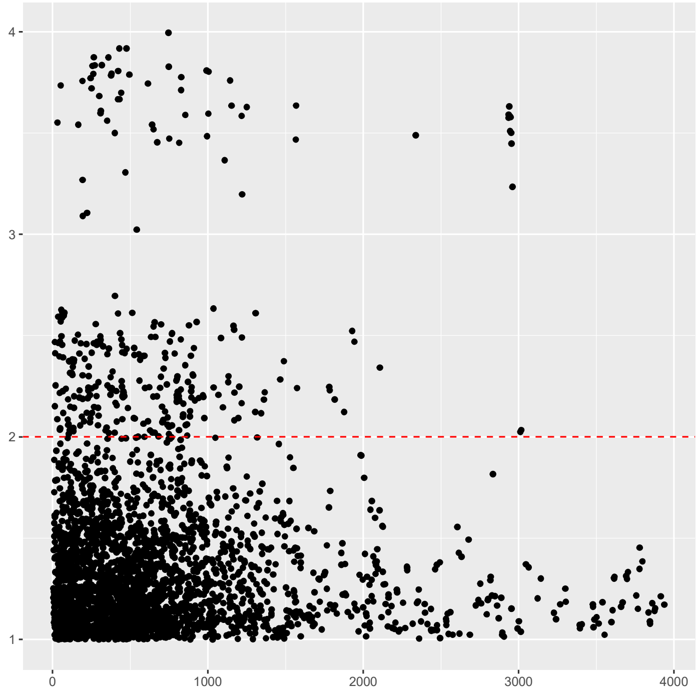

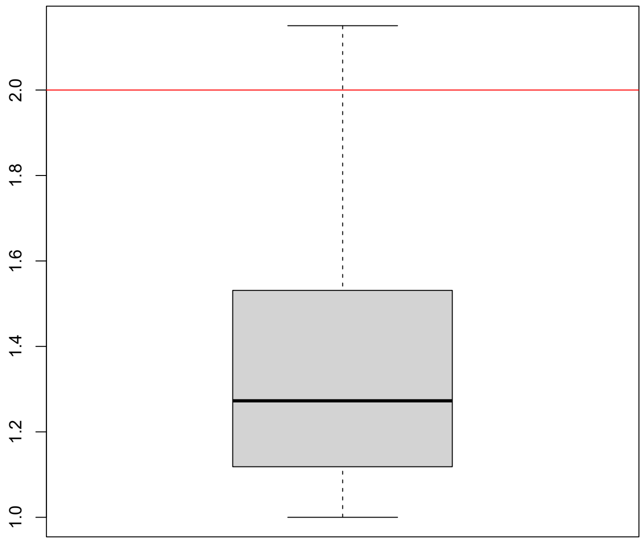

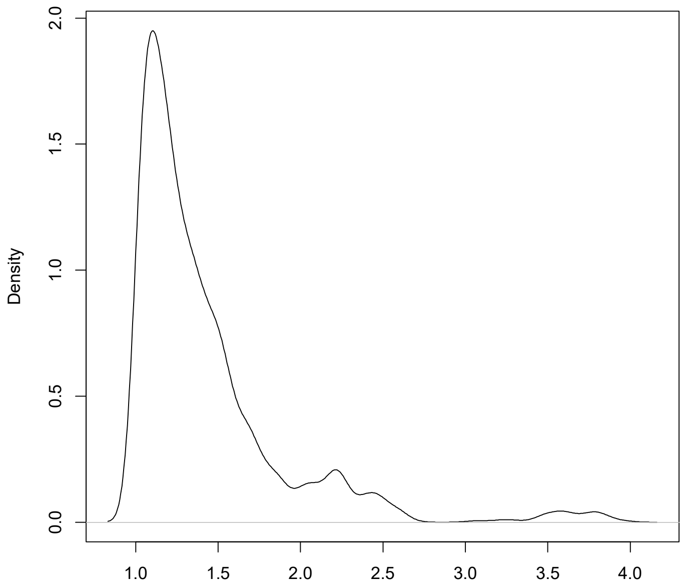
Density

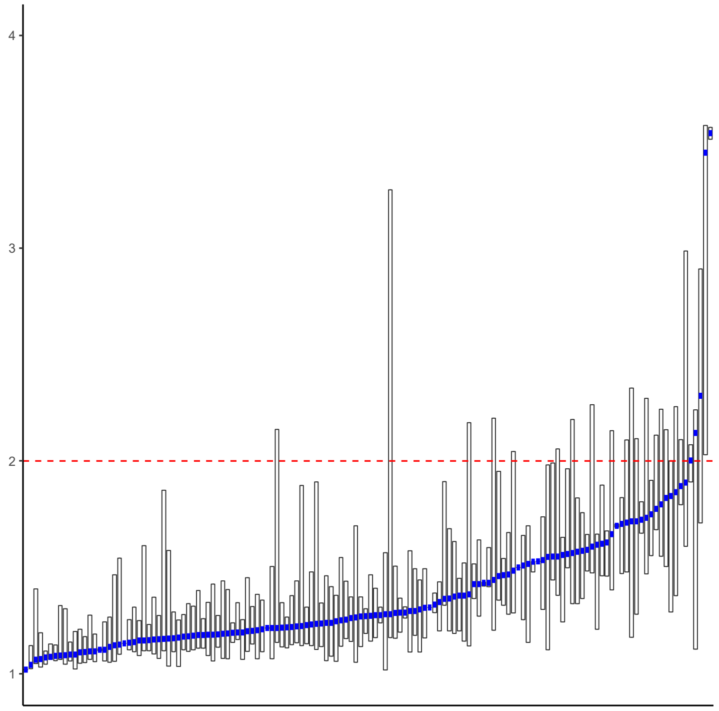

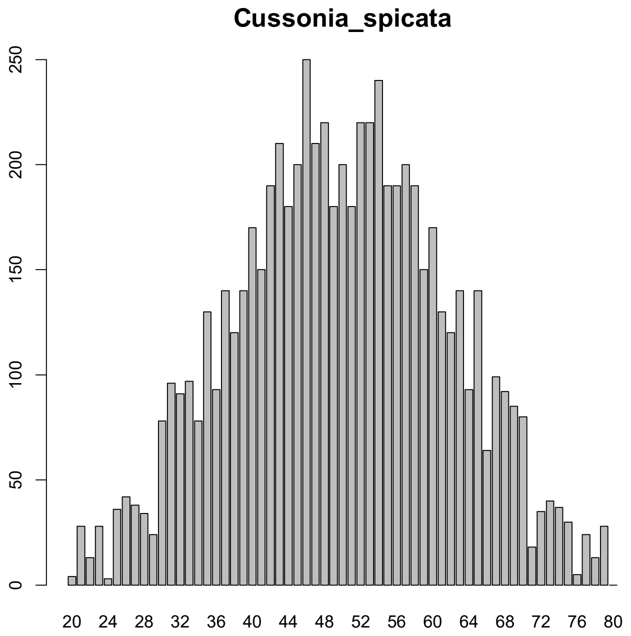
Cussonia_spicata
0
50
100
150
200
250
20 24 28 32 36 40 44 48 52 56 60 64 68 72 76 80

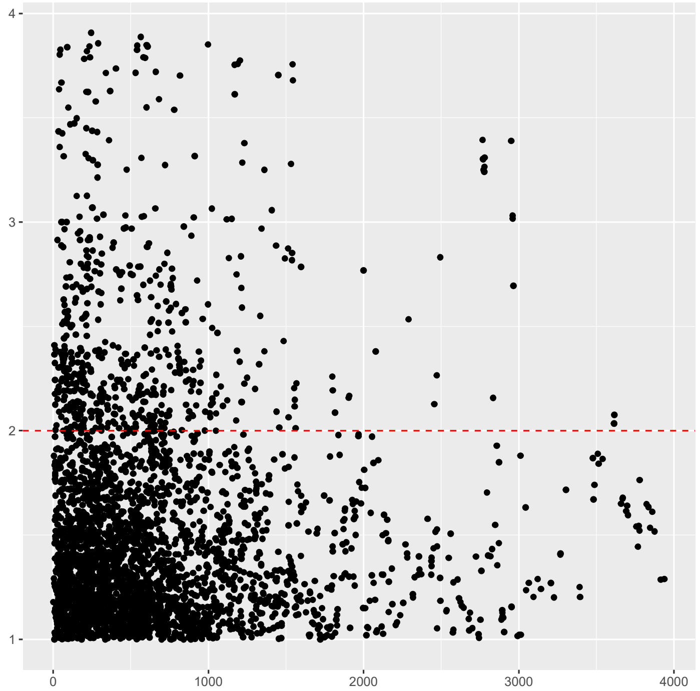
1
2
3
4
0 1000 2000 3000 4000

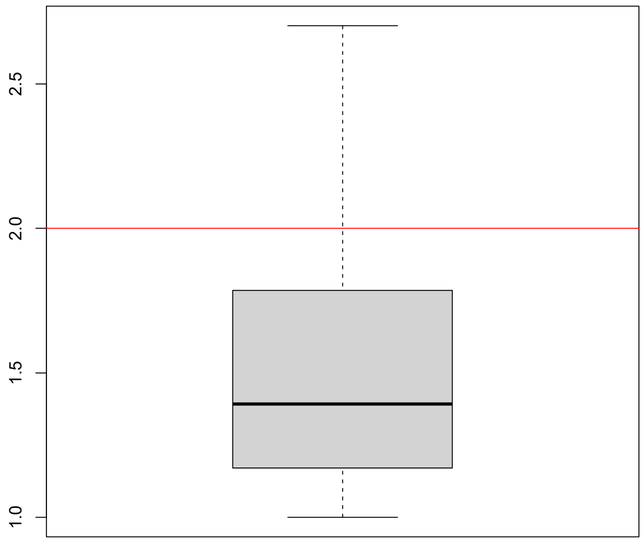
1.0
1.5
2.0
2.5

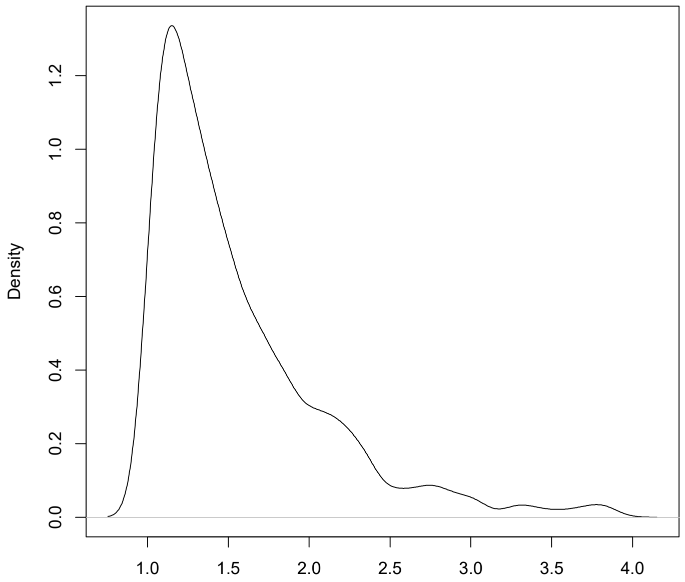
Density
0.0
0.2
0.4
0.6
0.8
1.0
1.2
1.0 1.5 2.0 2.5 3.0 3.5 4.0

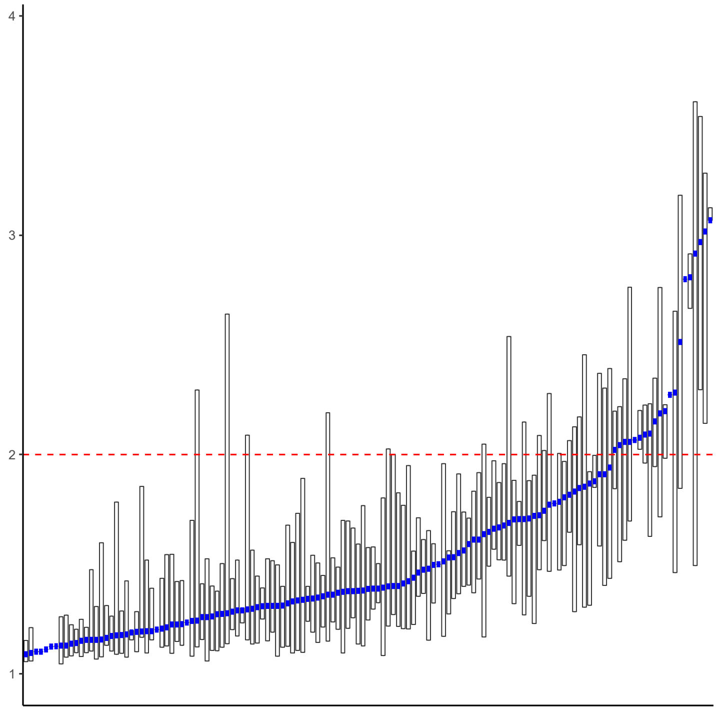
1
2
3
4

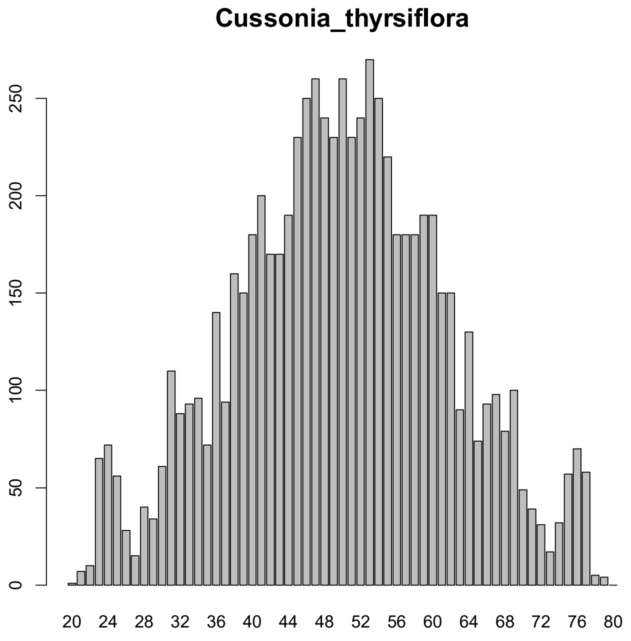
Cussonia_thyrsiflora
0
50
100
150
200
250
20 24 28 32 36 40 44 48 52 56 60 64 68 72 76 80

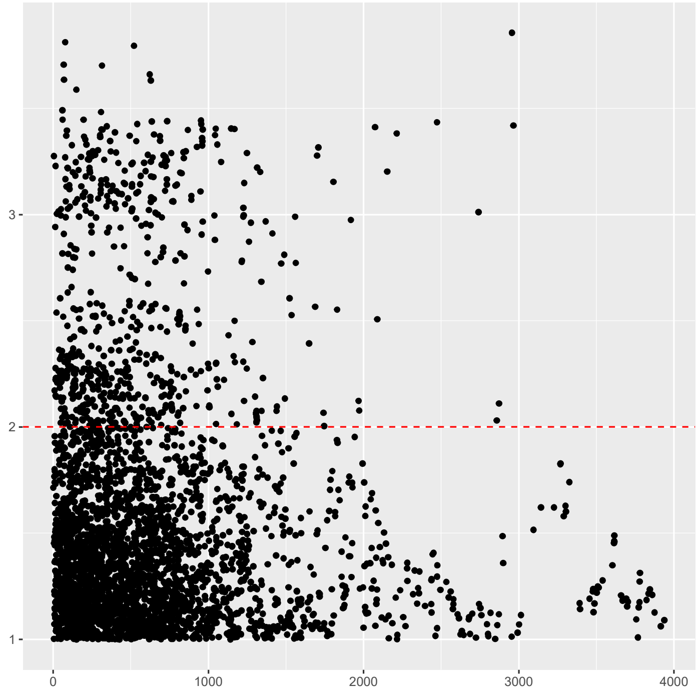
1
2
3
0 1000 2000 3000 4000

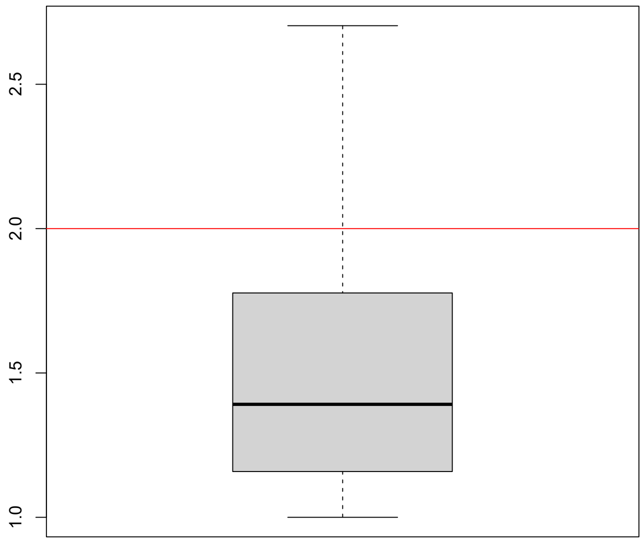
1.0
1.5
2.0
2.5

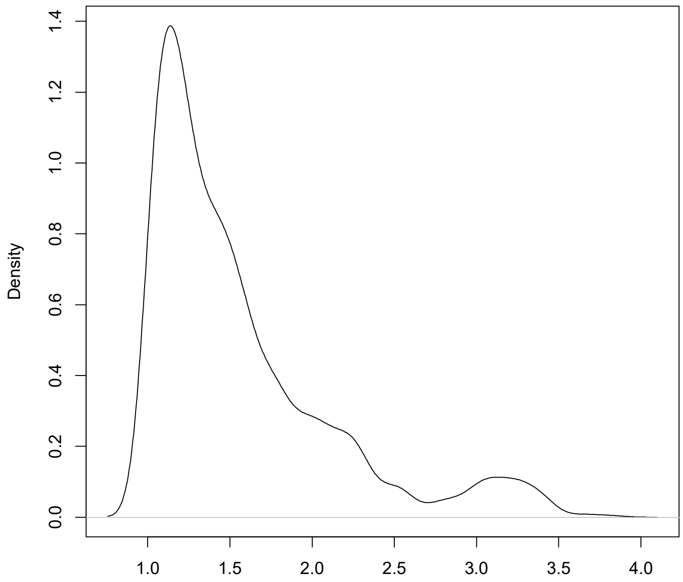
Density
0.0 0.2 0.4 0.6 0.8 1.0 1.2 1.4
1.0 1.5 2.0 2.5 3.0 3.5 4.0

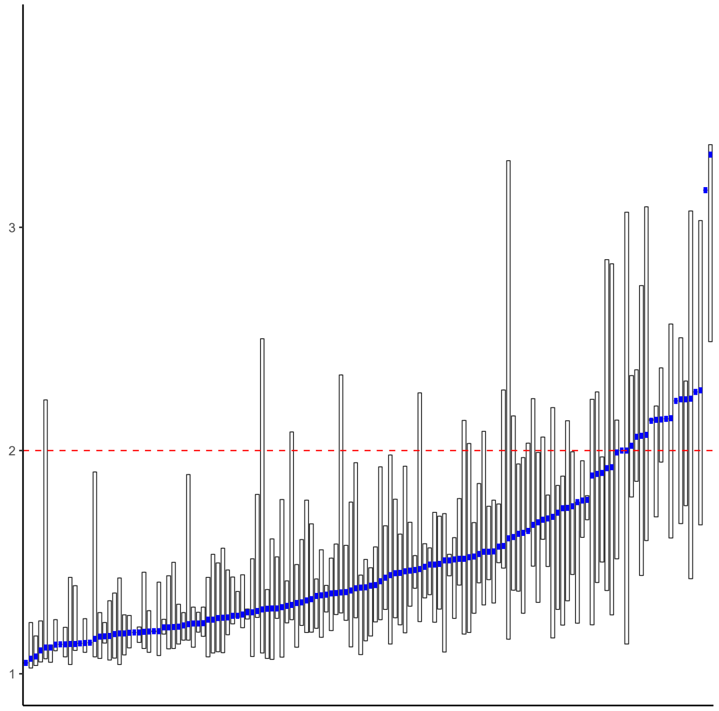
1
2
3

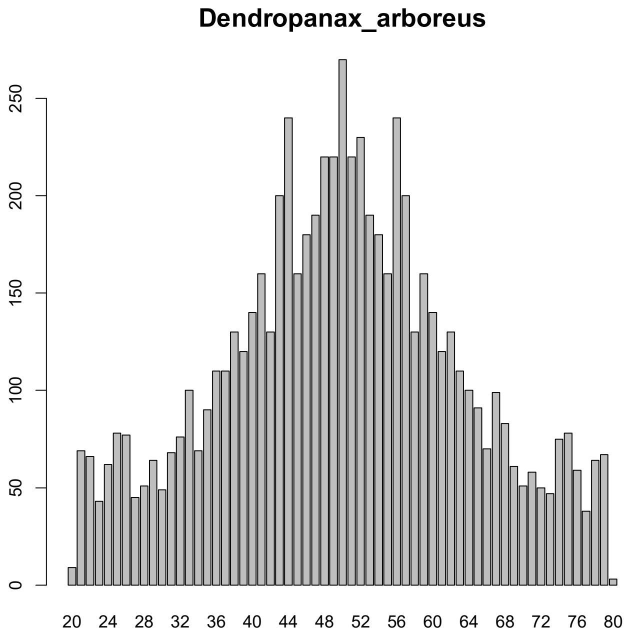
Dendropanax_arboreus
0
50
100
150
200
250
20 24 28 32 36 40 44 48 52 56 60 64 68 72 76 80

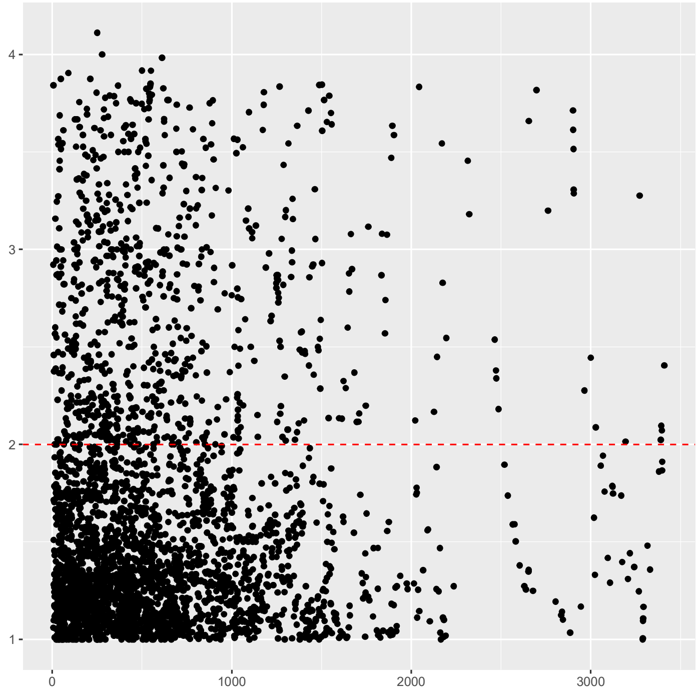
1
2
3
4
0
1000
2000
3000

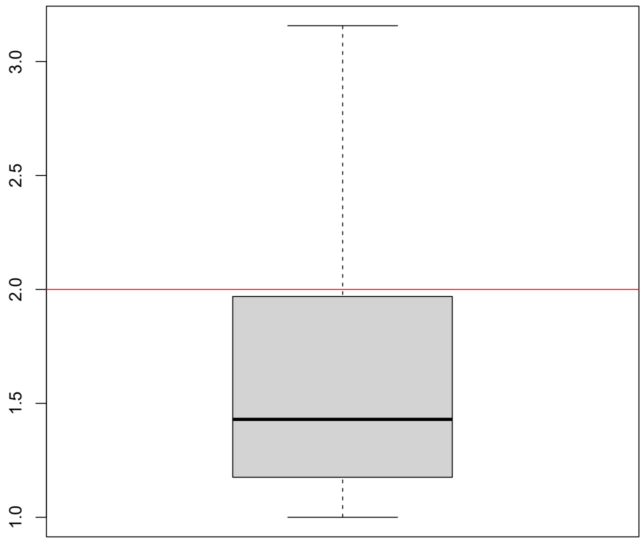
1.0
1.5
2.0
2.5
3.0

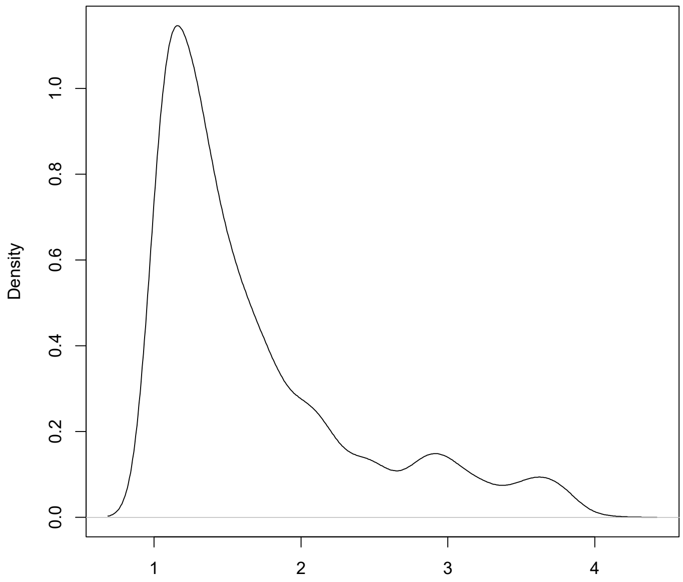
Density
0.0
0.2
0.4
0.6
0.8
1.0
1 2 3 4

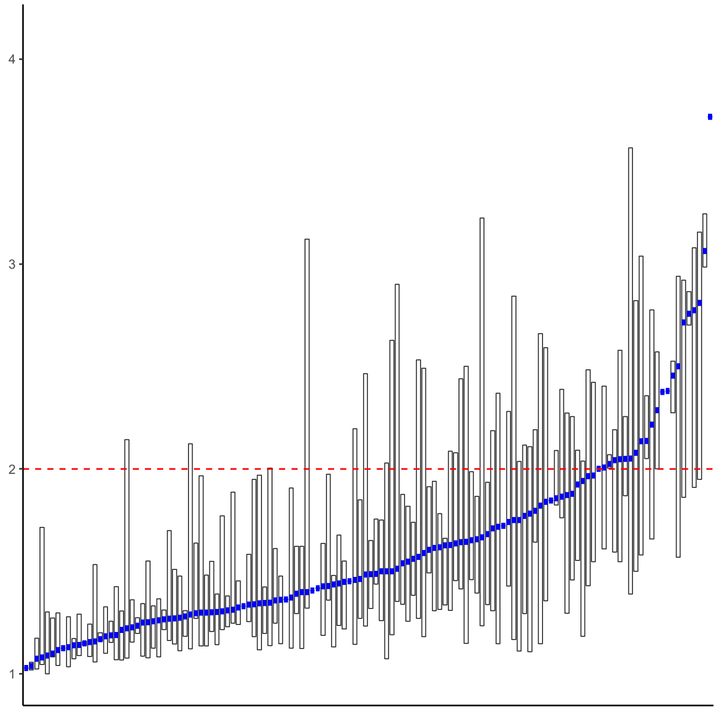
1
2
3
4

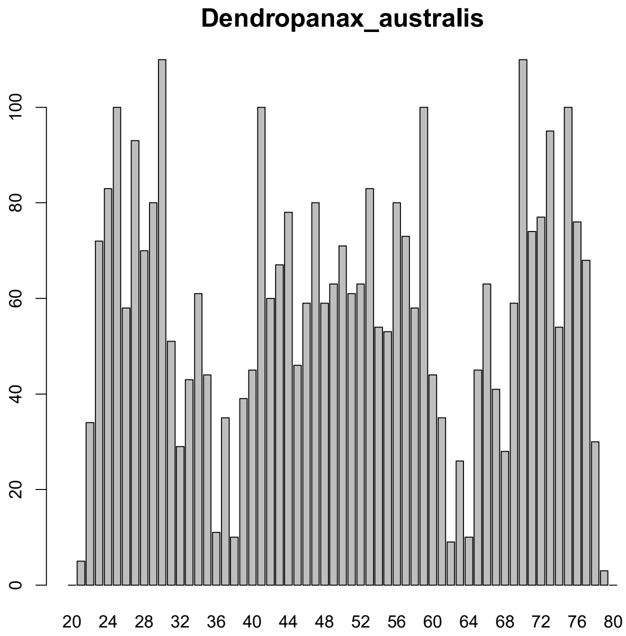
Dendropanax_australis

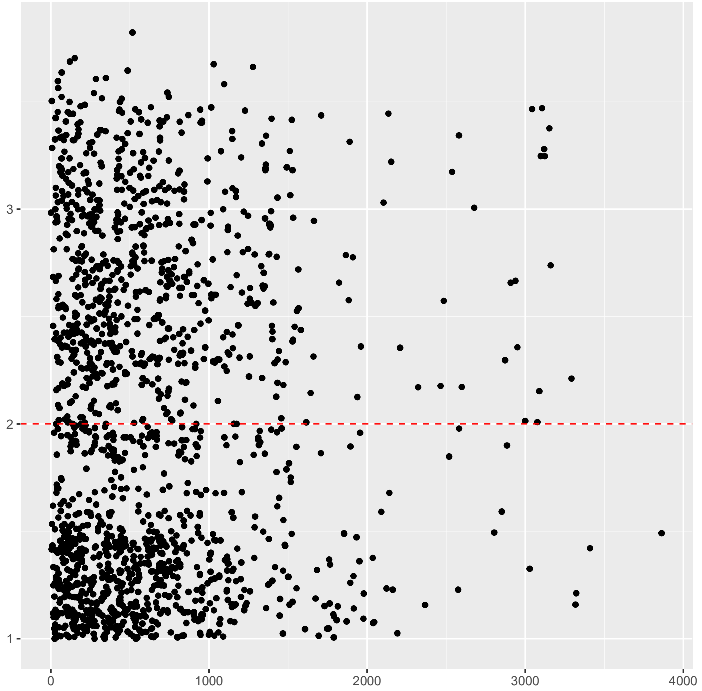

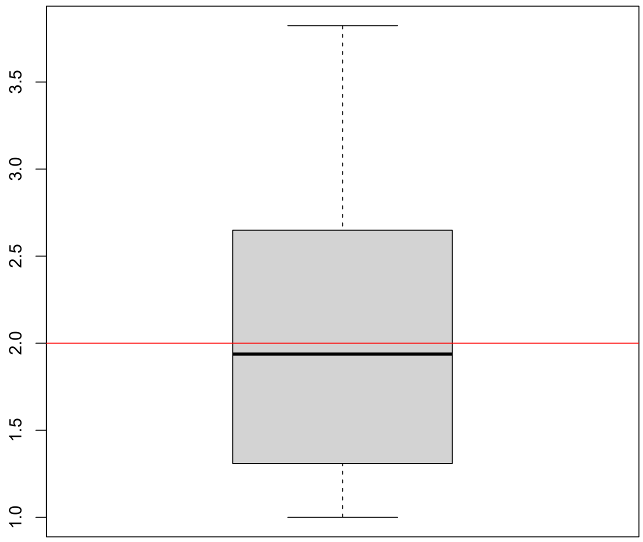

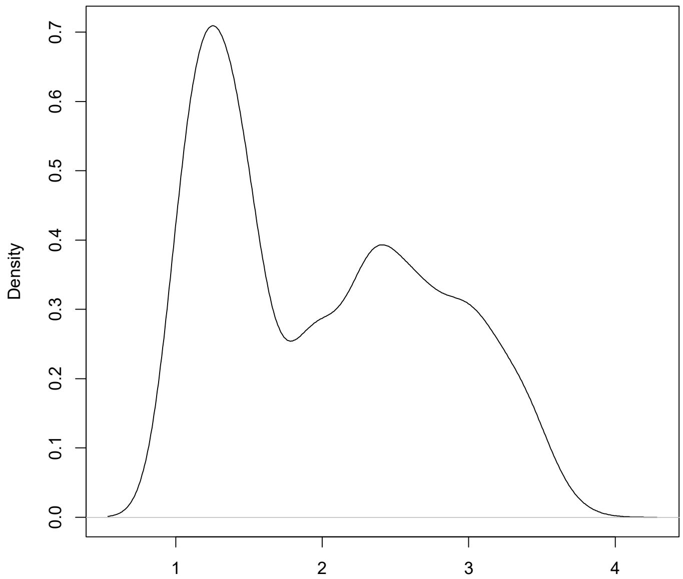
Density

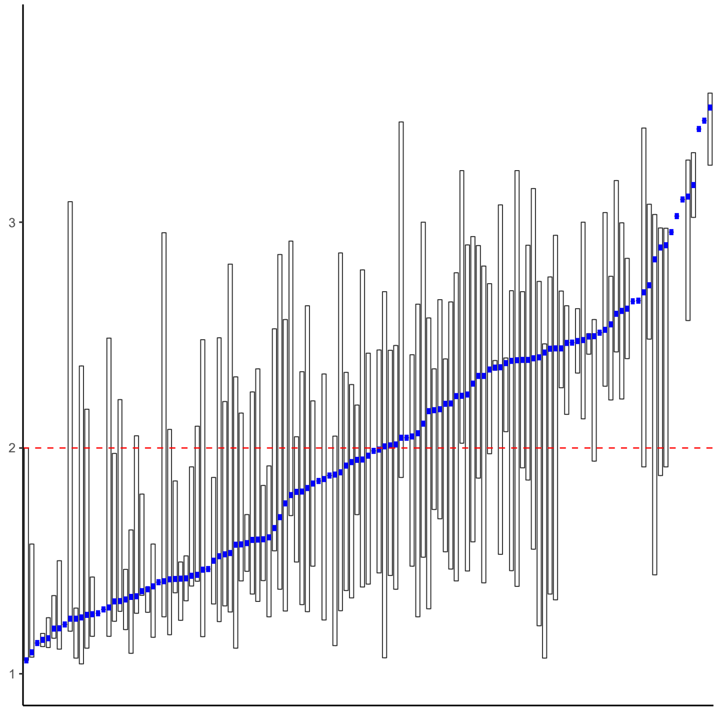

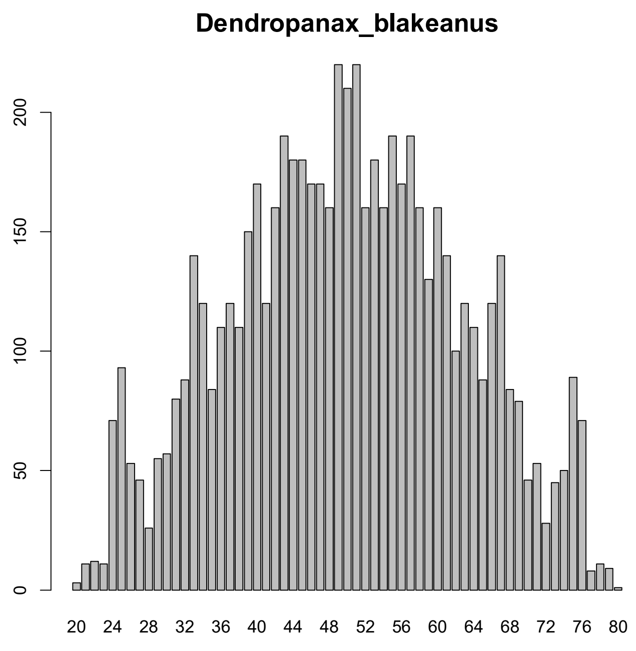
Dendropanax_blakeanus
0
50
100
150
200
20 24 28 32 36 40 44 48 52 56 60 64 68 72 76 80

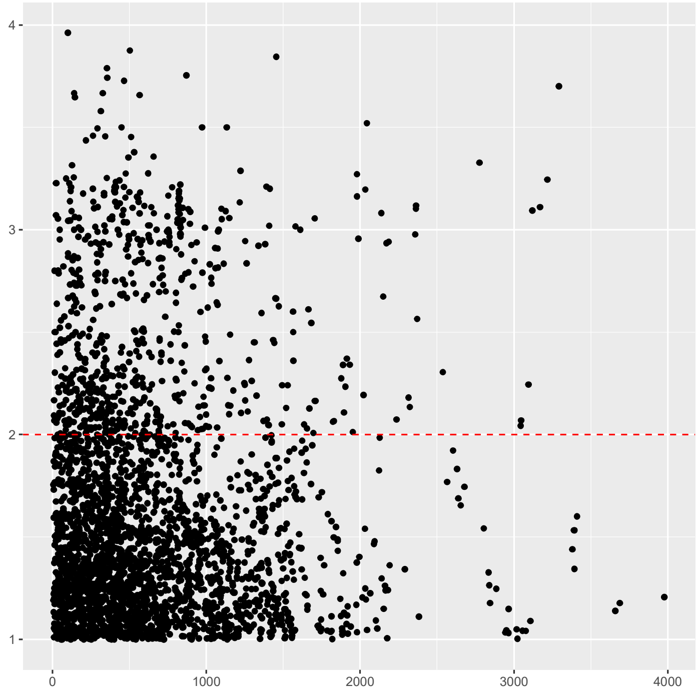
1
2
3
4
0 1000 2000 3000 4000

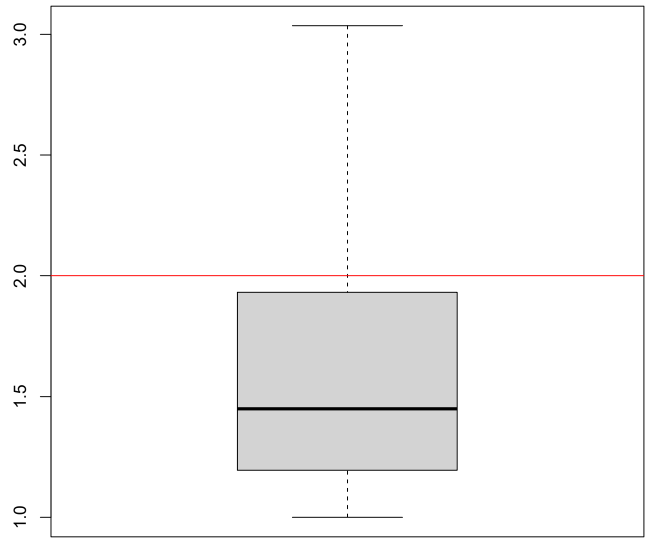
1.0
1.5
2.0
2.5
3.0

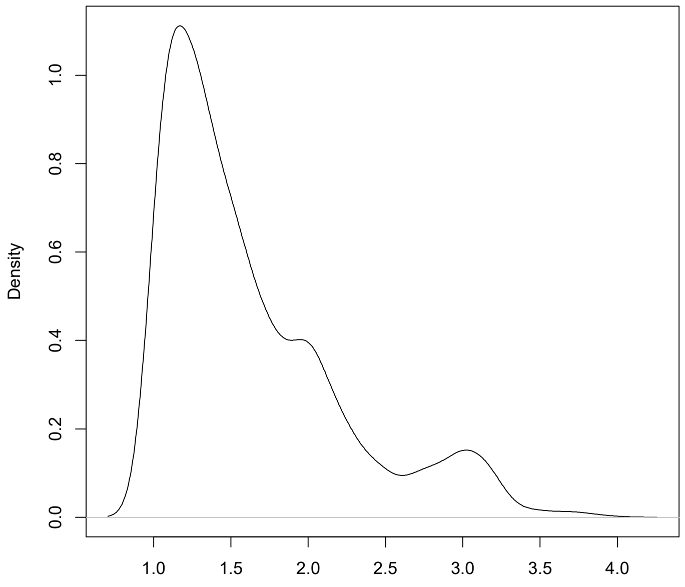
Density
0.0
0.2
0.4
0.6
0.8
1.0
1.0 1.5 2.0 2.5 3.0 3.5 4.0

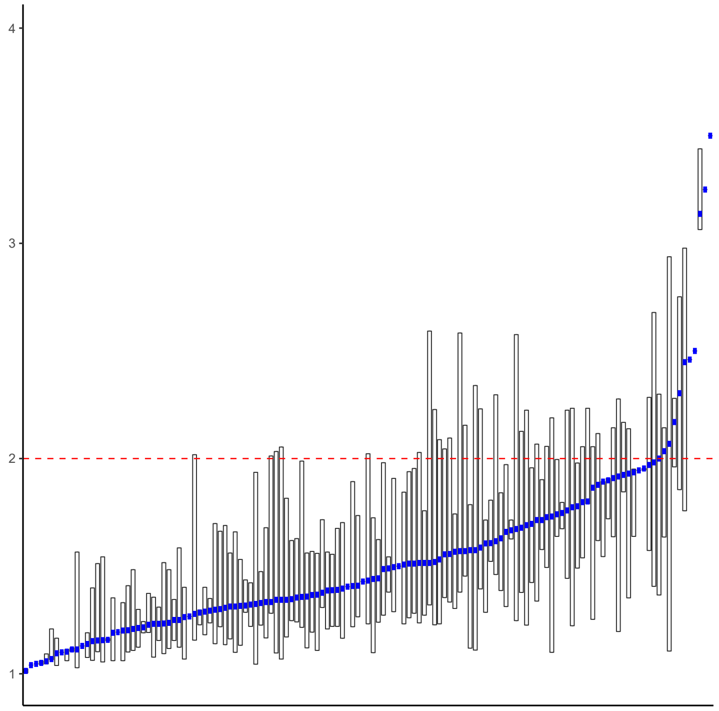
1
2
3
4

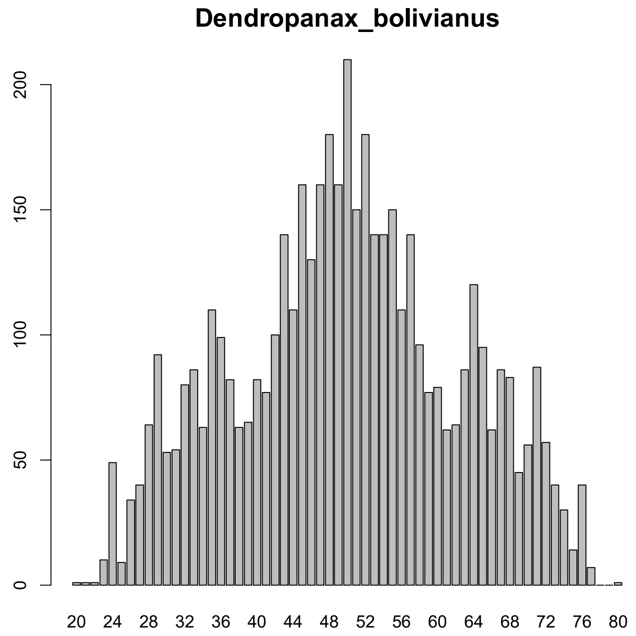
Dendropanax_bolivianus
0
50
100
150
200
20 24 28 32 36 40 44 48 52 56 60 64 68 72 76 80

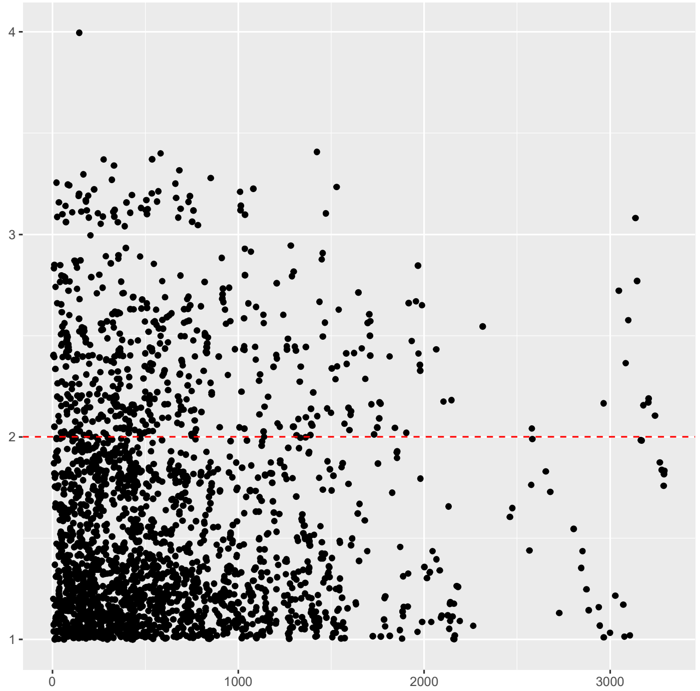
1
2
3
4
0
1000
2000
3000

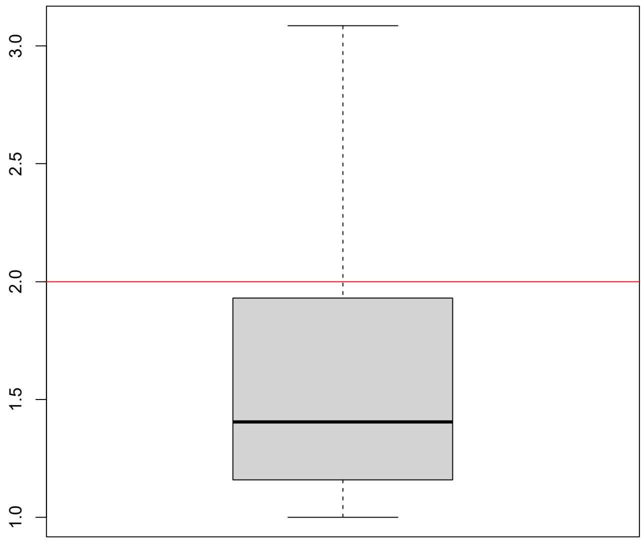
1.0
1.5
2.0
2.5
3.0

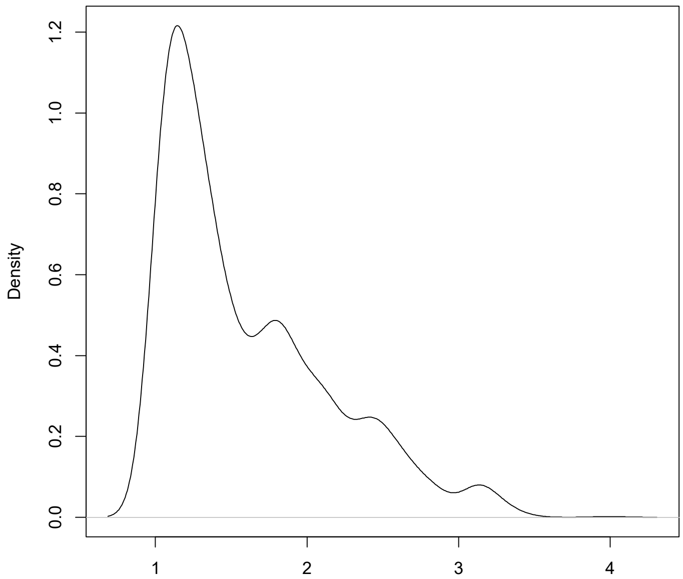
Density
0.0
0.2
0.4
0.6
0.8
1.0
1.2
1 2 3 4

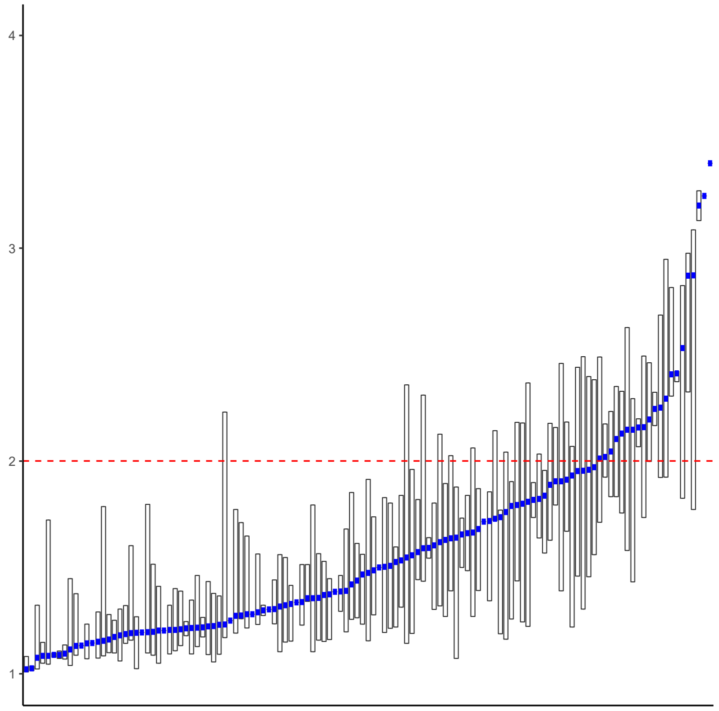
1
2
3
4

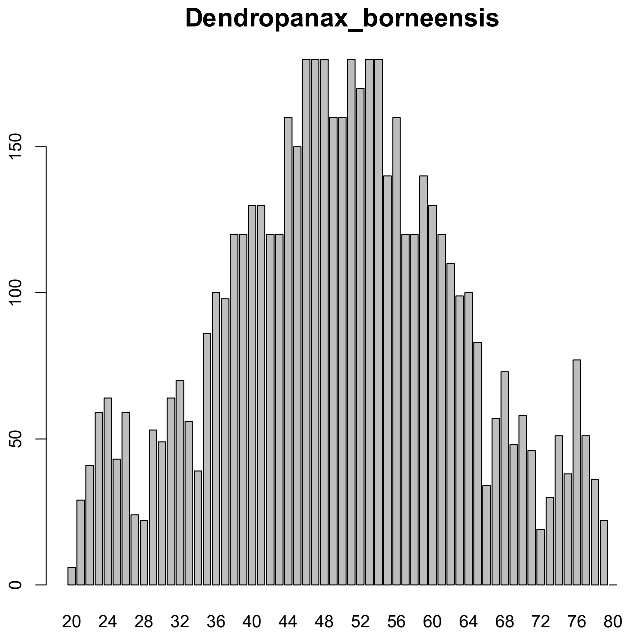
Dendropanax_borneensis
0
50
100
150
20 24 28 32 36 40 44 48 52 56 60 64 68 72 76 80

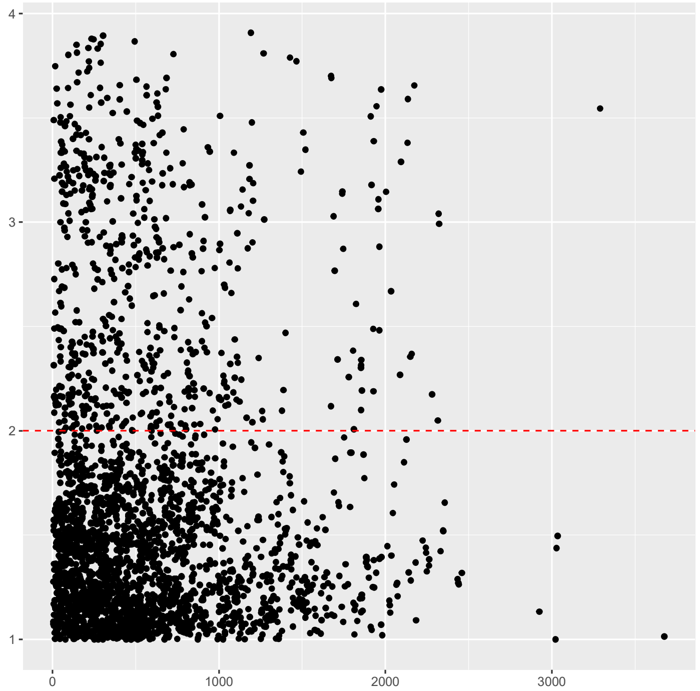
1
2
3
4
0 1000 2000 3000

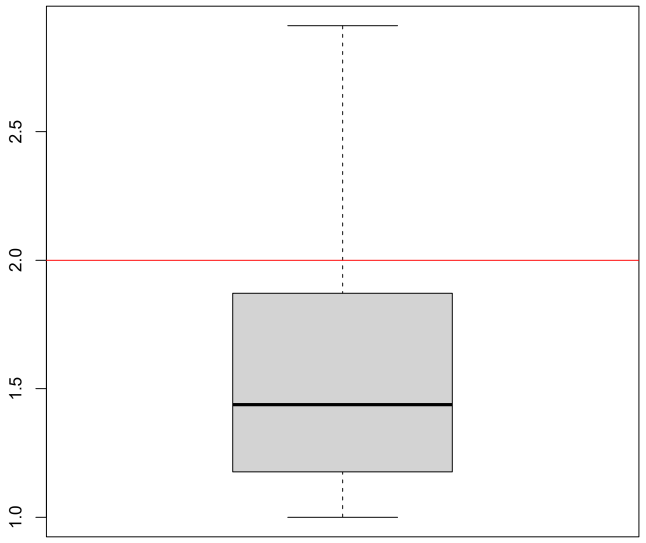
1.0
1.5
2.0
2.5

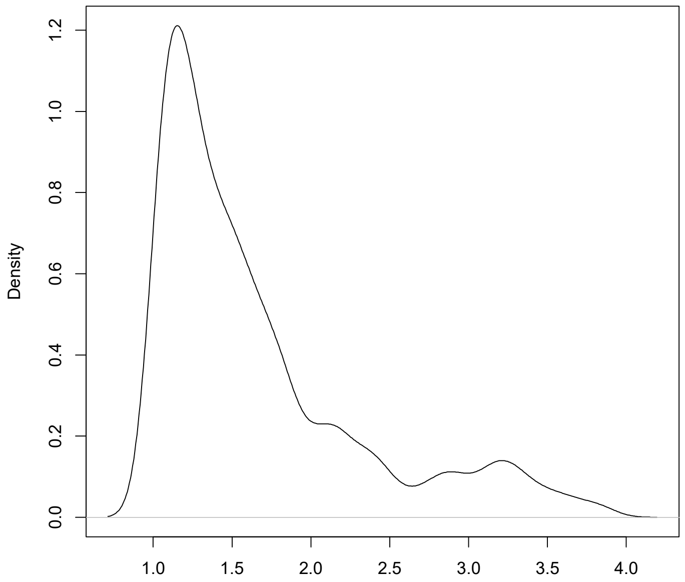
Density
0.0 0.2 0.4 0.6 0.8 1.0 1.2
1.0 1.5 2.0 2.5 3.0 3.5 4.0

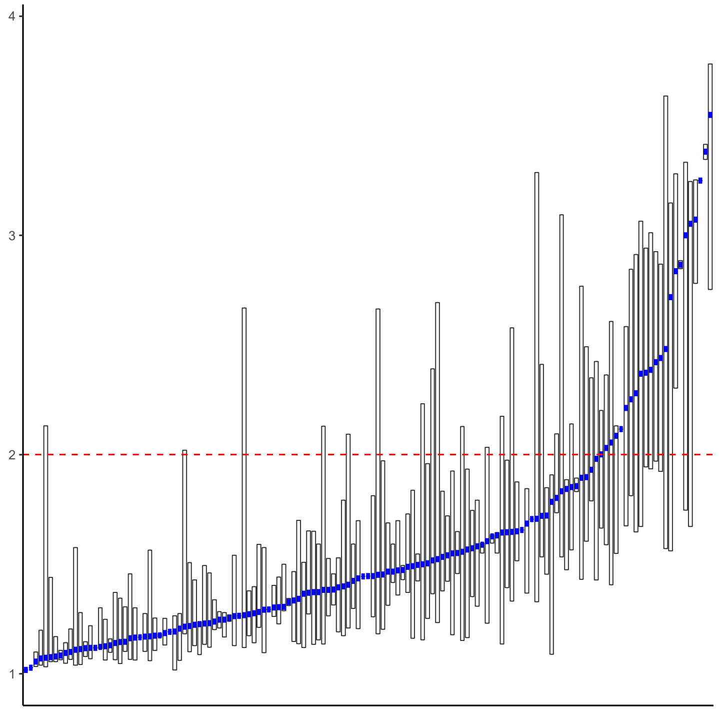
1
2
3
4

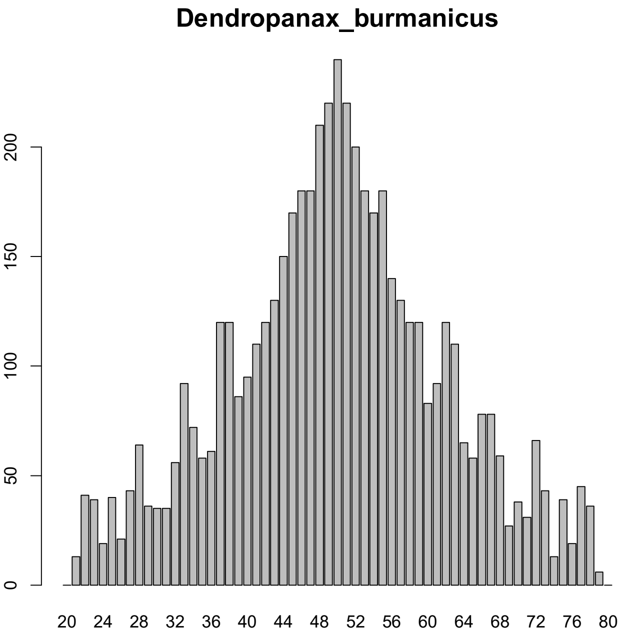
Dendropanax_burmanicus
0
50
100
150
200
20 24 28 32 36 40 44 48 52 56 60 64 68 72 76 80

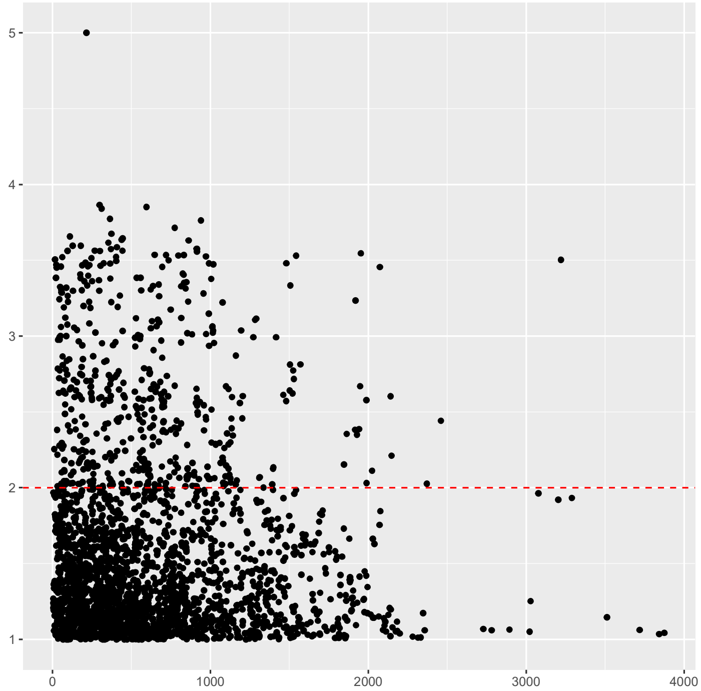
1
2
3
4
5
0 1000 2000 3000 4000

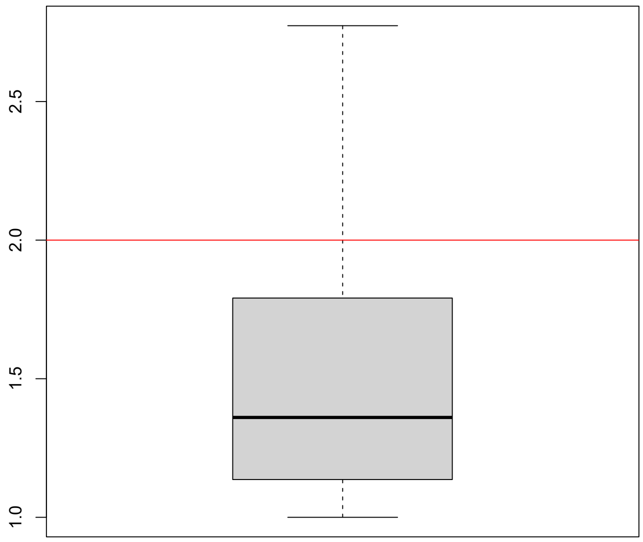
1.0
1.5
2.0
2.5

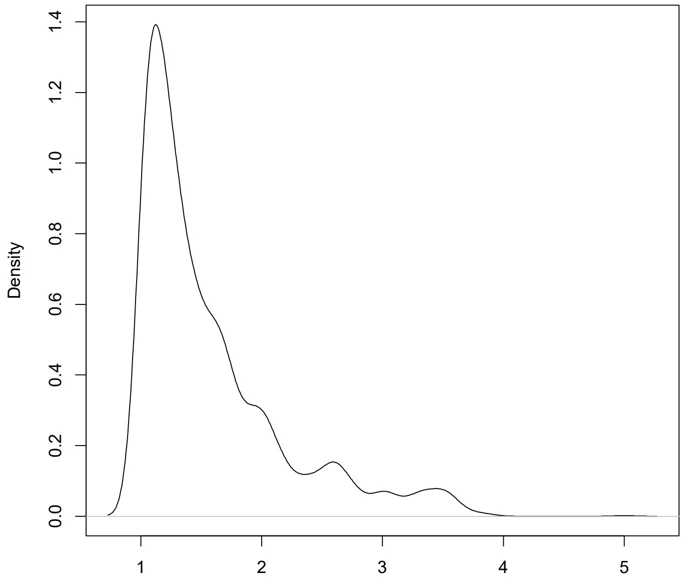
Density
0.0 0.2 0.4 0.6 0.8 1.0 1.2 1.4
1 2 3 4 5

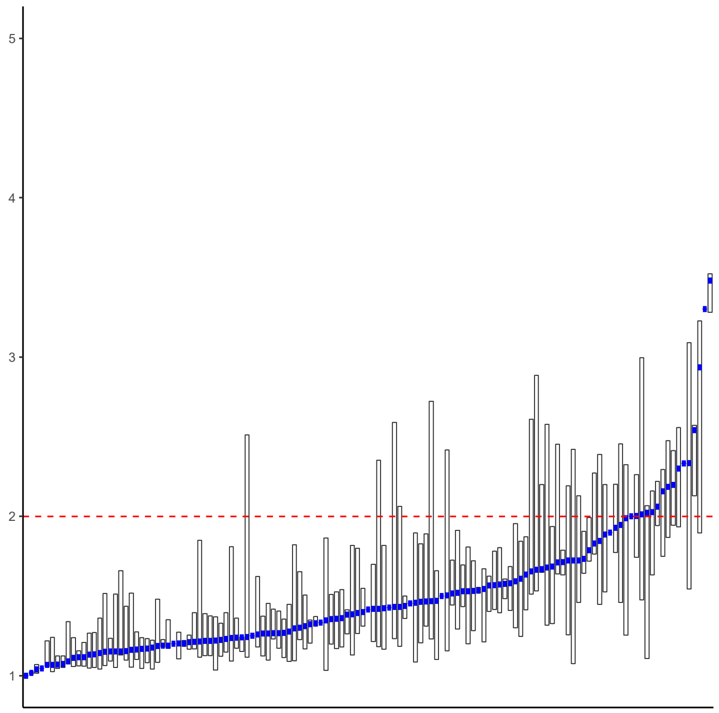
1
2
3
4
5

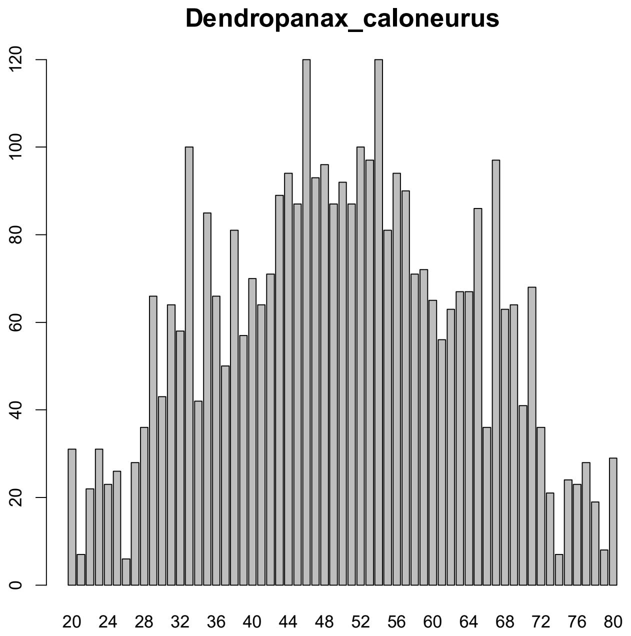
Dendropanax_caloneurus

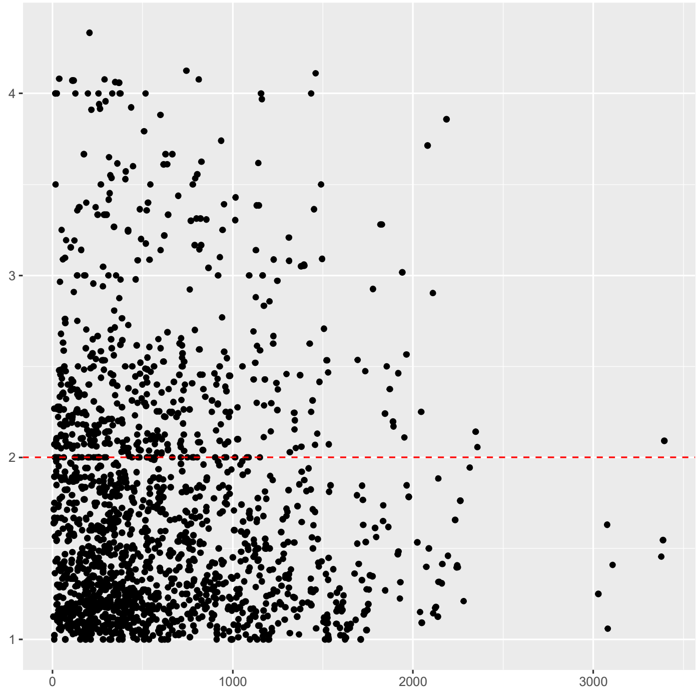

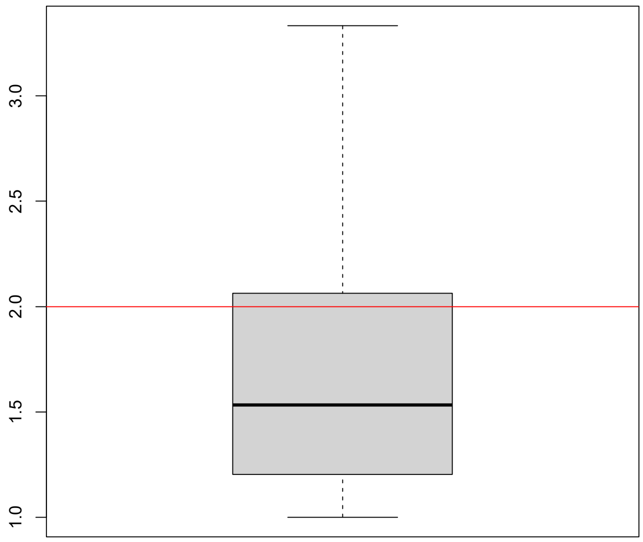

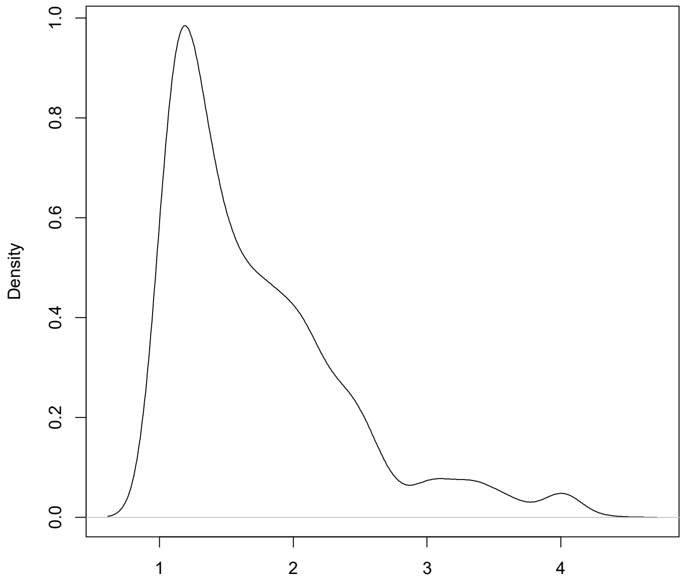
Density

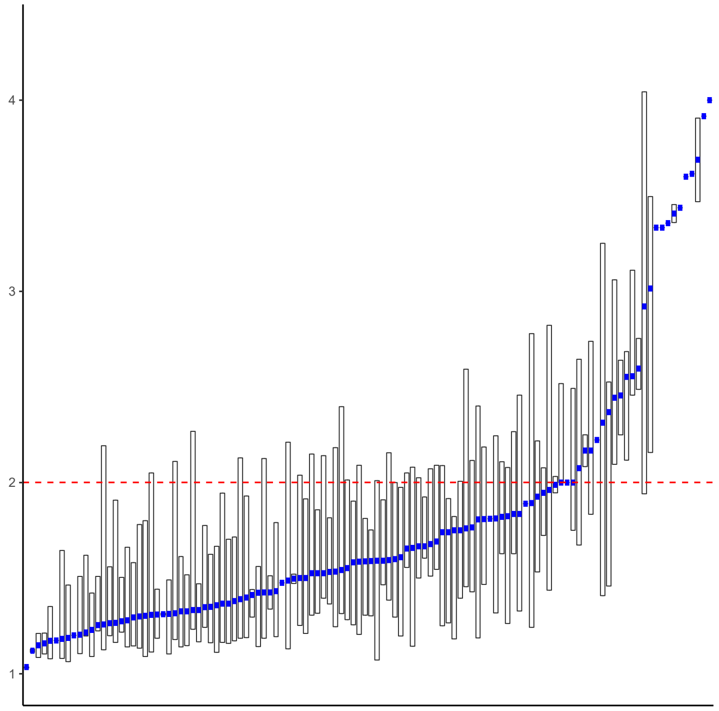

Dendropanax_caucanus

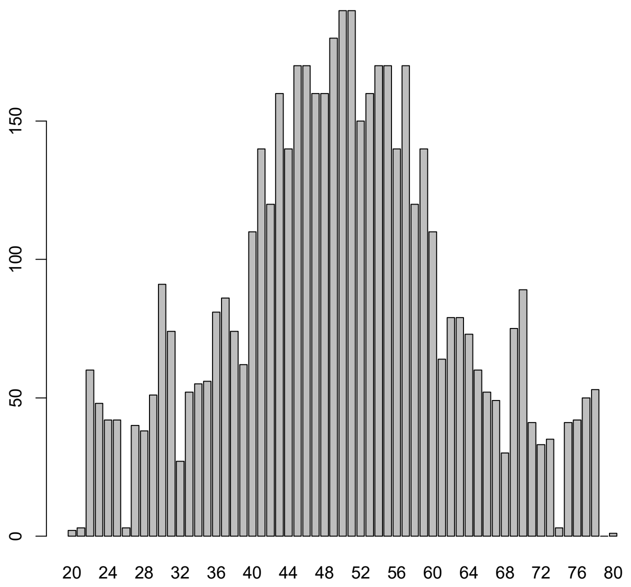

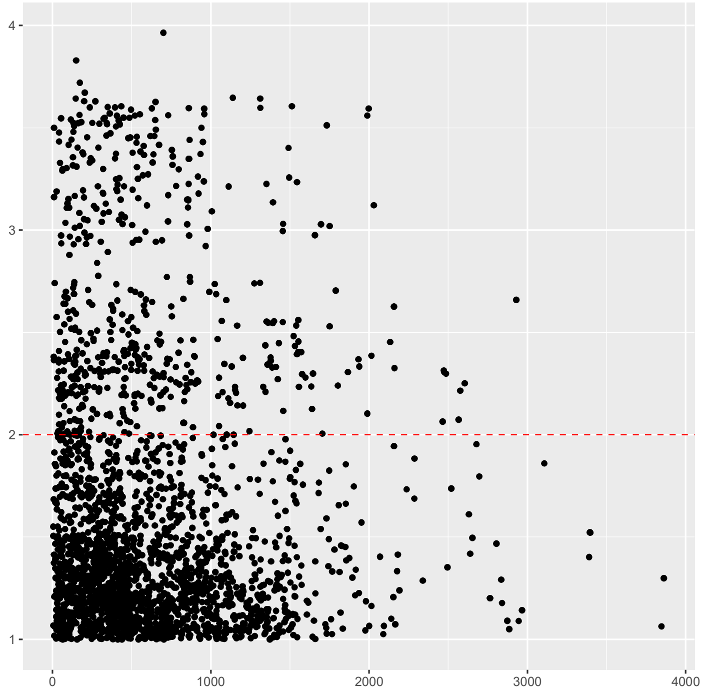

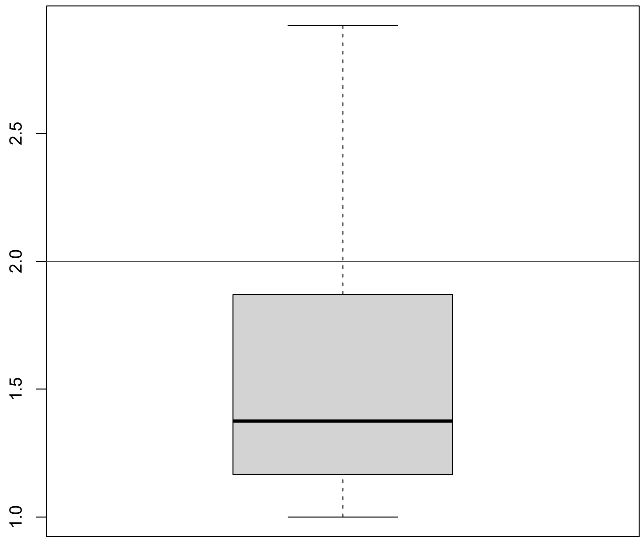

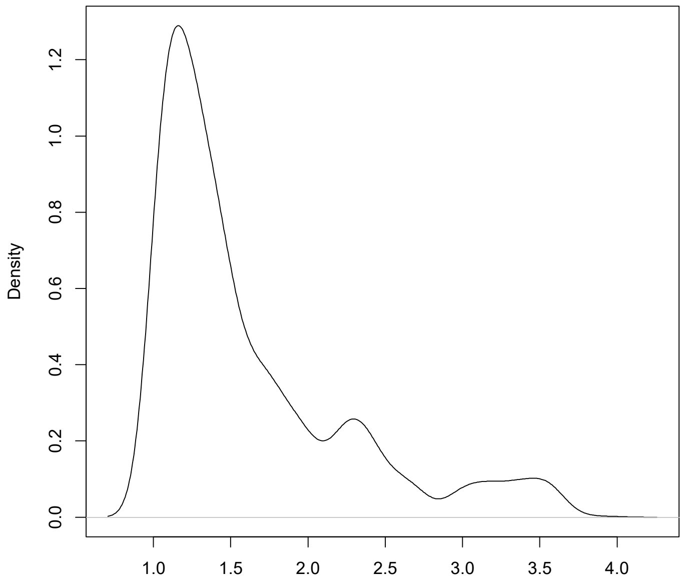

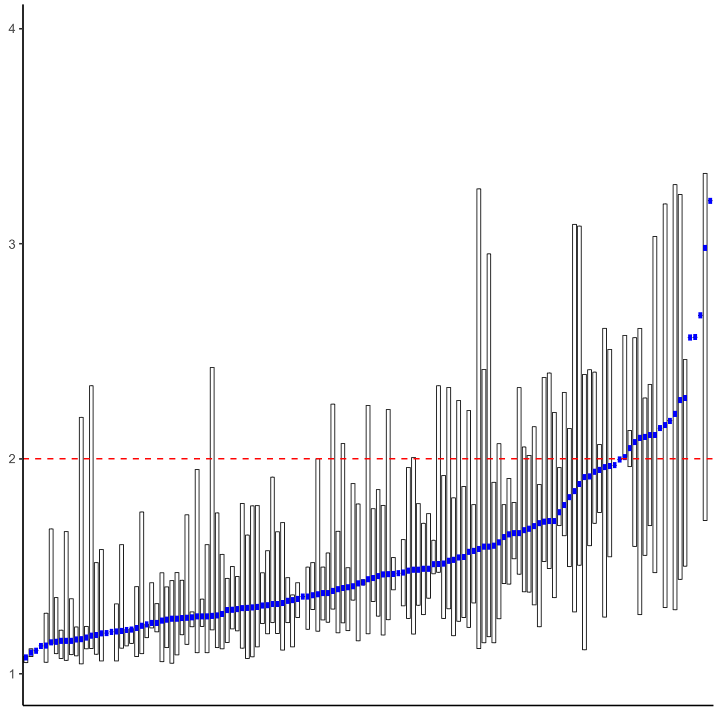

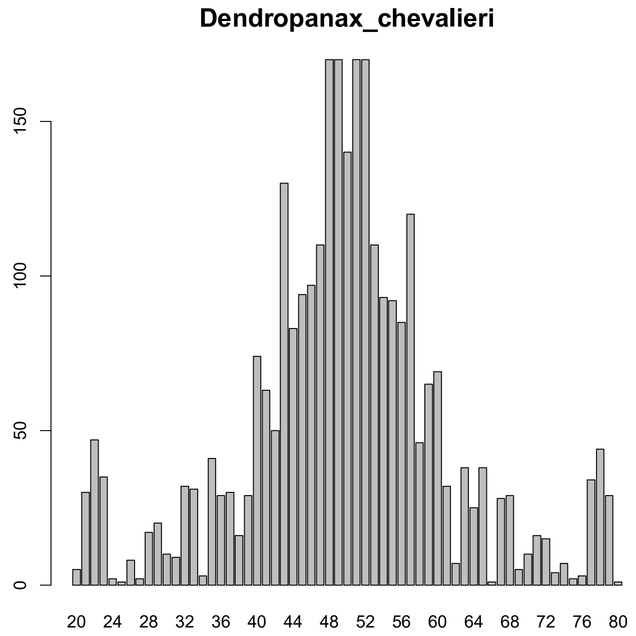
Dendropanax_chevalieri
0
50
100
150
20 24 28 32 36 40 44 48 52 56 60 64 68 72 76 80

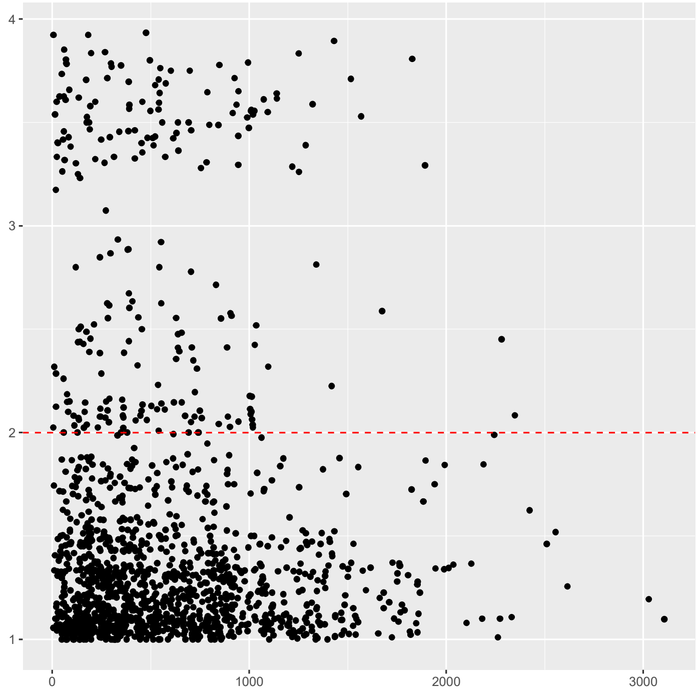
1
2
3
4
0
1000
2000
3000

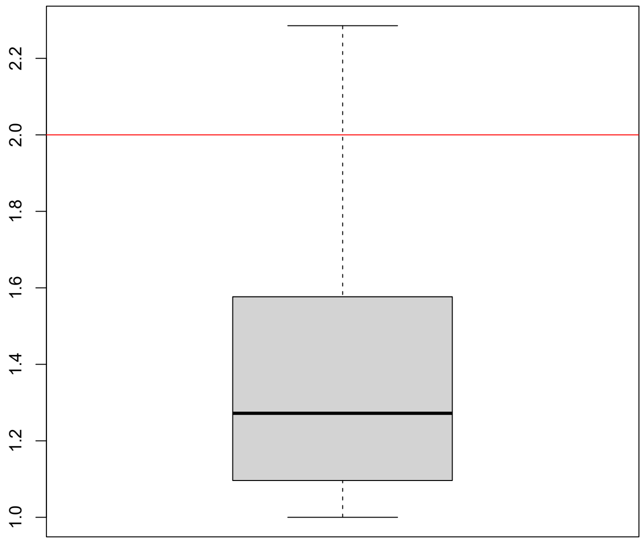
1.0
1.2
1.4
1.6
1.8
2.0
2.2

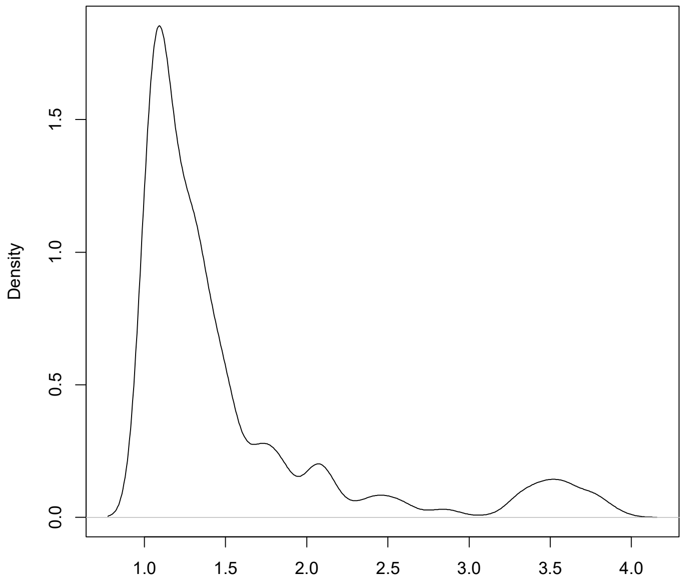
Density
0.0
0.5
1.0
1.5
1.0 1.5 2.0 2.5 3.0 3.5 4.0

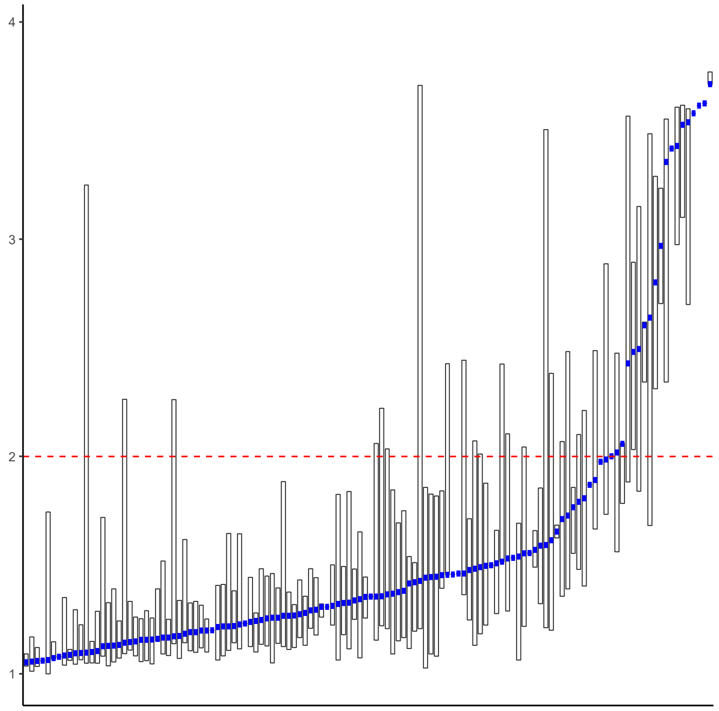
1
2
3
4

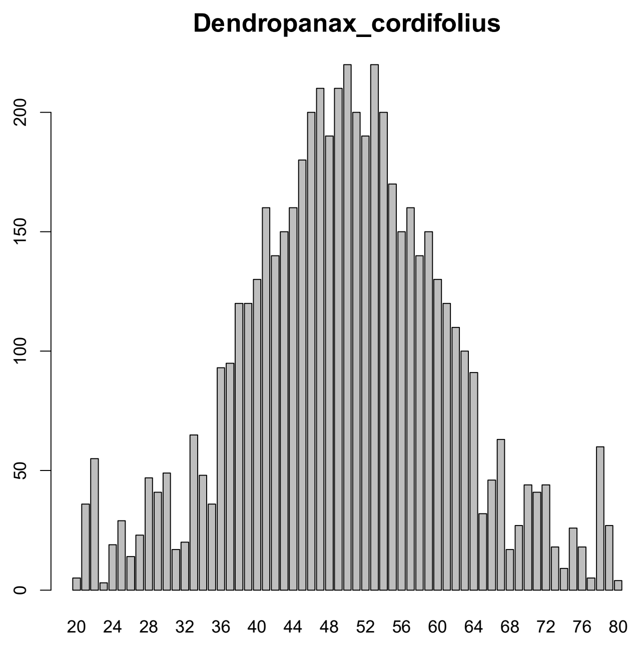
Dendropanax_cordifolius
0
50
100
150
200
20 24 28 32 36 40 44 48 52 56 60 64 68 72 76 80

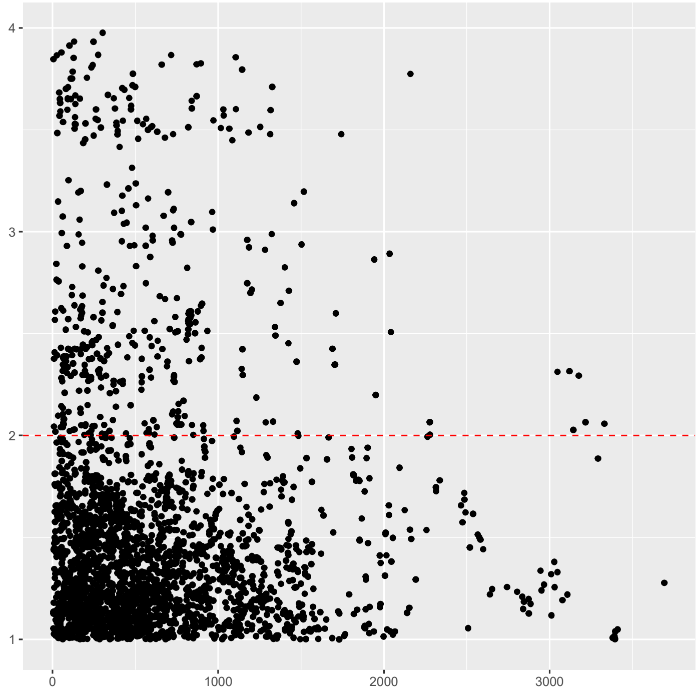
1
2
3
4
0 1000 2000 3000

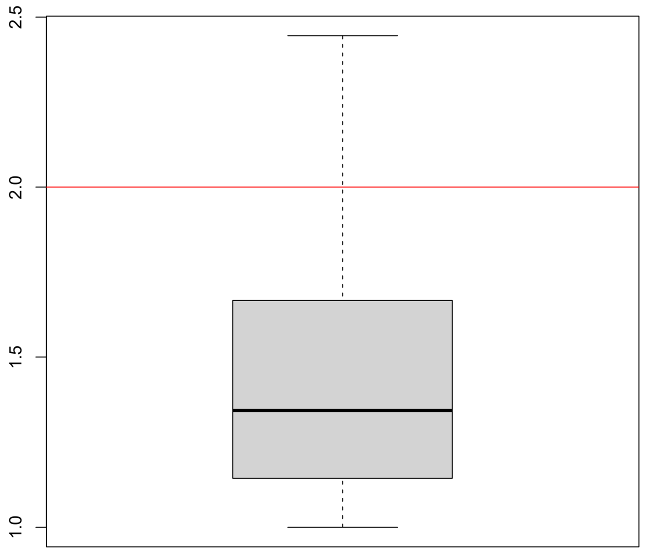
1.0
1.5
2.0
2.5

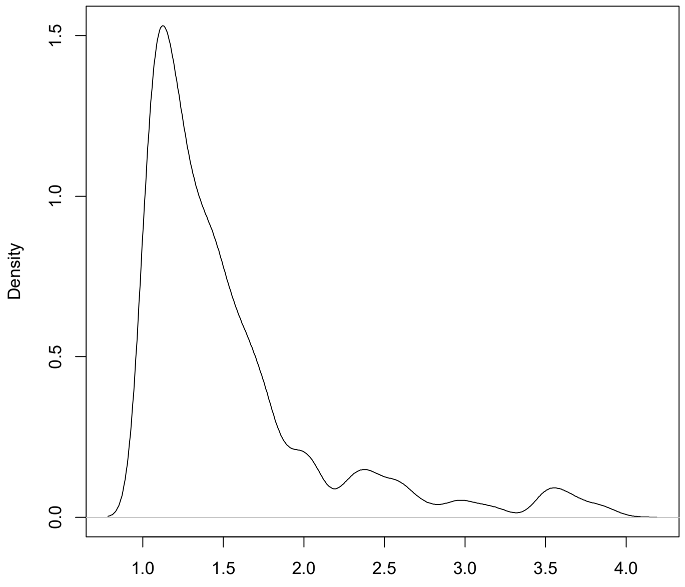
Density
0.0
0.5
1.0
1.5
1.0 1.5 2.0 2.5 3.0 3.5 4.0

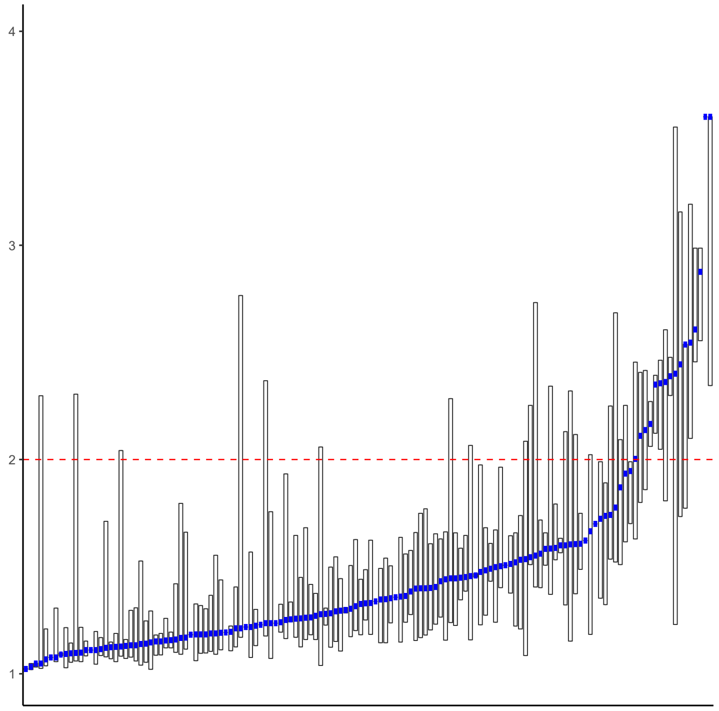
1
2
3
4

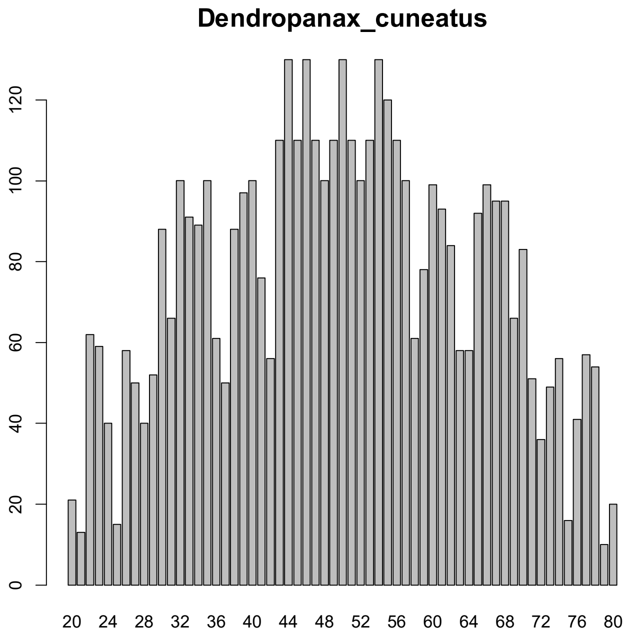
Dendropanax_cuneatus
0
20
40
60
80
100
120
20 24 28 32 36 40 44 48 52 56 60 64 68 72 76 80

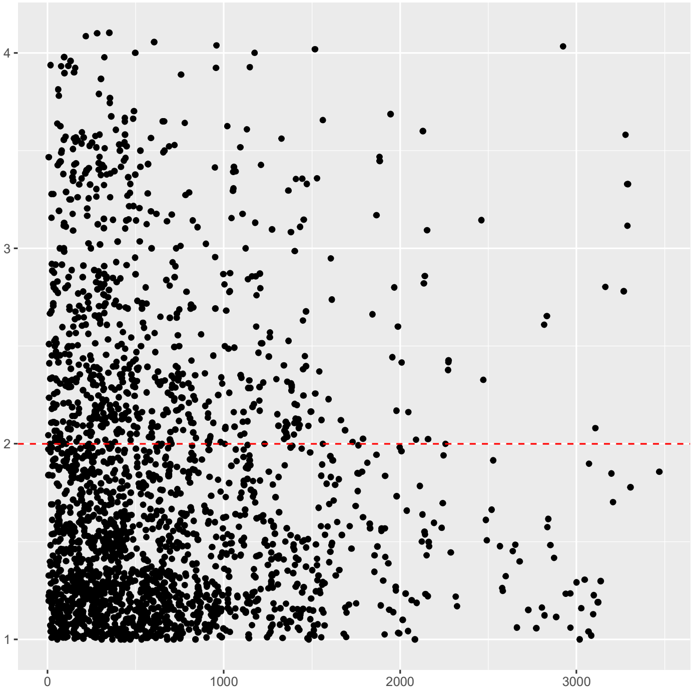
1
2
3
4
0 1000 2000 3000

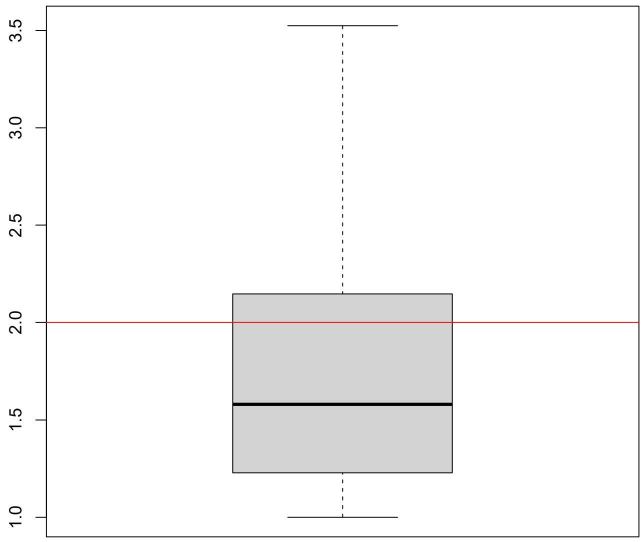
1.0
1.5
2.0
2.5
3.0
3.5

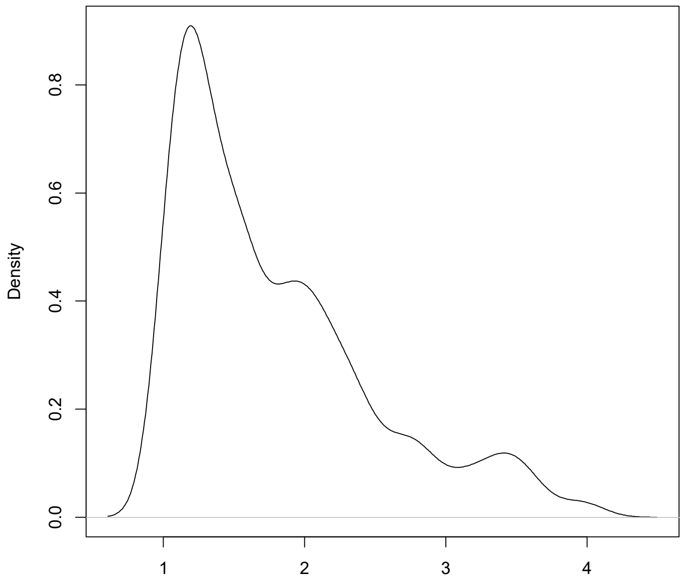
Density
0.0
0.2
0.4
0.6
0.8
1 2 3 4

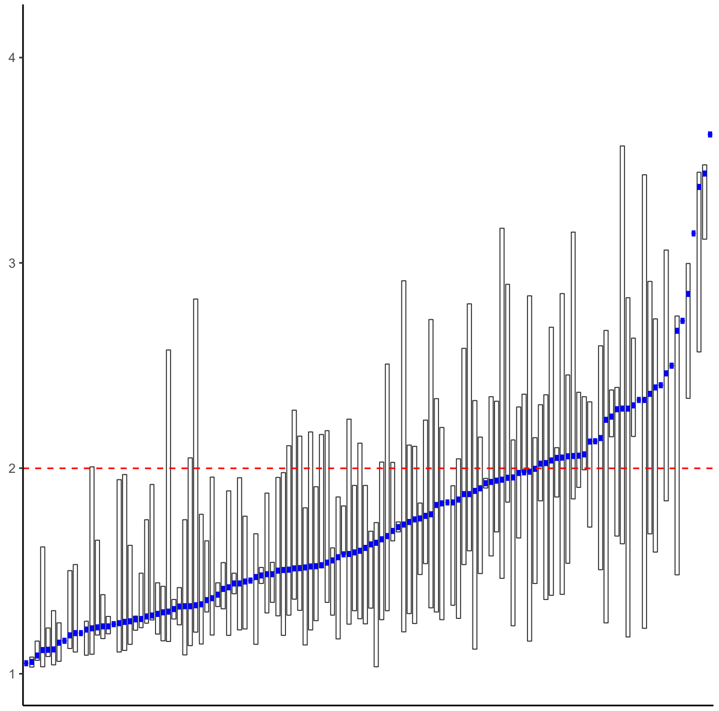
1
2
3
4

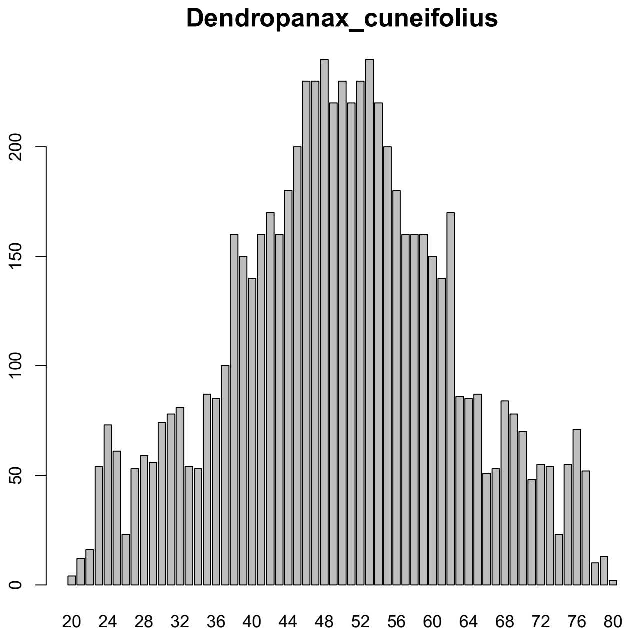
Dendropanax_cuneifolius
0
50
100
150
200
20 24 28 32 36 40 44 48 52 56 60 64 68 72 76 80

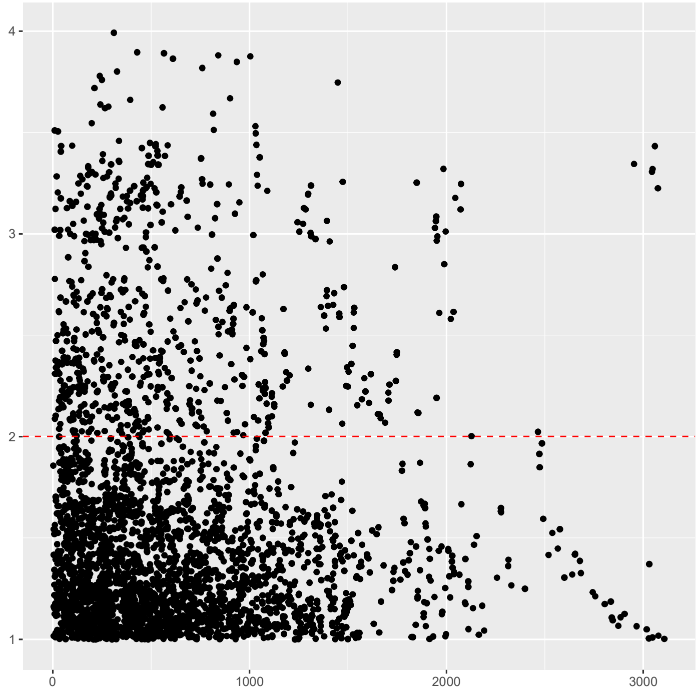
1
2
3
4
0 1000 2000 3000

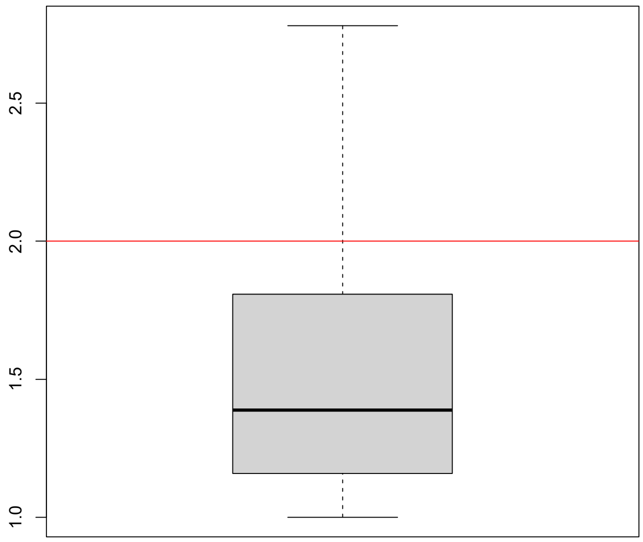
1.0
1.5
2.0
2.5

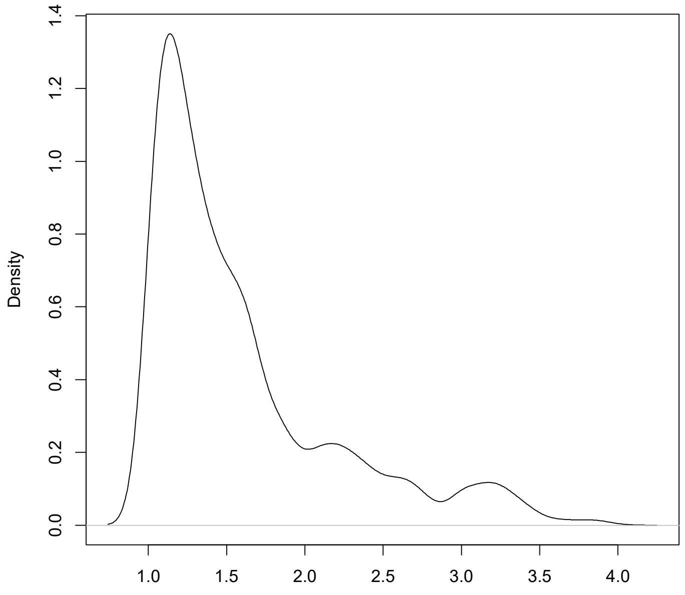
Density
0.0
0.2
0.4
0.6
0.8
1.0
1.2
1.4
1.0 1.5 2.0 2.5 3.0 3.5 4.0

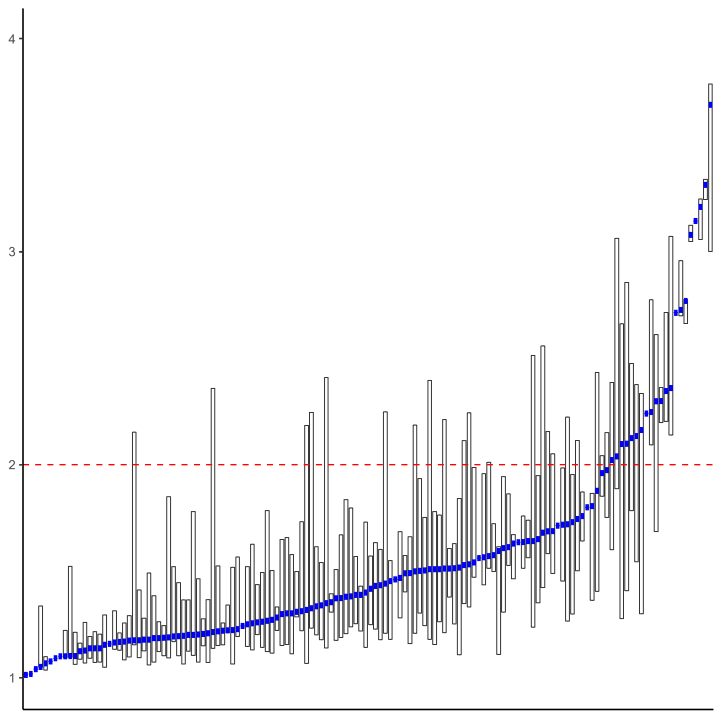
1
2
3
4

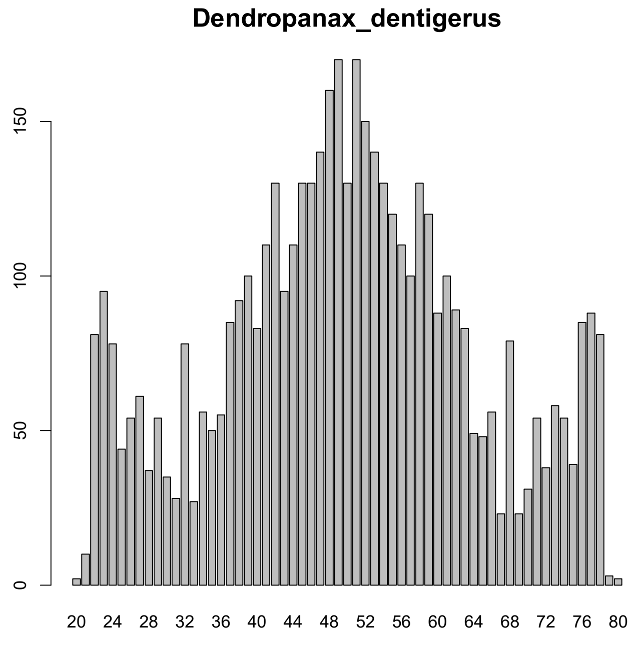
Dendropanax_dentigerus
0
50
100
150
20 24 28 32 36 40 44 48 52 56 60 64 68 72 76 80

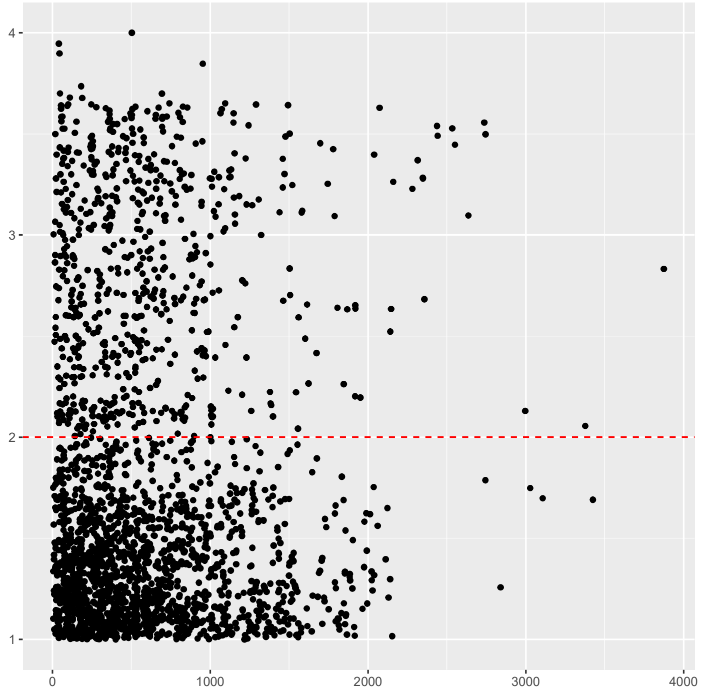
1
2
3
4
0 1000 2000 3000 4000

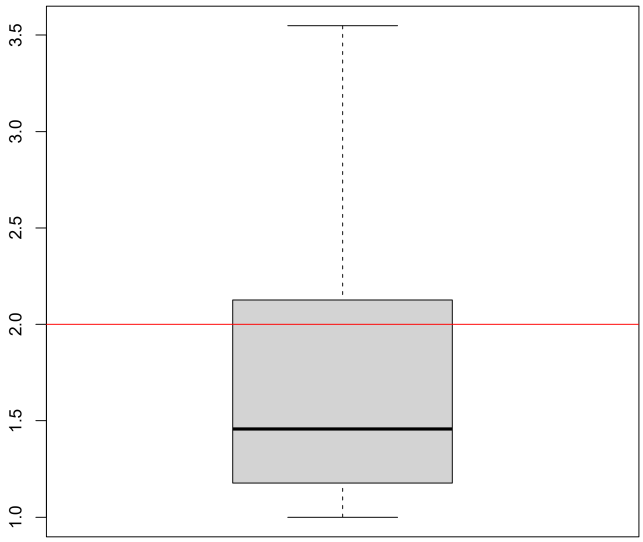
1.0
1.5
2.0
2.5
3.0
3.5

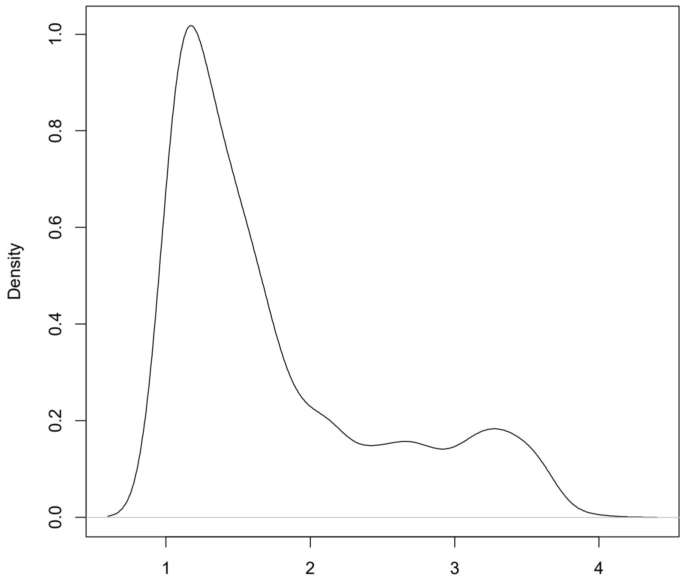
Density
0.0
0.2
0.4
0.6
0.8
1.0
1 2 3 4

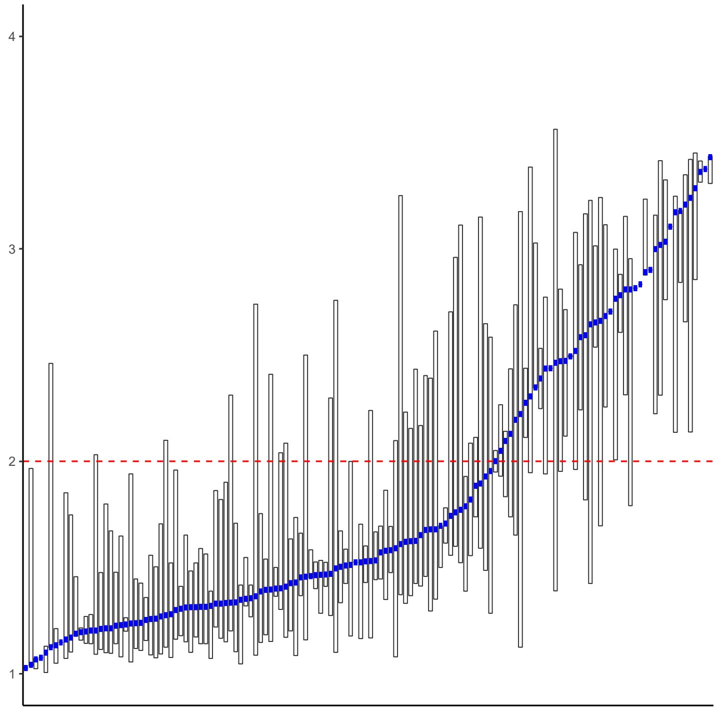
1
2
3
4

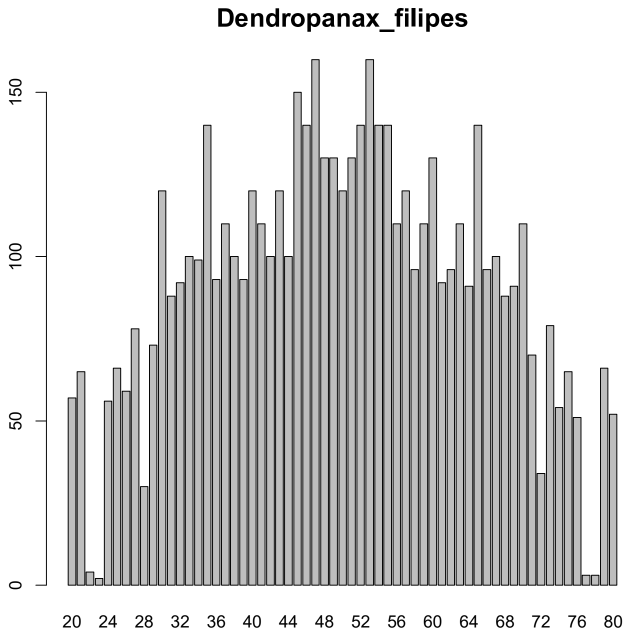
Dendropanax_filipes
0
50
100
150
20 24 28 32 36 40 44 48 52 56 60 64 68 72 76 80

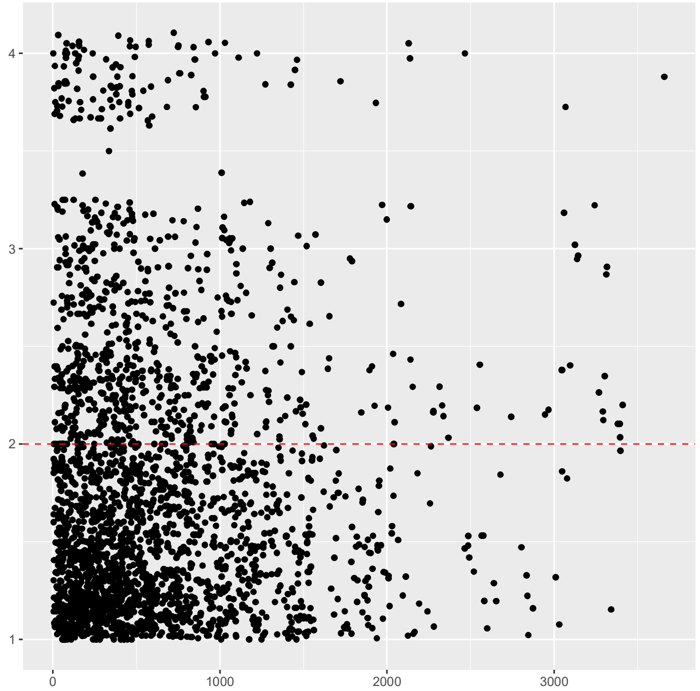
1
2
3
4
0 1000 2000 3000

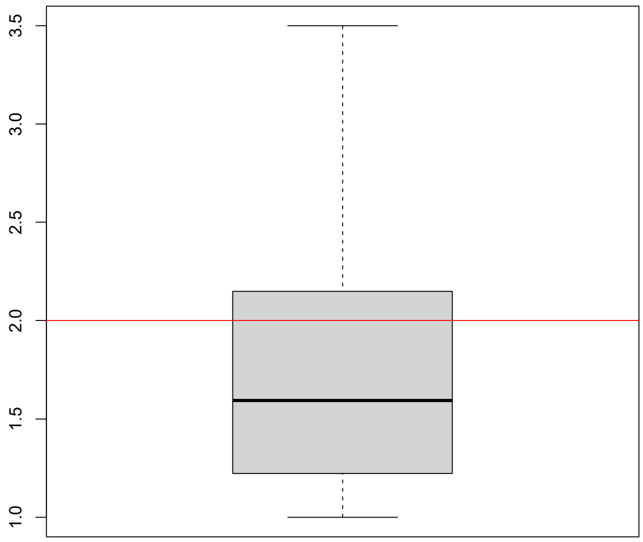
1.0
1.5
2.0
2.5
3.0
3.5

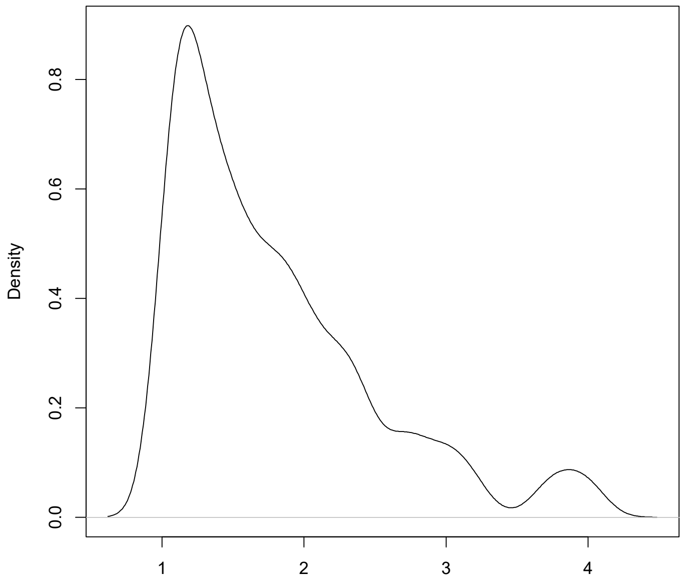
Density
0.0
0.2
0.4
0.6
0.8
1 2 3 4

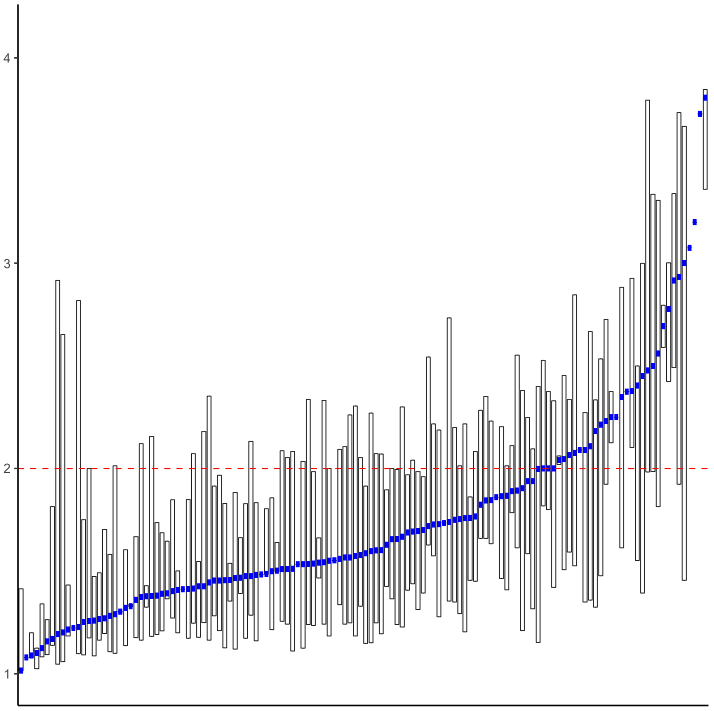
1
2
3
4

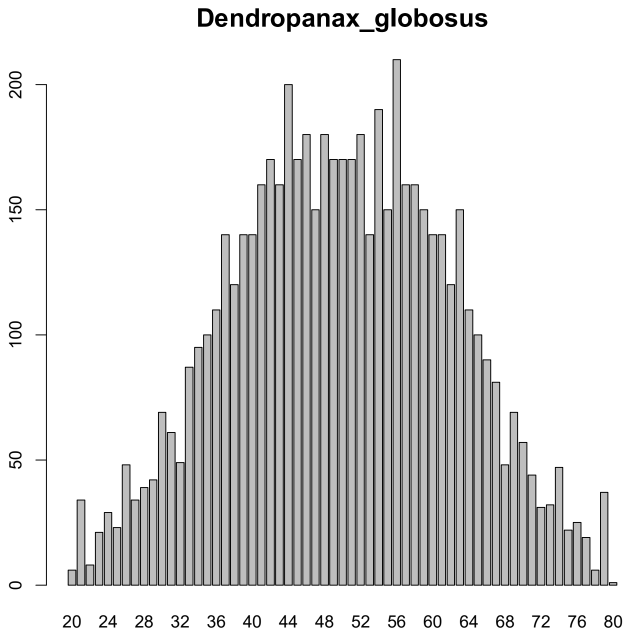
Dendropanax_globosus
0
50
100
150
200
20 24 28 32 36 40 44 48 52 56 60 64 68 72 76 80

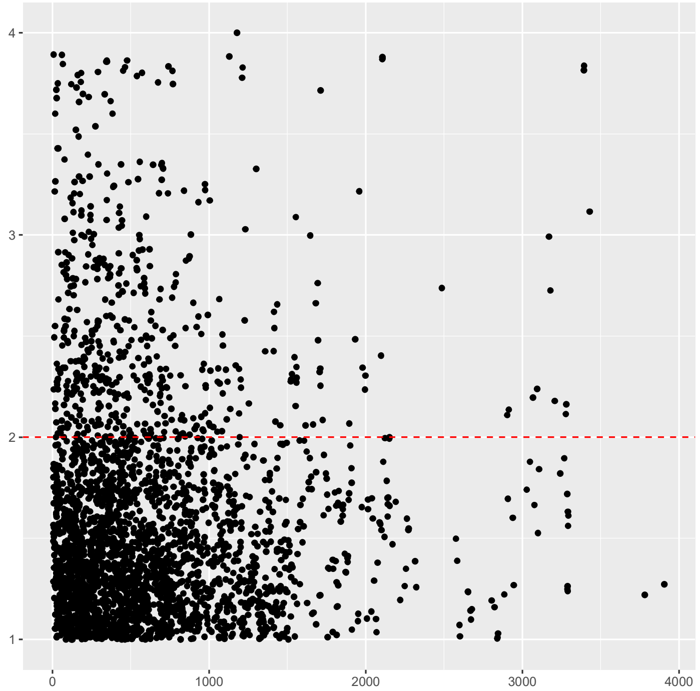
1
2
3
4
0 1000 2000 3000 4000

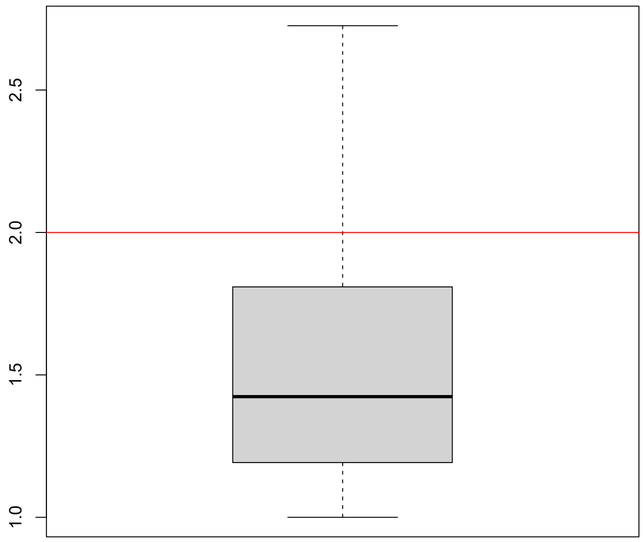
1.0
1.5
2.0
2.5

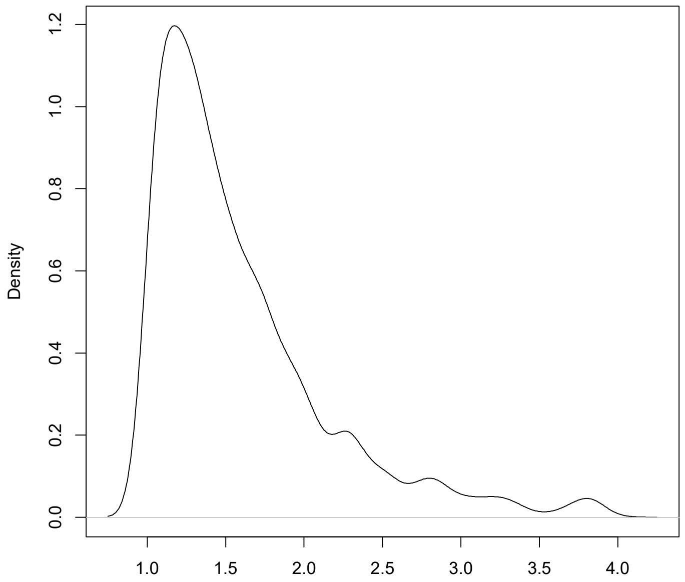
Density
0.0
0.2
0.4
0.6
0.8
1.0
1.2
1.0 1.5 2.0 2.5 3.0 3.5 4.0

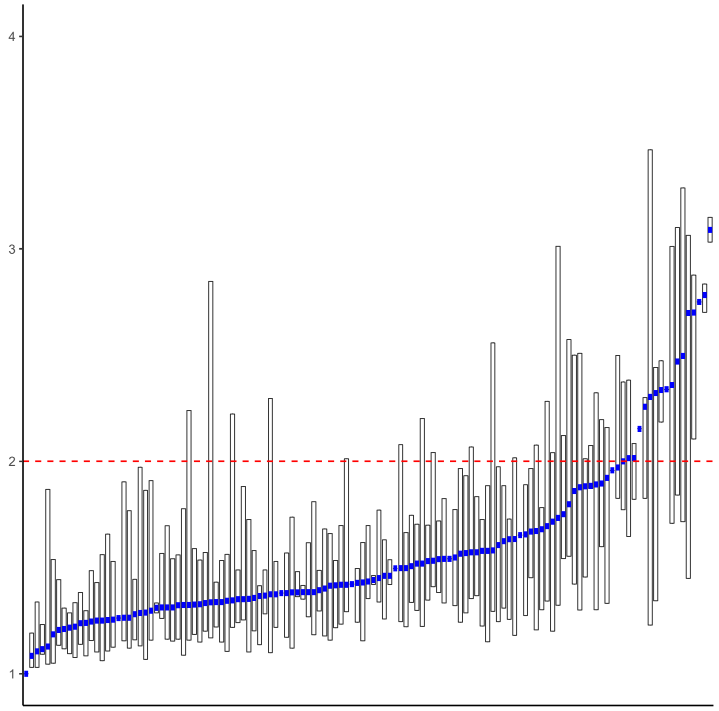
1
2
3
4

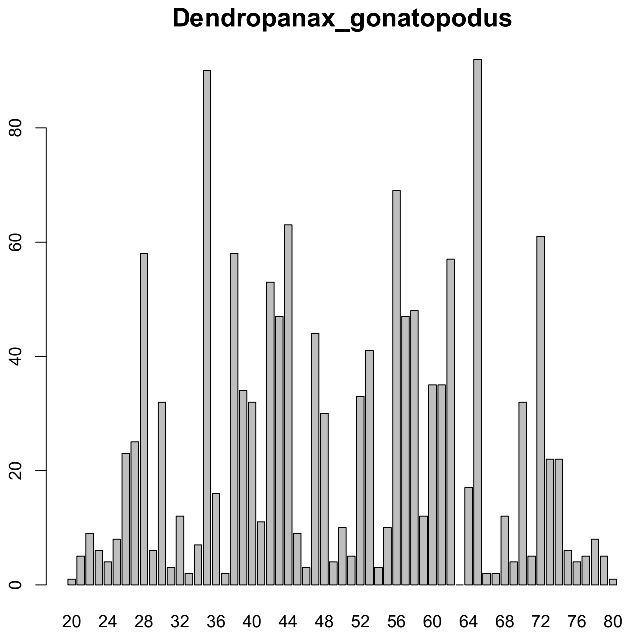
Dendropanax_gonatopodus
0
20
40
60
80
20 24 28 32 36 40 44 48 52 56 60 64 68 72 76 80

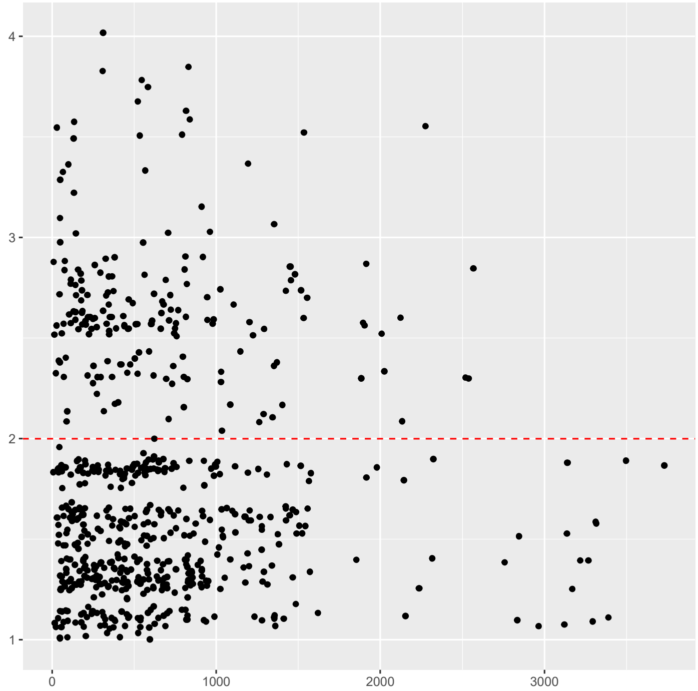
1
2
3
4
0 1000 2000 3000

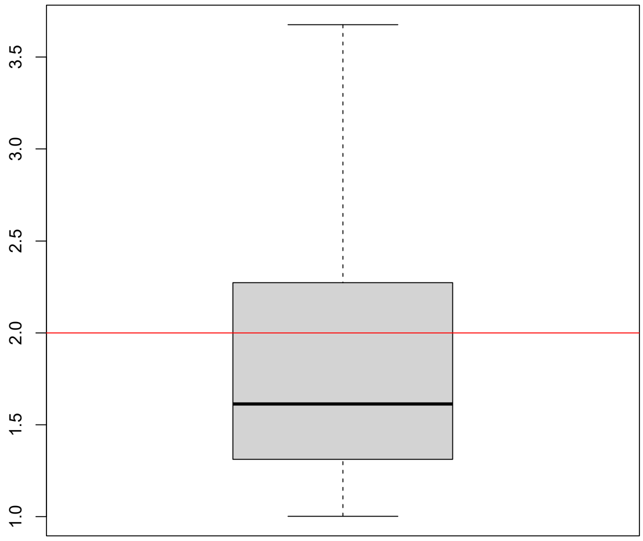
1.0
1.5
2.0
2.5
3.0
3.5

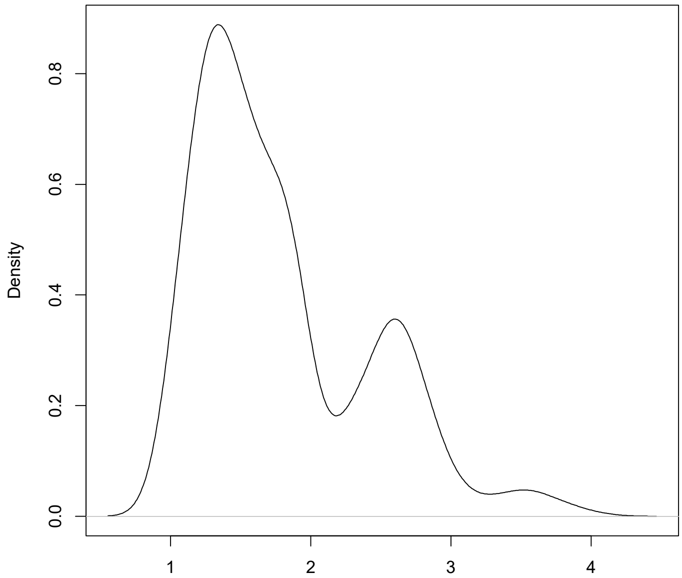
Density
0.0
0.2
0.4
0.6
0.8
1 2 3 4

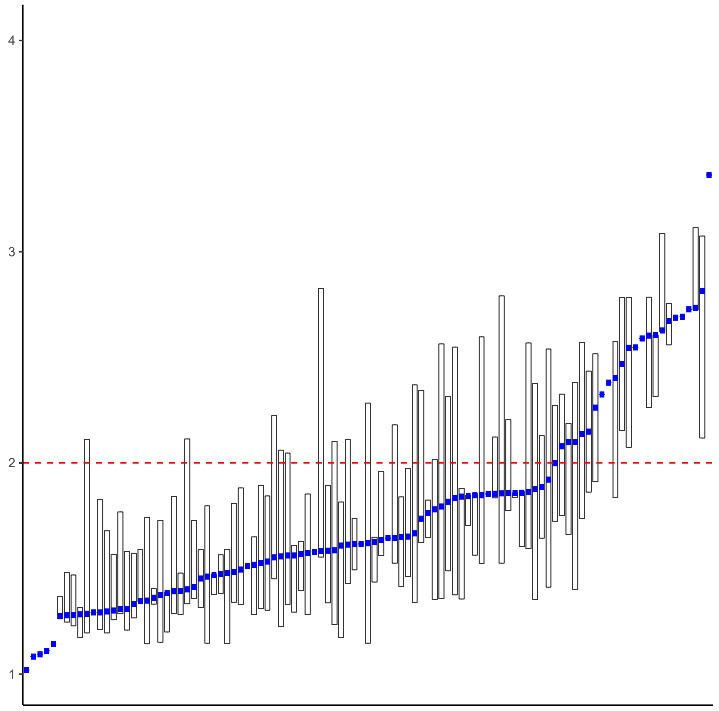
1
2
3
4

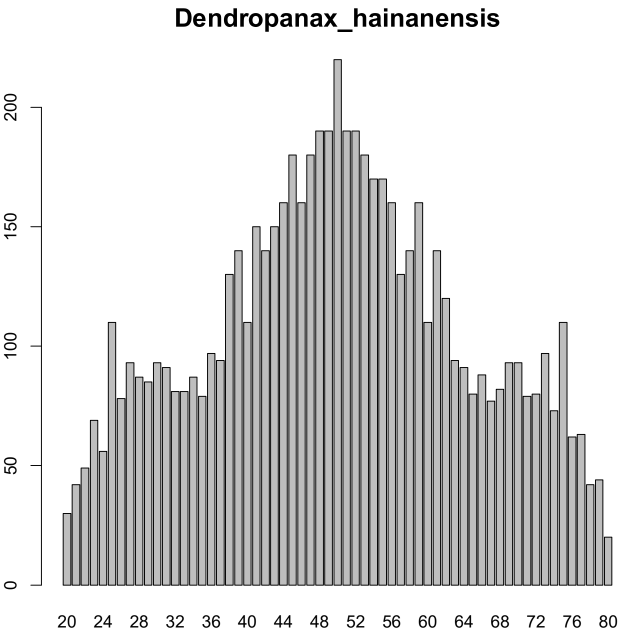
Dendropanax_hainanensis
0
50
100
150
200
20 24 28 32 36 40 44 48 52 56 60 64 68 72 76 80

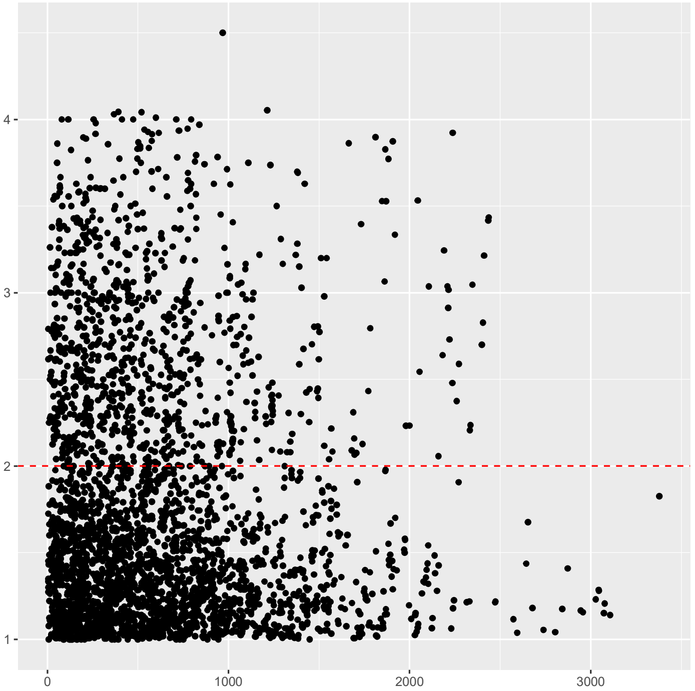
1
2
3
4
0
1000
2000
3000

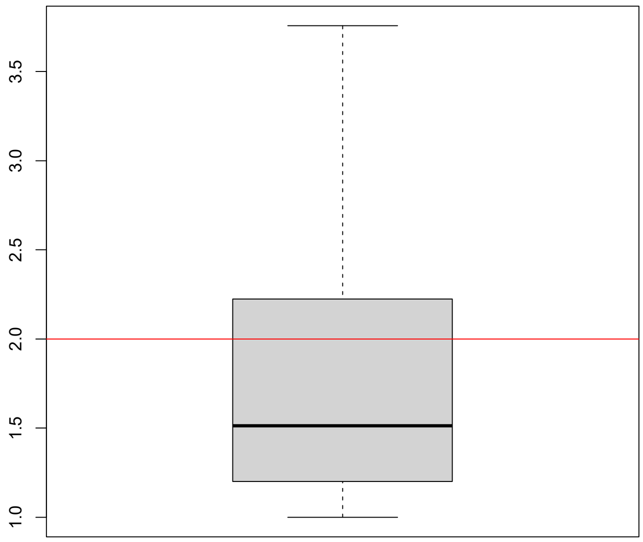
1.0
1.5
2.0
2.5
3.0
3.5

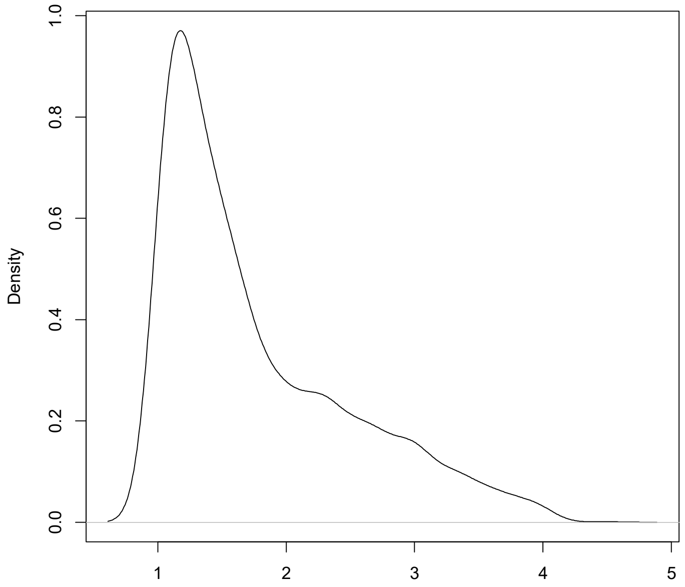
Density
0.0
0.2
0.4
0.6
0.8
1.0
1 2 3 4 5

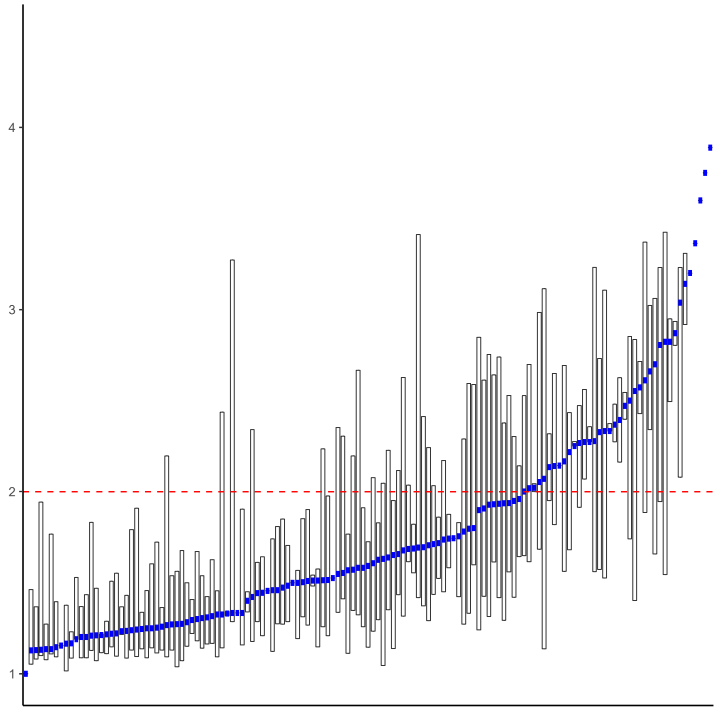
1
2
3
4

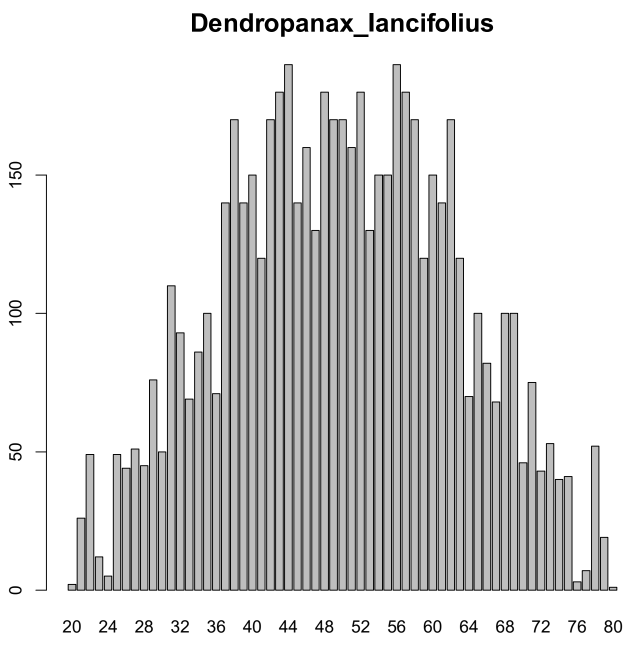
Dendropanax_lancifolius
0
50
100
150
20
24
28
32
36
40
44
48
52
56
60
64
68
72
76
80

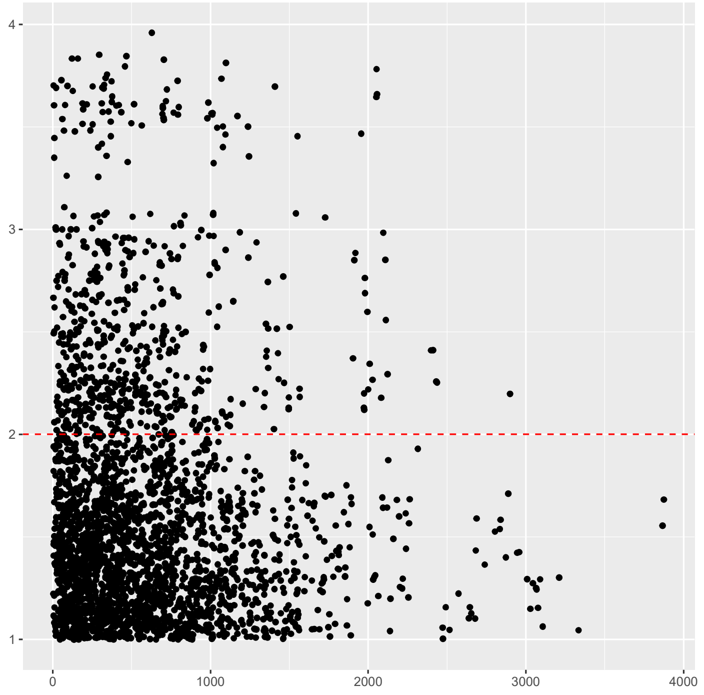
1
2
3
4
0
1000
2000
3000
4000

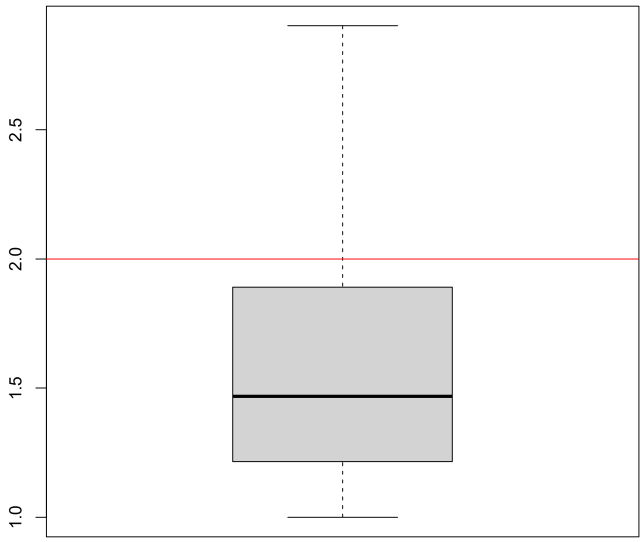
1.0
1.5
2.0
2.5

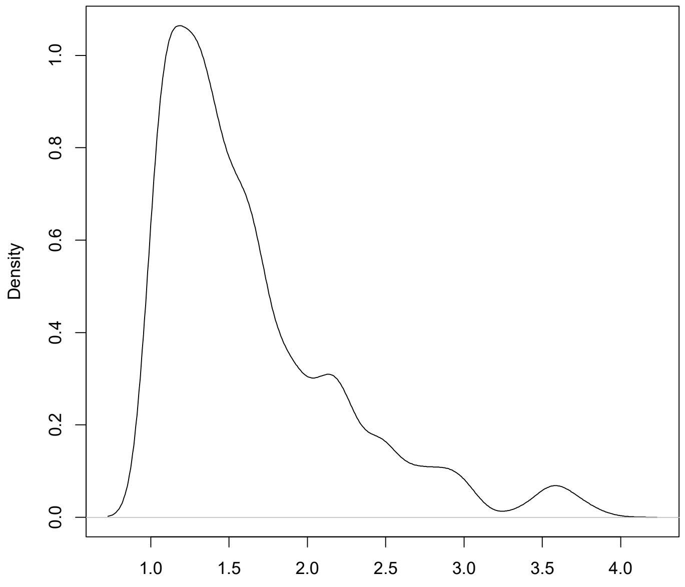
Density
0.0
0.2
0.4
0.6
0.8
1.0
1.0
1.5
2.0
2.5
3.0
3.5
4.0

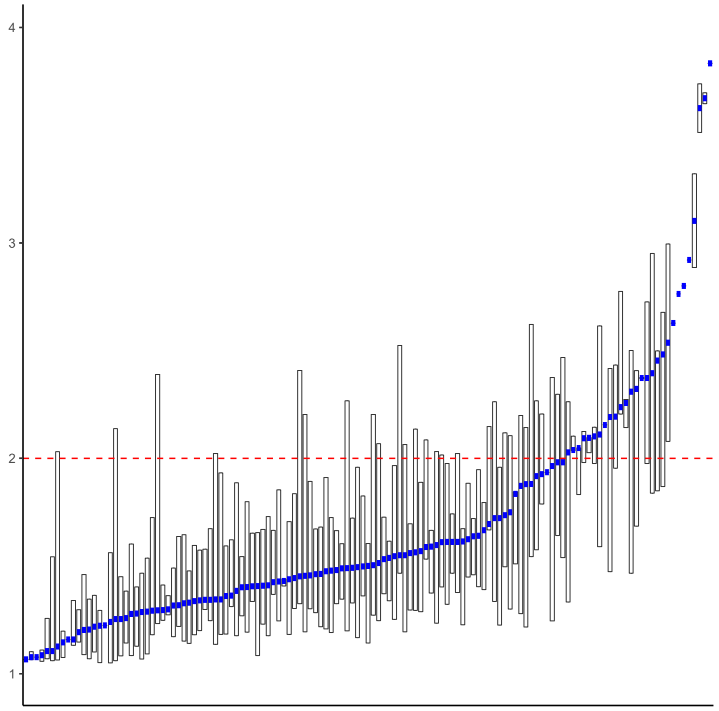
1
2
3
4

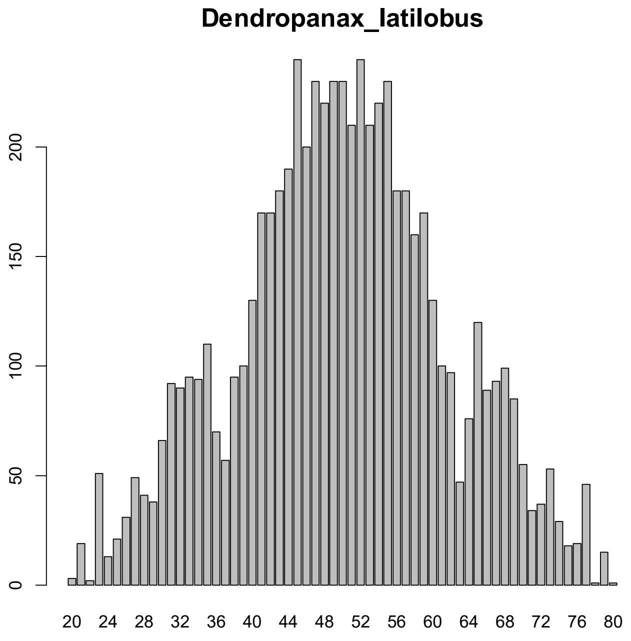

Dendropanax_latilobus

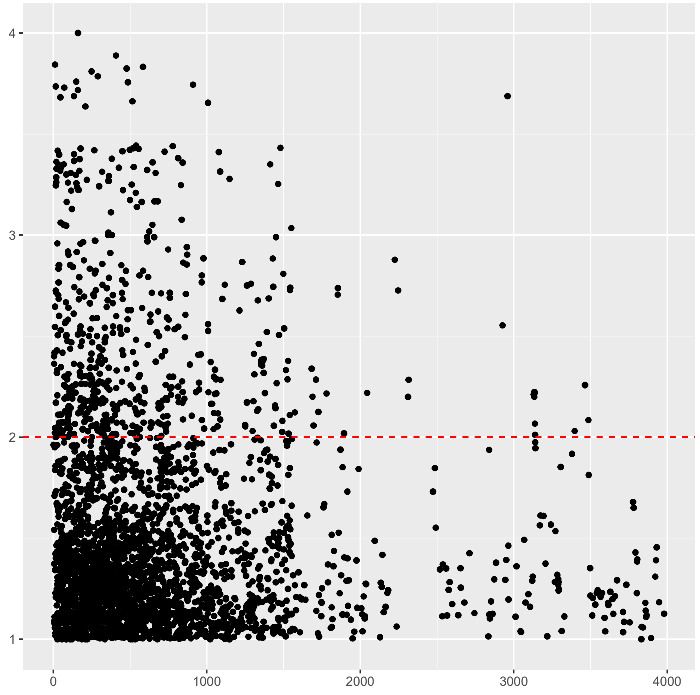

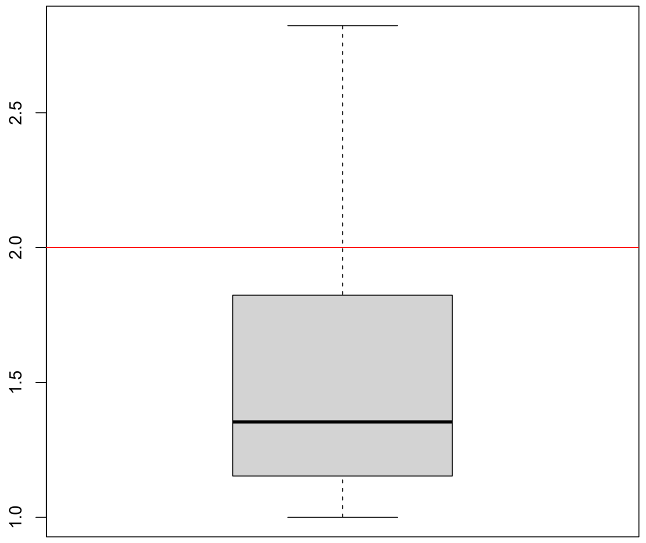

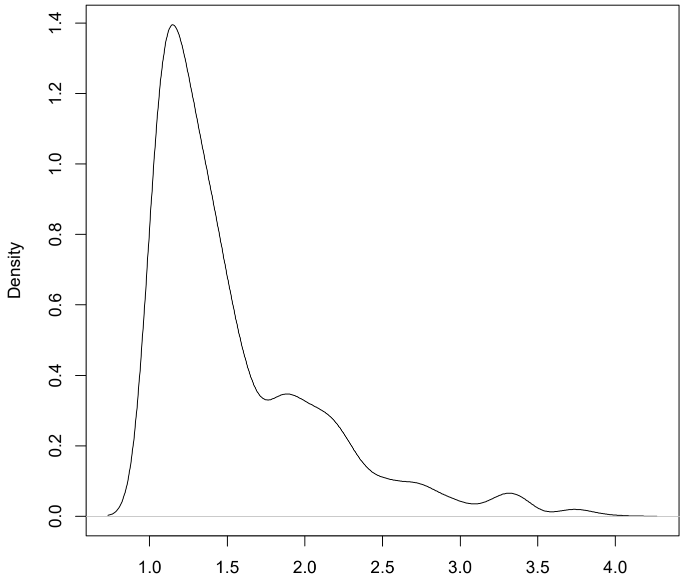

Density

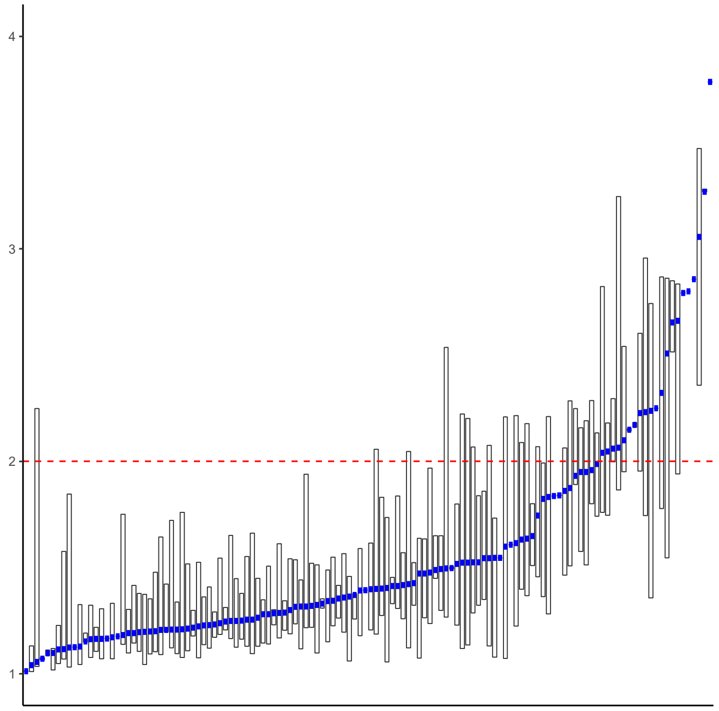

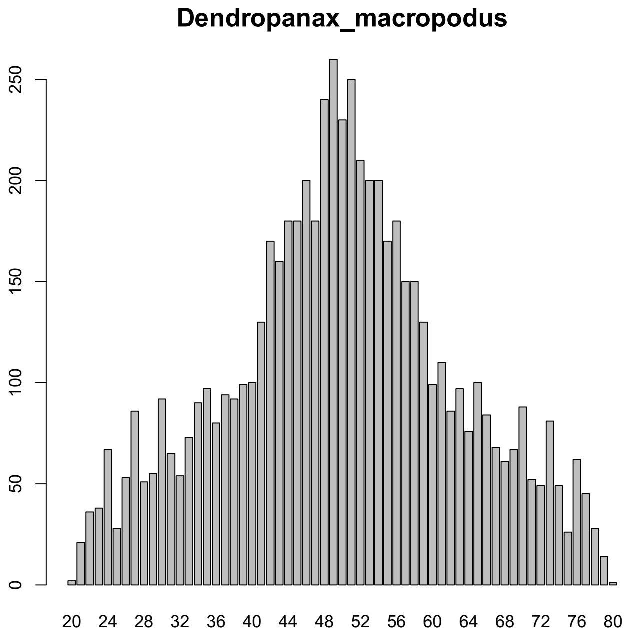
Dendropanax_macropodus
0
50
100
150
200
250
20 24 28 32 36 40 44 48 52 56 60 64 68 72 76 80

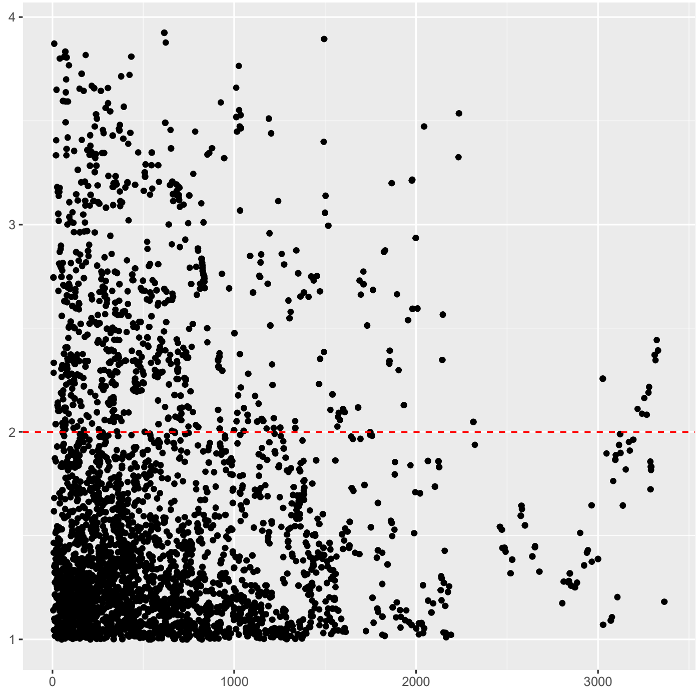
1
2
3
4
0 1000 2000 3000

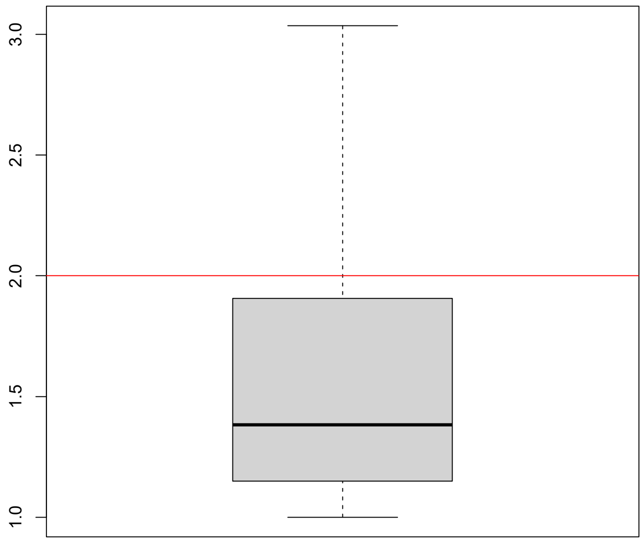
1.0
1.5
2.0
2.5
3.0

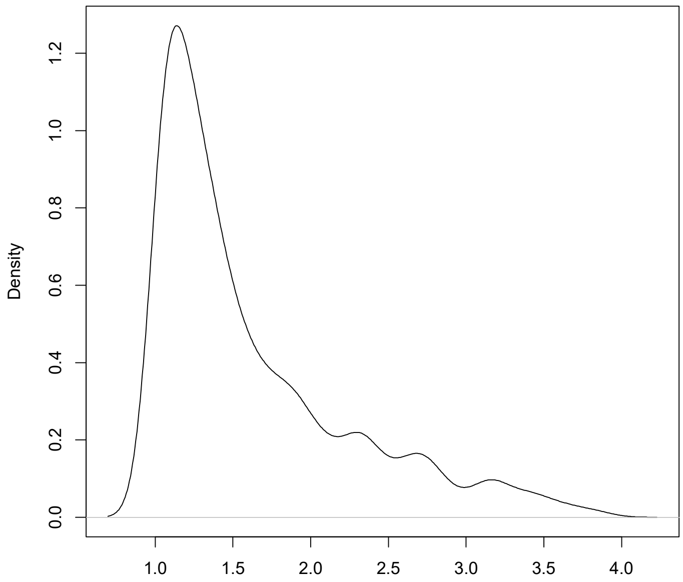
Density
0.0
0.2
0.4
0.6
0.8
1.0
1.2
1.0 1.5 2.0 2.5 3.0 3.5 4.0

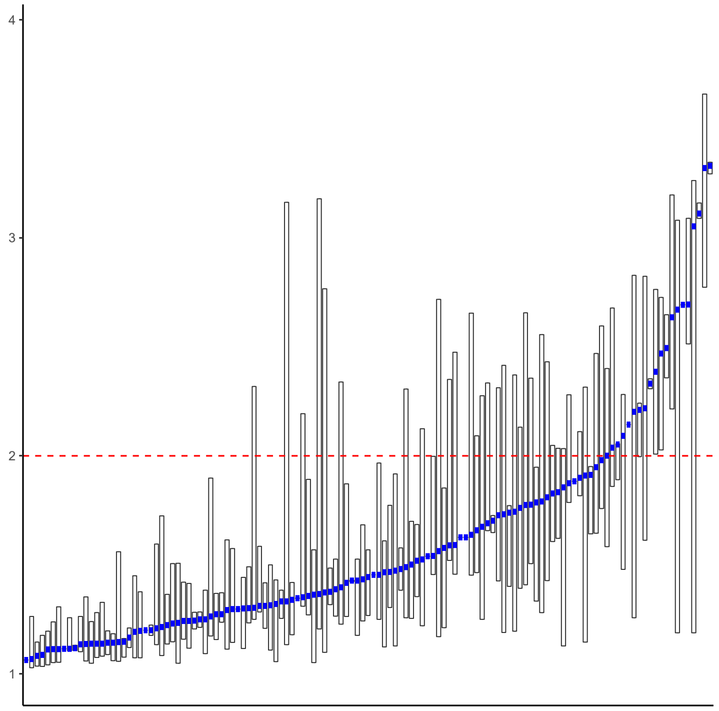
1
2
3
4

Dendropanax_maingayi

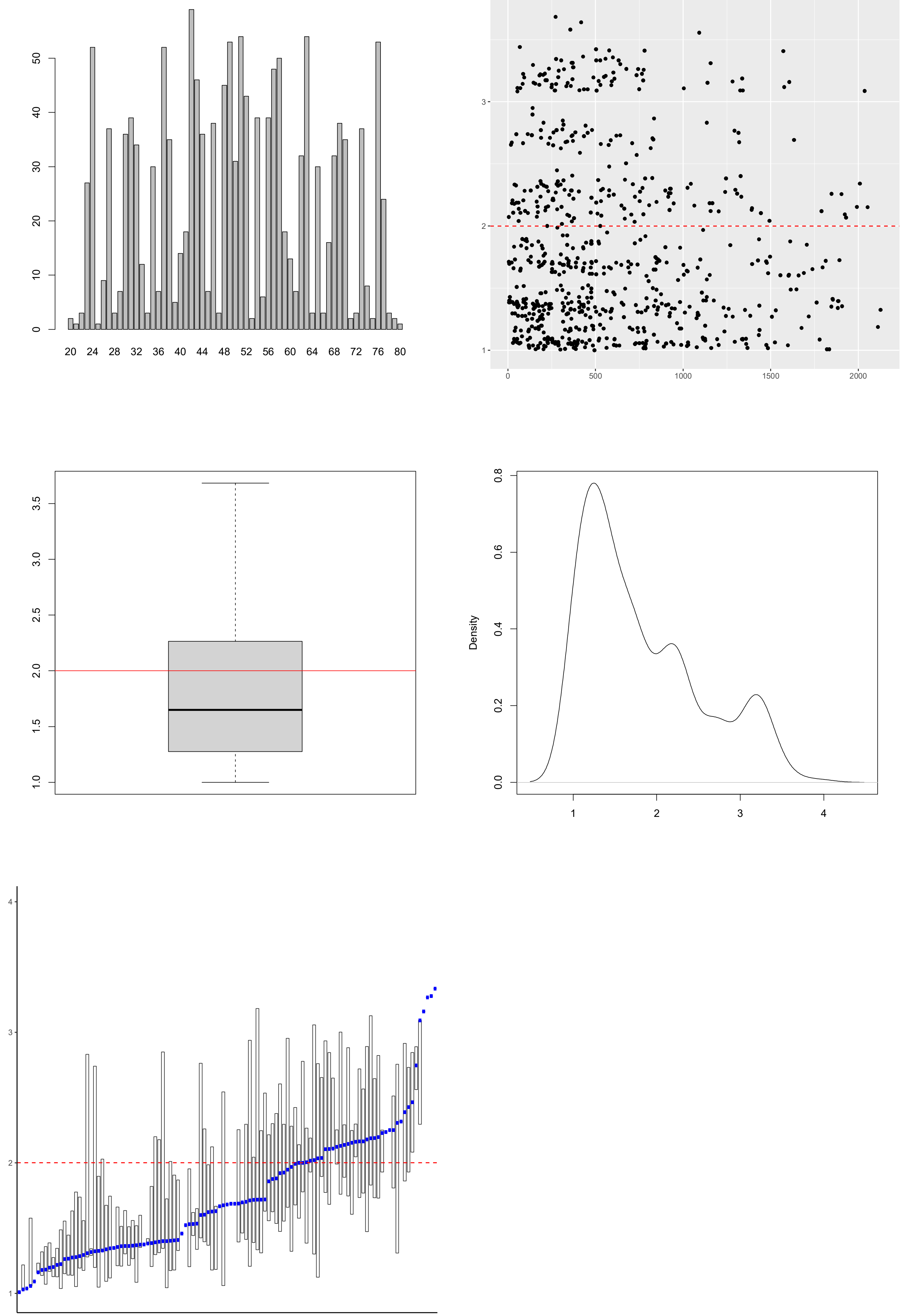

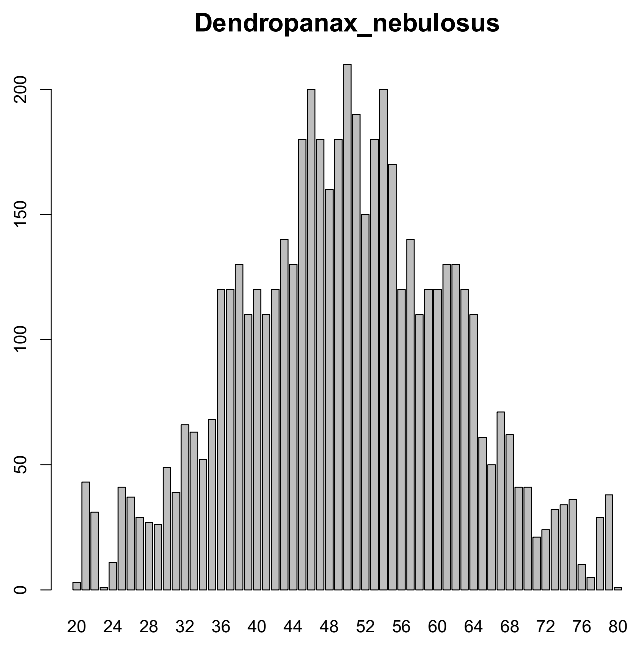
Dendropanax_nebulosus
0
50
100
150
200
20 24 28 32 36 40 44 48 52 56 60 64 68 72 76 80
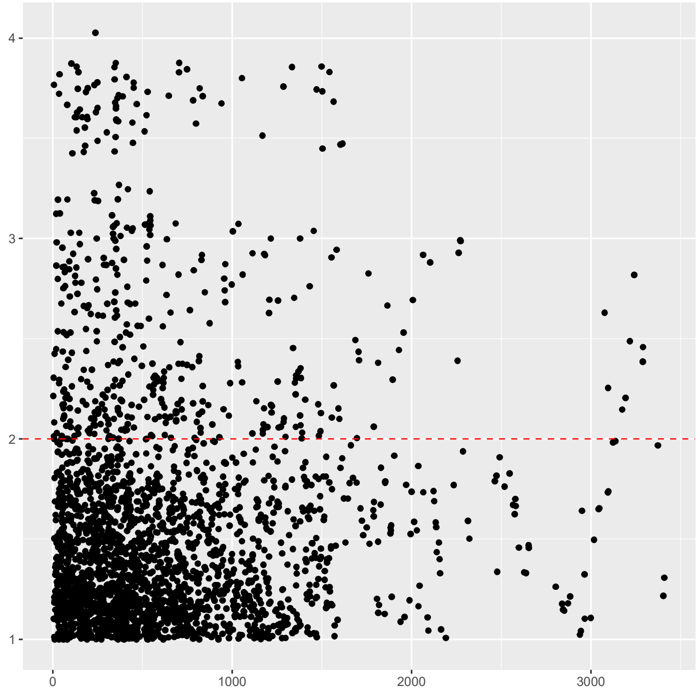
1
2
3
4
0
1000
2000
3000
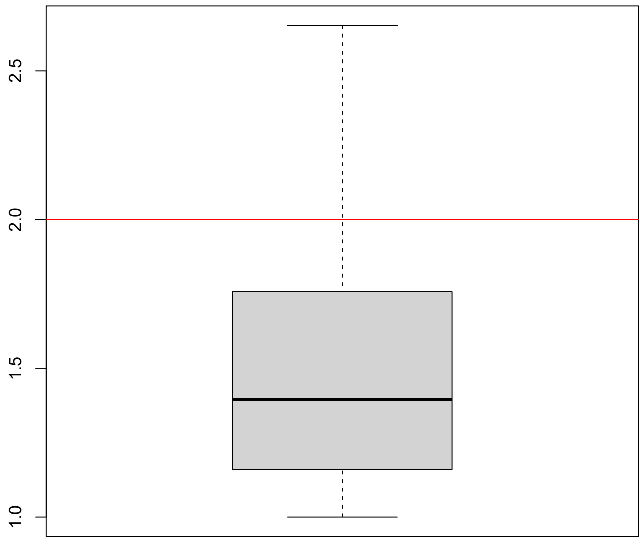
1.0
1.5
2.0
2.5
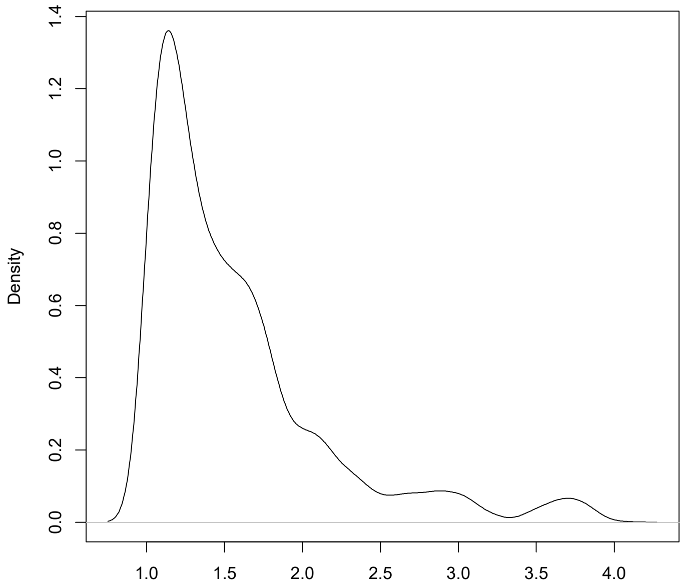
Density
0.0
0.2
0.4
0.6
0.8
1.0
1.2
1.4
1.0 1.5 2.0 2.5 3.0 3.5 4.0
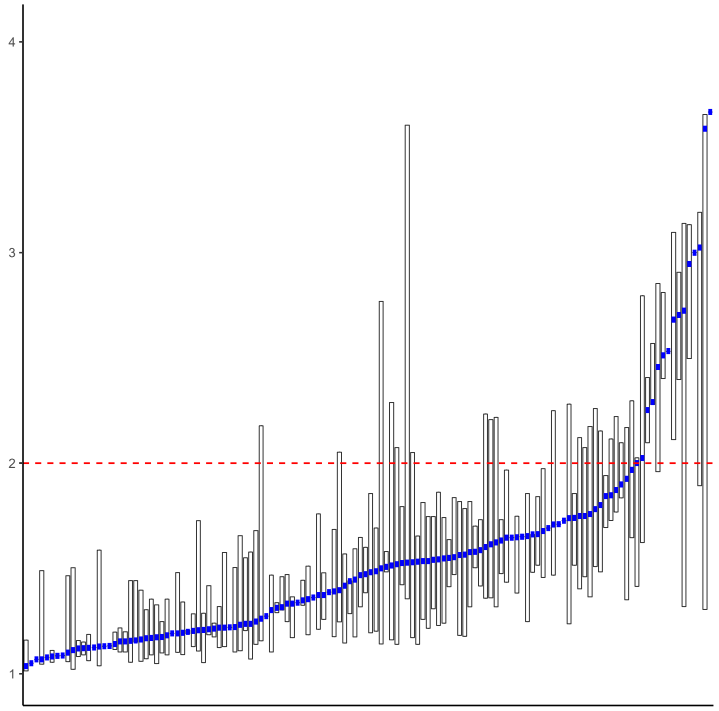
1
2
3
4

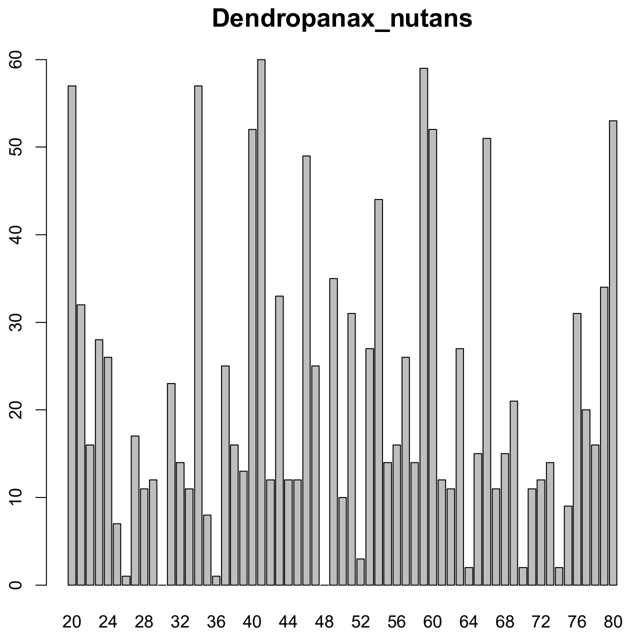
Dendropanax_nutans

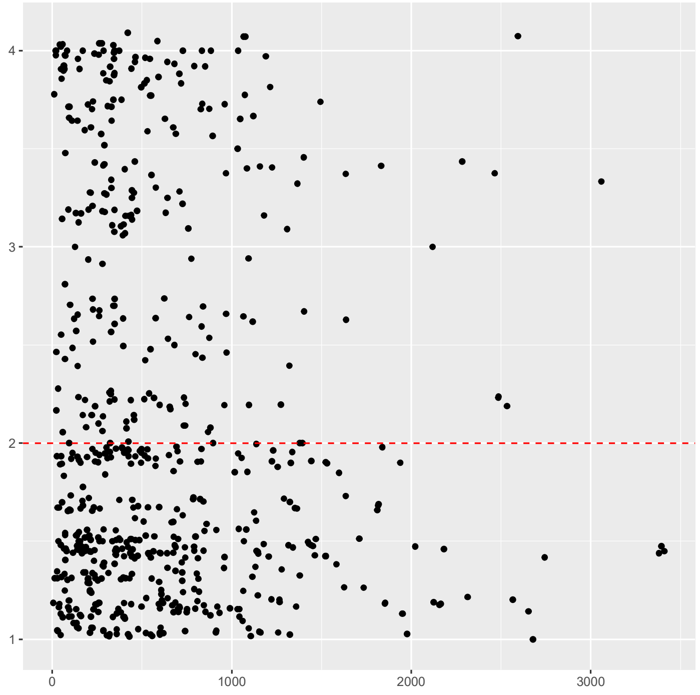

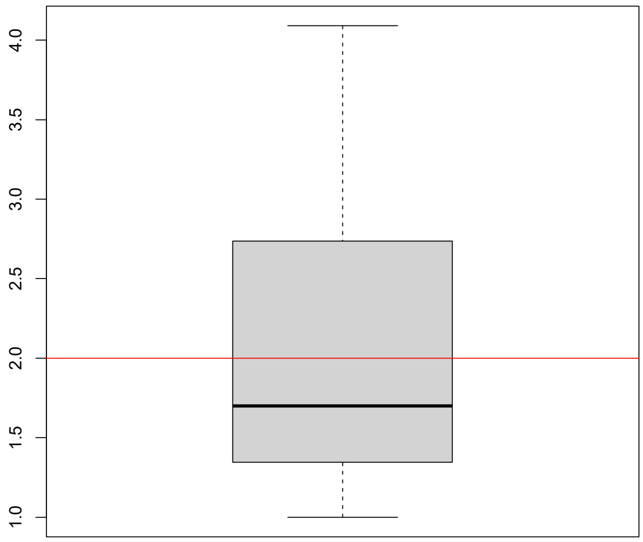

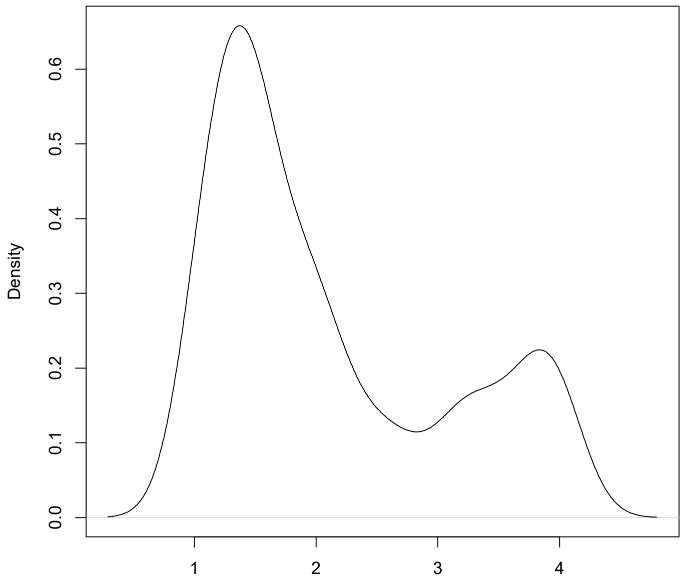
Density

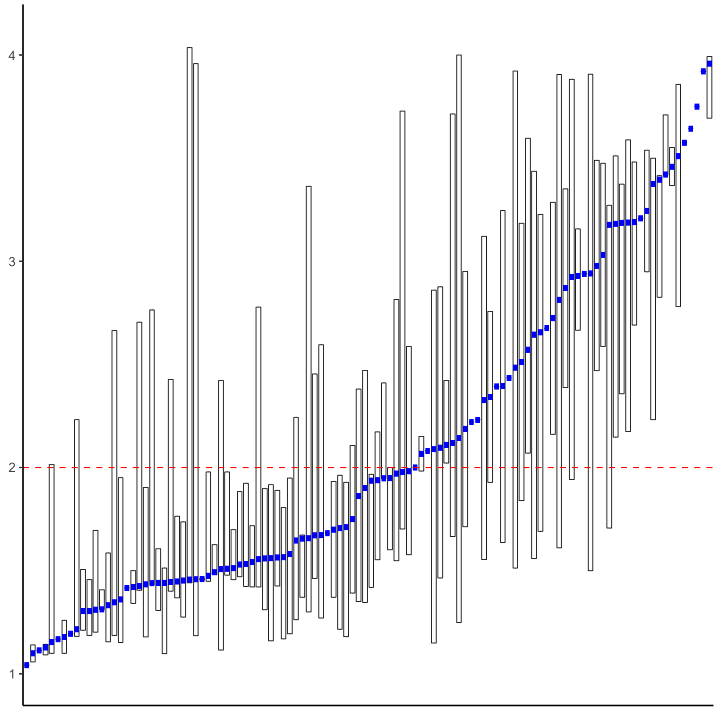

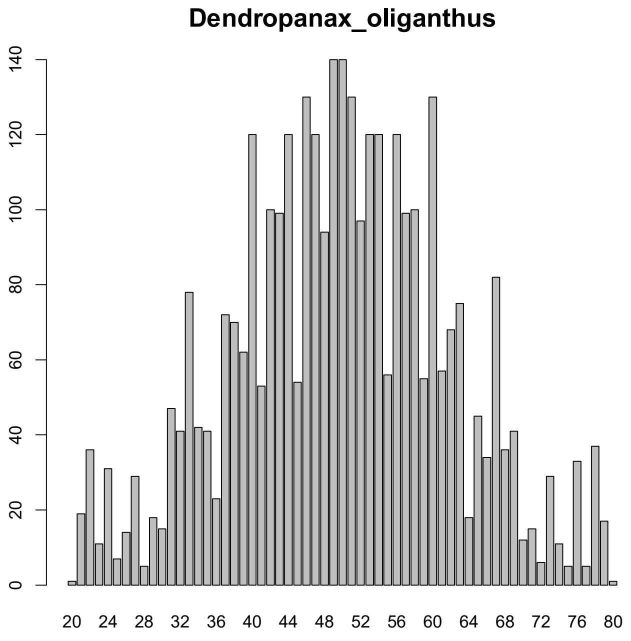
Dendropanax_oliganthus
0 20 40 60 80 100 120 140
20 24 28 32 36 40 44 48 52 56 60 64 68 72 76 80

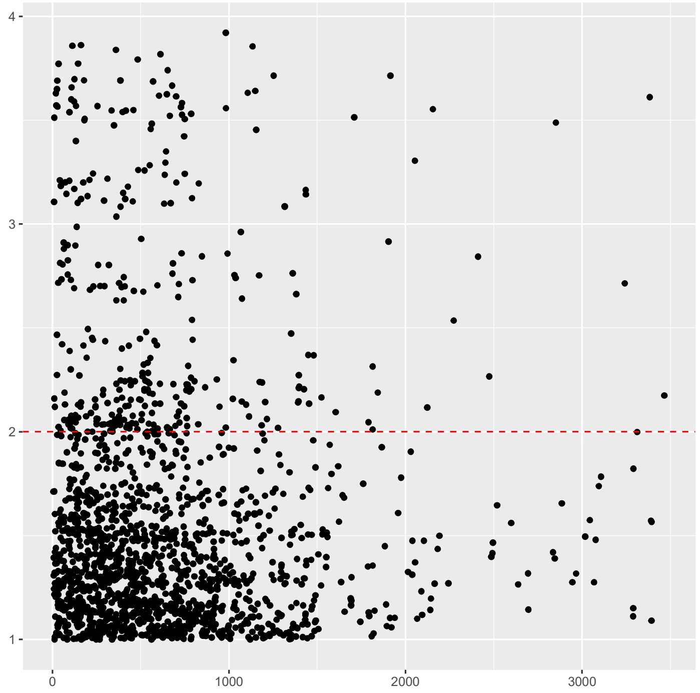
1 2 3 4
0 1000 2000 3000

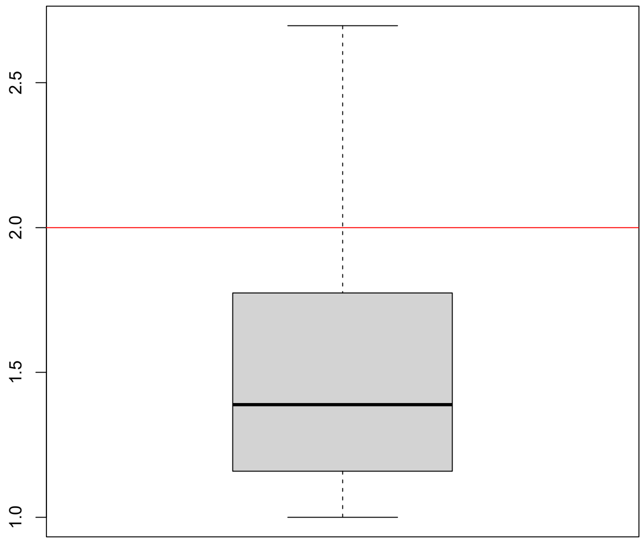
1.0 1.5 2.0 2.5

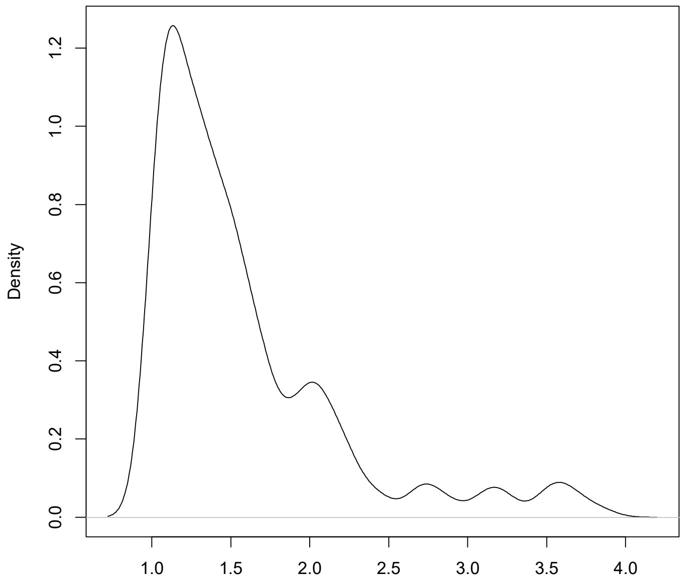
Density
0.0 0.2 0.4 0.6 0.8 1.0 1.2
1.0 1.5 2.0 2.5 3.0 3.5 4.0

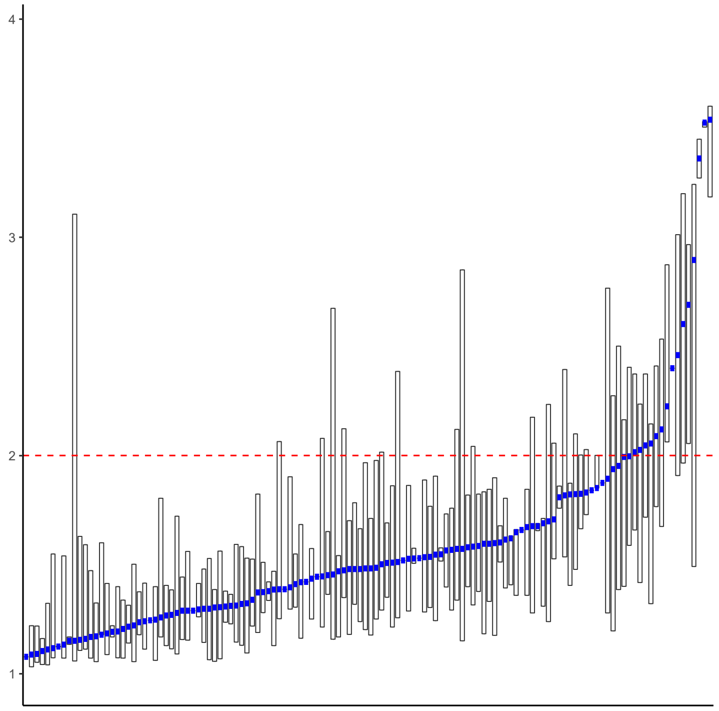
1 2 3 4

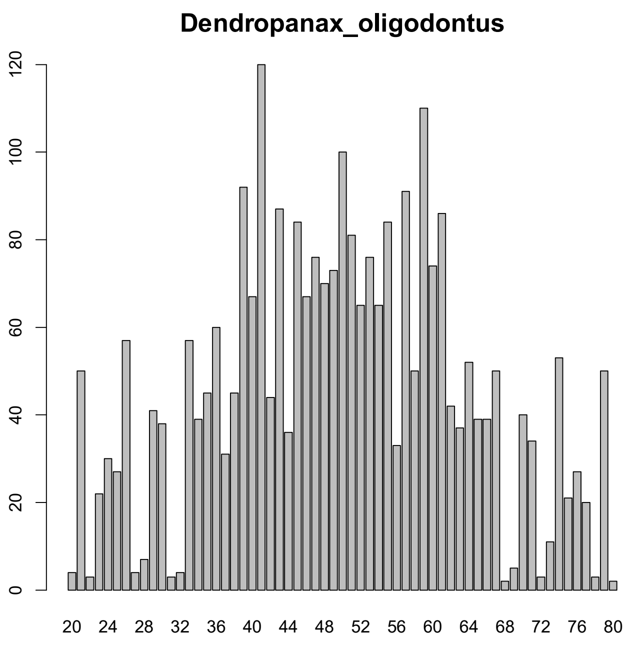
Dendropanax_oligodontus

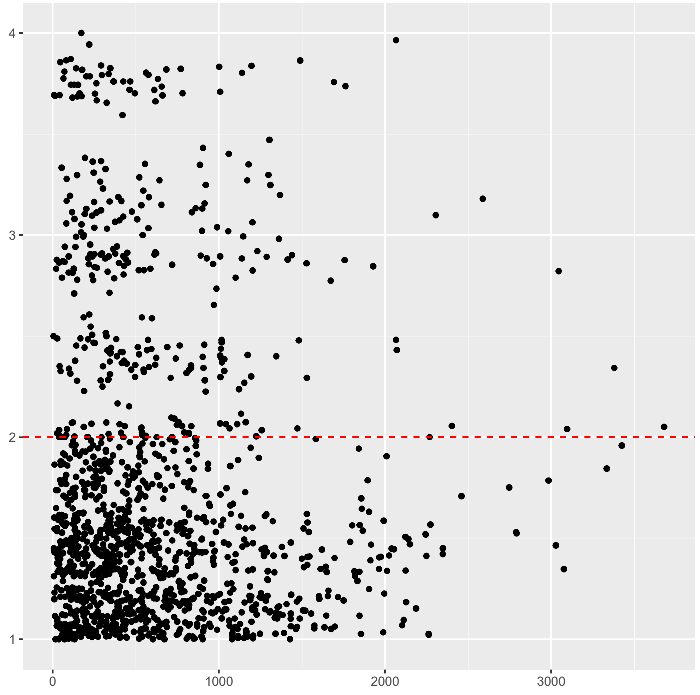

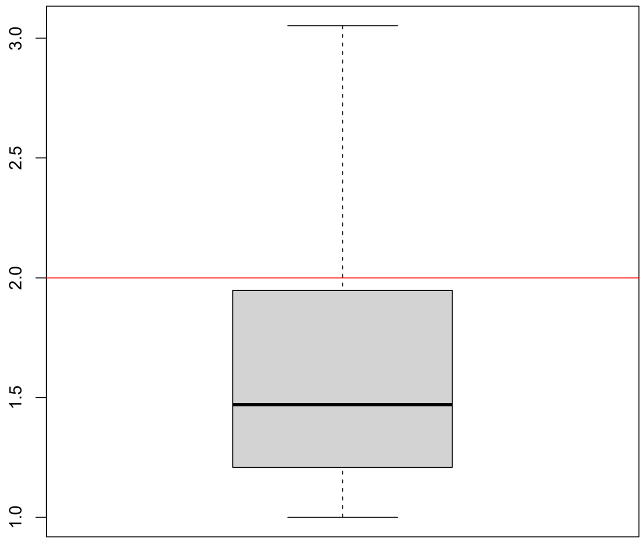

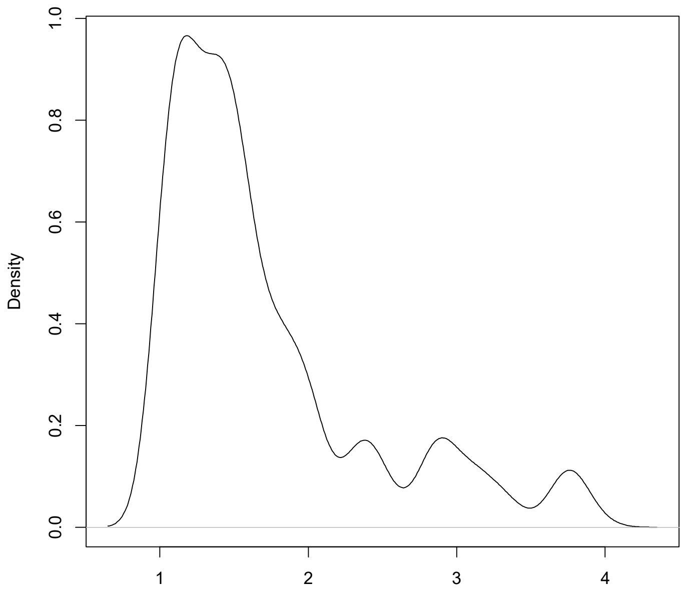
Density

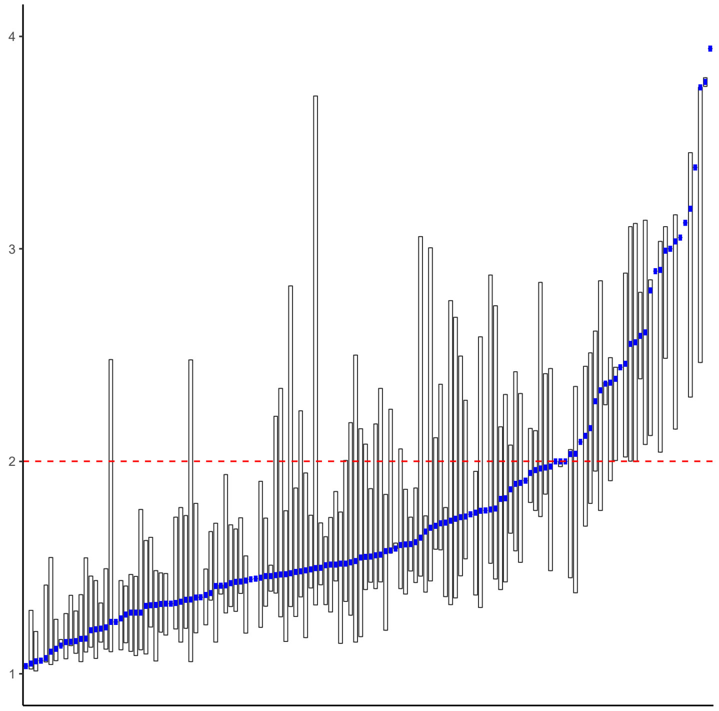

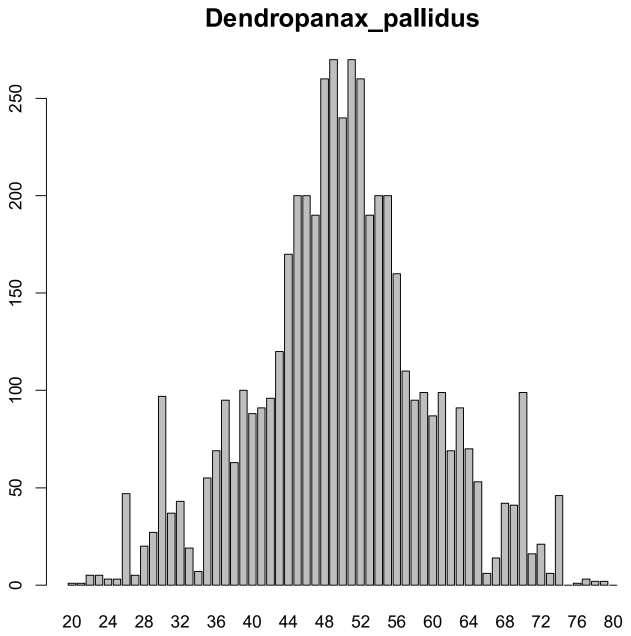
Dendropanax_pallidus

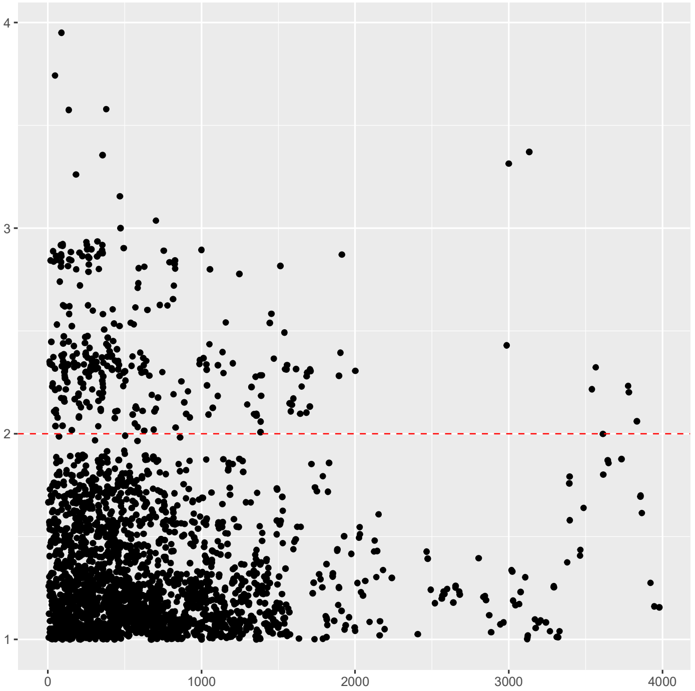

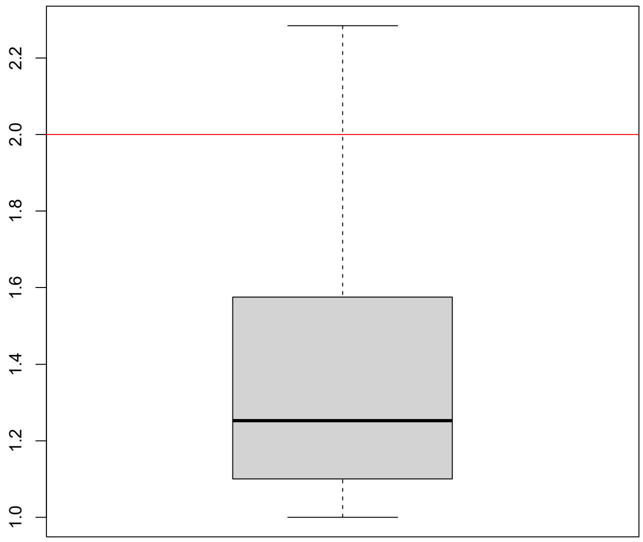

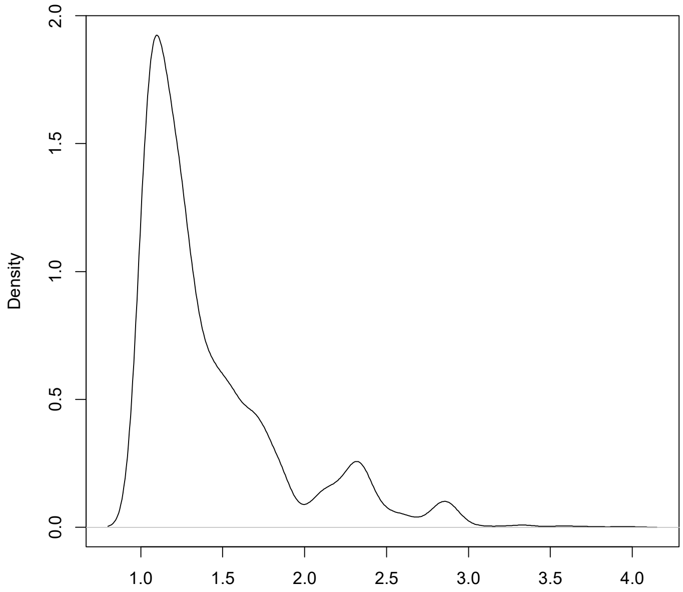
Density

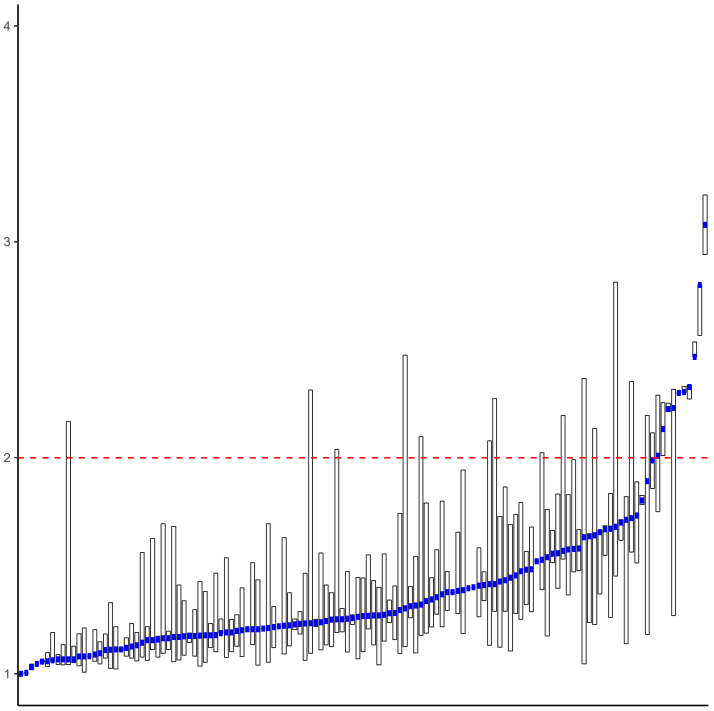

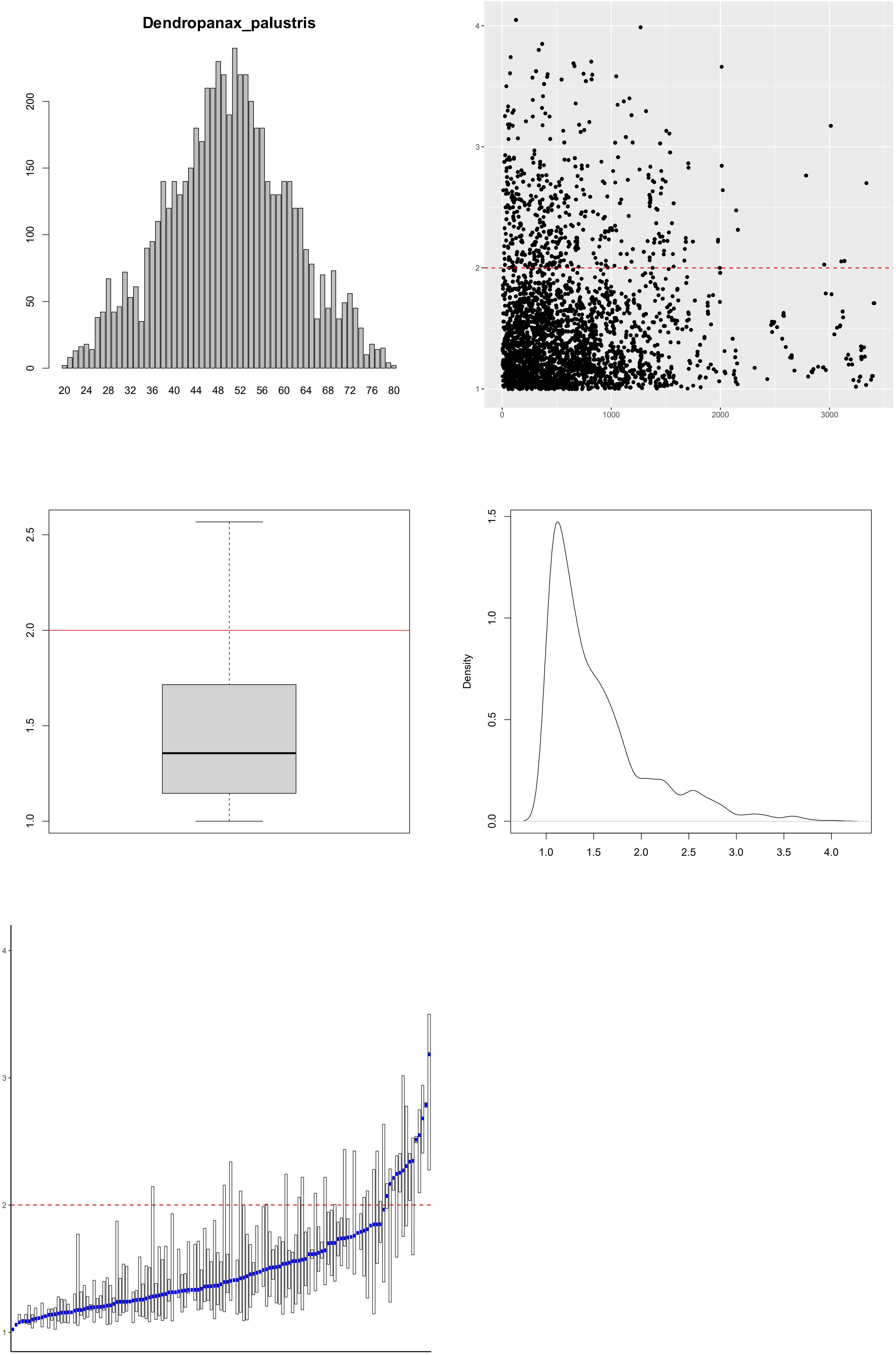
Dendropanax_palustris
Density

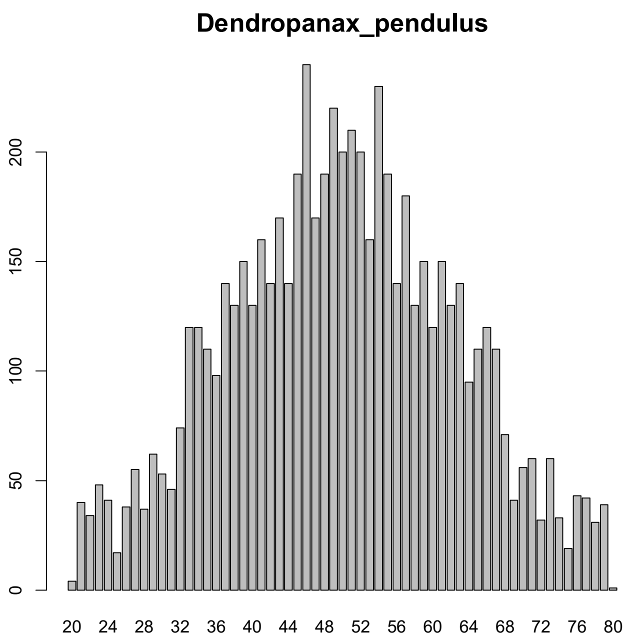
Dendropanax_pendulus

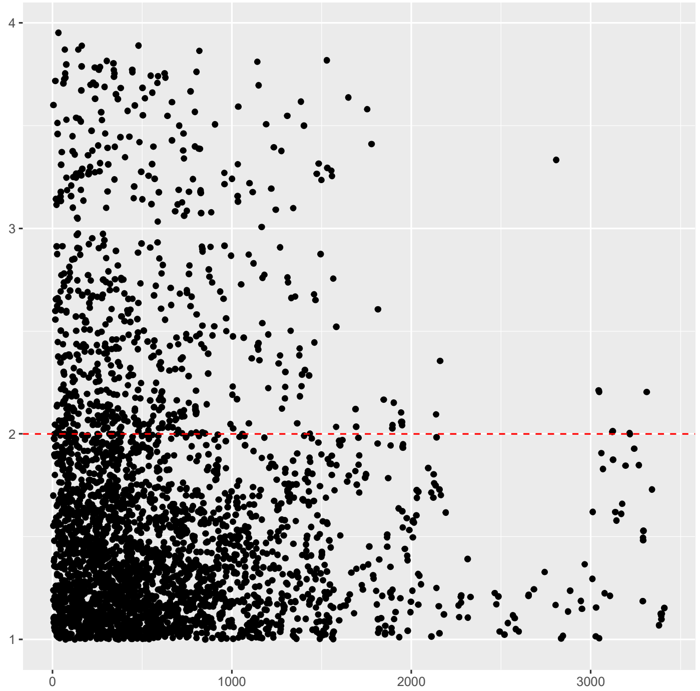

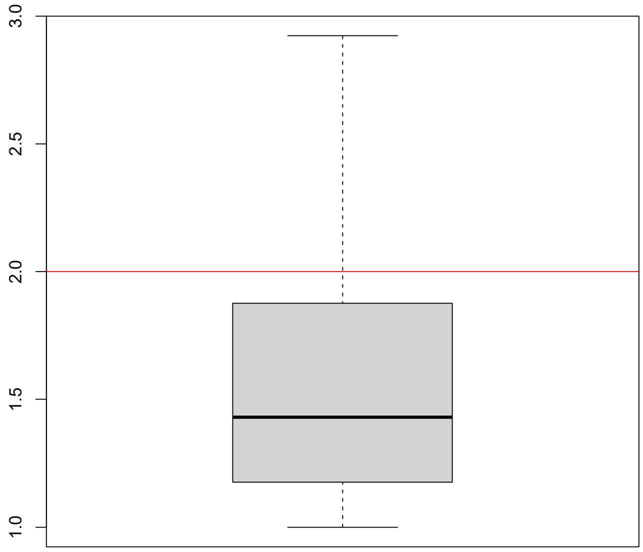

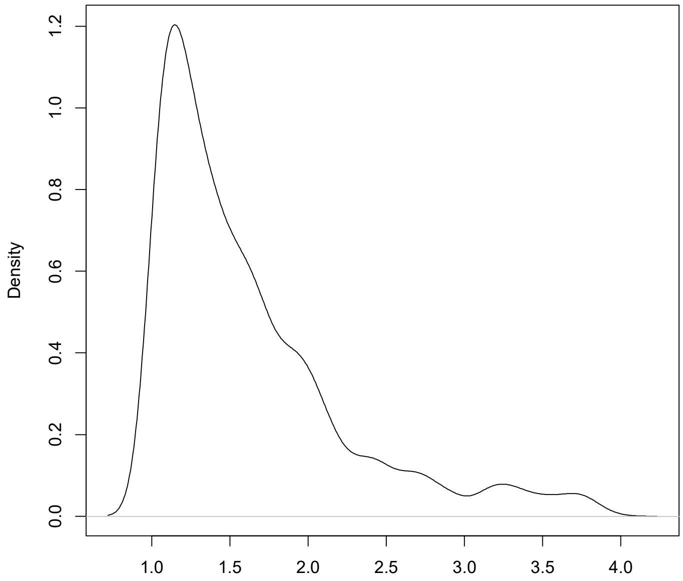
Density

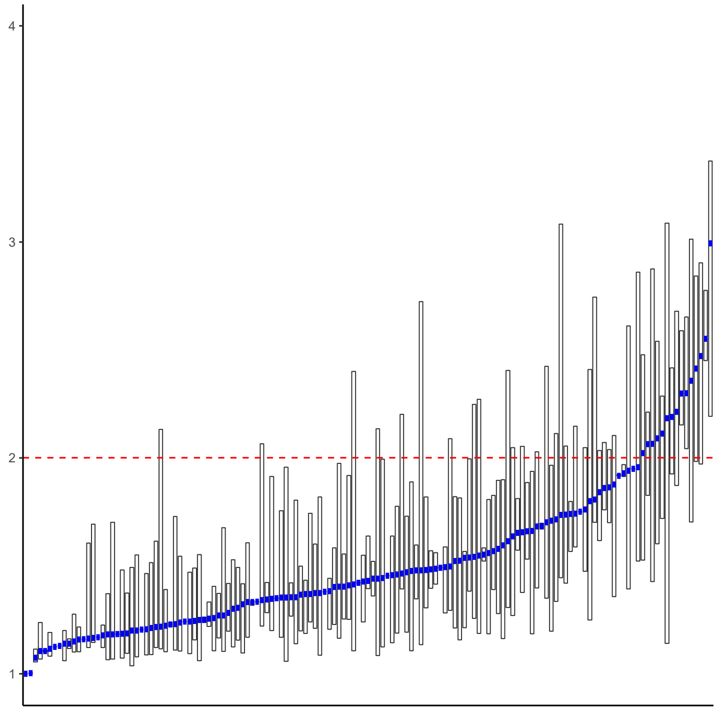

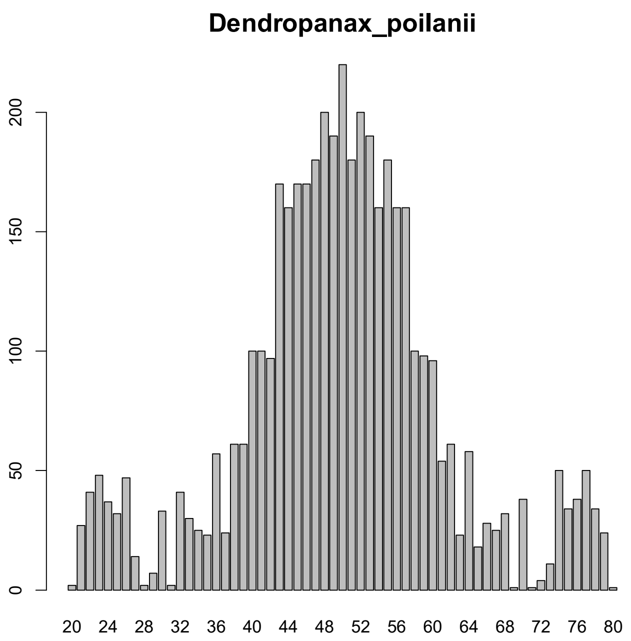
Dendropanax_poilanii

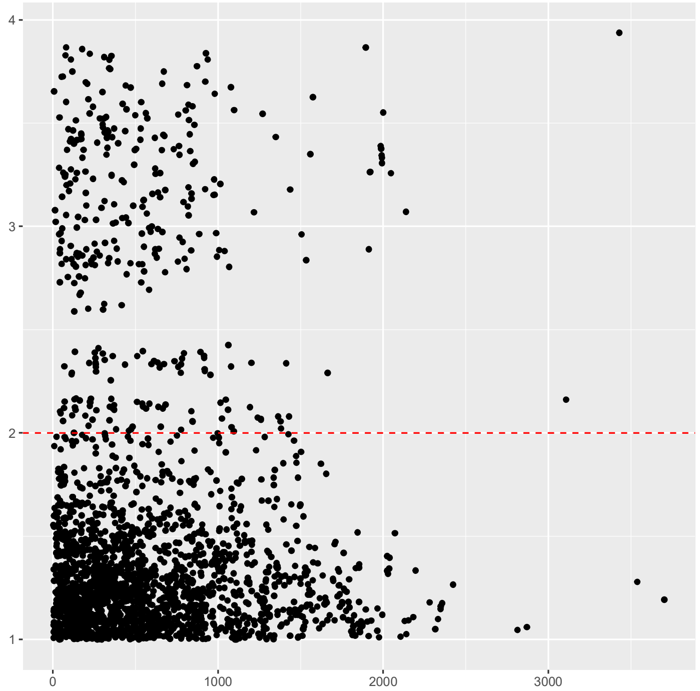

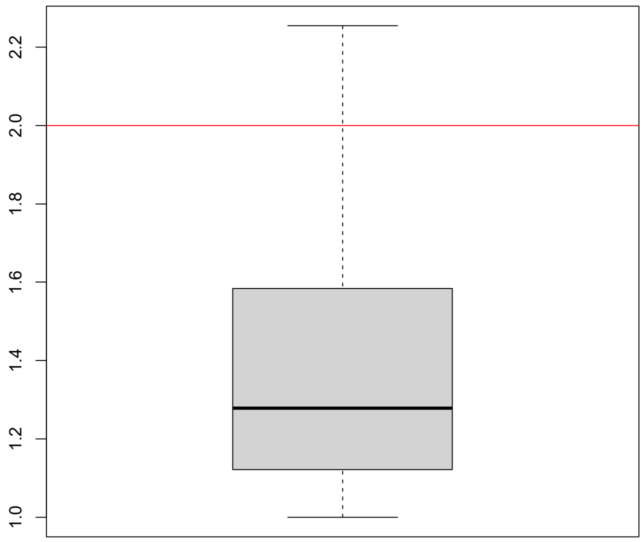

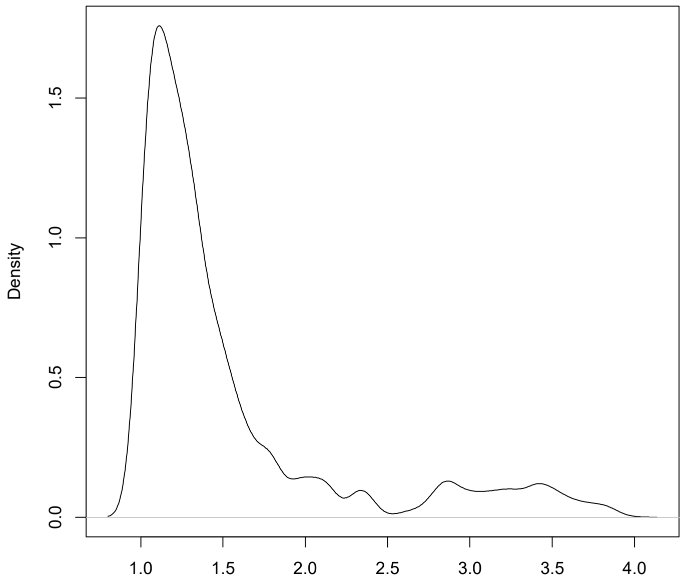
Density

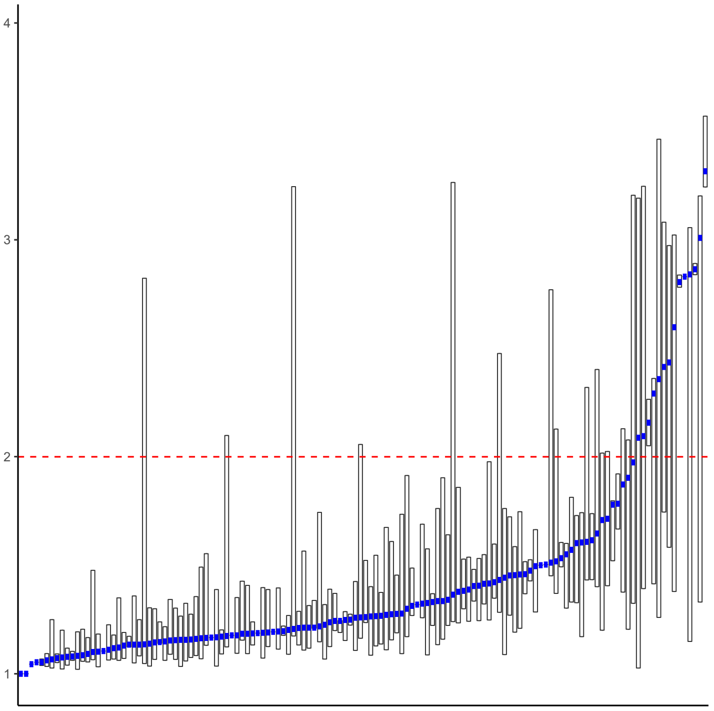

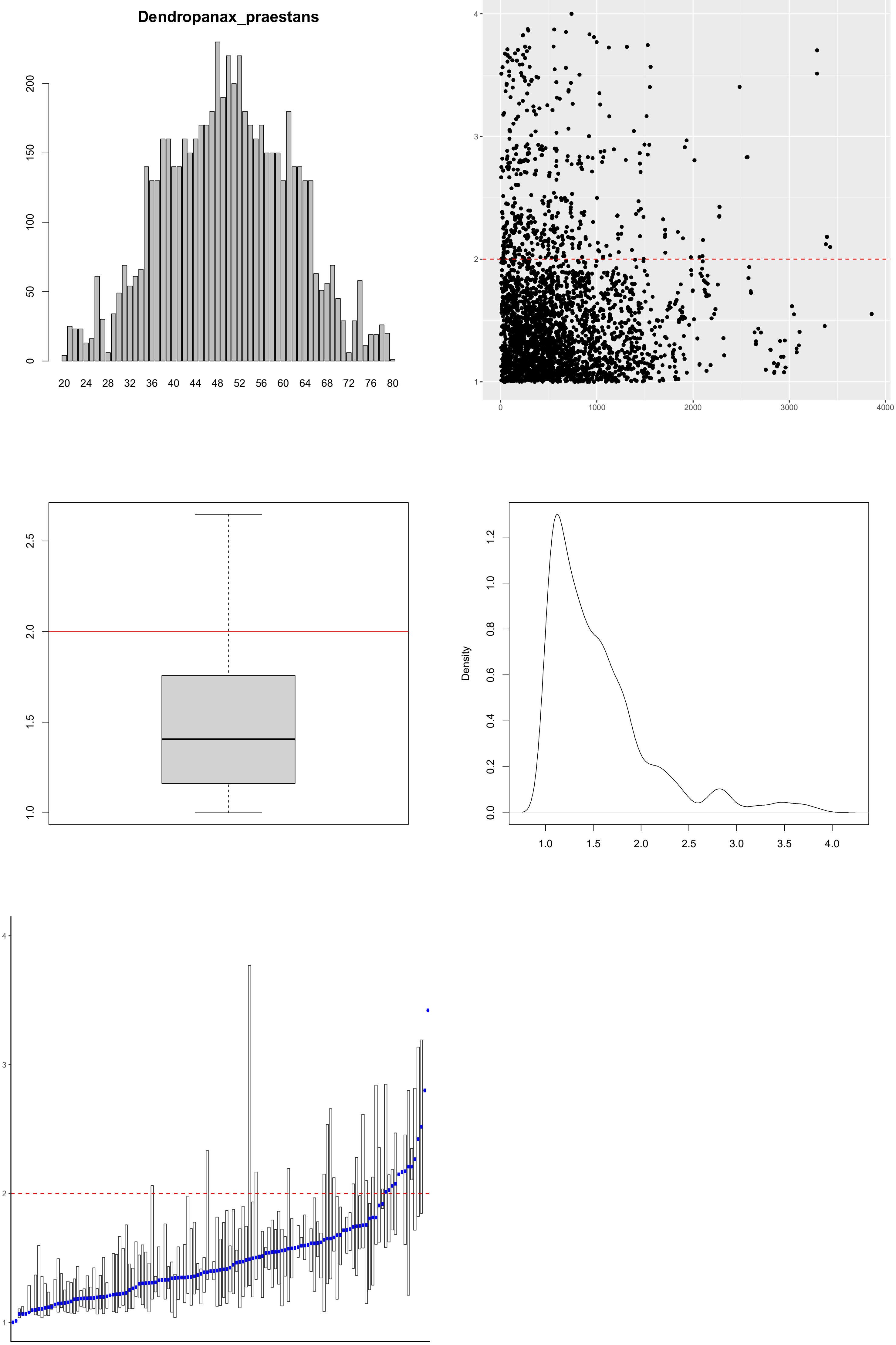
Dendropanax_praestans
Density

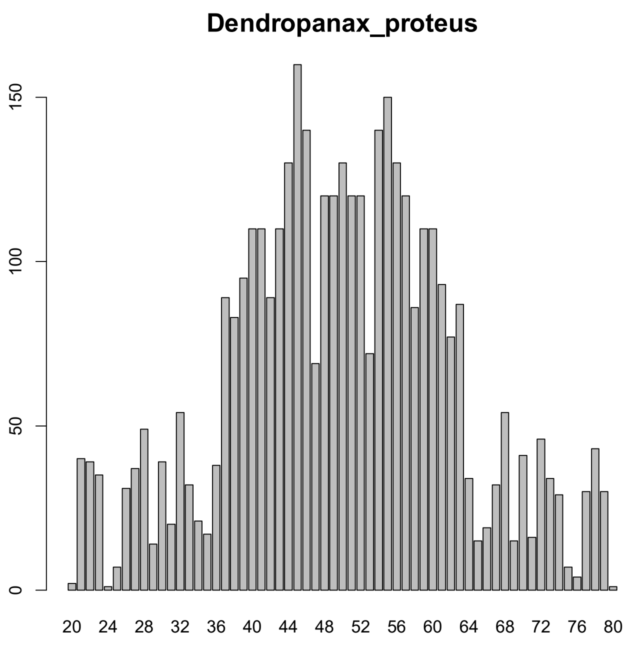
Dendropanax_proteus

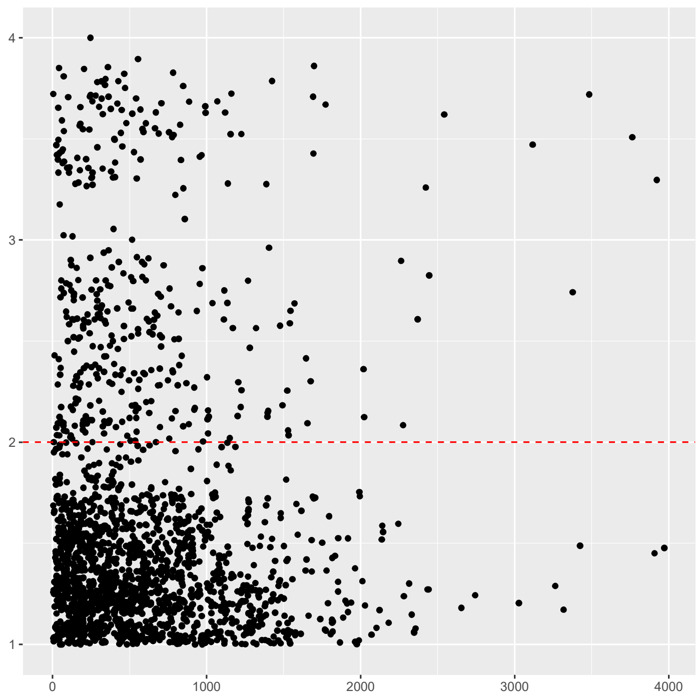

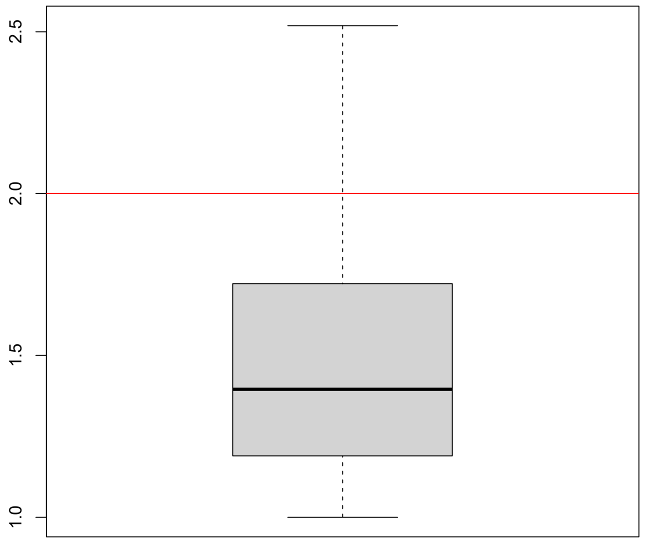

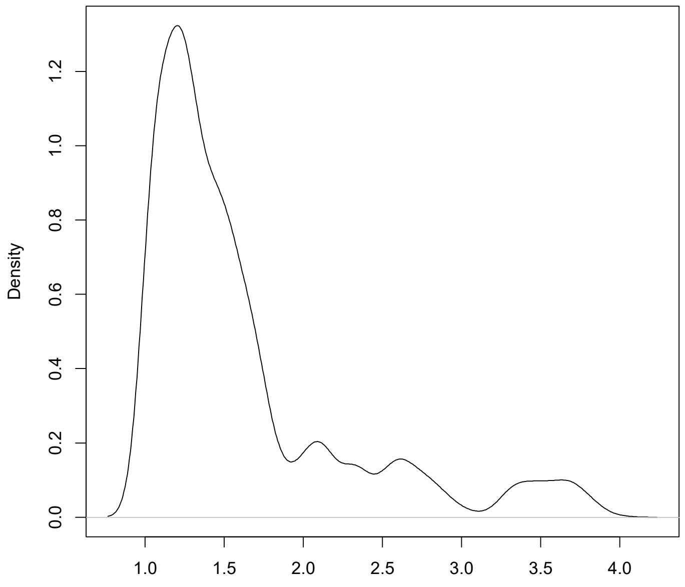
Density

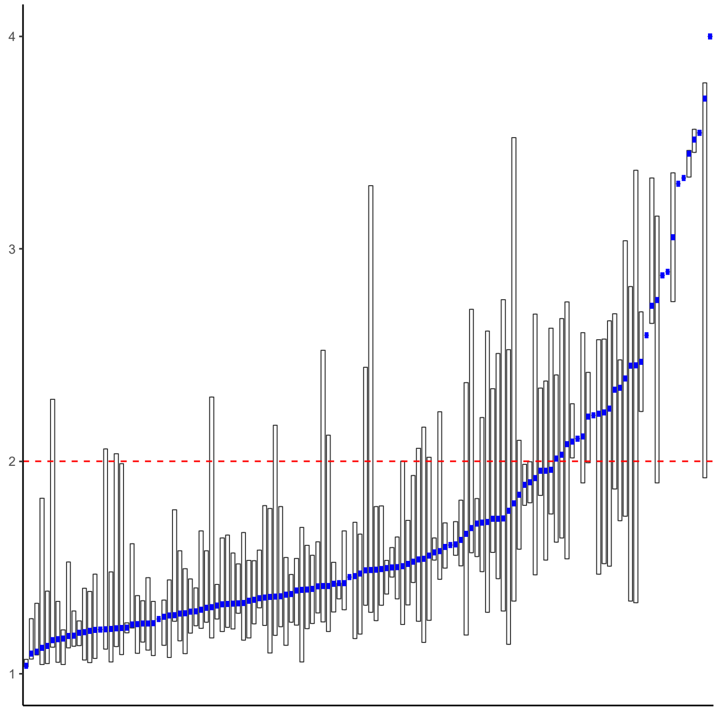

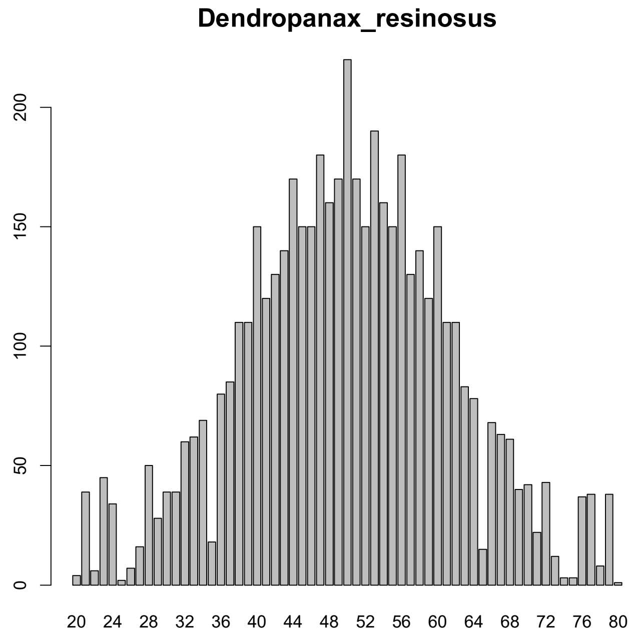
Dendropanax_resinosus

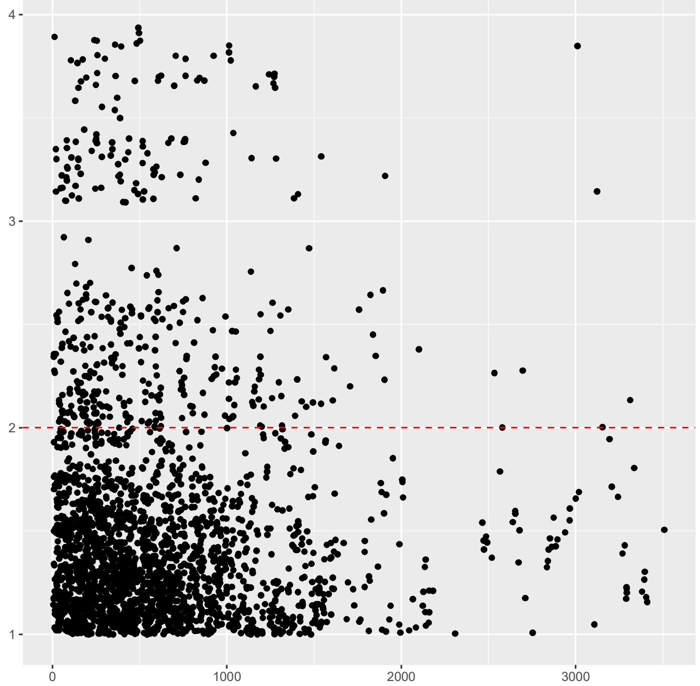

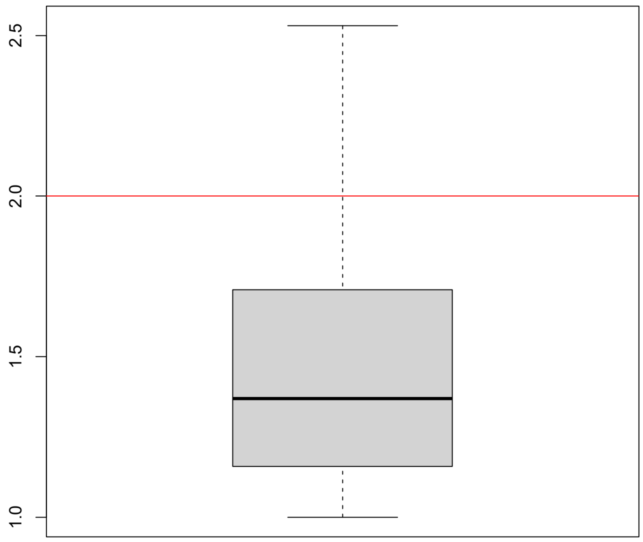

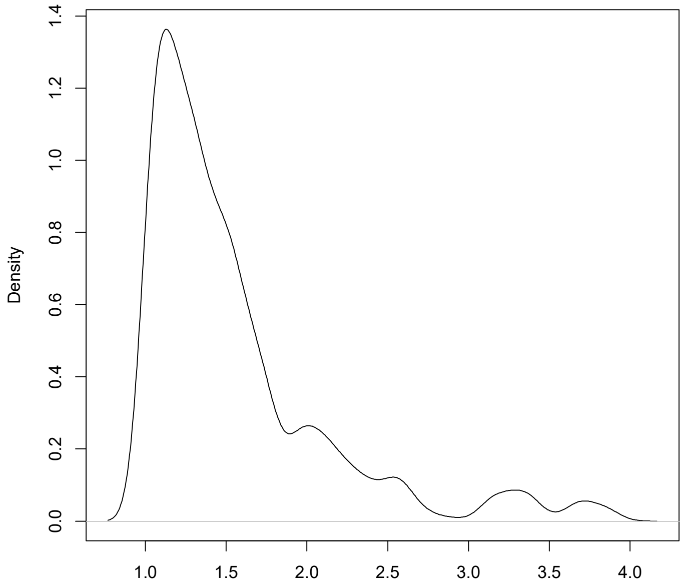
Density

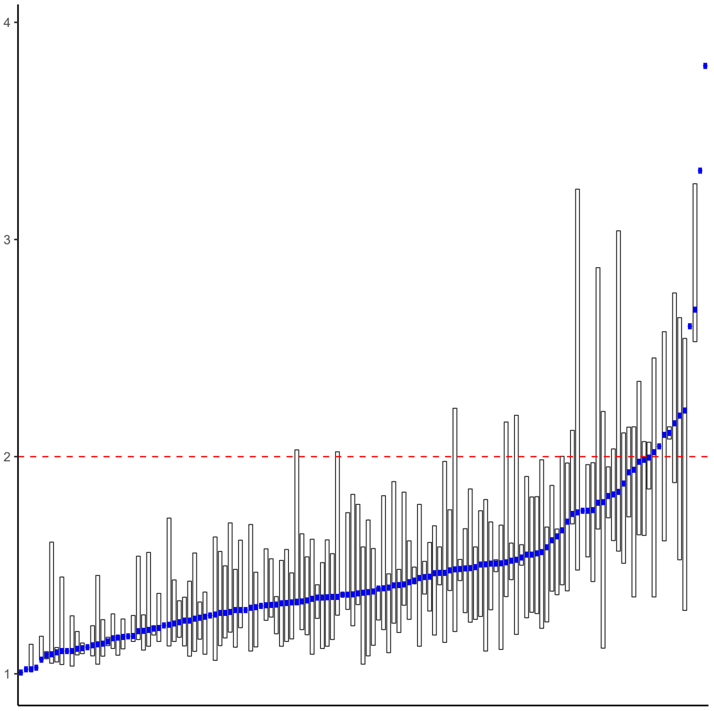

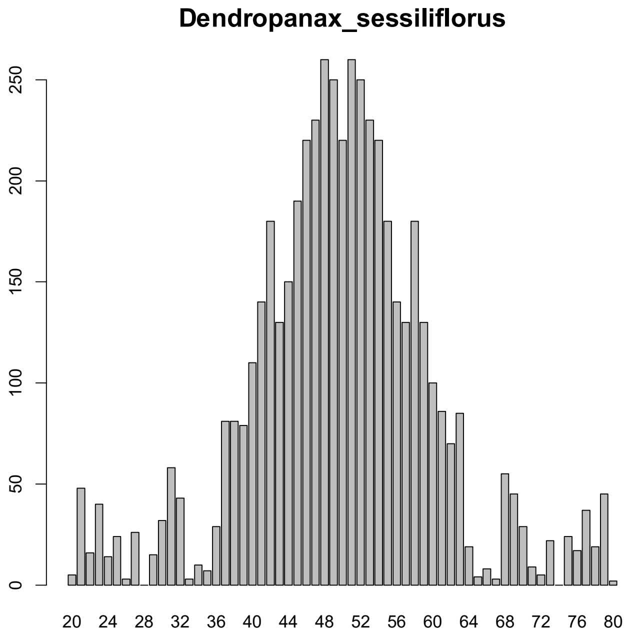
Dendropanax_sessiliflorus
0
50
100
150
200
250
20
24
28
32
36
40
44
48
52
56
60
64
68
72
76
80

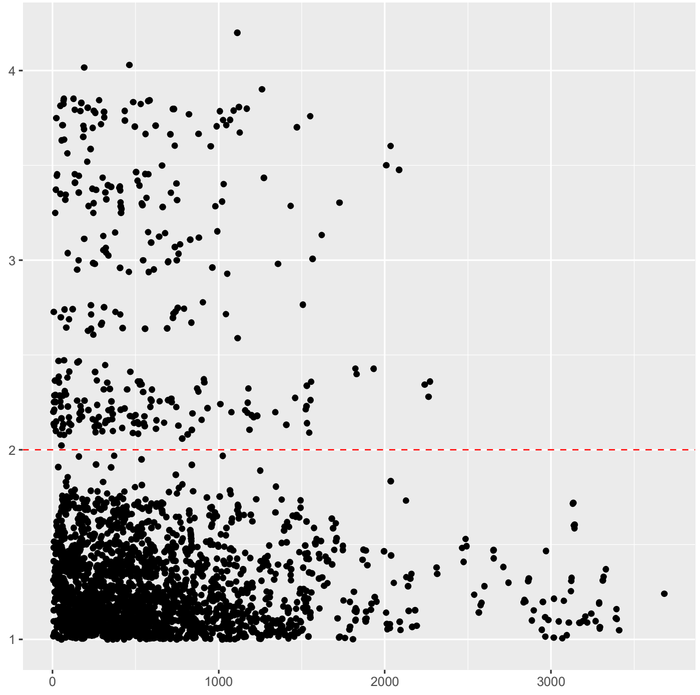
1
2
3
4
0
1000
2000
3000

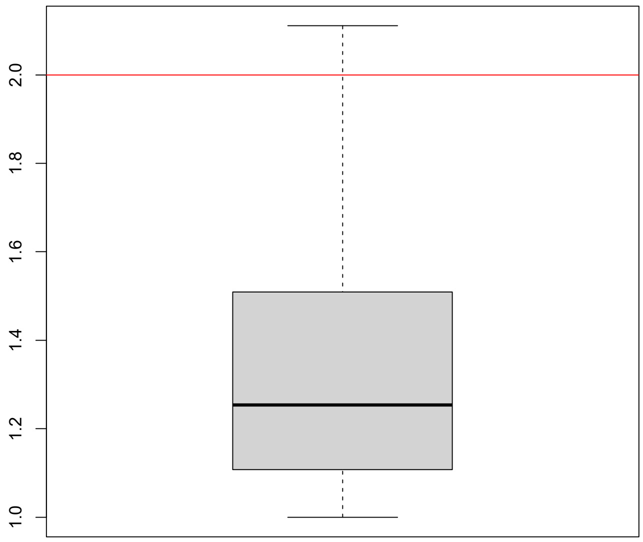
1.0
1.2
1.4
1.6
1.8
2.0

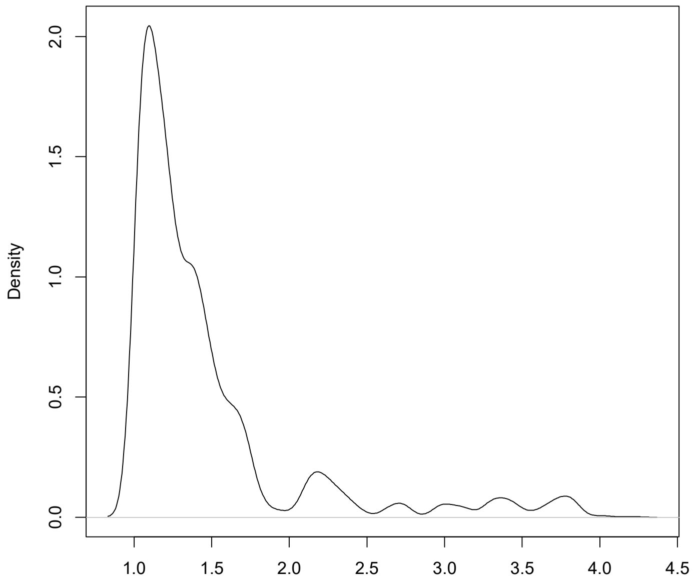
Density
0.0
0.5
1.0
1.5
2.0
1.0
1.5
2.0
2.5
3.0
3.5
4.0
4.5

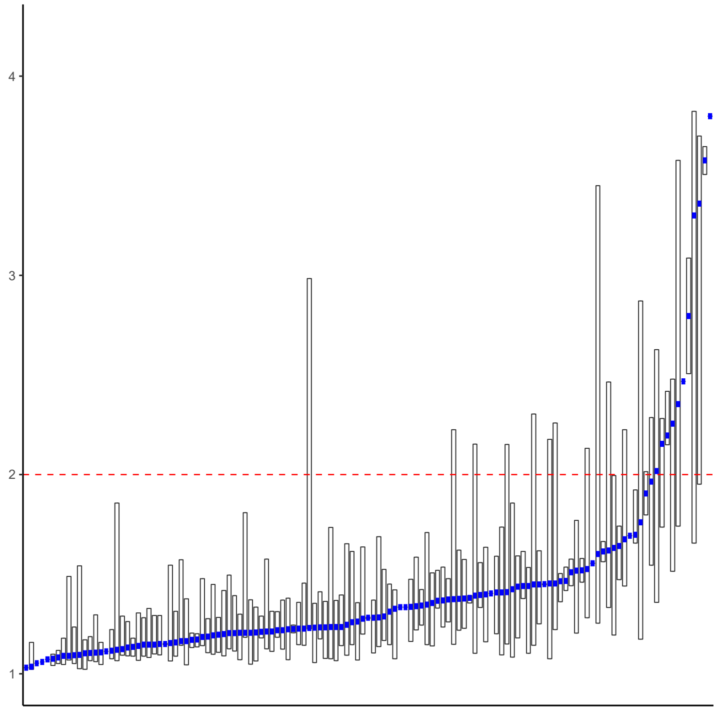
1
2
3
4

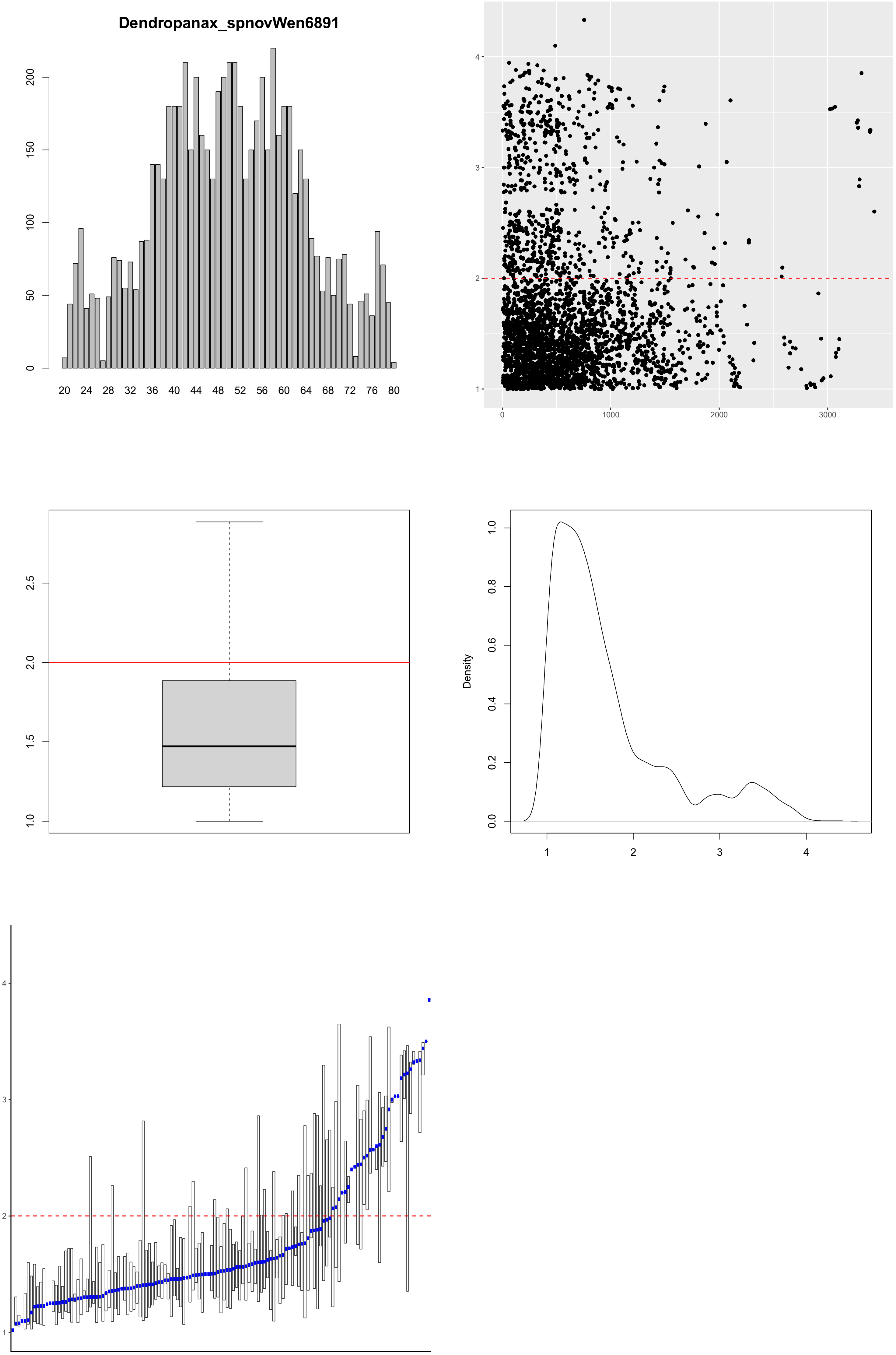
Dendropanax_spnovWen6891
Density

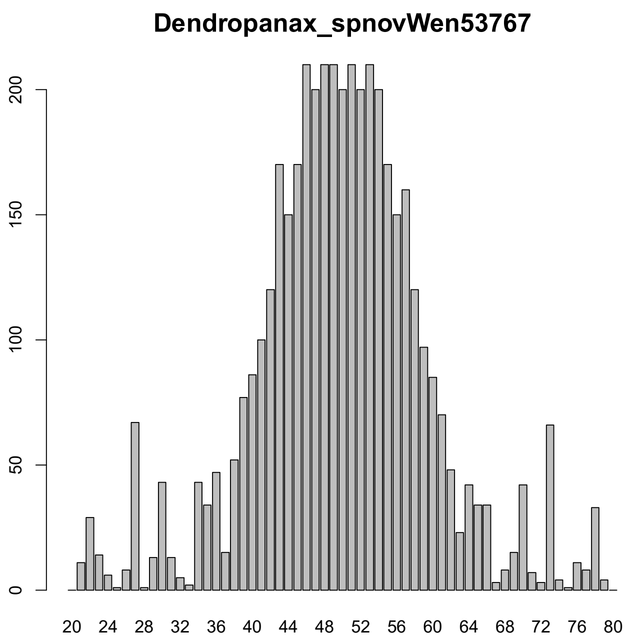
Dendropanax_spnovWen53767
0
50
100
150
200
20 24 28 32 36 40 44 48 52 56 60 64 68 72 76 80

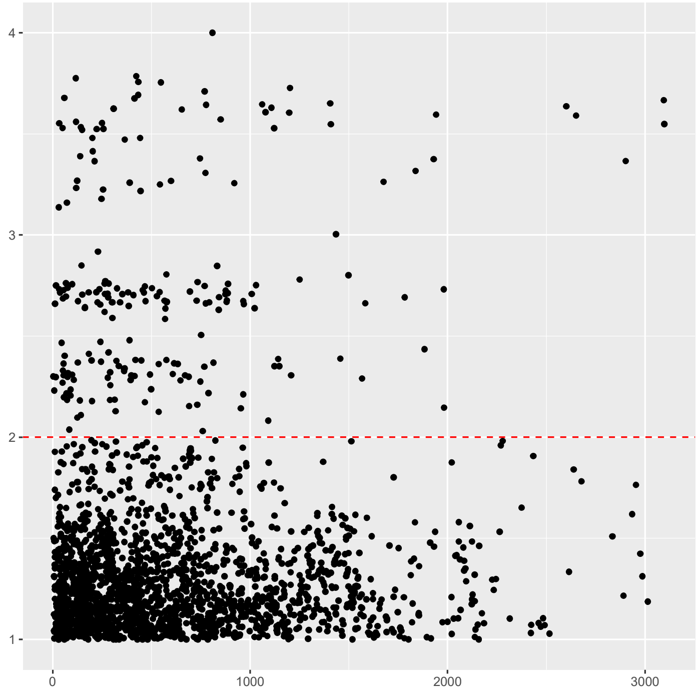
1
2
3
4
0
1000
2000
3000

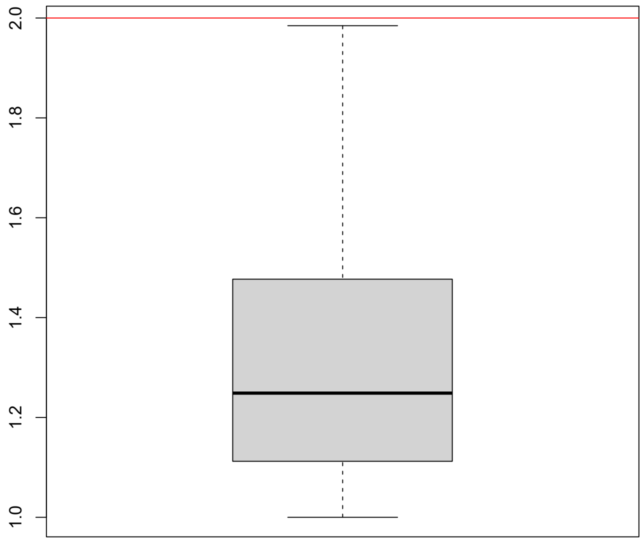
1.0
1.2
1.4
1.6
1.8
2.0

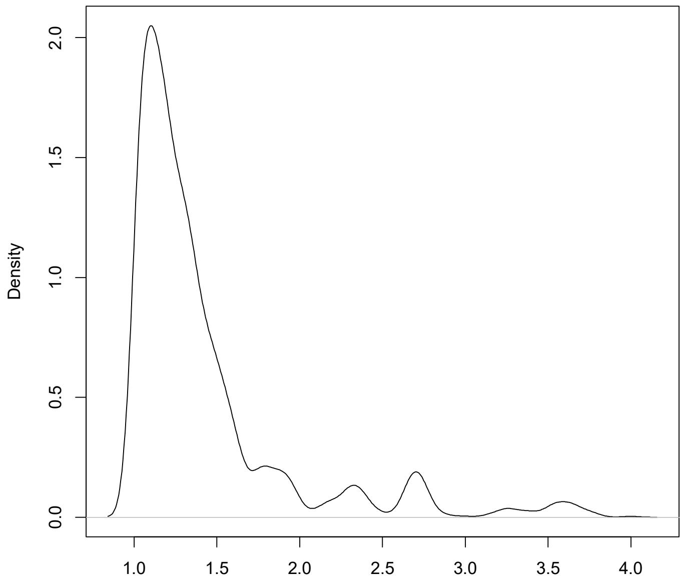
Density
0.0
0.5
1.0
1.5
2.0
1.0 1.5 2.0 2.5 3.0 3.5 4.0

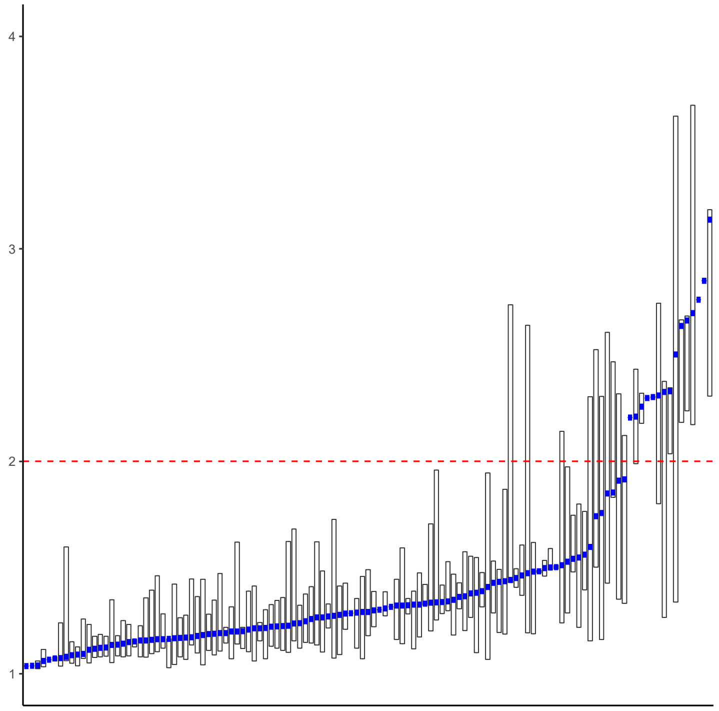
1
2
3
4

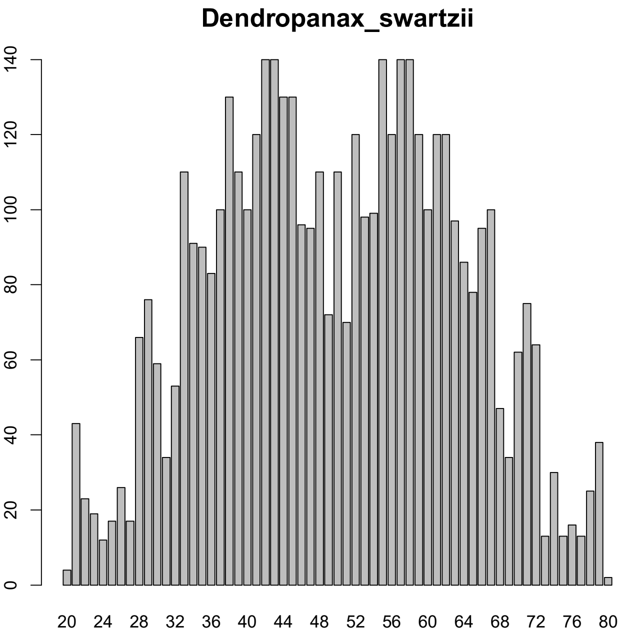
Dendropanax_swartzii
0
20
40
60
80
100
120
140
20 24 28 32 36 40 44 48 52 56 60 64 68 72 76 80

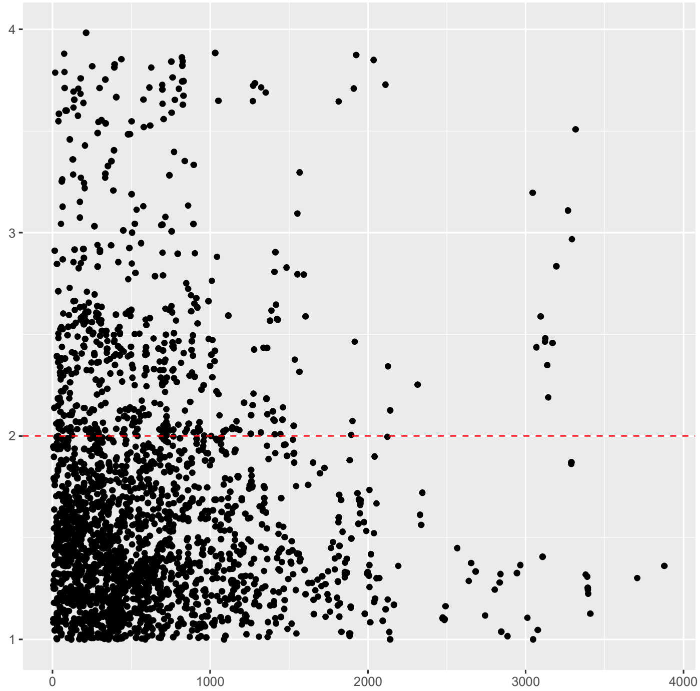
1
2
3
4
0 1000 2000 3000 4000

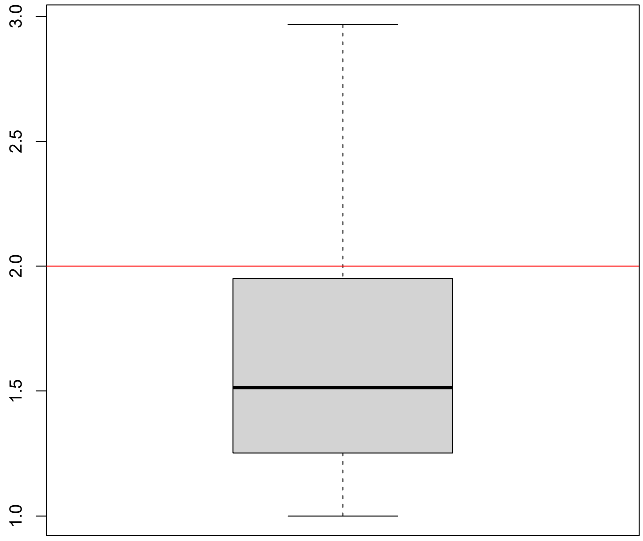
1.0
1.5
2.0
2.5
3.0

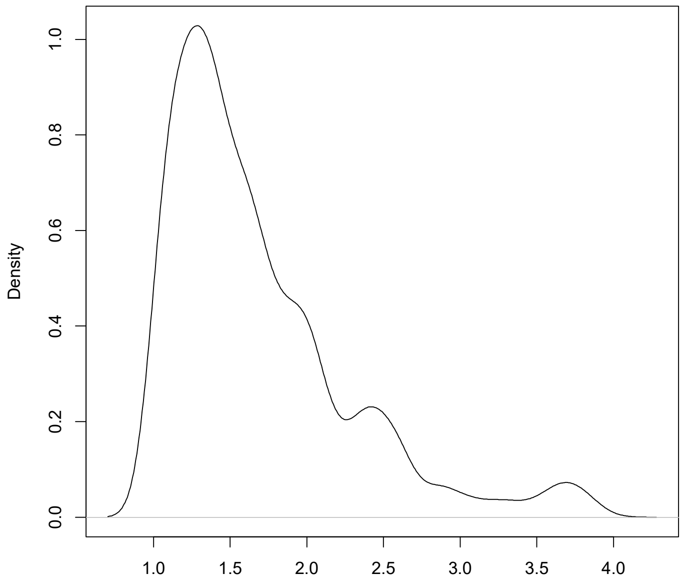
Density
0.0
0.2
0.4
0.6
0.8
1.0
1.0 1.5 2.0 2.5 3.0 3.5 4.0

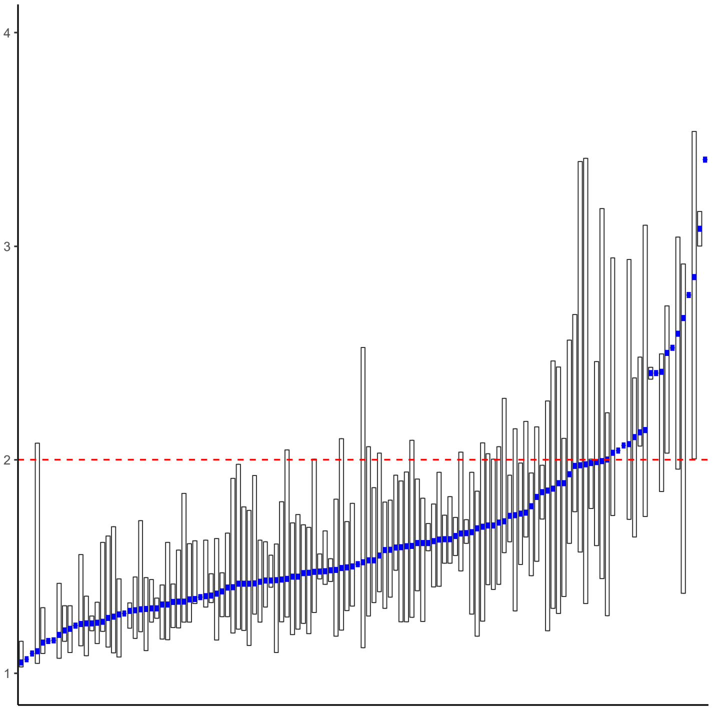
1
2
3
4

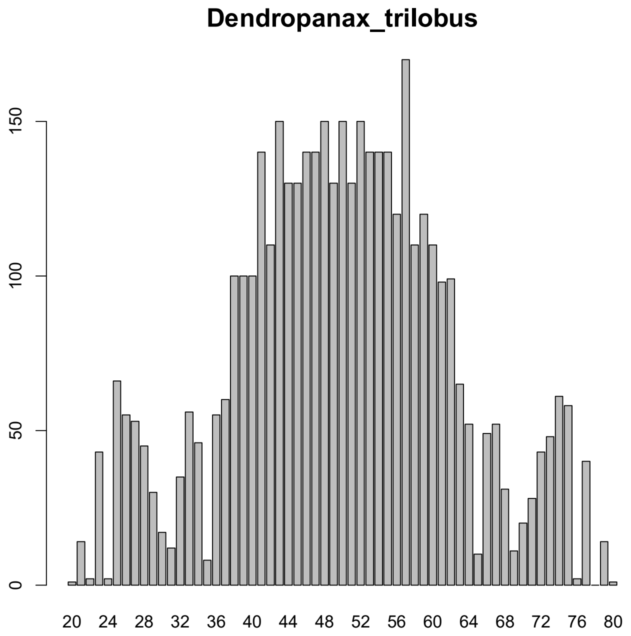
Dendropanax_trilobus
0
50
100
150
20 24 28 32 36 40 44 48 52 56 60 64 68 72 76 80

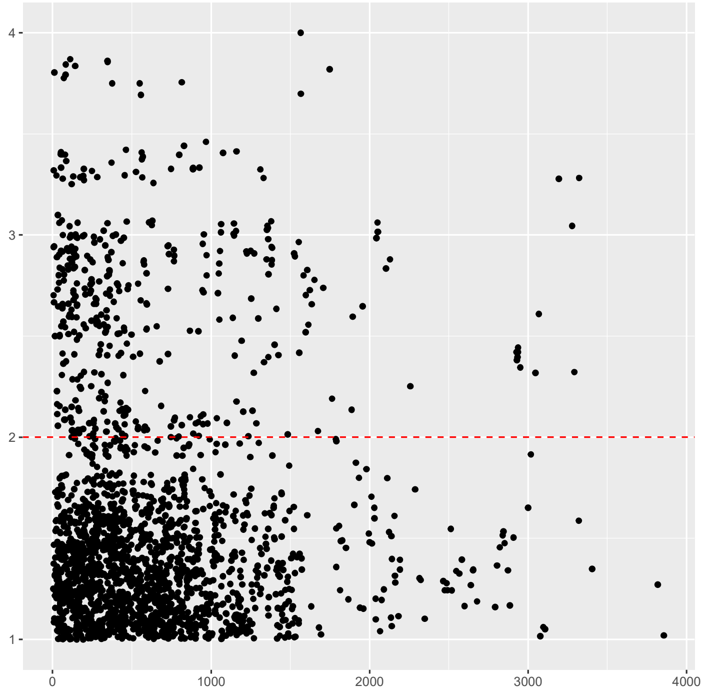
1
2
3
4
0 1000 2000 3000 4000

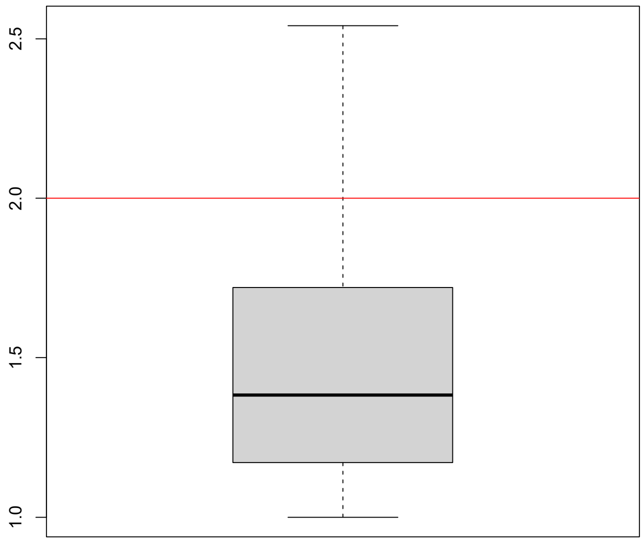
1.0
1.5
2.0
2.5

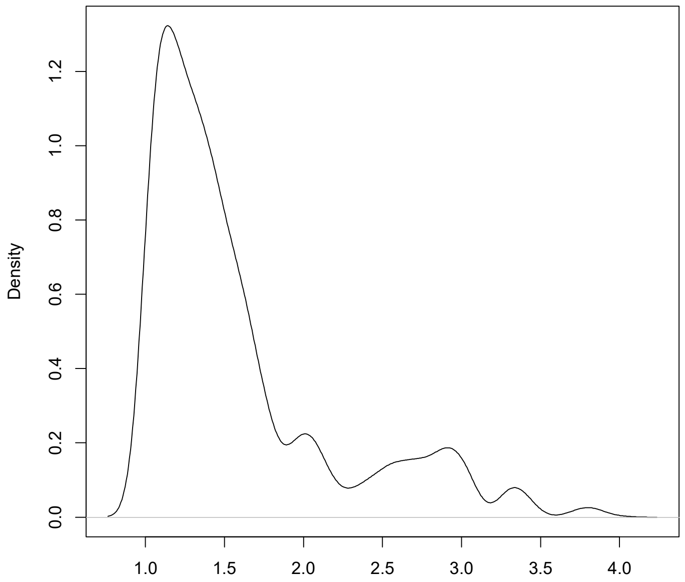
Density
0.0 0.2 0.4 0.6 0.8 1.0 1.2
1.0 1.5 2.0 2.5 3.0 3.5 4.0

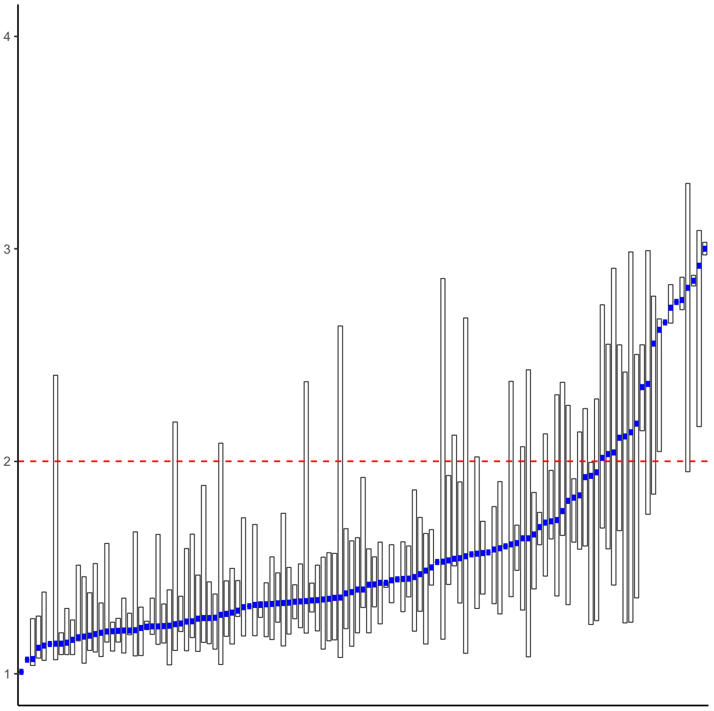
1
2
3
4

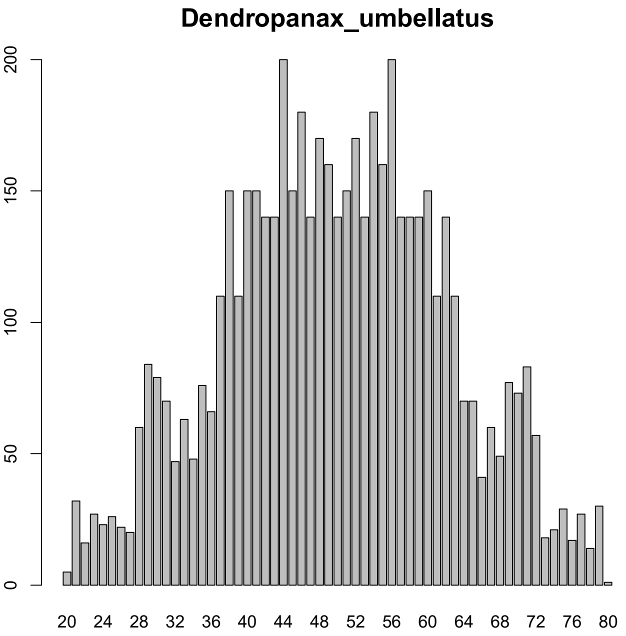
Dendropanax_umbellatus
0
50
100
150
200
20 24 28 32 36 40 44 48 52 56 60 64 68 72 76 80

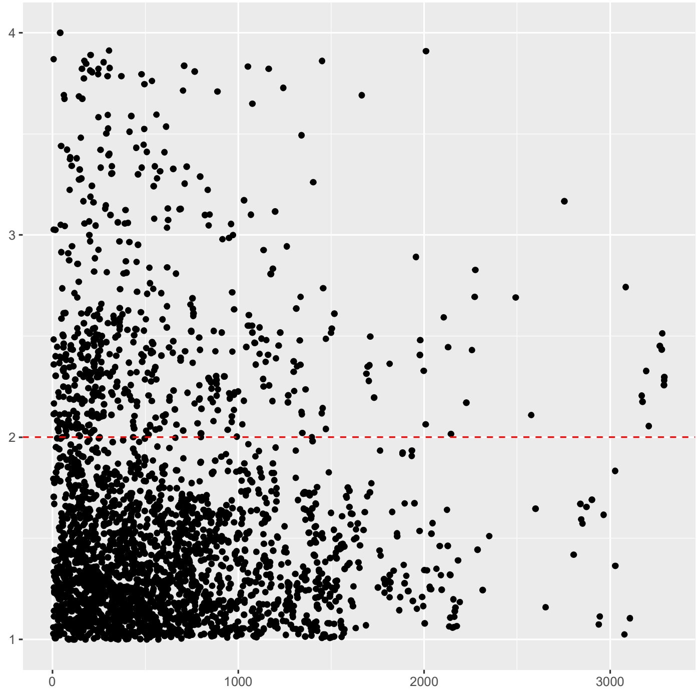
1
2
3
4
0 1000 2000 3000

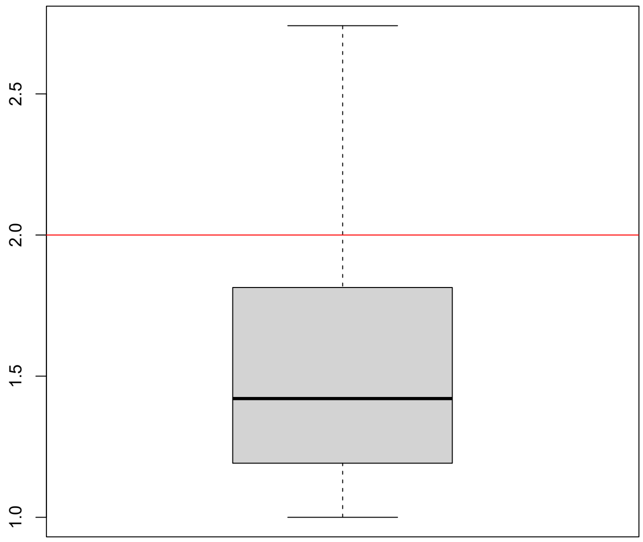
1.0
1.5
2.0
2.5

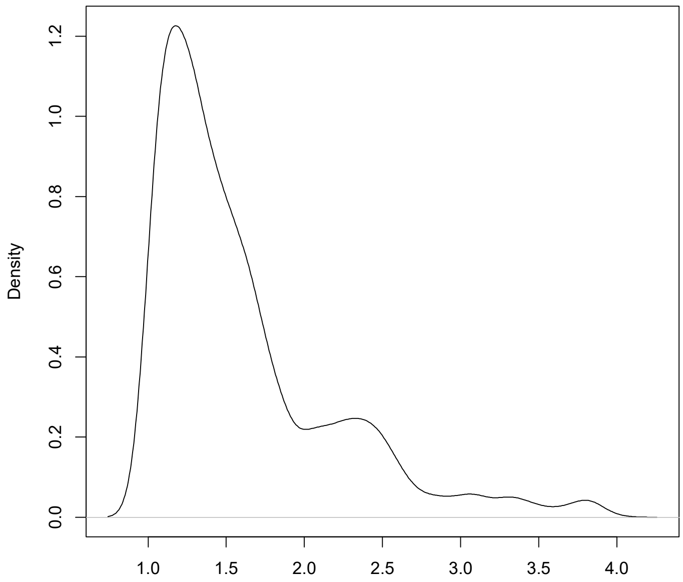
Density
0.0
0.2
0.4
0.6
0.8
1.0
1.2
1.0 1.5 2.0 2.5 3.0 3.5 4.0

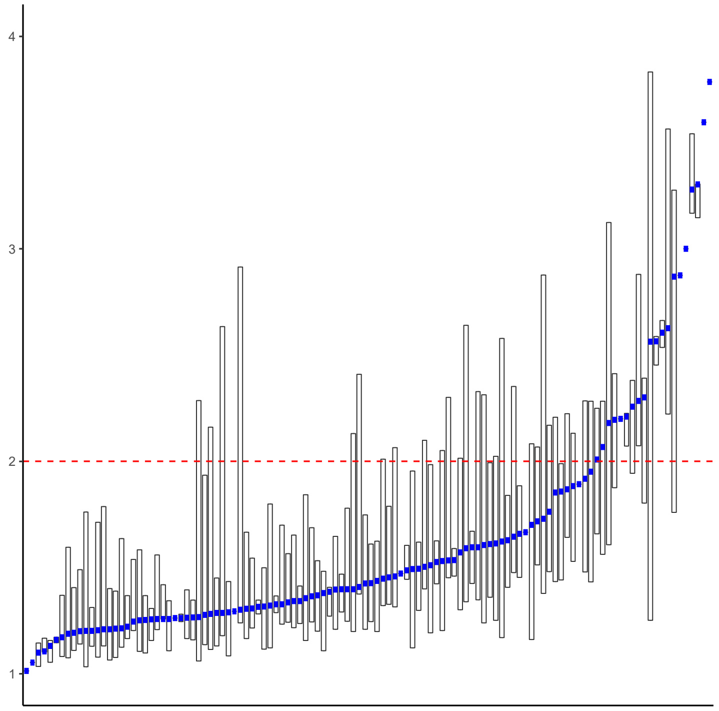
1
2
3
4

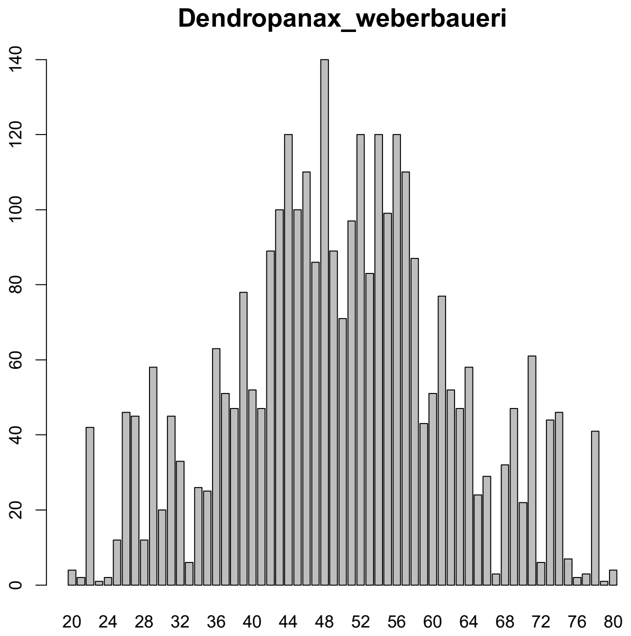
Dendropanax_weberbaueri

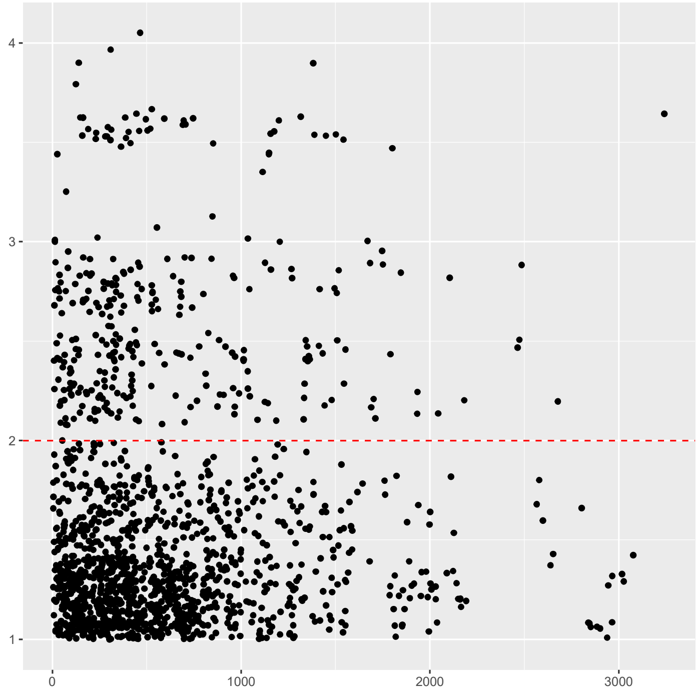

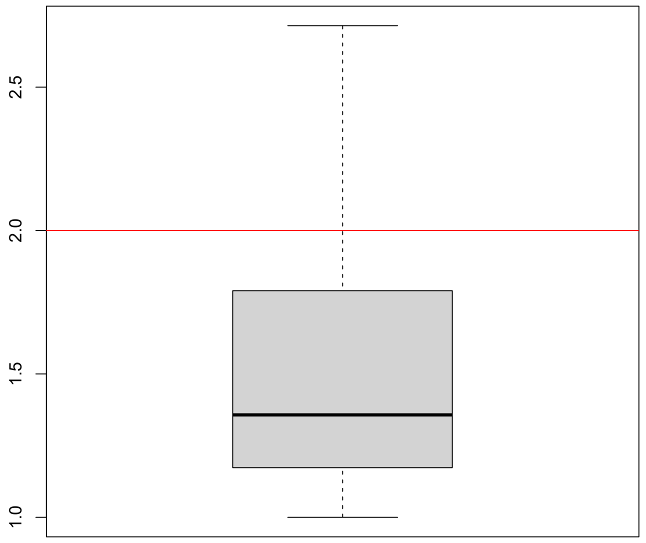

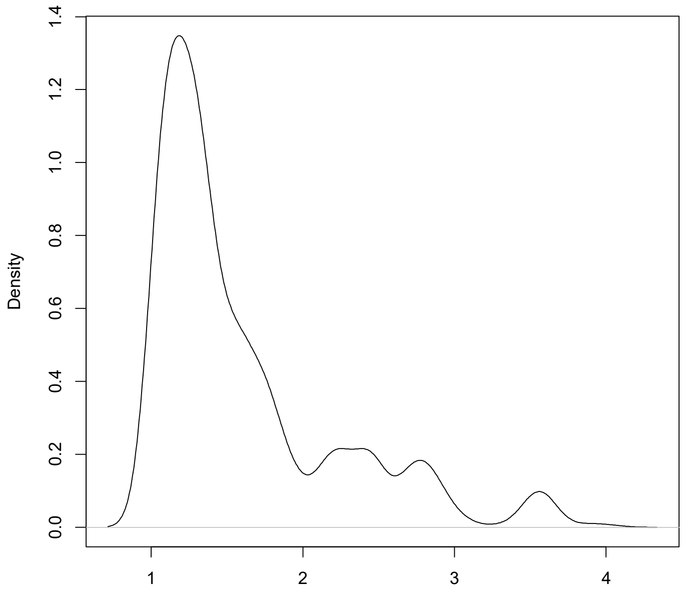
Density

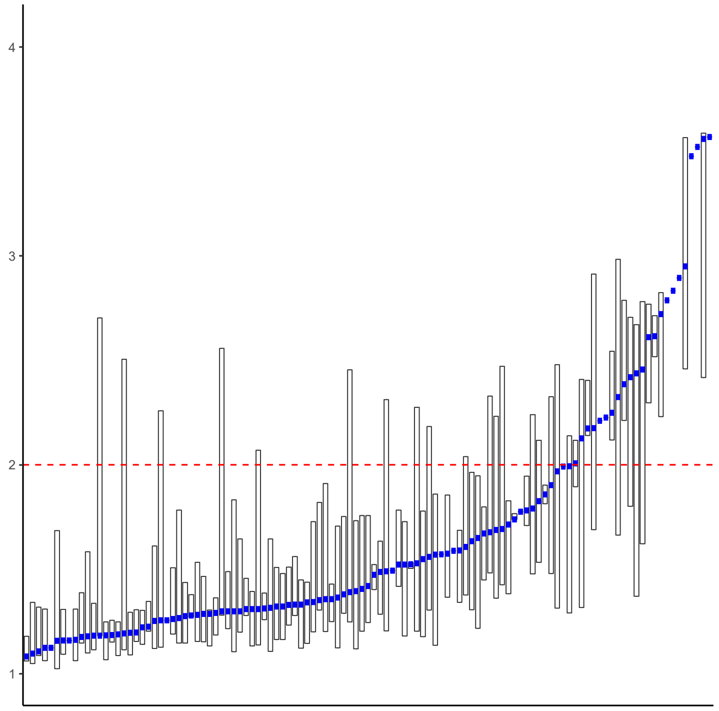

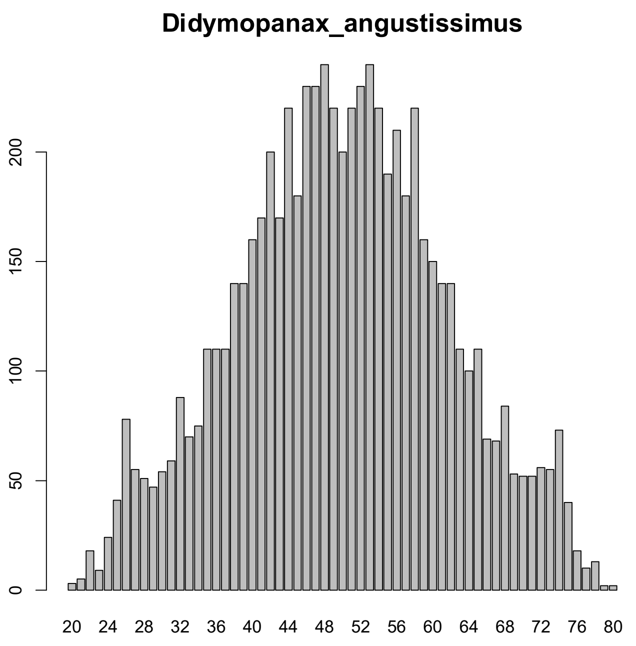
Didymopanax_angustissimus
0
50
100
150
200
20 24 28 32 36 40 44 48 52 56 60 64 68 72 76 80

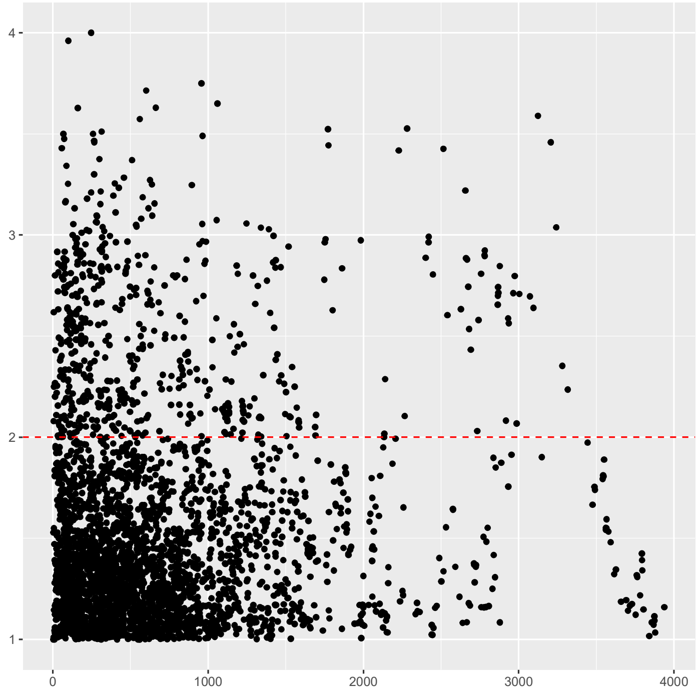
1
2
3
4
0
1000
2000
3000
4000

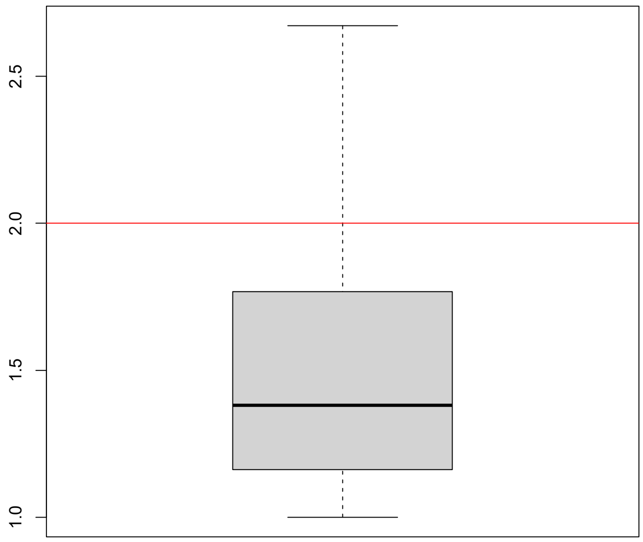
1.0
1.5
2.0
2.5

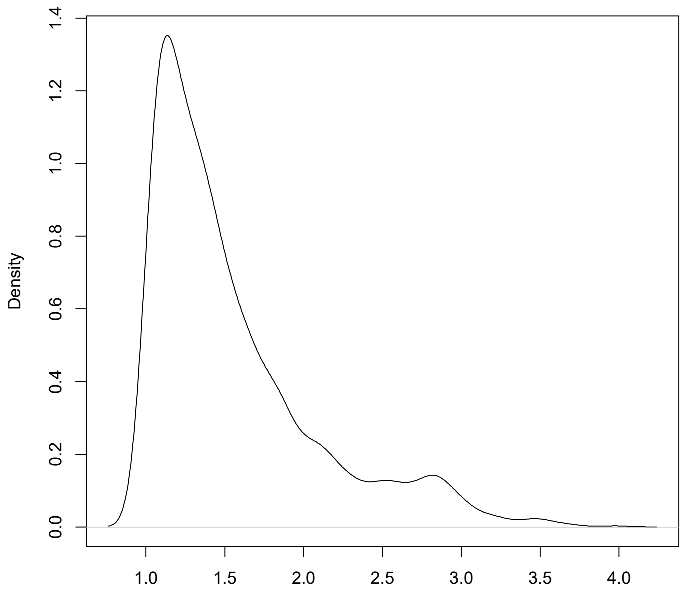
Density
0.0
0.2
0.4
0.6
0.8
1.0
1.2
1.4
1.0 1.5 2.0 2.5 3.0 3.5 4.0

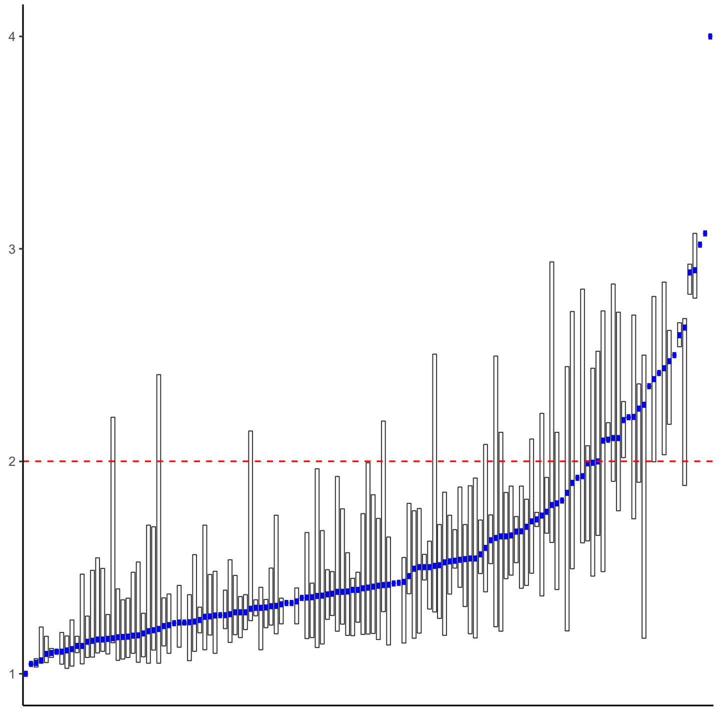
1
2
3
4

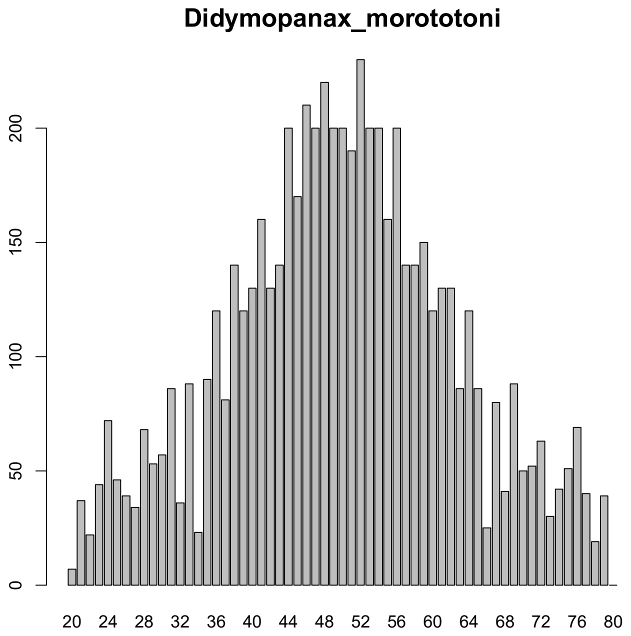
Didymopanax_morototoni
0
50
100
150
200
20 24 28 32 36 40 44 48 52 56 60 64 68 72 76 80

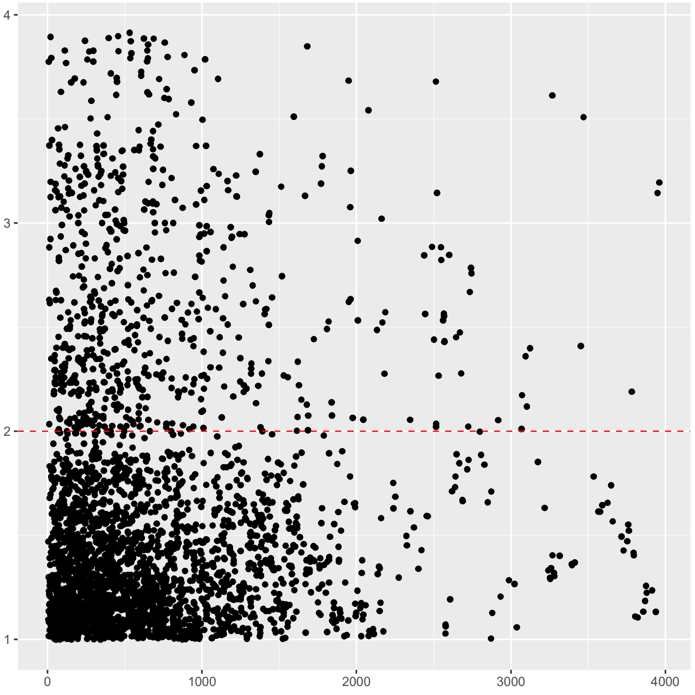
1
2
3
4
0 1000 2000 3000 4000

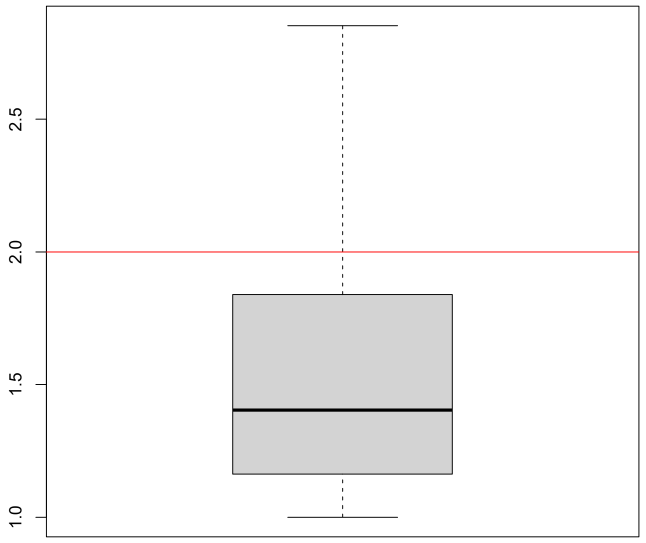
1.0
1.5
2.0
2.5

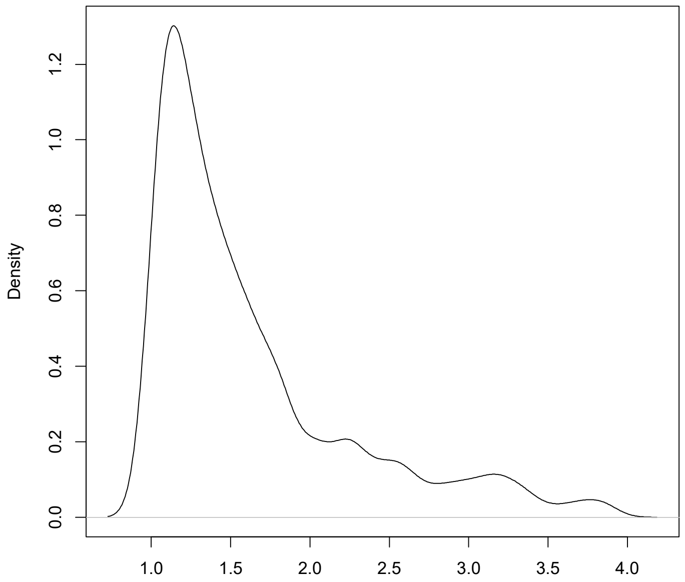
Density
0.0
0.2
0.4
0.6
0.8
1.0
1.2
1.0 1.5 2.0 2.5 3.0 3.5 4.0

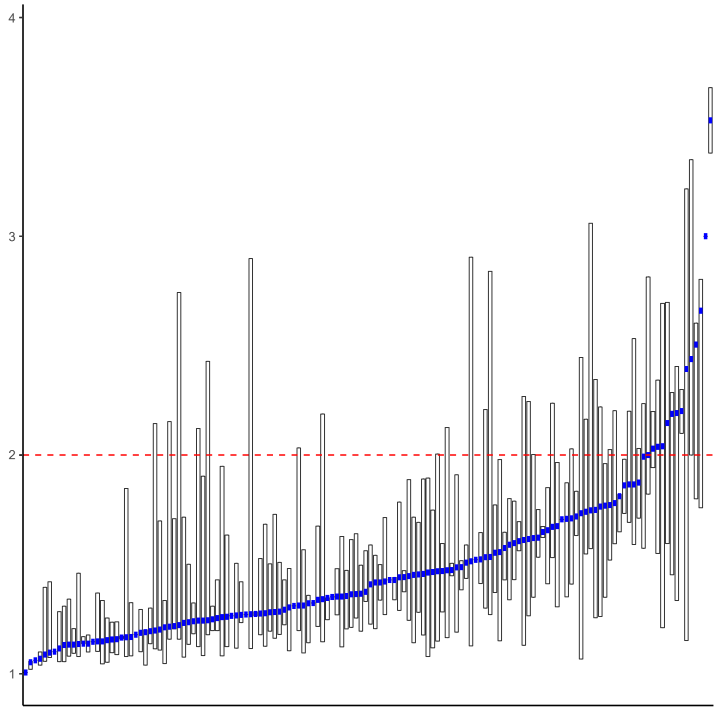
1
2
3
4

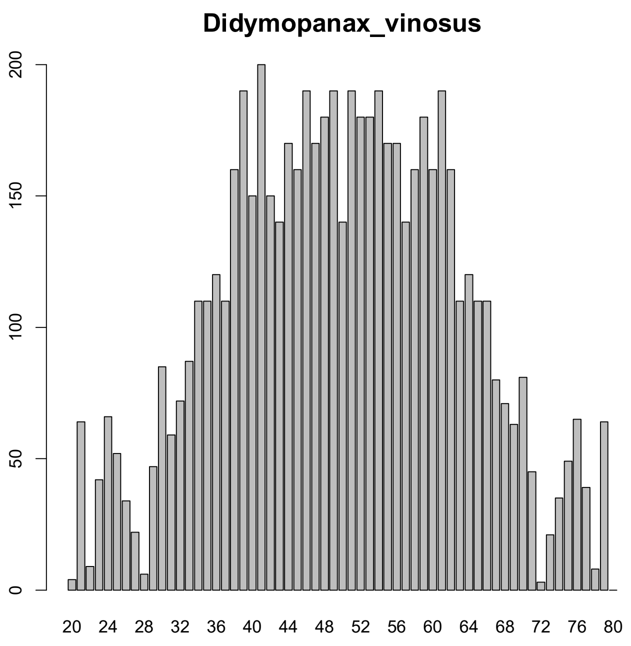
Didymopanax_vinosus
0
50
100
150
200
20 24 28 32 36 40 44 48 52 56 60 64 68 72 76 80

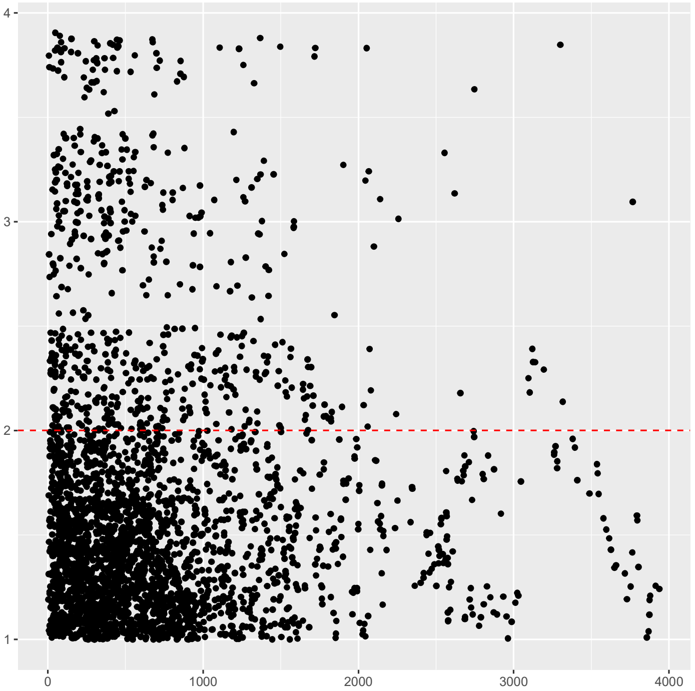
1
2
3
4
0 1000 2000 3000 4000

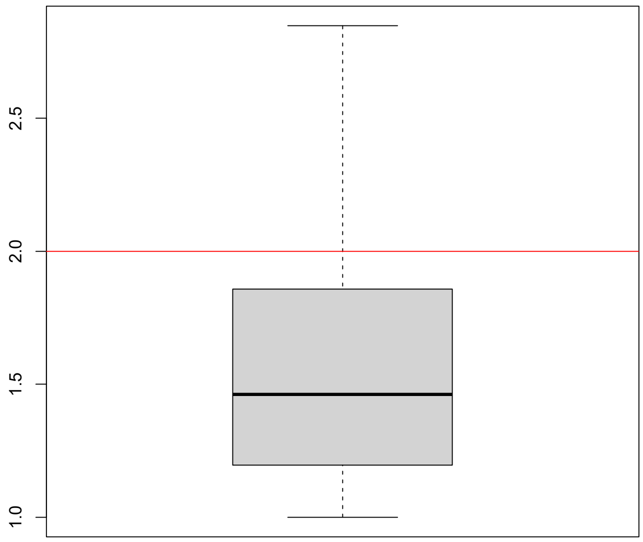
1.0
1.5
2.0
2.5

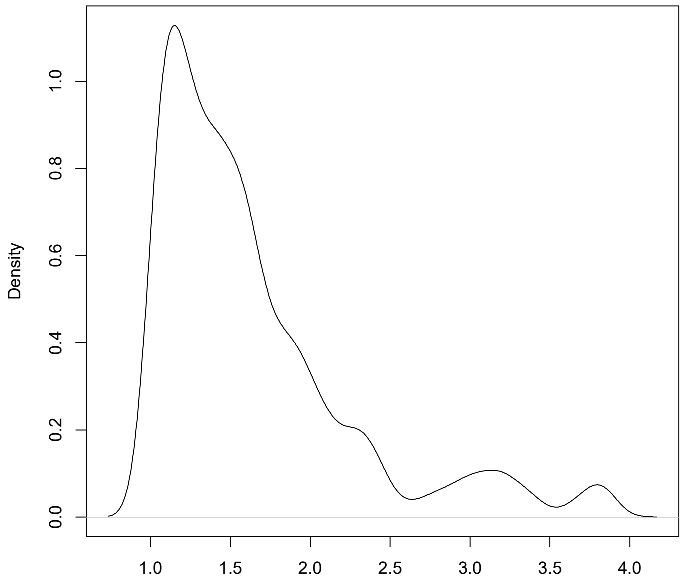
Density
0.0
0.2
0.4
0.6
0.8
1.0
1.0 1.5 2.0 2.5 3.0 3.5 4.0

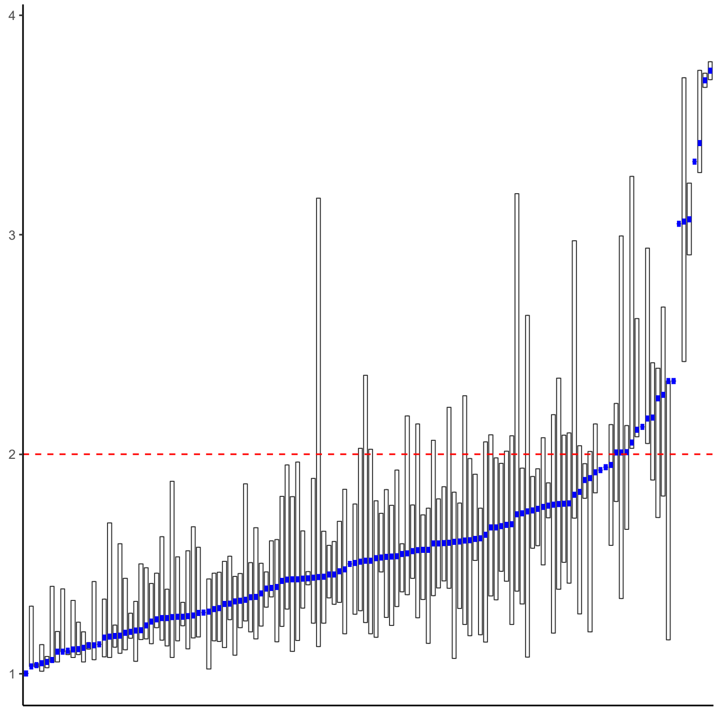
1
2
3
4

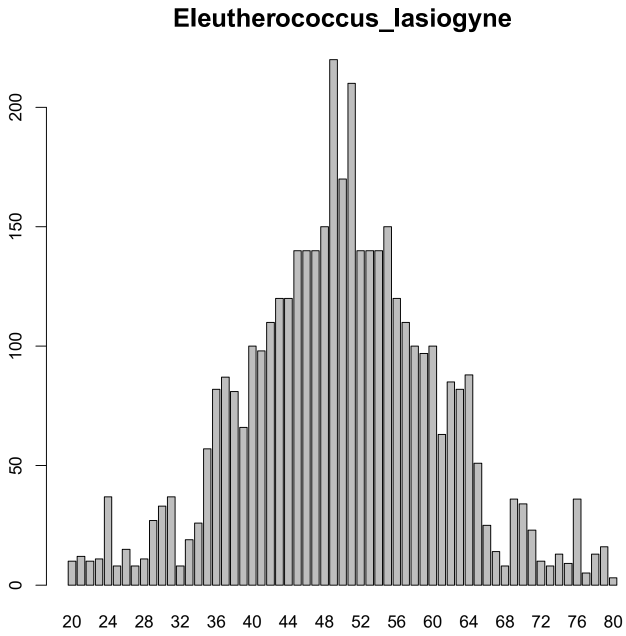
Eleutherococcus_lasiogyne
0
50
100
150
200
20 24 28 32 36 40 44 48 52 56 60 64 68 72 76 80

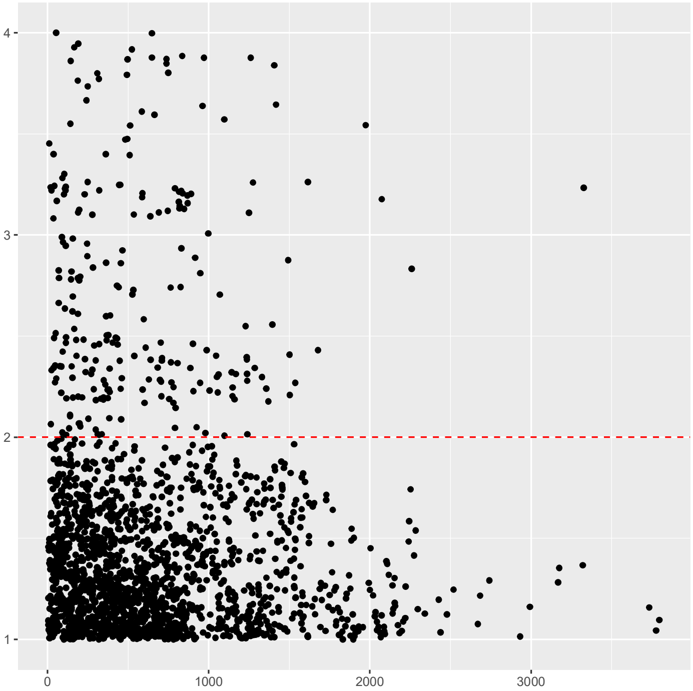
1
2
3
4
0 1000 2000 3000

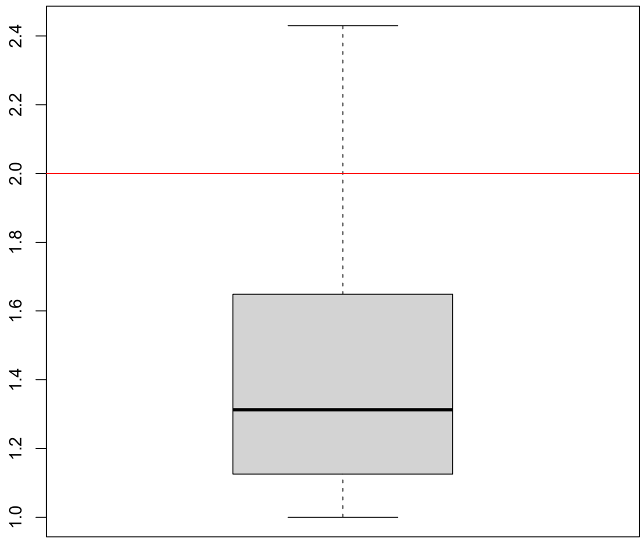
1.0
1.2
1.4
1.6
1.8
2.0
2.2
2.4

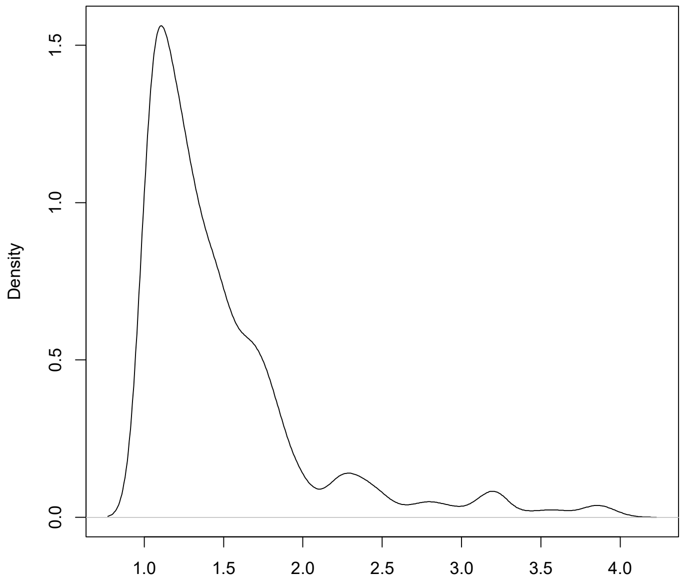
Density
0.0
0.5
1.0
1.5
1.0 1.5 2.0 2.5 3.0 3.5 4.0

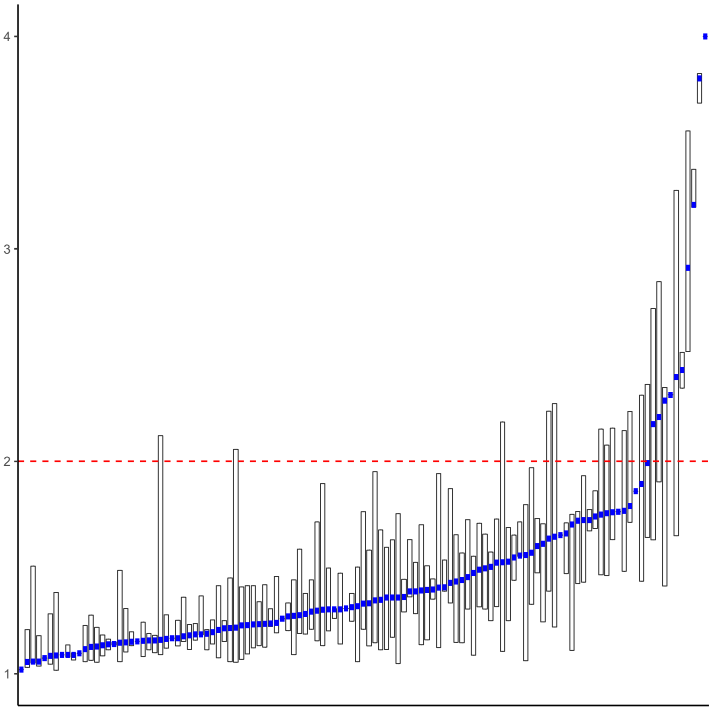
1
2
3
4

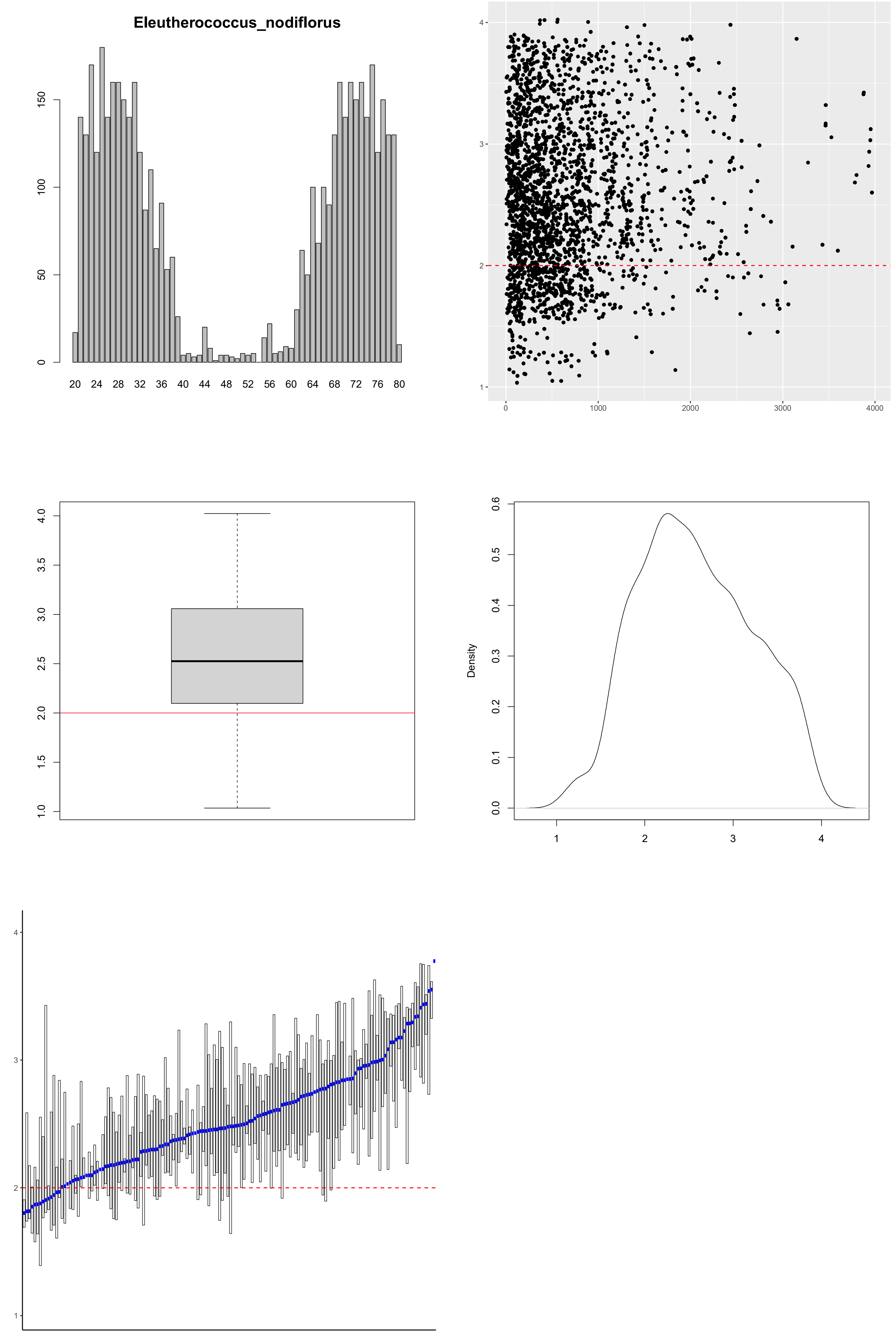

Eleutherococcus_nodiflorus
0
50
100
150
20
24
28
32
36
40
44
48
52
56
60
64
68
72
76
80
1
2
3
4
0
1000
2000
3000
4000
1.0
1.5
2.0
2.5
3.0
3.5
4.0
Density
0.0
0.1
0.2
0.3
0.4
0.5
0.6
1
2
3
4

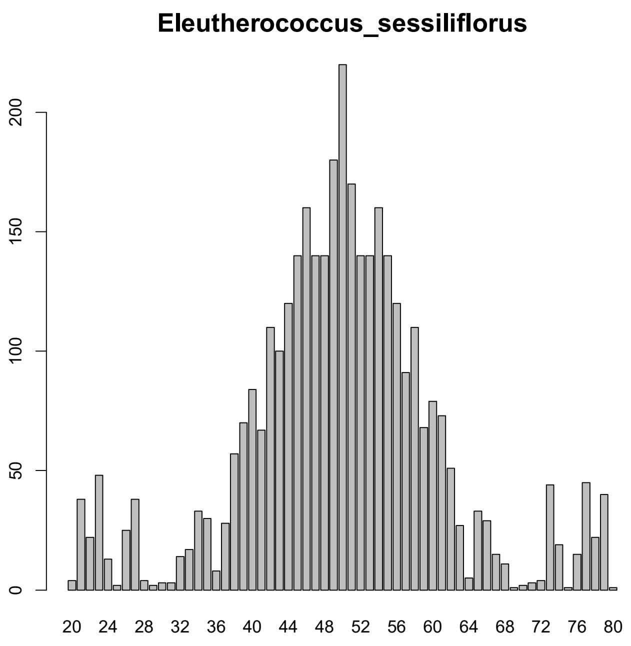
Eleutherococcus_sessiliflorus

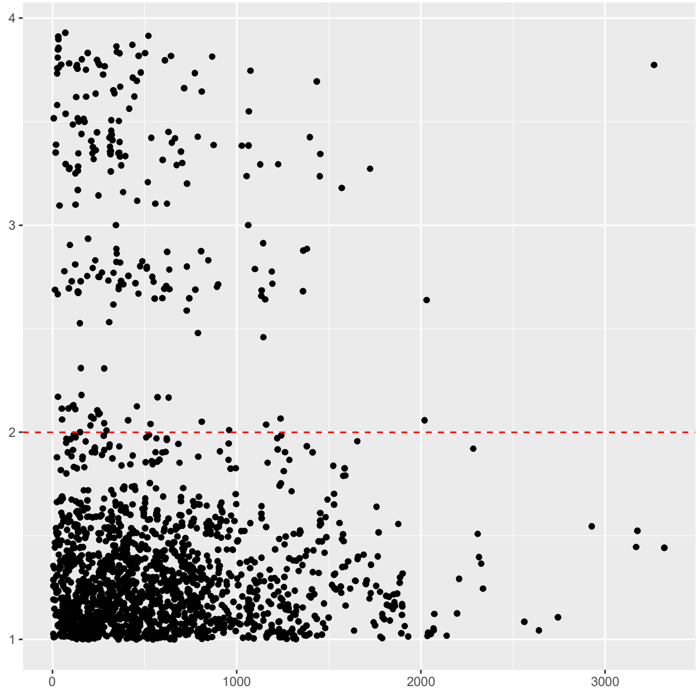

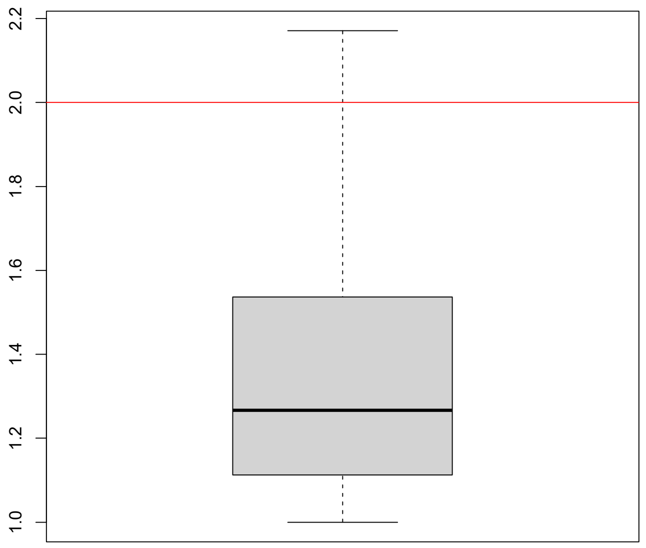

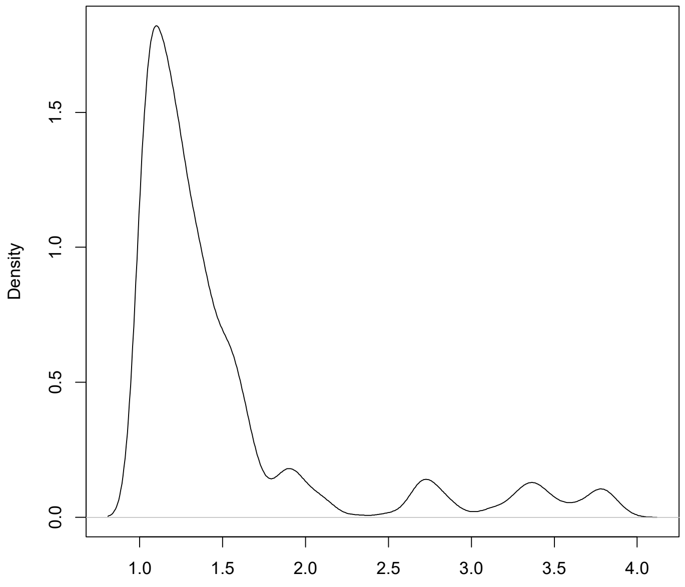
Density

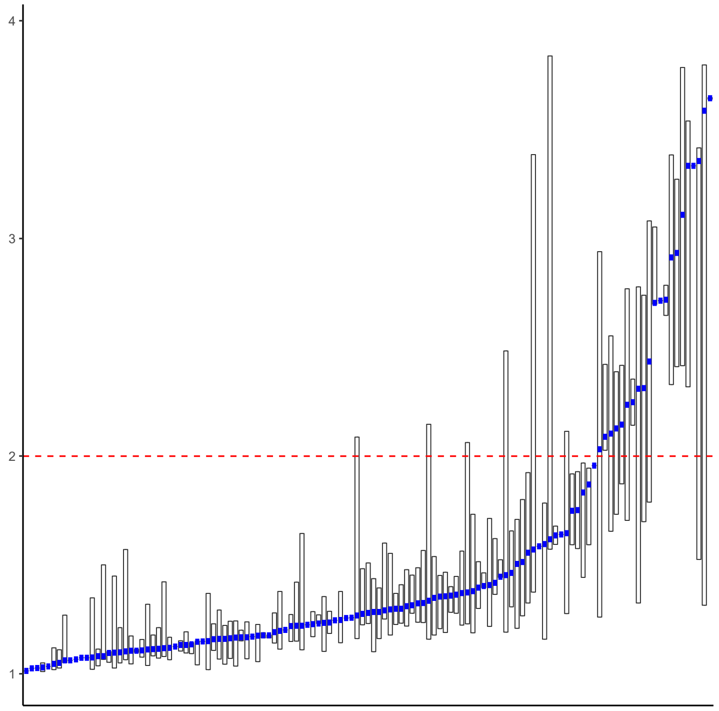

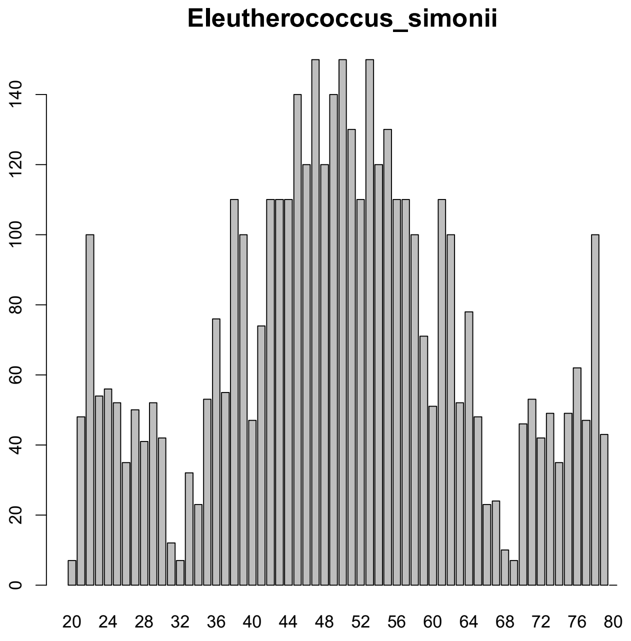
Eleutherococcus_simonii
0 20 40 60 80 100 120 140
20 24 28 32 36 40 44 48 52 56 60 64 68 72 76 80
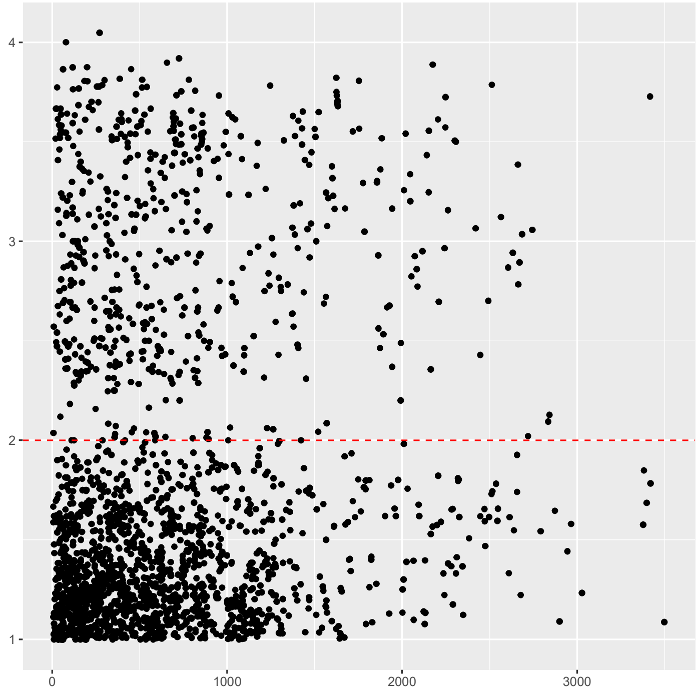
1 2 3 4
0 1000 2000 3000
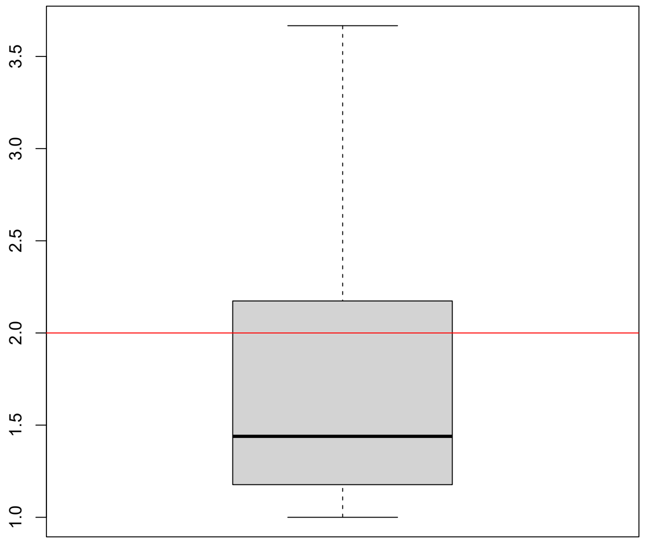
1.0 1.5 2.0 2.5 3.0 3.5
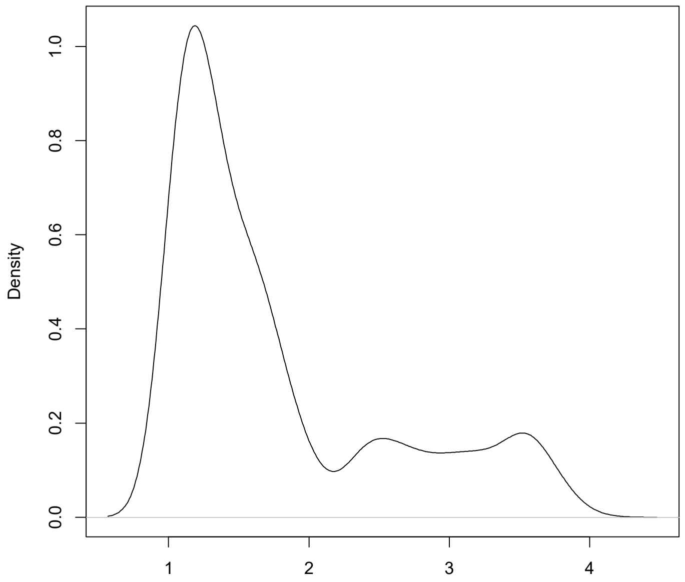
Density
0.0 0.2 0.4 0.6 0.8 1.0
1 2 3 4
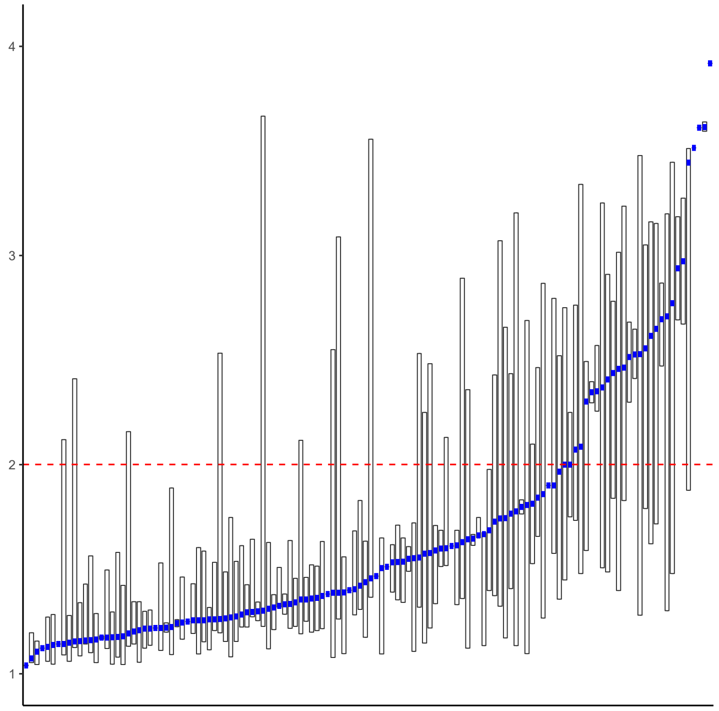
1 2 3 4

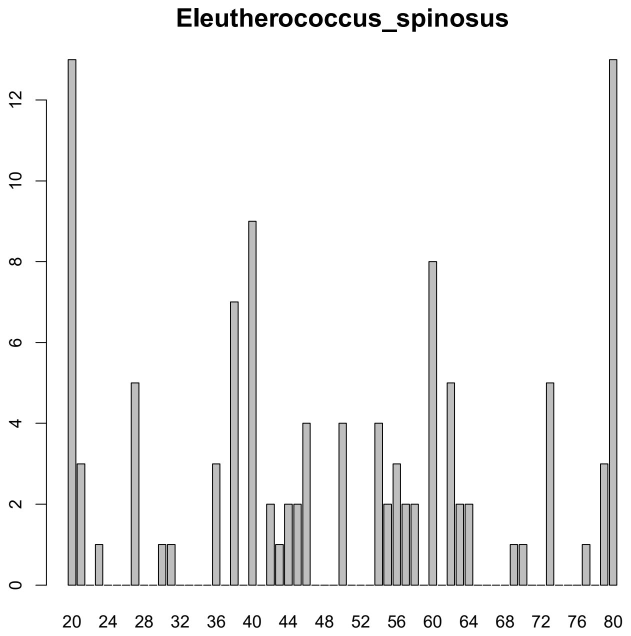
Eleutherococcus_spinosus
0 2 4 6 8 10 12
20 24 28 32 36 40 44 48 52 56 60 64 68 72 76 80

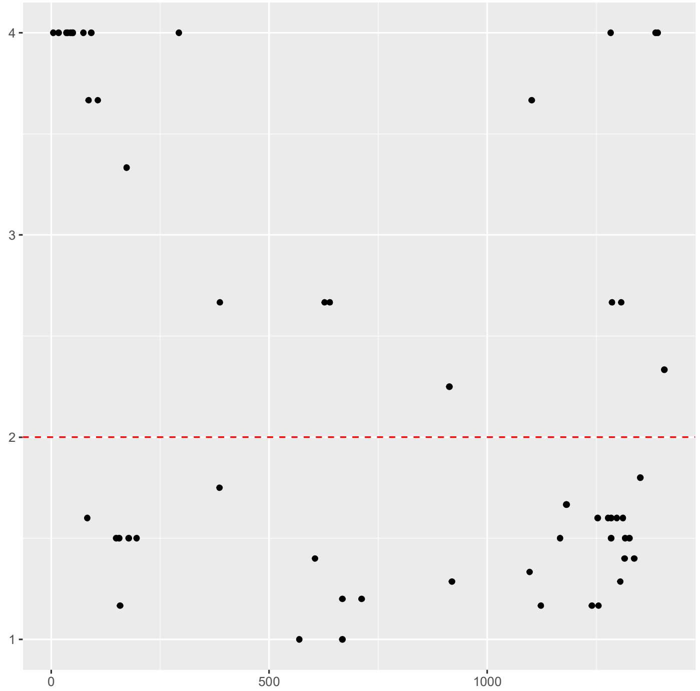
1 2 3 4
0 500 1000

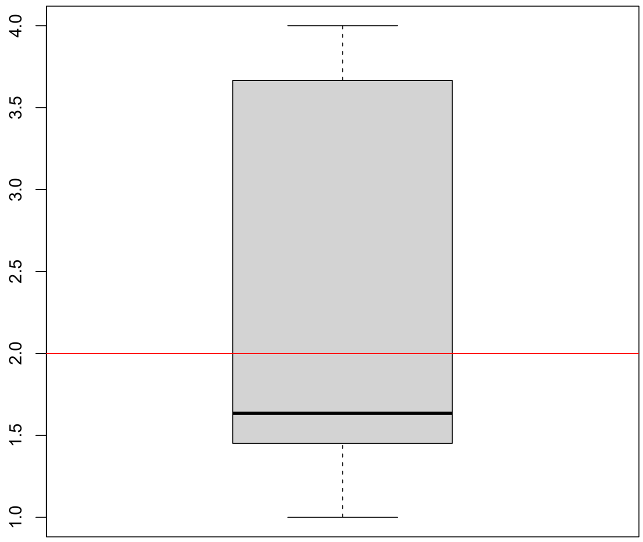
1.0 1.5 2.0 2.5 3.0 3.5 4.0

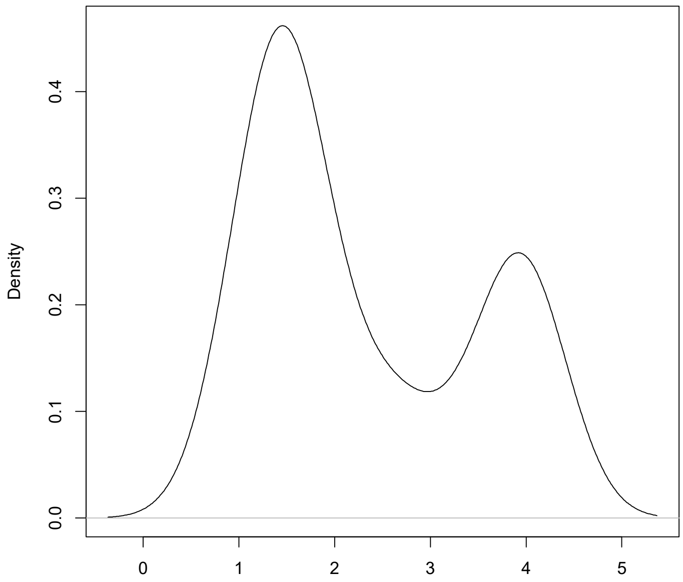
Density
0.0 0.1 0.2 0.3 0.4
0 1 2 3 4 5

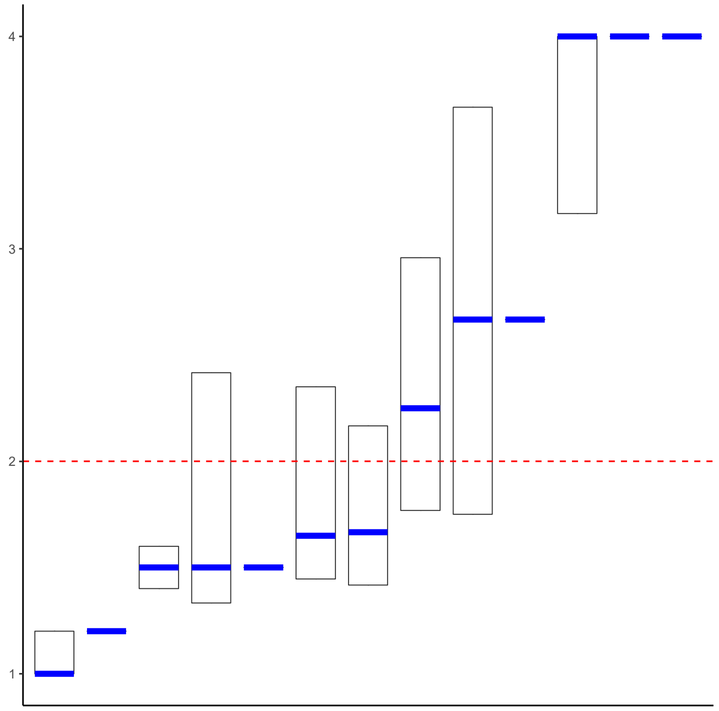
1 2 3 4

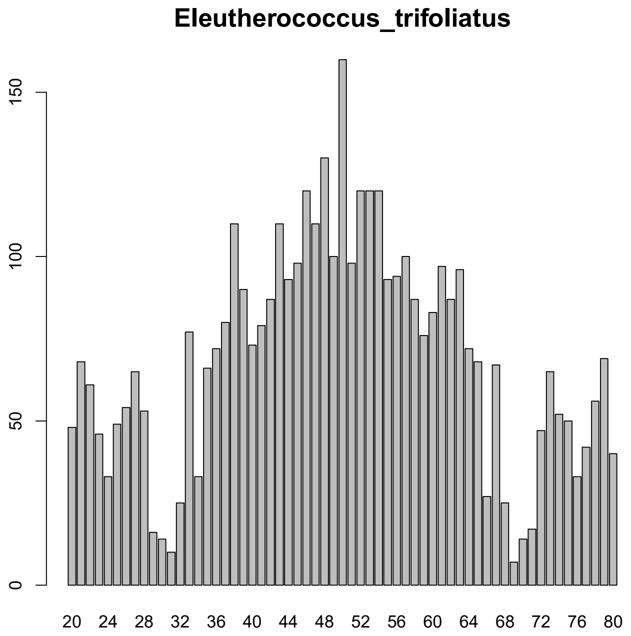
Eleutherococcus_trifoliatus
0
50
100
150
20 24 28 32 36 40 44 48 52 56 60 64 68 72 76 80

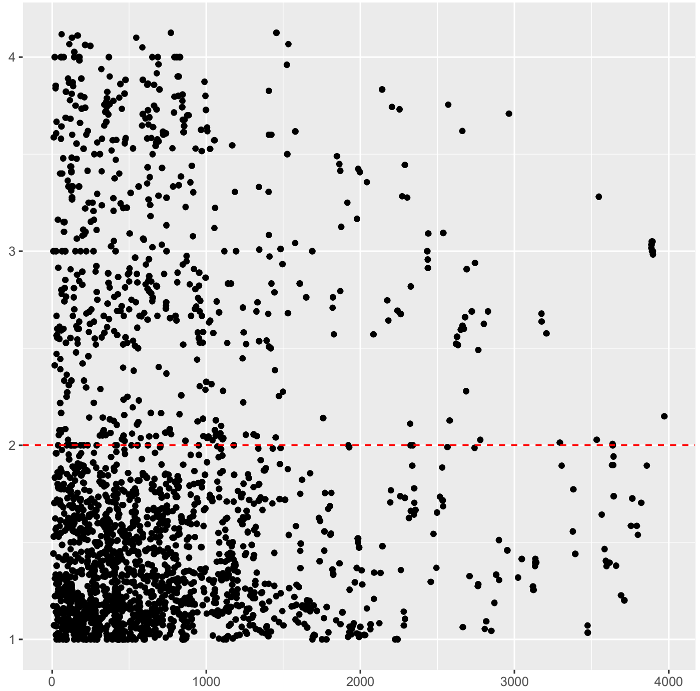
1
2
3
4
0 1000 2000 3000 4000

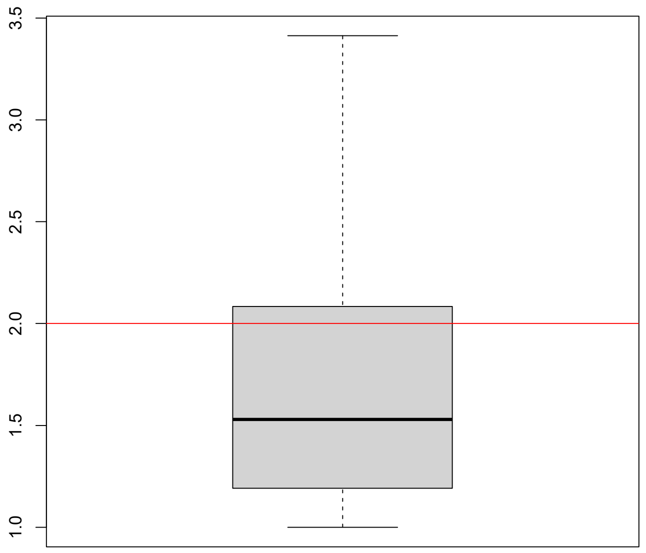
1.0
1.5
2.0
2.5
3.0
3.5

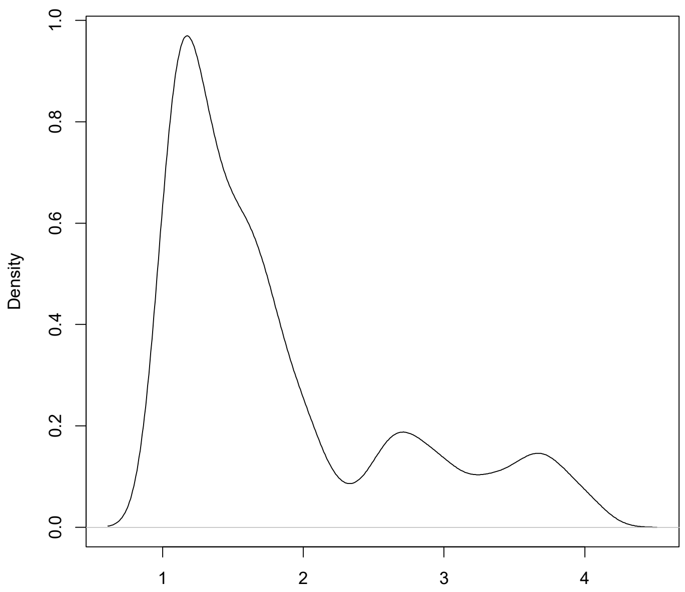
Density
0.0
0.2
0.4
0.6
0.8
1.0
1 2 3 4

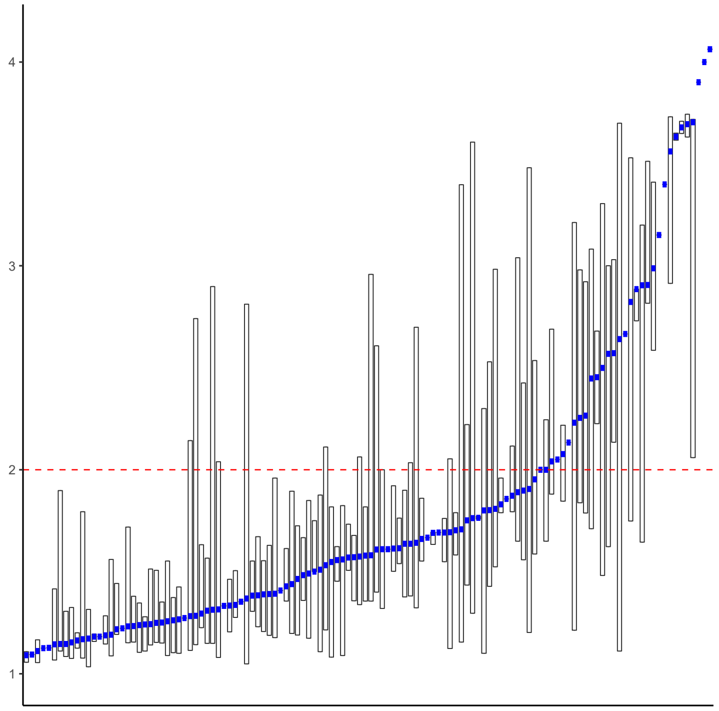
1
2
3
4

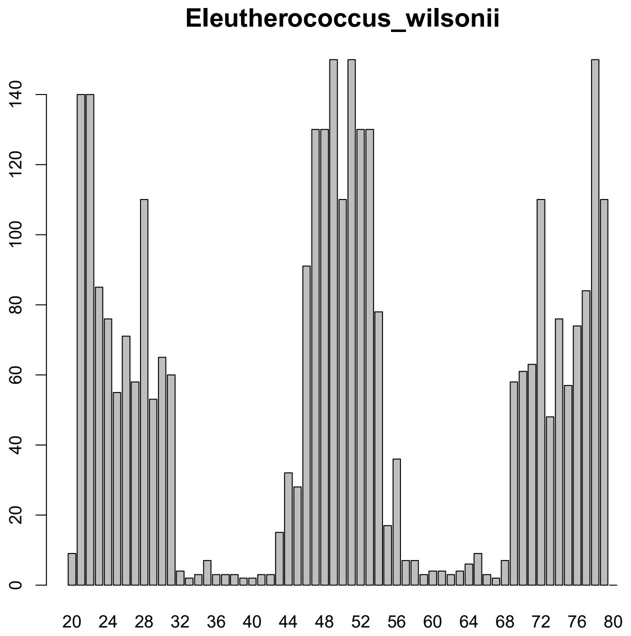
Eleutherococcus_wilsonii
0 20 40 60 80 100 120 140
20 24 28 32 36 40 44 48 52 56 60 64 68 72 76 80

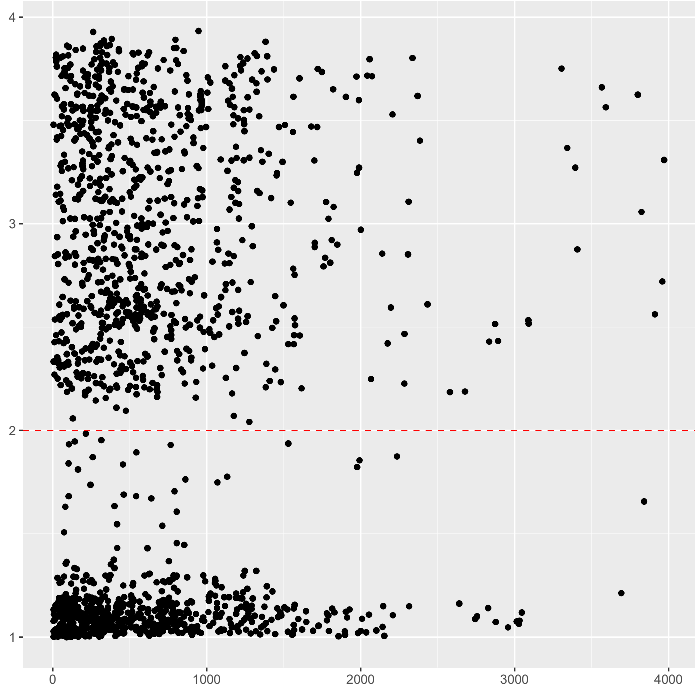
1 2 3 4
0 1000 2000 3000 4000

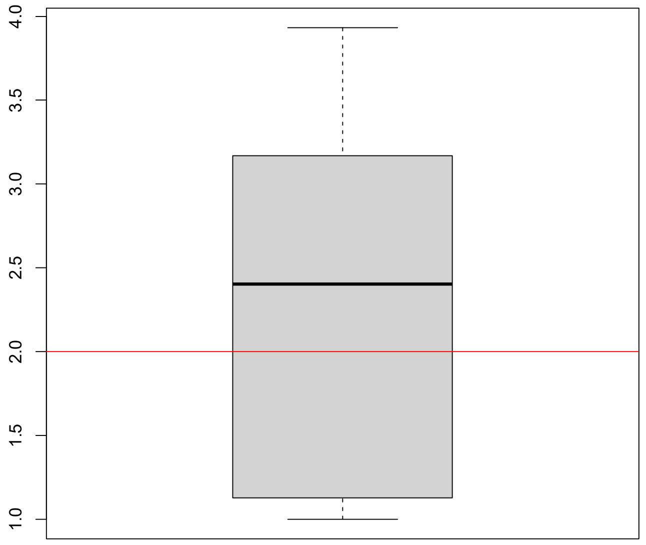
1.0 1.5 2.0 2.5 3.0 3.5 4.0

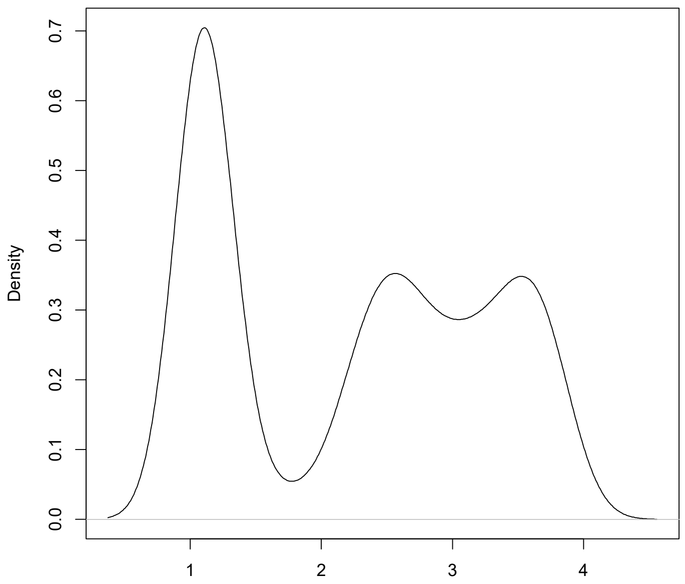
Density
0.0 0.1 0.2 0.3 0.4 0.5 0.6 0.7
1 2 3 4

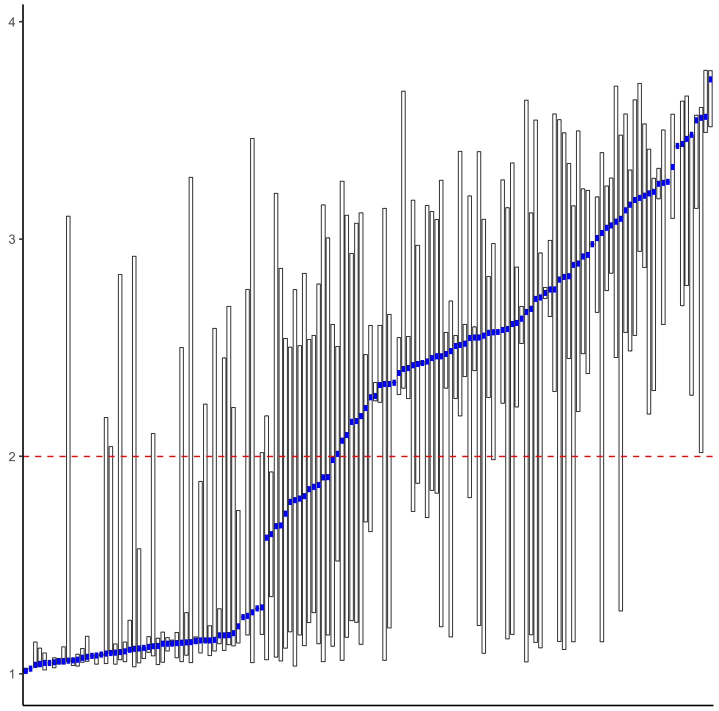
1 2 3 4

Fatsia_japonica

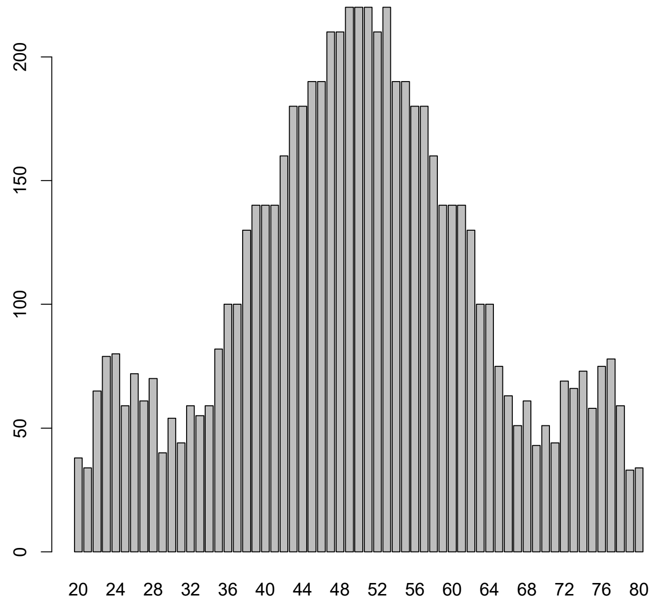

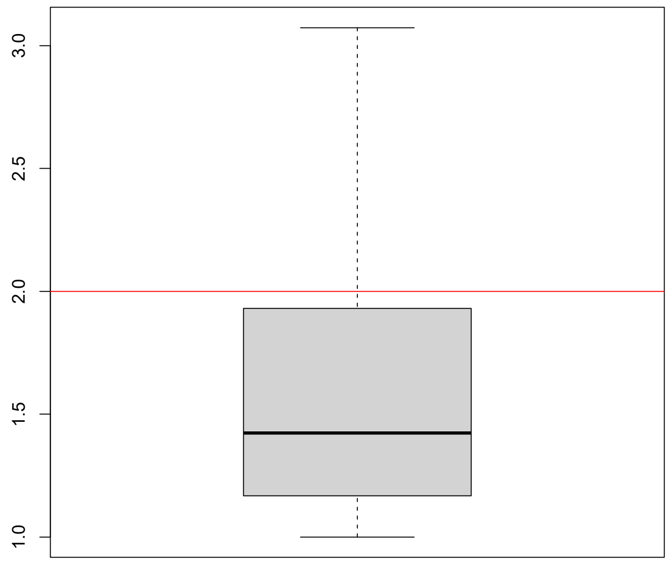

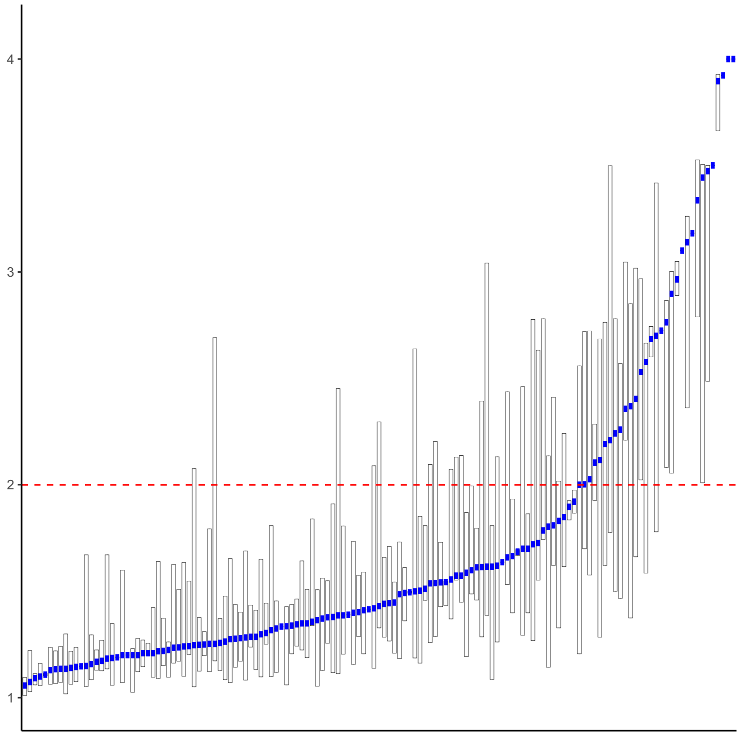

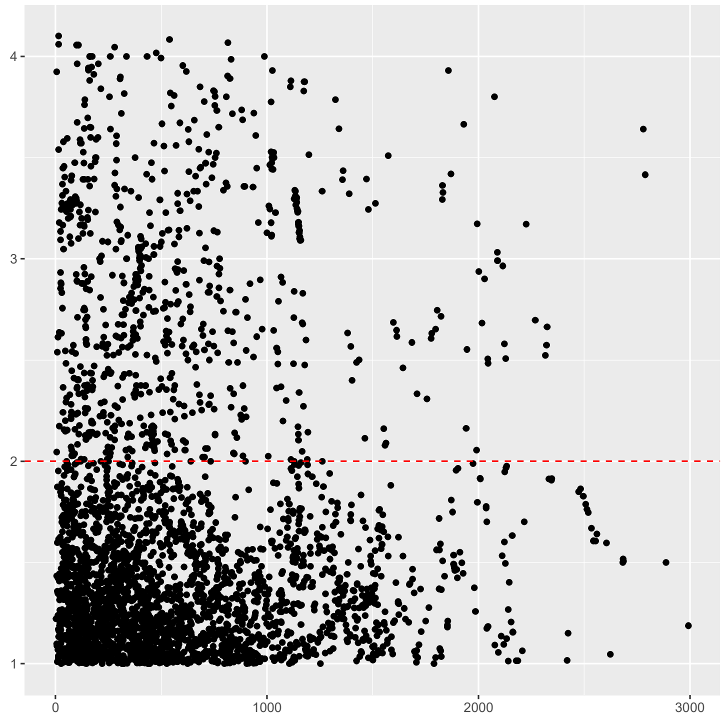

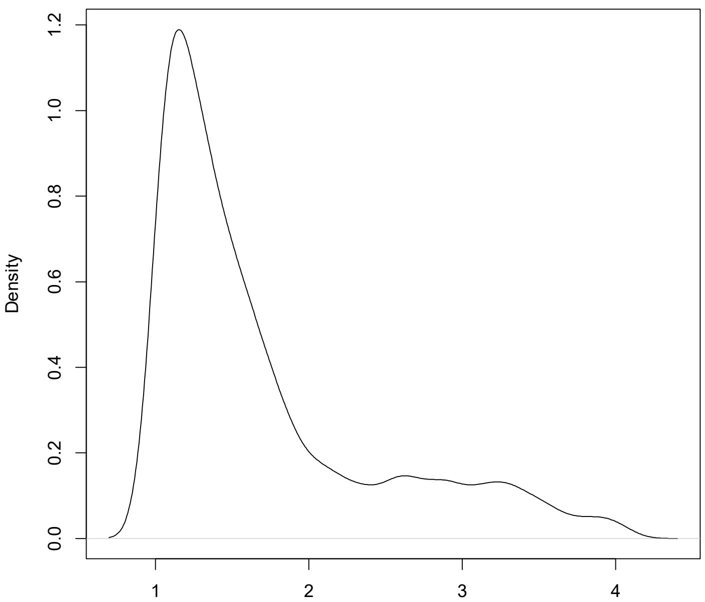

Fatsia_oligocarpella

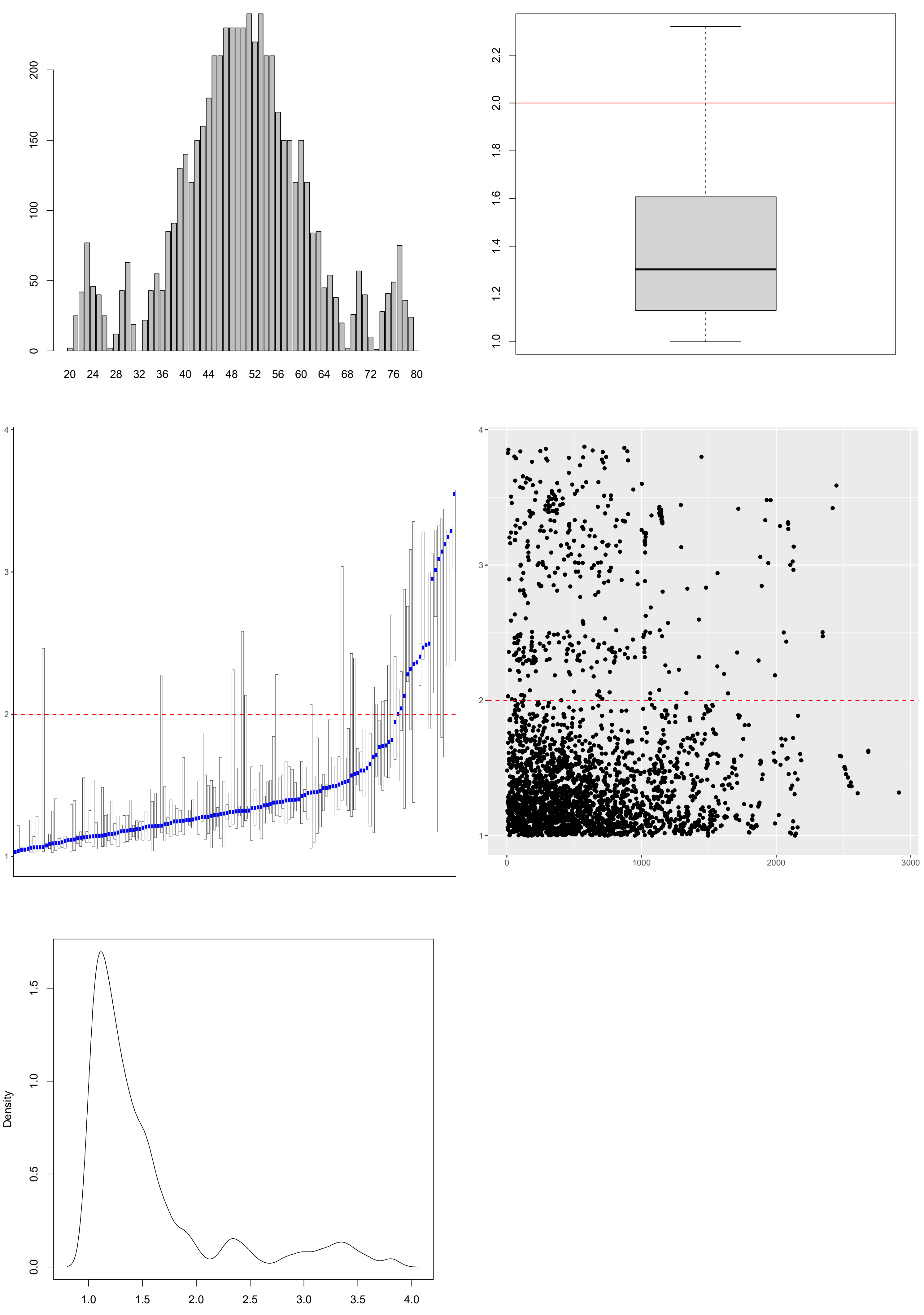

Fatsia_polycarpa

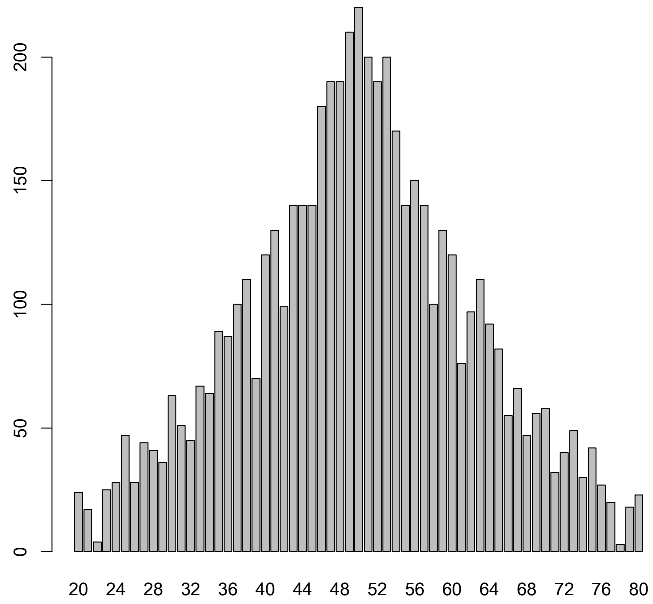

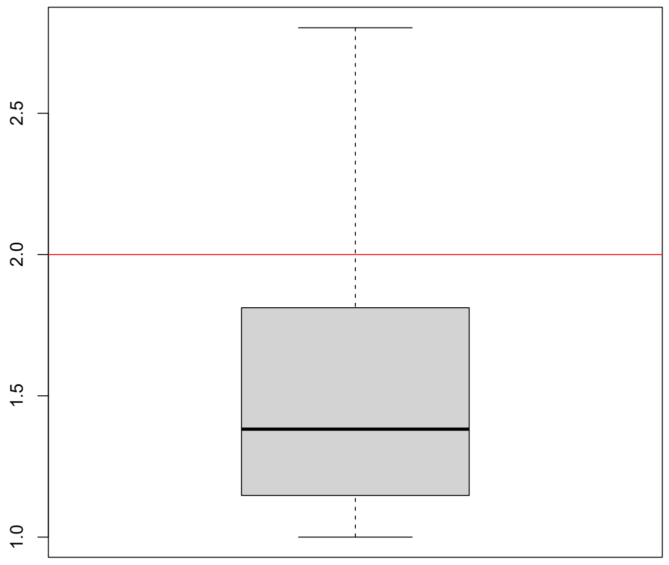

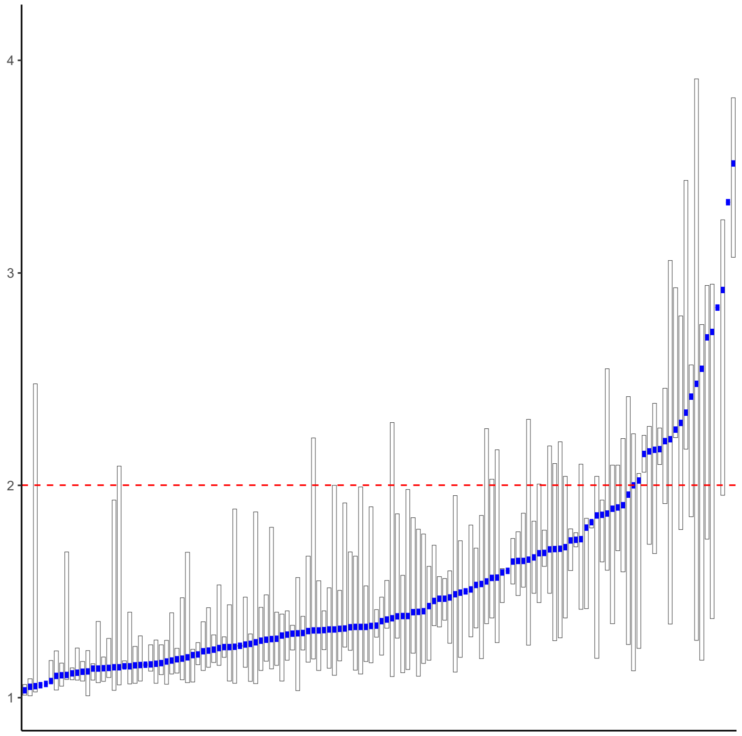

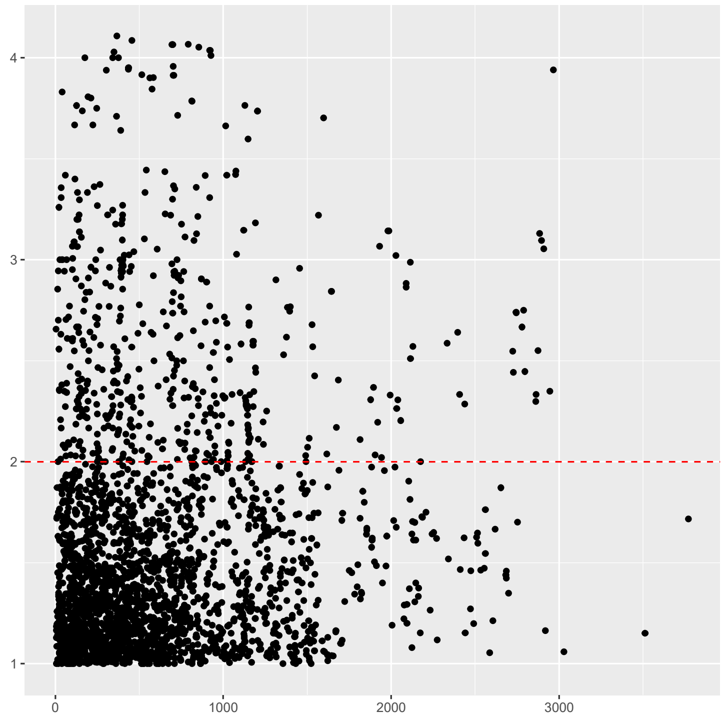

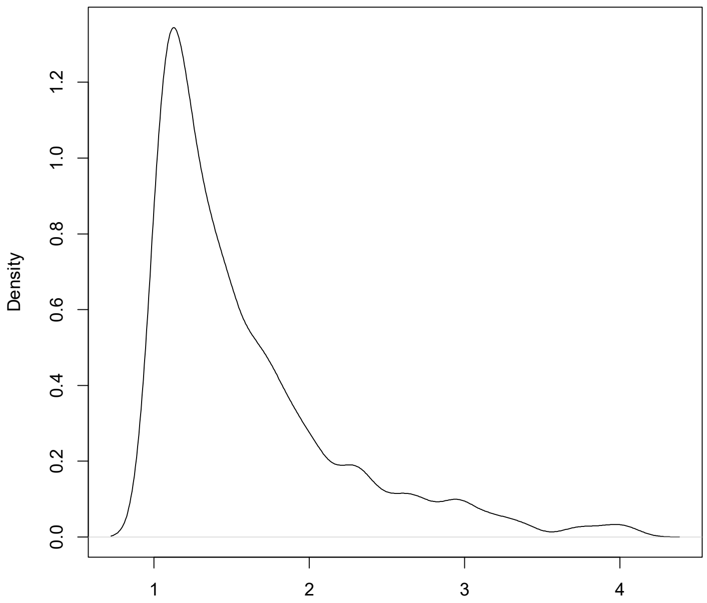

Frodinia_gleasonii

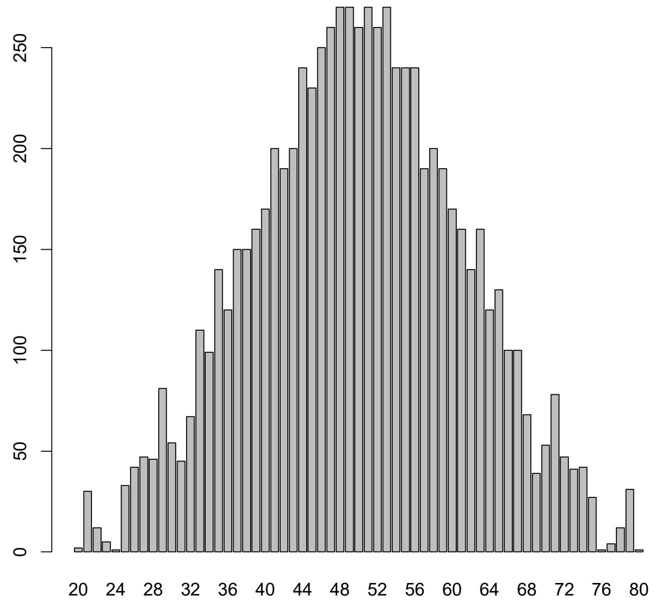

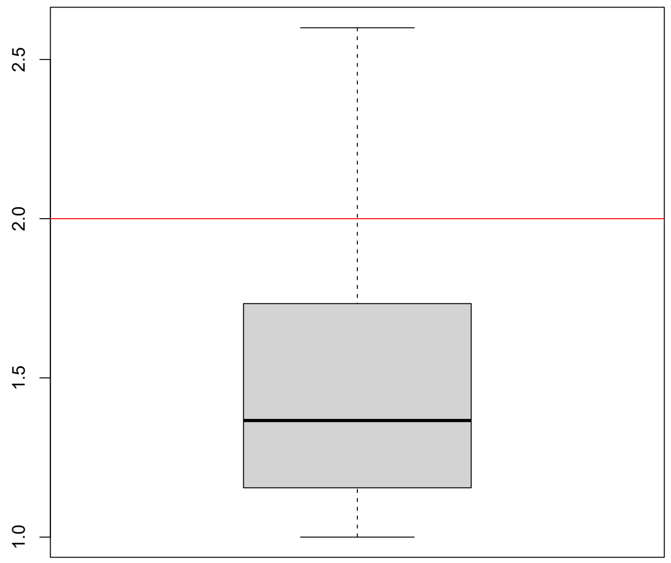

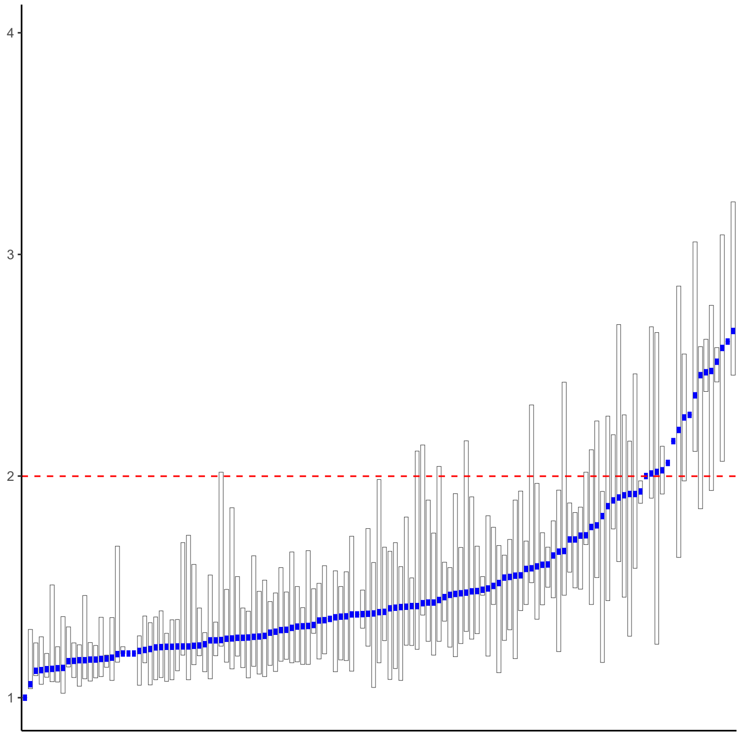

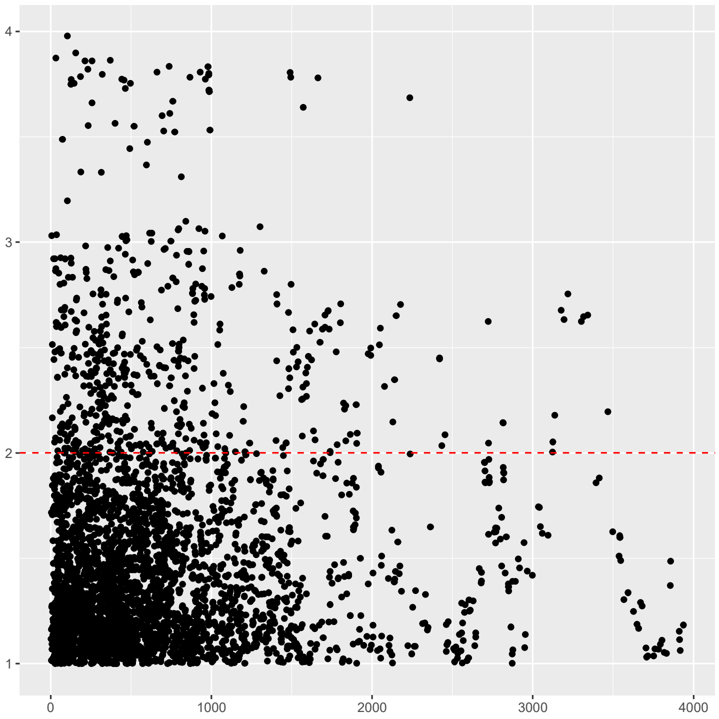

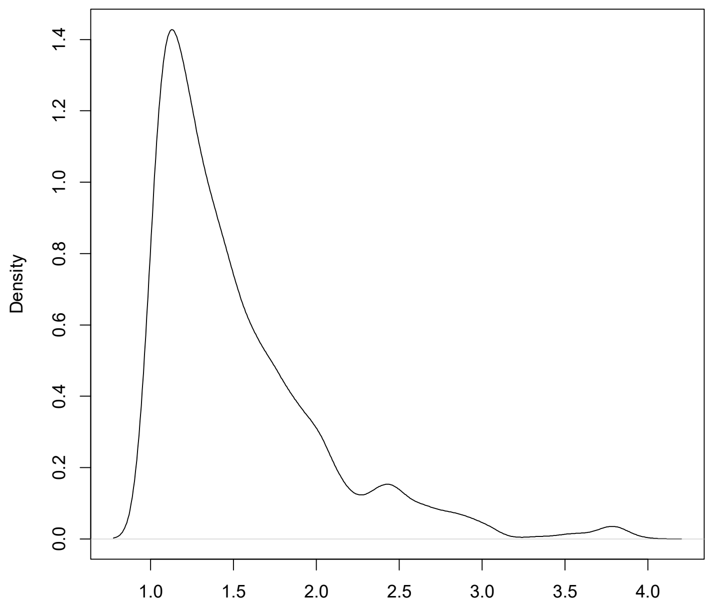

Gamblea_ciliata

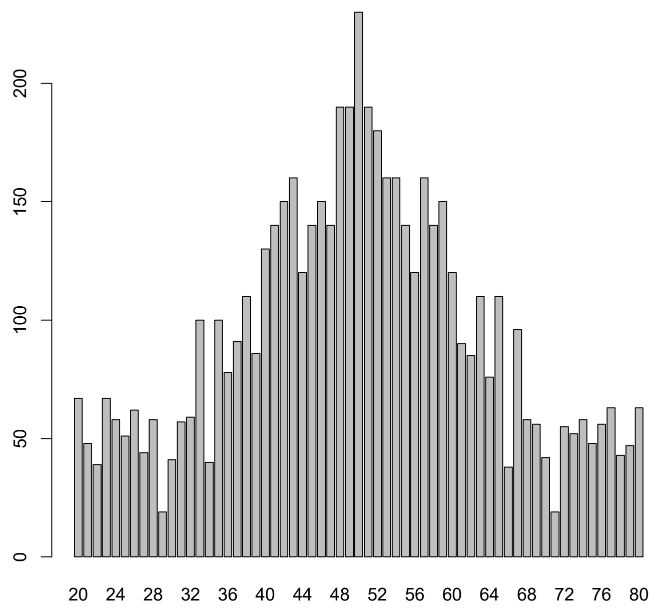

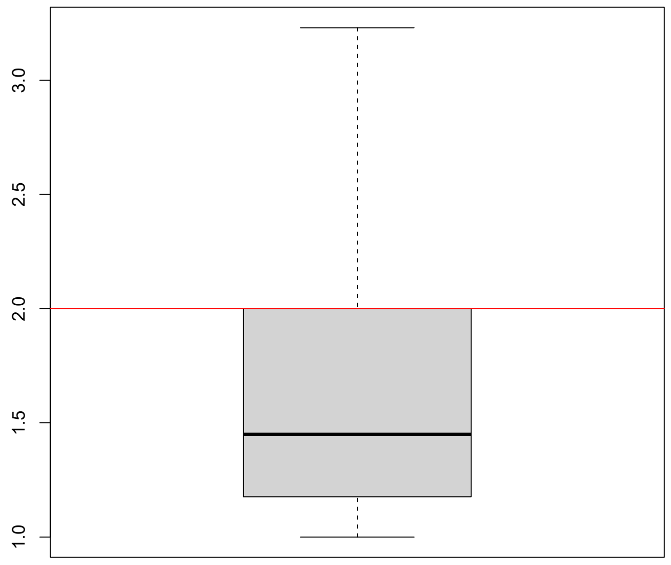

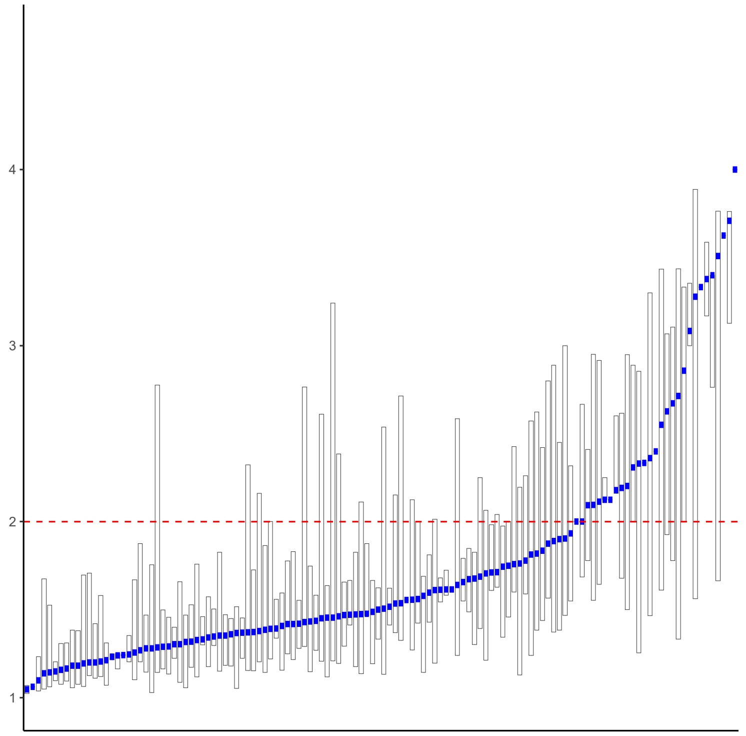

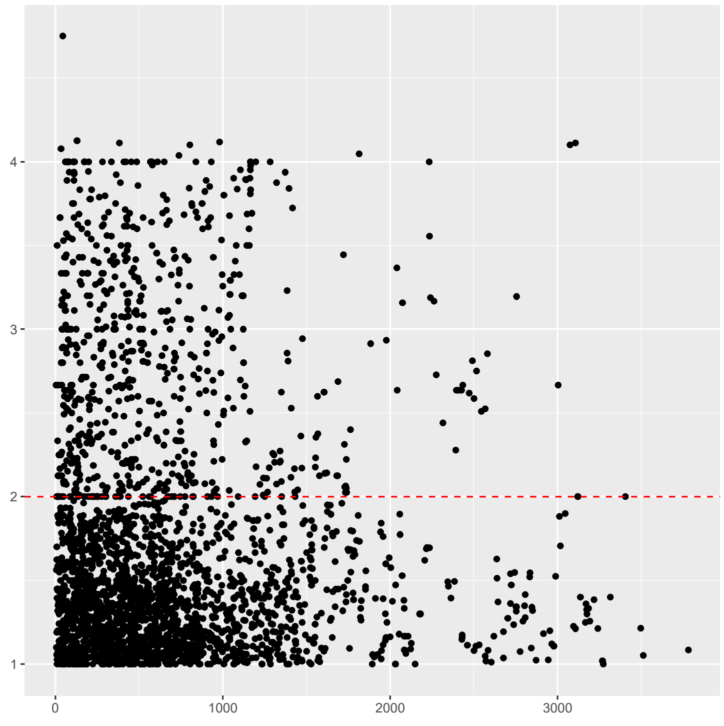

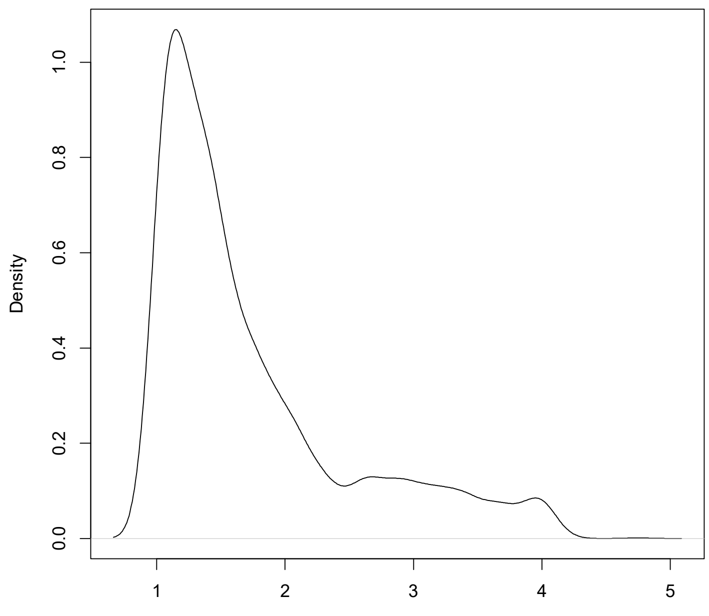

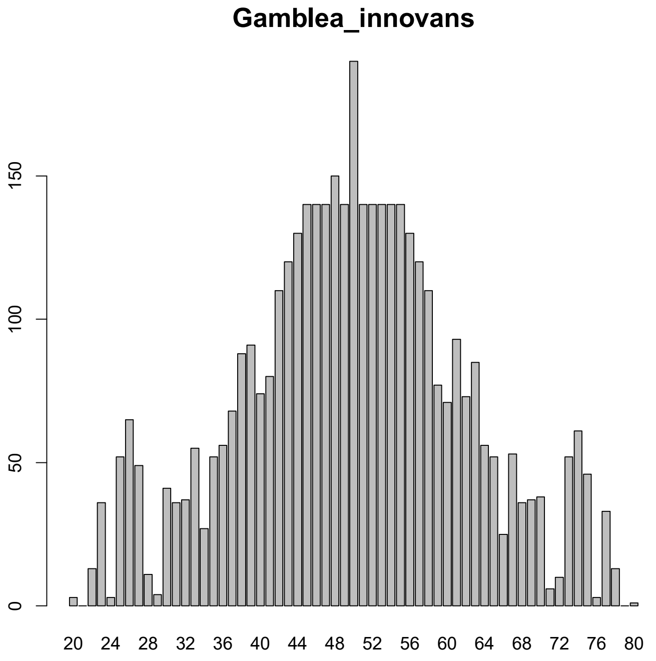
Gamblea_innovans
0
50
100
150
20
24
28
32
36
40
44
48
52
56
60
64
68
72
76
80

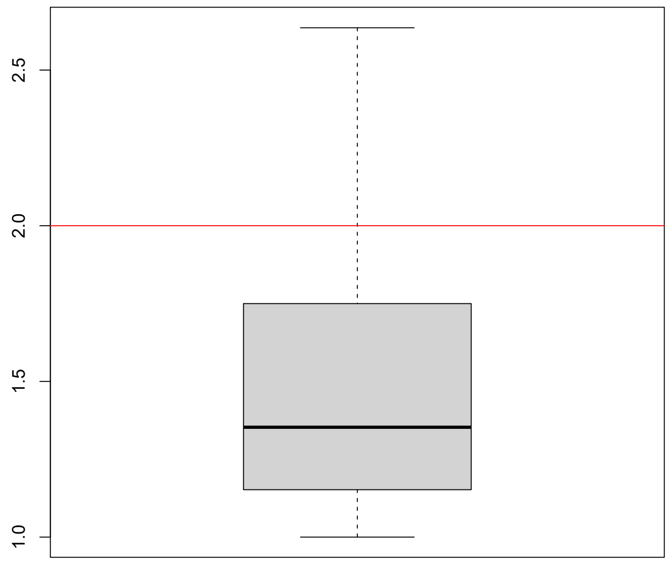
1.0
1.5
2.0
2.5

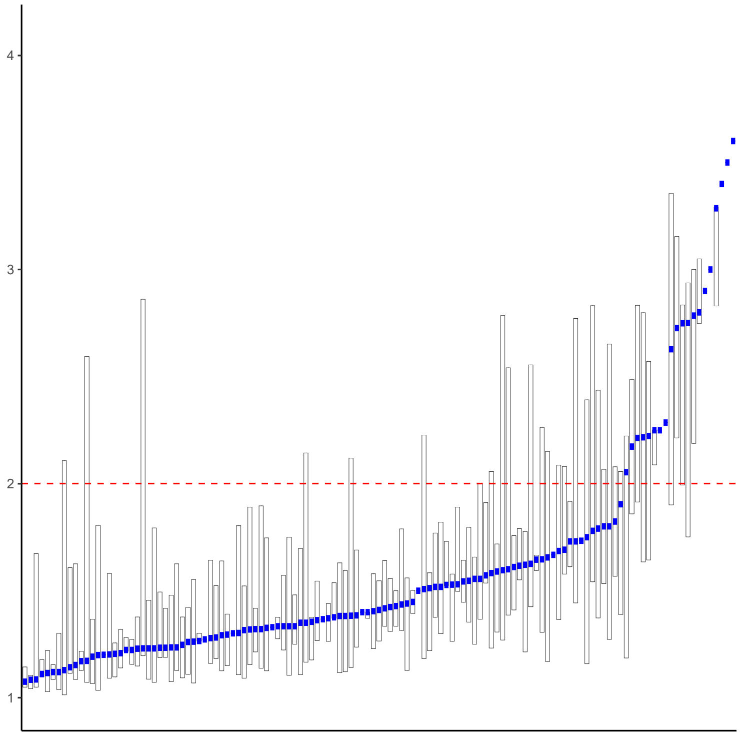
1
2
3
4

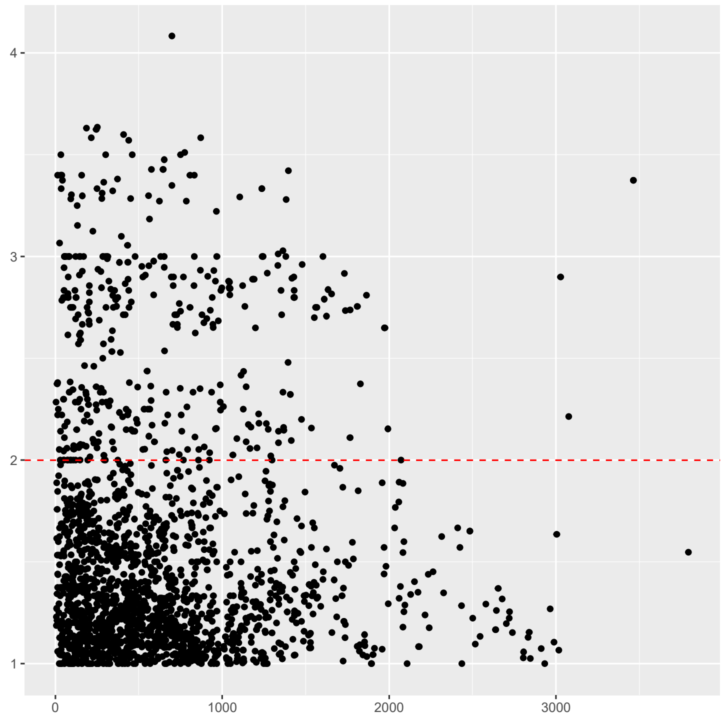
1
2
3
4
0
1000
2000
3000

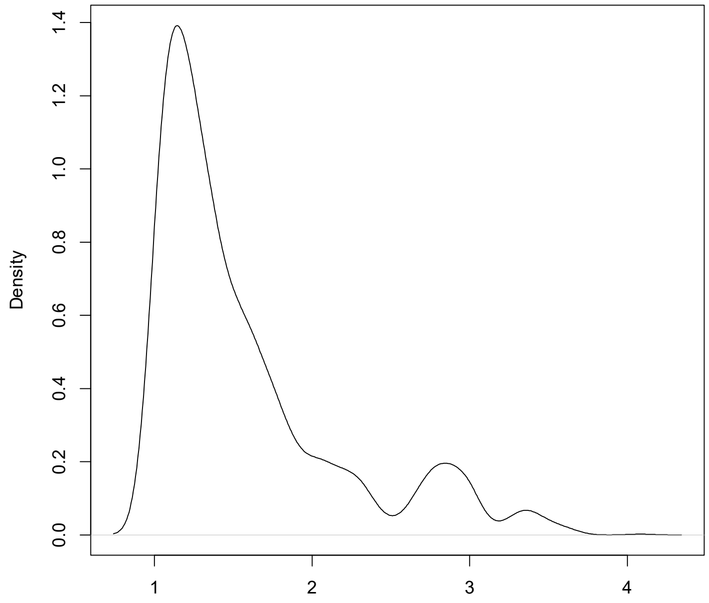
Density
0.0
0.2
0.4
0.6
0.8
1.0
1.2
1.4
1
2
3
4

Gamblea_malayana

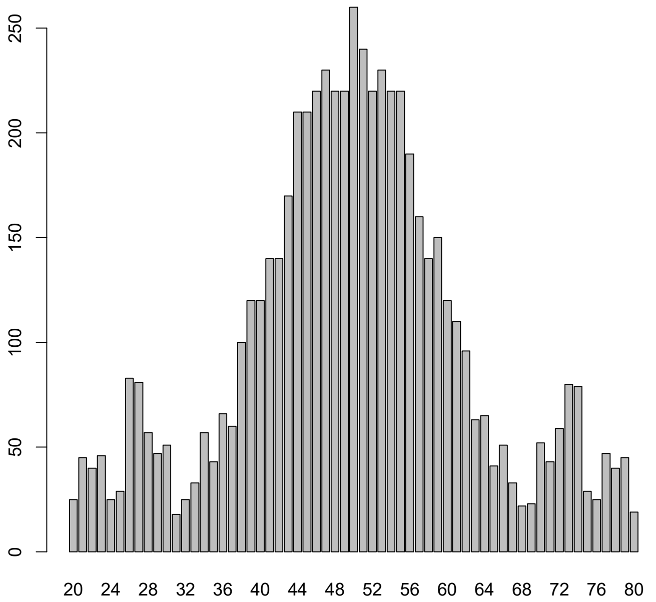

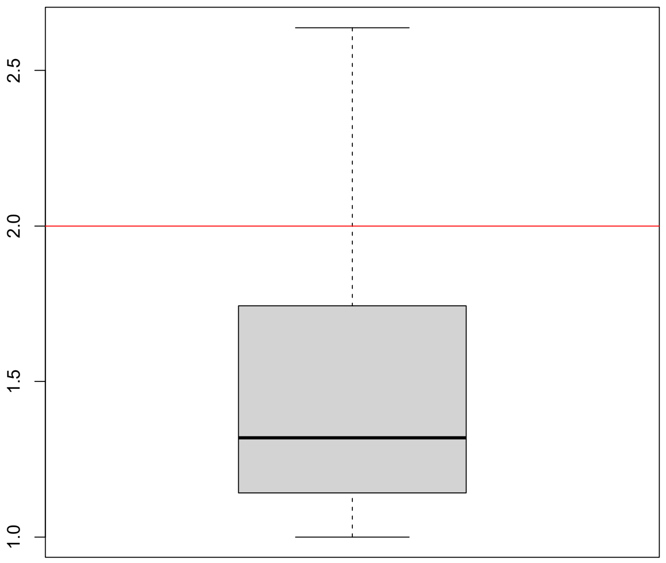

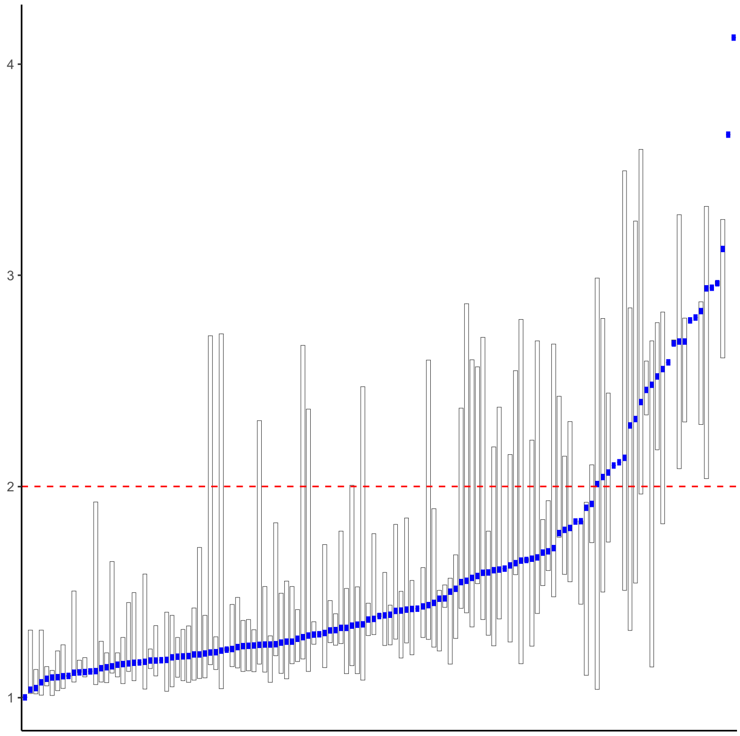

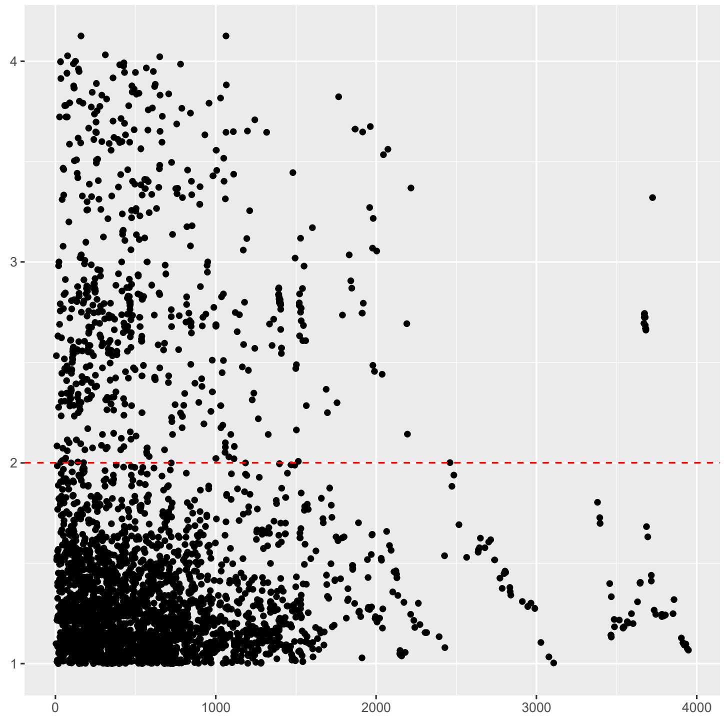

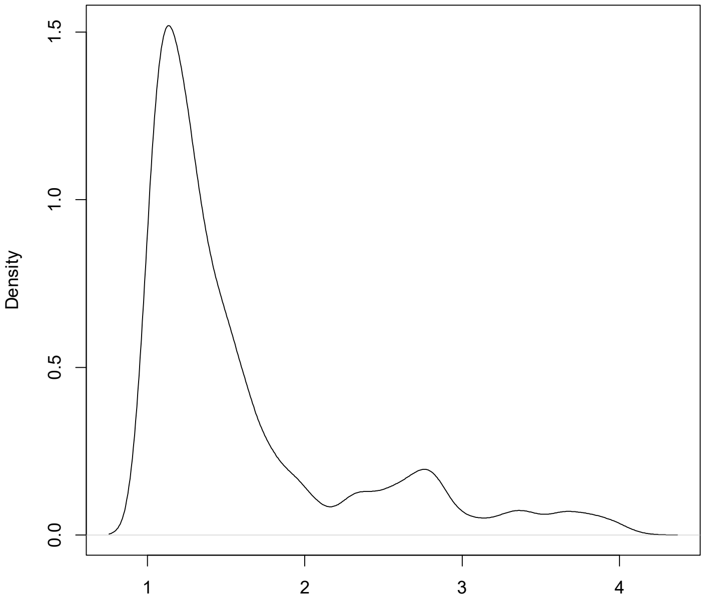

Gamblea_pseudoevodiifolia

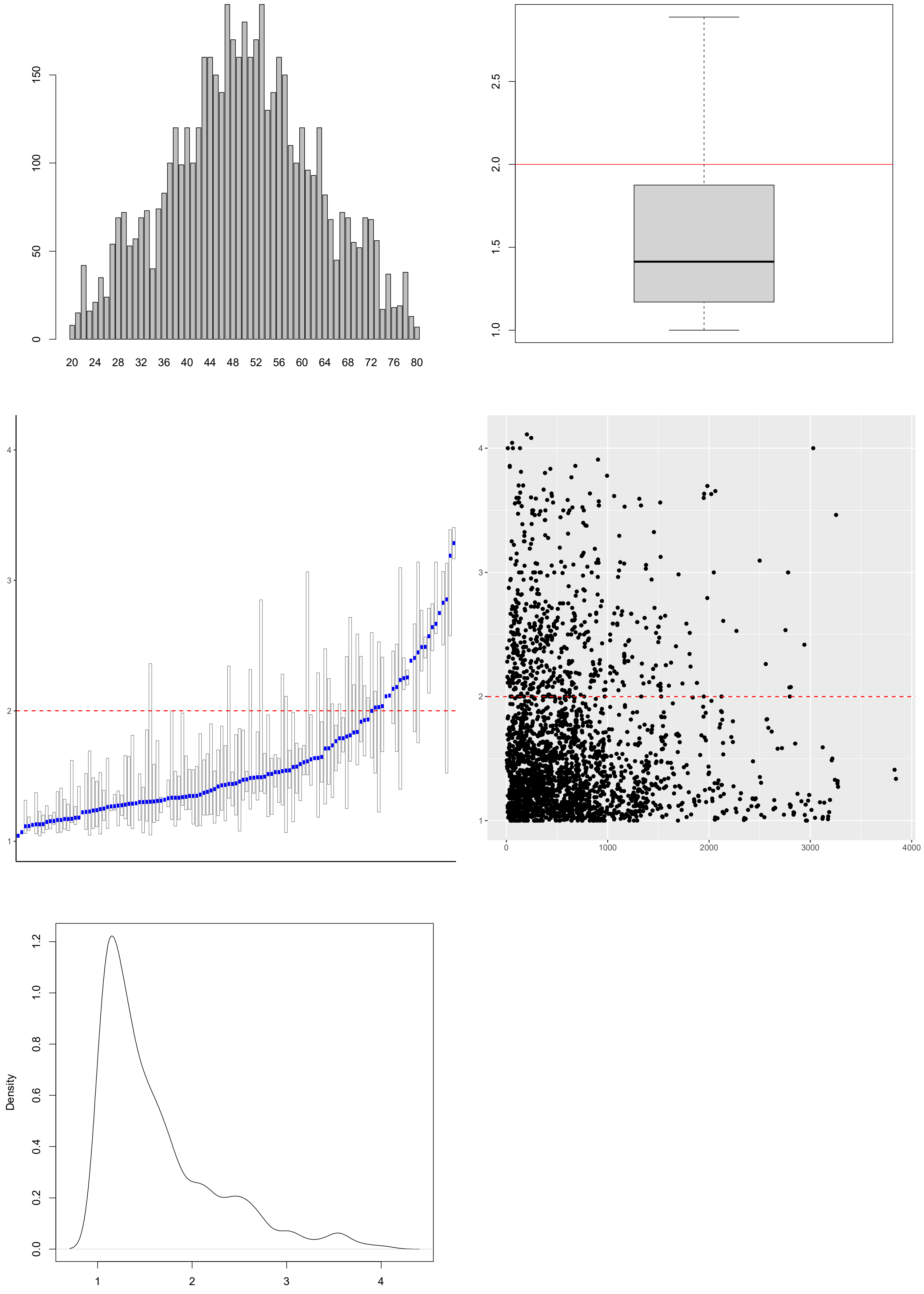

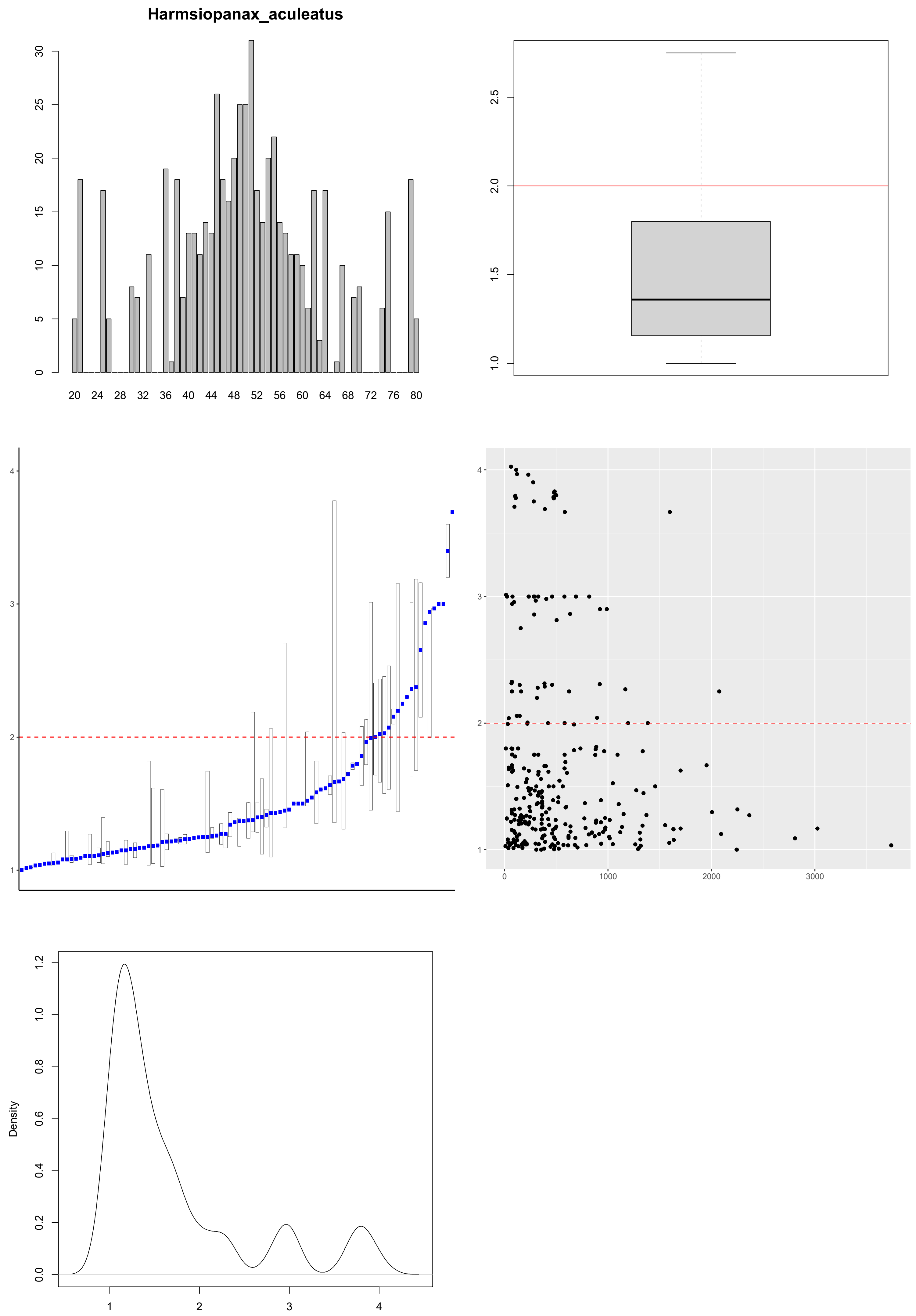
Harmsiopanax_aculeatus
Density

Harmsiopanax_ingens

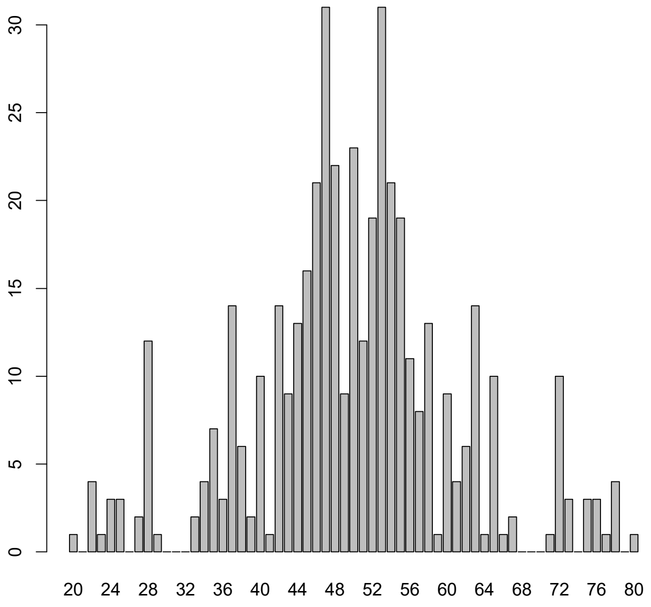

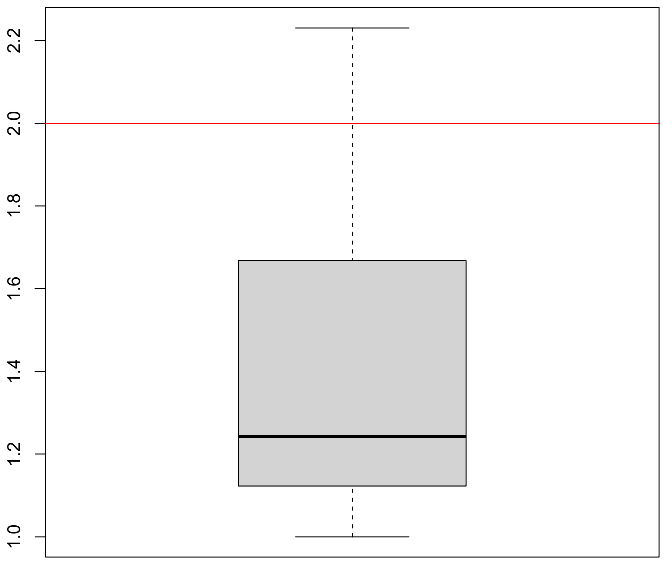

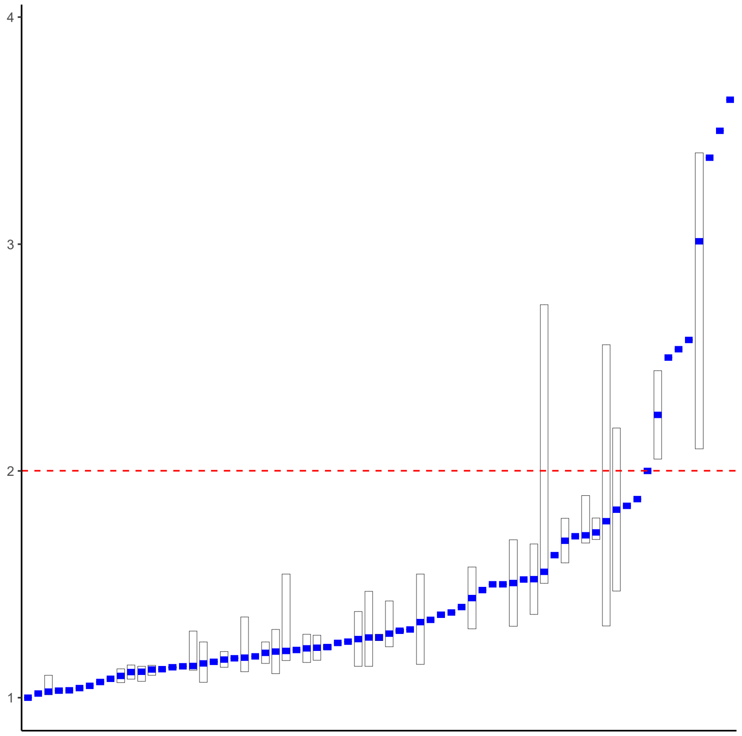

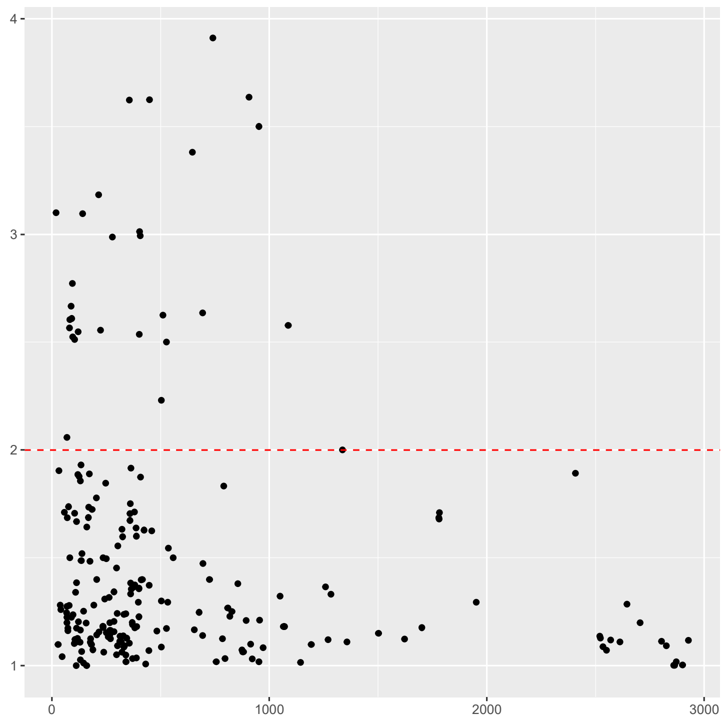

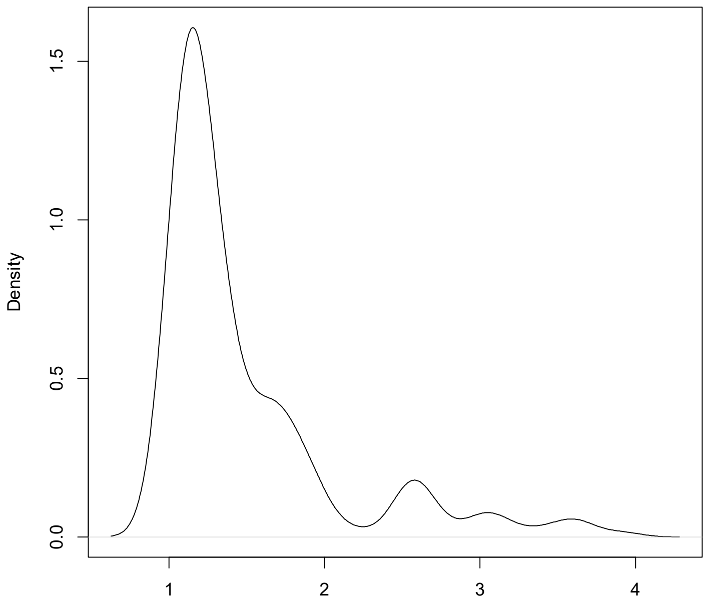

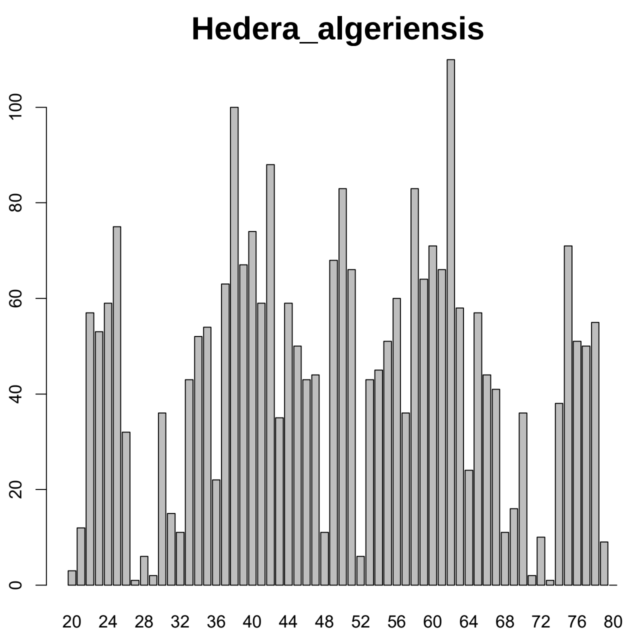
Hedera_algeriensis
0
20
40
60
80
100
20 24 28 32 36 40 44 48 52 56 60 64 68 72 76 80

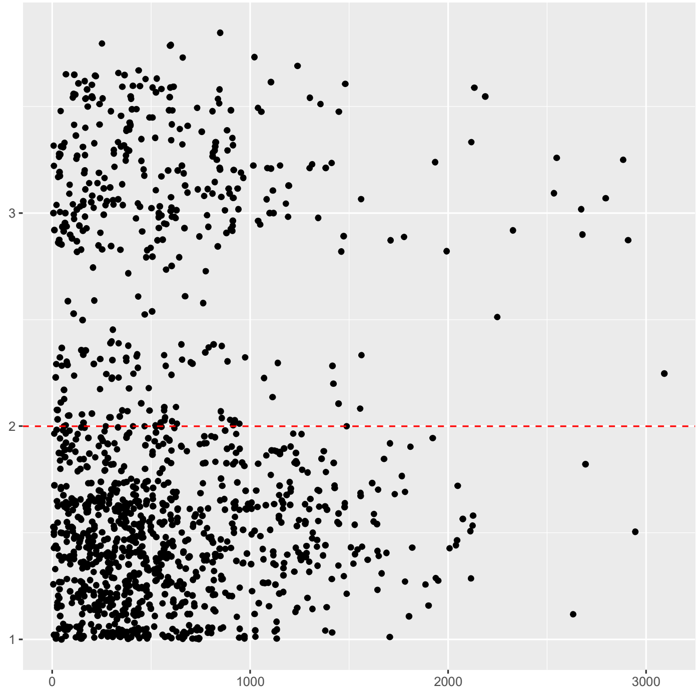
1
2
3
0 1000 2000 3000

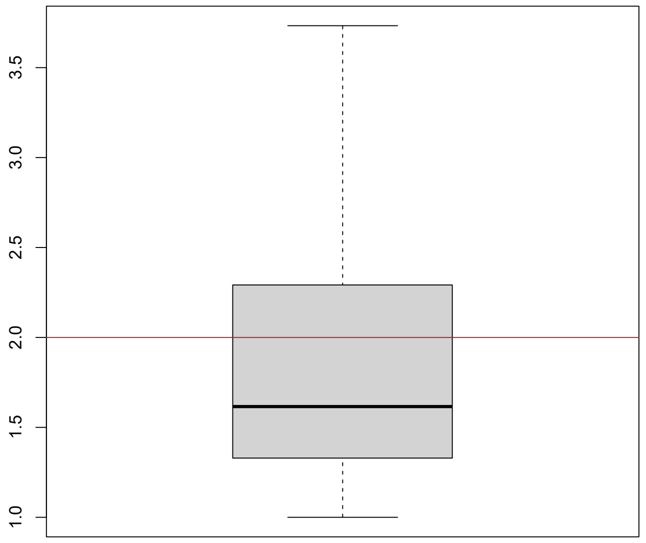
1.0
1.5
2.0
2.5
3.0
3.5

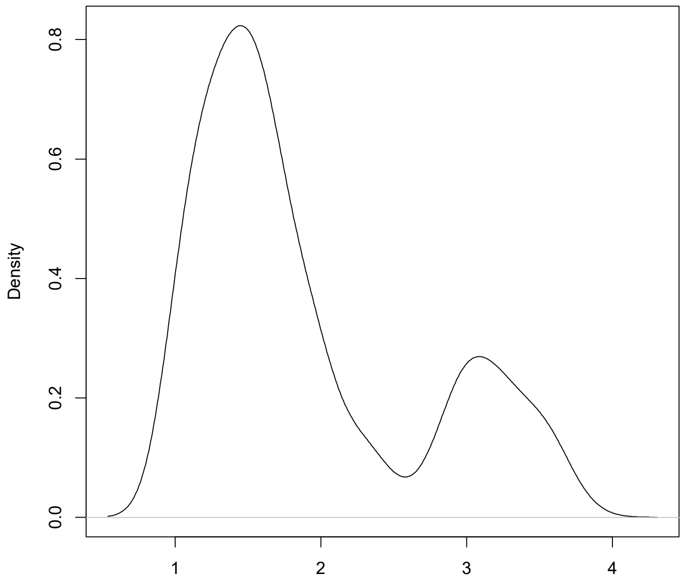
Density
0.0
0.2
0.4
0.6
0.8
1 2 3 4

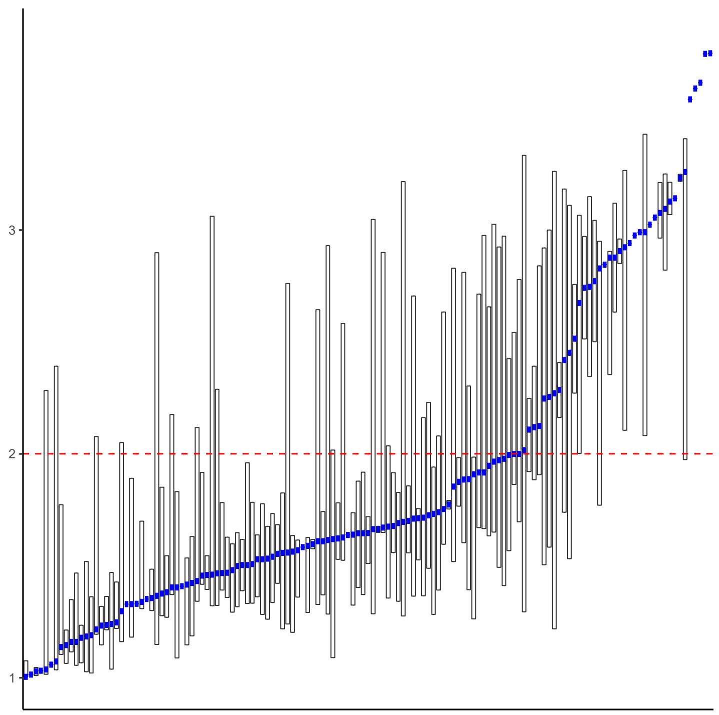
1
2
3

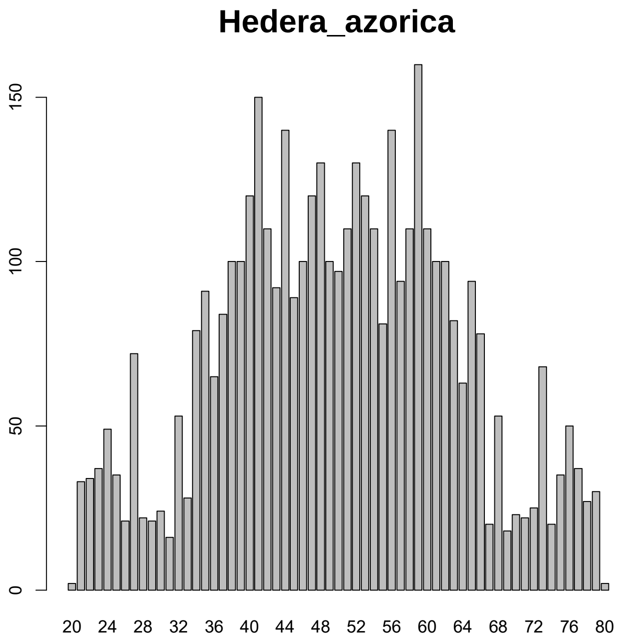
Hedera_azorica

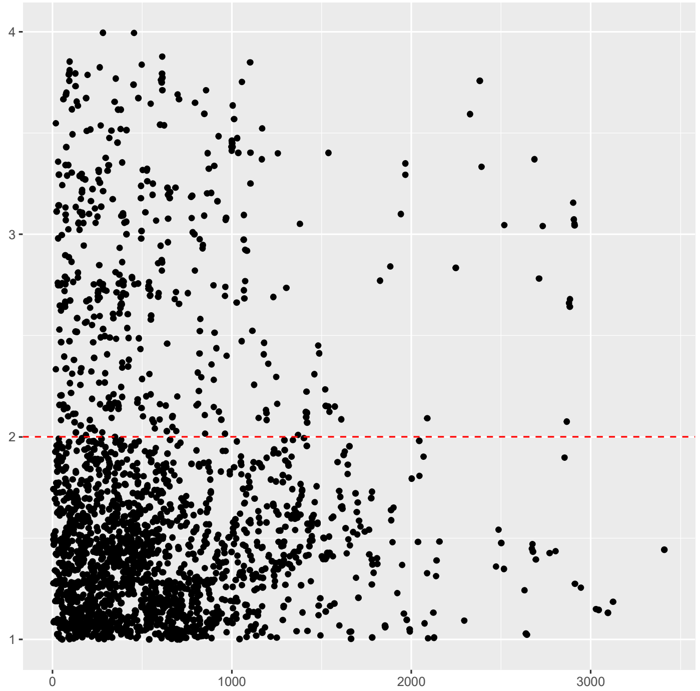

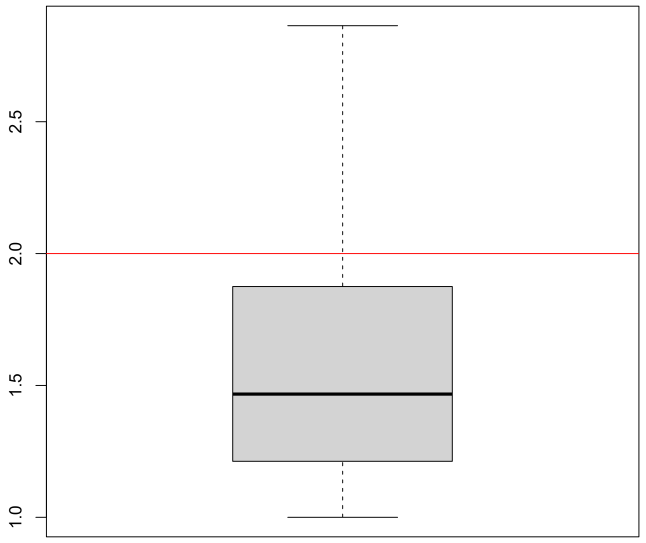

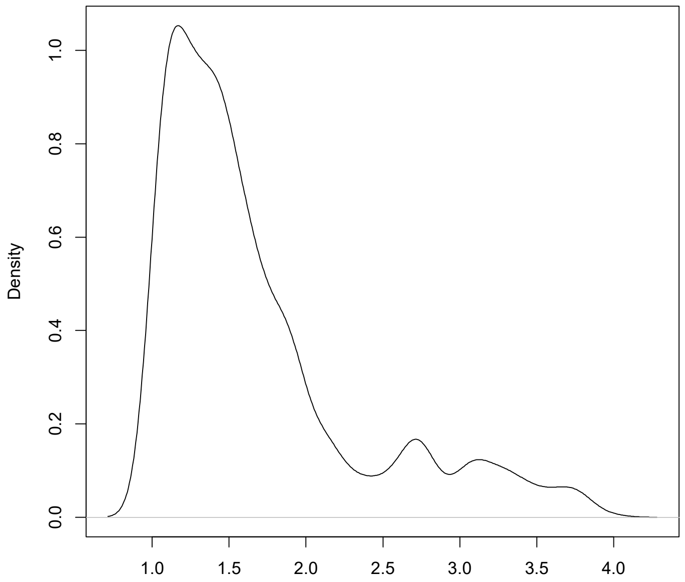
Density

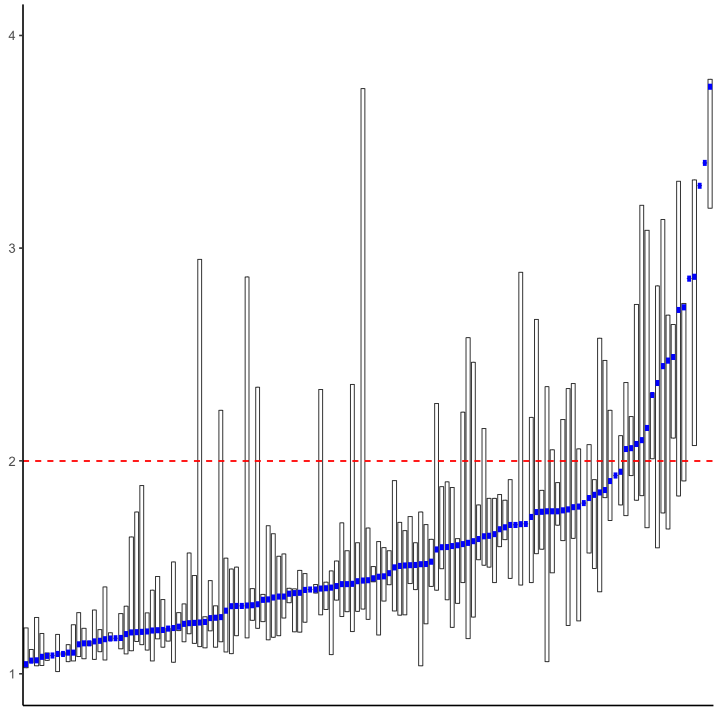

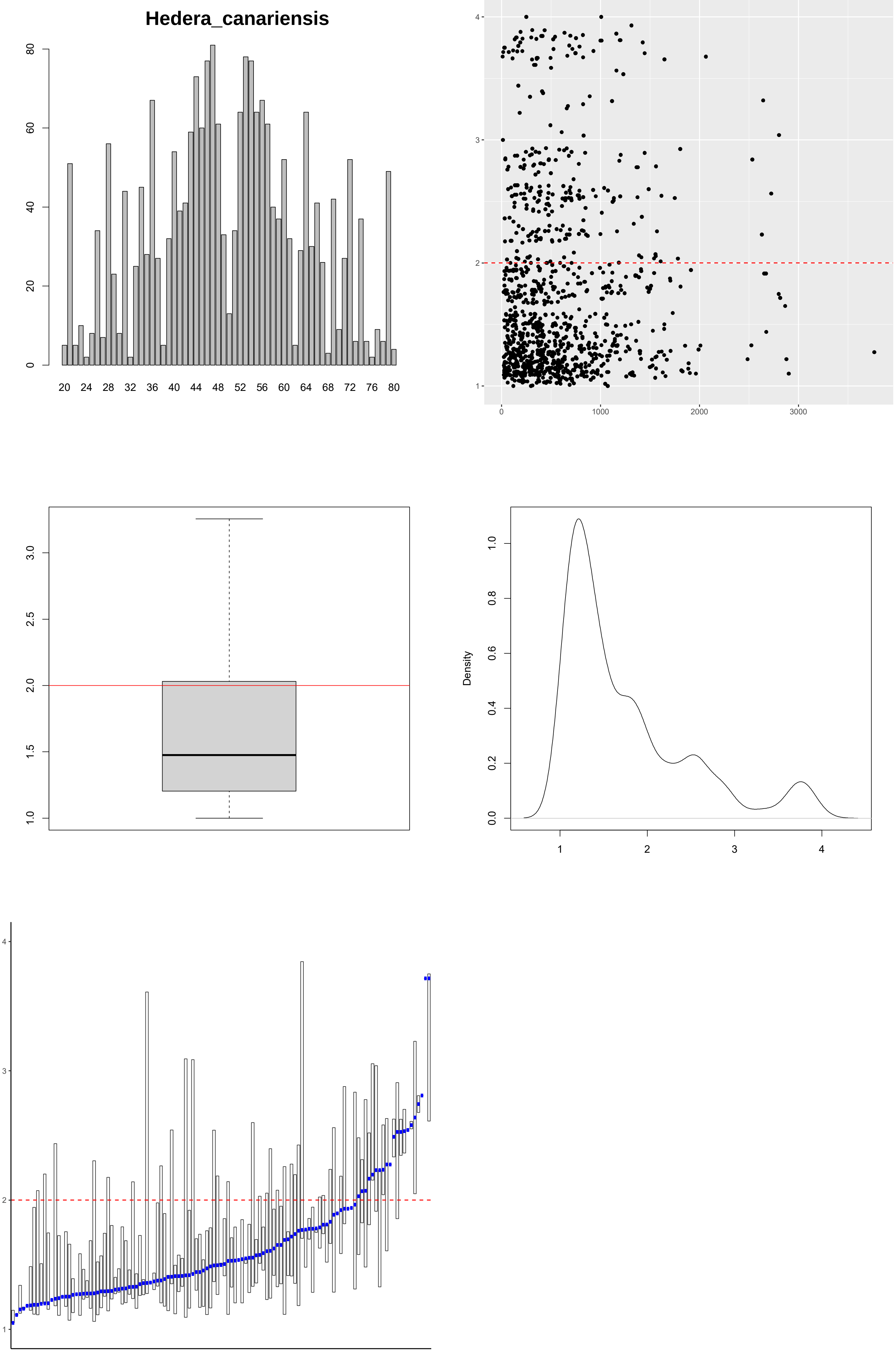
Hedera_canariensis
Density

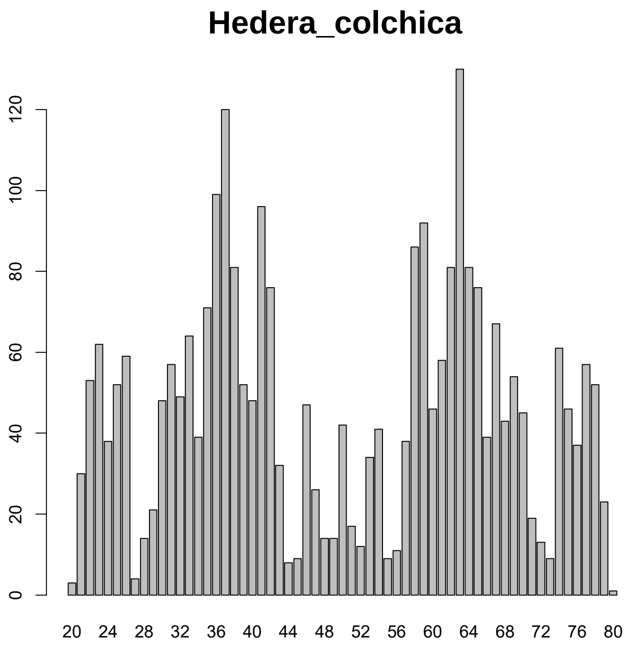
Hedera_colchica

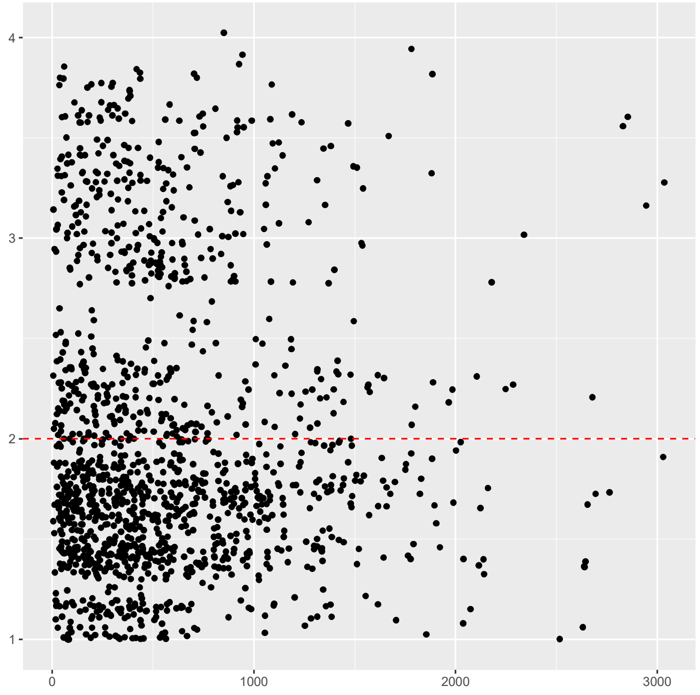

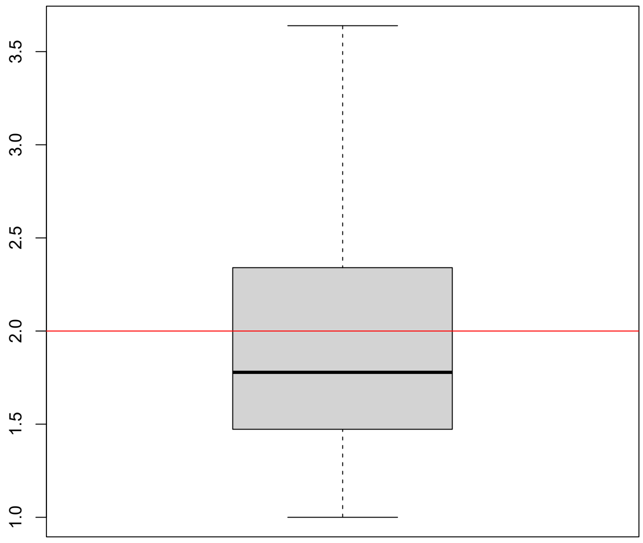

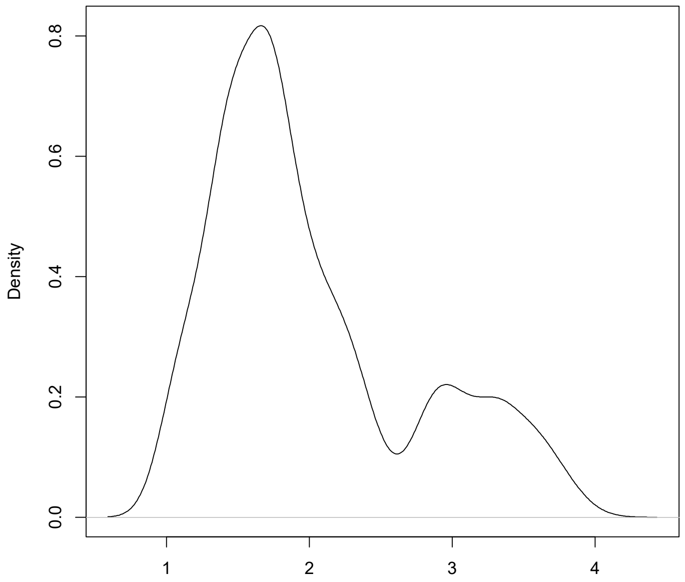
Density

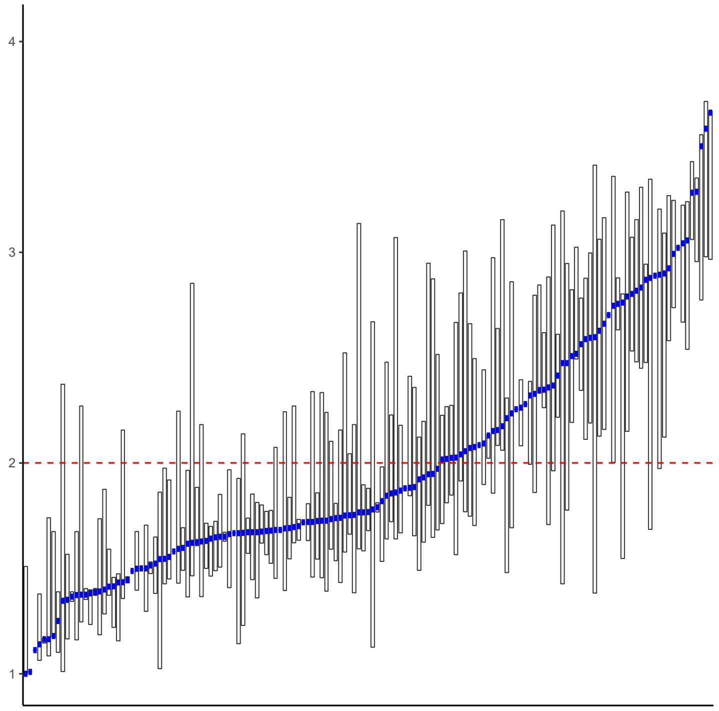

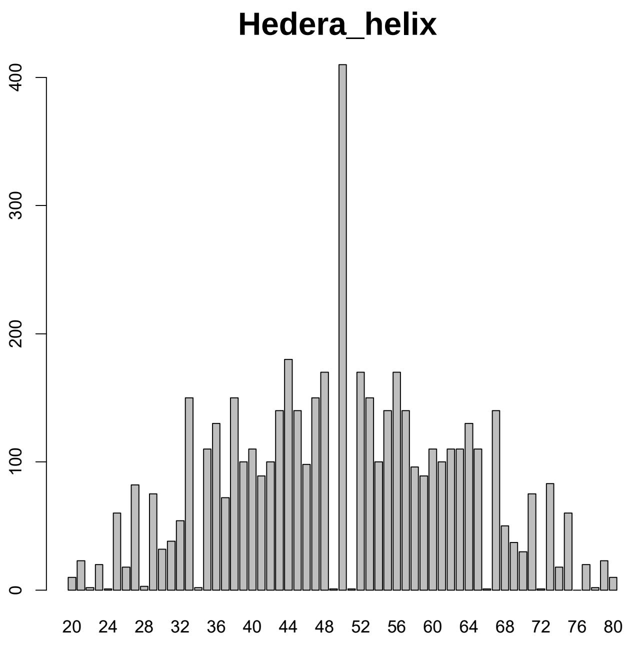
Hedera_helix
0
100
200
300
400
20 24 28 32 36 40 44 48 52 56 60 64 68 72 76 80

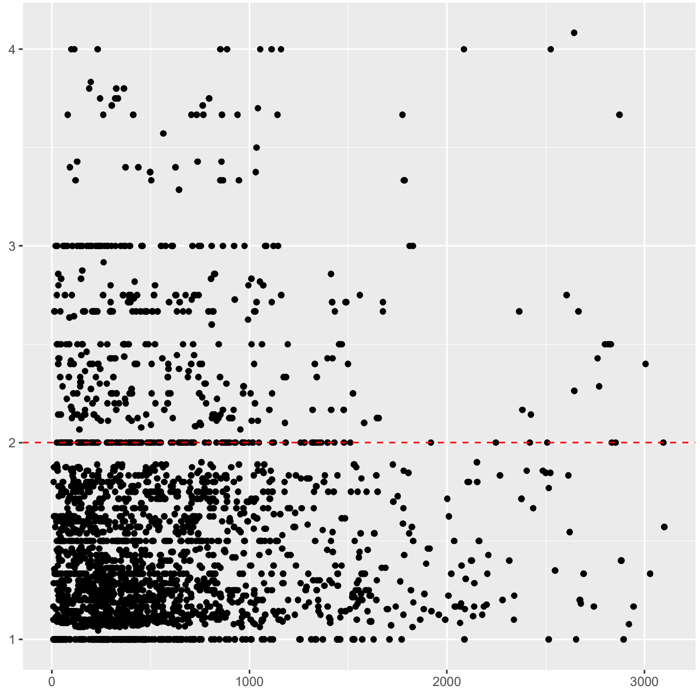
1
2
3
4
0 1000 2000 3000

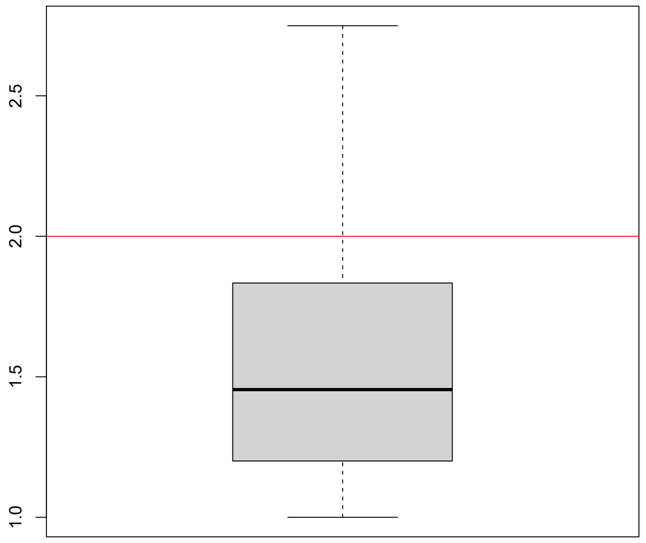
1.0
1.5
2.0
2.5

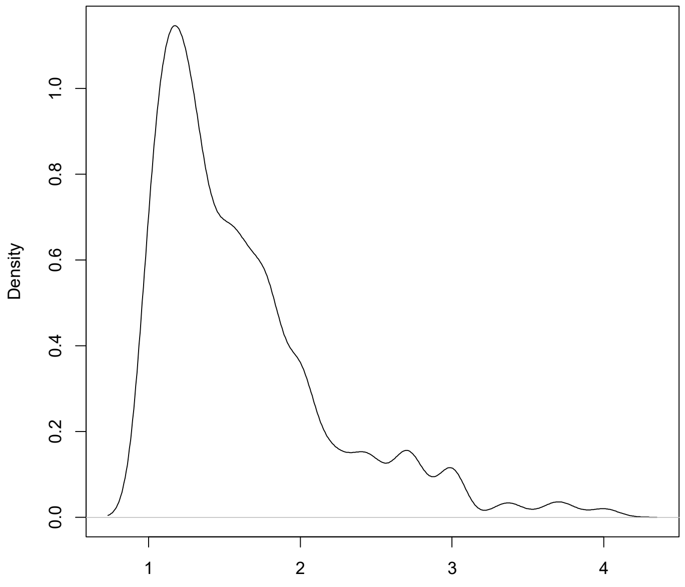
Density
0.0
0.2
0.4
0.6
0.8
1.0
1 2 3 4

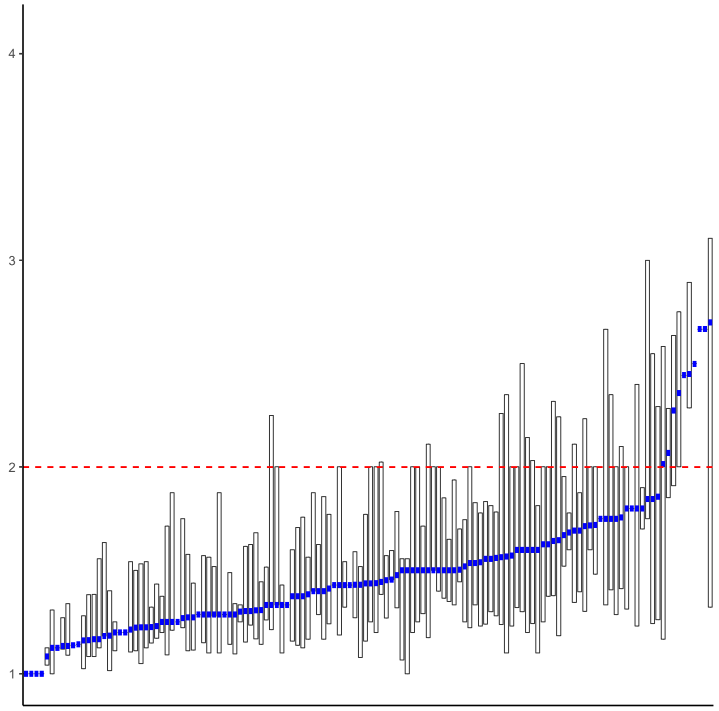
1
2
3
4

Hedera_hibernica

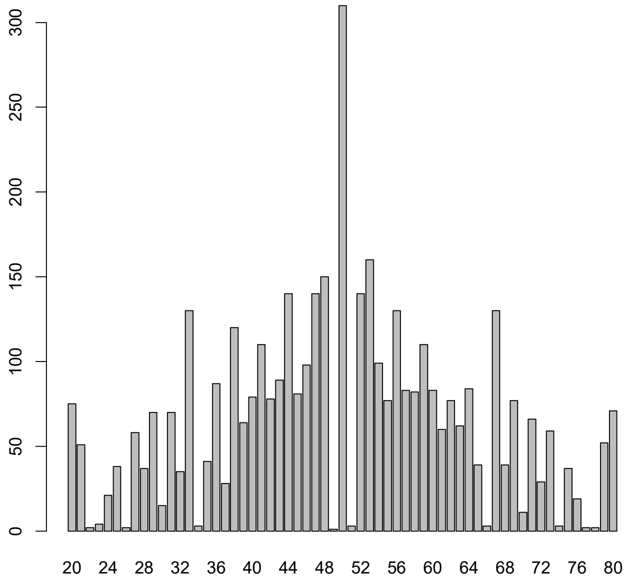

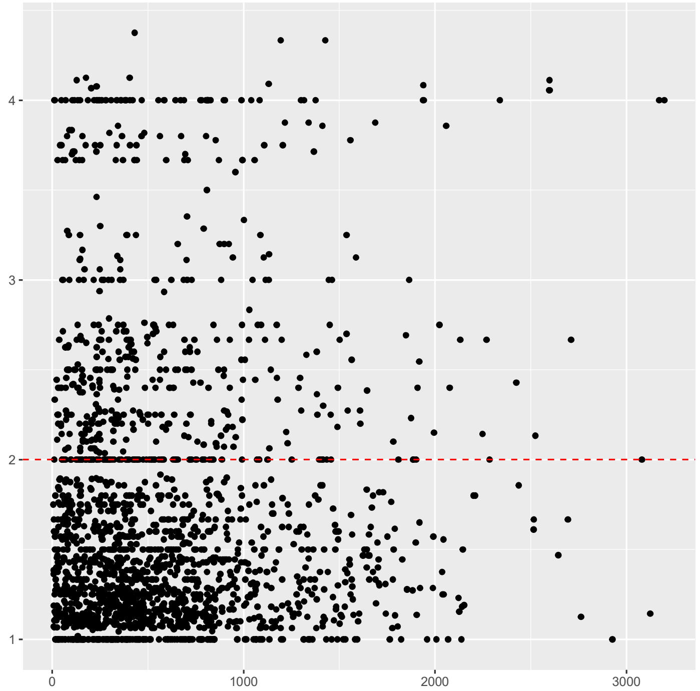

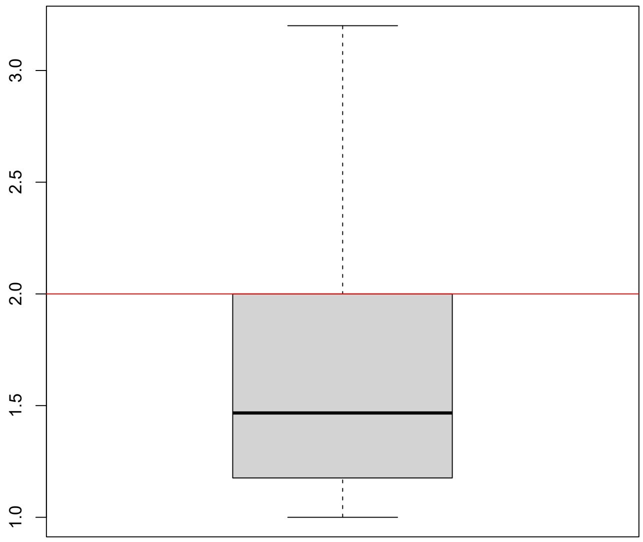

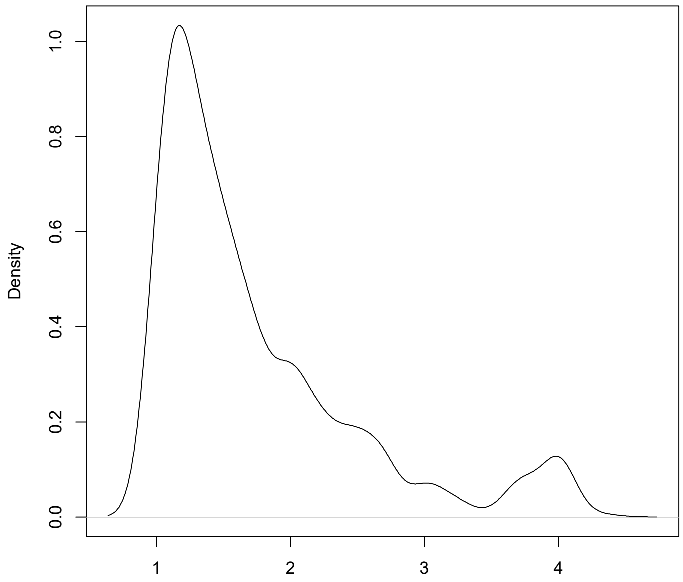

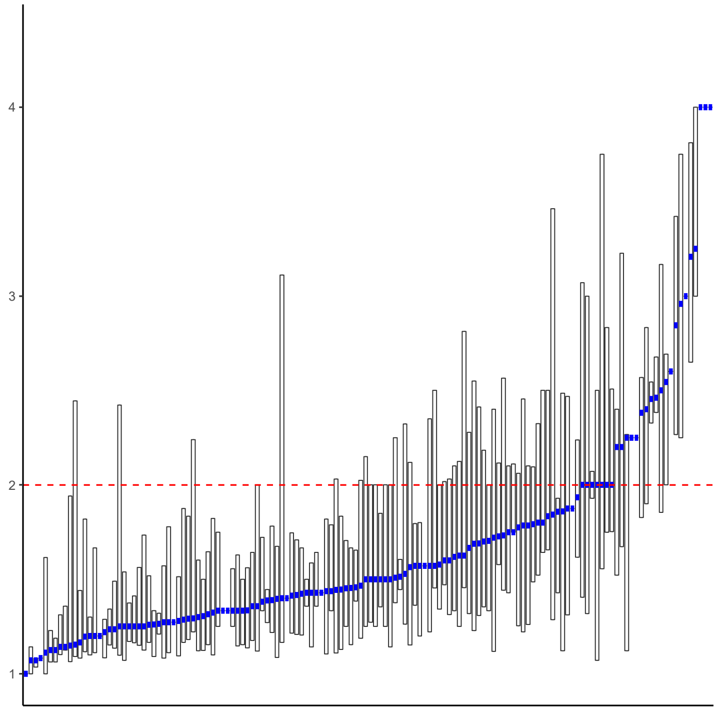

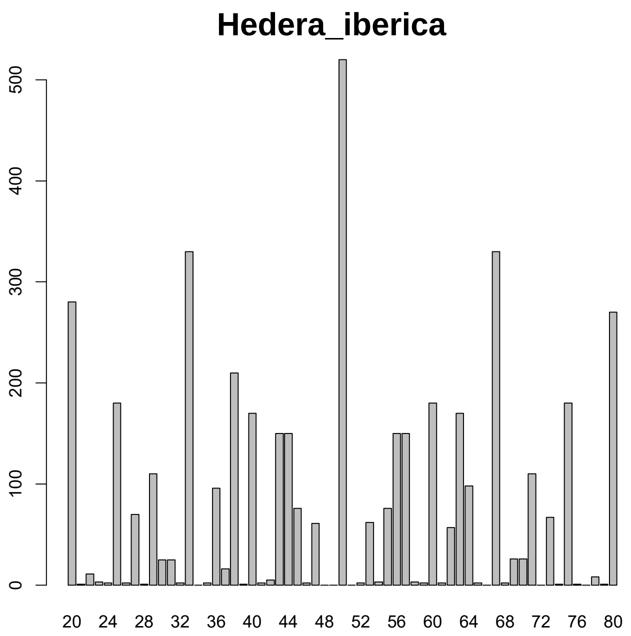
Hedera_iberica
0
100
200
300
400
500
20 24 28 32 36 40 44 48 52 56 60 64 68 72 76 80

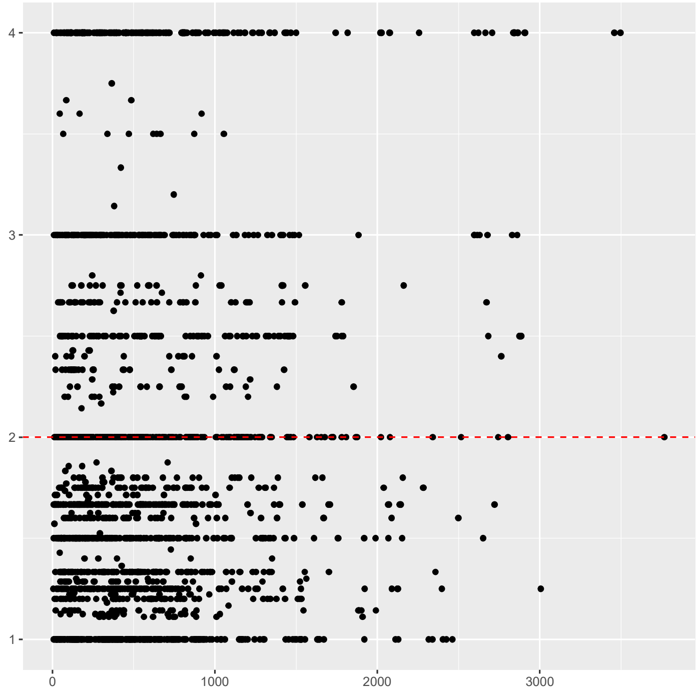
1
2
3
4
0
1000
2000
3000

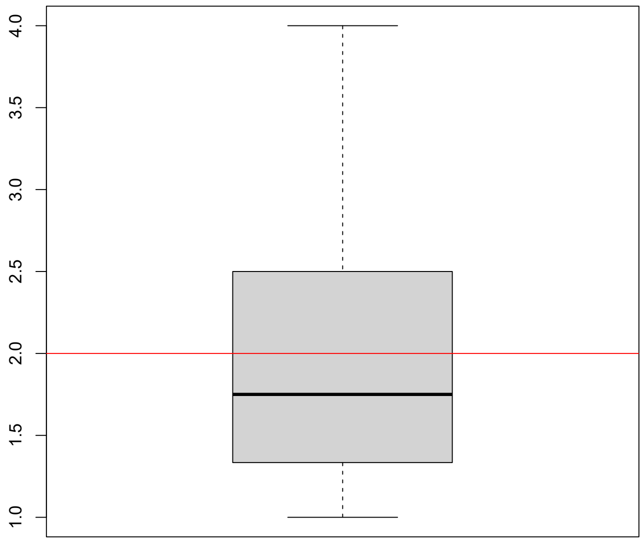
1.0
1.5
2.0
2.5
3.0
3.5
4.0

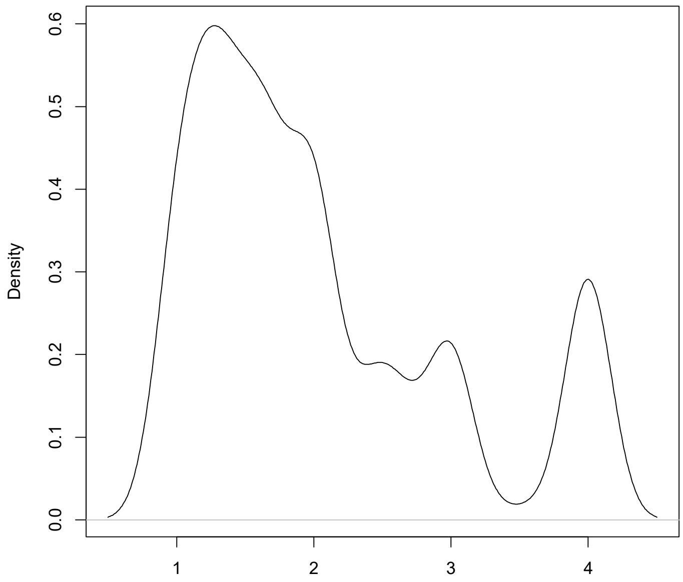
Density
0.0
0.1
0.2
0.3
0.4
0.5
0.6
1 2 3 4

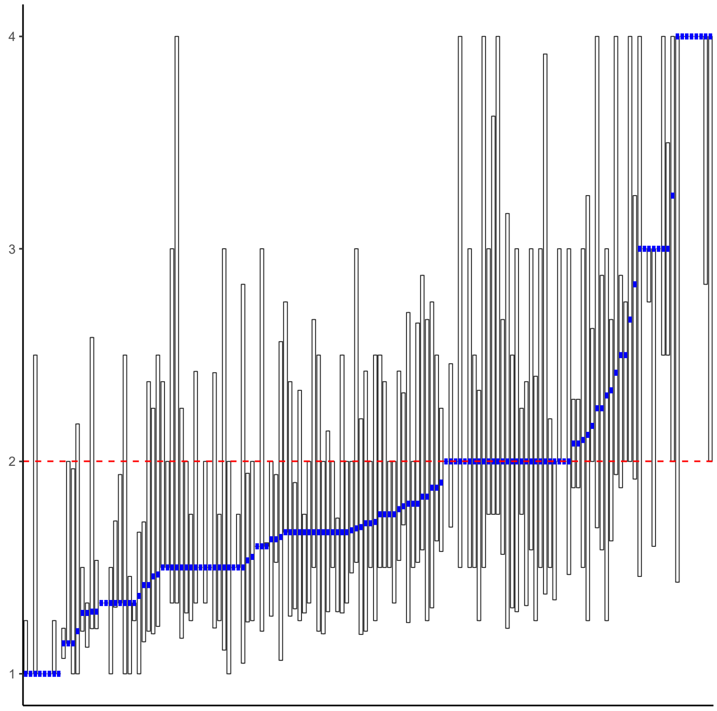
1
2
3
4

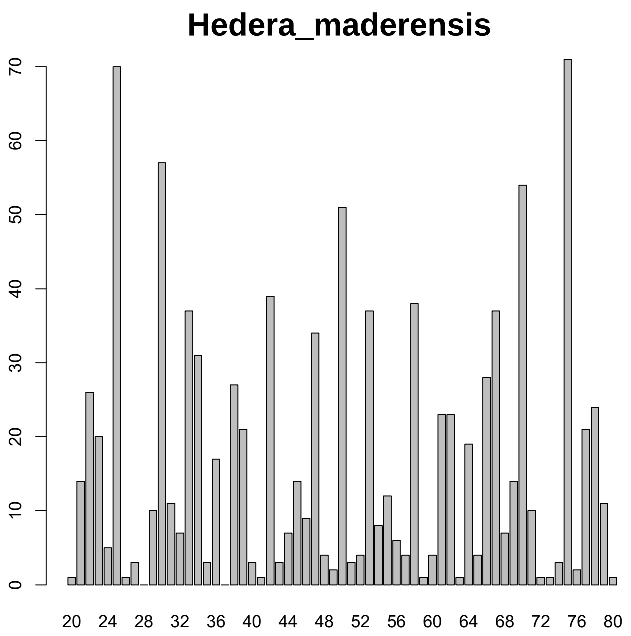
Hedera_maderensis
0
10
20
30
40
50
60
70
20
24
28
32
36
40
44
48
52
56
60
64
68
72
76
80

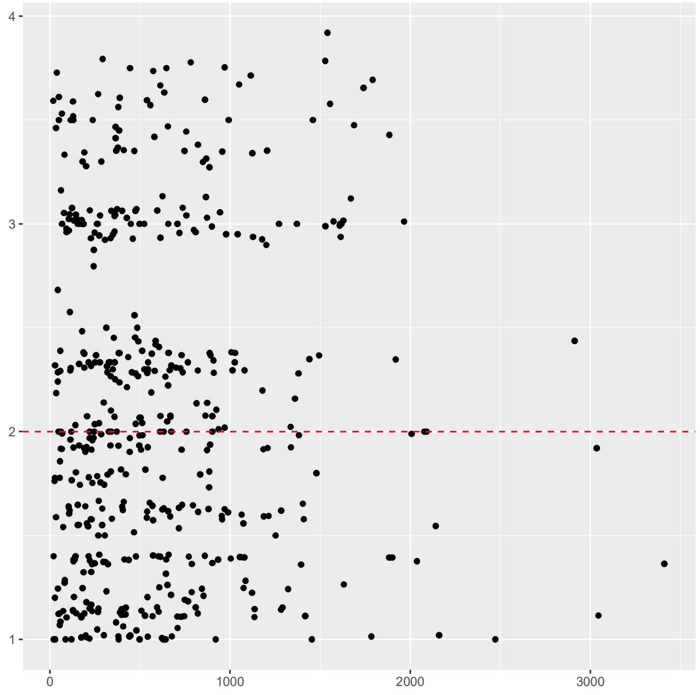
1
2
3
4
0
1000
2000
3000

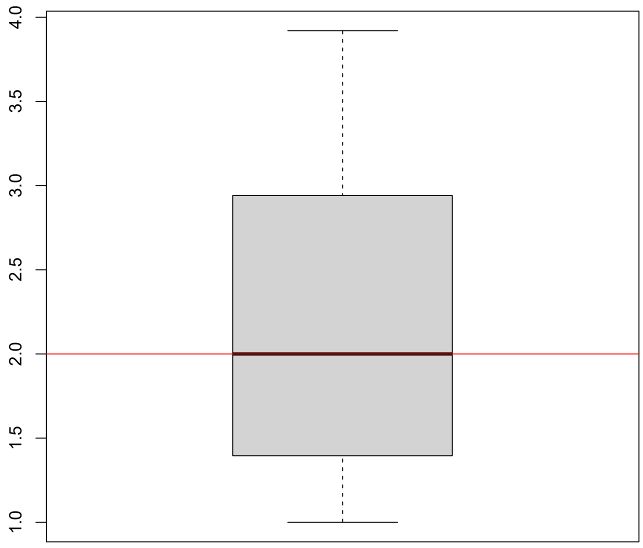
1.0
1.5
2.0
2.5
3.0
3.5
4.0

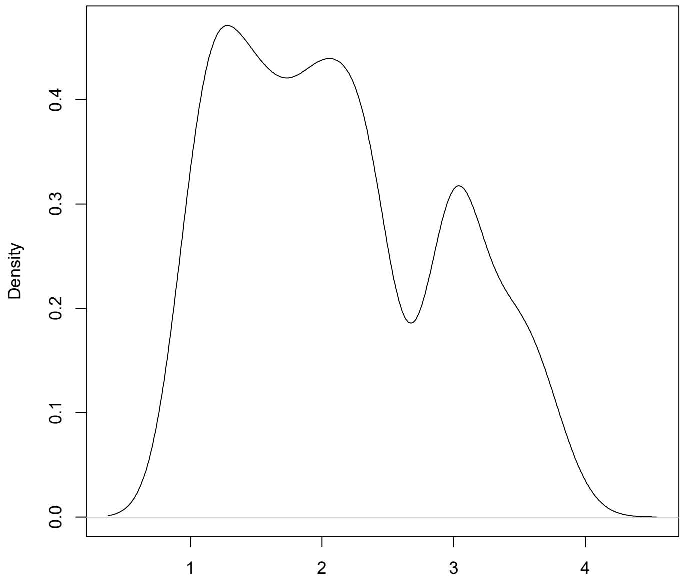
Density
0.0
0.1
0.2
0.3
0.4
1
2
3
4

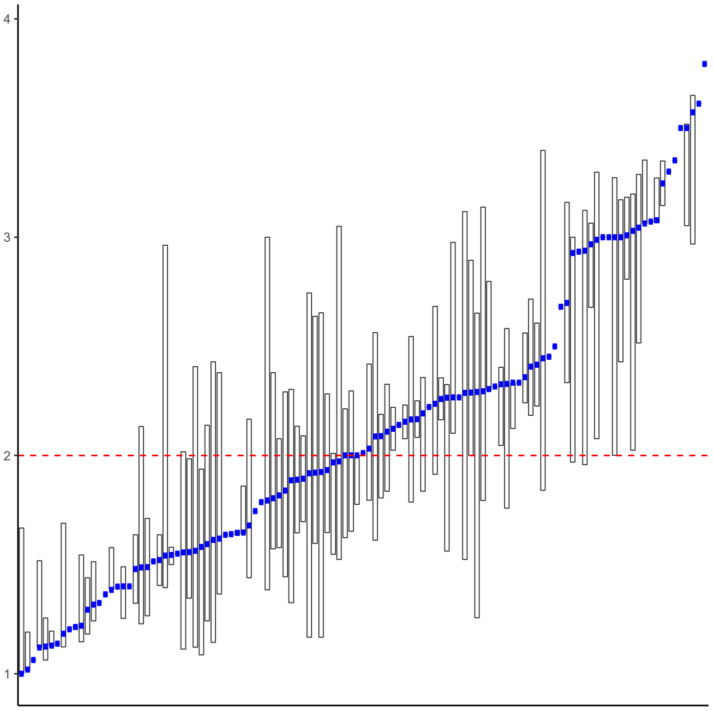
1
2
3
4

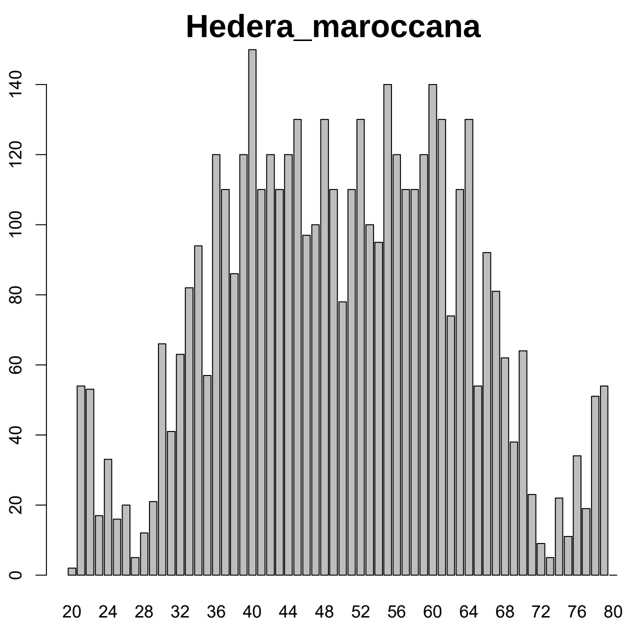
Hedera_maroccana
0
20
40
60
80
100
120
140
20 24 28 32 36 40 44 48 52 56 60 64 68 72 76 80

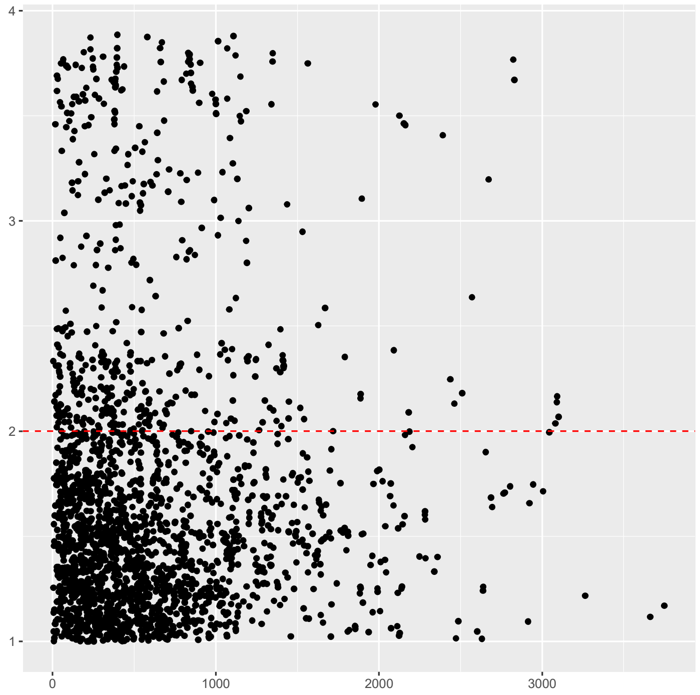
1
2
3
4
0
1000
2000
3000

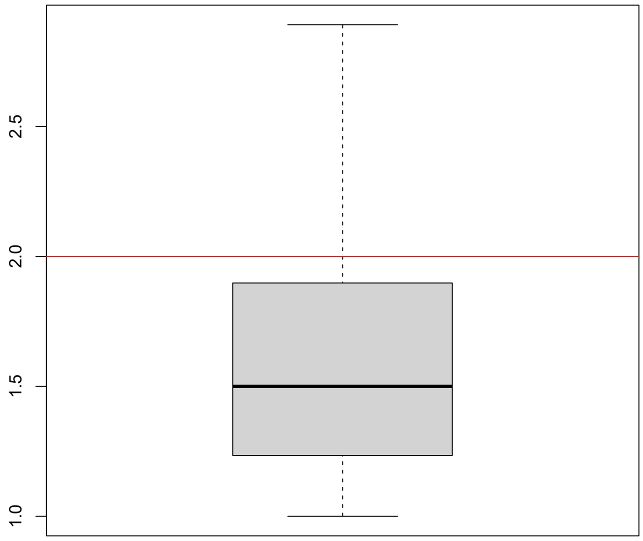
1.0
1.5
2.0
2.5

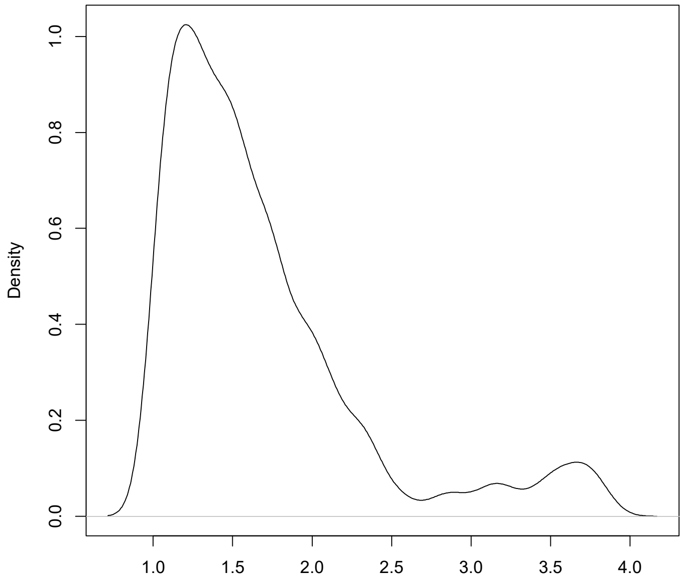
Density
0.0
0.2
0.4
0.6
0.8
1.0
1.0 1.5 2.0 2.5 3.0 3.5 4.0

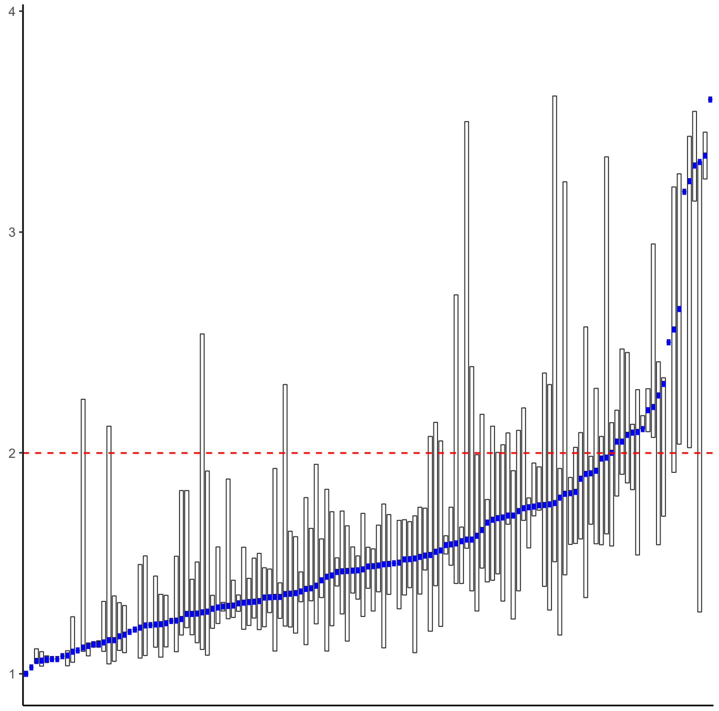
1
2
3
4

Hedera_nepalensis_nepalensis

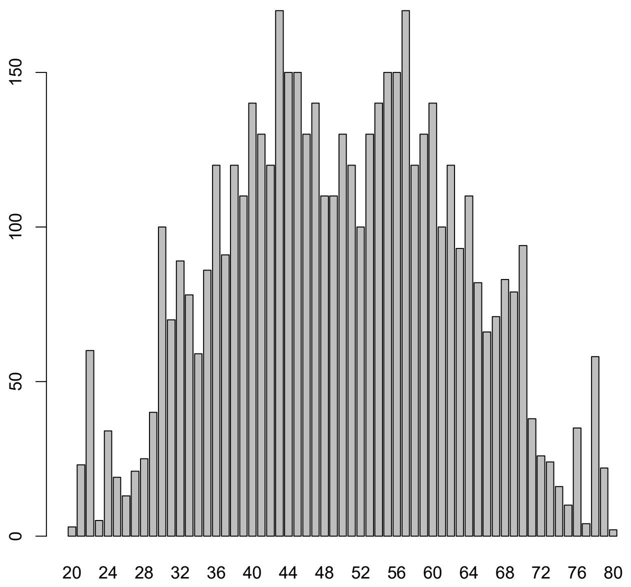

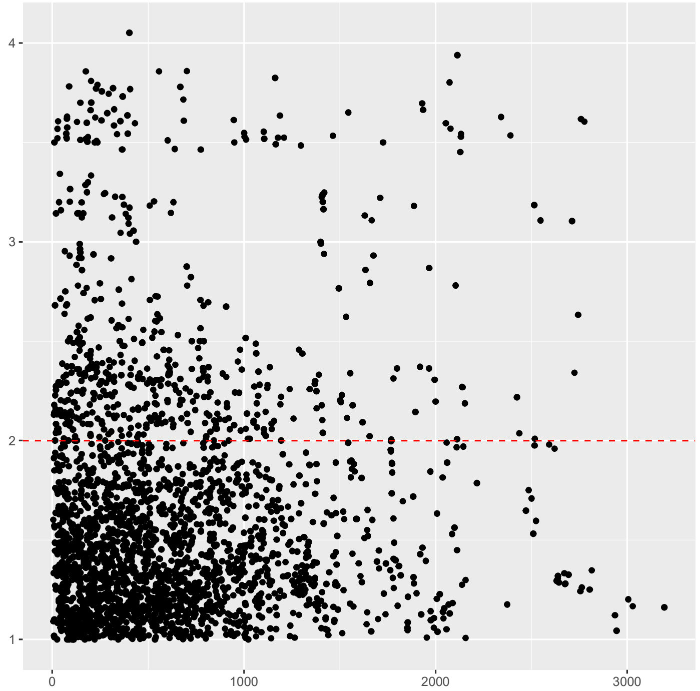

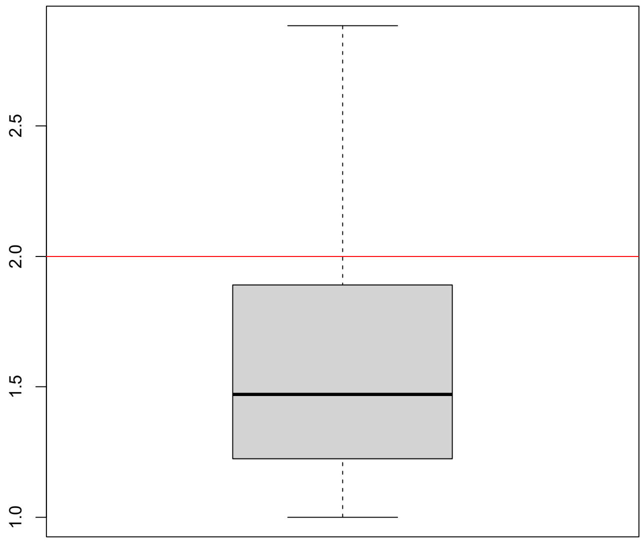

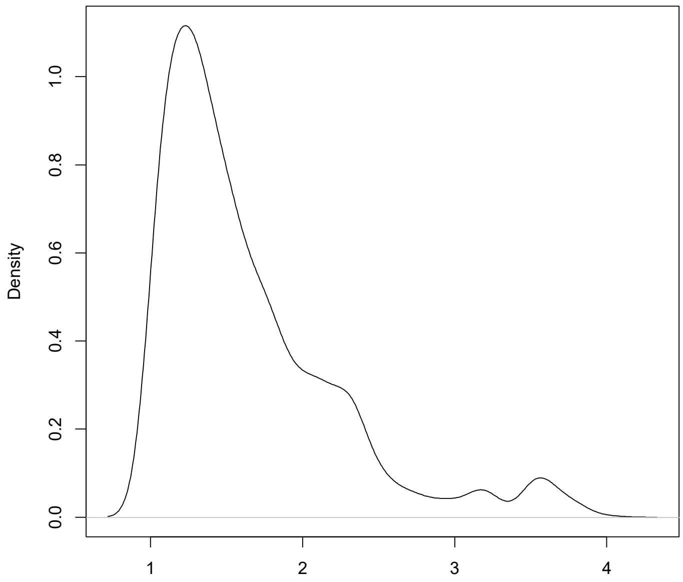

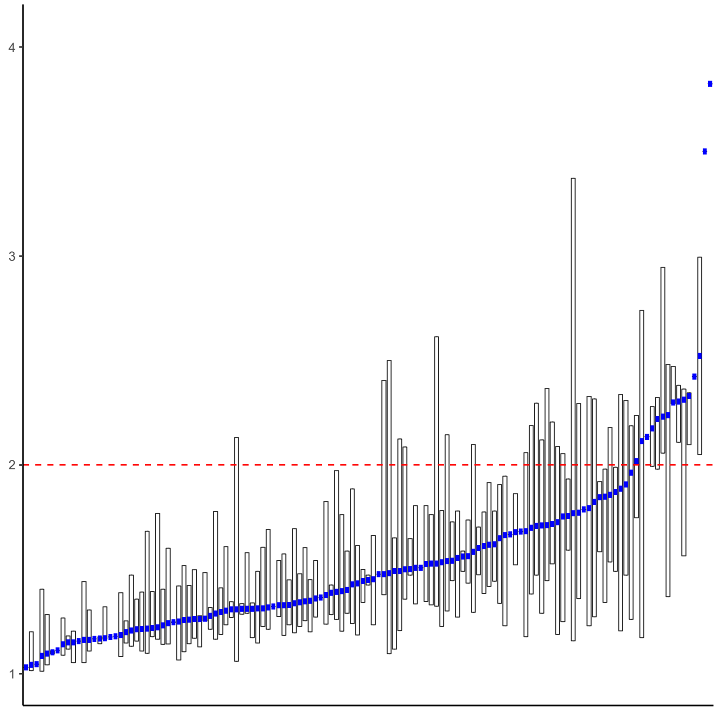

Hedera_nepalensis_sinensis

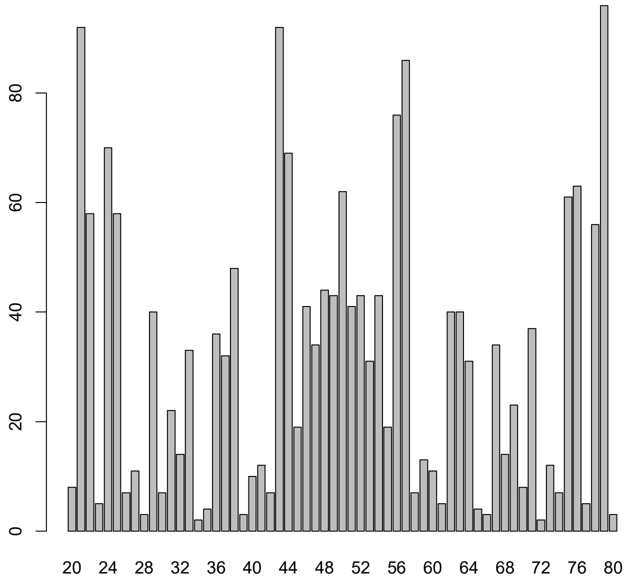

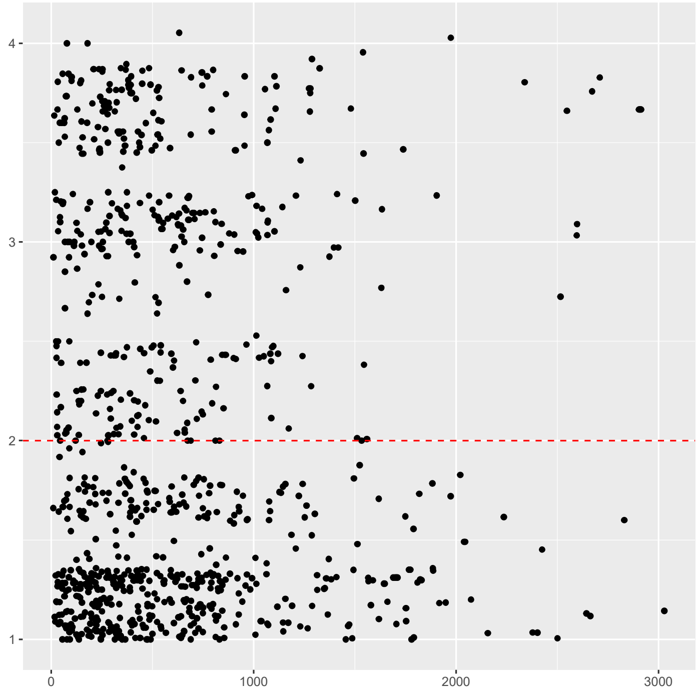

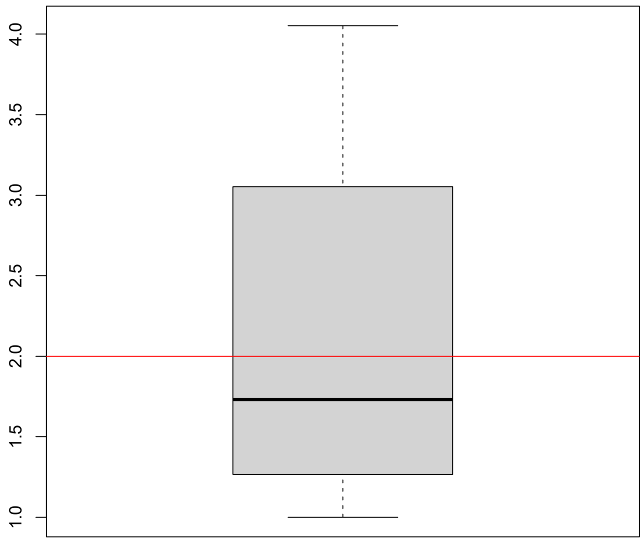

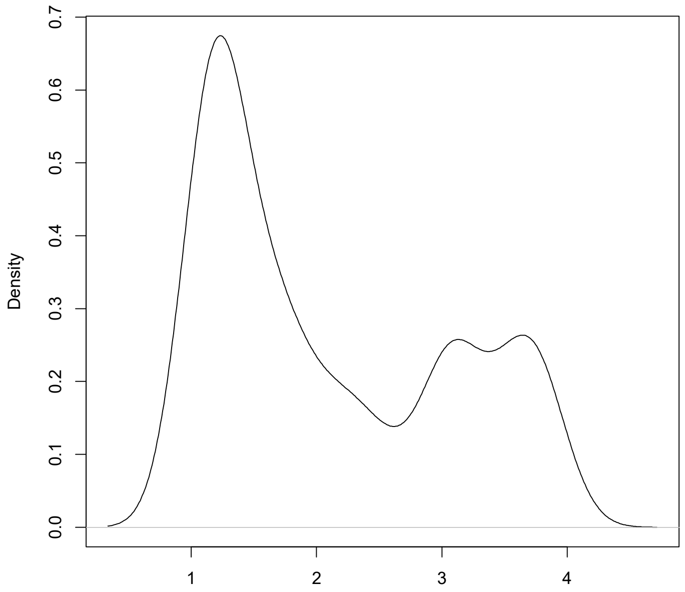

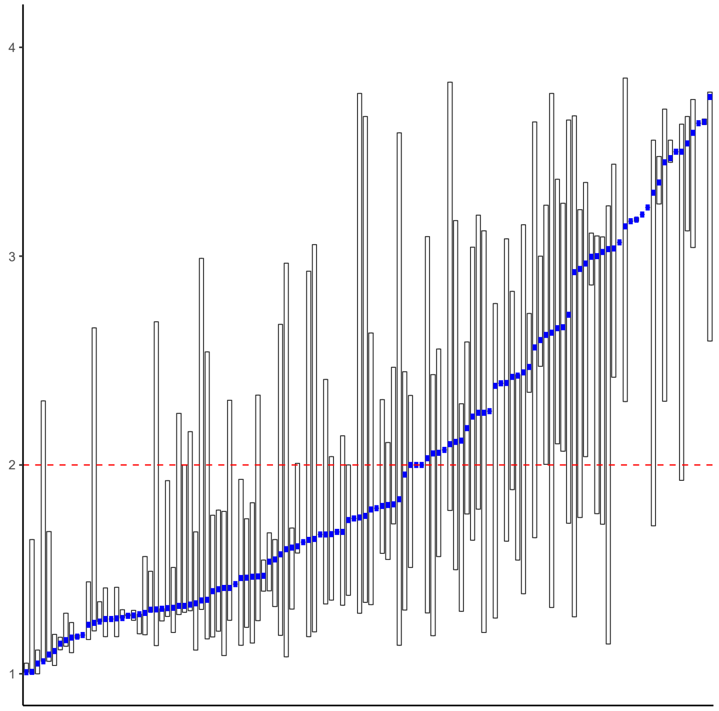

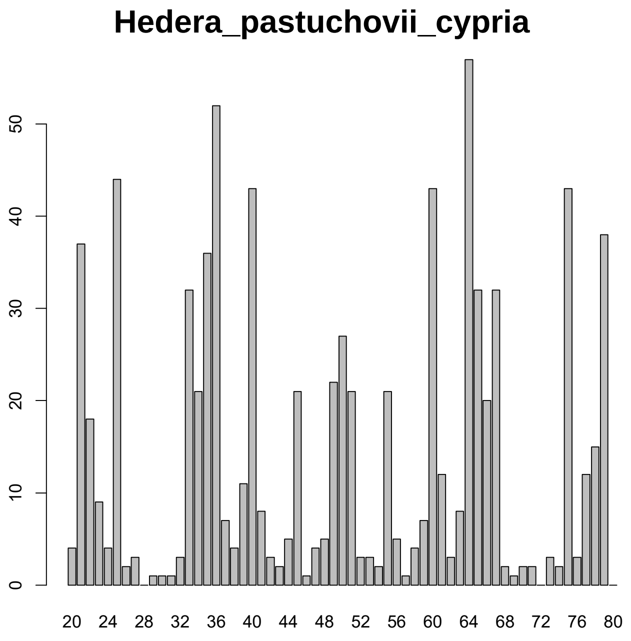
Hedera_pastuchovii_cypria
0
10
20
30
40
50
20 24 28 32 36 40 44 48 52 56 60 64 68 72 76 80

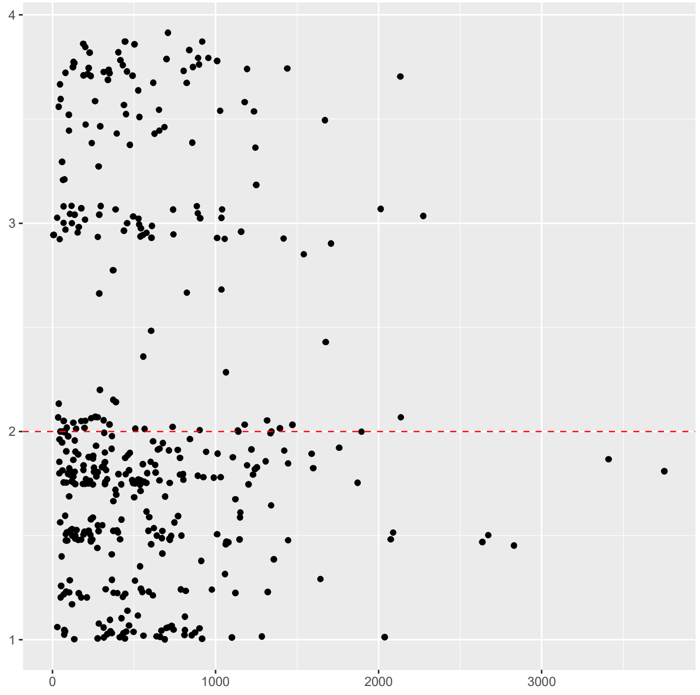
1
2
3
4
0 1000 2000 3000

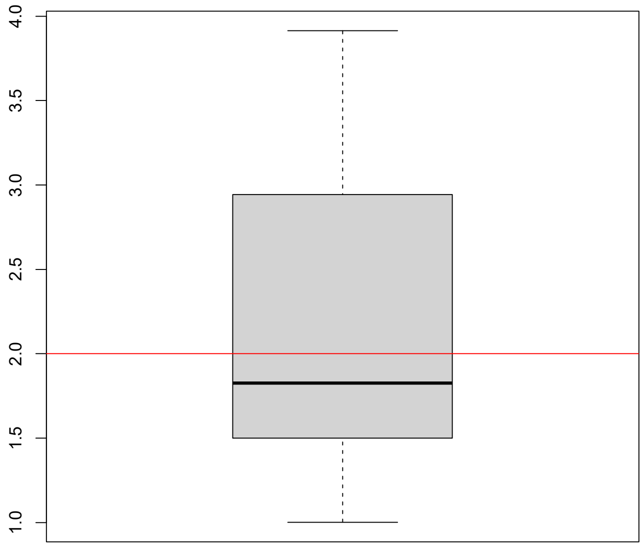
1.0
1.5
2.0
2.5
3.0
3.5
4.0

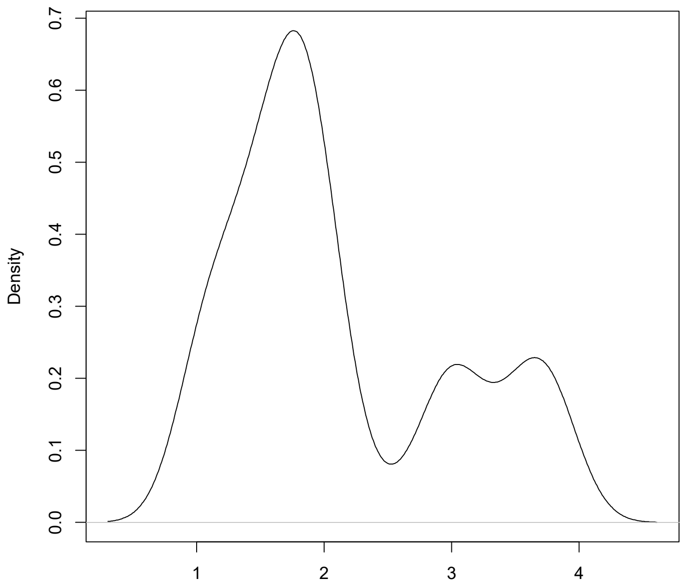
Density
0.0
0.1
0.2
0.3
0.4
0.5
0.6
0.7
1 2 3 4

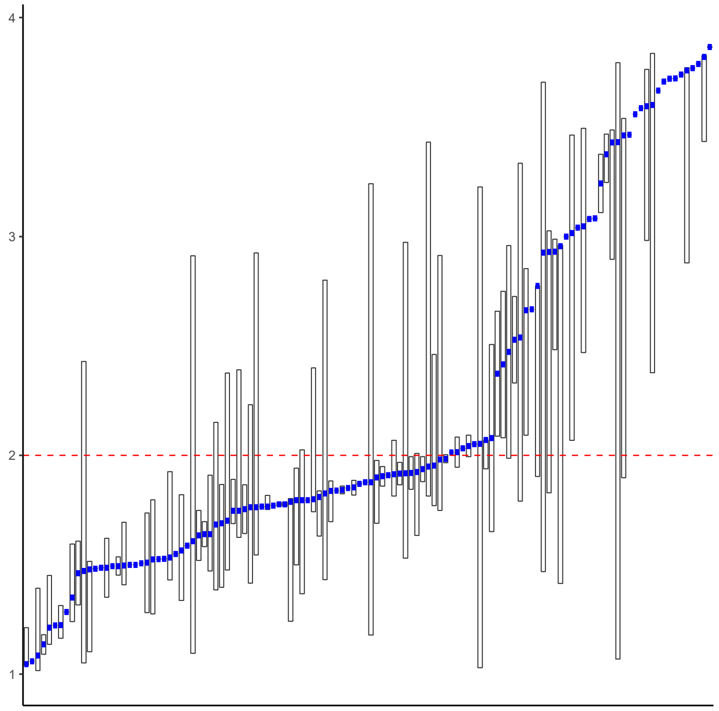
1
2
3
4

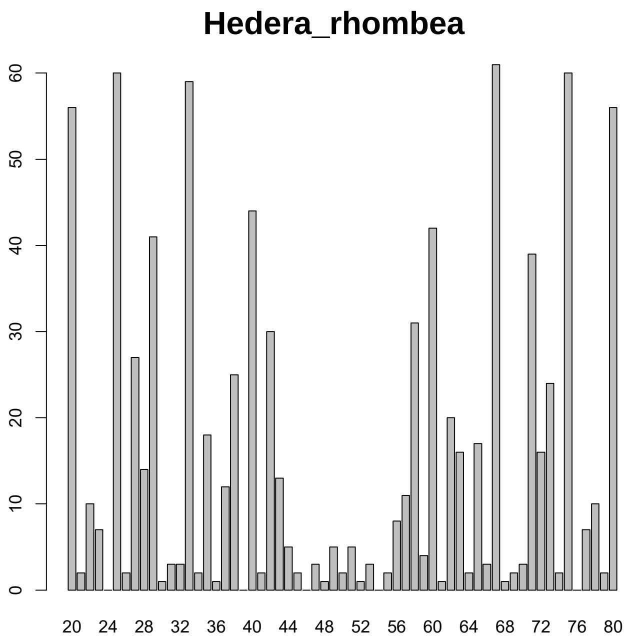
Hedera_rhombea
0
10
20
30
40
50
60
20
24
28
32
36
40
44
48
52
56
60
64
68
72
76
80

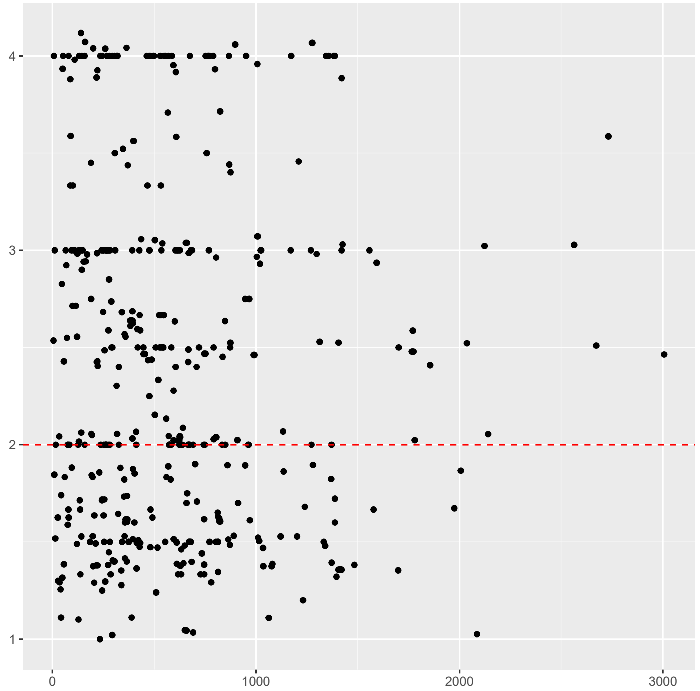
1
2
3
4
0
1000
2000
3000

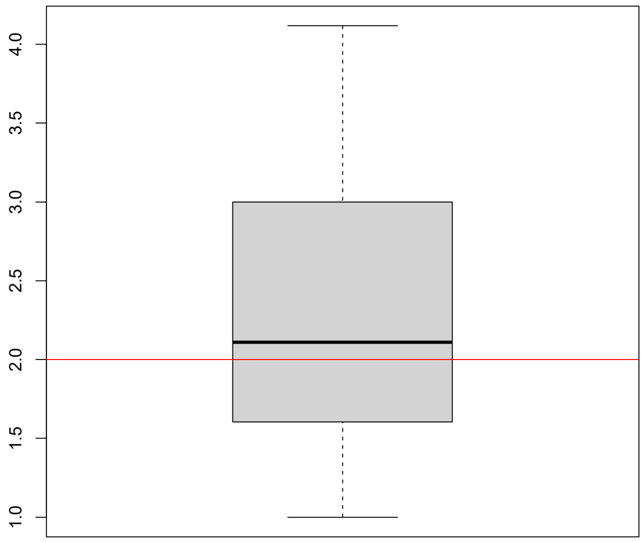
1.0
1.5
2.0
2.5
3.0
3.5
4.0

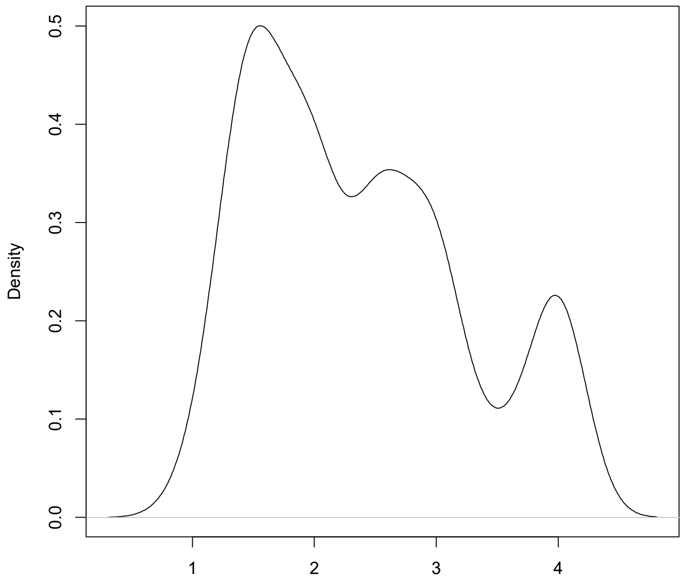
Density
0.0
0.1
0.2
0.3
0.4
0.5
1
2
3
4

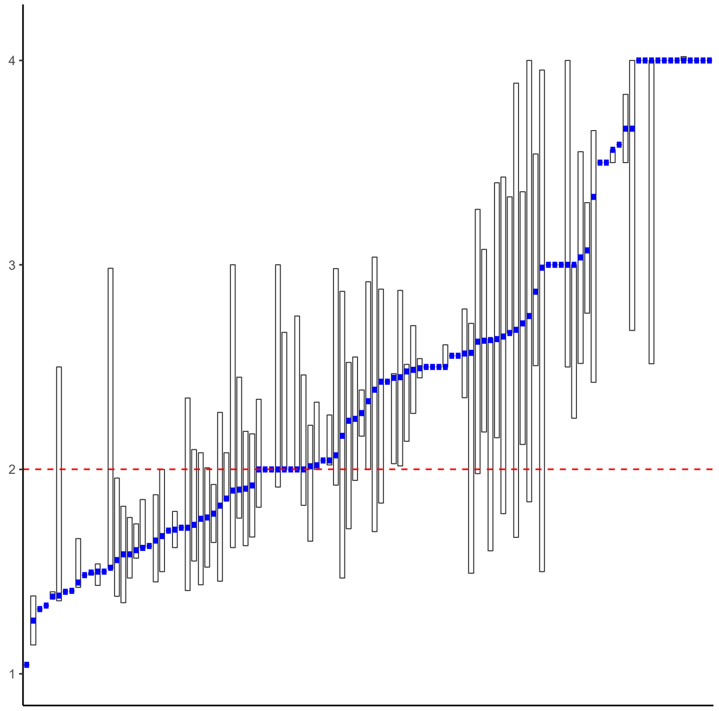
1
2
3
4

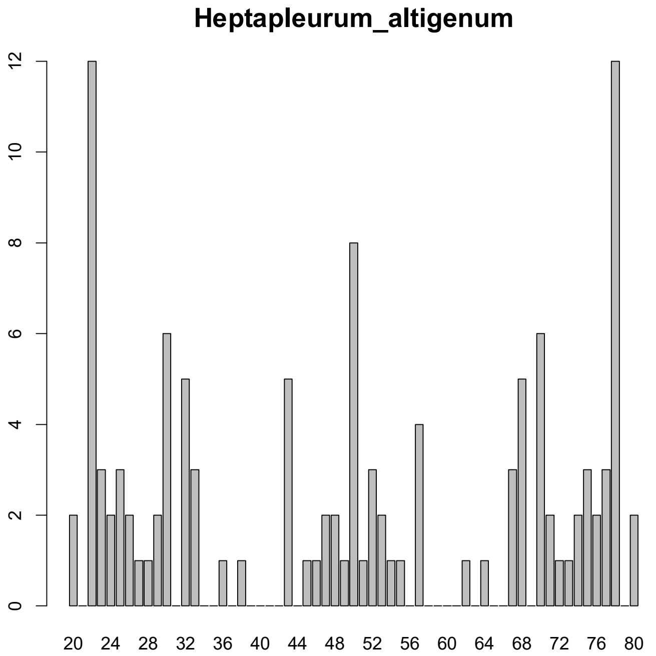
Heptapleurum_altigenum
0
2
4
6
8
10
12
20 24 28 32 36 40 44 48 52 56 60 64 68 72 76 80

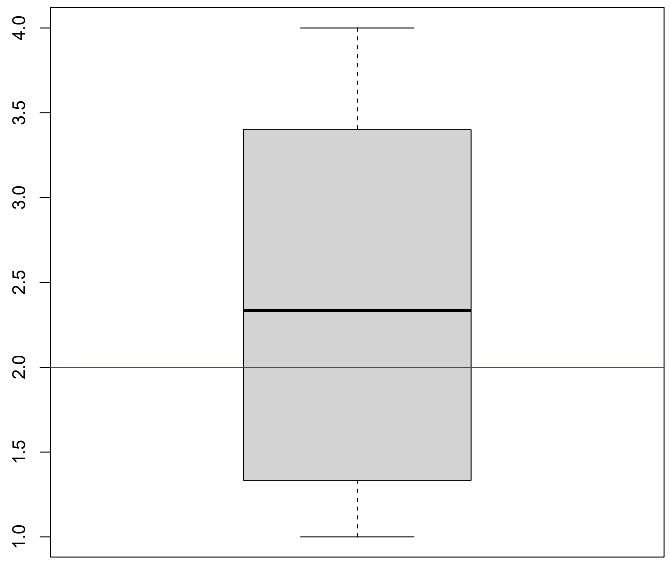
1.0
1.5
2.0
2.5
3.0
3.5
4.0

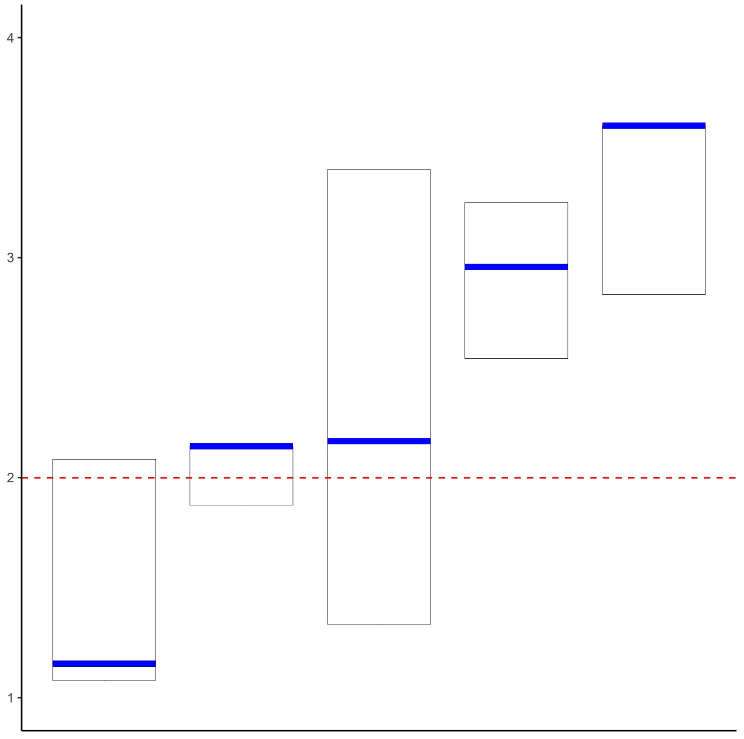
1
2
3
4

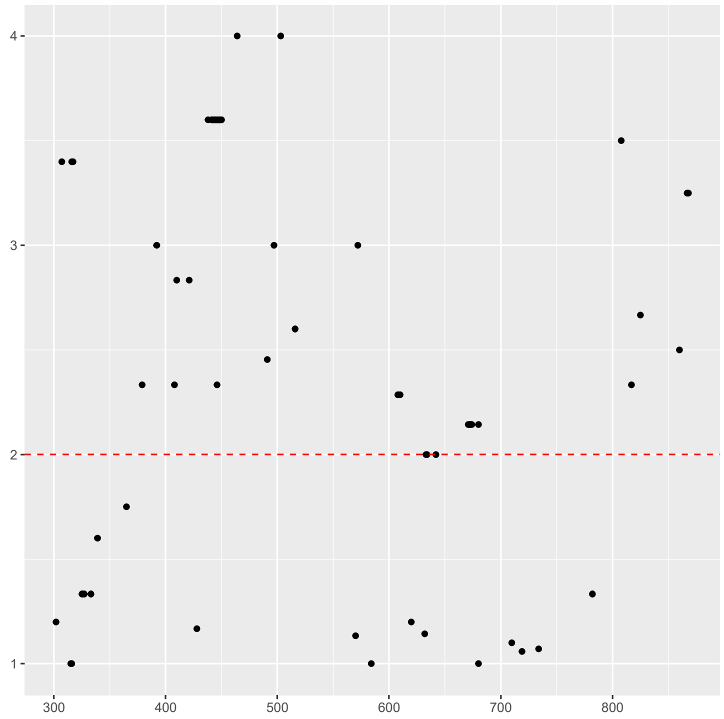
1
2
3
4
300 400 500 600 700 800

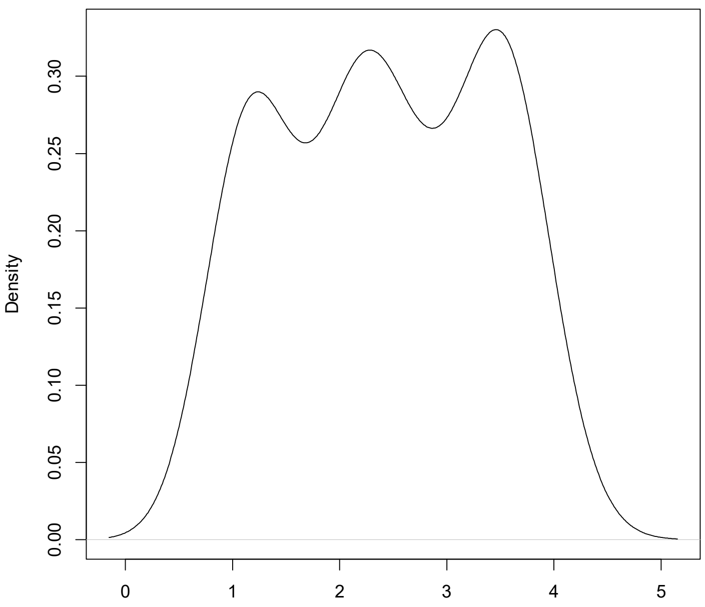
Density
0.00
0.05
0.10
0.15
0.20
0.25
0.30
0 1 2 3 4 5

Heptapleurum_calyptratum

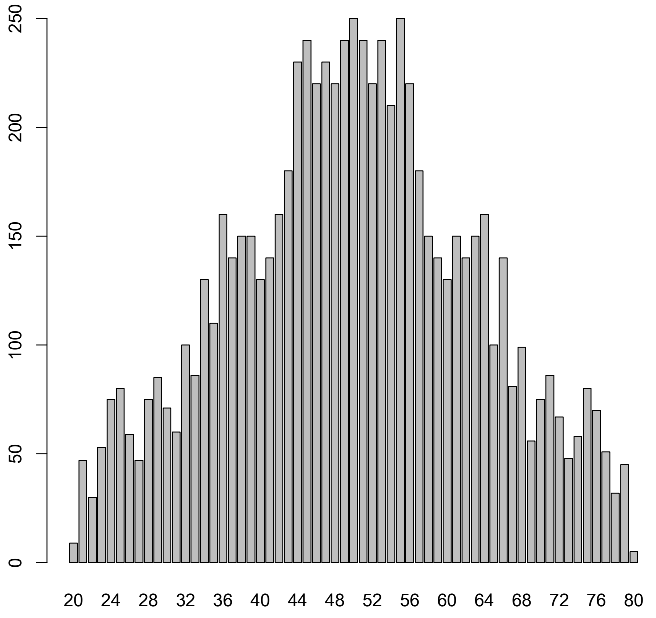

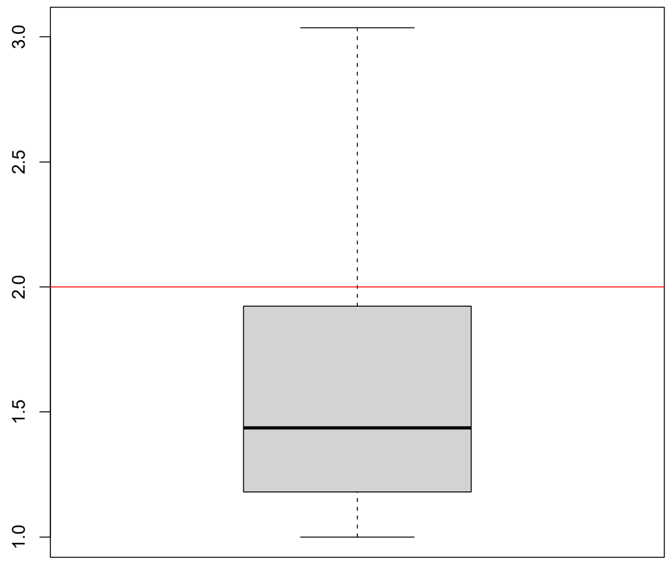

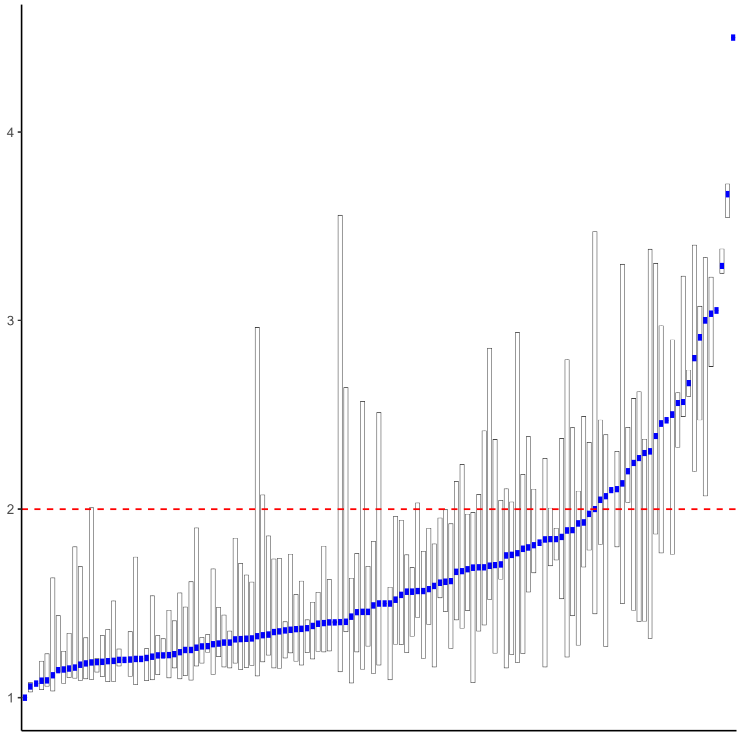

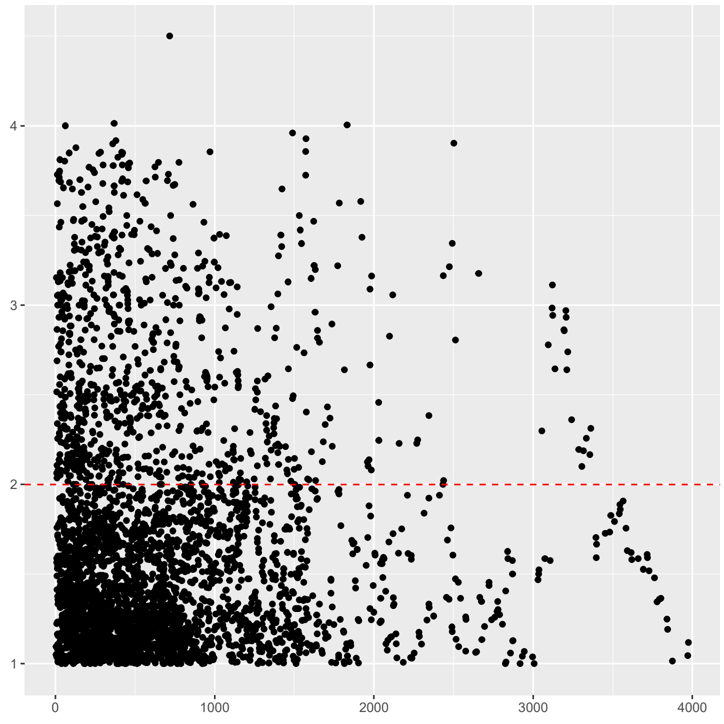

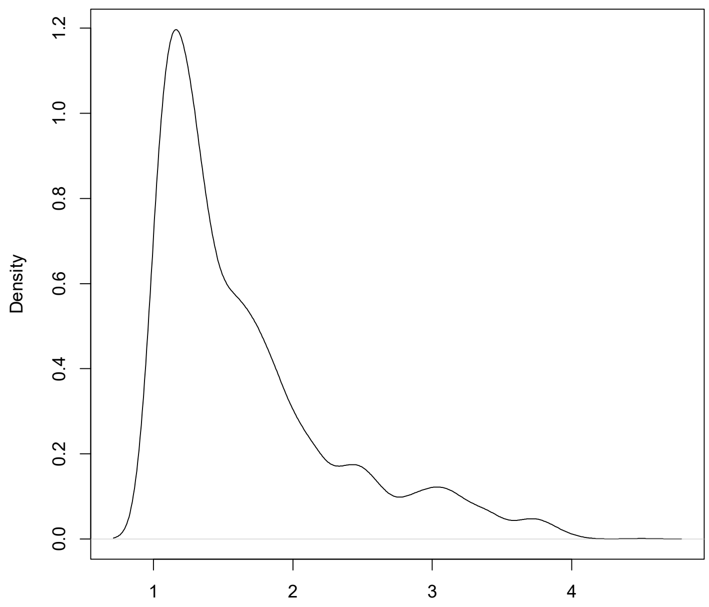

Heptapleurum_delavayi

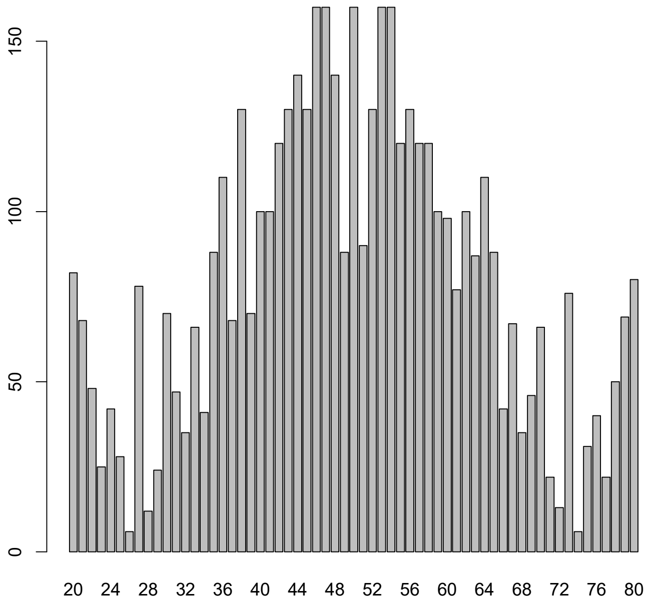

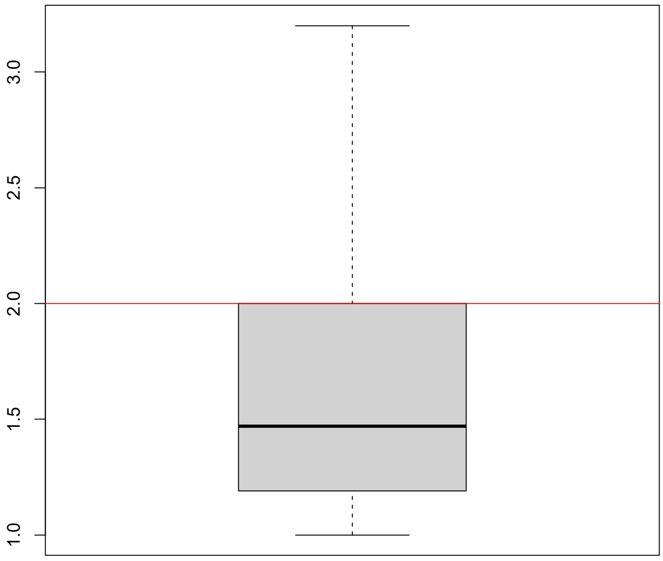

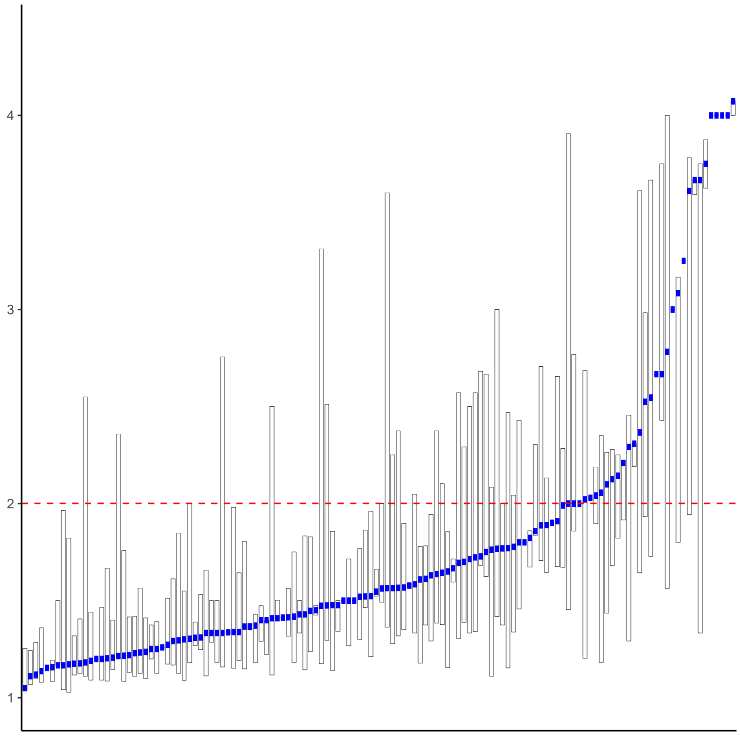

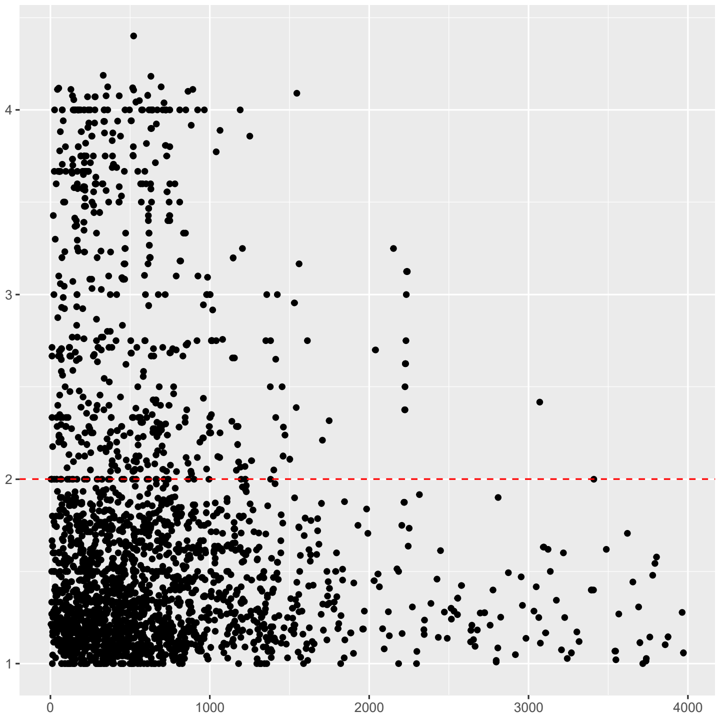

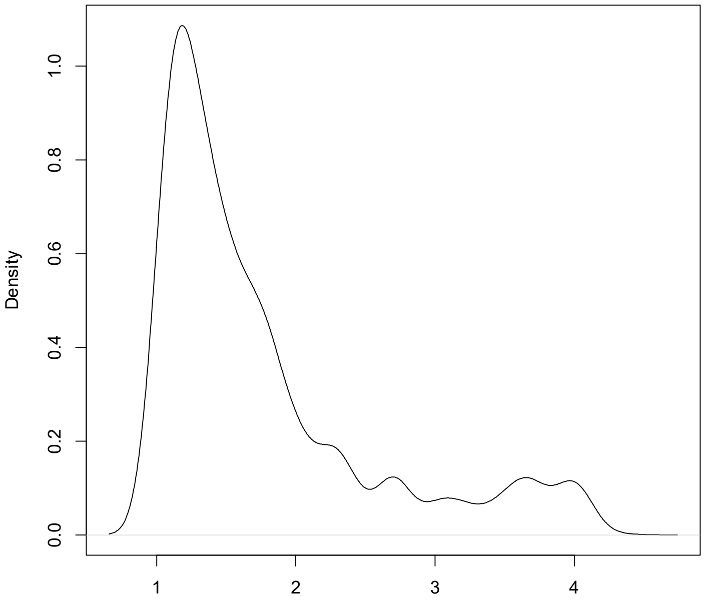

Heptapleurum_forbesii

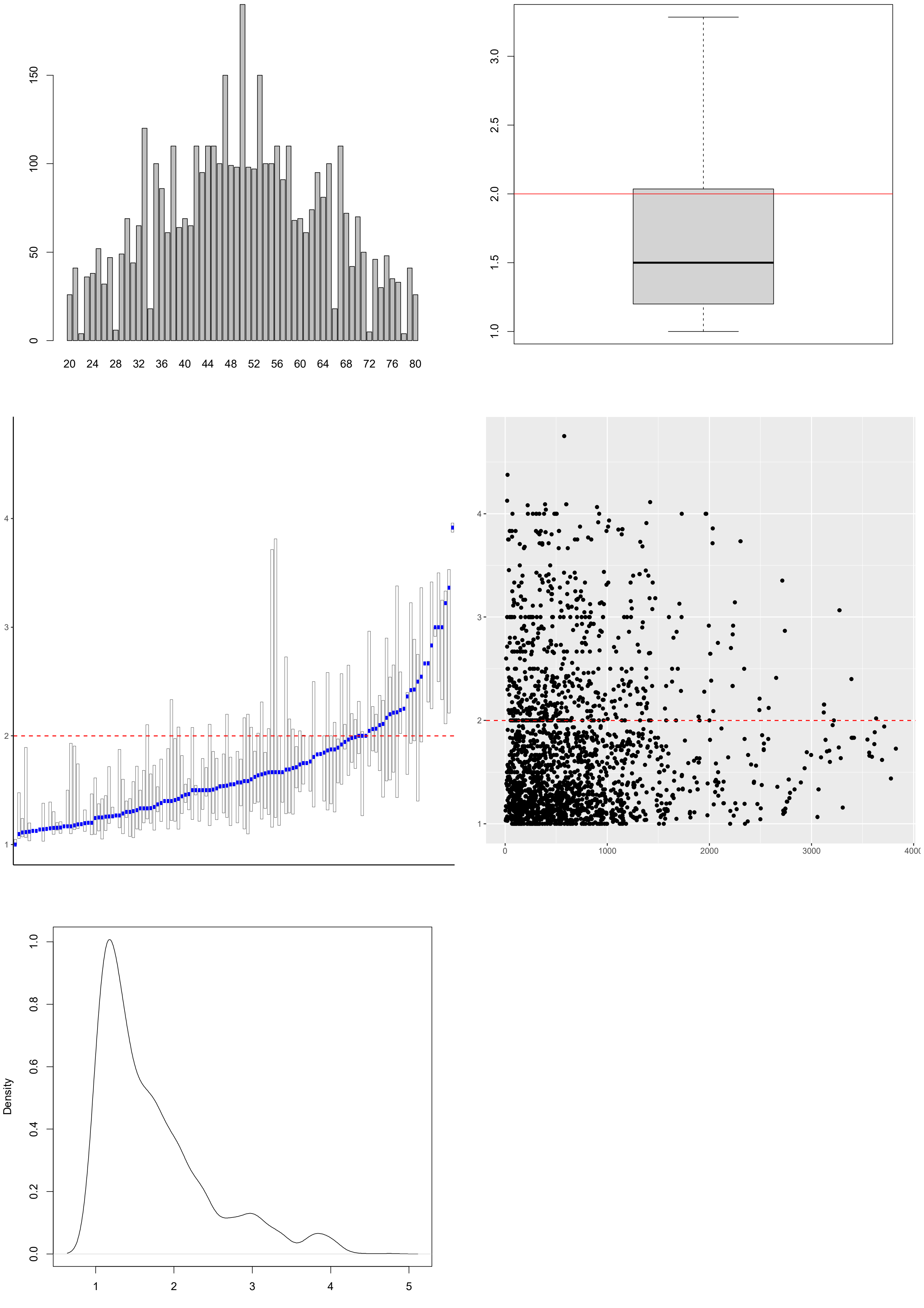

Heptapleurum_heptaphyllum

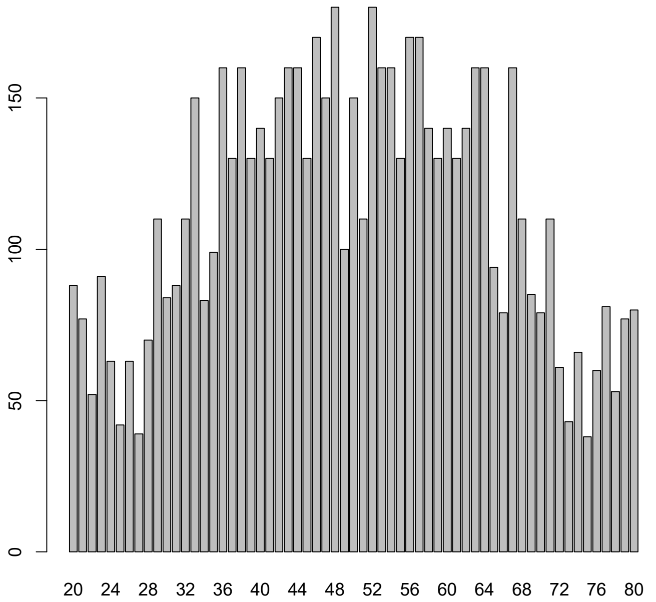

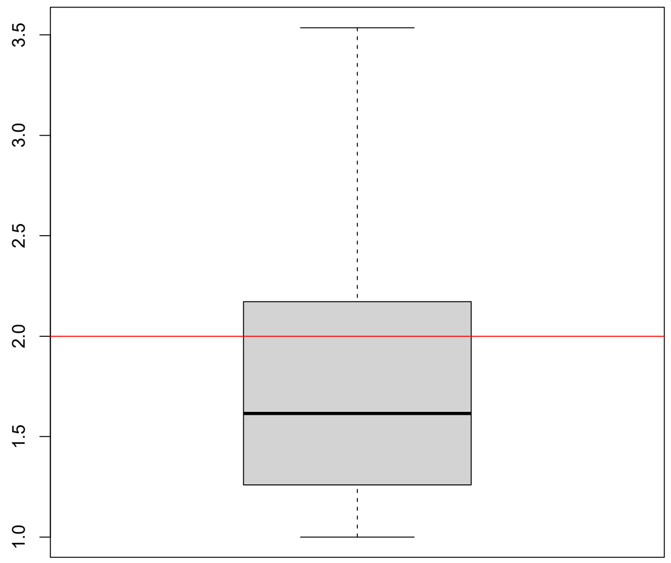

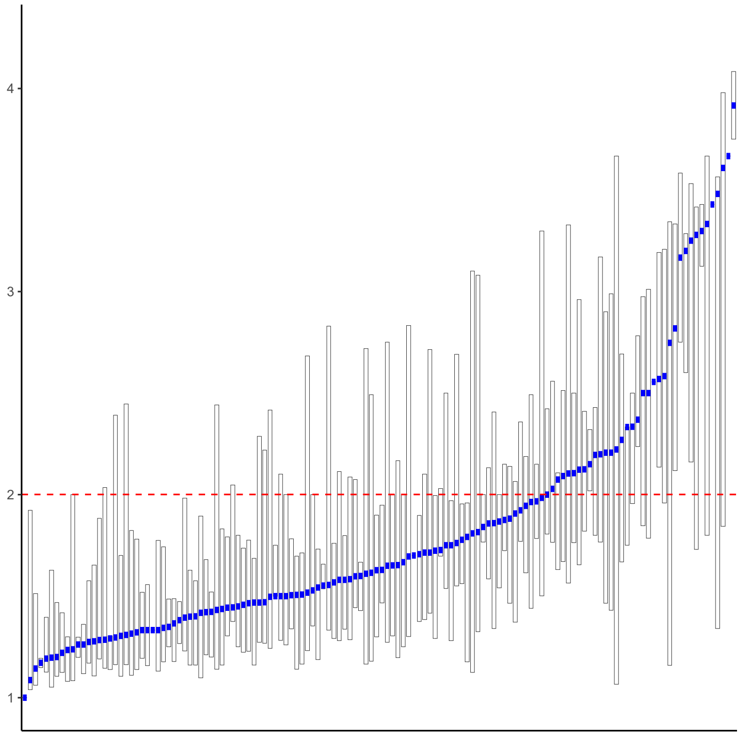

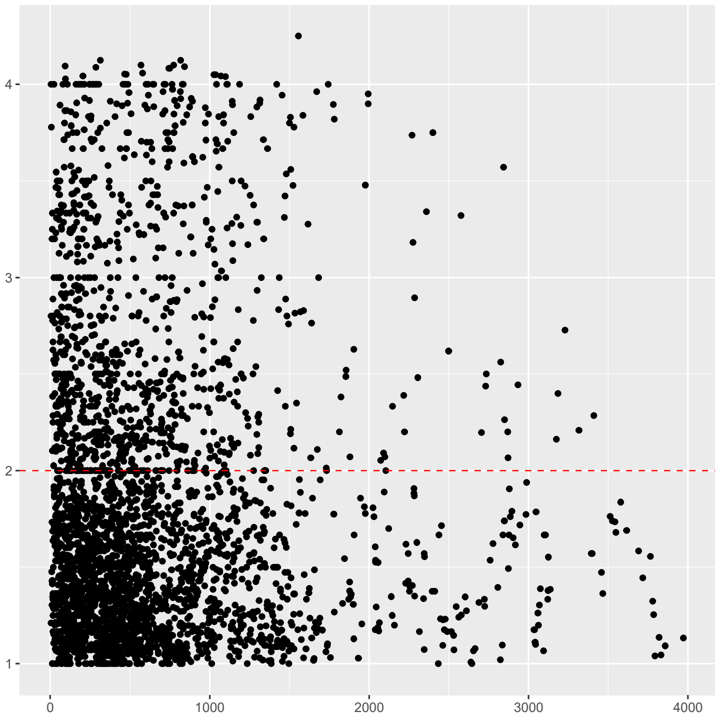

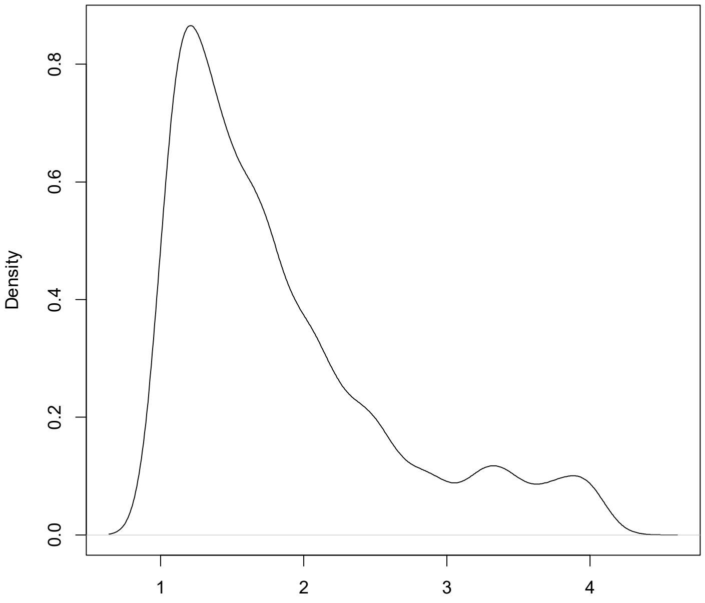

Heptapleurum_heterophyllum

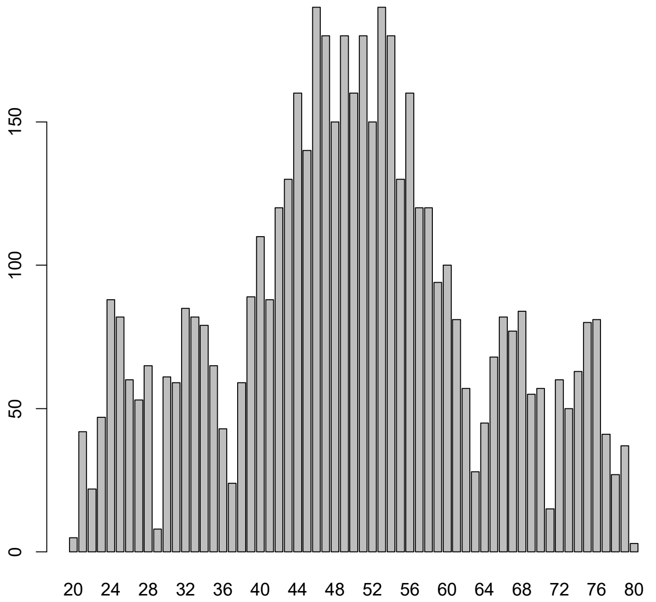

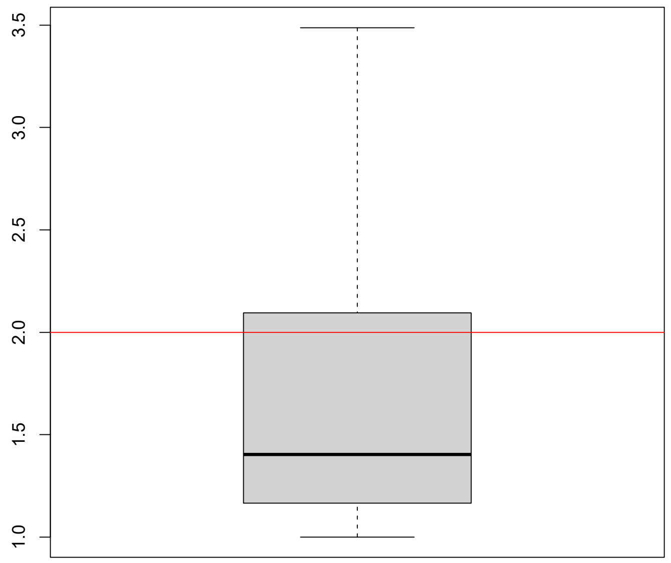

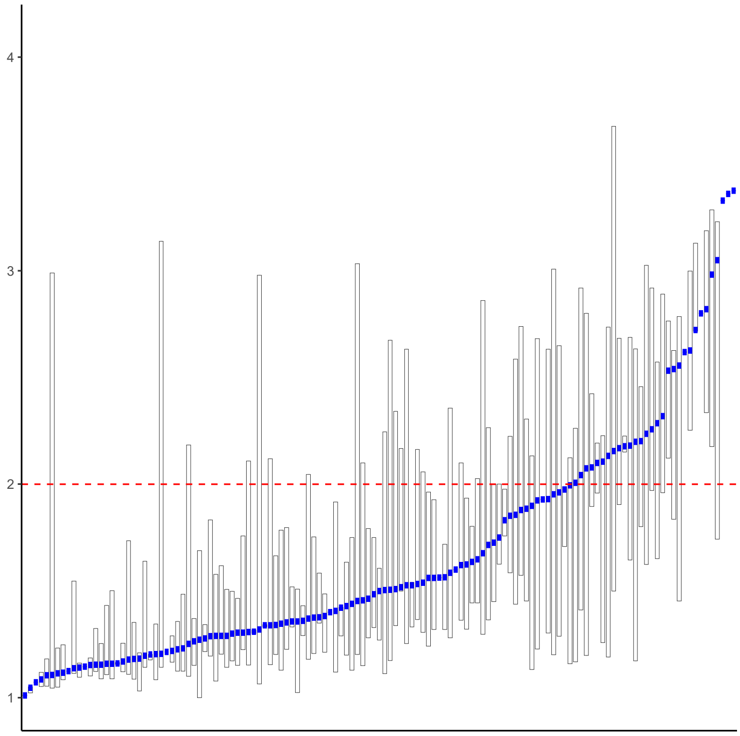

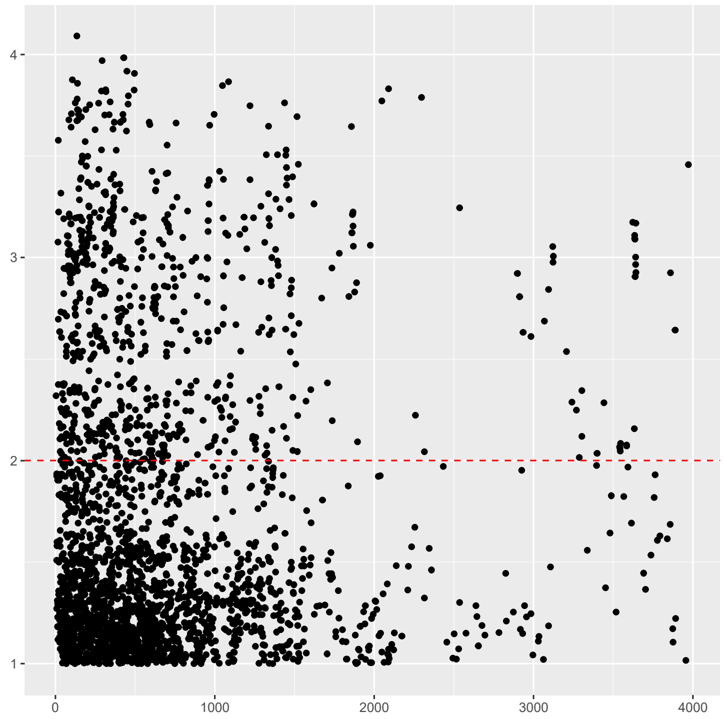

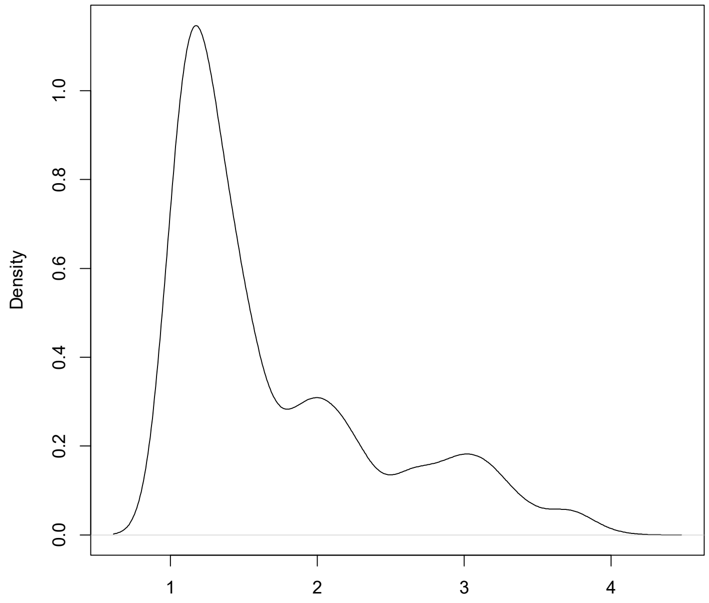

Heptapleurum_ischnoacrum

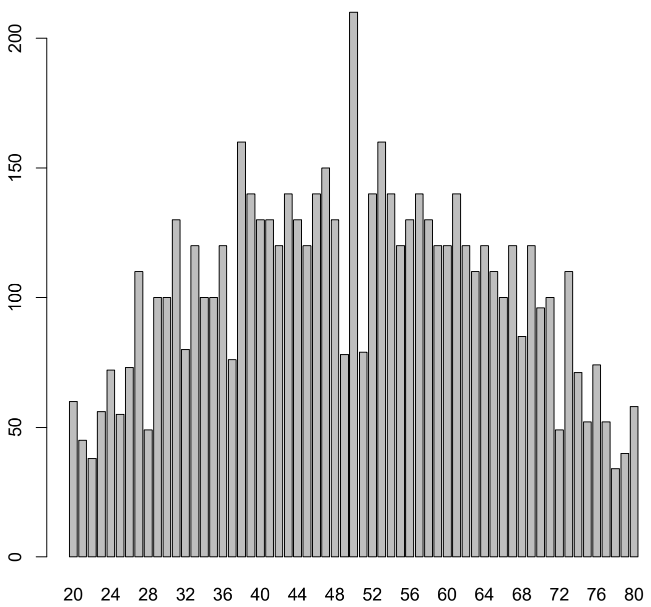

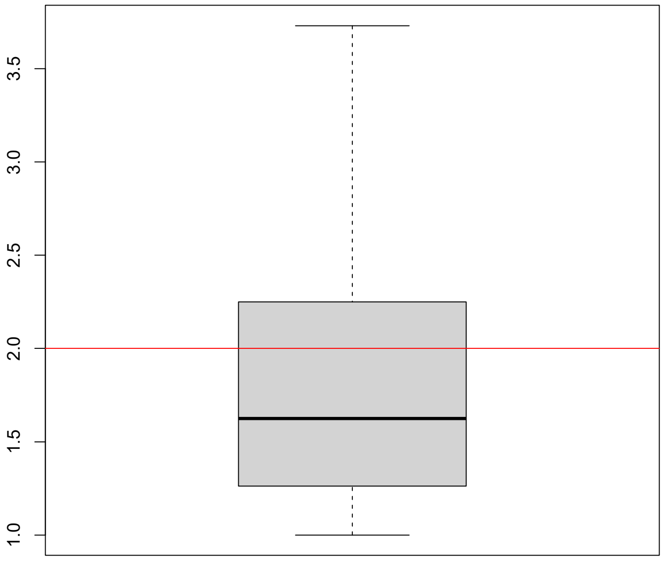

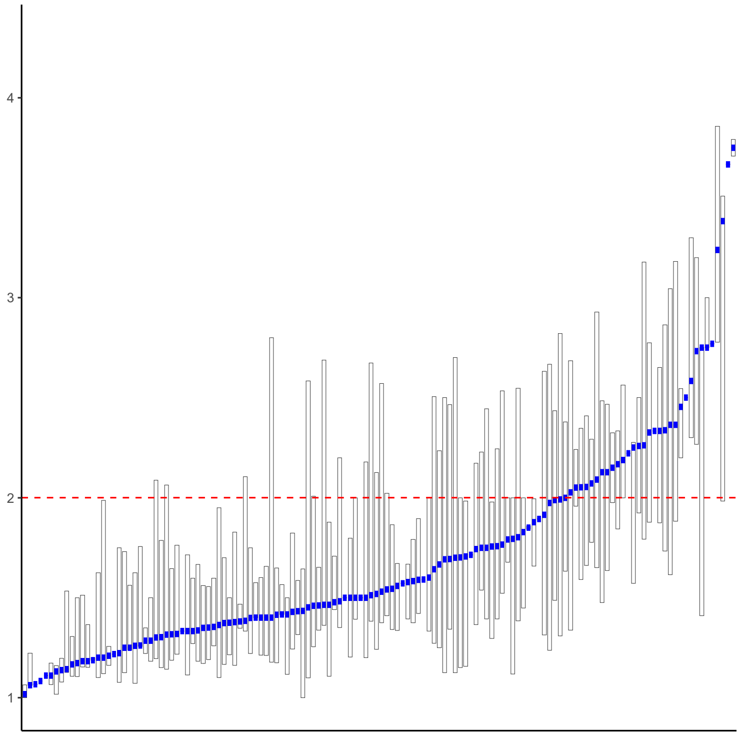

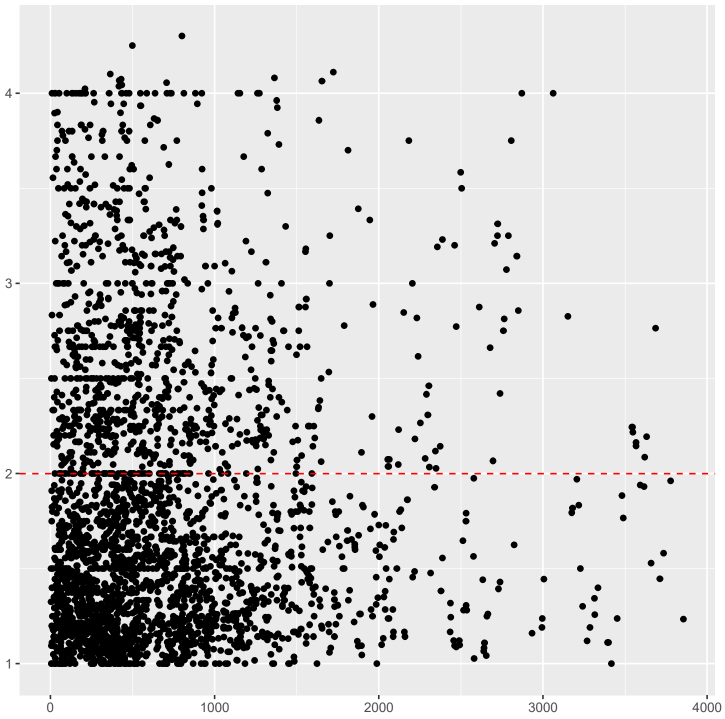

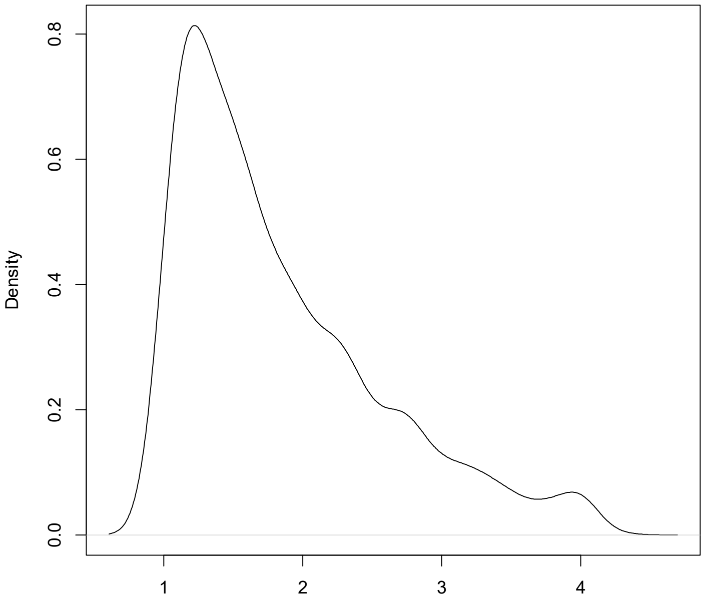

Heptapleurum_kornasii

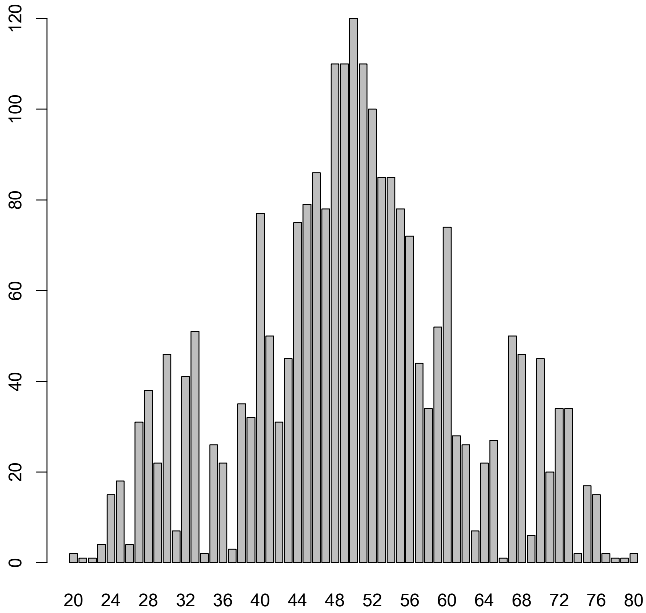

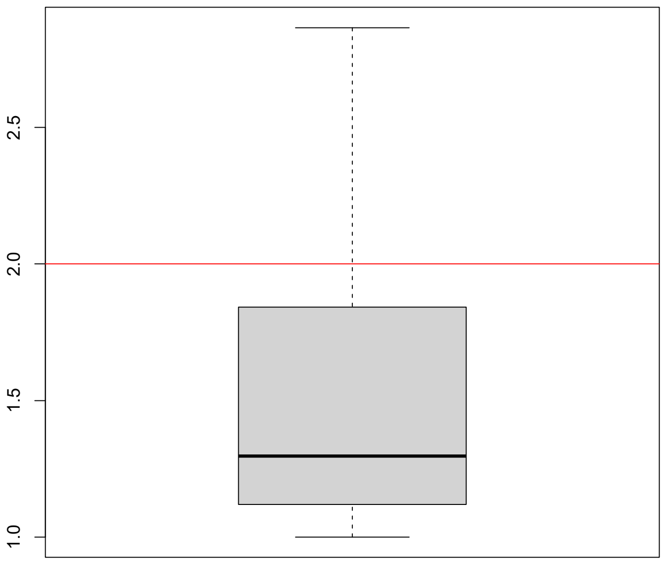

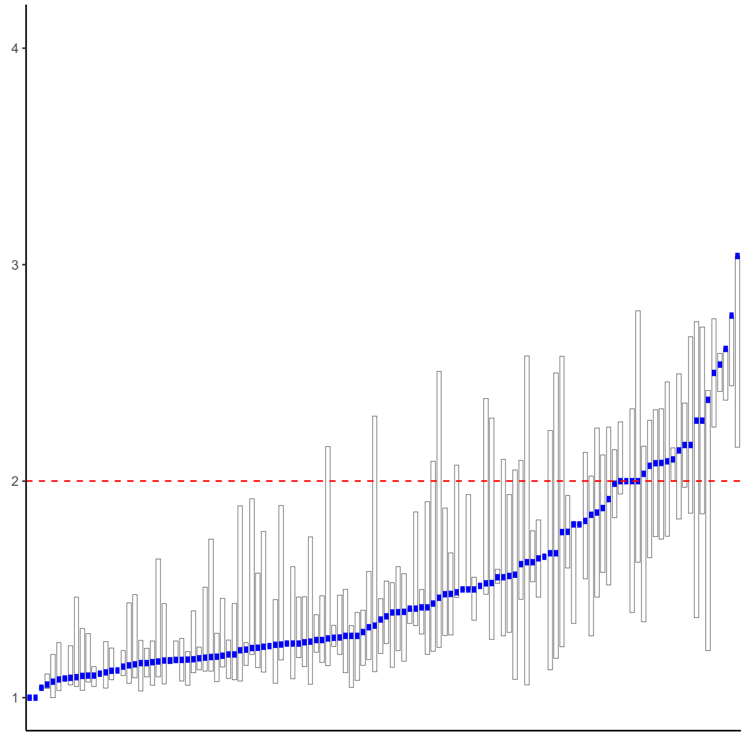

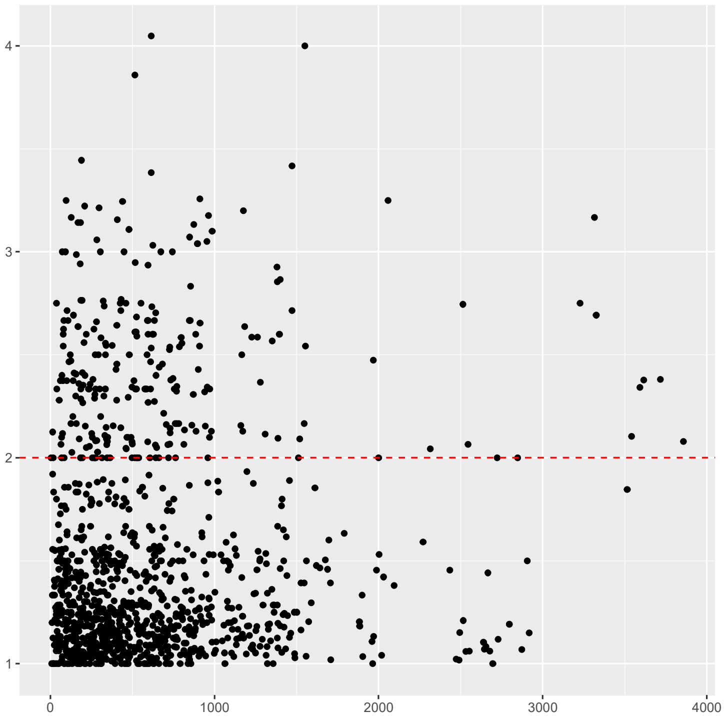

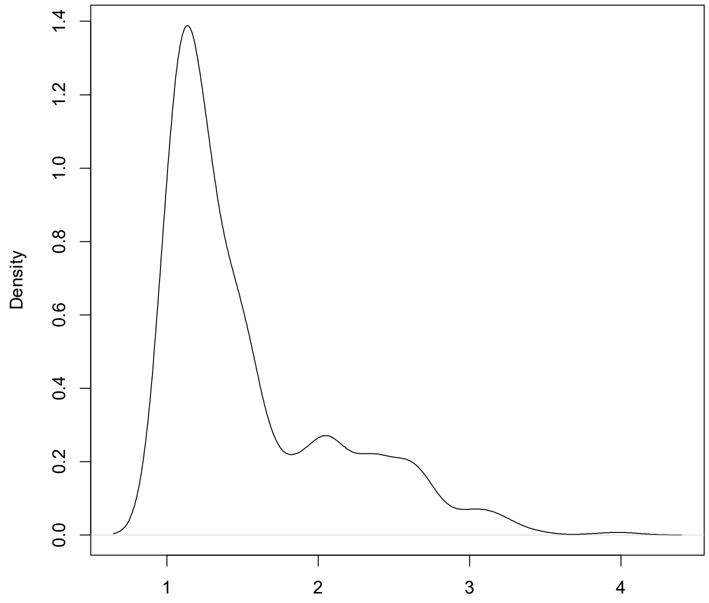

Heptapleurum_minutistellatum

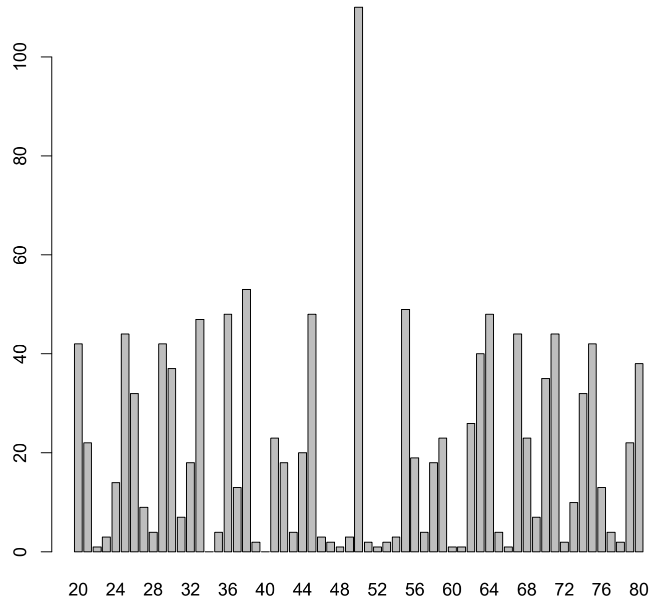

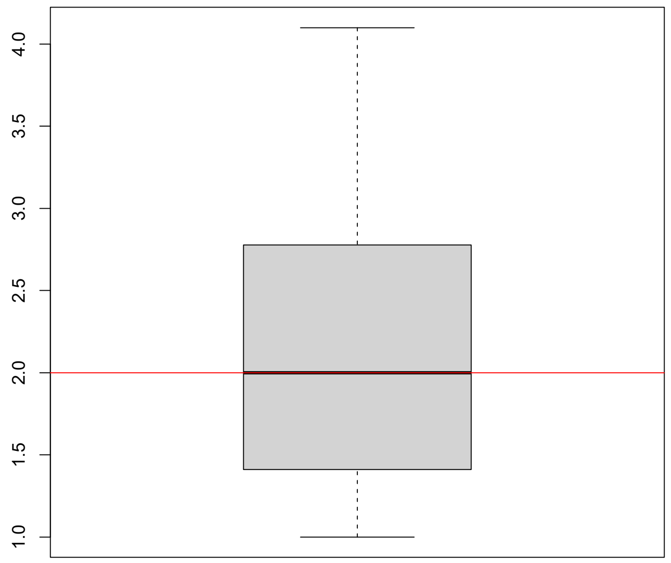

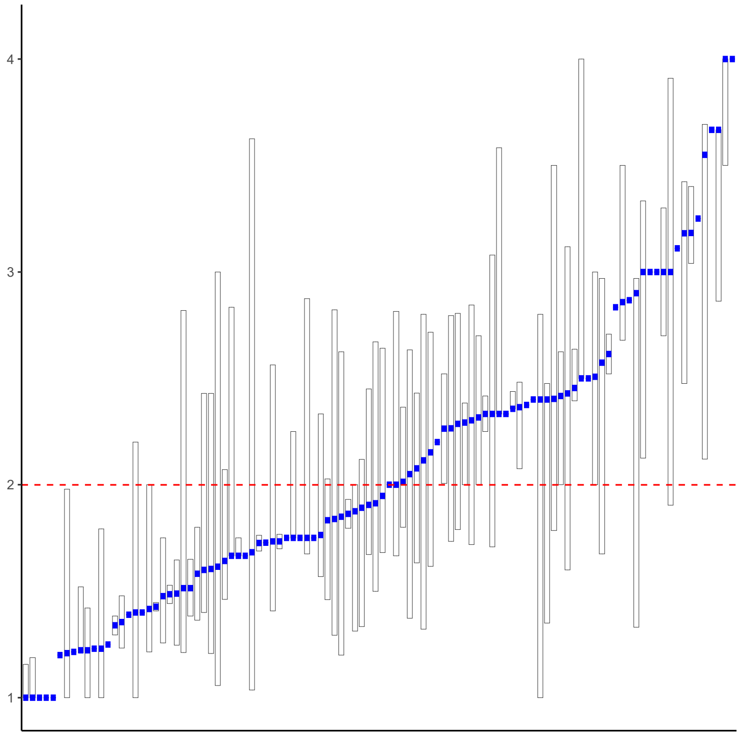

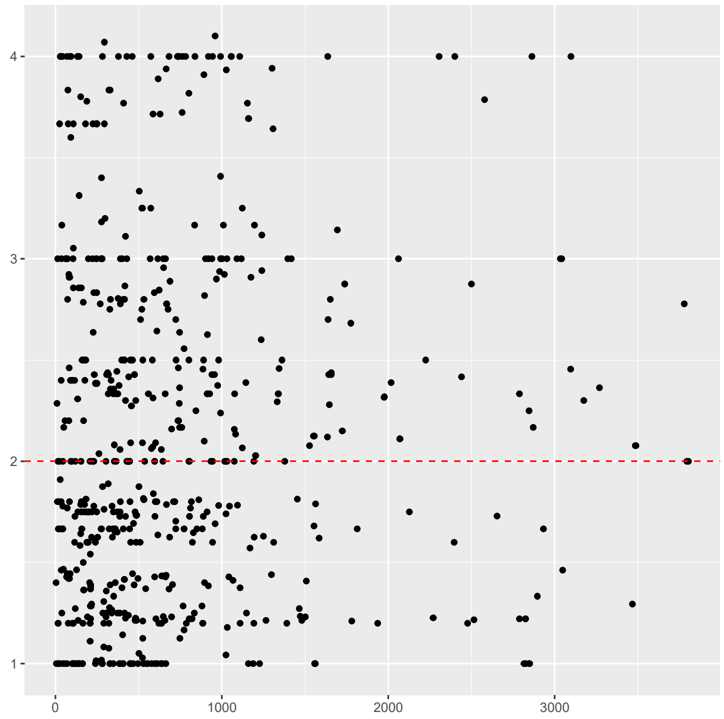

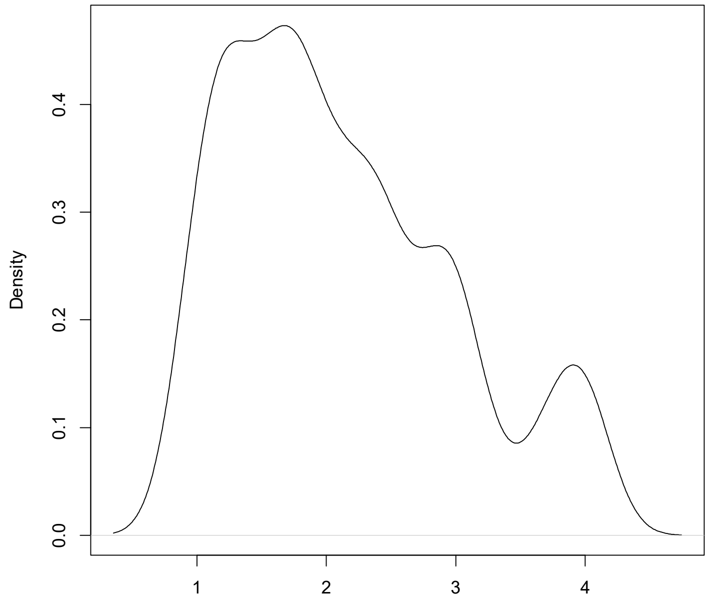

Heptapleurum_pachyphlebium

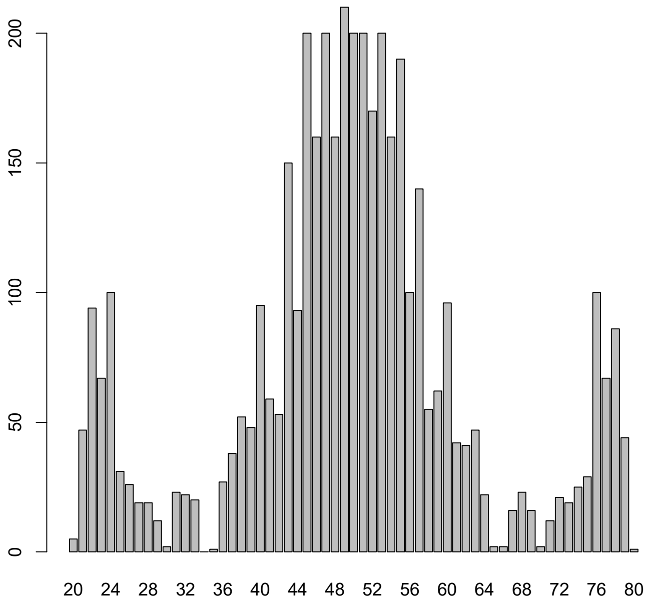

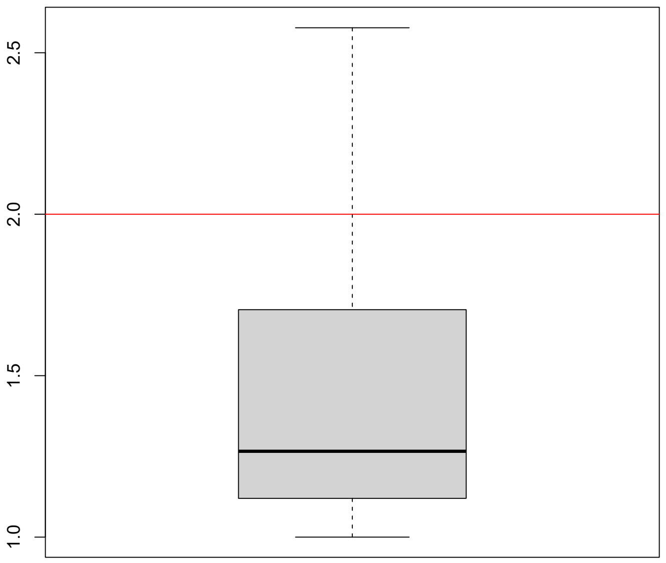

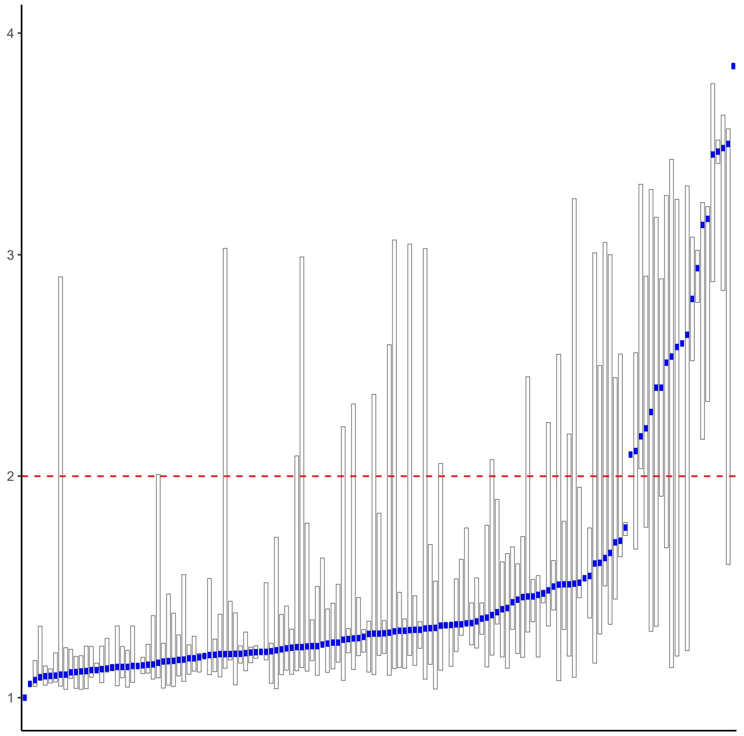

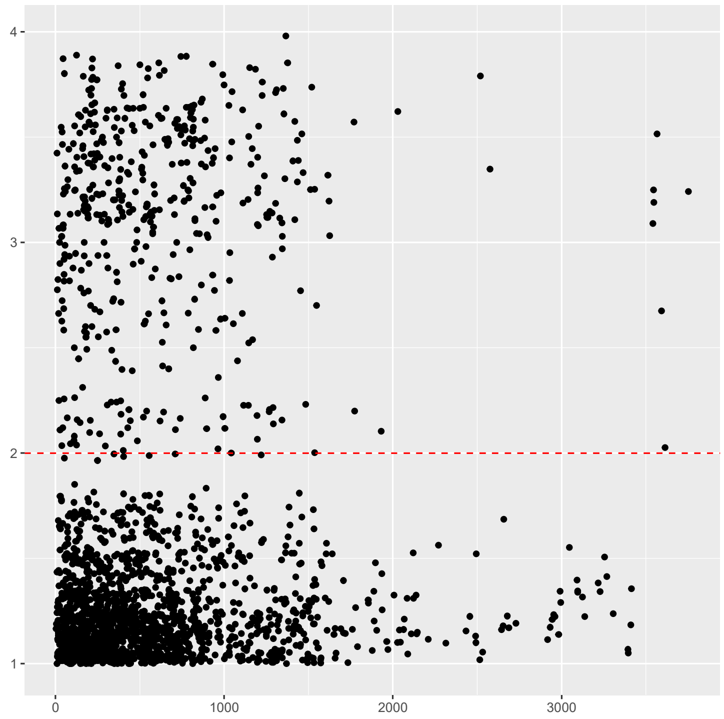

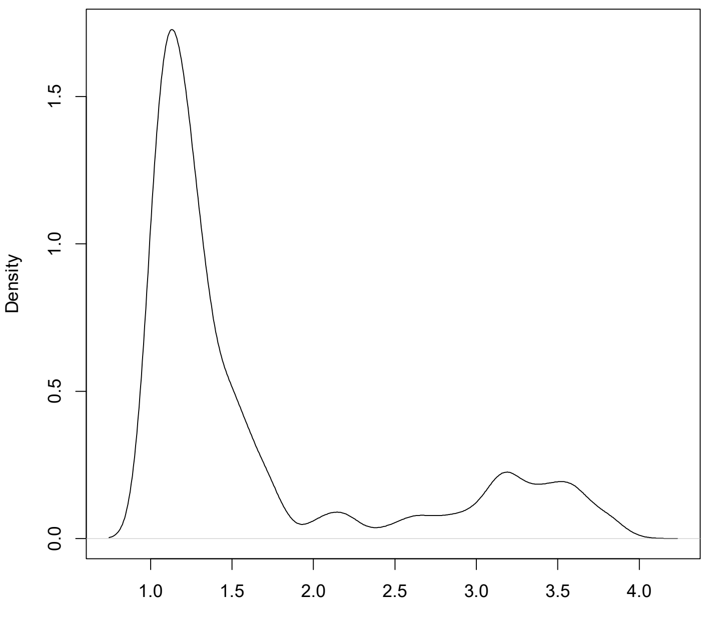

Heptapleurum_petelotii

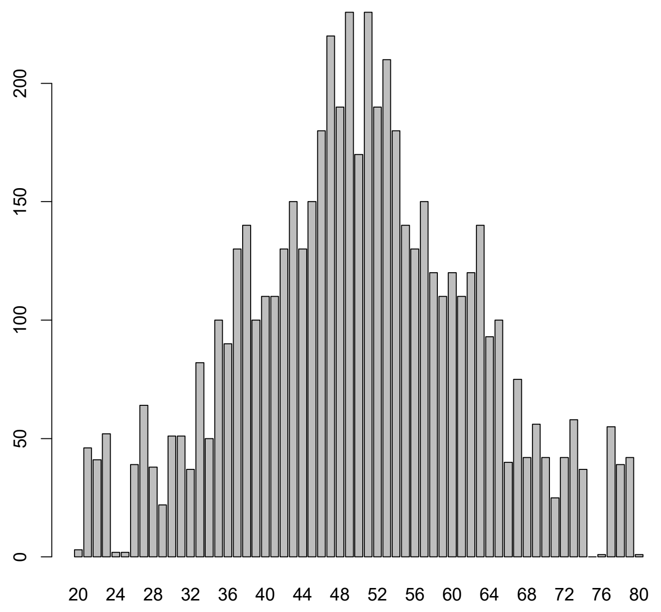

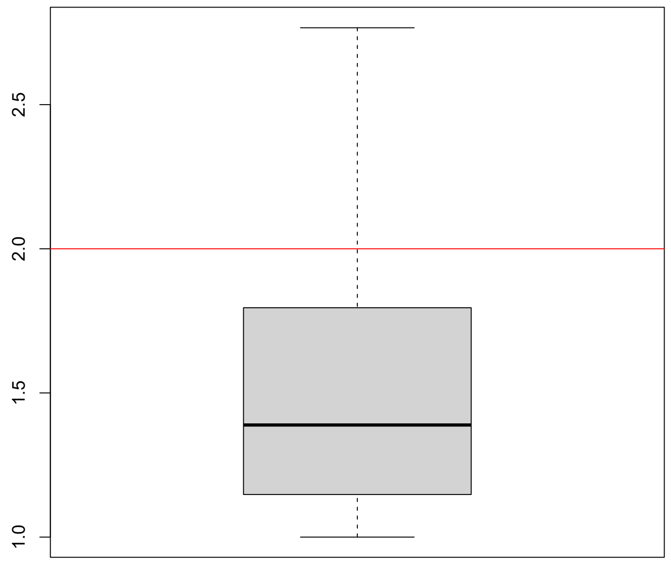

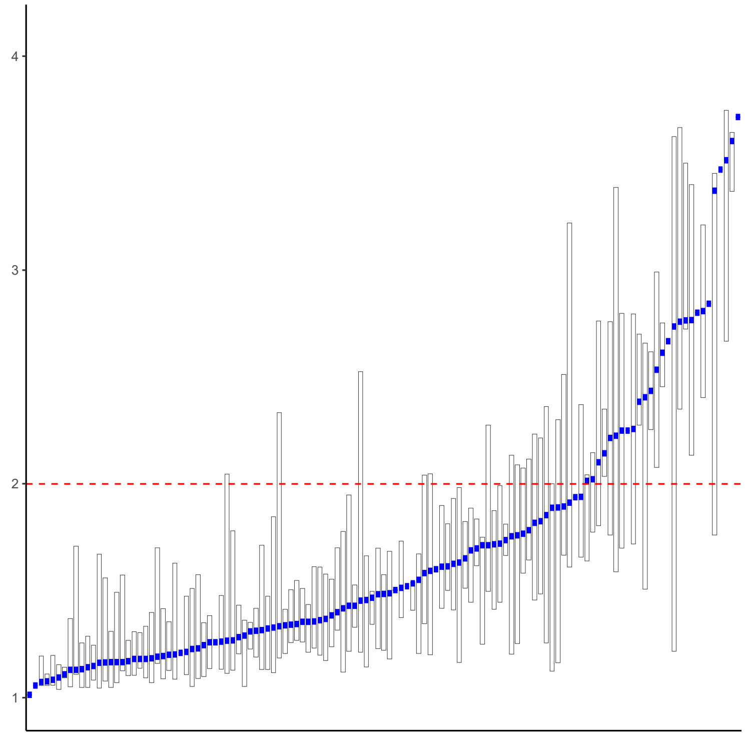

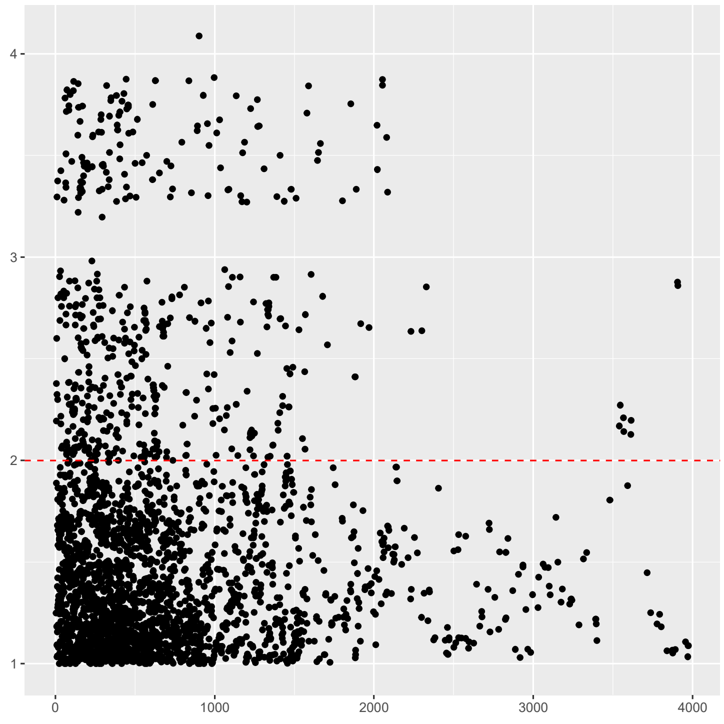

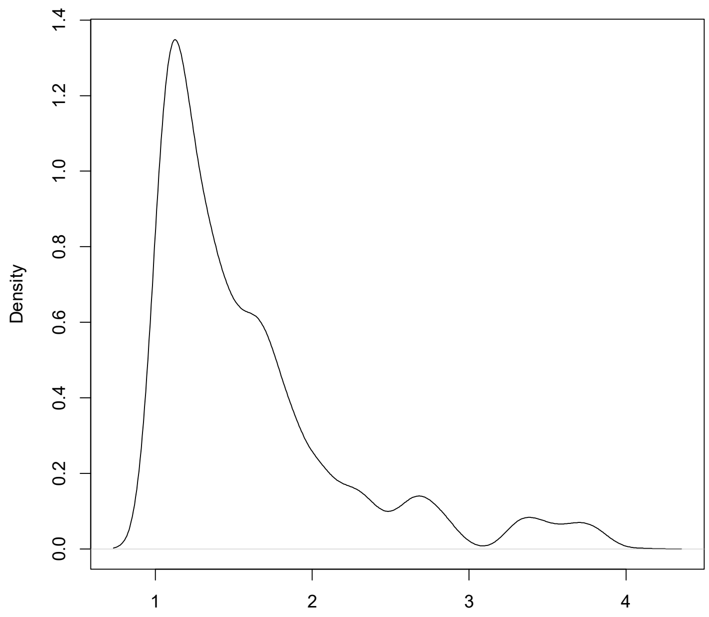

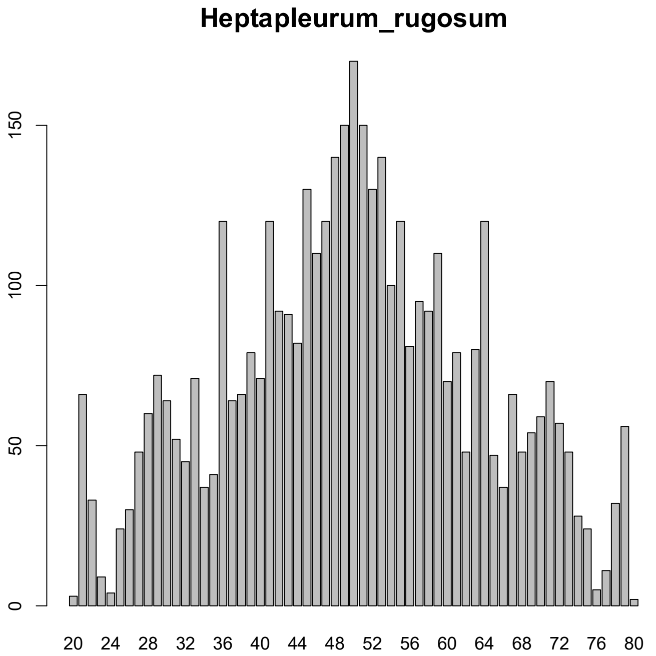
Heptapleurum_rugosum
0
50
100
150
20 24 28 32 36 40 44 48 52 56 60 64 68 72 76 80

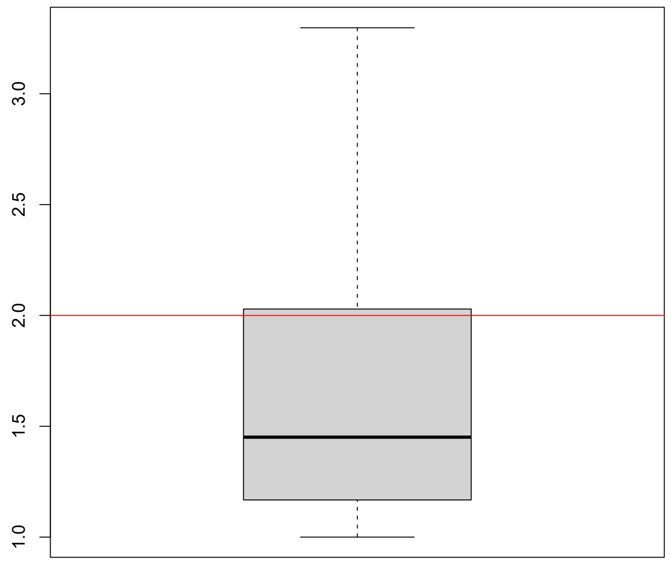
1.0
1.5
2.0
2.5
3.0

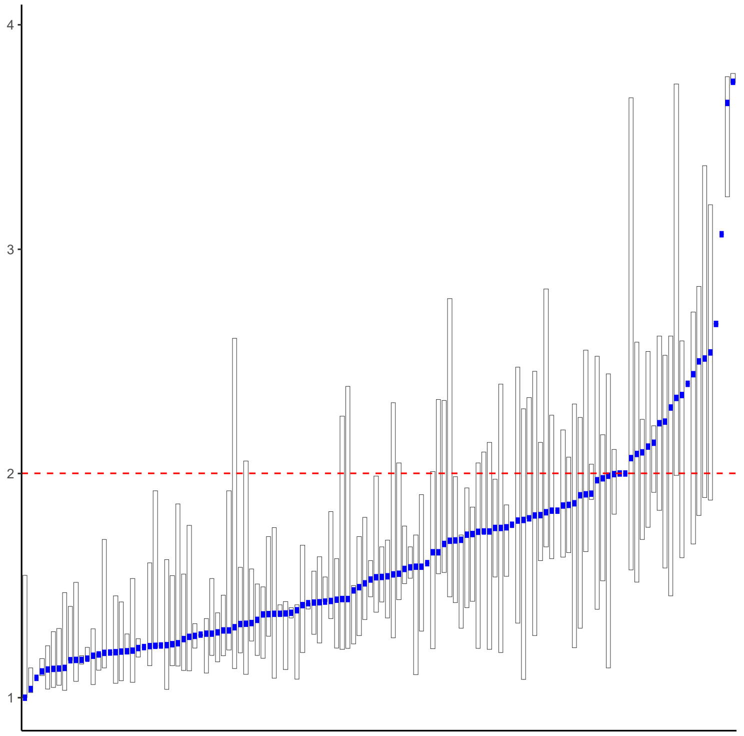
1
2
3
4

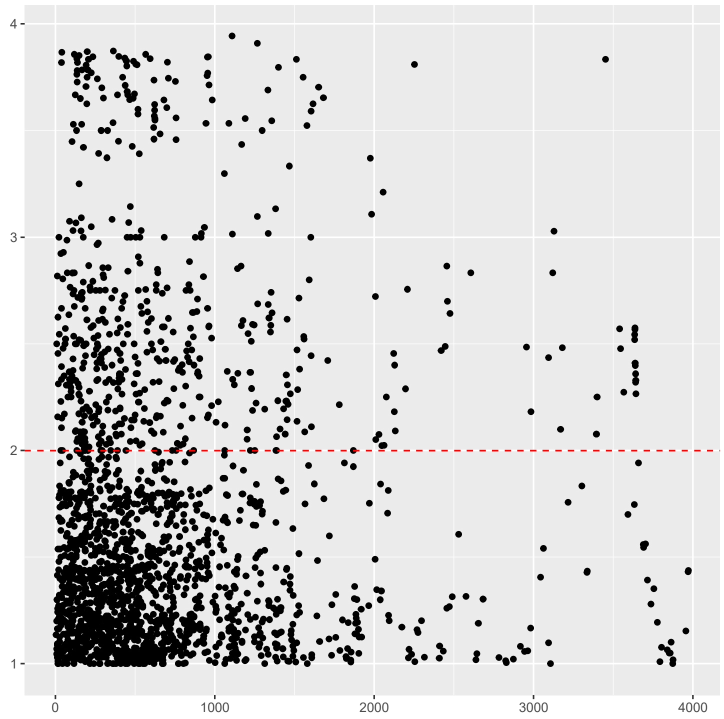
1
2
3
4
0 1000 2000 3000 4000

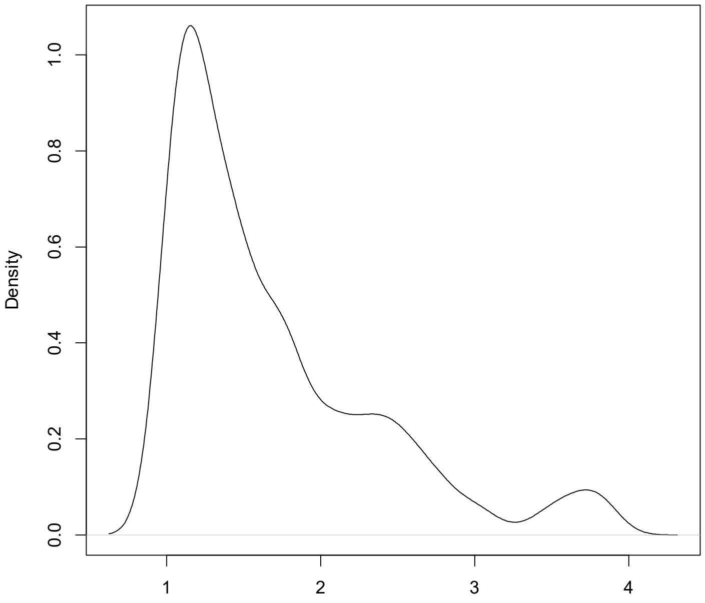
Density
0.0
0.2
0.4
0.6
0.8
1.0
1 2 3 4

Heptapleurum_scandens

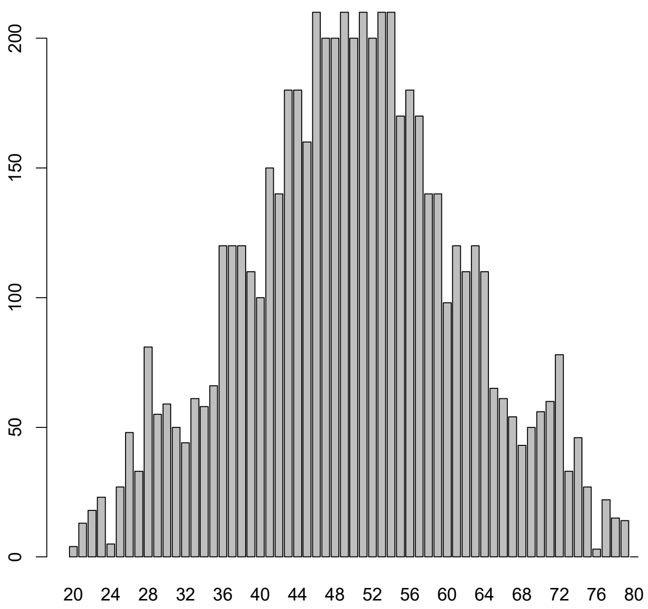

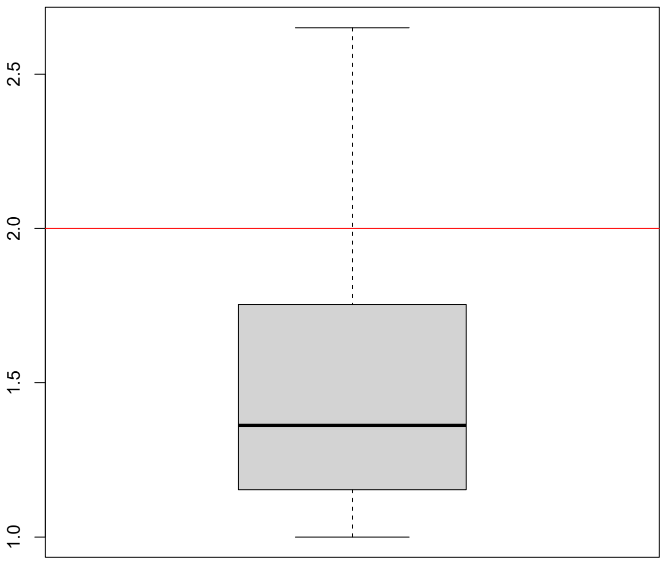

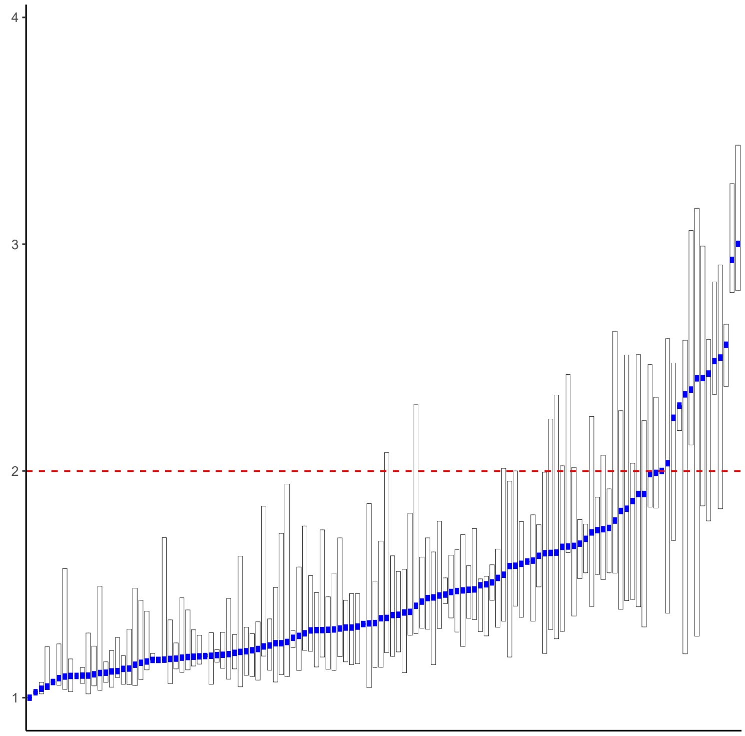

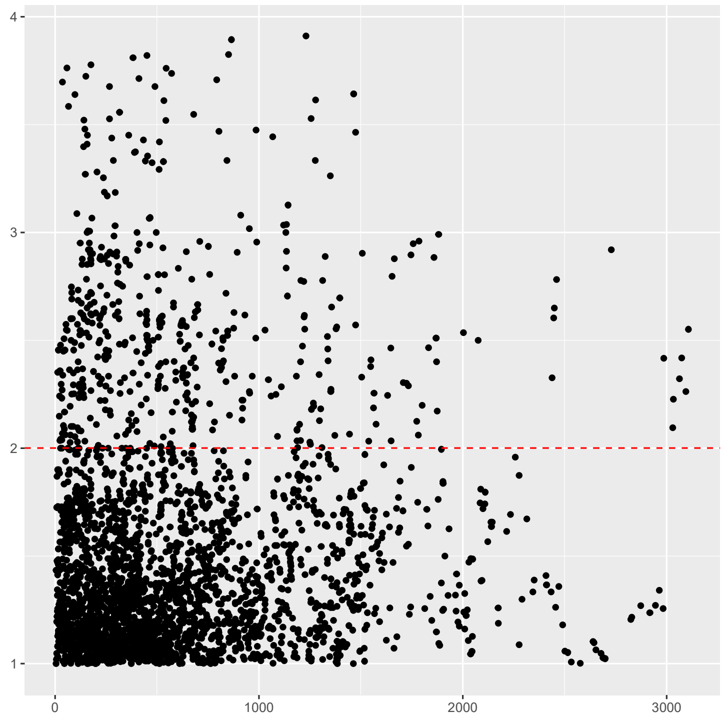

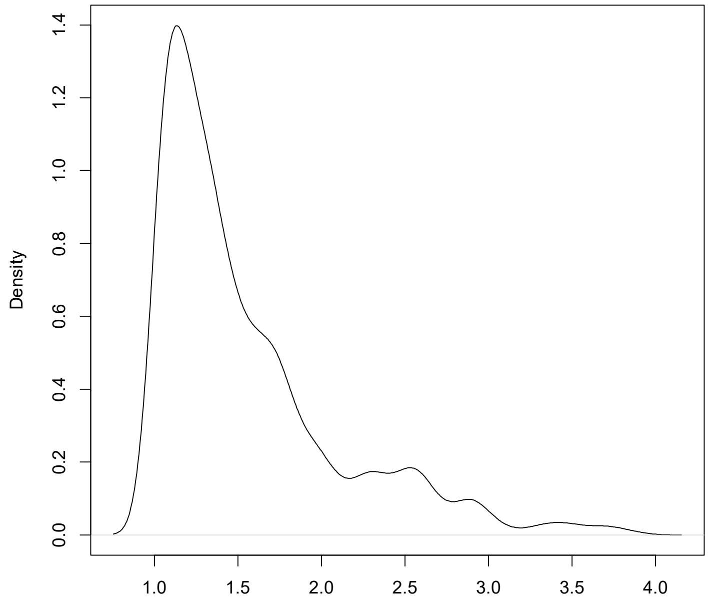

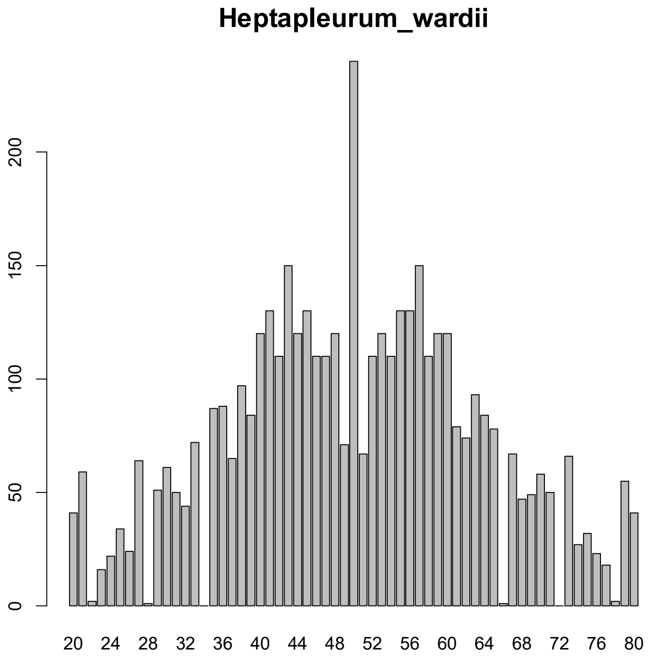

Heptapleurum_wardii
0
50
100
150
200
20
24
28
32
36
40
44
48
52
56
60
64
68
72
76
80

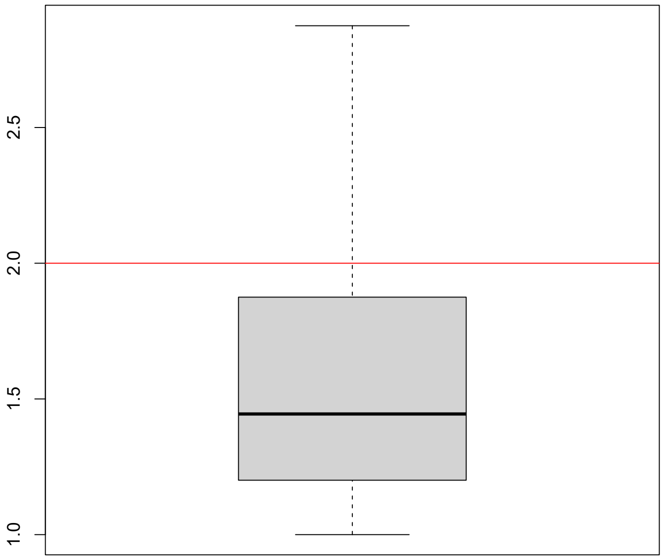

1.0
1.5
2.0
2.5

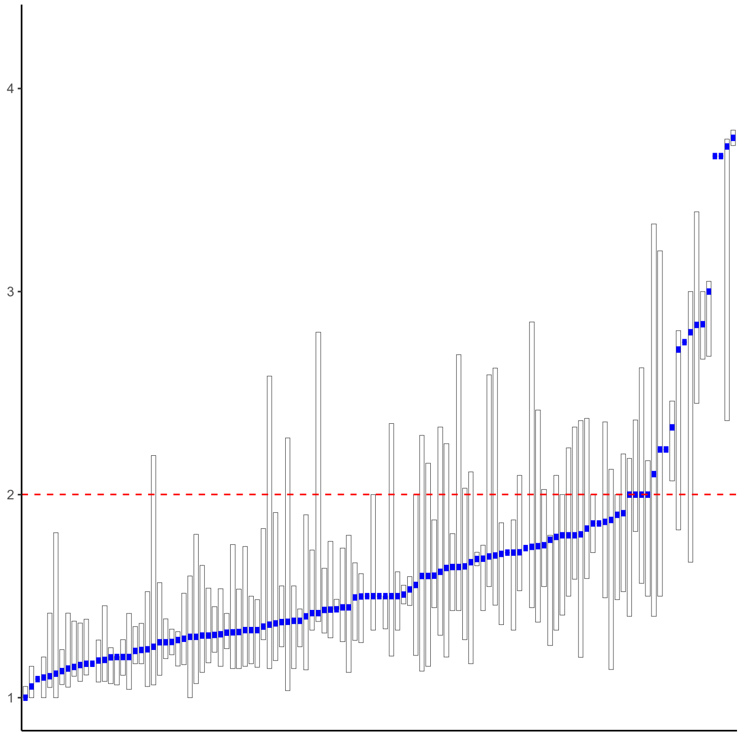

1
2
3
4

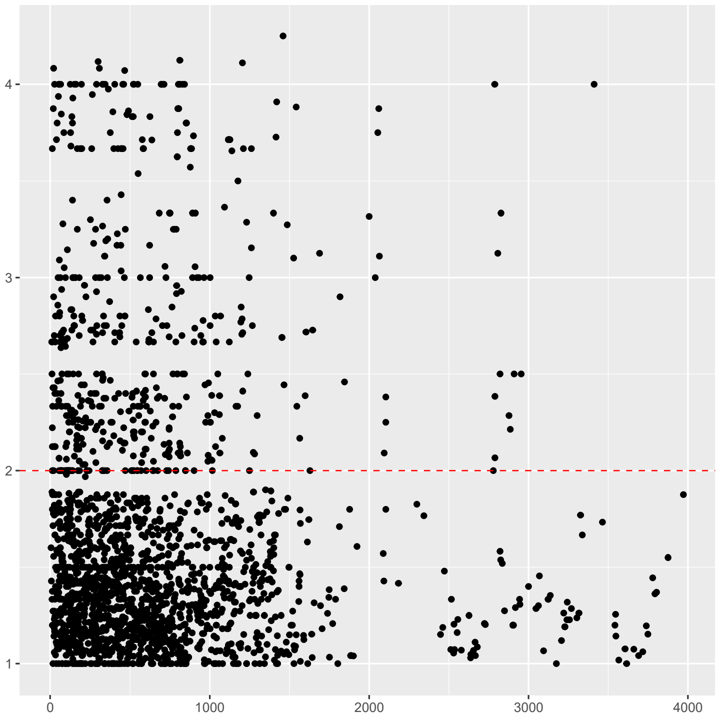

1
2
3
4
0
1000
2000
3000
4000

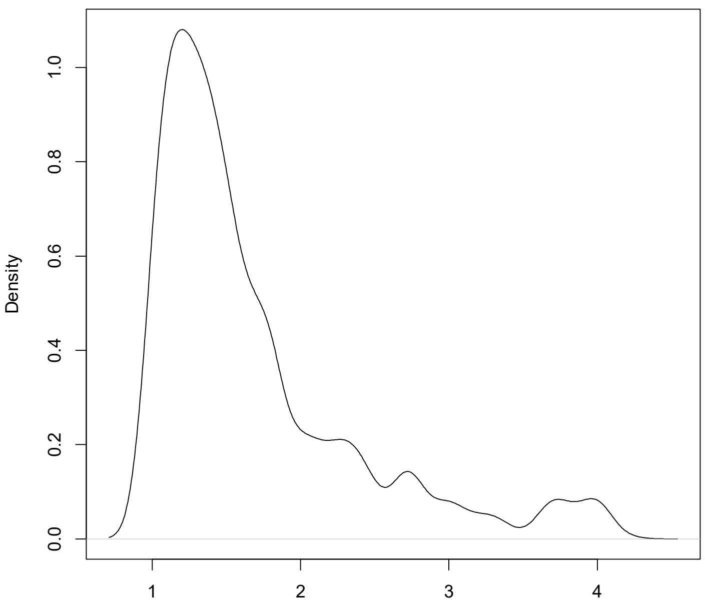

Density
0.0
0.2
0.4
0.6
0.8
1.0
1
2
3
4

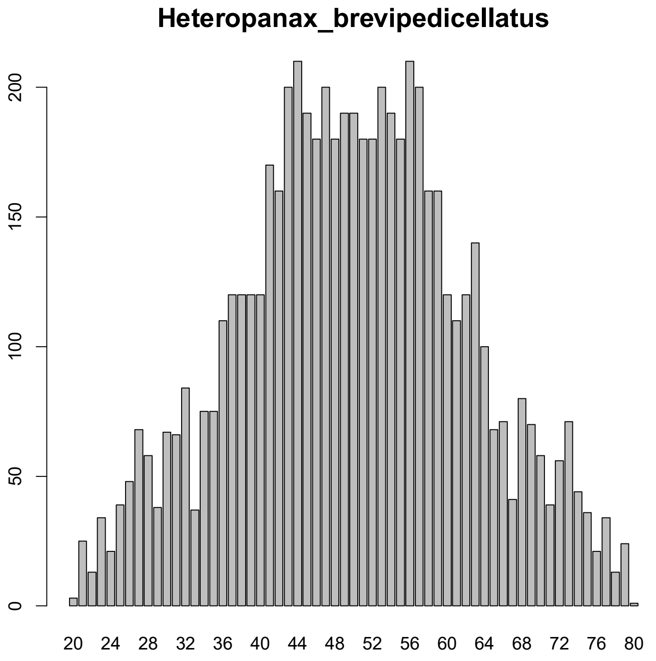
Heteropanax_brevipedicellatus
0
50
100
150
200
20 24 28 32 36 40 44 48 52 56 60 64 68 72 76 80

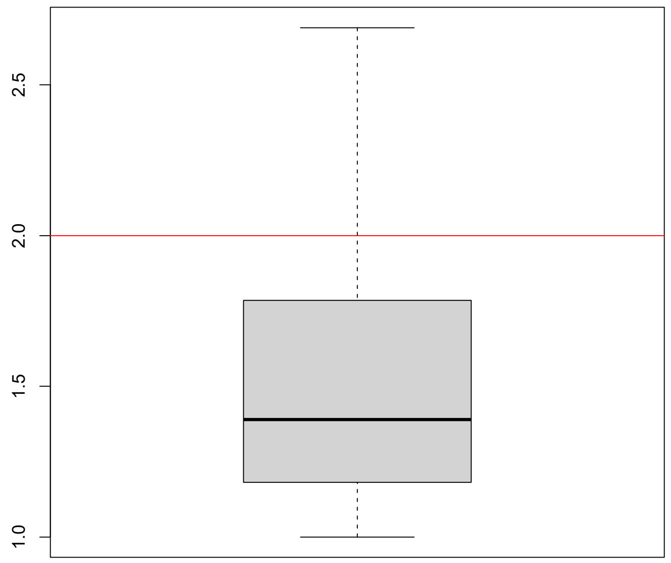
1.0
1.5
2.0
2.5

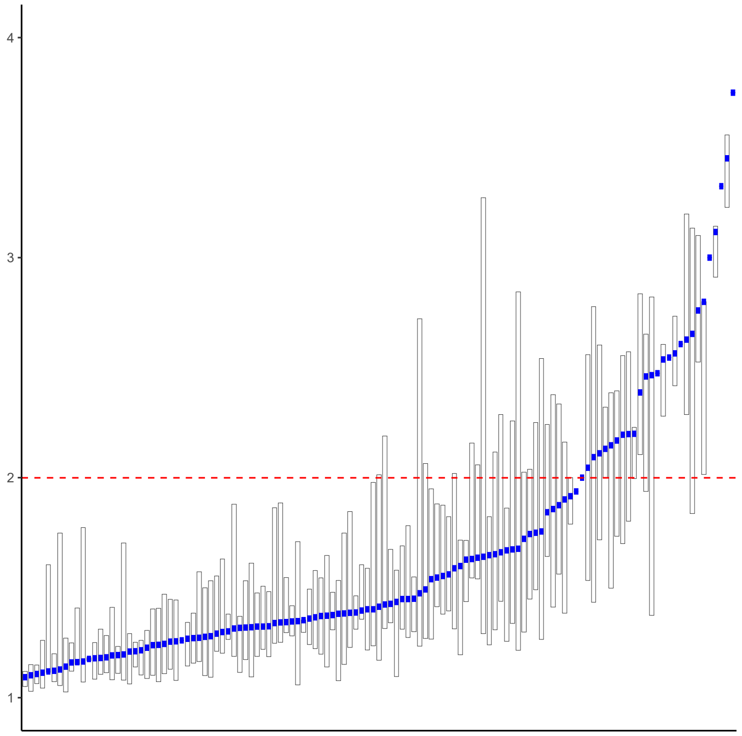
1
2
3
4

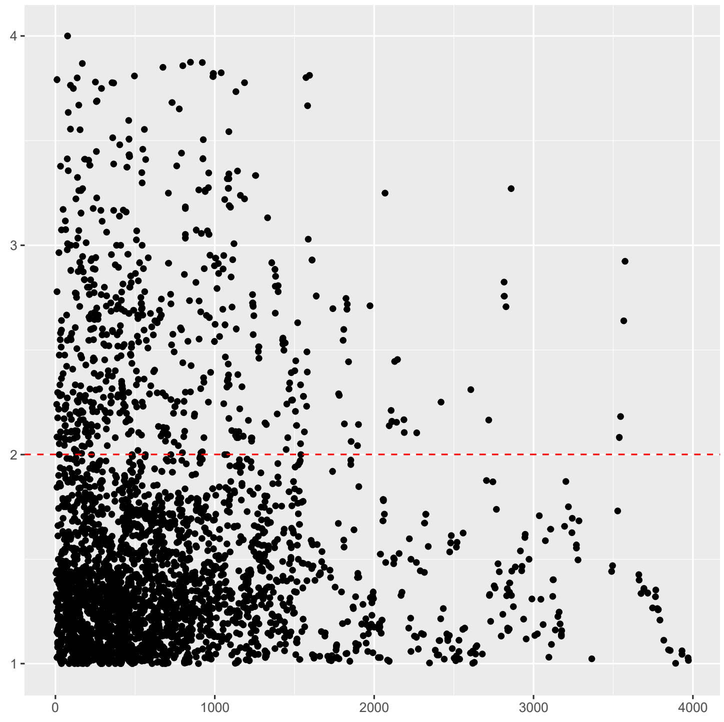
1
2
3
4
0 1000 2000 3000 4000

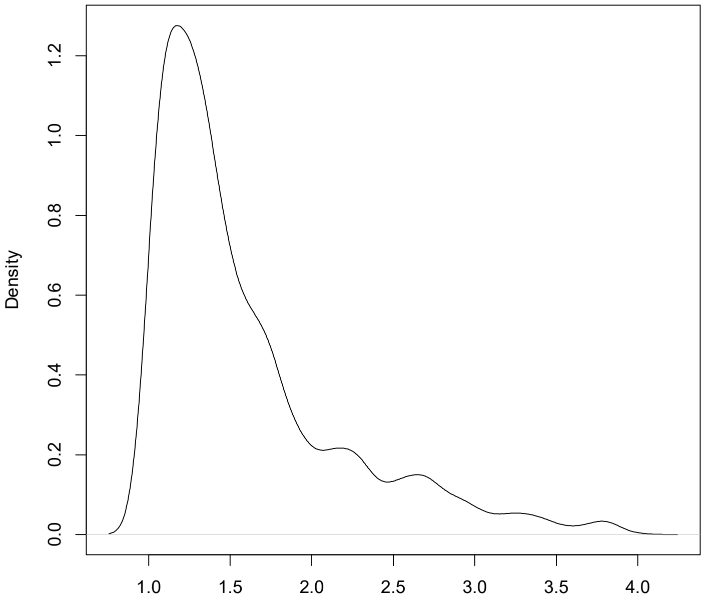
Density
0.0
0.2
0.4
0.6
0.8
1.0
1.2
1.0 1.5 2.0 2.5 3.0 3.5 4.0

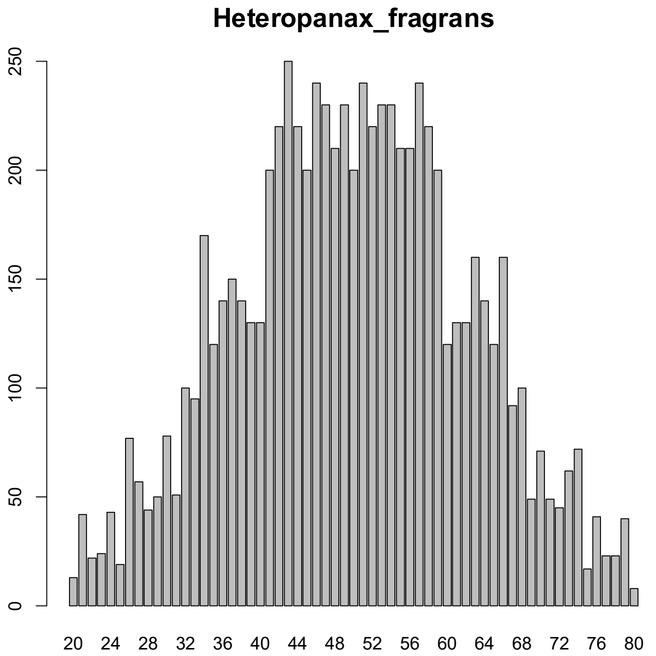
Heteropanax_fragrans
0
50
100
150
200
250
20 24 28 32 36 40 44 48 52 56 60 64 68 72 76 80

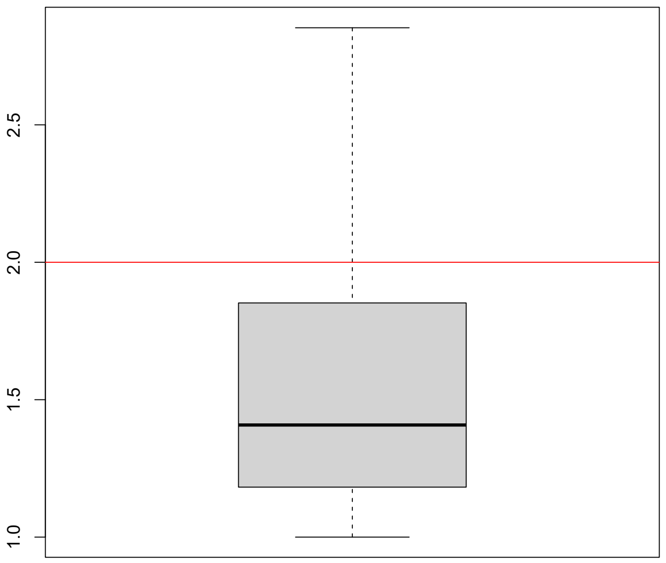
1.0
1.5
2.0
2.5

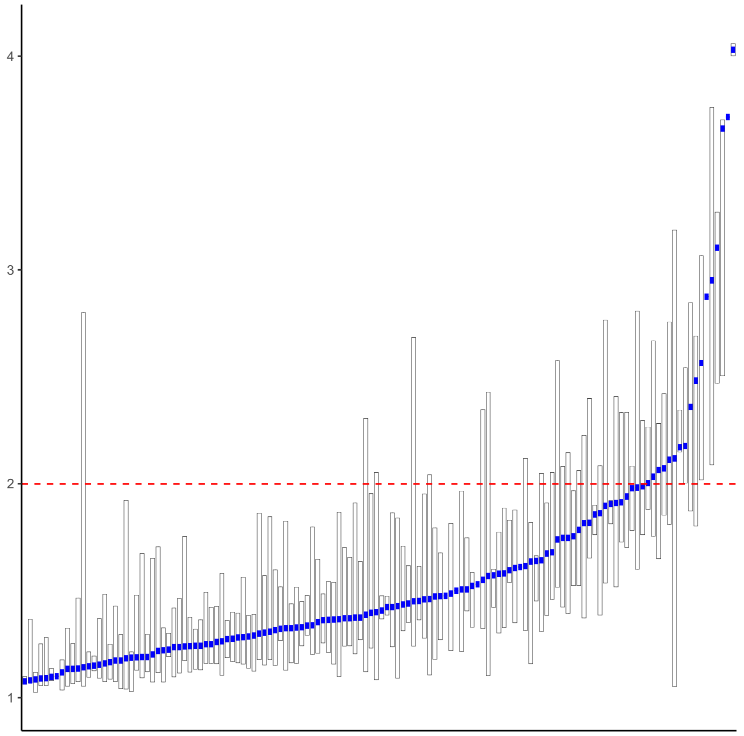
1
2
3
4

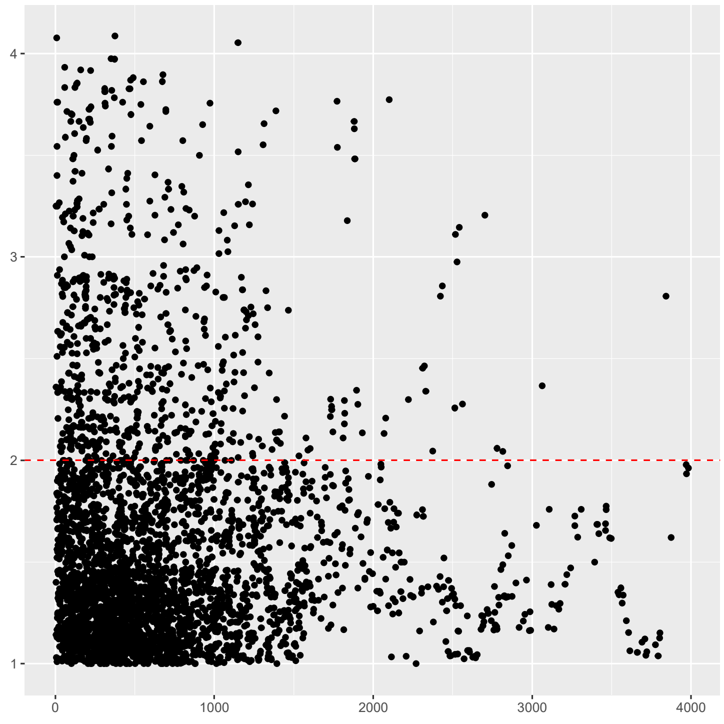
1
2
3
4
0 1000 2000 3000 4000

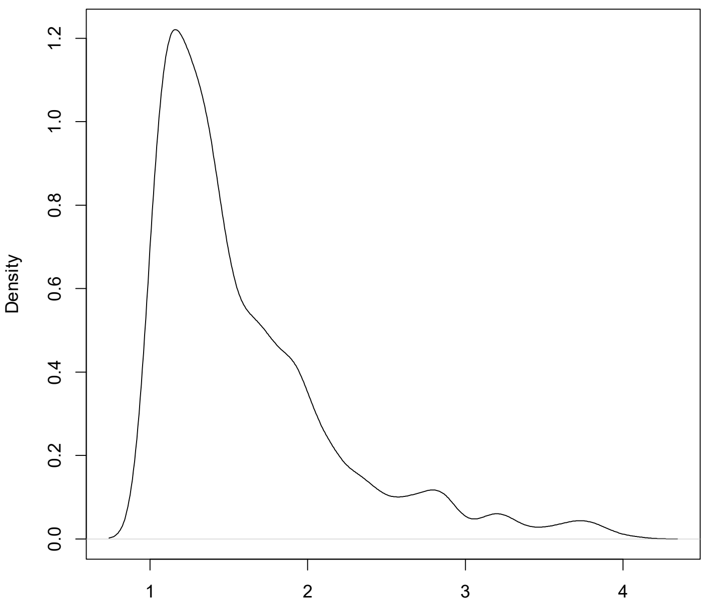
Density
0.0
0.2
0.4
0.6
0.8
1.0
1.2
1 2 3 4

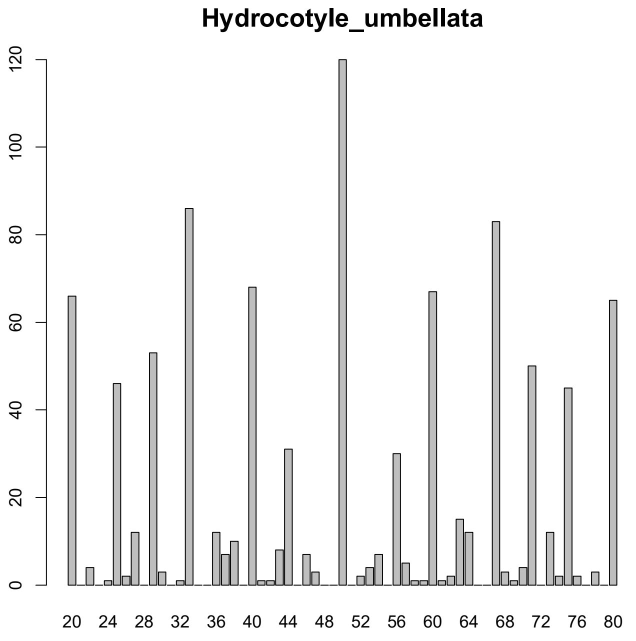
Hydrocotyle_umbellata
0
20
40
60
80
100
120
20 24 28 32 36 40 44 48 52 56 60 64 68 72 76 80

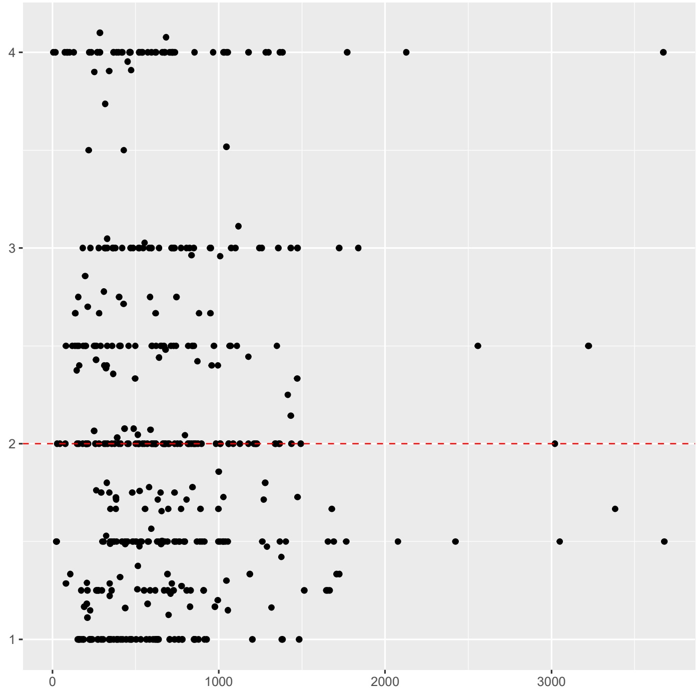
1
2
3
4
0
1000
2000
3000

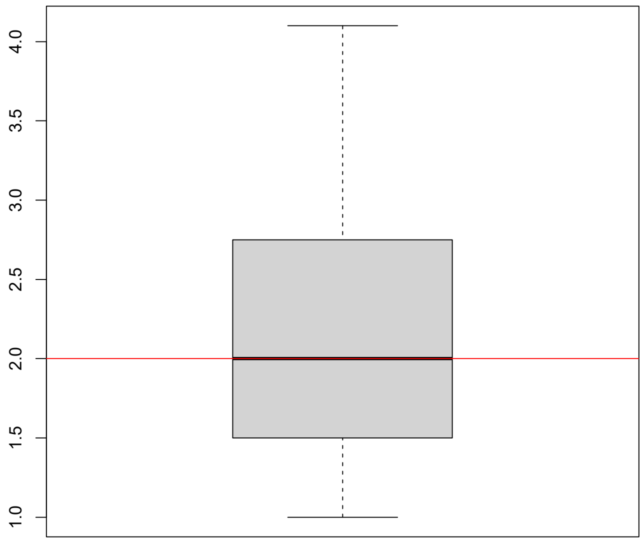
1.0
1.5
2.0
2.5
3.0
3.5
4.0

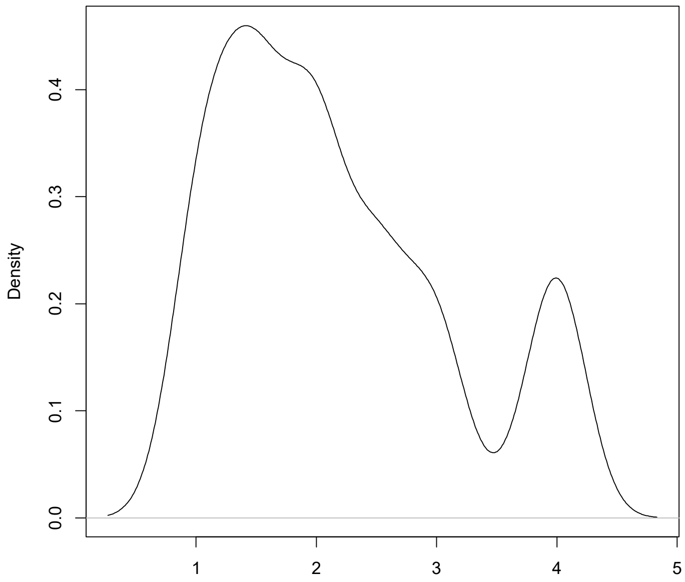
Density
0.0
0.1
0.2
0.3
0.4
1 2 3 4 5

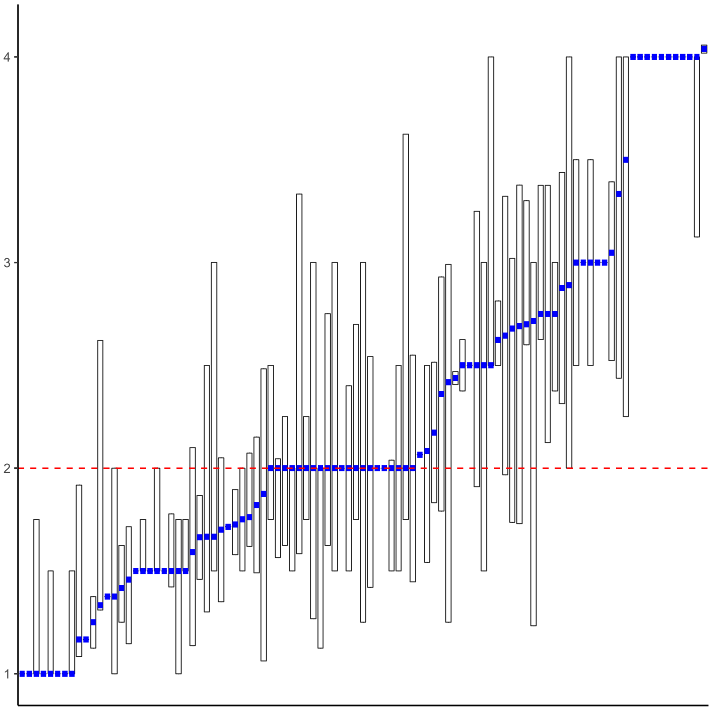
1
2
3
4

Hydrocotyle_cf_nepalensis

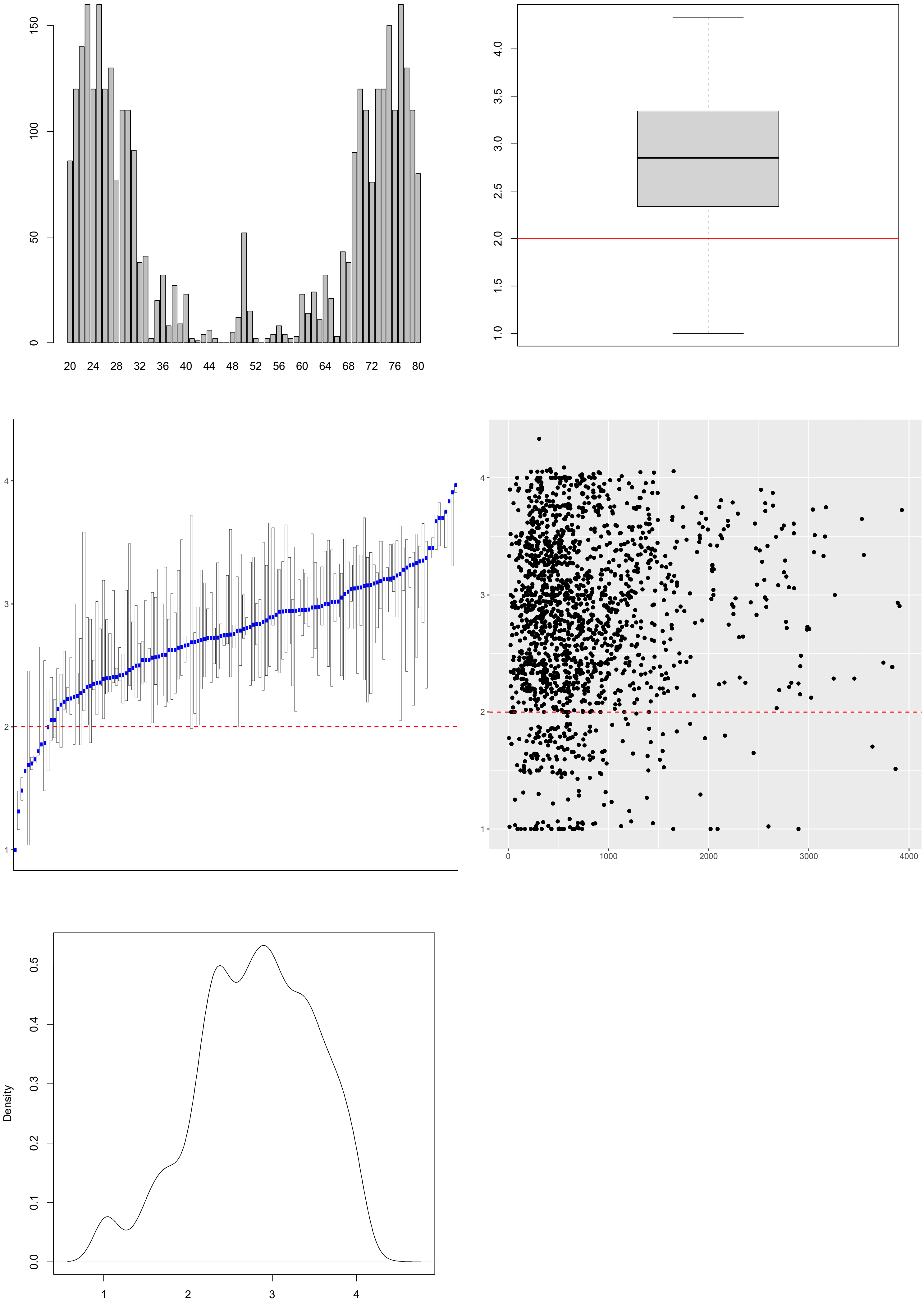

Kalopanax_septemlobus

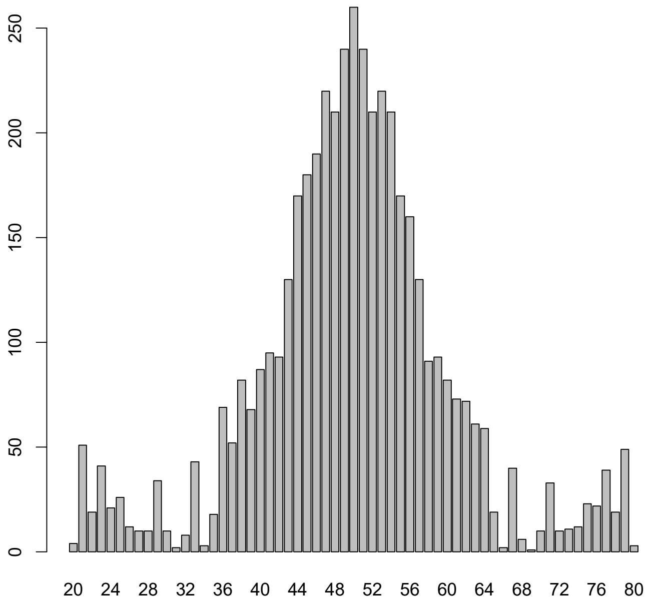

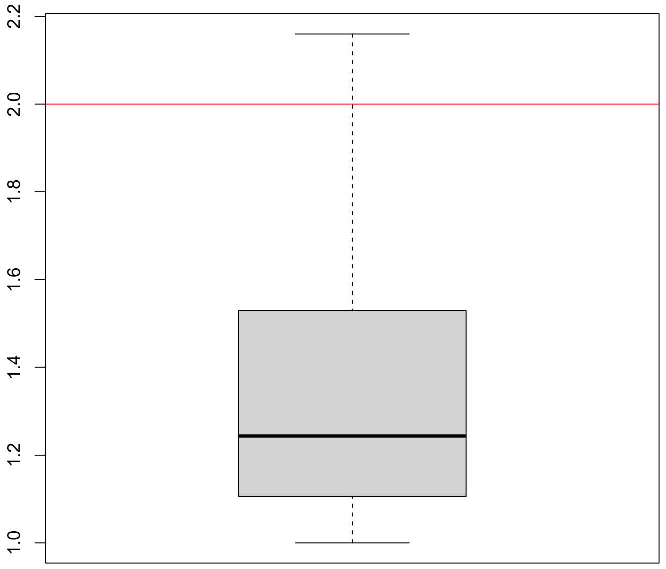

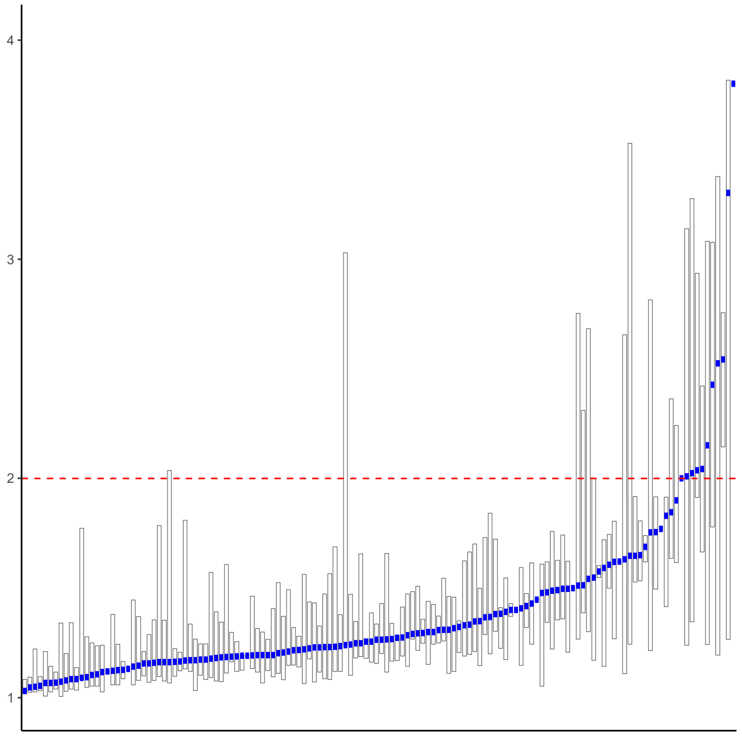

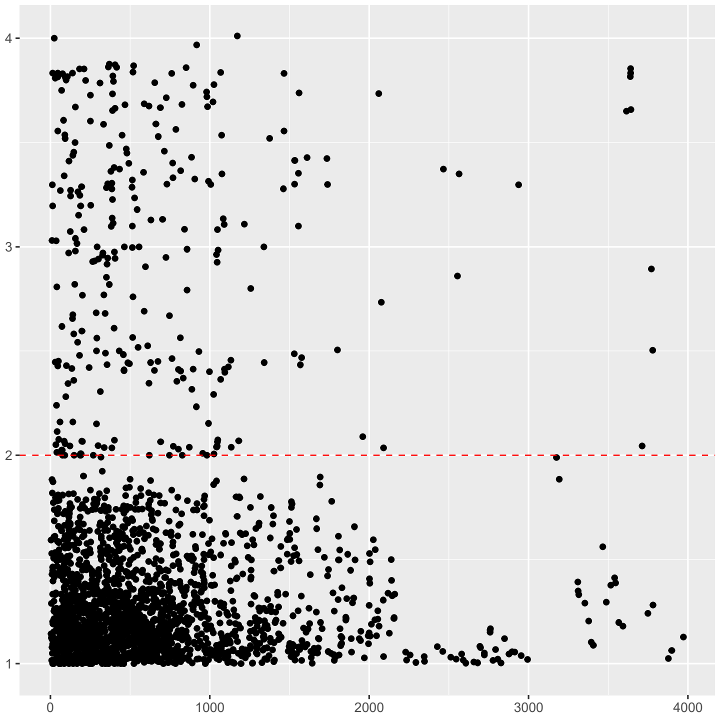

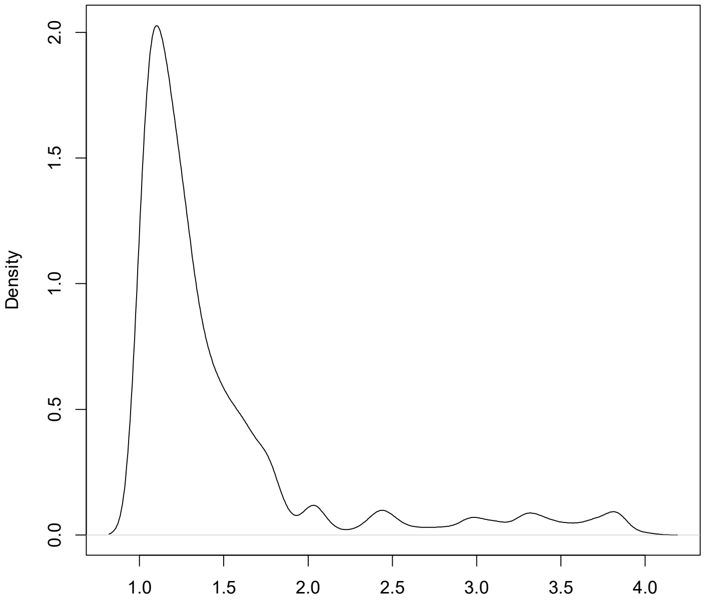

Mackinlaya_schlechteri

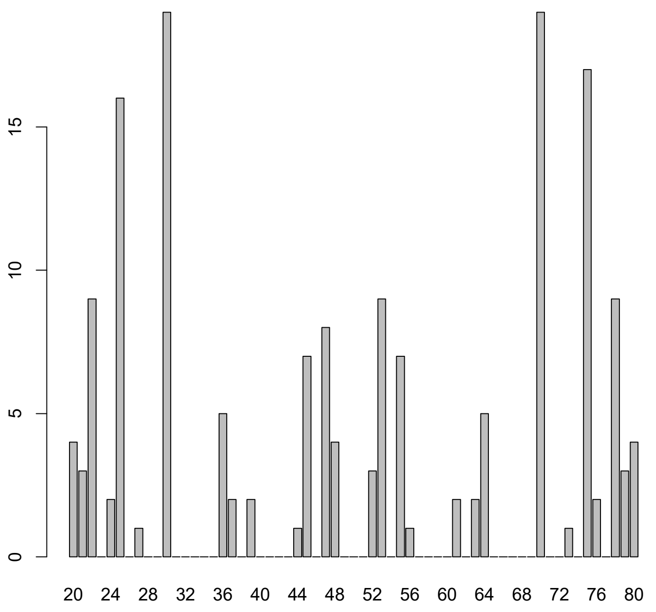

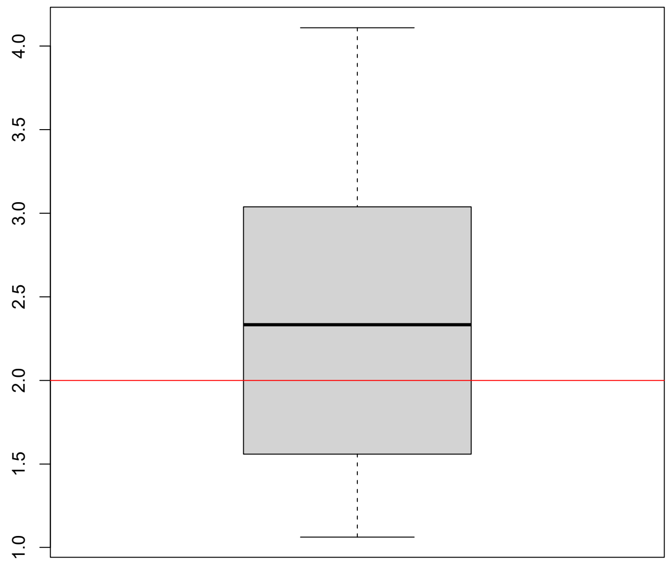

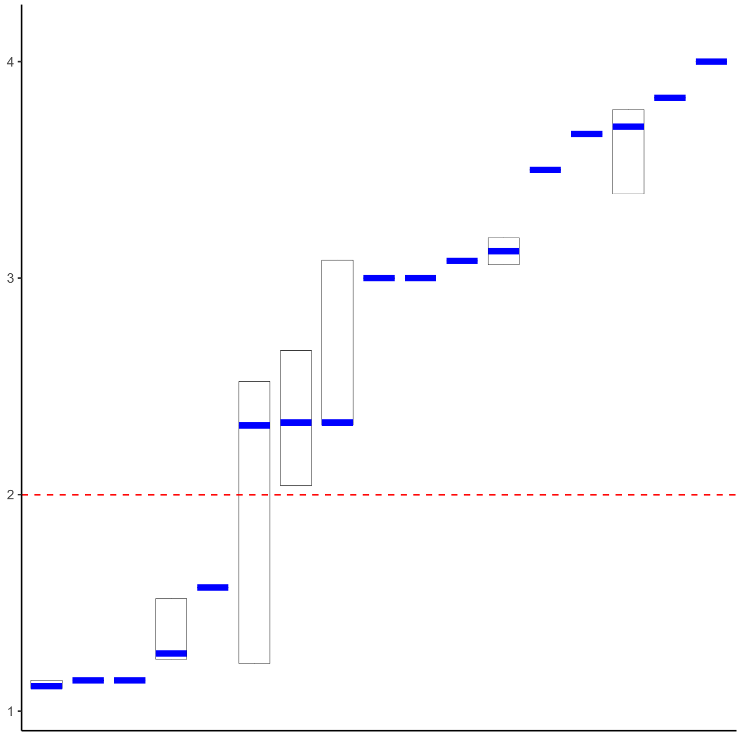

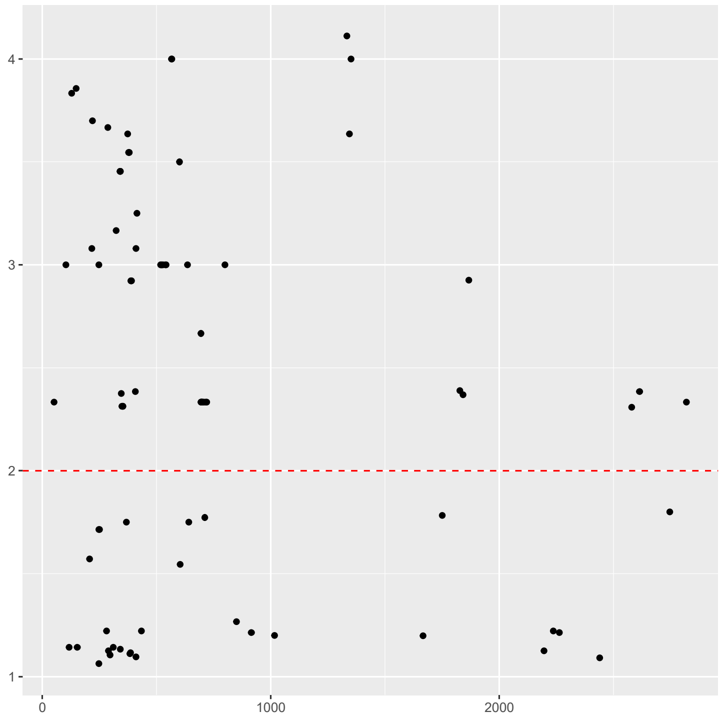

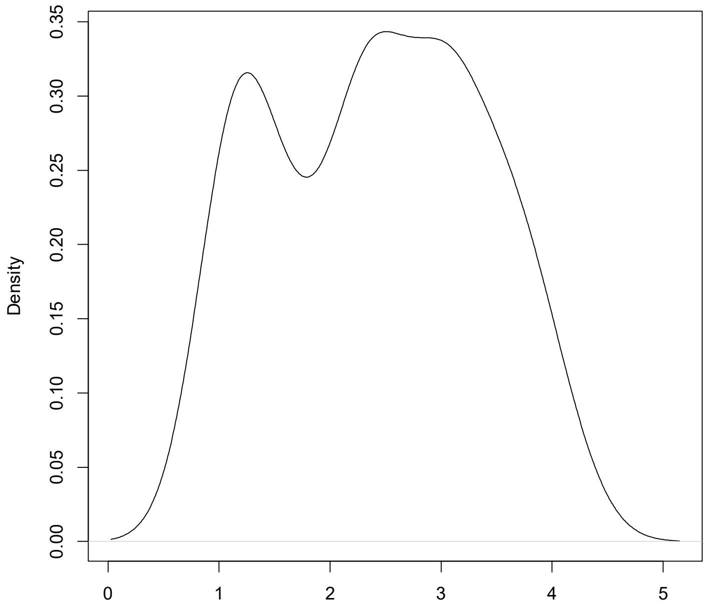

Macropanax_chienii

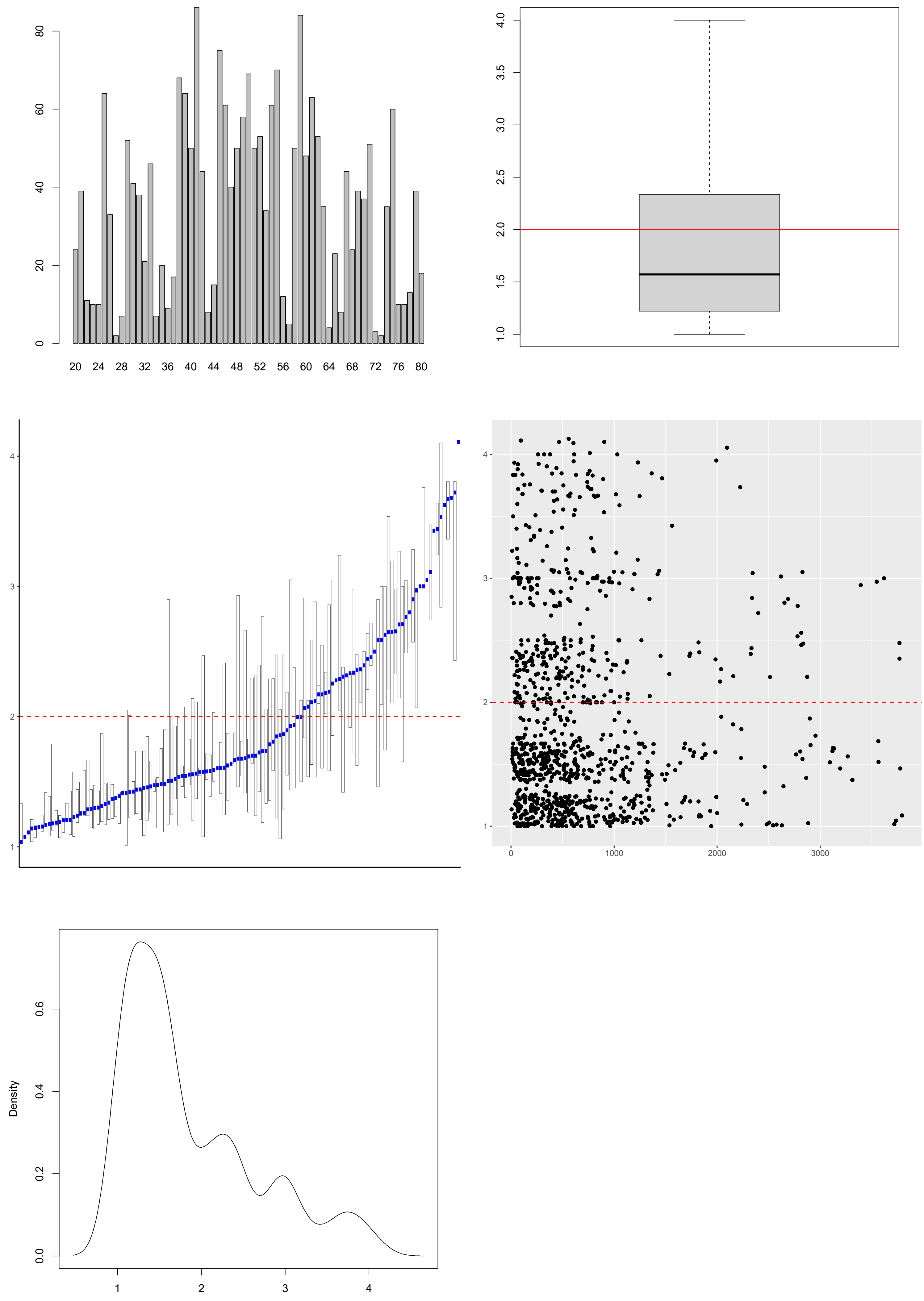

Macropanax_dispermus

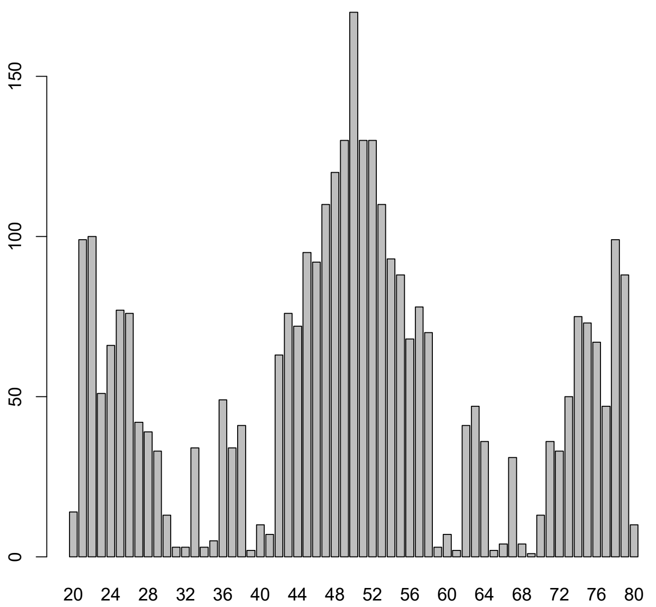

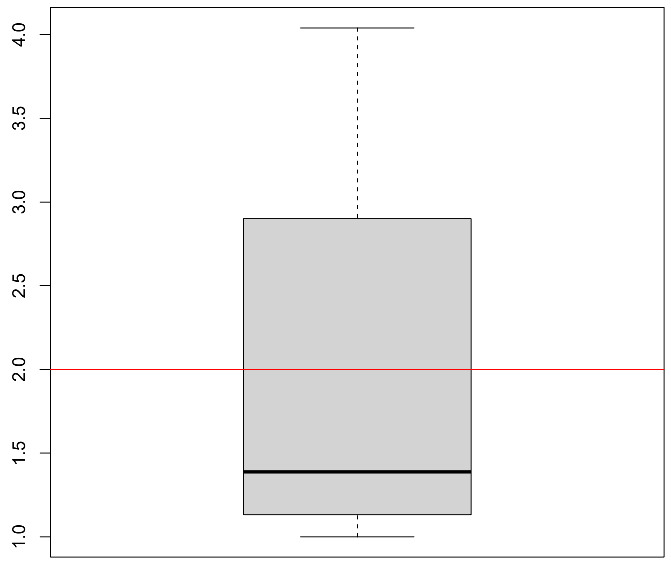

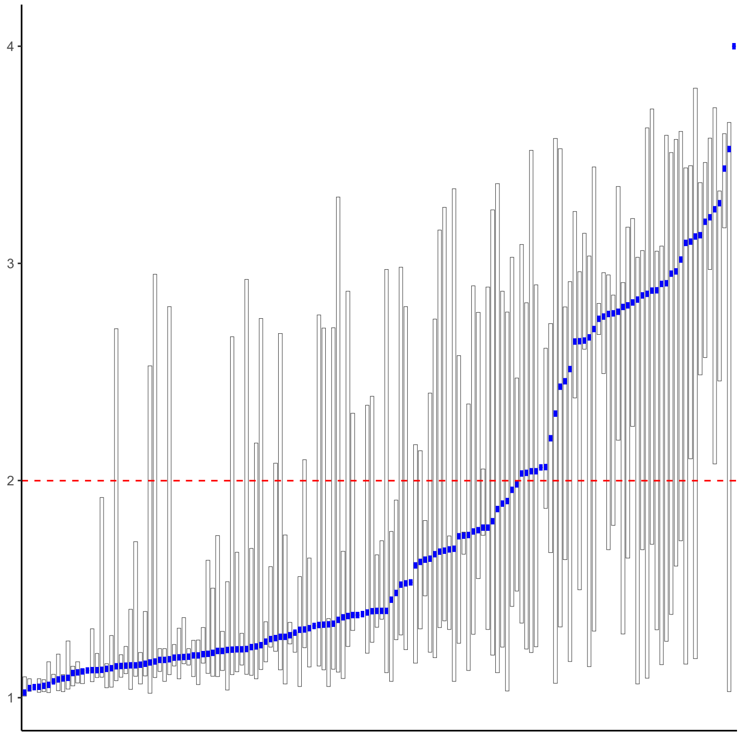

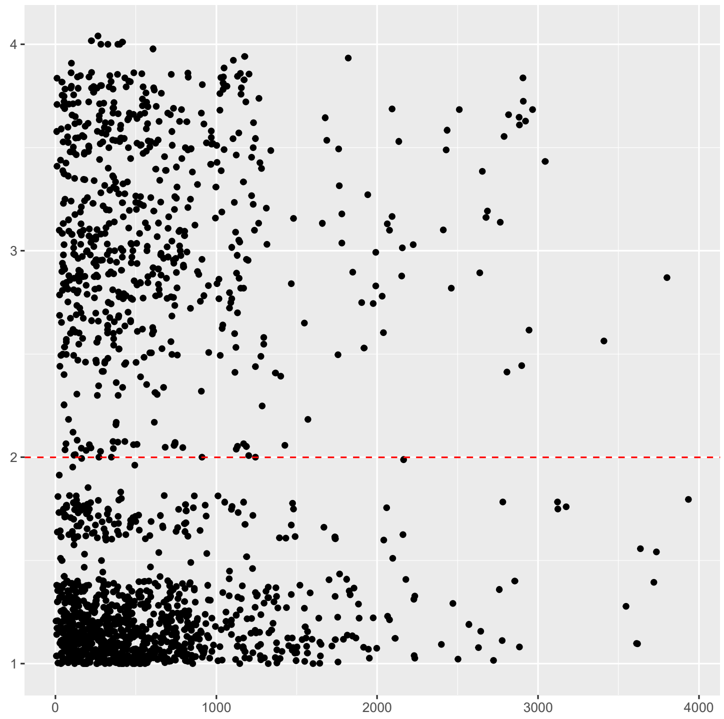

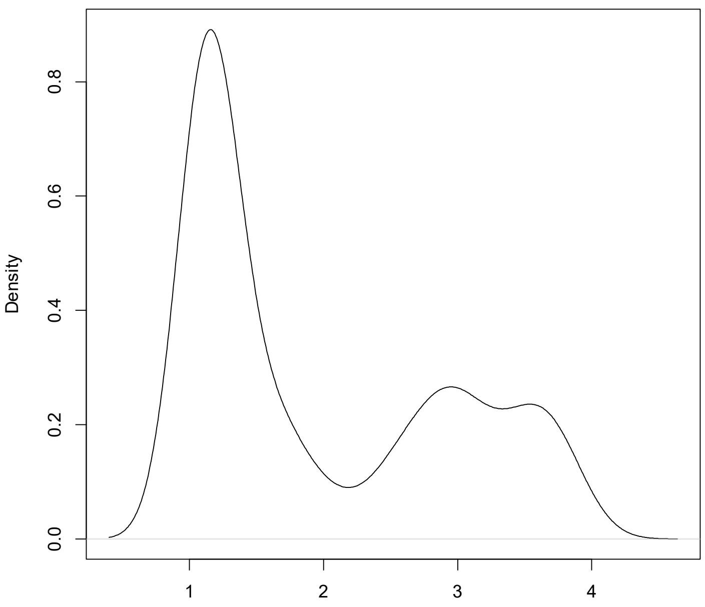

Macropanax_maingayi

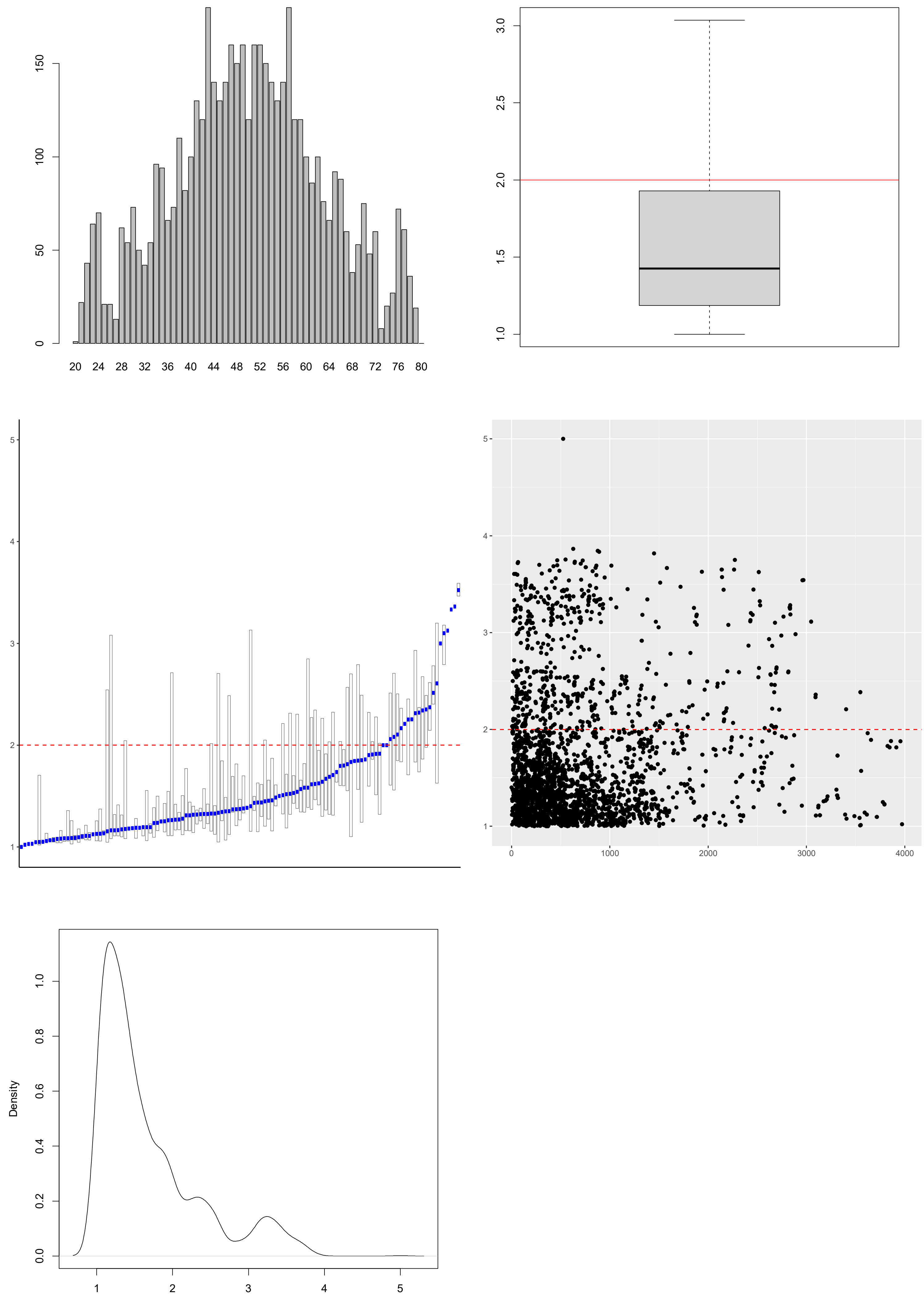

Macropanax_rosthornii

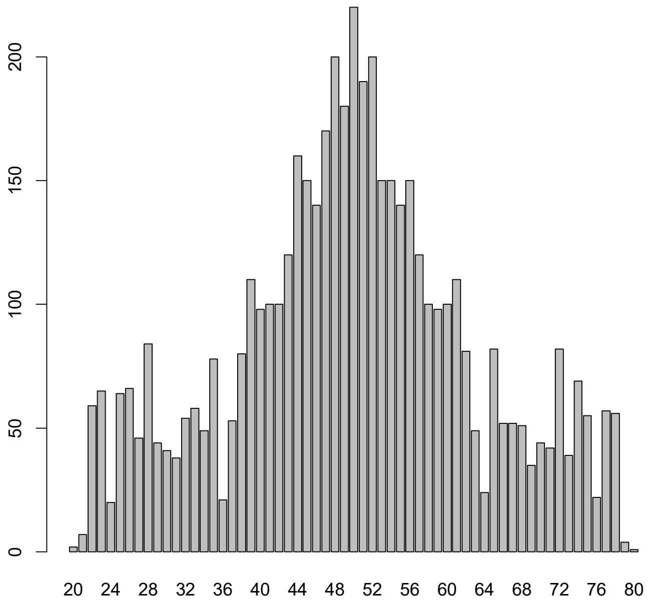

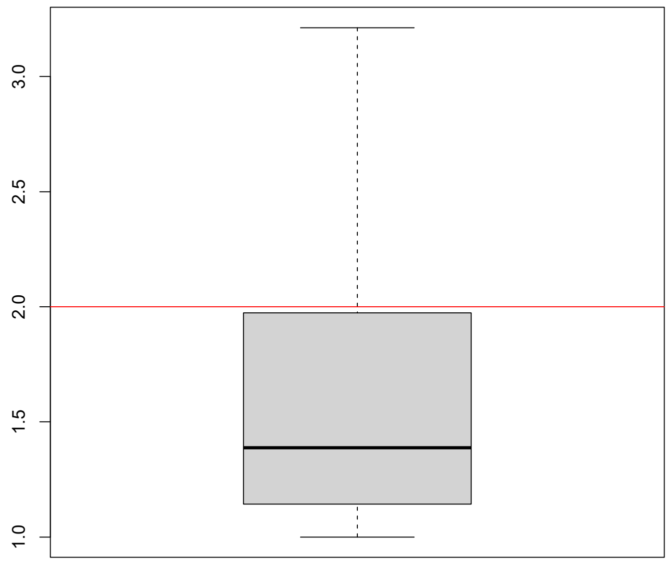

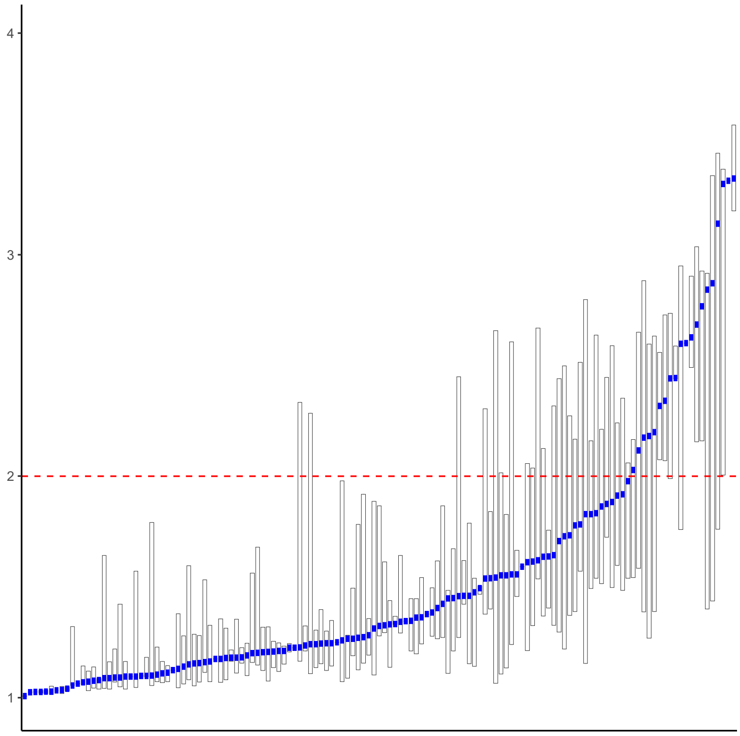

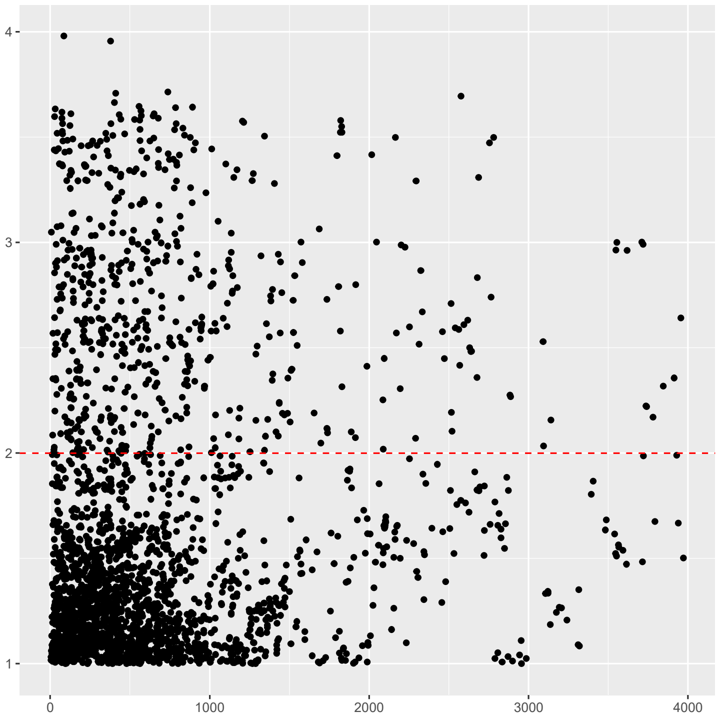

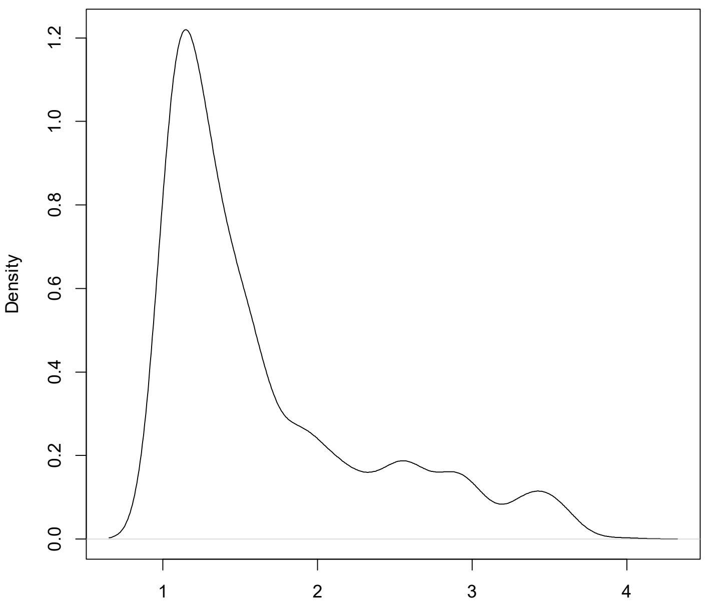

Macropanax_serratifolius

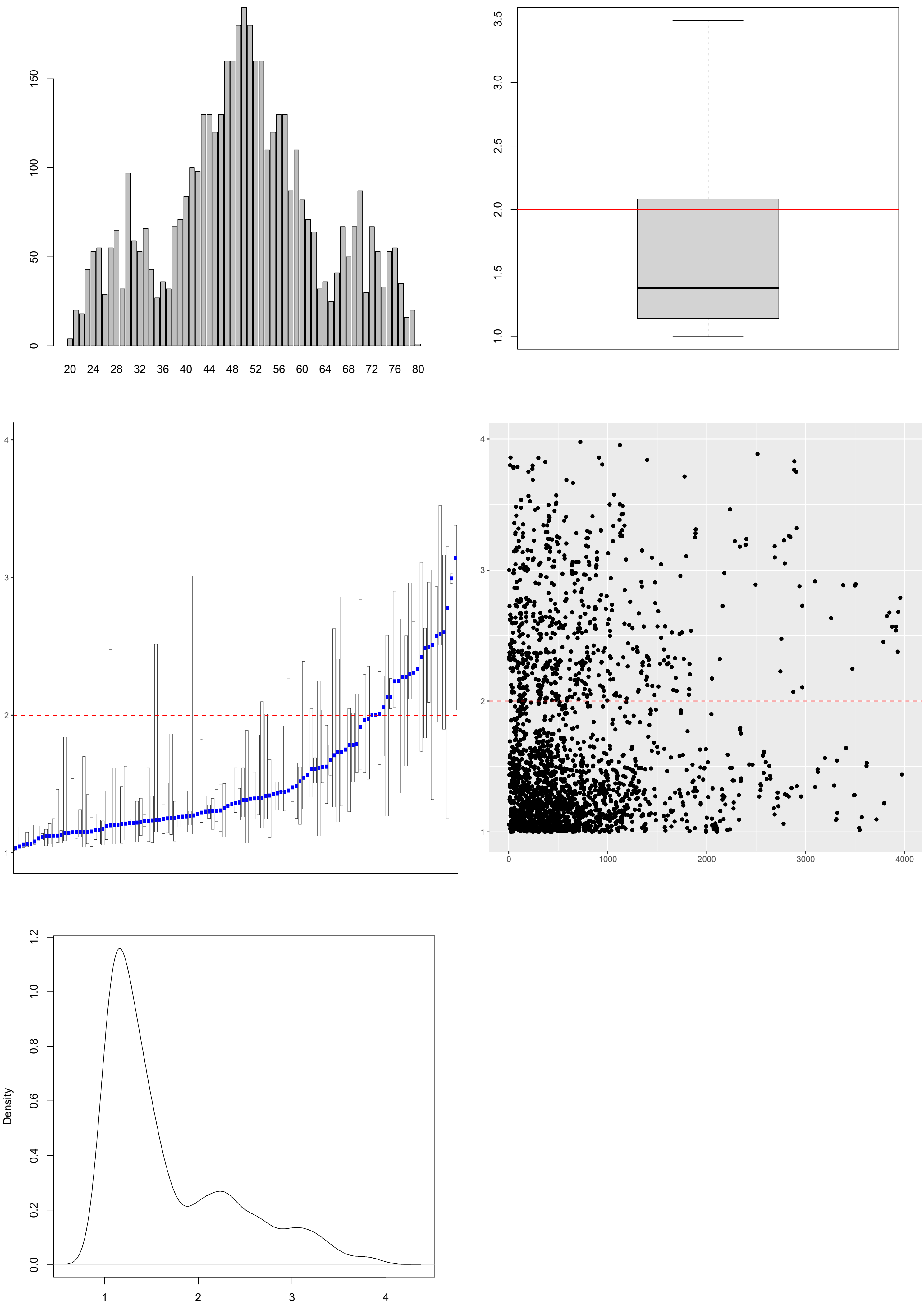

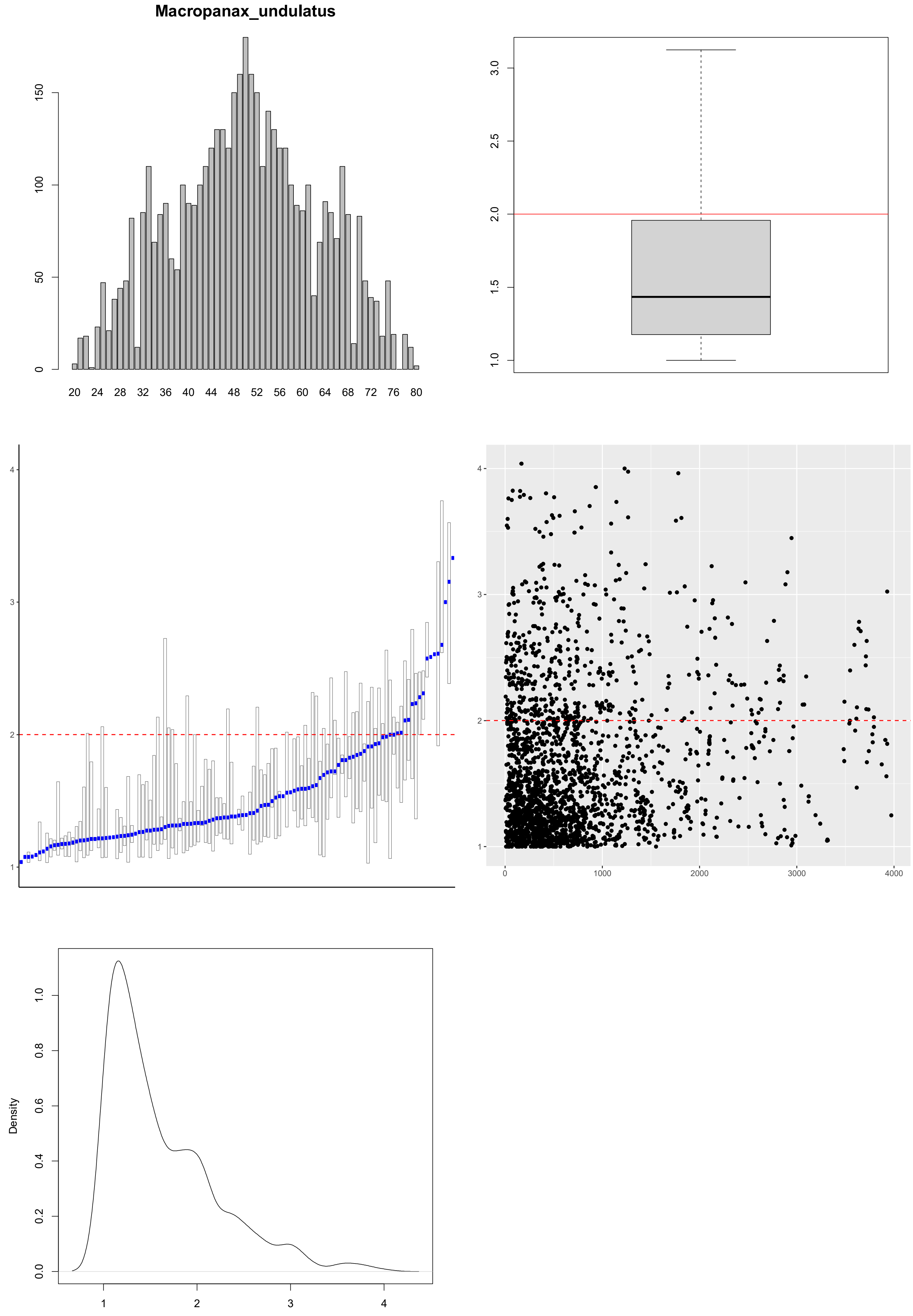
Macropanax_undulatus
0
50
100
150
20 24 28 32 36 40 44 48 52 56 60 64 68 72 76 80
1.0
1.5
2.0
2.5
3.0
1
2
3
4
0
1000
2000
3000
4000
Density
0.0
0.2
0.4
0.6
0.8
1.0

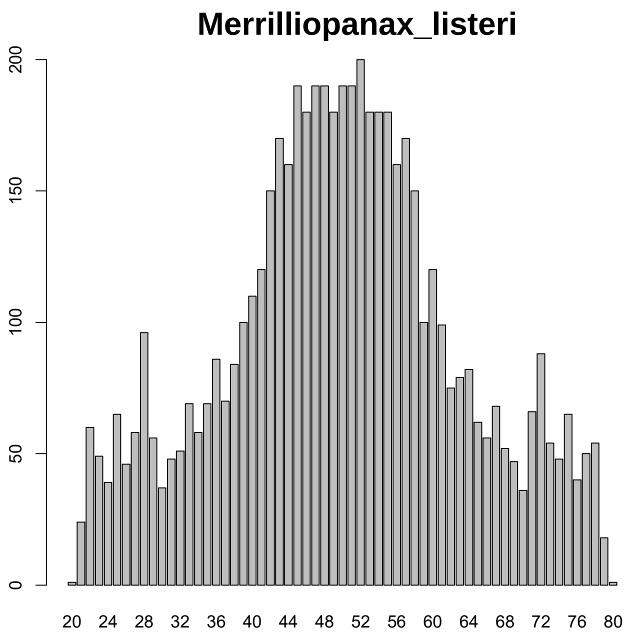
Merrilliopanax_listeri
0
50
100
150
200
20 24 28 32 36 40 44 48 52 56 60 64 68 72 76 80

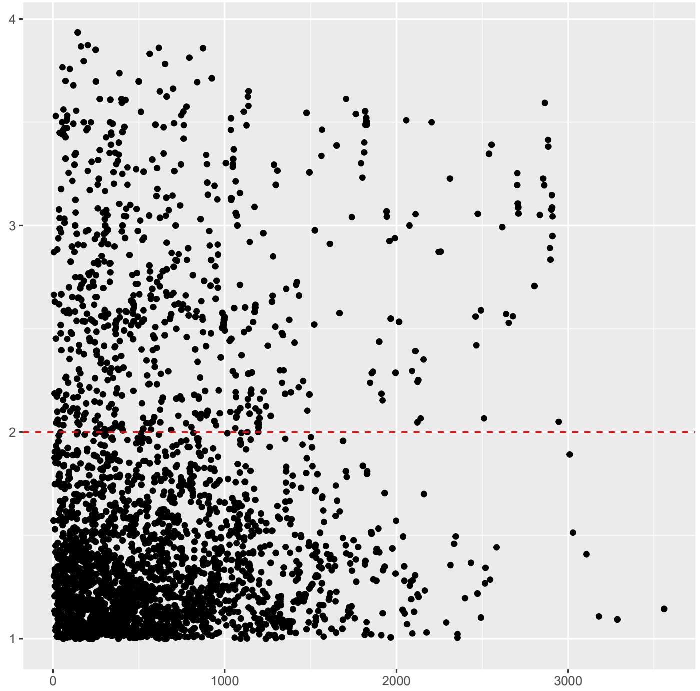
1
2
3
4
0 1000 2000 3000

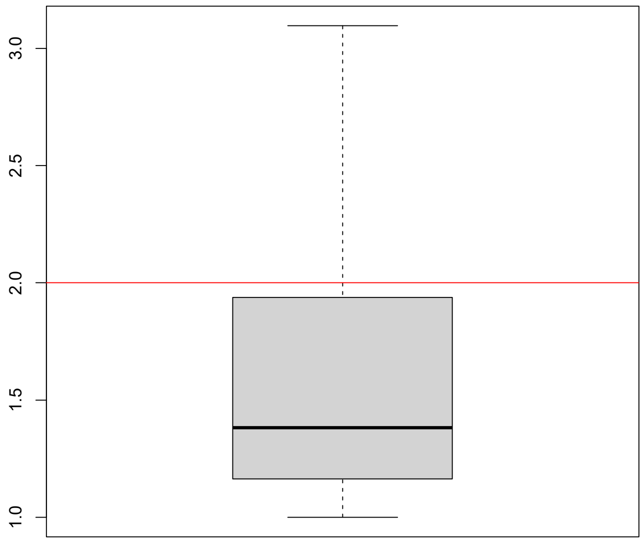
1.0
1.5
2.0
2.5
3.0

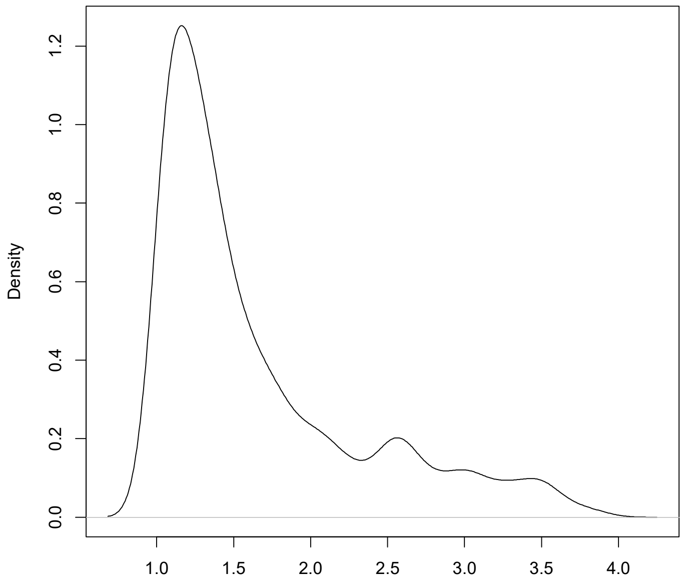
Density
0.0
0.2
0.4
0.6
0.8
1.0
1.2
1.0 1.5 2.0 2.5 3.0 3.5 4.0

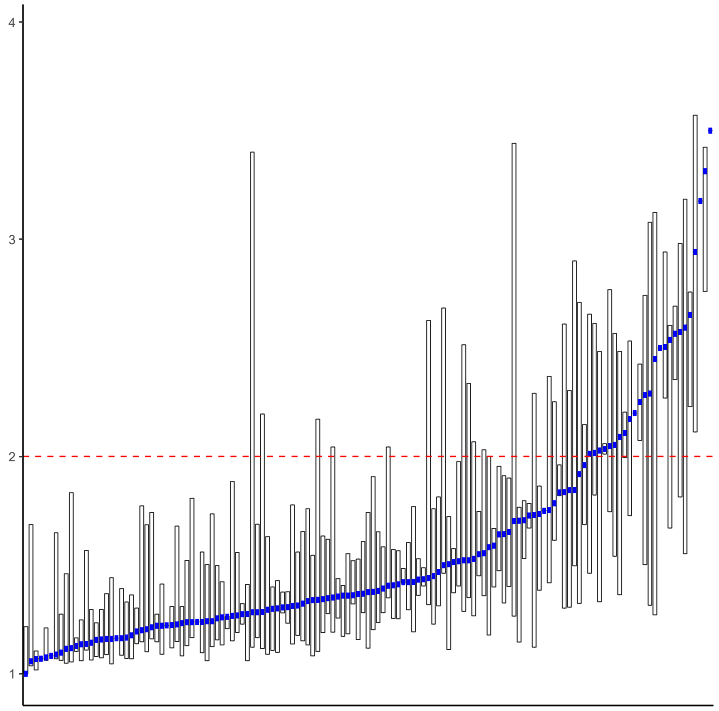
1
2
3
4

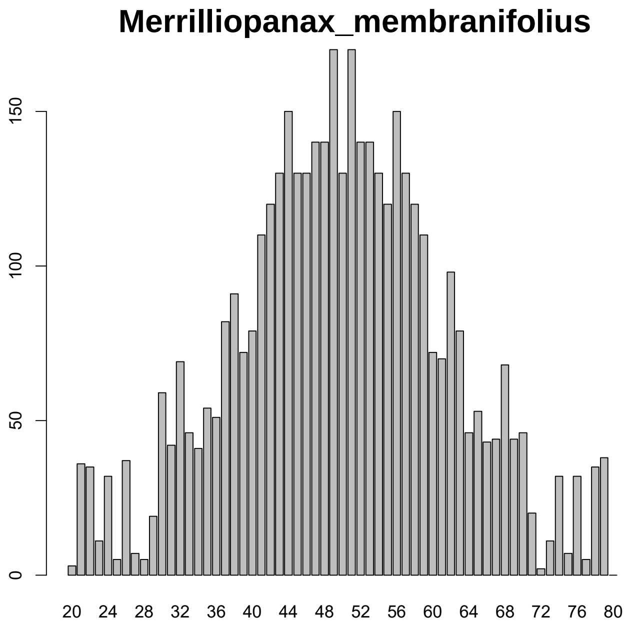
Merrilliopanax_membranifolius
0
50
100
150
20 24 28 32 36 40 44 48 52 56 60 64 68 72 76 80

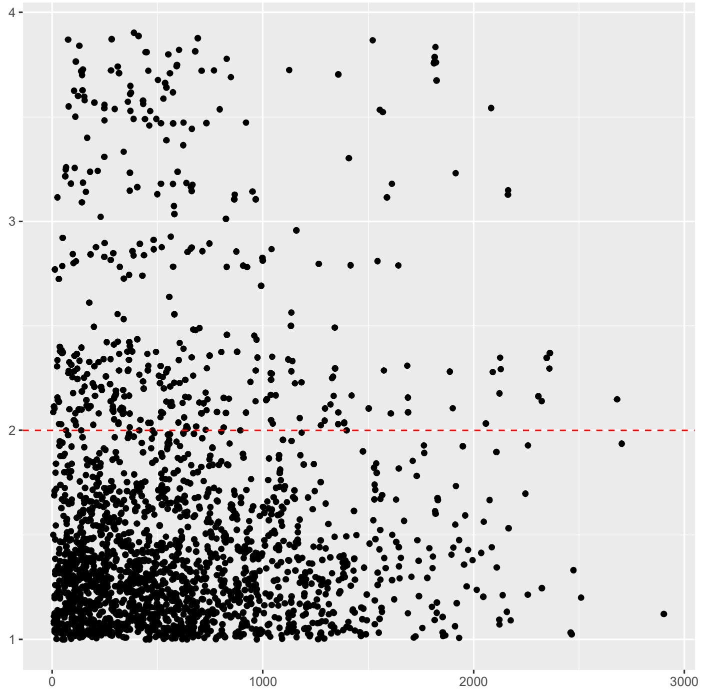
1
2
3
4
0 1000 2000 3000

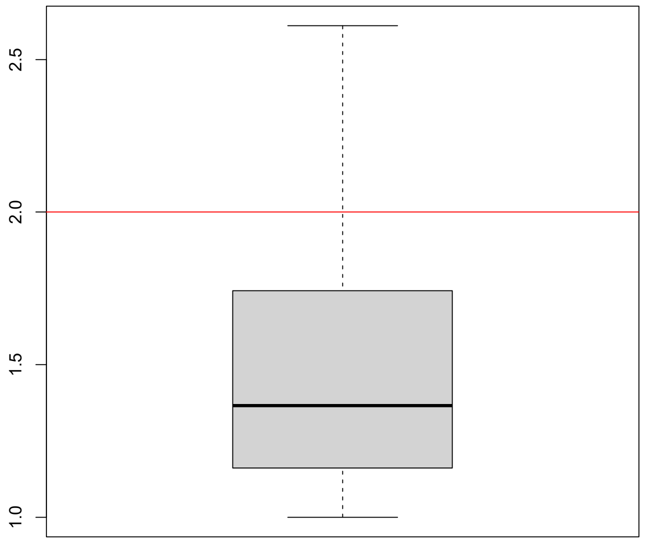
1.0
1.5
2.0
2.5

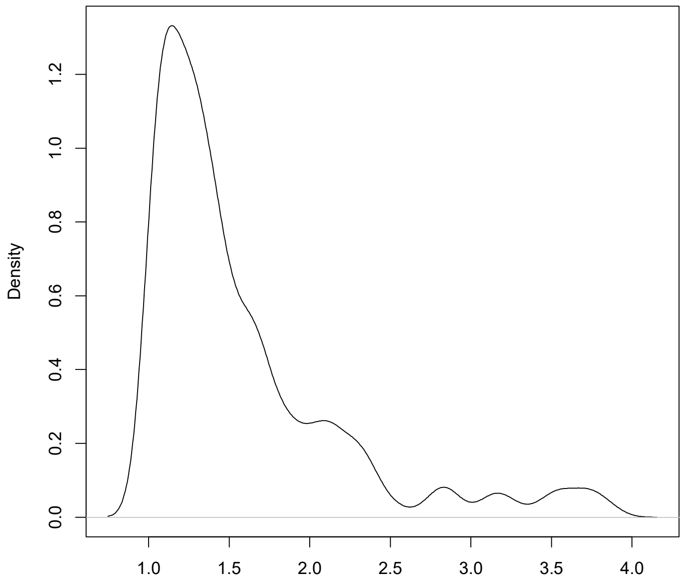
Density
0.0
0.2
0.4
0.6
0.8
1.0
1.2
1.0 1.5 2.0 2.5 3.0 3.5 4.0

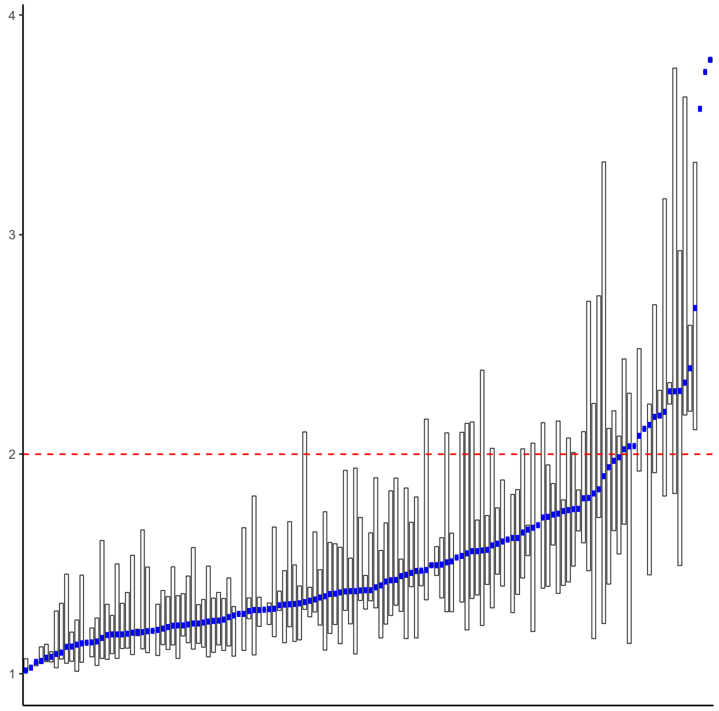
1
2
3
4

Meryta_pastoralis

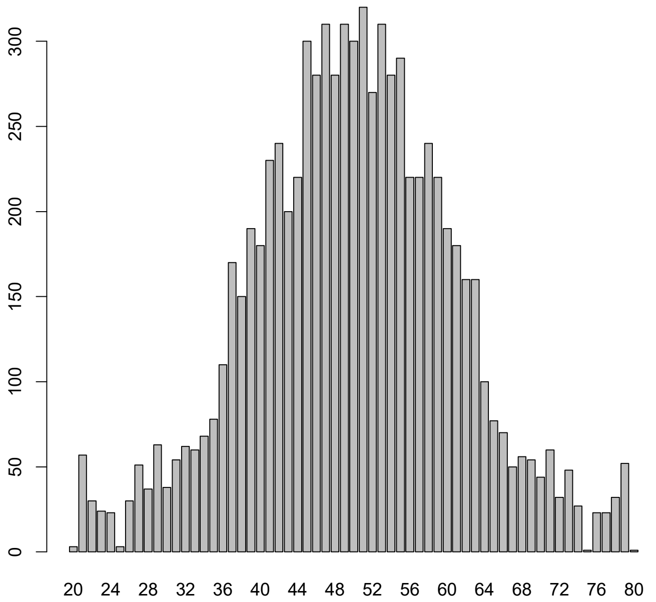

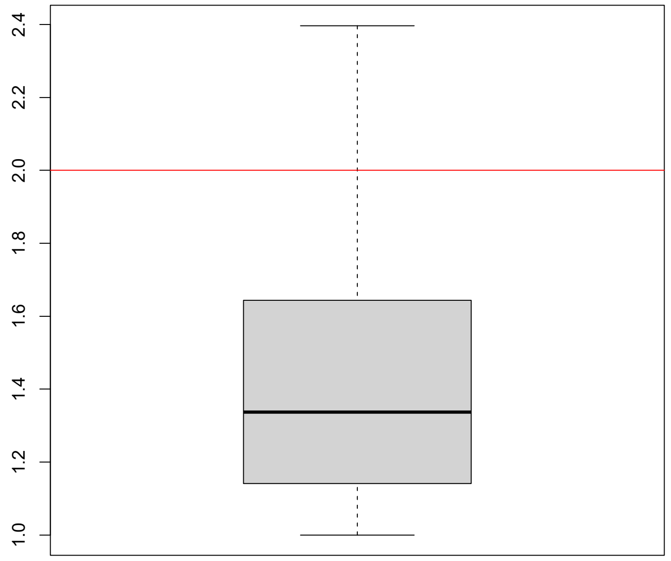

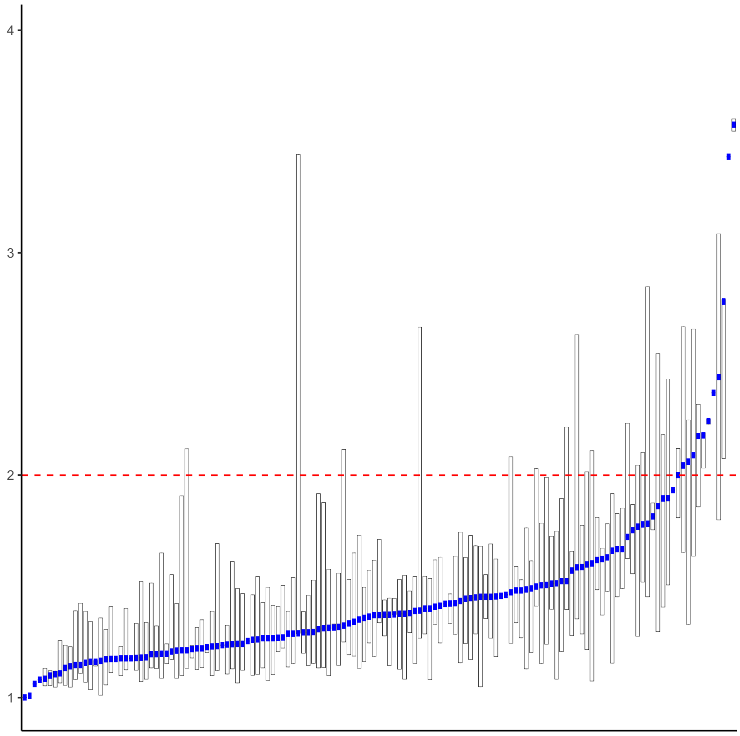

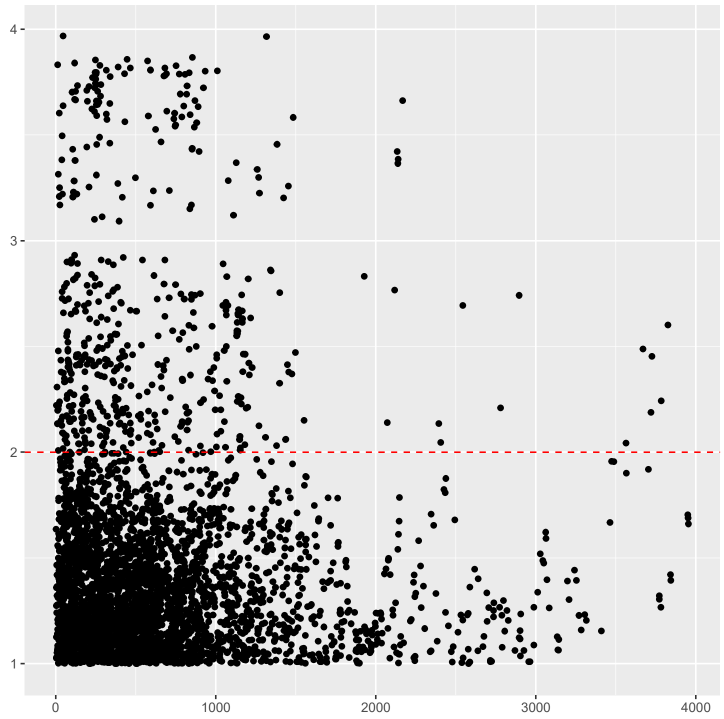

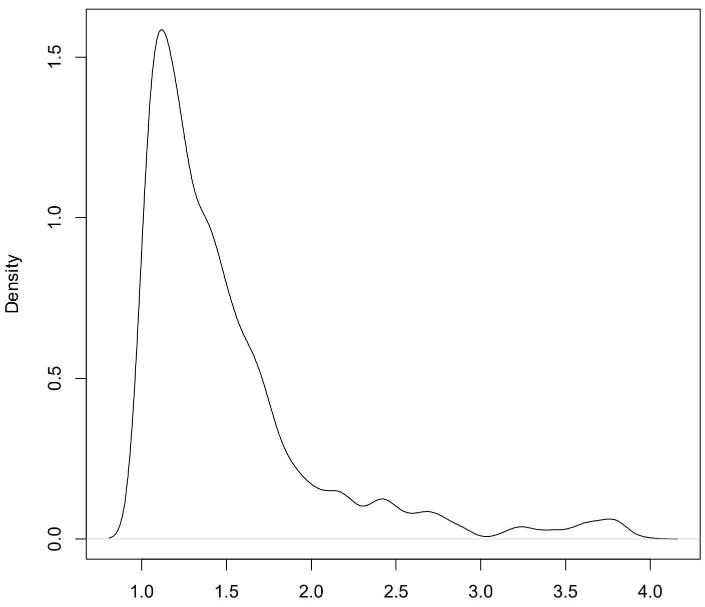

Metapanax_davidii

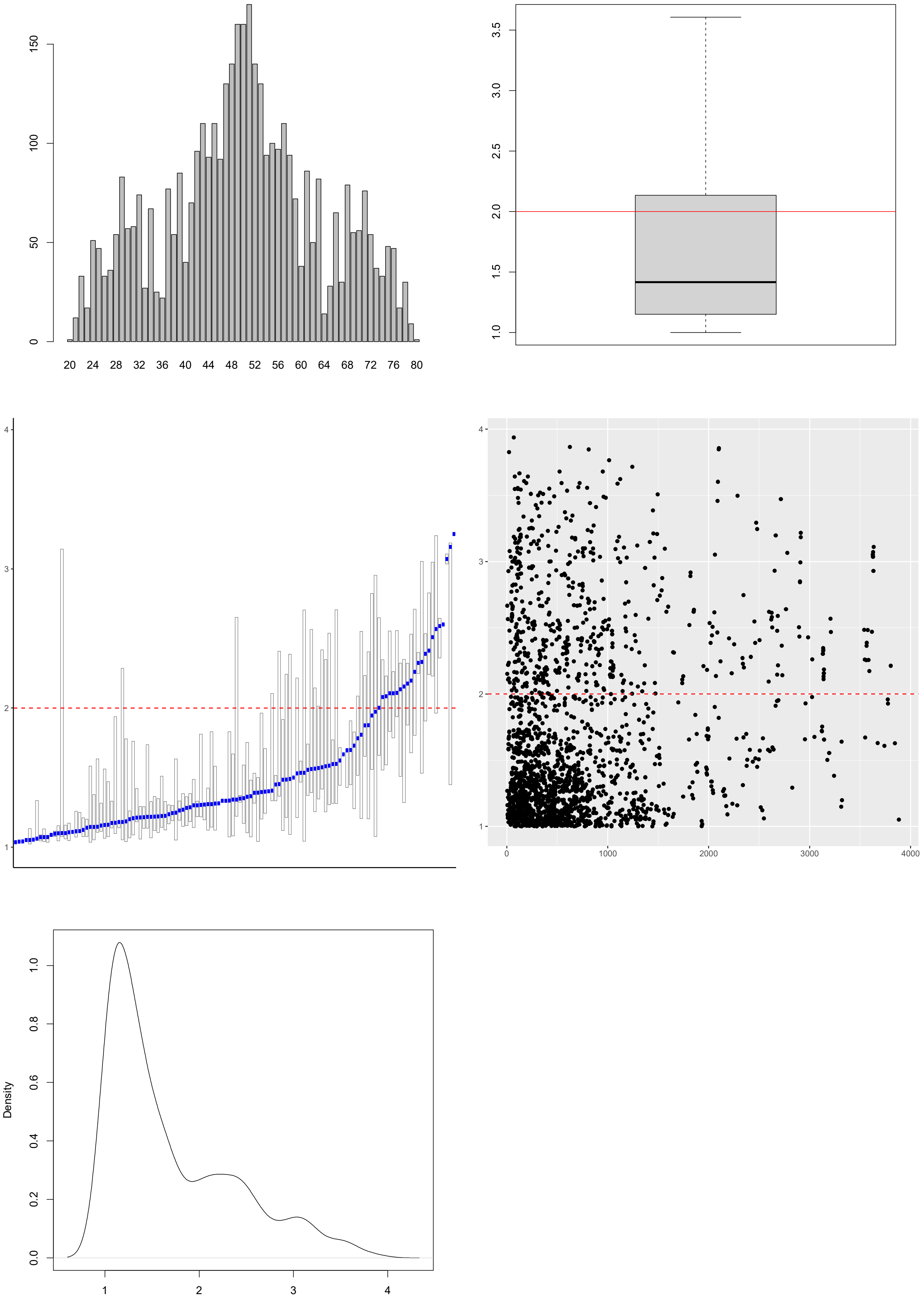

Metapanax_delavayi

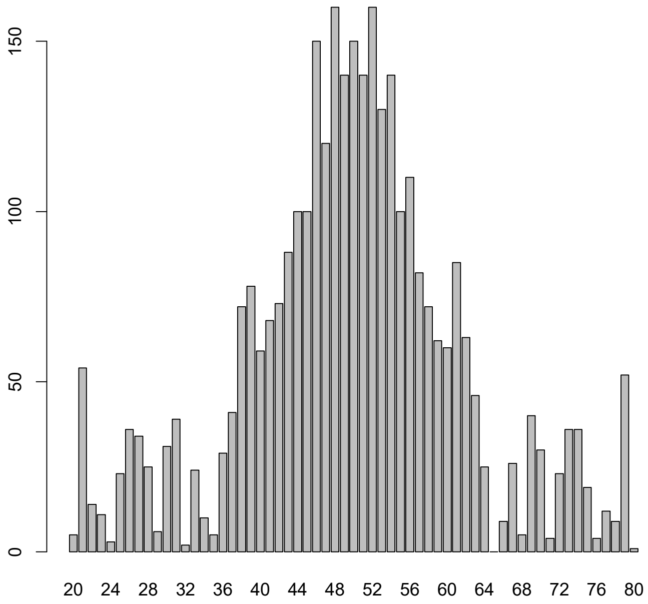

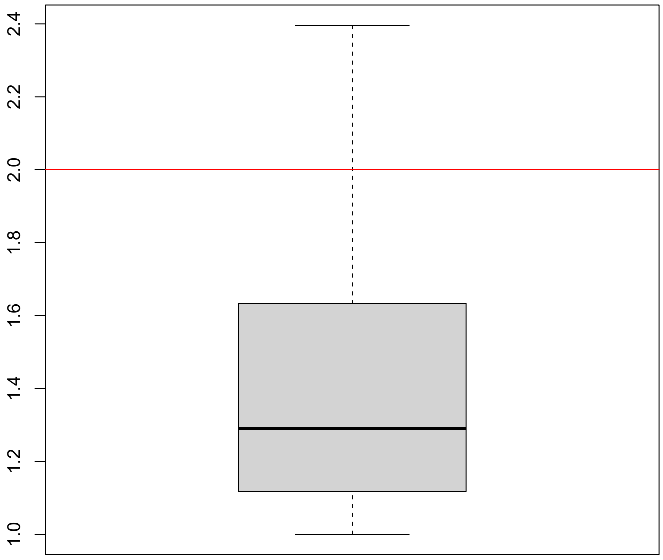

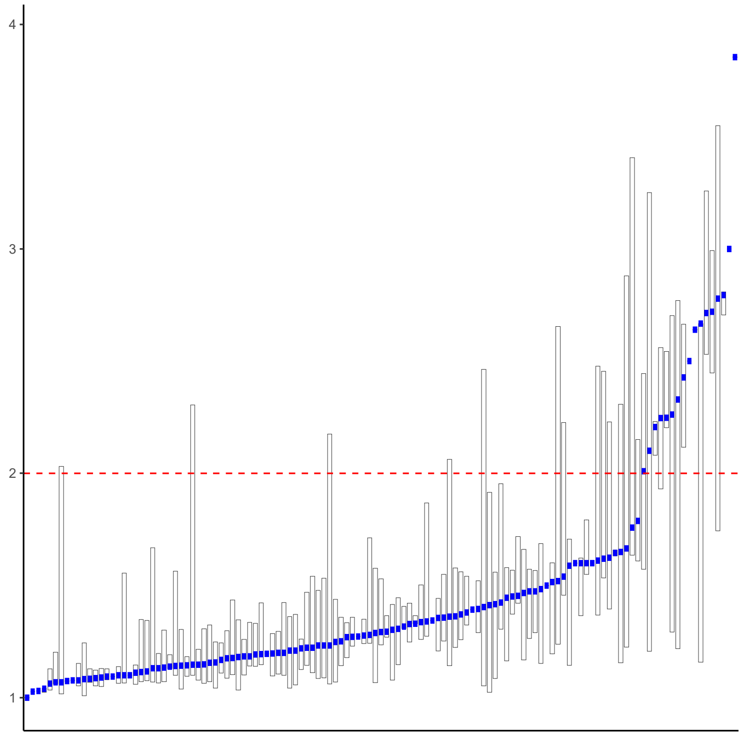

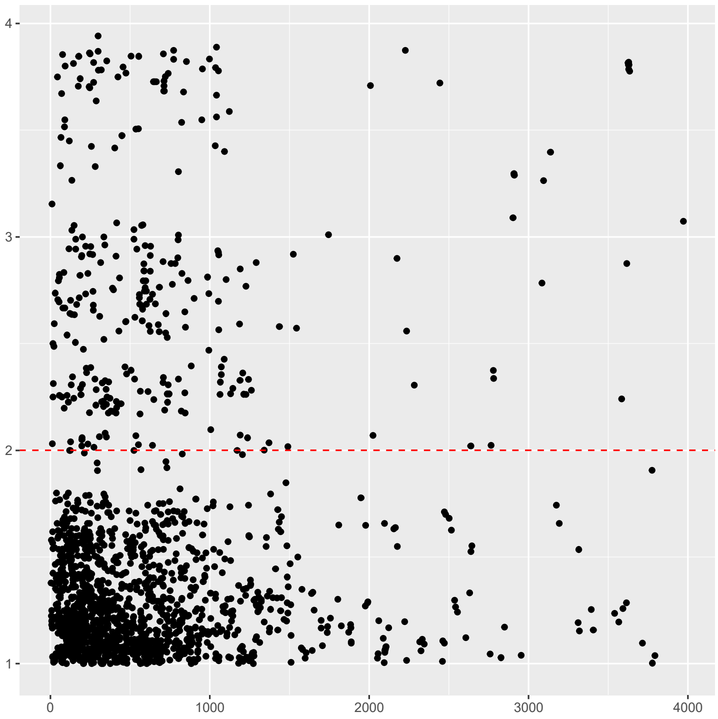

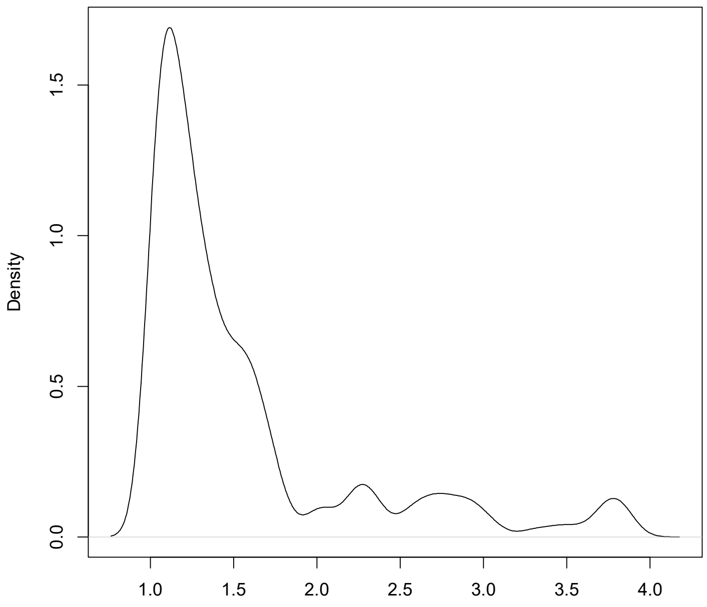

Oplopanax_elatus

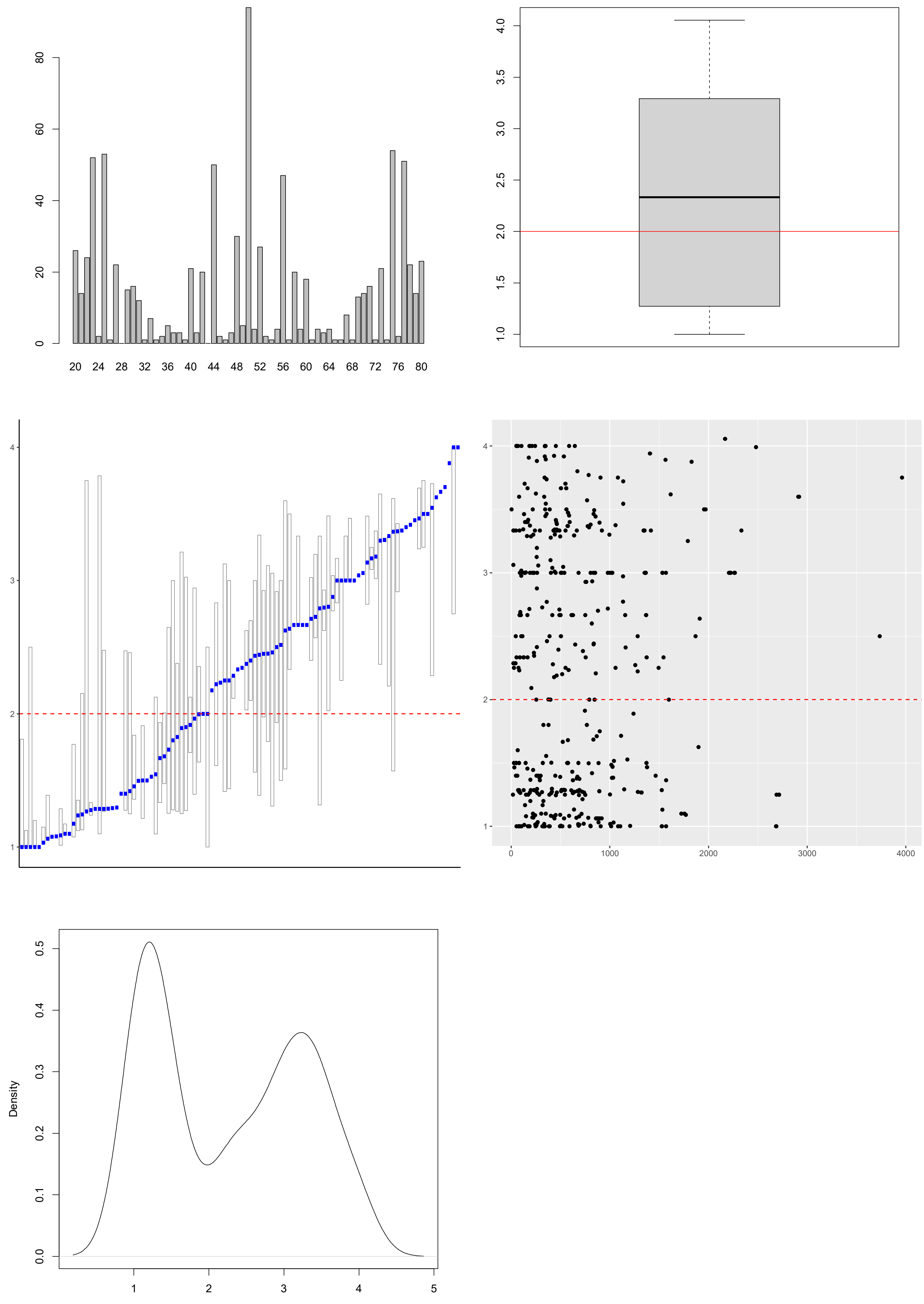

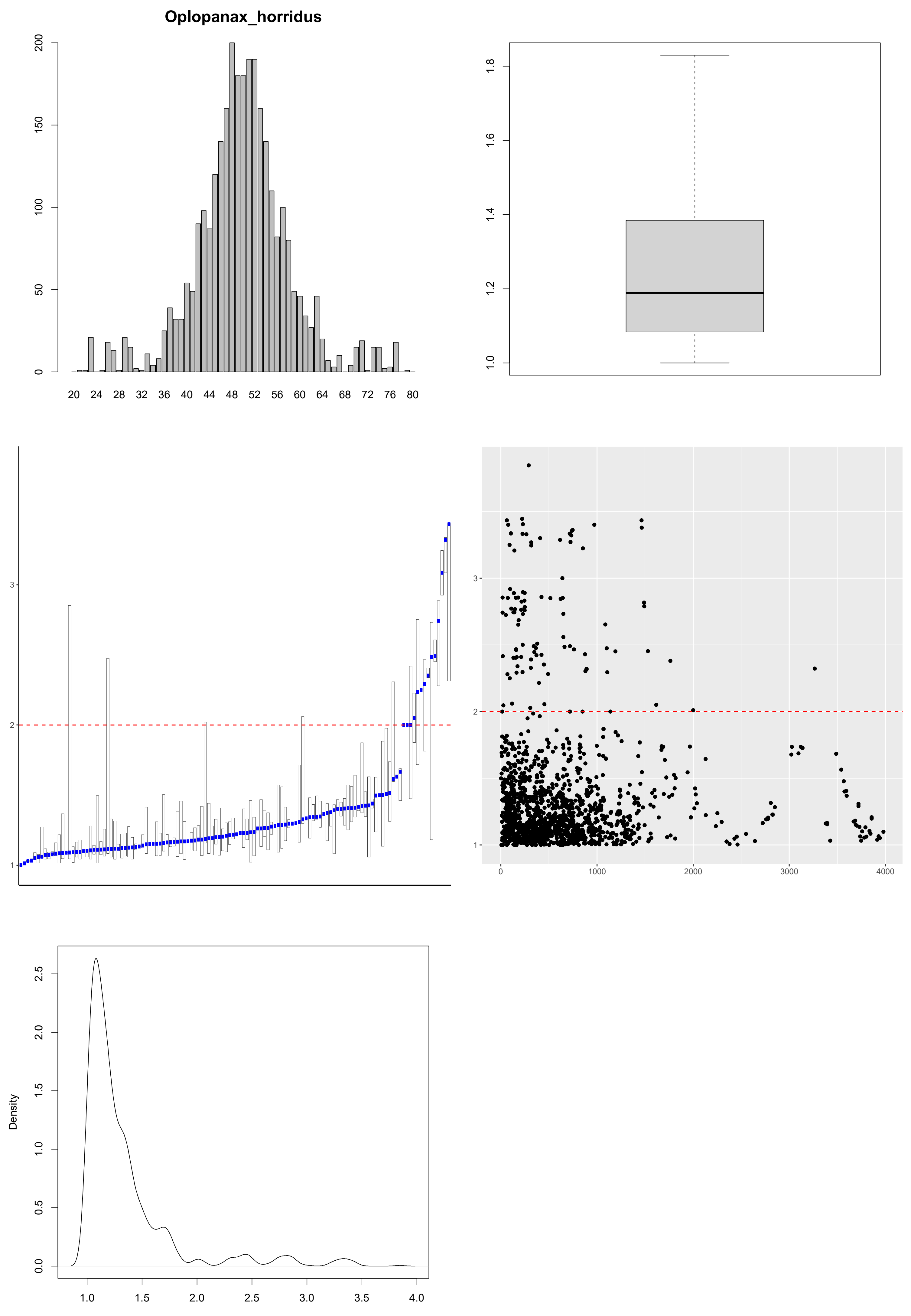
Oplopanax_horridus
Density

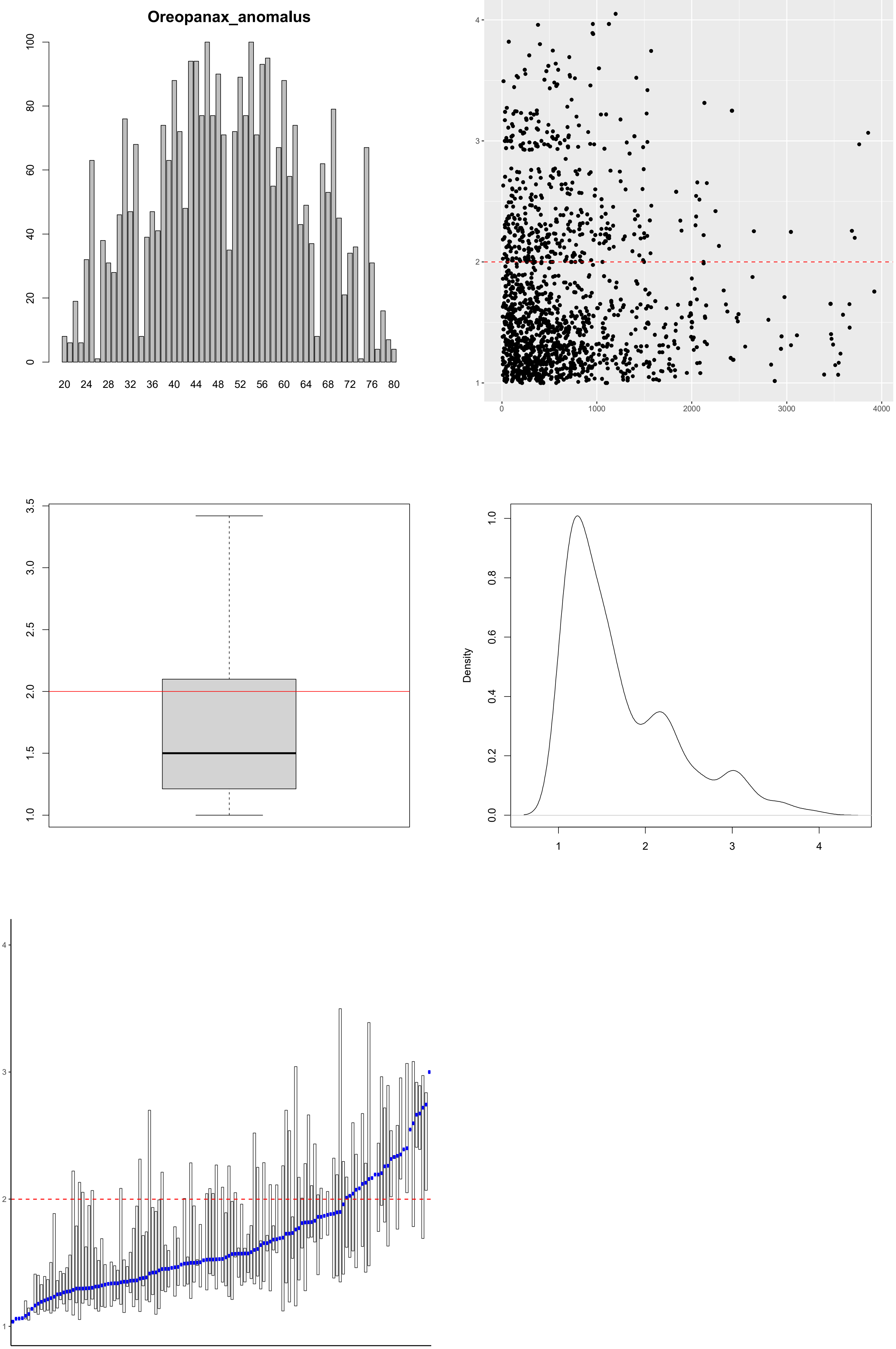
Oreopanax_anomalus
Density

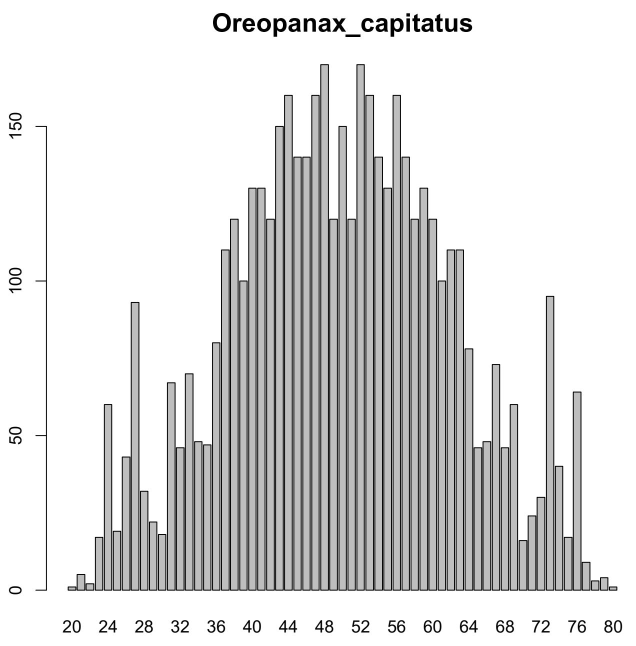
Oreopanax_capitatus
0
50
100
150
20 24 28 32 36 40 44 48 52 56 60 64 68 72 76 80

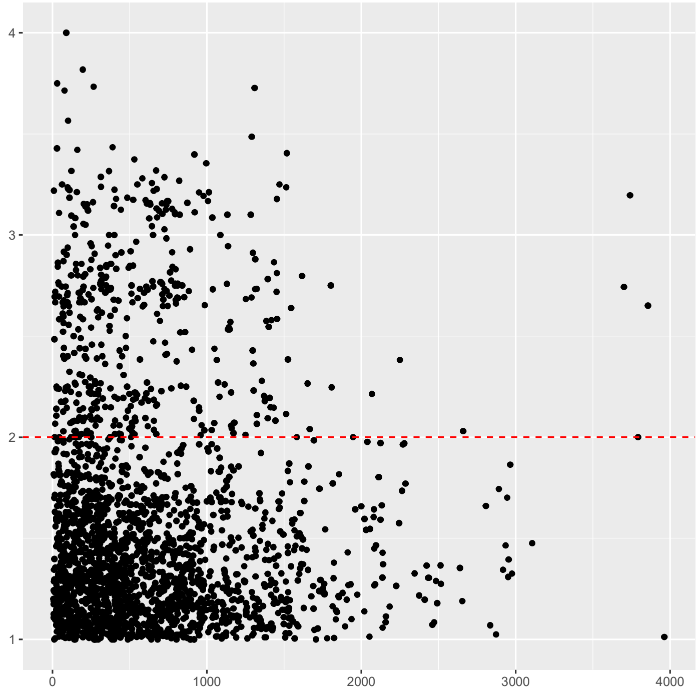
1
2
3
4
0 1000 2000 3000 4000

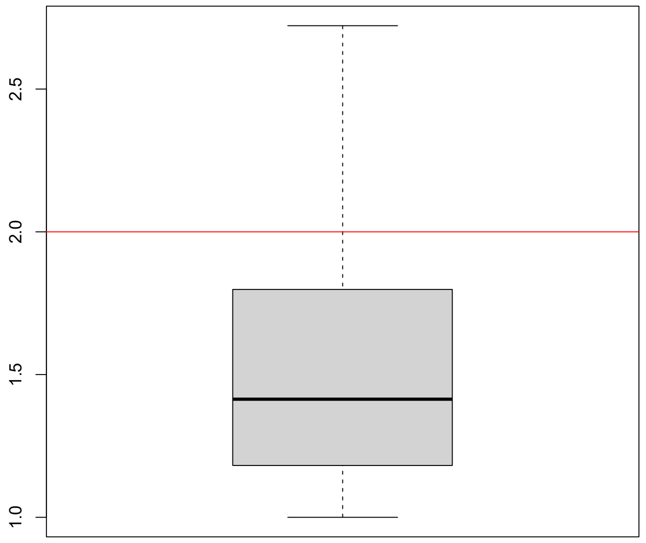
1.0
1.5
2.0
2.5

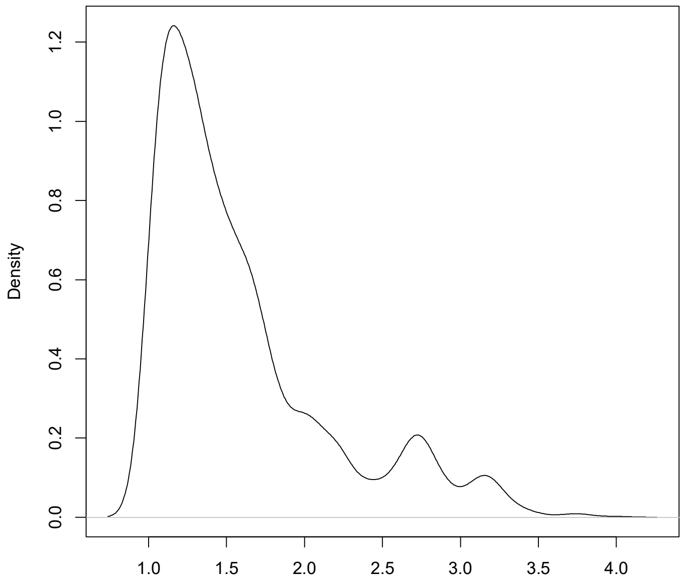
Density
0.0
0.2
0.4
0.6
0.8
1.0
1.2
1.0 1.5 2.0 2.5 3.0 3.5 4.0

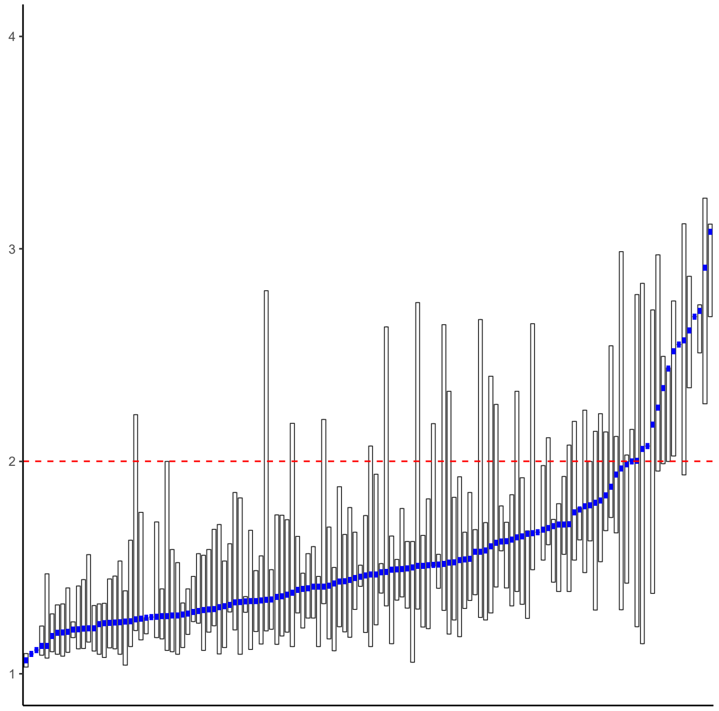
1
2
3
4

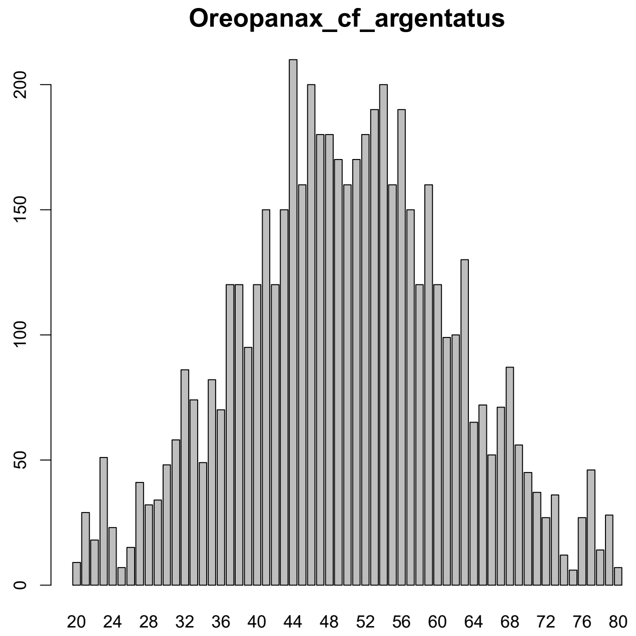
Oreopanax_cf_argentatus

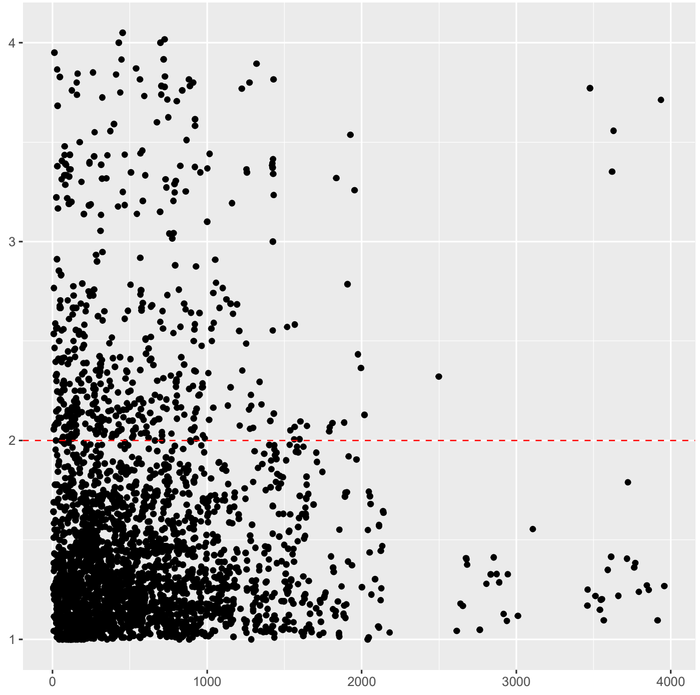

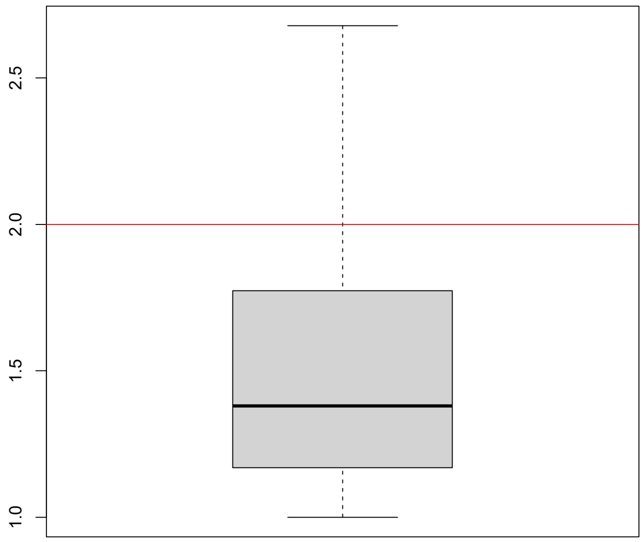

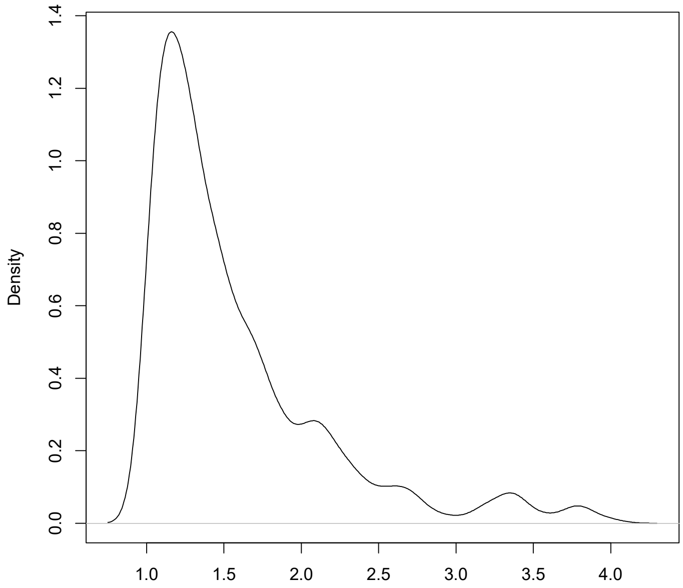
Density

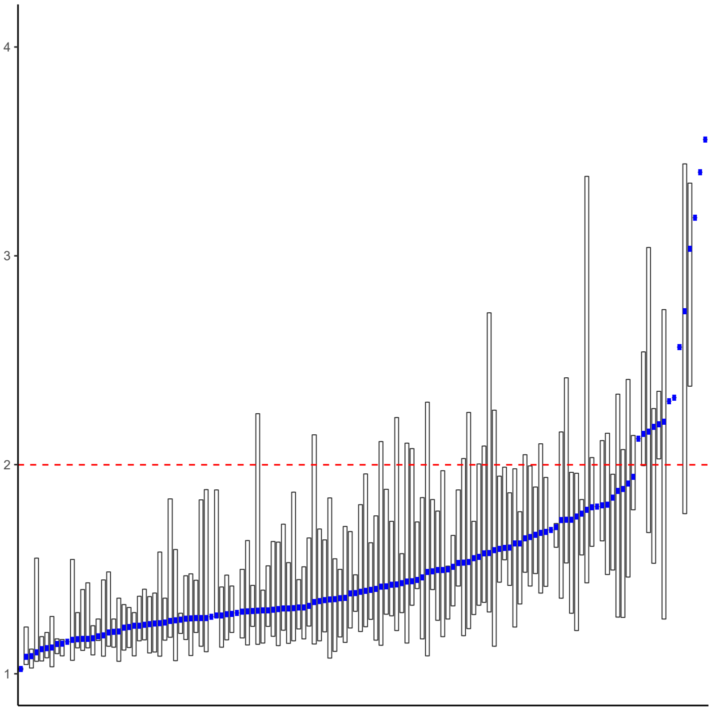

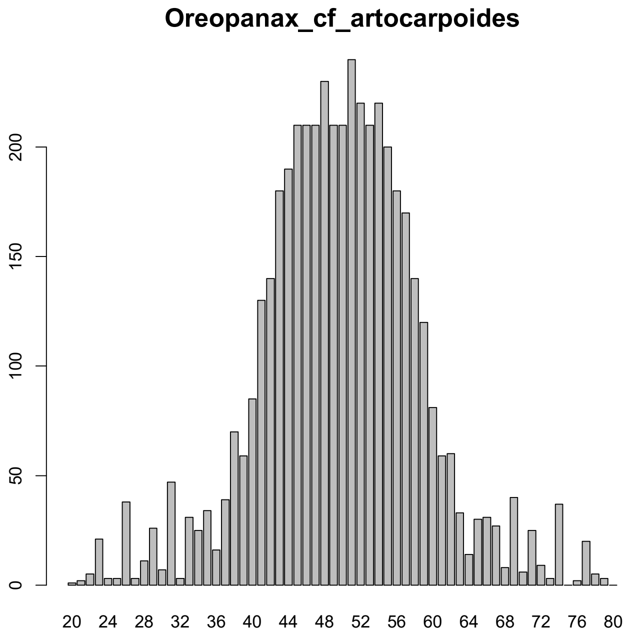
Oreopanax_cf_artocarpoides
0
50
100
150
200
20 24 28 32 36 40 44 48 52 56 60 64 68 72 76 80

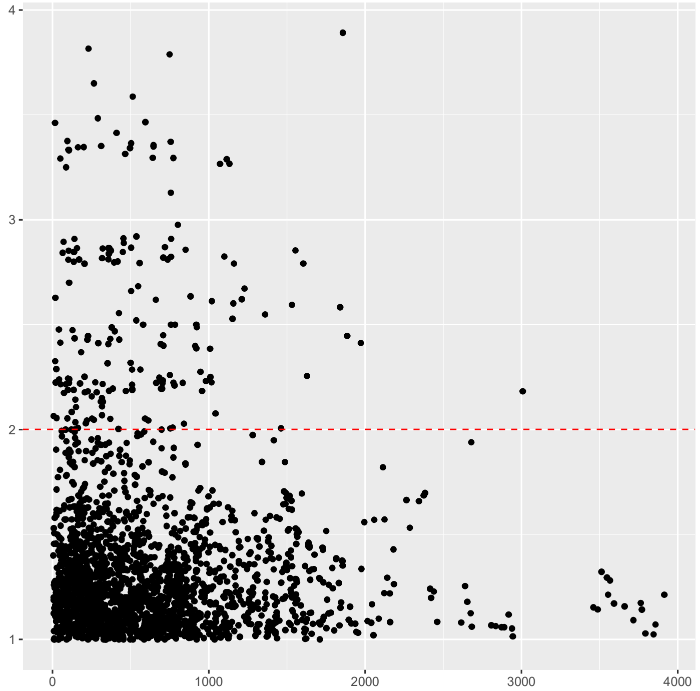
1
2
3
4
0 1000 2000 3000 4000

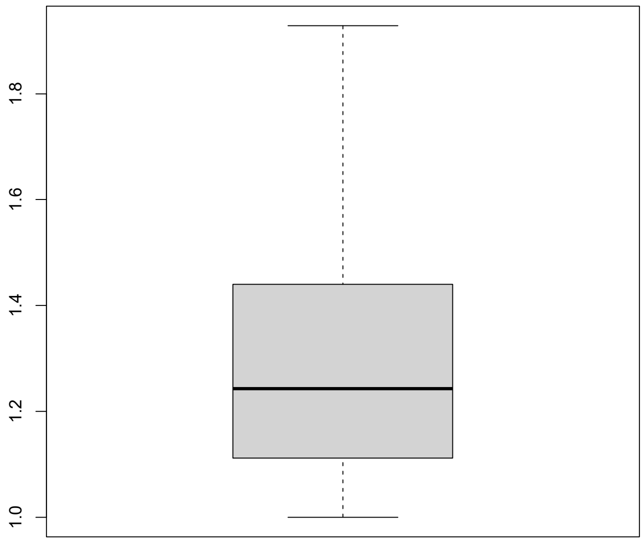
1.0
1.2
1.4
1.6
1.8

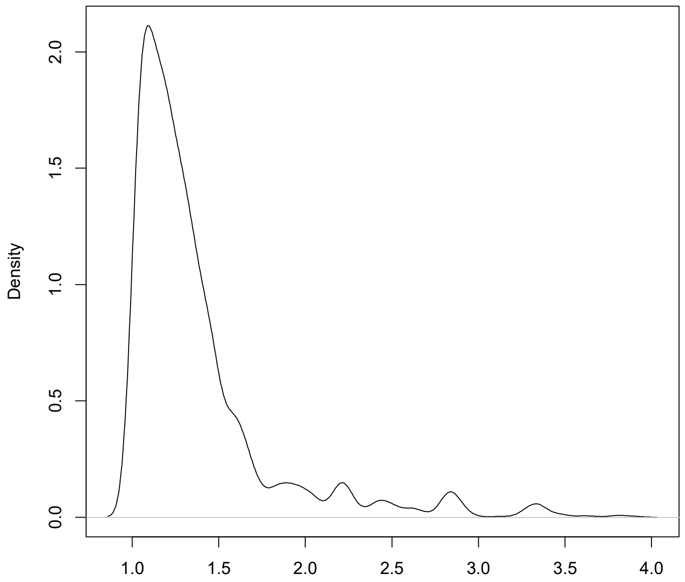
Density
0.0
0.5
1.0
1.5
2.0
1.0 1.5 2.0 2.5 3.0 3.5 4.0

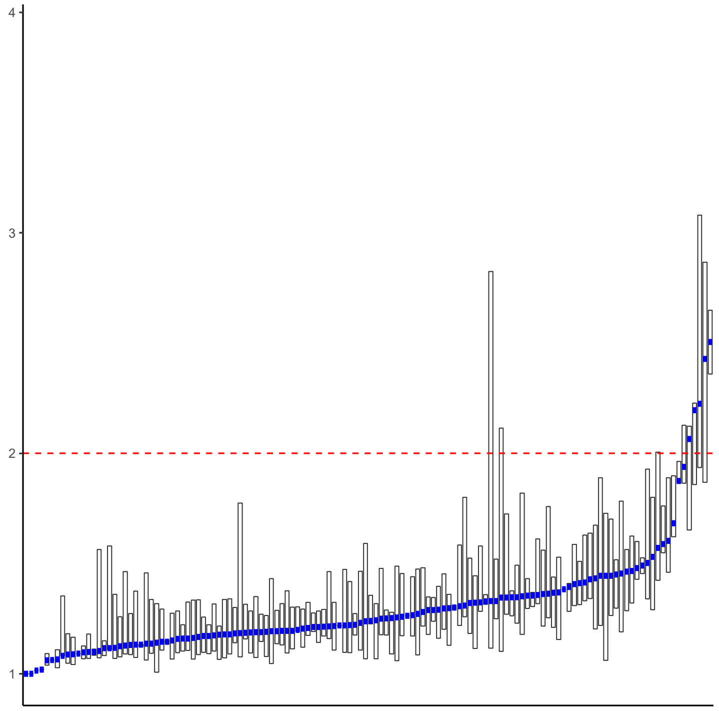
1
2
3
4

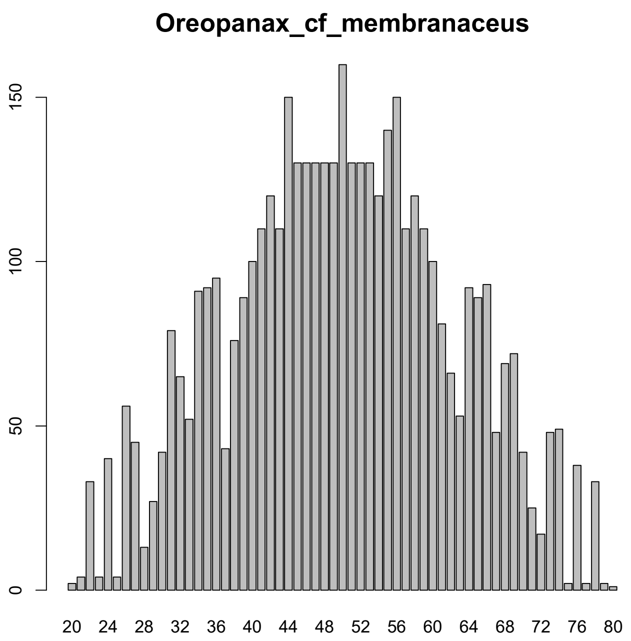
Oreopanax_cf_membranaceus
0
50
100
150
20 24 28 32 36 40 44 48 52 56 60 64 68 72 76 80

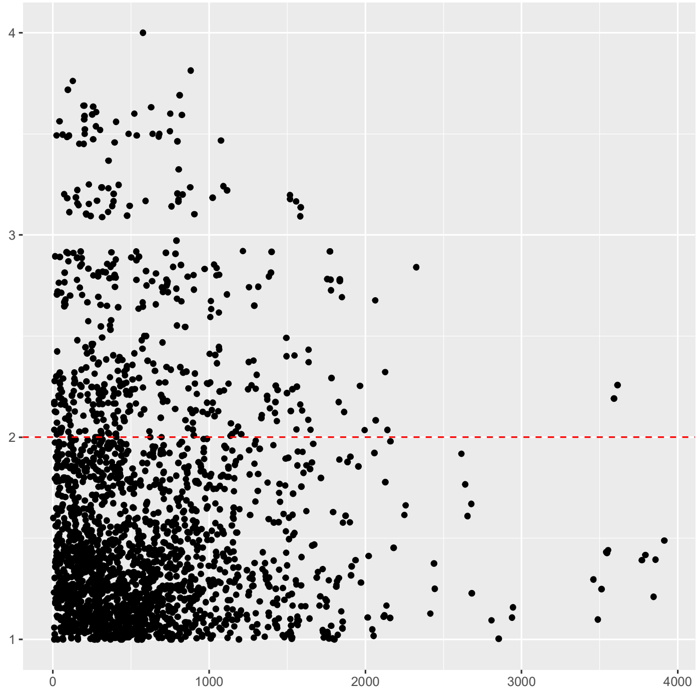
1
2
3
4
0 1000 2000 3000 4000

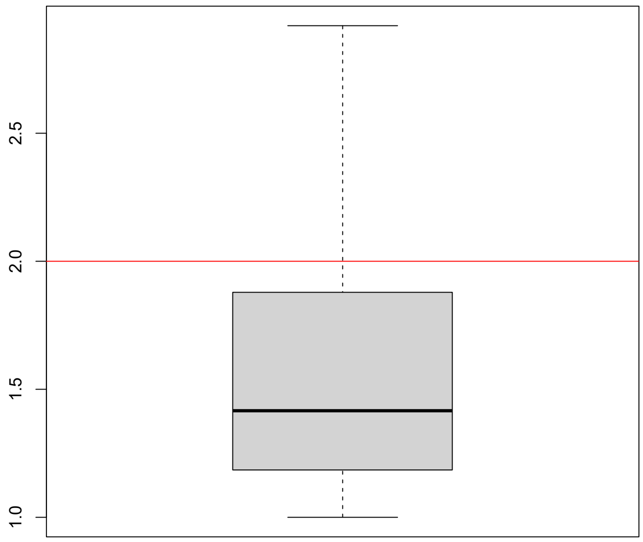
1.0
1.5
2.0
2.5

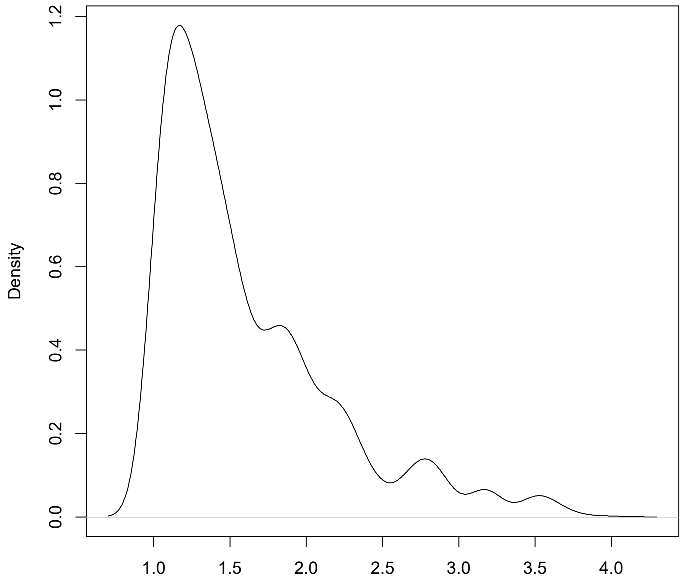
Density
0.0
0.2
0.4
0.6
0.8
1.0
1.2
1.0 1.5 2.0 2.5 3.0 3.5 4.0

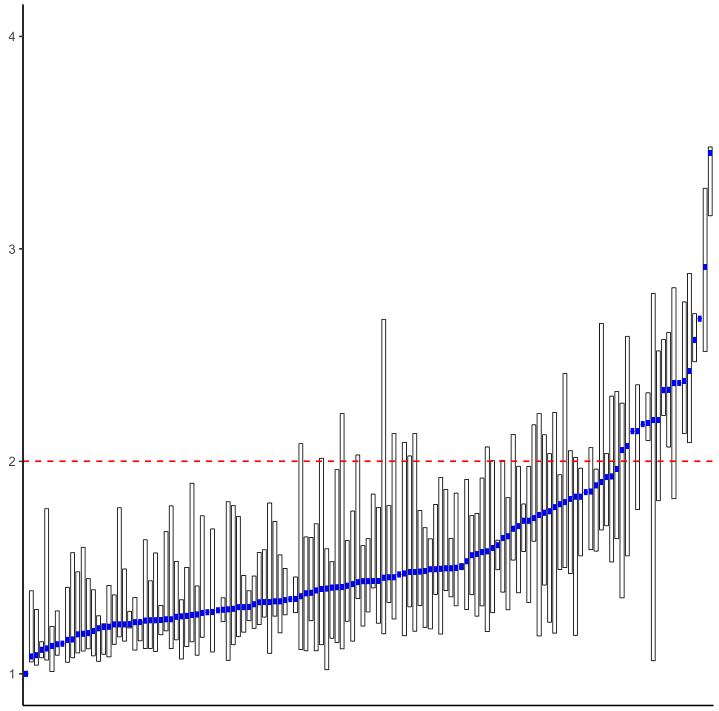
1
2
3
4

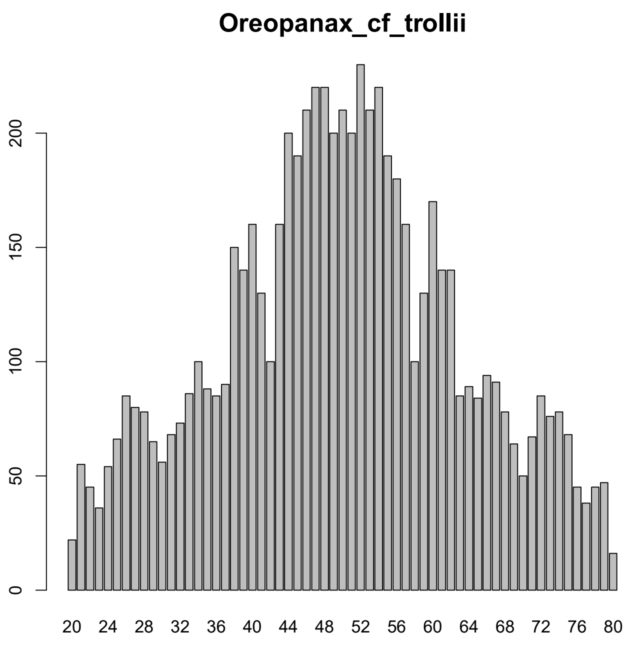

Oreopanax_cf_trollii
0
50
100
150
200
20
24
28
32
36
40
44
48
52
56
60
64
68
72
76
80

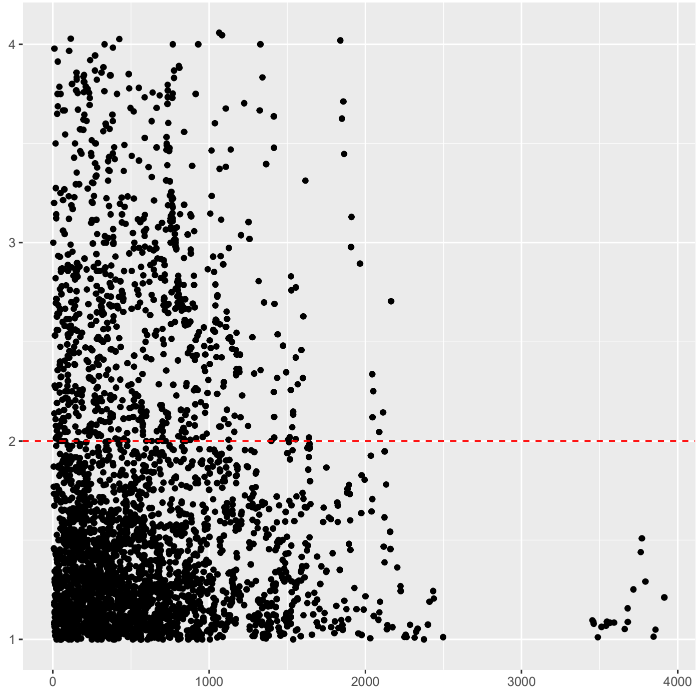

1
2
3
4
0
1000
2000
3000
4000

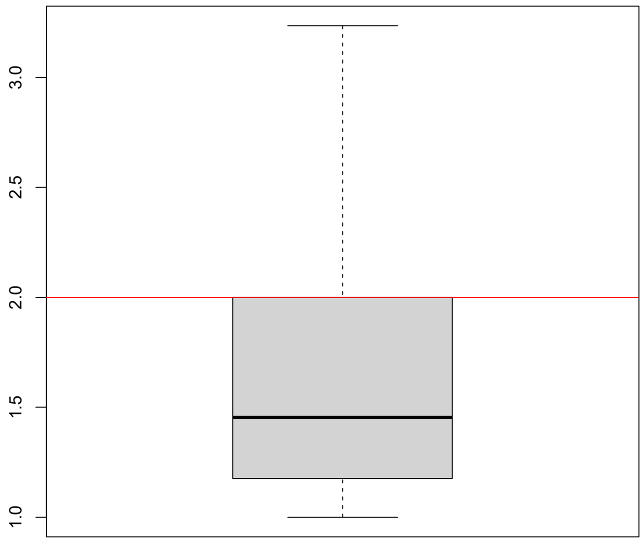

1.0
1.5
2.0
2.5
3.0

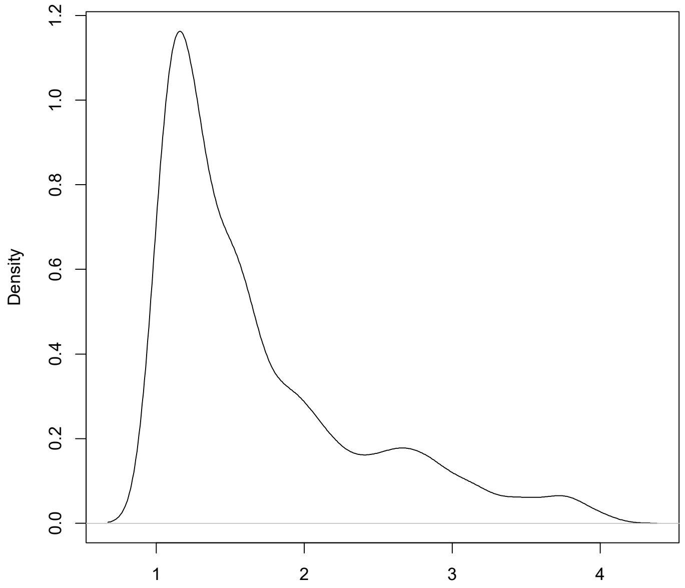

Density
0.0
0.2
0.4
0.6
0.8
1.0
1.2
1
2
3
4

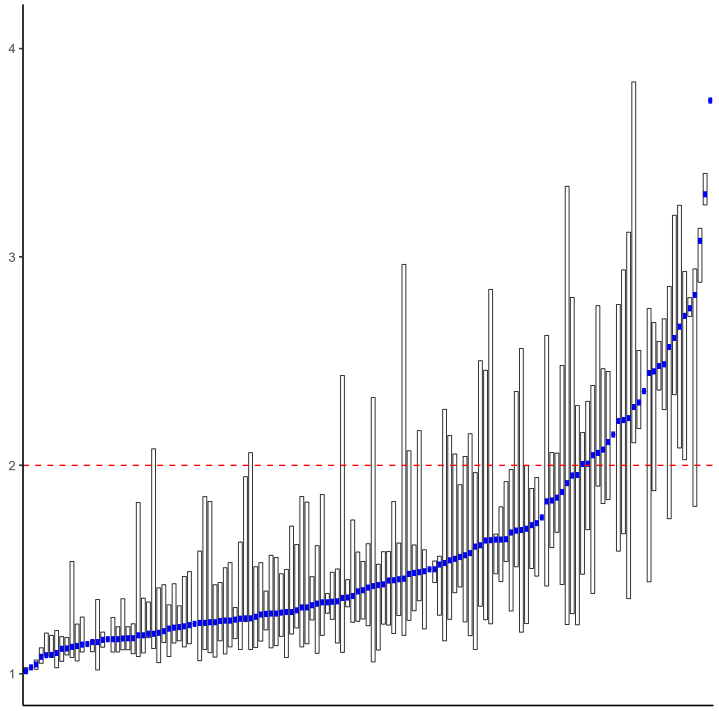

1
2
3
4

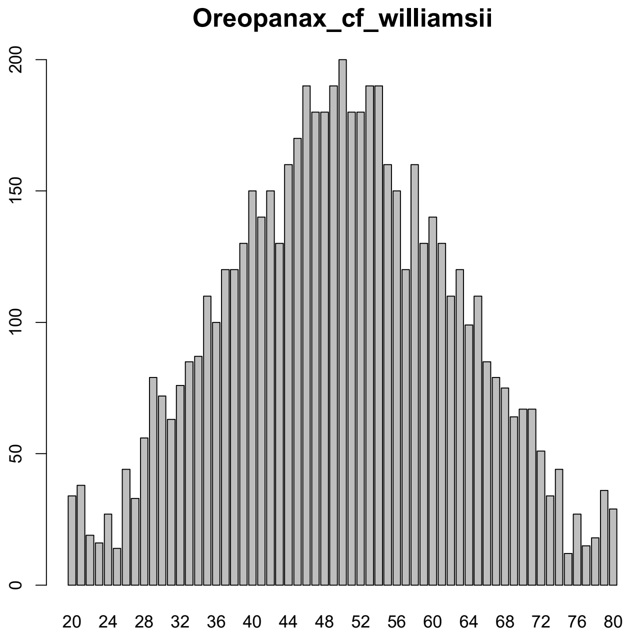
Oreopanax_cf_williamsii
0
50
100
150
200
20 24 28 32 36 40 44 48 52 56 60 64 68 72 76 80

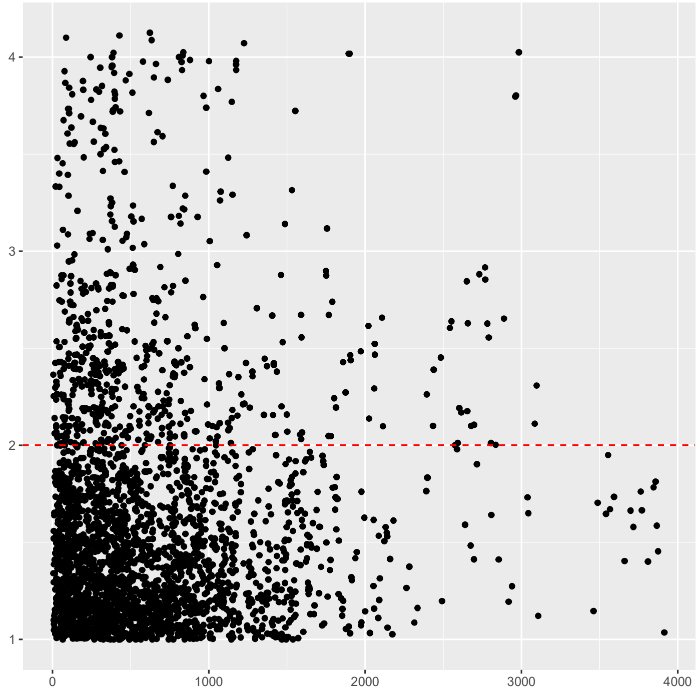
1
2
3
4
0 1000 2000 3000 4000

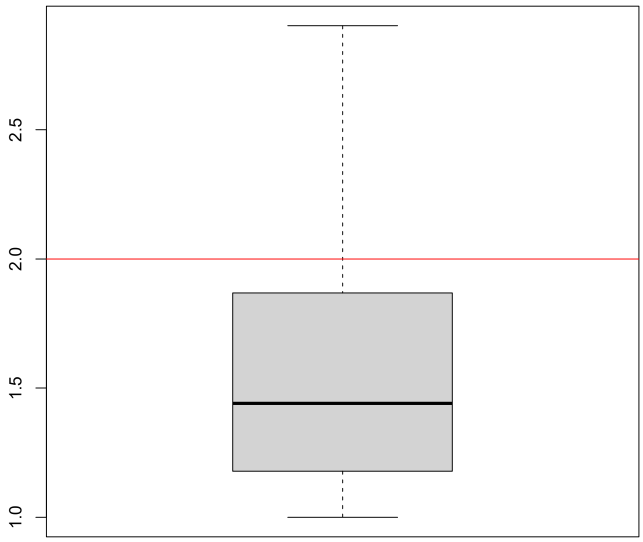
1.0
1.5
2.0
2.5

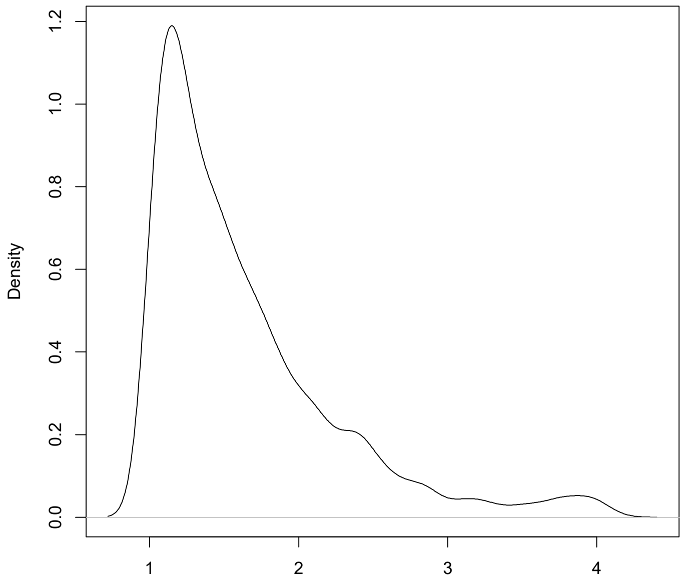
Density
0.0
0.2
0.4
0.6
0.8
1.0
1.2
1 2 3 4

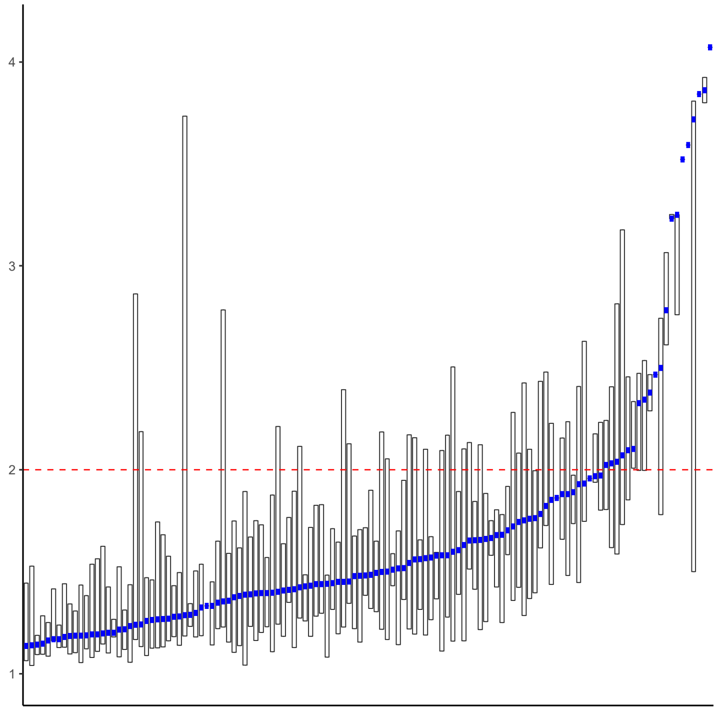
1
2
3
4

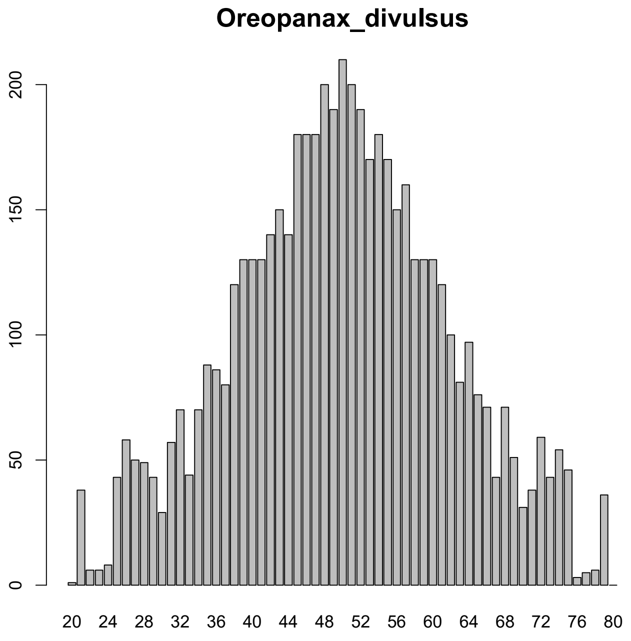
Oreopanax_divulsus
0
50
100
150
200
20 24 28 32 36 40 44 48 52 56 60 64 68 72 76 80

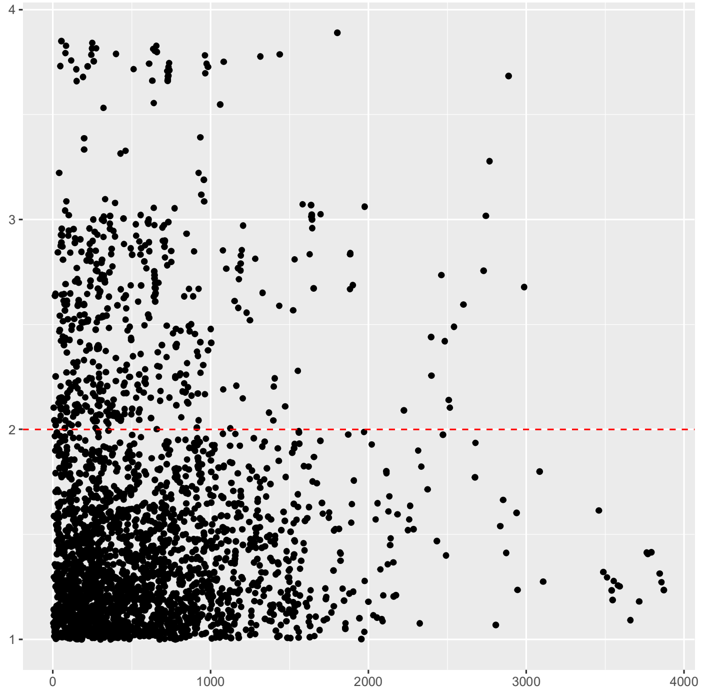
1
2
3
4
0 1000 2000 3000 4000

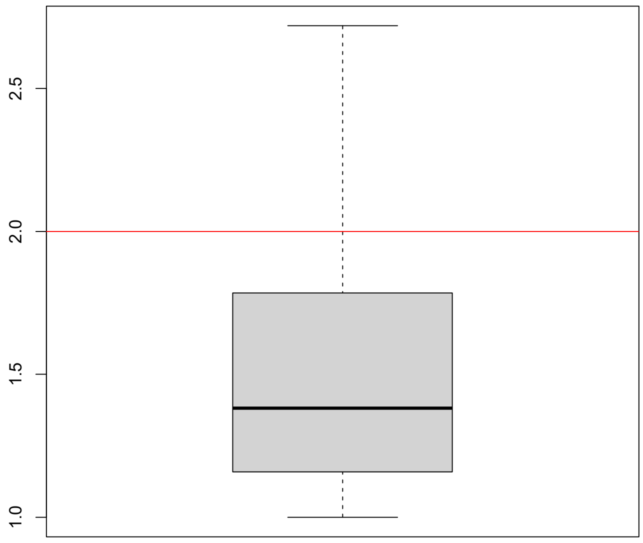
1.0
1.5
2.0
2.5

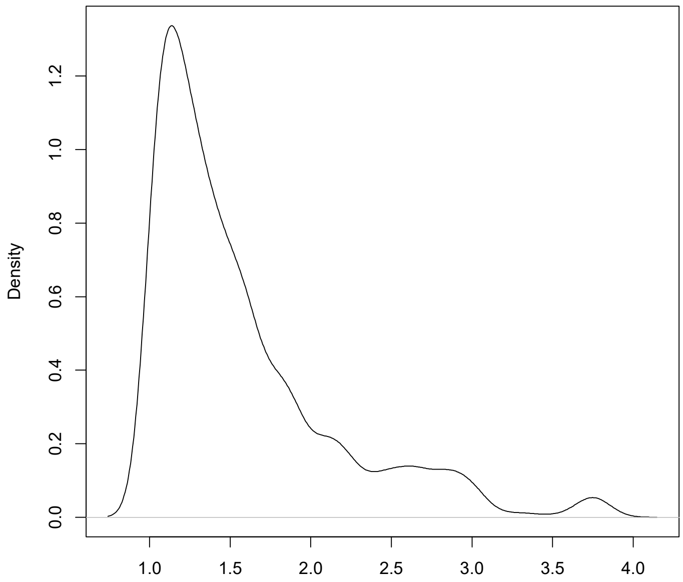
Density
0.0
0.2
0.4
0.6
0.8
1.0
1.2
1.0 1.5 2.0 2.5 3.0 3.5 4.0

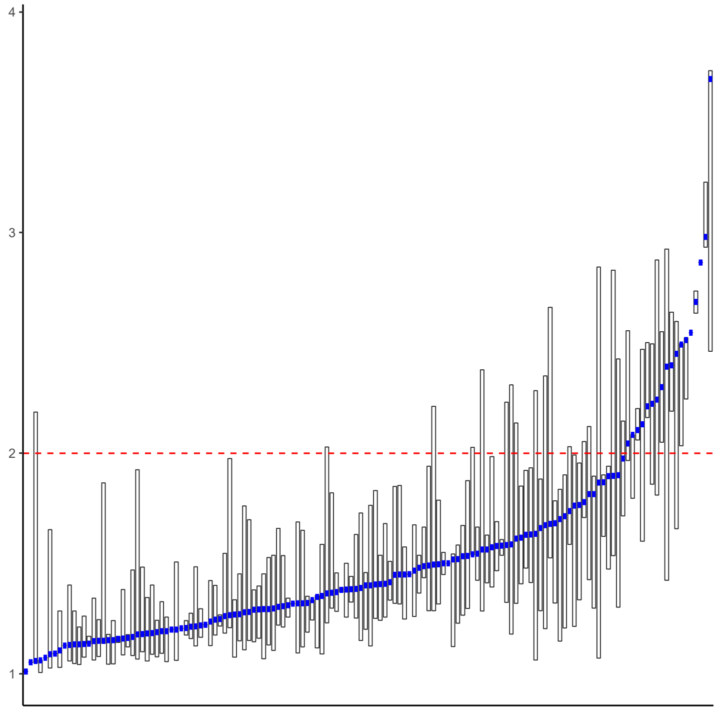
1
2
3
4

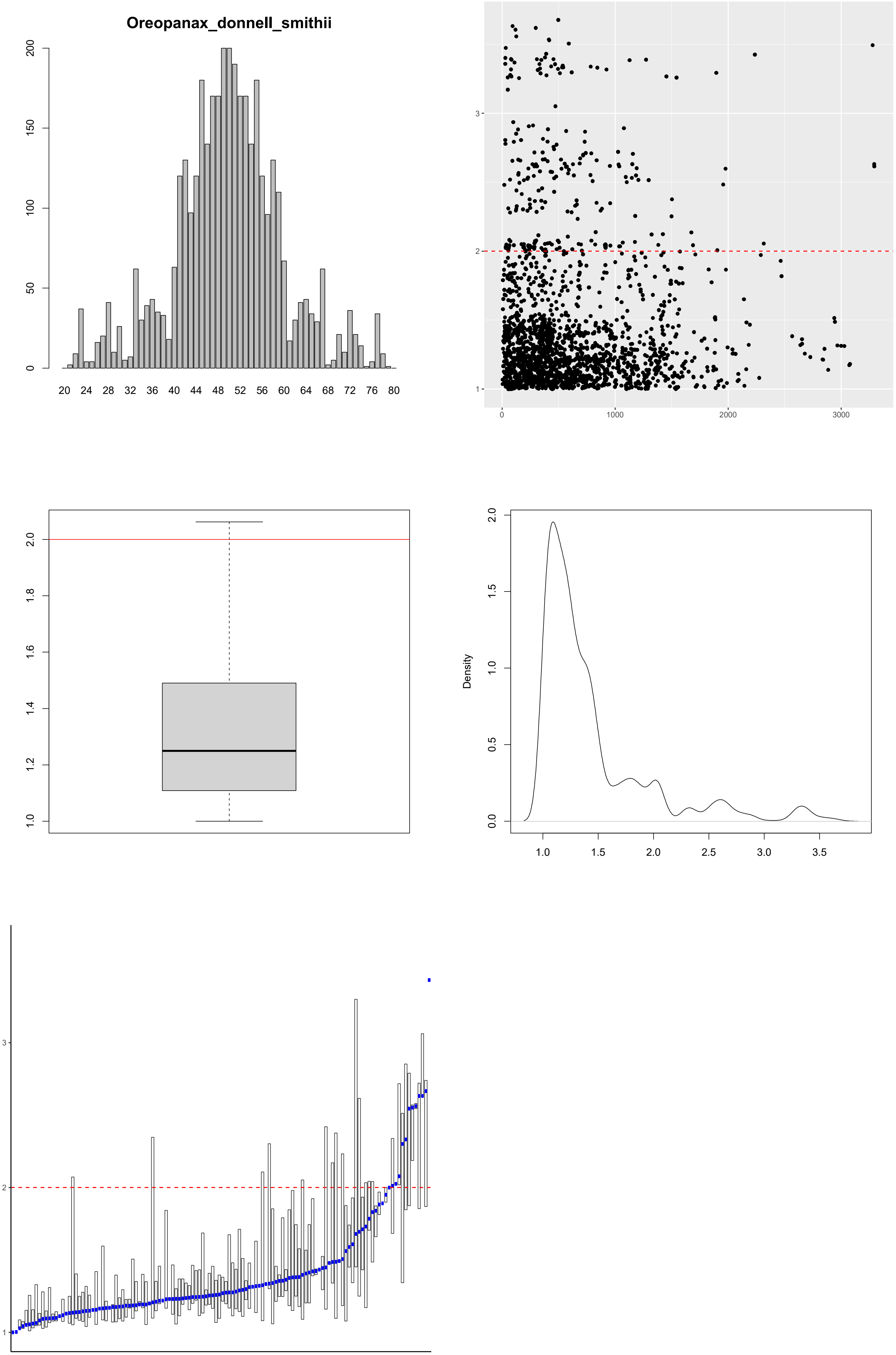
Oreopanax_donnell_smithii
Density

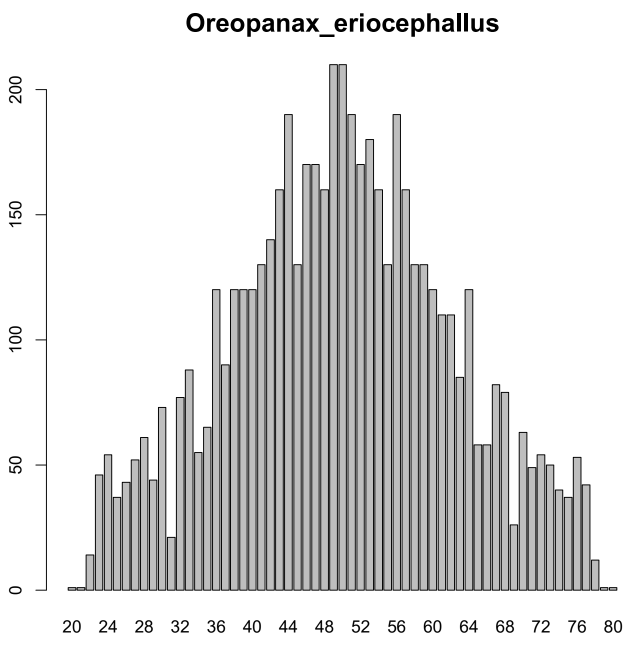
Oreopanax_eriocephallus
0
50
100
150
200
20 24 28 32 36 40 44 48 52 56 60 64 68 72 76 80

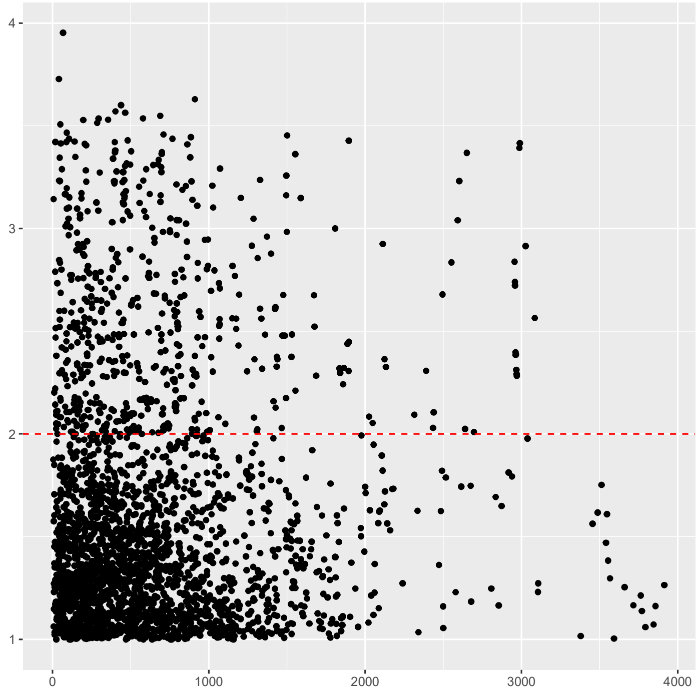
1
2
3
4
0 1000 2000 3000 4000

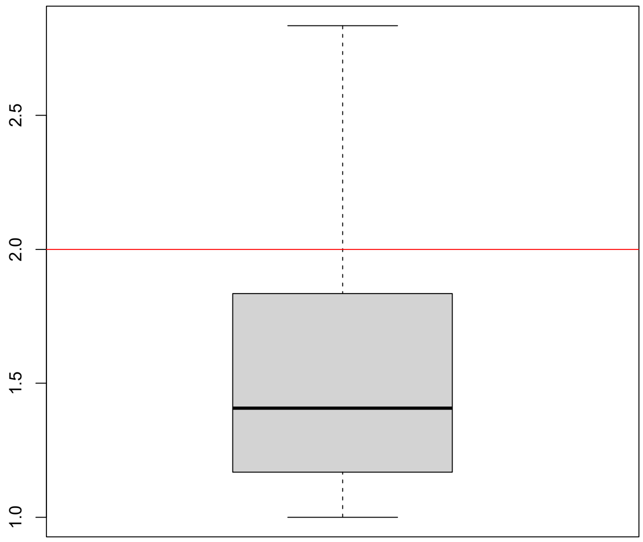
1.0
1.5
2.0
2.5

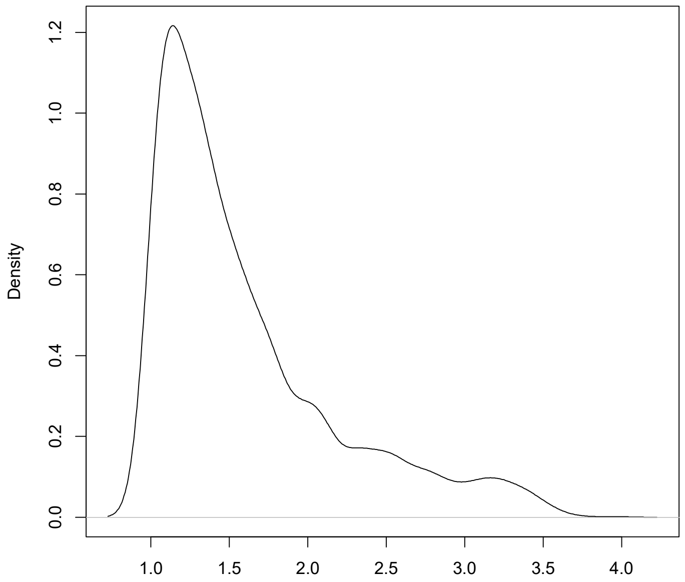
Density
0.0
0.2
0.4
0.6
0.8
1.0
1.2
1.0 1.5 2.0 2.5 3.0 3.5 4.0

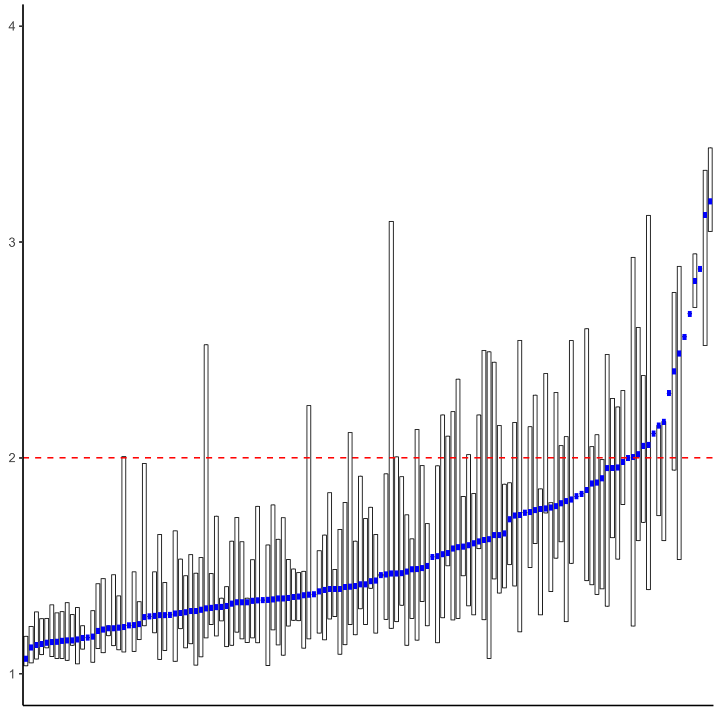
1
2
3
4

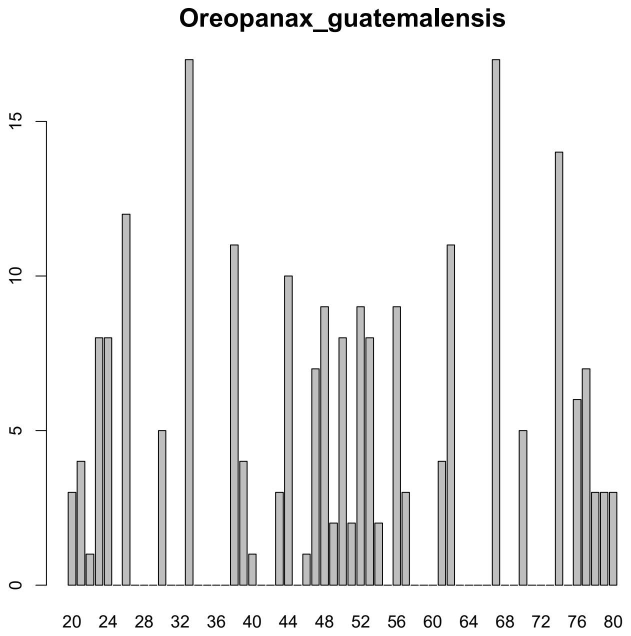
Oreopanax_guatemalensis

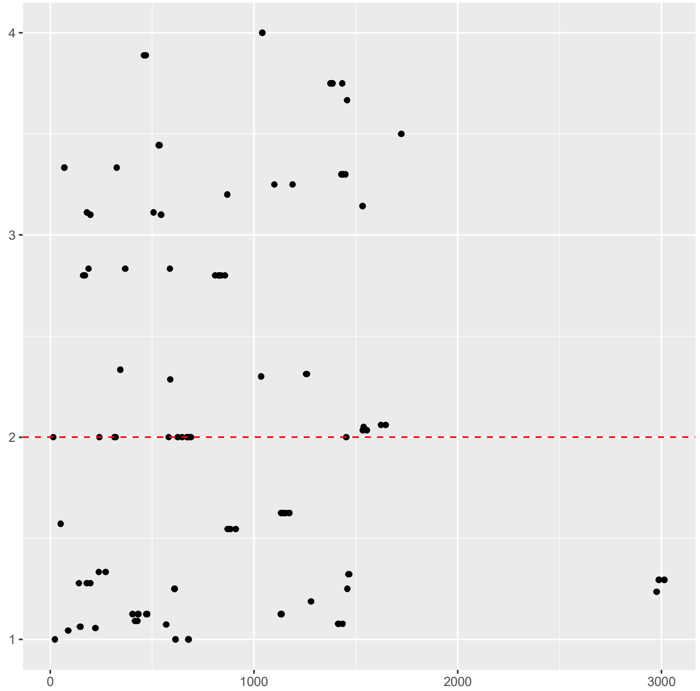

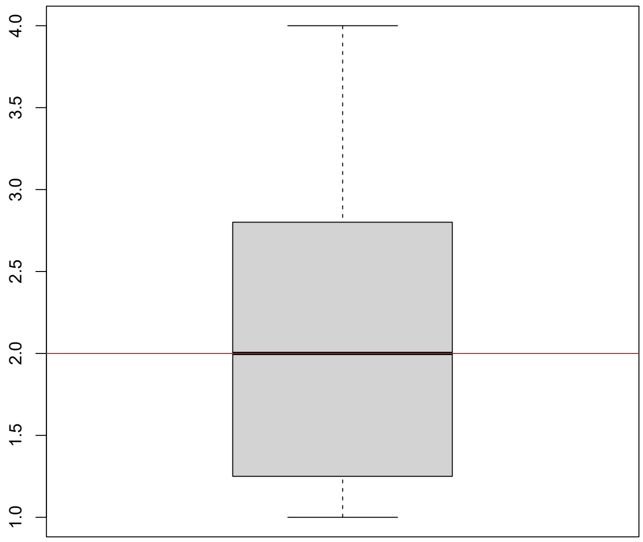

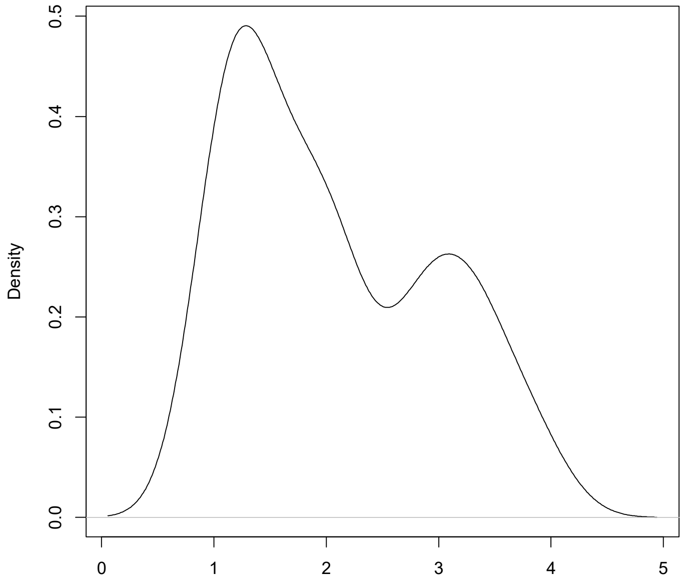
Density

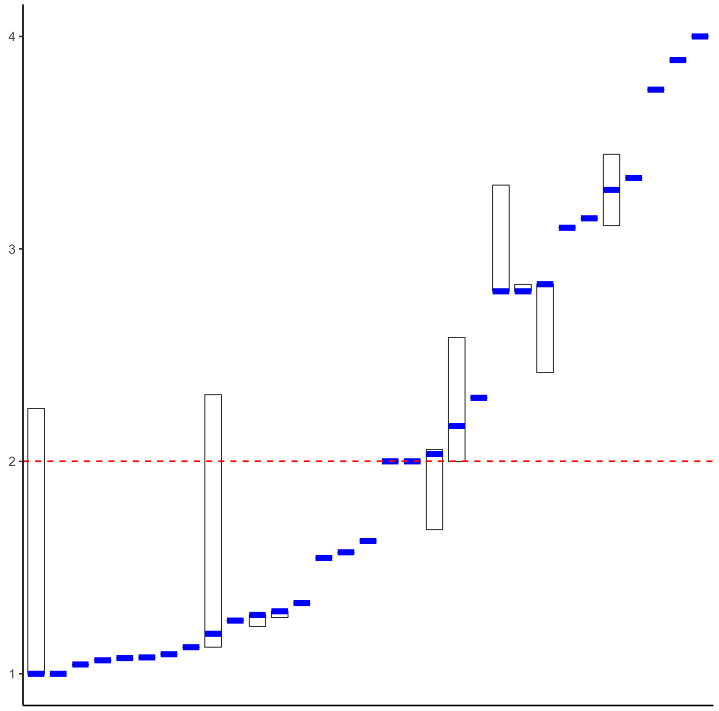

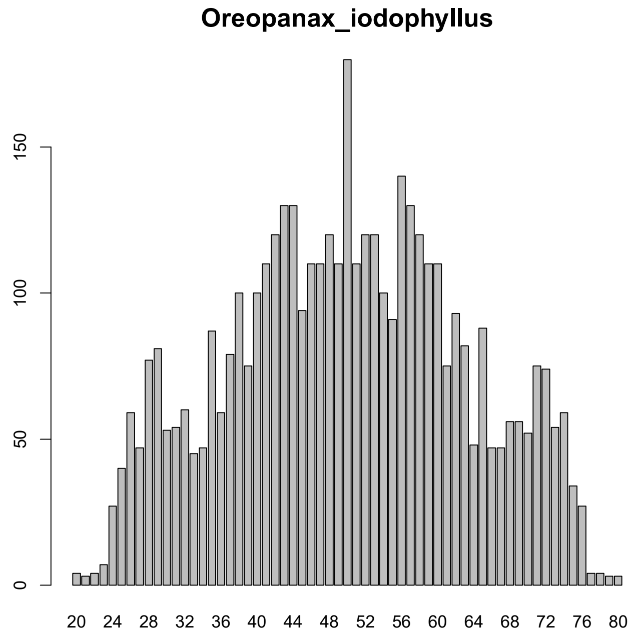
Oreopanax_iodophyllus
0
50
100
150
20 24 28 32 36 40 44 48 52 56 60 64 68 72 76 80

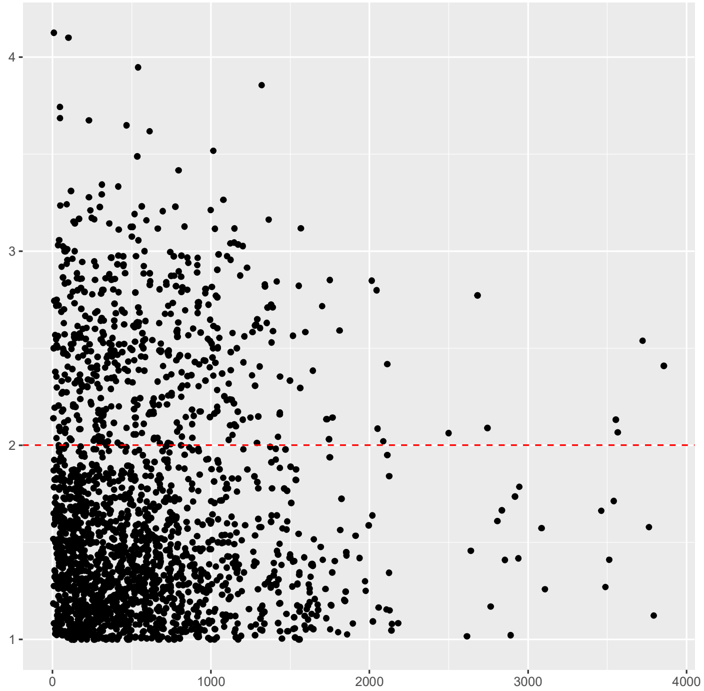
1
2
3
4
0 1000 2000 3000 4000

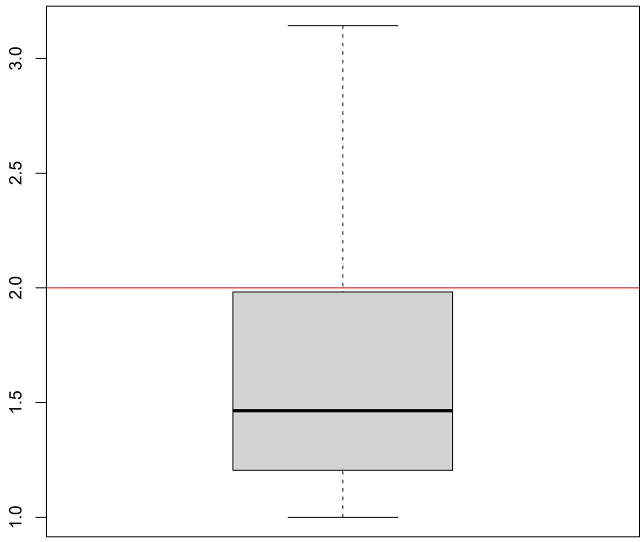
1.0
1.5
2.0
2.5
3.0

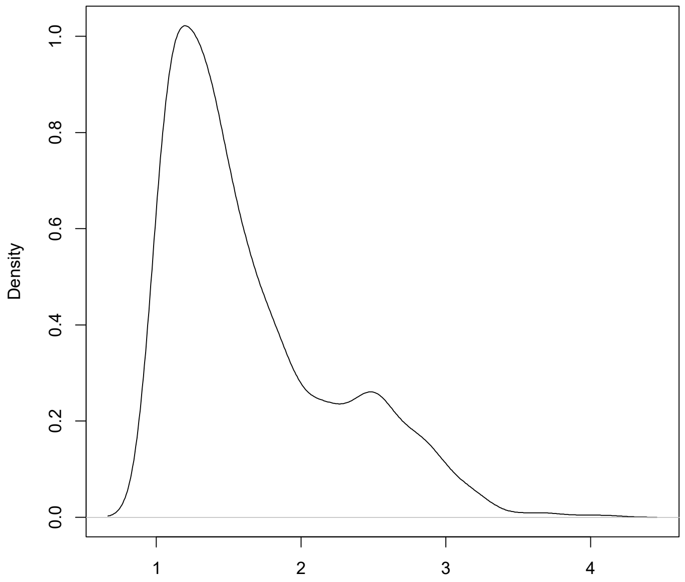
Density
0.0
0.2
0.4
0.6
0.8
1.0
1 2 3 4

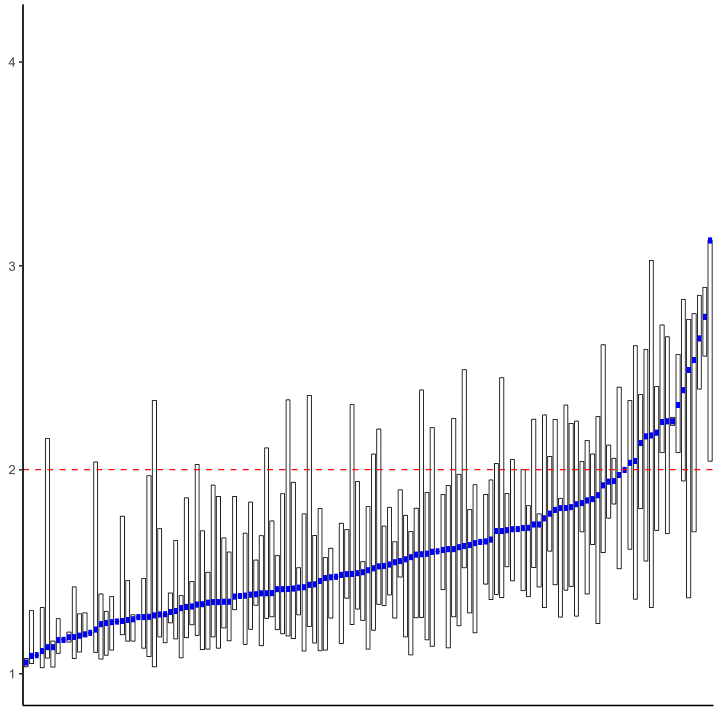
1
2
3
4

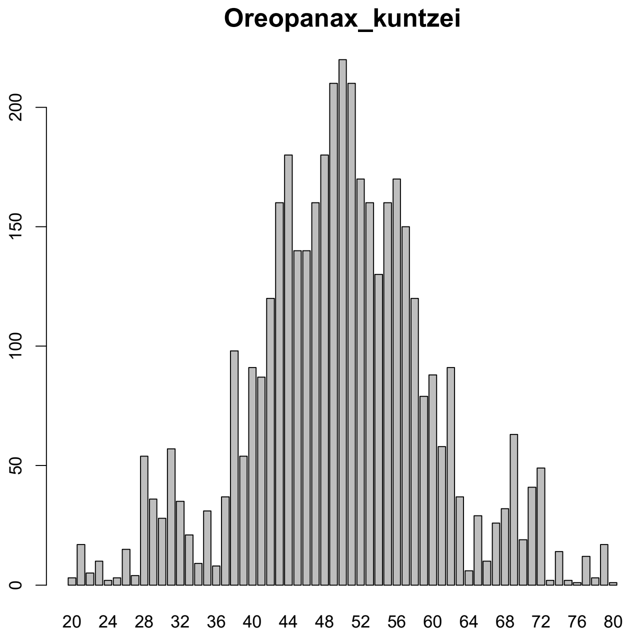
Oreopanax_kuntzei
0
50
100
150
200
20 24 28 32 36 40 44 48 52 56 60 64 68 72 76 80

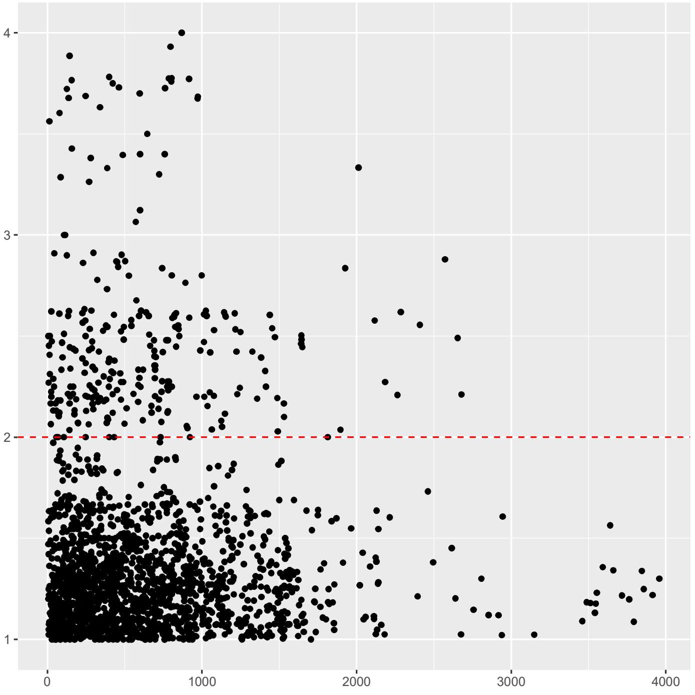
1
2
3
4
0 1000 2000 3000 4000

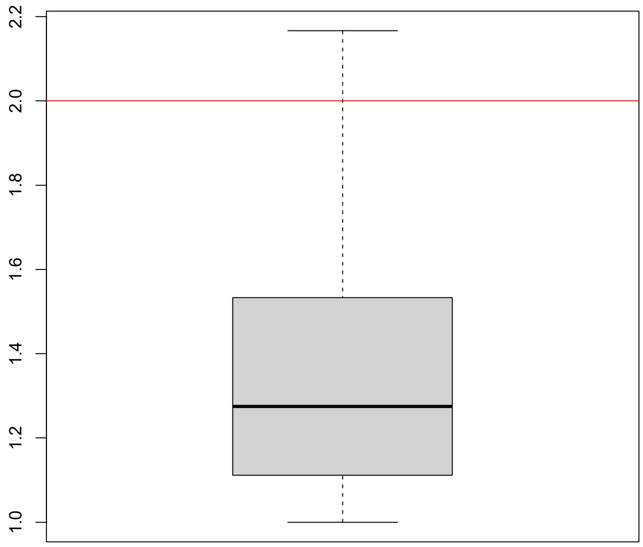
1.0
1.2
1.4
1.6
1.8
2.0
2.2

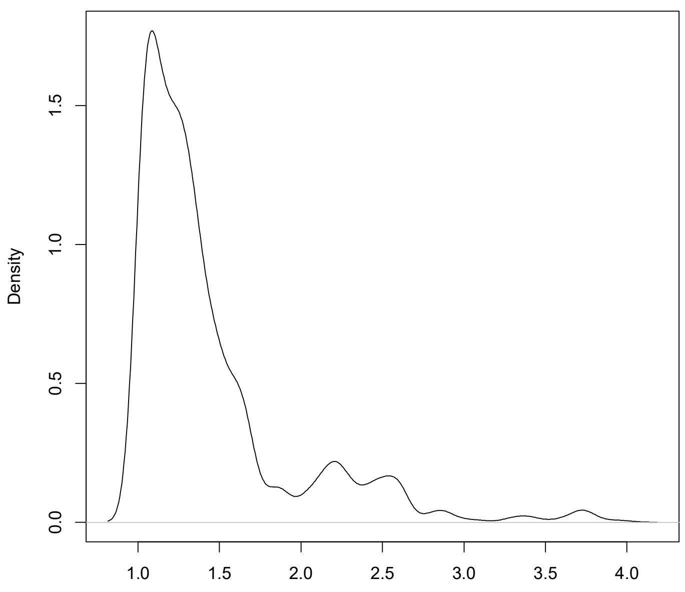
Density
0.0
0.5
1.0
1.5
1.0 1.5 2.0 2.5 3.0 3.5 4.0

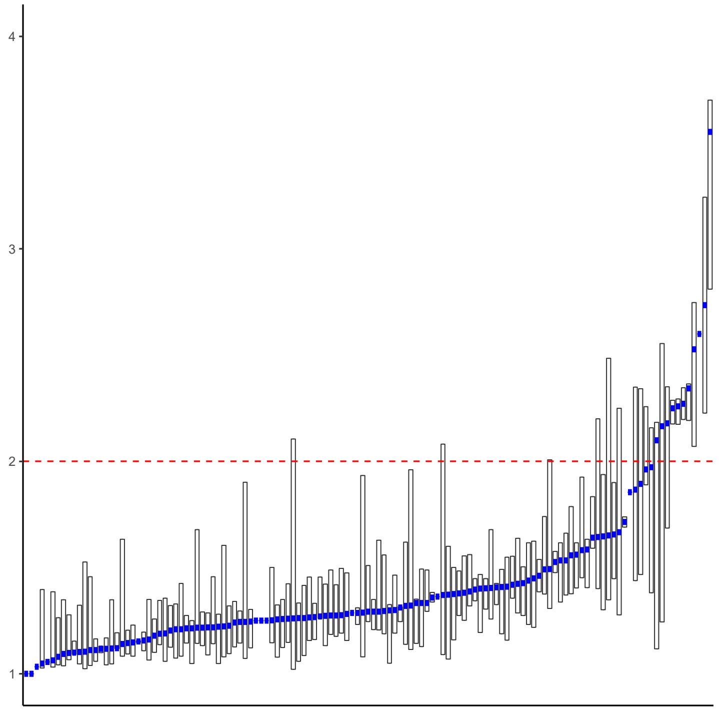
1
2
3
4

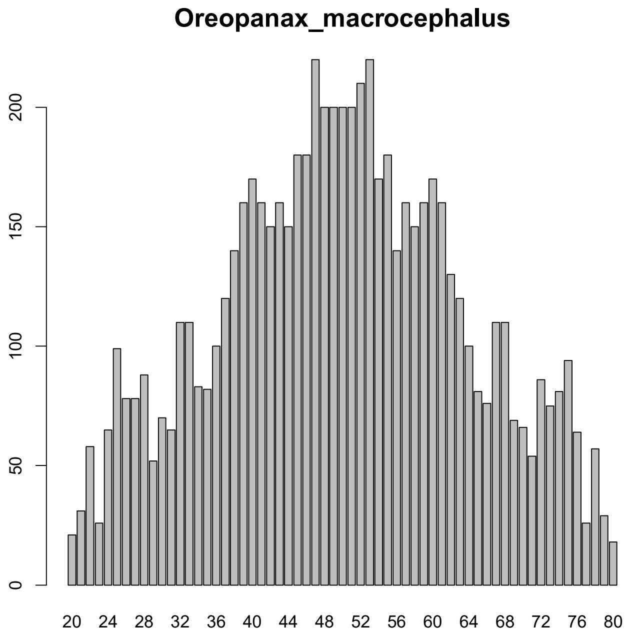
Oreopanax_macrocephalus
0
50
100
150
200
20 24 28 32 36 40 44 48 52 56 60 64 68 72 76 80

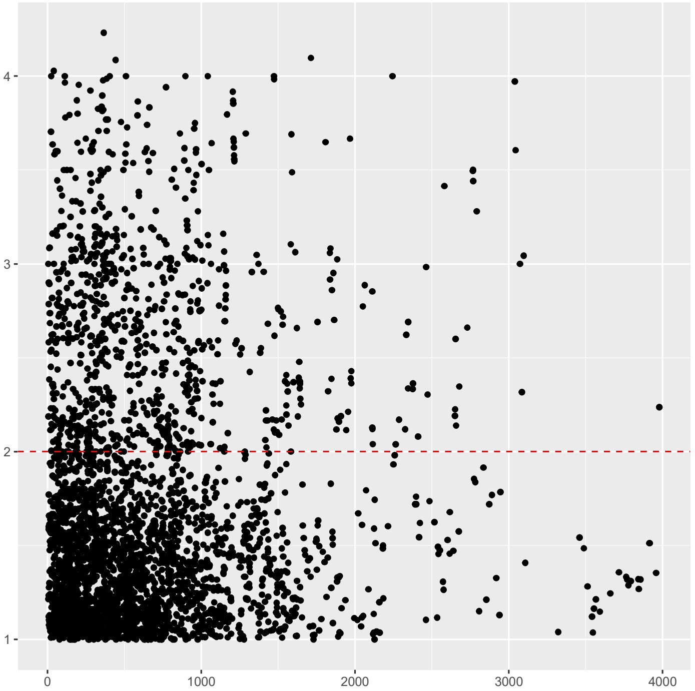
1
2
3
4
0 1000 2000 3000 4000

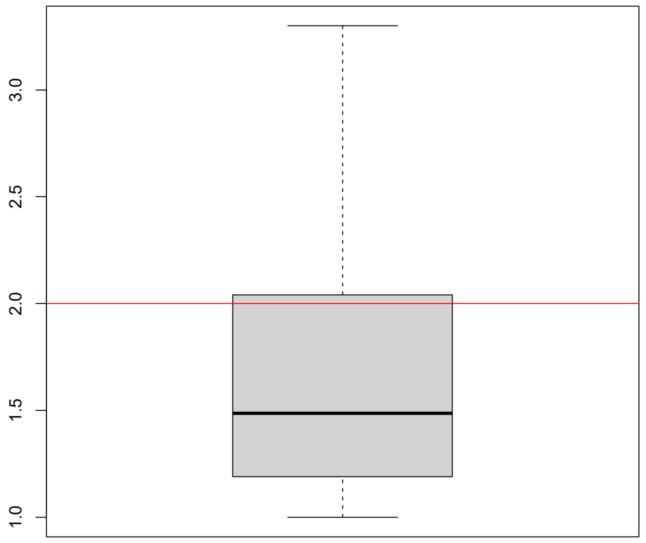
1.0
1.5
2.0
2.5
3.0

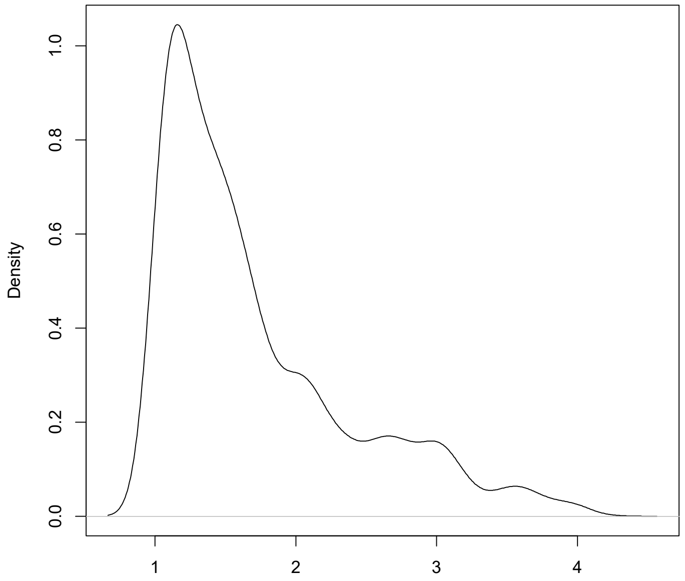
Density
0.0
0.2
0.4
0.6
0.8
1.0
1 2 3 4

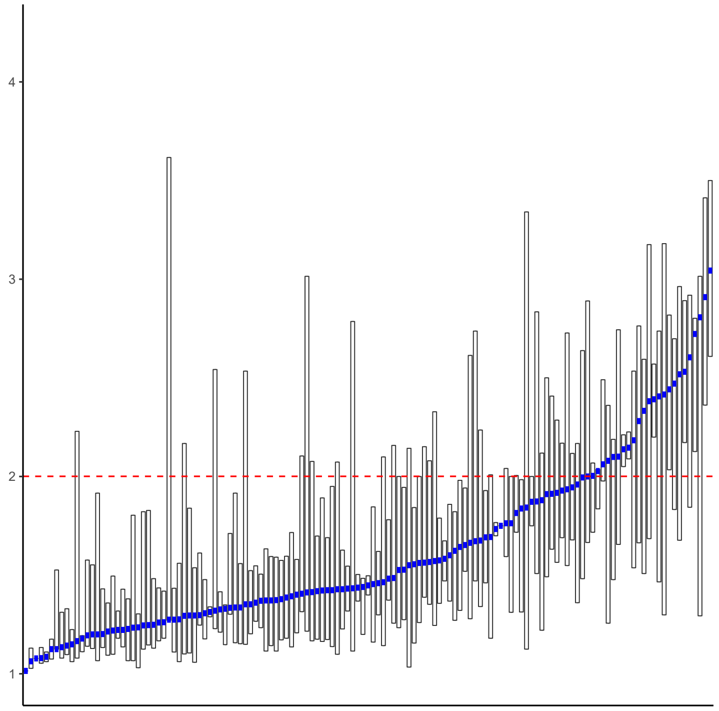
1
2
3
4

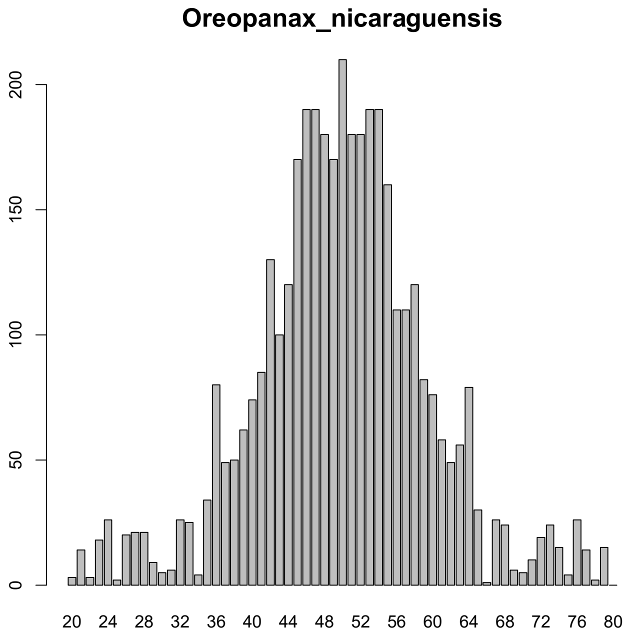
Oreopanax_nicaraguensis

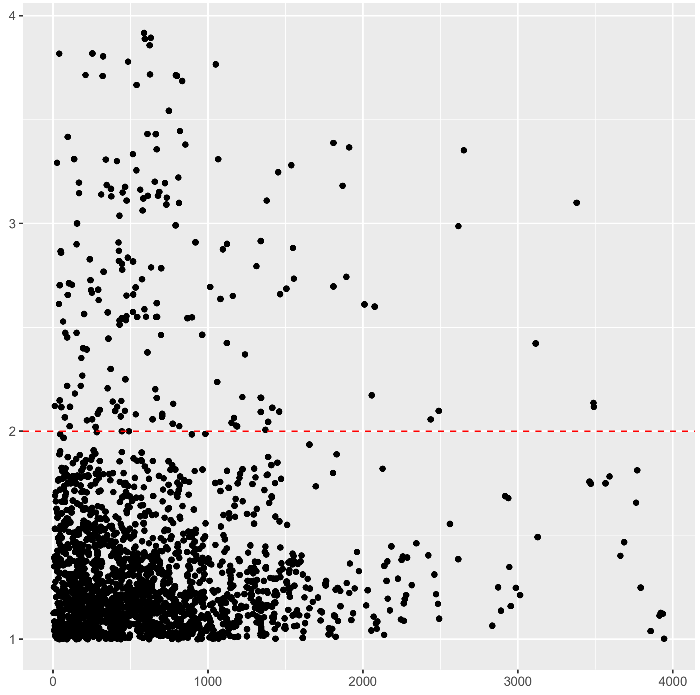

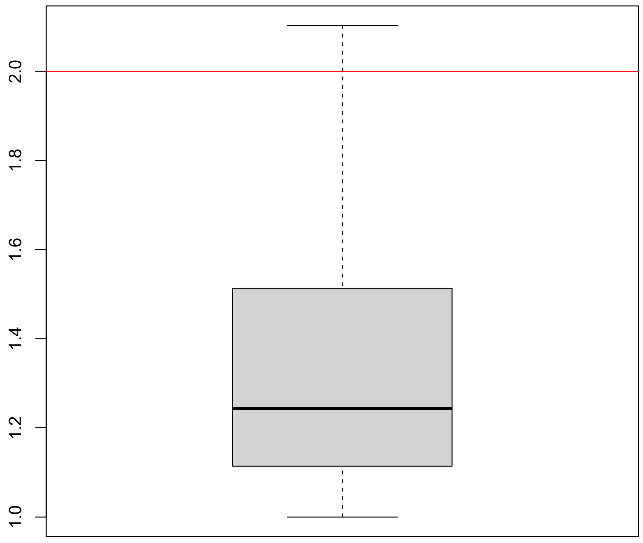

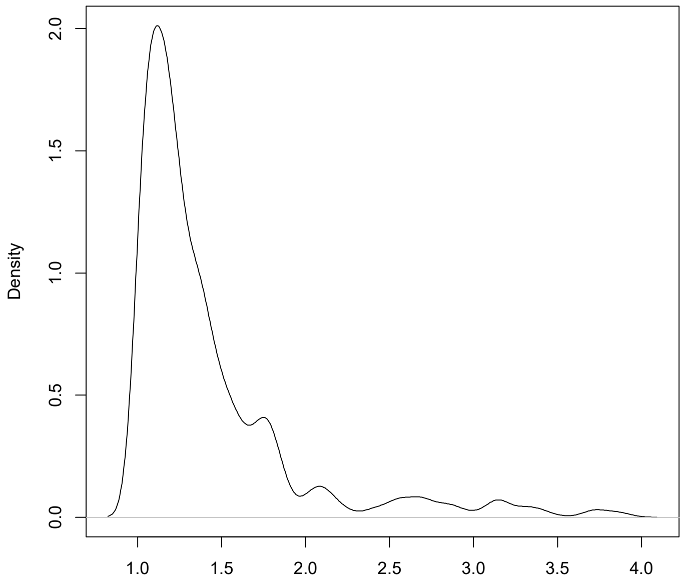
Density

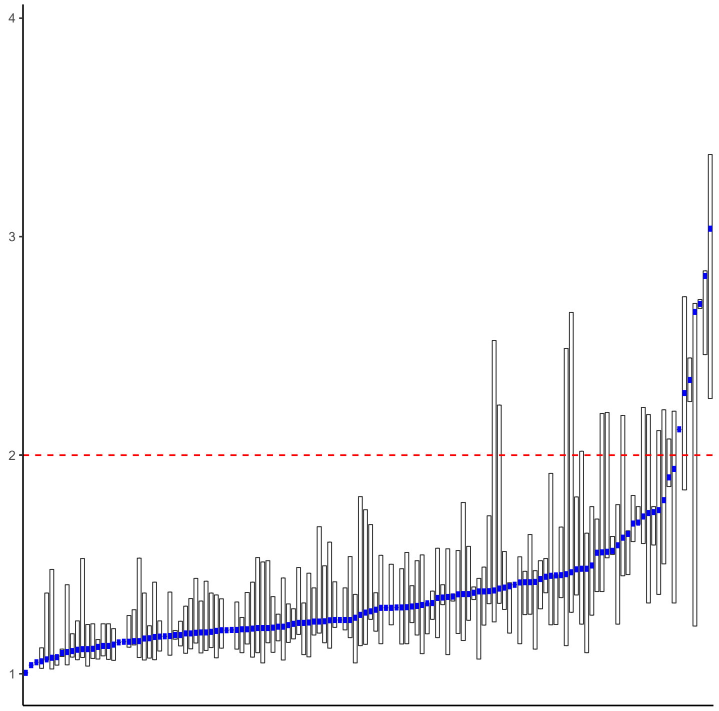

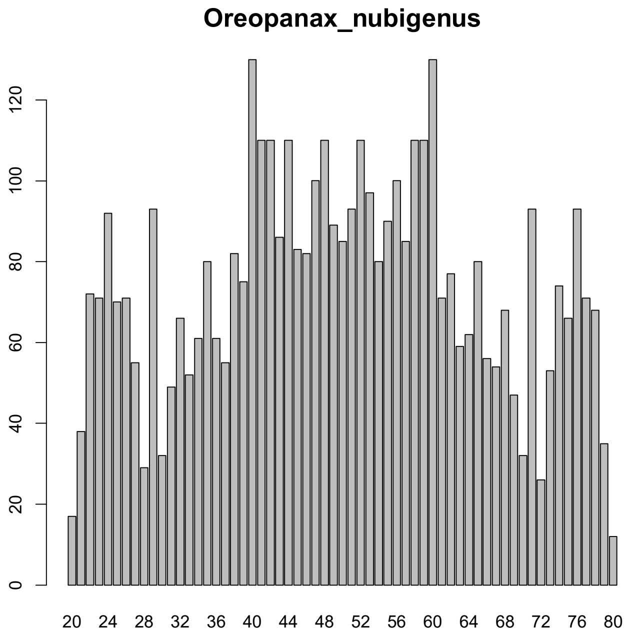
Oreopanax_nubigenus
0
20
40
60
80
100
120
20 24 28 32 36 40 44 48 52 56 60 64 68 72 76 80

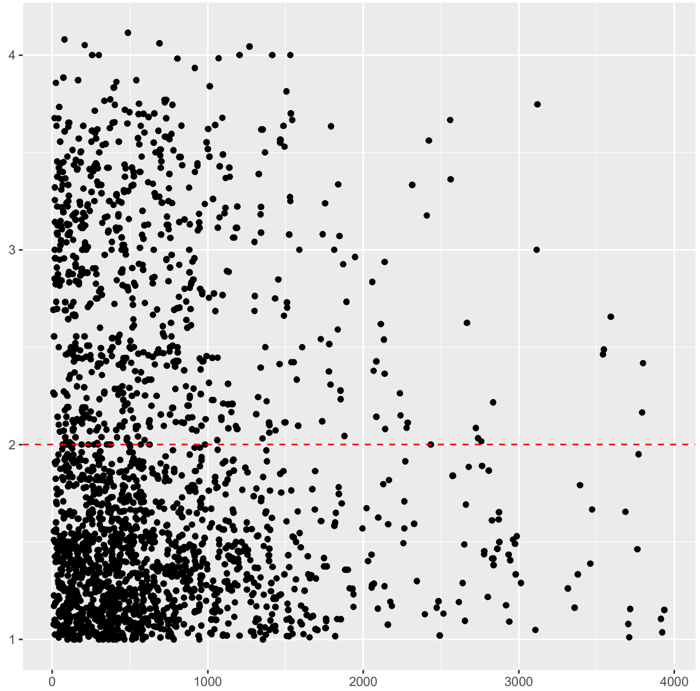
1
2
3
4
0 1000 2000 3000 4000

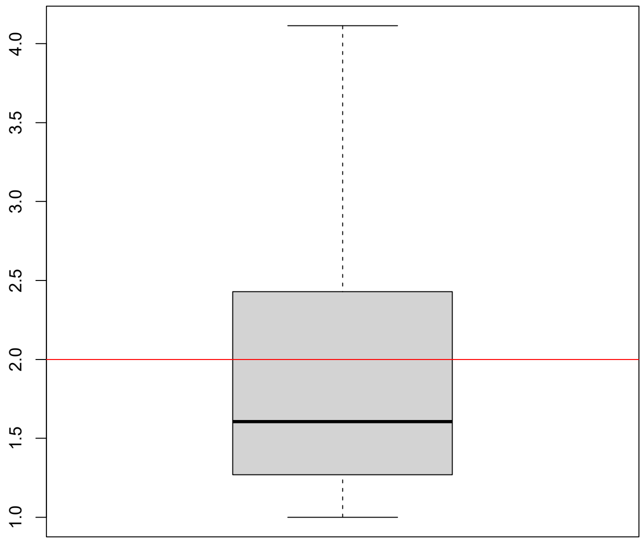
1.0
1.5
2.0
2.5
3.0
3.5
4.0

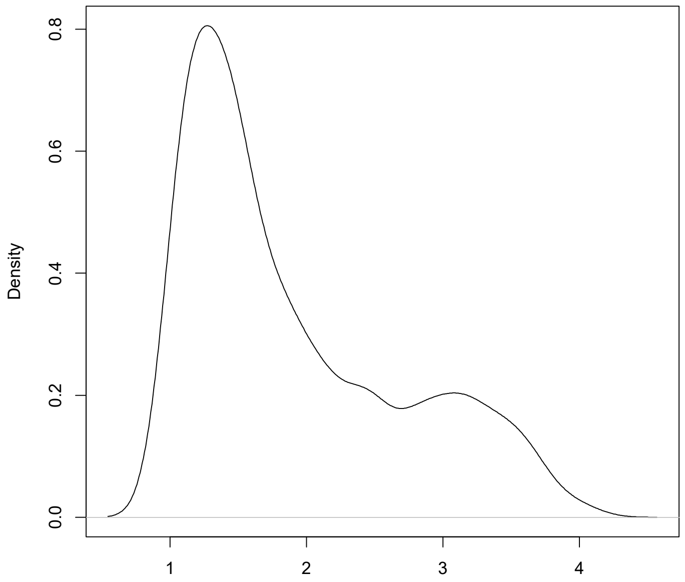
Density
0.0
0.2
0.4
0.6
0.8
1 2 3 4

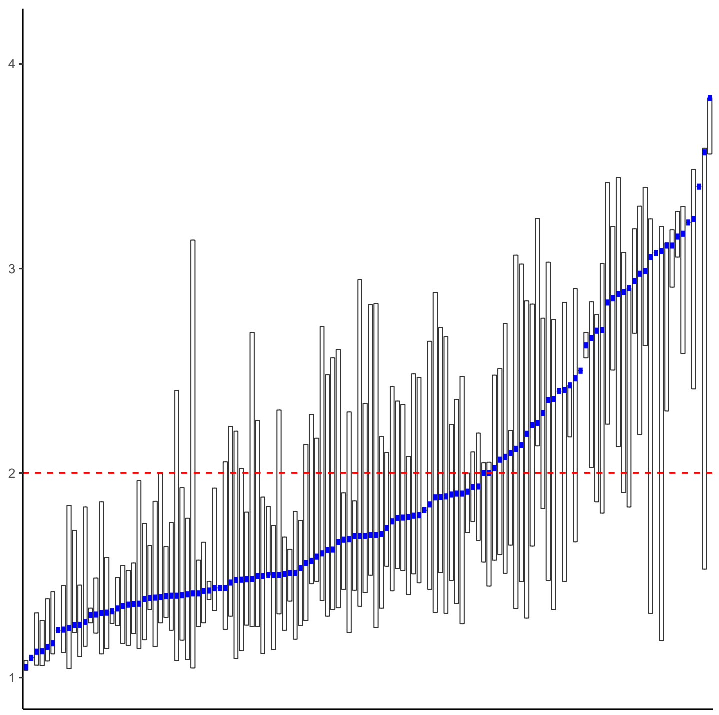
1
2
3
4

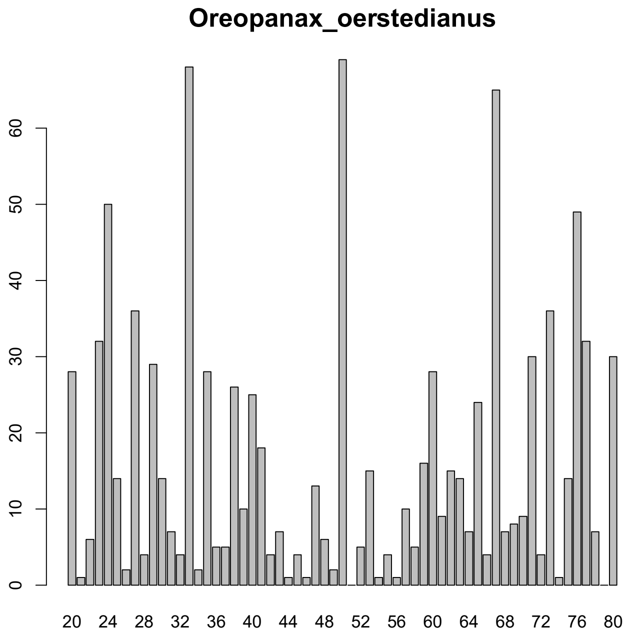
Oreopanax_oerstedianus

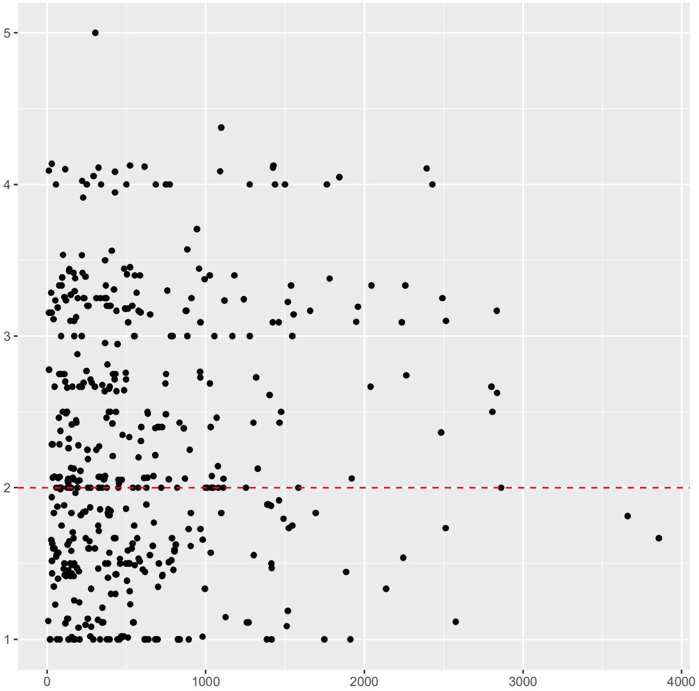

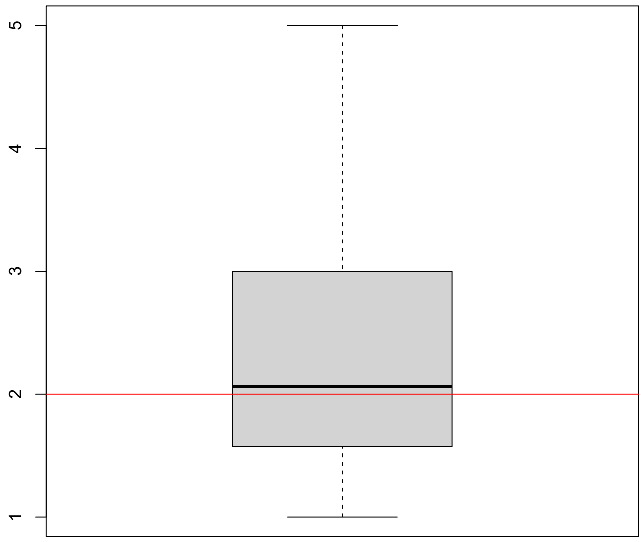

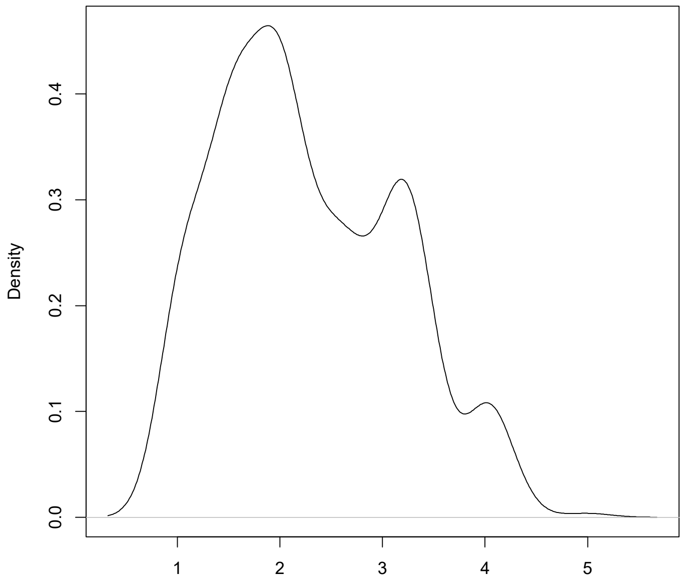
Density

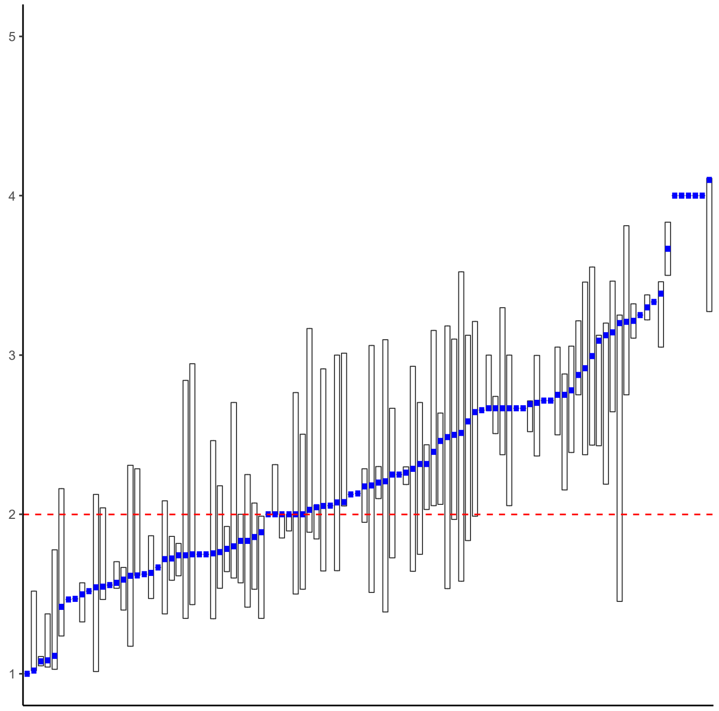

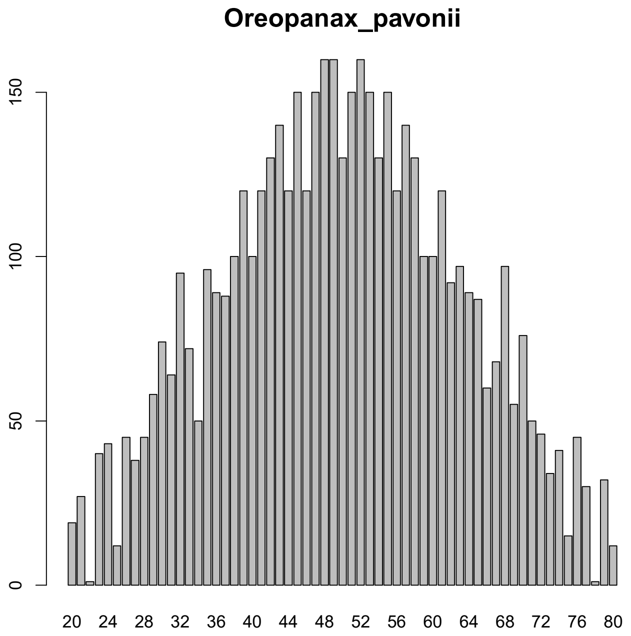
Oreopanax_pavonii

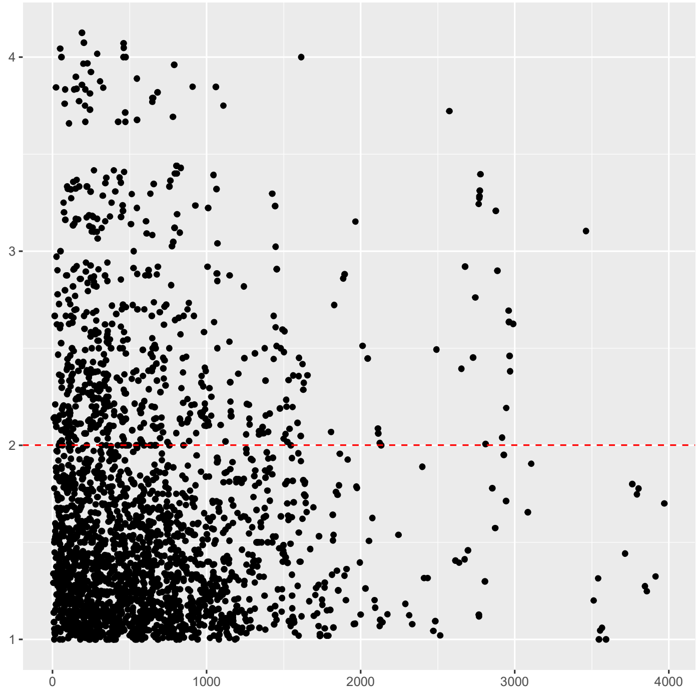

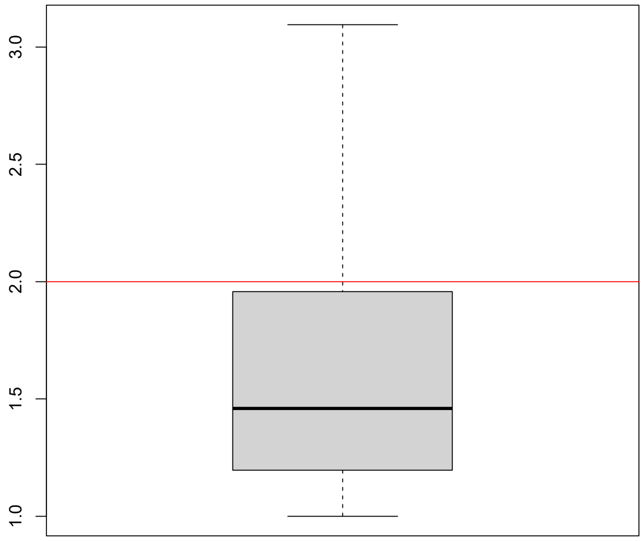

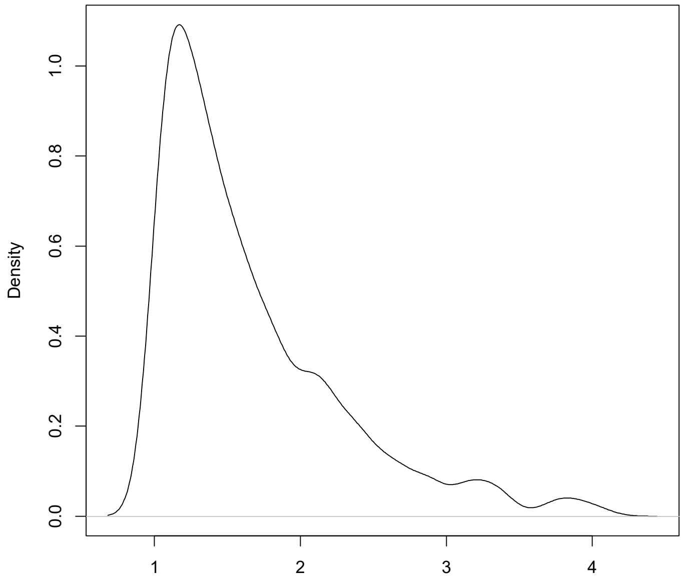
Density

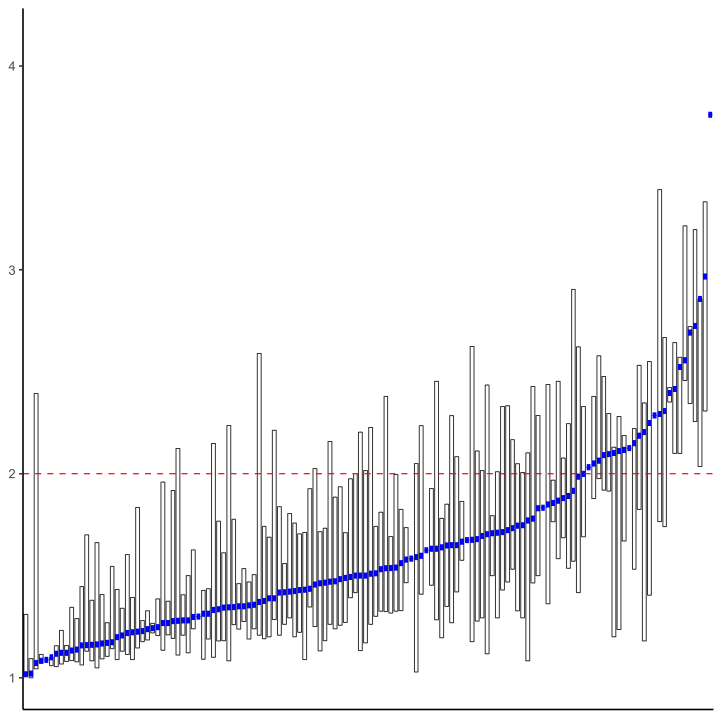

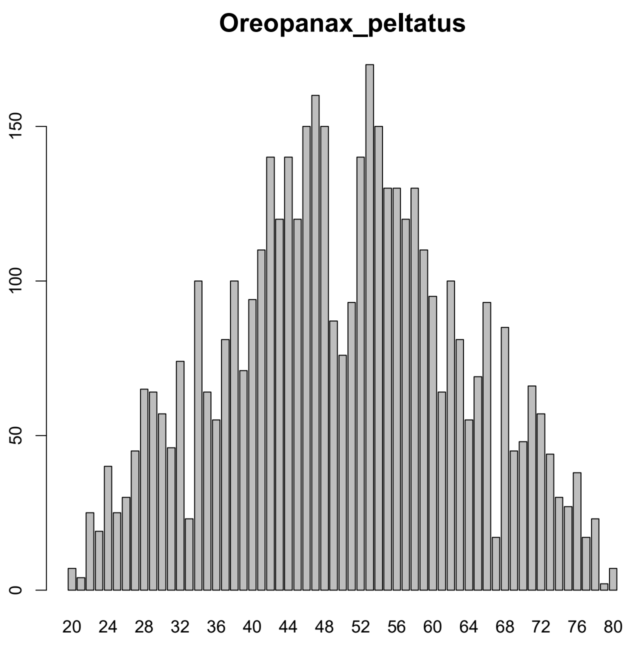
Oreopanax_peltatus

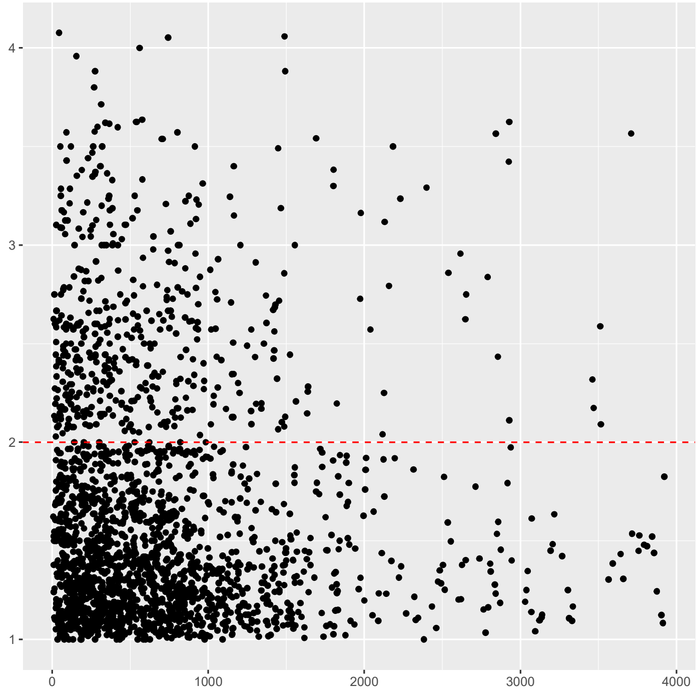

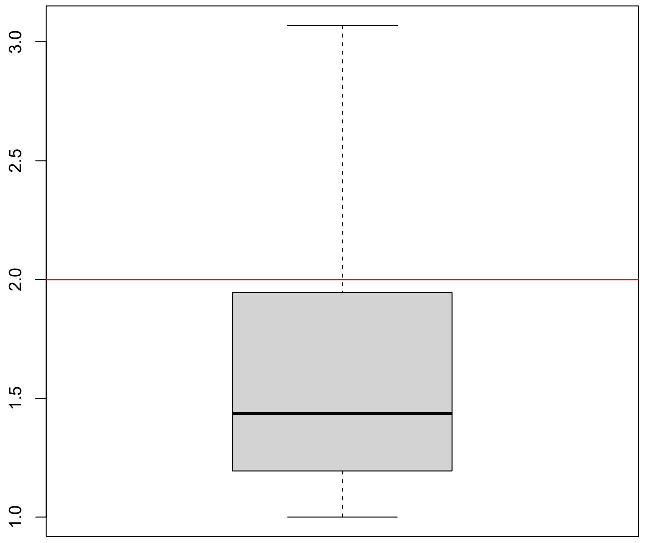

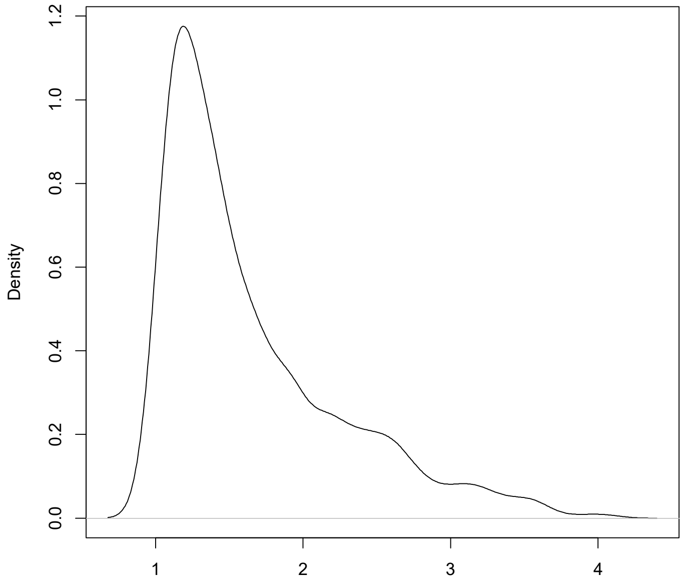
Density

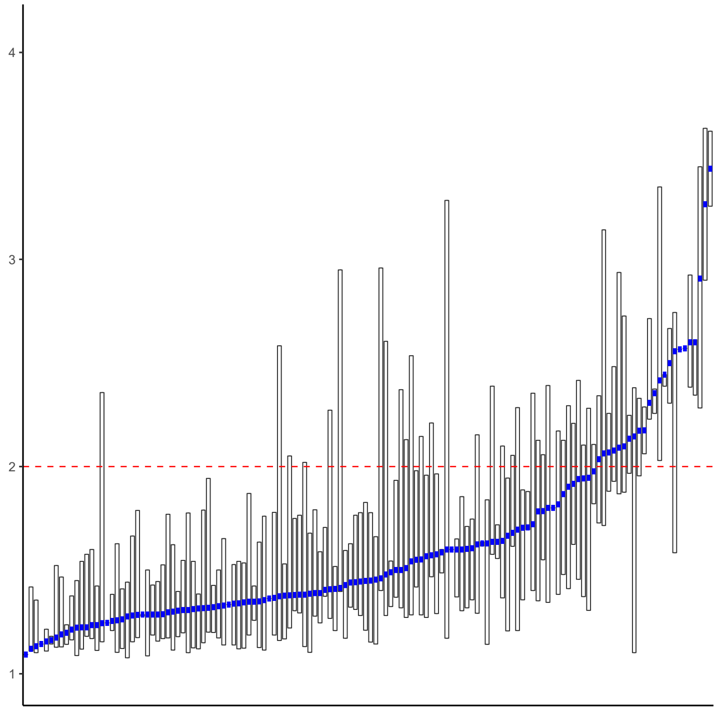

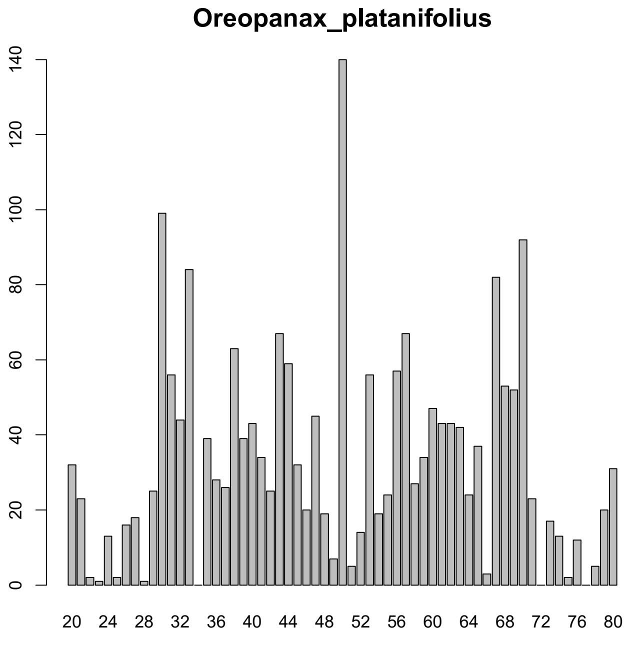
Oreopanax_platanifolius
0
20
40
60
80
100
120
140
20 24 28 32 36 40 44 48 52 56 60 64 68 72 76 80

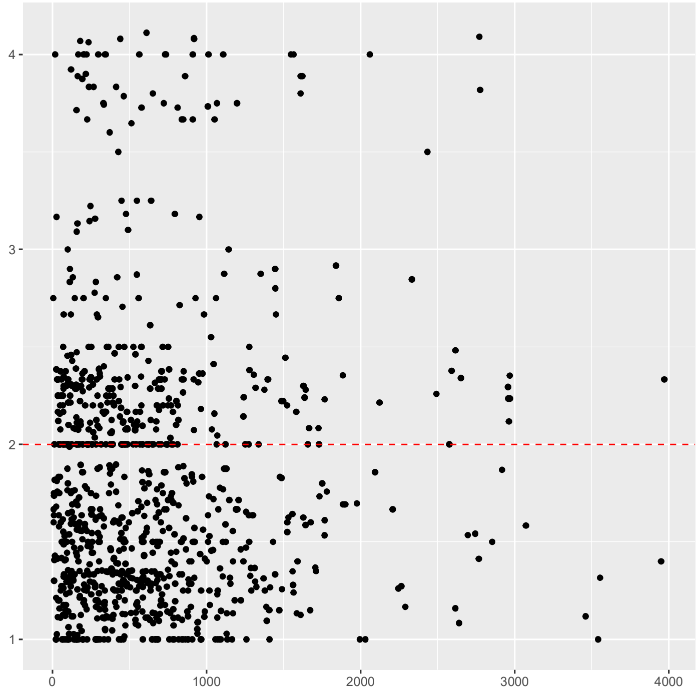
1
2
3
4
0 1000 2000 3000 4000

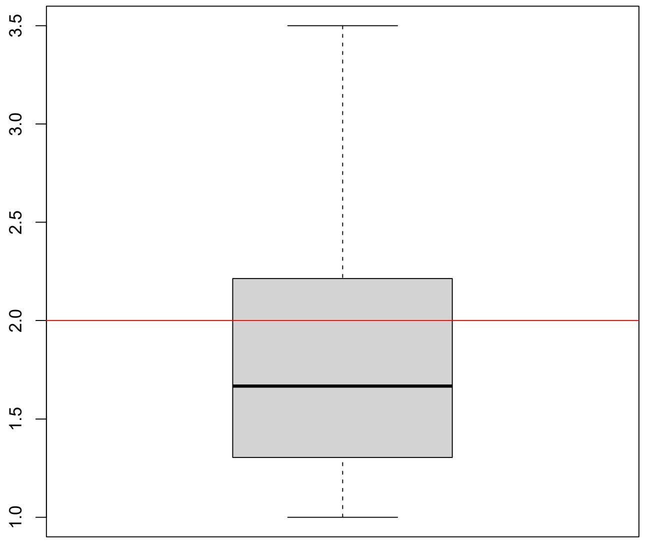
1.0
1.5
2.0
2.5
3.0
3.5

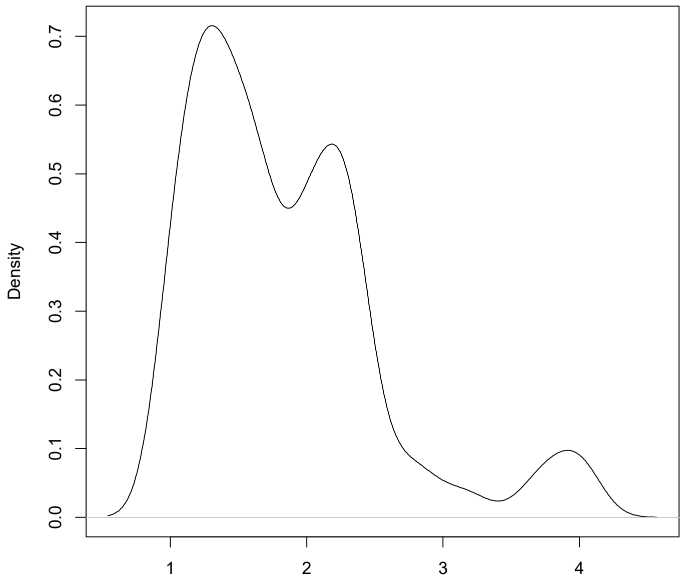
Density
0.0
0.1
0.2
0.3
0.4
0.5
0.6
0.7
1 2 3 4

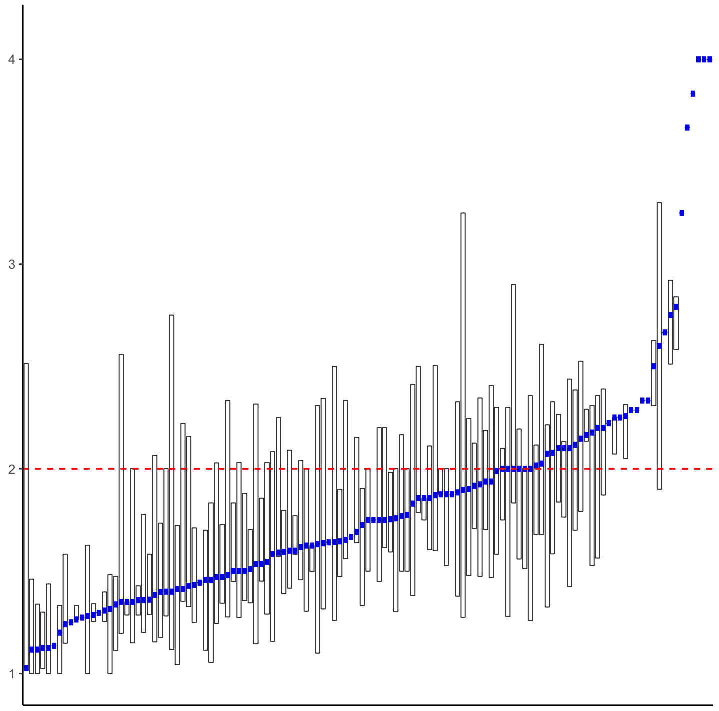
1
2
3
4

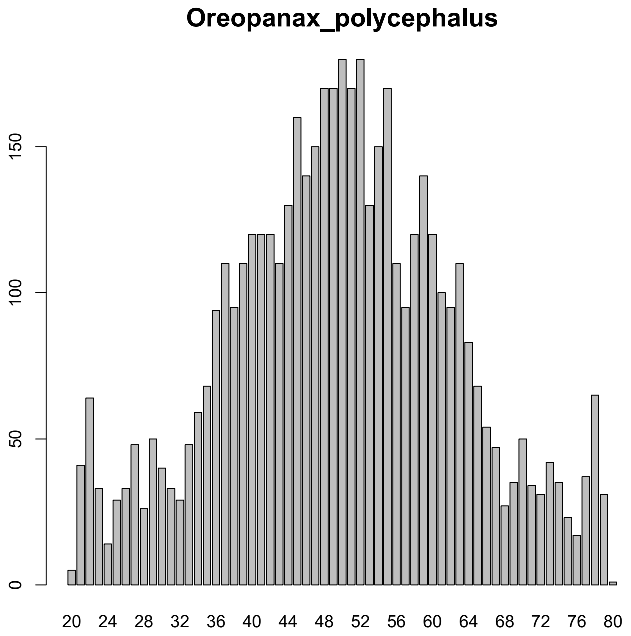
Oreopanax_polycephalus
0
50
100
150
20 24 28 32 36 40 44 48 52 56 60 64 68 72 76 80

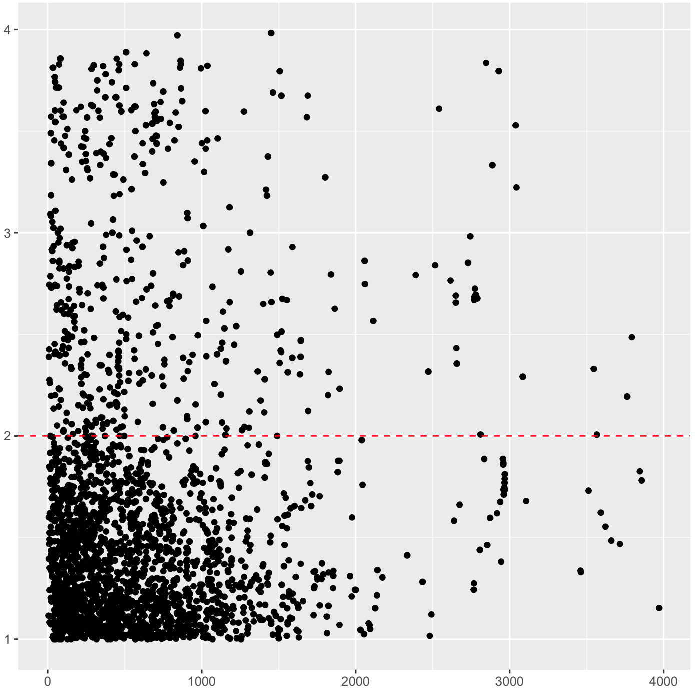
1
2
3
4
0 1000 2000 3000 4000

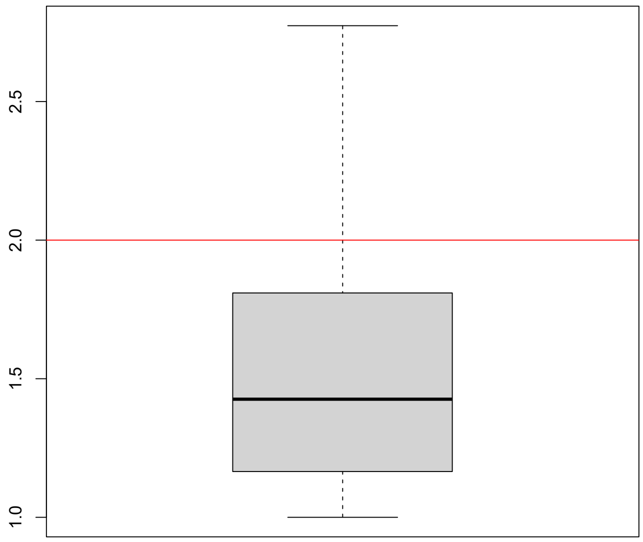
1.0
1.5
2.0
2.5

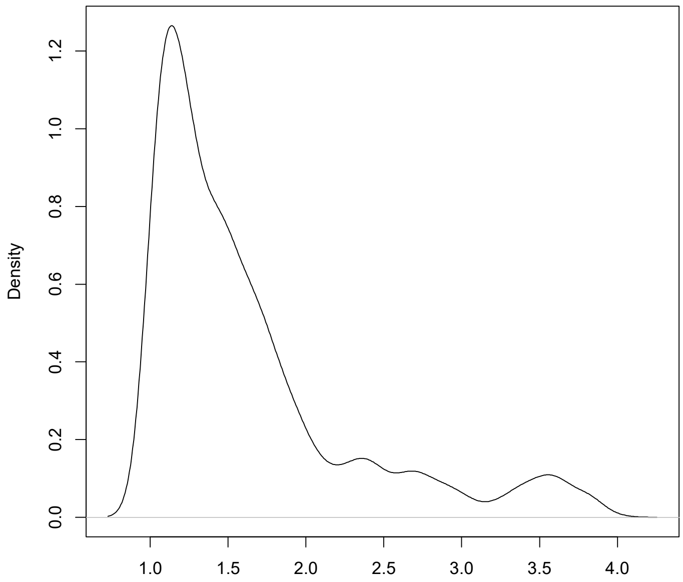
Density
0.0
0.2
0.4
0.6
0.8
1.0
1.2
1.0 1.5 2.0 2.5 3.0 3.5 4.0

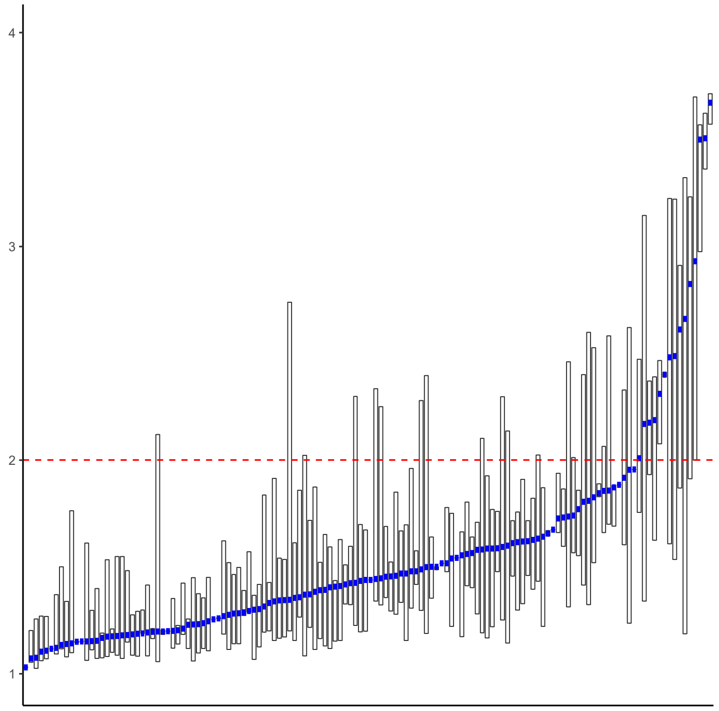
1
2
3
4

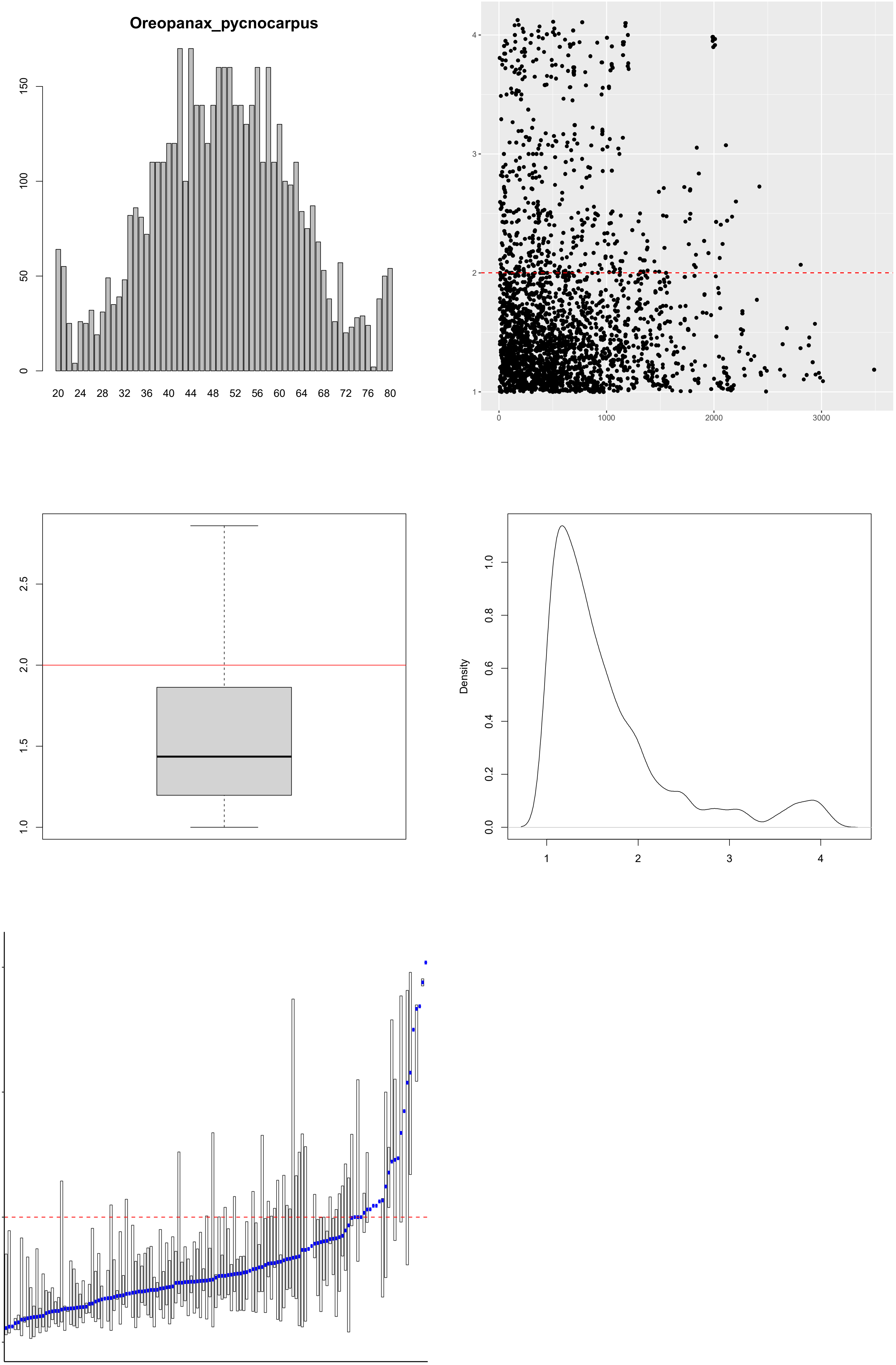
Oreopanax_pycnocarpus
Density

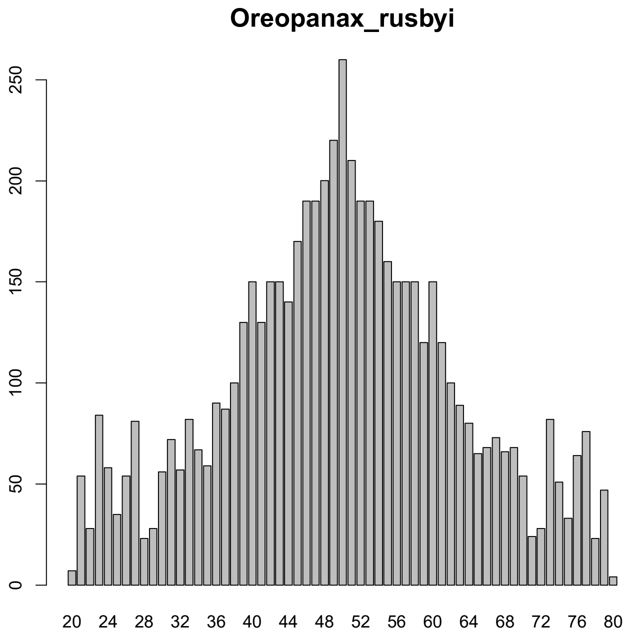
Oreopanax_rusbyi

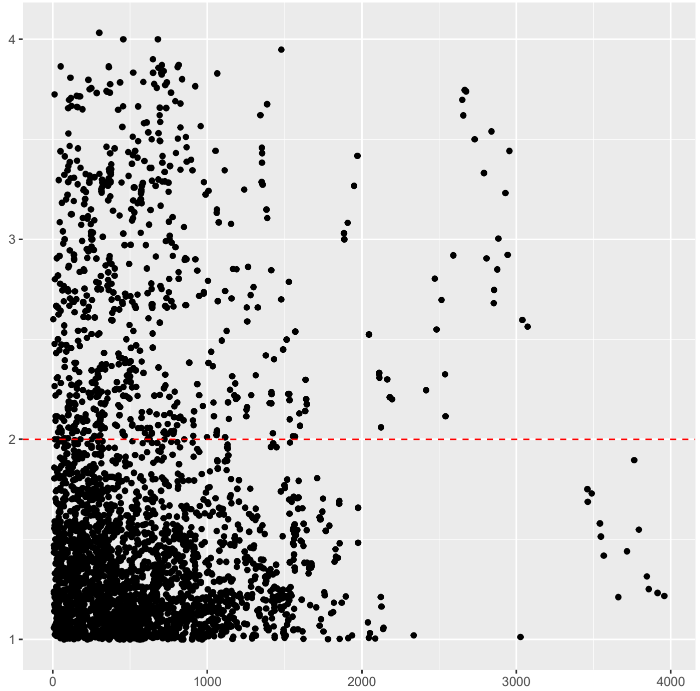

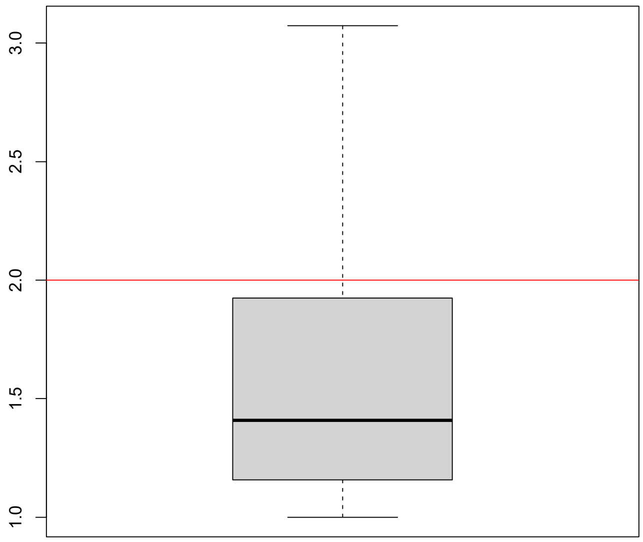

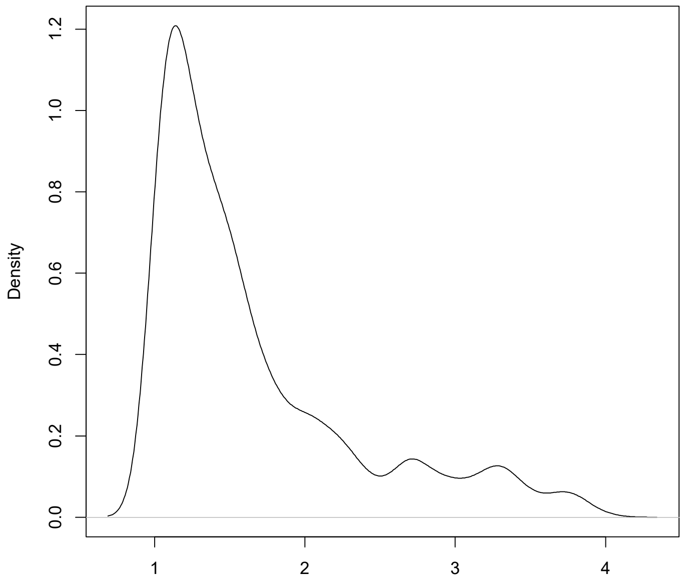
Density

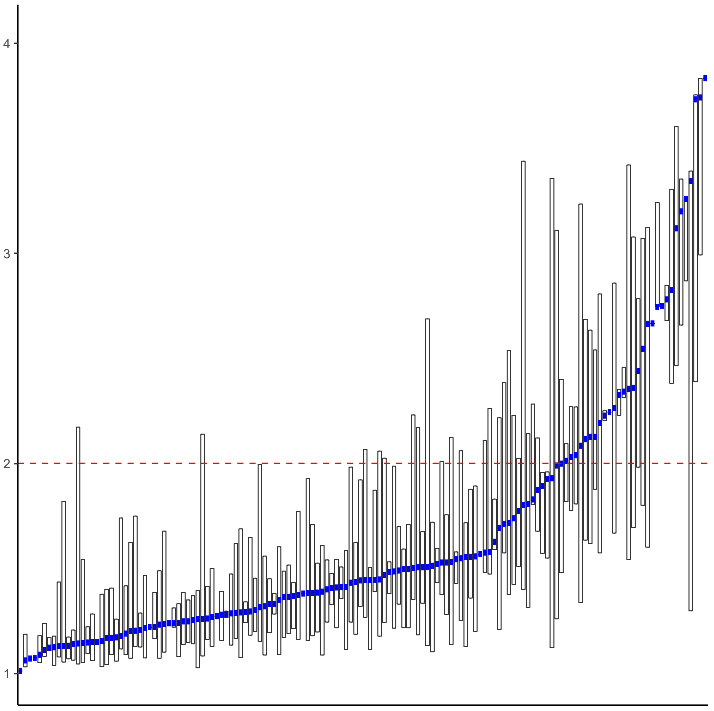

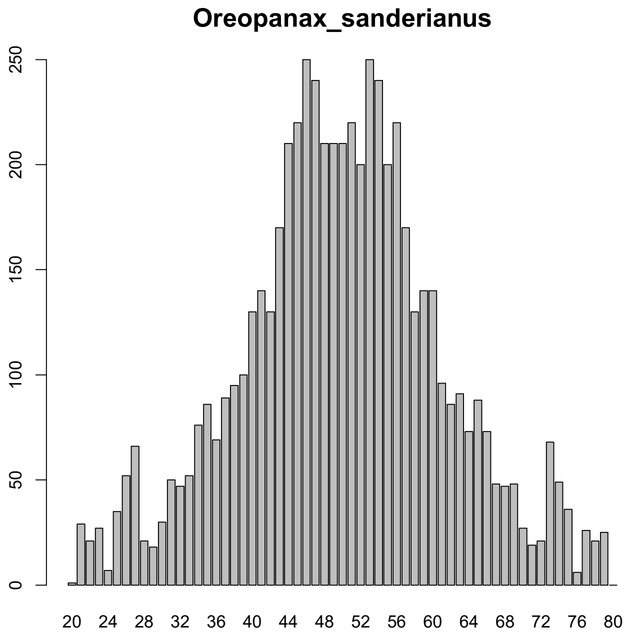
Oreopanax_sanderianus

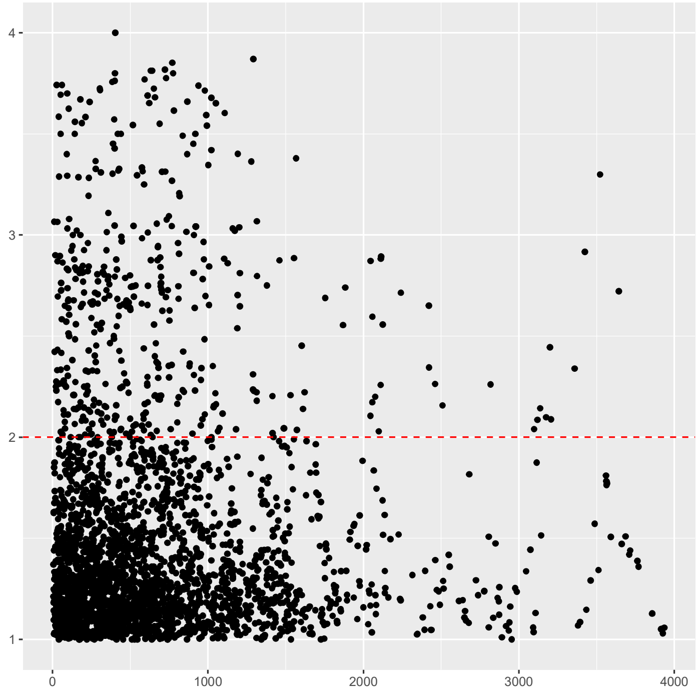

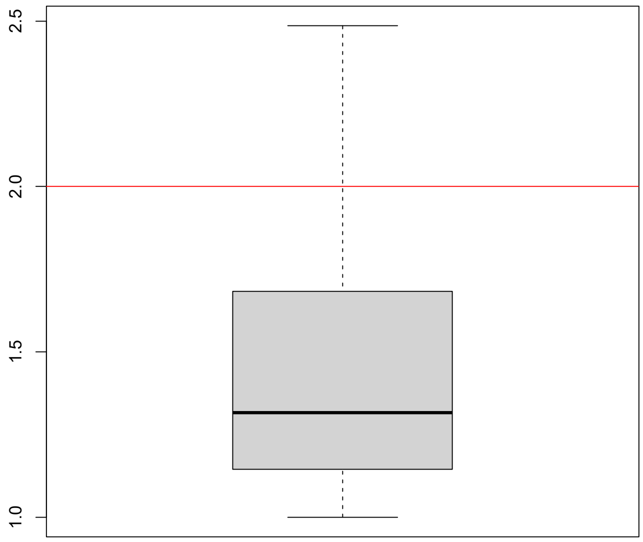

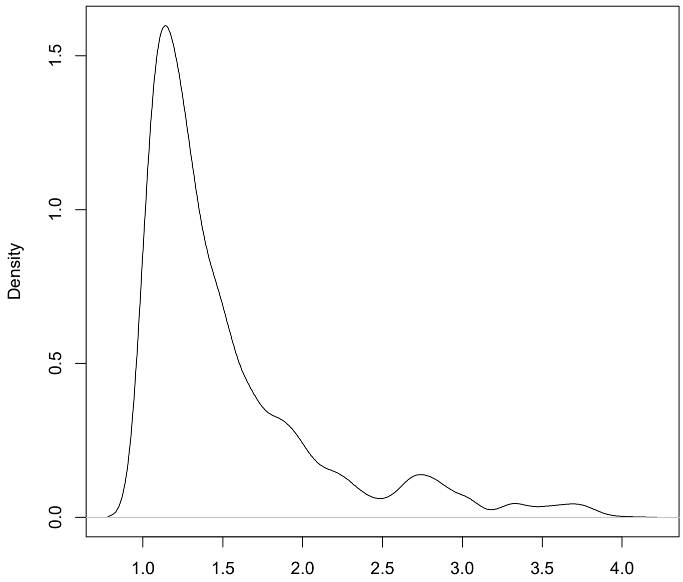
Density

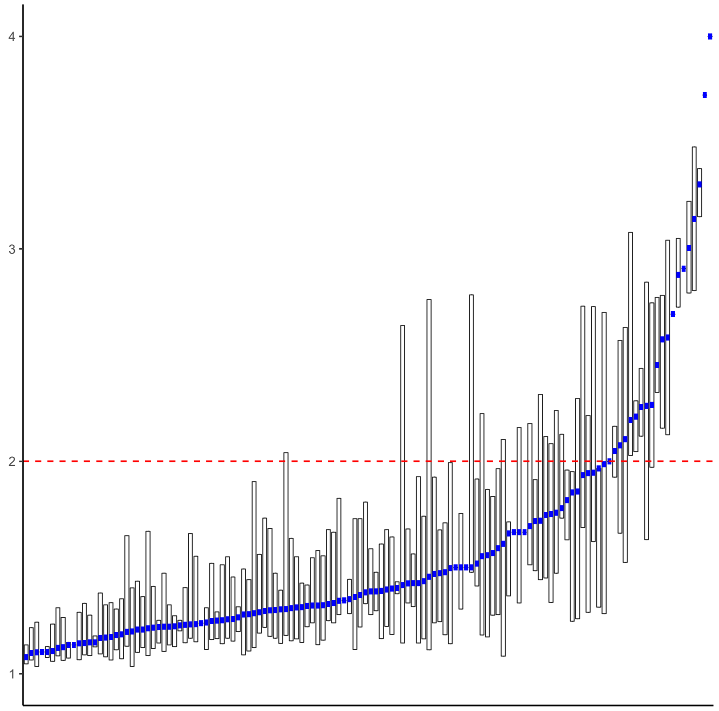

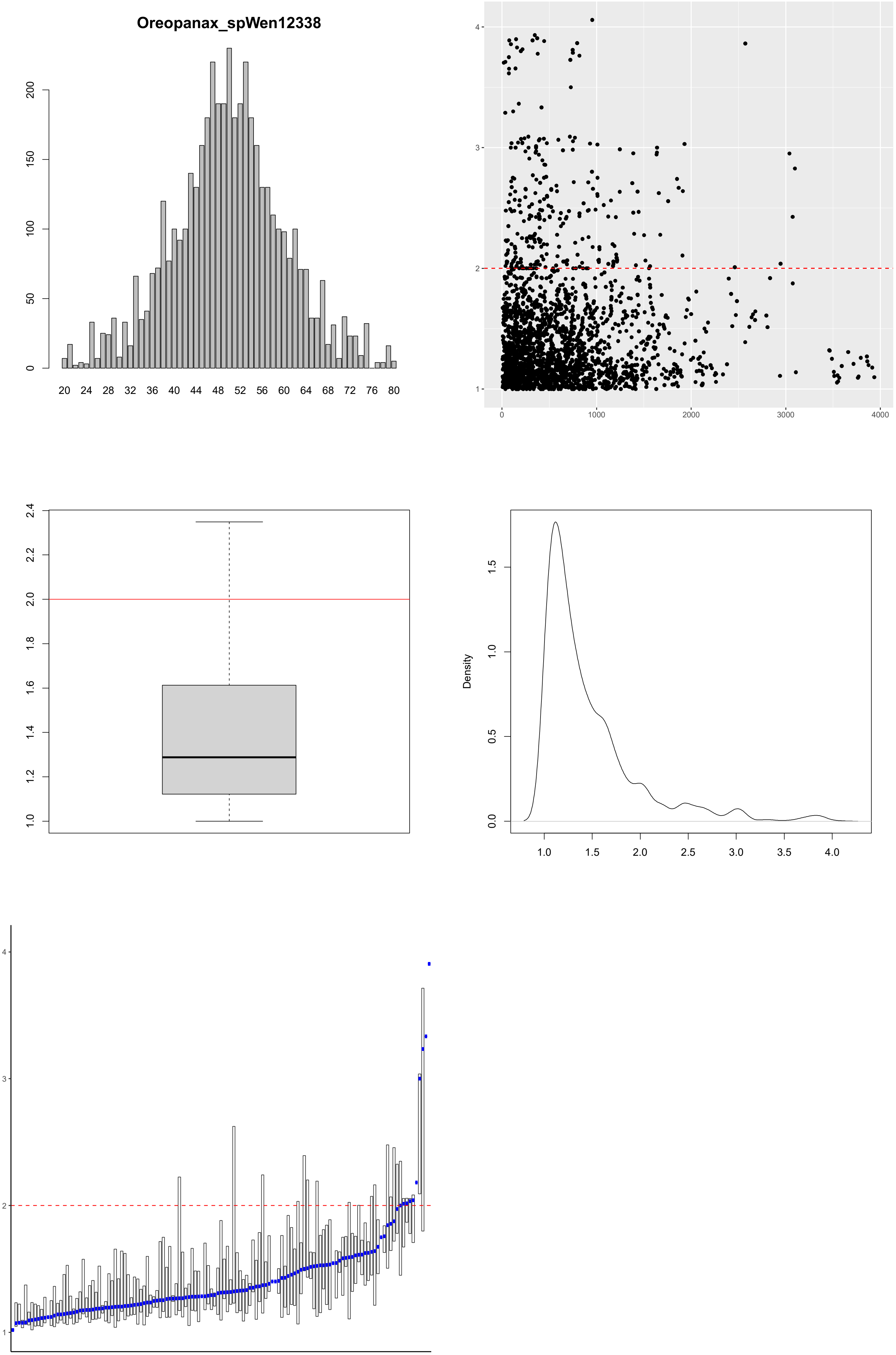
Oreopanax_spWen12338
Density

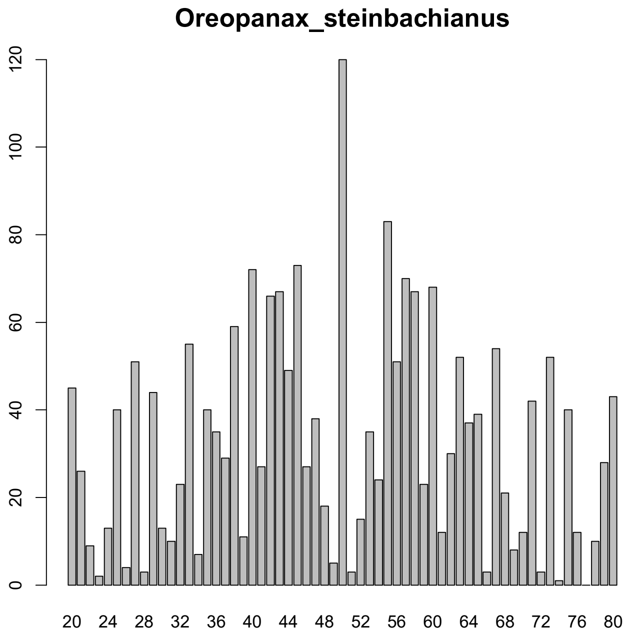
Oreopanax_steinbachianus
0
20
40
60
80
100
120
20 24 28 32 36 40 44 48 52 56 60 64 68 72 76 80

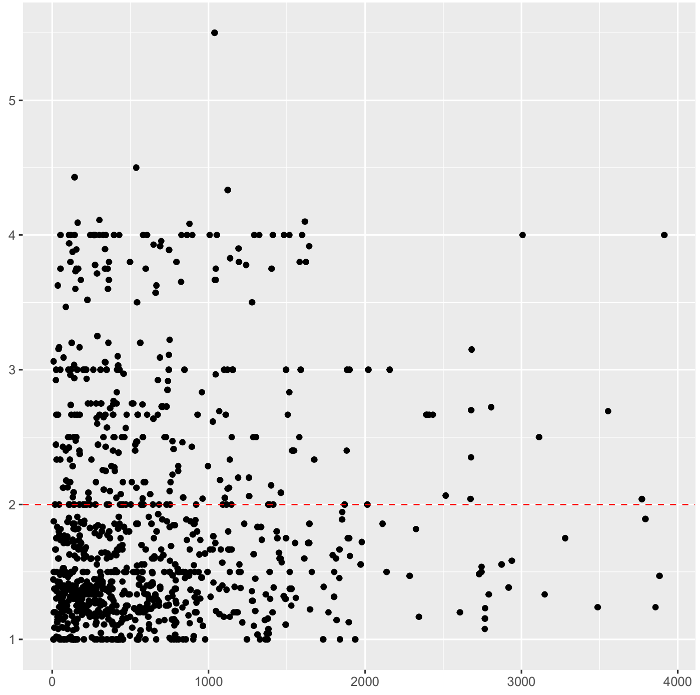
1
2
3
4
5
0 1000 2000 3000 4000

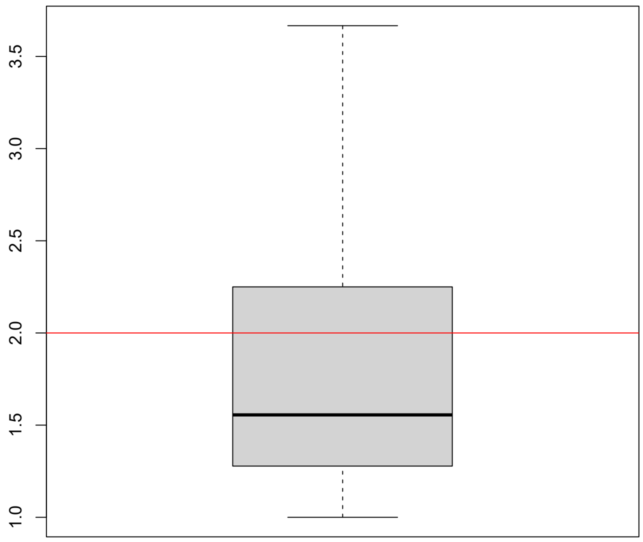
1.0
1.5
2.0
2.5
3.0
3.5

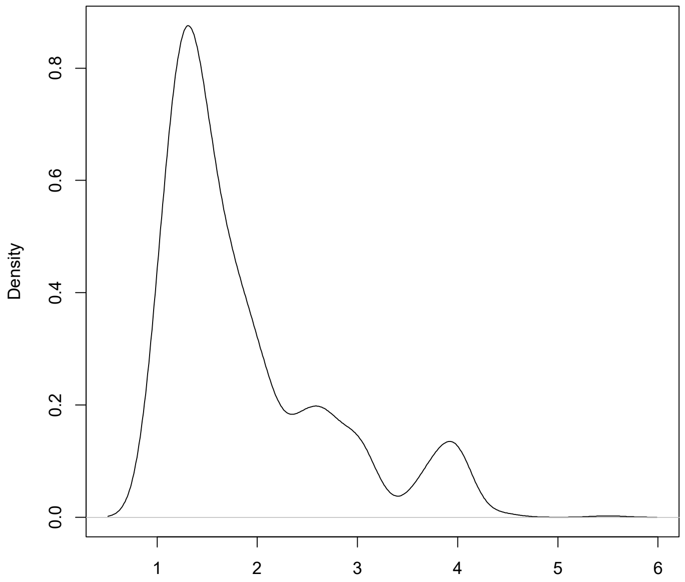
Density
0.0
0.2
0.4
0.6
0.8
1 2 3 4 5 6

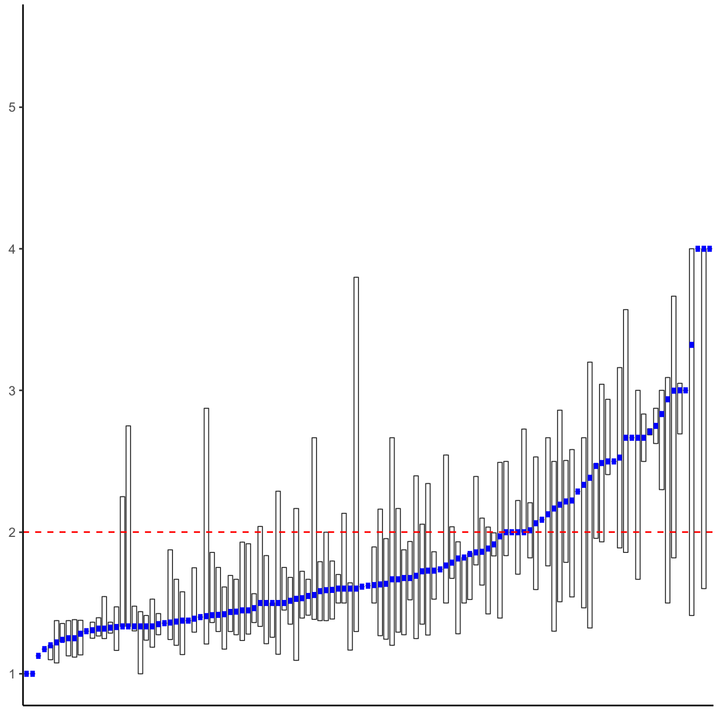
1
2
3
4
5

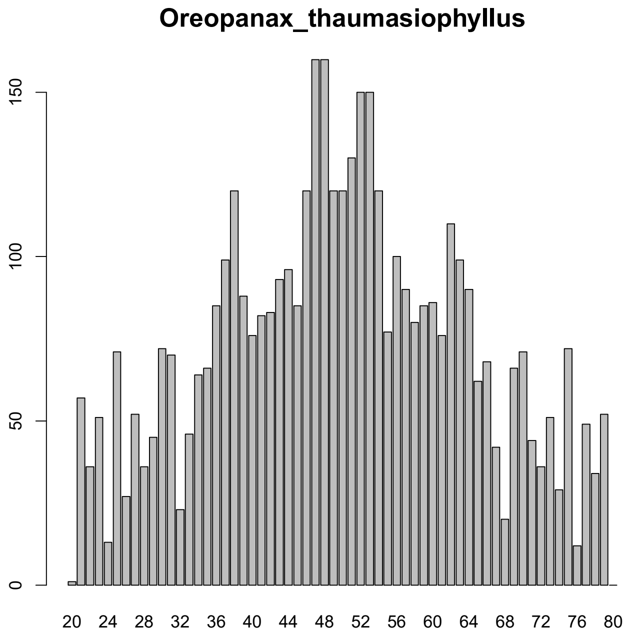
Oreopanax_thaumasiophyllus
0
50
100
150
20 24 28 32 36 40 44 48 52 56 60 64 68 72 76 80

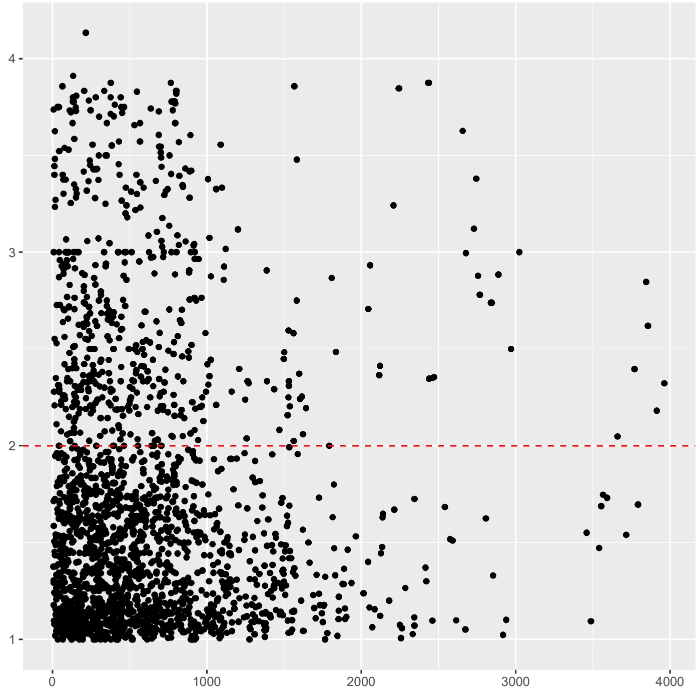
1
2
3
4
0
1000
2000
3000
4000

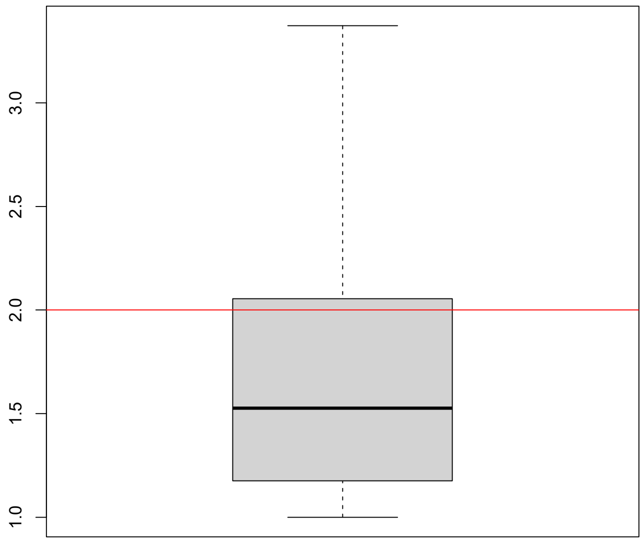
1.0
1.5
2.0
2.5
3.0

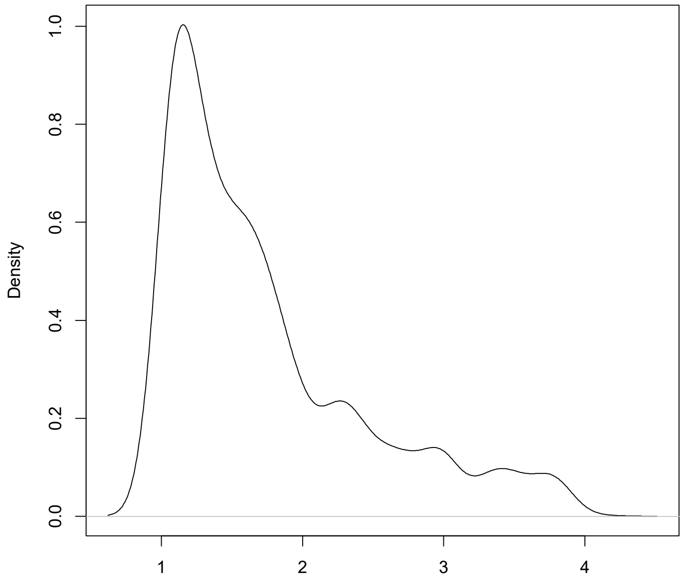
Density
0.0
0.2
0.4
0.6
0.8
1.0
1 2 3 4

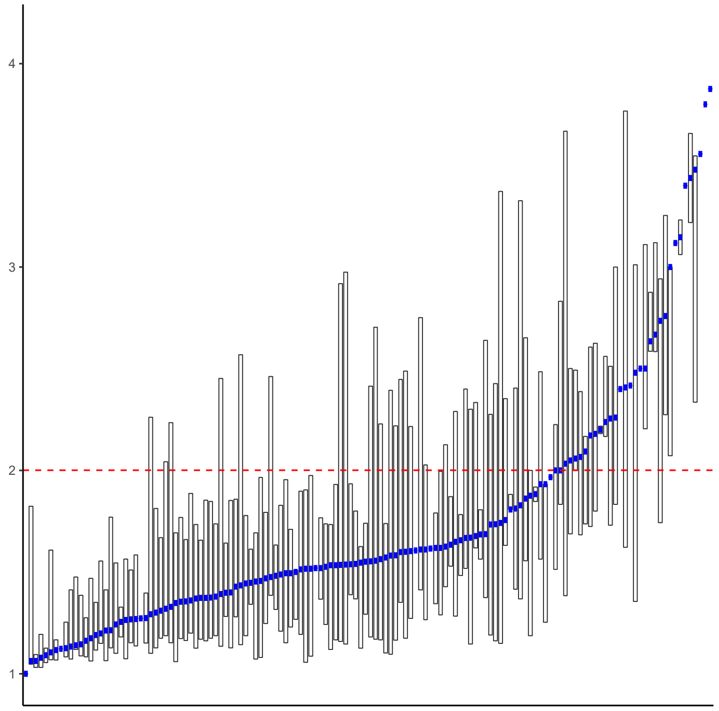
1
2
3
4

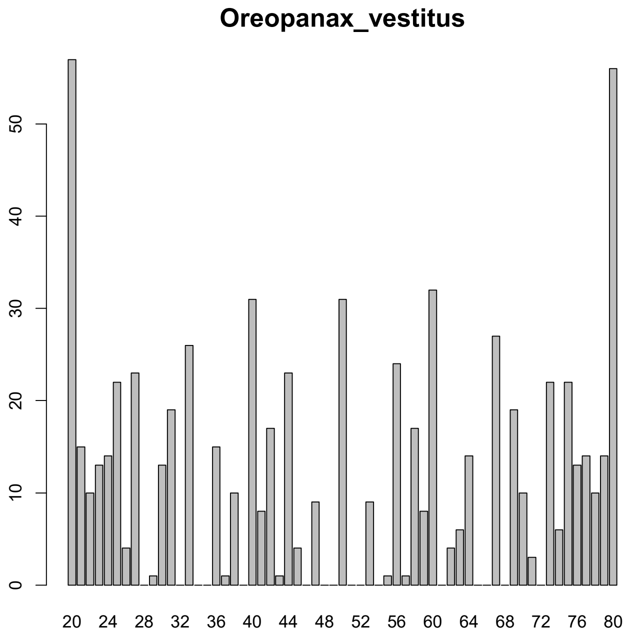
Oreopanax_vestitus
0
10
20
30
40
50
20 24 28 32 36 40 44 48 52 56 60 64 68 72 76 80

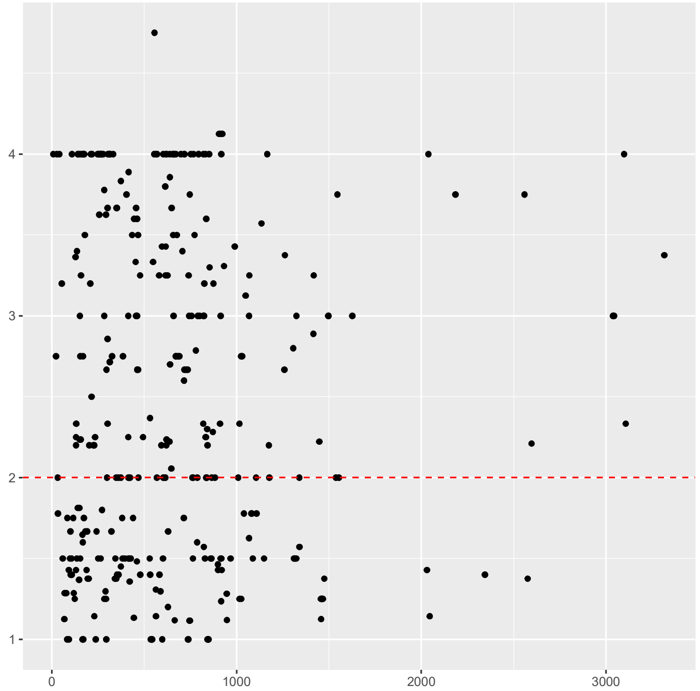
1
2
3
4
0 1000 2000 3000

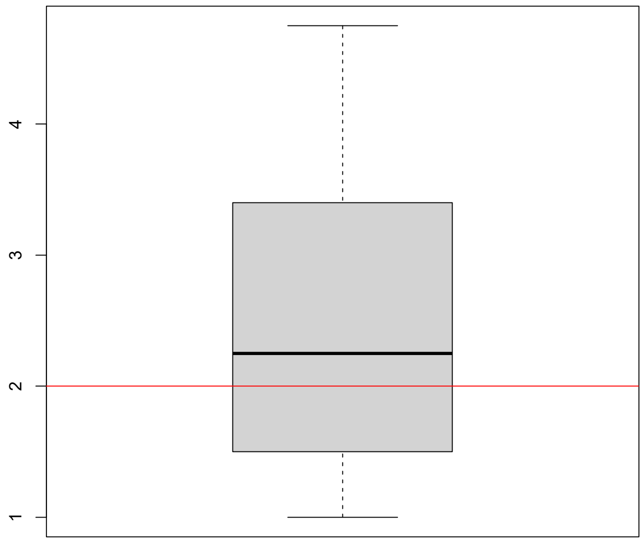
1
2
3
4

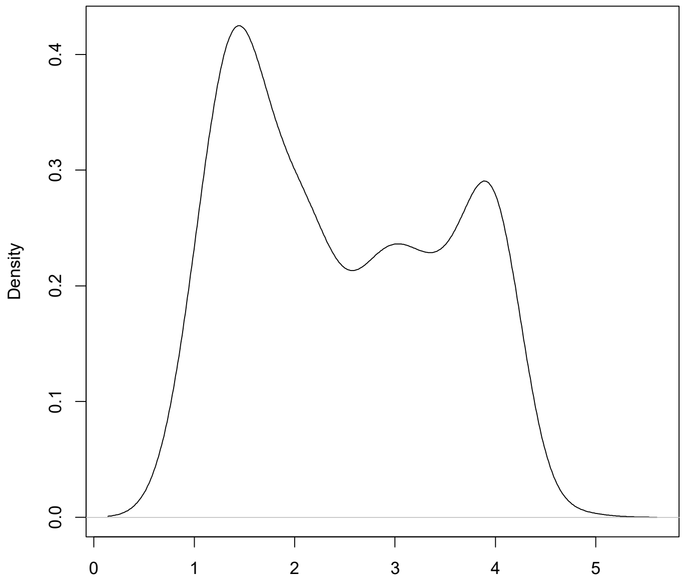
Density
0.0
0.1
0.2
0.3
0.4
0 1 2 3 4 5

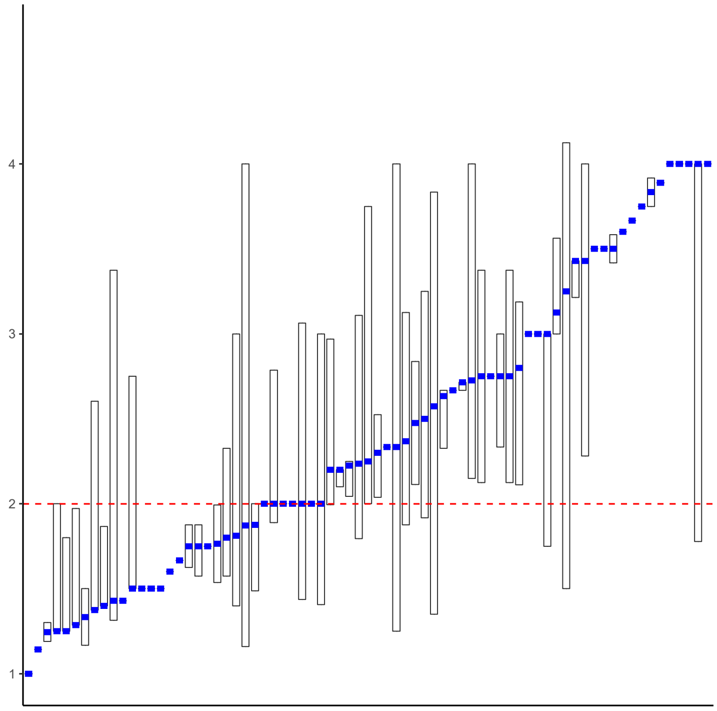
1
2
3
4

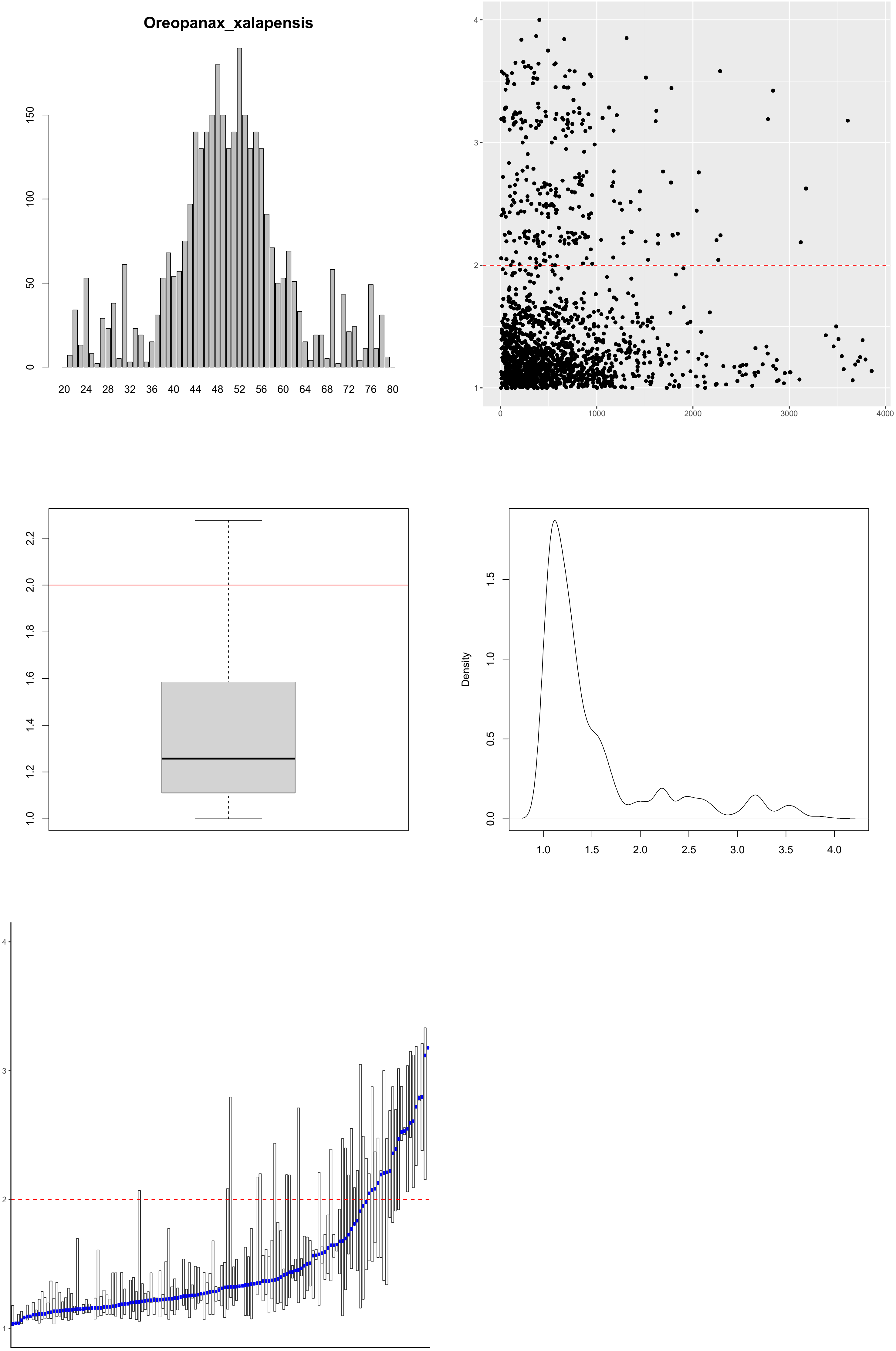
Oreopanax_xalapensis
Density

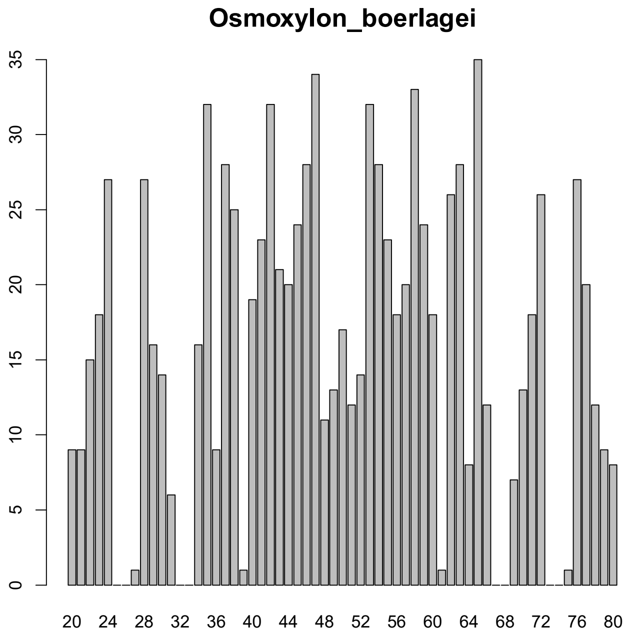
Osmoxylon_boerlagei

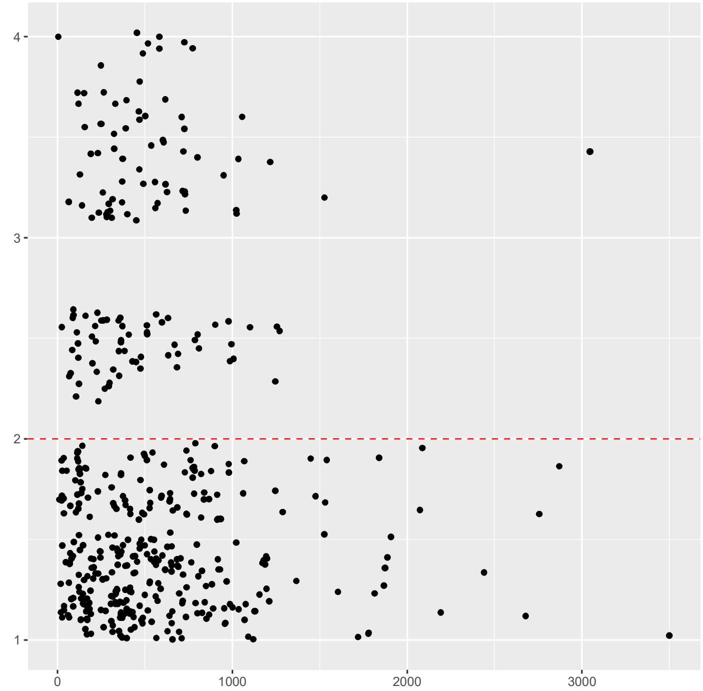

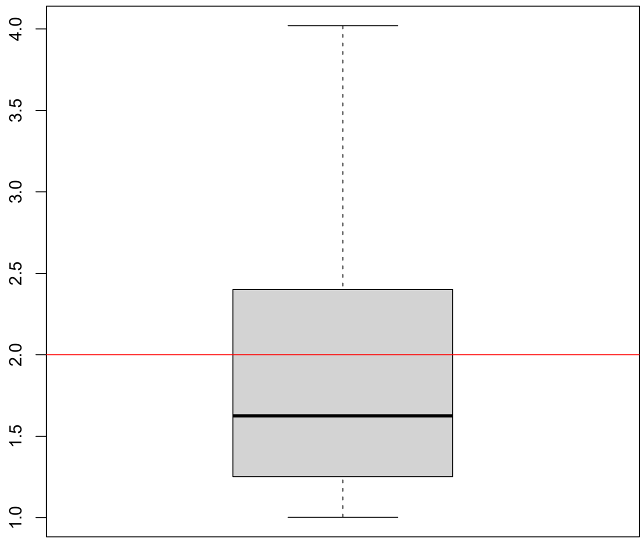

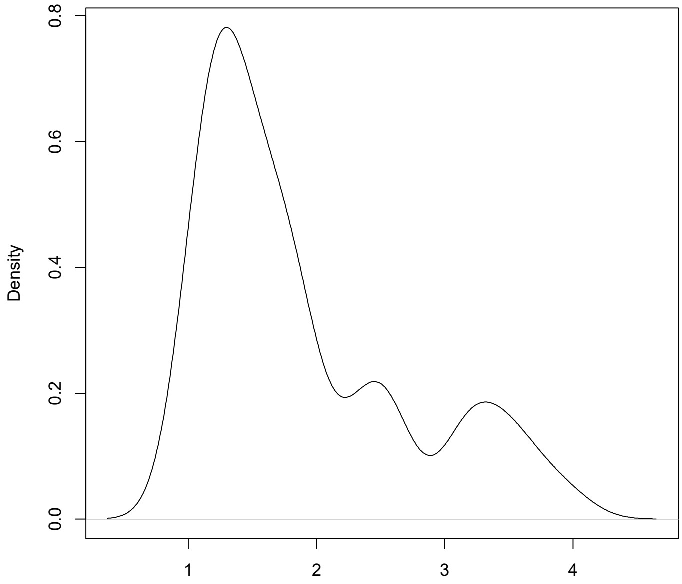
Density

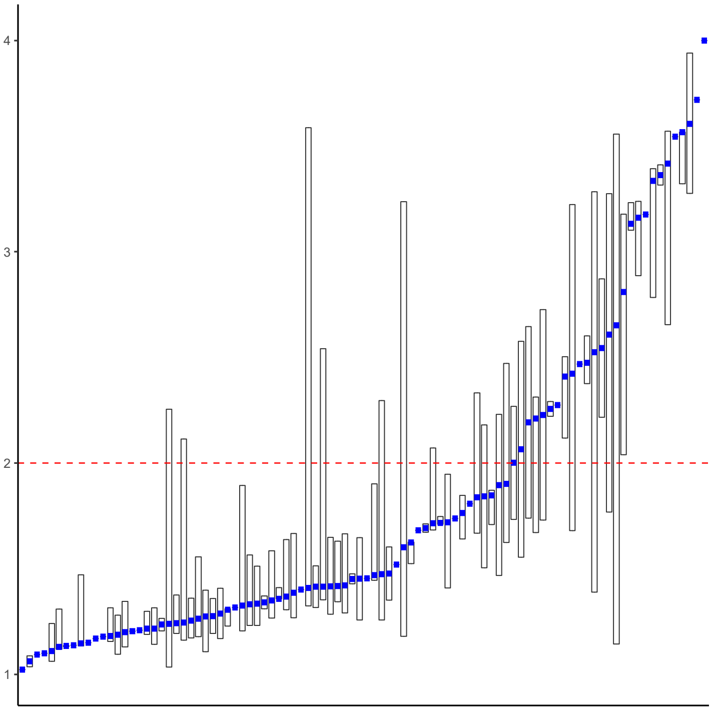

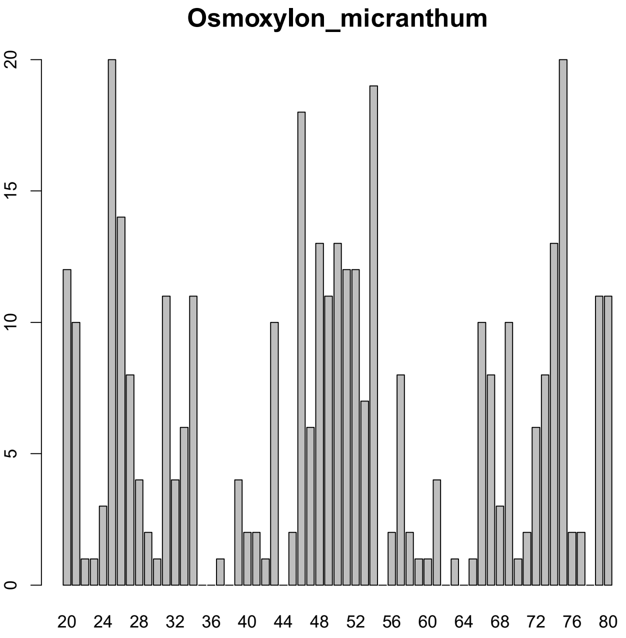
Osmoxylon_micranthum
0
5
10
15
20
20 24 28 32 36 40 44 48 52 56 60 64 68 72 76 80

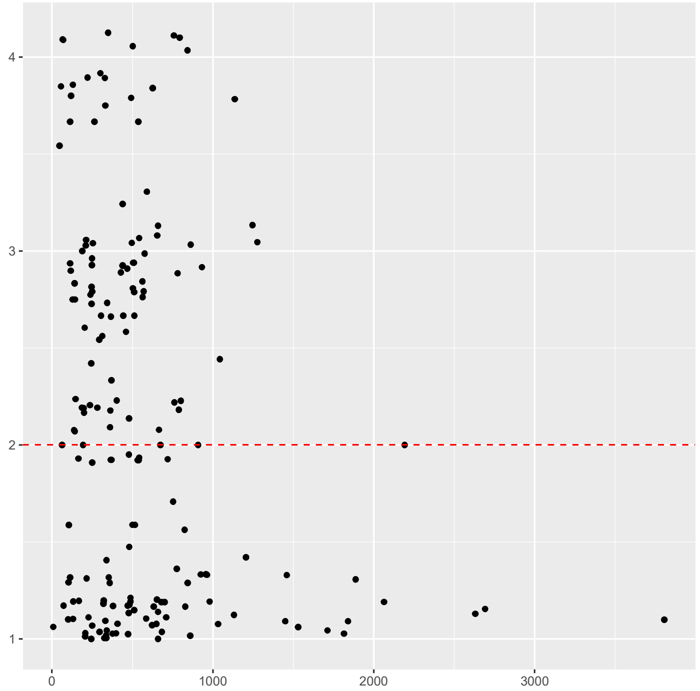
1
2
3
4
0 1000 2000 3000

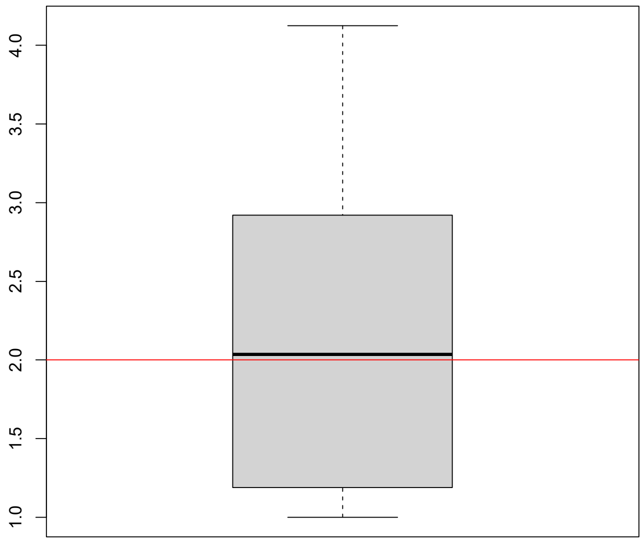
1.0
1.5
2.0
2.5
3.0
3.5
4.0

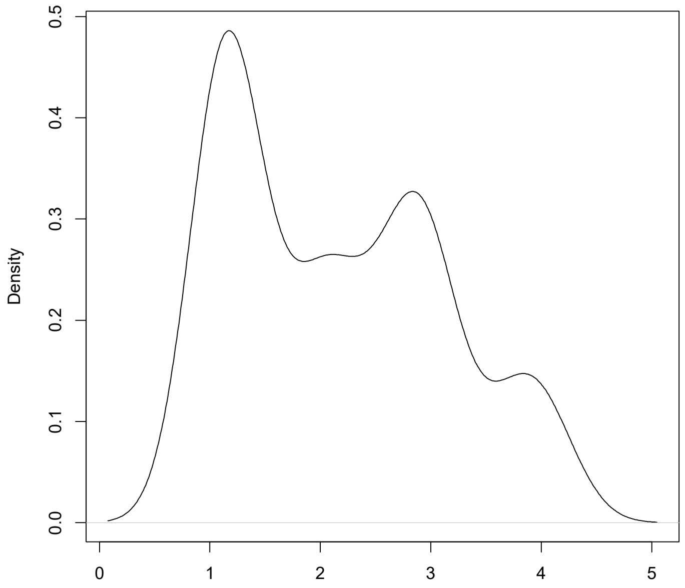
Density
0.0
0.1
0.2
0.3
0.4
0.5
0 1 2 3 4 5

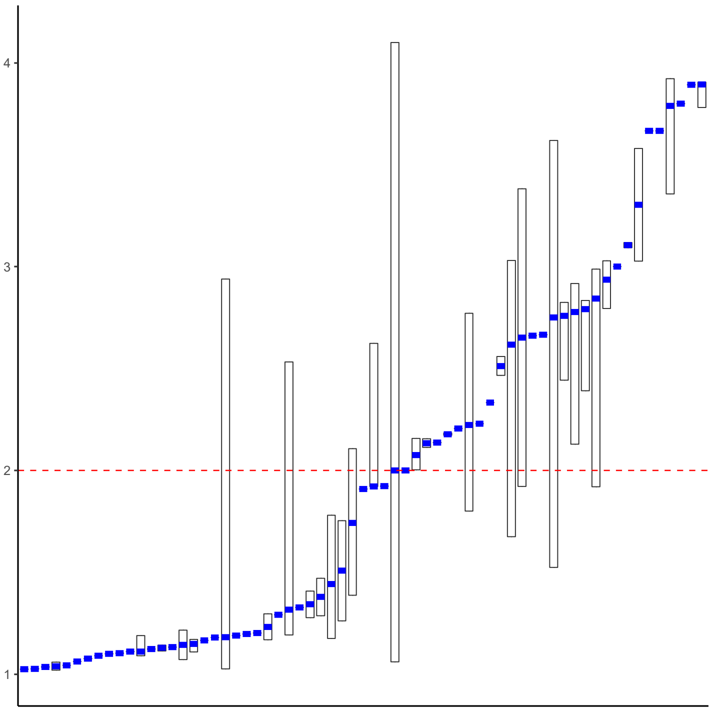
1
2
3
4

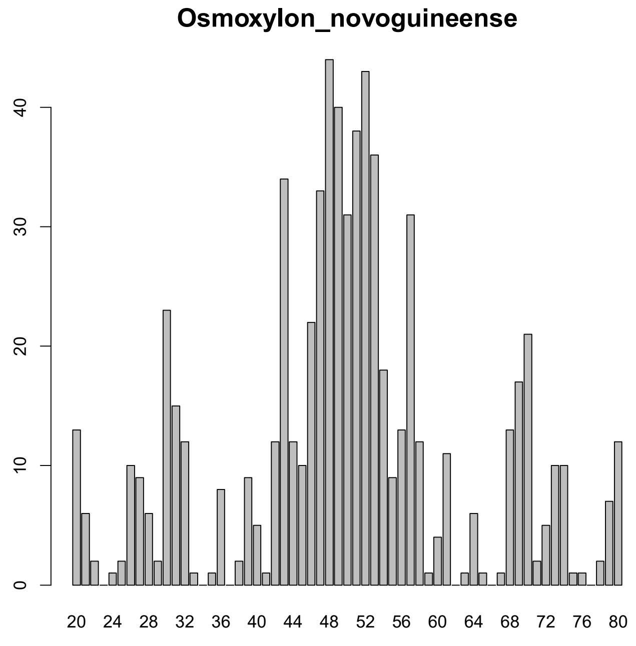
Osmoxylon_novoguineense

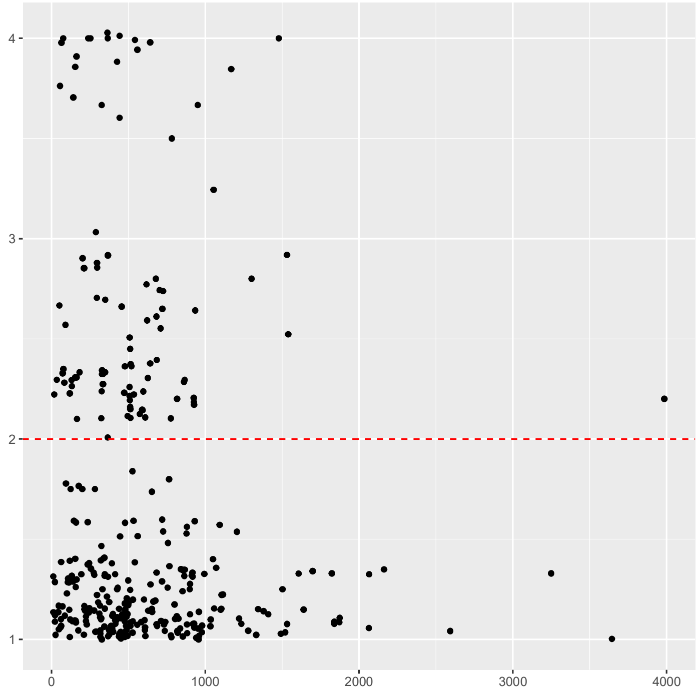

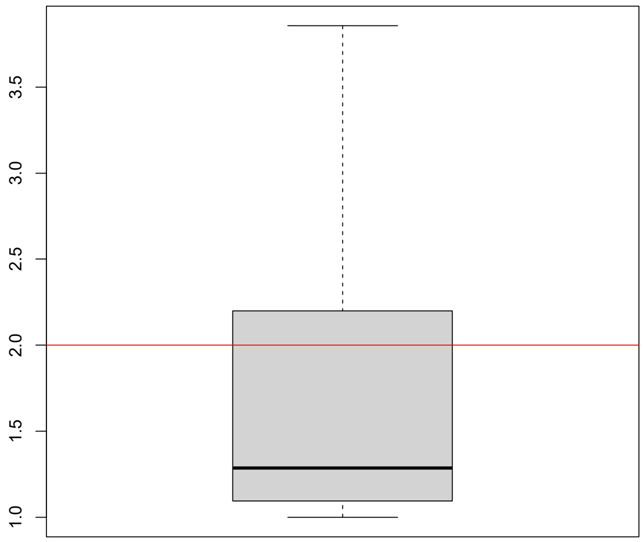

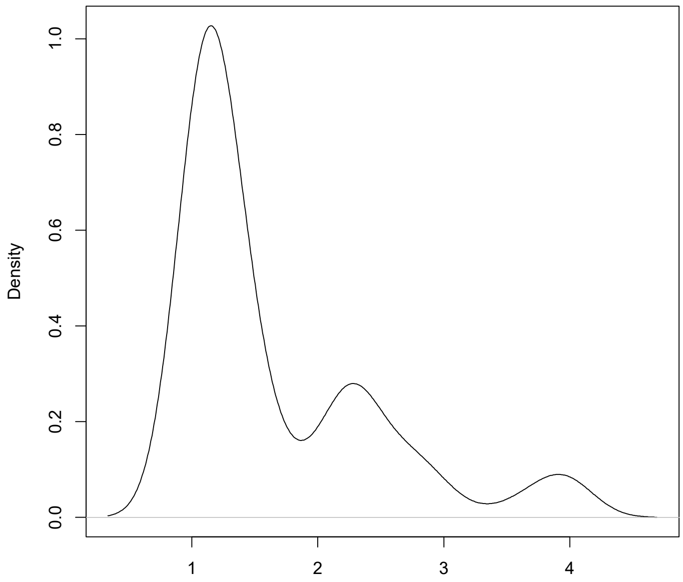
Density

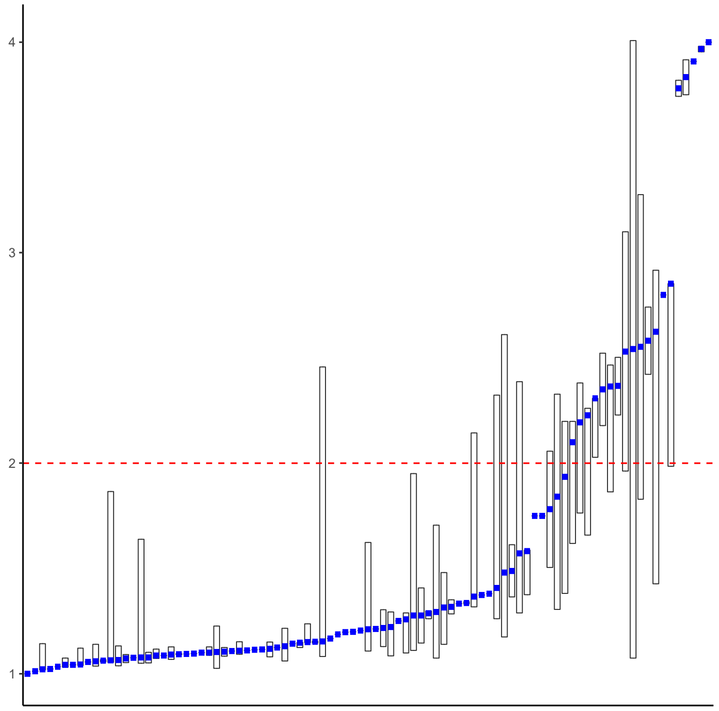

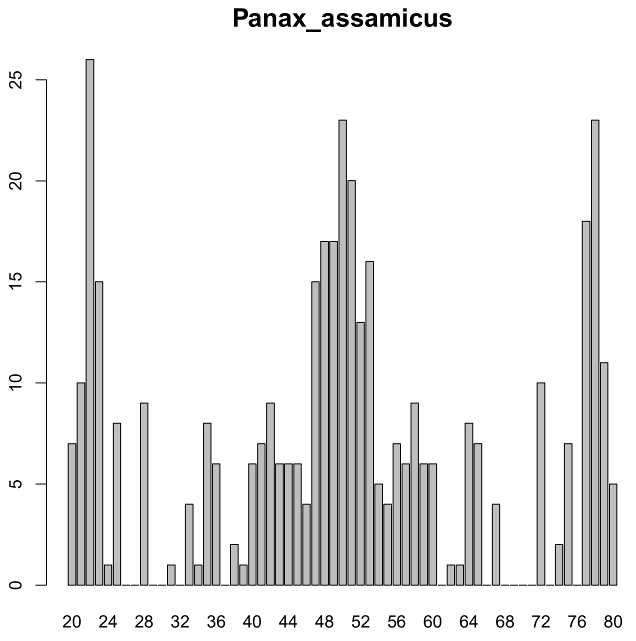
Panax_assamicus
0
5
10
15
20
25
20 24 28 32 36 40 44 48 52 56 60 64 68 72 76 80

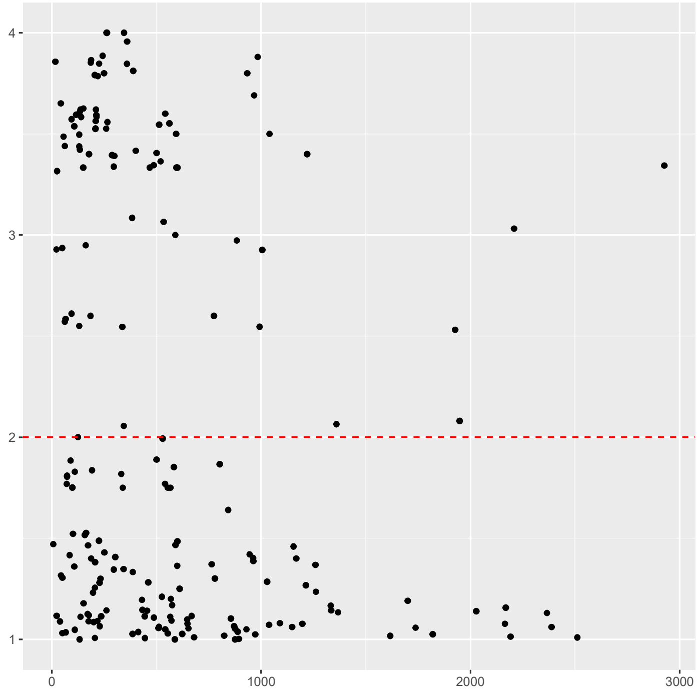
1
2
3
4
0 1000 2000 3000

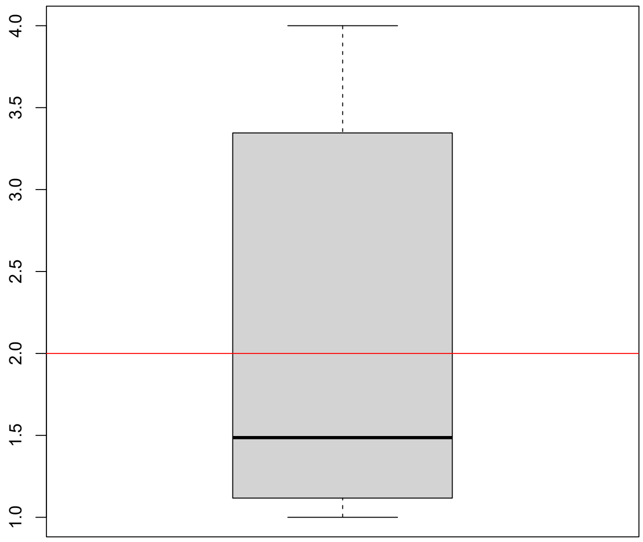
1.0
1.5
2.0
2.5
3.0
3.5
4.0

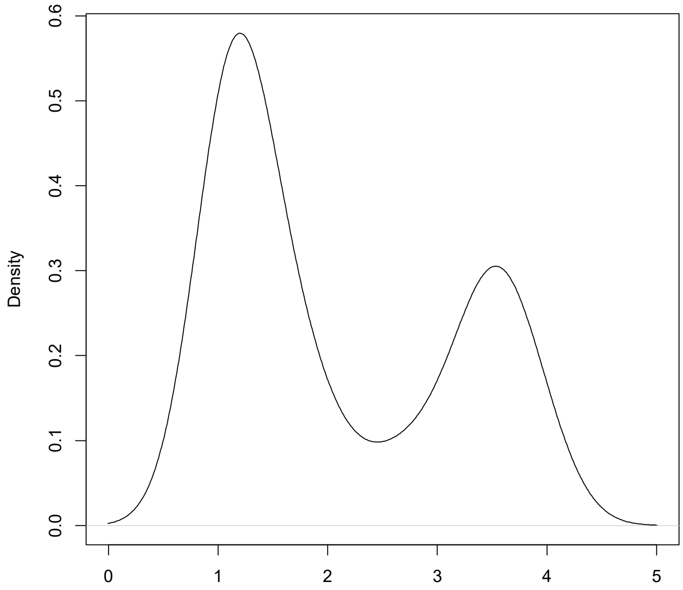
Density
0.0
0.1
0.2
0.3
0.4
0.5
0.6
0 1 2 3 4 5

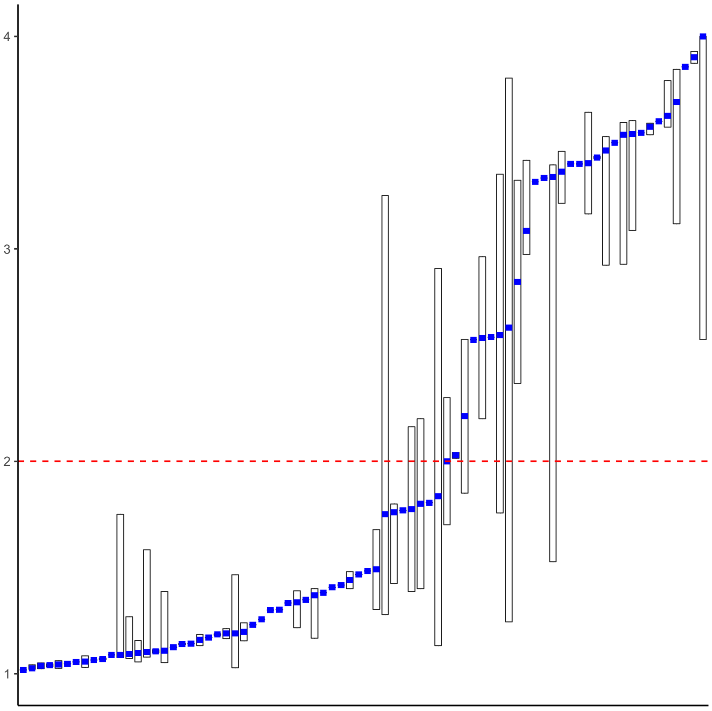
1
2
3
4

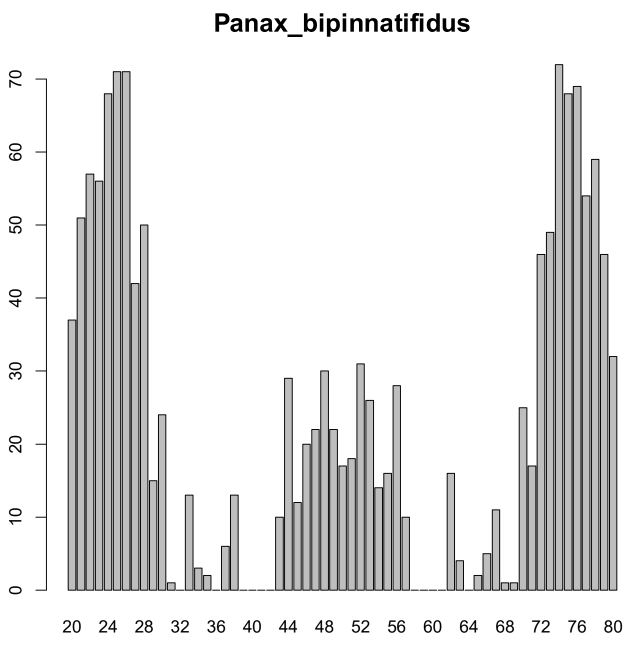
Panax_bipinnatifidus

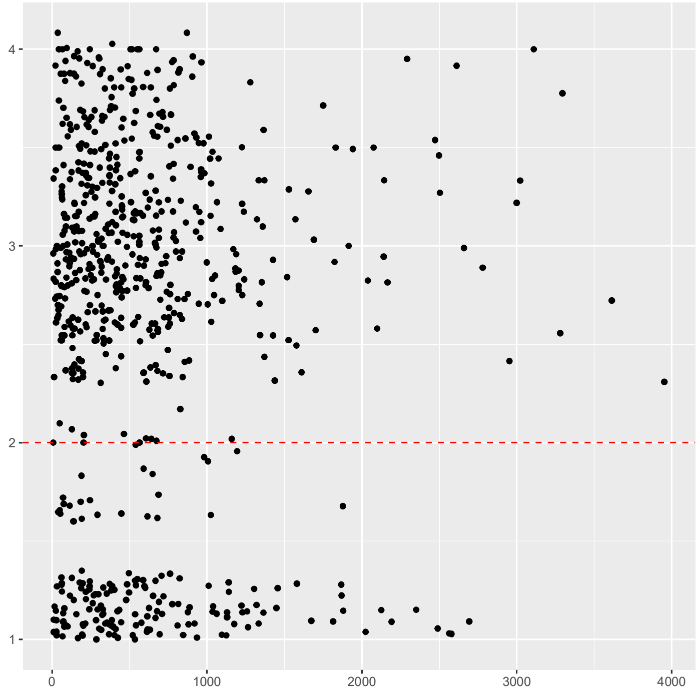

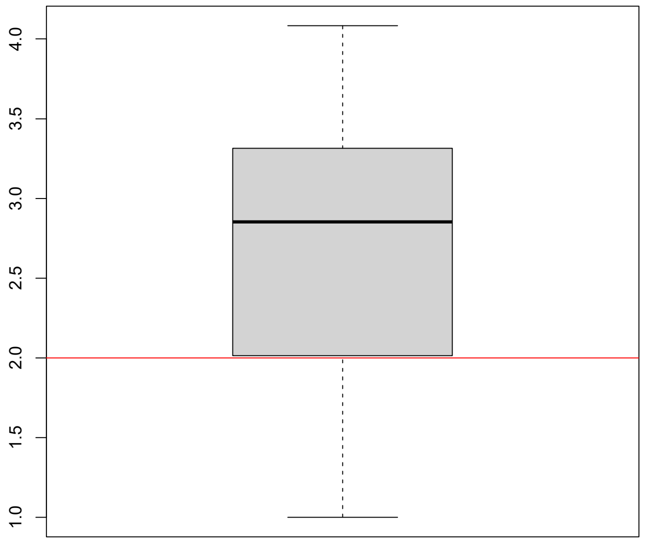

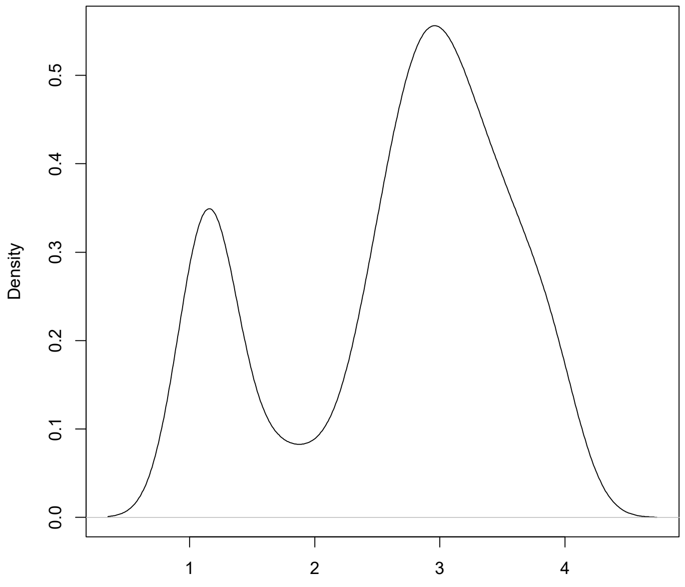
Density

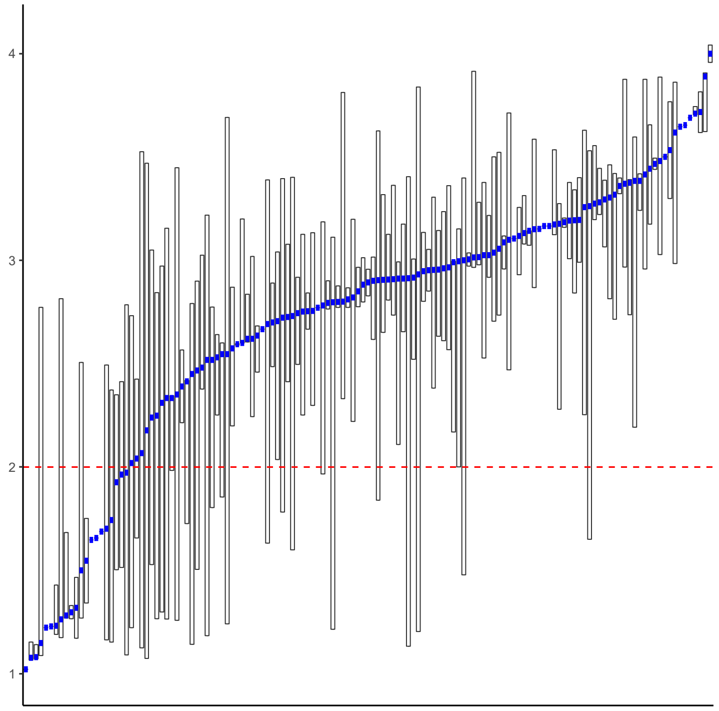

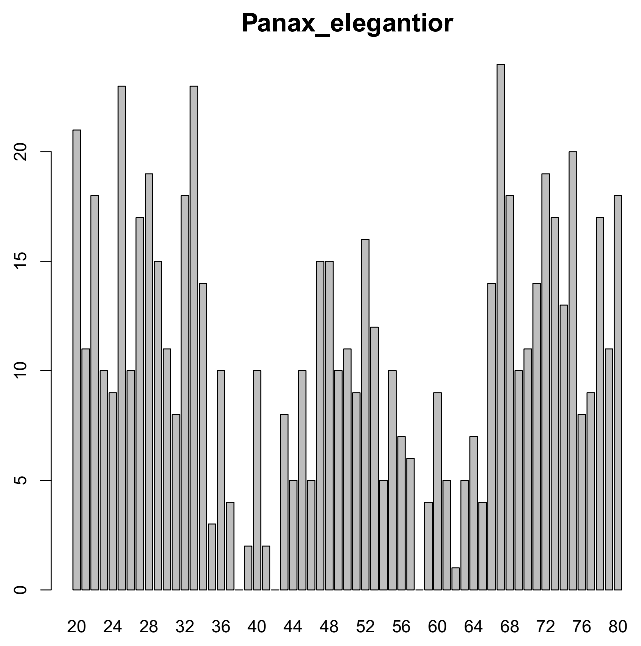
Panax_elegantior

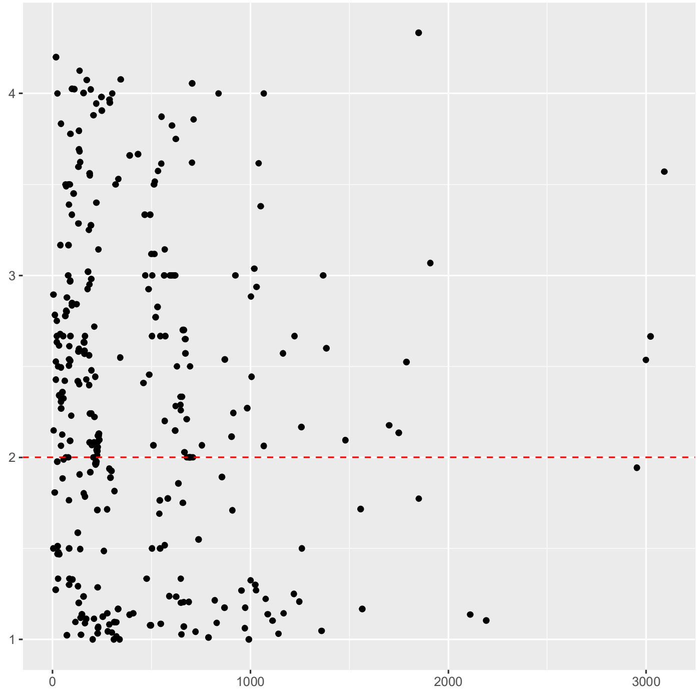

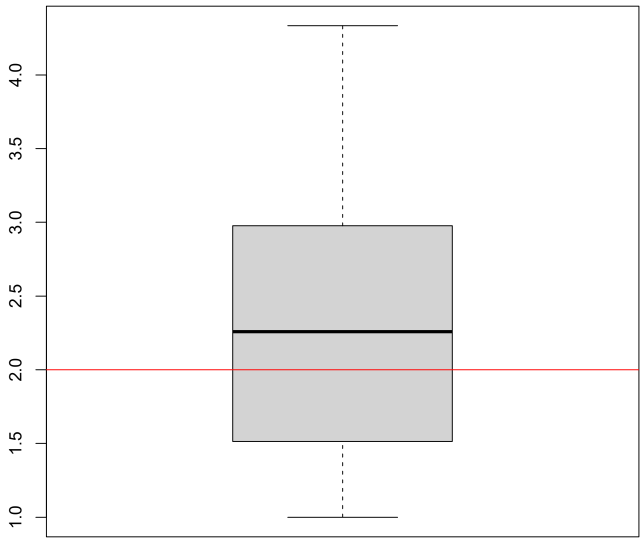

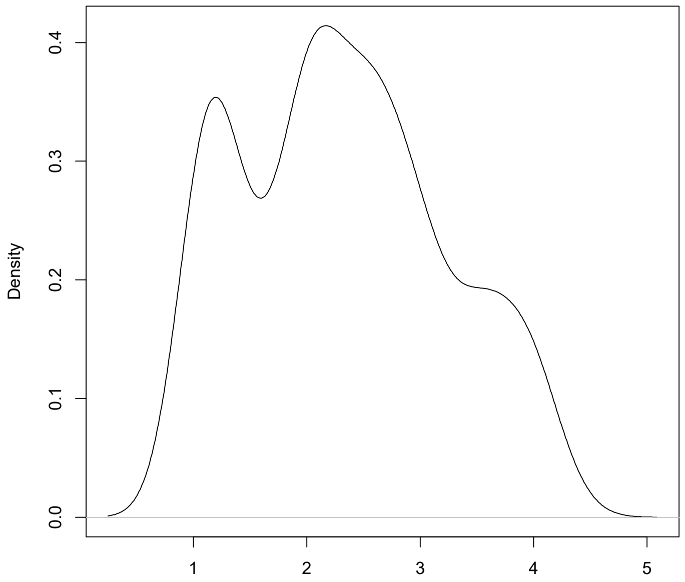
Density

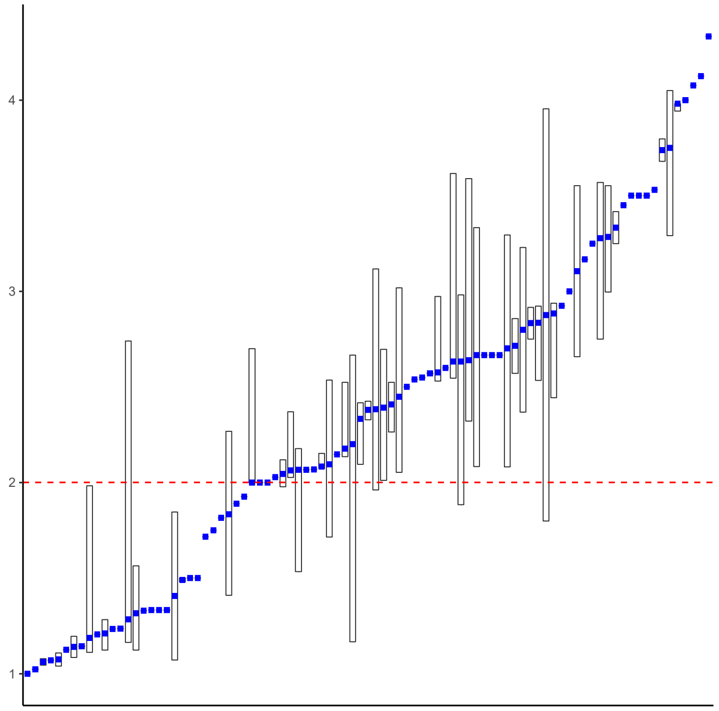

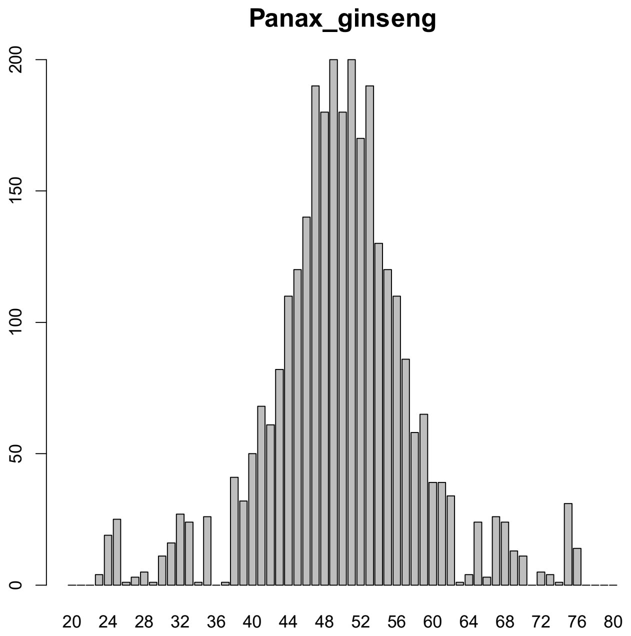
Panax_ginseng
0
50
100
150
200
20 24 28 32 36 40 44 48 52 56 60 64 68 72 76 80

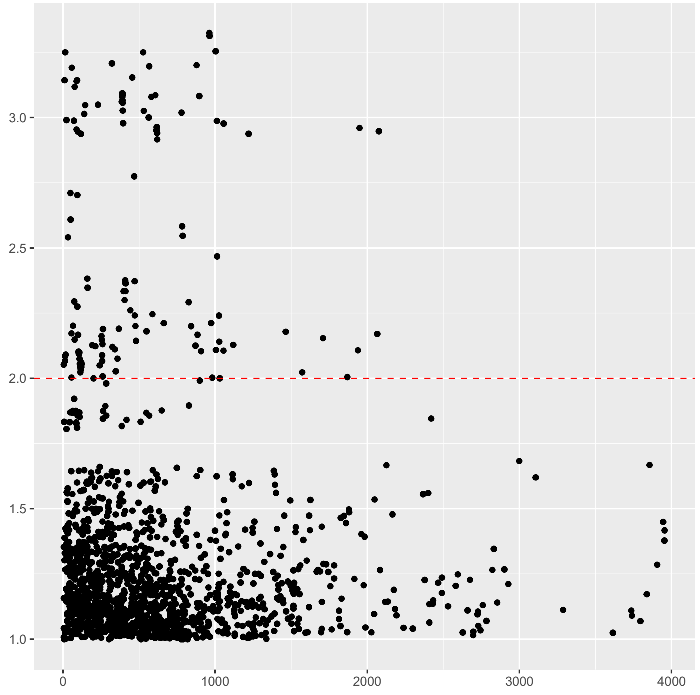
1.0
1.5
2.0
2.5
3.0
0 1000 2000 3000 4000

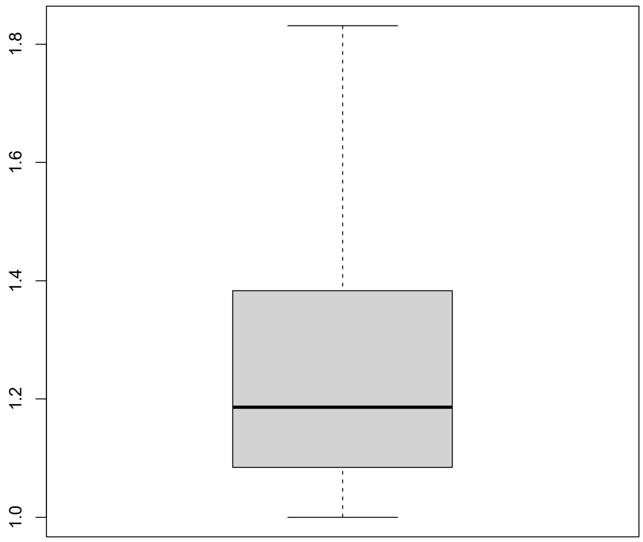
1.0
1.2
1.4
1.6
1.8

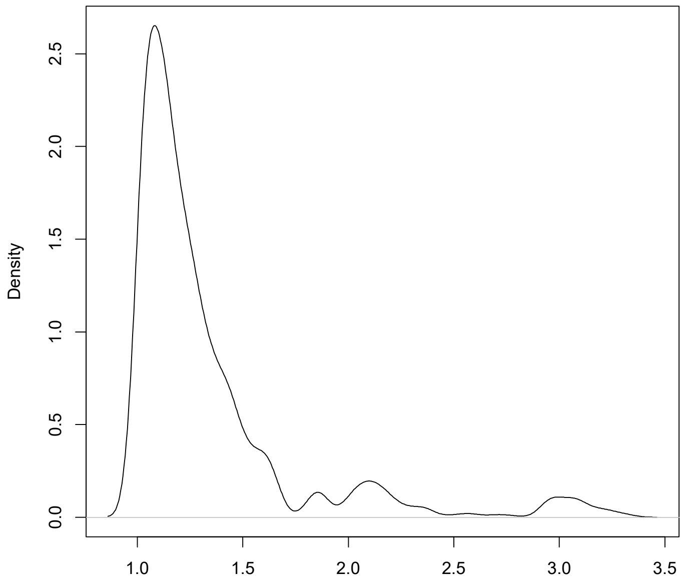
Density
0.0
0.5
1.0
1.5
2.0
2.5
1.0 1.5 2.0 2.5 3.0 3.5

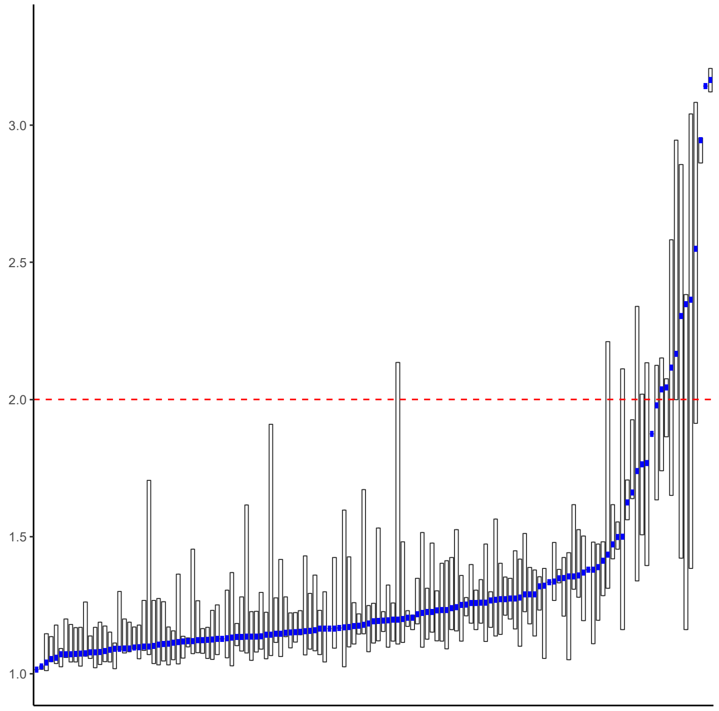
1.0
1.5
2.0
2.5
3.0

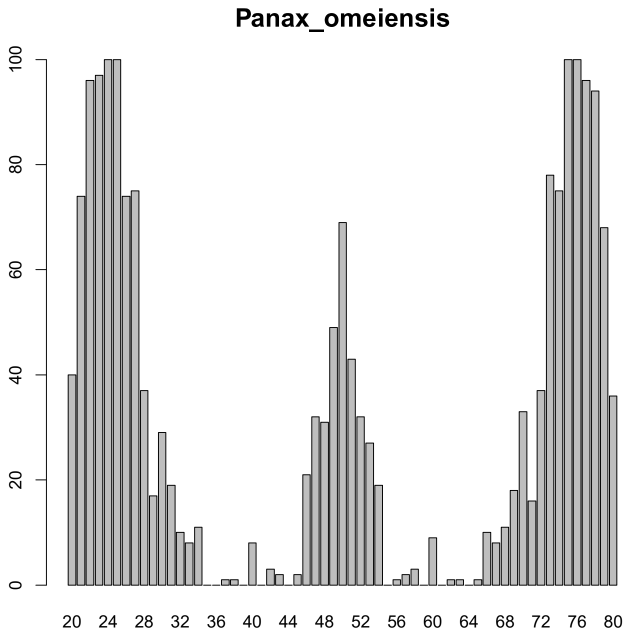
Panax_omeiensis
0
20
40
60
80
100
20 24 28 32 36 40 44 48 52 56 60 64 68 72 76 80

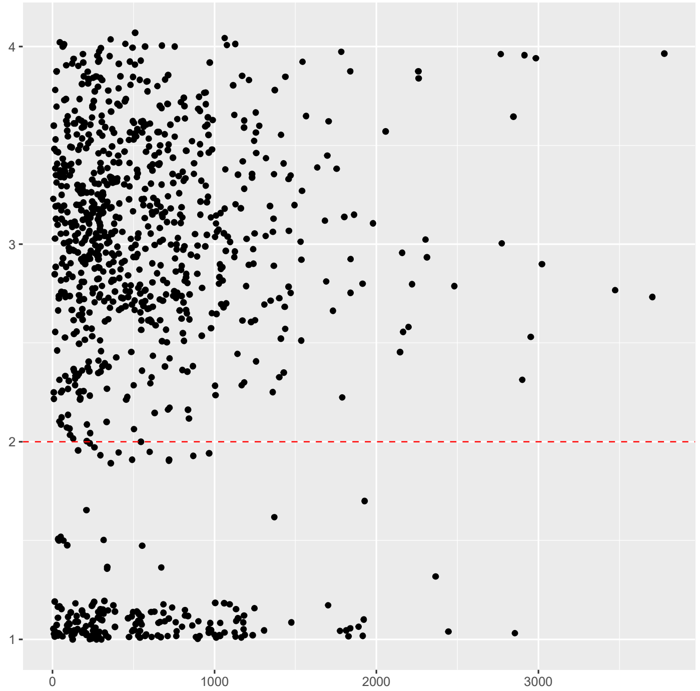
1
2
3
4
0 1000 2000 3000

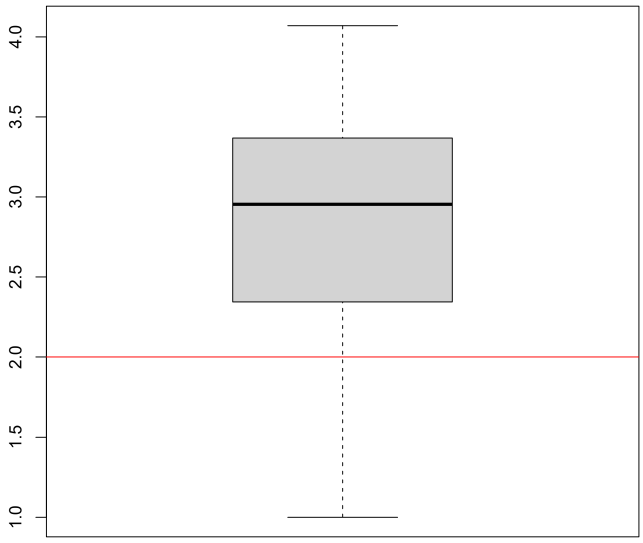
1.0
1.5
2.0
2.5
3.0
3.5
4.0

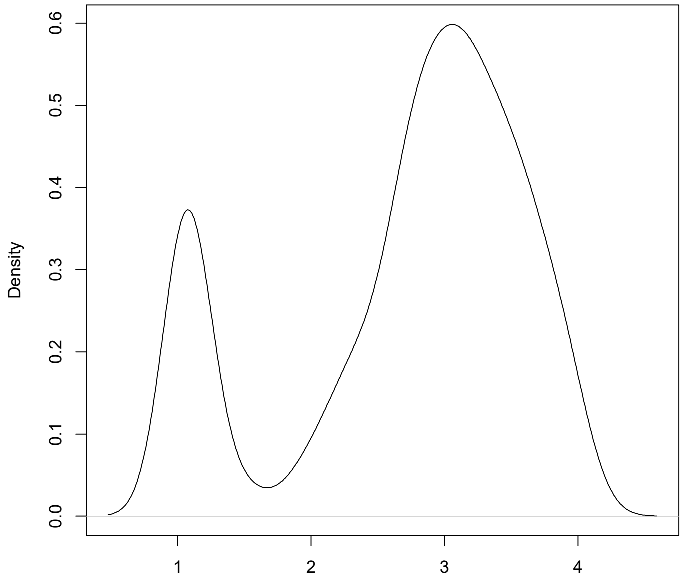
Density
0.0
0.1
0.2
0.3
0.4
0.5
0.6
1 2 3 4

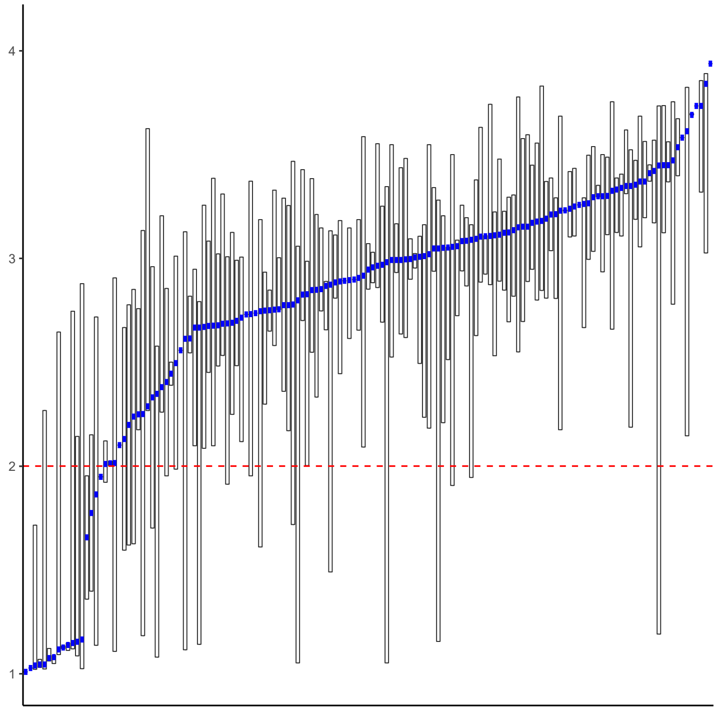
1
2
3
4

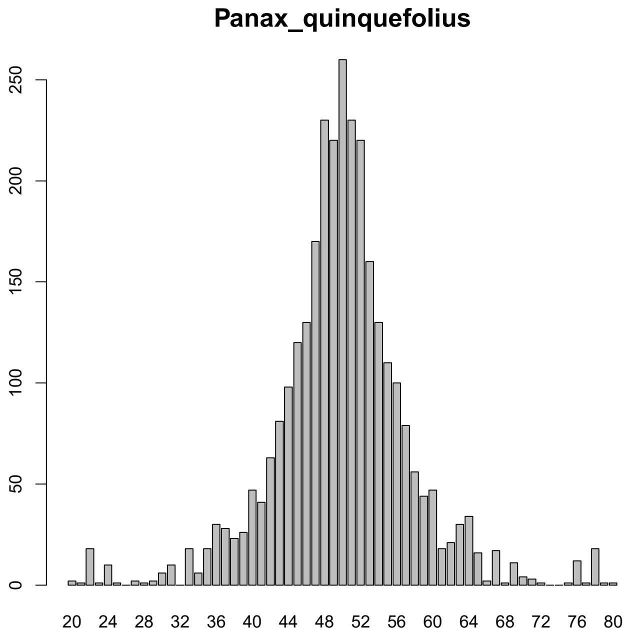
Panax_quinquefolius

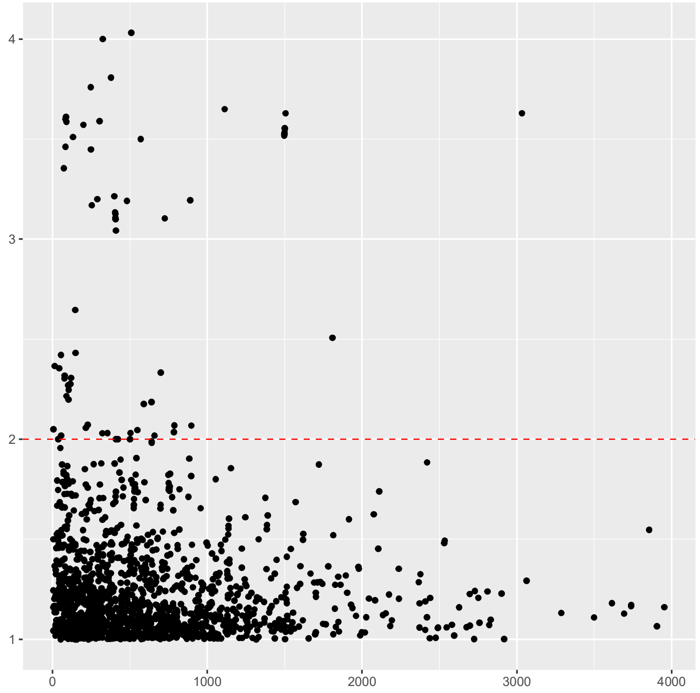

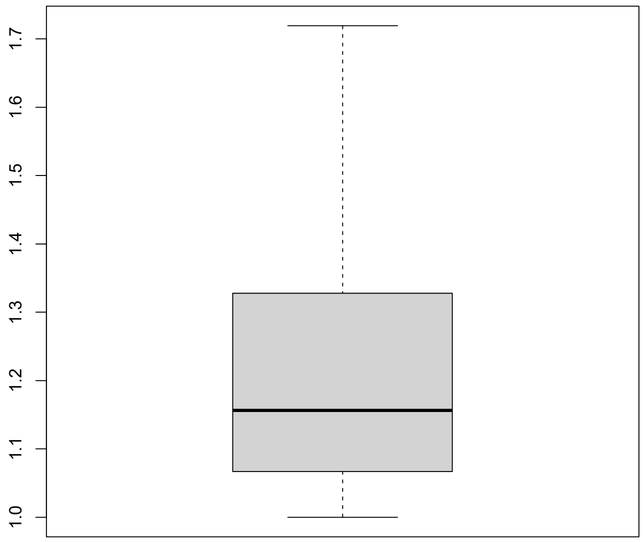

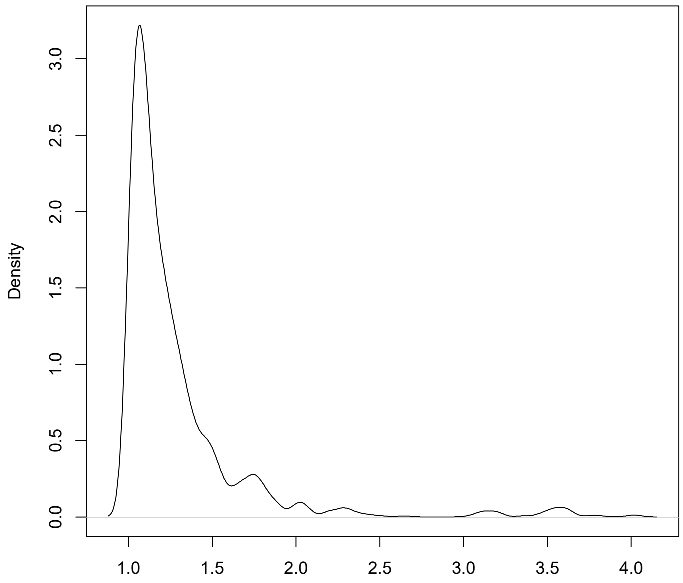
Density

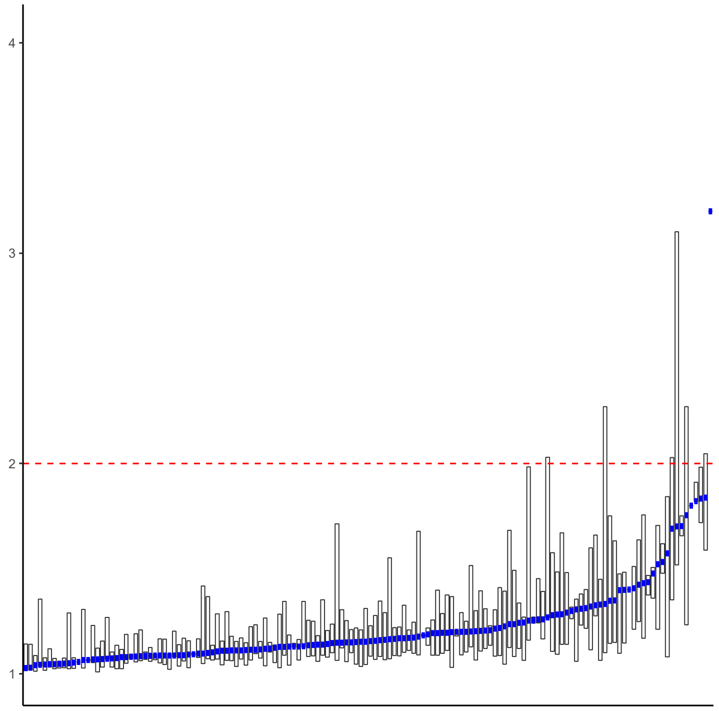

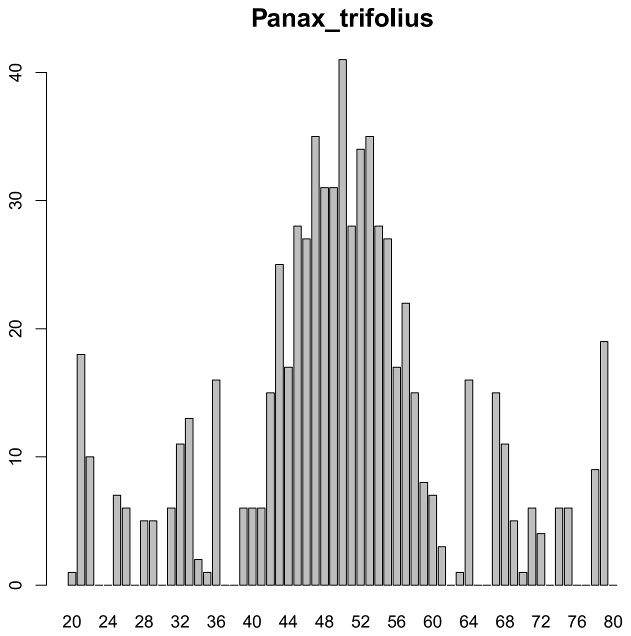
Panax_trifolius
0
10
20
30
40
20 24 28 32 36 40 44 48 52 56 60 64 68 72 76 80

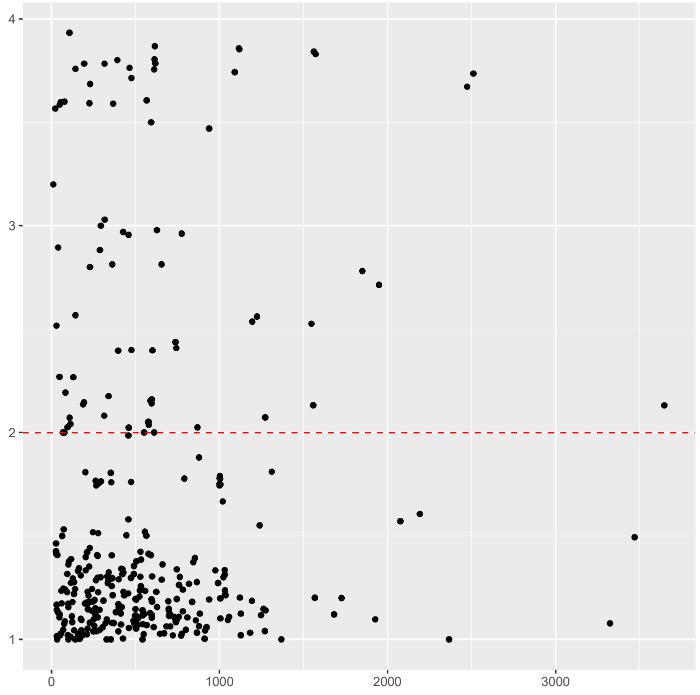
1
2
3
4
0
1000
2000
3000

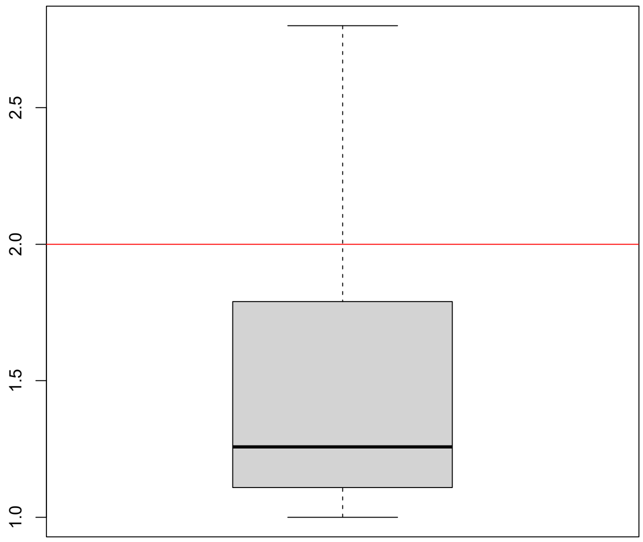
1.0
1.5
2.0
2.5

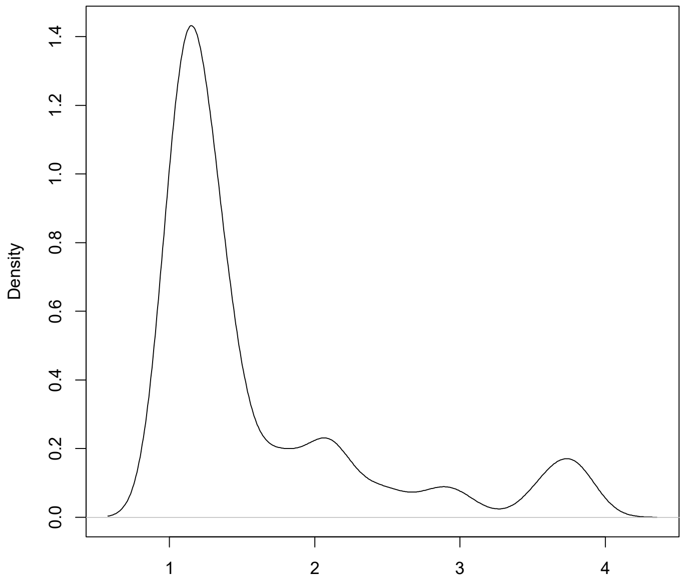
Density
0.0
0.2
0.4
0.6
0.8
1.0
1.2
1.4
1
2
3
4

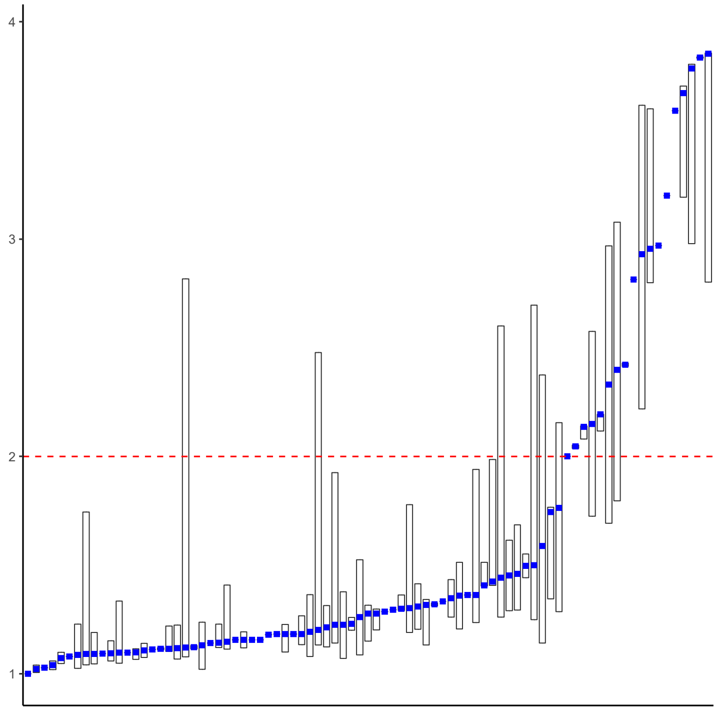
1
2
3
4

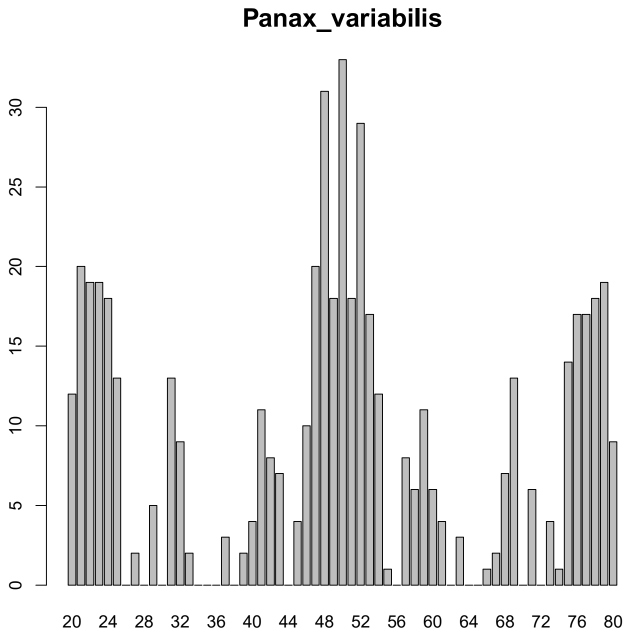
Panax_variabilis
0
5
10
15
20
25
30
20 24 28 32 36 40 44 48 52 56 60 64 68 72 76 80

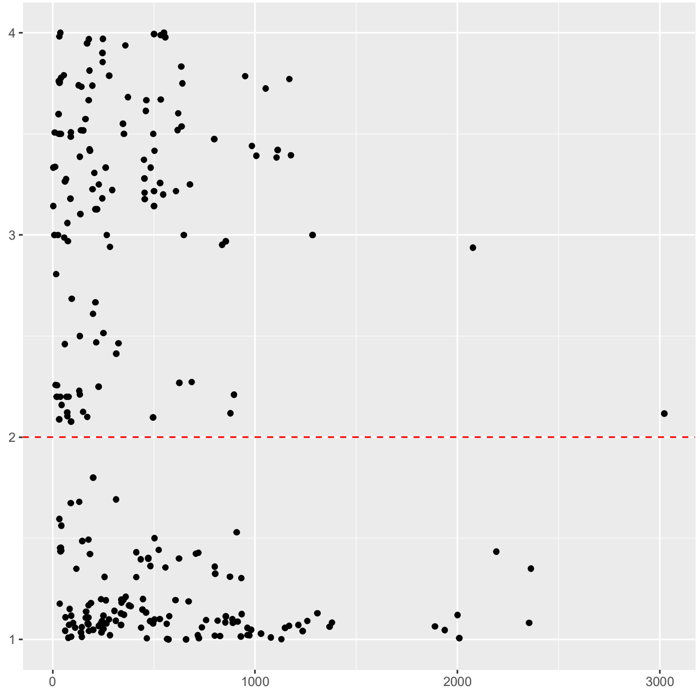
1
2
3
4
0 1000 2000 3000

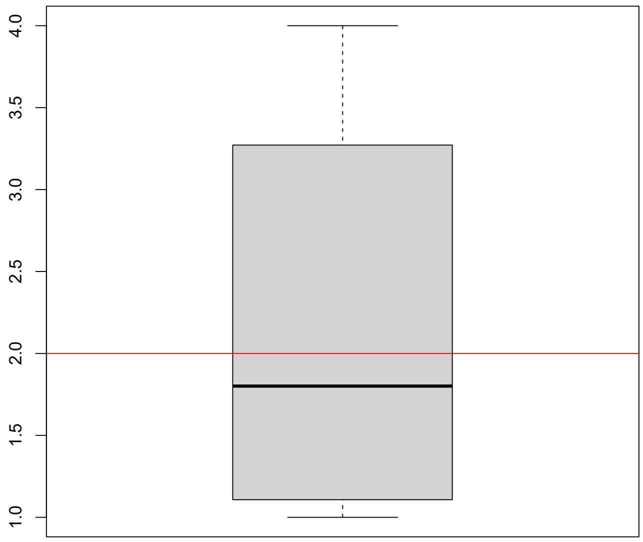
1.0
1.5
2.0
2.5
3.0
3.5
4.0

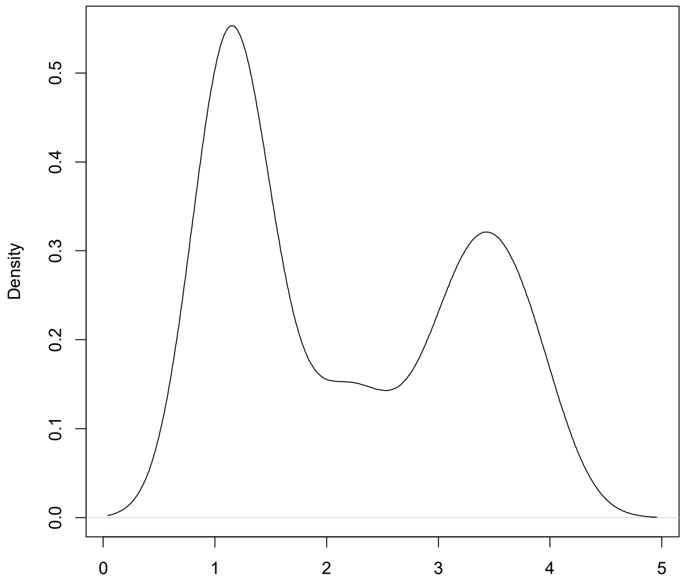
Density
0.0
0.1
0.2
0.3
0.4
0.5
0 1 2 3 4 5

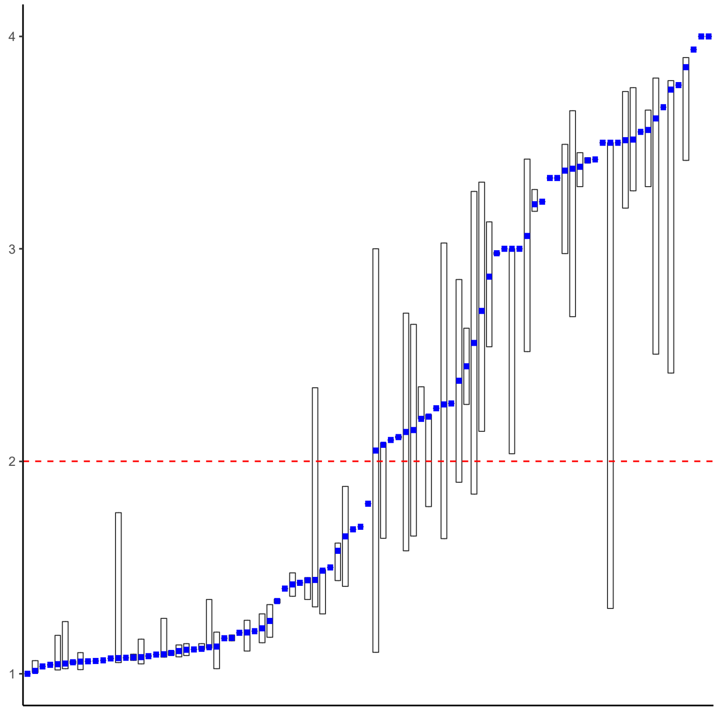
1
2
3
4

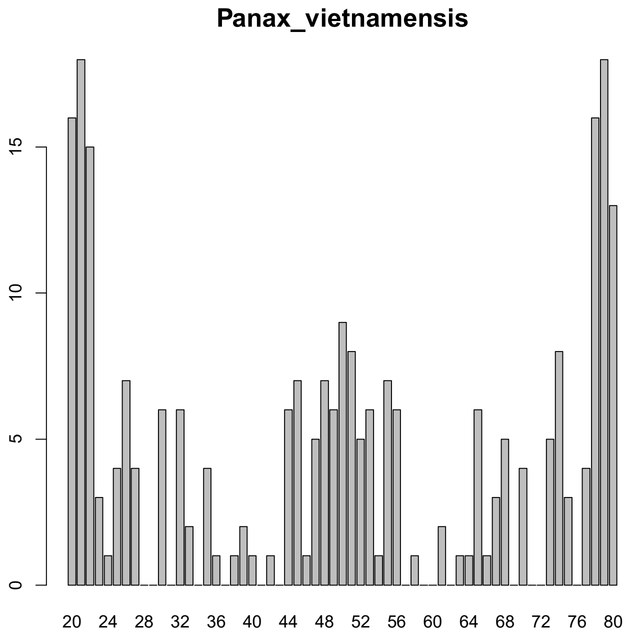
Panax_vietnamensis
0
5
10
15
20 24 28 32 36 40 44 48 52 56 60 64 68 72 76 80

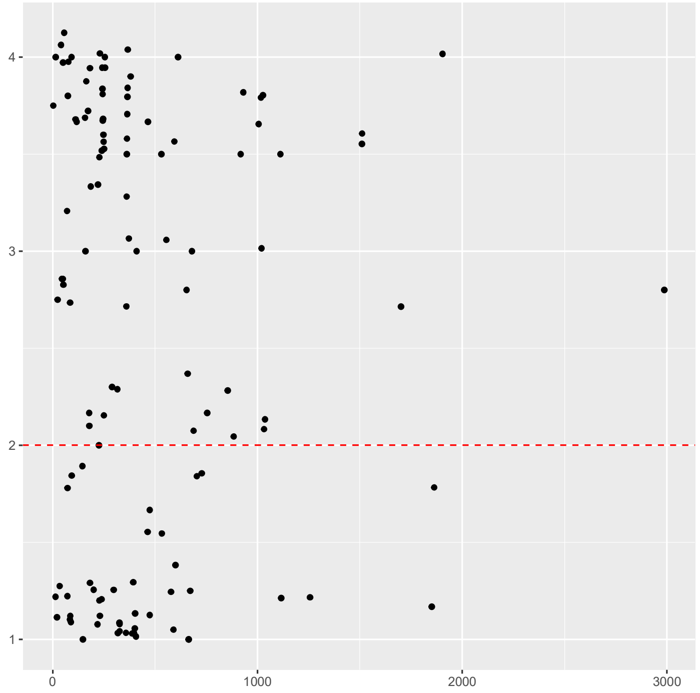
1
2
3
4
0 1000 2000 3000

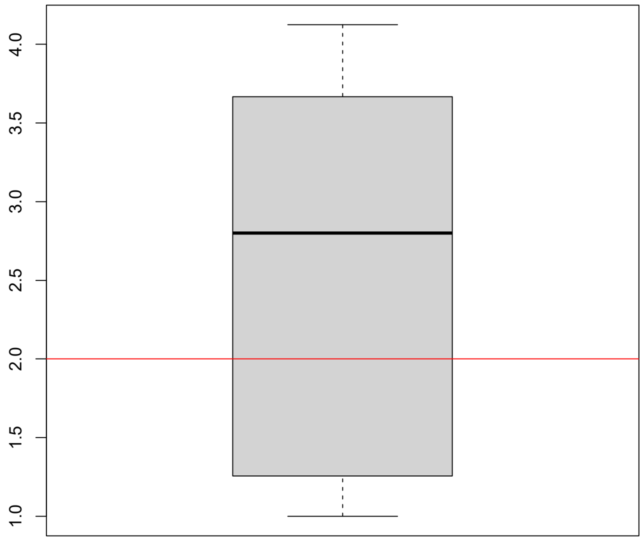
1.0
1.5
2.0
2.5
3.0
3.5
4.0

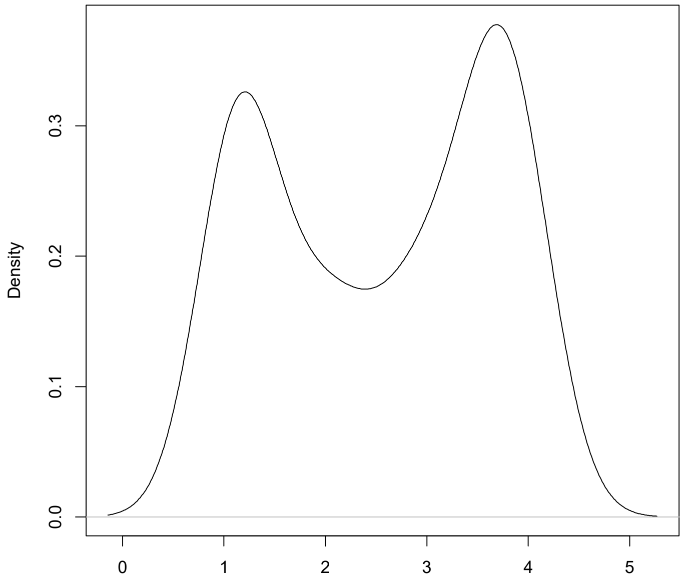
Density
0.0
0.1
0.2
0.3
0 1 2 3 4 5

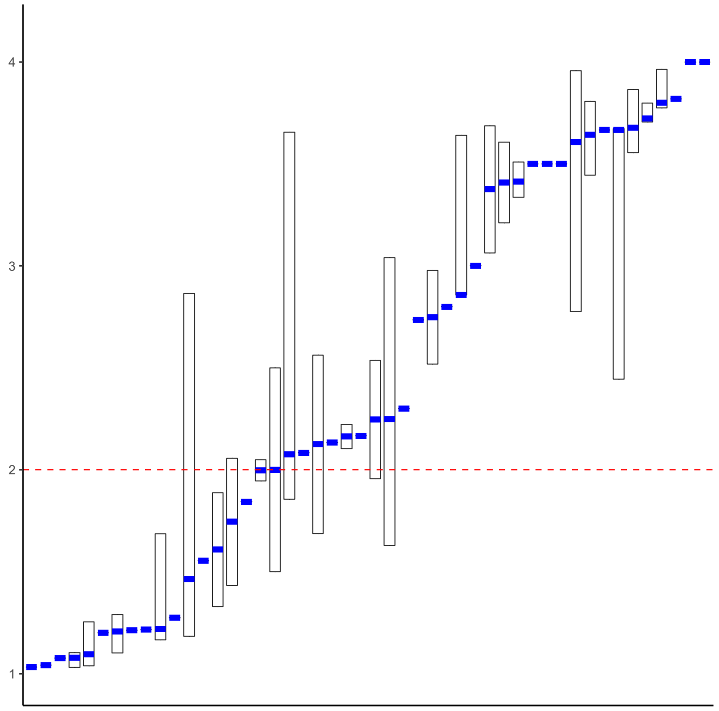
1
2
3
4

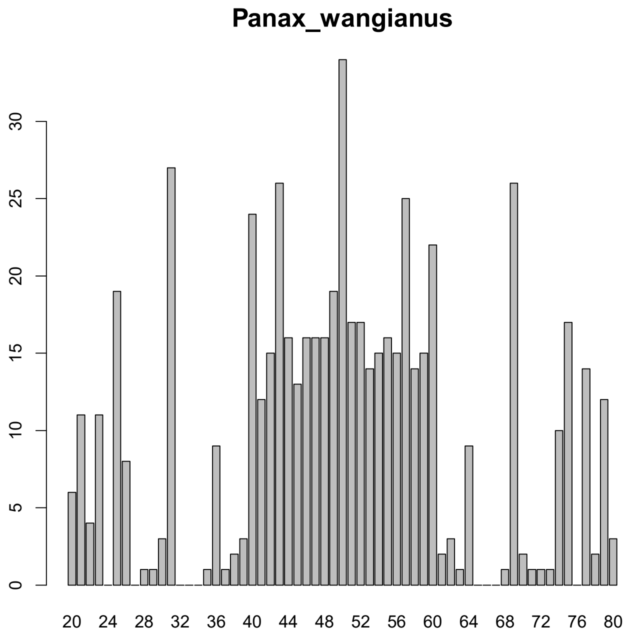
Panax_wangianus
0
5
10
15
20
25
30
20 24 28 32 36 40 44 48 52 56 60 64 68 72 76 80

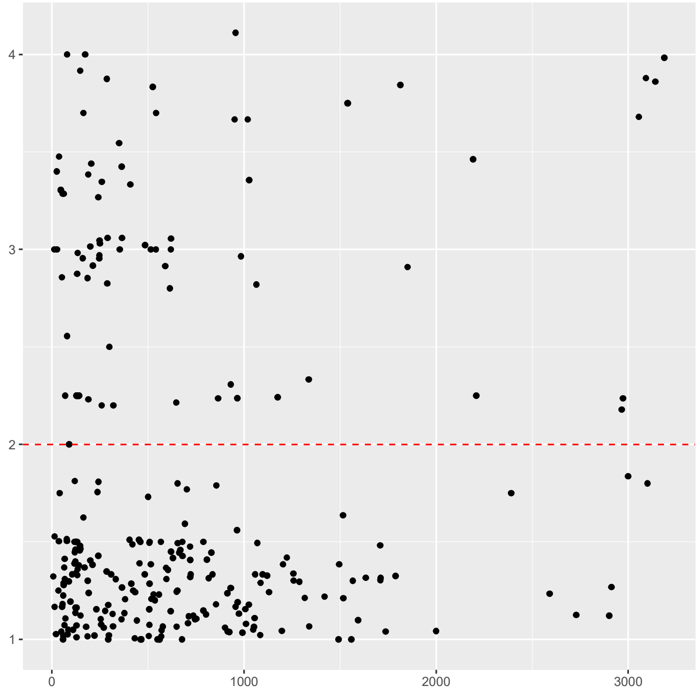
1
2
3
4
0 1000 2000 3000

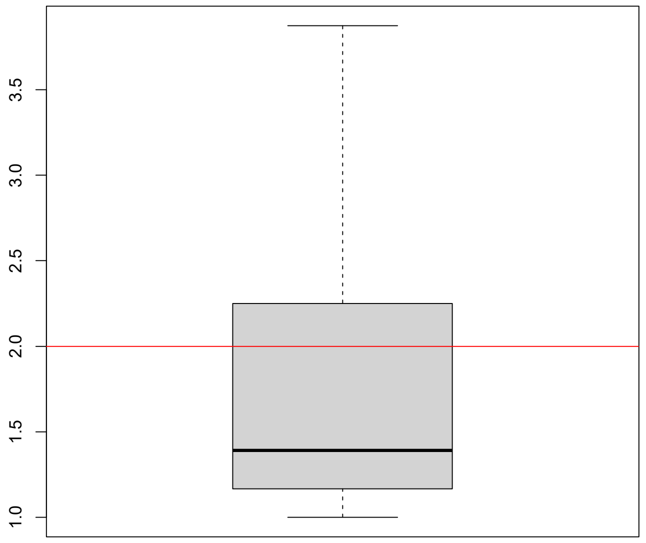
1.0
1.5
2.0
2.5
3.0
3.5

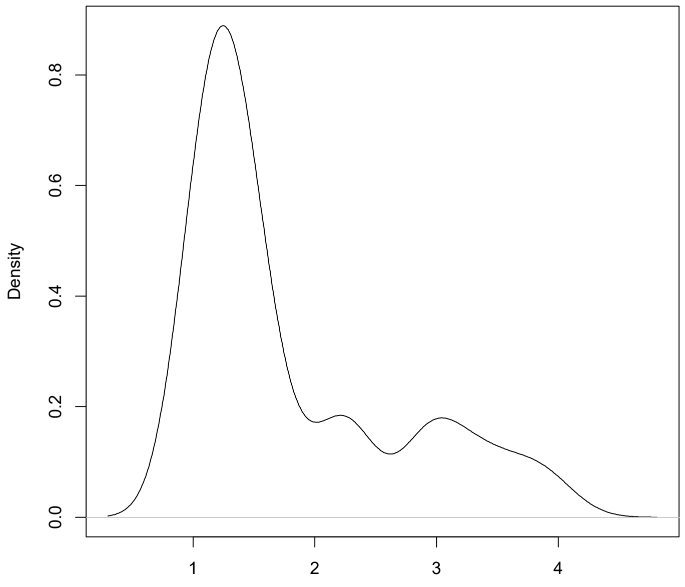
Density
0.0
0.2
0.4
0.6
0.8
1 2 3 4

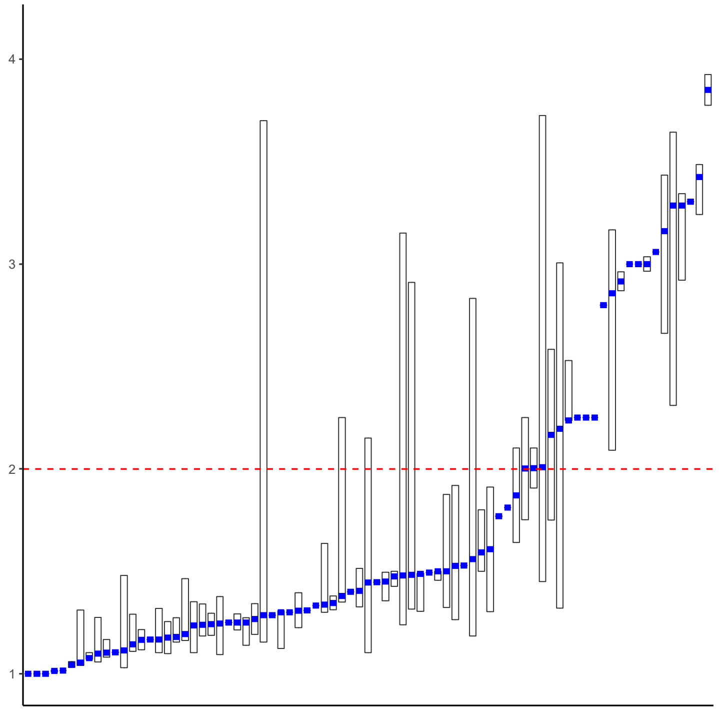
1
2
3
4

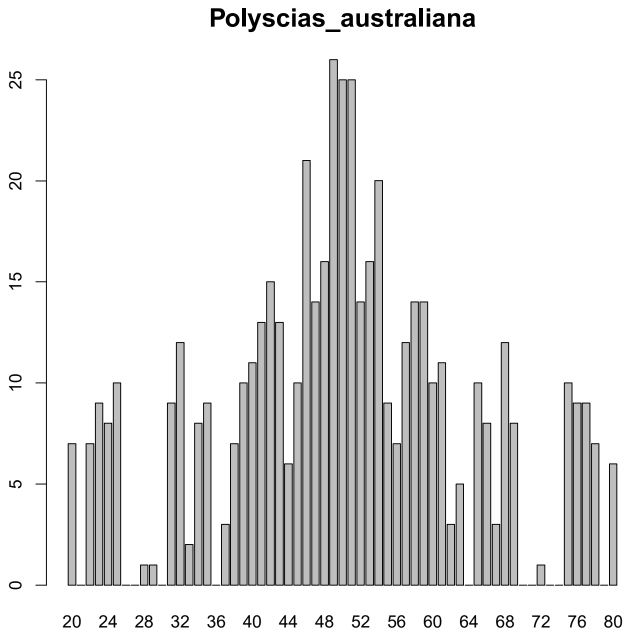
Polyscias_australiana
0
5
10
15
20
25
20 24 28 32 36 40 44 48 52 56 60 64 68 72 76 80

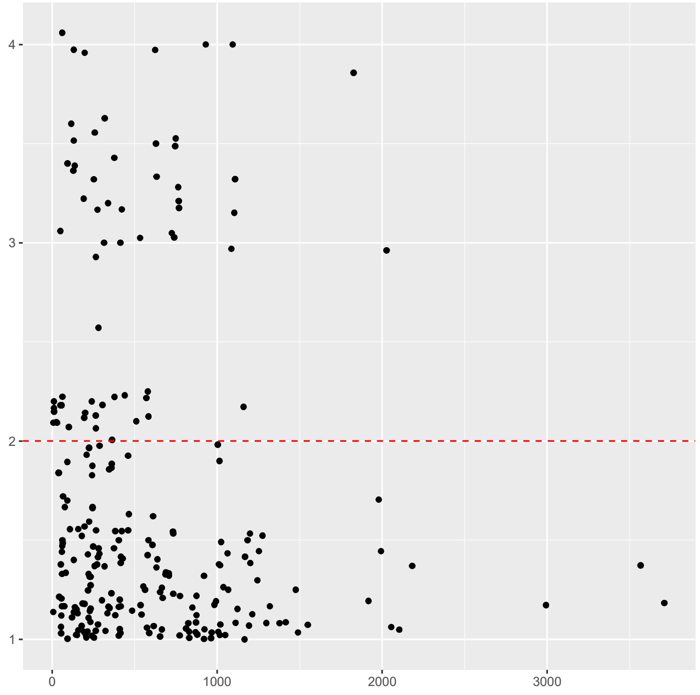
1
2
3
4
0 1000 2000 3000

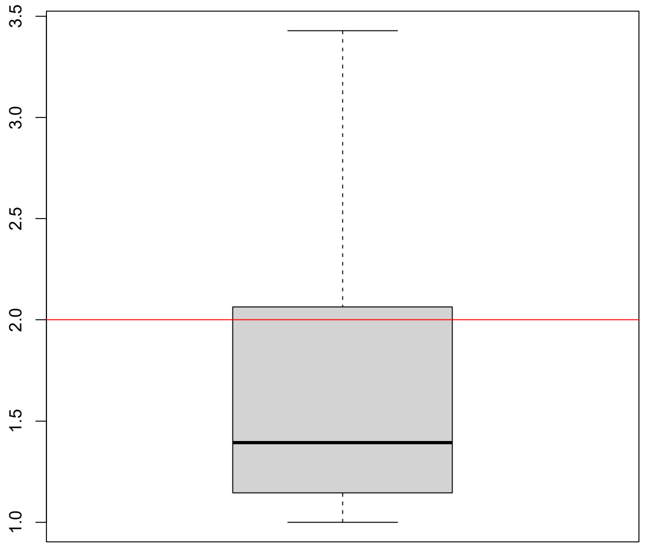
1.0
1.5
2.0
2.5
3.0
3.5

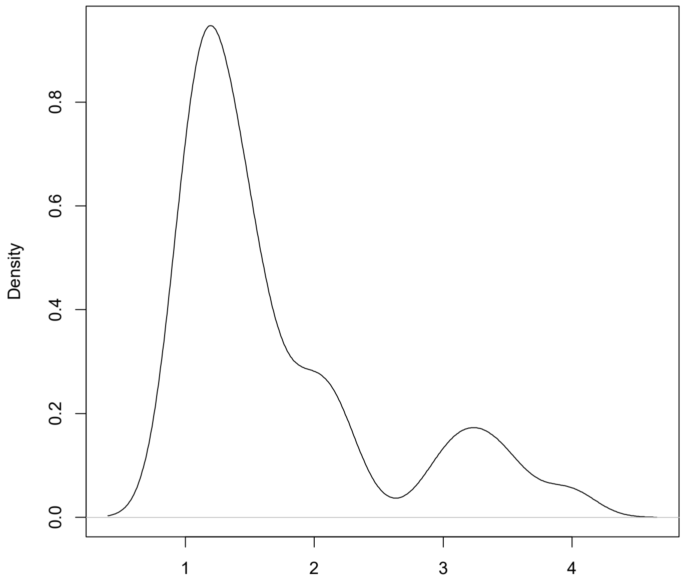
Density
0.0
0.2
0.4
0.6
0.8
1 2 3 4

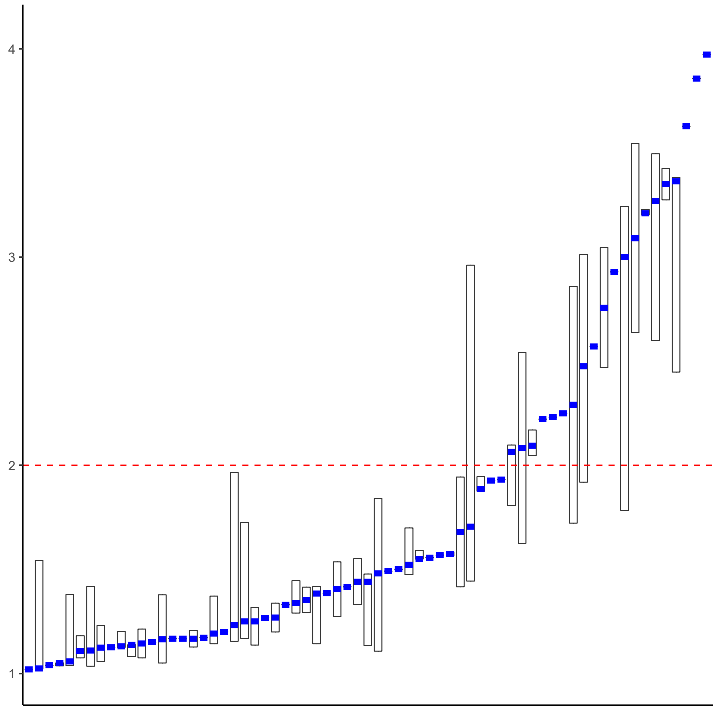
1
2
3
4

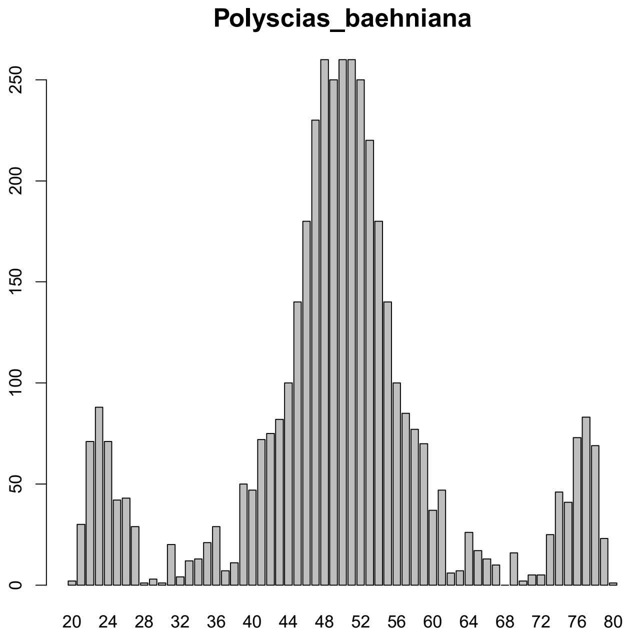
Polyscias_baehniana
0
50
100
150
200
250
20
24
28
32
36
40
44
48
52
56
60
64
68
72
76
80

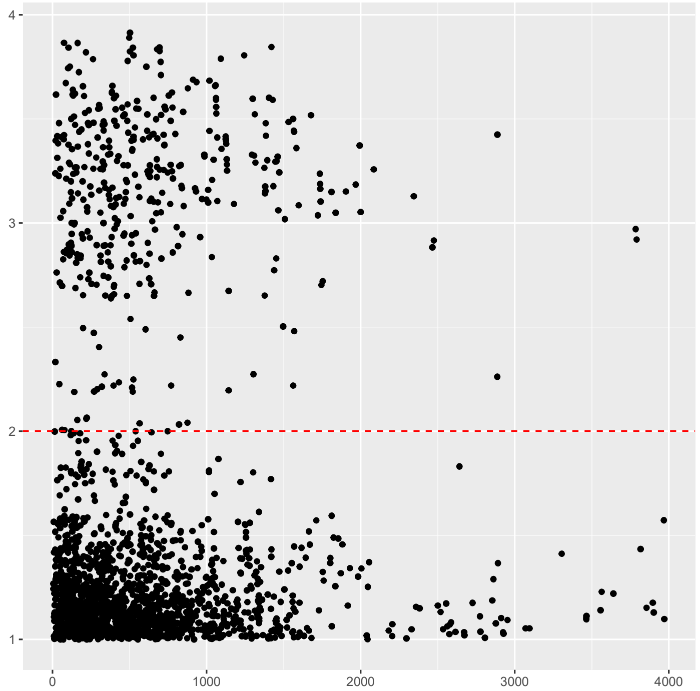
1
2
3
4
0
1000
2000
3000
4000

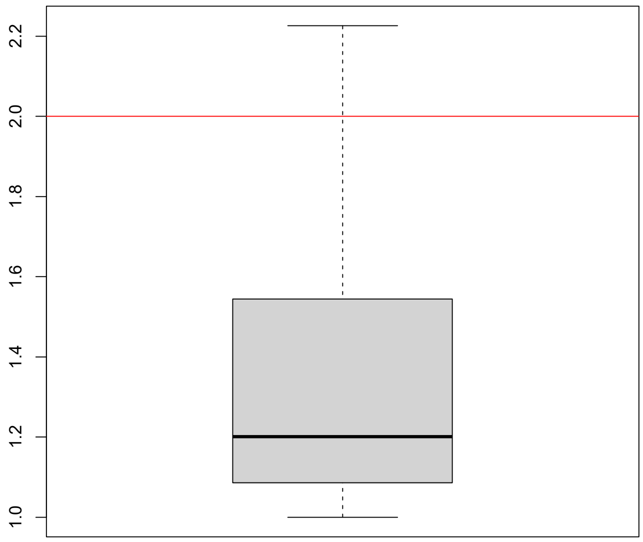
1.0
1.2
1.4
1.6
1.8
2.0
2.2

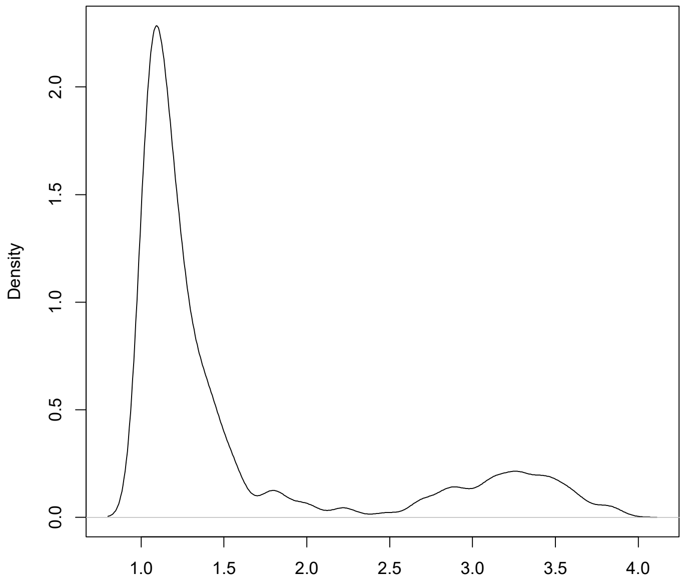
Density
0.0
0.5
1.0
1.5
2.0
1.0
1.5
2.0
2.5
3.0
3.5
4.0

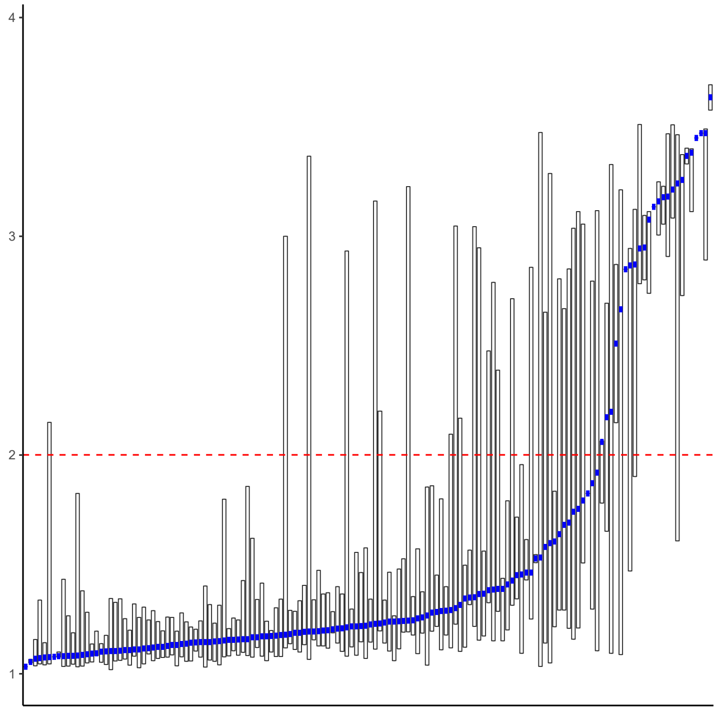
1
2
3
4

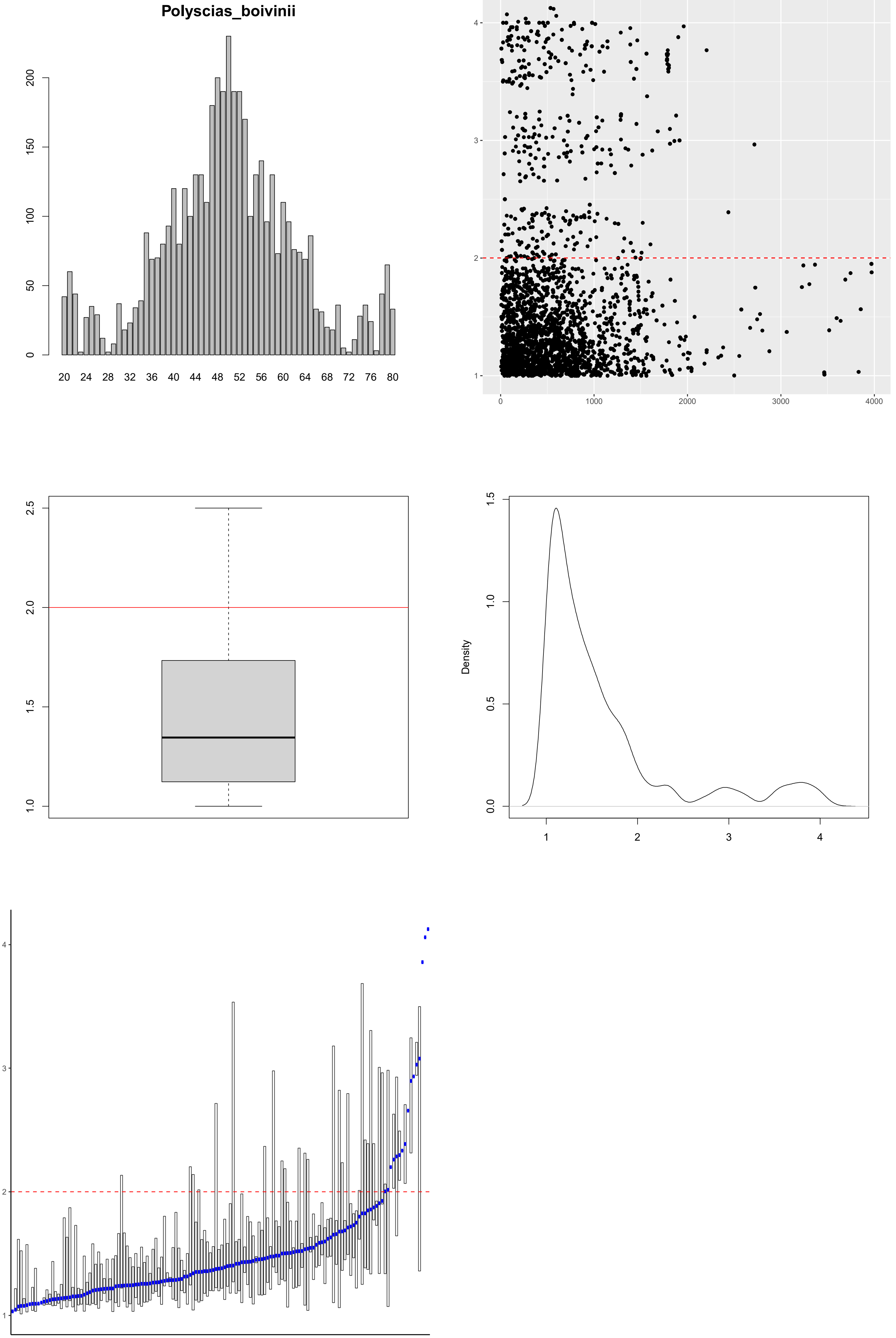
Polyscias_boivinii
Density

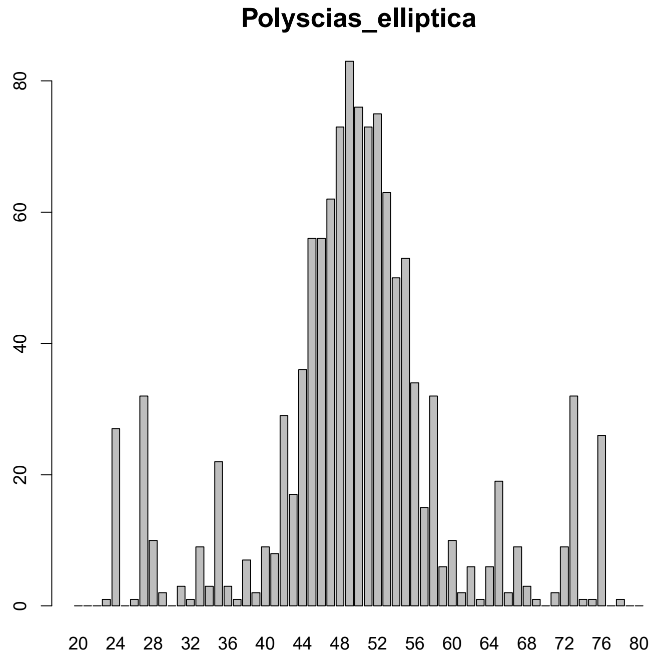
Polyscias_elliptica

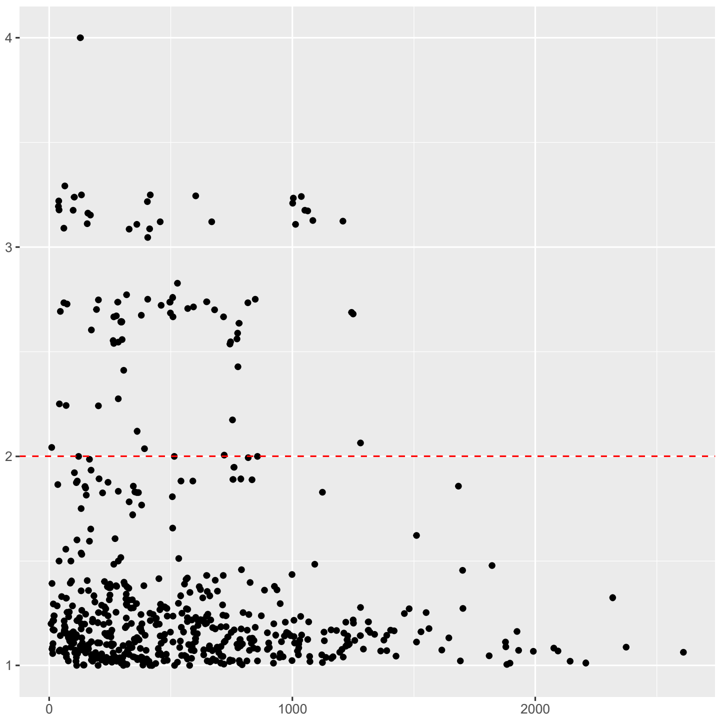

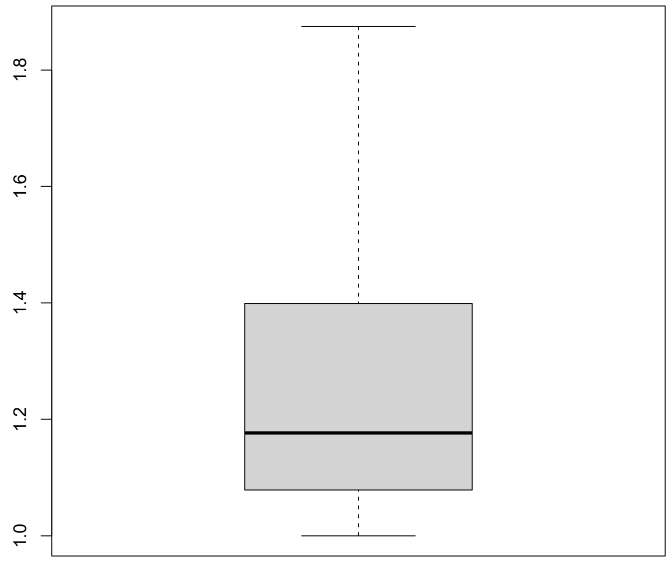

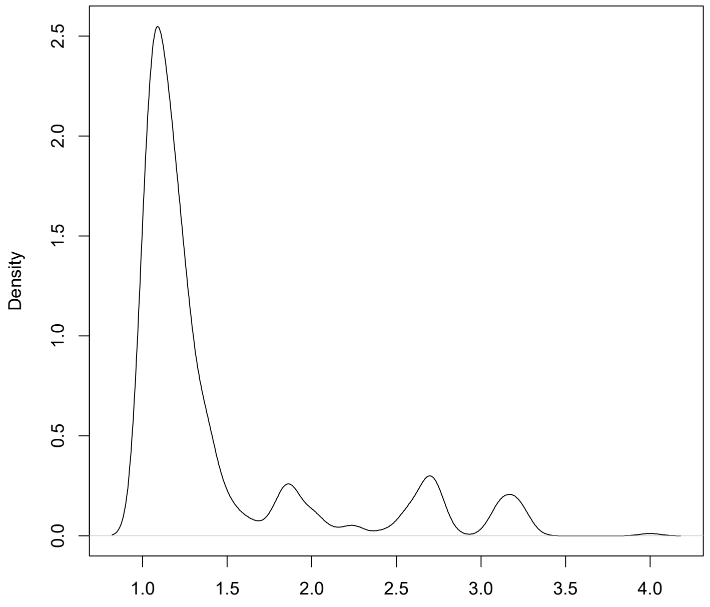
Density

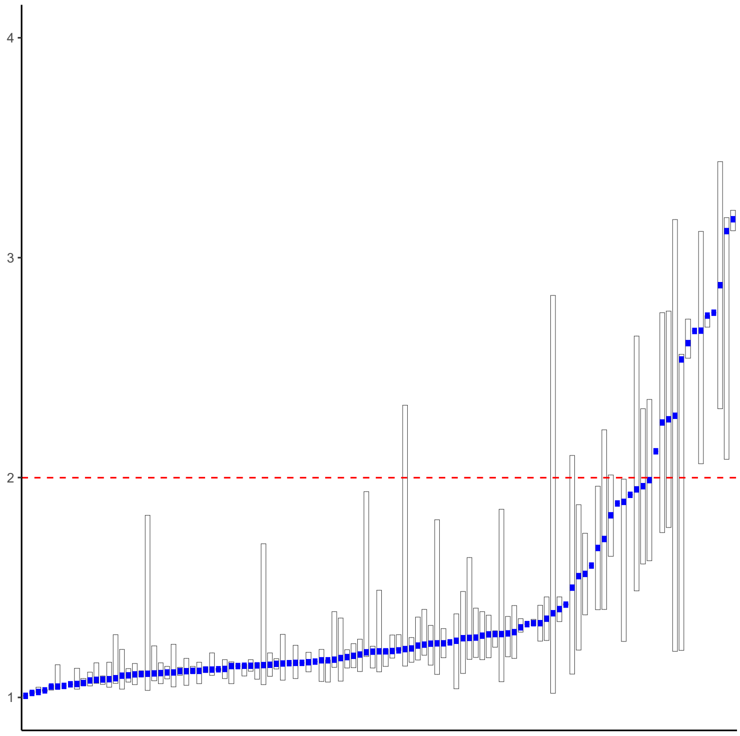

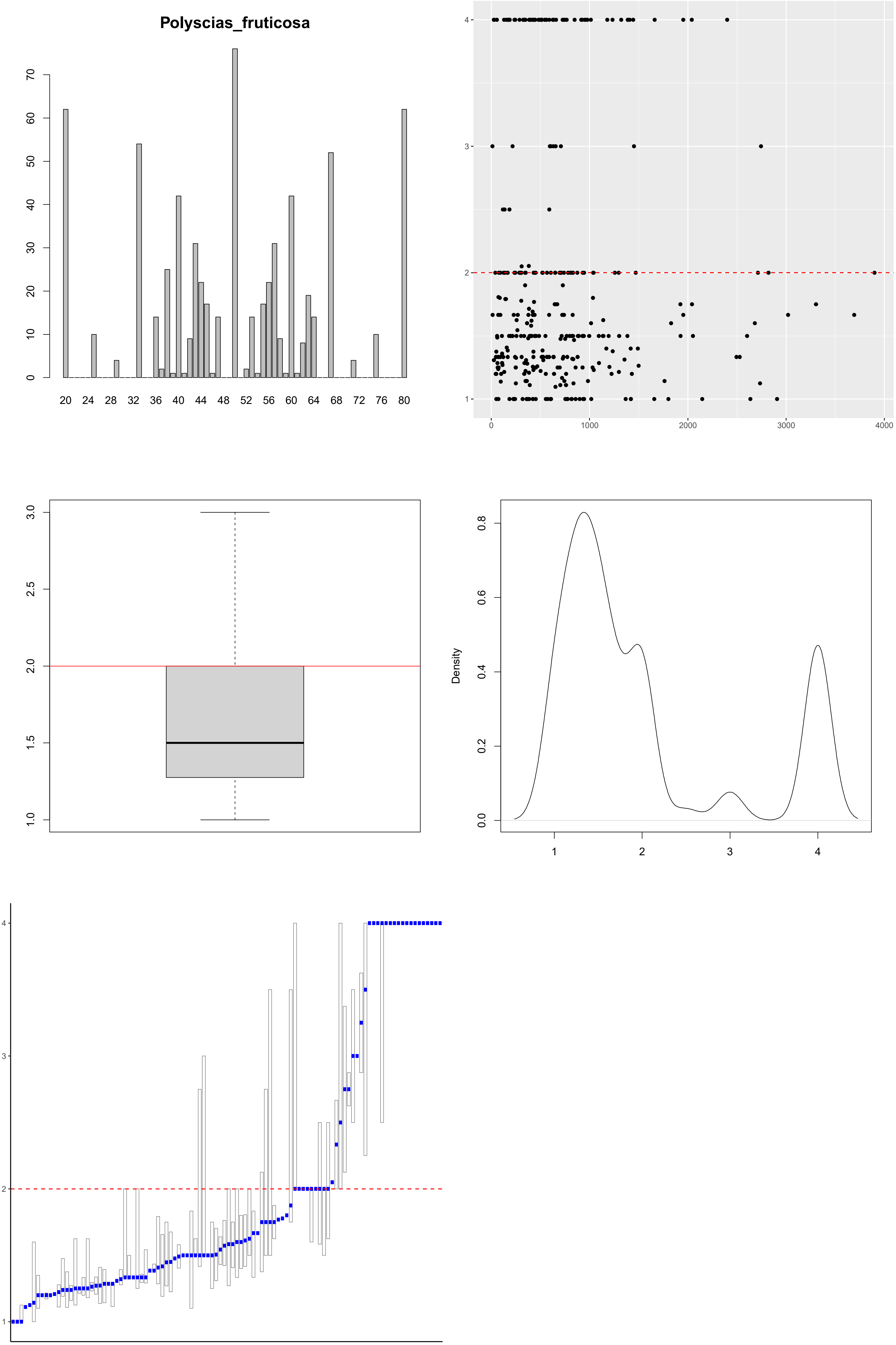

Polyscias_fruticosa
Density

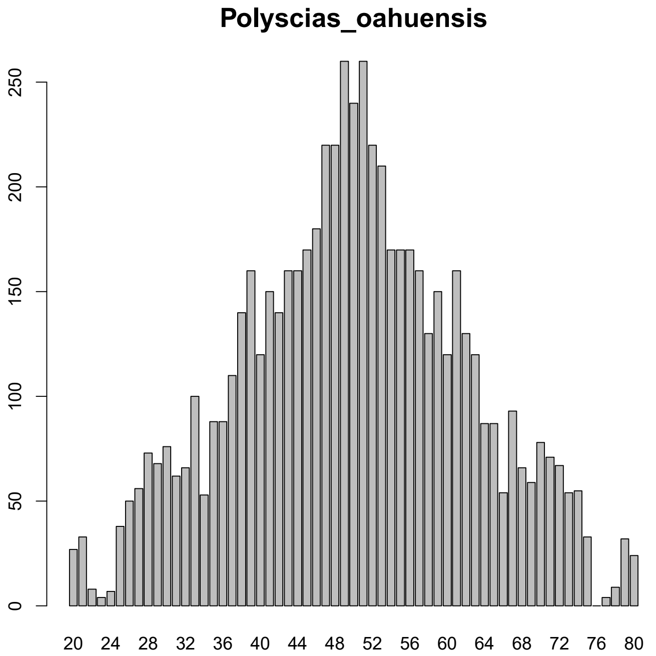
Polyscias_oahuensis

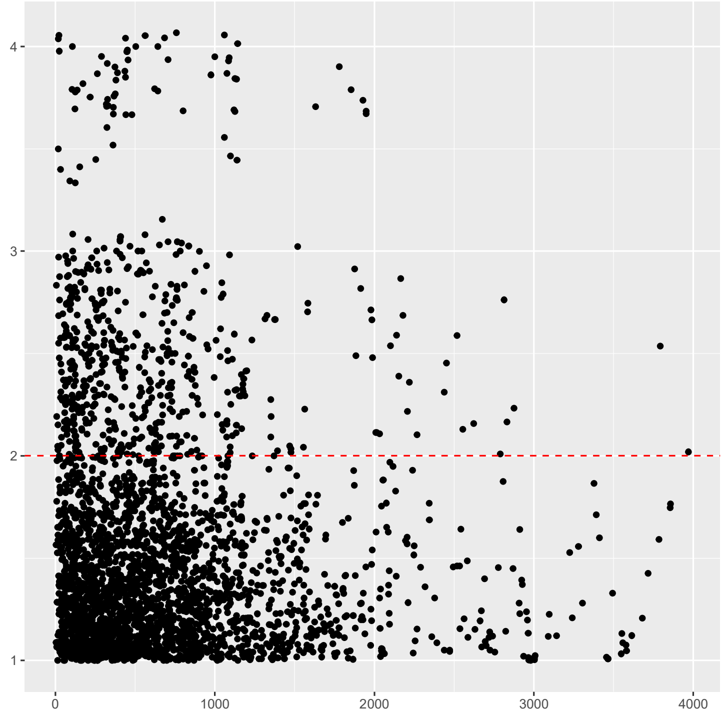

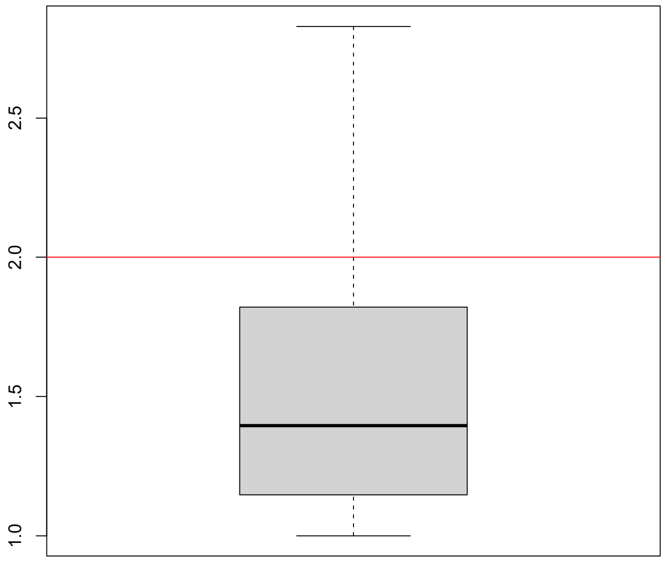

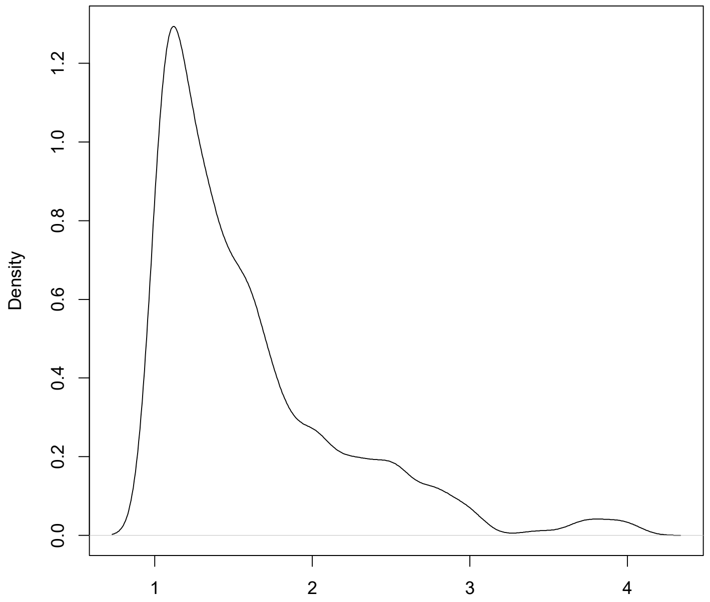
Density

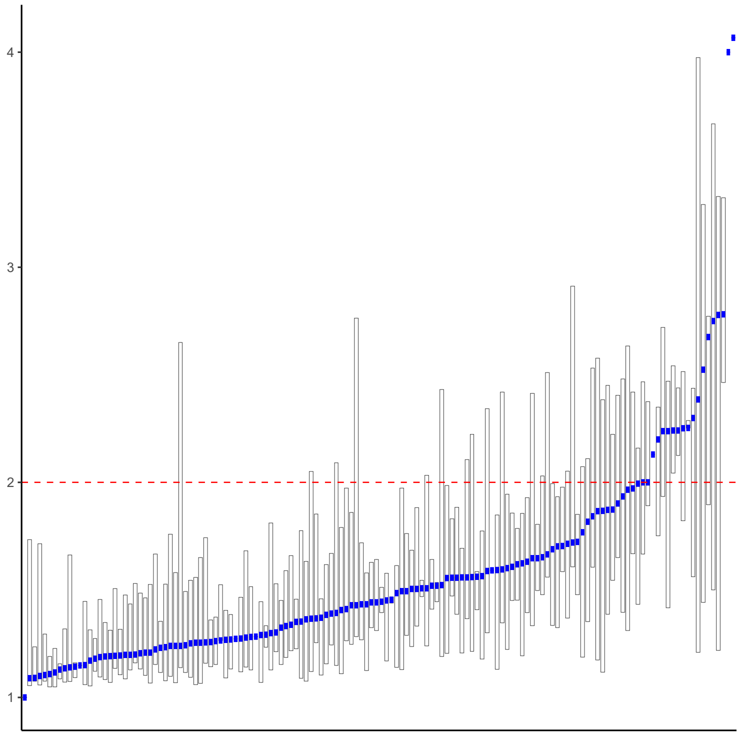

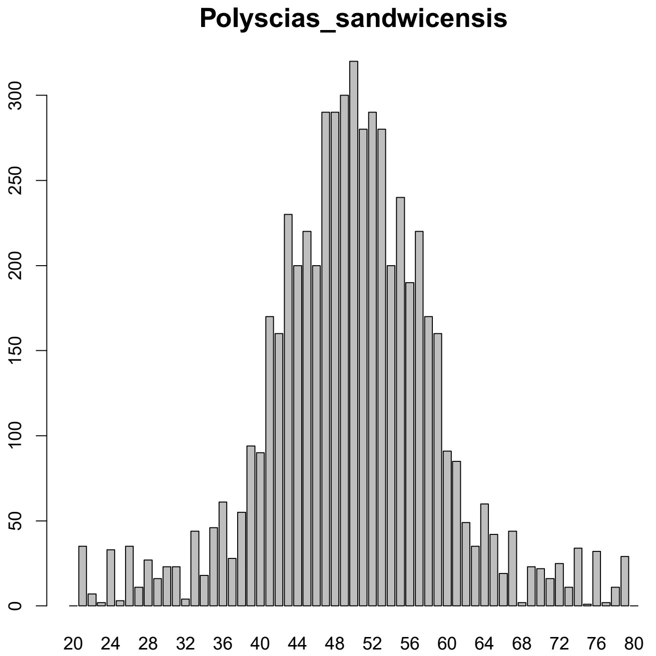
Polyscias_sandwicensis
0
50
100
150
200
250
300
20 24 28 32 36 40 44 48 52 56 60 64 68 72 76 80

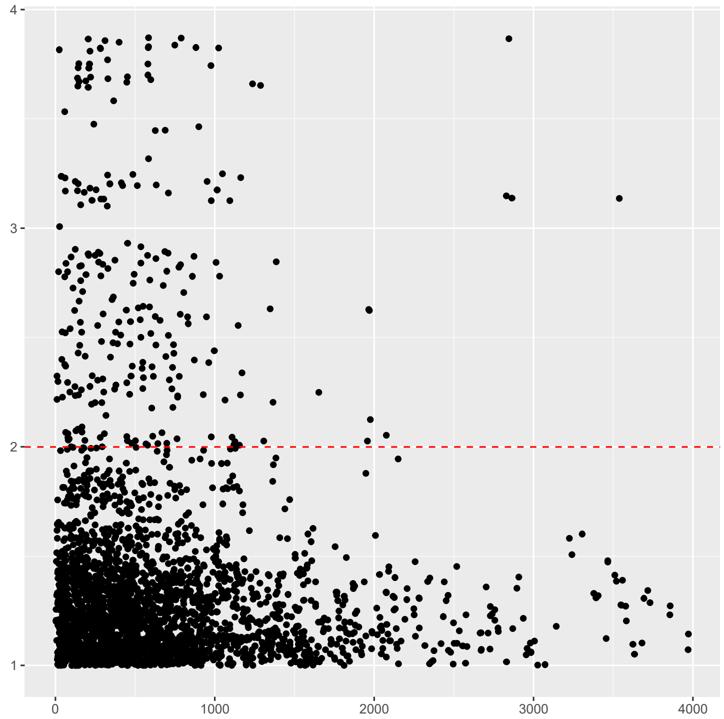
1
2
3
4
0
1000
2000
3000
4000

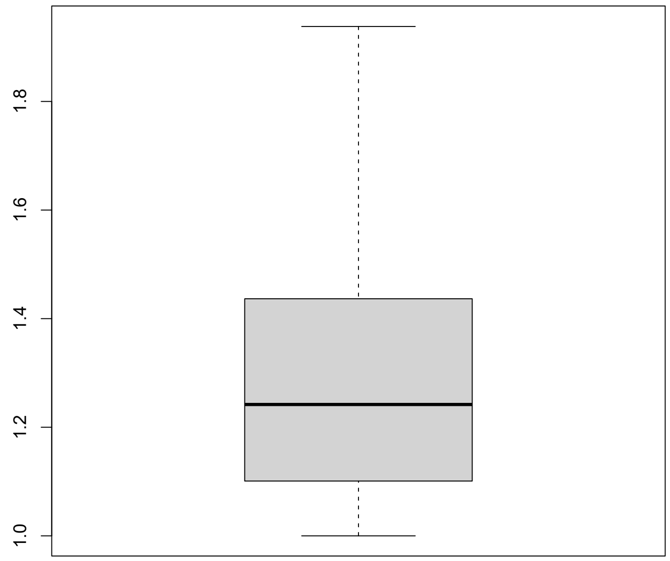
1.0
1.2
1.4
1.6
1.8

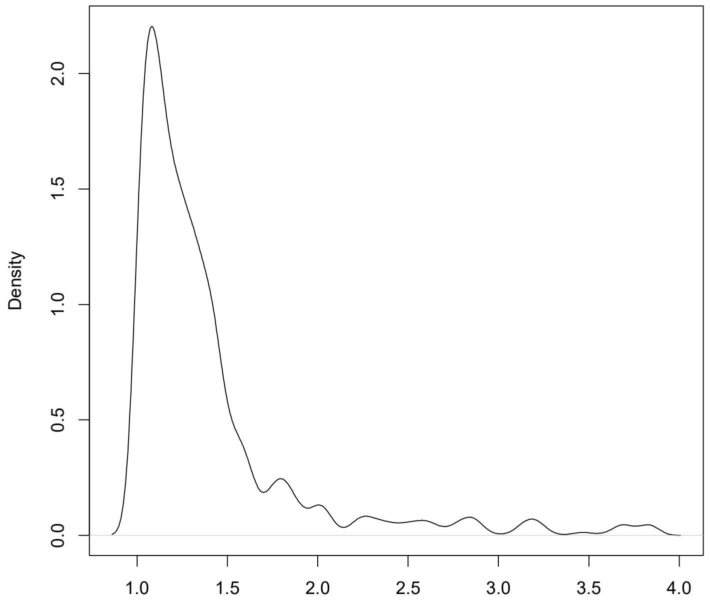
Density
0.0
0.5
1.0
1.5
2.0
1.0 1.5 2.0 2.5 3.0 3.5 4.0

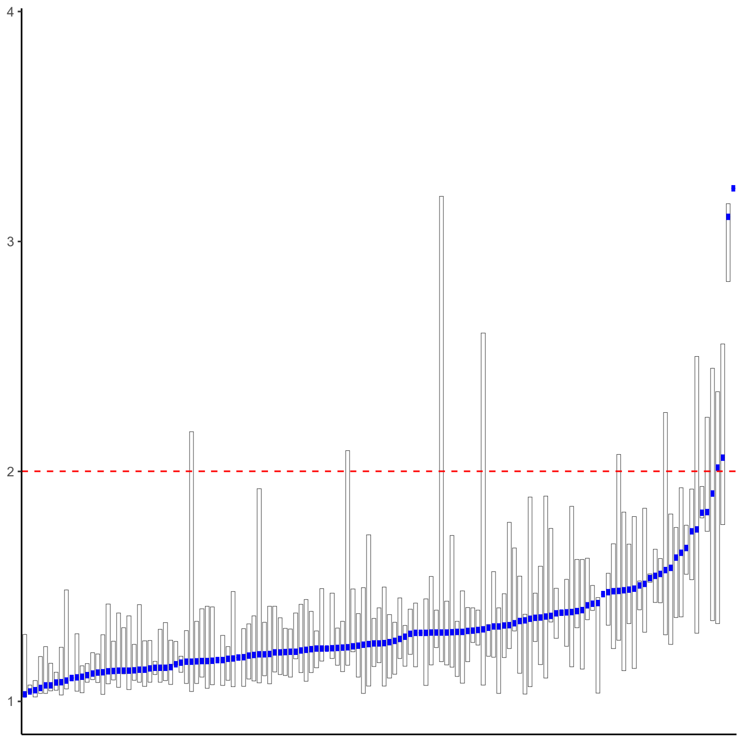
1
2
3
4

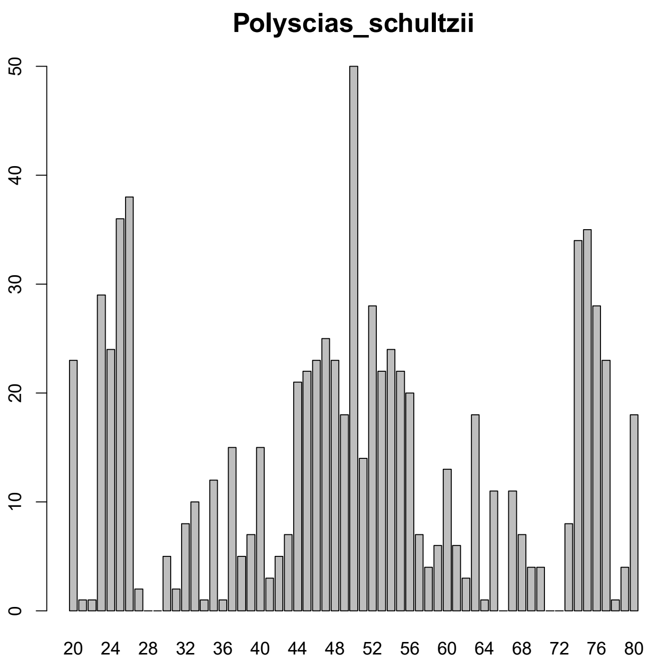
Polyscias_schultzii
0
10
20
30
40
50
20 24 28 32 36 40 44 48 52 56 60 64 68 72 76 80

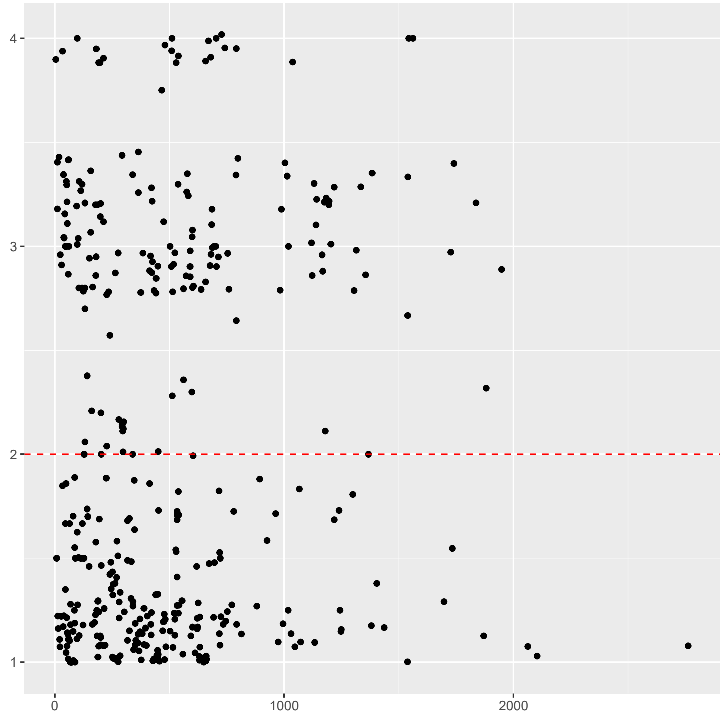
1
2
3
4
0
1000
2000

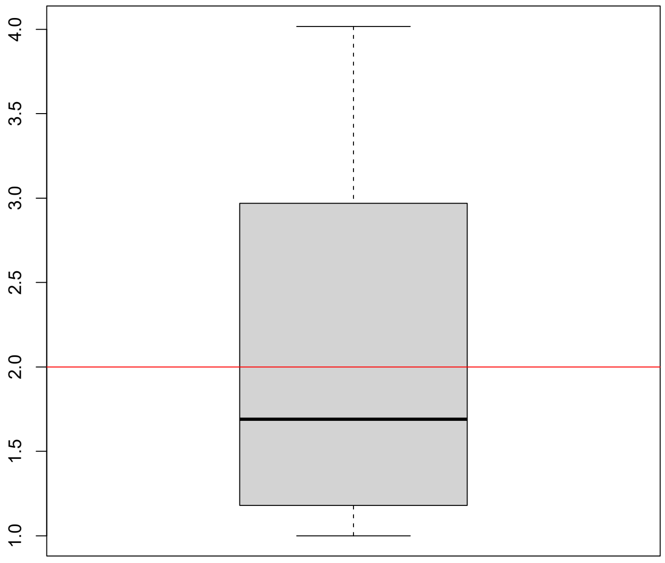
1.0
1.5
2.0
2.5
3.0
3.5
4.0

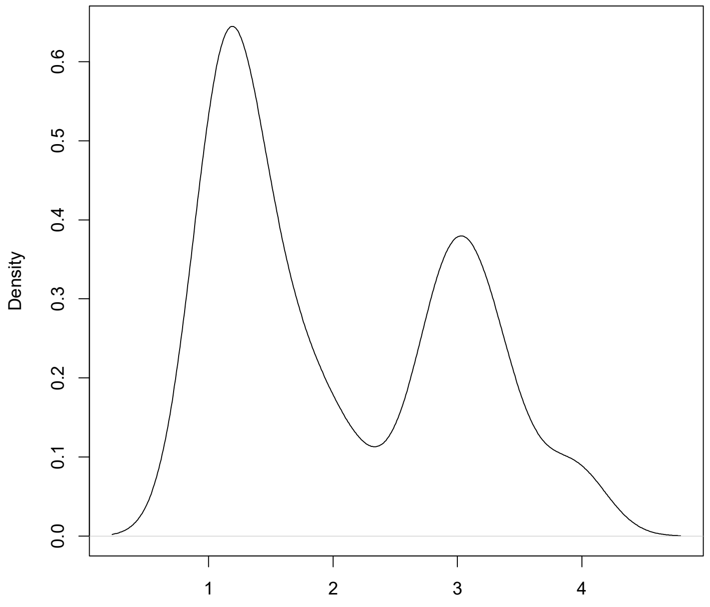
Density
0.0
0.1
0.2
0.3
0.4
0.5
0.6
1
2
3
4

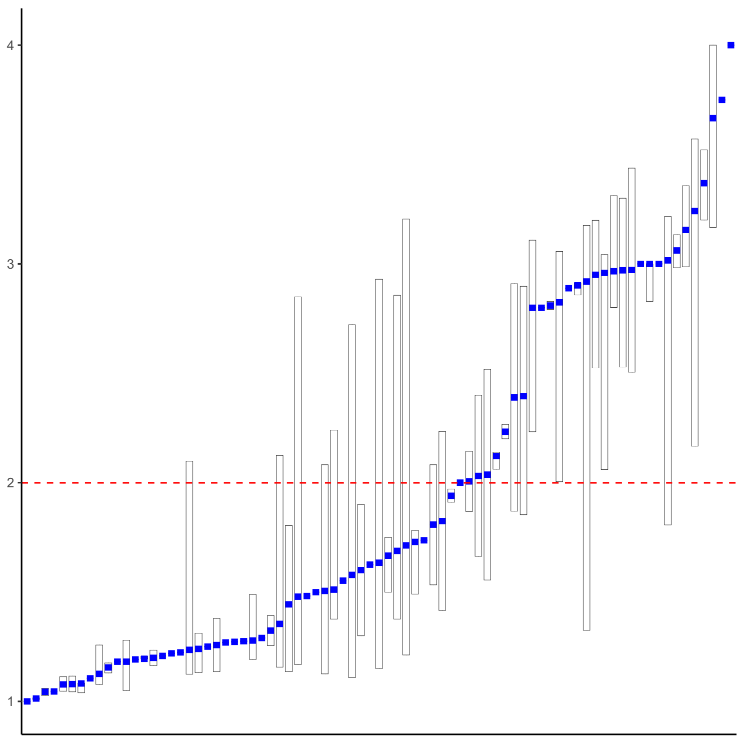
1
2
3
4

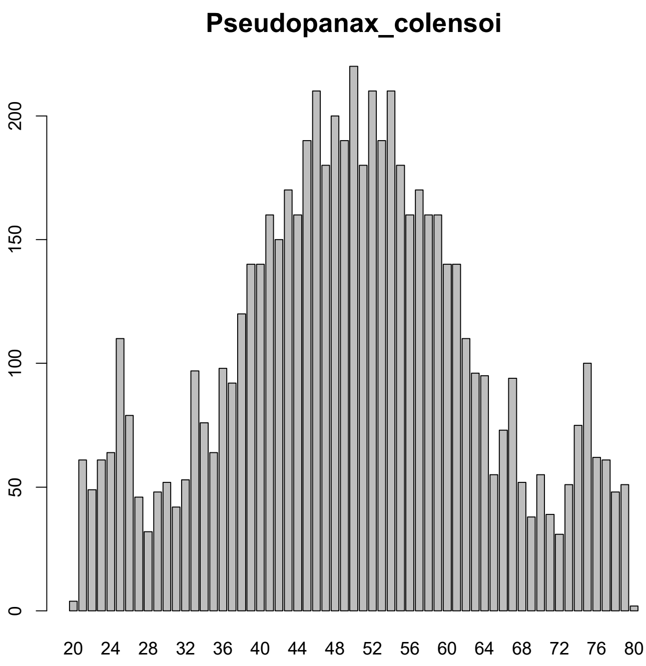
Pseudopanax_colensoi
0
50
100
150
200
20 24 28 32 36 40 44 48 52 56 60 64 68 72 76 80

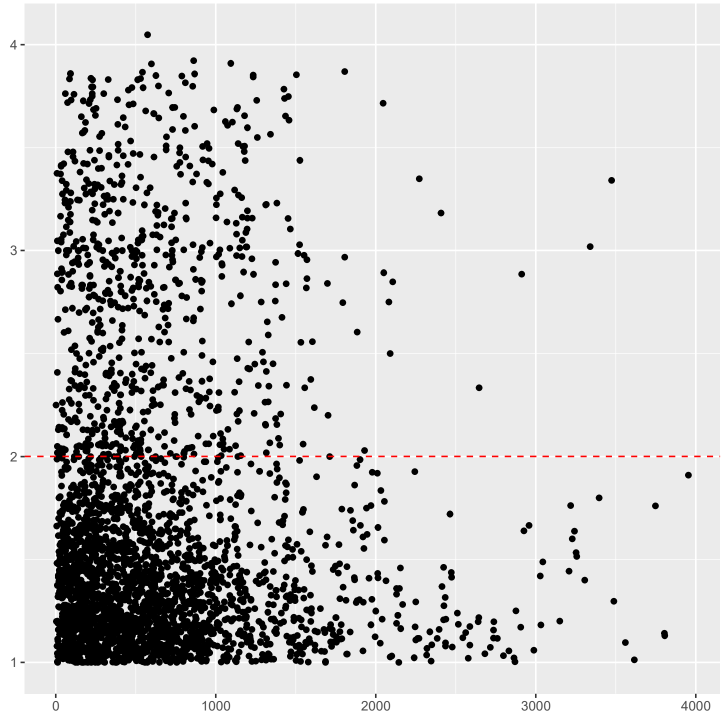
1
2
3
4
0 1000 2000 3000 4000

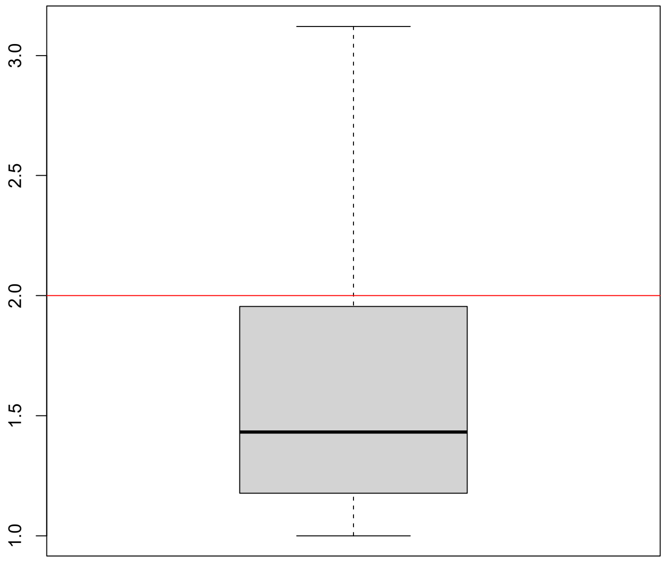
1.0
1.5
2.0
2.5
3.0

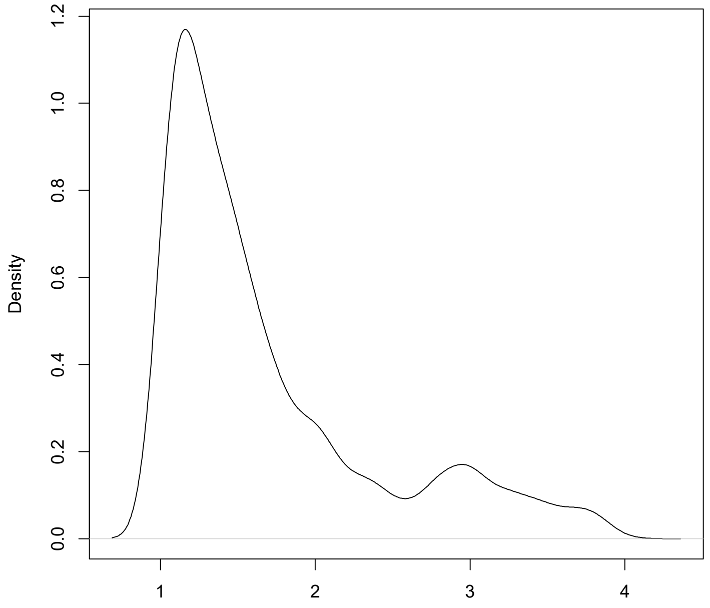
Density
0.0
0.2
0.4
0.6
0.8
1.0
1.2
1 2 3 4

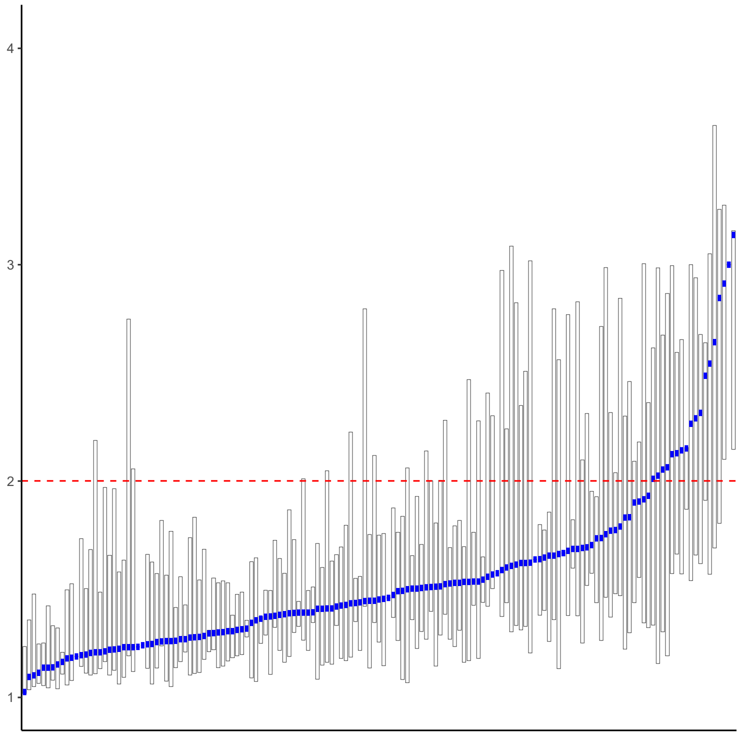
1
2
3
4

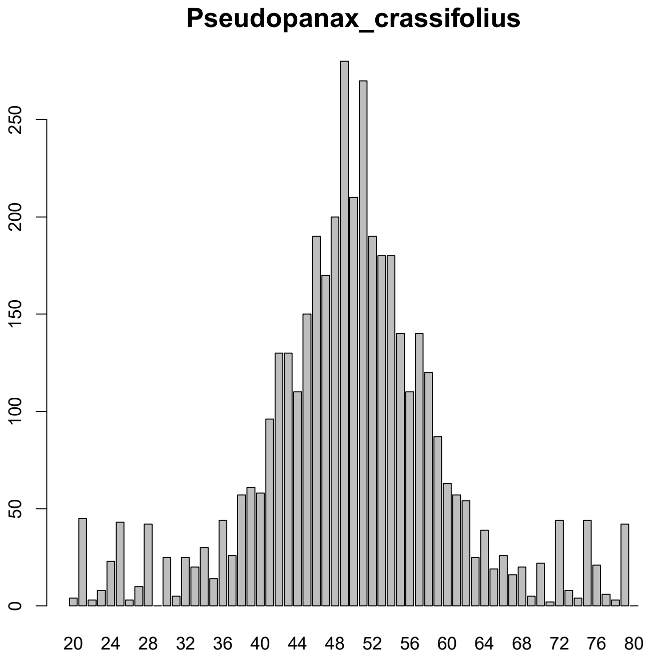
Pseudopanax_crassifolius
0
50
100
150
200
250
20 24 28 32 36 40 44 48 52 56 60 64 68 72 76 80

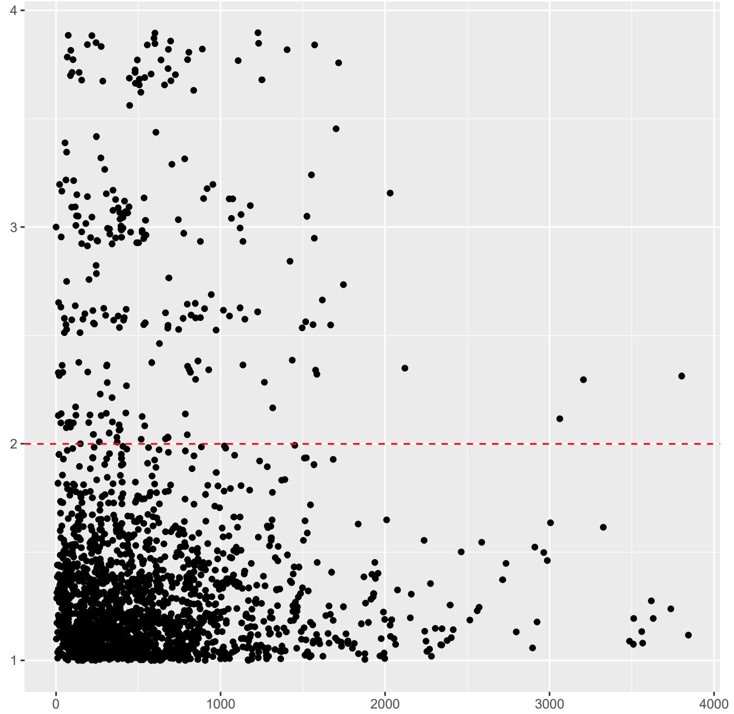
1
2
3
4
0 1000 2000 3000 4000

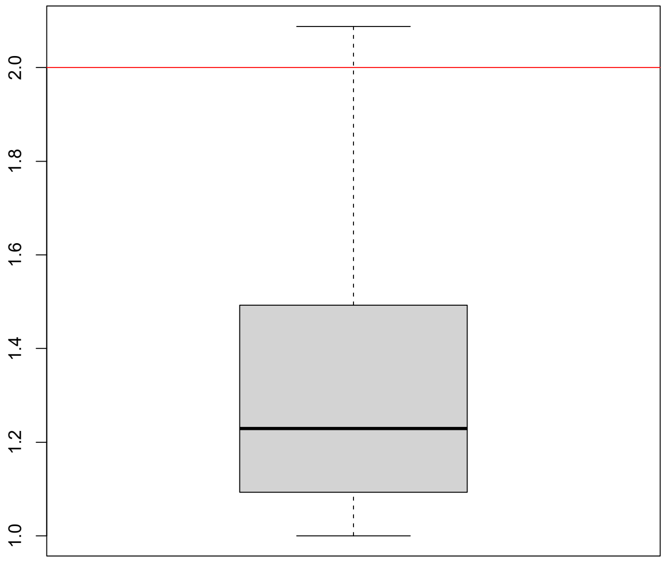
1.0
1.2
1.4
1.6
1.8
2.0

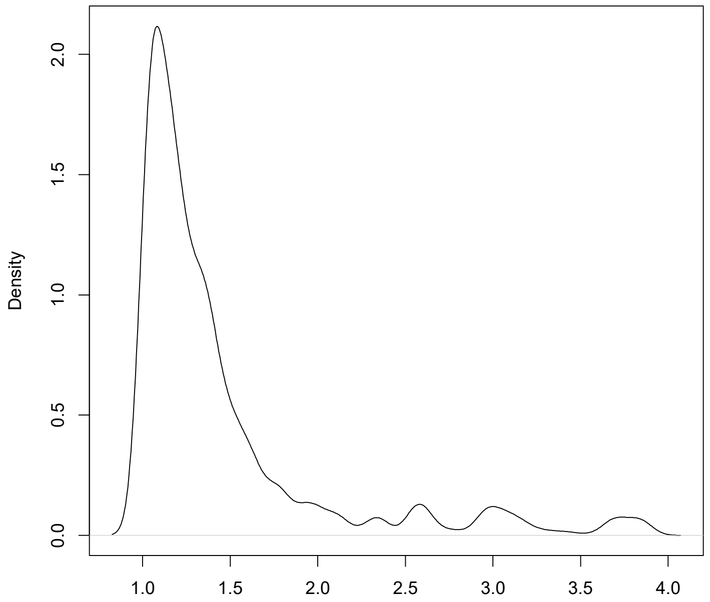
Density
0.0
0.5
1.0
1.5
2.0
1.0 1.5 2.0 2.5 3.0 3.5 4.0

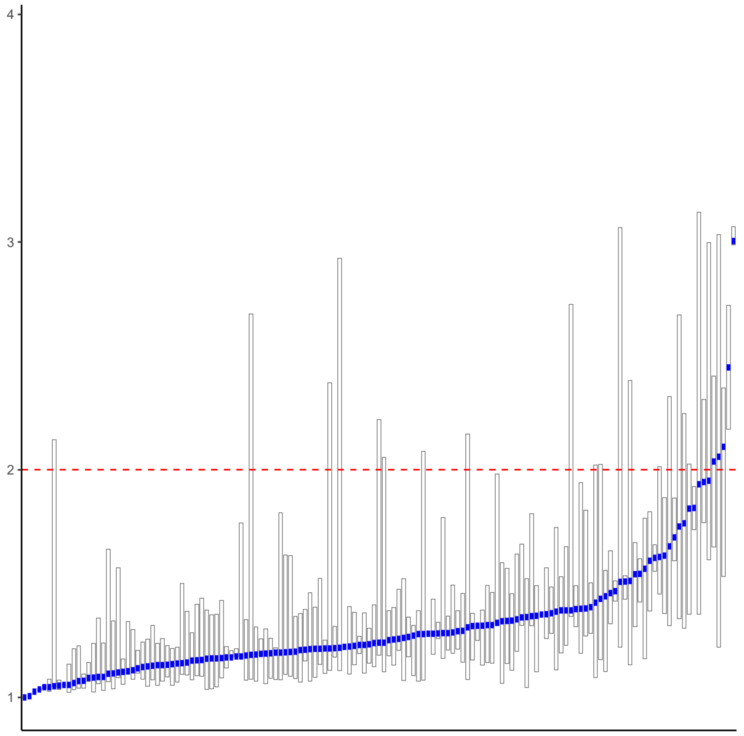
1
2
3
4

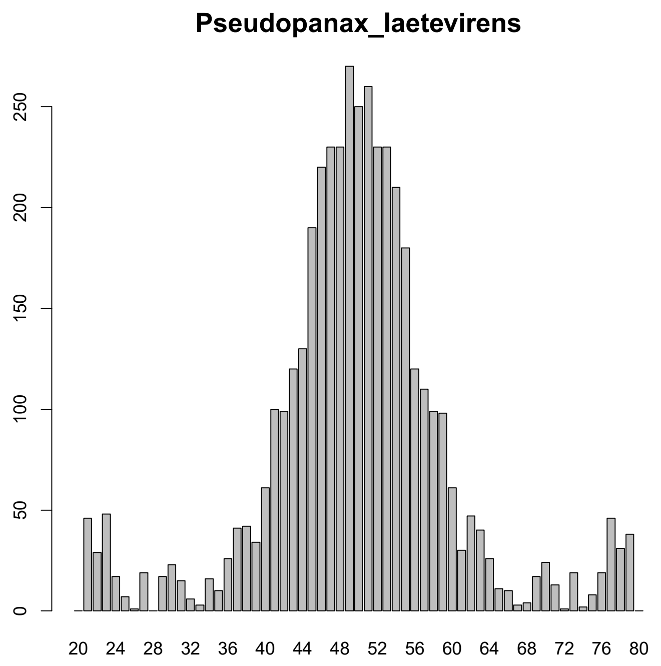
Pseudopanax_laetevirens

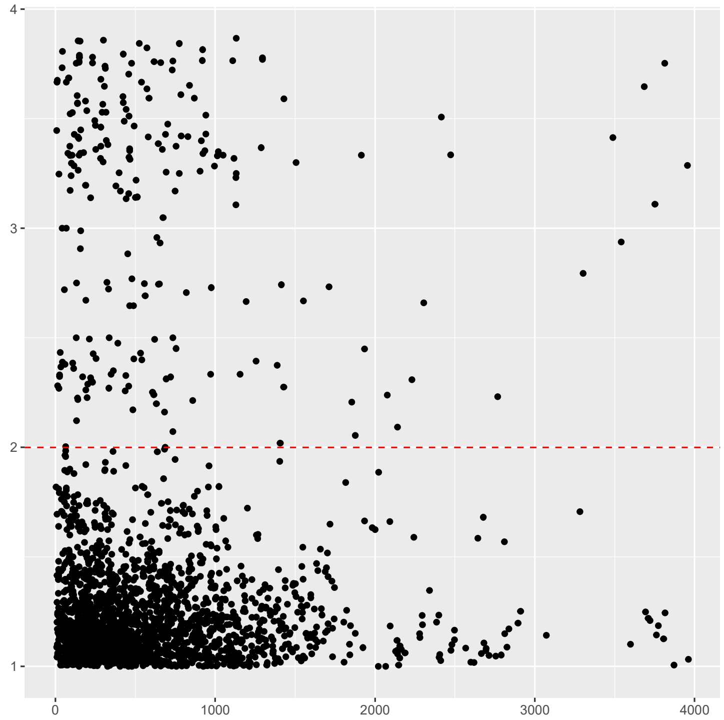

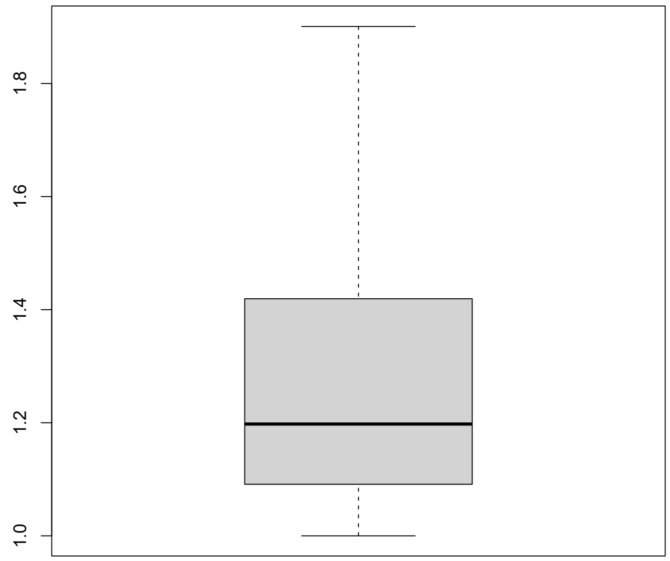

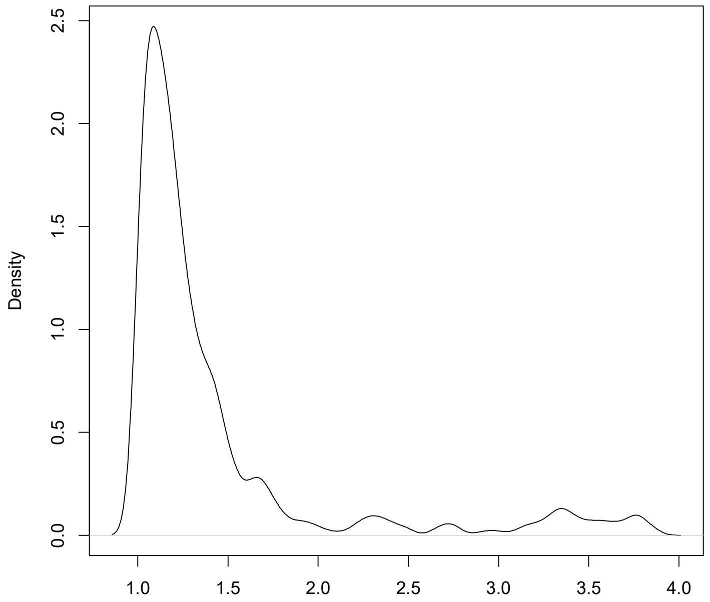
Density

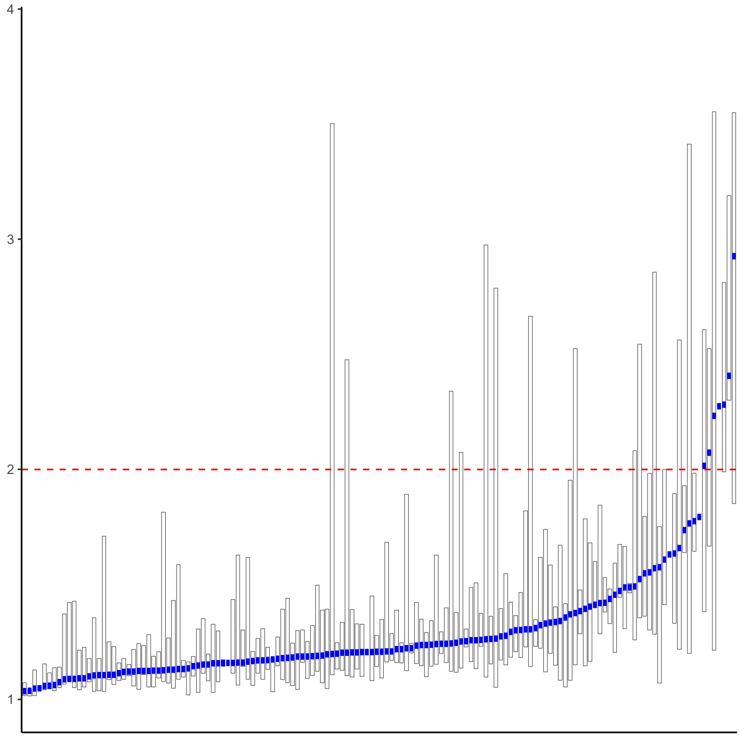

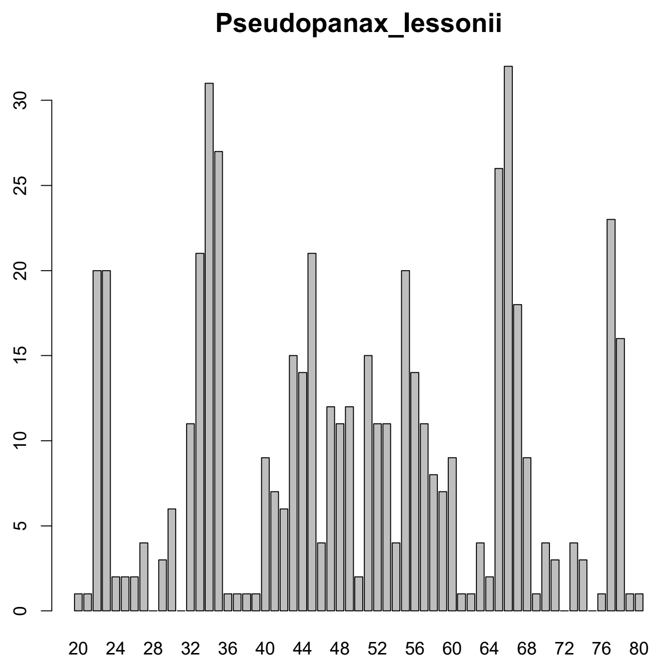
Pseudopanax_lessonii

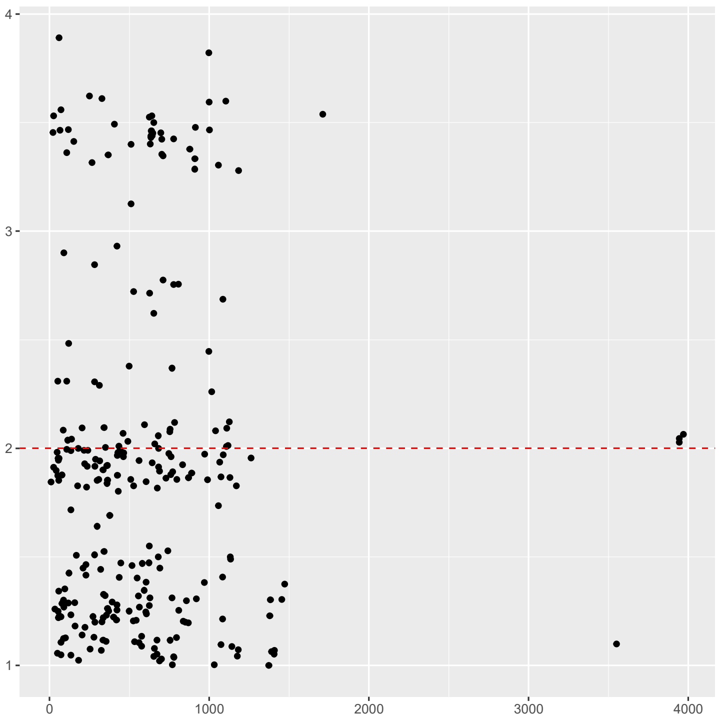

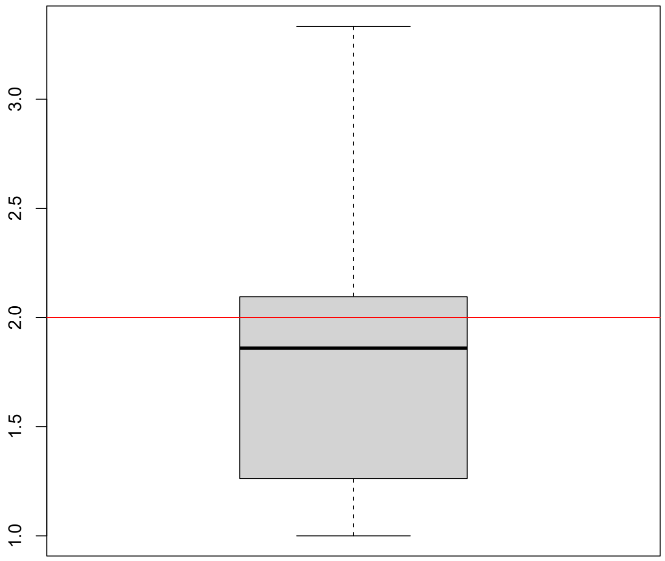

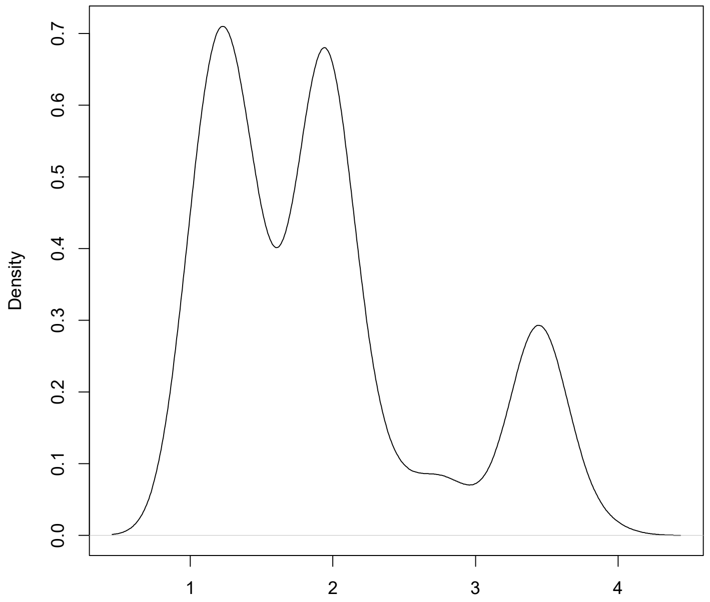
Density

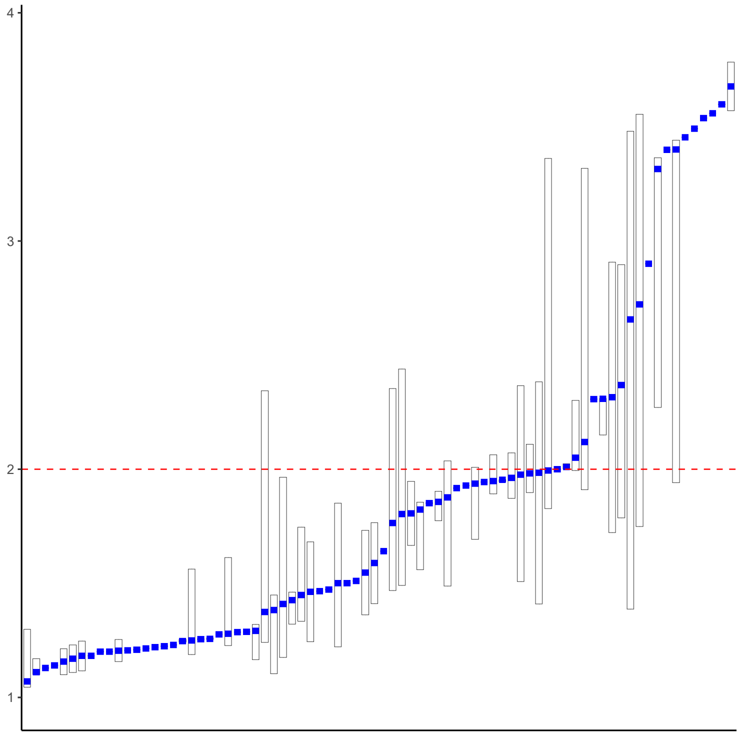

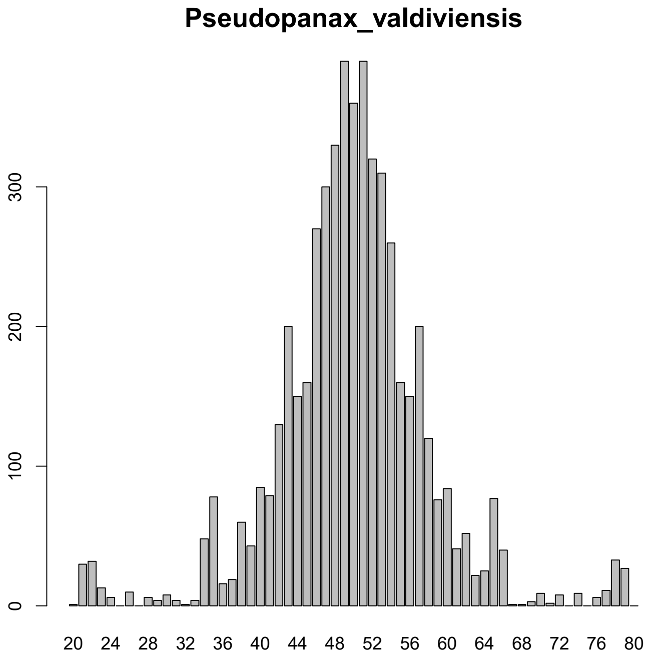
Pseudopanax_valdiviensis
0
100
200
300
20 24 28 32 36 40 44 48 52 56 60 64 68 72 76 80

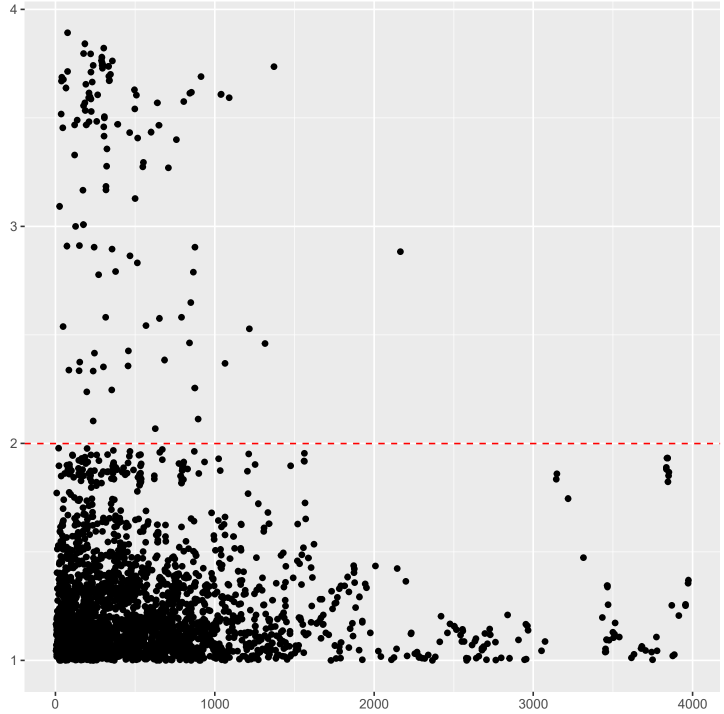
1
2
3
4
0 1000 2000 3000 4000

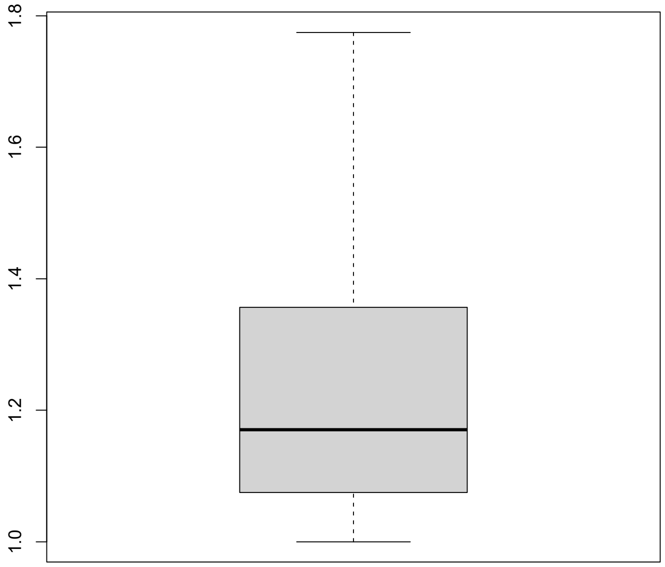
1.0
1.2
1.4
1.6
1.8

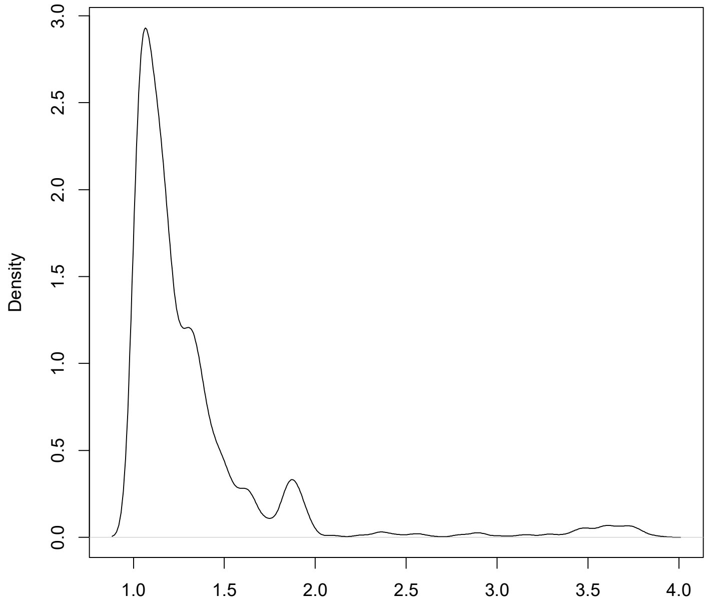
Density
0.0
0.5
1.0
1.5
2.0
2.5
3.0
1.0 1.5 2.0 2.5 3.0 3.5 4.0

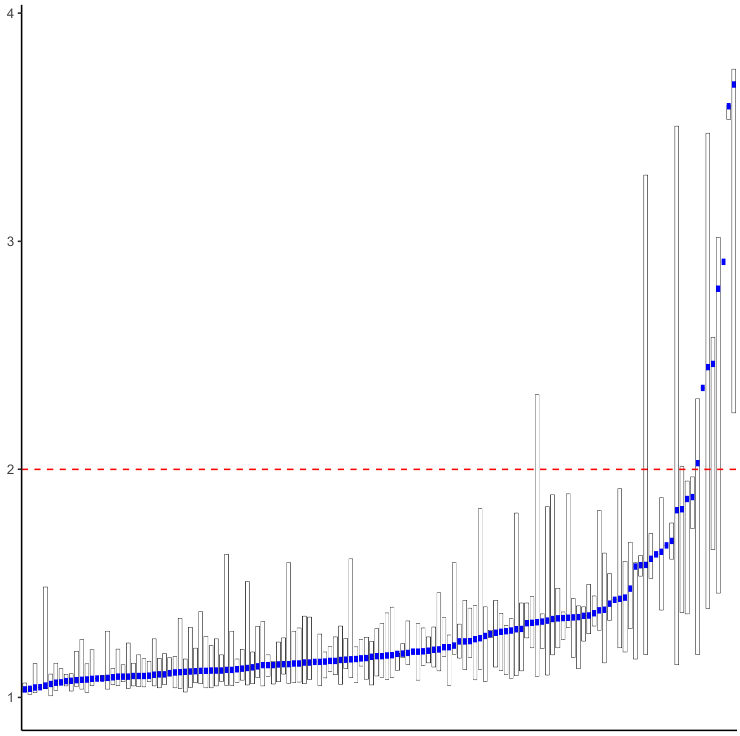
1
2
3
4

Raukaua_anomalus

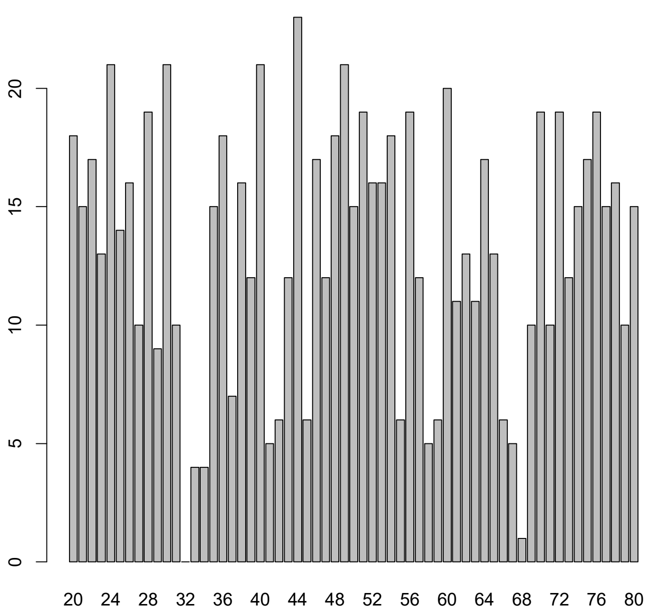

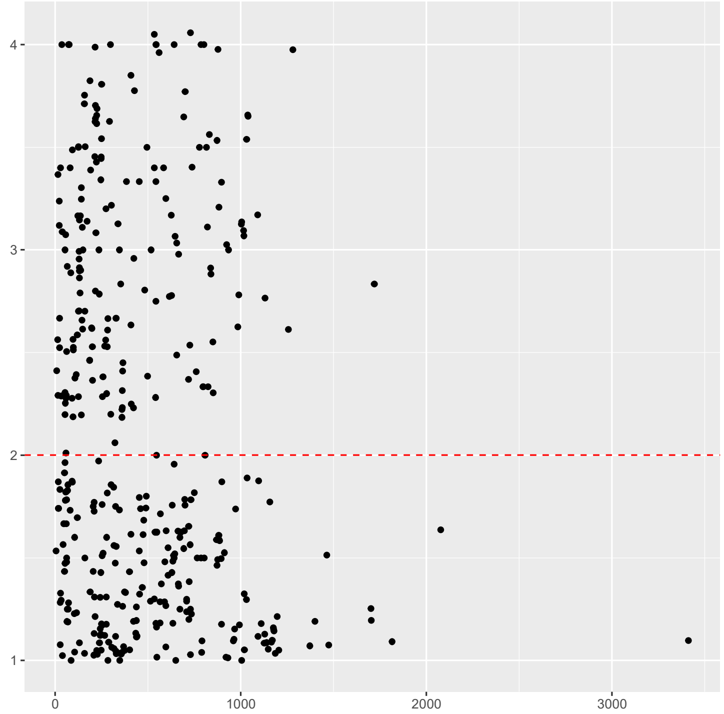

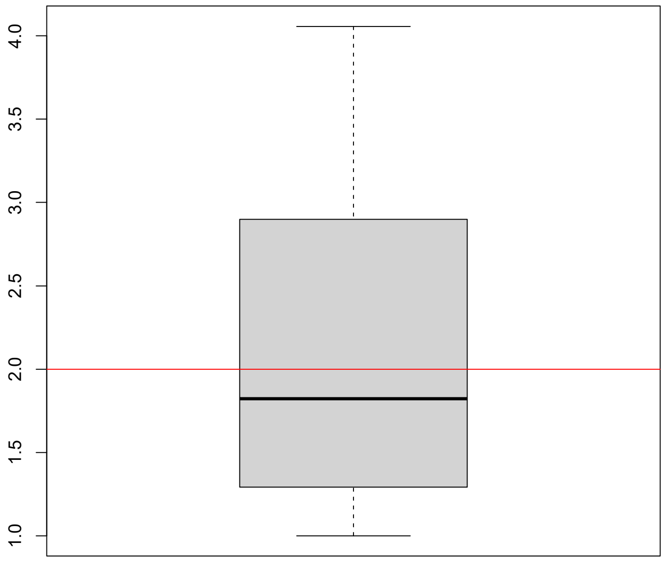

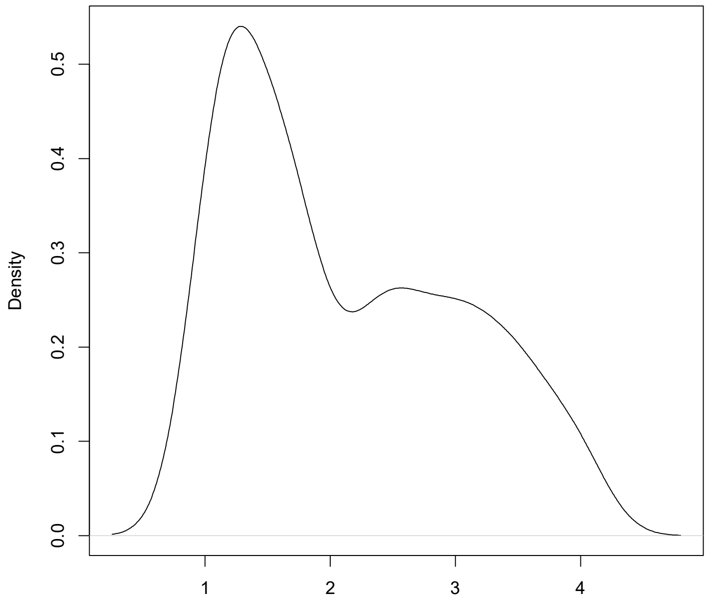

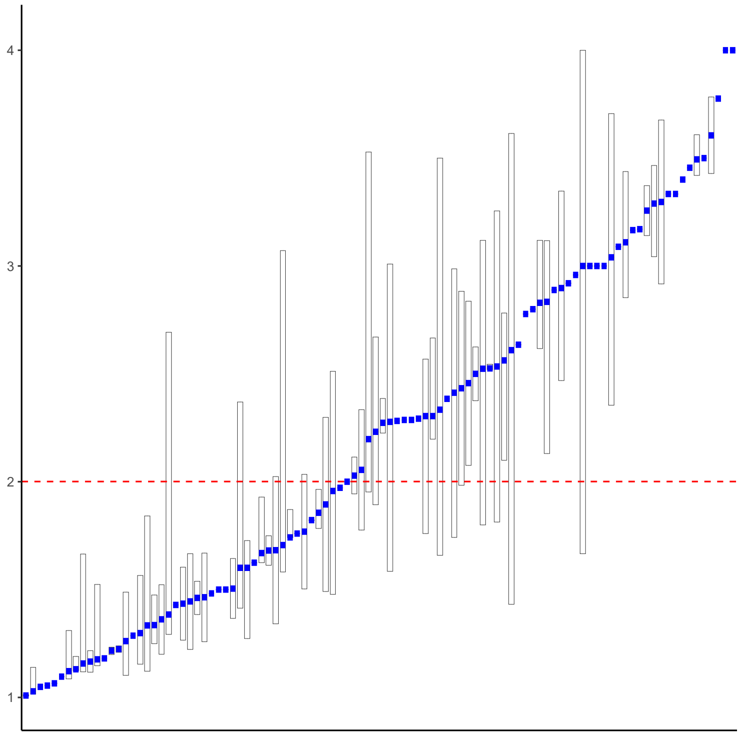

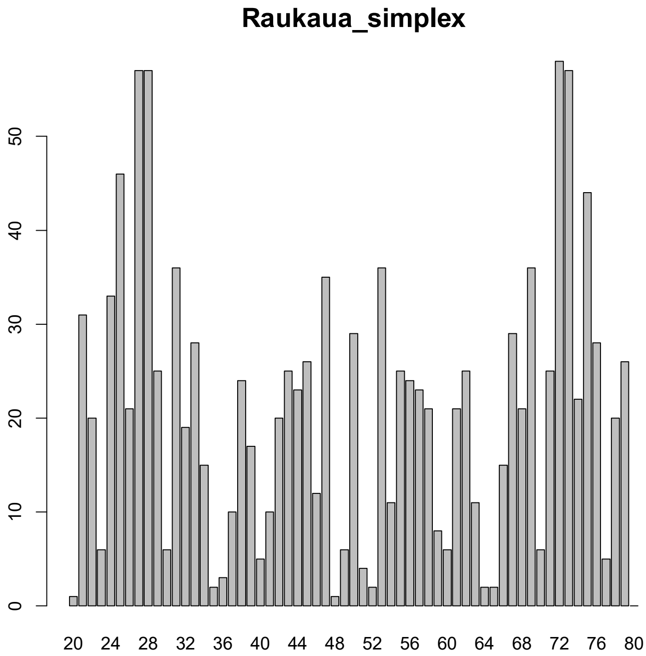
Raukaua_simplex
0
10
20
30
40
50
20 24 28 32 36 40 44 48 52 56 60 64 68 72 76 80

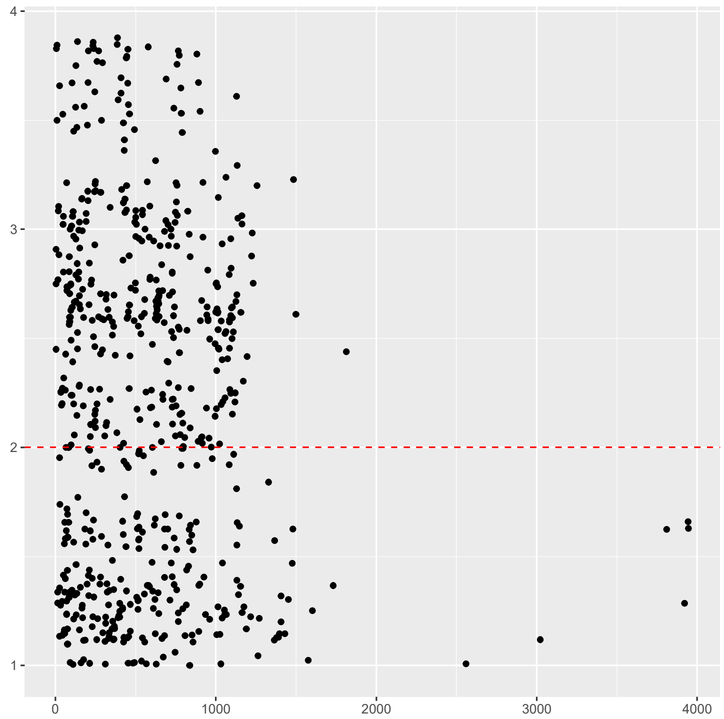
1
2
3
4
0
1000
2000
3000
4000

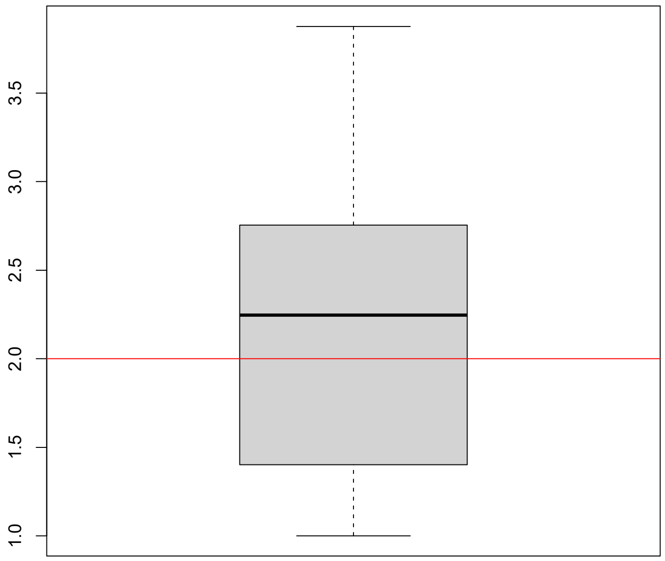
1.0
1.5
2.0
2.5
3.0
3.5

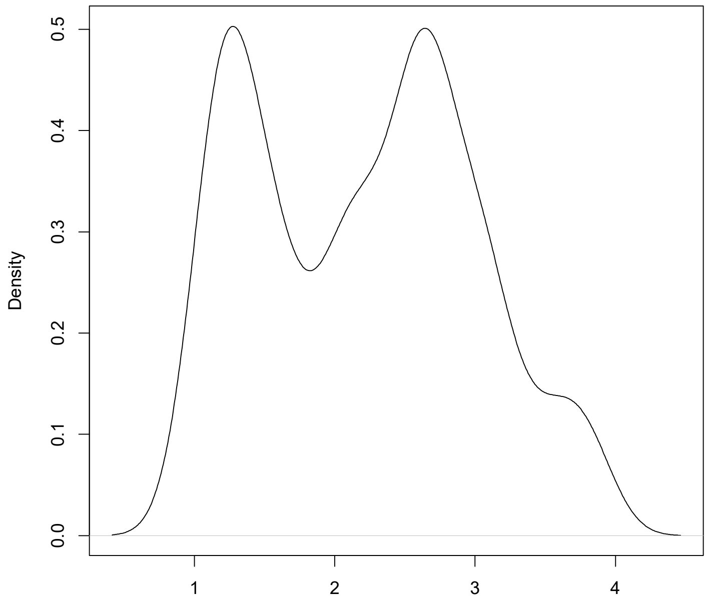
Density
0.0
0.1
0.2
0.3
0.4
0.5
1
2
3
4

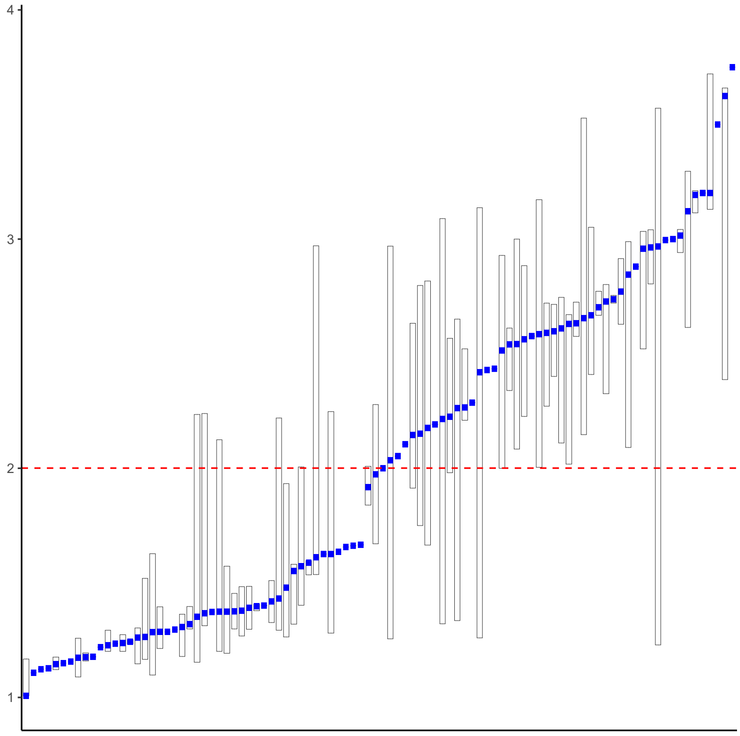
1
2
3
4

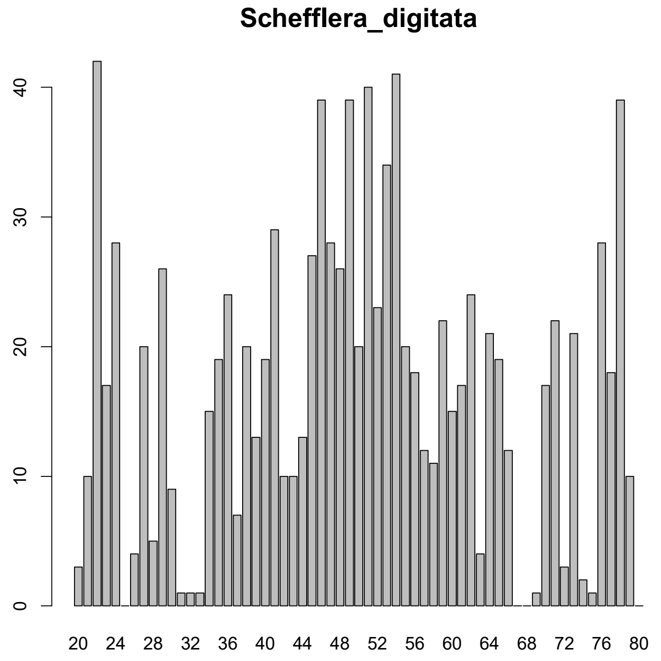
Schefflera_digitata
0
10
20
30
40
20 24 28 32 36 40 44 48 52 56 60 64 68 72 76 80

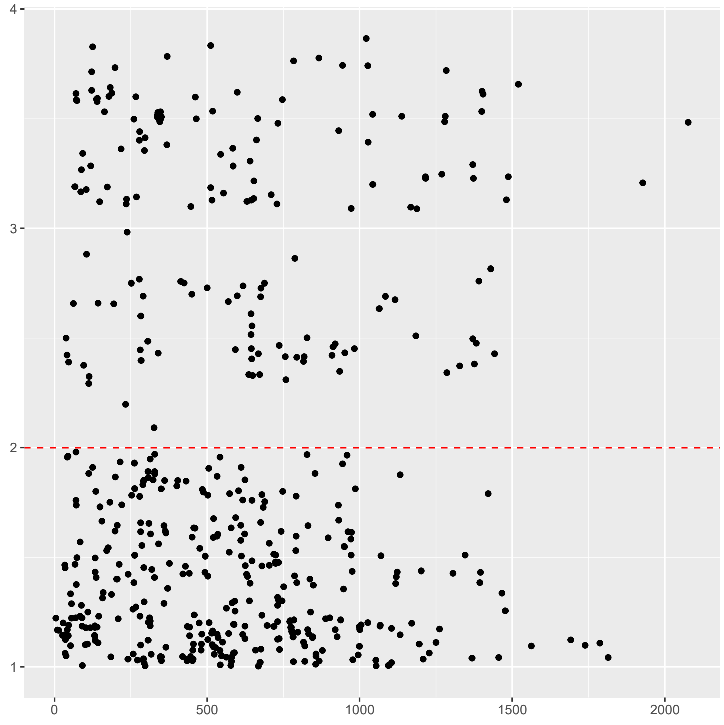
1
2
3
4
0 500 1000 1500 2000

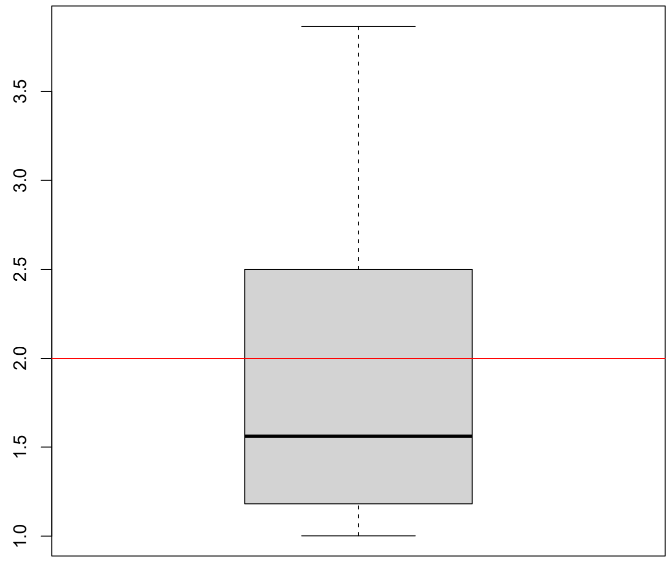
1.0
1.5
2.0
2.5
3.0
3.5

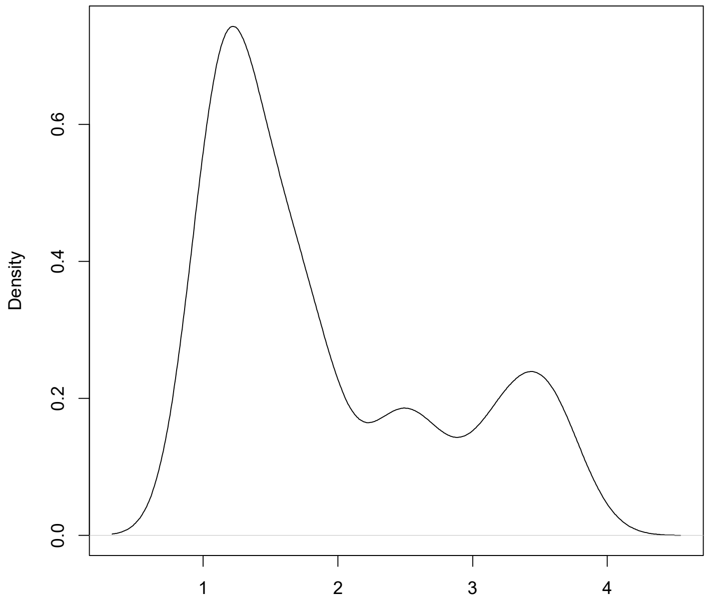
Density
0.0
0.2
0.4
0.6
1 2 3 4

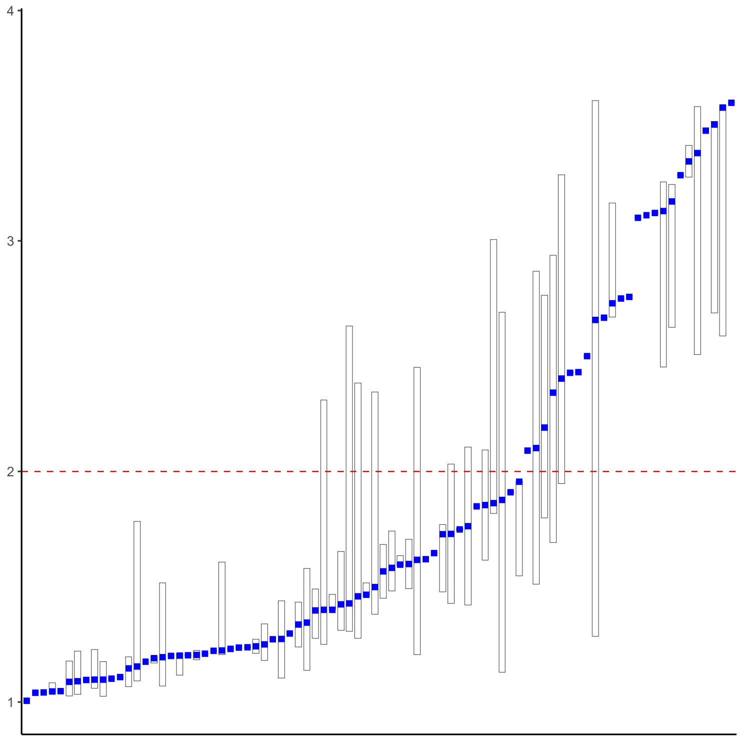
1
2
3
4

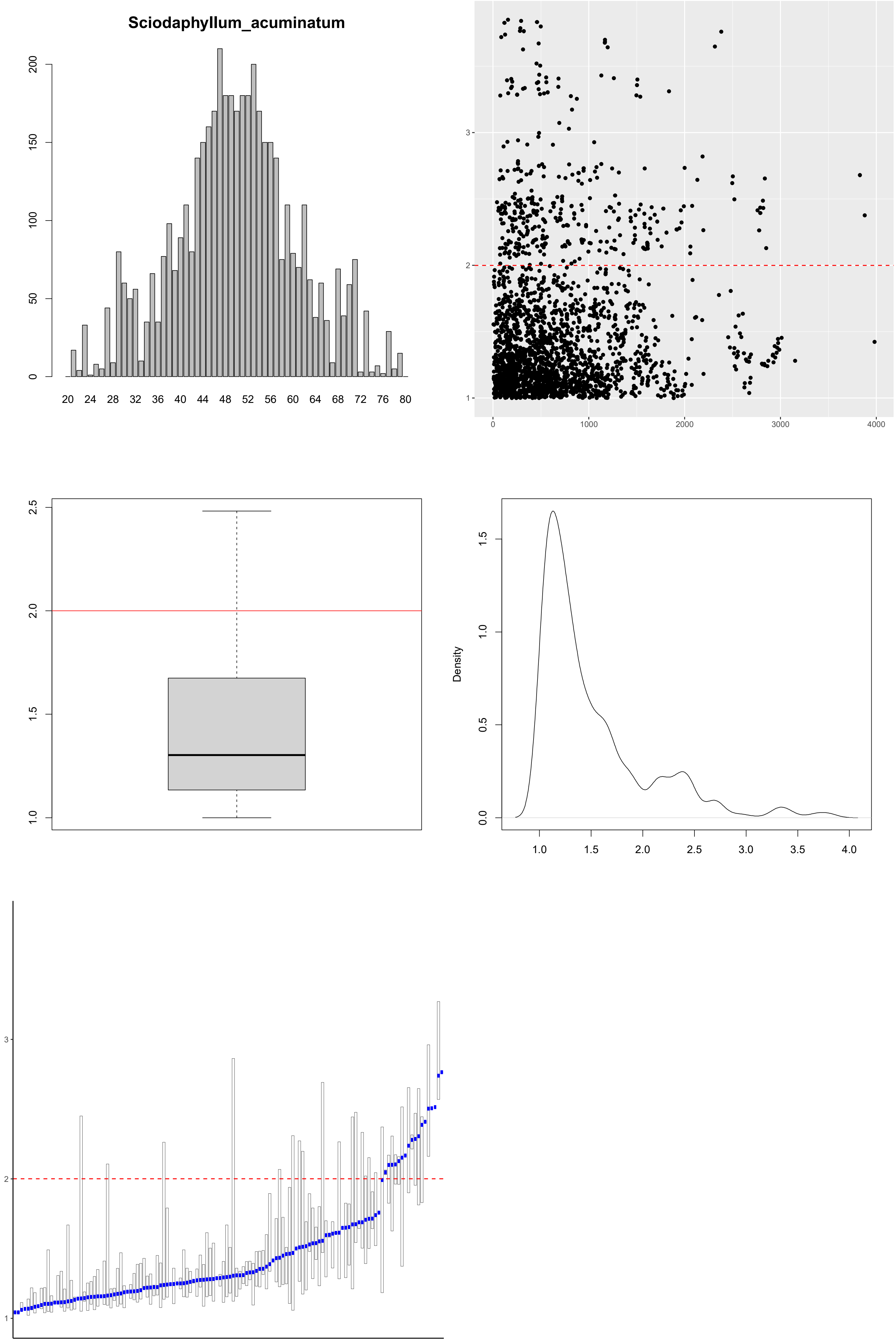
Sciodaphyllum_acuminatum
Density

Sciodaphyllum_angulatum

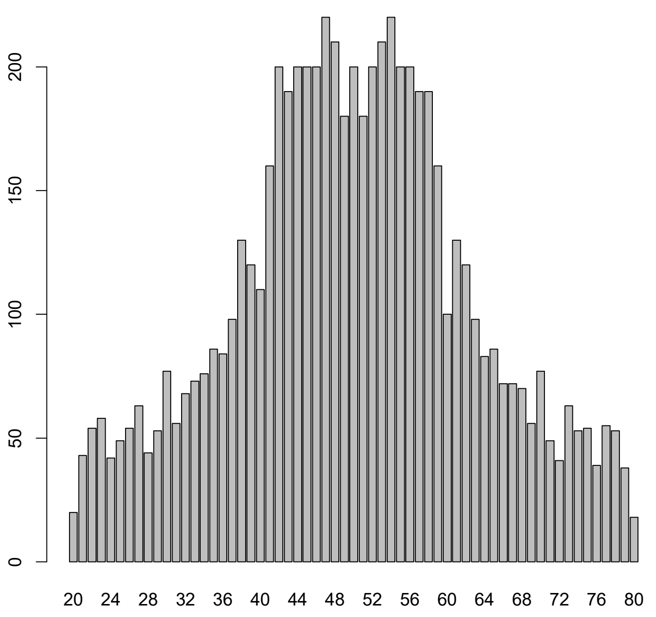

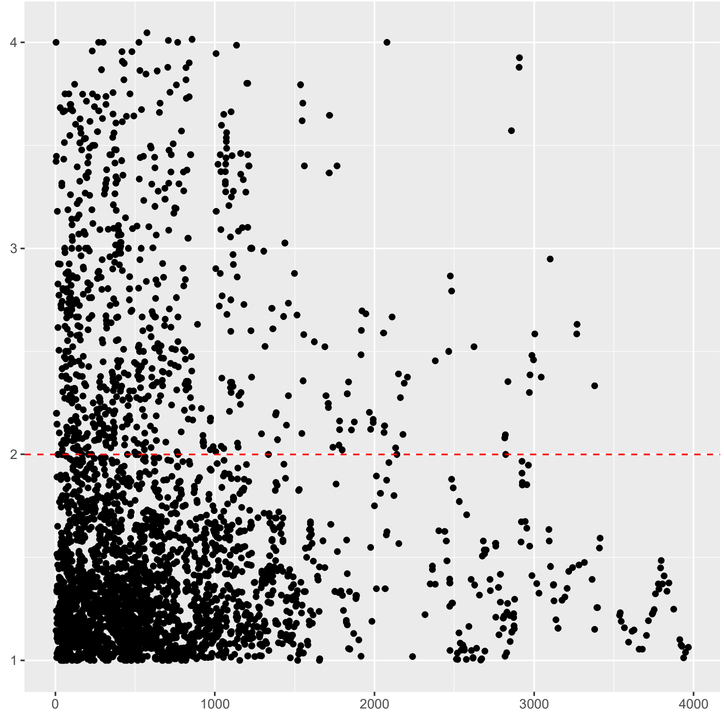

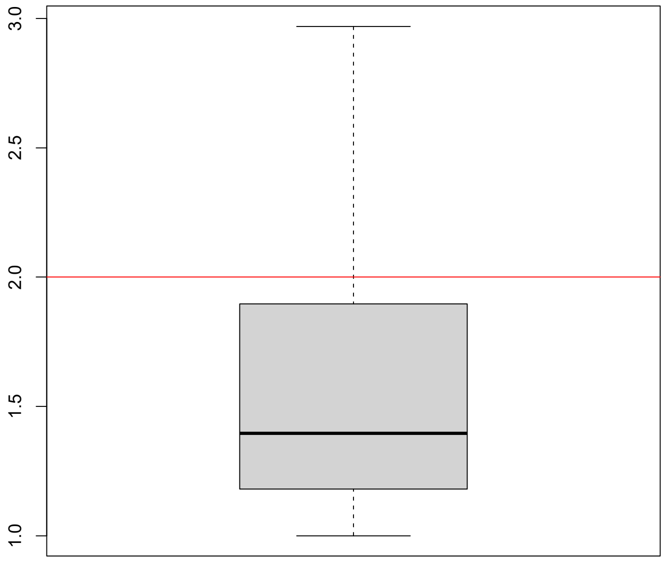

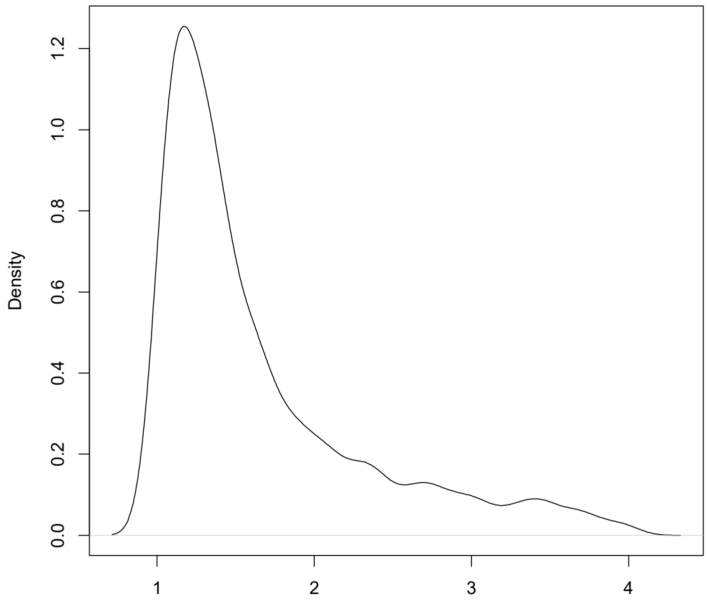

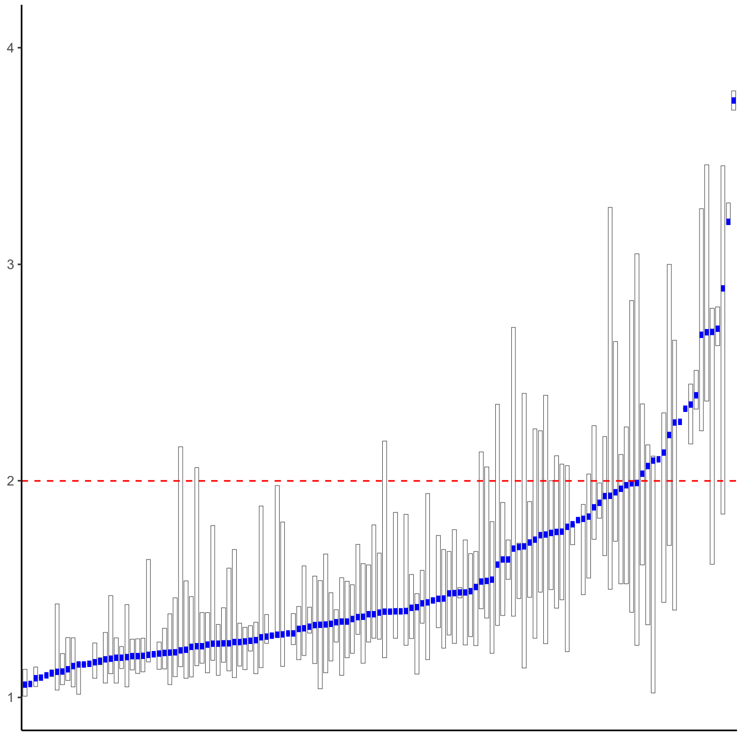

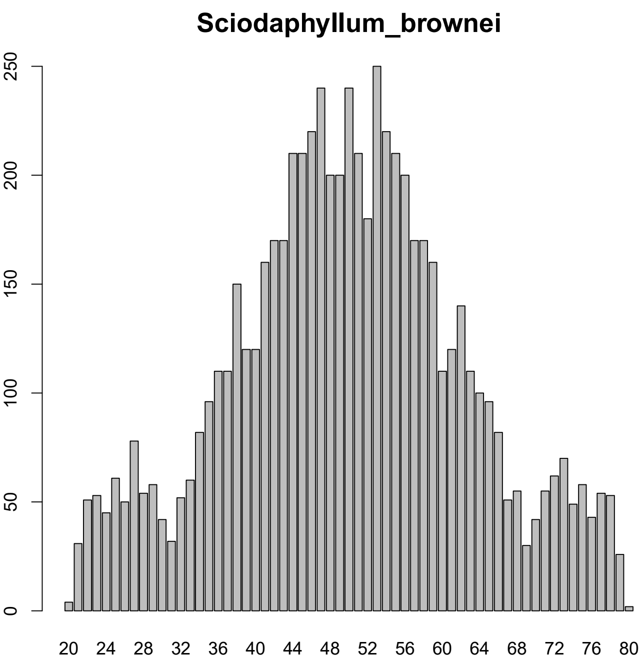
Sciodaphyllum_brownei
0
50
100
150
200
250
20 24 28 32 36 40 44 48 52 56 60 64 68 72 76 80

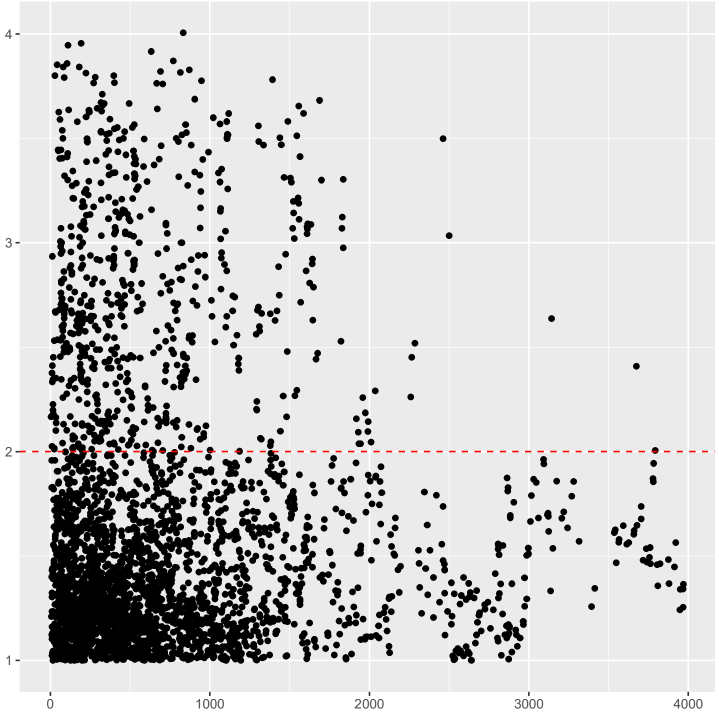
1
2
3
4
0 1000 2000 3000 4000

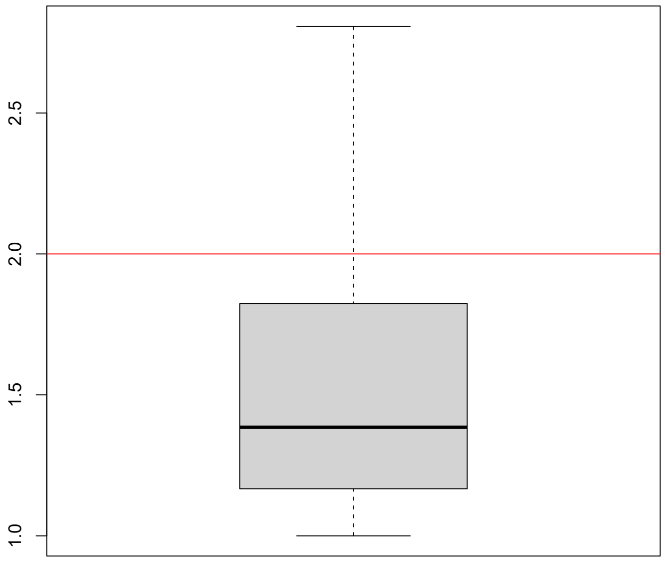
1.0
1.5
2.0
2.5

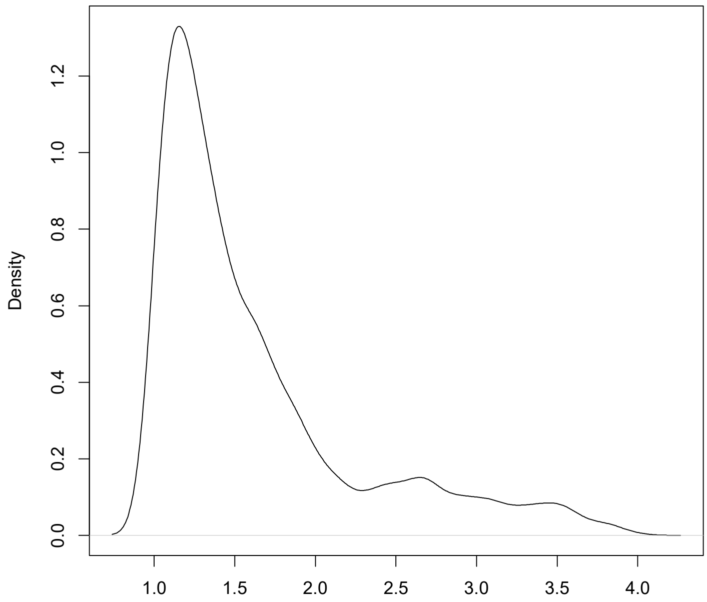
Density
0.0
0.2
0.4
0.6
0.8
1.0
1.2
1.0 1.5 2.0 2.5 3.0 3.5 4.0

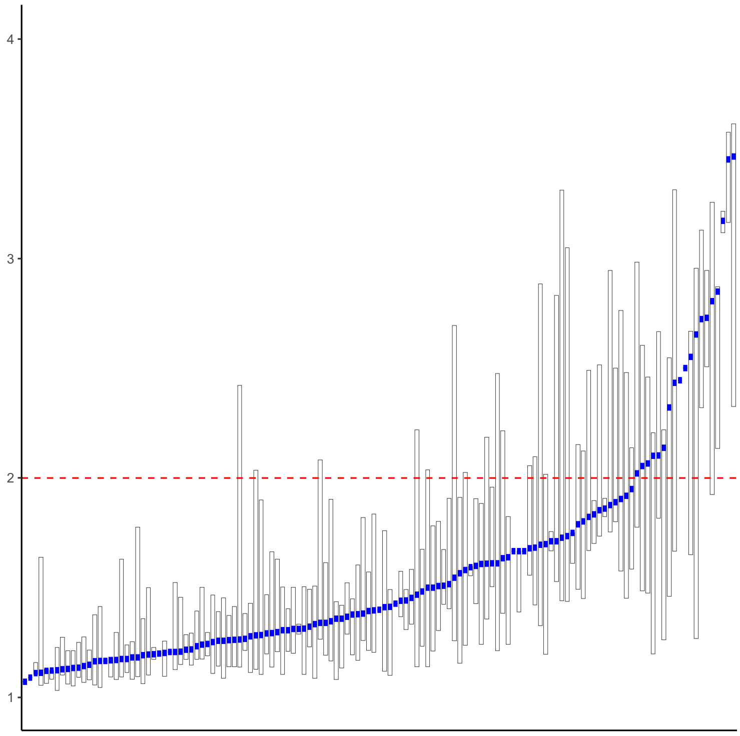
1
2
3
4

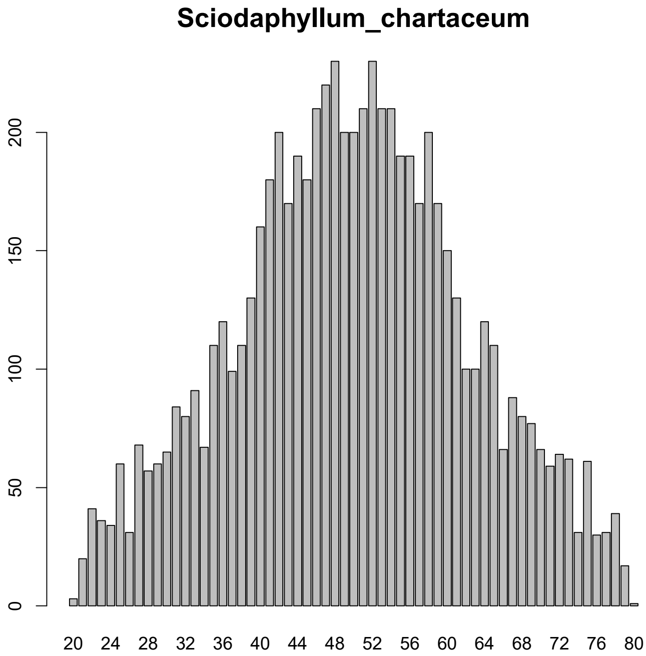
Sciodaphyllum_chartaceum

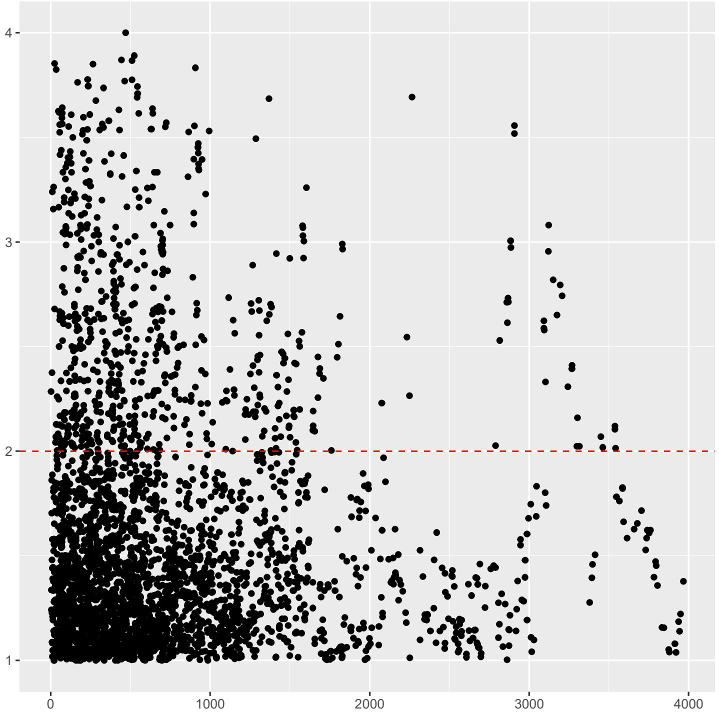

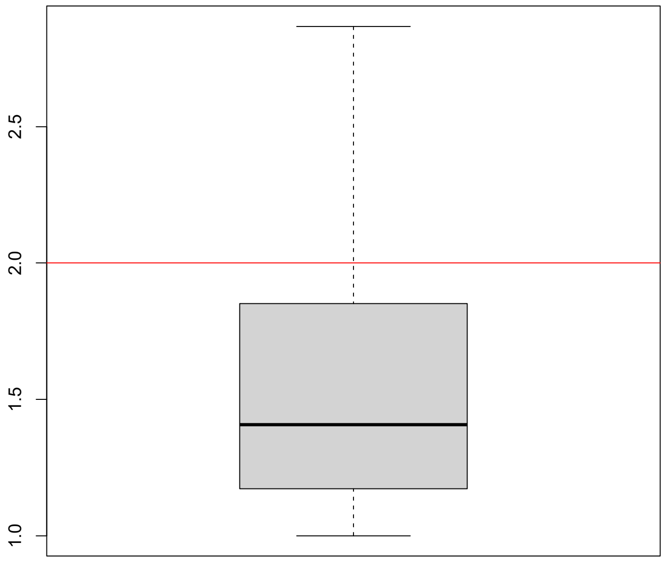

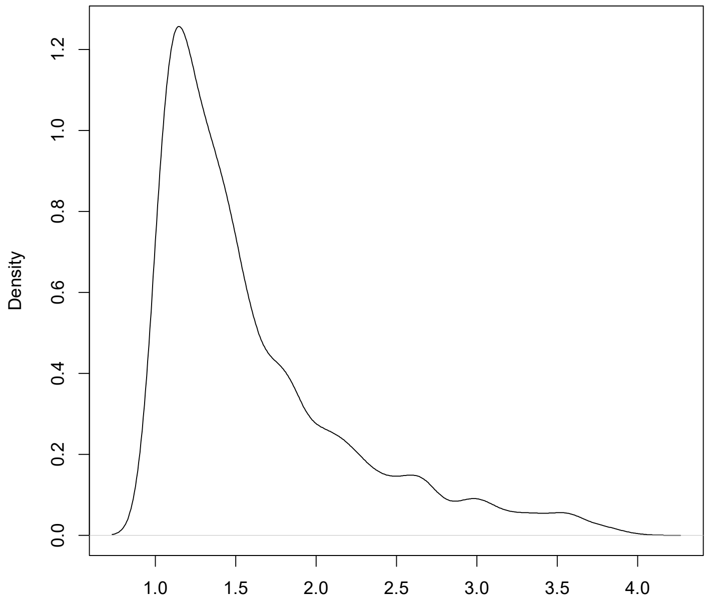
Density

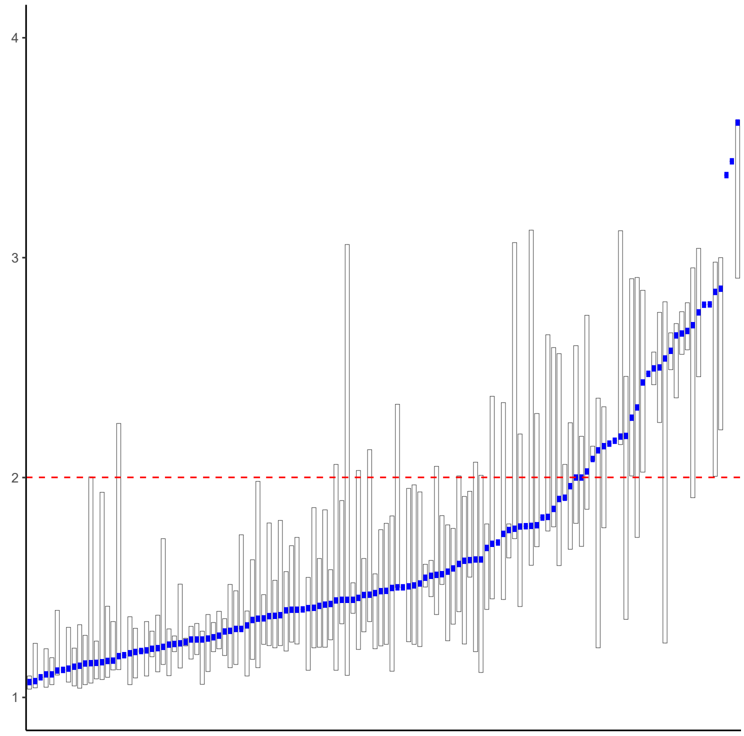

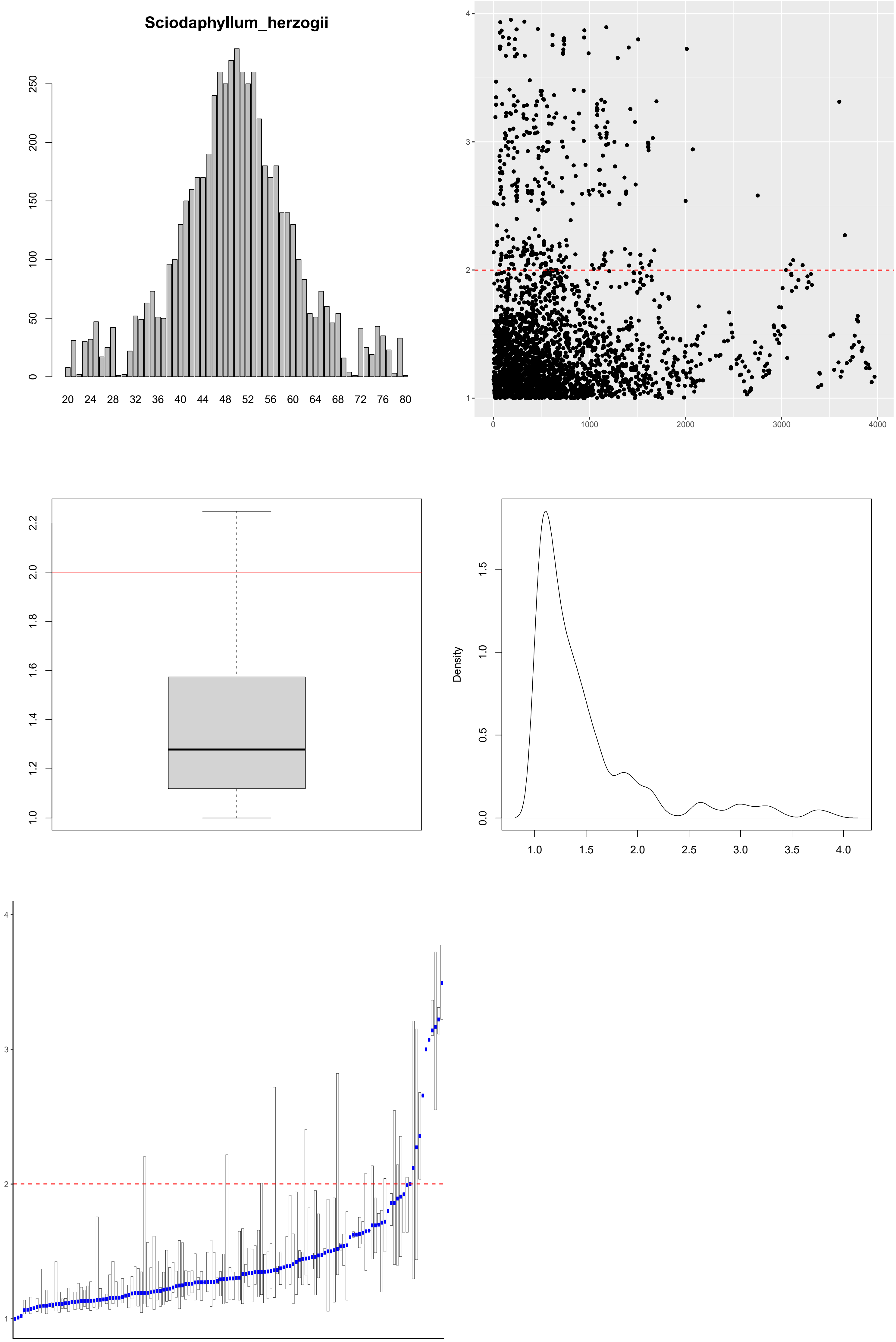
Sciodaphyllum_herzogii
Density

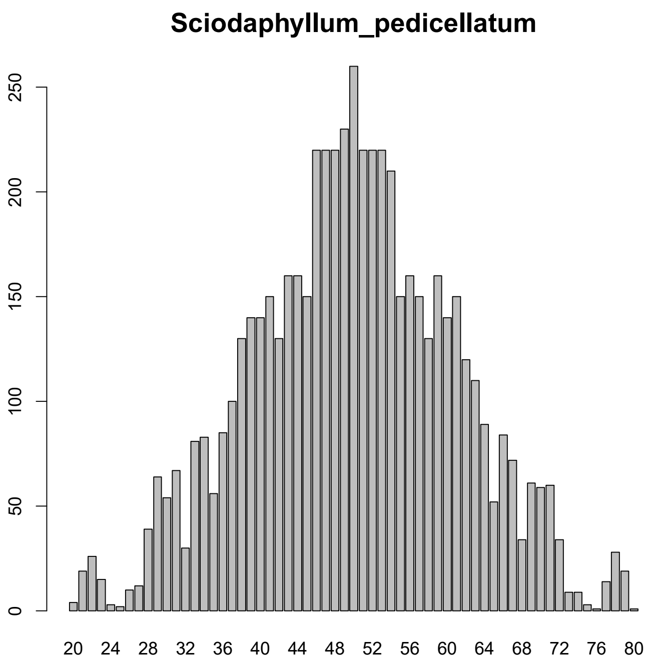
Sciodaphyllum_pedicellatum
0
50
100
150
200
250
20 24 28 32 36 40 44 48 52 56 60 64 68 72 76 80

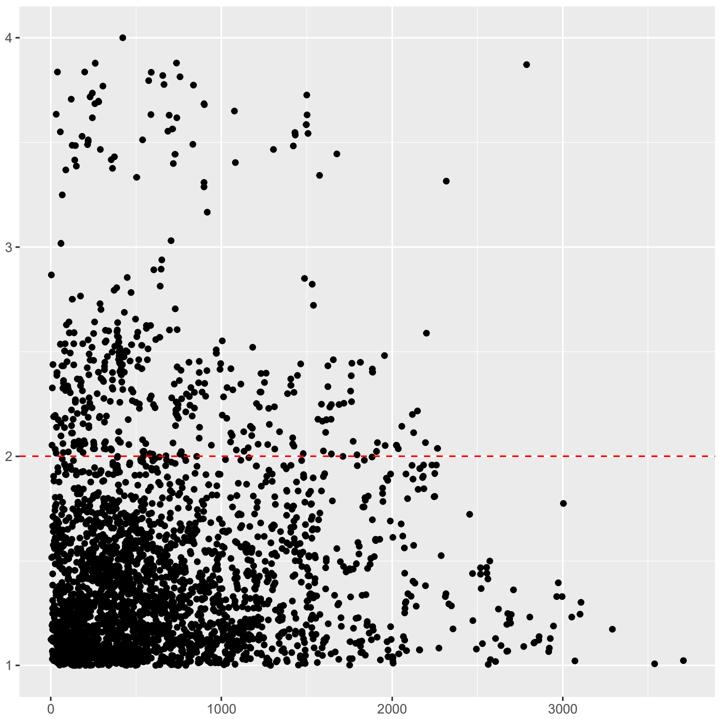
1
2
3
4
0 1000 2000 3000

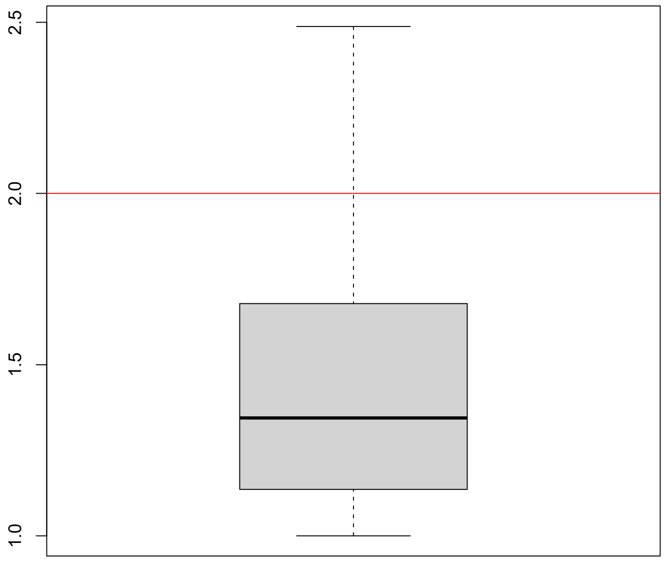
1.0
1.5
2.0
2.5

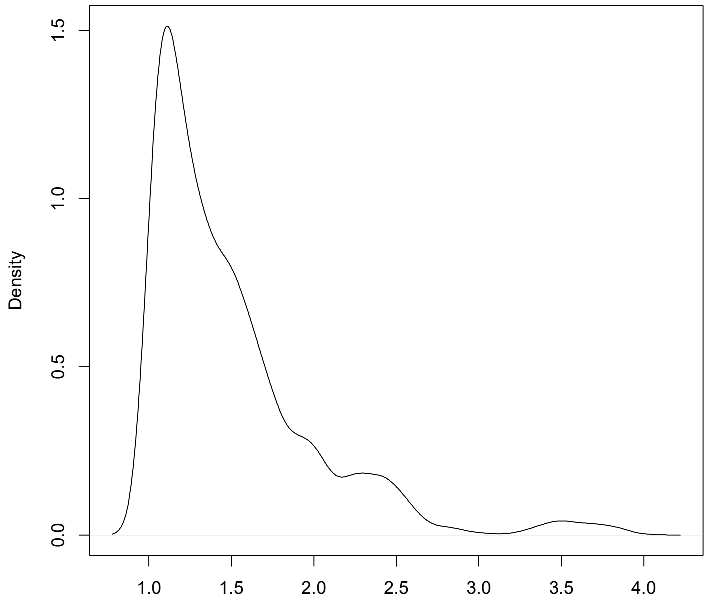
Density
0.0
0.5
1.0
1.5
1.0 1.5 2.0 2.5 3.0 3.5 4.0

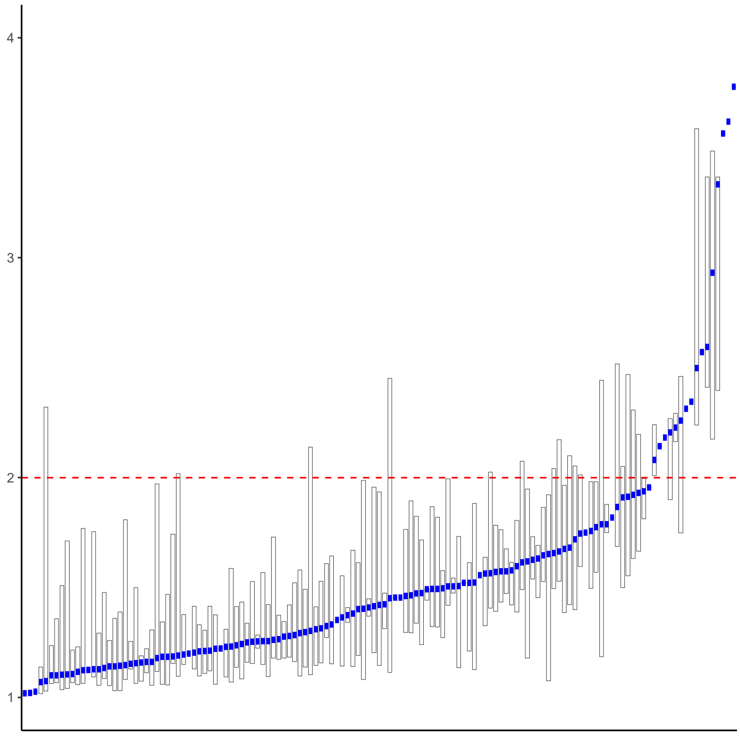
1
2
3
4

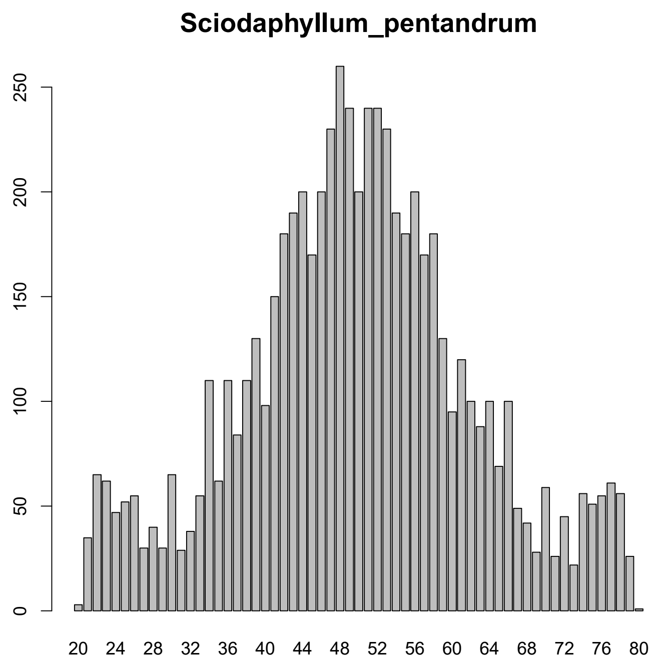
Sciodaphyllum_pentandrum
0
50
100
150
200
250
20 24 28 32 36 40 44 48 52 56 60 64 68 72 76 80

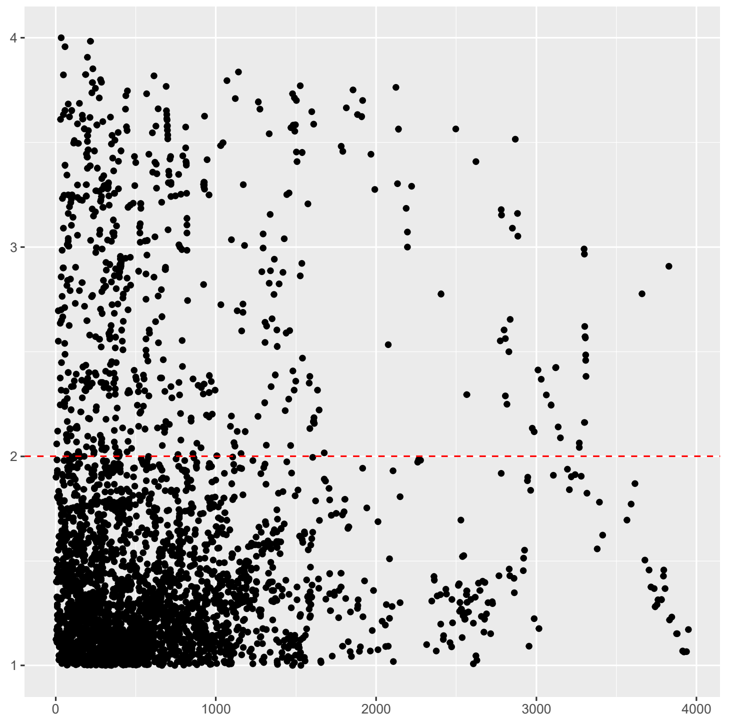
1
2
3
4
0 1000 2000 3000 4000

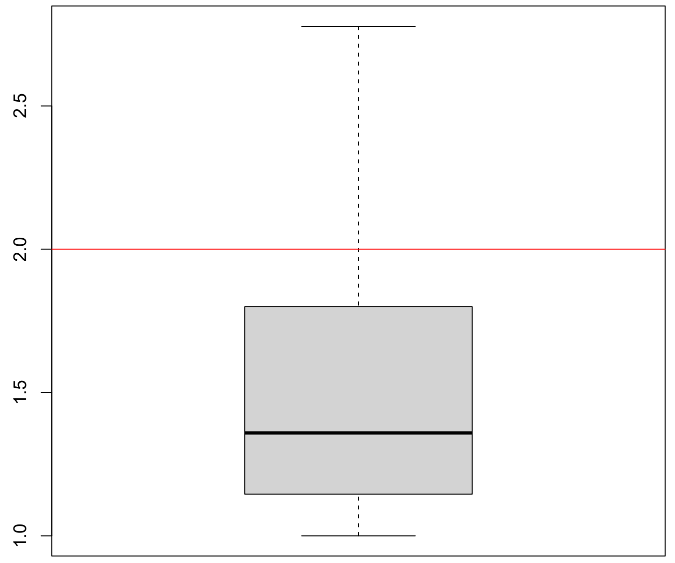
1.0
1.5
2.0
2.5

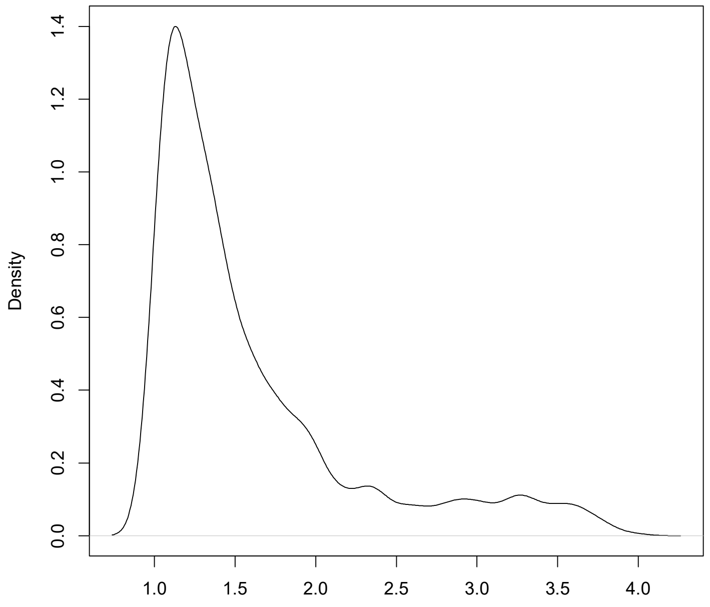
Density
0.0 0.2 0.4 0.6 0.8 1.0 1.2 1.4
1.0 1.5 2.0 2.5 3.0 3.5 4.0

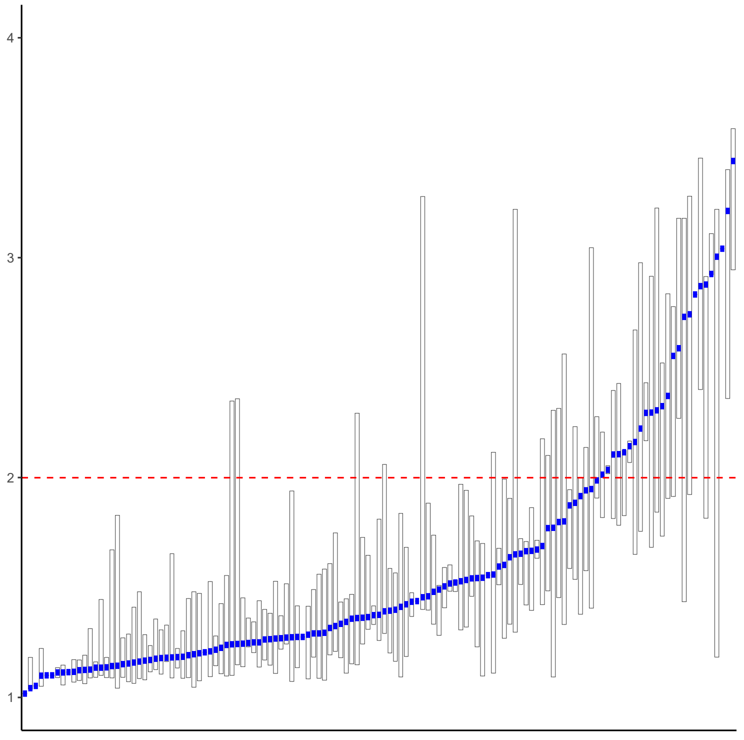
1
2
3
4

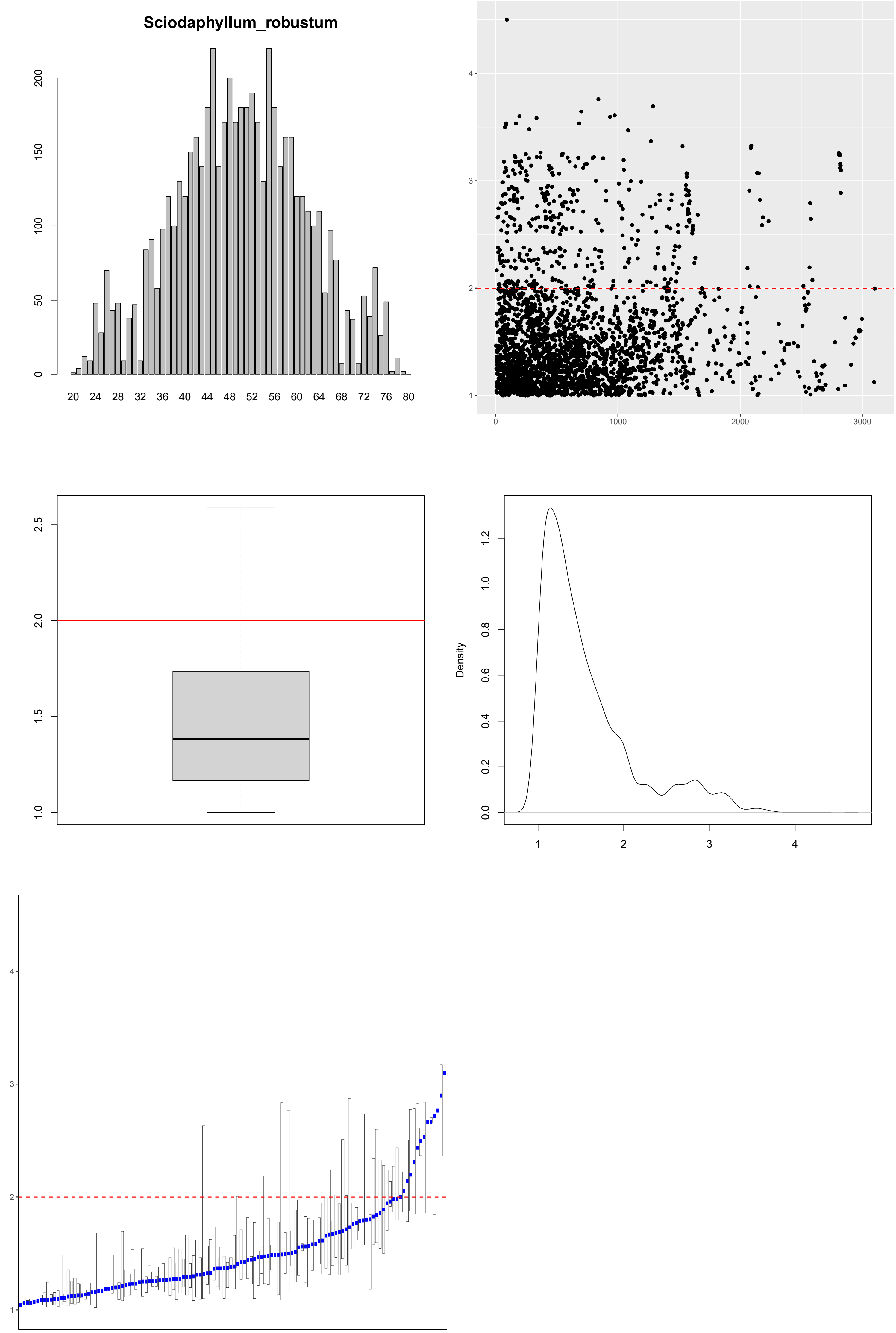
Sciodaphyllum_robustum
Density

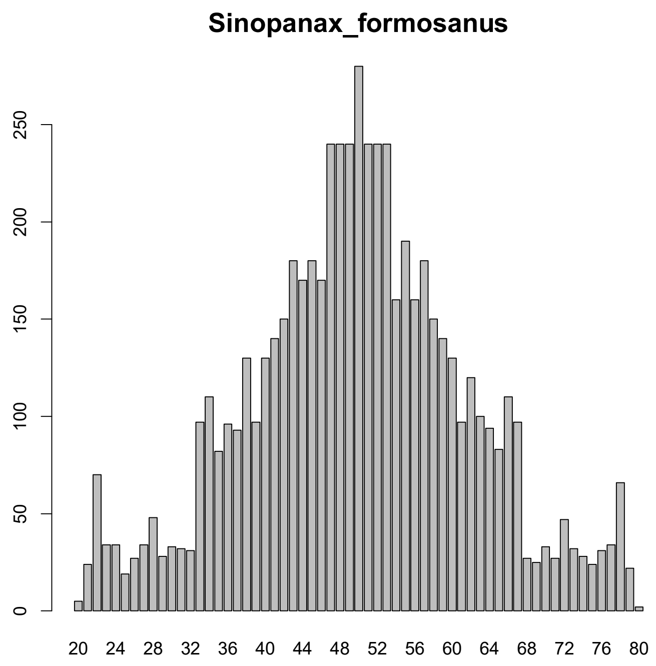
Sinopanax_formosanus
0
50
100
150
200
250
20 24 28 32 36 40 44 48 52 56 60 64 68 72 76 80

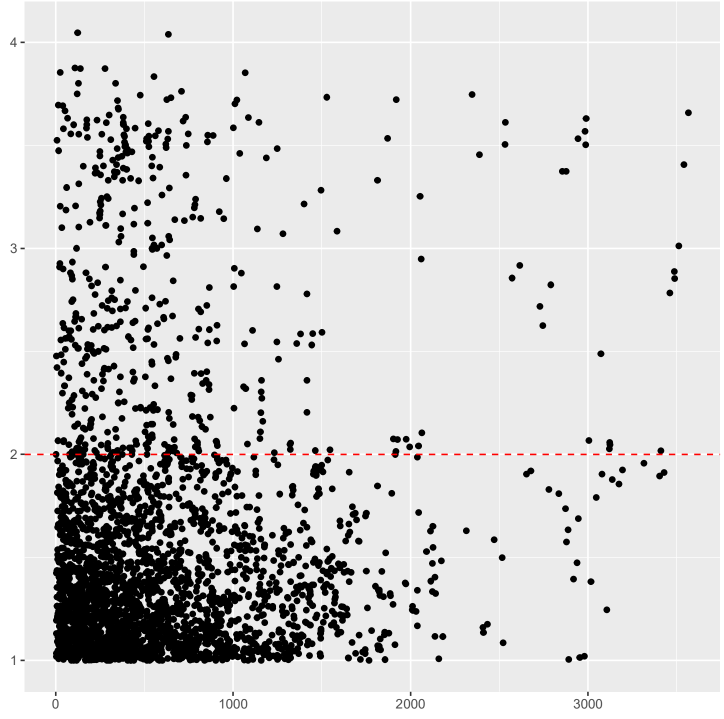
1
2
3
4
0 1000 2000 3000

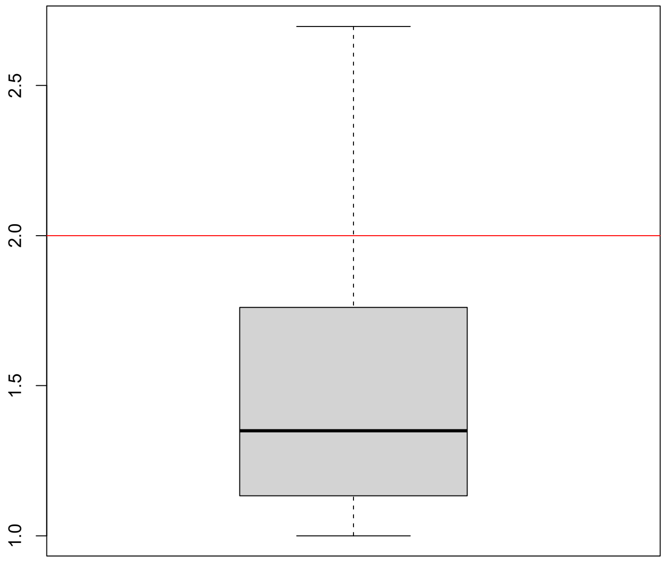
1.0
1.5
2.0
2.5

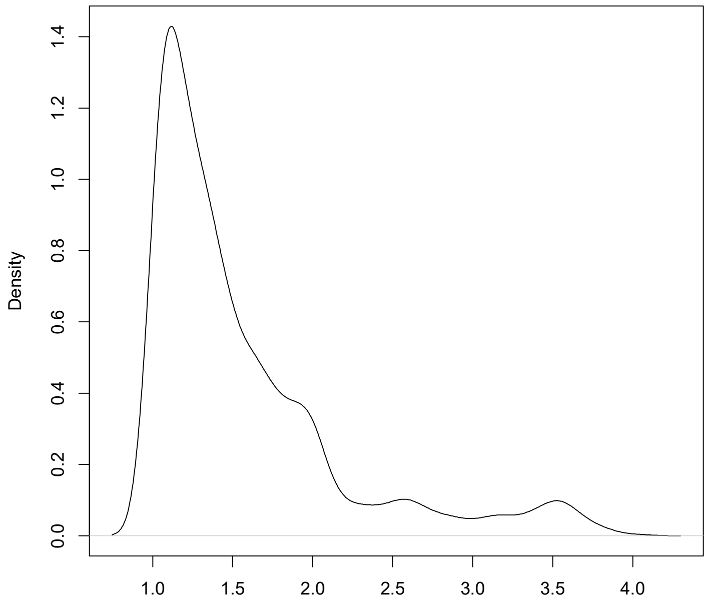
Density
0.0 0.2 0.4 0.6 0.8 1.0 1.2 1.4
1.0 1.5 2.0 2.5 3.0 3.5 4.0

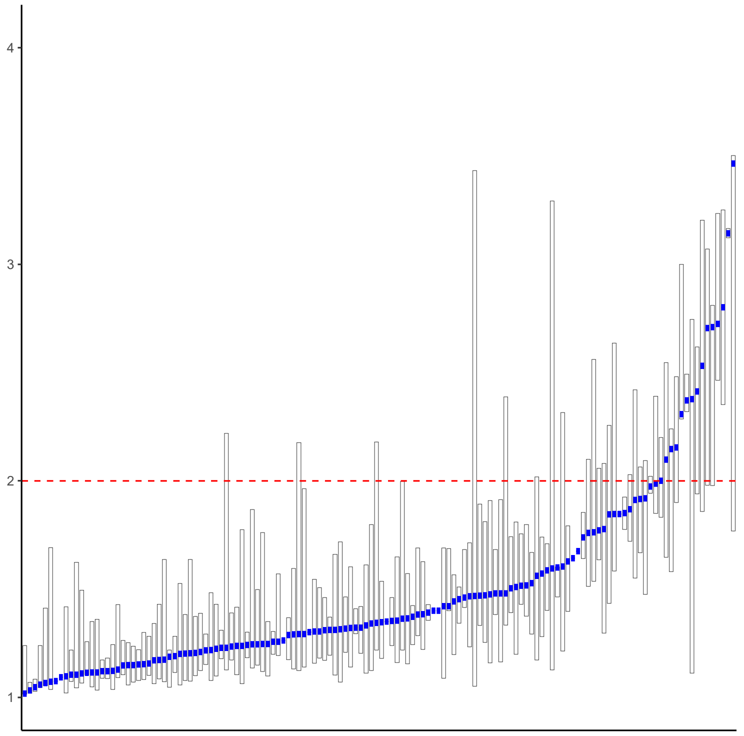
1
2
3
4

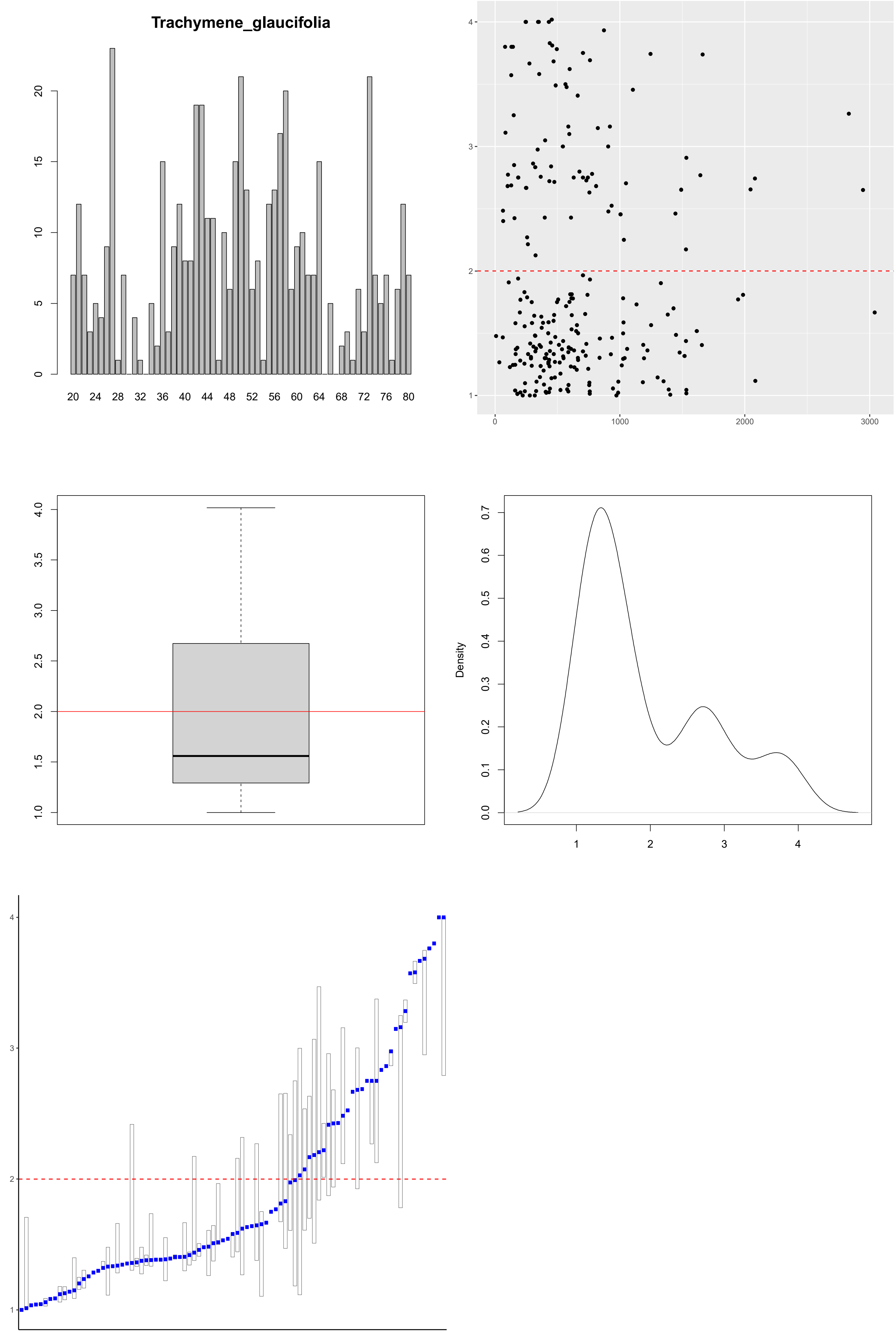
Trachymene_glaucifolia
Density

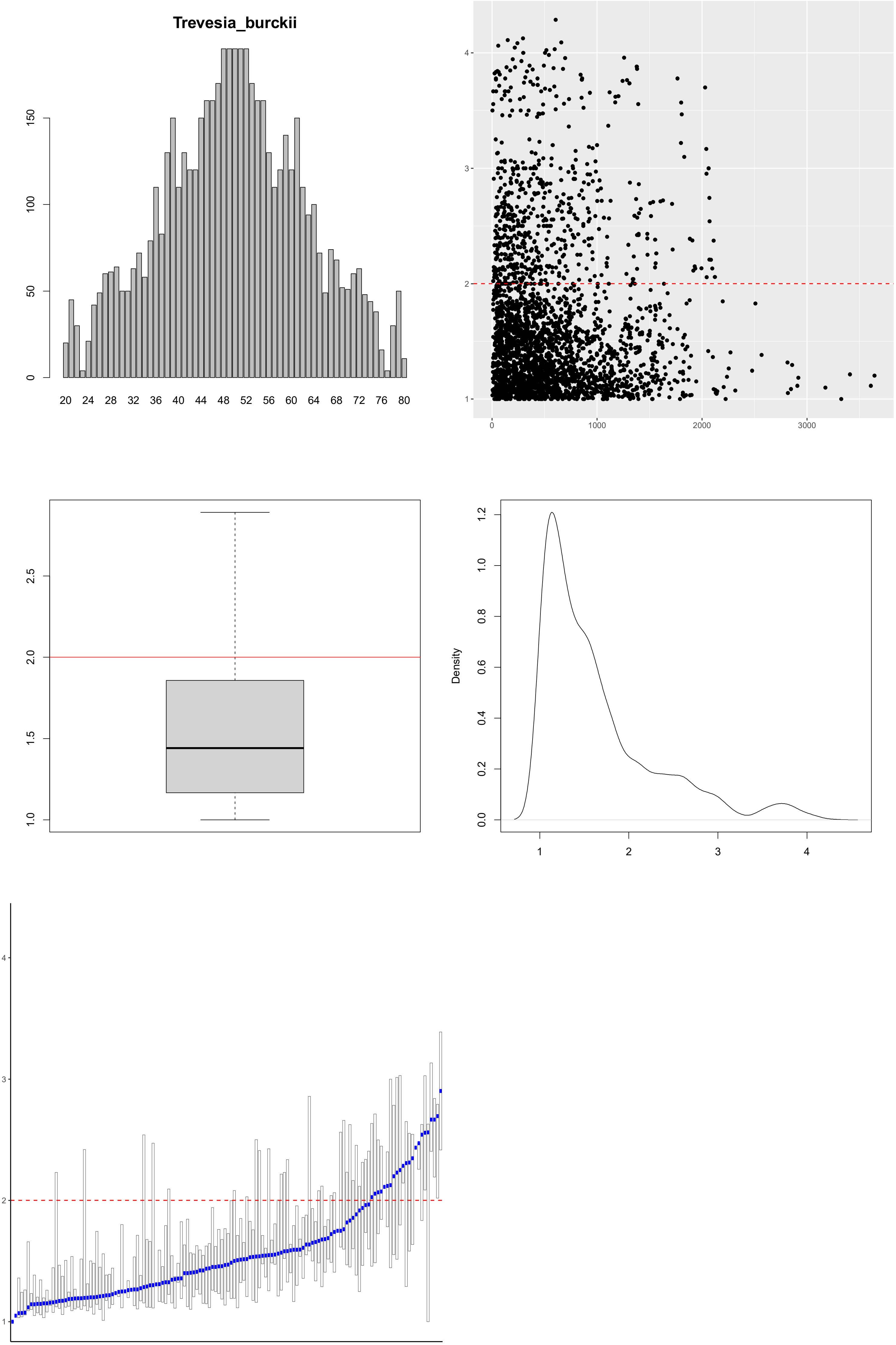
Trevesia_burckii
0
50
100
150
20 24 28 32 36 40 44 48 52 56 60 64 68 72 76 80
1
2
3
4
0
1000
2000
3000
1.0
1.5
2.0
2.5
Density
0.0
0.2
0.4
0.6
0.8
1.0
1.2
1 2 3 4

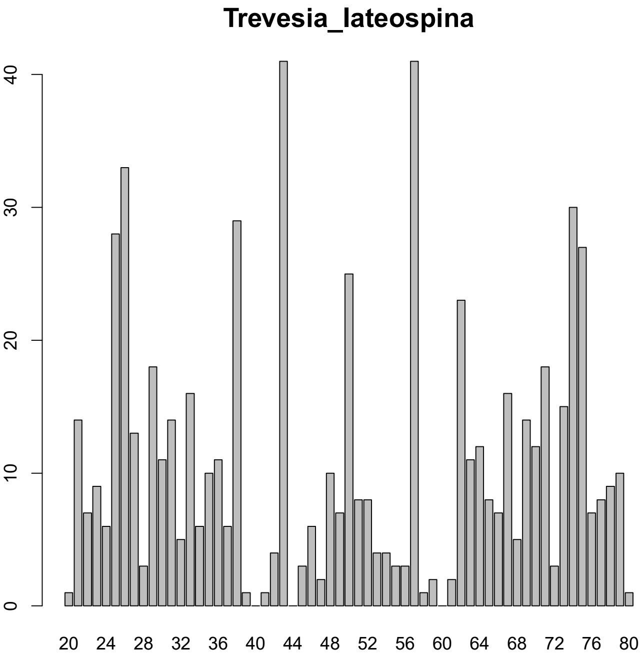
Trevesia_lateospina

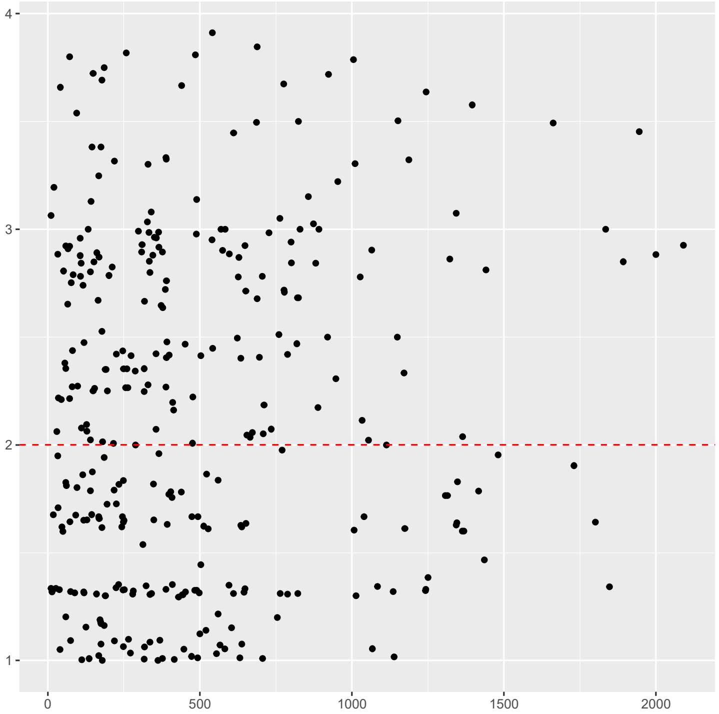

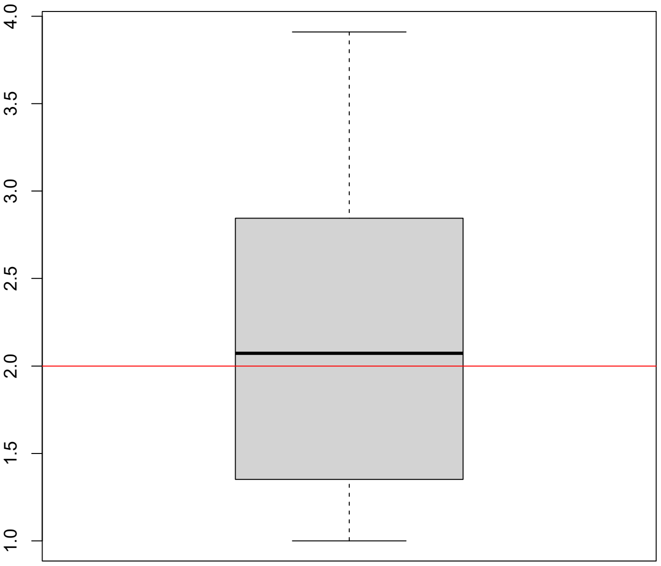

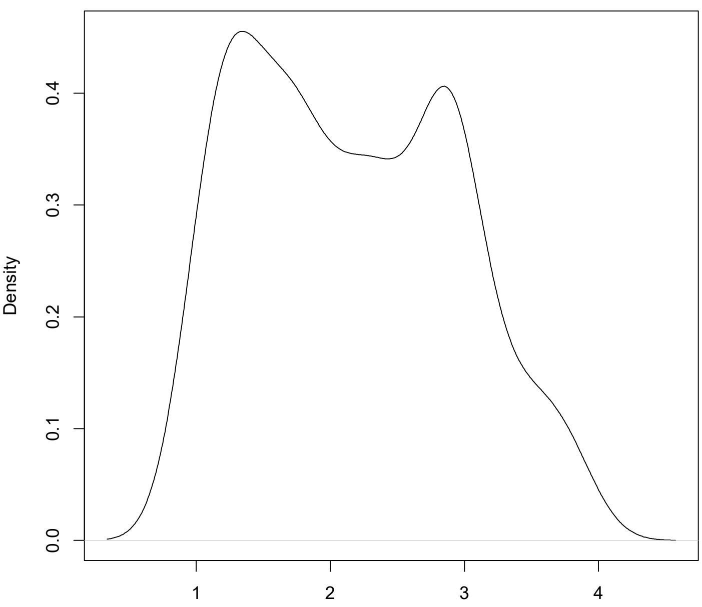
Density

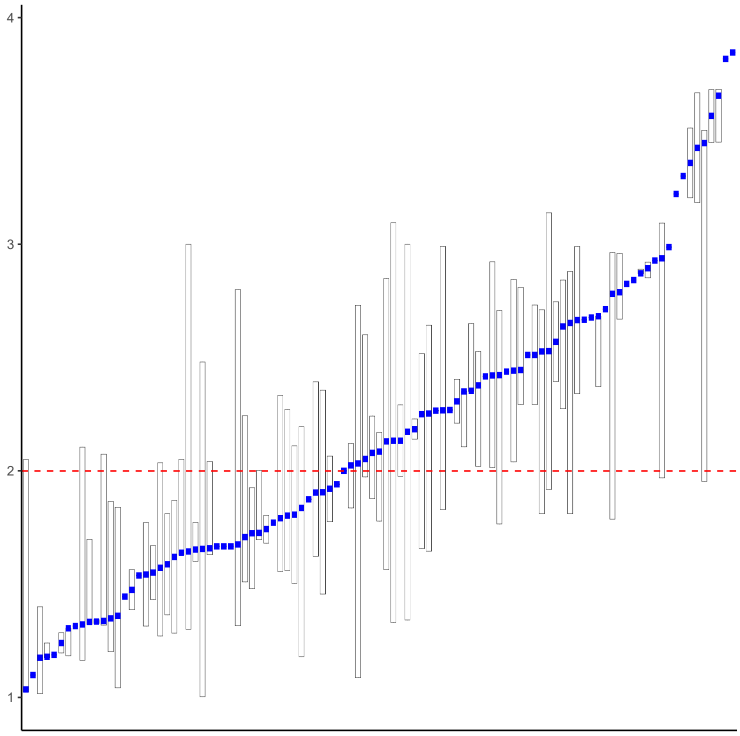

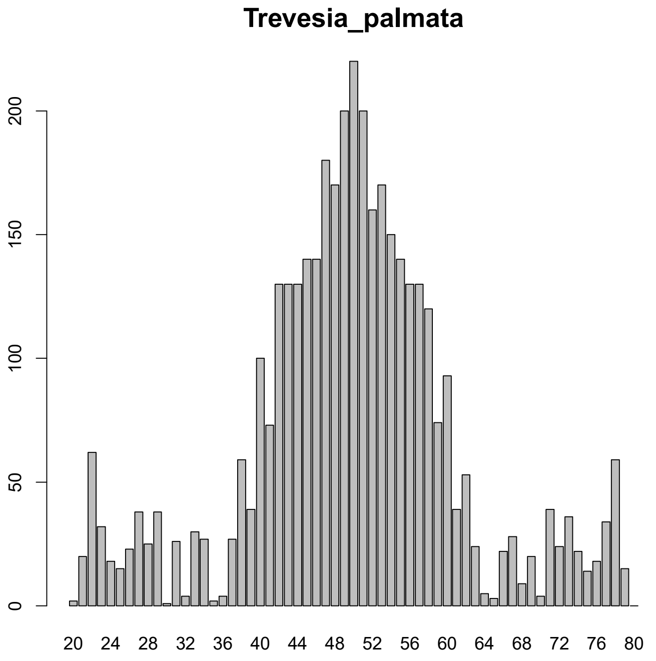
Trevesia_palmata
0
50
100
150
200
20 24 28 32 36 40 44 48 52 56 60 64 68 72 76 80

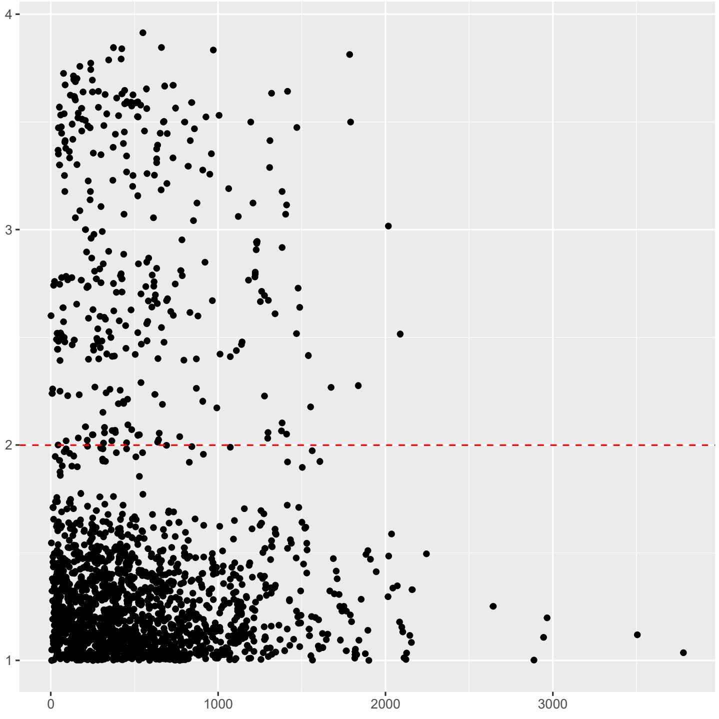
1
2
3
4
0
1000
2000
3000

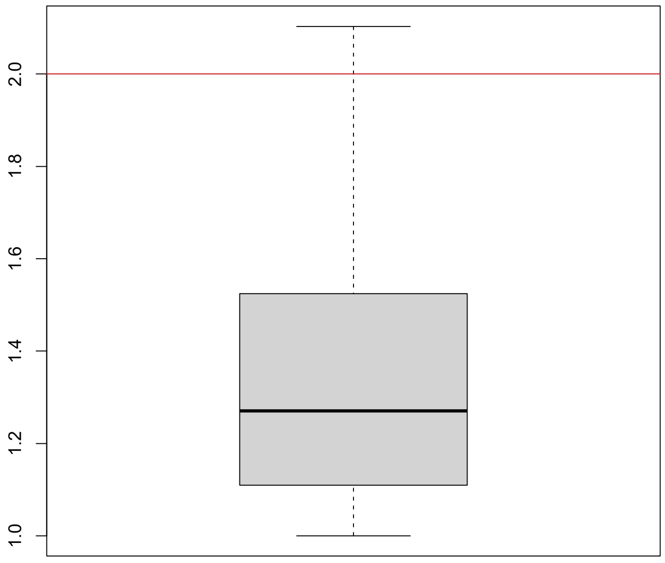
1.0
1.2
1.4
1.6
1.8
2.0

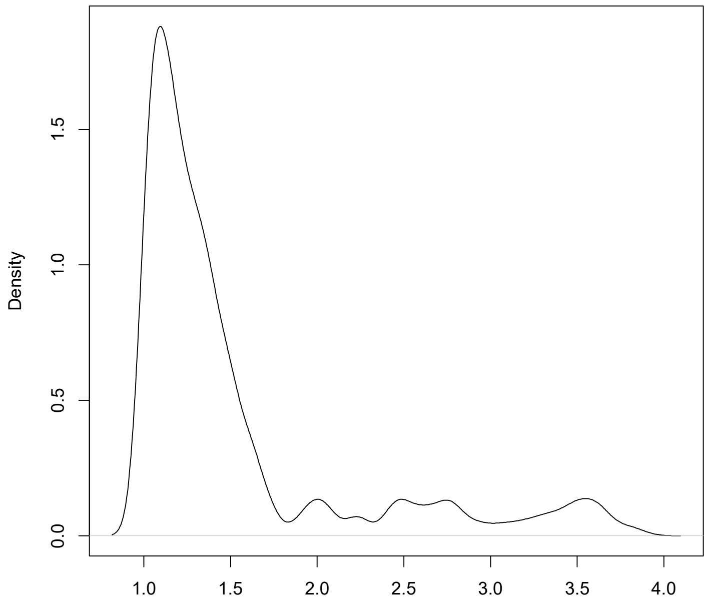
Density
0.0
0.5
1.0
1.5
1.0 1.5 2.0 2.5 3.0 3.5 4.0

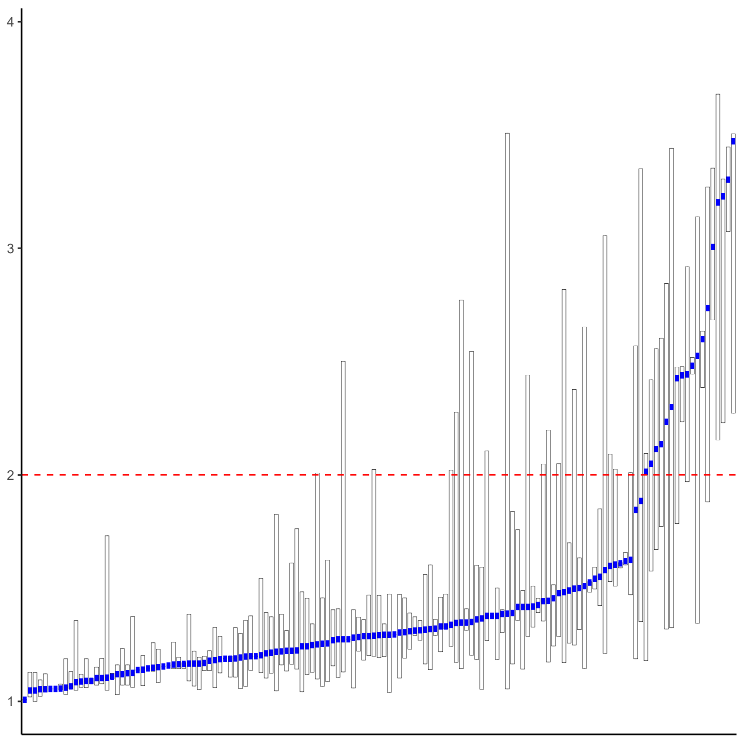
1
2
3
4

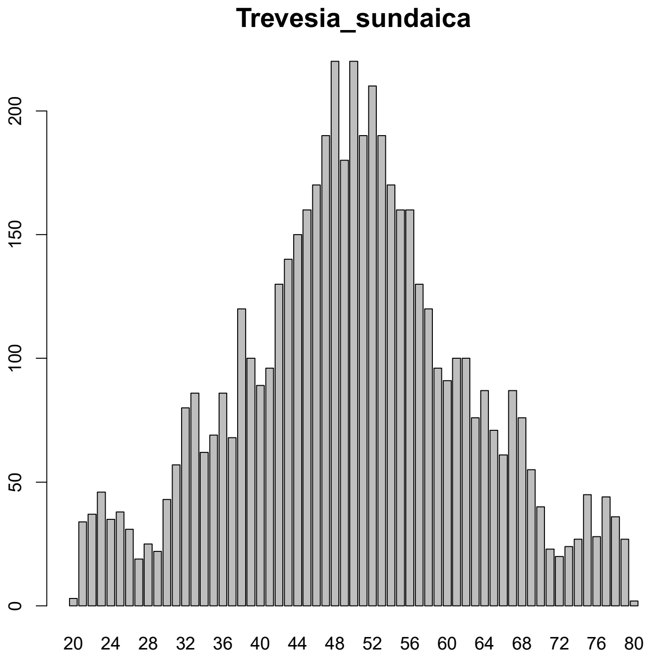
Trevesia_sundaica

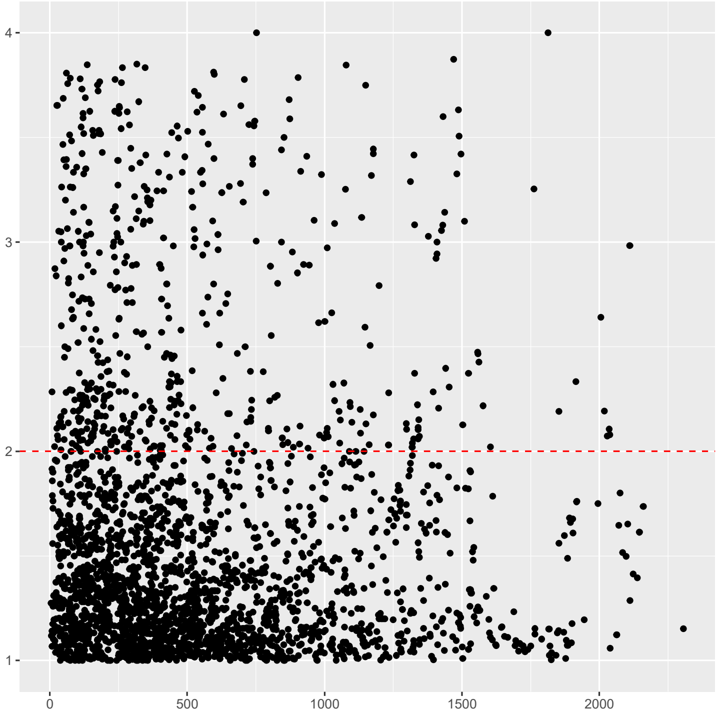

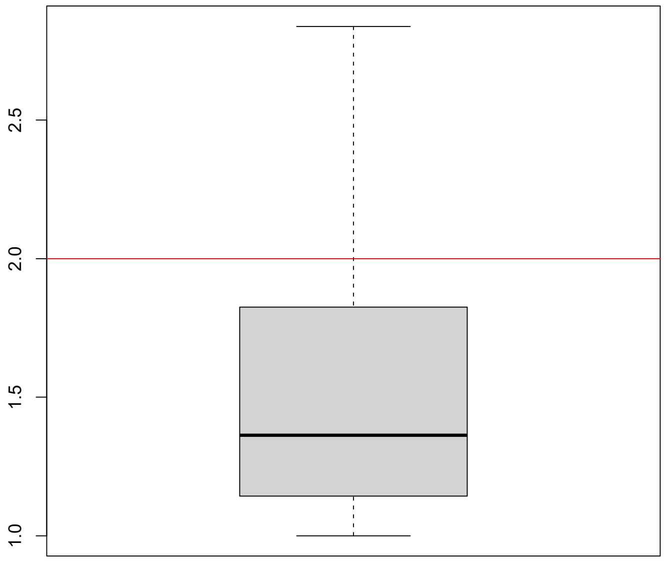

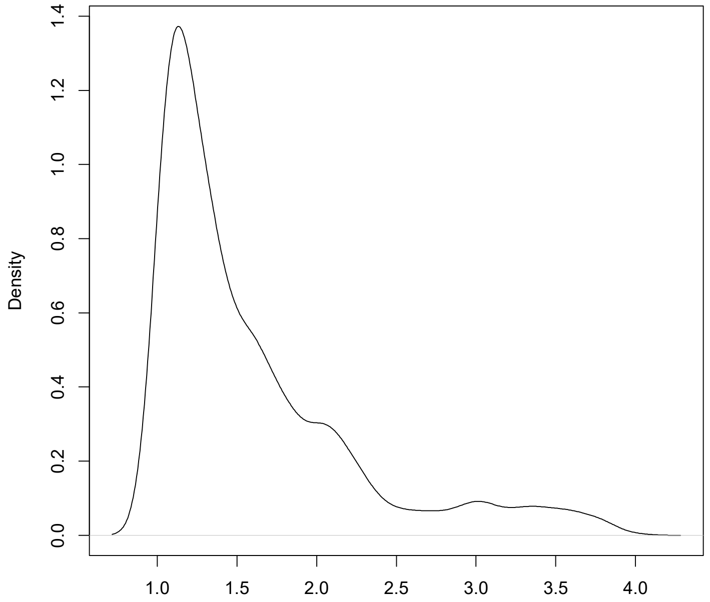
Density

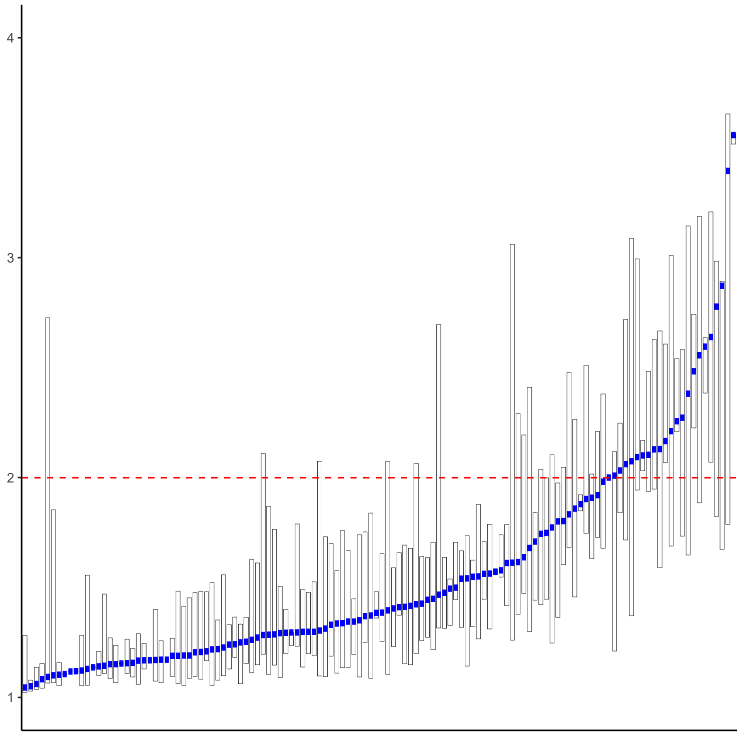

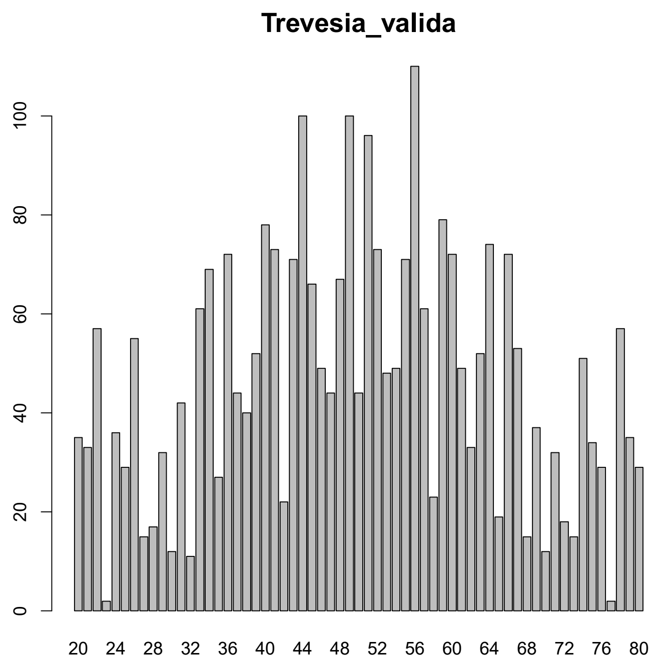
Trevesia_valida
0
20
40
60
80
100
20 24 28 32 36 40 44 48 52 56 60 64 68 72 76 80

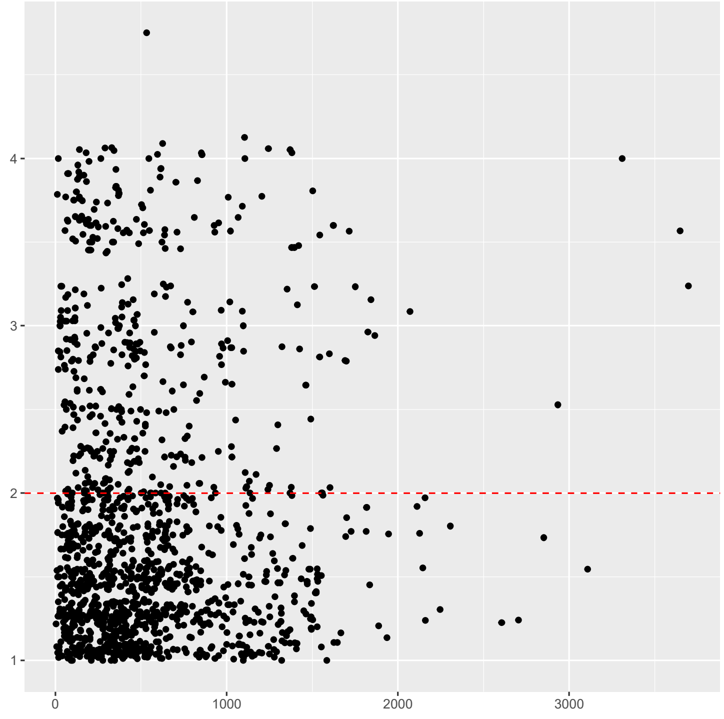
1
2
3
4
0
1000
2000
3000

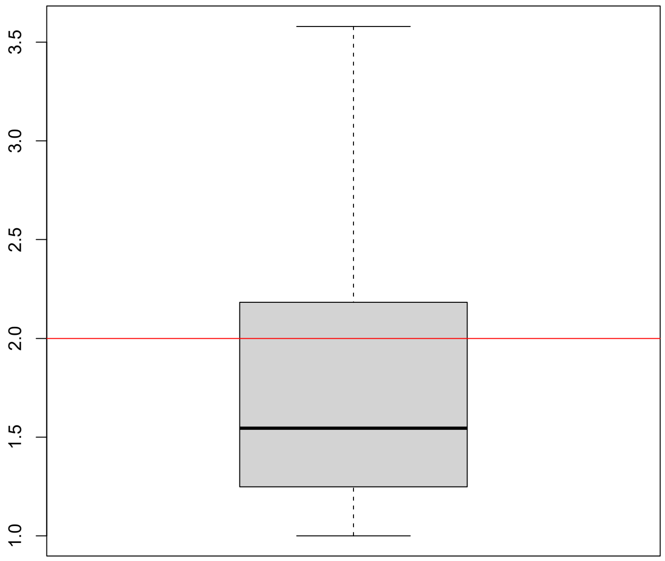
1.0
1.5
2.0
2.5
3.0
3.5

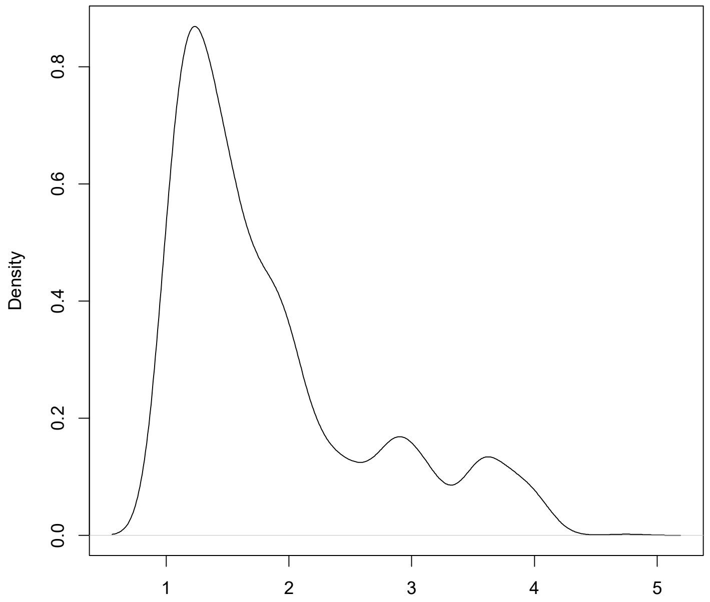
Density
0.0
0.2
0.4
0.6
0.8
1 2 3 4 5

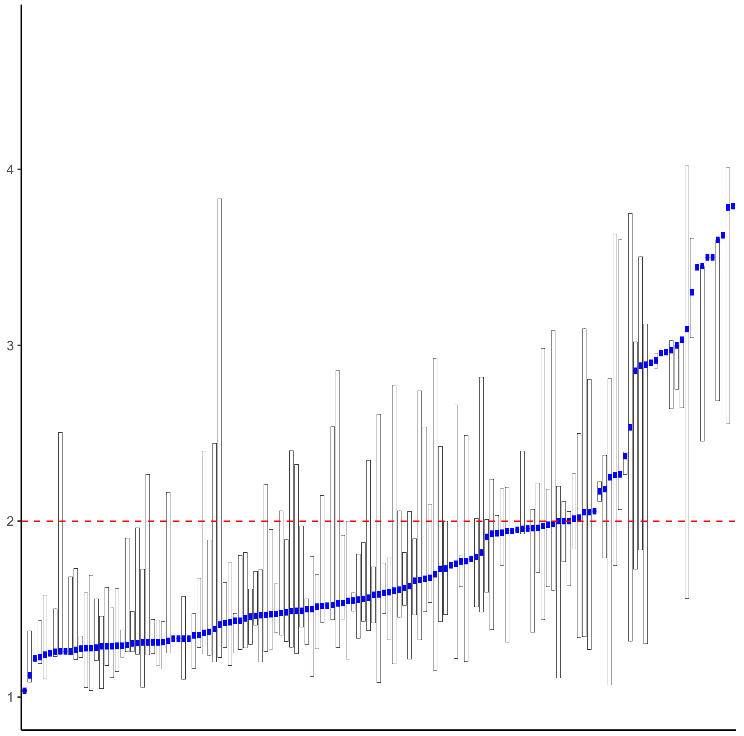
1
2
3
4

Tetrapanax_papyrifer

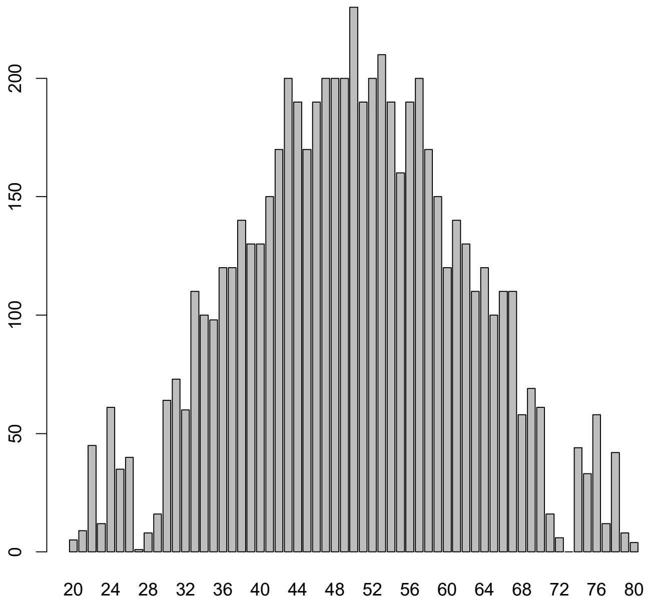

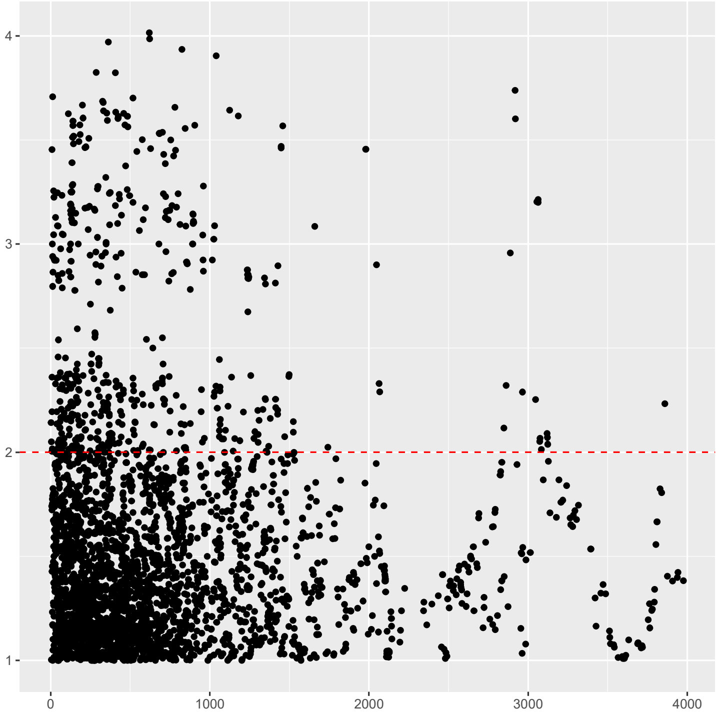

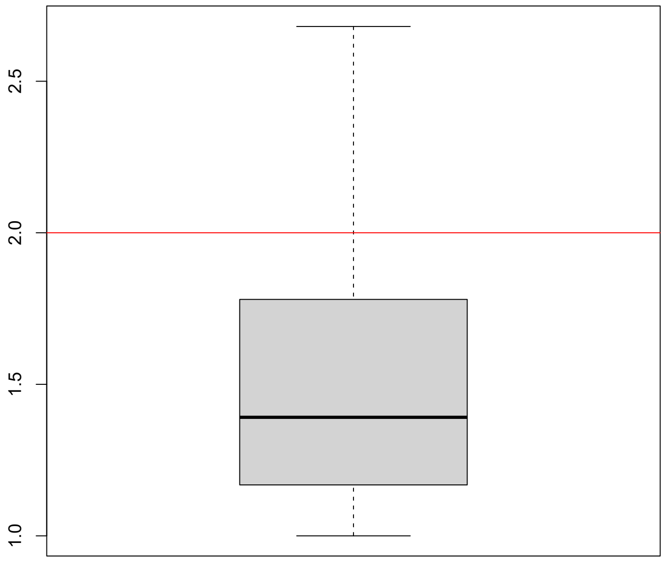

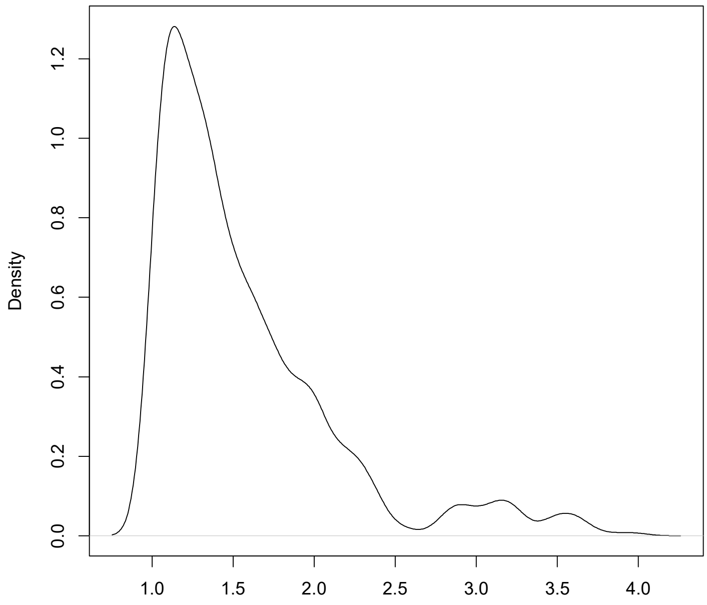

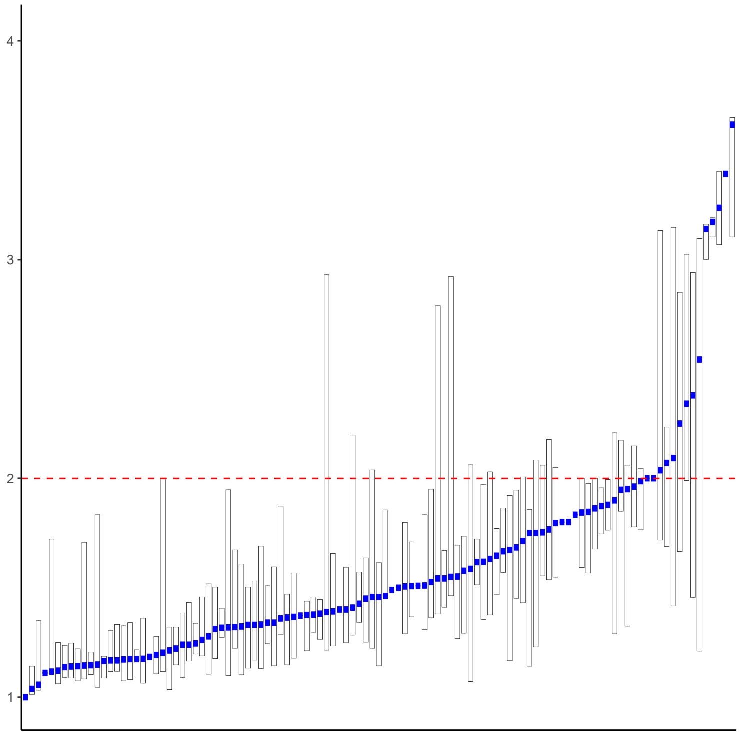

Supplement: Supplementary file 11 [file Presentation4.pdf]
